# Mapping geographical inequalities in childhood diarrhoeal morbidity and mortality in low-income and middle-income countries, 2000–17: analysis for the Global Burden of Disease Study 2017

**DOI:** 10.1016/S0140-6736(20)30114-8

**Published:** 2020-06-06

**Authors:** Local Burden of Disease Diarrhoea Collaborators, Robert C Reiner, Kirsten E Wiens, Aniruddha Deshpande, Mathew M Baumann, Paulina A Lindstedt, Brigette F Blacker, Christopher E Troeger, Lucas Earl, Sandra B Munro, Degu Abate, Hedayat Abbastabar, Foad Abd-Allah, Ahmed Abdelalim, Ibrahim Abdollahpour, Rizwan Suliankatchi Abdulkader, Getaneh Abebe, Kedir Hussein Abegaz, Lucas Guimarães Abreu, Michael R M Abrigo, Manfred Mario Kokou Accrombessi, Dilaram Acharya, Maryam Adabi, Oladimeji M Adebayo, Rufus Adesoji Adedoyin, Victor Adekanmbi, Olatunji O Adetokunboh, Beyene Meressa Adhena, Mohsen Afarideh, Keivan Ahmadi, Mehdi Ahmadi, Anwar E Ahmed, Muktar Beshir Ahmed, Rushdia Ahmed, Olufemi Ajumobi, Chalachew Genet Akal, Temesgen Yihunie Akalu, Ali S Akanda, Genet Melak Alamene, Turki M Alanzi, James R Albright, Jacqueline Elizabeth Alcalde Rabanal, Birhan Tamene Alemnew, Zewdie Aderaw Alemu, Beriwan Abdulqadir Ali, Muhammad Ali, Mehran Alijanzadeh, Vahid Alipour, Syed Mohamed Aljunid, Ali Almasi, Amir Almasi-Hashiani, Hesham M Al-Mekhlafi, Khalid Altirkawi, Nelson Alvis-Guzman, Nelson J Alvis-Zakzuk, Azmeraw T Amare, Saeed Amini, Arianna Maever Loreche Amit, Catalina Liliana Andrei, Masresha Tessema Anegago, Mina Anjomshoa, Fereshteh Ansari, Carl Abelardo T Antonio, Ernoiz Antriyandarti, Seth Christopher Yaw Appiah, Jalal Arabloo, Olatunde Aremu, Bahram Armoon, Krishna K Aryal, Afsaneh Arzani, Mohsen Asadi-Lari, Alebachew Fasil Ashagre, Hagos Tasew Atalay, Suleman Atique, Sachin R Atre, Marcel Ausloos, Leticia Avila-Burgos, Ashish Awasthi, Nefsu Awoke, Beatriz Paulina Ayala Quintanilla, Getinet Ayano, Martin Amogre Ayanore, Asnakew Achaw Ayele, Yared A Asmare Aynalem, Samad Azari, Ebrahim Babaee, Alaa Badawi, Shankar M Bakkannavar, Senthilkumar Balakrishnan, Ayele Geleto Bali, Maciej Banach, Aleksandra Barac, Till Winfried Bärnighausen, Huda Basaleem, Quique Bassat, Mohsen Bayati, Neeraj Bedi, Masoud Behzadifar, Meysam Behzadifar, Yibeltal Alemu Bekele, Michelle L Bell, Derrick A Bennett, Dessalegn Ajema Berbada, Tina Beyranvand, Anusha Ganapati Bhat, Krittika Bhattacharyya, Suraj Bhattarai, Soumyadeep Bhaumik, Ali Bijani, Boris Bikbov, Raaj Kishore Biswas, Kassawmar Angaw Bogale, Somayeh Bohlouli, Oliver J Brady, Nicola Luigi Bragazzi, Nikolay Ivanovich Briko, Andrey Nikolaevich Briko, Sharath Burugina Nagaraja, Zahid A Butt, Ismael R Campos-Nonato, Julio Cesar Campuzano Rincon, Rosario Cárdenas, Félix Carvalho, Franz Castro, Collins Chansa, Pranab Chatterjee, Vijay Kumar Chattu, Bal Govind Chauhan, Ken Lee Chin, Devasahayam J Christopher, Dinh-Toi Chu, Rafael M Claro, Natalie M Cormier, Vera M Costa, Giovanni Damiani, Farah Daoud, Lalit Dandona, Rakhi Dandona, Amira Hamed Darwish, Ahmad Daryani, Jai K Das, Rajat Das Gupta, Tamirat Tesfaye Dasa, Claudio Alberto Davila, Nicole Davis Weaver, Dragos Virgil Davitoiu, Jan-Walter De Neve, Feleke Mekonnen Demeke, Asmamaw Bizuneh Demis, Gebre Teklemariam Demoz, Edgar Denova-Gutiérrez, Kebede Deribe, Assefa Desalew, Getenet Ayalew Dessie, Samath Dhamminda Dharmaratne, Preeti Dhillon, Meghnath Dhimal, Govinda Prasad Dhungana, Daniel Diaz, Eric L Ding, Helen Derara Diro, Shirin Djalalinia, Huyen Phuc Do, David Teye Doku, Christiane Dolecek, Manisha Dubey, Eleonora Dubljanin, Bereket Duko Adema, Susanna J Dunachie, Andre R Durães, Senbagam Duraisamy, Andem Effiong, Aziz Eftekhari, Iman El Sayed, Maysaa El Sayed Zaki, Maha El Tantawi, Demelash Abewa Elemineh, Shaimaa I El-Jaafary, Hajer Elkout, Aisha Elsharkawy, Shymaa Enany, Aklilu Endalamfaw, Daniel Adane Endalew, Sharareh Eskandarieh, Alireza Esteghamati, Arash Etemadi, Tamer H Farag, Emerito Jose A Faraon, Mohammad Fareed, Roghiyeh Faridnia, Andrea Farioli, Andre Faro, Hossein Farzam, Ali Akbar Fazaeli, Mehdi Fazlzadeh, Netsanet Fentahun, Seyed-Mohammad Fereshtehnejad, Eduarda Fernandes, Irina Filip, Florian Fischer, Masoud Foroutan, Joel Msafiri Francis, Richard Charles Franklin, Joseph Jon Frostad, Takeshi Fukumoto, Reta Tsegaye Gayesa, Kidane Tadesse Gebremariam, Ketema Bizuwork Bizuwork Gebremedhin, Gebreamlak Gebremedhn Gebremeskel, Getnet Azeze Gedefaw, Yilma Chisha Dea Geramo, Birhanu Geta, Kebede Embaye Gezae, Ahmad Ghashghaee, Fariba Ghassemi, Paramjit Singh Gill, Ibrahim Abdelmageed Ginawi, Srinivas Goli, Nelson G M Gomes, Sameer Vali Gopalani, Bárbara Niegia Garcia Goulart, Ayman Grada, Harish Chander Gugnani, Davide Guido, Rafael Alves Guimares, Yuming Guo, Rajeev Gupta, Rahul Gupta, Nima Hafezi-Nejad, Michael Tamene Haile, Gessessew Bugssa Hailu, Arvin Haj-Mirzaian, Arya Haj-Mirzaian, Brian James Hall, Demelash Woldeyohannes Handiso, Hamidreza Haririan, Ninuk Hariyani, Ahmed I Hasaballah, Md. Mehedi Hasan, Amir Hasanzadeh, Hadi Hassankhani, Hamid Yimam Hassen, Desta Haftu Hayelom, Behnam Heidari, Nathaniel J Henry, Claudiu Herteliu, Fatemeh Heydarpour, Hagos D de Hidru, Chi Linh Hoang, Praveen Hoogar, Mojtaba Hoseini-Ghahfarokhi, Naznin Hossain, Mostafa Hosseini, Mehdi Hosseinzadeh, Mowafa Househ, Guoqing Hu, Ayesha Humayun, Syed Ather Hussain, Segun Emmanuel Ibitoye, Olayinka Stephen Ilesanmi, Milena D Ilic, Leeberk Raja Inbaraj, Seyed Sina Naghibi Irvani, Sheikh Mohammed Shariful Islam, Chinwe Juliana Iwu, Anelisa Jaca, Nader Jafari Balalami, Nader Jahanmehr, Mihajlo Jakovljevic, Amir Jalali, Achala Upendra Jayatilleke, Ensiyeh Jenabi, Ravi Prakash Jha, Vivekanand Jha, John S Ji, Peng Jia, Kimberly B Johnson, Jost B Jonas, Jacek Jerzy Jozwiak, Ali Kabir, Zubair Kabir, Amaha Kahsay, Hamed Kalani, Tanuj Kanchan, Behzad Karami Matin, André Karch, Surendra Karki, Amir Kasaeian, Gebremicheal Gebreslassie Kasahun, Gbenga A Kayode, Ali Kazemi Karyani, Peter Njenga Keiyoro, Daniel Bekele Ketema, Yousef Saleh Khader, Morteza Abdullatif Khafaie, Nauman Khalid, Ali Talha Khalil, Ibrahim Khalil, Rovshan Khalilov, Md Nuruzzaman Khan, Ejaz Ahmad Khan, Gulfaraz Khan, Junaid Khan, Khaled Khatab, Amir Khater, Mona M Khater, Alireza Khatony, Maryam Khayamzadeh, Mohammad Khazaei, Salman Khazaei, Ehsan Khodamoradi, Mohammad Hossein Khosravi, Jagdish Khubchandani, Aliasghar A Kiadaliri, Yun Jin Kim, Ruth W Kimokoti, Sezer Kisa, Adnan Kisa, Niranjan Kissoon, Shivakumar KM KM M Kondlahalli, Margaret N Kosek, Ai Koyanagi, Moritz U G Kraemer, Kewal Krishan, Nuworza Kugbey, G Anil Kumar, Manasi Kumar, Pushpendra Kumar, Dian Kusuma, Carlo La Vecchia, Ben Lacey, Aparna Lal, Dharmesh Kumar Lal, Faris Hasan Lami, Van C Lansingh, Savita Lasrado, Paul H Lee, Mostafa Leili, Tsegaye Tsegaye Lolaso Lolaso Lenjebo, Aubrey J Levine, Sonia Lewycka, Shanshan Li, Shai Linn, Rakesh Lodha, Joshua Longbottom, Platon D Lopukhov, Sameh Magdeldin, Phetole Walter Mahasha, Narayan Bahadur Mahotra, Deborah Carvalho Malta, Abdullah A Mamun, Navid Manafi, Farzad Manafi, Ana-Laura Manda, Mohammad Ali Mansournia, Chabila Christopher Mapoma, Dadi Marami, Laurie B Marczak, Francisco Rogerlândio Martins-Melo, Winfried März, Anthony Masaka, Manu Raj Mathur, Pallab K Maulik, Benjamin K Mayala, Colm McAlinden, Man Mohan Mehndiratta, Ravi Mehrotra, Kala M Mehta, Gebrekiros Gebremichael Meles, Addisu Melese, Ziad A Memish, Alemayehu Toma Mena, Ritesh G Menezes, Melkamu Merid Mengesha, Desalegn Tadese Mengistu, Getnet Mengistu, Tuomo J Meretoja, Bartosz Miazgowski, Kebadnew Mulatu M Mihretie, Molly K Miller-Petrie, Edward J Mills, Seyed Mostafa Mir, Parvaneh Mirabi, Erkin M Mirrakhimov, Amjad Mohamadi-Bolbanabad, Karzan Abdulmuhsin Mohammad, Yousef Mohammad, Dara K Mohammad, Aso Mohammad Darwesh, Naser Mohammad Gholi Mezerji, Noushin Mohammadifard, Ammas Siraj Mohammed, Shafiu Mohammed, Jemal Abdu Mohammed, Farnam Mohebi, Ali H Mokdad, Lorenzo Monasta, Yoshan Moodley, Masoud Moradi, Ghobad Moradi, Mohammad Moradi-Joo, Maziar Moradi-Lakeh, Paula Moraga, Abbas Mosapour, Simin Mouodi, Seyyed Meysam Mousavi, Miliva Mozaffor Mozaffor Mozaffor, Atalay Goshu Muluneh, Moses K Muriithi, Christopher J L Murray, GVS Murthy, Kamarul Imran Musa, Ghulam Mustafa, Saravanan Muthupandian, Mehdi Naderi, Ahamarshan Jayaraman Nagarajan, Mohsen Naghavi, Farid Najafi, Vinay Nangia, Javad Nazari, Duduzile Edith Ndwandwe, Ionut Negoi, Josephine W Ngunjiri, QuynhAnh P Nguyen, Trang Huyen Nguyen, Cuong Tat Nguyen, Dabere Nigatu, Dina Nur Anggraini Ningrum, Chukwudi A Nnaji, Marzieh Nojomi, Jean Jacques Noubiap, In-Hwan Oh, Oluchi Okpala, Andrew T Olagunju, Ahmed Omar Bali, Obinna E Onwujekwe, Doris D V Ortega-Altamirano, Osayomwanbo Osarenotor, Frank B Osei, Mayowa Ojo Owolabi, Mahesh P A, Jagadish Rao Padubidri, Adrian Pana, Tahereh Pashaei, Sanghamitra Pati, Ajay Patle, George C Patton, Kebreab Paulos, Veincent Christian Filipino Pepito, Alexandre Pereira, Norberto Perico, Konrad Pesudovs, David M Pigott, Bakhtiar Piroozi, James A Platts-Mills, Mario Poljak, Maarten J Postma, Hadi Pourjafar, Farshad Pourmalek, Akram Pourshams, Hossein Poustchi, Sergio I Prada, Liliana Preotescu, Hedley Quintana, Navid Rabiee, Mohammad Rabiee, Amir Radfar, Alireza Rafiei, Fakher Rahim, Vafa Rahimi-Movaghar, Muhammad Aziz Rahman, Fatemeh Rajati, Kiana Ramezanzadeh, Saleem M Rana, Chhabi Lal Ranabhat, Davide Rasella, Salman Rawaf, David Laith Rawaf, Lal Rawal, Giuseppe Remuzzi, Vishnu Renjith, Andre M N Renzaho, Melese Abate Reta, Satar Rezaei, Ana Isabel Ribeiro, Jennifer Rickard, Carlos Miguel Rios González, Maria Jesus Rios-Blancas, Leonardo Roever, Luca Ronfani, Elias Merdassa Roro, Ali Rostami, Dietrich Rothenbacher, Enrico Rubagotti, Salvatore Rubino, Anas M Saad, Siamak Sabour, Ehsan Sadeghi, Saeed Safari, Mahdi Safdarian, Rajesh Sagar, Mohammad Ali Sahraian, S. Mohammad Sajadi, Mohammad Reza Salahshoor, Nasir Salam, Farkhonde Salehi, Saleh Salehi Zahabi, Marwa R Rashad Salem, Hosni Salem, Yahya Salimi, Hamideh Salimzadeh, Evanson Zondani Sambala, Abdallah M Samy, Juan Sanabria, Itamar S Santos, Sivan Yegnanarayana Iyer Saraswathy, Abdur Razzaque Sarker, Benn Sartorius, Brijesh Sathian, Maheswar Satpathy, Alyssa N Sbarra, Lauren E Schaeffer, David C Schwebel, Anbissa Muleta Senbeta, Subramanian Senthilkumaran, Hosein Shabaninejad, Amira A Shaheen, Masood Ali Shaikh, Ali S Shalash, Seifadin Ahmed Shallo, Mehran Shams-Beyranvand, MohammadBagher Shamsi, Morteza Shamsizadeh, Mehdi Sharif, Muki Shehu Shey, Kenji Shibuya, Wondimeneh Shibabaw Shibabaw Shiferaw, Mika Shigematsu, Apurba Shil, Jae Il Shin, Rahman Shiri, Reza Shirkoohi, Si Si, Soraya Siabani, Jasvinder A Singh, Narinder Pal Singh, Dhirendra Narain Sinha, Malede Mequanent Sisay, Eirini Skiadaresi, David L Smith, Mohammad Reza Sobhiyeh, Anton Sokhan, Moslem Soofi, Joan B Soriano, Muluken Bekele Sorrie, Ireneous N Soyiri, Chandrashekhar T Sreeramareddy, Agus Sudaryanto, Mu'awiyyah Babale Sufiyan, Hafiz Ansar Rasul Suleria, Bryan L Sykes, Koku Sisay Tamirat, Aberash Abay Tassew, Nuno Taveira, Bineyam Taye, Arash Tehrani-Banihashemi, Mohamad-Hani Temsah, Berhe etsay Tesfay, Fisaha Haile Tesfay, Zemenu Tadesse Tessema, Kavumpurathu Raman Thankappan, Sathish Thirunavukkarasu, Nihal Thomas, Kenean Getaneh Tlaye, Boikhutso Tlou, Marcos Roberto Tovani-Palone, Eugenio Traini, Khanh Bao Tran, Indang Trihandini, Irfan Ullah, Bhaskaran Unnikrishnan, Sahel Valadan Tahbaz, Pascual R Valdez, Santosh Varughese, Yousef Veisani, Francesco S Violante, Sebastian Vollmer, Theo Vos, Fiseha Wadilo Wada, Yasir Waheed, Yafeng Wang, Yuan-Pang Wang, Girmay Teklay Weldesamuel, Catherine A Welgan, Ronny Westerman, Taweewat Wiangkham, Tissa Wijeratne, Charles Shey Shey Wiysonge, Haileab Fekadu Wolde, Dawit Zewdu Wondafrash, Tewodros Eshete Wonde, Ai-Min Wu, Gelin Xu, Ali Yadollahpour, Seyed Hossein Yahyazadeh Jabbari, Tomohide Yamada, Mehdi Yaseri, Muluken Azage Yenesew, Alex Yeshaneh, Mekdes Tigistu Yilma, Ebrahim M Yimer, Paul Yip, Biruck Desalegn Yirsaw, Engida Yisma, Naohiro Yonemoto, Mustafa Z Younis, Hebat-Allah Salah A Yousof, Chuanhua Yu, Hasan Yusefzadeh, Mohammad Zamani, Carlos Zambrana-Torrelio, Hamed Zandian, Ayalew Jejaw Zeleke, Nejimu Biza Zepro, Taye Abuhay Zewale, Dongyu Zhang, Yunquan Zhang, Xiu-Ju Zhao, Arash Ziapour, Sanjay Zodpey, Simon I Hay

## Abstract

**Background:**

Across low-income and middle-income countries (LMICs), one in ten deaths in children younger than 5 years is attributable to diarrhoea. The substantial between-country variation in both diarrhoea incidence and mortality is attributable to interventions that protect children, prevent infection, and treat disease. Identifying subnational regions with the highest burden and mapping associated risk factors can aid in reducing preventable childhood diarrhoea.

**Methods:**

We used Bayesian model-based geostatistics and a geolocated dataset comprising 15 072 746 children younger than 5 years from 466 surveys in 94 LMICs, in combination with findings of the Global Burden of Diseases, Injuries, and Risk Factors Study (GBD) 2017, to estimate posterior distributions of diarrhoea prevalence, incidence, and mortality from 2000 to 2017. From these data, we estimated the burden of diarrhoea at varying subnational levels (termed units) by spatially aggregating draws, and we investigated the drivers of subnational patterns by creating aggregated risk factor estimates.

**Findings:**

The greatest declines in diarrhoeal mortality were seen in south and southeast Asia and South America, where 54·0% (95% uncertainty interval [UI] 38·1–65·8), 17·4% (7·7–28·4), and 59·5% (34·2–86·9) of units, respectively, recorded decreases in deaths from diarrhoea greater than 10%. Although children in much of Africa remain at high risk of death due to diarrhoea, regions with the most deaths were outside Africa, with the highest mortality units located in Pakistan. Indonesia showed the greatest within-country geographical inequality; some regions had mortality rates nearly four times the average country rate. Reductions in mortality were correlated to improvements in water, sanitation, and hygiene (WASH) or reductions in child growth failure (CGF). Similarly, most high-risk areas had poor WASH, high CGF, or low oral rehydration therapy coverage.

**Interpretation:**

By co-analysing geospatial trends in diarrhoeal burden and its key risk factors, we could assess candidate drivers of subnational death reduction. Further, by doing a counterfactual analysis of the remaining disease burden using key risk factors, we identified potential intervention strategies for vulnerable populations. In view of the demands for limited resources in LMICs, accurately quantifying the burden of diarrhoea and its drivers is important for precision public health.

**Funding:**

Bill & Melinda Gates Foundation.

## Introduction

Across low-income and middle-income countries (LMICs), diarrhoea causes more than half a million childhood deaths annually.^1^ In addition to this staggering loss of life, more than 910 million childhood cases of diarrhoea per year^2^ are distributed unequally across the population, causing not only acute morbidity but also long-term disability in children who suffer repeatedly with enteric infections.^3^ National-level analyses of the burden of childhood diarrhoea, measured by both death rates and incidence, have exposed substantial variation. In LMICs in 2017, the incidence of diarrhoea ranged from less than one episode per child per year to more than four episodes per child per year.^2^ In the same population, the case-fatality rate of diarrhoea can vary from one per 10 000 infections to more than 20 per 10 000 infections.^4^

WHO's integrated Global Action Plan for Pneumonia and Diarrhoea (GAPPD) identified three approaches to reduce the burden of diarrhoea: protect, prevent, and treat.^5^ Healthy children are less likely to have severe diarrhoea episodes,^6^ so diarrhoeal burden can be reduced by prioritising good health practices from birth. As such, reducing general health risk factors, such as child growth failure (CGF) indicators of stunting, wasting, and underweight,^4^, ^7^ can protect a child from diarrhoea. Preventing illness by promoting vaccination and improved water, sanitation, and hygiene (WASH) can similarly reduce diarrhoeal burden.^8^, ^9^ Finally, appropriate treatment, such as oral rehydration solution (ORS), the efficacy of which exceeds 90%,^10^ can substantially reduce death resulting from disease-associated dehydration.^11^, ^12^

**Research in context**

**Evidence before this study**

In the Global Burden of Diseases, Injuries, and Risk Factors Study (GBD) 2017, diarrhoea was the third leading cause of death among children younger than 5 years and was reported to have caused an estimated 534 000 deaths. WHO's integrated Global Action Plan for the Prevention and Control of Pneumonia and Diarrhoea calls for protection of children from disease by establishing good health practices, preventing infection from occurring, and treating infections when they occur. Over the past decade, large reductions in childhood mortality due to diarrhoea have been recorded across low-income and middle-income countries (LMICs), in part attributable to strategies to reduce child growth failure (CGF), improve water, sanitation, and hygiene (WASH), and increase access to oral rehydration therapy and vaccines. Several studies have recorded substantial between-country variation in both the likelihood of a child experiencing a diarrhoea episode and that episode resulting in death. To reduce the burden of childhood diarrhoea, the remaining subnational regions with the highest prevalence and those with the lowest levels of interventions should be identified.

**Added value of this study**

We present the first high-resolution subnational estimates of diarrhoeal morbidity and mortality from 2000 to 2017 in LMICs. We used Bayesian model-based geostatistics and an extensive geolocated dataset in combination with established methods from GBD 2017 for both burden estimation and risk factor association. We did a systematic assessment of local variation to estimate the distribution of diarrhoea prevalence, incidence, and mortality. Our estimates show considerable subnational variation in the diarrhoeal burden for children younger than 5 years. We synthesised new subnational estimates of the key risk factors of diarrhoea to discern averted deaths attributable to improvements in these drivers of diarrhoeal morbidity and mortality. Finally, when focusing on subnational regions with the highest remaining burden, we identified not only which regions of the world have the highest diarrhoeal burden and continued geographical inequalities but also the subnational risk factors that require targeted interventions to alleviate this burden.

**Implications of all the available evidence**

By providing estimates of remaining diarrhoeal burden at various spatial scales, we have identified countries and locations that are still most in need of preventive and protective measures. Our results indicate that regions with the highest burden had varied exposure to select risk factors; however, similar to previous studies, most high-burden areas showed some combination of poor WASH, high CGF, and low oral rehydration solution coverage. In view of the limited resources in many LMICs, quantification of both the local burden of diarrhoea and its drivers is important to maximise impact.

Distal determinants of diarrhoeal mortality, such as measurable indicators of child welfare,^13^ have been geospatially mapped at the local level in Africa, including under-5 mortality,^14^ CGF,^15^ and education levels of the broader population.^16^ Country-level assessment of these determinants can mask subnational variation and provide limited information with which to formulate policy.^17^ Furthermore, mapping interventions such as malaria nets^18^ and vaccines^19^ has shown the positive effects of these strategies on reducing diseases. Subsequently, precise mapping of diarrhoea-related interventions, including ORS coverage^20^ and access to safe water and sanitation (Deshpande A, unpublished data), in addition to diarrhoea incidence and death, provides in-depth analysis to aid in the prevention of deaths associated with diarrhoea.

National trends in diarrhoeal burden are associated with (and in many cases driven by) national trends in risk factors associated with the protect, prevent, and treat strategy. Childhood stunting, poor sanitation access, and low ORS coverage are risk factors most strongly associated with changes in diarrhoeal burden.^4^ To date, no comprehensive attempt has been made to quantify either the subnational variation in diarrhoea or these key risk factors across LMICs. Several isolated studies of subnational variation in diarrhoea,^21^ childhood stunting,^15^ WASH,^22^ and ORS coverage^23^ have shown striking variation at the spatial scale investigated. However, without estimates designed to be comparable across space and time, it is difficult to analyse such scattered information as a cohesive body of knowledge.

Reducing morbidity and mortality could be accomplished by targeting regions with the highest mortality rate, or those with the greatest total number of deaths. At the national scale, for example, Central African Republic was estimated to have the highest childhood mortality rate attributable to diarrhoea globally, at 6·9 deaths per 1000 children. Because of this country's relatively small population, however, this rate translates to approximately 4156 children dying per year.^21^ By contrast, in Nigeria, which has a much larger population than Central African Republic, an estimated 104 000 children a year die from diarrhoea, but the mortality rate is less than half that of Central African Republic (3·0 deaths per 1000 children).^24^ A location within a country could have a relatively low risk of mortality but a sufficiently large population so it is a greater contributor to overall burden than other areas in that country. Thus, decisions aimed at optimum burden reduction might overlook those at highest risk. Mapping both rates and counts can aid in the design of intervention strategies that efficiently save lives while also highlighting entrenched geographical disparities in diarrhoeal burden.

Here, we present an analysis of local variation in diarrhoeal morbidity and mortality in children younger than 5 years across 94 LMICs between 2000 and 2017. We used Bayesian model-based geostatistics and an extensive geolocated dataset (describing 3 738 327 diarrhoea episodes across 15 072 746 children) in combination with methods from the Global Burden of Diseases, Injuries, and Risk Factors Study (GBD) 2017 to estimate posterior distributions of continuous continent-wide surfaces of diarrhoea prevalence, incidence, and mortality.^1^, ^2^ We then aggregated our estimates at second administrative-level units (eg, districts in Uganda or divisions in Kenya; henceforth referred to as units), to identify regions with the most pronounced rate of burden reduction versus those that continue to have higher-than-average burden. We combined this analysis with published estimates of subnational CGF variation^15^ and new estimates of subnational variation in WASH (Deshpande A, unpublished data) and ORS^20^ to break down diarrhoeal burden. Finally, through these linked analyses, we identified regions most in need of tailored interventions to reduce the burden of this largely preventable disease.

## Methods

### Definitions

Diarrhoea episodes were defined as three or more loose stools over a 24-h period.^4^ Diarrhoea prevalence was defined as the point prevalence of children younger than 5 years with diarrhoea. Incidence was defined as the number of cases of diarrhoea in children younger than 5 years per child per year. Mortality was defined as the number of deaths among children younger than 5 years due to diarrhoea per child per year. Rates per 1000 are presented in the figures and represent prevalence, incidence, or mortality rates per child multiplied by 1000). Diarrhoea burden is used throughout this Article to refer to the combined burden of prevalence, incidence, and mortality.

### Data

We included 94 LMICs in our analysis; these countries were defined according to the Socio-demographic Index (SDI), which assesses development based on education, fertility, and income.^24^ Where appropriate, we use designated ISO 3166-1 alpha-3 codes for countries. Our study complies with the Guidelines for Accurate and Transparent Health Estimates Reporting (GATHER) recommendations (appendix 1 pp 84–85).^25^

#### Surveys

We compiled 466 household surveys (including the Demographic and Health Survey [DHS], Multiple Indicator Cluster Survey [MICS], and other country-specific surveys) from 2000 to 2017 with geocoded information from 207 021 coordinates corresponding to survey clusters and 17 954 subnational polygon boundaries. We included surveys that asked if children younger than 5 years had diarrhoea, typically within the preceding 2 weeks. Potential bias attributable to seasonal variation in diarrhoea was addressed, as described in appendix 1 (p 5). Data were vetted for representativeness at the national level and subnational level, as appropriate. Data inclusion, coverage, and validation are further described in appendix 1 (pp 3, 9).

#### Spatial covariates

We compiled 15 covariates that were indexed at the subnational level and could possibly be related to diarrhoea prevalence, including access to roads, ratio of child dependents (aged 0–14 years) to working-age adults (aged 15–64 years), distance from rivers or lakes, night-time lights (time-varying covariate), elevation, population ratio of women of maternal age to children, population (time-varying covariate), aridity (time-varying covariate), urban or rural (time-varying covariate), urban proportion of the location (time-varying covariate), irrigation, number of people whose daily vitamin A needs could be met, prevalence of under-5 stunting (time-varying covariate), prevalence of under-5 wasting (time-varying covariate), and diphtheria-tetanus-pertussis immunisation coverage (time-varying covariate). We also included the Healthcare Access and Quality Index,^26^ percentage of the population with access to improved toilet types, and percentage of the population with access to improved water sources (as defined by WHO and UNICEF's Joint Monitoring Programme) as national-level time-varying covariates. We filtered these covariates for multicollinearity in each modelling region (appendix 1 pp 5–6) using variance inflation factor (VIF) analysis with a VIF threshold of 3.^27^ Covariate information, including plots of all covariates, is detailed in the appendix 1 (pp 25–26, 90–96).

### Statistical analysis

#### Geostatistical model

Prevalence data were used as inputs to a Bayesian model-based geostatistical framework. Briefly, this framework uses a spatially and temporally explicit hierarchical logistic regression model to predict prevalence. Potential interactions and non-linear relations between covariates and diarrhoea prevalence were incorporated using a stacked generalisation technique.^28^ Posterior distributions of all parameters and hyperparameters were estimated using R-INLA version 19.05.30.9000.^29^, ^30^ Uncertainty was calculated by taking 250 draws from the estimated posterior joint distribution of the model, and each uncertainty interval (UI) reported represents the 2·5th and 97·5th percentiles of those draws. Models were run independently in 14 geographically distinct modelling regions based on the GBD 2010 study,^31^ and one country-specific model in India. Analyses were done using R version 3.5.0. Maps were produced using ArcGIS Desktop 10.6. Additional details are provided in appendix 1 (pp 6–8).

#### Post estimation

Estimated prevalence was converted into incidence using an average duration of a diarrhoea episode of 4·2 days^4^ (appendix 1 p 9). We converted incidence surfaces to mortality surfaces by multiplying the incidence values by country-specific and year-specific case-fatality rates (which did not vary subnationally). We calibrated our continuous prevalence estimates to those of prevalence, mortality, and incidence from GBD 2017. However, we did not calibrate prevalence or incidence in South Africa because of unreasonably low estimates in this location in the GBD 2017 study. We then calculated population-weighted aggregations of the 250 draws of diarrhoea prevalence, mortality, and incidence estimates at the country level, first administrative-level unit, and second administrative-level unit (hereafter referred to as unit). This calculation resulted in estimates for 24 143 units within 94 countries. Geographical inequalities were quantified as the relative difference between each unit and the respective country average. We also estimated inequality using the Gini coefficient,^32^ which summarises the distribution of each indicator across the population, with a value of 0 representing perfect equality and 1 representing maximum inequality (appendix 1 p 12).

#### Counterfactual analyses using diarrhoea risk factors

Following the GAPPD framework, we did a post-hoc counterfactual analysis using subnational estimates of risk factors according to GBD 2017, including reducing prevalence of childhood stunting and childhood wasting (protect), access to improved sanitation and improved water (prevent), and increasing ORS coverage (treat). Some known diarrhoea risk factors (eg, low coverage of rotavirus vaccine, or no or partial breastfeeding) were not included because subnational estimates are currently not available for all 94 LMICs included in this study. We used the counterfactual analysis to estimate the number of deaths averted because of changes in CGF and WASH risk factors (appendix 1 pp 61–62).

#### Model validation

Models were validated using source-stratified five-fold cross validation. Holdout sets were created by combining randomised sets of second administrative unit cluster-level datapoints. Model performance was summarised by the bias (mean error), total variance (root-mean-square error), 95% data coverage within prediction intervals, and correlation between observed data and predictions. When possible, estimates were compared against existing estimates. All validation procedures and corresponding results are provided in appendix 1 (p 9).

### Role of the funding source

The funder had no role in study design, data collection, data analysis, data interpretation, or writing of the report. RCR had full access to all data in the study and had final responsibility for the decision to submit for publication.

## Results

Our model produced estimates of local diarrhoea prevalence, incidence, and mortality for 94 LMICs yearly from 2000 to 2017, showing subnational spatial and temporal variation. A large variation in diarrhoeal burden was seen, both between and within countries, and striking differences in trends were noted over time by location. Although, in many countries, rates of diarrhoeal morbidity and mortality were disproportionally high in less-populated rural areas, the absolute burden of diarrhoeal mortality was typically concentrated in highly populated urban centres. By integrating these subnational estimates of mortality with similar estimates of leading risk factors, improvements in WASH (Deshpande A, unpublished data) and prevention of CGF (relative to levels in 2000) were estimated to avert 46 000 (95% UI 32 000–170 000) and 245 000 (177 000–940 000) child deaths in 2017, respectively. The full array of our model outputs is provided in appendix 2 (pp 1–950), and online.

### Incidence of diarrhoea

**Figure 1:**
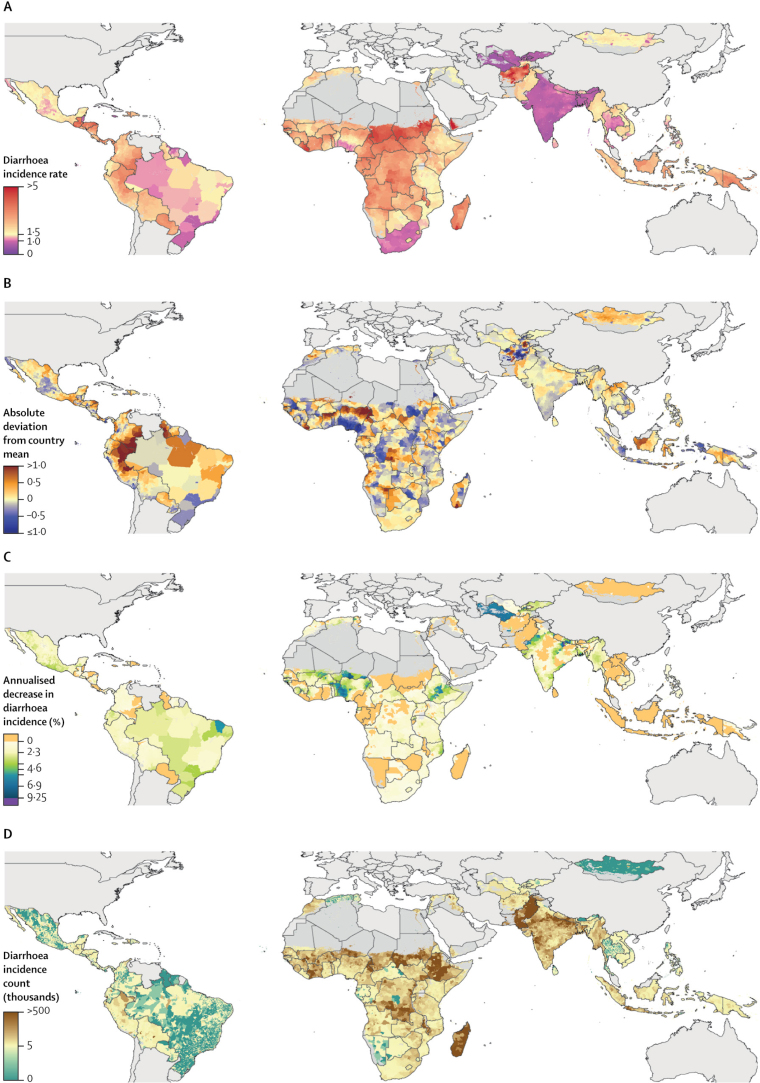
Mapping of diarrhoea incidence among children younger than 5 years in low-income and middle-income countries by second administrative-level unit, 2017 Estimated mean incidence rate per 1000 children in 2017 (A). Absolute deviation from mean incidence rate by country in 2017 (B). Annualised decrease in diarrhoea incidence rate from 2000 to 2017 (C). Estimated mean number of cases of diarrhoea among children in 2017 (D). All panels are aggregated to the second administrative-level unit. Maps reflect administrative boundaries, land cover, lakes, and population; grey-coloured grid cells were classified as barren or sparsely vegetated and had fewer than ten people per 1 × 1 km grid cell, or were not included in these analyses.^33^, ^34^, ^35^, ^36^, ^37^, ^38^

In 2017, Yemen had the most units exceeding five cases of diarrhoea per child per year (124 units), with Afghanistan (16 units) the only other country with such high incidence (figure 1A). It is unsurprising that Yemen had the most subnational units with high incidence, because the country had had the highest national incidence of diarrhoea globally, with 4·7 (95% UI [4·0–5·7]) cases per child per year. In 2017, the highest incidence of diarrhoea for sub-Saharan Africa was in Cameroon (4·8 [95% UI 2·9–7·4] cases per child per year in Mayo-Danay department, Extrême-Nord); for Latin America the highest incidence was in Guatemala (4·7 [3·7–5·8] cases per child per year in San Antonio Suchitepéquez department, Suchitepéquez; and 4·4 [3·5–5·5] cases per child per year in San Miguel Panán department, Suchitepéquez); and for southeast Asia the highest incidence of diarrhoea was in Papua New Guinea (3·5 [2·7–4·5] cases per child per year in Koroba-Kopiago district, Hela). Massive variation within regions is exemplified in central Asia and south Asia, where the highest incidence of diarrhoea by country spanned from 2506th to 24 391st across all LMICs (2·8 [95% UI 2·1–3·6] cases per child per year in Moskva district, Khatlon, Tajikistan; and 0·7 [0·4–1·3] cases per child per year in Aşgabat district, Aşgabat, Turkmenistan; figure 1A). Maps of upper and lower bounds for the uncertainty on incidence can be found in appendix 1 (p 47).

As with variation between countries, substantial variation was seen within most countries. 16 countries had at least one unit with an estimated incidence of diarrhoea more than 1·0 case per child per year higher than the national average (figure 1B). The district of Darqad, Takhar, Afghanistan, had an incidence of 6·3 (95% UI 4·2–9·5) cases per child per year, which was 2·3 cases per child per year higher than the national average (4·0 [2·8–5·3] cases per child per year). Conversely, only nine countries had units with incidence less than 1·0 case per child per year lower than their country average (appendix 2 pp 3–4, 478–950). Countries with large relative geographical inequality include Guyana, where the rate in the Marudi council, Upper Takutu-Upper Essequibo, was 2·4 (95% UI 2·0–3·1) cases per child per year, which is much higher than the country average of 1·2 (0·9–1·5) cases per child per year. It is important to note that the comparison in Afghanistan also illustrates a technical difficulty in summarising correlated uncertainty. In Afghanistan, the 95% UI for the estimated incidence of diarrhoea in Darqad overlaps that for average incidence across the country, but these UIs are based on summarising aggregations from draws of correlated incidence surfaces. In every draw from the posterior distribution of incidence, Darqad had an incidence at least 86·0% higher than that draw's estimated country incidence.

The substantial reduction in overall diarrhoeal burden since 2000 has not translated into a consistent reduction in incidence of diarrhoea. 5729 (24%) of 24 139 units had an increase in childhood diarrhoea incidence from 2000 to 2017 (figure 1C). Laos in particular contained 24 units with annual rates of change in diarrhoea incidence exceeding 5% per year. Conversely, among all units that had decreases in incidence, Nigeria saw the greatest number of units (n=40) with annual declines in diarrhoea incidence of 7% or more.

Incidence data provide information on the per person risk of disease. However, some units with the highest incidence of diarrhoea are sparsely populated. On the other hand, many units with the highest incidence of diarrhoea and moderate rates of diarrhoea have considerable populations. For example, in 2017, five units in Punjab, Pakistan (Dera Ghazi Khan, Faisalabad, Gujranwala, Lahore, and Multan) were estimated to have more than 21 (95% UI 14·8–28·9) million cases of diarrhoea in children younger than 5 years (figure 1D). Each of these units had an incidence less than 1·9 (95% UI 1·3–3·0) cases per child per year. By contrast, Wadhrah district in Hajjah, Yemen, had a high incidence of diarrhoea (5·5 [95% UI 4·3–7·0] cases per child per year), but because of this district's relatively small child population, there were only 9890 (7766–12 723) cases of diarrhoea (figure 1D). These incidence data suggest that interventions focused on lowering the absolute burden of diarrhoea might best be focused on urban areas, although this focus risks exacerbating existing geographical disparities.

### Mortality from diarrhoea

**Figure 2:**
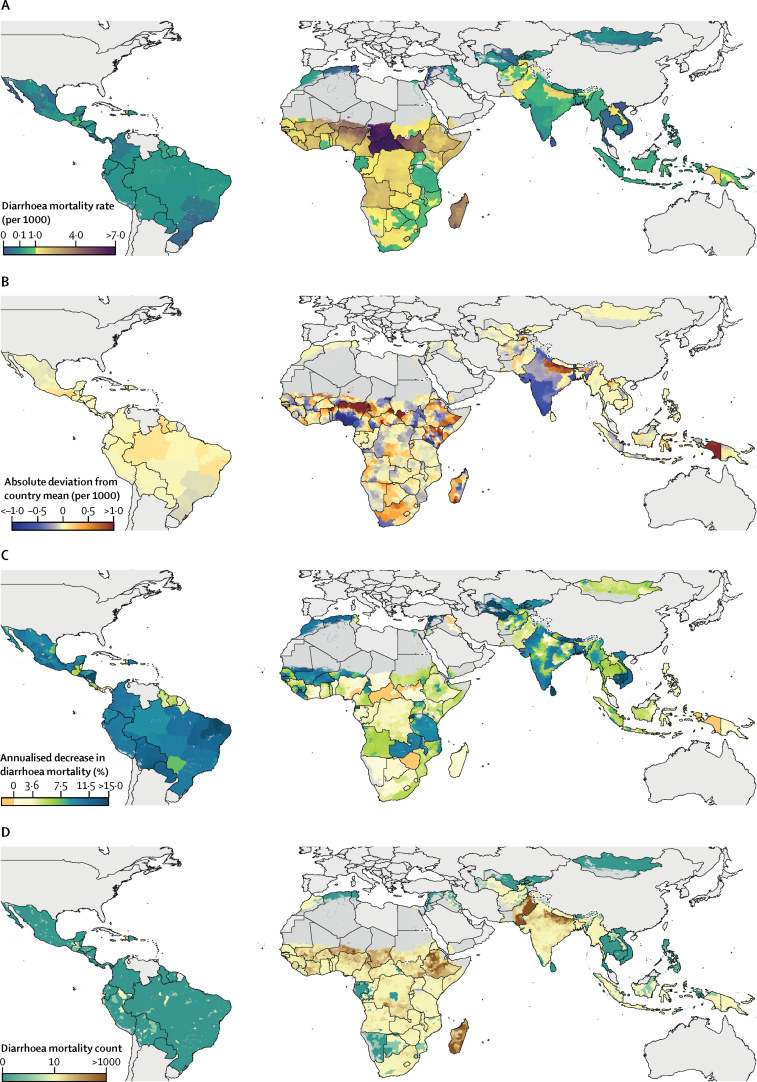
Mapping of diarrhoeal mortality among children younger than 5 years in low-income and middle-income countries by second administrative-level unit, 2017 Estimated mean mortality rate per 1000 children in 2017 (A). Absolute deviation from the mean mortality rate by country in 2017 (B). Annualised decrease in diarrhoeal mortality rate from 2000 to 2017 (C). Estimated mean number of diarrhoeal deaths among children in 2017 (D). All panels are aggregated to the second administrative-level unit. Maps reflect administrative boundaries, land cover, lakes, and population; grey-coloured grid cells were classified as barren or sparsely vegetated and had fewer than ten people per 1 × 1 km grid cell, or were not included in these analyses.^33^, ^34^, ^35^, ^36^, ^37^, ^38^

Similar to patterns noted previously on a subnational map of diarrhoeal mortality in Africa,^21^ substantial diarrhoeal burden was seen in several countries in the Sahel region of Africa, with Birao in Vakaga, Central African Republic, having the highest mortality rate globally of 8·2 (95% UI 6·8–9·7) deaths per 1000 children in 2017 (figure 2A). Seven countries had at least one unit exceeding five deaths per 1000 children, and all were located in Africa. For 46 countries, the GAPPD goal of decreasing childhood diarrhoeal mortality to less than one death per 1000 children was achieved in every second administrative-level unit by 2017 (appendix 2 pp 5–477). Global variation in diarrhoea mortality was so vast that rates for many countries remain several orders of magnitude lower than those in central sub-Saharan Africa (figure 2A).

**Figure 3:**
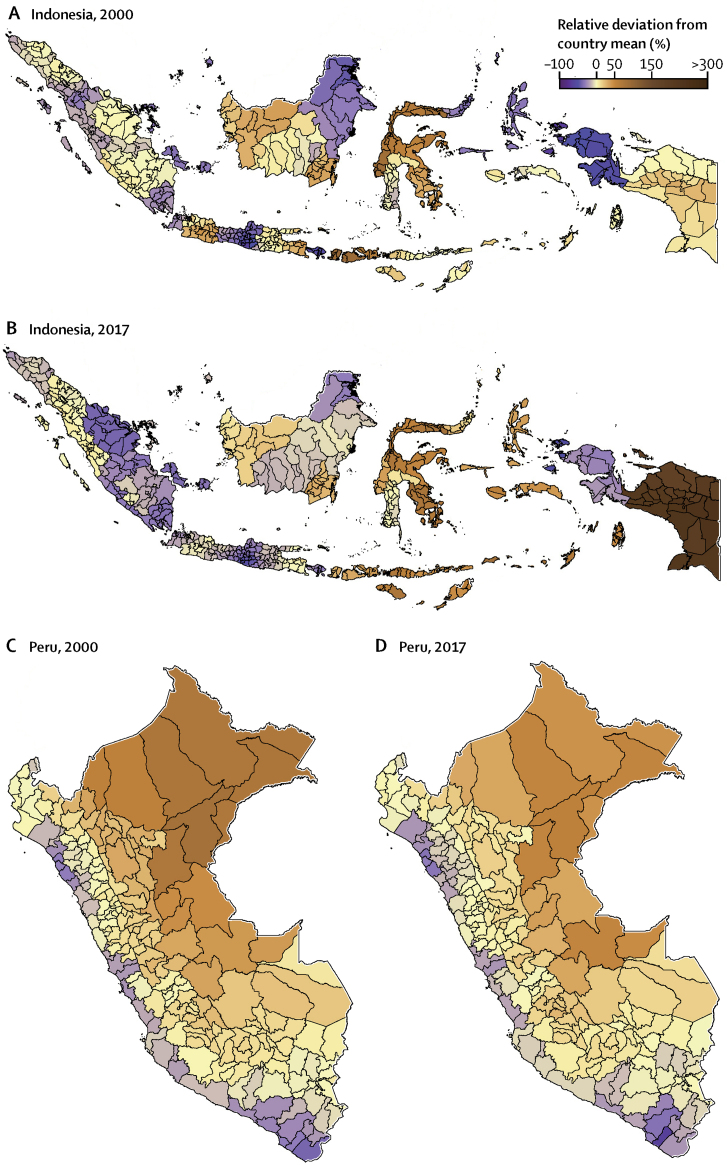
Relative geographical inequality of childhood diarrhoeal mortality in Indonesia and Peru in 2000 and 2017 Relative deviation of second administrative-level units from country mean for Indonesia in 2000 (A), Indonesia in 2017 (B), Peru in 2000 (C), and Peru in 2017 (D). Maps reflect administrative boundaries, land cover, lakes, and population; grey-coloured grid cells were classified as barren or sparsely vegetated and had fewer than ten people per 1 × 1 km grid cell, or were not included in these analyses.^33^, ^34^, ^35^, ^36^, ^37^, ^38^

Similar to incidence, substantial within-country variation was noted in diarrhoeal mortality. As previously highlighted in our Africa-focused analysis,^21^ some units in Nigeria in 2017 were far above the country average. Of the 100 largest deviations above the national mean mortality rate, 86 occurred in northern Nigeria (figure 2B). Only units in Chad, Kenya, and Nigeria had rates greater than one death per 1000 less than their country average mortality rate (figure 2B). When the analysis was done in terms of relative deviation from the mean, different patterns of subnational variation became apparent. Indonesia stood out as having many units within Papua that were more than three-fold the country average; in particular, the Boven Digoel Regency of Papua, Indonesia, was estimated to have a diarrhoeal mortality rate 3·4 times the national average (figures 3A, B). Similarly, 736 units of Mexico were estimated to have mortality rates more than double the national average (figure 2B). Although Nigeria had massive absolute deviations, units with the highest absolute deviations were 169·0% (95% UI 114·2–256·5) the national average (figure 2B). Maps of upper and lower bounds for uncertainty on incidence can be found in appendix 1 (p 48).

Unlike incidence of diarrhoea, diarrhoeal mortality declined in most units from 2000 to 2017. 8658 (36%) of 24 143 units showed reduced rates of childhood diarrhoeal mortality, by more than 10% per year (figure 2C). The greatest declines in diarrhoeal mortality were seen in south and southeast Asia and South America, where 54·0% (95% UI 38·1–65·8), 17·4% (7·7–28·4), and 59·5% (34·2–86·9) of units, respectively, recorded decreases in deaths from diarrhoea greater than 10%. Diarrhoeal mortality was estimated to have increased in only 112 (0·5%) units over this time, exclusively in Central African Republic, Indonesia, Kenya, South Sudan, and Tunisia. Although massive imbalances in mortality rates within Africa persisted in 2017, most diarrhoeal deaths in LMICs occurred outside of Africa. Importantly, because of the juxtaposition of mortality rate to population size, the five units with the largest number of diarrhoeal deaths were all outside of Africa, specifically in Punjab, Pakistan (Dera Ghazi Khan, Faisalabad, Gujranwala, Lahore, and Multan; figure 2D). By comparison, the total number of deaths in these five units was more than double the total estimated diarrhoeal deaths in Liberia, Rwanda, and Togo.

### Geographical inequality in diarrhoeal mortality

**Figure 4:**
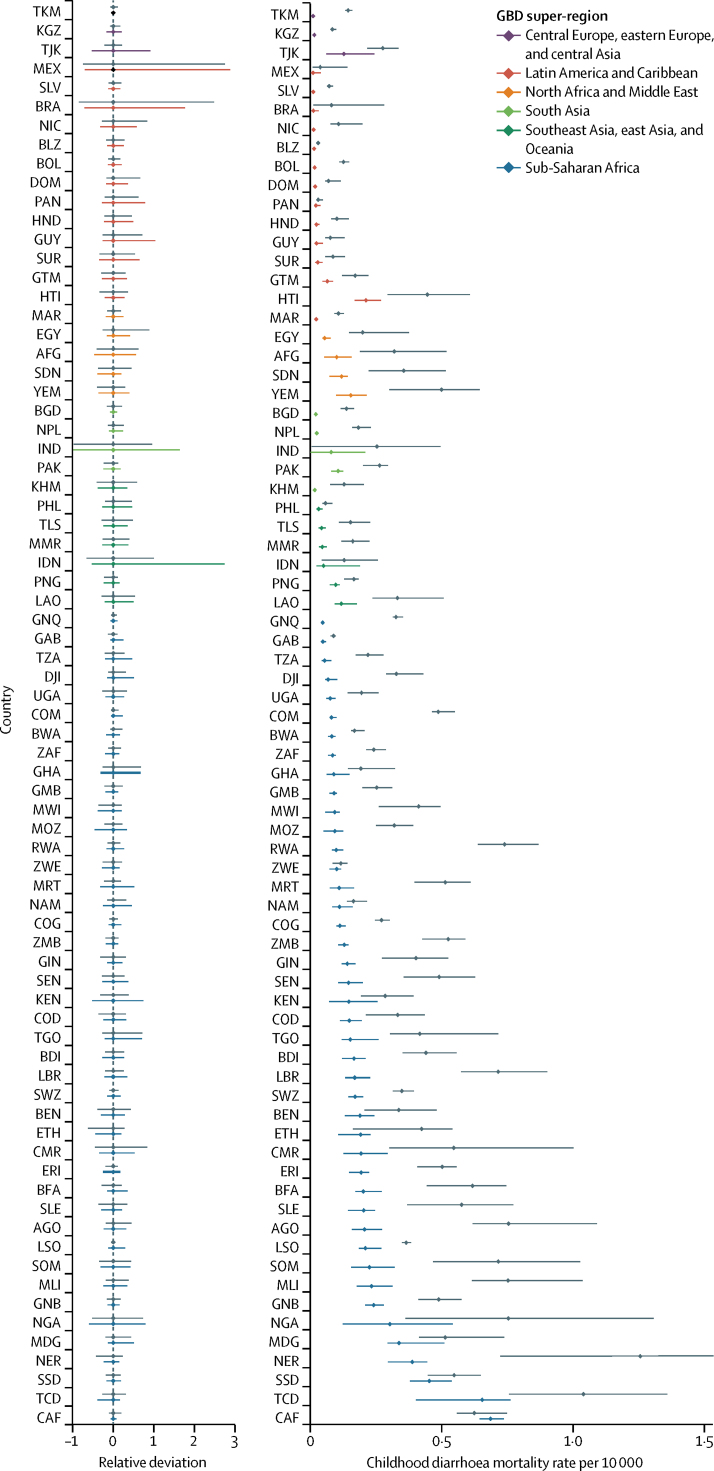
Geographical inequality of childhood diarrhoeal mortality at the second administrative-level unit The left panel shows the range of relative deviation from the country mean diarrhoea mortality rate for each country in 2000 (upper bar) and 2017 (lower bar, coloured by GBD super-region). Each bar represents the range from the lowest to highest second administrative-level unit deviation for each country. The right panel shows LMICs with at least one death from diarrhoea per 10 000 children at the second administrative-level unit ranked by childhood diarrhoea mortality rate in 2017. Mean mortality rates are shown as dark grey dots and are national-level aggregations that correspond to the results shown in figure 3. Each bar represents the range from the lowest to highest second administrative-level unit childhood diarrhoeal mortality rate for each country in 2000 (upper bar) and 2017 (lower bar, coloured by GBD super-region). Country names in both panels are the designated ISO 3166-1 alpha-3 codes. GBD=Global Burden of Diseases, Injuries, and Risk Factors Study. LMICs=low-income and middle-income countries.

Within analyses of geographical inequality, focusing on maximum deviations from the country mean can mask subnational variation in space and time. Two exemplars of this masking are Indonesia (where units with the greatest deviation changed over time) and Peru (where the shape of the distribution of inequality changed even though the maximum deviation remained mostly stable; figure 3). In 2000, the units within Indonesia farthest from the mean were all within the first administrative-level units (provinces) of modern-day Gorontalo, Nusa Tenggara Barat, Sulawesi Barat, and Sulawesi Tengah, with the largest relative deviation in the East Lombok Regency in Nusa Tenggara Barat (101·4% the national mortality rate; Figure 3, Figure 4). By 2017, units in Papua were almost four times the Indonesian national average (Figure 3, Figure 4). Units in Papua went from not ranking in the 60 units with the highest deviation in Indonesia in 2000 to having the 29 units with the highest deviations from the country average in 2017.

In Peru, several units had substantial deviations from the national average in 2000. The maximum relative geographical inequality occurred in Requena province, Loreto, with 0·80 (95% UI 0·65–0·97) deaths per 1000, versus a country mortality rate of 0·4 (0·4–0·5) deaths per 1000 children, a relative deviation of 83·7%. Since 2000, Peru has seen substantial reductions in diarrhoeal mortality, and yet, in 2017, mortality in Requena province, Loreto, was 56·8% higher than the country average. Although the maximum relative deviation increased over this period, the distribution of inequality shows a different pattern. In 2000, 58 of 196 provinces in Peru had mortality rates at least 20% higher than the country average (figure 3C); however, in 2017, only 34 provinces had mortality rates at least 20% higher than the country average (figure 3D).

### Drivers of geographical inequality in diarrhoeal mortality

A risk factor can drive the risk of diarrhoeal mortality by increasing the chance that either a child is infected, infection develops into a disease episode, or an episode results in death. Both CGF and WASH risk factors were used as covariates in the diarrhoea prevalence model because they are predictive of infections that lead to diarrhoea.^7^, ^22^ Conversely, ORS coverage was not used because there is clinical evidence that ORS prevents mortality from diarrhoea,^11^, ^12^ but there is no evidence that it affects diarrhoea prevalence or incidence. Because of the possibility for circularity, post-hoc correlative analyses between the subnational variation in diarrhoeal mortality and the subnational variation in CGF and WASH must be interpreted carefully. However, consistent with the logic of previous risk factor analysis,^3^ excluding these known drivers of diarrhoea incidence would diminish the fit and usefulness of the output more than the potential loss of interpretation due to circularity. It is important to note that by using both stacked generalisation and the Gaussian process, which incorporates estimates of spatial and temporal autocorrelation, diarrhoeal mortality patterns are not a simple direct function of the risk factors used. Most importantly, the counterfactual analysis is based on externally derived risk ratios for each level of each risk factor.

**Figure 5:**
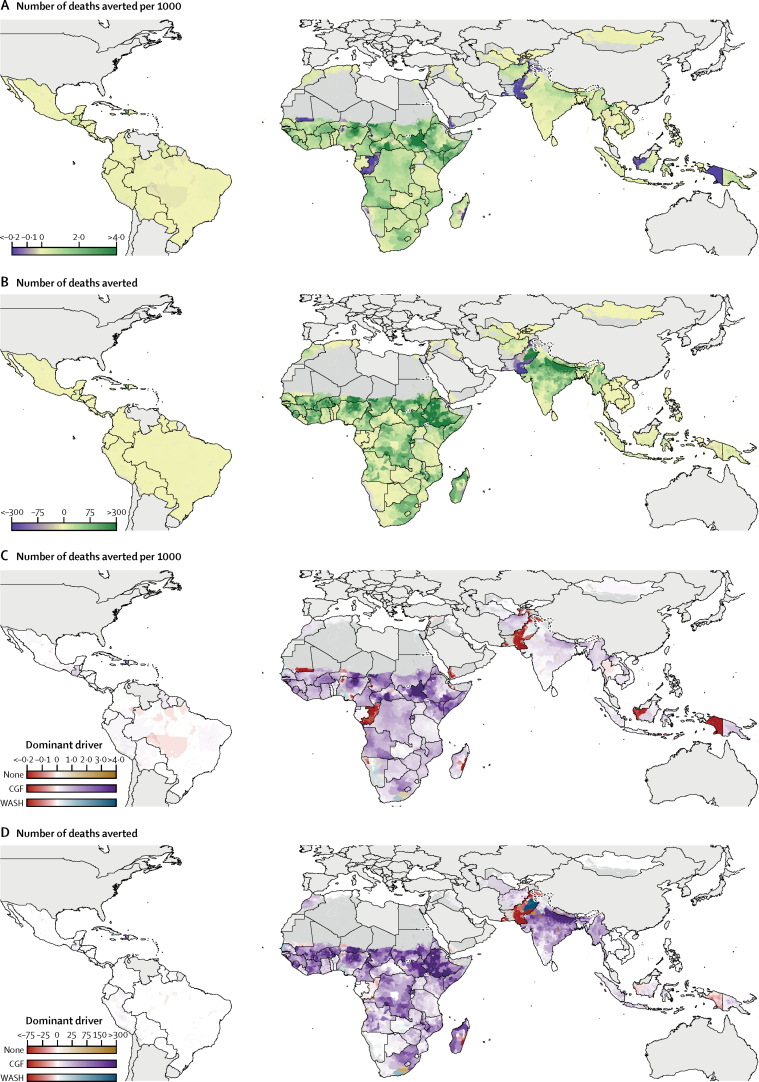
Averted diarrhoeal deaths in 2017 attributable to improvements in key risk factors implemented from 2000 to 2017 Number of deaths averted per 1000 children (A). Number of total deaths averted (B). Number of deaths averted per 1000 children with colour scale driven by dominant driver (C). Number of total deaths averted with colour scale driven by dominant driver (D). The risk factor contributing most of the reduction is indicated as either WASH (blue), CGF (purple), and none (gold), in which none represents locations where neither risk factor is dominant. Maps reflect administrative boundaries, land cover, lakes, and population; grey-coloured grid cells were classified as barren or sparsely vegetated and had fewer than ten people per 1 × 1 km grid cell, or were not included in these analyses.^33^, ^34^, ^35^, ^36^, ^37^, ^38^ WASH water, sanitation, and hygiene. CGF=child growth failure.

To assess drivers of the temporal trends in diarrhoeal mortality, we did a counterfactual analysis by comparing the estimated number of diarrhoeal deaths in 2017 to the scenario in which these risk factors had been at their 2000 levels. For the primary counterfactual analysis, we did not include ORS because only a few studies have quantified ORS efficacy precisely and, thus, there is no universally accepted risk ratio for its efficacy. A counterfactual analysis that includes ORS is presented in appendix 1 (pp 61–62). Additional factors that affect death rates and counts, such as changes in population structure and size and sociodemographic factors, were kept at their 2017 levels. Reductions in CGF averted 245 000 deaths, and 46 000 deaths were averted by improvements in WASH (figure 5D). In units where one or both risk factor groups improved from 2000 to 2017, we estimated 297 000 deaths were averted because of combined changes in WASH and CGF risk factors (figure 5B). The largest attributable relative reductions in diarrhoeal mortality in units where at least one child was estimated to have died from diarrhoea in 2017 were seen in India, Myanmar, Rwanda, and Somalia, where gains were mainly attributable to concurrent reductions in CGF (figures 5A, C). Conversely, the largest absolute attributable reductions in diarrhoeal mortality were in Ethiopia, India, Niger, and Pakistan. In Lahore, within the Punjab province of Pakistan, these gains were almost entirely due to improvements in WASH, whereas in the units within Ethiopia, India, and Niger, the averted deaths were almost entirely due to reductions in CGF (figures 5B, D). Although many regions that saw deaths averted because of WASH also had improvements associated with CGF, there were regions in Angola and Pakistan where the reduction in diarrhoea-related mortality was mainly driven by WASH (figure 5C). In 2000, across all LMIC units, 68·0–99·2% of childhood diarrhoeal deaths were attributable to either CGF or WASH risk factors. In 2017, the range increased slightly to 60·1–99·0% (appendix 1 pp 61–62).

**Figure 6:**
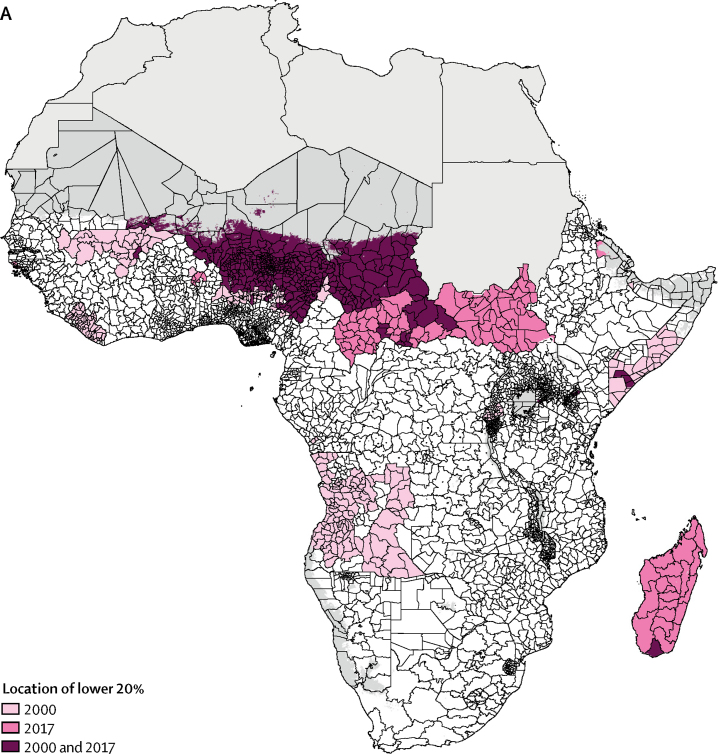

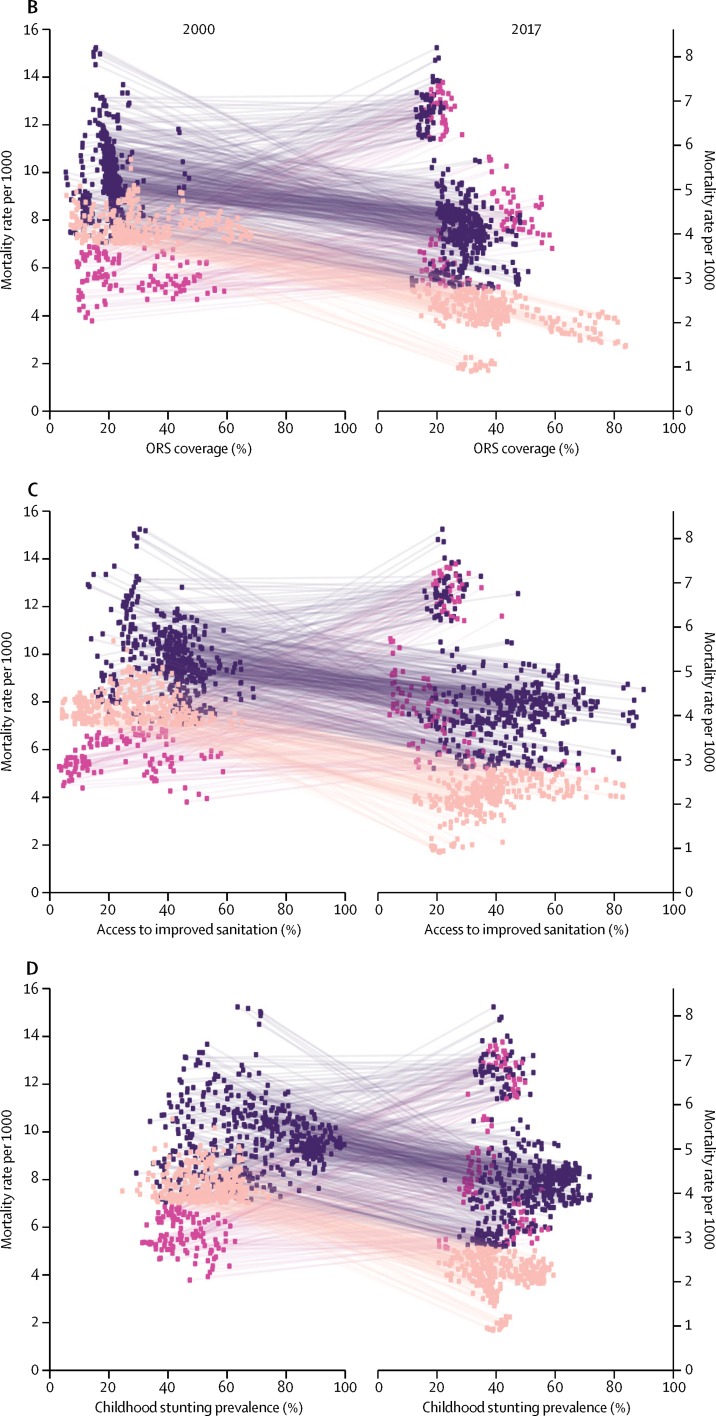
Second administrative-level units in sub-Saharan Africa with childhood mortality rates in the lower 20% Second administrative units are coloured according to where children are most likely to die of diarrhoea, or the lower 20% (A). Scatter plots of mortality rates against ORS coverage (B),^21^ access to improved sanitation (C), and childhood stunting prevalence (D). The left axes are based on 2000 values whereas the right axes are based on 2017 values. The scale change in the y axis is due to substantial decline in mortality rates across most of sub-Saharan Africa. Because lower 20% is itself a relative distinction, scales are adjusted accordingly. Maps reflect administrative boundaries, land cover, lakes, and population; grey-coloured grid cells were classified as barren or sparsely vegetated and had fewer than ten people per 1 × 1 km grid cell, or were not included in these analyses.^33^, ^34^, ^35^, ^36^, ^37^, ^38^ ORS=oral rehydration solution.

Compared with other modelled regions, much of sub-Saharan Africa had a disproportionally high burden of diarrhoeal disease. Inequality, as measured by the Gini coefficient across units within sub-Saharan Africa, remained mostly constant in sub-Saharan Africa from 2000 (0·30) to 2017 (0·33). We identified units with child populations at highest risk of death due to diarrhoea, defined as units with 20% of the population in Africa living in areas with the highest mortality rates (figure 6A). No combination of risk factors that drove high diarrhoeal mortality was discernible; however, units had at least one risk factor at a high level (figures 6B–D). Of 565 units accounting for 20% of children with the highest diarrhoeal mortality risk in 2017, 447 were also among those with the highest risk in 2000. The other 118 units that became relatively worse from 2000 to 2017 were predominantly in South Sudan (n=45), Central African Republic (n=39), and Madagascar (n=21). In units in South Sudan, although ORS decreased slightly on average (2·7%), there was a notable decline in average prevalence of childhood stunting across the 45 units (9·6%; figure 6D). As with high-burden areas in 2017, the risk factors that correlated with improvements from 2000 to 2017 were varied. For example, of the 295 units that transitioned out of the lower 20% from 2000 to 2017, 53 came from Liberia. In these units, surprisingly, both ORS coverage and access to improved sanitation declined on average from 2000 to 2017 (average ORS coverage declined by 14·1% and average access to improved sanitation declined by 11·7%; figures 6B, C). Conversely, and more consistent with the improvements in these units of Liberia, childhood stunting consistently improved from 2000 to 2017 (childhood stunting decreases ranged from 14·4% to 25·4%; figure 6D).

## Discussion

Over the past 18 years, substantial reductions have been noted in diarrhoeal mortality, but these improvements have not been recorded uniformly across LMICs. Although only 112 (0·5%) of 24 143 units had increases in mortality rates from 2000 to 2017, 5729 (24%) units saw an increase in incidence of childhood diarrhoea over this period. While some units with high diarrhoeal burden in 2000 have subsequently noted impressive reductions, other units with historically high diarrhoeal burden have seen some of the most meagre improvements. Globally, most of the diarrhoeal burden is in sub-Saharan Africa and south Asia, but we recorded substantial variation within countries in these subcontinents. Moreover, even in regions with relatively low diarrhoeal burden, we identified units that far exceeded their respective country's averages. Our estimates identified the units of each country where diarrhoeal burden was disproportionally high, pinpointing the locations most in need of targeted interventions.

Identifying a country's worst-performing units also leads to awareness of the extent of geographical inequality, measured by the range of relative deviation from the mean. It likewise pinpoints if these units are left behind consistently over time. In Peru, some metrics of geographical inequality seem to be mostly consistent from 2000 to 2017. However, deeper analysis into the distribution of burden across the country showed that more than half of its worst-performing units substantially improved relative to others in the country. Only a few Peruvian units east of the Andes seem to be left behind. Conversely, in Indonesia, the worst-performing units in 2000 actually improved more than average, whereas units in Papua became substantially worse relative to the rest of the country, leading to units exceeding the country average by almost 350%.

The different subnational patterns that emerge between relative and absolute deviations are echoed when comparing units with the highest mortality rates versus those units where most children die from diarrhoea. Across all LMICs, even though units with the highest mortality risk were all in sub-Saharan Africa, the five units where most children died were all in Pakistan. These same patterns hold within many countries. In the Democratic Republic of the Congo, most deaths from diarrhoea occurred in the capital city of Kinshasa, where the death rate was 1·5 (95% UI 1·3–1·9) deaths per 1000 children; however, the second administrative-level unit with the highest death rate (Kazumba, Kasaï; 2·0 [1·6–2·4] deaths per 1000 children) had an estimated 307 (251–368) childhood deaths in 2017 because of its small population size (figure 2). When attempting to further reduce diarrhoeal burden in a country or region, interventionists, policy makers, and other stakeholders must consider and balance the needs of both locations with the highest risk and locations with the highest burden.

Changes in diarrhoeal burden are due to myriad related drivers, but findings of a study^3^ showed that CGF and poor access to improved WASH were most associated with global reductions in the burden of diarrhoea. Although there are other important risk factors for diarrhoea (eg, poor rotavirus vaccine coverage), we did a counterfactual CGF and WASH risk factor analysis. Using newly available subnational estimates, we have provided a deeper understanding of the drivers of past success and location-specific needs to prevent future deaths. Large portions of sub-Saharan Africa have seen improvements because of reductions in CGF. Likewise, reductions in diarrhoeal deaths in Ethiopia have coincided with improvements in access to better sanitation. We identified second administrative-level units of Ethiopia, India, Niger, and Pakistan where reductions in CGF and WASH risk factors since 2000 have averted more than 1000 childhood deaths due to diarrhoea. Some of the regions that have seen the slowest improvements can also be linked to risk factors. In much of Pakistan, for example, small improvements in WASH have been overwhelmed by increases in CGF (figure 5). Although it is unlikely that risk factors will be eliminated completely, and thus counting all deaths still attributable to a risk factor is slightly misleading, we did identify patterns relating disproportionately high values of risk factors with disproportionally high burden. In sub-Saharan Africa, no combination of risk factors was found that needed reduction across the region; rather, in different locations of high burden, a different suite of risk factors seemed to be associated with the high risk of death due to diarrhoea (figure 6).

In the future, our analysis could aid in targeting of site-specific interventions, for example, to units of India, Indonesia, and Nigeria that did worse than their respective country average and had higher than country-average levels of childhood stunting. Although nationwide campaigns to reduce childhood stunting have a role in averting further unnecessary deaths, focused interventions in the worst-performing units might reduce the recorded substantial geographical inequality in diarrhoeal burden. Our results did not always indicate that every unit needing improvements required reductions in all risk factors, even within one country. As an example, although most poorly performing units within Nigeria had lower than average access to improved sanitation and ORS coverage, almost 10% of children in poorly performing units lived in locations estimated to have better than average sanitation and ORS coverage. Careful consideration of location-specific risk factors is necessary to optimally design intervention programmes.

Limitations associated with our analysis include inherent biases in survey data, which are associated with data obtained with recall biases. There is also uneven data coverage in space and time, in particular from zones of conflict and political instability (eg, Afghanistan, Iraq, Pakistan, Syria, and Yemen). Regarding the geospatial modelling framework, our approach is designed to optimise out-of-sample predictive validity and, as such, it is difficult to do inferential analyses. Our spatial and temporal autocorrelation assumptions might smooth over focal epidemics. Additionally, our model does not distinguish differences in rates of disease or death by causes of diarrhoea because we are currently unable to fully model all causes of diarrhoea. For this study, we assume that the case-fatality rate is constant for any particular year within any particular country. This assumption is unlikely, but since it is more likely that the places with higher than average prevalence are likely to be the same places with a higher than average case-fatality rate, our observations about subnational inequality in diarrhoea mortality probably underestimate these quantities. As previously mentioned, the risk factor analyses must be interpreted with care. CGF and WASH risk factors are used as covariates within the diarrhoea model, so it is unsurprising that the final diarrhoeal burden estimates correlate with those covariates. On the other hand, because of both the spatiotemporal smoothing that occurs through the Gaussian process and the stacked generalisation beforehand, it is not necessary for the final output to correspond with the covariates used in the regression. Although ORS was not used in the prevalence model, many of the base covariates used in diarrhoea (eg, elevation or population density) were used in the ORS model.

Our counterfactual analysis assumed that each risk factor affects diarrhoeal mortality and changes through time independently of all other risk factors. Accurately capturing and quantifying the covariation of these risk factors in space and time would further improve the use of that analysis. Our study also does not address the protective effect of breastfeeding with potential for the reduction of diarrhoeal burden.^39^ Breastfeeding can account for some of the lower rates of reduction in diarrhoea incidence and would be useful to investigate in future studies. Diarrhoea is a common symptom triggered by different causes and, to further focus preventive health-care strategies, a more in-depth analysis of diarrhoea causes should be done in future studies. Finally, despite the availability of vaccines to rotavirus, which is the leading cause of diarrhoea, we did not include coverage of this vaccine in our risk factor analysis because subnational estimates of rotavirus vaccine coverage are not yet available for all LMICs.

Because geospatial information is available for some causes of diarrhoea, estimating the subnational variation in those pathogens would help the interpretations and recommendations resultant from this work. Our current modelling framework aggregates ages to all children younger than 5 years but, in view of the strong relation between the case-fatality ratio and age, an age-specific model would be more informative. Our current framework prioritises prediction over inference. There is an increased need in building inferential models that can be used to infer the effect of interventions. Finally, our model assumes that every child within a population is equally likely to become infected and, on infection, is equally likely to develop disease or die. It does not address the vicious cycle of repeated enteric infections in the same individual that causes more severe symptoms. Incorporating these dynamics into our modelling framework can improve accurate accounting of the long-term burden of diarrhoea and quantification of those who are most vulnerable.

Every year, more than half a million children in LMICs die from diarrhoea; however, with treatment, most of these deaths can be averted. Our results serve as a new tool to pinpoint where these deaths occur. By establishment of good health practices from birth, children can be protected from enteric infections resulting in serious diarrhoeal episodes. Finally, by ensuring access to healthy environments, exposure to enteric pathogens can be prevented. Optimising reduction of diarrhoeal burden can be achieved by focusing on locations with the highest risk or those with the highest burden; either way, a detailed understanding of diarrhoeal morbidity and mortality, in addition to risk factors that drive diarrhoea, is necessary at the spatial scale at which policy is implemented. This work provides the data necessary to formulate effective policies and precision public health programmes to ultimately stop the preventable loss of so many young lives.

Correspondence to: Dr Robert C Reiner Jr, Institute for Health Metrics and Evaluation, Department of Health Metrics Sciences, School of Medicine, University of Washington, Seattle, WA 98121, USA bcreiner@uw.edu

**This online publication has been corrected. The corrected version first appeared at thelancet.com on June 4, 2020, and further corrections have been made on July 23, 2020**

## Data sharing

The source code and data used to generate estimates are available online. The full sets of estimates at all geographical levels produced can be found online.
