# Department of Error

**DOI:** 10.1016/S0140-6736(20)31569-5

**Published:** 2020-07-25

*Local Burden of Disease Diarrhoea Collaborators. Mapping geographical inequalities in childhood diarrhoeal morbidity and mortality in low-income and middle-income countries, 2000–17: analysis for the Global Burden of Disease Study 2017.* Lancet *2020; **395:** 1779–801—*In this Article, Lorenzo Monasta was added to the author list as shown: “… Ali H Mokdad, Lorenzo Monasta, Yoshan Moodley, …”. The respective affiliation section has been amended. These changes have been made to the online version as of July 23, 2020.

