# Supplementary material for: Mapping geographical inequalities in childhood diarrhoeal morbidity and mortality in low-income and middle-income countries, 2000–17: analysis for the Global Burden of Disease Study 2017
Source: Lancet. 2020 Jun 6;395(10239):1779–801. doi: 10.1016/S0140-6736(20)30114-8 (PMC7314599; doi:10.1016/S0140-6736(20)30114-8)
Supplement: Supplementary appendix 1 [file mmc1.pdf]

THE LANCET

Supplementary appendix

This appendix formed part of the original submission and has been peer reviewed. We post it as supplied by the authors.

Supplement to: Reiner Jr RC, Hay SI, on behalf of the Local Burden of Disease Diarrhoea Collaborators. Mapping geographical inequalities in childhood diarrhoeal morbidity and mortality in low-income and middle-income countries, 2000–17: analysis for the Global Burden of Disease Study 2017. *Lancet* 2020; published online May 6. https://doi.org/10.1016/S0140-6736(20)30114-8.

Mapping geographic inequalities in childhood diarrhoeal morbidity and mortality in low- and middle-income countries, 2000–2017

Appendix

Contents

0.0 GATHER compliance 3

1.0 Case definition of modelled outputs 3

1.1 Prevalence 3

1.2 Incidence 3

1.3 Mortality 3

2.0 Data 3

2.1 Summary of included data sources 3

2.2 Standardising case definitions 4

2.3 Standardising ages 4

2.4 Standardising recall periods 5

2.5 Seasonality adjustment 5

2.6 Period prevalence to point prevalence conversion 5

2.7 Aggregation to finest possible geography 5

2.8 Creation of pseudo-points within areal units 5

2.9 Assigning covariates to points 5

2.10 Administrative boundaries 6

3.0 Geostatistical model 6

3.1 Model geographies 6

3.2 Ensemble covariate modelling via stacked generalisation 6

3.3 Geostatistical model 6

3.4 Priors 7

3.5 Mesh creation 8

3.6 Fitted parameters and estimate generation 8

4.0 Post-estimation 9

4.1 Calibration to the Global Burden of Disease (GBD) 2017 9

4.2 Conversion of point prevalence to other measures 9

5.0 Model validation **9**
5.1 Vetting stacker models and time trends 9
5.2 In-sample validation 9
5.3 Out-of-sample validation 9
6.0 Supplemental results **10**
6.1 Prevalence, incidence, and mortality due to diarrhoea 10
6.2 Annualised rate of change (AROC) in diarrhoea prevalence, incidence, and mortality 10
6.3 Relative and absolute geographic inequality 10
6.4 Mild, moderate, and severe child growth failure 10
6.5 Counterfactual analysis of deaths averted 10
6.6 GINI coefficient 12
7.0 Appendix Figures **13**
8.0 Appendix Tables **83**
9.0 Appendix References **115**
10.0 Author Contributions **117**

0.0 GATHER compliance

Please see Appendix Table 1 for discussion on how this study meets the conditions for GATHER[1] guidelines.

1.0 Case definition of modelled outputs

1.1 Prevalence

Prevalence is defined as number of children under the age of 5 who had diarrhoea, defined as three or more abnormally loose or watery stools within the previous 24 hours.[2]

1.2 Incidence

Incidence is defined as the number of cases of diarrhoea in children under the age of 5 per child per year.

1.3 Mortality

Mortality is defined as the number of deaths per year of children under 5 due to diarrhoea.

2.0 Data

2.1 Summary of included data sources

This analysis selected 94 countries based on their Socio-demographic Index (SDI) published in the Global Burden of Disease (GBD) (see Appendix Table 3). The SDI is a measure of development that combines education, fertility, and income.[3] We primarily aimed to include all countries in the middle, lower-middle, or low SDI quintiles, with several exceptions. Albania and Moldova were excluded despite middle SDI status due to geographic discontinuity with other included countries and lack of available survey data. Despite the requisite SDI quintiles for inclusion, we excluded the countries of Cape Verde, Cuba, French Guiana, Iran, Palestine, Trinidad and Tobago, and Venezuela as they had no relevant data available. We also excluded island nations with fewer than one million inhabitants, which were the nations of Fiji, Solomon Islands, Maldives, Vanuatu, Samoa, Saint Lucia, Kiribati, St. Vincent and the Grenadines, Grenada, Micronesia, Tonga, Seychelles, Dominica, Marshall Islands, and America Samoa because they typically lacked survey data and did not have sufficient geographic continuity for a geospatial analytic approach to be advantageous over a national one. We present most estimates in this study at the second administrative level (e.g., districts, counties), with the exception of estimates for Brazil, which we present at the first administrative level (e.g., provinces, states). Data from Brazil was available only at the region level (between first administrative level and national level). Therefore, we only present results for Brazil at the first administrative level, which have been calibrated to results from GBD 2017.[3]

The household surveys used to model diarrhoea prevalence can be found at http://ghdx.healthdata.org/record/ihme-data/lmic-under-5-diarrhea-incidence-prevalence-and-mortality-geospatial-estimates-2000-2017 and visualised in Appendix Figure 3. For a survey to be considered for this analysis, we required that it fit our country inclusion criteria outlined above, include geography information more granular than the national level, data collected during the time frame of 2000 to 2017, have survey sample weights if the survey was not self-weighted, and have data on whether a child had diarrhoea in the past few weeks preceding survey collection. A survey was considered self-weighted if the documentation stated that the survey was self-weighted or that random sampling was conducted. After screening 1,611 sources that were tagged for diarrhoea relevance in the Global Health Data Exchange (GHDx)[4] and met the country and time frame criteria for this analysis, 515 sources met all of the inclusion criteria and were extracted and collated for analysis.

Select data sources were excluded from the analysis for missing survey sample weights for areal data, non-geographically representative sampling, or untrustworthy data. Untrustworthy data were determined by the survey administrator or systematic review of the data, visualised in Appendix Figure 2, and further explained in Section 5.1. After systematic review, 466 sources were included in the final diarrhoea dataset.

2.2 Standardising case definitions

We used "diarrhoea" as the preferred definition of diarrhoea from our survey data as it was the most common survey definition. Of the 466 surveys included in the diarrhoea model, 64 had a non-standard diarrhoea definition (Appendix Table 8). For observations for which other definitions were available, we applied an adjustment to account for differences in case detection rates relative to the standard definition. Definitions were first classified as standard ("diarrhoea" or "diarrhoeal disease"), non-standard definition 1 (explicitly specifying symptoms characterising diarrhoea, for example: "diarrhoea is determined according to the perception of illness by the mother, or person in charge, or three liquid stools a day, or blood in the stool"), or non-standard definition 2 ("loose watery motion"). Studies with the latter definition were excluded because this definition was deemed to be potentially subject to a high false positive rate, given the characteristics of healthy infant stools. A single logistic regression model was fit to the remaining surveys (both those with standard or with non-standard definition 1), regressing diarrhoea prevalence on definition and country-level fixed effects and a natural cubic spline (R splines package; three internal knots placed at default quantiles) on calendar year, to account for broad temporal trends. Prevalence reported by non-standard surveys was then reduced by the estimated coefficient of the fixed effect for non-standard definition 1, in logit space.

2.3 Standardising ages

We used ages 0 to 5 (0 to 59 months) as the preferred age range from our survey data. Of the 466 surveys included in the diarrhoea model, 36 had a non-standard age range from 0 to under 5 years, or 0.5 to under 5 years (Appendix Table 9). Prevalence reported by surveys with non-standard age ranges were adjusted to derive corresponding estimates of under-5 prevalence using a prevalence-by-age crosswalk model. For each survey with a non-standard age range, we obtained population age distribution and diarrhoea prevalence estimates for the survey country and year from the GBD study.[5] Age distributions are available for single age-years and GBD age categories, while prevalence is available only for GBD age categories. We first made the simplifying assumption that the age distribution ($P(A)$, or probability of age A) within a study sample and within the population from which it is drawn followed the age distribution of the surveyed country as a whole, in the sample year: $P(A)_{study} = P(A)_{country}$.

GBD diarrhoea prevalence estimates were used to derive prevalence-by-age models ($P(D|A)$, or the probability of diarrhoea, D, at age A) for each survey country and year. Model estimation was accomplished by minimising the sum of absolute differences between age category-specific prevalence (population-weighted GBD estimates, spanning age 0 through age 15) and prevalence estimated by integration across ages 0–15 using proposed age-by-prevalence curves. These proposed curves consisted of linear interpolations between population-weighted midpoints of each GBD age category, with prevalence at these midpoints estimated via iterative optimisation. (Prevalence curves were estimated out to age 15, despite ultimate interest only in ages 0–5, in order to improve estimates of prevalence at age 5.)

We next assumed that the prevalence-by-age relationship in the study population follows the shape of the country-level relationship but may differ in magnitude by a scaling factor, α, calculated on the logit scale:

$$logit\left(P(D|A)_{study}\right) = \alpha + logit\left(P(D|A)_{country}\right)$$

From the study we have a prevalence-by-age estimate for an age range bounded by ages $A1$ inclusive and $A2$ exclusive, given as $P(D|A1 \leq A < A2)_{study}$, or prevalence of diarrhoea given that age is between $A1$ and $A2$. We similarly define a baseline (country-level) prevalence between ages $A1$ and $A2$ as $P(D|A1 \leq A < A2)_{country}$. We are interested in estimating disease prevalence within a hypothetical sample from the study population drawn from target ages 0 to 5, given as $P(D|0 \leq A < 5)_{study}$. The country-level prevalence is similarly given as $P(D|0 \leq A < 5)_{country}$.

We then calculate α, the study-level scaling factor, using the reported study-level prevalence and baseline prevalence for age range $A1$–$A2$:

$$\alpha = logit(P(D|A1 \leq A < A2)_{country}) - logit(P(D|A1 \leq A < A2)_{study})$$

Crosswalk is then performed from reported study-level prevalence for age range $A1$–$A2$ to hypothetical study-level prevalence in the target age range (0–5) as follows:

$$logit(P(D|0 \leq A < 5)_{study}) = \alpha + logit(P(D|0 \leq A < 5)_{country})$$

2.4 Standardising recall periods

We used a two-week recall period as the preferred recall period from our survey data. Of the 466 surveys included in the diarrhoea model, 64 had a non-standard recall period. Recall periods were accounted for in the point prevalence calculation, described in Section 2.6.

2.5 Seasonality adjustment

Because surveys are rarely conducted over the entire year, estimates of diarrhoea may be biased by seasonal trends. We accounted for intra-annual variation in diarrhoea prevalence by fitting a sine-cosine regression with a period of six months by region, weighted by the standard error of the data. We generated and applied a scalar per month and region based on the percent difference between the regression fit and observed diarrhoea prevalence to adjust for seasonal biases (Appendix Figures 7a–n).

2.6 Period prevalence to point prevalence conversion

Data were converted from period prevalence (e.g., "did child x have y symptoms in the last z days?") to point prevalence using the following formula:

$$Point\ Prevalence = \frac{Period\ Prevalence\ *\ Duration}{(Recall\ Period + Duration - 1)}$$

Where duration is assumed to be 4.2 days and recall period is the number of days the question asks over (e.g., 2 weeks).

2.7 Aggregation to finest possible geography

We aggregated/summarised the individual-level microdata to the finest possible spatial resolution available—preferably, a latitude and longitude pair representing the location of the survey cluster/primary sampling unit. Where point-level referencing was not available, we matched survey microdata to the smallest polygon/areal unit possible. We calculated the effective sample size for each spatial aggregation (point and polygon) via the Kish approximation considering the underlying complex survey design.[6] After aggregation, the adjustments described above (Section 2.2–2.6) and below (Section 2.8–2.9) were applied.

2.8 Creation of pseudo-points within areal units

We created pseudo-points for areal data via a population-weighted resampling process as our desired model requires data of a single geometric type (e.g., latitude/longitude point). Specifically, we randomly generated 10,000 candidate points from within each areal unit using the WorldPop total population raster as a spatial distribution weight.[7] K-means clustering was performed to aggregate candidate points into the pseudo-points used for modelling. These pseudo-points were assigned analytical weights proportional to the number of candidate points that entered into the k-means cluster. Each pseudo-point generated by this process was assigned the diarrhoea prevalence observed from the survey for that polygon.

2.9 Assigning covariates to points

We assembled a number of remotely sensed and modelled products to use as predictors. Where possible we selected covariates that were used to model the burden of diarrhoea for GBD 2017, existed at the temporal (yearly) and

spatial resolution of interest (5 × 5-km). The covariates included: access to roads, ratio of children dependents (age 0 to 14) to working adults (age 15 to 64), distance from rivers or lakes, nighttime lights^{TV} (^{TV}= time-varying covariates), elevation, population ratio of women of maternal age to children, population^{TV}, aridity^{TV}, urban or rural^{TV}, urban proportion of the location^{TV}, irrigation, number of people whose daily vitamin A needs could be met (nutrient yield), prevalence of under-5 stunting^{TV}, prevalence of under-5 wasting^{TV}, and diphtheria-tetanus-pertussis (DTP3) immunisation coverage^{TV}. We included the Healthcare Access and Quality Index,[8] the percent of population with access to improved water sources as defined by the Joint Monitoring Program, and percent of population with access to improved toilet types as defined by the Joint Monitoring Program as national-level time-varying covariates. Appendix Figure 4 displays the final selection of covariates while Appendix Table 4 lists the source information. We filtered these covariates for multi-collinearity within each modelling region (see Appendix Figure 5) using variance inflation factor (VIF)[9] analysis using a threshold of VIF < 3. Appendix Figure 4 displays the spatial patterns of the covariates and Appendix Table 4 lists the source information. Appendix Table 5a-b shows the final covariates selected for each region following VIF analysis. Once assembled, we conducted a spatial query to match covariate values spatially and temporally to our collection of points and pseudo points. For numerical stability, all covariates were centred and scaled to mean 0, with a standard deviation of 1.

2.10 Administrative boundaries

For this analysis we use shapefiles from the Database of Global Administrative Areas (GADM) to define country boundaries and the relevant subnational/administrative divisions.[10] Slight adjustments to ensure proper nesting of administrative units were made, and larger adjustments were made in the Democratic Republic of the Congo and India where collaborators in these countries indicated mistakes in administrative boundaries in the shapefiles.

3.0 Geostatistical model

3.1 Model geographies

We stratified our data and analyses into 14 contiguous regions selected to align with the GBD 2010 study.[11] This was done to improve computational tractability and to take advantage of the a priori grouping based on country-level epidemiological profiles. Appendix Figure 5 shows the configuration of the regions. India was removed from these regions and modelled separately, given the distinct spatial and temporal patterns that the diarrhoea prevalence data exhibited here.

3.2 Ensemble covariate modelling via stacked generalisation

We used a stacked generalisation ensemble model framework to capture non-linear effects and complex interactions among our covariates.[12] For each region (Section 3.1), we fit three child models to our dataset: a generalised additive model (GAM), a penalised regression with the elastic net penalty, and a boosted regression tree (BRT). As described below in Section 3.3, we use a spatio-temporal Gaussian process regression as the parent ensembler.

Parameters for the GAM model (spline type and number of knots) was selected by expert prior with a maximum of 4 knots and the lambda parameter for the elastic net regression was selected by cross validation. Initial hyperparameters for the BRT (namely tree complexity, learning rate, and number of trees) were selected using non-parametric Bayesian optimisation over a finite space, where the objective function was the negative mean absolute error of the BRT fit.[13] See Appendix Table 6 for all hyperparameters that were selected by modelling region.[13]

Each child model was fit using five-fold cross validation to reduce overfitting and the out-of-sample predictions across the child model hold outs were compiled into a single set of model predictions. Additionally, each child model was fit on 100% of the data and a full set of in-sample predictions were created. The out-of-sample predictions per child model were fed to the parent geostatistical model (see below) as covariates for fitting while the in-sample predictions from the child models are used during the parent model's predict step.

3.3 Geostatistical model

Binomial count data are modelled within a Bayesian hierarchical modelling framework using a logit link function and a spatially and temporally explicit hierarchical generalised linear regression model to estimate the point prevalence of diarrhoea in the 14 regions of LMICs. Our model was constructed as follows:

$$C_i | p_i,\ N_i \sim \text{Binomial}(p_i,\ N_i)$$

$$\text{logit}(p_i) = \beta_0 + \boldsymbol{X_i \beta} + \epsilon_{GP_i} + \epsilon_{ctry_i} + \epsilon_{study_i} + \epsilon_i$$

$$\sum \boldsymbol{\beta} = 1$$

$$\epsilon_{ctry_i} \sim \text{N}(0, \sigma^2_{ctry})$$

$$\epsilon_i \sim \text{N}(0, \sigma^2_{nug})$$

$$\boldsymbol{\epsilon_{GP}} | \boldsymbol{\Sigma}_{\text{space}}, \boldsymbol{\Sigma}_{time} \sim \text{GP}(0, \boldsymbol{\Sigma}_{\text{space}} \otimes \boldsymbol{\Sigma}_{time})$$

$$\boldsymbol{\Sigma}_{\text{space}} = \frac{2^{1-\nu}}{\tau \times \Gamma(\nu)} \times (\kappa \boldsymbol{D})^{\nu} \times \text{K}_{\nu}(\kappa \boldsymbol{D})$$

$$\Sigma_{time\,j,k} = \rho^{|\,t_k - t_j|}.$$

For each region, we modelled the number of children at location-time i, among a sample size, N_i, who had diarrhoea as binomial count data, C_i. The counts, C_i, probabilities, p_i, predictions from the three child models $\boldsymbol{X_i}$, and residual terms ϵ_* are all indexed at a space-time coordinate. The term p_i represents both the annual prevalence and the annual probability that an individual child will have diarrhoea given the child resides at that particular location. The logit of annual prevalence, $\text{logit}(p_i)$, was modelled as a linear combination of the three child models, $\boldsymbol{X_i}$; a correlated spatio-temporal error term, ϵ_{GP_i}; and an independent error term, ϵ_i. Coefficients, $\boldsymbol{\beta}$, on the child models represent their respective predictive weighting in the mean logit link and are constrained to sum to one. ϵ_{ctry_i} is a country random effect, and ϵ_i, is an independent error term. ϵ_{GP}, is modelled as a three-dimensional Gaussian process in space-time centred at zero and with a covariance matrix constructed from a Kroenecker product of spatial and temporal covariance kernels. The spatial covariance, Σ_{space}, is modelled using an isotropic and stationary Matérn function,[14] and temporal covariance, Σ_{time}, as an autoregressive order 1 (AR1) function represented in the model with four equally spaced knots.

This approach leveraged the data's residual correlation structure to more accurately predict prevalence estimates for locations with no data, while also propagating the dependence in the data through to uncertainty estimates.[15] The posterior distributions were fit using computationally efficient and accurate approximations in R-INLA[16,17] (integrated nested Laplace approximation) with the stochastic partial differential equations (SPDE)[18] approximation to the Gaussian process residuals.

3.4 Priors

The following priors were used:

- $\beta_0 \sim N(\mu = 0, \sigma^2 = 1000)$,
- $\boldsymbol{\beta} \sim N(\boldsymbol{\mu}, \Sigma\ )$,
  - $\boldsymbol{\mu} = (\frac{1}{3}, \frac{1}{3}, \frac{1}{3})'$
  - $\Sigma\ = 1{,}000 * \ I_{3\,x\,3}$

- $log\left(\frac{1+\rho}{1-\rho}\right) \sim N(\mu = 2, \sigma^2 = 1/1.2^2)$,
- $\left(\frac{1}{\sigma^2_{ctry}}\right) \sim gamma(\alpha = 1, \gamma = 0.00005)$,
- $\left(\frac{1}{\sigma^2_{study}}\right) \sim gamma(\alpha = 1, \gamma = 0.00005)$,
- $\left(\frac{1}{\sigma^2_{nugget}}\right) \sim gamma(\alpha = 1, \gamma = 0.00005)$,
- $\theta_1 = \log(\tau) \sim N(\mu_{\theta_1}, \sigma^2_{\theta_1})$,
- $\theta_2 = \log(\kappa) \sim N(\mu_{\theta_2}, \sigma^2_{\theta_2})$.

We used the uncorrelated multivariate normal priors that INLA automatically determines based on the finite elements mesh for the log-transformed spatial hyperparameters κ and τ. The mean (μ) and variance (σ^2) parameters for the hyperpriors selected by INLA for the meshes in each region can be found in Appendix Table 7. In our parameterisation we represent α and γ in the *gamma* distribution as rate and shape, respectively. The starting set of hyperparameters were selected using INLA defaults, as well as a previously performed sensitivity analysis.[19] We used the uncorrelated multivariate normal priors that INLA automatically determines based on the finite elements mesh for the log-transformed spatial hyperparameters κ and τ. The mean (μ) and variance (σ^2) parameters for the hyperpriors selected by INLA for the meshes in each region can be found in Appendix Table 7. In our parameterisation we represent α and γ in the *gamma* distribution as rate and shape, respectively. The starting set of hyperparameters were selected using INLA defaults, as well as a previously performed sensitivity analysis.[19]

3.5 Mesh creation

We constructed the finite elements mesh for the stochastic partial differential equation approximation to the Gaussian process regression using a simplified polygon boundary (in which coastlines and complex boundaries were smoothed) for each of the regions within our model. This paper uses an improved mesh that is constructed on the S2 domain. This allows distance to be calculated along the sphere instead of using Euclidean distance between latitude and longitude coordinates. This mesh also generates denser vertices in data rich areas. We set the minimum triangle edge length to 25 km, the maximum triangle length to 1,000 km, with the mesh extending 500 km past the region's boundary. An example of finite elements mesh-constructed for the South sub-Saharan Africa region can be found in Appendix Figure 6.

3.6 Fitted parameters and estimate generation

Fitted parameters and hyperparameters, as well as their 95% credible intervals are shown by indicator and region in Appendix Table 7. Spatial hyperparameters (τ and κ) and their uncertainties have been transformed into the more interpretable nominal variance and range parameters. Nominal variance, approximating the variance at any single point, is calculated as $nom.var = 4\pi\kappa^2\tau^2$, and nominal range, approximating the distance before spatial correlation decays by 90%, as $range = \sqrt{8}/\kappa$.

All estimates were generated by taking 250 draws from the posterior distribution. For estimates at the 5 × 5-km grid cell level, these draws were used directly to generate estimates and uncertainty. 95% credible intervals around the mean of our estimates (Appendix Figures 8–16) were generated by taking the 2.5% and 97.5% quantiles of each of the draws, at the grid cell or administrative level.

To aggregate our results to second administrative-level units for each draw, we fractionally assigned each grid cell to any intersecting unit by examining the starting area of the grid cell and the relative areas of the resulting geometric intersections, and took population-weighted averages of grid cells assigned to each unit. To aggregate to first administrative-level units, we computed population-weighted averages of nested second administrative-level units. To aggregate to country levels, we computed population-weighted averages of nested first administrative-level units.

4.0 Post-estimation

4.1 Calibration to the Global Burden of Disease (GBD) 2017

To leverage national-level data included in GBD 2017, but outside the scope of our current geospatial modelling framework and to ensure agreement between these estimates and GBD 2017 national-level estimates, we performed a post-hoc calibration such that the population weighted mean of the 5 × 5-km estimates within a particular country-year recovers the corresponding mean estimate from the GBD.[20,21]

Specifically, for each posterior draw we calculated population-weighted grid cell aggregations to a national level and compared these country-year estimates to the GBD 2017 country-years.[20,21] We defined the raking factor to be the ratio between the GBD 2017 estimate and our current estimates. Finally, we multiplied each of our grid cells in a country-year by its associated raking factor. This ensures perfect calibration between our geospatial estimates and GBD 2017 national-level estimates, while preserving our estimated within-country geospatial and temporal variation.

4.2 Conversion of point prevalence to other measures

We converted our calibrated estimates of diarrhoea point prevalence (the output of the model + raking step) to incidence by finding translation factors for each country-year derived from the relationship estimated as part of the GBD. Finally, we converted incidence to mortality due to diarrhoea by using the country-year specific case-fatality rate estimated by GBD. As such, this translation assumes identical relative spatial patterns between diarrhoea prevalence, incidence, and mortality within a particular country-year.

5.0 Model validation

5.1 Vetting stacker models and time trends

For each intermediate model and for final models, we created line plots of our estimates for each of the stacking models and the final INLA model including uncertainty overlaid on the input data. We created and reviewed these plots for each country and for each first administrative unit. These plots allowed us to (1) identify unreasonable time trends caused by covariates in the absence of data, allowing us to remove those covariates; (2) identify outlier data caused by non-representative surveys or mistakes in data extraction; (3) identify countries with unique patterns in diarrhoea, which deserved individual country analyses; and (4) understand how the individual stacking models and final geostatistical model each contributed to the spatial and temporal estimates.

5.2 In-sample validation

We plotted our predictions vs. the observed data by modelling regions and by year at the country-level, first administrative-level, and second administrative-level aggregations (Appendix Figures 27–35). We also calculated mean error (ME, or bias), root-mean-squared-error (RMSE, which summarises total variance), and 95% coverage of our predictive intervals (the proportion of observed in-sample data that fall within our predicted 95% credible intervals). The in-sample fit statistics are shown in Appendix Table 10a–f.

5.3 Out-of-sample validation

We examined the predictive validity of our modelling strategy using five-fold out-of-sample cross-validation. Folds were created by randomly assigning entire second administrative units, stratified by region, to one of five folds. For each modelling region, we ran the entire modelling process once per fold, in addition to the full in-sample runs described above, generating a complete set of out-of-sample predictions. Using these out-of-sample predictions, we then calculated mean error (ME, or bias), root-mean-squared-error (RMSE, which summarises total variance), and 95% coverage of our predictive intervals (the proportion of observed out-of-sample data that fall within our predicted 95% credible intervals) aggregated to the spatial holdout level. Appendix Figures 36–44 show out-of-sample prediction vs. observed data. Similarly, Appendix Table 11a–f summarises out-of-sample statistics.

6.0 Supplemental results

6.1 Prevalence, incidence, and mortality due to diarrhoea

Appendix Figures 8–16 provide additional visualisation of calibrated estimates for diarrhoea incidence, prevalence, and mortality respectively.

6.2 Annualised rate of change (AROC) in diarrhoea prevalence, incidence, and mortality

We computed the AROC in diarrhoea incidence and mortality from 2000 to 2017. Appendix Figures 24 and 25 show estimates of the AROC from 2000 to 2017 including mean, upper, and lower estimates.

For each grid cell, log-transformed the posterior mean prevalence estimates from each year from 2000 to 2017, $prev_{i,yr}^{l}$, and determined the rate of change between each pair of adjacent years (beginning with yr=2001):

$$AROC_{i,yr}^{l} = prev_{i,yr}^{l} - prev_{i,yr-1}^{l}.$$

Next, we took a weighted average AROC across the study period to calculate grid-cell-level AROCs. Weight is defined as:

$$w_{yr} = \frac{(yr-2000)^{\gamma}}{\sum_{2001}^{2017}(yr-2000)^{\gamma}},$$

in which different weights can be given to years across the study period by selecting the appropriate γ. For this analysis, we chose to use empirical weighting, such that weights are proportional to the amount of data in each year by modeling region. Finally, we calculated grid-cell-level weighted-AROC:

$$AROC_{i} = \sum_{2001}^{2017} w_{yr} AROC_{i,yr}^{l}.$$

6.3 Relative and absolute geographic inequality

We also quantified geographic inequalities within countries over time as both the relative and absolute difference between diarrhoeal mortality rates in each second administrative unit and its country mean using the following formulas:

$$Absolute\ inequality = mortality_{unit} - mortality_{country}$$

$$Relative\ inequality = \frac{mortality_{unit} - mortality_{country}}{mortality_{country}}$$

6.4 Mild, moderate, and severe child growth failure

Following from the recent study estimating subnational variation in moderate and severe child growth failure across all LMICs[22], we reanalysed that data to break out the percent of the population experiencing mild, moderate, and severe growth failure. In particular, for both childhood stunting and childhood wasting we individually mapped the probability of finding a child with a height-age z-score or a weight-age z-score of: less than -3 (severe); between -2 and -3 (moderate); and between -1 and -2 (mild) (Appendix Figures 17–22). For each location-year, we normalised the estimated values to ensure the total did not exceed 100% of the population.

6.5 Counterfactual analysis of deaths averted

We performed a counterfactual analysis of the estimated total number of diarrhoeal deaths averted that were associated with changes in risk factors from 2000 to 2017.

The population attributable fractions (PAF) of differences in diarrhoeal deaths attributable to changes in risk factors were made using the following formulas:

$$PAF_O = \frac{\sum_1^i (RR_i * E_{i,17}) - 1}{\sum_1^i (RR_i * E_{i,17})}$$

$$PAF_C = \frac{\sum_1^i (RR_i * E_{i,00}) - 1}{\sum_1^i (RR_i * E_{i,00})}$$

$$Deaths\ Averted = Deaths_{O,17} * (\frac{1 - PAF_O}{1 - PAF_C} * PAF_C - PAF_O)$$

$$PAF: Population\ Attributable\ Fraction$$

$$E_{i,YY}: Exposure\ of\ level\ i\ in\ 20YY$$

$$Deaths_{O,YY}: Deaths\ observed\ in\ 20YY$$

$$RR_i: Risk\ Ratio\ associated\ with\ baseline\ to\ exposure\ level\ i$$

The estimation of WASH- and CGF-attributable diarrhoeal deaths averted as well as the total estimates of diarrhoeal deaths averted across all risk factors were made using the following formulas:

$$PAF_{O,WASH} = 1 - (1 - PAF_{O,Sanitation}) * (1 - PAF_{O,Water}) * (1 - PAF_{O,Stunting}) * (1 - PAF_{O,Wasting})$$

$$PAF_{C,WASH} = 1 - (1 - PAF_{O,Sanitation}) * (1 - PAF_{O,Water}) * (1 - PAF_{O,Stunting}) * (1 - PAF_{O,Wasting})$$

$$Deaths\ Averted_{WASH} = Deaths_{O,17} * (\frac{1 - PAF_{O,WASH}}{1 - PAF_{C,WASH}} * PAF_{C,WASH} - PAF_{O,WASH})$$

$$Rate\ averted_{WASH} = \frac{Number\ averted_{WASH}}{Total\ number\ of\ children\ under\ 5} * 1000$$

$$PAF_{O,CGF} = 1 - (1 - PAF_{O,Stunting}) * (1 - PAF_{O,Wasting})$$

$$PAF_{C,CGF} = 1 - (1 - PAF_{O,Stunting}) * (1 - PAF_{O,Wasting})$$

$$Deaths\ Averted_{CGF} = Deaths_{O,17} * (\frac{1 - PAF_{O,CGF}}{1 - PAF_{C,CGF}} * PAF_{C,CGF} - PAF_{O,CGF})$$

$$Rate\ averted_{CGF} = \frac{Number\ averted_{CGF}}{Total\ number\ of\ children\ under\ 5} * 1000$$

$$PAF_{O,Total} = 1 - (1 - PAF_{O,Sanitation}) * (1 - PAF_{O,Water}) * (1 - PAF_{O,Stunting}) * (1 - PAF_{O,Wasting})$$

$$PAF_{C,Total} = 1 - (1 - PAF_{C,Sanitation}) * (1 - PAF_{C,Water}) * (1 - PAF_{C,Stunting}) * (1 - PAF_{C,Wasting})$$

$$Deaths\ Averted_{Total} = Deaths_{O,17} * (\frac{1 - PAF_{O,Total}}{1 - PAF_{C,Total}} * PAF_{C,Total} - PAF_{O,Total})$$

$$Rate\ averted_{Total} = \frac{Number\ averted_{Total}}{Total\ number\ of\ children\ under\ 5} * 1000$$

6.6 GINI coefficient

The Gini coefficient[2] is a popular measure of inequality, originally applied to economics. For income inequality, the Gini coefficient assesses the magnitude of disparity between the richest and poorest individuals. In this context, equality corresponds to wealth uniformly distributed across the population and inequality corresponds to a small number of individuals possessing the majority of the wealth. The Gini coefficient for wealth can be calculated directly from the Lorenz curve, which sorts individuals by their income and plots cumulative percentages of individuals against their corresponding fraction of wealth. The Gini coefficient is then calculated as one minus twice the area under the Lorenz curve. An alternative formulation of the Gini coefficient calculates the relative mean absolute difference in wealth, and then observes that the Gini coefficient is half the resulting quantity. If x_i is the wealth of the ith individual (out of n individuals), the Gini coefficient, G, is given as:

$$G = \frac{\sum_{i=1}^{n} \sum_{j=1}^{n} |x_i - x_j|}{2n \sum_{i=1}^{n} x_i}$$

More recently, the Gini coefficient has been applied beyond economics to many fields of science, including population health.[24] Instead of calculating the cumulative fraction of wealth held by a fraction of the population, the cumulative burden of disease can be used for both the Lorenz curve and the Gini coefficient. For diarrhoeal mortality at the second administrative unit, for example, the creation of the Lorenz curve first sorts all units by mortality rate. The curve is generated by plotting cumulative population against cumulative mortality count and normalising both sums by their respective totals.

7.0 Appendix Figures

Appendix Figure 1. Geospatial modelling flowchart....15
Appendix Figure 2. Data inclusion and exclusion flowchart....17
Appendix Figure 3a–e. Diarrhoea data availability by type and country....19
Appendix Figure 4. Covariates....25
Appendix Figure 5. Map of modelling regions....27
Appendix Figure 6. Finite elements mesh....28
Appendix Figure 7a–n. Seasonal pattern adjustments for diarrhoea prevalence....29
Appendix Figure 8. Posterior means and 95% uncertainty intervals for diarrhoea prevalence by grid cell, 2017....43
Appendix Figure 9. Posterior means and 95% uncertainty intervals for diarrhoea incidence by grid cell, 2017....44
Appendix Figure 10. Posterior means and 95% uncertainty intervals for diarrhoeal mortality by grid cell, 2017....45
Appendix Figure 11. Posterior means and 95% uncertainty intervals for diarrhoea prevalence at the second administrative level, 2017....46
Appendix Figure 12. Posterior means and 95% uncertainty intervals for diarrhoea incidence at the second administrative level, 2017....47
Appendix Figure 13. Posterior means and 95% uncertainty intervals for diarrhoeal mortality at the second administrative level, 2017....48
Appendix Figure 14. Posterior means and 95% uncertainty intervals for diarrhoea prevalence at the first administrative level, 2017....49
Appendix Figure 15. Posterior means and 95% uncertainty intervals for diarrhoea incidence at the first administrative level, 2017....50
Appendix Figure 16. Posterior means and 95% uncertainty intervals for diarrhoeal mortality at the first administrative level, 2017....51
Appendix Figure 17. Prevalence of mild stunting in children under 5 at the second administrative level, 2017....52
Appendix Figure 18. Prevalence of moderate stunting in children under 5 at the second administrative level, 2017.53
Appendix Figure 19. Prevalence of severe stunting in children under 5 at the second administrative level, 2017....54
Appendix Figure 20. Prevalence of mild wasting in children under 5 at the second administrative level, 2017....55
Appendix Figure 21. Prevalence of moderate wasting in children under 5 at the second administrative level, 2017.56
Appendix Figure 22. Prevalence of severe wasting in children under 5 at the second administrative level, 2017....57
Appendix Figure 23. Lorenz curves of inequality for sub-Saharan Africa....58
Appendix Figure 24. Incidence rate annualised rate of change....59
Appendix Figure 25. Mortality rate annualised rate of change....60
Appendix Figure 26. Averted diarrhoeal deaths in 2017 attributable to improvements in water and sanitation, child growth failure, and oral rehydration solution implemented from 2000 to 2017....61
Appendix Figure 27. In-sample validation plot of diarrhoea by country....64
Appendix Figure 28. In-sample validation plot of diarrhoea by first administrative unit....65
Appendix Figure 29. In-sample validation plot of diarrhoea by second administrative unit....66
Appendix Figure 30. In-sample validation plot of diarrhoea by country and modelling region....67
Appendix Figure 31. In-sample validation plot of diarrhoea by first administrative unit and modelling region....68

Appendix Figure 32. In-sample validation plot of diarrhoea by second administrative unit and modelling region 69
Appendix Figure 33. In-sample validation plot of diarrhoea by country and year..70
Appendix Figure 34. In-sample validation plot of diarrhoea by first administrative unit and year 71
Appendix Figure 35. In-sample validation plot of diarrhoea by second administrative unit and year.........................72
Appendix Figure 36. Out-of-sample validation plot of diarrhoea by country 74
Appendix Figure 37. Out-of-sample validation plot of diarrhoea by first administrative unit....................................75
Appendix Figure 38. Out-of-sample validation plot of diarrhoea by second administrative unit 76
Appendix Figure 39. Out-of-sample validation plot of diarrhoea by country and modelling region 77
Appendix Figure 40. Out-of-sample validation plot of diarrhoea by first administrative unit and modelling region..78
Appendix Figure 41. Out-of-sample validation plot of diarrhoea by second administrative unit and modelling region 79
Appendix Figure 42. Out-of-sample validation plot of diarrhoea by country and year 80
Appendix Figure 43. Out-of-sample validation plot of diarrhoea by first administrative unit and year 81
Appendix Figure 44. Out-of-sample validation plot of diarrhoea by second administrative division and year...........82

Appendix Figure 1. Geospatial modelling flowchart

The geospatial modelling process consists of four sections. First (in blue), we compile all available survey data that can be referenced to a coordinate/point (e.g., survey cluster) or small polygon unit and calculate the diarrhoea prevalence at the respective level (Section 2.0). Data are then adjusted for differential age ranges, case definitions, seasonality, and differential recall periods (Section 2.2–2.5). Data matched to polygons are resampled into pseudo points using a k-means clustering algorithm (Section 2.8). Covariates are subsequently merged to the points and pseudo points via a spatial join (Section 2.9). Second (green), we use the point data and their associated covariates and a stacked generalisation ensemble model (Section 3.2). The children models, boosted regression trees, generalised additive models, and elastic net regression are fit using a 5-fold cross validation process (Section 3.3). The cross-validated predictions from each model then serve as the covariate values for the main/parent model (Spatio-temporal GPR model) (Section 3.3–3.4). The predictions from when the child models are fit on all the data (rather than 4/5ths implied by the cross validation) are then used to create posterior predictions of diarrhoea prevalence in a 5 × 5-km grid for the years 2000–2017 (Section 3.6). Third (purple and circled orange), we combine the predictions from step 2 and calibrate them such that the population weighted mean diarrhoea prevalence for a particular country-year from our model matches the GBD estimates (Section 4.1).[2,20,25] Finally (orange), we aggregate our estimates to first and second administrative units.

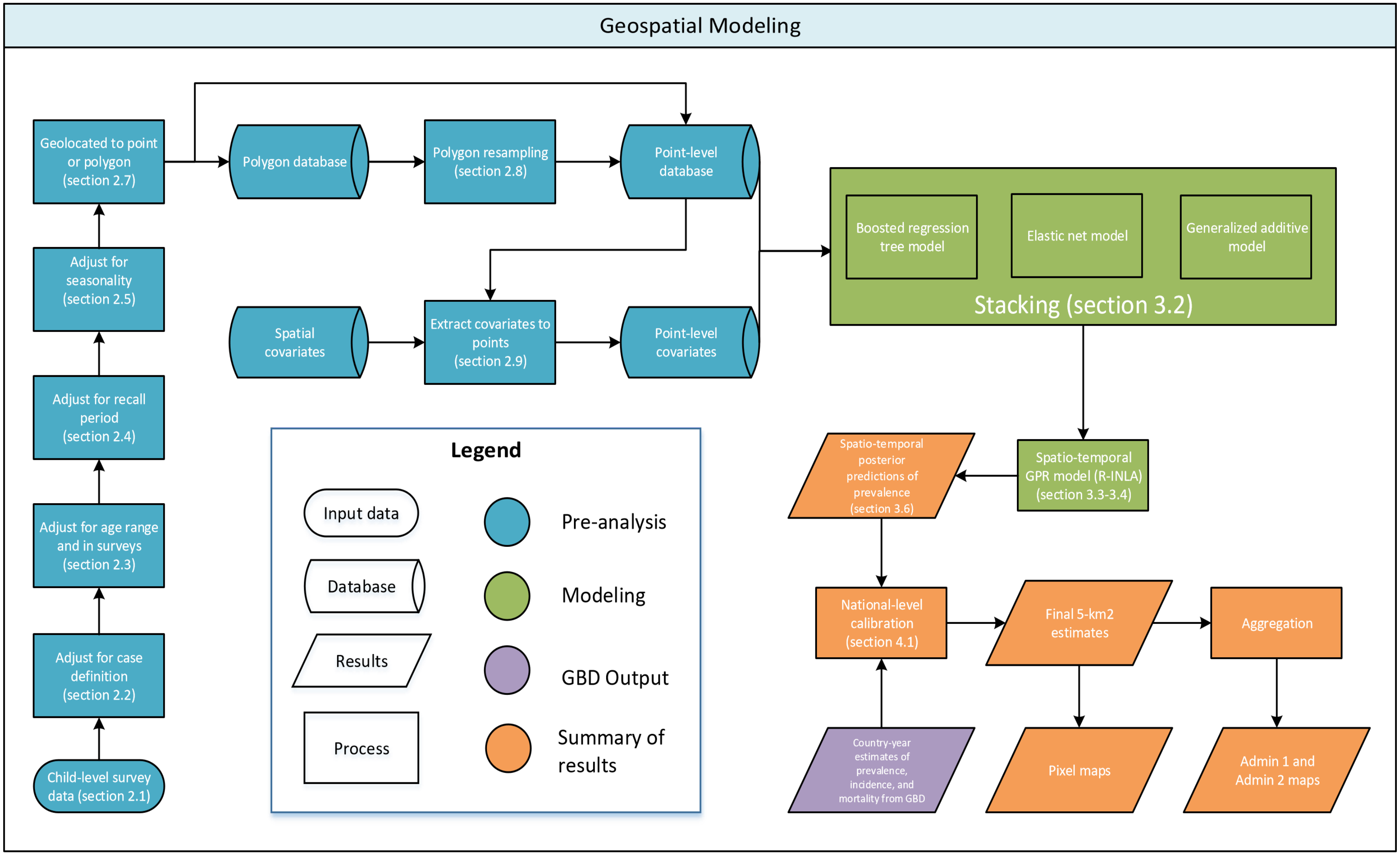
Geospatial Modeling
Geolocated to point or polygon (section 2.7)
Polygon database
Polygon resampling (section 2.8)
Point-level database
Adjust for seasonality (section 2.5)
Adjust for recall period (section 2.4)
Adjust for age range and in surveys (section 2.3)
Adjust for case definition (section 2.2)
Child-level survey data (section 2.1)
Spatial covariates
Extract covariates to points (section 2.9)
Point-level covariates
Boosted regression tree model
Elastic net model
Generalized additive model
Stacking (section 3.2)
Spatio-temporal GPR model (R-INLA) (section 3.3-3.4)
Spatio-temporal posterior predictions of prevalence (section 3.6)
National-level calibration (section 4.1)
Final 5-km2 estimates
Aggregation
Country-year estimates of prevalence, incidence, and mortality from GBD
Pixel maps
Admin 1 and Admin 2 maps
Legend
Input data
Database
Results
Process
Pre-analysis
Modeling
GBD Output
Summary of results

Appendix Figure 2. Data inclusion and exclusion flowchart

The data vetting process used in this analysis includes multiple steps. First (in green), we compiled all sources tagged as diarrhoea relevant from the GHDx and extracted data sources that met all of our inclusion criteria (Section 2.1). If a data source did not meet all of our inclusion criteria that source was excluded (in orange). Second (in light blue) the data was processed according to the geospatial modelling framework (Section 2.1–4.2, Appendix Figure 1). Third (in dark blue), line plots were created for each country and independently scrutinised for data quality over time (Section 5.1). Next, each survey that was flagged as off trend from the line plots was reviewed. If a survey was found to have a data processing mistake, the mistake would be fixed and the process would start over. Additionally, if there seemed to be a reasonable explanation for why a survey was off trend (e.g., a natural disaster that could potentially explain an uptick in diarrhoea prevalence) then that survey was included in the final data set. If a survey did not have any processing errors and there was no reasonable explanation for why it was off trend, that survey was then excluded from the final data set.

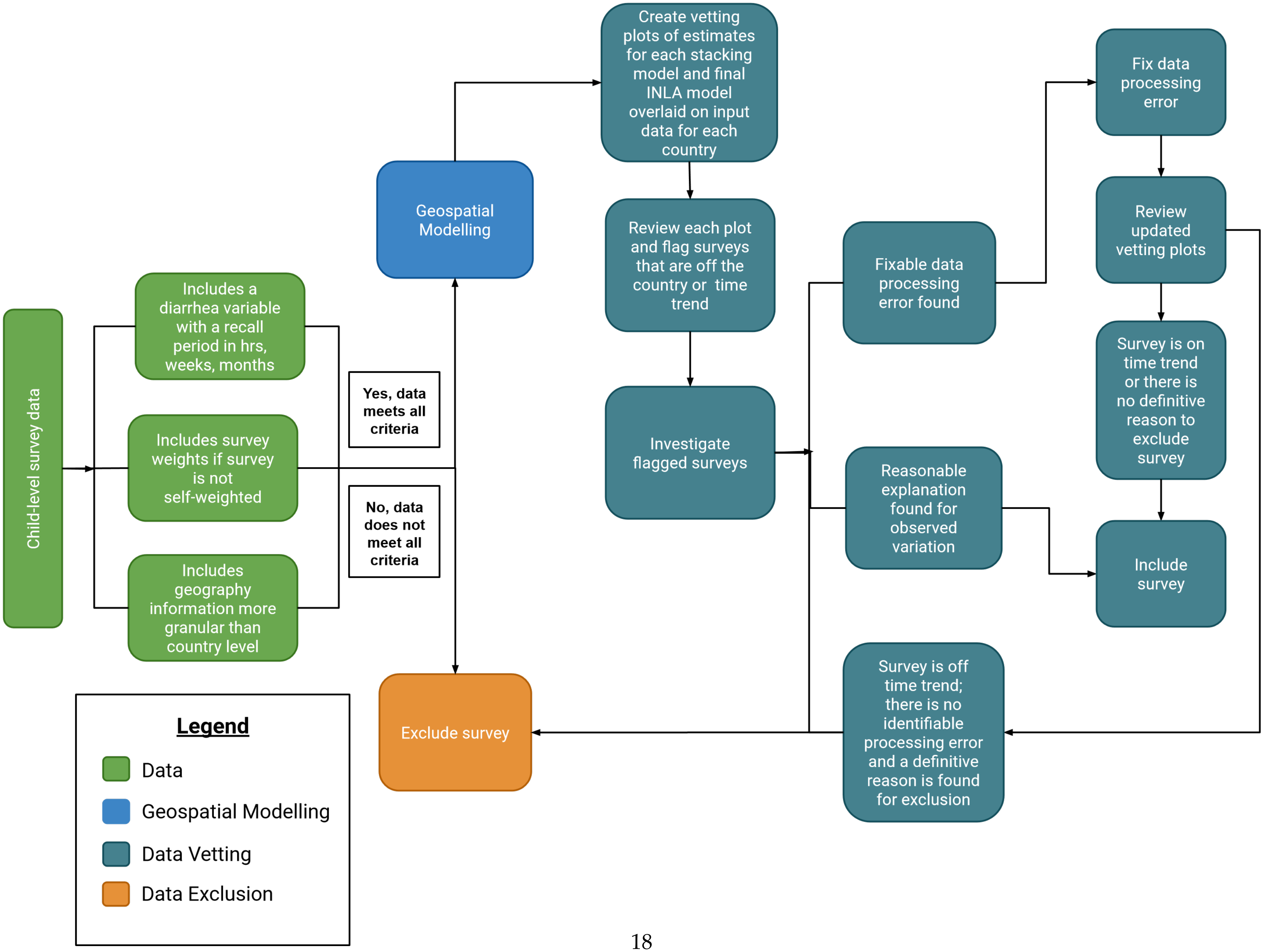
Child-level survey data
Includes a diarrhea variable with a recall period in hrs, weeks, months
Includes survey weights if survey is not self-weighted
Includes geography information more granular than country level
Yes, data meets all criteria
No, data does not meet all criteria
Geospatial Modelling
Exclude survey
Create vetting plots of estimates for each stacking model and final INLA model overlaid on input data for each country
Review each plot and flag surveys that are off the country or time trend
Investigate flagged surveys
Fixable data processing error found
Reasonable explanation found for observed variation
Survey is off time trend; there is no identifiable processing error and a definitive reason is found for exclusion
Fix data processing error
Review updated vetting plots
Survey is on time trend or there is no definitive reason to exclude survey
Include survey
Legend
Data
Geospatial Modelling
Data Vetting
Data Exclusion

Appendix Figure 3a–e. Diarrhoea data availability by type and country

All data are shown by country and year of survey and mapped at their corresponding geopositioned coordinate or area. In the left panel, the total number of points and polygons (areal) for each country are plotted by data source, type and sample size. Sample size represents the number of individual microdata records for each survey. In the right panel, mean diarrhoea prevalence for the input coordinate or area are mapped. Figure **a)** shows diarrhoea data availability in Africa by type and country from 2000–2017. Figure **b)** shows diarrhoea data availability in Latin America and the Caribbean by type and country from 2000–2017. Figure **c)** shows diarrhoea data availability in southeast Asia by type and country from 2000–2017. Figure **d)** shows diarrhoea data availability in south Asia by type and country from 2000–2017. Figure **e)** shows diarrhoea data availability in the Middle East and central Asia by type and country from 2000–2017.

a)

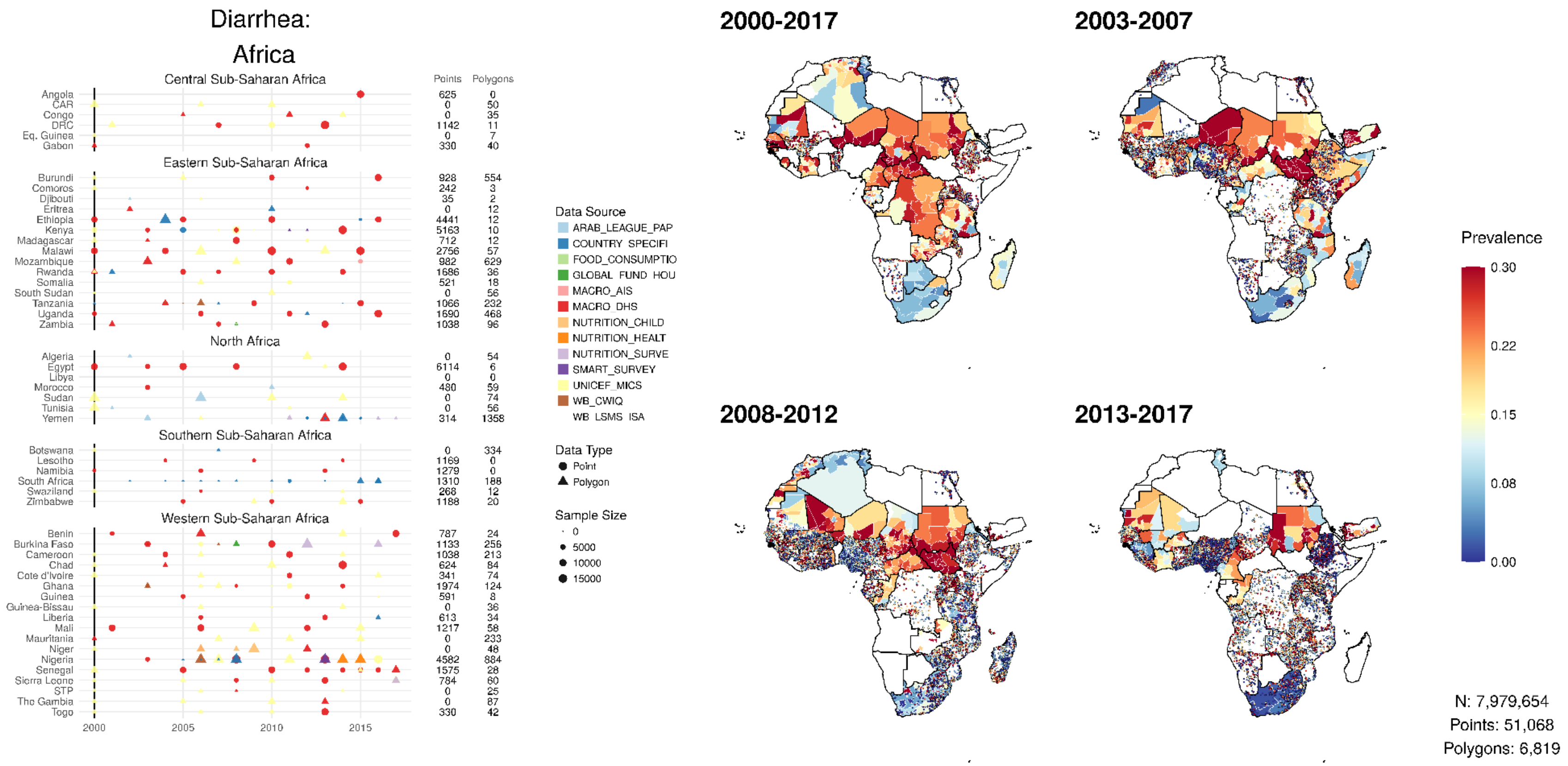

Diarrhea:
Africa
Central Sub-Saharan Africa
Eastern Sub-Saharan Africa
North Africa
Southern Sub-Saharan Africa
Western Sub-Saharan Africa
Points
Polygons
Data Source
ARAB_LEAGUE_PAP
COUNTRY_SPECIFI
FOOD_CONSUMPTIO
GLOBAL_FUND_HOU
MACRO_AIS
MACRO_DHS
NUTRITION_CHILD
NUTRITION_HEALT
NUTRITION_SURVE
SMART_SURVEY
UNICEF_MICS
WB_CWIQ
WB_LSMS_ISA
Data Type
Point
Polygon
Sample Size
0
5000
10000
15000
2000-2017
2003-2007
2008-2012
2013-2017
Prevalence
0.30
0.22
0.15
0.08
0.00
N: 7,979,654
Points: 51,068
Polygons: 6,819

b)

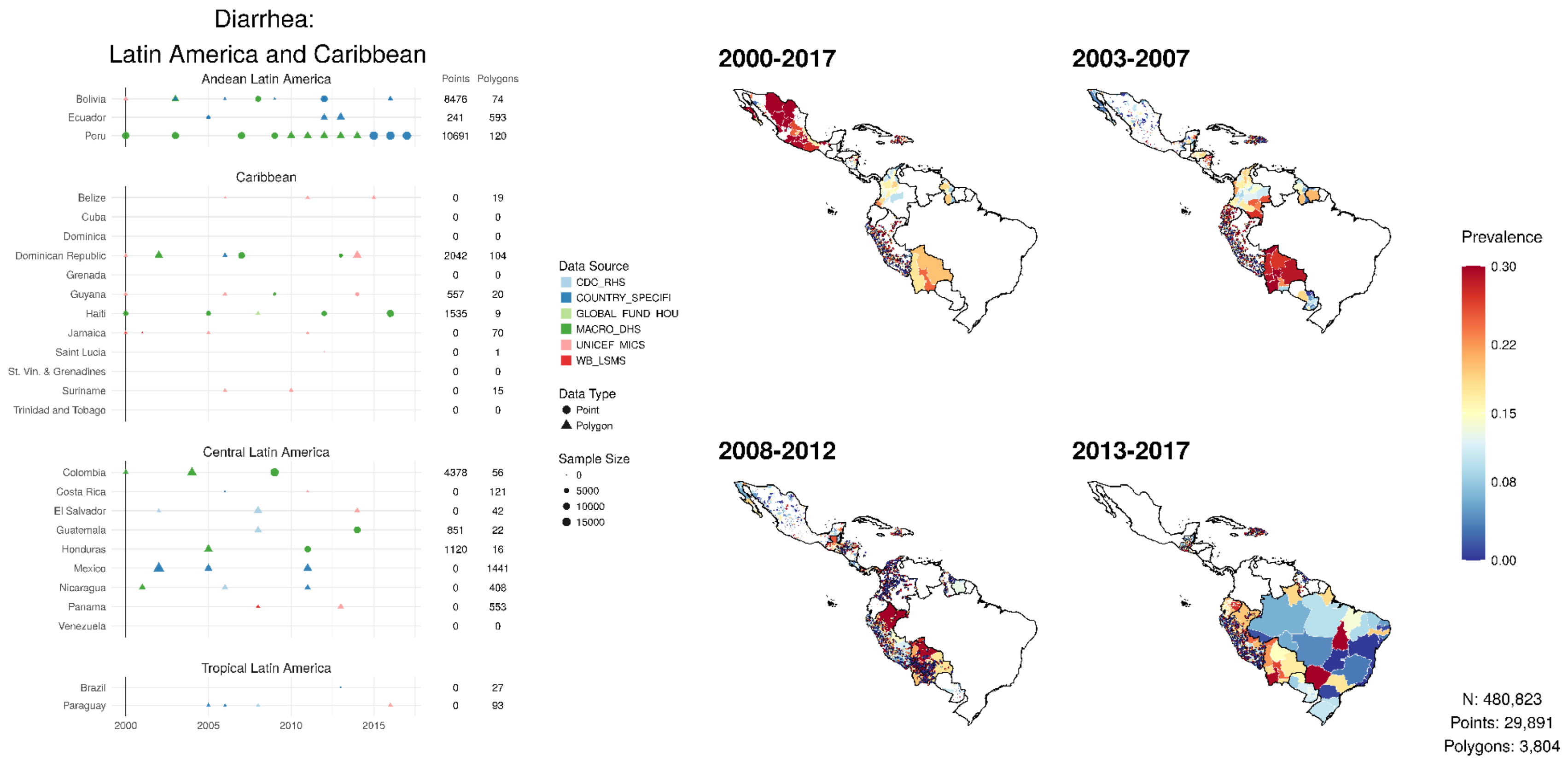

Diarrhea:
Latin America and Caribbean
Andean Latin America
Points
Polygons
Bolivia 8476 74
Ecuador 241 593
Peru 10691 120
Caribbean
Belize 0 19
Cuba 0 0
Dominica 0 0
Dominican Republic 2042 104
Grenada 0 0
Guyana 557 20
Haiti 1535 9
Jamaica 0 70
Saint Lucia 0 1
St. Vin. & Grenadines 0 0
Suriname 0 15
Trinidad and Tobago 0 0
Central Latin America
Colombia 4378 56
Costa Rica 0 121
El Salvador 0 42
Guatemala 851 22
Honduras 1120 16
Mexico 0 1441
Nicaragua 0 408
Panama 0 553
Venezuela 0 0
Tropical Latin America
Brazil 0 27
Paraguay 0 93
2000
2005
2010
2015
Data Source
CDC_RHS
COUNTRY_SPECIFI
GLOBAL_FUND_HOU
MACRO_DHS
UNICEF_MICS
WB_LSMS
Data Type
Point
Polygon
Sample Size
0
5000
10000
15000
2000-2017
2003-2007
2008-2012
2013-2017
Prevalence
0.30
0.22
0.15
0.08
0.00
N: 480,823
Points: 29,891
Polygons: 3,804

c)

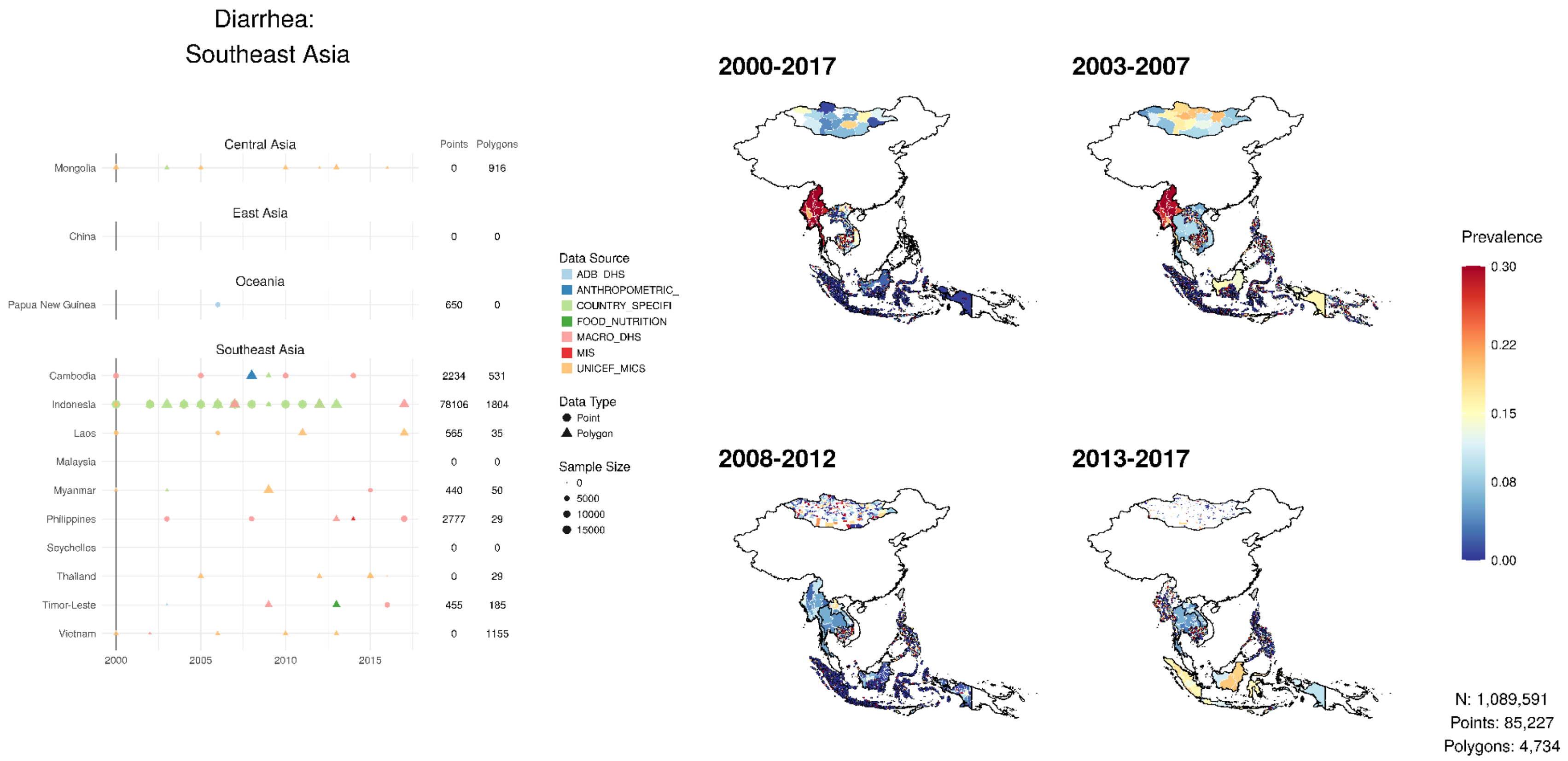
Diarrhea:
Southeast Asia
Central Asia
Points
Polygons
Mongolia
0
916
East Asia
China
0
0
Oceania
Papua New Guinea
650
0
Southeast Asia
Cambodia
2234
531
Indonesia
78106
1804
Laos
565
35
Malaysia
0
0
Myanmar
440
50
Philippines
2777
29
Seychelles
0
0
Thailand
0
29
Timor-Leste
455
185
Vietnam
0
1155
2000
2005
2010
2015
Data Source
ADB_DHS
ANTHROPOMETRIC_
COUNTRY_SPECIFI
FOOD_NUTRITION
MACRO_DHS
MIS
UNICEF_MICS
Data Type
Point
Polygon
Sample Size
0
5000
10000
15000
2000-2017
2003-2007
2008-2012
2013-2017
Prevalence
0.30
0.22
0.15
0.08
0.00
N: 1,089,591
Points: 85,227
Polygons: 4,734

d)

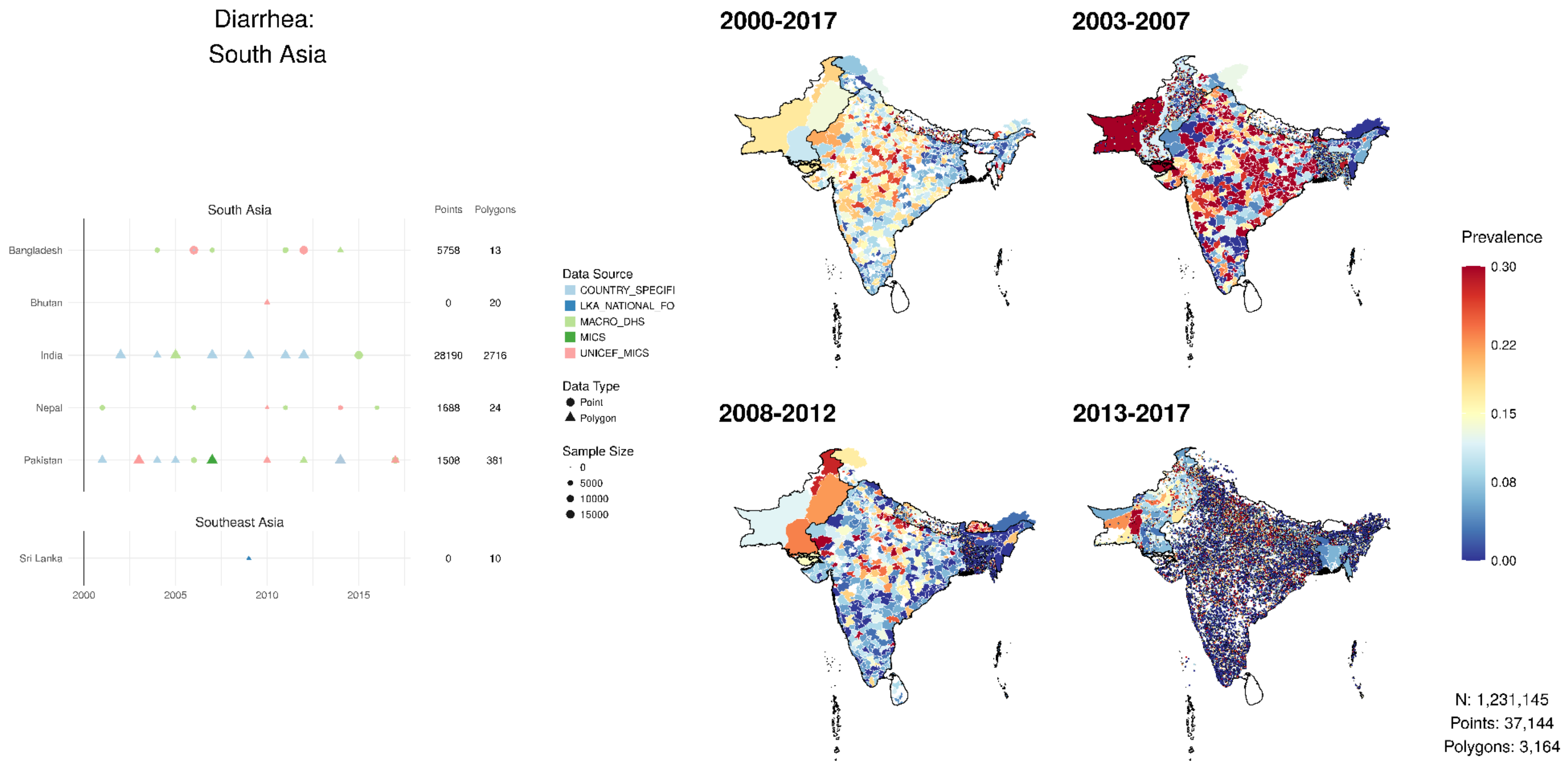
Diarrhea:
South Asia
South Asia
Points
Polygons
Bangladesh
5758
13
Bhutan
0
20
India
28190
2716
Nepal
1688
24
Pakistan
1508
381
Southeast Asia
Sri Lanka
0
10
2000
2005
2010
2015
Data Source
COUNTRY_SPECIFI
LKA_NATIONAL_FO
MACRO_DHS
MICS
UNICEF_MICS
Data Type
Point
Polygon
Sample Size
0
5000
10000
15000
2000-2017
2003-2007
2008-2012
2013-2017
Prevalence
0.30
0.22
0.15
0.08
0.00
N: 1,231,145
Points: 37,144
Polygons: 3,164

e)

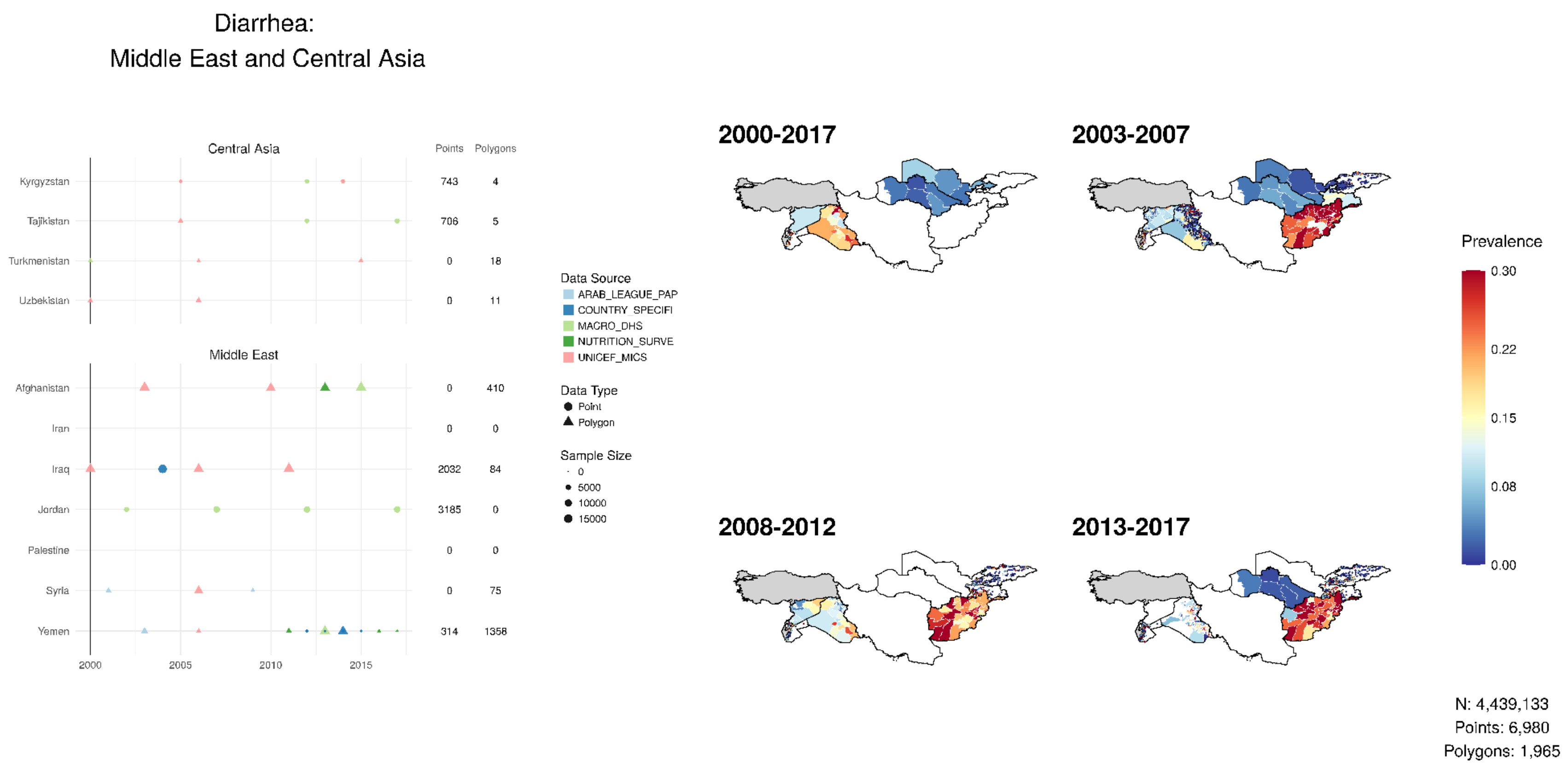
Diarrhea:
Middle East and Central Asia
Central Asia
Points
Polygons
Kyrgyzstan 743 4
Tajikistan 706 5
Turkmenistan 0 18
Uzbekistan 0 11
Middle East
Afghanistan 0 410
Iran 0 0
Iraq 2032 84
Jordan 3185 0
Palestine 0 0
Syria 0 75
Yemen 314 1358
2000
2005
2010
2015
Data Source
ARAB_LEAGUE_PAP
COUNTRY_SPECIFI
MACRO_DHS
NUTRITION_SURVE
UNICEF_MICS
Data Type
Point
Polygon
Sample Size
0
5000
10000
15000
2000-2017
2003-2007
2008-2012
2013-2017
Prevalence
0.30
0.22
0.15
0.08
0.00
N: 4,439,133
Points: 6,980
Polygons: 1,965

Appendix Figure 4. Covariates

A total of 15 covariate raster layers of possible socio-economic and environmental correlates of diarrhoea prevalence were used as inputs for the stacking modelling process. Time-varying covariates are presented for the year 2017. For the year of production of non-time-varying covariates, please refer to the individual covariate citation inAppendix Table 4.3 for additional detail. Covariates are labelled as follows: access to roads [*access2*], aridity[TV] [*aridity*], ratio of children dependents (age 0 to 14) to working adults (age 15 to 64) [*depratio*], distance from rivers or lakes [*distriverslakes*], night-time lights[TV] [*dmspntl*], diphtheria-tetanus-pertussis immunisation coverage [*dpt3_cov*], elevation [*elevation*], number of children under 5 per woman of childbearing age [*fertility*], urban or rural[TV] [ghlsurbanicity], number of people whose daily vitamin A needs could be met [*herreronyield*], irrigation [*irrigation*], urban proportion of the location[TV] [*landcover*], prevalence of under-5 stunting[TV] [*stunting_mod_b*], prevalence of under-5 wasting[TV] [*wasting_mod_b*], and population[TV] [*worldpop_raked*]. Maps reflect administrative boundaries, land cover, lakes, and population; grey-coloured grid cells were classified as “barren or sparsely vegetated” and had fewer than ten people per 1 × 1-km grid cell, or were not included in these analyses.[26–31]

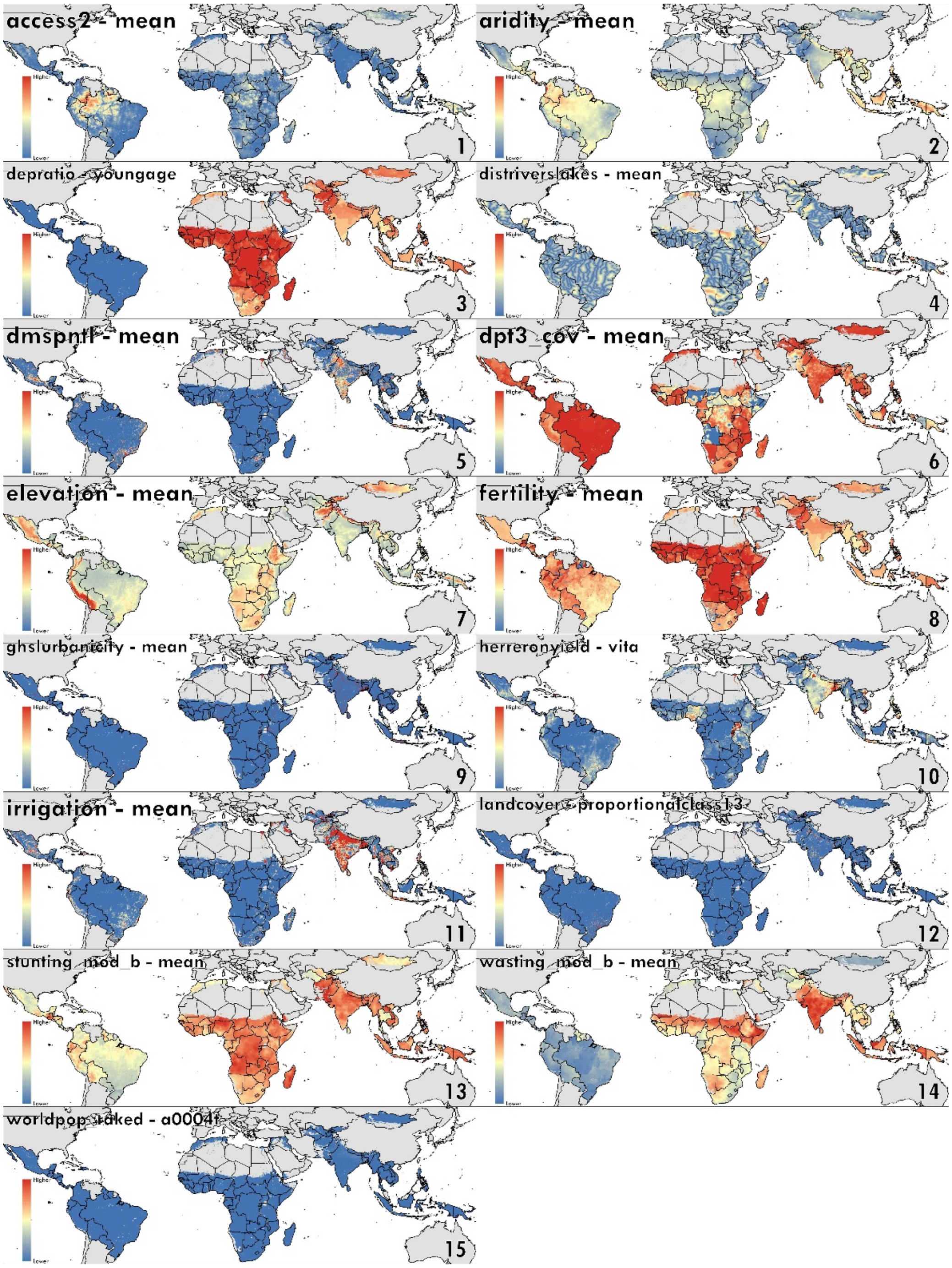
access2 - mean
1
aridity - mean
2
depratio - youngage
3
distriverslakes - mean
4
dmspntl - mean
5
dpt3_cov - mean
6
elevation - mean
7
fertility - mean
8
ghslurbanicity - mean
9
herreronyield - vita
10
irrigation - mean
11
landcover - proportionalclass13
12
stunting_mod_b - mean
13
wasting_mod_b - mean
14
worldpop_raked - a0004t
15

Appendix Figure 5. Map of modelling regions

We stratified our data and analyses into 15 regions selected to align with the Global Burden of Disease study and to allow for country-specific models in India. Each colour represents a different modelling region, where grey shows countries that we did not included in this stage of our analysis.

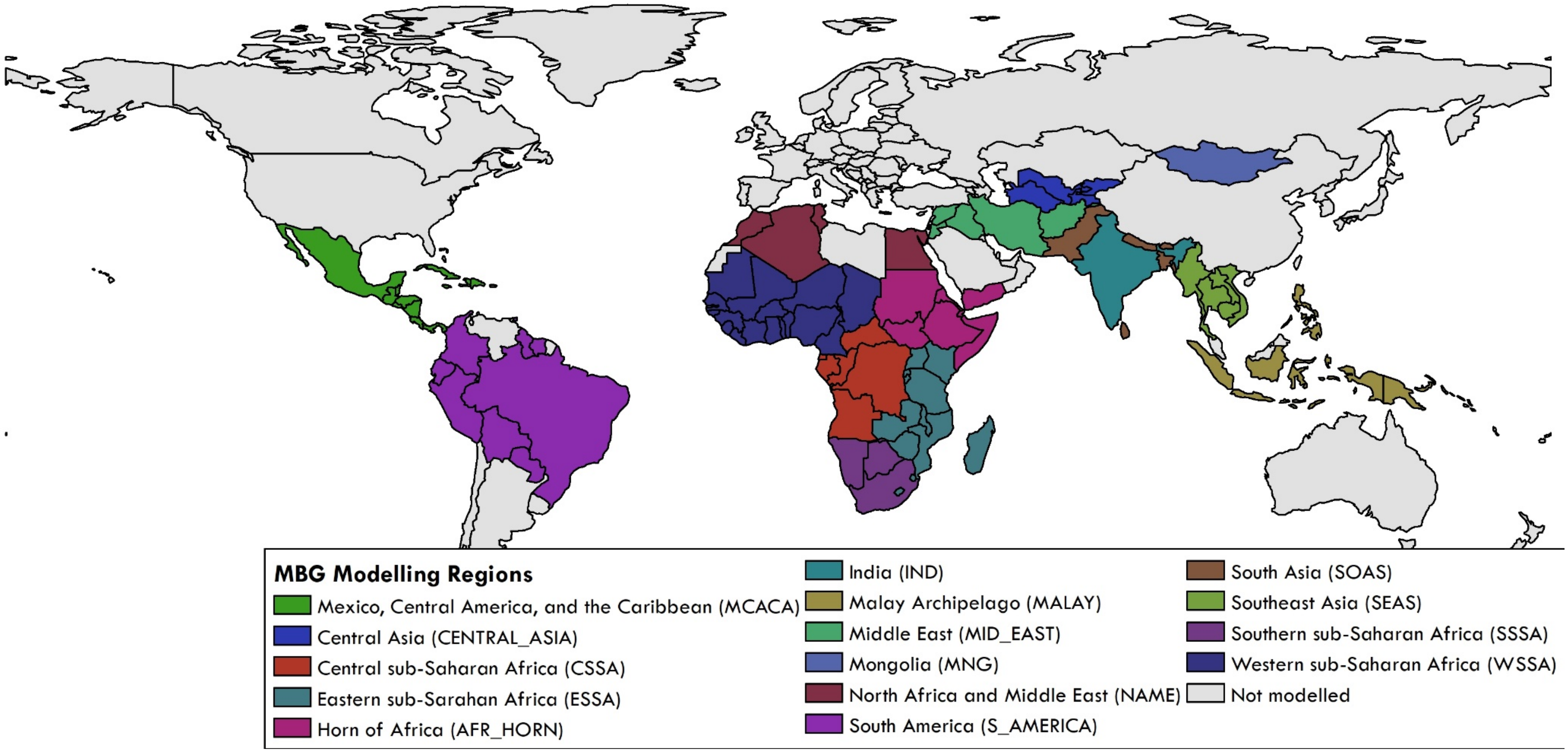

Appendix Figure 6. Finite elements mesh

The finite elements mesh used to fit the space-time correlated error for the southern sub-Saharan Africa region. Both the fine-scale mesh over land in the modelling region and the coarser buffer region mesh are shown.

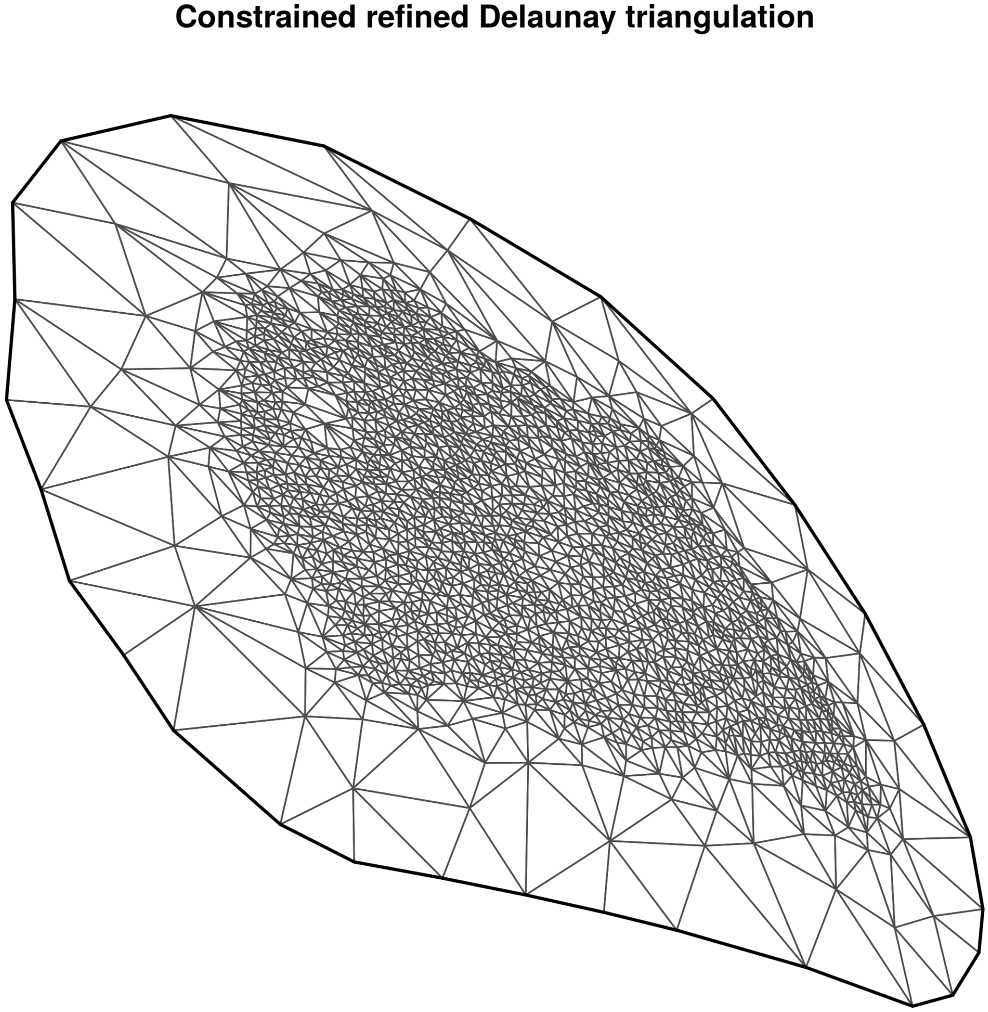

Appendix Figure 7a–n. Seasonal pattern adjustments for diarrhoea prevalence

Seasonality adjustments were made for each modelling region (Section 2.5). Each colour represents a specific country, while a circle represents unadjusted data and a diamond represents data adjusted for seasonality. Additionally, the curved black line represents a sinusoidal regression fit to the data, while the dashed horizontal line represents the mean of the predicted prevalence. Each individual plot **(a–n)** shows diarrhoea prevalence adjustments for seasonality by modelling region.

a) Middle East region

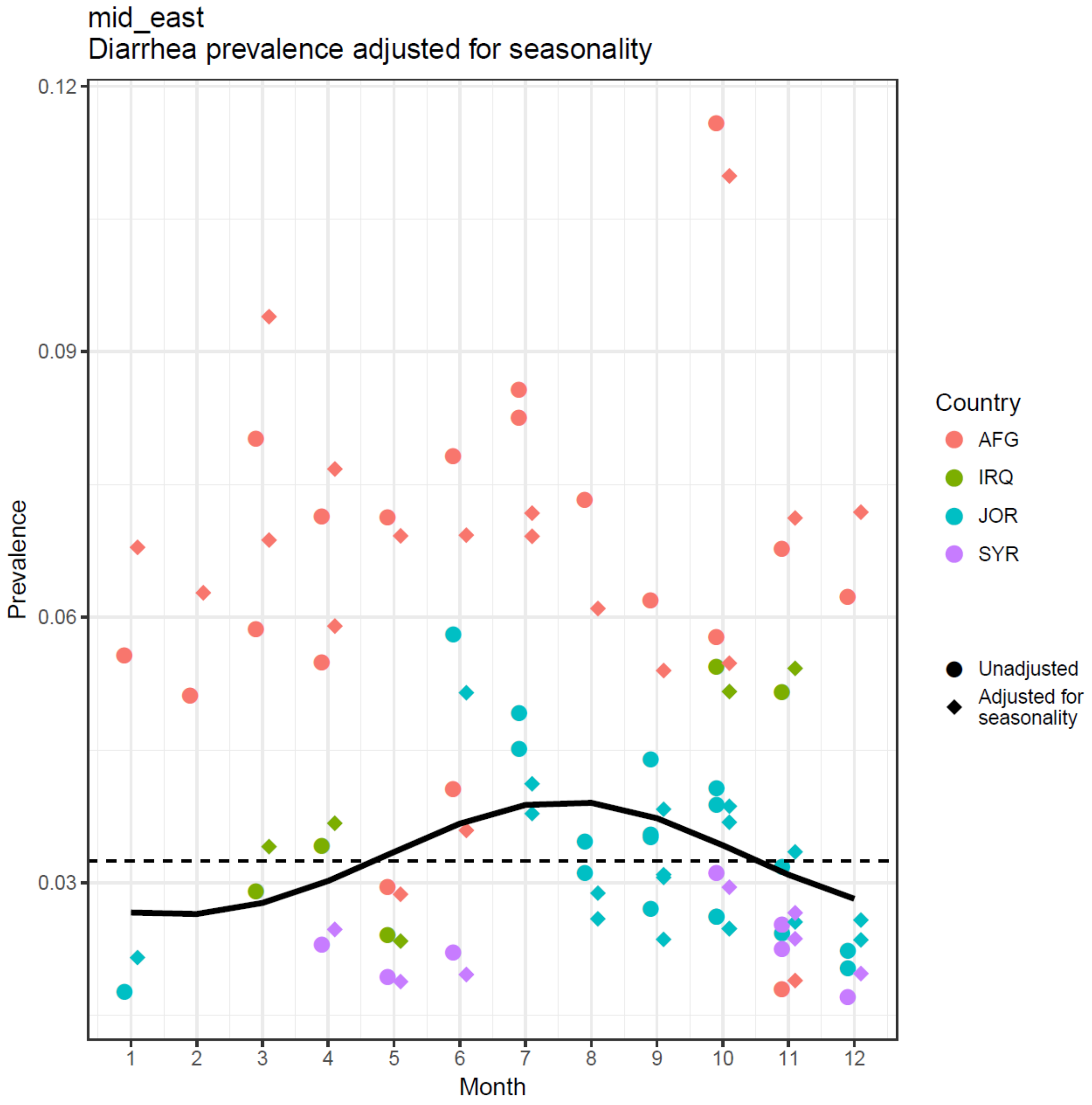

b) Central sub-Saharan Africa region

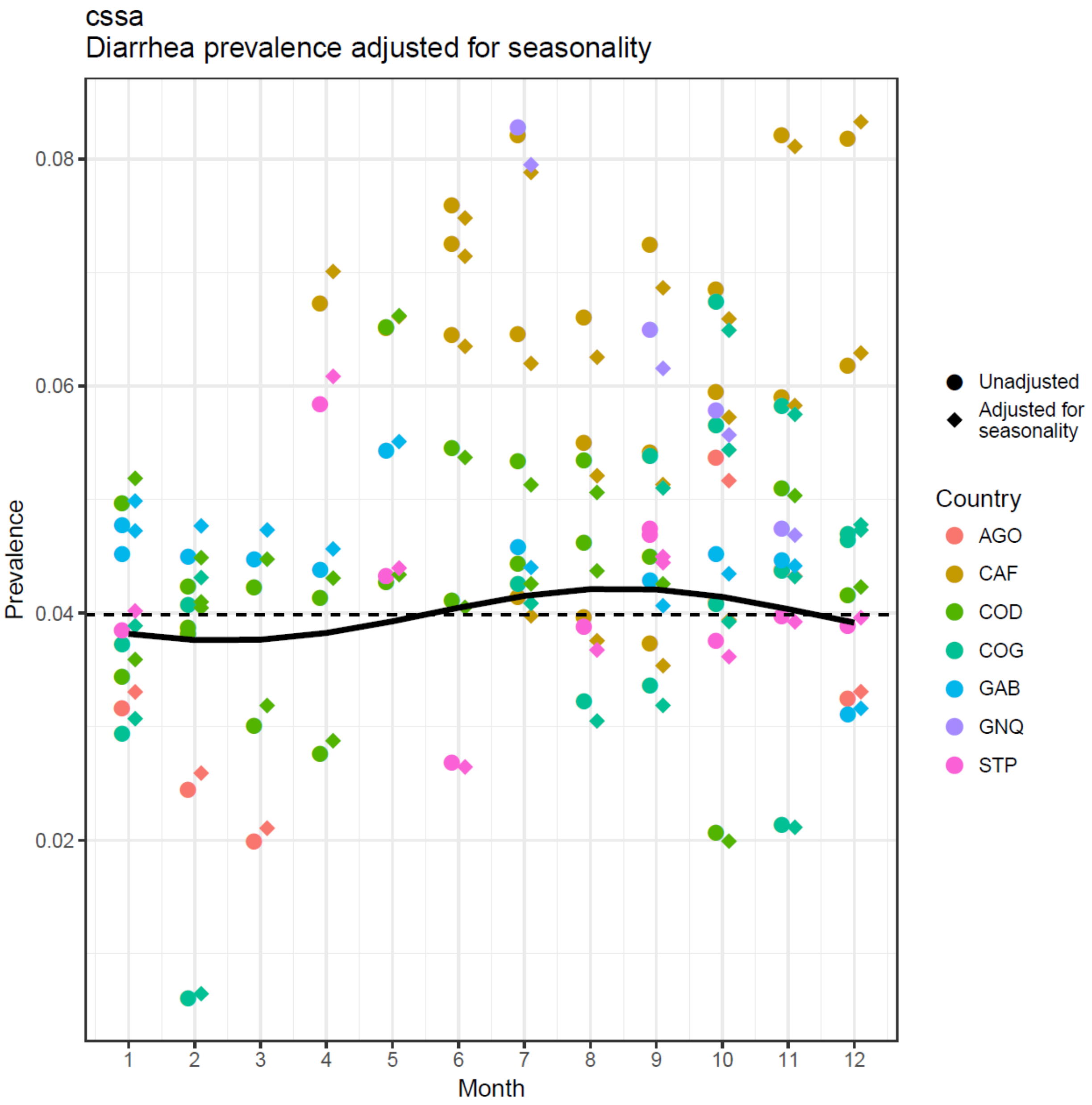

c) Eastern sub-Saharan Africa region

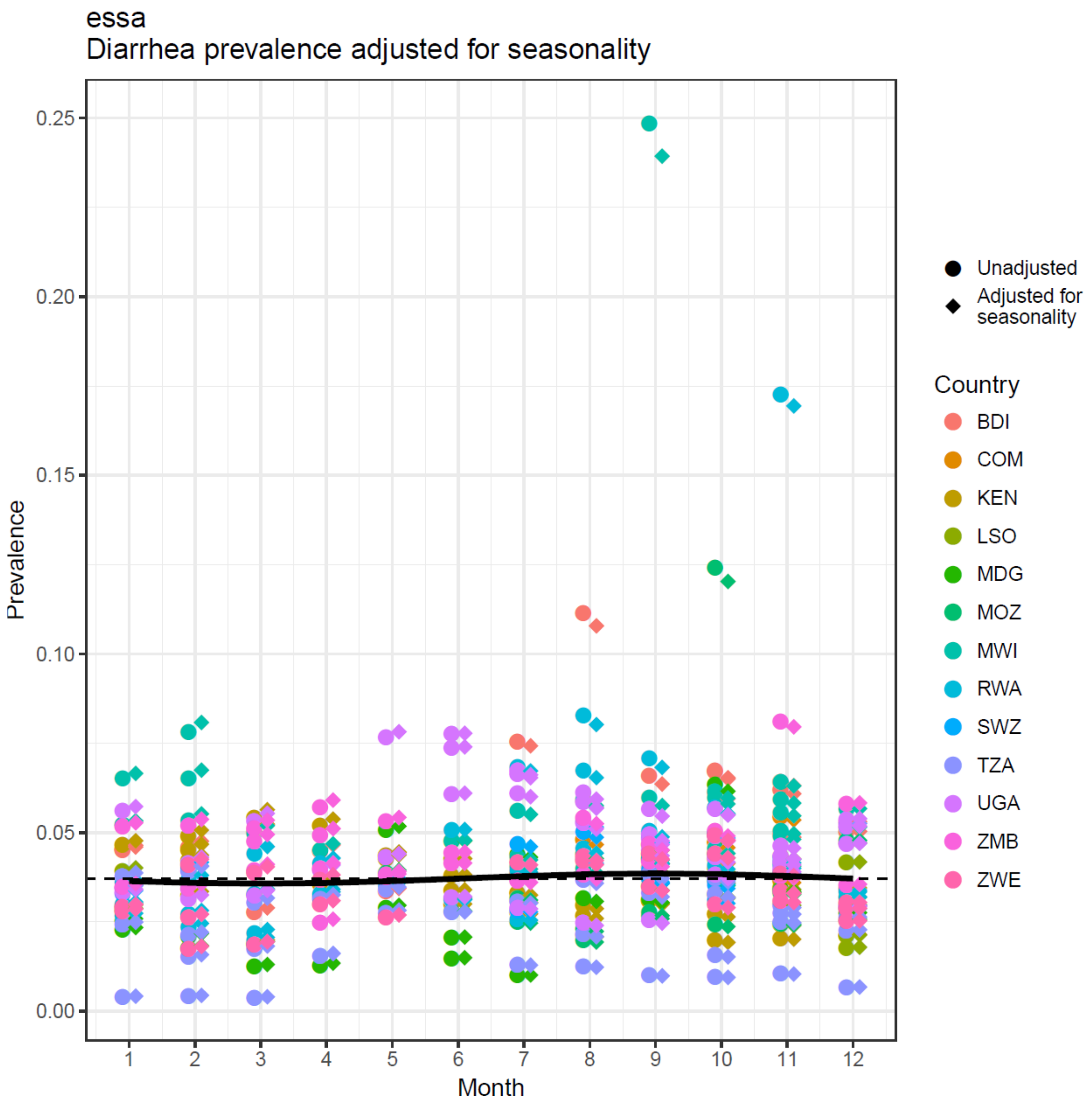

d) Western sub-Saharan Africa region

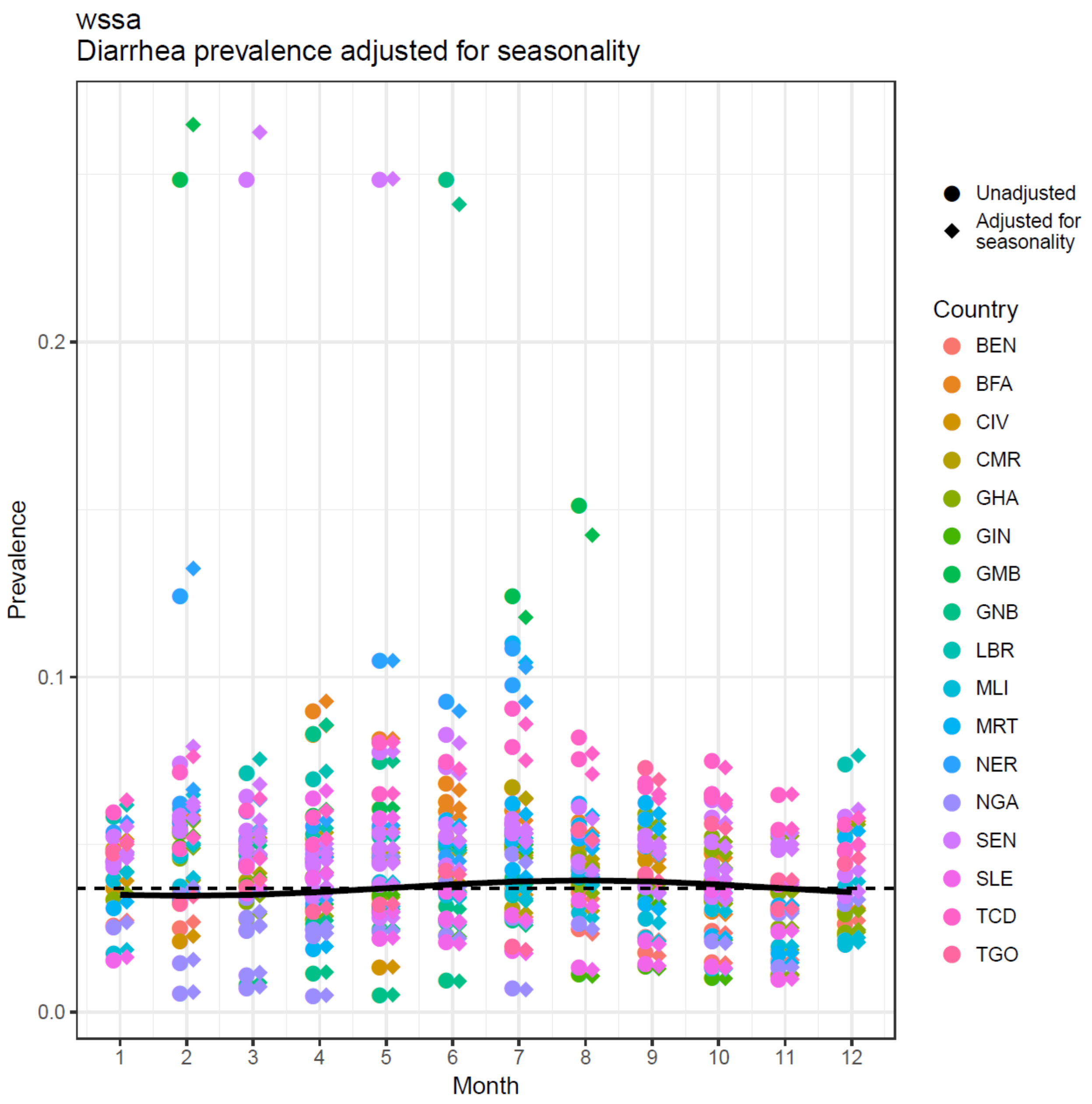

e) **South Asia region**

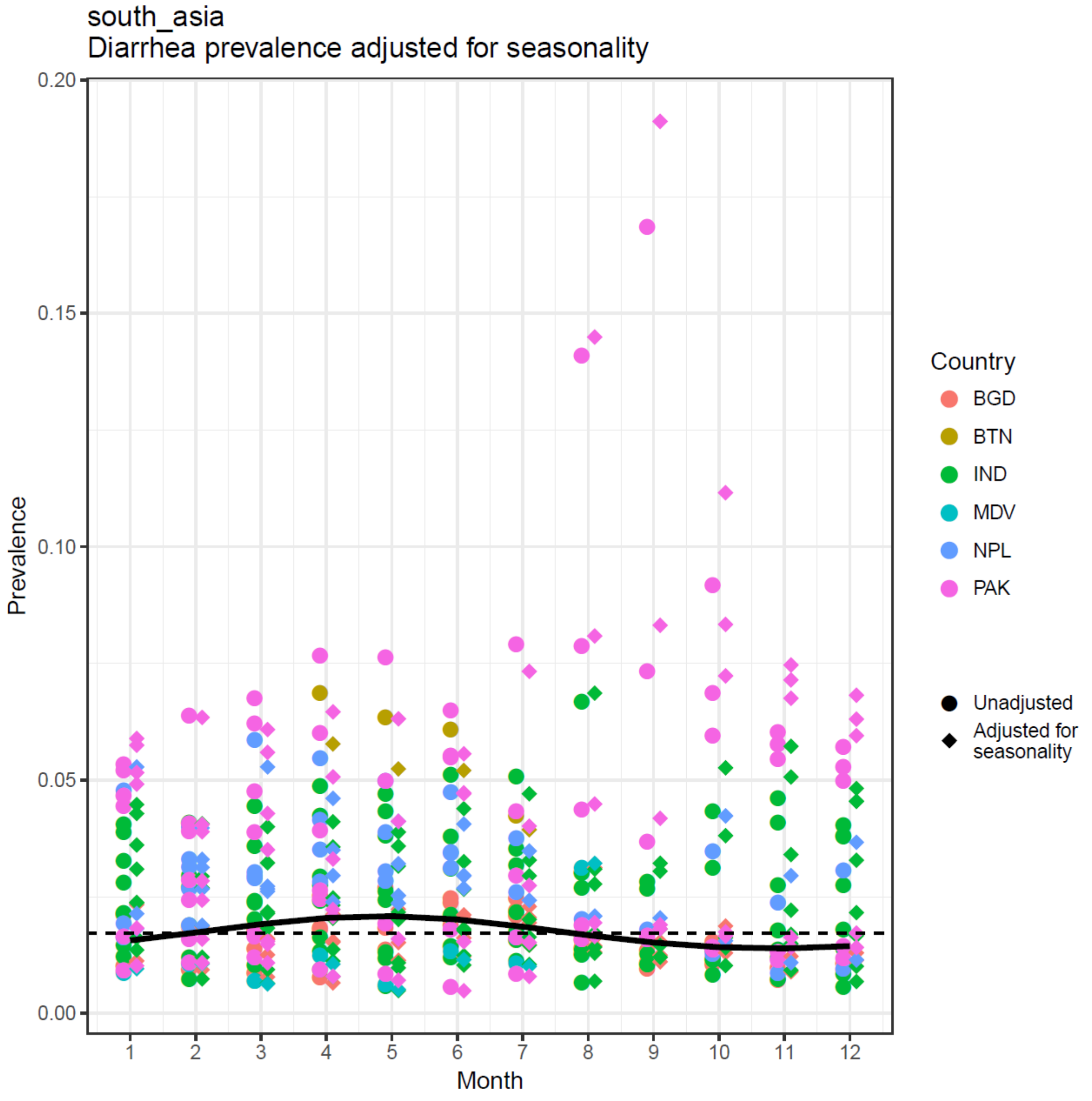

f) **Mexico, central America, and the Caribbean region**

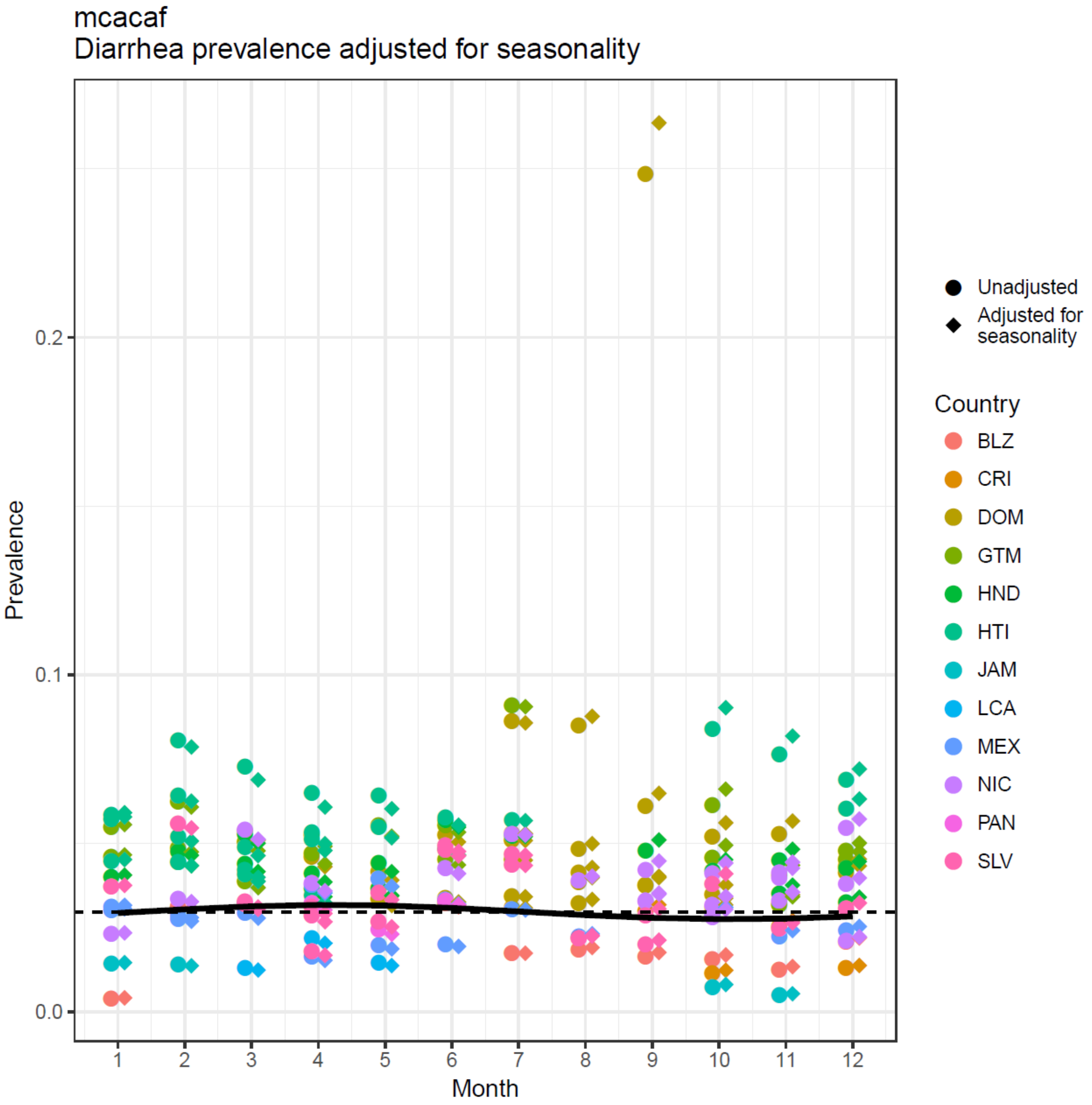

g) **South America region**

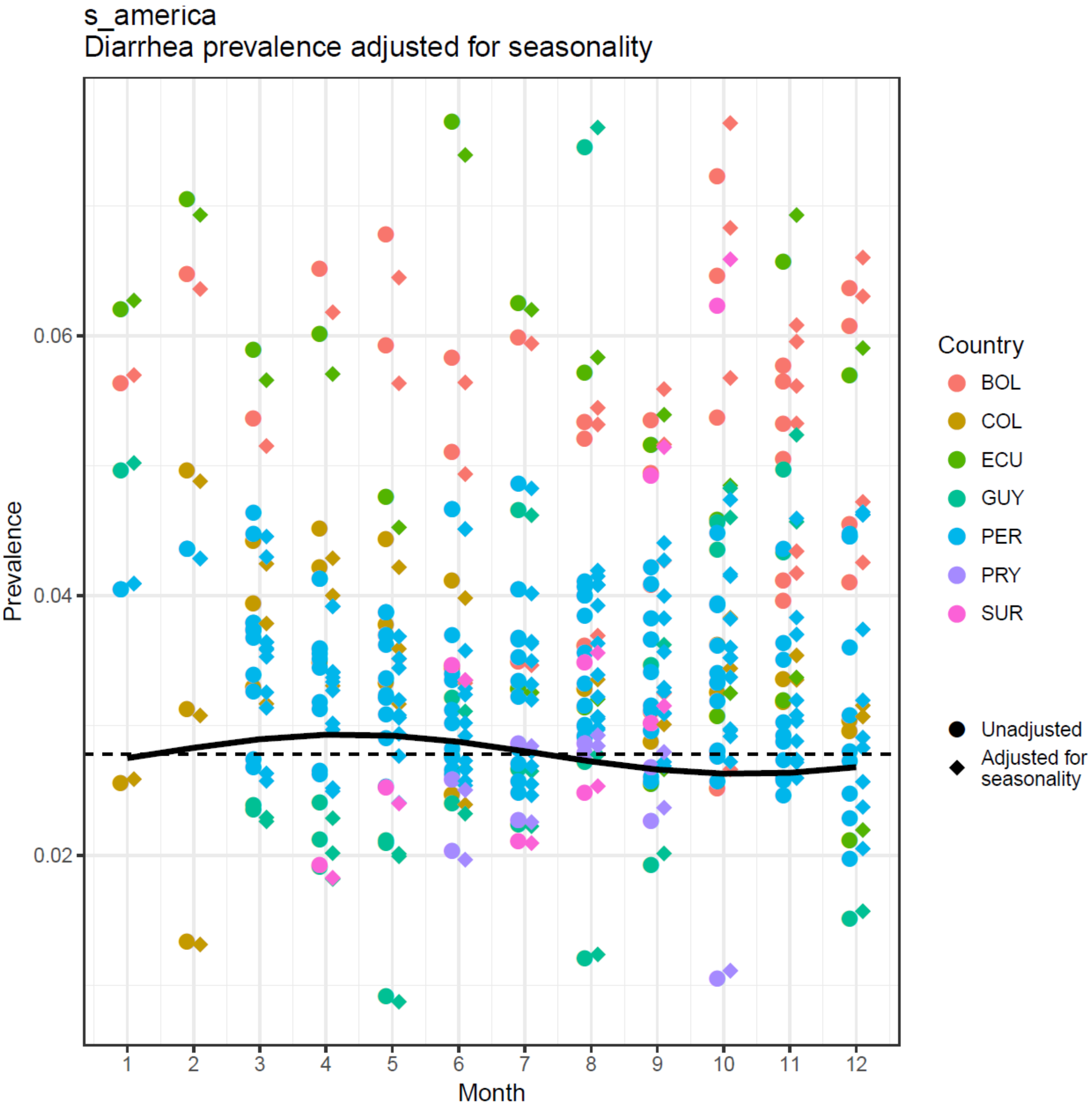

h) Southern sub-Saharan Africa region

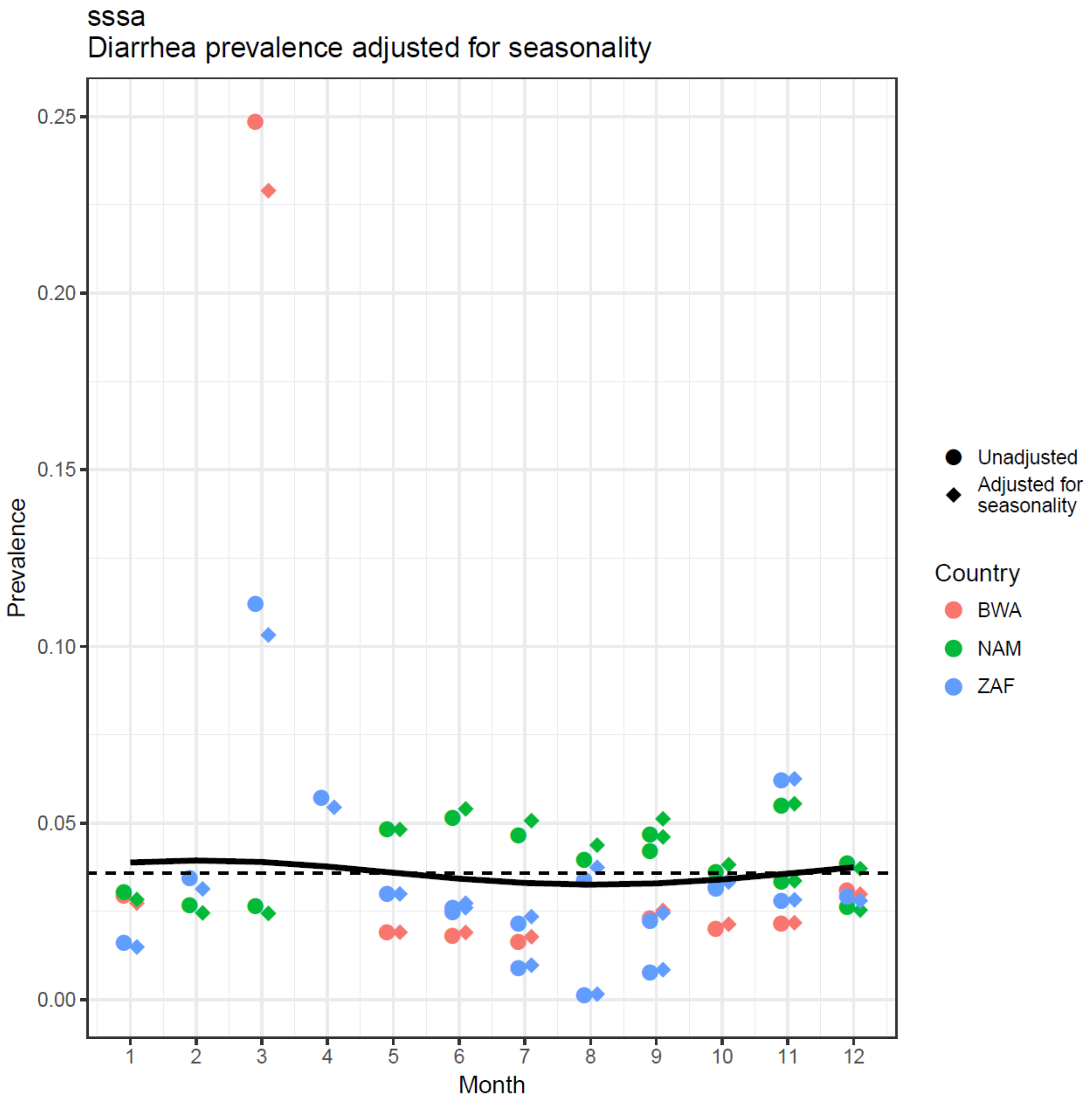

i) **Horn of Africa region**

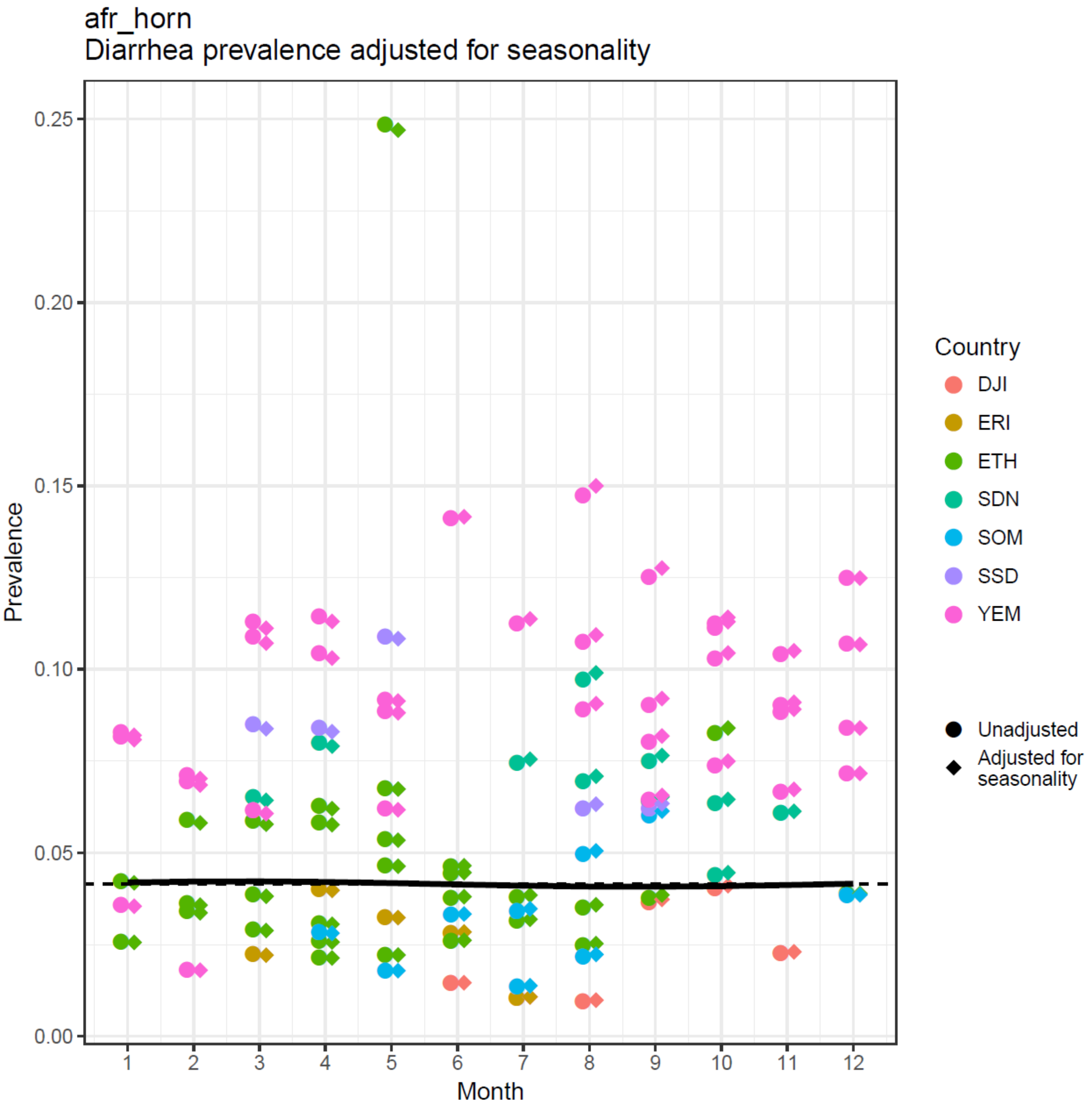

j) North Africa and Middle East region

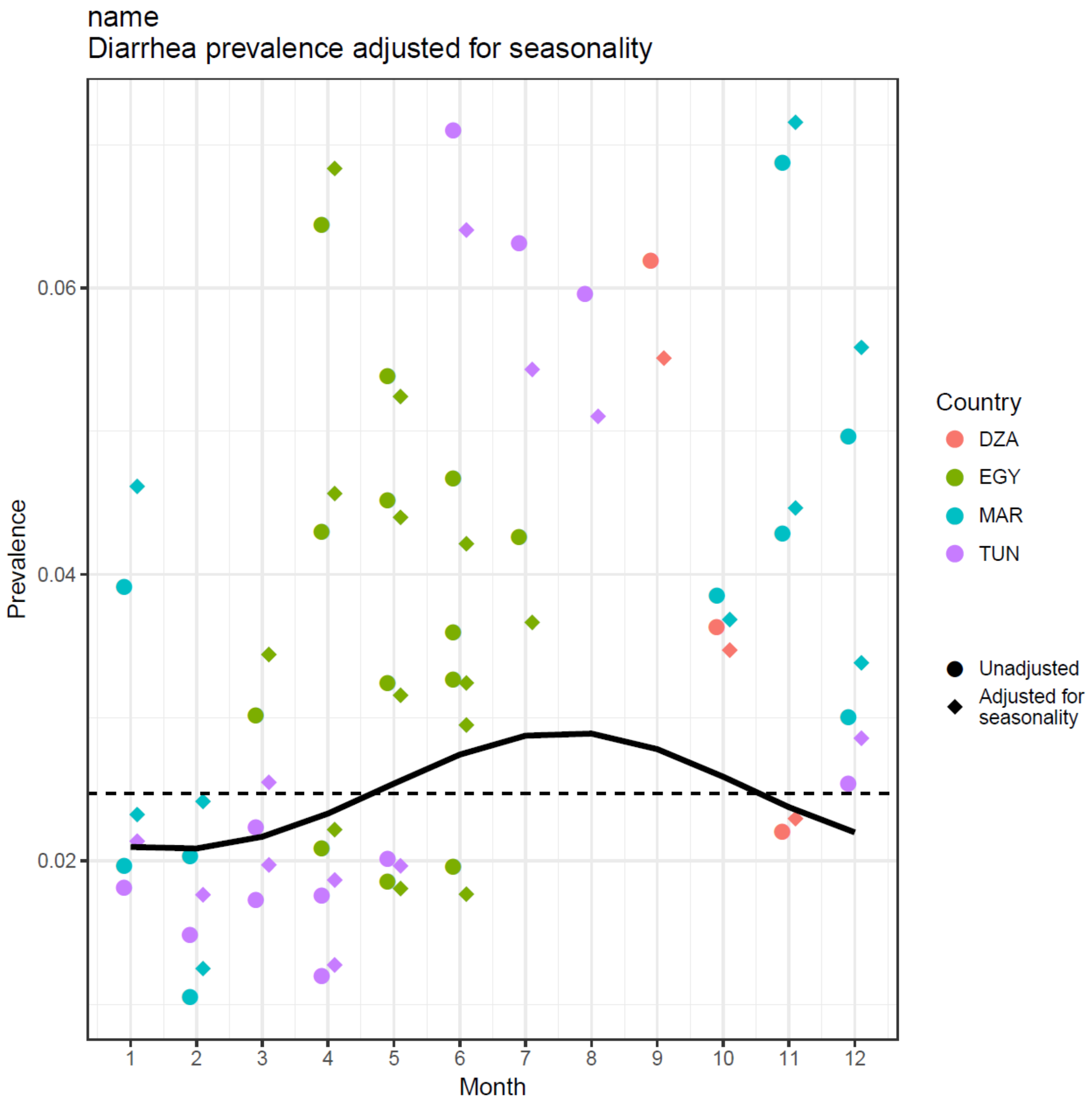

k) Malay Archipelago region

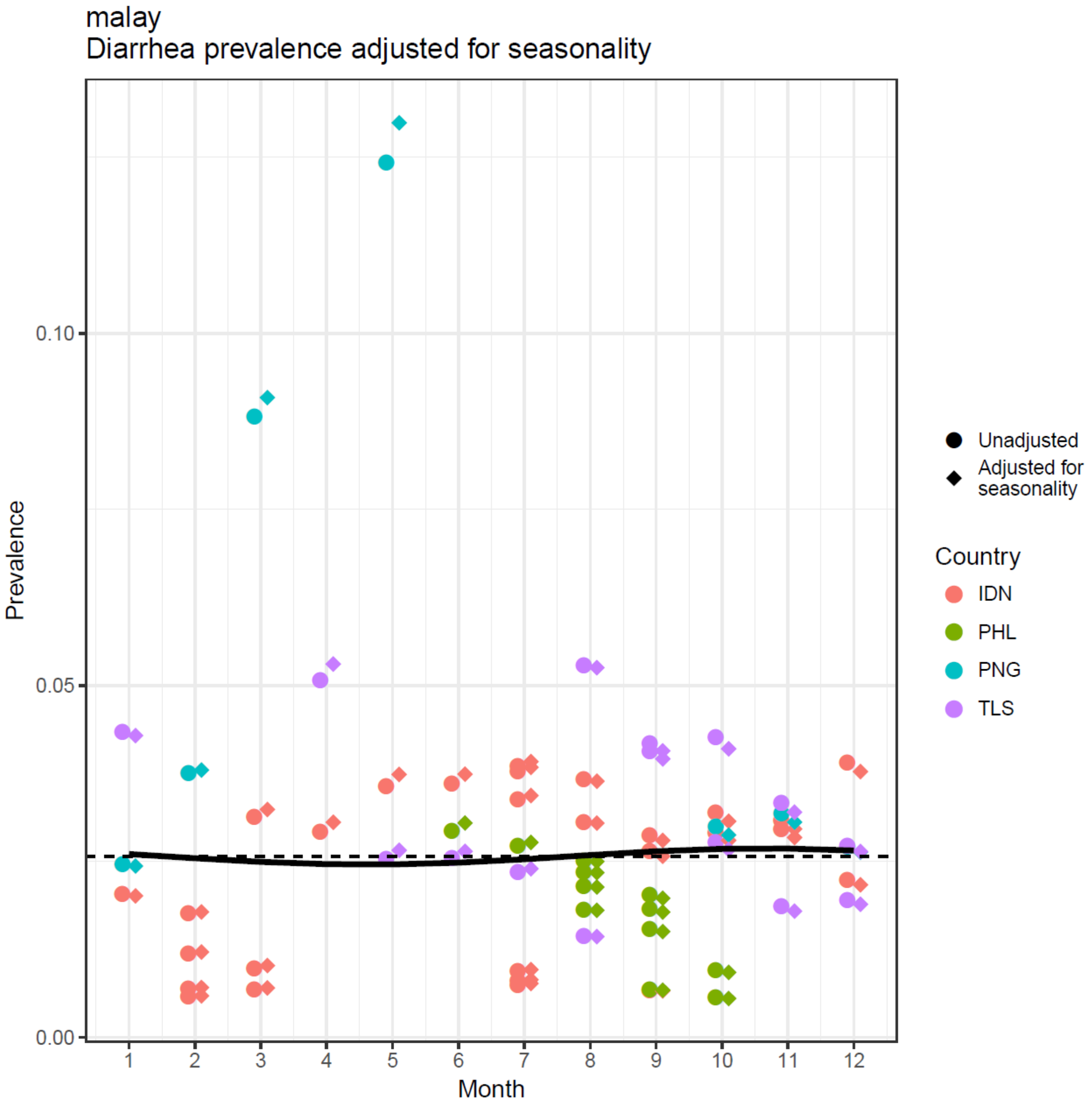

l) Central Asia region

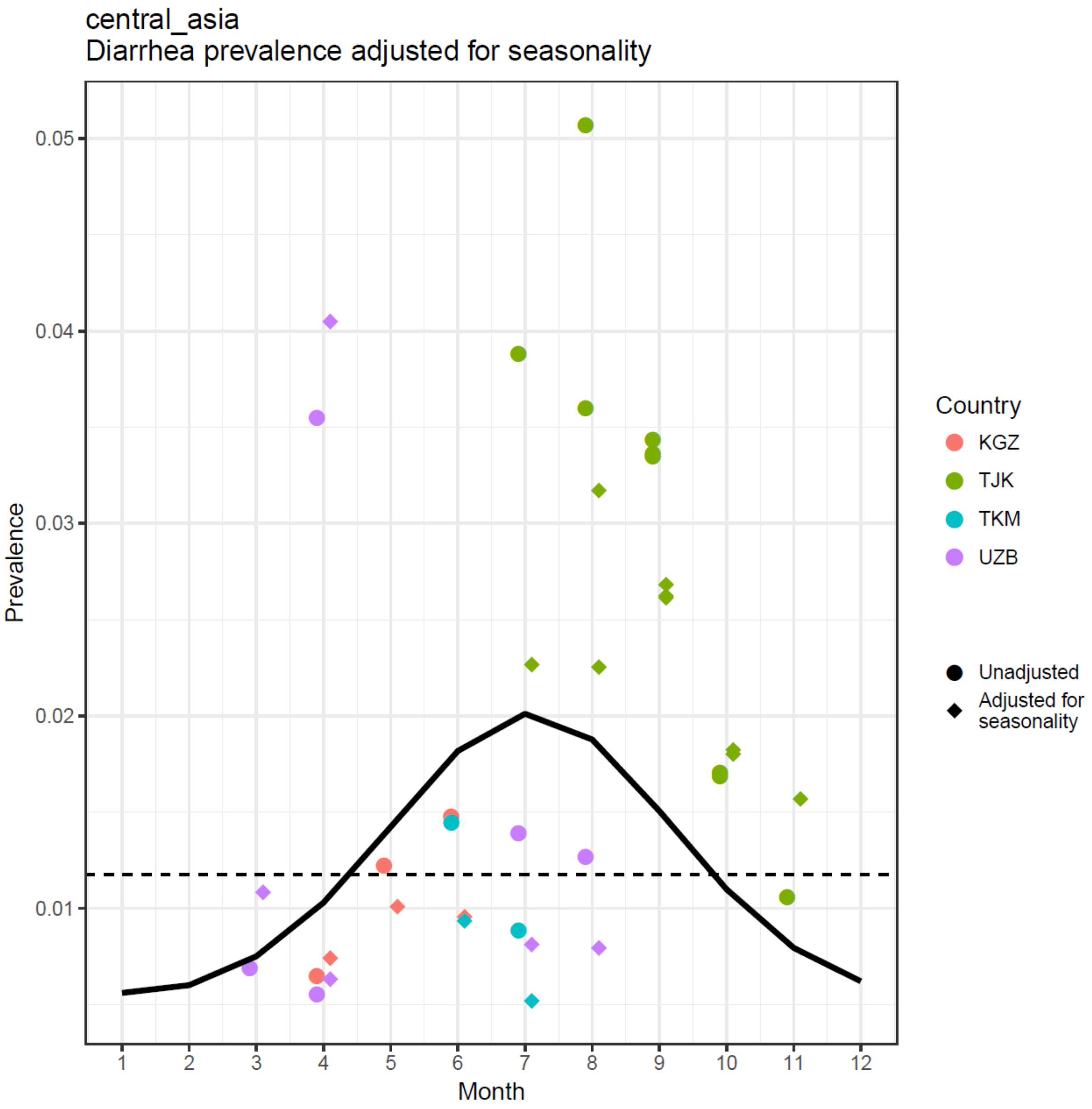

m) Southeast Asia region

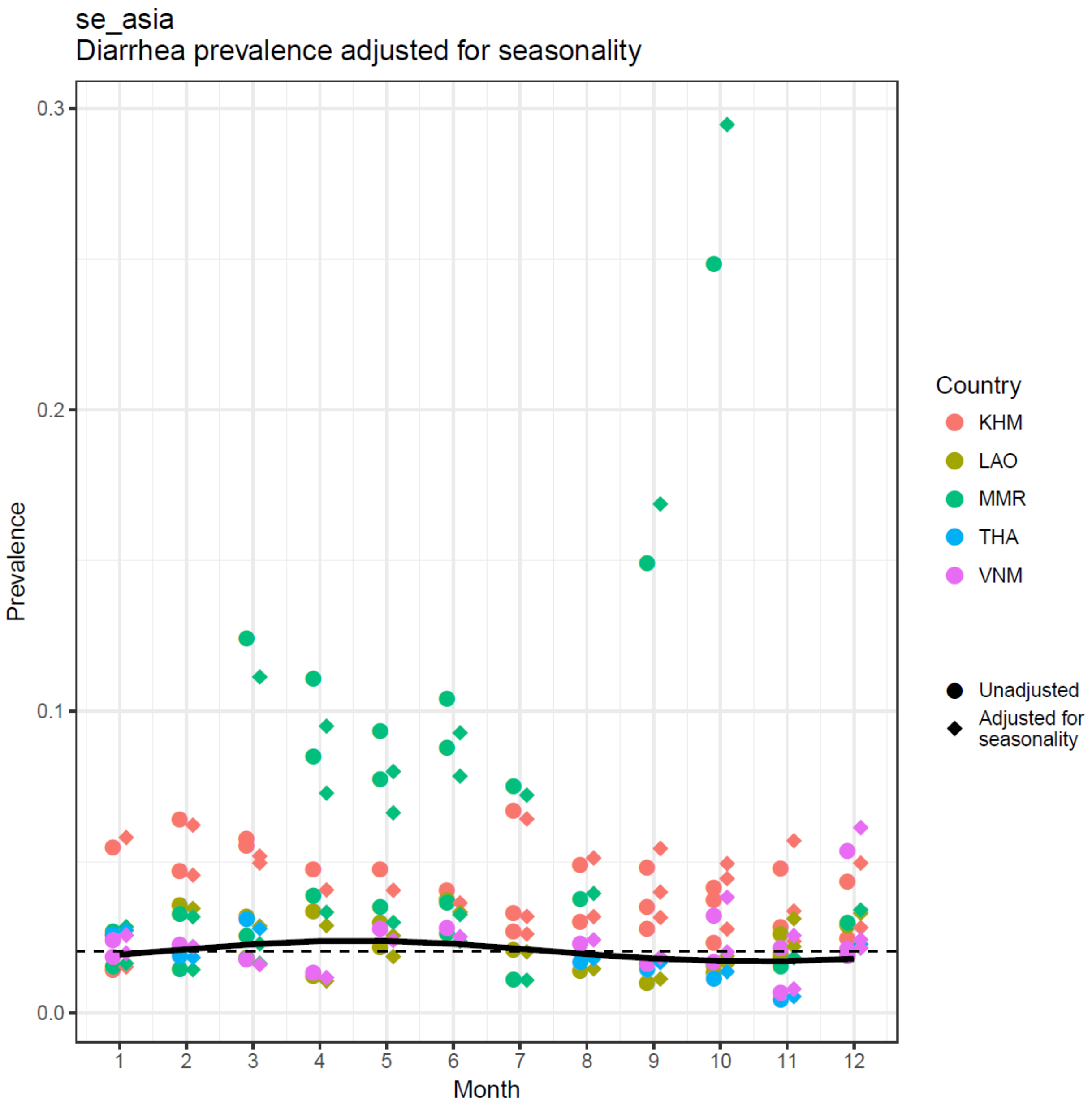

n) Mongolia region

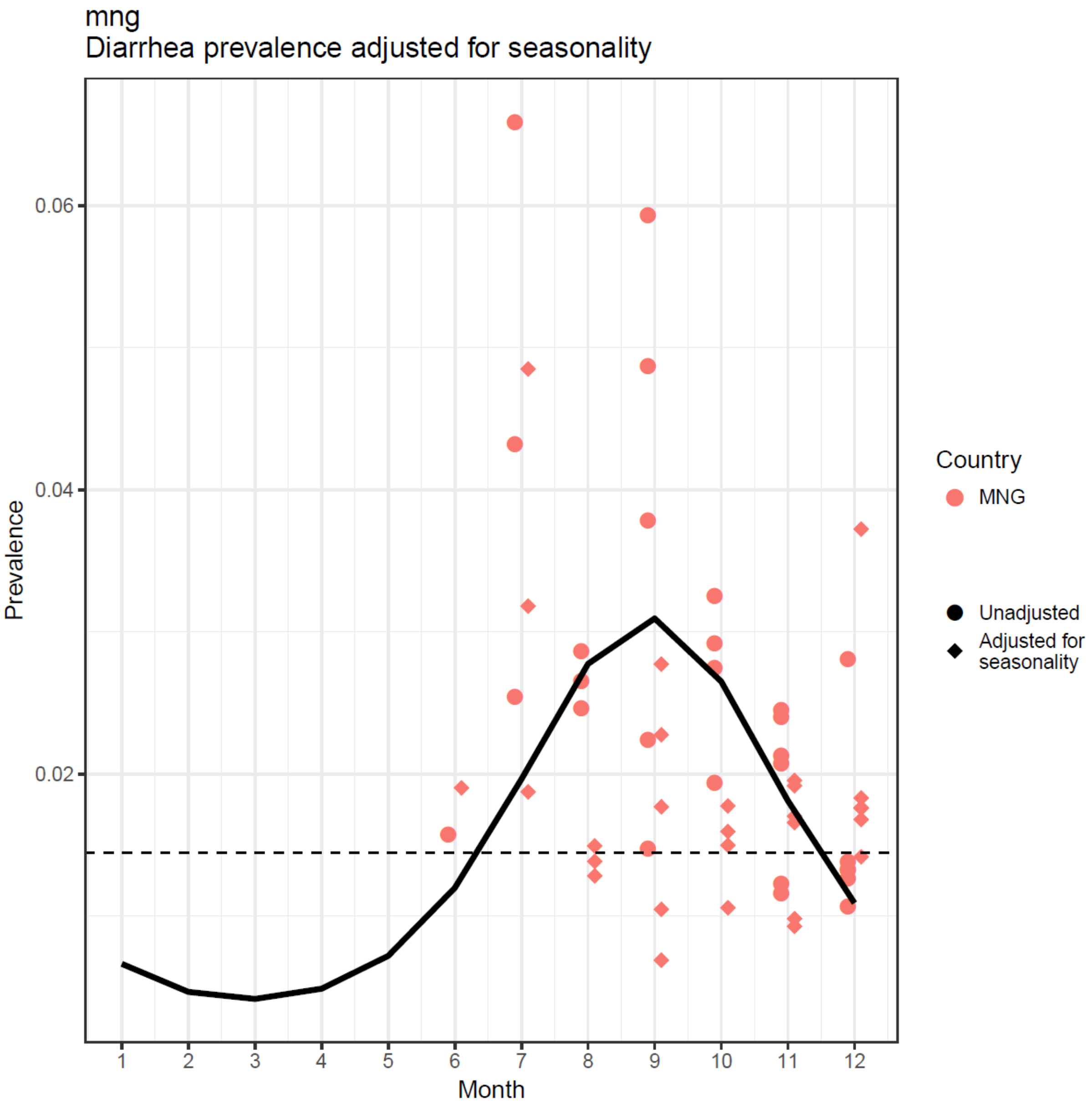

Appendix Figure 8. Posterior means and 95% uncertainty intervals for diarrhoea prevalence by grid cell, 2017

Maps reflect administrative boundaries, land cover, lakes, and population; grey-coloured grid cells were classified as "barren or sparsely vegetated" and had fewer than ten people per 1 × 1-km grid cell, or were not included in these analyses.[26–31]

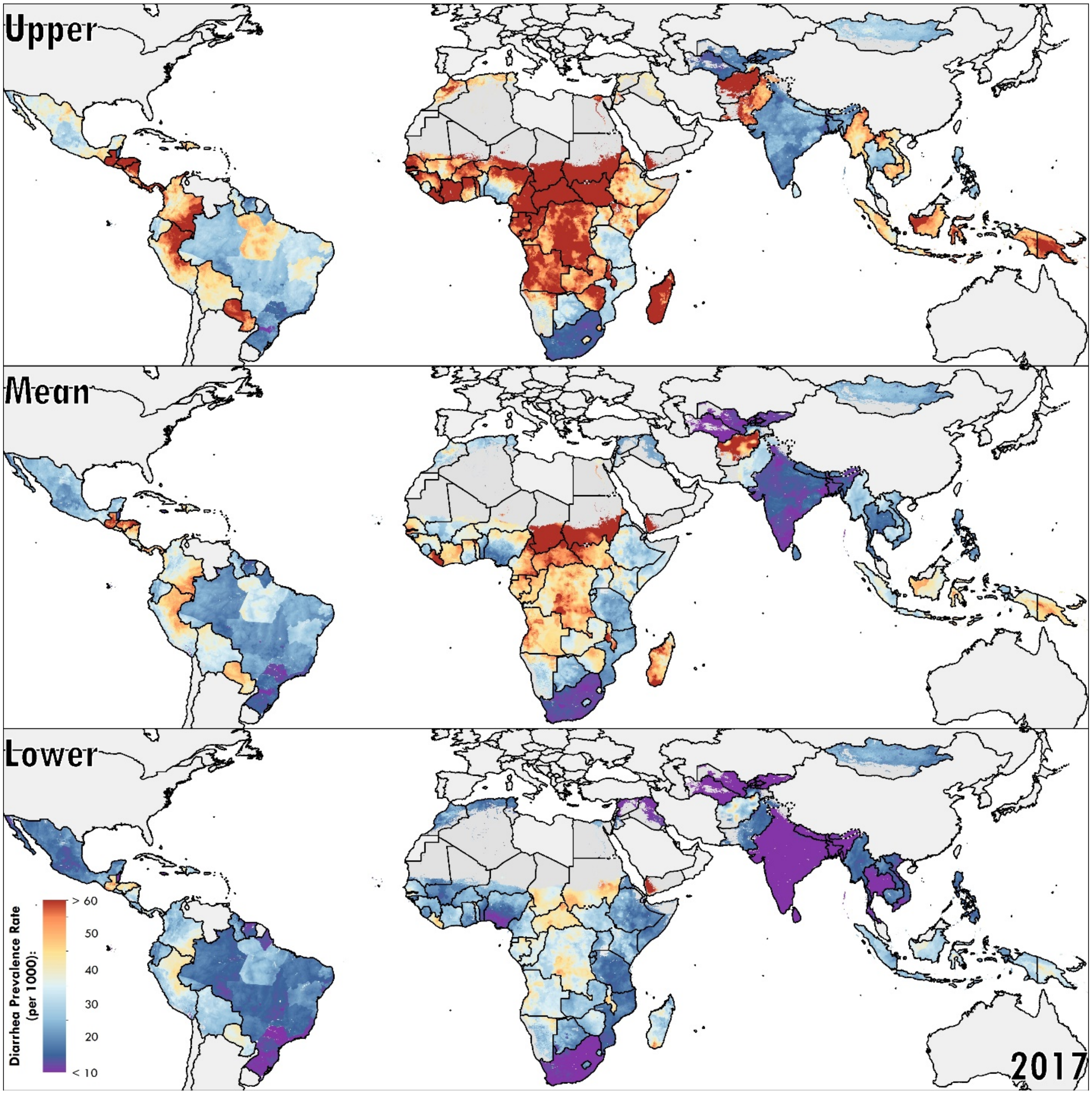

Appendix Figure 9. Posterior means and 95% uncertainty intervals for diarrhoea incidence by grid cell, 2017

Maps reflect administrative boundaries, land cover, lakes, and population; grey-coloured grid cells were classified as "barren or sparsely vegetated" and had fewer than ten people per 1 × 1-km grid cell, or were not included in these analyses.[26–31]

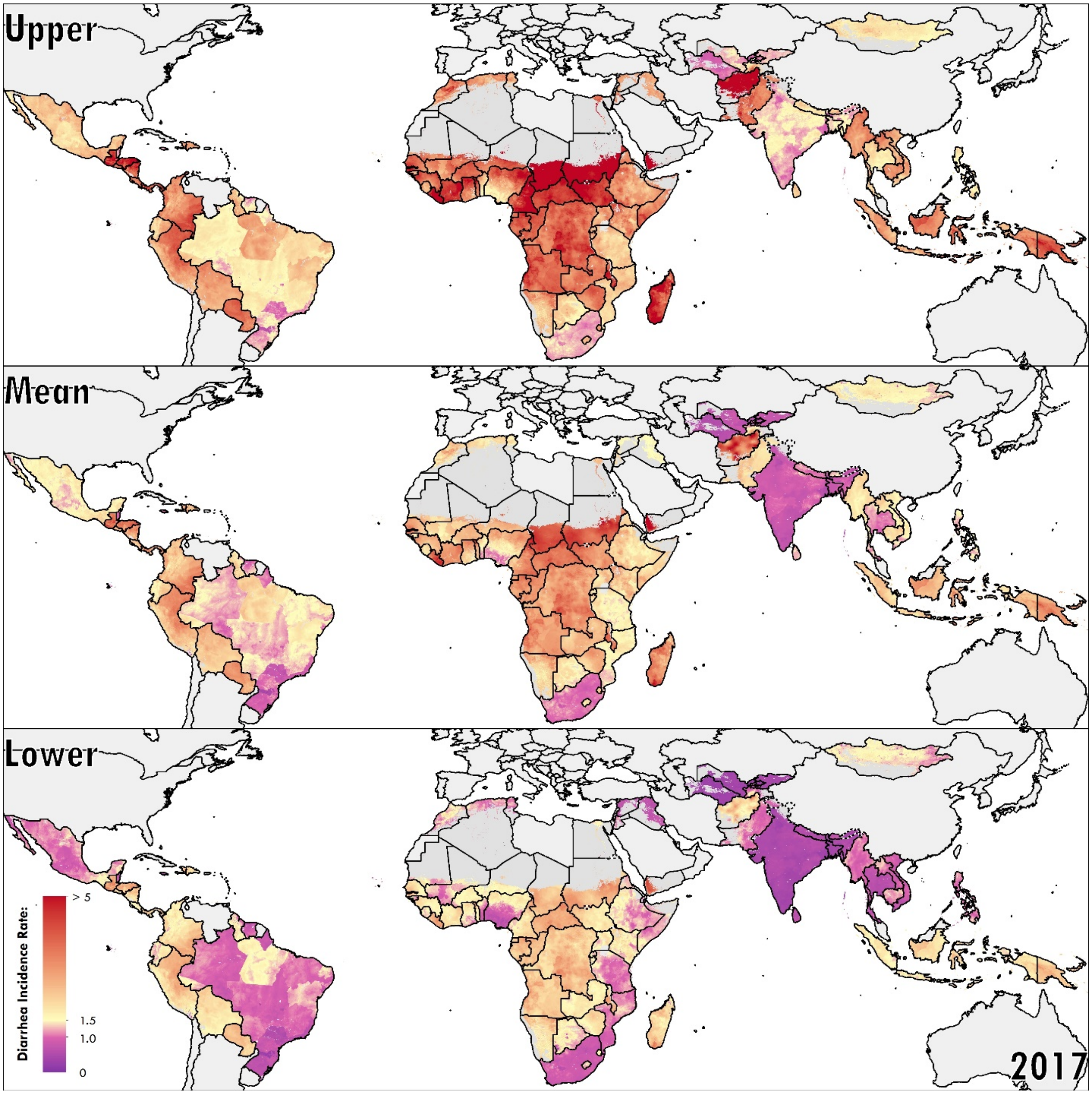

Appendix Figure 10. Posterior means and 95% uncertainty intervals for diarrhoeal mortality by grid cell, 2017

Maps reflect administrative boundaries, land cover, lakes, and population; grey-coloured grid cells were classified as "barren or sparsely vegetated" and had fewer than ten people per 1 × 1-km grid cell, or were not included in these analyses.[26–31]

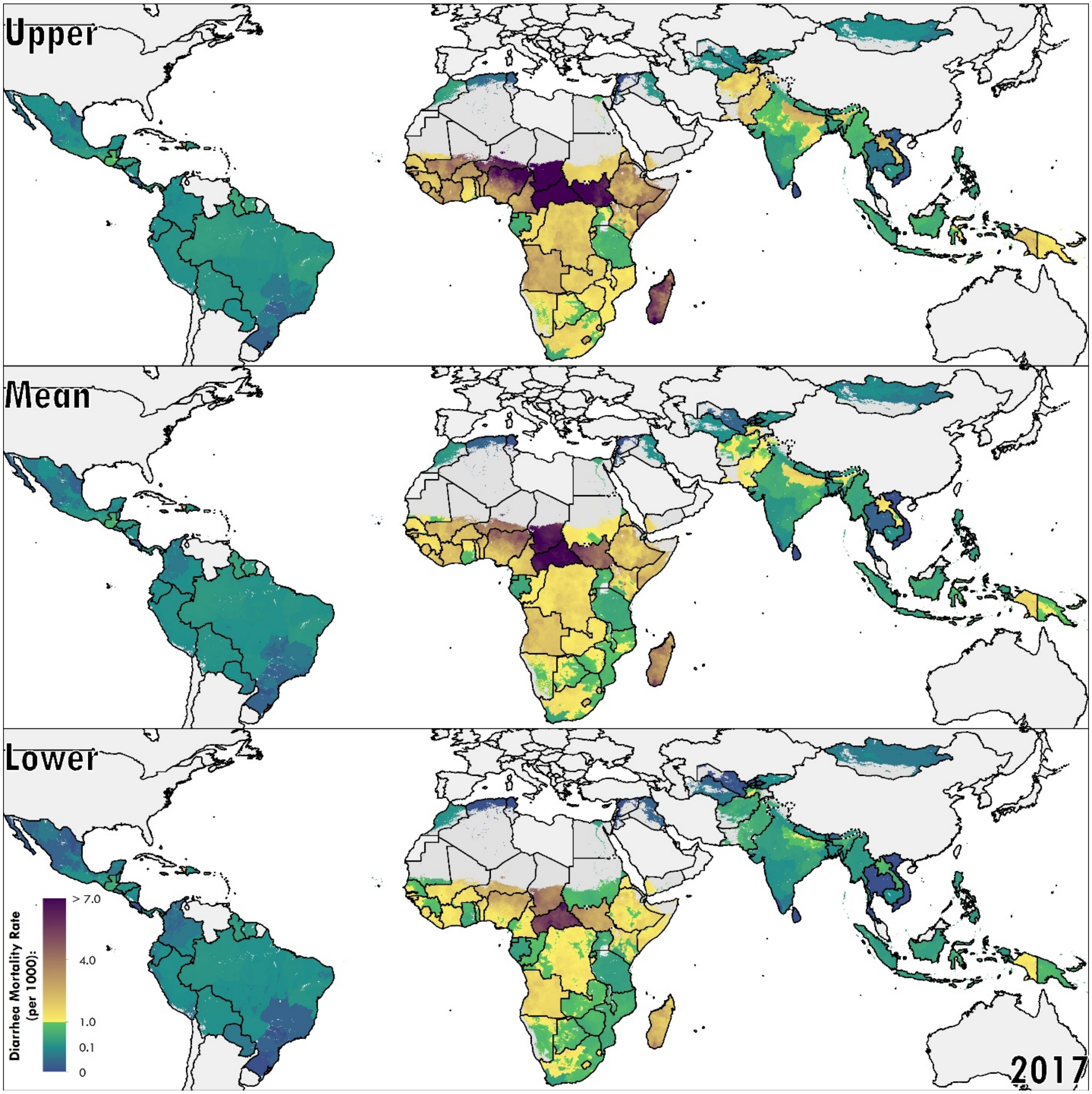

Appendix Figure 11. Posterior means and 95% uncertainty intervals for diarrhoea prevalence at the second administrative level, 2017

Maps reflect administrative boundaries, land cover, lakes, and population; grey-coloured grid cells were classified as "barren or sparsely vegetated" and had fewer than ten people per 1 × 1-km grid cell, or were not included in these analyses.[26–31]

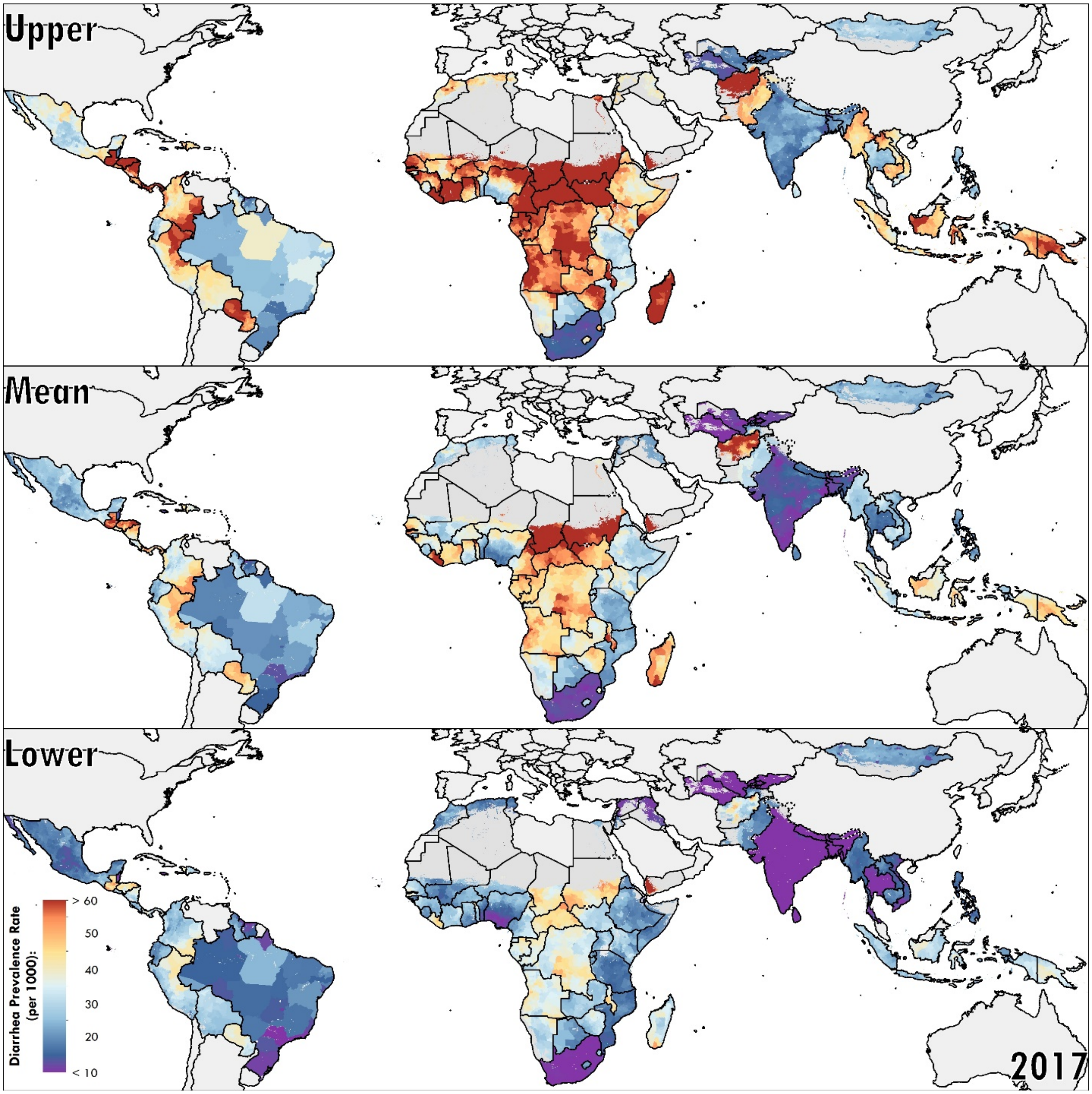

Appendix Figure 12. Posterior means and 95% uncertainty intervals for diarrhoea incidence at the second administrative level, 2017

Maps reflect administrative boundaries, land cover, lakes, and population; grey-coloured grid cells were classified as "barren or sparsely vegetated" and had fewer than ten people per 1 × 1-km grid cell, or were not included in these analyses.[26–31]

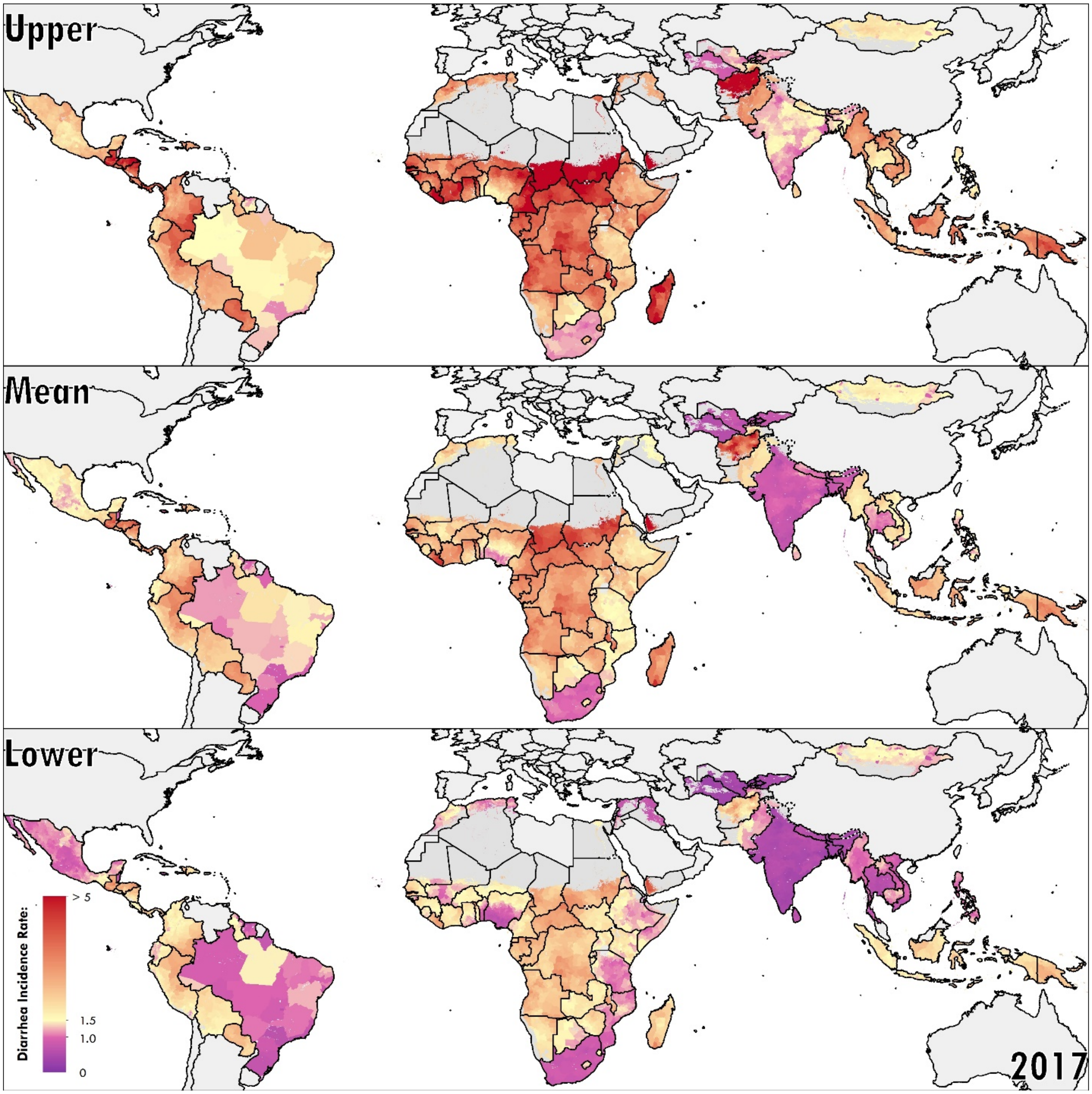

Appendix Figure 13. Posterior means and 95% uncertainty intervals for diarrhoeal mortality at the second administrative level, 2017

Maps reflect administrative boundaries, land cover, lakes, and population; grey-coloured grid cells were classified as "barren or sparsely vegetated" and had fewer than ten people per 1 × 1-km grid cell, or were not included in these analyses.[26–31]

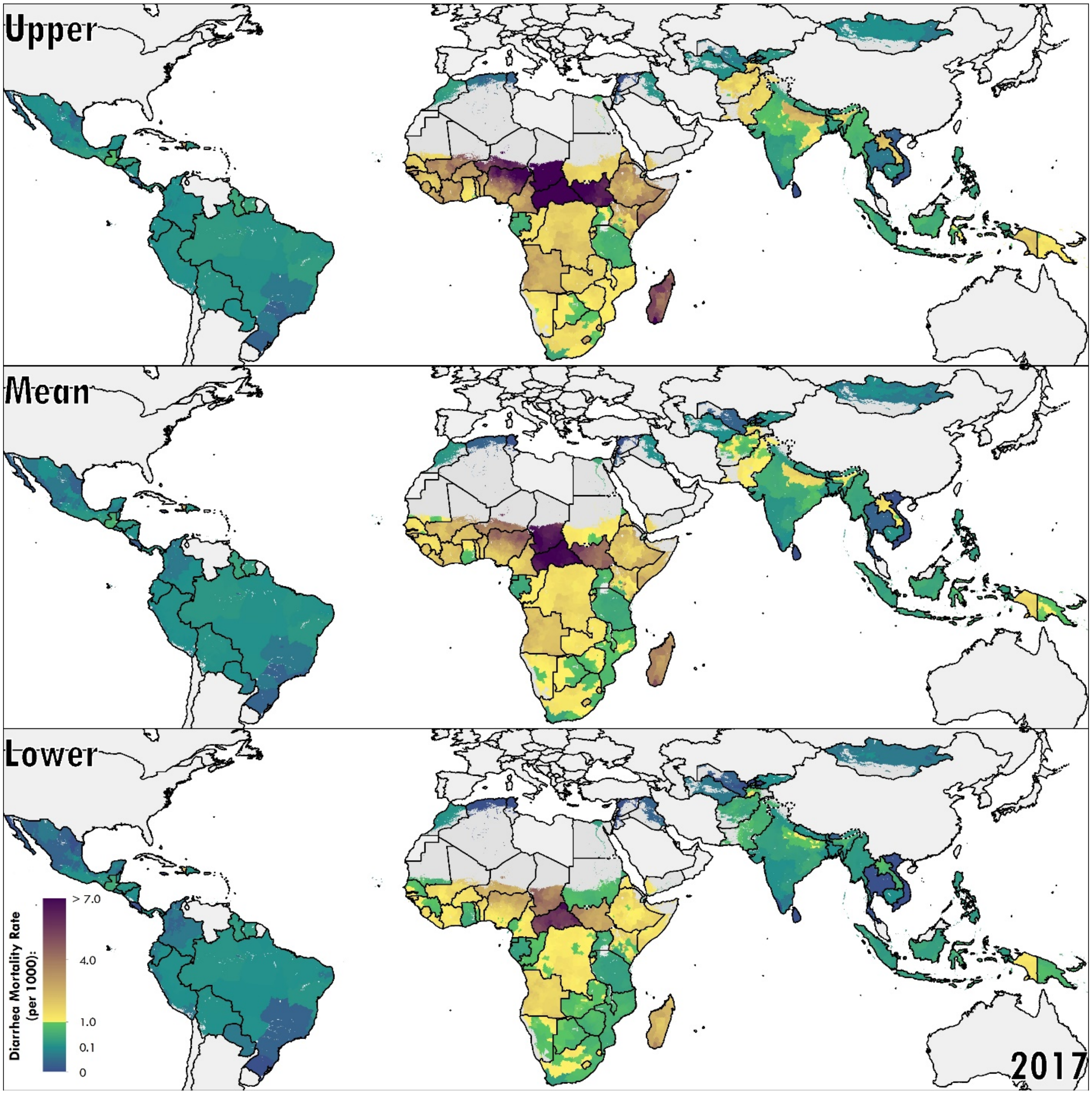

Appendix Figure 14. Posterior means and 95% uncertainty intervals for diarrhoea prevalence at the first administrative level, 2017

Maps reflect administrative boundaries, land cover, lakes, and population; grey-coloured grid cells were classified as "barren or sparsely vegetated" and had fewer than ten people per 1 × 1-km grid cell, or were not included in these analyses.[26–31]

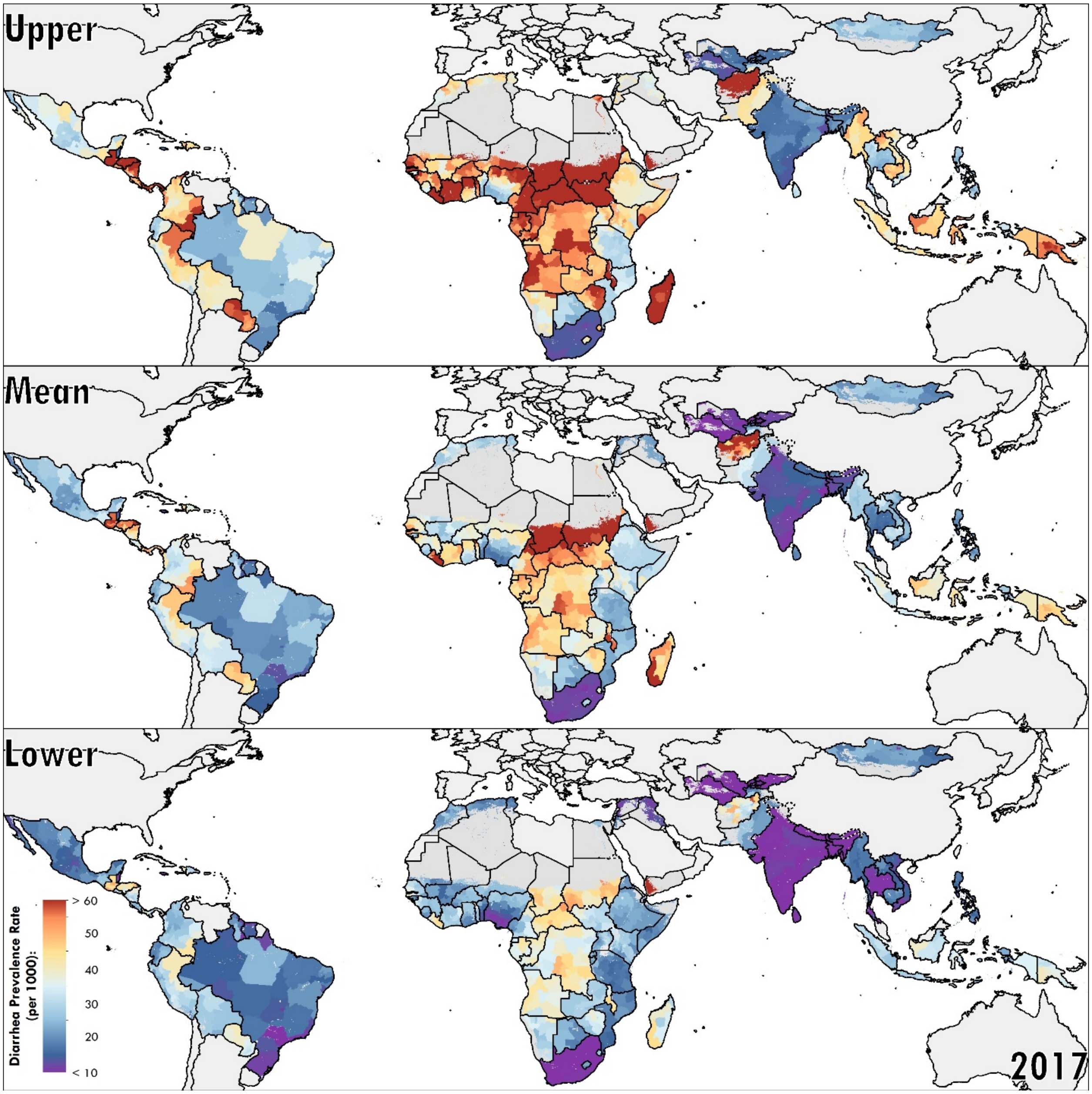

Appendix Figure 15. Posterior means and 95% uncertainty intervals for diarrhoea incidence at the first administrative level, 2017

Maps reflect administrative boundaries, land cover, lakes, and population; grey-coloured grid cells were classified as "barren or sparsely vegetated" and had fewer than ten people per 1 × 1-km grid cell, or were not included in these analyses.[26–31]

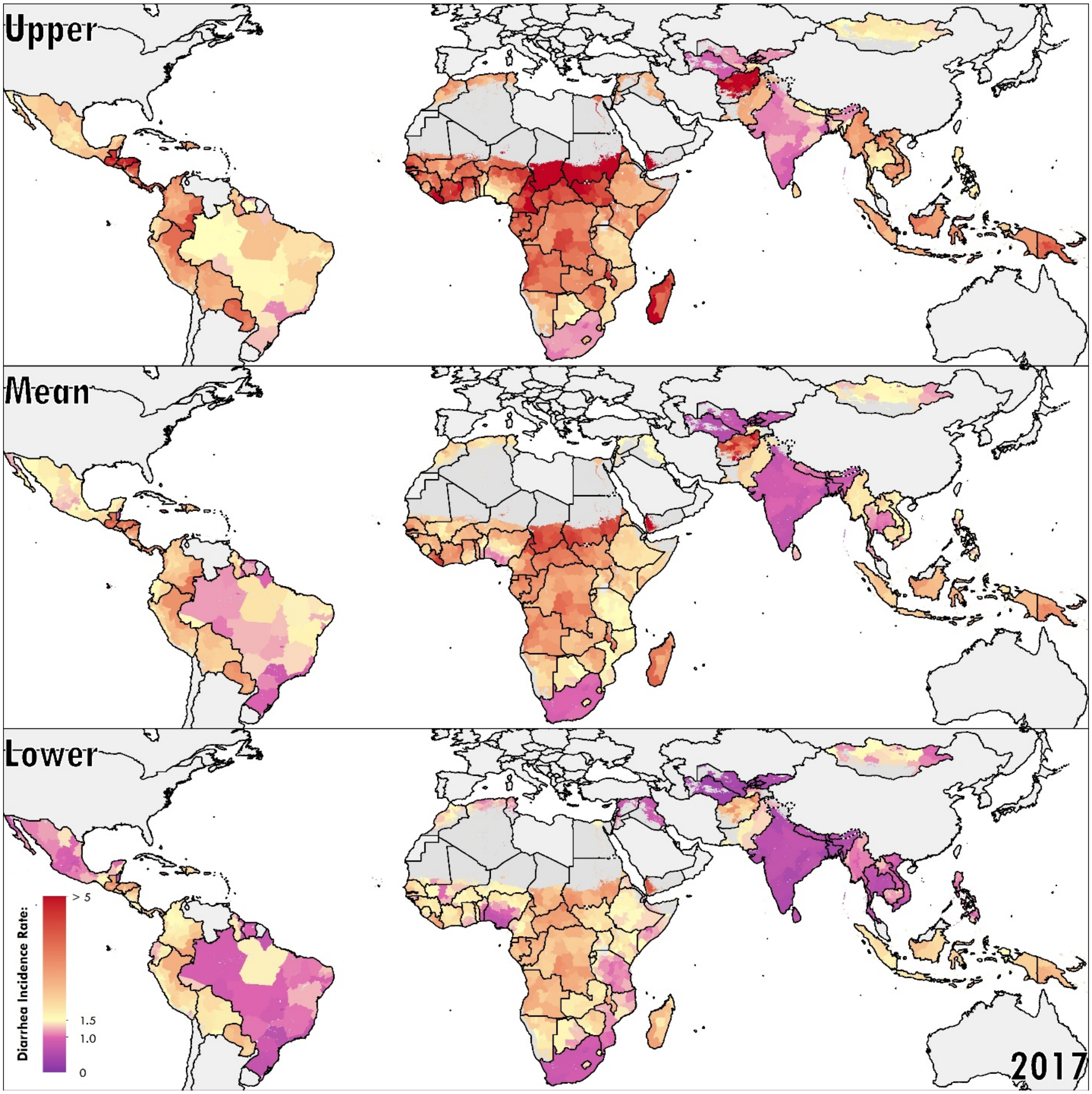

Appendix Figure 16. Posterior means and 95% uncertainty intervals for diarrhoeal mortality at the first administrative level, 2017

Maps reflect administrative boundaries, land cover, lakes, and population; grey-coloured grid cells were classified as "barren or sparsely vegetated" and had fewer than ten people per 1 × 1-km grid cell, or were not included in these analyses.[26–31]

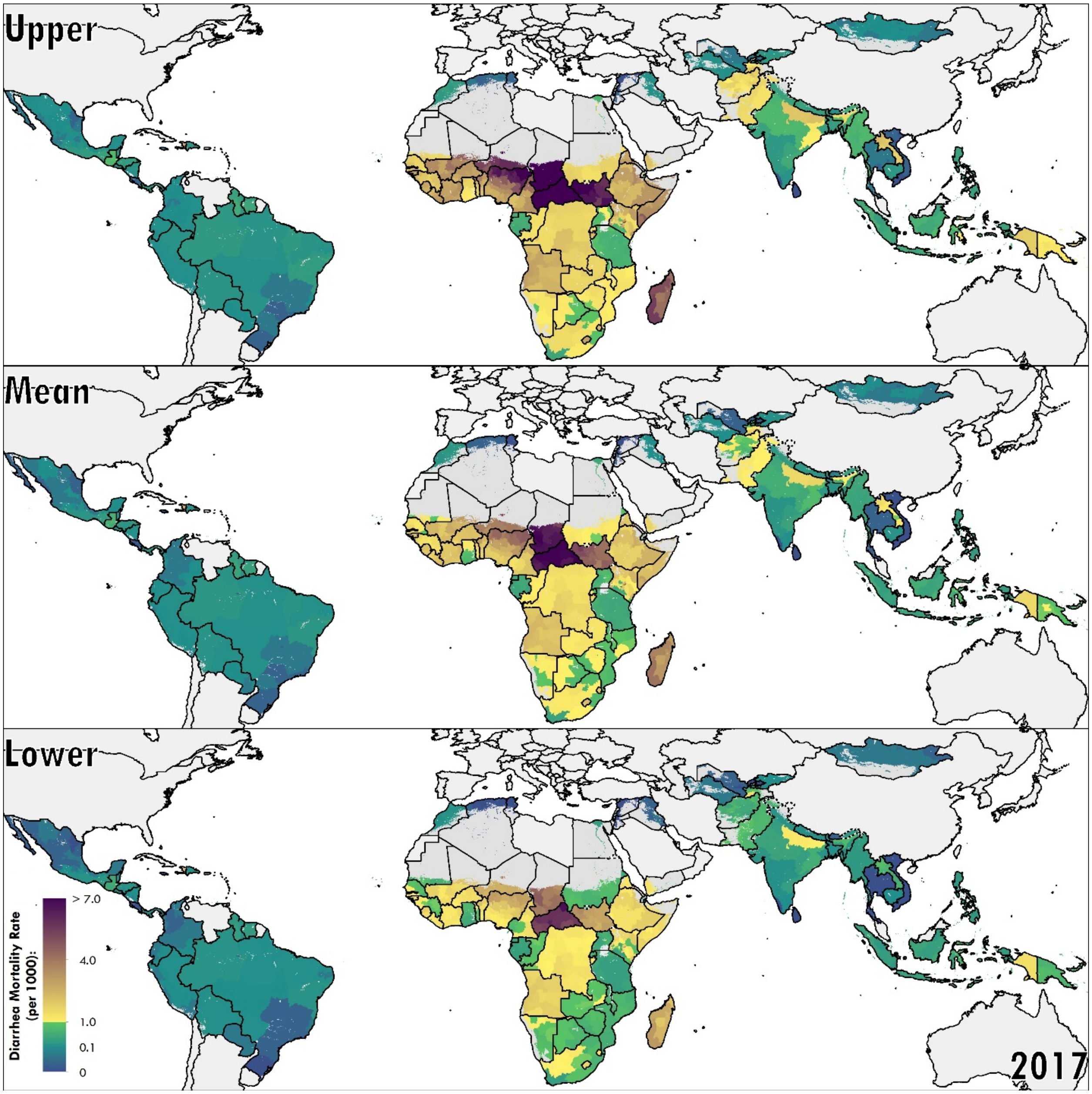

Appendix Figure 17. Prevalence of mild stunting in children under 5 at the second administrative level, 2017

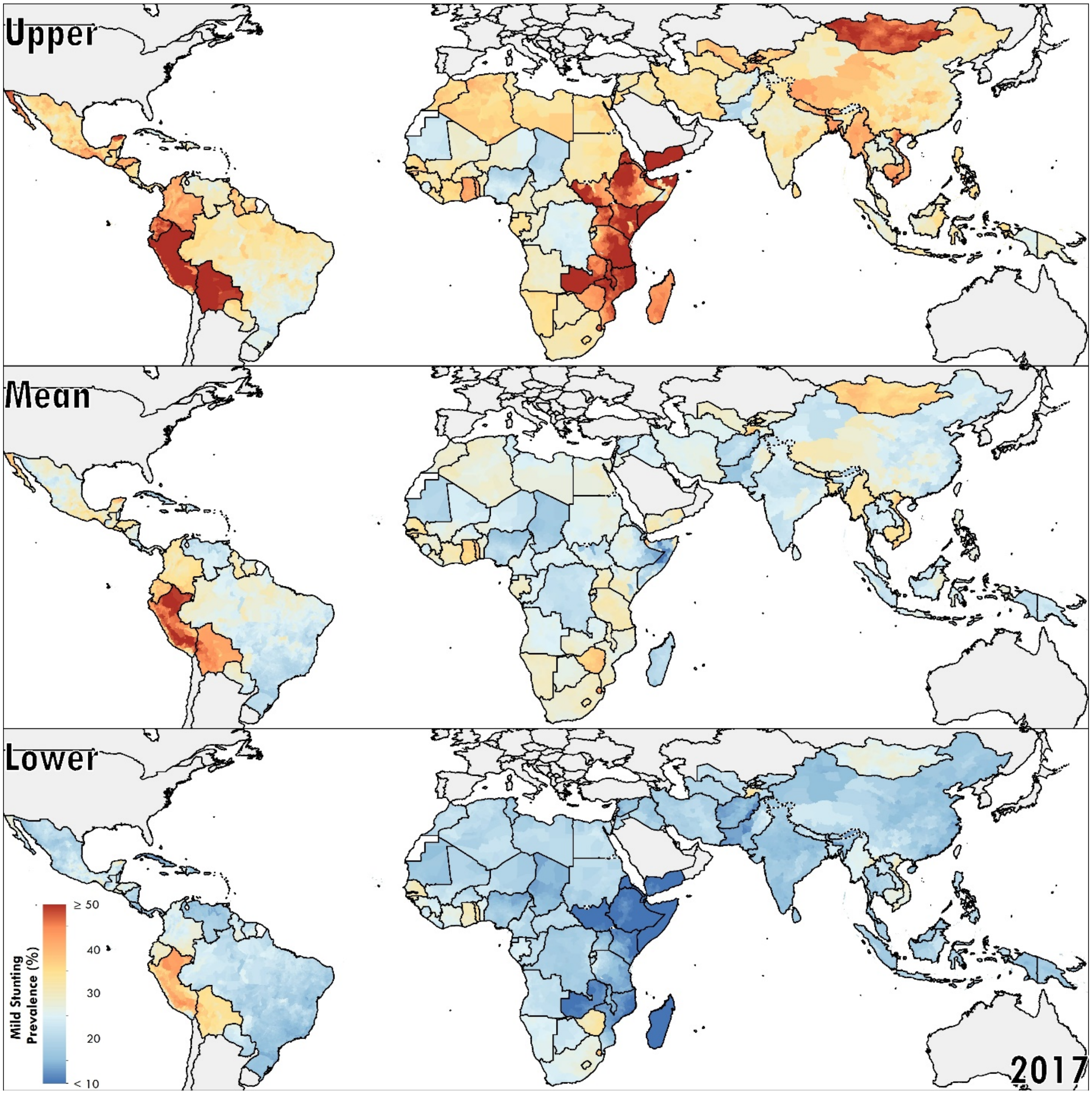

Appendix Figure 18. Prevalence of moderate stunting in children under 5 at the second administrative level, 2017

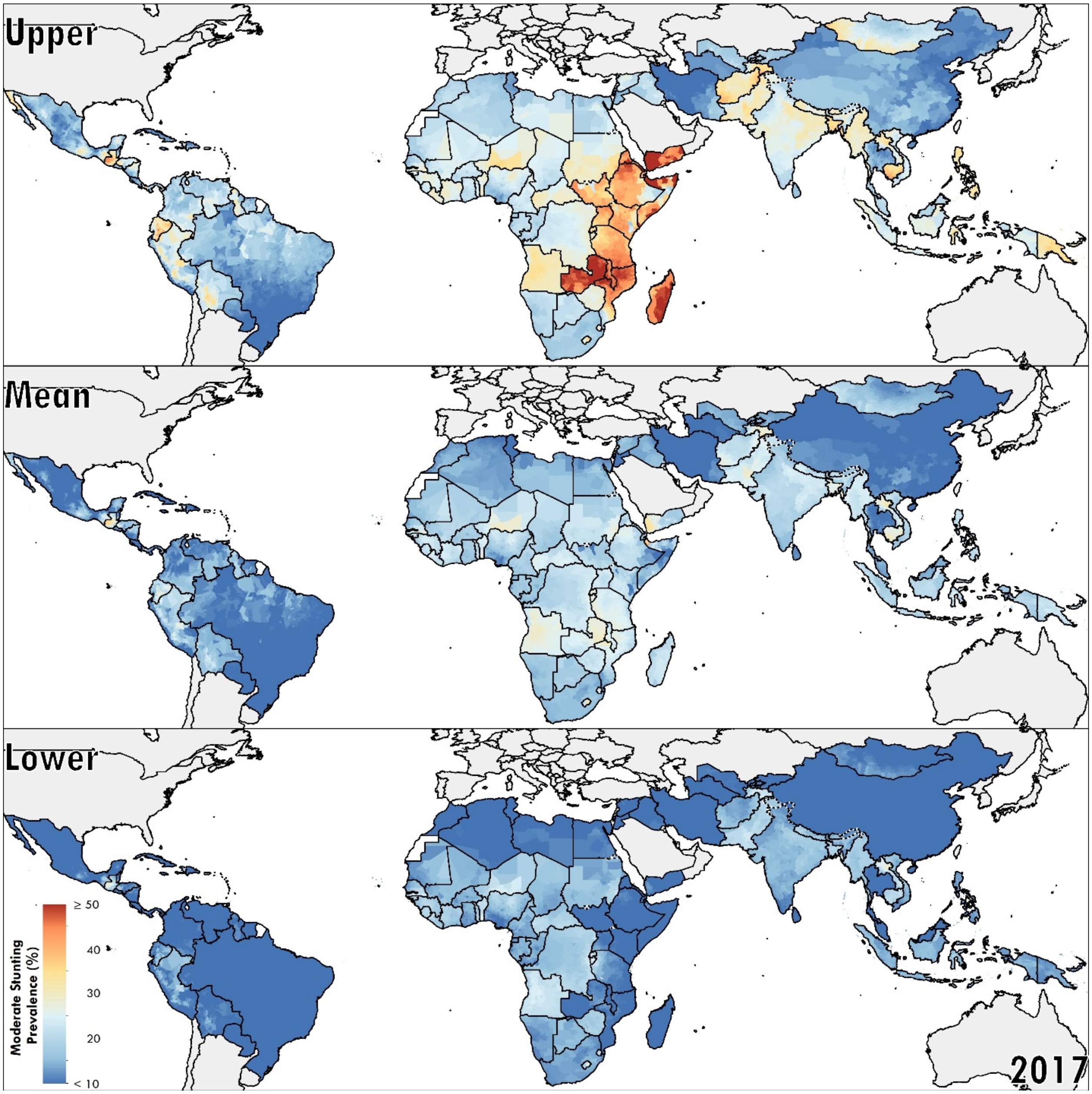

Appendix Figure 19. Prevalence of severe stunting in children under 5 at the second administrative level, 2017

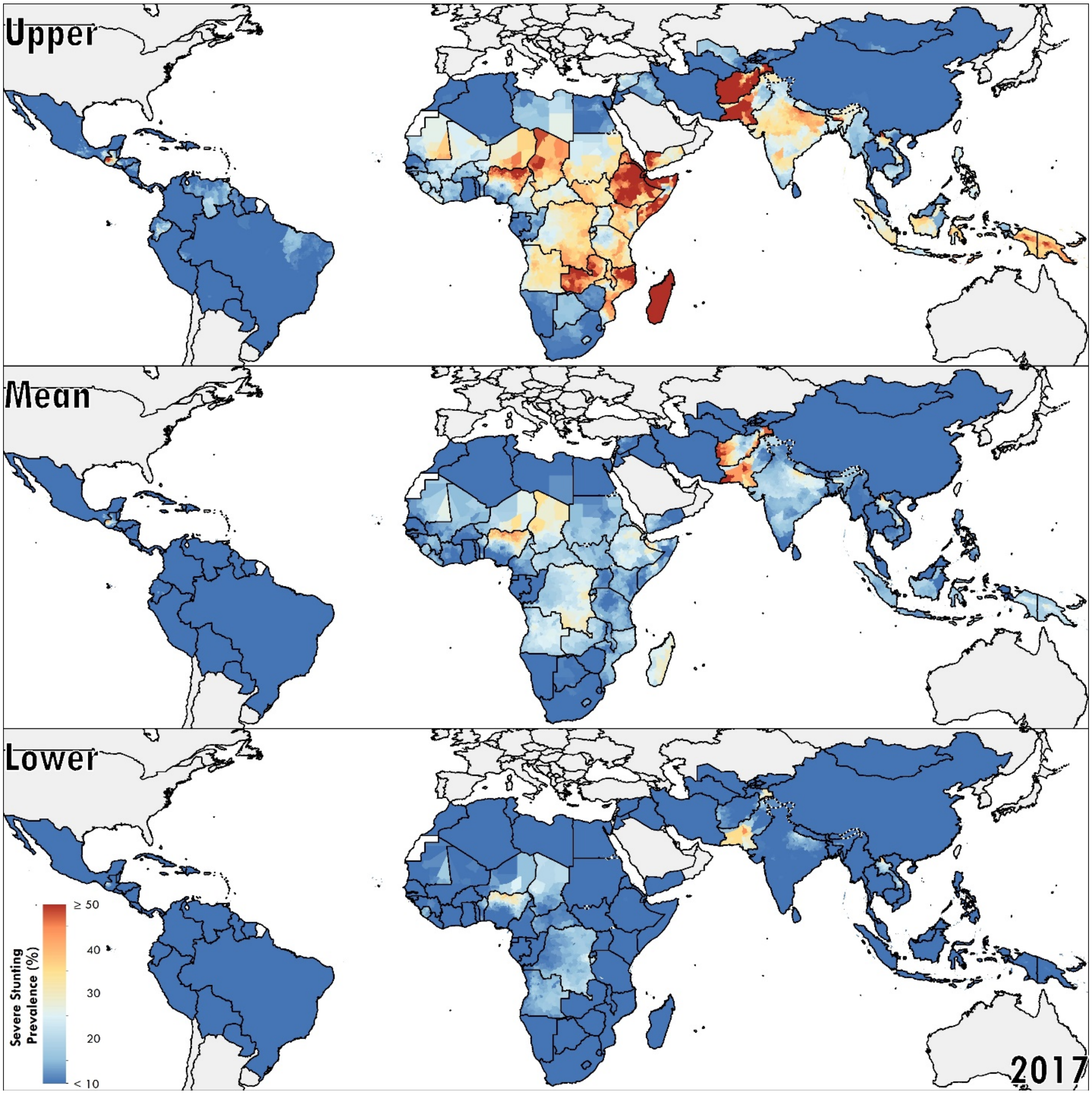

Appendix Figure 20. Prevalence of mild wasting in children under 5 at the second administrative level, 2017

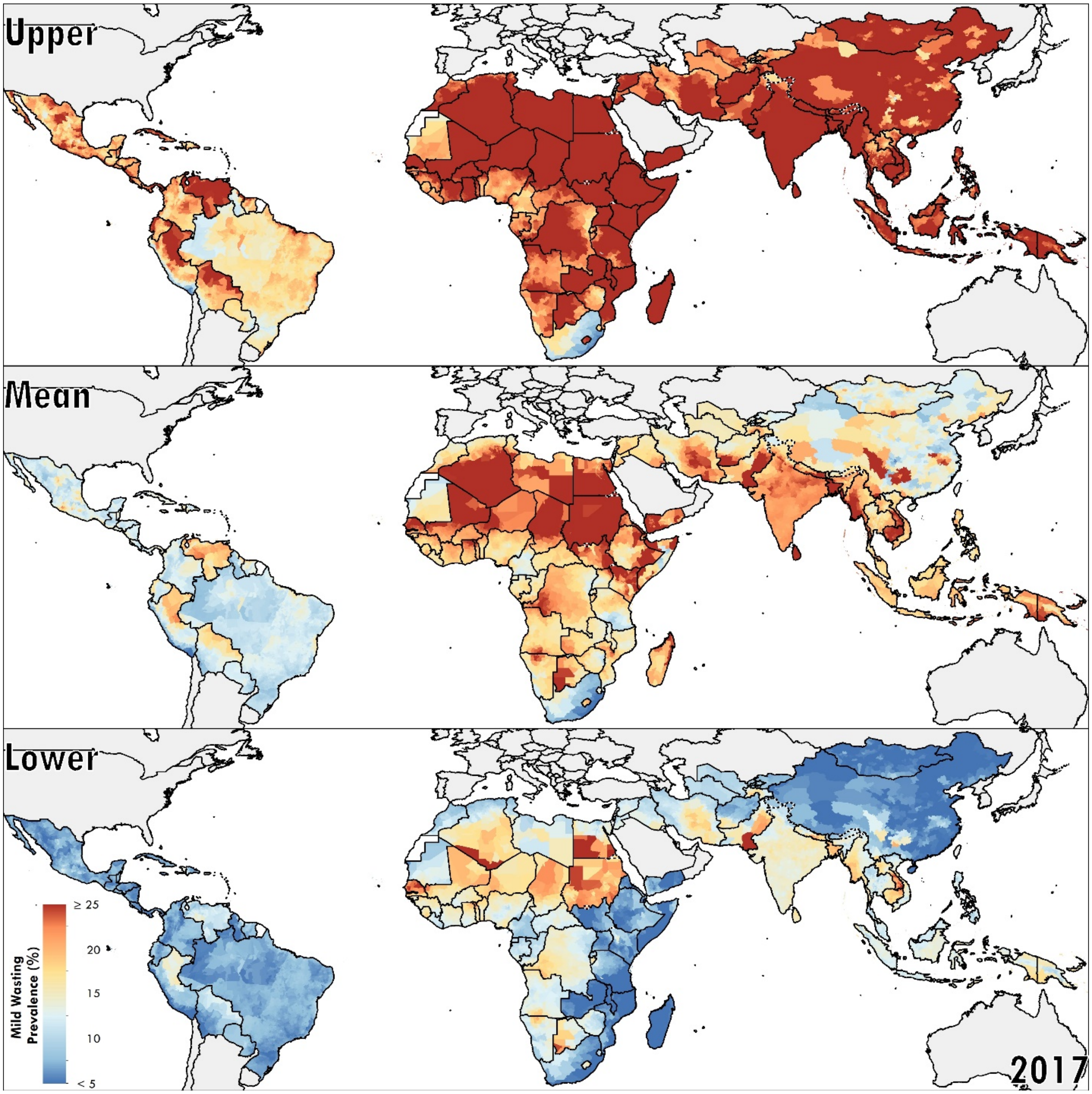

Appendix Figure 21. Prevalence of moderate wasting in children under 5 at the second administrative level, 2017

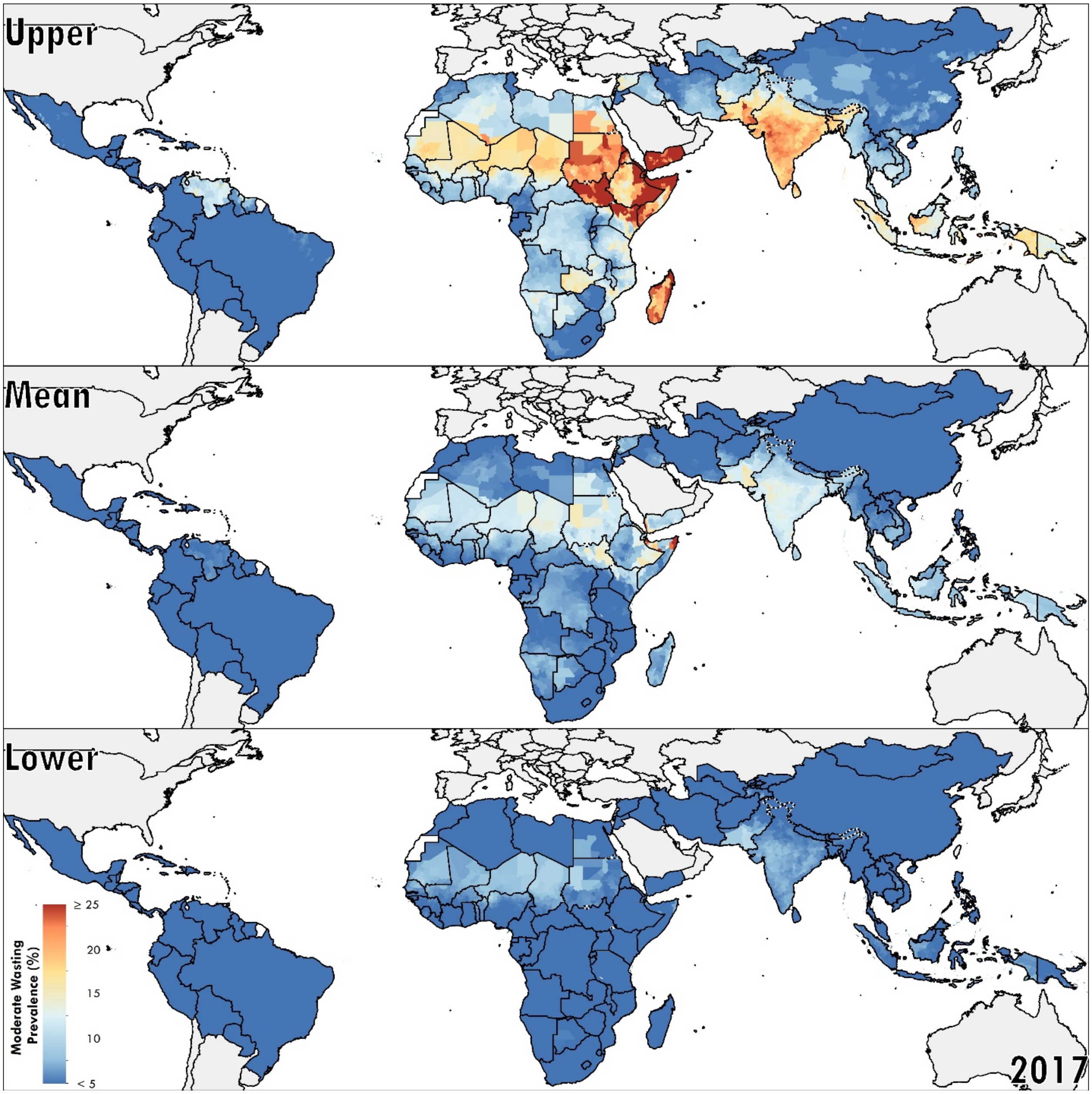

Appendix Figure 22. Prevalence of severe wasting in children under 5 at the second administrative level, 2017

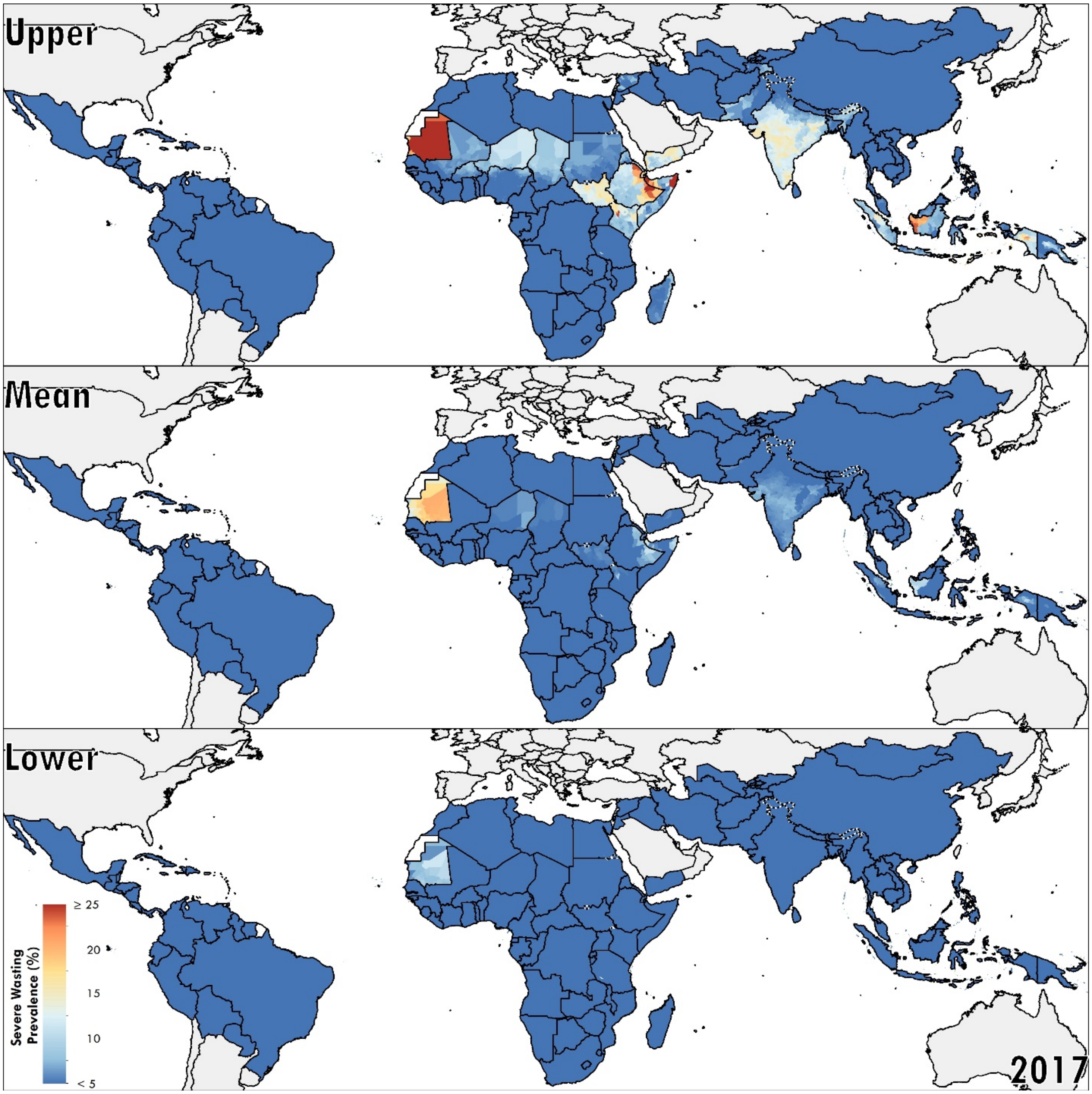

Appendix Figure 23. Lorenz curves of inequality for sub-Saharan Africa

(a–b) Lorenz curves calculated for mortality risk against population across second administrative units in sub-Saharan Africa for 2000 **(a)**, and 2017 **(b)**. Segments of the curve corresponding to units that are in the bottom 20% in 2000, 2017, or both are shaded light pink, magenta, or purple, respectively.

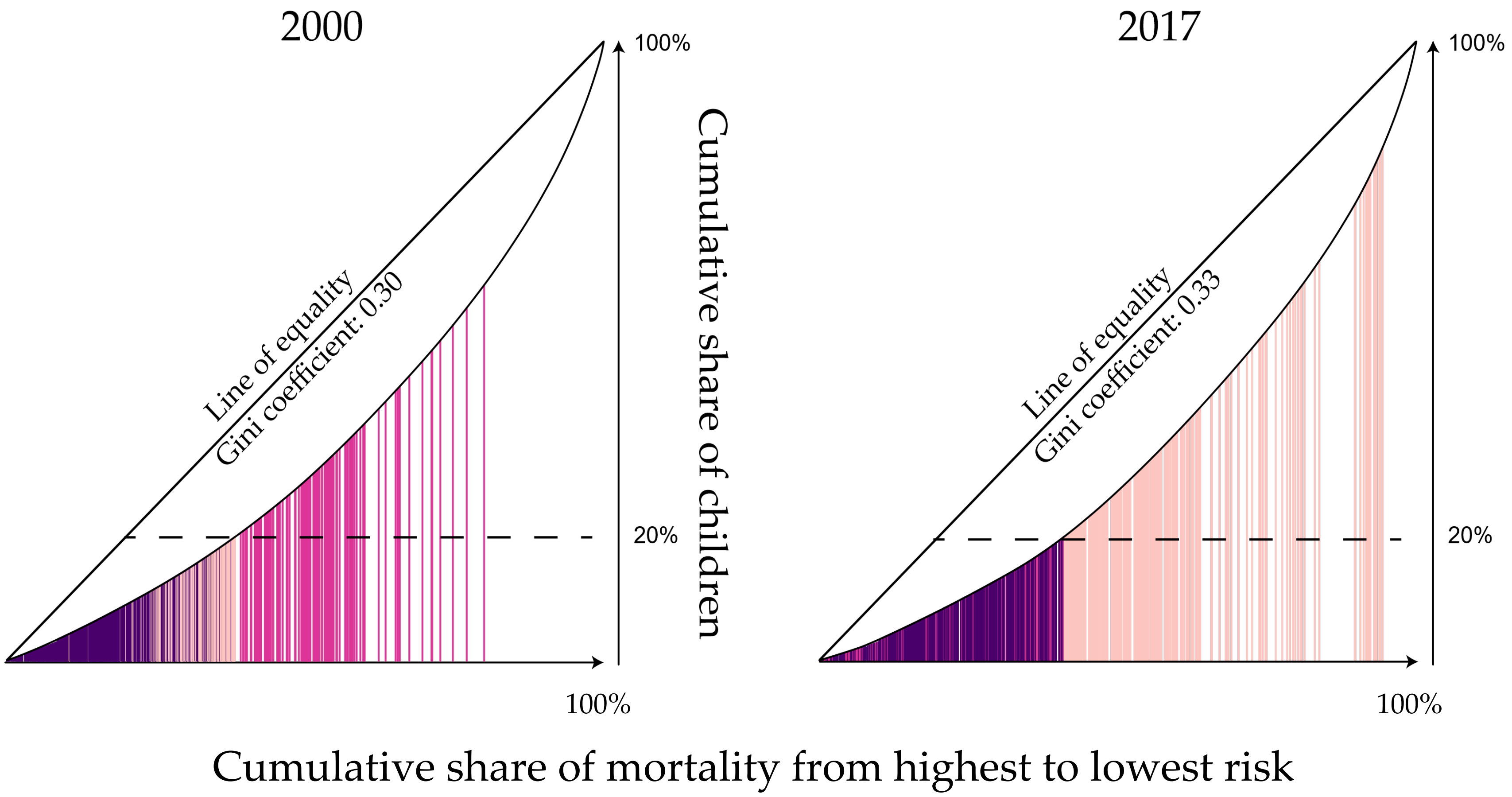

Appendix Figure 24. Incidence rate annualised rate of change

Maps reflect administrative boundaries, land cover, lakes, and population; grey-coloured grid cells were classified as "barren or sparsely vegetated" and had fewer than ten people per 1 × 1-km grid cell, or were not included in these analyses.[26–31]

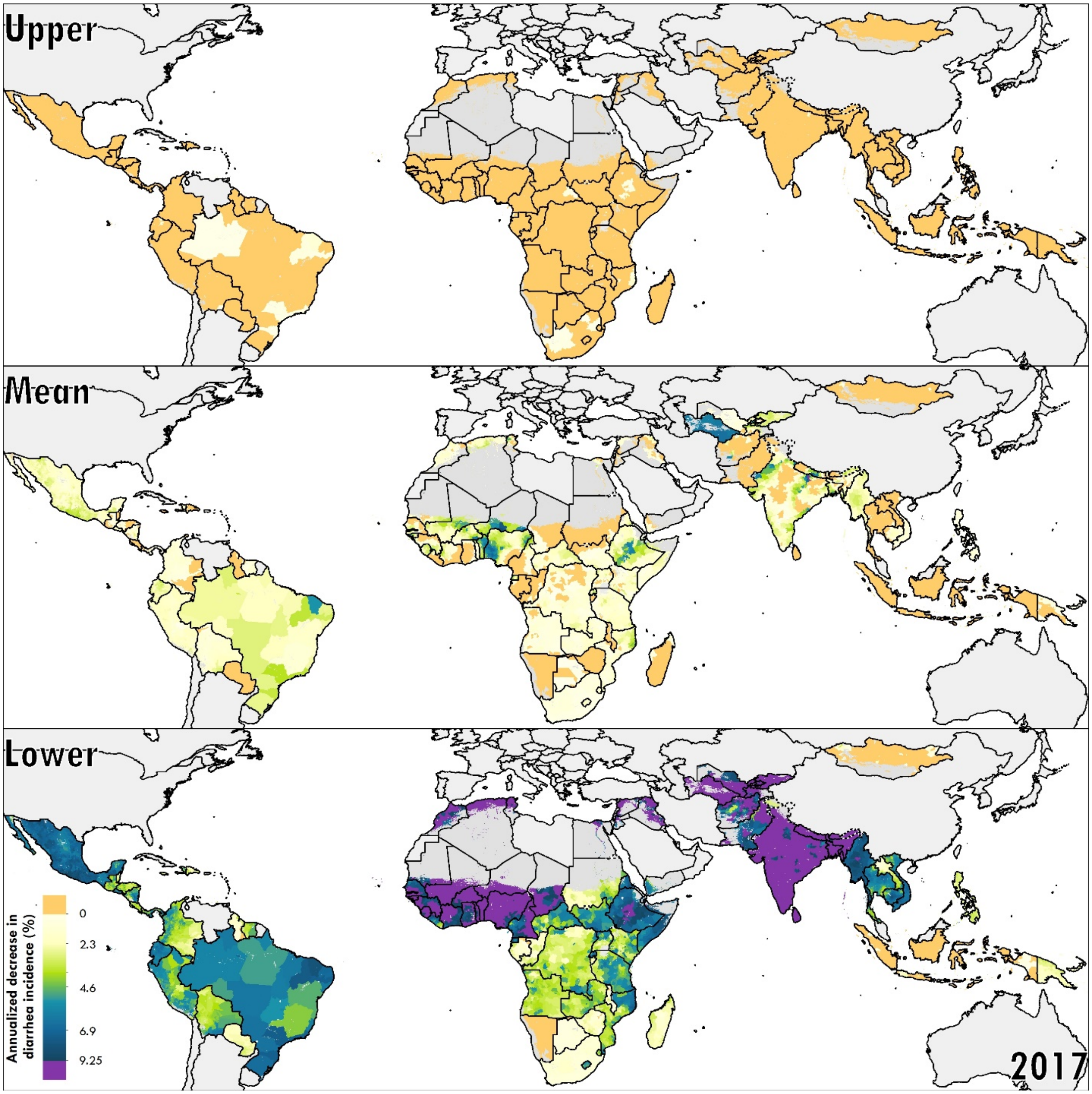

Appendix Figure 25. Mortality rate annualised rate of change

Maps reflect administrative boundaries, land cover, lakes, and population; grey-coloured grid cells were classified as "barren or sparsely vegetated" and had fewer than ten people per 1 × 1-km grid cell, or were not included in these analyses.[26–31]

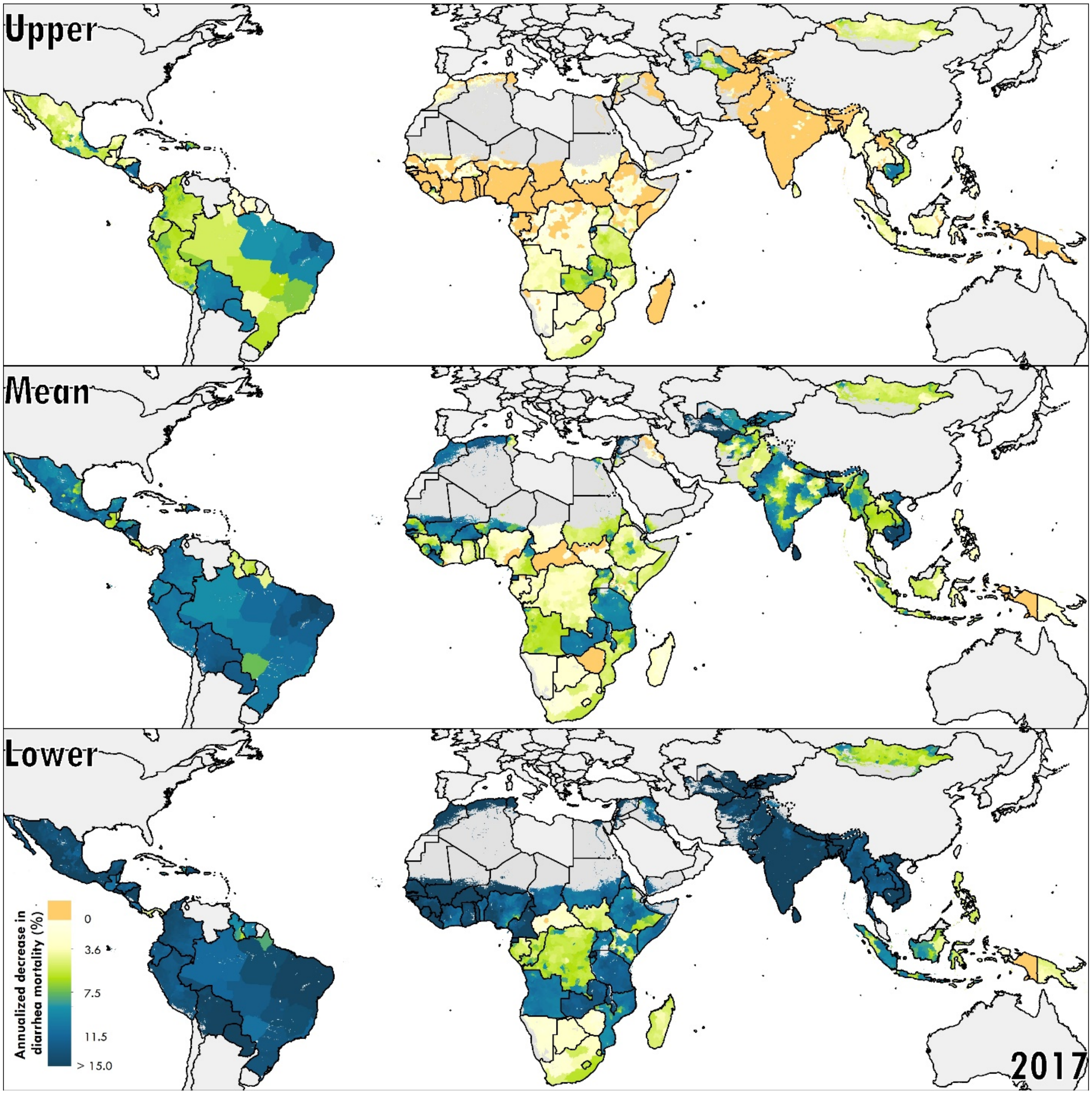

Appendix Figure 26. Averted diarrhoeal deaths in 2017 attributable to improvements in water and sanitation, child growth failure, and oral rehydration solution implemented from 2000 to 2017

(a) Number of deaths averted per 1,000 children. (b) Number of total deaths averted. (c) Number of deaths averted per 1,000 children with colour scale driven by dominant driver. (d) Number of total deaths averted with colour scale driven by dominant driver. The risk factor contributing the majority of the reduction is indicated as water and sanitation=blue, child growth failure=purple, oral rehydration solution=pink, none=gold. Deaths averted were calculated in the same manner as described in Section 6.5, with oral rehydration solution (ORS) added as an additional risk factor.. Maps reflect administrative boundaries, land cover, lakes, and population; grey-coloured grid cells were classified as “barren or sparsely vegetated” and had fewer than ten people per 1 × 1-km grid cell, or were not included in these analyses.

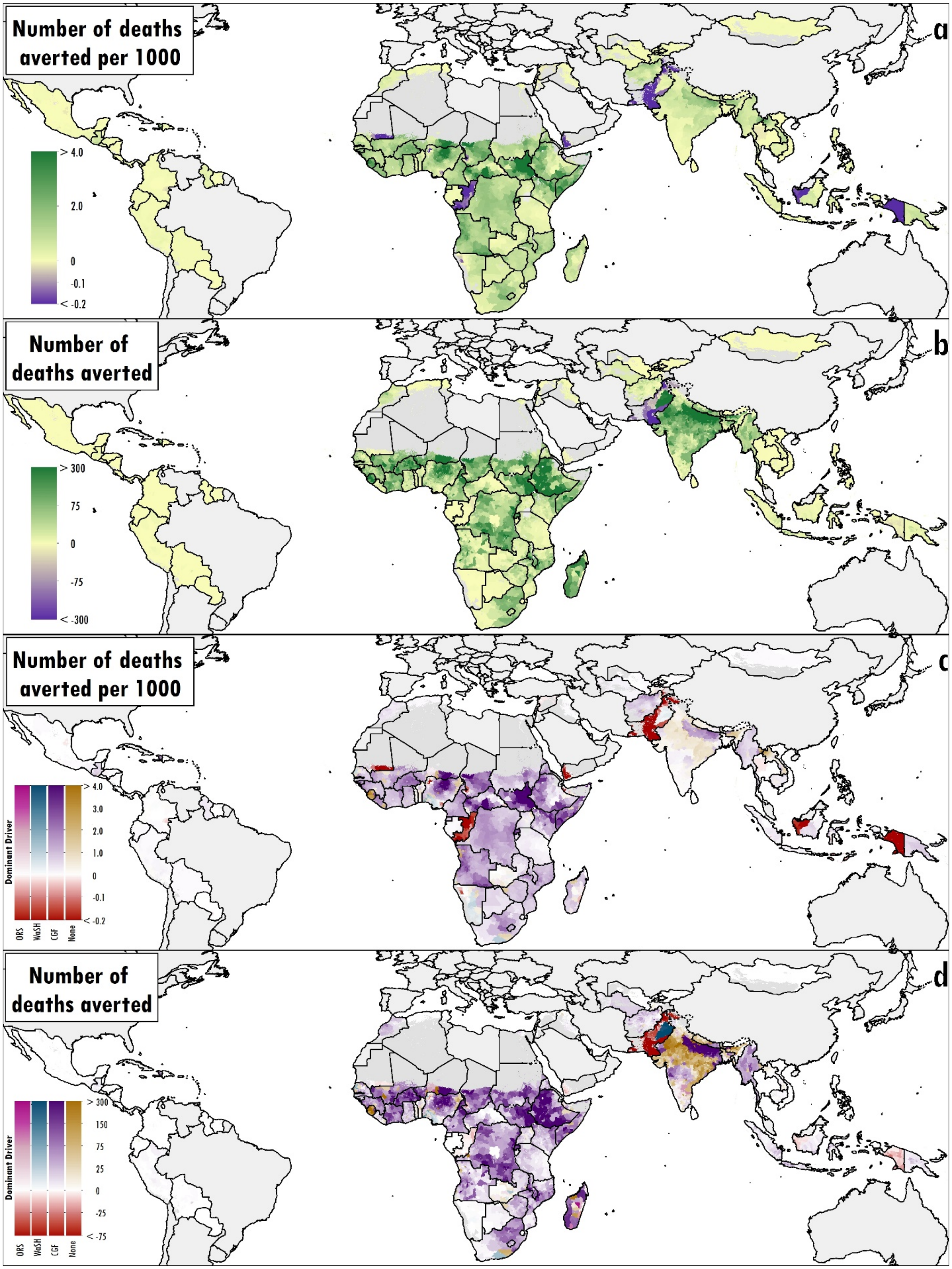

Number of deaths averted per 1000
a
> 4.0
2.0
0
-0.1
< -0.2
Number of deaths averted
b
> 300
75
0
-75
< -300
Number of deaths averted per 1000
c
Dominant Driver
ORS
WaSH
CGF
None
> 4.0
3.0
2.0
1.0
0
-0.1
< -0.2
Number of deaths averted
d
Dominant Driver
ORS
WaSH
CGF
None
> 300
150
75
25
0
-25
< -75

Appendix Figures 27–35. In-sample validation plots

Each plot shows diarrhoea prevalence estimates from the survey data on the x-axis and mean posterior predictions of diarrhoea prevalence prior to calibration to GBD estimates on the y-axis. The size of each dot is proportional to sample size in the underlying data. Estimates are shown aggregated to country, first administrative, and second administrative levels. Estimates are shown across all regions and years, as well as stratified by region and by year. For corresponding in-sample fit statistics see Appendix Table 9a–f.

Regions are labelled in the following manner: the horn of Africa [*dia_afr_horn*], central Asia [*dia_central_asia*], central sub-Saharan Africa [*dia_cssa*], eastern sub-Saharan Africa [*dia_essa*], Malay Archipelago [*dia_malay*], Mexico, the Caribbean, and central America [*dia_mcaca*], the Middle East [*dia_mid_east*], north Africa Middle East [*dia_name*], South America [*dia_s_america*], southeast Asia [*dia_se_asia*], south Asia [*dia_south_asia-ind*], southern sub-Saharan Africa [*dia_sssa*], western sub-Saharan Africa [*dia_wssa*], and India [*IND*].

Appendix Figure 27. In-sample validation plot of diarrhoea by country

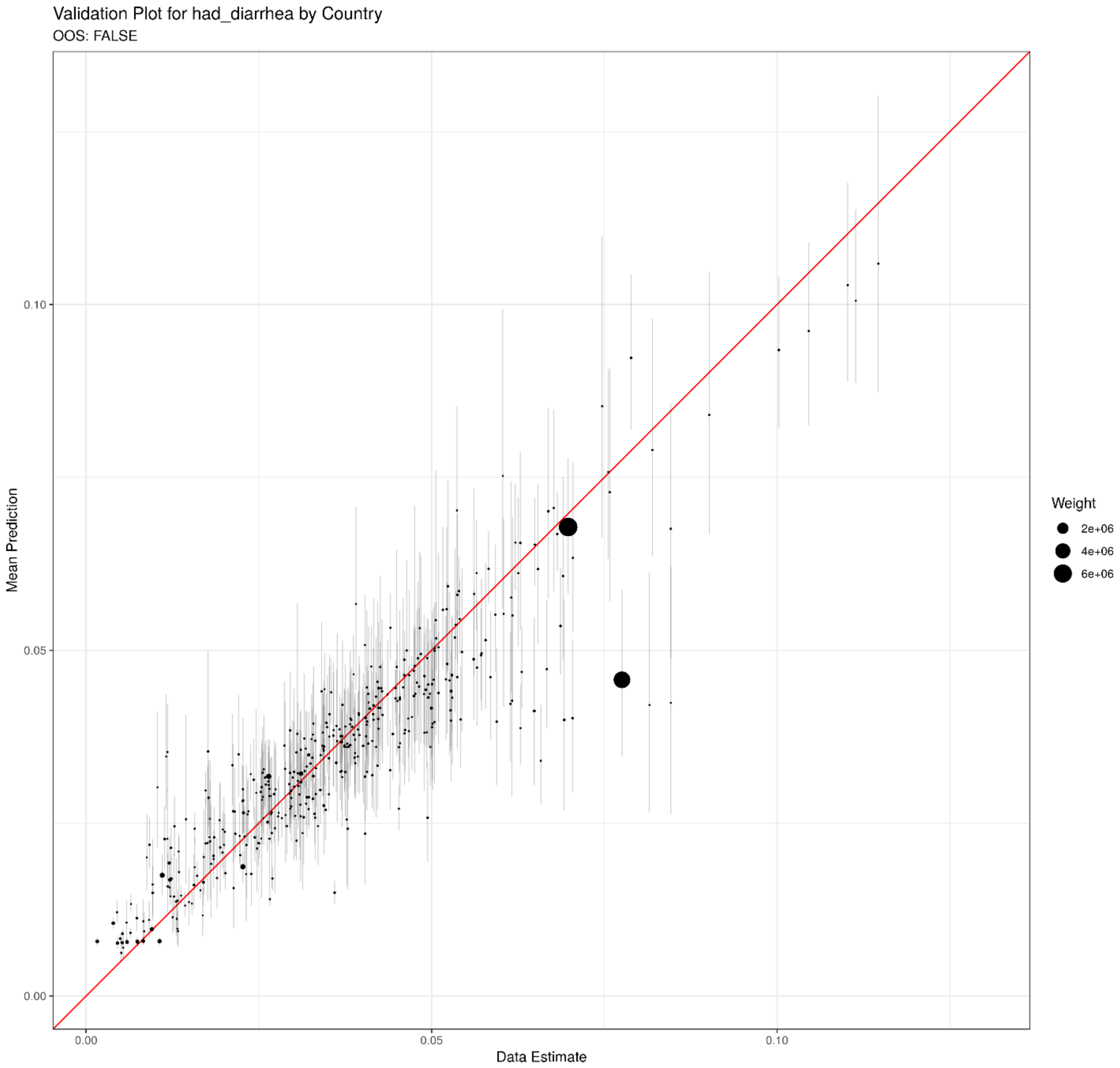

Appendix Figure 28. In-sample validation plot of diarrhoea by first administrative unit

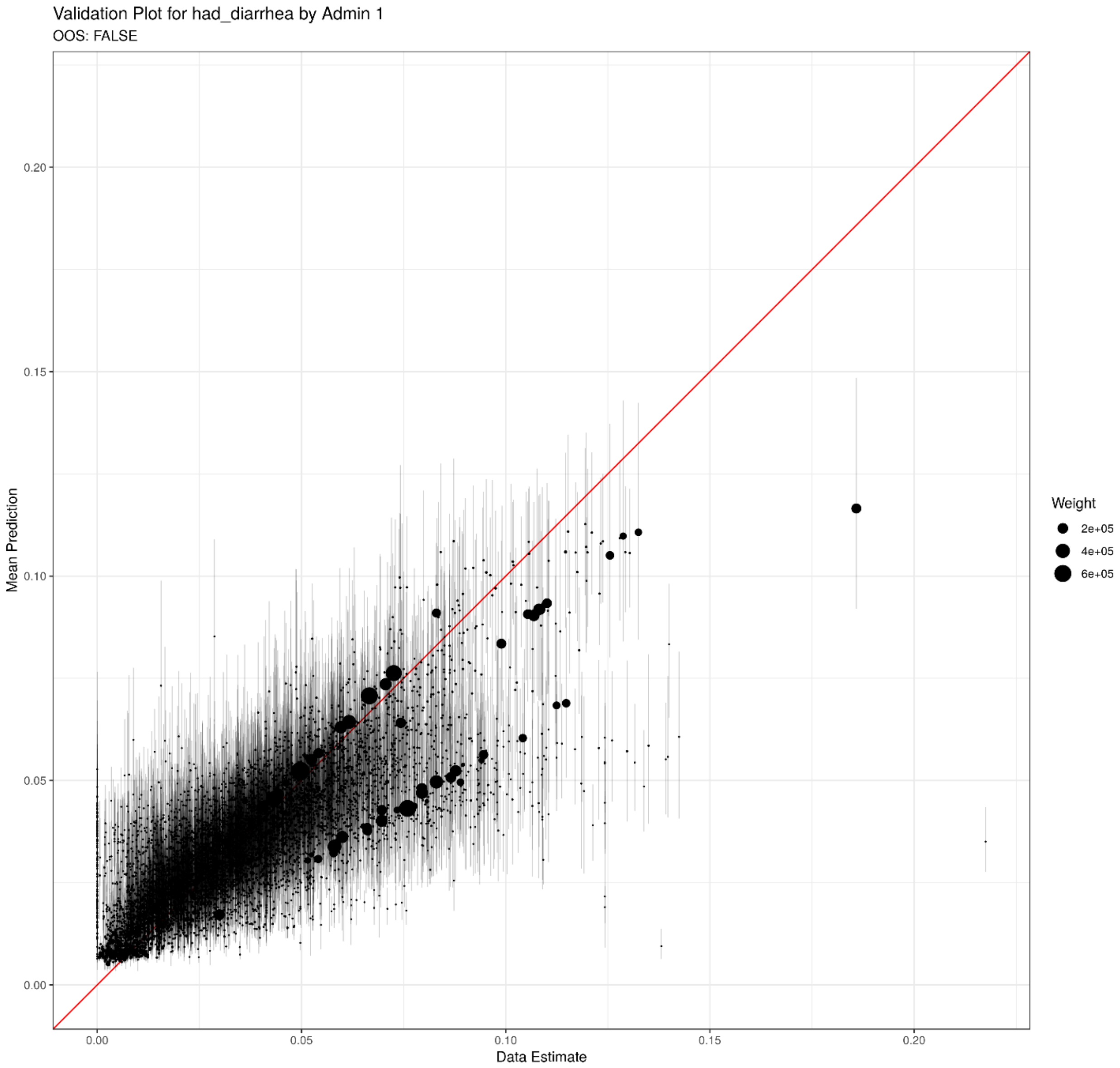

Appendix Figure 29. In-sample validation plot of diarrhoea by second administrative unit

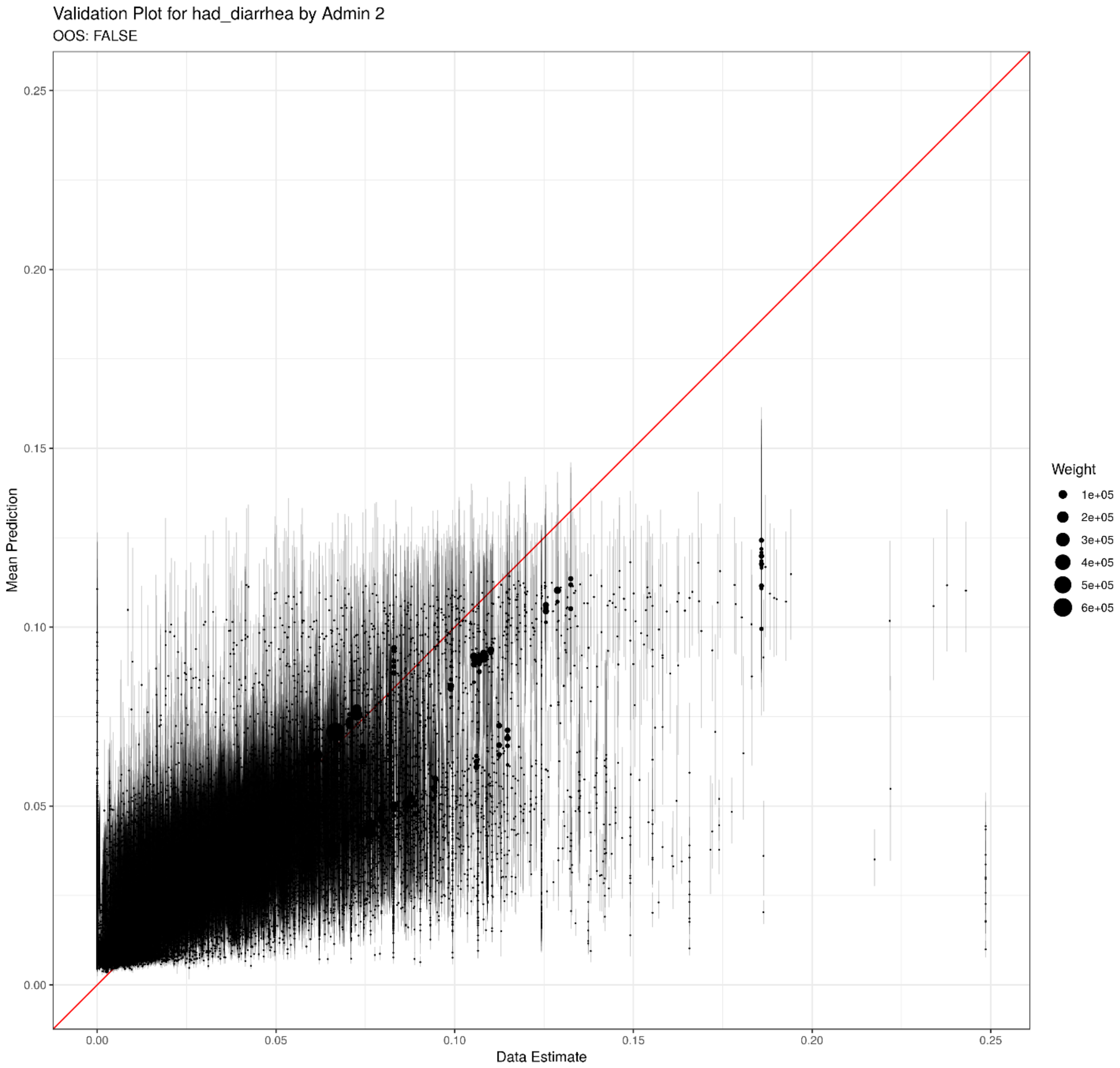

Appendix Figure 30. In-sample validation plot of diarrhoea by country and modelling region

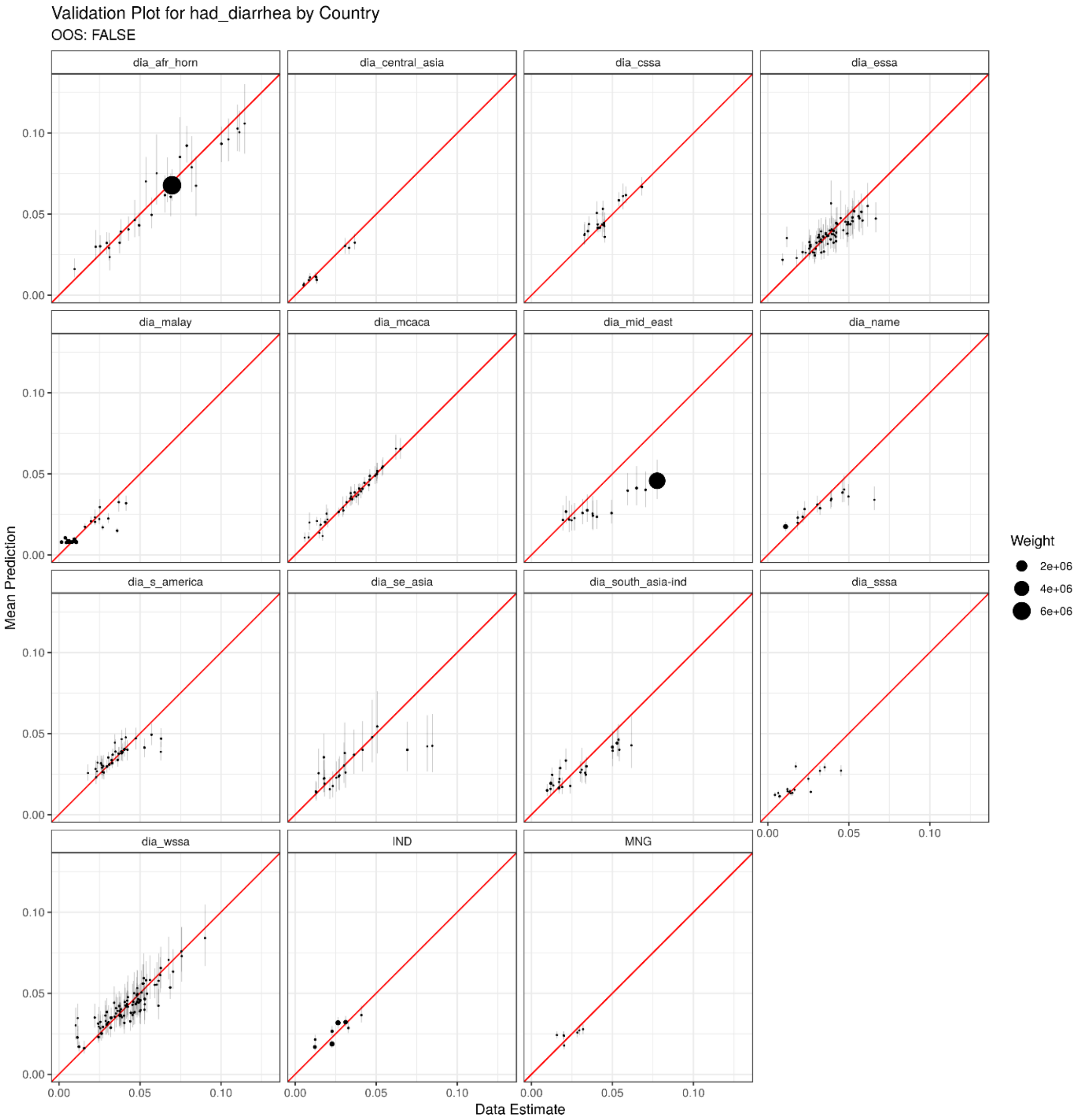

Appendix Figure 31. In-sample validation plot of diarrhoea by first administrative unit and modelling region

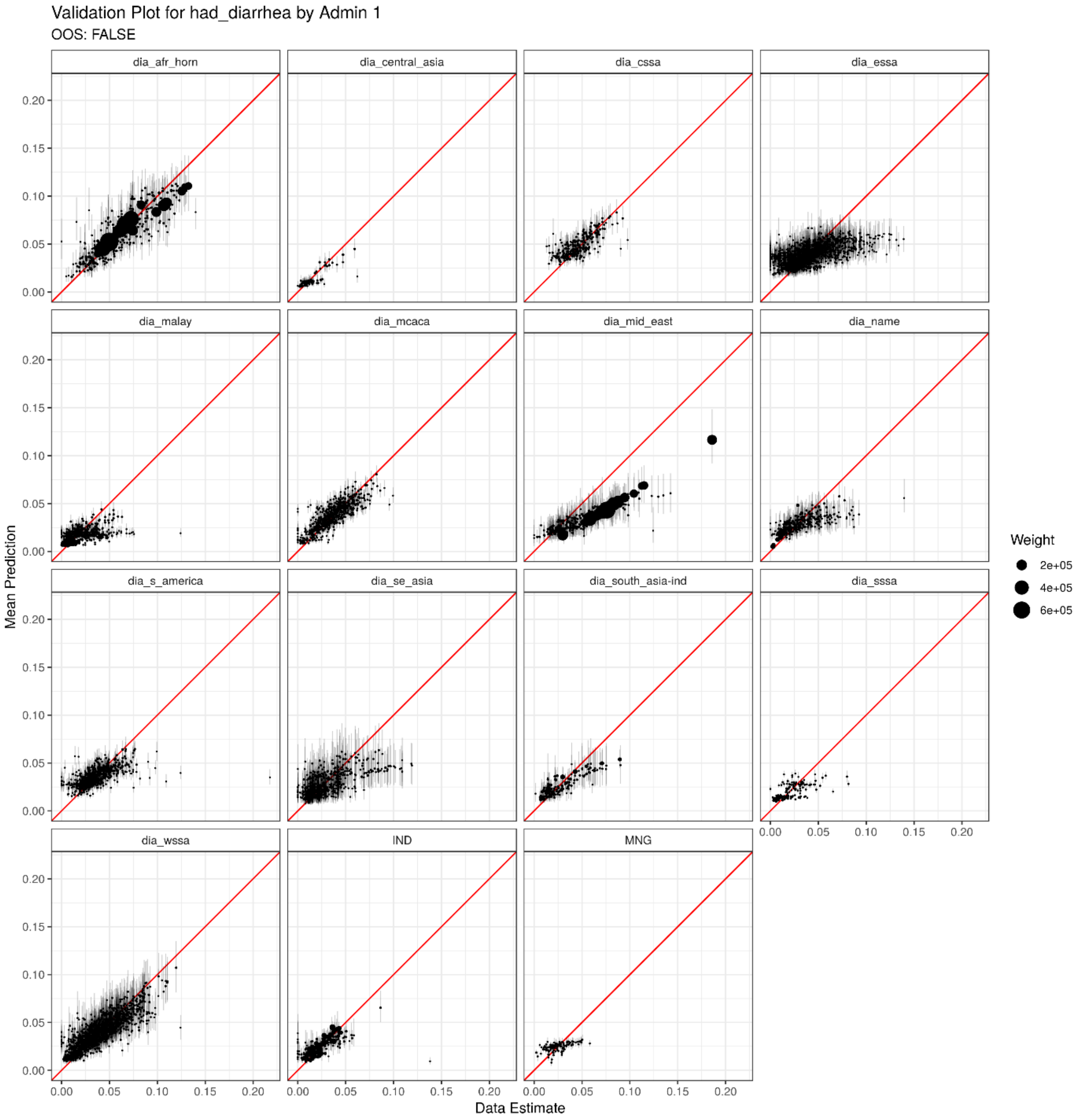

Appendix Figure 32. In-sample validation plot of diarrhoea by second administrative unit and modelling region

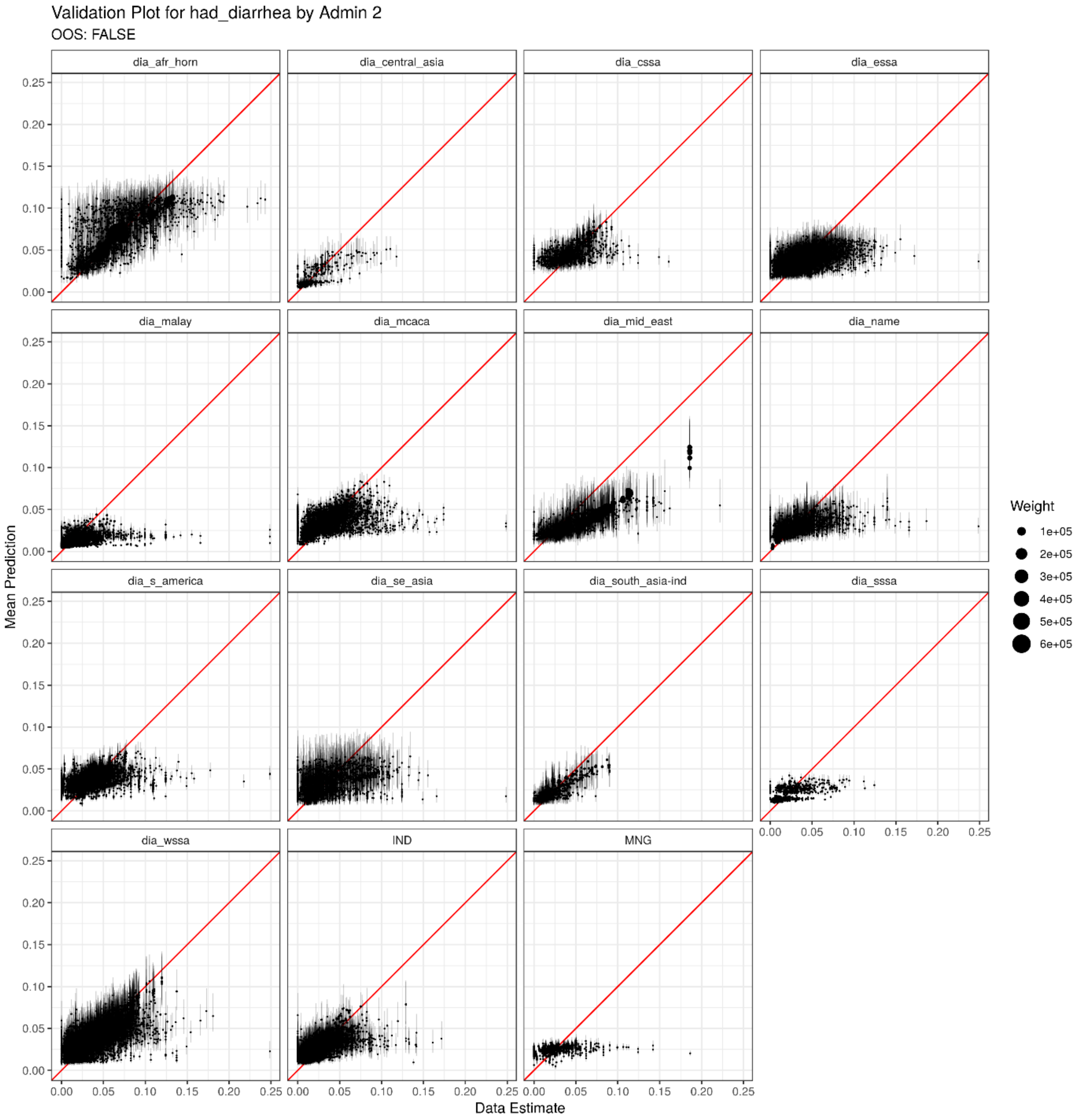

Appendix Figure 33. In-sample validation plot of diarrhoea by country and year

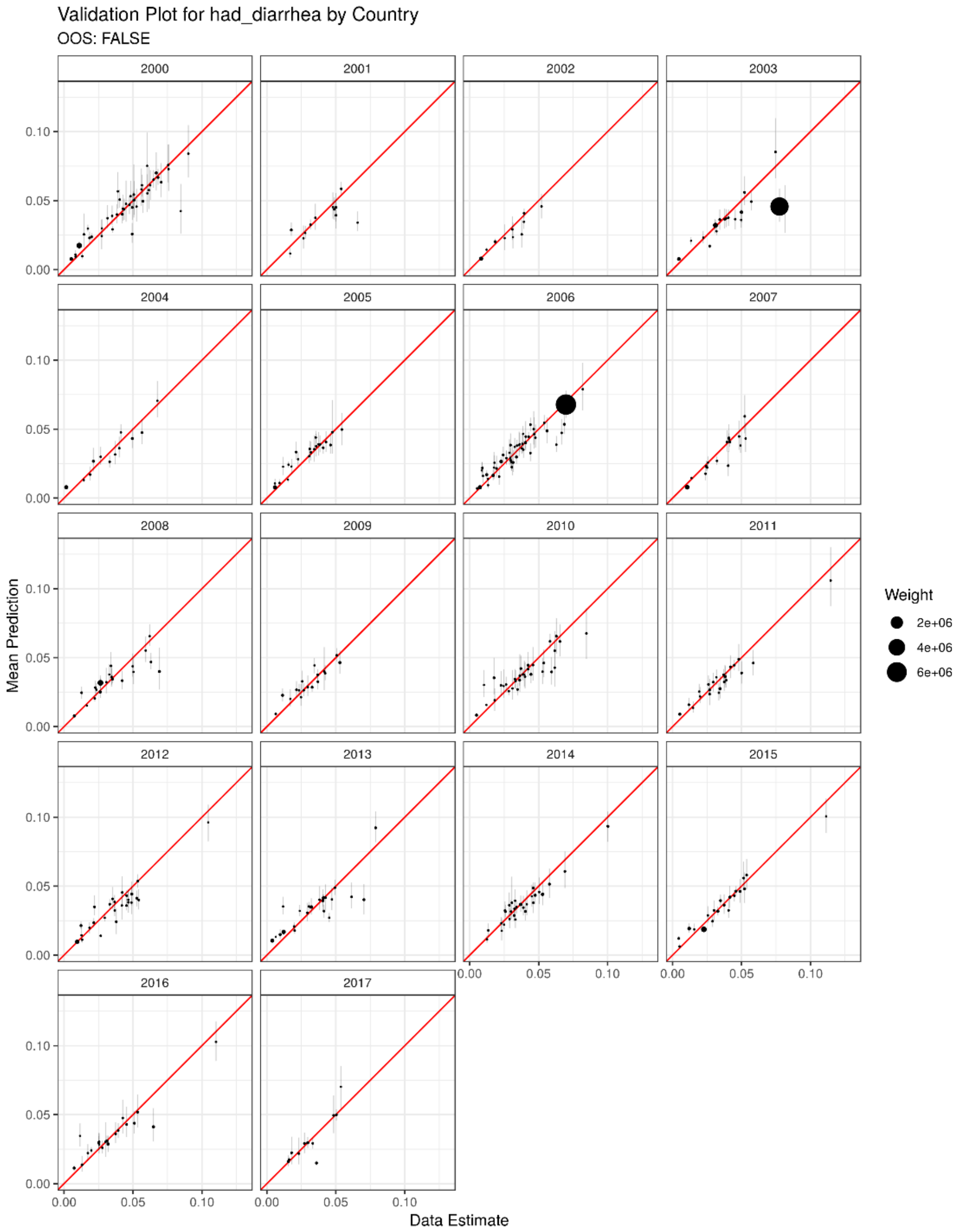

Appendix Figure 34. In-sample validation plot of diarrhoea by first administrative unit and year

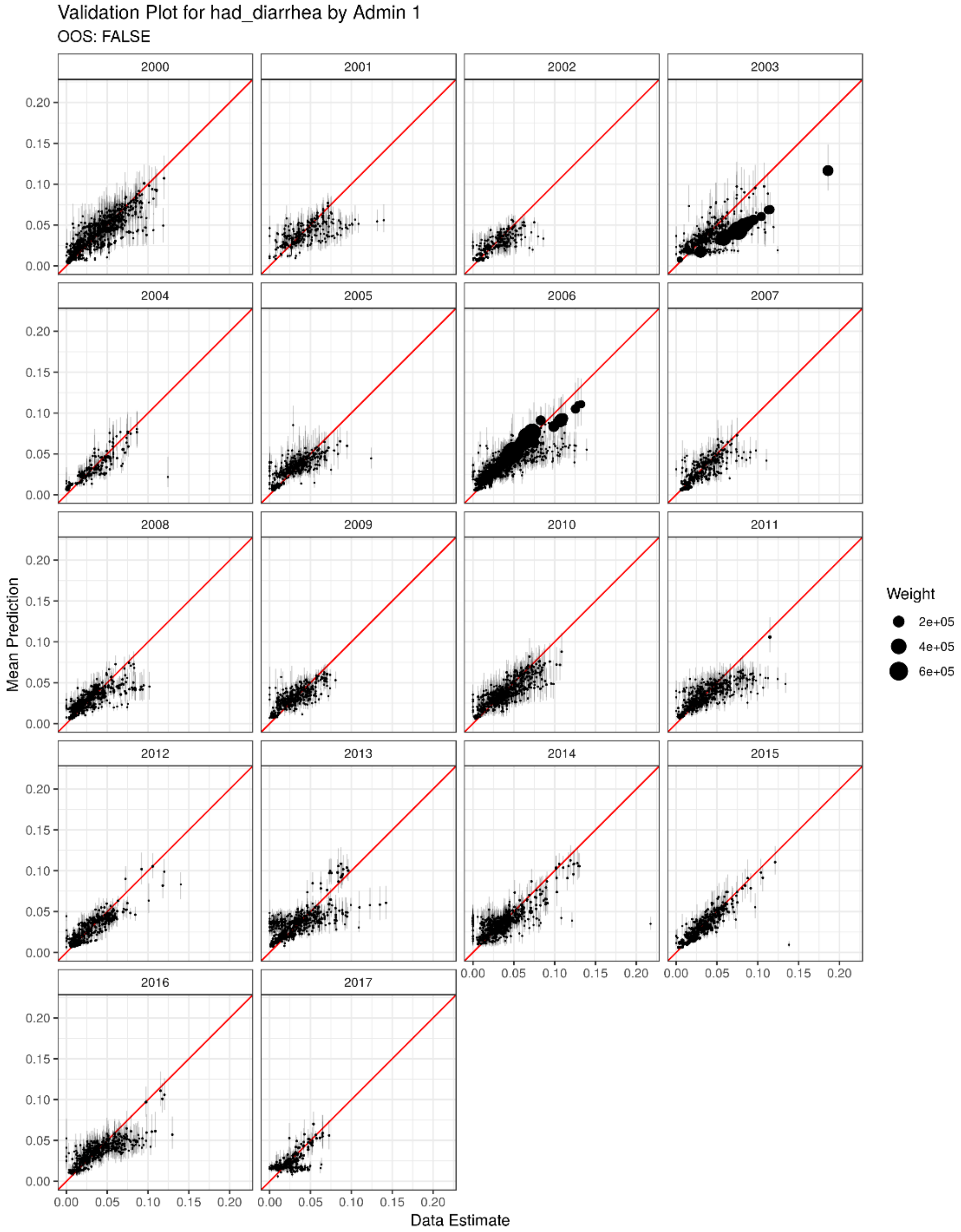

Appendix Figure 35. In-sample validation plot of diarrhoea by second administrative unit and year

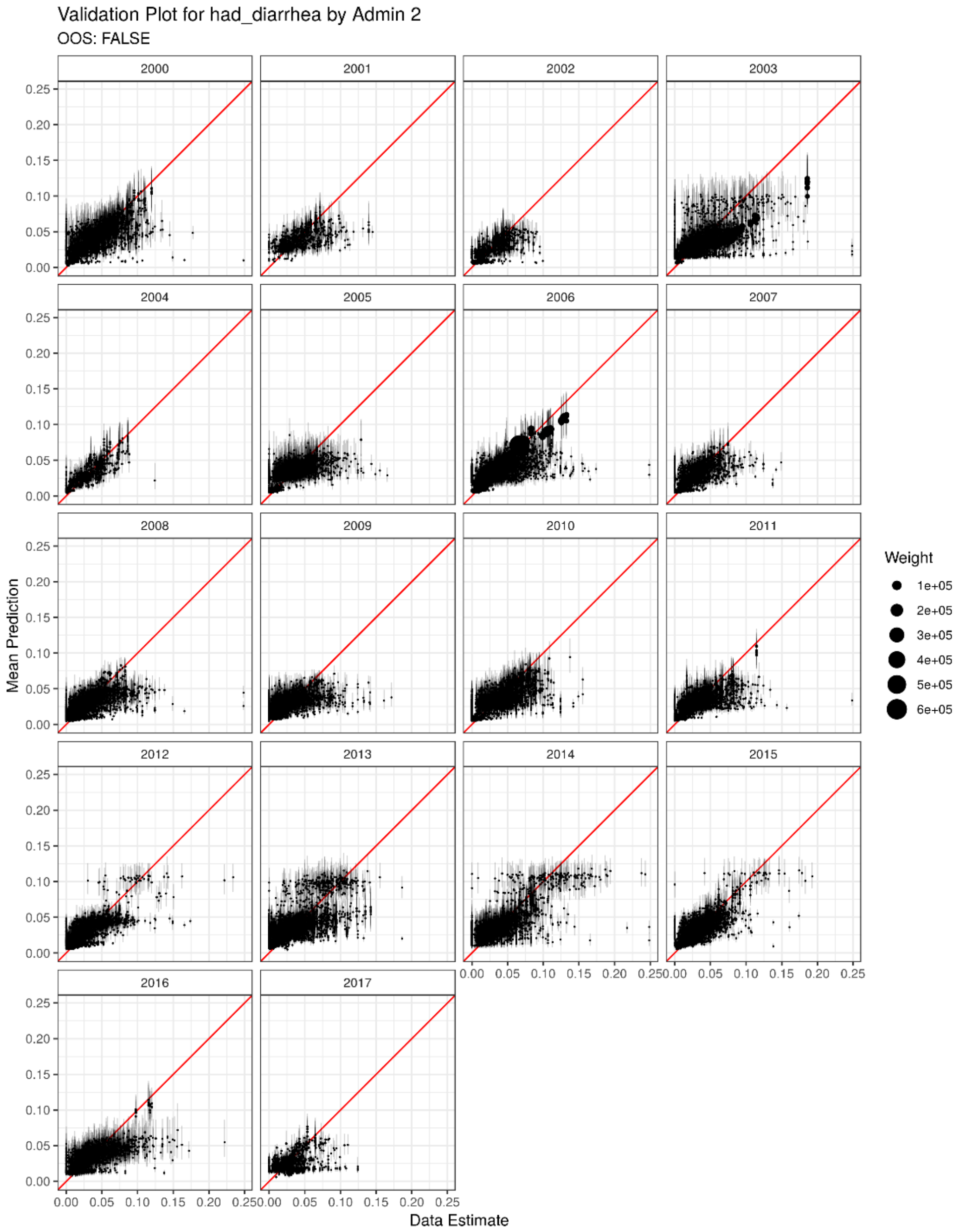

Figures 36–44. Out-of-sample validation plots

Each plot shows diarrhoea prevalence estimates from the survey data on the x-axis and mean posterior predictions of diarrhoea prevalence prior to calibration to GBD estimates on the y-axis. The size of each dot is proportional to sample size in the underlying data. Estimates are shown aggregated to country, first administrative, and second administrative levels. Estimates are shown across all regions and years, as well as stratified by region and by year. For corresponding out-of-sample fit statistics see Appendix Table 10a–f.

Regions are labelled in the following manner: the horn of Africa [*dia_afr_horn*], central Asia [*dia_central_asia*], central sub-Saharan Africa [*dia_cssa*], eastern sub-Saharan Africa [*dia_essa*], Malay Archipelago [*dia_malay*], Mexico, the Caribbean, and central America [*dia_mcaca*], the Middle East [*dia_mid_east*], north Africa Middle East [*dia_name*], South America [*dia_s_america*], southeast Asia [*dia_se_asia*], south Asia [*dia_south_asia-ind*], southern sub-Saharan Africa [*dia_sssa*], western sub-Saharan Africa [*dia_wssa*], and India [*IND*].

Appendix Figure 36. Out-of-sample validation plot of diarrhoea by country

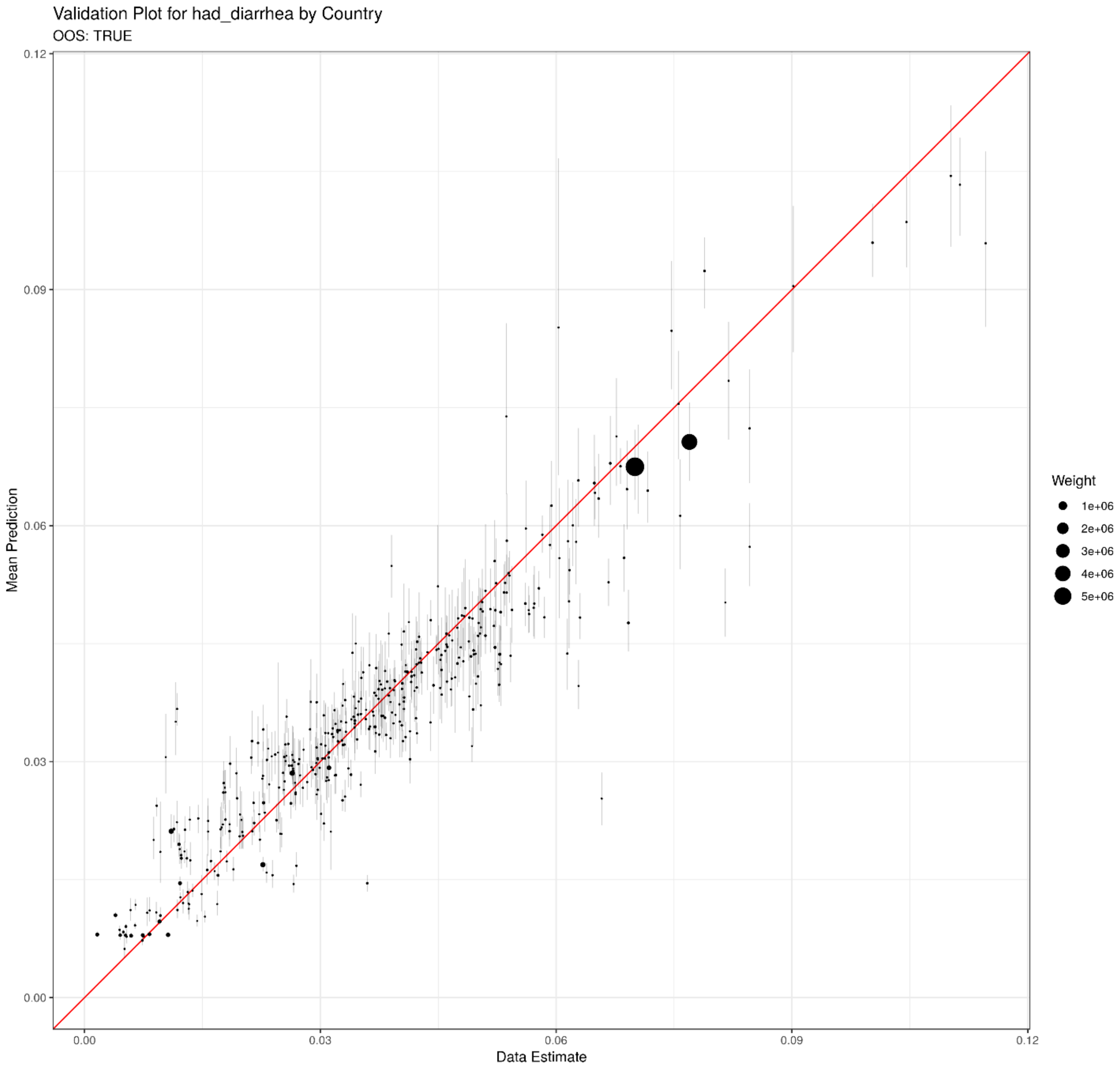

Appendix Figure 37. Out-of-sample validation plot of diarrhoea by first administrative unit

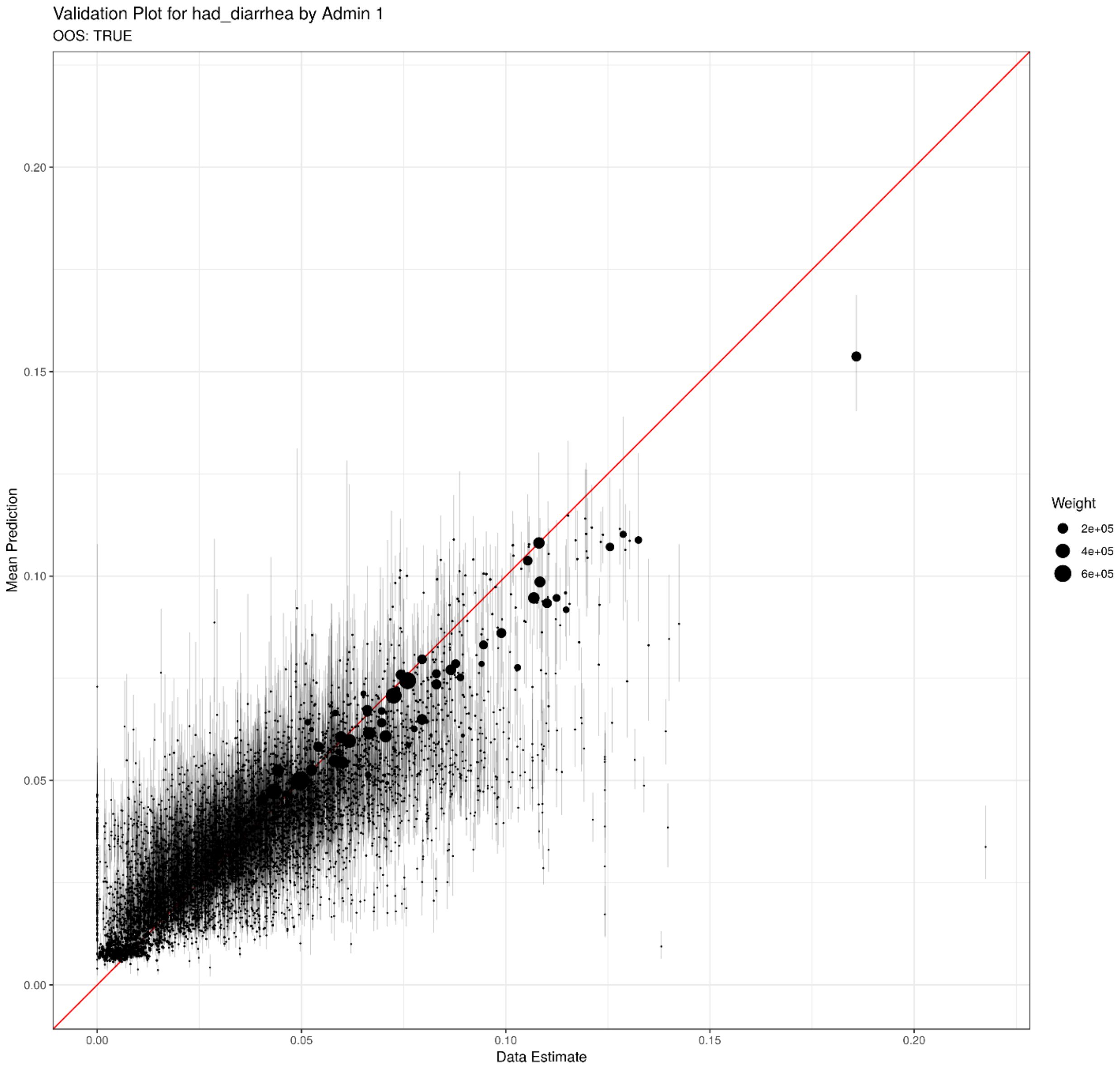

Appendix Figure 38. Out-of-sample validation plot of diarrhoea by second administrative unit

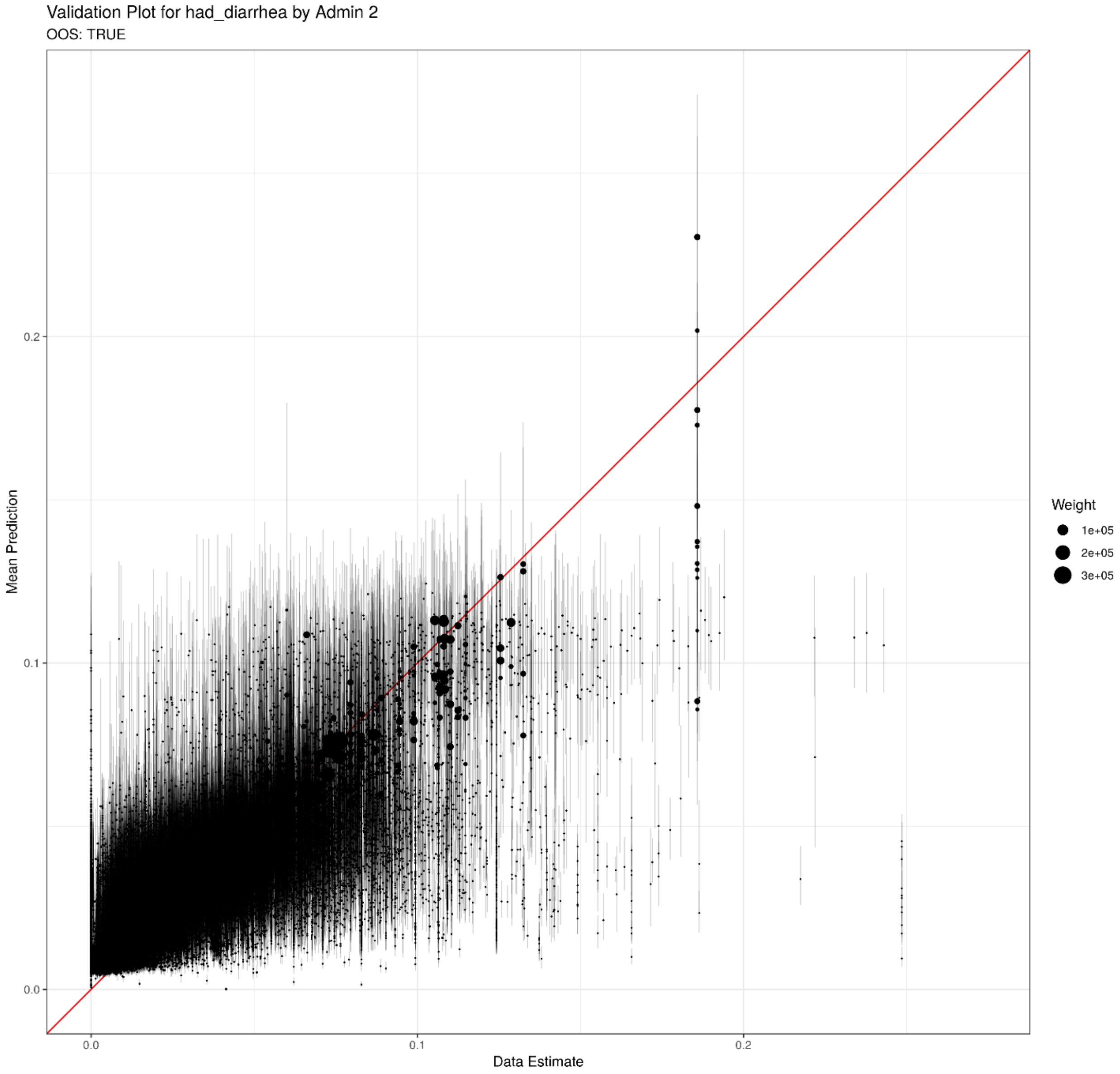

Appendix Figure 39. Out-of-sample validation plot of diarrhoea by country and modelling region

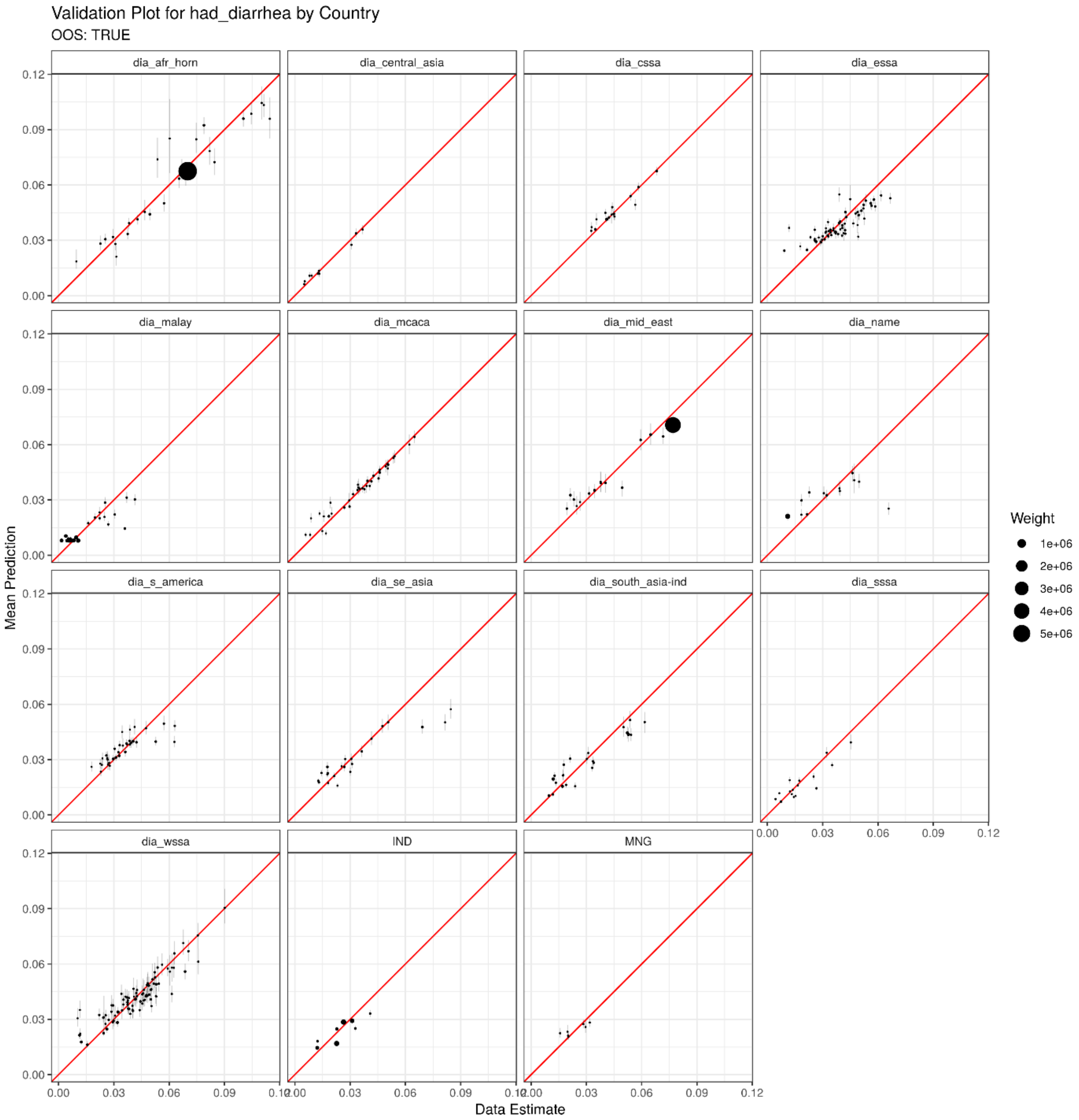

Appendix Figure 40. Out-of-sample validation plot of diarrhoea by first administrative unit and modelling region

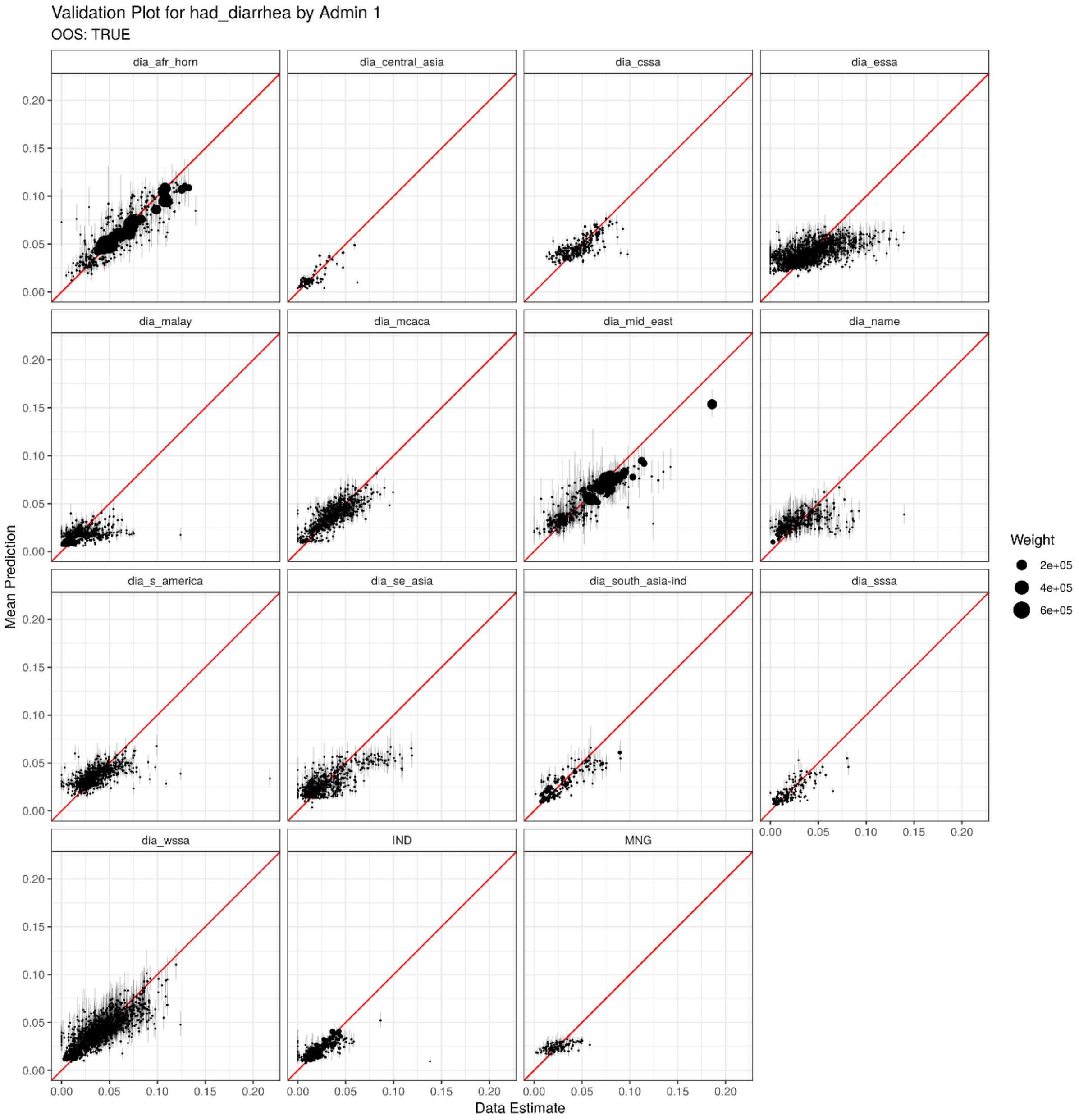

Appendix Figure 41. Out-of-sample validation plot of diarrhoea by second administrative unit and modelling region

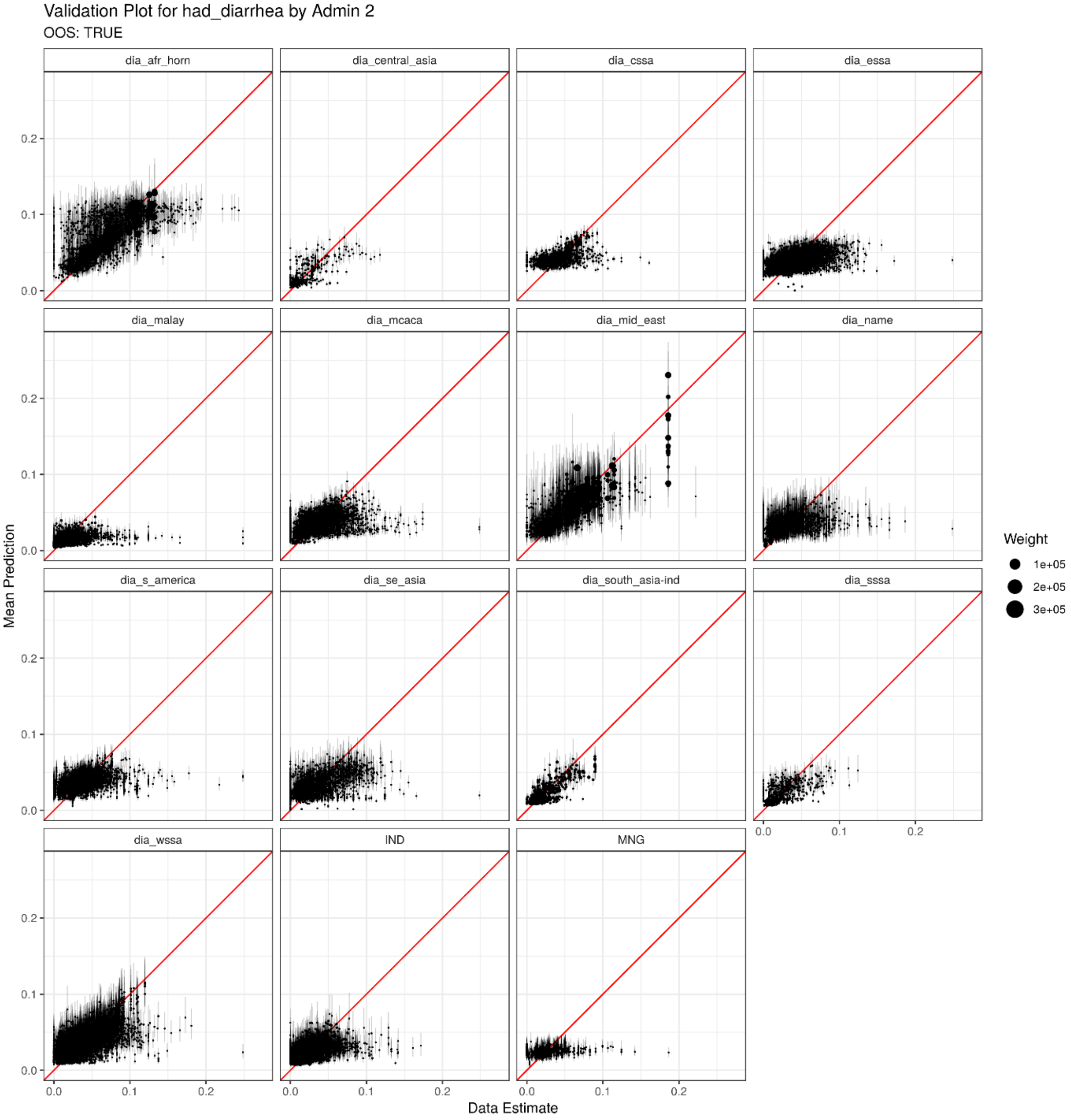

Appendix Figure 42. Out-of-sample validation plot of diarrhoea by country and year

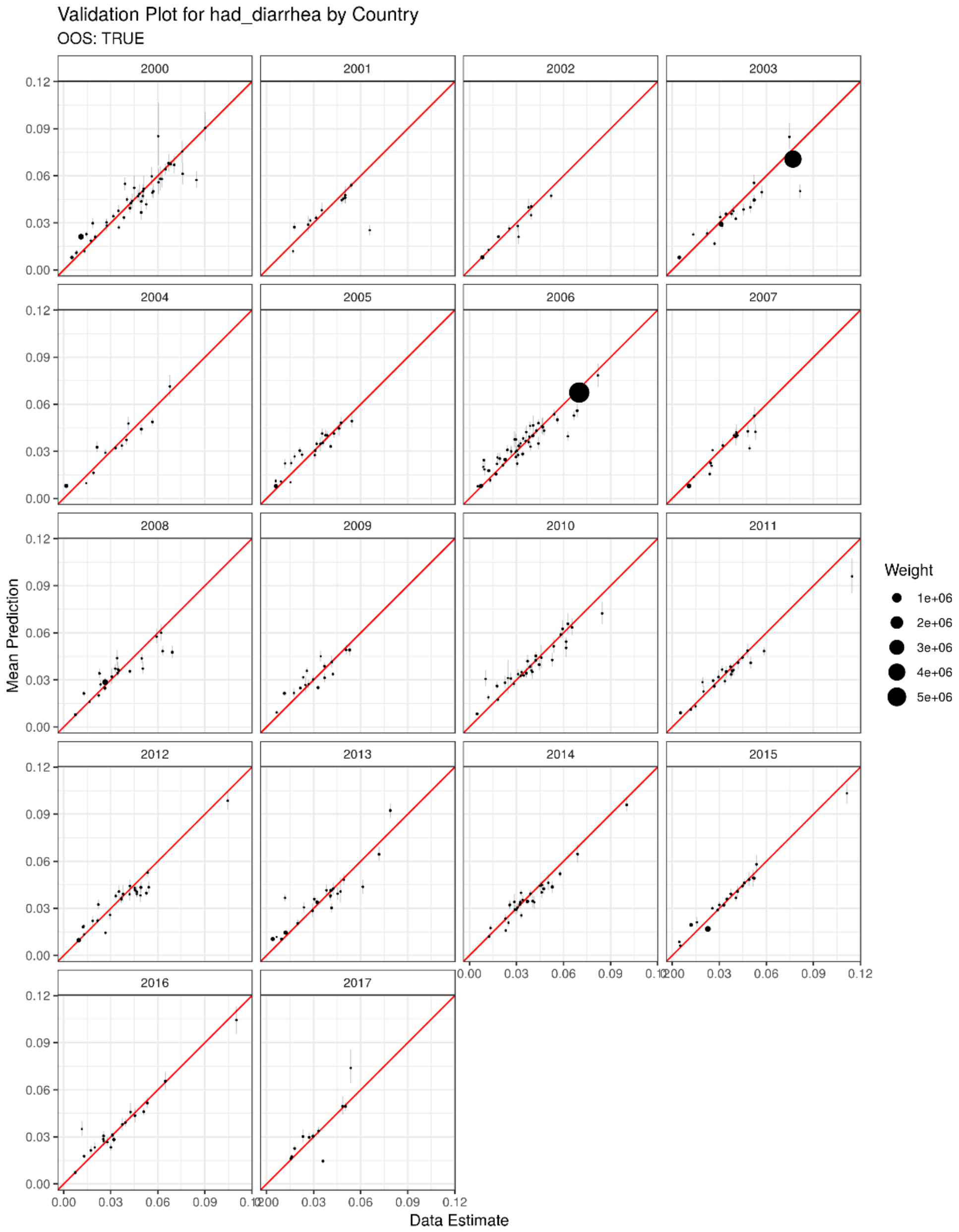

Appendix Figure 43. Out-of-sample validation plot of diarrhoea by first administrative unit and year

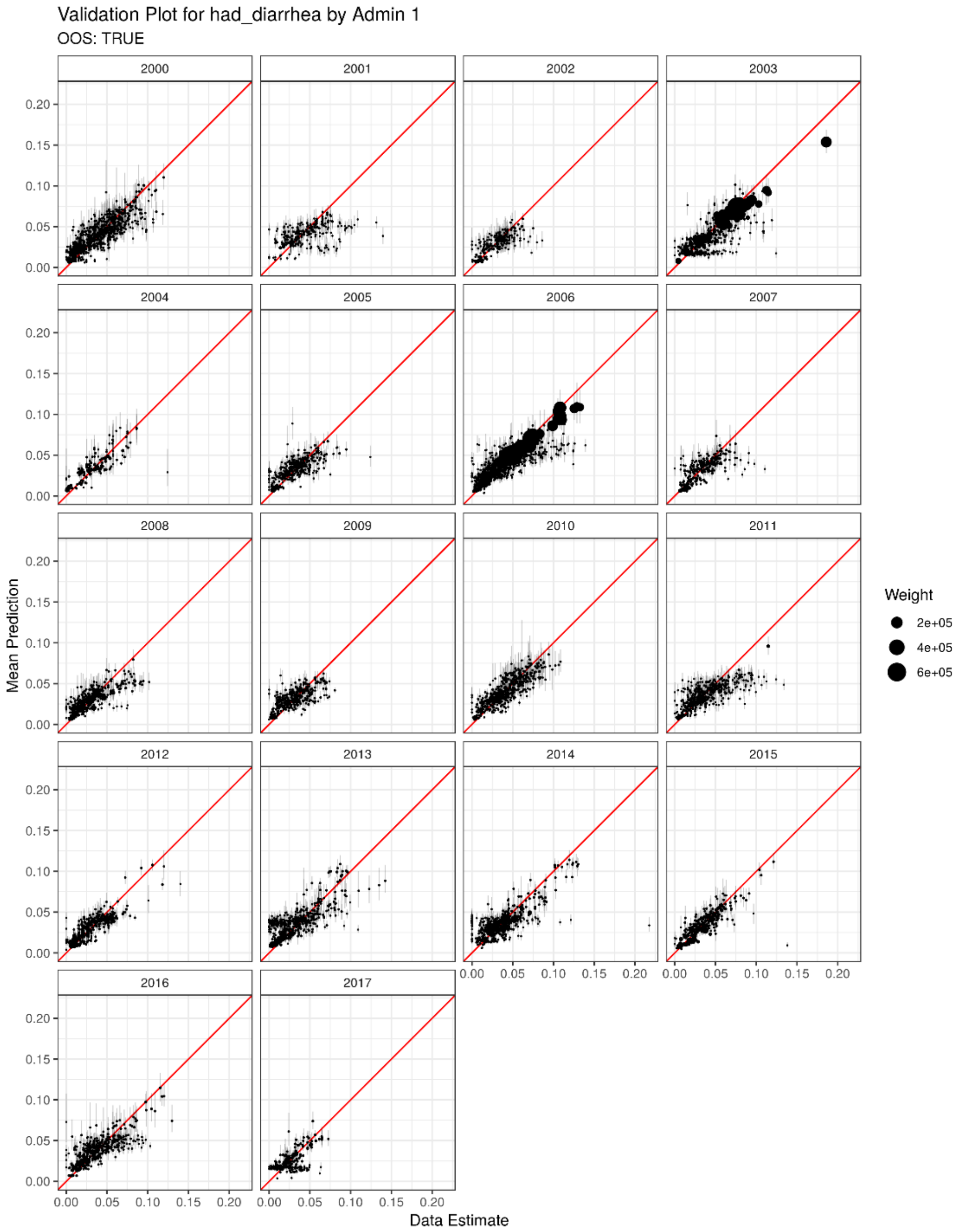

Appendix Figure 44. Out-of-sample validation plot of diarrhoea by second administrative division and year

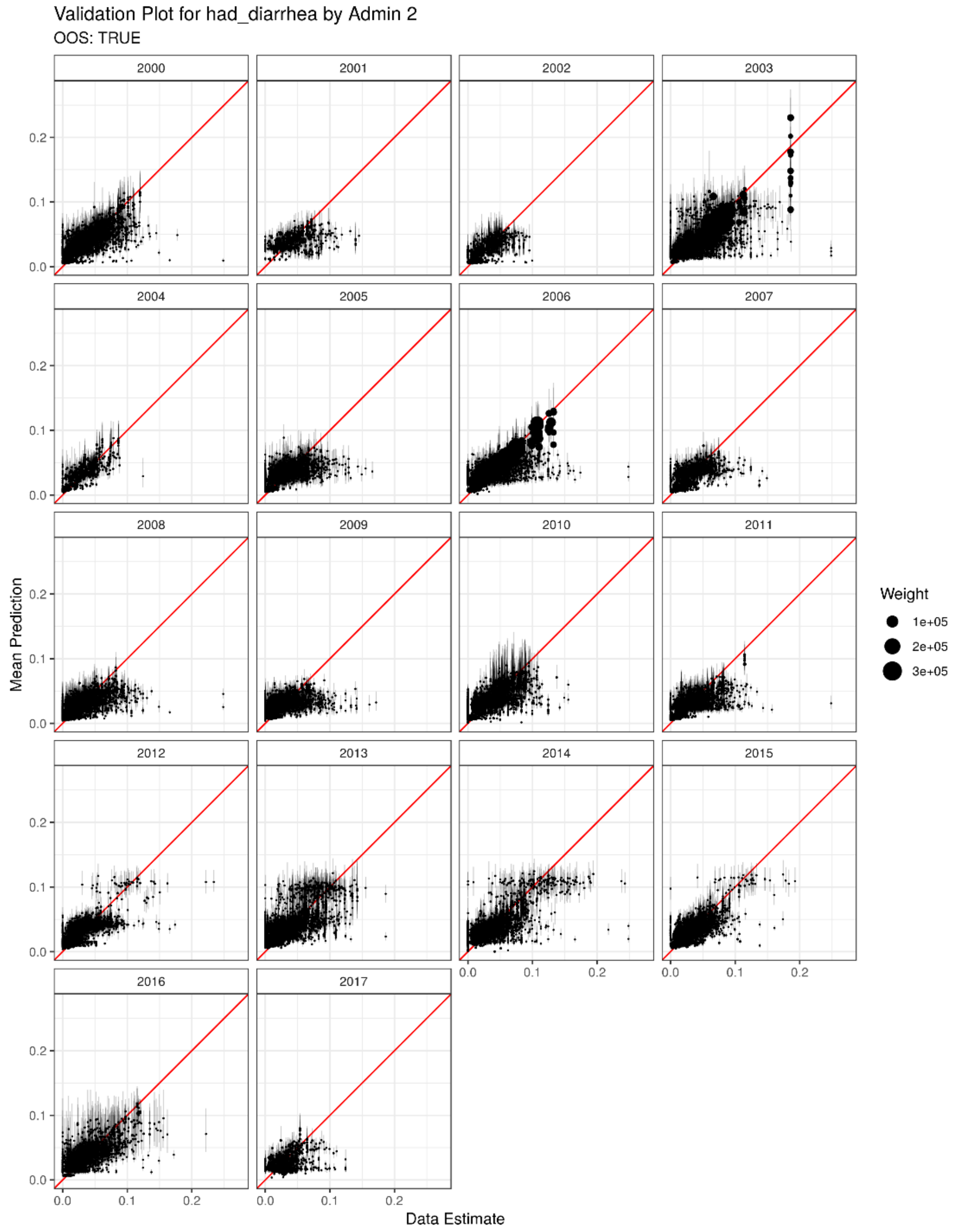

8.0 Appendix Tables

Appendix Table 1. Compliance for the Guidelines for Accurate and Transparent Health Estimates Reporting (GATHER) 84

Appendix Table 2. ISO3 codes and corresponding country names 86

Appendix Table 3. Countries included in analysis, stratified by Socio-demographic Index (SDI)[2] 88

Appendix Table 4. Covariates used in mapping 90

Appendix Table 5a–b. Covariates used in ensemble covariate modelling via stacked generalisation, stratified by modelling region 93

Appendix Table 6. Parameters used for boosted regression trees 96

Appendix Table 7. Fitted parameters 97

Appendix Table 8. Diarrhoea definition adjustment 99

Appendix Table 9. Age adjustment Table 101

Appendix Table 10a–f. In-sample fit statistics 103

Appendix Table 11a–f. Out-of-sample fit statistics 109

Appendix Table 1. Compliance for the Guidelines for Accurate and Transparent Health Estimates Reporting[1] (GATHER)

Item #	Checklist item	Reported
**Objectives and funding**		
**1**	Define the indicator(s), populations (including age, sex, and geographic entities), and time period(s) for which estimates were made.	Manuscript: Methods<br>Appendix: Section 1.0, 2.1
**2**	List the funding sources for the work.	Manuscript: Methods
**Data Inputs**		
For all data inputs from multiple sources that are synthesised as part of the study:		
**3**	Describe how the data were identified and how the data were accessed.	Manuscript: Methods<br>Appendix: Section 1.0, 2.0
**4**	Specify the inclusion and exclusion criteria. Identify all ad-hoc exclusions.	Manuscript: Methods<br>Appendix: Section 1.0, 2.1
**5**	Provide information on all included data sources and their main characteristics. For each data source used, report reference information or contact name/institution, population represented, data collection method, year(s) of data collection, sex and age range, diagnostic criteria or measurement method, and sample size, as relevant.	Manuscript: Methods<br>Appendix: Section 1.0 and 2.1, and available at: http://ghdx.healthdata.org/record/ihme-data/lmic-under-5-diarrhea-incidence-prevalence-and-mortality-geospatial-estimates-2000-2017
**6**	Identify and describe any categories of input data that have potentially important biases (e.g., based on characteristics listed in item 5).	Appendix: Section 2.0
For data inputs that contribute to the analysis but were not synthesised as part of the study:		
**7**	Describe and give sources for any other data inputs.	Manuscript: Methods<br>Appendix: Section 2.0, 3.0
**8**	Provide all data inputs in a file format from which data can be efficiently extracted (e.g., a spreadsheet rather than a PDF), including all relevant meta-data listed in item 5. For any data inputs that cannot be shared because of ethical or legal reasons, such as third-party ownership, provide a contact name or the name of the institution that retains the right to the data.	Available at: http://ghdx.healthdata.org/record/ihme-data/lmic-under-5-diarrhea-incidence-prevalence-and-mortality-geospatial-estimates-2000-2017
**9**	Provide a conceptual overview of the data analysis method. A diagram may be helpful.	Appendix: Section 3.0, Figure 1
**10**	Provide a detailed description of all steps of the analysis, including mathematical formulae. This description should cover, as relevant, data cleaning, data pre-processing, data adjustments and weighting of data sources, and mathematical or statistical model(s).	Manuscript: Methods
**11**	Describe how candidate models were evaluated and how the final model(s) were selected.	Manuscript: Methods Appendix: Sections 3.0, 4.0
**12**	Provide the results of an evaluation of model performance, if done, as well as the results of any relevant sensitivity analysis.	Manuscript: Methods<br>Appendix: Sections 5.0
**13**	Describe methods for calculating uncertainty of the estimates. State which sources of uncertainty were, and were not, accounted for in the uncertainty analysis.	Manuscript: Methods<br>Appendix: Sections 3.0
**14**	State how analytic or statistical source code used to generate estimates can be accessed.	Available at: http://ghdx.healthdata.org/record/ihme-data/lmic-under-5-diarrhea-incidence-prevalence-and-mortality-geospatial-estimates-2000-2017

Results and Discussion		
**15**	Provide published estimates in a file format from which data can be efficiently extracted.	Raster files for spatial data and CSVs of first- and second-administrative estimates available at http://ghdx.healthdata.org/record/ihme-data/lmic-under-5-diarrhea-incidence-prevalence-and-mortality-geospatial-estimates-2000-2017
**16**	Report a quantitative measure of the uncertainty of the estimates (e.g., credible intervals).	Manuscript: Results<br>Appendix: Section 3.0
**17**	Interpret results in light of existing evidence. If updating a previous set of estimates, describe the reasons for changes in estimates.	Manuscript: Discussion
**18**	Discuss limitations of the estimates. Include a discussion of any modelling assumptions or data limitations that affect interpretation of the estimates.	Manuscript: Discussion

Appendix Table 2. ISO3 codes and corresponding country names

ISO3 Code	Country Name
AFG	Afghanistan
AGO	Angola
BDI	Burundi
BEN	Benin
BFA	Burkina Faso
BGD	Bangladesh
BLZ	Belize
BOL	Bolivia
BRA	Brazil
BTN	Bhutan
BWA	Botswana
CAF	Central African Republic
CHN	China
CIV	Côte d'Ivoire
CMR	Cameroon
COD	Democratic Republic of the Congo
COG	Republic of the Congo
COL	Colombia
COM	Comoros
CPV	Cape Verde
CRI	Costa Rica
CUB	Cuba
DJI	Djibouti
DOM	Dominican Republic
DZA	Algeria
ECU	Ecuador
EGY	Egypt
ERI	Eritrea
ESH	Western Sahara
ETH	Ethiopia
GAB	Gabon
GHA	Ghana
GIN	Guinea
GMB	The Gambia
GNB	Guinea-Bissau
GNQ	Equatorial Guinea
GTM	Guatemala
GUF	French Guiana

ISO3 Code	Country Name
GUY	Guyana
HND	Honduras
HTI	Haiti
IDN	Indonesia
IND	India
IRN	Iran
IRQ	Iraq
JAM	Jamaica
JOR	Jordan
KEN	Kenya
KGZ	Kyrgyzstan
KHM	Cambodia
LAO	Laos
LBR	Liberia
LBY	Libya
LKA	Sri Lanka
LSO	Lesotho
MAR	Morocco
MDG	Madagascar
MEX	Mexico
MLI	Mali
MMR	Myanmar
MNG	Mongolia
MOZ	Mozambique
MRT	Mauritania
MWI	Malawi
MYS	Malaysia
NAM	Namibia
NER	Niger
NGA	Nigeria
NIC	Nicaragua
NPL	Nepal
PAK	Pakistan
PAN	Panama
PER	Peru
PHL	Philippines
PNG	Papua New Guinea
PRY	Paraguay

ISO3 Code	Country Name
PSE	Palestine
RWA	Rwanda
SDN	Sudan
SEN	Senegal
SLE	Sierra Leone
SLV	El Salvador
SOM	Somalia
SSD	South Sudan
STP	São Tomé and Príncipe
SUR	Suriname
SWZ	Swaziland (eSwatini)
SYR	Syria
TCD	Chad
TGO	Togo
THA	Thailand
TJK	Tajikistan
TKM	Turkmenistan
TLS	Timor-Leste
TTO	Trinidad and Tobago
TUN	Tunisia
TZA	Tanzania
UGA	Uganda
UZB	Uzbekistan
VEN	Venezuela
VNM	Vietnam
YEM	Yemen
ZAF	South Africa
ZMB	Zambia
ZWE	Zimbabwe

Appendix Table 3. Countries included in analysis, stratified by Socio-demographic Index (SDI)[3]

Low SDI	Low Middle SDI	Middle SDI
Afghanistan	Angola	Algeria
Bangladesh	Belize	Botswana
Benin	Bhutan	Brazil
Burkina Faso	Bolivia	Colombia
Burundi	Cambodia	Costa Rica
Central African Republic	Cameroon	Ecuador
Chad	Djibouti	Equatorial Guinea
Comoros	Dominican Republic	Gabon
Côte d'Ivoire	Egypt	Indonesia
Democratic Republic of the Congo	El Salvador	Jamaica
Eritrea	Ghana	Jordan
Ethiopia	Guatemala	Mexico
Guinea	Guyana	Mongolia
Guinea-Bissau	Honduras	Namibia
Haiti	India	Panama
Liberia	Iraq	Paraguay
Madagascar	Kenya	Peru
Malawi	Kyrgyzstan	Philippines
Mali	Laos	South Africa
Mozambique	Lesotho	Sri Lanka
Nepal	Mauritania	Suriname
Niger	Morocco	Syria
Papua New Guinea	Myanmar	Thailand
Rwanda	Nicaragua	Tunisia
Senegal	Nigeria	Turkmenistan
Sierra Leone	Pakistan	Uzbekistan
Somalia	Republic of the Congo	Vietnam
South Sudan	São Tomé and Príncipe	
Tanzania	Sudan	
The Gambia	Swaziland (eSwatini)	
Togo	Tajikistan	
Uganda	Timor-Leste	

Yemen	Zambia
	Zimbabwe

Appendix Table 4. Covariates used in mapping

A variety of socioeconomic and environmental variables were used to predict diarrhoea prevalence. Where available, the finest spatio-temporal resolution of gridded data sets was used.

Covariate	Temporal Resolution	Source	Reference
**Access to roads**		Oxford	Weiss, D. J. *et al.* A global map of travel time to cities to assess inequalities in accessibility in 2015. *Nature* **533**, 333–336 (2018).
**Ratio of children dependents (age 0 to 14) to working adults (age 1 to 64)**	Static	WorldPop	Available for Africa at: http://www.worldpop.org.uk/data/summary/?id=332
**Distance from rivers or lakes**	Static	Natural Earth Data (derived)	Natural Earth. Rivers and lake centerlines dataset. Available at: http://www.naturalearthdata.com/downloads/10mphysical-vectors/10m-rivers-lake-centerlines/. (Accessed: 24th July 2017)
**Nighttime lights**[TV]	Annual	NOAA DMSP satellite program (derived)	Savory et al. Intercalibration and Gaussian Process Modeling of Nighttime Lights Imagery for Measuring Urbanisation Trends in Africa 2000–2013. Remote Sens. 9, (2017).<br><br>Available at: https://www.ngdc.noaa.gov/eog/dmsp/downloadV4composites.html
**Elevation**	Static	NOAA GLOBE	Hastings, David A., and Paula K. Dunbar. Global Land One-kilometer Base Elevation (GLOBE) Digital Elevation Model, Documentation, Volume 1.0. Key to Geophysical Records Documentation (KGRD) 34. National Oceanic and Atmospheric Administration, National Geophysical Data Center, 325 Broadway, Boulder, Colorado 80303, U.S.A (1999).<br><br>GLOBE Task Team and others (Hastings, David A., Paula K. Dunbar, Gerald M. Elphingstone, Mark Bootz, Hiroshi Murakami, Hiroshi Maruyama, Hiroshi Masaharu, Peter Holland, John Payne, Nevin A. Bryant, Thomas L. Logan, J.-P. Muller, Gunter Schreier, and John S. MacDonald), eds., 1999. The Global Land One-kilometer Base Elevation (GLOBE) Digital Elevation Model, Version 1.0. National Oceanic and Atmospheric Administration, National Geophysical Data Center, 325 Broadway, Boulder, Colorado 80303, U.S.A. Available at: https://www.ngdc.noaa.gov/mgg/topo/globe.html. (Accessed: 16th February 2017)

Covariate	Temporal Resolution	Source	Reference
**Population ratio of women of maternal age to children (fertility)**	Annual	WorldPop (derived)	Lloyd, C. T., Sorichetta, A. & Tatem, A. J. High resolution global gridded data for use in population studies. *Sci. Data* **4**, sdata20171 (2017).<br>Available at: http://www.worldpop.org.uk/data/get_data/. (Accessed: 25th July 2017)
**Population**^{TV}	Annual	WorldPop	Lloyd, C. T., Sorichetta, A. & Tatem, A. J. High resolution global gridded data for use in population studies. Sci. Data 4, sdata20171 (2017).<br>World Pop. Get data. Available at: http://www.worldpop.org.uk/data/get_data/. (Accessed: 25th July 2017)
**Aridity**^{TV}	Annual	WorldClim (derived)	Zomer, R.J., Trabucco, A., Bossio, D.A. & Verchot, L.V. Climate change mitigation: A spatial analysis of global land suitability for clean development mechanism afforestation and reforestation. *Agriculture Ecosystems & Environment* **126**, 67–80 (2008).<br>Global Aridity Index (Global-Aridity) and Global Potential Evapo-Transpiration (Global-PET) Methodology and Geospatial Dataset Description (2009). Available at: http://www.cgiar-csi.org/data/global-aridity-and-pet-database
**Urban or rural**^{TV}	Annual	European Commission/ GHS	Pesaresi, M. et al. Operating procedure for the production of the Global Human Settlement Layer from Landsat data of the epochs 1975, 1990, 2000, and 2014. (Publications Office of the European Union, 2016).<br>Available at: http://ghsl.jrc.ec.europa.eu/data.php
**Urban proportion of the location**^{TV} **(landcover)**		MODIS	Available at: https://lpdaac.usgs.gov/dataset_discovery/modis/modis_products_table/mcd12q1
**Irrigation**	Static	University of Frankfurt and FAO	Siebert, S., Doll, P., Hoogeveen, J., Faures, J.-M., Frenken, K., & Feick, S. Development and validation of the global map of irrigation areas. *Hydrology and Earth System Sciences* **9**, 535–547 (2005).<br>Goethe-Universität. Generation of a digital global map of irrigation areas. Available at: https://www.unifrankfurt.de/45218039/Global_Irrigation_Map. (Accessed: 25th July 2017). Also from: http://www.fao.org/nr/water/aquastat/irrigationmap/index10.stm
**Number of people whose daily vitamin A**		Herrero et al. (modelled)	Herrero, M. et al. Farming and the geography of nutrient production for human use: a transdisciplinary analysis. *Lancet Planet. Health* **1**, e33–e42 (2017).

Covariate	Temporal Resolution	Source	Reference
**needs could be met (nutrient yield)**			
**Prevalence of under-5 stunting[TV]**		Internally modelled	Osgood-Zimmerman A, Millear AI, Stubbs RW, Shields C, Pickering BV, Earl L, Graetz N, Kinyoki DK, Ray SE, Bhatt S, Browne AJ, Burstein R, Cameron E, Casey DC, Deshpande A, Fullman N, Gething PW, Gibson HS, Henry NJ, Herrero M, Krause LK, Letourneau ID, Levine AJ, Liu PY, Longbottom J, Mayala BK, Mosser JF, Noor AM, Pigott DM, Piwoz EG, Rao P, Rawat R, Reiner RC, Smith DL, Weiss DJ, Wiens KE, Mokdad AH, Lim SS, Murray CJL, Kassebaum NJ, Hay SI. Mapping child growth failure in Africa between 2000 and 2015. Nature. 28 Feb 2018. doi:10.1038/nature25760<br>IHME CGF Team (Damaris K.), generated on 2018_12_05 using run_date 2018_10_30_13_12_18
**Prevalence of under-5 wasting[TV]**		Internally modelled	IHME CGF Team (Damaris K.), generated on 2018_12_05 using run_date 2018_10_30_13_12_18
**Diphtheria-tetanus-pertussis (DTP3) immunisation coverage[TV]**		Internally modelled	IHME Vaccine Team (Alyssa S.), generated on 2019_03_11 using run_date 2018_12_21_21_20_34
[TV] Time-varying			

Appendix Table 5a–b. Covariates used in ensemble covariate modelling via stacked generalisation, stratified by modelling region

Table **a)** presents the first 9 covariates and Table **b)** presents the following 9 covariates that were used in the generalised additive model (GAM), penalised regression with the elastic net penalty, and boosted regression tree (BRT) models.

a)

Region	Access to roads	Ratio of children dependents to working adults	Distance to rivers or lakes	Night-time lights^{TV}	Elevation	Fertility	Urbanicity^{TV}	Nutrient yield	Irrigation
**Horn of Africa**	TRUE	FALSE	TRUE	FALSE	TRUE	TRUE	TRUE	TRUE	TRUE
**Central sub-Saharan Africa**	TRUE	TRUE	TRUE	TRUE	FALSE	FALSE	TRUE	TRUE	TRUE
**Western sub-Saharan Africa**	TRUE	FALSE	TRUE	FALSE	TRUE	TRUE	TRUE	TRUE	TRUE
**North Africa and Middle East**	TRUE	FALSE	TRUE	FALSE	TRUE	TRUE	TRUE	TRUE	TRUE
**Southern sub-Saharan Africa**	TRUE	FALSE	TRUE	FALSE	TRUE	TRUE	TRUE	TRUE	TRUE
**Mexico, Central America, and the Caribbean**	TRUE	FALSE	TRUE	TRUE	TRUE	TRUE	TRUE	TRUE	TRUE
**South America**	TRUE	FALSE	TRUE	FALSE	TRUE	TRUE	TRUE	TRUE	TRUE
**Central Asia**	TRUE	TRUE	TRUE	FALSE	FALSE	FALSE	TRUE	TRUE	TRUE
**Mongolia**	TRUE	FALSE	TRUE	FALSE	TRUE	TRUE	TRUE	TRUE	TRUE
**India**	TRUE	FALSE	TRUE	TRUE	TRUE	TRUE	TRUE	TRUE	TRUE
**SE Asia**	TRUE	FALSE	TRUE	TRUE	TRUE	TRUE	TRUE	TRUE	TRUE
**Malay Archipelago**	TRUE	TRUE	TRUE	FALSE	TRUE	FALSE	TRUE	TRUE	TRUE

Region	Access to roads	Ratio of children dependents to working adults	Distance to rivers or lakes	Night-time lights^{TV}	Elevation	Fertility	Urbanicity^{TV}	Nutrient yield	Irrigation
**South Asia**	TRUE	TRUE	TRUE	TRUE	TRUE	FALSE	TRUE	TRUE	TRUE
**Middle East**	TRUE	FALSE	TRUE	FALSE	TRUE	TRUE	TRUE	TRUE	TRUE
**Eastern sub-Saharan Africa**	TRUE	TRUE	TRUE	TRUE	TRUE	FALSE	TRUE	TRUE	TRUE

*GBD covariate included in the VIF selection

^{TV} Time-varying

b)

Region	Land cover	Aridity^{TV}	Population^{TV}	Stunting^{TV}	Wasting^{TV}	DTP3 vaccine coverage	% of population with access to improved water sources	% of population with access to improved sanitation facilities	Haqi*
**Horn of Africa**	FALSE	FALSE	TRUE	TRUE	TRUE	TRUE	FALSE	TRUE	FALSE
**Central sub-Saharan Africa**	FALSE	TRUE	TRUE	TRUE	TRUE	TRUE	TRUE	FALSE	TRUE
**Western sub-Saharan Africa**	FALSE	TRUE	TRUE	TRUE	TRUE	TRUE	TRUE	FALSE	TRUE
**North Africa and Middle East**	FALSE	TRUE	TRUE	TRUE	TRUE	FALSE	FALSE	FALSE	TRUE
**Southern sub-Saharan Africa**	TRUE	TRUE	TRUE	FALSE	TRUE	TRUE	TRUE	TRUE	FALSE
**Mexico, Central**	FALSE	TRUE	TRUE	FALSE	TRUE	TRUE	FALSE	TRUE	FALSE

Region	Land cover	Aridity^{TV}	Population^{TV}	Stunting^{TV}	Wasting^{TV}	DTP3 vaccine coverage	% of population with access to improved water sources	% of population with access to improved sanitation facilities	Haqi*
**America, and the Caribbean**									
**South America**	TRUE	TRUE	TRUE	TRUE	TRUE	TRUE	FALSE	TRUE	FALSE
**Central Asia**	FALSE	TRUE	TRUE	FALSE	TRUE	TRUE	TRUE	FALSE	FALSE
**Mongolia**	TRUE	TRUE	FALSE	FALSE	TRUE	TRUE	TRUE	FALSE	FALSE
**India**	FALSE	TRUE	TRUE	TRUE	TRUE	TRUE	TRUE	TRUE	FALSE
**SE Asia**	FALSE	TRUE	TRUE	FALSE	TRUE	TRUE	TRUE	FALSE	FALSE
**Malay Archipelago**	FALSE	TRUE	TRUE	TRUE	TRUE	TRUE	TRUE	TRUE	FALSE
**South Asia**	FALSE	TRUE	TRUE	TRUE	TRUE	TRUE	TRUE	TRUE	FALSE
**Middle East**	TRUE	TRUE	TRUE	FALSE	TRUE	FALSE	TRUE	FALSE	FALSE
**Eastern sub-Saharan Africa**	FALSE	TRUE	TRUE	TRUE	TRUE	TRUE	TRUE	TRUE	TRUE

*GBD covariate included in the VIF selection

^{TV} Time-varying

Appendix Table 6. Parameters used for boosted regression trees

Region	Bagging fraction	Tree complexity	Number of trees	Learning rate	Min. observations per node
Central sub-Saharan Africa	0.5	4	997	0.061	13
Eastern sub-Saharan Africa	0.5	4	4448	0.041	8
Horn of Africa	0.5	4	933	0.088	12
North Africa Middle East	0.5	4	1680	0.058	6
Southern sub-Saharan Africa	0.5	4	758	0.067	9
Western sub-Saharan Africa	0.5	4	982	0.073	14
Mexico, Central America, and the Caribbean	0.5	4	1374	0.035	12
South America	0.5	4	3996	0.090	14
Central Asia	0.5	4	1397	0.037	9
Middle East	0.5	4	778	0.069	11
Mongolia	0.5	4	1479	0.020	12
South Asia	0.5	4	1323	0.044	15
Southeast Asia	0.5	4	1883	0.040	13
Malay Archipelago	0.5	4	5780	0.032	14
India	0.5	4	9662	0.011	15

Appendix Table 7. Fitted parameters

Lower, median, and upper quantiles (0.025%, 0.50%, 0.975%) are displayed for the main parameters by region. The first four columns provide information on the fixed effects: the intercept (int) and the covariates (gam, gbm, and enet) corresponding to the predicted ensemble rasters. Fitted values for the spatio-temporal field hyperparameters and the precisions (inverse variance) for our random effects are shown in the next four columns.

	Quantiles	int	gam	gbm	enet	Nominal Range	Nominal Variance	Ar1 ρ	precis	Country Random Effect Precision
**Central sub-Saharan Africa quantiles**	0.025	-0.0886	0.0705	0.3891	0.2191	0.2955	-0.3819	0.7169	842.9741	23.4788
	0.500	-0.0314	0.1877	0.4714	0.3408	0.5494	0.1160	0.8939	13143.2288	48.1609
	0.975	0.0256	0.3050	0.5538	0.4621	0.9071	0.4610	0.9648	68813.7643	95.3734
**Eastern sub-Saharan Africa quantiles**	0.025	-0.2308	0.1487	0.1842	0.3620	0.4067	-0.6690	0.9185	1147.5586	7.8353
	0.500	-0.1379	0.2719	0.2352	0.4927	0.6502	-0.3145	0.9614	12679.4904	12.6842
	0.975	-0.0452	0.3957	0.2863	0.6228	0.8788	0.0543	0.9842	75651.0104	20.2242
**Horn of Africa quantiles**	0.025	-0.5808	-0.0500	0.2815	0.5792	0.6547	-1.0195	0.8548	930.3892	10.9600
	0.500	-0.4380	0.0062	0.3390	0.6547	0.7915	-0.7082	0.9225	1810.3702	23.1016
	0.975	-0.2957	0.0626	0.3965	0.7301	0.9143	-0.3794	0.9589	4466.2463	50.6154
**North Africa Middle East quantiles**	0.0749	0.1664	-0.0694	0.3312	0.2413	-0.9193	0.3822	-0.5853	826.0058	6.8683
	0.2093	0.2676	0.1117	0.4422	0.4459	-0.5830	0.7707	-0.0672	11303.5765	14.8836
	0.3432	0.3678	0.2927	0.5532	0.6505	-0.2230	1.1364	0.5383	65246.7832	32.0212
**Southern sub-Saharan Africa quantiles**	0.025	-0.1320	-0.0813	-0.0009	0.3257	0.9881	-0.3959	-0.0482	1463.2432	2.4434
	0.500	-0.0483	0.2702	0.0556	0.6736	2.3495	2.1246	0.7705	14945.0866	4.3667
	0.975	0.0337	0.6237	0.1131	1.0190	4.7445	6.7720	0.9792	71203.3650	7.5538
**Western sub-Saharan Africa quantiles**	0.025	-0.0948	-0.1478	0.5400	0.2894	0.6811	-1.0268	0.3450	3762.6308	11.4176
	0.500	-0.0211	-0.0282	0.6272	0.4010	0.8442	-0.7962	0.5638	21401.5247	21.7635
	0.975	0.0526	0.0916	0.7142	0.5122	0.9979	-0.5499	0.7552	83697.6148	33.2766
**Mexico, Central America, and the Caribbean quantiles**	0.025	-0.1950	-0.0506	0.2434	0.2600	0.7259	-1.9563	0.5816	2136.2162	982.5331
	0.500	-0.0592	0.1821	0.3349	0.4830	1.6673	-1.2873	0.8604	15704.5585	12036.3112
	0.975	0.0764	0.4160	0.4264	0.7042	2.3694	-0.5411	0.9569	73301.5720	65428.6688

	Quantiles	int	gam	gbm	enet	Nominal Range	Nominal Variance	Ar1 ρ	precis	Country Random Effect Precision
**South America quantiles**	0.025	-0.1956	0.3415	0.0431	0.2239	1.5704	-2.2363	0.8047	3080.3541	21.9895
	0.500	-0.0839	0.5261	0.0667	0.4078	2.1855	-1.5850	0.9244	17126.0933	47.4096
	0.975	0.0276	0.7124	0.0888	0.5899	2.7379	-0.9261	0.9812	79371.8188	116.6675
**Central Asia quantiles**	0.025	-0.7328	-0.1352	0.1902	0.4642	-0.5402	-0.2720	-0.1473	107.0631	1088.7073
	0.500	-0.5121	0.0046	0.3440	0.6509	-0.1399	0.2469	0.4438	276.6643	11491.0210
	0.975	-0.2939	0.1451	0.4981	0.8371	0.2603	0.7487	0.7726	743.1322	63244.8907
**Middle East quantiles**	0.025	-0.9871	-0.0077	0.0072	0.7681	-0.8873	0.4064	0.4146	140.2626	1.8549
	0.500	-0.8549	0.0543	0.0796	0.8638	-0.7394	0.5726	0.6412	265.3059	3.4692
	0.975	-0.7222	0.1243	0.1519	0.9565	-0.6093	0.7637	0.7770	580.7055	6.5046
**Mongolia quantiles**	0.025	-0.0045	-0.0334	0.0782	-0.1312	-1.2813	-1.4449	0.1459	1484.8496	--
	0.500	0.1164	0.4541	0.1877	0.3558	1.4519	1.4551	0.7529	14038.4000	--
	0.975	0.2347	0.9575	0.3009	0.8278	3.4203	6.1773	0.9491	69651.9570	--
**South Asia quantiles**	0.025	-0.2193	-0.1116	0.3695	0.4725	-0.5459	0.0654	-0.1160	235.8261	4.5442
	0.500	-0.1335	-0.0352	0.4584	0.5765	-0.3548	0.2452	0.2147	552.3542	7.5084
	0.975	-0.0480	0.0418	0.5473	0.6802	-0.2082	0.4712	0.5651	1796.0736	13.8463
**Southeast Asia quantiles**	0.025	-0.3366	-0.1004	0.0065	0.7936	0.2318	-1.1720	0.6487	2145.9710	1.9920
	0.500	-0.1711	0.0464	0.0119	0.9417	0.7130	-0.5234	0.8835	16480.1070	3.5187
	0.975	-0.0079	0.1946	0.0172	1.0882	1.2176	0.1041	0.9649	78564.8125	6.0458
**Malay Archipelago quantiles**	0.025	0.0221	0.4149	-0.0037	0.2941	0.2425	-0.7570	0.9201	2586.1534	1.3418
	0.500	0.0880	0.5594	0.0022	0.4385	0.6626	0.0249	0.9571	16187.5878	2.2262
	0.975	0.1536	0.7038	0.0081	0.5826	1.2129	0.6209	0.9822	110542.2131	3.7787
**India quantiles**	0.025	-0.0031	-0.1565	0.3147	0.4699	-0.0358	-0.3318	-0.2628	3402.7821	--
	0.500	0.0681	-0.0057	0.3856	0.6200	0.1397	-0.0848	-0.0445	19465.9450	--
	0.975	0.1389	0.1453	0.4565	0.7696	0.3553	0.1137	0.2024	84647.6970	--

Appendix Table 8. Diarrhoea definition adjustment

Below are all surveys that were adjusted for their definition of diarrhoea shown with source sample size, age adjusted prevalence and diarrhoea adjusted prevalence.

Country	Source	Year	Sample Size	Age Adjusted Prevalence	Definition Adjusted Prevalence
**Burundi**	UNICEF MICS	2005	6550.13	0.197082	0.189892
**Burkina Faso**	UNICEF MICS	2006	5216	0.19862	0.191043
**Bangladesh**	UNICEF MICS	2006	29675	0.071205	0.068102
**Belize**	UNICEF MICS	2006	780.9562	0.12358	0.118141
**Belize**	UNICEF MICS	2011	1902.836	0.075849	0.072339
**Bolivia**	HOUSEHOLD SURVEY	2016	609.2184	0.220988	0.212279
**Côte d'Ivoire**	UNICEF MICS	2006	6886.423	0.169944	0.162891
**Cameroon**	UNICEF MICS	2006	5853.078	0.183348	0.176476
**Democratic Republic of the Congo**	UNICEF MICS	2001	9385.646	0.22511	0.21628
**Comoros**	UNICEF MICS	2000	4321.497	0.188021	0.180248
**Djibouti**	UNICEF MICS	2006	1949.202	0.041447	0.03946
**Dominican Republic**	UNICEF MICS	2000	1995	0.210526	0.202192
**Ghana**	UNICEF MICS	2006	3048.836	0.162407	0.155575
**The Gambia**	UNICEF MICS	2000	3616	0.221239	0.21258
**The Gambia**	UNICEF MICS	2005	6485.514	0.192505	0.184804
**Guinea-Bissau**	UNICEF MICS	2000	5597.045	0.319706	0.308915
**Guinea-Bissau**	UNICEF MICS	2006	5291.861	0.122233	0.116802
**Equatorial Guinea**	UNICEF MICS	2000	2469.057	0.235834	0.226661
**Guyana**	UNICEF MICS	2006	2295.757	0.111238	0.106345
**India**	DISTRICT LEVEL HOUSEHOLD SURVEY	2007	255902.6	0.110598	0.105792
**Iraq**	UNICEF MICS	2000	14338.29	0.206359	0.198166
**Iraq**	UNICEF MICS	2006	14286.11	0.13196	0.126234
**Iraq**	UNICEF MICS	2011	24866.95	0.144014	0.137882
**Jamaica**	UNICEF MICS	2005	1419	0.024665	0.02346
**Kenya**	UNICEF MICS	2008	13996	0.110389	0.105705
**Kenya**	UNICEF MICS	2009	450.2132	0.193378	0.185408
**Kyrgyzstan**	UNICEF MICS	2005	2854.543	0.038772	0.037039
**Laos**	UNICEF MICS	2000	5101	0.060576	0.057992
**Laos**	UNICEF MICS	2006	4134	0.129657	0.12453
**Madagascar**	UNICEF MICS	2000	5746.31	0.132394	0.126581
**Myanmar**	MULTIPLE INDICATOR CLUSTER SURVEY	2003	1852.875	0.339108	0.328085
**Myanmar**	UNICEF MICS	2009	15275.07	0.07432	0.070863
**Mongolia**	UNICEF MICS	2005	3542.681	0.06609	0.062991

**Mozambique**	UNICEF MICS	2008	11407	0.175068	0.168237
**Mauritania**	UNICEF MICS	2007	8561.69	0.218515	0.209927
**Malawi**	UNICEF MICS	2006	20137.5	0.233921	0.224899
**Niger**	UNICEF MICS	2000	4613.821	0.373406	0.361804
**Nigeria**	WB_CWIQ	2006	25194.4	0.05184	0.049361
**Nigeria**	UNICEF MICS	2007	16487	0.105841	0.101178
**Sudan**	UNICEF MICS	2000	20789.45	0.278391	0.268252
**Sudan**	UNICEF MICS	2010	11952.26	0.272342	0.26228
**Senegal**	UNICEF MICS	2000	8453.075	0.293031	0.282557
**Sierra Leone**	UNICEF MICS	2000	2669.364	0.251824	0.242272
**Sierra Leone**	UNICEF MICS	2005	5232.73	0.14395	0.137842
**Somalia**	UNICEF MICS	2006	6195.955	0.195114	0.187579
**South Sudan**	UNICEF MICS	2000	1390	0.25036	0.241886
**South Sudan**	UNICEF MICS	2010	8197.817	0.351049	0.339436
**São Tomé and Príncipe**	UNICEF MICS	2000	2189.46	0.182501	0.174893
**Suriname**	UNICEF MICS	2006	2238.076	0.111963	0.107071
**Syria**	UNICEF MICS	2006	10933	0.081498	0.077759
**Chad**	UNICEF MICS	2000	4114.743	0.313937	0.303138
**Togo**	UNICEF MICS	2006	3499.781	0.151746	0.145265
**Thailand**	UNICEF MICS	2012	4018.802	0.053646	0.051075
**Thailand**	UNICEF MICS	2015	2883.827	0.05251	0.049992
**Thailand**	UNICEF MICS	2016	472.607	0.036036	0.034275
**Tajikistan**	UNICEF MICS	2005	3897.297	0.128908	0.123202
**Turkmenistan**	UNICEF MICS	2006	2054.736	0.056043	0.053373
**Uganda**	WB_LSMS_ISA	2013	2181.79	0.136815	0.131967
**Uzbekistan**	UNICEF MICS	2006	4569.577	0.022602	0.021491
**Vietnam**	UNICEF MICS	2000	3082.22	0.114597	0.109499
**Vietnam**	UNICEF MICS	2006	2678	0.073936	0.070473

Appendix Table 9. Age adjustment Table

Below are all surveys that were adjusted for age difference (e.g., the source sampled children age 0–24 months instead of 0–59 months) shown with source sample size, age range of the children sampled, unadjusted prevalence, and adjusted prevalence.

Country	Source	Year	Sample Size	Age Range	Unadjusted Prevalence	Age Adjusted Prevalence
**Burkina Faso**	WB CWIQ	2003	729	0–4	0.325	0.312
**Ethiopia**	WB LSMS ISA	2013	2906	0.5–5	0.151	0.154
**India**	DISTRICT LEVEL HOUSEHOLD SURVEY	2002	162193	0–3	0.136	0.125
**India**	DISTRICT LEVEL HOUSEHOLD SURVEY	2007	255903	0–4	0.117	0.111
**India**	COVERAGE EVALUATION SURVEY	2009	15425	1-2	0.154	0.131
**Kenya**	SMART SURVEY	2011	472	0–3	0.124	0.131
**Kenya**	SMART SURVEY	2011	678	0.5–5	0.124	0.131
**Kenya**	SMART SURVEY	2011	313	0.5–5	0.128	0.136
**Kenya**	SMART SURVEY	2011	221	0.5–5	0.303	0.321
**Kenya**	SMART SURVEY	2011	725	0.5–5	0.060	0.064
**Kenya**	SMART SURVEY	2012	614	0.5–5	0.096	0.102
**Kenya**	SMART SURVEY	2012	441	0.5–5	0.320	0.339
**Kenya**	SMART SURVEY	2012	373	0.5–5	0.129	0.137
**Kenya**	SMART SURVEY	2012	238	0.5–5	0.210	0.223
**Mozambique**	MACRO AIS	2015	4980	1-3	0.111	0.103
**Nigeria**	HOUSEHOLD, SCHOOL AND HEALTH FACILITY SURVEY	2005	614	0-3	0.055	0.047
**Nigeria**	HOUSEHOLD, SCHOOL AND HEALTH FACILITY SURVEY	2007	909	0-3	0.043	0.037
**Sierra Leone**	NUTRITION SURVEY	2017	8462	0.5-5	0.047	0.047
**Vietnam**	MACRO DHS	2002	1230	0-3	0.109	0.102
**Yemen**	NUTRITIONAL STATUS & MORTALITY SURVEY	2011	4721	0.5-5	0.454	0.461
**Yemen**	NUTRITIONAL STATUS & MORTALITY SURVEY	2012	711	0.5-5	0.370	0.375
**Yemen**	NUTRITIONAL STATUS & MORTALITY SURVEY	2012	411	0.5-5	0.287	0.291
**Yemen**	NUTRITIONAL STATUS & MORTALITY SURVEY	2012	1439	0.5-5	0.469	0.475
**Yemen**	NUTRITIONAL STATUS & MORTALITY SURVEY	2012	1648	0.5-5	0.419	0.425
**Yemen**	NUTRITIONAL STATUS & MORTALITY SURVEY	2013	949	0.5-5	0.348	0.352
**Yemen**	NUTRITIONAL STATUS & MORTALITY SURVEY	2013	861	0.5-5	0.354	0.358
**Yemen**	NUTRITIONAL STATUS & MORTALITY SURVEY	2014	325	0.5-5	0.357	0.360

**Yemen**	NUTRITIONAL STATUS & MORTALITY SURVEY	2014	1547	0.5-5	0.456	0.461
**Yemen**	NUTRITIONAL STATUS & MORTALITY SURVEY	2015	695	0.5-5	0.414	0.418
**Yemen**	NUTRITIONAL STATUS & MORTALITY SURVEY	2015	1115	0.5-5	0.483	0.487
**Yemen**	NUTRITIONAL STATUS & MORTALITY SURVEY	2015	1034	0.5-5	0.423	0.426
**Yemen**	NUTRITIONAL STATUS & MORTALITY SURVEY	2016	738	0.5-5	0.480	0.483
**Yemen**	NUTRITION_SURVEY	2016	1746	0.5-5	0.390	0.392
**Yemen**	NUTRITION_SURVEY	2016	1190	0.5-5	0.470	0.473
**Yemen**	NUTRITION_SURVEY	2016	1234	0.5-5	0.461	0.464
**Yemen**	NUTRITIONAL STATUS & MORTALITY SURVEY	2017	1315	0.5-5	0.215	0.216

Appendix Table 10a–f. In-sample fit statistics

In-sample fit statistics are shown for country-level **(a,b)**, first-administrative level **(c,d)**, and second administrative level **(e,f)** aggregations. Metrics are shown by year **(a,d,e)** and by modelling region **(b,d,e)**.

a) Predictive in sample metrics by year aggregated to the country level for diarrhoea prevalence.

Year	Mean Err.	RMSE	Median SS	Corr.	95% Cov.
**2000**	-0.00258	36461.76	4422.872	0.959677	0.600903
**2001**	0.000403	6842.664	5122.5	0.81014	0.948672
**2002**	0.000896	26077.07	5486.588	0.976267	0.944371
**2003**	0.029838	1144636	6500	0.88682	0.05286
**2004**	-0.00205	25359.22	6586.5	0.982204	0.896659
**2005**	-0.0012	19500.86	5245.365	0.972327	0.969033
**2006**	0.001824	902712.1	5225.304	0.990794	0.993457
**2007**	0.002828	33534.66	5724	0.96358	0.911643
**2008**	-0.00183	55413.21	6066.49	0.779837	0.882409
**2009**	-0.00061	10314.59	6029.213	0.941342	0.960504
**2010**	0.00145	9855.338	7655.195	0.89581	0.942704
**2011**	0.001853	10730.01	8439.388	0.968006	0.976789
**2012**	0.001372	22222.66	6243.5	0.950423	0.918817
**2013**	-0.0017	28721.44	6838.862	0.909784	0.931013
**2014**	0.002724	13282.26	6917	0.965095	0.956792
**2015**	0.00138	56992.99	10085.63	0.952785	0.96839
**2016**	0.003331	12252	7010.024	0.918266	0.918563
**2017**	0.001883	12617.23	11126.29	0.814551	0.818453

b) Predictive in sample metrics by region aggregated to the country level for diarrhoea prevalence.

Region	Mean Err.	RMSE	Median SS	Corr.	95% Cov.
**India**	-0.00141	142571.4	68721.18	0.794424	0.940274
**Mongolia**	-0.00066	4099.28	3581.603	0.597622	0.933523
**Horn of Africa**	0.00192	1215904	6616.013	0.957704	0.995036
**Central Asia**	0.001292	4116.442	4167	0.987361	0.993177
**Central sub-Saharan Africa**	-0.0018	10291.73	7328.384	0.915071	0.978634
**Eastern sub-Saharan Africa**	0.001535	9395.286	5803.655	0.879773	0.978874
**Malay Archipelago**	-0.00087	53755.23	17696.02	0.822281	0.883395
**Mexico, central America, and the Caribbean**	-0.00051	7013.744	4892.403	0.989369	0.9897
**The Middle East**	0.03113	1287518	12399.27	0.979286	0.030938
**Northern Africa**	-0.00402	65160.69	5850	0.973299	0.410153
**South America**	0.001039	10155.17	4599.155	0.854705	0.986645
**Southeast Asia**	0.002658	7958.273	5038.556	0.713112	0.804503
**South Asia**	0.001022	18234.85	8239	0.94253	0.863633
**Southern sub-Saharan Africa**	0.000477	4815.835	1545.903	0.832675	0.682705
**Western sub-Saharan Africa**	0.000579	11761.66	6983.188	0.916647	0.940076

c) Predictive in sample metrics by year aggregated to the first administrative level for diarrhoea prevalence.

Year	Mean Err.	RMSE	Median SS	Corr.	95% Cov.
**2000**	-0.00258	2333.669	274	0.932465	0.600903
**2001**	0.000403	584.7609	68.17568	0.783579	0.948672
**2002**	0.000896	1342.125	161.7645	0.926455	0.944371
**2003**	0.029838	54702.24	153	0.972523	0.05286
**2004**	-0.00205	1686.012	368.2709	0.964964	0.896659
**2005**	-0.0012	1156.03	272.4639	0.917135	0.969033
**2006**	0.001824	50498.47	250.6849	0.968795	0.993457
**2007**	0.002828	1643.3	312.4817	0.907021	0.911643
**2008**	-0.00183	3548.19	377.02	0.783915	0.882409
**2009**	-0.00061	703.4821	291.0782	0.874766	0.960504
**2010**	0.00145	906.9722	371	0.866054	0.942704
**2011**	0.001853	670.9522	331.4673	0.907993	0.976789
**2012**	0.001372	1248.834	355.9981	0.913408	0.918817
**2013**	-0.0017	1534.853	305.3576	0.882256	0.931013
**2014**	0.002724	1326.408	349.1717	0.90844	0.956792
**2015**	0.00138	4019.886	644.6587	0.924898	0.96839
**2016**	0.003331	589.3396	282.2504	0.874914	0.918563
**2017**	0.001883	921.1147	444.6032	0.748113	0.818453

d) Predictive in sample metrics by region aggregated to the first administrative level for diarrhoea prevalence.

Region	Mean Err.	RMSE	Median SS	Corr.	95% Cov.
**India**	-0.00141	6658.789	1133.135	0.857302	0.940274
**Mongolia**	-0.00066	371.2068	162.5	0.618169	0.933523
**Horn of Africa**	0.00192	85527.09	642	0.967772	0.995036
**Central Asia**	0.001292	579.7716	386.2754	0.931556	0.993177
**Central sub-Saharan Africa**	-0.0018	719.0324	529.4174	0.824306	0.978634
**Eastern sub-Saharan Africa**	0.001535	773.8414	97	0.78371	0.978874
**Malay Archipelago**	-0.00087	2382.332	270.124	0.760727	0.883395
**Mexico, central America, and the Caribbean**	-0.00051	464.1463	271.4587	0.877892	0.9897
**The Middle East**	0.03113	64224.77	689.3445	0.991596	0.030938
**Northern Africa**	-0.00402	3137.011	247	0.836688	0.410153
**South America**	0.001039	555.3194	329.3172	0.790738	0.986645
**Southeast Asia**	0.002658	386.785	57.736	0.662882	0.804503
**South Asia**	0.001022	4256.645	928	0.911795	0.863633
**Southern sub-Saharan Africa**	0.000477	515.1575	140.5291	0.762385	0.682705
**Western sub-Saharan Africa**	0.000579	922.161	536.7694	0.885348	0.940076

e) Predictive in sample metrics by year aggregated to the second administrative level for diarrhoea prevalence.

Year	Mean Err.	RMSE	Median SS	Corr.	95% Cov.
**2000**	-0.00258	703.0055	38	0.909764	0.600903
**2001**	0.000403	156.9732	29.24542	0.72693	0.948672
**2002**	0.000896	158.4306	50.04706	0.851144	0.944371
**2003**	0.029838	9759.431	22	0.964552	0.05286
**2004**	-0.00205	216.2431	103.096	0.945658	0.896659
**2005**	-0.0012	127.6838	29	0.835027	0.969033
**2006**	0.001824	13224.14	44	0.965545	0.993457
**2007**	0.002828	179.2696	51.9902	0.843329	0.911643
**2008**	-0.00183	266.5709	45	0.744177	0.882409
**2009**	-0.00061	137.024	26.84685	0.7587	0.960504
**2010**	0.00145	178.7098	33.19359	0.800524	0.942704
**2011**	0.001853	141.7656	39	0.850136	0.976789
**2012**	0.001372	165.2871	42.88677	0.851378	0.918817
**2013**	-0.0017	143.9977	20.09701	0.819787	0.931013
**2014**	0.002724	251.7417	43	0.829846	0.956792
**2015**	0.00138	344.6095	90.60172	0.834851	0.96839
**2016**	0.003331	134.3992	43	0.81033	0.918563
**2017**	0.001883	184.4761	25.39926	0.652647	0.818453

f) Predictive in sample metrics by region aggregated to the second administrative level for diarrhoea prevalence.

Region	Mean Err.	RMSE	Median SS	Corr.	95% Cov.
**India**	-0.00141	273.2747	103	0.752488	0.940274
**Mongolia**	-0.00066	89.50022	7.662	0.434734	0.933523
**Horn of Africa**	0.00192	21244.38	68	0.961905	0.995036
**Central Asia**	0.001292	162.6019	49.76313	0.820718	0.993177
**Central sub-Saharan Africa**	-0.0018	197.5417	65	0.681959	0.978634
**Eastern sub-Saharan Africa**	0.001535	172.9063	42	0.681539	0.978874
**Malay Archipelago**	-0.00087	215.071	61	0.655253	0.883395
**Mexico, central America, and the Caribbean**	-0.00051	134.9077	17.77103	0.716695	0.9897
**The Middle East**	0.03113	14396.95	87	0.985651	0.030938
**Northern Africa**	-0.00402	831.1926	26	0.76702	0.410153
**South America**	0.001039	105.8462	12.12976	0.674362	0.986645
**Southeast Asia**	0.002658	132.0324	16.75897	0.601928	0.804503
**South Asia**	0.001022	836.2025	254.6374	0.901628	0.863633
**Southern sub-Saharan Africa**	0.000477	105.226	26.88727	0.680792	0.682705
**Western sub-Saharan Africa**	0.000579	192.1447	49.31013	0.810058	0.940076

Appendix Table 11a–f. Out-of-sample fit statistics

Out-of-sample fit statistics are shown for country-level **(a,b)**, first-administrative level **(c,d)**, and second administrative level **(e,f)** aggregations. Metrics are shown by year **(a,d,e)** and by modelling region **(b,d,e)**.

a) Predictive out of sample metrics by year aggregated to the country level for diarrhoea prevalence.

Year	Mean Err.	RMSE	Median SS	Corr.	95% Cov.
**2000**	-0.00425	36458.76	4218.12	0.964018	0.620194
**2001**	3.41E-05	6842.664	5122.5	0.834617	0.827049
**2002**	9.44E-05	26077.07	5486.588	0.989783	0.935431
**2003**	0.005974	954648	6500	0.995106	0.881182
**2004**	-0.00333	25359.22	6586.5	0.97326	0.890665
**2005**	-0.00103	19500.86	5245.365	0.981503	0.947297
**2006**	0.002344	851002.3	5225.304	0.992665	0.911571
**2007**	0.002501	33534.66	5724	0.988127	0.903439
**2008**	-0.00034	55415.28	6066.49	0.906486	0.849729
**2009**	-0.00048	10310.2	6029.213	0.923582	0.906068
**2010**	0.000143	9855.339	7655.195	0.968654	0.946022
**2011**	0.001062	10730.5	8439.388	0.983355	0.938904
**2012**	0.000884	22222.66	6243.5	0.968307	0.887391
**2013**	-0.00192	28659.89	6838.862	0.96361	0.94324
**2014**	0.002699	13282.26	6917	0.973032	0.929452
**2015**	0.002195	56992.99	10085.63	0.946642	0.948599
**2016**	0.000313	12252	7010.024	0.986801	0.945851
**2017**	0.00084	12617.23	11126.29	0.781495	0.785643

b) Predictive out of sample metrics by region aggregated to the country level for diarrhoea prevalence.

Region	Mean Err.	RMSE	Median SS	Corr.	95% Cov.
**India**	0.001171	142573.6	68721.18	0.811362	0.897341
**Mongolia**	-0.00064	4099.28	3581.603	0.881029	0.929388
**Horn of Africa**	0.002478	1146232	6616.013	0.959602	0.915092
**Central Asia**	-0.00017	4008.679	3897.297	0.991849	0.967378
**Central sub-Saharan Africa**	-0.0005	10291.73	7328.384	0.980656	0.935896
**Eastern sub-Saharan Africa**	0.001355	9395.286	5803.655	0.901306	0.941136
**Malay Archipelago**	-0.00087	53755.23	17696.02	0.817942	0.881355
**Mexico, central America, and the Caribbean**	0.00014	7013.744	4892.403	0.985377	0.974018
**The Middle East**	0.006007	1073554	12399.27	0.988061	0.876713
**Northern Africa**	-0.0076	65160.83	5850	0.910718	0.403638
**South America**	0.00073	10155.17	4599.155	0.844213	0.97932
**Southeast Asia**	0.002253	7958.273	5038.556	0.926858	0.811533
**South Asia**	0.001037	17909	8239	0.952221	0.758832
**Southern sub-Saharan Africa**	0.001411	4815.835	1545.903	0.942626	0.864376
**Western sub-Saharan Africa**	0.000612	11761.66	6983.188	0.933927	0.90621

c) Predictive out of sample metrics by year aggregated to the first administrative level for diarrhoea prevalence.

Year	Mean Err.	RMSE	Median SS	Corr.	95% Cov.
**2000**	-0.00425	2333.108	273.0552	0.918726	0.620194
**2001**	3.41E-05	584.7611	68.17568	0.760727	0.827049
**2002**	9.44E-05	1342.125	161.7645	0.927088	0.935431
**2003**	0.005974	46707.79	153	0.972276	0.881182
**2004**	-0.00333	1686.012	368.2709	0.945878	0.890665
**2005**	-0.00103	1156.03	272.4639	0.910665	0.947297
**2006**	0.002344	46979.63	250.6849	0.979726	0.911571
**2007**	0.002501	1643.3	312.4817	0.913911	0.903439
**2008**	-0.00034	3548.19	377.02	0.858565	0.849729
**2009**	-0.00048	703.0071	288.9412	0.814088	0.906068
**2010**	0.000143	906.973	371	0.896042	0.946022
**2011**	0.001062	670.9541	331.4673	0.901112	0.938904
**2012**	0.000884	1248.834	355.9981	0.909424	0.887391
**2013**	-0.00192	1533.059	305.3576	0.917968	0.94324
**2014**	0.002699	1326.408	349.1717	0.89463	0.929452
**2015**	0.002195	4019.886	644.6587	0.894225	0.948599
**2016**	0.000313	589.3417	282.2504	0.899462	0.945851
**2017**	0.00084	921.1153	444.6032	0.682852	0.785643

d) Predictive out of sample metrics by region aggregated to the first administrative level for diarrhoea prevalence.

Region	Mean Err.	RMSE	Median SS	Corr.	95% Cov.
**India**	0.001171	6658.788	1133.135	0.849075	0.897341
**Mongolia**	-0.00064	371.2068	162.5	0.573659	0.929388
**Horn of Africa**	0.002478	79565.33	642	0.977492	0.915092
**Central Asia**	-0.00017	563.4224	378.2914	0.879921	0.967378
**Central sub-Saharan Africa**	-0.0005	719.0314	529.4174	0.770593	0.935896
**Eastern sub-Saharan Africa**	0.001355	773.8415	97	0.747539	0.941136
**Malay Archipelago**	-0.00087	2382.332	270.124	0.734983	0.881355
**Mexico, central America, and the Caribbean**	0.00014	464.1459	271.4587	0.825572	0.974018
**The Middle East**	0.006007	54811.43	676	0.970489	0.876713
**Northern Africa**	-0.0076	3137.012	247	0.784779	0.403638
**South America**	0.00073	555.3197	329.3172	0.746219	0.97932
**Southeast Asia**	0.002253	386.7852	57.736	0.824825	0.811533
**South Asia**	0.001037	4121.078	928	0.923278	0.758832
**Southern sub-Saharan Africa**	0.001411	515.157	140.5291	0.870643	0.864376
**Western sub-Saharan Africa**	0.000612	922.161	536.7694	0.850218	0.90621

e) Predictive out of sample metrics by year aggregated to the second administrative level for diarrhoea prevalence.

Year	Mean Err.	RMSE	Median SS	Corr.	95% Cov.
**2000**	-0.00425	702.6304	38	0.886211	0.620194
**2001**	3.41E-05	156.9734	29.24542	0.67771	0.827049
**2002**	9.44E-05	158.4311	50.04706	0.827853	0.935431
**2003**	0.005974	8839.53	22	0.863173	0.881182
**2004**	-0.00333	216.244	103.096	0.92079	0.890665
**2005**	-0.00103	127.6837	29	0.807653	0.947297
**2006**	0.002344	10909.46	44	0.949832	0.911571
**2007**	0.002501	179.2698	51.9902	0.840909	0.903439
**2008**	-0.00034	266.5702	45	0.721166	0.849729
**2009**	-0.00048	136.6978	26.84685	0.686407	0.906068
**2010**	0.000143	178.7104	33.19359	0.786656	0.946022
**2011**	0.001062	141.767	39	0.826509	0.938904
**2012**	0.000884	165.2874	42.88677	0.831417	0.887391
**2013**	-0.00192	143.582	20	0.834853	0.94324
**2014**	0.002699	251.7417	43	0.790646	0.929452
**2015**	0.002195	344.609	90.60172	0.775888	0.948599
**2016**	0.000313	134.4009	43	0.80694	0.945851
**2017**	0.00084	184.4765	25.39926	0.566322	0.785643

f) Predictive out of sample metrics by region aggregated to the second administrative level for diarrhoea prevalence.

Region	Mean Err.	RMSE	Median SS	Corr.	95% Cov.
**India**	0.001171	273.273	103	0.594099	0.897341
**Mongolia**	-0.00064	89.50022	7.662	0.343701	0.929388
**Horn of Africa**	0.002478	17525.46	68	0.9394	0.915092
**Central Asia**	-0.00017	151.2232	49.76313	0.726618	0.967378
**Central sub-Saharan Africa**	-0.0005	197.5409	65	0.599988	0.935896
**Eastern sub-Saharan Africa**	0.001355	172.9064	42	0.605435	0.941136
**Malay Archipelago**	-0.00087	215.071	61	0.625057	0.881355
**Mexico, central America, and the Caribbean**	0.00014	134.9074	17.77103	0.637025	0.974018
**The Middle East**	0.006007	13039.31	85.12207	0.849265	0.876713
**Northern Africa**	-0.0076	831.1935	26	0.683297	0.403638
**South America**	0.00073	105.8463	12.12976	0.611133	0.97932
**Southeast Asia**	0.002253	132.0325	16.75897	0.728319	0.811533
**South Asia**	0.001037	808.336	251.7394	0.862669	0.758832
**Southern sub-Saharan Africa**	0.001411	105.2256	26.88727	0.76654	0.864376
**Western sub-Saharan Africa**	0.000612	192.1447	49.31013	0.749553	0.90621

9.0 Appendix References

1 Stevens GA, Alkema L, Black RE, *et al.* Guidelines for Accurate and Transparent Health Estimates Reporting: the GATHER statement. *Lancet* 2016; **388**: e19–23.

2 Troeger CE, Khalil IA, Blacker BF, *et al.* Quantifying risks and interventions that have affected the burden of diarrhoea among children younger than 5 years: an analysis of the Global Burden of Disease Study 2017. *Lancet Infect Dis* 2020; **20**: 37–59.

3 Dicker D, Nguyen G, Abate D, *et al.* Global, regional, and national age-sex-specific mortality and life expectancy, 1950–2017: a systematic analysis for the Global Burden of Disease Study 2017. *Lancet* 2018; **392**: 1684–735.

4 Global Health Data Exchange | GHDx. http://ghdx.healthdata.org/ (accessed May 23, 2019).

5 Murray CJL, Callender CSKH, Kulikoff XR, *et al.* Population and fertility by age and sex for 195 countries and territories, 1950–2017: a systematic analysis for the Global Burden of Disease Study 2017. *Lancet* 2018; **392**: 1995–2051.

6 Kish L. Survey sampling. New York, London: John Wiley & Sons, Inc., 1965.

7 Worldpop: get data. http://www.worldpop.org.uk/data/get_data/ (accessed June 13, 2018).

8 Fullman N, Yearwood J, Abay SM, *et al.* Measuring performance on the Healthcare Access and Quality Index for 195 countries and territories and selected subnational locations: a systematic analysis from the Global Burden of Disease Study 2016. *Lancet* 2018; **391**: 2236–71.

9 Faraway J. Problems with the predictors. In: Linear Models with R. CRC Press, 2004.

10 Database of Global Administrative Areas. GADM data version 3.6. https://gadm.org/data.html (accessed June 1, 2019).

11 Murray CJ, Ezzati M, Flaxman AD, *et al.* GBD 2010: design, definitions, and metrics. *Lancet* 2012; **380**: 2063–6.

12 Bhatt S, Cameron E, Flaxman SR, Weiss DJ, Smith DL, Gething PW. Improved prediction accuracy for disease risk mapping using Gaussian process stacked generalization. *J R Soc Interface* 2017; **14**: 20170520.

13 Head T, MechCoder, Louppe G, *et al.* scikit-optimize/scikit-optimize: v0.5.2 (Version v0.5.2). Zenodo. Zenodo, 2018 DOI:10.5281/zenodo.1207017.

14 Stein M. Interpolation of Spatial Data - Some Theory for Kriging [Internet]. Springer, 1999.

15 Gelfand AE, Diggle P, Guttorp P, Fuentes M, editors. Handbook of Spatial Statistics, 1 edition. Boca Raton: CRC Press, 2010.

16 Rue H, Martino S, Chopin N. Approximate Bayesian inference for latent Gaussian models by using integrated nested Laplace approximations. *J R Stat Soc Ser B Stat Methodol* 2009; **71**: 319–92.

17 Martins TG, Simpson D, Lindgren F, Rue H. Bayesian computing with INLA: New features. *Comput Stat Data Anal* 2013; **67**: 68–83.

18 Lidngren F, Rue H, Lindström J. An explicit link between Gaussian fields and Gaussian Markov random fields: the stochastic partial differential equation approach. *J R Stat Soc Ser B Stat Methodol* 2011; **73**: 423–98.

19 Dwyer-Lindgren L, Cork MA, Sligar A, *et al.* Mapping HIV prevalence in sub-Saharan Africa between 2000 and 2017. *Nature* 2019; **570**: 189–93.

20 Roth GA, Abate D, Abate KH, *et al.* Global, regional, and national age-sex-specific mortality for 282 causes of death in 195 countries and territories, 1980–2017: a systematic analysis for the Global Burden of Disease Study 2017. *The Lancet* 2018; **392**: 1736–88.

21 James SL, Abate D, Abate KH, *et al.* Global, regional, and national incidence, prevalence, and years lived with disability for 354 diseases and injuries for 195 countries and territories, 1990–2017: a systematic analysis for the Global Burden of Disease Study 2017. *Lancet* 2018; **392**: 1789–858.

22 Osgood-Zimmerman A, Millear AI, Stubbs RW, *et al.* Mapping child growth failure in Africa between 2000 and 2015. *Nature* 2018; **555**: 41–7.

23 De Maio FG. Income inequality measures. *J Epidemiol Community Health* 2007; **61**: 849.

24 Asada Y. Assessment of the health of Americans: the average health-related quality of life and its inequality across individuals and groups. *Popul Health Metr* 2005; **3**: 7–7.

25 Kyu HH, Abate D, Abate KH, *et al.* Global, regional, and national disability-adjusted life-years (DALYs) for 359 diseases and injuries and healthy life expectancy (HALE) for 195 countries and territories, 1990–2017: a systematic analysis for the Global Burden of Disease Study 2017. *Lancet* 2018; **392**: 1859–922.

26 GeoNetwork. The Global Administrative Unit Layers (GAUL). 2015. http://www.fao.org/geonetwork/srv/en/metadata.show?id=12691 (accessed June 1, 2017).

27 Land Processes Distributed Active Archive Center. Combined MODIS 5.1. MCD12Q1 LP DAAC NASA Land Data Prod. Serv. 2017; published online June 1. https://lpdaac.usgs.gov/products/mcd12q1v006/ (accessed June 1, 2017).

28 World Wildlife Fund. Global Lakes and Wetlands Database, Level 3. 2004. https://www.worldwildlife.org/pages/global-lakes-and-wetlands-database (accessed June 1, 2017).

29 WorldPop. WorldPop dataset. 2017; published online July 25. http://www.worldpop.org.uk/data/get_data/ (accessed July 24, 2017).

30 Tatem AJ. WorldPop, open data for spatial demography. *Sci Data* 2017; **4**: 170004.

31 Lehner B, Döll P. Development and validation of a global database of lakes, reservoirs and wetlands. *J Hydrol* 2004; **296**: 1–22.

10.0 Author Contributions

Managing the estimation or publication process

Brigette Blacker, Sandra Munro, Natalie Cormier, Kris Krohn, Laurie Marczak

Writing the first draft of the manuscript

Bobby Reiner, Simon Hay, Brigette Blacker, Sandra Munro, Kirsten Wiens

Primary responsibility for this manuscript focused on: applying analytical methods to produce estimates

Bobby Reiner, Kirsten Wiens

Primary responsibility for this manuscript focused on: seeking, cataloguing, extracting, or cleaning data; production or coding of figures and tables

Paulina Lindstedt, Mathew Baumann, Lucas Earl, Ian Pollock

Providing data or critical feedback on data sources

Foad Abd-Allah, Getaneh Alemu Abebe, Aklilu Abrham, Michael R M Abrigo, Manfred Accrombessi, maryam adabi, Daniel Adane, Oladimeji Adebayo, Rufus Adesoji Adedoyin, Victor Adekanmbi, Olatunji Adetokunboh, Mohsen Afarideh, Mohamed lemine Cheikh Brahim Ahmed, Muktar Ahmed, Turki Alanzi, Jacqueline E Alcalde-Rabanal, Birhan Alemnew, Beriwan Ali, Muhammad Ali, Vahid Alipour, Syed Aljunid, Amir Almasi-Hashiani, Nelson Alvis-Guzman, Nelson J Alvis-Zakzuk, Azmeraw T Amare, Saeed Amini, Catalina Liliana Andrei, Mina Anjomshoa, Fereshteh Ansari, Ernoiz Antriyandarti, Jalal Arabloo, Olatunde Aremu, Bahram Armoon, Krishna K Aryal, Marcel Ausloos, Leticia Avila-Burgos, Ashish Awasthi, Nefsu Awoke, Beatriz Paulina Ayala Quintanilla, Getinet Ayano, Yared Aynalem, Alaa Badawi, Senthilkumar Balakrishnan, Maciej Banach, Aleksandra Barac, Till Bärnighausen, Mohsen Bayati, Masoud Behzadifar, Meysam Behzadifar, Suraj Bhattarai, Boris Bikbov, Somayeh Bohlouli, Nicola Luigi Bragazzi, Andrey Briko, Nikolay Briko, Gessessew Bugssa, Zahid Butt, Luis Alberto Cámera, Julio Cesar Campuzano Rincon, Deborah Carvalho Malta, Franz Castro, Vijay Kumar Chattu, Bal Govind Chauhan, Devasahayam Christopher, Dinh-Toi Chu, Vera M Costa, Giovanni Damiani, Amira Hamed Darwish, Ahmad Daryani, Rajat Das Gupta, Asmamaw Demis, Kebede Deribe, Samath Dharmaratne, Preeti Dhillon, Meghnath Dhimal, Govinda Dhungana, Helen Diro, David Teye Doku, Manisha Dubey, Bereket Duko Adema, Susanna Dunachie, Senbagam Duraisamy, Aziz Eftekhari, Iman El Sayed, Maysaa El Sayed Zaki, Hajer Elkout, Sharareh Eskandarieh, Arash Etemadi, Oluchi Ezekannagha, Tamer Farag, Mohammad Fareed, Andrea Farioli, Seyed-Mohammad Fereshtehnejad, Irina Filip, Richard Franklin, Takeshi Fukumoto, Ketema Bizuwork Gebremedhin, Gebrekiros Gebremichael, Getnet Gedefaw, Ibrahim Ginawi, Addisu Gize, Nelson Gomes, Bárbara Goulart, Ayman Grada, Harish Gugnani, Yuming Guo, Rahul Gupta, Michael Tamene Haile, Arvin Haj-Mirzaian, Arya Haj-Mirzaian, Brian Hall, Hamidreza Haririan, Amir Hasanzadeh, Hadi Hassankhani, Hamid Y Hassen, Claudiu Herteliu, Hagos Hidru, Praveen Hoogar, Naznin Hossain, Mehdi Hosseinzadeh, Mowafa Househ, Guoqing Hu, Trang Huyen Nguyen, Nader Jafari Balalami, Nader Jahanmehr, Mihajlo Jakovljevic, Ahamarshan Jayaraman Nagarajan, Achala Jayatilleke, Ensiyeh Jenabi, John Ji, Jost B Jonas, Jacek Jóźwiak, Zubair Kabir, Tanuj Kanchan, André Karch, Amir Kasaeian, Gbenga Kayode, Peter Keiyoro,

Yousef Khader, Morteza Abdullatif Khafaie, Nauman Khalid, Ali Talha Khalil, Ibrahim Khalil, Rovshan Khalilov, Ejaz Khan, Junaid Khan, Md Nuruzzaman Khan, Khaled Khatab, Amir Khater, Ehsan Khodamoradi, Jagdish Khubchandani, Yun Jin Kim, Adnan Kisa, Sezer Kisa, Kewal Krishan, Pushpendra Kumar, Dian Kusuma, Aparna Lal, Dharmesh Lal, Savita Lasrado, Shanshan Li, Paulina Lindstedt, Chi Linh Hoang, Tsegaye Lolaso, Joshua Longbottom, Platon Lopukhov, Narayan Mahotra, Farzad Manafi, Navid Manafi, Mohammad Ali Mansournia, Francisco Rogerlândio Martins-Melo, Winfried März, Benjamin Mayala, Colm McAlinden, Man Mohan Mehndiratta, Ritesh G Menezes, Melkamu Merid Mengesha, Tuomo Meretoja, Bartosz Miazgowski, Edward Mills, Seyed Mostafa Mir, Amjad Mohamadi-Bolbanabad, Dara Mohammad, Karzan Mohammad, Aso Mohammad Darwesh, Naser Mohammad Gholi Mezerji, Noushin Mohammadifard, Shafiu Mohammed, Jemal Abdu Mohammed, Mehdi Mojadam, Ali Mokdad, Masoud Moradi, Mohammad Moradi-Joo, Simin Mouodi, Seyyed Meysam Mousavi, Miliva Mozaffor, GVS Murthy, Kamarul Imran Musa, Ghulam Mustafa, Saravanan Muthupandian, Mohsen Naghavi, Seyed Sina Naghibi Irvani, Javad Nazari, Ionut Negoi, Josephine Ngunjiri, QuynhAnh Nguyen, Dabere Nigatu, Chukwudi Nnaji, Marzieh Nojomi, Jean Jacques Noubiap, Andrew T Olagunju, Ahmed Omar Bali, Obinna Onwujekwe, Doris V Oretga-Altamirano, Mahesh P A, Jagadish Rao Padubidri, Adrian Pana, Ajay Patle, Veincent Christian Pepito, Alexandre Pereira, Bakhtiar Piroozi, Mario Poljak, Maarten Postma, Hossein Poustchi, Sergio Prada, Ashwini Pujar, Hedley Quintana, Mohammad Rabiee, Navid Rabiee, Amir Radfar, Alireza Rafiei, Fakher Rahim, Vafa Rahimi-Movaghar, Kiana Ramezanzadeh, Saleem Rana, Chhabi Lal Ranabhat, David Laith Rawaf, Salman Rawaf, Lal Rawal, Robert Reiner, Vishnu Renjith, Andre M N Renzaho, Seyed Mohammad Riahi, Ana Isabel Ribeiro, María Jesús Ríos-Blancas, Leonardo Roever, Luca Ronfani, Ali Rostami, Enrico Rubagotti, Salvatore Rubino, Ehsan Sadeghi, Rajesh Sagar, Mohammad Ali Sahraian, S Mohammad Sajadi, Mohammad Reza Salahshoor, Hosni Salem, Hamideh Salimzadeh, Evanson Sambala, Abdallah M Samy, Sivan Saraswathy, Abdur Razzaque Sarker, Brijesh Sathian, Maheswar Satpathy, David C Schwebel, Seyedmojtaba Seyedmousavi, Masood Ali Shaikh, Mehran Shams-Beyranvand, Mehdi Sharif, Wondimeneh Shiferaw, Mika Shigematsu, Zabta Khan Shinwari, Soraya Siabani, Jasvinder Singh, Malede Mequanent Sisay, Eirini Skiadaresi, Mohammadreza Sobhiyeh, Muluken Bekele Sorrie, Chandrashekhar T Sreeramareddy, Agus Sudaryanto, Hafiz Ansar Rasul Suleria, Desalegn Tadese, Cuong Tat Nguyen, Nuno Taveira, Bineyam Taye, Arash Tehrani-Banihashemi, Girmay Teklay, Berhe Tesfay, Sathish Thirunavukkarasu, Nihal Thomas, Alemayehu Toma, Marcos Roberto Tovani-Palone, Bach Tran, Christopher Troeger, Irfan Ullah, Bhaskaran Unnikrishnan, Sahel Valadan Tahbaz, Pascual Ruben Valdez, Yasser Vasseghian, Yasir Waheed, Yuan-Pang Wang, Ronny Westerman, Taweewat Wiangkham, Kirsten Wiens, Tissa Wijeratne, Charles Shey Wiysonge, Dawit Zewdu Wondafrash, Gelin Xu, Ali Yadollahpour, Alex Yeshaneh, Mekdes Tigistu Yilma, Naohiro Yonemoto, Mustafa Younis, Sojib Bin Zaman, Mohammad Zamani, Hamed Zandian, Nejimu Zepro, Xiu-Ju Zhao, Arash Ziapour

Development of methods or computational machinery

Hedayat Abbastabar, Aklilu Abrham, Muktar Ahmed, Saeed Amini, Suleman Atique, Mathew Baumann, Franz Castro, Giovanni Damiani, Farah Daoud, Rajat Das Gupta, Aniruddha Deshpande, Helen Diro, Lucas Earl, Ali Akbar Fazaeli, Joseph Frostad, Michael Tamene Haile, Nathaniel Henry, Hagos Hidru, Mowafa Househ, Ensiyeh Jenabi, Kimberly Johnson, Mohammad Khazaei, Adnan Kisa, Sezer Kisa, Paulina Lindstedt, Ali Mokdad, Miliva Mozaffor, Mohsen Naghavi, Javad Nazari, QuynhAnh Nguyen, Mohammad Rabiee, Navid Rabiee, Chhabi Lal Ranabhat, Robert Reiner, Vishnu Renjith, Seyed Mohammad Riahi, Mohammad Reza Salahshoor, Abdallah M Samy, Maheswar Satpathy, Alyssa Sbarra, Mohammadreza Sobhiyeh, Christopher Troeger, Sahel Valadan Tahbaz, Yasser Vasseghian, Theo Vos, Kirsten Wiens, Arash Ziapour

Providing critical feedback on methods or results

Degu Abate, Aberash Abay, Hedayat Abbastabar, Foad Abd-Allah, Ahmed Abdelalim, Ibrahim Abdollahpour, Lucas Guimarães Abreu, Michael R M Abrigo, Manfred Accrombessi, Dilaram Acharya, Daniel Adane, Oladimeji Adebayo, Rufus Adesoji Adedoyin, Victor Adekanmbi, Olatunji Adetokunboh, Mohsen Afarideh, Keivan Ahmadi, Mehdi Ahmadi, Anwar Ahmed, Muktar Ahmed, Dessalegn Ajema, Olufemi Ajumobi, Temesgen Yihunie Akalu, Ali S Akanda, Genet Melak Alamene, Jacqueline E Alcalde-Rabanal, Birhan Alemnew, Zewdie Aderaw Alemu, Muhammad Ali, Mehran Alijanzadeh, Vahid Alipour, Syed Aljunid, Ali Almasi, Amir Almasi-Hashiani, Hesham Al-Mekhlafi, Khalid Altirkawi, Nelson J Alvis-Zakzuk, Azmeraw T Amare, Saeed Amini, Arianna Maever Amit, Catalina Liliana Andrei, Mina Anjomshoa, Fereshteh Ansari, Carl Abelardo Antonio, Ernoiz Antriyandarti, Seth Christopher Yaw Appiah, Jalal Arabloo, Olatunde Aremu, Krishna K Aryal, Mehran Asadi-Aliabadi, Mohsen Asadi-Lari, Suleman Atique, Sachin Atre, Marcel Ausloos, Ashish Awasthi, Nefsu Awoke, Beatriz Paulina Ayala Quintanilla, Getinet Ayano, Martin Ayanore, Asnakew Ayele, Yared Aynalem, Muluken Azage, Samad Azari, EbRahim Babaee, Alaa Badawi, Frank Badu Osei, Shankar Bakkannavar, Senthilkumar Balakrishnan, Maciej Banach, Aleksandra Barac, Till Bärnighausen, Huda Basaleem, Quique Bassat, Mohsen Bayati, Masoud Behzadifar, Meysam Behzadifar, Yibeltal Alemu Bekele, Michelle Bell, Derrick Bennett, Tina Beyranvand, Anusha G Bhat, Krittika Bhattacharyya, Suraj Bhattarai, Ali Bijani, Boris Bikbov, Raaj Kishore Biswas, Kassawmar Angaw Bogale, Somayeh Bohlouli, Oliver Brady, Nicola Luigi Bragazzi, Andrey Briko, Nikolay Briko, Gessessew Bugssa, Zahid Butt, Ismael Campos-Nonato, Rosario Cárdenas, Felix Carvalho, Deborah Carvalho Malta, Franz Castro, Collins Chansa, Pranab Chatterjee, Vijay Kumar Chattu, Bal Govind Chauhan, Ken Chin, Yilma Chisha, Devasahayam Christopher, Dinh-Toi Chu, Rafael Claro, Giovanni Damiani, Amira Hamed Darwish, Ahmad Daryani, Jai Das, Rajat Das Gupta, Tamirat Tesfaye Dasa, Jan-Walter De Neve, Asmamaw Demis, Assefa Desalew, Aniruddha Deshpande, Preeti Dhillon, Meghnath Dhimal, Daniel Diaz, Eric L Ding, Shirin Djalalinia, David Teye Doku, Christiane Dolecek, Manisha Dubey, Bereket Duko Adema, Susanna Dunachie, Senbagam Duraisamy, Andem Effiong, Aziz Eftekhari, Iman El Sayed, Maysaa El Sayed Zaki, Maha El Tantawi, Demelash Elemineh, Aklilu Endalamaw, Sharareh Eskandarieh, Alireza Esteghamati, Arash Etemadi, Oluchi Ezekannagha, Tamer Farag, Emerito Jose A Faraon, Mohammad Fareed, Roghiyeh Faridnia, Andre Faro, Hossein Farzam, Alebachew Fasil, Mehdi Fazlzadeh, Netsanet Fentahun, Eduarda Fernandes, Irina Filip, Florian Fischer, Masoud Foroutan, Joel Francis, Richard Franklin, Takeshi Fukumoto, Kidane Tadesse Gebremariam, Ketema Bizuwork Gebremedhin, Gebreamlak Gebremeskel, Gebrekiros Gebremichael, Getnet Gedefaw, Ayele Geleto, Chalachew Genet, Kebede Embaye Gezae, Ahmad Ghashghaee, Fariba Ghassemi, Paramjit Gill, Addisu Gize, Srinivas Goli, Nelson Gomes, Sameer Gopalani, Bárbara Goulart, Ayman Grada, Davide Guido, Rafael Guimarães, Yuming Guo, Rahul Gupta, Rajeev Gupta, Nima Hafezi-Nejad, Desta Haftu, Michael Tamene Haile, Arvin Haj-Mirzaian, Arya Haj-Mirzaian, Brian Hall, Demelash Woldeyohannes Handiso, Ninuk Hariyani, Md Mehedi Hasan, Amir Hasanzadeh, Hadi Hassankhani, Hamid Y Hassen, Behnam Heidari, Delia Hendrie, Nathaniel Henry, Claudiu Herteliu, Fatemeh Heydarpour, Hagos Hidru, Praveen Hoogar, Mojtaba Hoseini-Ghahfarokhi, Naznin Hossain, Mostafa Hosseini, Mehdi Hosseinzadeh, Mowafa Househ, Guoqing Hu, Ayesha Humayun, Syed Ather Hussain, Trang Huyen Nguyen, Segun Emmanuel Ibitoye, Olayinka Ilesanmi, Milena Ilic, Leeberk Raja Inbaraj, Sheikh Mohammed Shariful Islam, Chinwe Juliana Iwu, Anelisa Jaca, Nader Jafari Balalami, Mihajlo Jakovljevic, Ahamarshan Jayaraman Nagarajan, Achala Jayatilleke, Ensiyeh Jenabi, Ravi Prakash Jha, Vivekanand Jha, John Ji, Peng Jia, Kimberly Johnson, Jacek Jóźwiak, Zubair Kabir, Amaha Kahsay, Hamed Kalani, Tanuj Kanchan, Behzad Karami Matin, André Karch, Surendra Karki, Amir Kasaeian, Gebremicheal Kasahun, Gbenga Kayode, Ali Kazemi Karyani, Peter Keiyoro, Daniel Bekele Ketema, Yousef Khader, Morteza Abdullatif Khafaie, Ali Talha Khalil, Ibrahim Khalil, Rovshan Khalilov, Gulfaraz Khan, Junaid Khan, Md Nuruzzaman Khan, Khaled Khatab, Mona Khater, Amir Khater, Alireza Khatony, Maryam Khayamzadeh, Mohammad Khazaei, Salman Khazaei, Mohammad Hossein Khosravi, Jagdish Khubchandani, Ali Kiadaliri, Yun Jin Kim, Ruth Kimokoti, Adnan Kisa, Sezer Kisa, Niranjan Kissoon, Margaret Kosek, Ai Koyanagi, Moritz

Kraemer, Kewal Krishan, Nuworza Kugbey, Manasi Kumar, Pushpendra Kumar, Dian Kusuma, Carlo La Vecchia, Aparna Lal, Dharmesh Lal, Faris Lami, Savita Lasrado, Mostafa Leili, Sonia Lewycka, Shanshan Li, Chi Linh Hoang, Shai Linn, Tsegaye Lolaso, Platon Lopukhov, Sameh Magdeldin, Mohamed Magdy Abd EL Razek, Hassan Magdy Abdel Razek, Phetole Mahasha, Abdullah Mamun, Farzad Manafi, Navid Manafi, Mohammad Ali Mansournia, Christopher Chabila Mapoma, Laurie Marczak, Francisco Rogerlândio Martins-Melo, Anthony Masaka, Manu Mathur, Pallab K Maulik, Benjamin Mayala, Colm McAlinden, Birhanu Geta Meharie, Kala M Mehta, Feleke Mekonnen, Addisu Melese, Ziad Memish, Ritesh G Menezes, Melkamu Merid Mengesha, Getnet Mengistu, Beyene Meressa, Tuomo Meretoja, Kebadnew Mulatu Mihretie, Ted R Miller, Edward Mills, Seyed Mostafa Mir, parvaneh mirabi, Erkin Mirrakhimov, Amjad Mohamadi-Bolbanabad, Dara Mohammad, Yousef Mohammad, Aso Mohammad Darwesh, Naser Mohammad Gholi Mezerji, Ammas Mohammed, Shafiu Mohammed, Jemal Abdu Mohammed, Farnam Mohebi, Mehdi Mojadam, Ali Mokdad, Yoshan Moodley, Ghobad Moradi, Masoud Moradi, Mohammad Moradi-Joo, Maziar Moradi-Lakeh, Paula Moraga, Abbas Mosapour, Seyyed Meysam Mousavi, Miliva Mozaffor, Atalay Muluneh, GVS Murthy, Kamarul Imran Musa, Ghulam Mustafa, Saravanan Muthupandian, Mehdi Naderi, Sharath B Nagaraja, Mohsen Naghavi, Farid Najafi, Vinay Nangia, Javad Nazari, Ionut Negoi, Josephine Ngunjiri, Dabere Nigatu, Dina Nur Anggraini Ningrum, Chukwudi Nnaji, In-Hwan Oh, Andrew T Olagunju, Ahmed Omar Bali, Obinna Onwujekwe, Doris V Oretga-Altamirano, Osayomwanbo Osarenotor, Mayowa Owolabi, Mahesh P A, Jagadish Rao Padubidri, Adrian Pana, Tahereh Pashaei, Sanghamitra Pati, kebreab Paulos, Veincent Christian Pepito, Alexandre Pereira, Norberto Perico, David Pigott, Bakhtiar Piroozi, James Platts-Mills, Mario Poljak, Maarten Postma, Farshad Pourmalek, Akram Pourshams, Sergio Prada, Ashwini Pujar, Hedley Quintana, Mohammad Rabiee, Navid Rabiee, Amir Radfar, Fakher Rahim, Vafa Rahimi-Movaghar, Fatemeh Rajati, Kiana Ramezanzadeh, Saleem Rana, Chhabi Lal Ranabhat, Davide Rasella, David Laith Rawaf, Salman Rawaf, Lal Rawal, Robert Reiner, Giuseppe Remuzzi, Vishnu Renjith, Andre M N Renzaho, Melese Reta, Seyed Mohammad Riahi, Ana Isabel Ribeiro, Jennifer Rickard, Carlos Rios-González, Leonardo Roever, Elias Roro, Ali Rostami, Dietrich Rothenbacher, Enrico Rubagotti, Salvatore Rubino, Siamak Sabour, Ehsan Sadeghi, Mahdi Safdarian, Rajesh Sagar, Mohammad Ali Sahraian, S Mohammad Sajadi, Hosni Salem, Marwa Salem, Yahya Salimi, Hamideh Salimzadeh, Evanson Sambala, Abdallah M Samy, Juan Sanabria, Itamar Santos, Sivan Saraswathy, Abdur Razzaque Sarker, Benn Sartorius, Brijesh Sathian, Maheswar Satpathy, Alyssa Sbarra, Lauren Schaeffer, David C Schwebel, Anbissa Muleta Senbeta, Subramanian Senthilkumaran, Seyedmojtaba Seyedmousavi, Hosein Shabaninejad, Amira Shaheen, Masood Ali Shaikh, Seifadin Ahmed Shallo, Mehran Shams-Beyranvand, Mohammadbagher Shamsi, Morteza Shamsizadeh, Mehdi Sharif, Muki Shey, Kenji Shibuya, Wondimeneh Shiferaw, Mika Shigematsu, Jae Il Shin, Zabta Khan Shinwari, Rahman Shiri, Reza Shirkoohi, Shivakumar KM, Si Si, Soraya Siabani, Jasvinder Singh, Narinder Pal Singh, Koku Sisay, Malede Mequanent Sisay, Eirini Skiadaresi, Mohammadreza Sobhiyeh, Shahin Soltani, Moslem Soofi, Joan B Soriano, Muluken Bekele Sorrie, Ireneous Soyiri, Chandrashekhar T Sreeramareddy, Agus Sudaryanto, Mu'awiyyah Babale Sufiyan, Hafiz Ansar Rasul Suleria, Rizwan Suliankatchi Abdulkader, Bryan L Sykes, Desalegn Tadese, Zemenu Tadesse, Cuong Tat Nguyen, Nuno Taveira, Bineyam Taye, Girmay Teklay, Mohamad-Hani Temsah, Berhe Tesfay, Fisaha Haile Tesfay, Masresha Tessema, Sathish Thirunavukkarasu, Nihal Thomas, Kenean Getaneh Tlaye, Boikhutso Tlou, Marcos Roberto Tovani-Palone, Bach Tran, Khanh Bao Tran, Indang Trihandini, Christopher Troeger, Reta Tsegaye, Irfan Ullah, Bhaskaran Unnikrishnan, Sahel Valadan Tahbaz, Santosh Varughese, Yasser Vasseghian, Sebastian Vollmer, Theo Vos, Fiseha Wadilo, Yasir Waheed, Yafeng Wang, Yuan-Pang Wang, Ronny Westerman, Taweewat Wiangkham, Kirsten Wiens, Tissa Wijeratne, Charles Shey Wiysonge, Haileab Fekadu Wolde, Dawit Zewdu Wondafrash, Ai-Min Wu, Ali Yadollahpour, Tomohide Yamada, Mehdi Yaseri, Alex Yeshaneh, Mekdes Tigistu Yilma, EbRahim M Yimer, Paul Yip, Biruck Desalegn Yirsaw, Engida Yisma, Naohiro Yonemoto, Mustafa Younis, Chuanhua Yu, Hasan Yusefzadeh, Sojib Bin Zaman, Carlos Zambrana-Torrelio, Hamed Zandian, Ayalew Jejaw Zeleke, Nejimu Zepro, Taye Abuhay Zewale, Dongyu Zhang, Yunquan Zhang, Yikun Zhu, Arash Ziapour, Sanjay Zodpey

Drafting the manuscript or revising it critically for important intellectual content

Hedayat Abbastabar, Ahmed Abdelalim, Getaneh Alemu Abebe, Kedir Abegaz, Lucas Guimarães Abreu, Daniel Adane, Oladimeji Adebayo, Victor Adekanmbi, Olatunji Adetokunboh, Mohsen Afarideh, Muktar Ahmed, Rushdia Ahmed, Olufemi Ajumobi, Temesgen Yihunie Akalu, Ali S Akanda, Genet Melak Alamene, Jacqueline E Alcalde-Rabanal, Birhan Alemnew, Beriwan Ali, Vahid Alipour, Ali Almasi, Amir Almasi-Hashiani, Hesham Al-Mekhlafi, Nelson Alvis-Guzman, Saeed Amini, Catalina Liliana Andrei, Mina Anjomshoa, Fereshteh Ansari, Carl Abelardo Antonio, Jalal Arabloo, Olatunde Aremu, Afsaneh Arzani, Marcel Ausloos, Ashish Awasthi, Nefsu Awoke, Samad Azari, EbRahim Babaee, Alaa Badawi, Shankar Bakkannavar, Senthilkumar Balakrishnan, Maciej Banach, Aleksandra Barac, Till Bärnighausen, Mohsen Bayati, Neeraj Bedi, Masoud Behzadifar, Yibeltal Alemu Bekele, Michelle Bell, Derrick Bennett, Anusha G Bhat, Krittika Bhattacharyya, Soumyadeep Bhaumik, Raaj Kishore Biswas, Brigette Blacker, Kassawmar Angaw Bogale, Oliver Brady, Andrey Briko, Nikolay Briko, Gessessew Bugssa, Ismael Campos-Nonato, Rosario Cárdenas, Felix Carvalho, Deborah Carvalho Malta, Franz Castro, Pranab Chatterjee, Vijay Kumar Chattu, Devasahayam Christopher, Rafael Claro, Giovanni Damiani, Ahmad Daryani, Rajat Das Gupta, Tamirat Tesfaye Dasa, Claudio Davila-Cervantes, Nicole Davis Weaver, Dragos Virgil Davitoiu, Jan-Walter De Neve, Asmamaw Demis, Edgar Denova-Gutiérrez, Kebede Deribe, Getenet Dessie, Daniel Diaz, David Teye Doku, Manisha Dubey, Eleonora Dubljanin, Susanna Dunachie, Andre Duraes, Senbagam Duraisamy, Lucas Earl, Andem Effiong, Iman El Sayed, Maysaa El Sayed Zaki, Maha El Tantawi, Shaimaa El-Jaafary, Aisha Elsharkawy, Shymaa Enany, Aklilu Endalamaw, Tewodros Eshete, Alireza Esteghamati, Arash Etemadi, Roghiyeh Faridnia, Andrea Farioli, Andre Faro, Seyed-Mohammad Fereshtehnejad, Eduarda Fernandes, Florian Fischer, Masoud Foroutan, Joel Francis, Richard Franklin, Takeshi Fukumoto, Kidane Tadesse Gebremariam, Gebreamlak Gebremeskel, Gebrekiros Gebremichael, Getnet Gedefaw, Ayele Geleto, Ahmad Ghashghaee, Nelson Gomes, Sameer Gopalani, Bárbara Goulart, Rafael Guimarães, Rajeev Gupta, Nima Hafezi-Nejad, Michael Tamene Haile, Brian Hall, Demelash Woldeyohannes Handiso, Ninuk Hariyani, Ahmed Hasaballah, Hadi Hassankhani, Claudiu Herteliu, Hagos Hidru, Praveen Hoogar, Mojtaba Hoseini-Ghahfarokhi, Mostafa Hosseini, Mowafa Househ, Guoqing Hu, Ayesha Humayun, Syed Ather Hussain, Trang Huyen Nguyen, Segun Emmanuel Ibitoye, Olayinka Ilesanmi, Milena Ilic, Sheikh Mohammed Shariful Islam, Chinwe Juliana Iwu, Nader Jafari Balalami, Nader Jahanmehr, Mihajlo Jakovljevic, Amir Jalali, Ensiyeh Jenabi, Ravi Prakash Jha, Vivekanand Jha, Jost B Jonas, Jacek Jóźwiak, Ali Kabir, Hamed Kalani, André Karch, Gbenga Kayode, Peter Keiyoro, Yousef Khader, Morteza Abdullatif Khafaie, Ejaz Khan, Gulfaraz Khan, Junaid Khan, Mona Khater, Jagdish Khubchandani, Ali Kiadaliri, Yun Jin Kim, Adnan Kisa, Margaret Kosek, Ai Koyanagi, Nuworza Kugbey, Dian Kusuma, Carlo La Vecchia, Ben Lacey, Dharmesh Lal, Savita Lasrado, Paul Lee, Chi Linh Hoang, Rakesh Lodha, Joshua Longbottom, Platon Lopukhov, Mohamed Magdy Abd EL Razek, Hassan Magdy Abdel Razek, Abdullah Mamun, Farzad Manafi, Navid Manafi, Ana-Laura Manda, Mohammad Ali Mansournia, Dadi Marami, Laurie Marczak, Francisco Rogerlândio Martins-Melo, Manu Mathur, Colm McAlinden, Ravi Mehrotra, Ritesh G Menezes, Melkamu Merid Mengesha, Tuomo Meretoja, Ted R Miller, Molly Miller-Petrie, Edward Mills, Seyed Mostafa Mir, Dara Mohammad, Karzan Mohammad, Yousef Mohammad, Shafiu Mohammed, Jemal Abdu Mohammed, Farnam Mohebi, Mehdi Mojadam, Ali Mokdad, Ghobad Moradi, Masoud Moradi, Mohammad Moradi-Joo, Maziar Moradi-Lakeh, Paula Moraga, Abbas Mosapour, Seyyed Meysam Mousavi, Miliva Mozaffor, Sandra Munro, Kamarul Imran Musa, Ghulam Mustafa, Mehdi Naderi, Sharath B Nagaraja, Seyed Sina Naghibi Irvani, Vinay Nangia, Javad Nazari, Duduzile Ndwandwe, Ionut Negoi, Josephine Ngunjiri, Dabere Nigatu, Jean Jacques Noubiap, In-Hwan Oh, Andrew T Olagunju, Obinna Onwujekwe, Doris V Oretga-Altamirano, Mayowa Owolabi, Mahesh P A, Jagadish Rao Padubidri, Adrian Pana, Sanghamitra Pati, George Patton, Veincent Christian Pepito, Alexandre Pereira, Konrad Pesudovs, David Pigott, James Platts-Mills, Mario Poljak, Maarten Postma, Farshad Pourmalek, Ashwini Pujar, Hedley Quintana, Mohammad Rabiee, Navid Rabiee, Fakher Rahim,

Vafa Rahimi-Movaghar, Muhammad Aziz Rahman, Fatemeh Rajati, Davide Rasella, David Laith Rawaf, Salman Rawaf, Lal Rawal, Robert Reiner, Vishnu Renjith, Andre M N Renzaho, Satar Rezaei, Seyed Mohammad Riahi, Ana Isabel Ribeiro, Jennifer Rickard, Leonardo Roever, Luca Ronfani, Dietrich Rothenbacher, Salvatore Rubino, Anas Saad, Saeed Safari, Mahdi Safdarian, Rajesh Sagar, Nasir Salam, Farkhonde Salehi, Saleh Salehi zahabi, Hosni Salem, Marwa Salem, Abdallah M Samy, Juan Sanabria, Itamar Santos, Maheswar Satpathy, David C Schwebel, Seyedmojtaba Seyedmousavi, Ali Shalash, Morteza Shamsizadeh, Mehdi Sharif, Wondimeneh Shiferaw, Mika Shigematsu, Apurba Shil, Jasvinder Singh, Dhirendra Narain Sinha, Eirini Skiadaresi, anton Sokhan, Joan B Soriano, Muluken Bekele Sorrie, Chandrashekhar T Sreeramareddy, Mu'awiyyah Babale Sufiyan, Bryan L Sykes, Desalegn Tadese, Hagos Tasew, Cuong Tat Nguyen, Nuno Taveira, Bineyam Taye, Arash Tehrani-Banihashemi, Berhe Tesfay, Fisaha Haile Tesfay, Kavumpurathu Thankappan, Marcos Roberto Tovani-Palone, Eugenio Traini, Bach Tran, Khanh Bao Tran, Reta Tsegaye, Irfan Ullah, Sahel Valadan Tahbaz, Yasser Vasseghian, Yousef Veisani, Francesco S Violante, Yuan-Pang Wang, Catherine Welgan, Ronny Westerman, Taweewat Wiangkham, Kirsten Wiens, Tissa Wijeratne, Charles Shey Wiysonge, Haileab Fekadu Wolde, Dawit Zewdu Wondafrash, Ai-Min Wu, Gelin Xu, Hossein Yahyazadeh, Mekdes Tigistu Yilma, Hebat-Allah Yousof, Hasan Yusefzadeh, Mohammad Zamani, Nejimu Zepro, Xiu-Ju Zhao, Arash Ziapour

Management of the overall research enterprise (for example, through membership in the Scientific Council)

Mohamed lemine Cheikh Brahim Ahmed, Saeed Amini, Huda Basaleem, Brigette Blacker, Deborah Carvalho Malta, Devasahayam Christopher, Gebre Demoz, Shymaa Enany, Ensiyeh Jenabi, Mohammad Hossein Khosravi, Van Lansingh, Aubrey Levine, Ali Mokdad, Miliva Mozaffor, Mohsen Naghavi, Javad Nazari, George Patton, Vishnu Renjith, Seyed Mohammad Riahi, Rajesh Sagar, Abdur Razzaque Sarker, Benn Sartorius, Kenji Shibuya, Sahel Valadan Tahbaz, Yasser Vasseghian, Tissa Wijeratne, Arash Ziapour
