# Supplementary material for: Mapping geographical inequalities in childhood diarrhoeal morbidity and mortality in low-income and middle-income countries, 2000–17: analysis for the Global Burden of Disease Study 2017
Source: Lancet. 2020 Jun 6;395(10239):1779–801. doi: 10.1016/S0140-6736(20)30114-8 (PMC7314599; doi:10.1016/S0140-6736(20)30114-8)
Supplement: Supplementary appendix 2 [file mmc2.pdf]

THE LANCET

Supplementary appendix

This appendix formed part of the original submission and has been peer reviewed. We post it as supplied by the authors.

Supplement to: Reiner Jr RC, Hay SI, on behalf of the Local Burden of Disease Diarrhoea Collaborators. Mapping geographical inequalities in childhood diarrhoeal morbidity and mortality in low-income and middle-income countries, 2000–17: analysis for the Global Burden of Disease Study 2017. *Lancet* 2020; published online May 6. https://doi.org/10.1016/S0140-6736(20)30114-8.

Supplementary results: Deaths, mortality rate (per 10,000), incident cases, and incidence rate (per 10,000) in 2000 and 2017 by country and unit. Upper and lower values indicate bounds of 95% UI.

Table 1: Diarrhoea Mortality by Country

	2000 mortality						2017 mortality					
Country	mean rate (per 10,000)	lower rate (per 10,000)	upper rate (per 10,000)	mean count	lower count	upper count	mean rate (per 10,000)	lower rate (per 10,000)	upper rate (per 10,000)	mean count	lower count	upper count
**Central Europe, Eastern Europe, and Central Asia**												
Kyrgyzstan	0.8	0.6	1.2	457	318	633	0.2	0.1	0.2	118	89	158
Mongolia	0.2	0.2	0.2	48	43	53	0.1	0.1	0.1	27	24	30
Tajikistan	2.8	2.0	3.8	2500	1820	3400	1.3	1.1	1.5	1590	1390	1820
Turkmenistan	1.4	1.1	1.8	637	493	806	0.1	0.1	0.1	58	43	80
Uzbekistan	0.2	0.2	0.3	694	538	887	0.0	0.0	0.1	156	109	224
**Latin America and Caribbean**												
Belize	0.3	0.2	0.4	9	7	13	0.1	0.1	0.2	5	4	7
Bolivia	1.3	1.1	1.4	1530	1310	1740	0.2	0.1	0.2	238	201	275
Brazil	0.8	0.7	1.0	13200	11200	15800	0.1	0.1	0.1	1800	1500	2120
Colombia	0.3	0.3	0.4	1450	1210	1730	0.1	0.1	0.1	298	237	366
Costa Rica	0.1	0.1	0.1	35	24	49	0.0	0.0	0.0	10	7	14
Dominican Republic	0.7	0.6	0.8	773	675	884	0.2	0.1	0.2	183	149	219
Ecuador	0.5	0.4	0.6	812	673	980	0.1	0.1	0.1	149	125	180
El Salvador	0.7	0.5	0.9	539	412	702	0.1	0.1	0.1	61	46	79
Guatemala	1.7	1.3	2.2	3050	2240	4000	0.6	0.5	0.8	1290	1040	1560
Guyana	0.8	0.6	0.9	80	63	99	0.2	0.2	0.3	17	14	21
Haiti	4.5	4.0	4.9	5280	4790	5830	2.1	1.9	2.3	3140	2870	3380
Honduras	1.0	0.8	1.3	966	756	1210	0.2	0.2	0.3	271	209	366
Jamaica	0.1	0.1	0.1	19	15	25	0.0	0.0	0.1	7	5	11
Mexico	0.4	0.3	0.4	4720	3920	5580	0.1	0.1	0.1	1250	987	1530
Nicaragua	1.1	0.9	1.3	728	609	872	0.1	0.1	0.2	86	65	111
Panama	0.3	0.2	0.4	94	66	128	0.2	0.2	0.3	77	57	101
Paraguay	0.5	0.4	0.6	312	247	394	0.1	0.1	0.1	61	50	76
Peru	0.4	0.4	0.5	1330	1100	1540	0.1	0.1	0.1	366	316	417
Suriname	0.9	0.7	1.1	39	30	50	0.3	0.2	0.3	13	11	16
**North Africa and Middle East**												
Afghanistan	3.2	2.3	4.1	12700	9320	16400	1.0	0.7	1.3	5910	4200	7920
Algeria	0.3	0.2	0.3	1040	852	1210	0.0	0.0	0.0	166	138	196
Egypt	2.0	1.6	2.4	16100	13100	19200	0.5	0.4	0.7	6460	5170	8260
Iraq	0.2	0.2	0.3	1040	778	1370	0.1	0.1	0.1	561	399	772
Jordan	0.1	0.1	0.2	66	41	107	0.0	0.0	0.0	35	22	54
Morocco	1.1	0.9	1.3	3730	3130	4500	0.2	0.2	0.3	687	564	797
Sudan	3.6	2.9	4.4	17100	14000	20900	1.2	0.9	1.5	7220	5520	9320
Syria	0.1	0.1	0.1	171	115	250	0.0	0.0	0.0	18	11	28
Tunisia	0.1	0.1	0.1	63	52	73	0.0	0.0	0.0	21	17	26
Yemen	5.0	3.5	6.9	17400	12300	24200	1.5	1.3	1.9	7400	6140	8900
**South Asia**												
Bangladesh	1.4	1.0	1.9	24700	17900	34400	0.2	0.1	0.3	3060	2170	4500
Bhutan	0.9	0.6	1.4	68	42	104	0.1	0.1	0.1	7	5	11
India	2.5	2.2	3.0	333000	284000	397000	0.8	0.7	0.9	103000	86300	119000
Nepal	1.8	1.4	2.5	6450	4770	8830	0.2	0.2	0.3	753	545	1030
Pakistan	2.6	2.1	3.3	61600	49900	77100	1.1	0.8	1.3	28900	22800	36100
**Southeast Asia, East Asia, and Oceania**												
Cambodia	1.3	0.9	1.8	2290	1570	3220	0.2	0.1	0.3	300	193	448
Indonesia	1.3	1.2	1.4	28100	25800	30600	0.5	0.4	0.6	10600	9390	11800
Laos	3.3	2.1	5.3	2620	1630	4190	1.2	0.7	1.8	949	600	1450
Myanmar	1.6	1.0	2.4	8400	5120	12500	0.5	0.3	0.7	2000	1250	2980
Papua New Guinea	1.7	1.4	1.9	1450	1260	1710	1.0	0.8	1.1	1310	1150	1510
Philippines	0.6	0.5	0.6	6310	5580	7040	0.3	0.3	0.4	3760	3330	4250
Sri Lanka	0.0	0.0	0.1	85	56	130	0.0	0.0	0.0	12	8	18
Thailand	0.1	0.1	0.2	497	323	763	0.0	0.0	0.1	129	80	188
Timor-Leste	1.5	1.2	1.9	247	201	309	0.4	0.4	0.5	75	63	89
Vietnam	0.1	0.1	0.1	739	448	1100	0.0	0.0	0.0	128	78	193

Table 1: Diarrhoea Mortality by Country *(continued)*

	2000 mortality						2017 mortality					
Country	mean rate (per 10,000)	lower rate (per 10,000)	upper rate (per 10,000)	mean count	lower count	upper count	mean rate (per 10,000)	lower rate (per 10,000)	upper rate (per 10,000)	mean count	lower count	upper count
**Sub-Saharan Africa**												
Angola	7.5	6.3	8.8	21400	17800	24800	2.1	1.8	2.3	10200	9010	11600
Benin	3.4	2.6	4.3	4300	3300	5440	1.9	1.4	2.4	3610	2760	4570
Botswana	1.7	1.6	1.8	364	338	399	0.8	0.7	0.9	196	175	220
Burkina Faso	6.2	4.5	8.0	14500	10600	18800	2.0	1.5	2.8	7520	5520	10400
Burundi	4.4	3.5	5.5	4670	3690	5830	1.7	1.3	2.1	3040	2300	3860
Cameroon	5.5	4.5	6.5	14200	11700	16800	1.9	1.4	2.6	7940	5850	10800
Central African Republic	6.2	5.7	6.8	3800	3460	4170	6.9	6.2	7.6	4160	3740	4600
Chad	10.4	8.6	12.4	18100	14900	21600	6.5	4.7	8.8	20100	14400	27000
Comoros	4.9	3.5	6.6	434	314	590	0.8	0.6	1.1	69	49	91
Côte d'Ivoire	3.1	2.5	4.0	9380	7310	11900	2.0	1.3	2.7	7610	5150	10400
Democratic Republic of the Congo	3.3	3.1	3.6	30100	28200	32400	1.5	1.4	1.6	19500	18100	21000
Djibouti	3.3	2.1	4.8	345	222	508	0.7	0.4	1.1	102	63	167
Equatorial Guinea	3.3	2.8	3.7	415	360	465	0.5	0.4	0.6	89	75	106
Eritrea	5.0	3.5	7.0	3160	2210	4440	1.9	1.3	2.9	1580	1060	2340
Ethiopia	4.2	3.5	5.2	54600	44800	67300	1.9	1.5	2.5	31800	24500	40900
Gabon	0.9	0.8	1.0	160	139	184	0.5	0.4	0.5	95	83	108
Gambia	2.5	2.0	3.2	607	492	762	0.9	0.6	1.2	288	196	387
Ghana	1.9	1.4	2.5	5780	4200	7630	0.9	0.7	1.2	3630	2640	4880
Guinea	4.0	3.2	5.0	6070	4890	7550	1.4	1.1	1.8	2760	2200	3540
Guinea-Bissau	4.9	3.9	6.1	1120	882	1380	2.4	1.8	3.3	738	543	1010
Kenya	2.8	2.4	3.4	14600	12400	17200	1.5	1.2	1.8	9550	8080	11400
Lesotho	3.6	2.7	4.9	922	693	1250	2.1	1.6	2.8	474	357	627
Liberia	7.2	5.3	9.1	3680	2730	4660	1.7	1.2	2.3	1200	877	1630
Madagascar	5.1	4.1	6.4	15100	12100	18700	3.4	2.6	4.3	14700	11300	18600
Malawi	4.1	3.3	5.1	8910	7170	11000	0.9	0.7	1.2	2540	2000	3170
Mali	7.5	5.6	9.4	16400	12300	20600	2.3	1.7	3.2	8700	6280	11900
Mauritania	5.1	4.2	6.2	2300	1880	2790	1.1	0.8	1.4	628	473	824
Mozambique	3.2	2.6	3.9	10500	8630	12900	0.9	0.8	1.1	4390	3580	5400
Namibia	1.6	1.5	1.8	423	387	462	1.1	1.0	1.2	321	281	360
Niger	12.6	10.0	15.7	30500	24200	38000	3.9	2.9	5.2	16900	12700	22600
Nigeria	7.5	6.0	9.4	161000	127000	201000	3.0	2.3	3.9	104000	79100	134000
Republic of Congo	2.7	2.4	3.1	1360	1190	1540	1.1	1.0	1.3	710	642	796
Rwanda	7.4	5.9	8.9	10500	8430	12700	1.0	0.7	1.3	1890	1420	2410
São Tomé and Príncipe	2.9	2.4	3.5	69	58	82	0.3	0.3	0.4	8	6	10
Senegal	4.9	4.1	5.9	8250	6850	9990	1.5	1.1	1.9	3300	2430	4230
Sierra Leone	5.8	4.3	7.2	4310	3230	5390	2.0	1.6	2.6	2430	1920	3100
Somalia	7.2	5.2	10.2	13000	9390	18700	2.3	1.7	3.1	6680	4950	9060
South Africa	2.4	2.1	2.7	12200	10800	13600	0.8	0.7	1.0	4620	3890	5420
South Sudan	5.5	4.0	7.3	7480	5400	9930	4.5	3.1	6.2	7910	5440	10900
Swaziland	3.5	2.6	4.5	535	406	686	1.7	1.3	2.2	247	185	320
Tanzania	2.2	1.8	2.6	13500	11300	15900	0.5	0.5	0.6	4900	4080	5760
Togo	4.2	3.1	5.4	3450	2600	4510	1.5	1.1	2.0	1620	1200	2120
Uganda	1.9	1.6	2.3	9860	8070	11800	0.8	0.6	0.9	5260	4320	6440
Zambia	5.2	4.2	6.4	9910	7890	12100	1.3	1.0	1.6	3710	2990	4500
Zimbabwe	1.2	0.9	1.5	2130	1710	2660	1.0	0.8	1.3	2220	1750	2810

Table 2: Diarrhoea Incidence by Country

Country	2000 incidence						2017 incidence					
	mean rate (per 10,000)	lower rate (per 10,000)	upper rate (per 10,000)	mean count	lower count	upper count	mean rate (per 10,000)	lower rate (per 10,000)	upper rate (per 10,000)	mean count	lower count	upper count
**Central Europe, Eastern Europe, and Central Asia**												
Kyrgyzstan	1234.7	860.1	1710.5	6.73e+05	4.69e+05	9.32e+05	786.0	596.1	1054.6	6.09e+05	462000	8.17e+05
Mongolia	1228.2	1104.4	1365.6	3.09e+05	2.78e+05	3.44e+05	1384.2	1235.8	1554.1	5.30e+05	473000	5.95e+05
Tajikistan	2063.1	1504.0	2808.7	1.87e+06	1.36e+06	2.54e+06	1609.3	1404.9	1838.3	2.01e+06	1750000	2.29e+06
Turkmenistan	1464.2	1133.5	1853.5	6.48e+05	5.02e+05	8.21e+05	615.0	456.9	846.4	3.39e+05	252000	4.67e+05
Uzbekistan	726.1	563.1	928.2	2.19e+06	1.70e+06	2.79e+06	698.2	489.0	1000.9	2.40e+06	1680000	3.44e+06
**Latin America and Caribbean**												
Belize	784.6	577.7	1099.9	2.51e+04	1.85e+04	3.52e+04	922.8	713.1	1193.2	3.49e+04	27000	4.52e+04
Bolivia	3292.9	2825.8	3744.3	3.99e+06	3.43e+06	4.54e+06	2174.1	1832.5	2507.3	3.15e+06	2660000	3.64e+06
Brazil	2036.7	1731.1	2388.7	3.33e+07	2.83e+07	3.91e+07	1258.5	1063.9	1472.2	1.93e+07	16300000	2.26e+07
Colombia	2546.9	2122.3	3033.4	1.20e+07	1.00e+07	1.43e+07	2129.4	1692.9	2614.4	9.00e+06	7160000	1.11e+07
Costa Rica	1858.3	1292.0	2592.2	7.38e+05	5.13e+05	1.03e+06	2291.4	1605.5	3309.6	8.04e+05	564000	1.16e+06
Dominican Republic	2079.5	1816.1	2377.4	2.33e+06	2.03e+06	2.66e+06	2234.0	1815.6	2676.9	2.23e+06	1810000	2.67e+06
Ecuador	3104.6	2574.9	3746.8	4.92e+06	4.08e+06	5.94e+06	1591.1	1330.7	1917.8	2.48e+06	2070000	2.99e+06
El Salvador	2956.5	2257.4	3846.9	2.22e+06	1.70e+06	2.90e+06	2520.1	1921.7	3255.3	1.37e+06	1040000	1.77e+06
Guatemala	3514.3	2577.9	4606.5	6.29e+06	4.61e+06	8.24e+06	3583.6	2886.1	4342.7	7.12e+06	5730000	8.63e+06
Guyana	1262.9	993.2	1564.5	1.32e+05	1.04e+05	1.63e+05	1181.5	929.9	1453.2	8.66e+04	68200	1.06e+05
Haiti	3632.8	3298.7	4016.6	4.30e+06	3.90e+06	4.75e+06	2460.9	2253.0	2651.9	3.65e+06	3340000	3.93e+06
Honduras	3452.1	2702.2	4313.7	3.31e+06	2.59e+06	4.13e+06	3238.4	2498.9	4370.1	3.80e+06	2930000	5.13e+06
Jamaica	579.4	438.2	745.8	1.62e+05	1.23e+05	2.09e+05	833.5	558.2	1177.9	1.58e+05	106000	2.23e+05
Mexico	2233.9	1848.9	2633.2	2.79e+07	2.31e+07	3.29e+07	1551.2	1212.8	1889.6	1.87e+07	14600000	2.28e+07
Nicaragua	2928.2	2448.4	3504.4	1.98e+06	1.66e+06	2.37e+06	2724.9	2038.6	3520.9	1.85e+06	1390000	2.40e+06
Panama	3244.5	2273.6	4388.1	1.03e+06	7.20e+05	1.39e+06	2975.9	2178.9	3888.7	1.08e+06	787000	1.41e+06
Paraguay	1885.1	1493.1	2382.7	1.22e+06	9.69e+05	1.55e+06	2771.8	2279.1	3444.7	2.06e+06	1690000	2.56e+06
Peru	2712.4	2246.3	3129.3	8.28e+06	6.86e+06	9.55e+06	2108.0	1819.9	2403.5	7.79e+06	6720000	8.88e+06
Suriname	1277.2	992.9	1655.2	5.76e+04	4.48e+04	7.47e+04	906.2	725.0	1106.8	4.34e+04	34800	5.31e+04
**North Africa and Middle East**												
Afghanistan	3516.8	2585.4	4549.7	1.40e+07	1.03e+07	1.81e+07	3987.1	2834.8	5345.6	2.35e+07	16700000	3.15e+07
Algeria	2015.6	1654.3	2347.2	7.56e+06	6.21e+06	8.80e+06	1884.9	1567.7	2227.7	8.44e+06	7020000	9.97e+06
Egypt	2704.3	2211.6	3230.9	2.18e+07	1.78e+07	2.61e+07	2571.3	2059.4	3289.6	3.03e+07	24300000	3.88e+07
Iraq	1741.7	1310.4	2298.9	7.58e+06	5.70e+06	1.00e+07	1530.4	1088.6	2106.3	9.08e+06	6460000	1.25e+07
Jordan	1509.3	930.0	2430.9	1.07e+06	6.57e+05	1.72e+06	1843.9	1176.9	2853.4	2.15e+06	1370000	3.33e+06
Morocco	2215.9	1862.0	2679.4	7.70e+06	6.47e+06	9.32e+06	1976.9	1621.6	2291.5	6.06e+06	4970000	7.02e+06
Sudan	3676.8	3013.1	4504.1	1.77e+07	1.45e+07	2.16e+07	4154.0	3179.9	5365.3	2.52e+07	19300000	3.25e+07
Syria	1360.3	913.5	1986.4	3.06e+06	2.05e+06	4.47e+06	1532.1	996.6	2421.2	2.20e+06	1430000	3.47e+06
Tunisia	1571.7	1318.8	1841.0	1.53e+06	1.28e+06	1.79e+06	1778.5	1442.1	2204.3	1.62e+06	1310000	2.01e+06
Yemen	4910.1	3465.3	6817.7	1.71e+07	1.21e+07	2.37e+07	4749.3	3938.3	5711.5	2.28e+07	18900000	2.74e+07
**South Asia**												
Bangladesh	1228.9	888.0	1711.0	2.21e+07	1.60e+07	3.08e+07	850.5	603.9	1250.2	1.24e+07	8810000	1.82e+07
Bhutan	1256.1	771.5	1935.0	9.06e+04	5.57e+04	1.40e+05	1312.6	856.2	1999.7	1.17e+05	76600	1.79e+05
India	1166.2	1019.8	1332.6	1.53e+08	1.34e+08	1.75e+08	872.6	762.4	986.2	1.13e+08	98600000	1.28e+08
Nepal	1774.9	1313.8	2430.0	6.25e+06	4.63e+06	8.56e+06	1076.2	779.7	1471.0	3.29e+06	2390000	4.50e+06
Pakistan	1422.7	1152.1	1781.9	3.32e+07	2.69e+07	4.16e+07	1918.2	1514.0	2395.1	5.24e+07	41400000	6.54e+07
**Southeast Asia, East Asia, and Oceania**												
Cambodia	2422.5	1657.4	3402.5	4.32e+06	2.95e+06	6.06e+06	1932.4	1243.6	2886.9	3.46e+06	2230000	5.17e+06
Indonesia	1637.4	1497.1	1784.2	3.58e+07	3.27e+07	3.90e+07	2269.6	2013.4	2530.3	4.77e+07	42300000	5.31e+07
Laos	1382.0	859.7	2215.7	1.09e+06	6.78e+05	1.75e+06	1771.7	1120.2	2708.7	1.43e+06	902000	2.18e+06
Myanmar	2210.8	1347.9	3282.9	1.15e+07	7.00e+06	1.71e+07	1794.1	1116.5	2674.1	7.91e+06	4920000	1.18e+07
Papua New Guinea	3066.6	2662.6	3601.4	2.69e+06	2.33e+06	3.16e+06	3002.4	2631.0	3446.4	4.07e+06	3570000	4.67e+06
Philippines	1502.2	1327.6	1674.4	1.64e+07	1.45e+07	1.83e+07	1359.2	1203.1	1535.3	1.62e+07	14400000	1.83e+07
Sri Lanka	855.8	567.0	1314.5	1.49e+06	9.89e+05	2.29e+06	1261.0	794.7	1822.6	2.04e+06	1280000	2.95e+06
Thailand	1053.3	685.4	1618.1	5.27e+06	3.43e+06	8.10e+06	1111.3	688.5	1619.1	3.76e+06	2330000	5.48e+06
Timor-Leste	1869.8	1521.8	2336.5	3.02e+05	2.46e+05	3.78e+05	2004.9	1681.3	2380.2	3.49e+05	293000	4.14e+05
Vietnam	1400.0	848.7	2094.2	1.09e+07	6.62e+06	1.63e+07	1489.2	904.2	2250.3	1.15e+07	7000000	1.74e+07
**Sub-Saharan Africa**												
Angola	4418.1	3690.2	5126.2	1.25e+07	1.05e+07	1.45e+07	2919.0	2569.5	3294.0	1.45e+07	12800000	1.63e+07
Benin	2545.8	1949.7	3221.1	3.25e+06	2.49e+06	4.12e+06	1459.1	1114.8	1844.5	2.78e+06	2130000	3.52e+06
Botswana	1708.2	1585.0	1867.7	3.71e+05	3.44e+05	4.05e+05	1432.2	1275.7	1601.0	3.43e+05	306000	3.84e+05

Table 2: Diarrhoea Incidence by Country *(continued)*

Country	2000 incidence						2017 incidence					
	mean rate (per 10,000)	lower rate (per 10,000)	upper rate (per 10,000)	mean count	lower count	upper count	mean rate (per 10,000)	lower rate (per 10,000)	upper rate (per 10,000)	mean count	lower count	upper count
Burkina Faso	3753.0	2743.7	4875.7	8.81e+06	6.44e+06	1.14e+07	2404.1	1764.1	3332.6	8.98e+06	6590000	1.24e+07
Burundi	3022.8	2386.2	3776.3	3.21e+06	2.53e+06	4.01e+06	2154.1	1628.5	2727.5	3.94e+06	2980000	4.99e+06
Cameroon	3371.6	2782.9	3986.7	8.76e+06	7.23e+06	1.04e+07	3079.0	2266.2	4201.7	1.27e+07	9320000	1.73e+07
Central African Republic	4060.7	3701.0	4456.7	2.47e+06	2.25e+06	2.71e+06	3362.8	3023.4	3725.2	2.04e+06	1830000	2.26e+06
Chad	4207.5	3476.0	5026.2	7.31e+06	6.04e+06	8.73e+06	4048.2	2904.0	5442.4	1.24e+07	8900000	1.67e+07
Comoros	2903.7	2099.4	3943.3	2.59e+05	1.87e+05	3.52e+05	2231.0	1602.5	2944.1	1.90e+05	137000	2.51e+05
Côte d'Ivoire	3181.1	2477.2	4037.7	9.48e+06	7.38e+06	1.20e+07	2897.3	1960.1	3955.5	1.12e+07	7590000	1.53e+07
Democratic Republic of the Congo	3403.1	3183.6	3662.6	3.09e+07	2.89e+07	3.32e+07	2958.0	2743.2	3178.3	3.90e+07	36100000	4.19e+07
Djibouti	1856.1	1195.0	2735.2	1.96e+05	1.26e+05	2.88e+05	1448.2	889.5	2366.0	2.19e+05	134000	3.57e+05
Equatorial Guinea	3841.5	3336.9	4310.9	4.88e+05	4.24e+05	5.48e+05	2547.1	2147.1	3033.6	4.83e+05	407000	5.76e+05
Eritrea	2806.0	1957.5	3935.8	1.77e+06	1.23e+06	2.48e+06	2096.0	1402.5	3097.4	1.72e+06	1150000	2.54e+06
Ethiopia	3200.4	2637.1	3926.6	4.12e+07	3.40e+07	5.06e+07	1984.1	1523.5	2562.7	3.30e+07	25300000	4.26e+07
Gabon	2450.3	2137.0	2815.7	4.44e+05	3.88e+05	5.11e+05	2942.0	2576.5	3347.2	5.86e+05	513000	6.66e+05
Gambia	3169.8	2570.5	3979.3	7.61e+05	6.17e+05	9.55e+05	2583.3	1757.9	3472.2	8.24e+05	560000	1.11e+06
Ghana	2469.9	1795.4	3258.9	7.42e+06	5.39e+06	9.79e+06	2197.2	1599.6	2954.8	8.92e+06	6490000	1.20e+07
Guinea	3022.4	2434.6	3762.1	4.56e+06	3.67e+06	5.68e+06	2538.5	2018.5	3252.8	4.99e+06	3970000	6.40e+06
Guinea-Bissau	3163.7	2497.5	3919.0	7.23e+05	5.71e+05	8.96e+05	2024.0	1488.5	2773.2	6.20e+05	456000	8.49e+05
Kenya	2581.2	2163.0	3063.8	1.32e+07	1.11e+07	1.57e+07	2171.8	1835.5	2622.3	1.41e+07	12000000	1.71e+07
Lesotho	2452.6	1842.6	3316.8	6.20e+05	4.66e+05	8.38e+05	1831.9	1380.2	2422.8	4.14e+05	312000	5.48e+05
Liberia	2950.0	2192.4	3739.5	1.52e+06	1.13e+06	1.92e+06	3563.3	2613.3	4868.8	2.52e+06	1850000	3.44e+06
Madagascar	2420.5	1947.6	3012.9	7.10e+06	5.71e+06	8.84e+06	3039.2	2349.2	3861.6	1.32e+07	10200000	1.68e+07
Malawi	2801.2	2256.4	3463.3	6.05e+06	4.87e+06	7.48e+06	3420.5	2699.0	4272.6	9.37e+06	7390000	1.17e+07
Mali	3487.1	2616.9	4358.2	7.62e+06	5.72e+06	9.52e+06	2019.5	1456.8	2764.8	7.54e+06	5440000	1.03e+07
Mauritania	3094.2	2536.0	3762.1	1.38e+06	1.13e+06	1.68e+06	2161.1	1628.1	2837.0	1.24e+06	931000	1.62e+06
Mozambique	2442.9	2000.9	2997.3	8.05e+06	6.60e+06	9.88e+06	1640.3	1336.5	2016.9	7.71e+06	6280000	9.48e+06
Namibia	1893.5	1731.8	2065.3	4.89e+05	4.47e+05	5.34e+05	2227.8	1954.4	2498.3	6.45e+05	566000	7.23e+05
Niger	5059.2	4011.5	6313.0	1.23e+07	9.74e+06	1.53e+07	2462.2	1853.0	3288.2	1.08e+07	8090000	1.44e+07
Nigeria	2865.1	2263.3	3569.5	6.12e+07	4.84e+07	7.63e+07	1875.2	1422.8	2407.1	6.46e+07	49000000	8.29e+07
Republic of Congo	2480.2	2166.7	2810.4	1.24e+06	1.09e+06	1.41e+06	3021.6	2733.2	3384.9	1.91e+06	1730000	2.14e+06
Rwanda	3051.1	2450.7	3680.8	4.33e+06	3.48e+06	5.22e+06	1997.8	1498.3	2548.0	3.82e+06	2870000	4.88e+06
São Tomé and Príncipe	2645.8	2199.1	3143.4	6.24e+04	5.19e+04	7.41e+04	2317.0	1874.1	2841.0	5.75e+04	46500	7.05e+04
Senegal	3269.0	2713.9	3957.6	5.50e+06	4.57e+06	6.66e+06	3003.1	2214.8	3848.1	6.81e+06	5020000	8.72e+06
Sierra Leone	3084.9	2309.9	3854.2	2.31e+06	1.73e+06	2.89e+06	1975.2	1557.6	2516.1	2.36e+06	1860000	3.01e+06
Somalia	2677.1	1927.9	3832.8	4.88e+06	3.51e+06	6.98e+06	2074.2	1535.5	2813.8	6.15e+06	4550000	8.35e+06
South Africa	1377.3	1223.7	1536.7	6.97e+06	6.19e+06	7.78e+06	377.0	317.2	442.2	2.05e+06	1720000	2.40e+06
South Sudan	3647.3	2636.3	4844.0	4.98e+06	3.60e+06	6.62e+06	3036.1	2088.4	4184.2	5.31e+06	3650000	7.31e+06
Swaziland	2647.5	2011.7	3395.6	4.06e+05	3.09e+05	5.21e+05	2214.3	1658.6	2864.9	3.21e+05	241000	4.16e+05
Tanzania	1985.0	1658.4	2324.6	1.23e+07	1.02e+07	1.43e+07	1657.4	1378.0	1946.7	1.49e+07	12400000	1.75e+07
Togo	3622.0	2726.6	4735.5	3.00e+06	2.26e+06	3.92e+06	2797.7	2070.5	3642.8	2.99e+06	2210000	3.90e+06
Uganda	2987.6	2445.1	3573.8	1.51e+07	1.24e+07	1.81e+07	2183.4	1792.1	2672.9	1.52e+07	12400000	1.86e+07
Zambia	3150.1	2508.4	3855.9	5.95e+06	4.74e+06	7.28e+06	2436.8	1963.3	2958.9	6.99e+06	5630000	8.49e+06
Zimbabwe	1994.0	1600.4	2489.7	3.66e+06	2.93e+06	4.56e+06	2577.0	2037.6	3264.7	5.68e+06	4490000	7.19e+06

Table 3: Diarrhoea Mortality by Unit

		2000 mortality						2017 mortality					
Country	Unit	mean rate (per 10,000)	lower rate (per 10,000)	upper rate (per 10,000)	mean count	lower count	upper count	mean rate (per 10,000)	lower rate (per 10,000)	upper rate (per 10,000)	mean count	lower count	upper count
**Central Europe, Eastern Europe, and Central Asia**													
Kyrgyzstan	Ak-Suu	0.8	0.5	1.2	5	3	7	0.2	0.1	0.2	1	1	2
Kyrgyzstan	Ak-Talaa	0.8	0.5	1.2	3	2	4	0.1	0.1	0.2	1	0	1
Kyrgyzstan	Aksyi	0.9	0.5	1.3	13	8	20	0.2	0.1	0.2	3	2	5
Kyrgyzstan	Ala-Buka	0.9	0.5	1.4	8	5	12	0.1	0.1	0.2	2	1	2
Kyrgyzstan	Alai	0.8	0.5	1.3	6	4	10	0.2	0.1	0.2	2	1	3
Kyrgyzstan	Alamüdün	0.8	0.5	1.3	7	4	11	0.2	0.1	0.2	2	2	4
Kyrgyzstan	Aravan	0.9	0.5	1.4	11	7	18	0.1	0.1	0.2	2	1	3
Kyrgyzstan	At-Bashi	0.8	0.5	1.2	5	3	7	0.2	0.1	0.2	1	1	2
Kyrgyzstan	Bakai-Ata	0.8	0.5	1.3	4	2	6	0.2	0.1	0.3	1	1	2
Kyrgyzstan	Batken	1.1	0.7	1.6	13	8	20	0.2	0.1	0.3	3	2	4
Kyrgyzstan	Bazar-Korgon	0.9	0.5	1.4	13	8	21	0.2	0.1	0.2	3	2	5
Kyrgyzstan	Biškek	0.8	0.4	1.3	40	23	64	0.2	0.1	0.2	16	10	25
Kyrgyzstan	Chatkal	0.9	0.6	1.3	3	2	5	0.1	0.1	0.2	1	0	1
Kyrgyzstan	Chong-Alay	0.9	0.5	1.4	3	2	5	0.2	0.1	0.2	1	0	1
Kyrgyzstan	Chui	0.8	0.5	1.2	8	5	13	0.2	0.1	0.2	2	1	4
Kyrgyzstan	Djety-Oguz	0.8	0.5	1.2	7	4	11	0.2	0.1	0.2	2	1	3
Kyrgyzstan	Jaiyl	0.8	0.5	1.3	4	2	6	0.2	0.1	0.2	1	1	2
Kyrgyzstan	Jumgal	0.8	0.5	1.2	3	2	5	0.1	0.1	0.2	1	0	1
Kyrgyzstan	Kadamjai	0.9	0.5	1.3	24	14	35	0.1	0.1	0.2	5	3	7
Kyrgyzstan	Kara-Buura	0.8	0.5	1.3	3	2	5	0.2	0.1	0.2	1	1	2
Kyrgyzstan	Kara-Kuldja	0.8	0.5	1.2	9	6	14	0.2	0.1	0.3	2	1	4
Kyrgyzstan	Kara-Suu	0.9	0.5	1.3	41	23	63	0.2	0.1	0.2	9	6	14
Kyrgyzstan	Kemin	0.8	0.5	1.2	4	3	6	0.2	0.1	0.2	1	1	2
Kyrgyzstan	Kochkor	0.8	0.4	1.2	5	3	8	0.2	0.1	0.2	1	1	2
Kyrgyzstan	Lailak	1.0	0.6	1.5	17	10	26	0.1	0.1	0.2	3	2	5
Kyrgyzstan	Manas	0.8	0.5	1.3	4	2	6	0.2	0.1	0.2	1	1	2
Kyrgyzstan	Moskovsky	0.8	0.4	1.3	5	3	7	0.2	0.1	0.2	2	1	3
Kyrgyzstan	Naryn	0.8	0.5	1.3	8	5	13	0.2	0.1	0.2	2	1	3
Kyrgyzstan	Nookat	0.9	0.5	1.4	24	14	38	0.2	0.1	0.2	5	3	7
Kyrgyzstan	Nooken	0.9	0.5	1.3	18	11	28	0.2	0.1	0.2	4	2	5
Kyrgyzstan	Osh	0.9	0.5	1.4	20	11	32	0.2	0.1	0.2	5	3	7
Kyrgyzstan	Panfilov	0.8	0.5	1.2	2	1	3	0.2	0.1	0.2	1	0	1
Kyrgyzstan	Sokuluk	0.8	0.5	1.3	8	5	13	0.2	0.1	0.2	3	2	4
Kyrgyzstan	Song-Kol	0.8	0.5	1.3	0	0	0	0.1	0.1	0.2	0	0	0
Kyrgyzstan	Suzak	0.8	0.5	1.3	30	17	46	0.2	0.1	0.2	7	5	12
Kyrgyzstan	Talas	0.8	0.5	1.4	8	5	14	0.2	0.1	0.2	3	2	4
Kyrgyzstan	Togus-Toro	0.8	0.5	1.2	2	1	3	0.2	0.1	0.3	1	0	1
Kyrgyzstan	Toktogul	0.8	0.5	1.4	12	7	19	0.2	0.1	0.2	3	2	4
Kyrgyzstan	Ton	0.8	0.5	1.2	7	5	11	0.2	0.1	0.2	2	1	3
Kyrgyzstan	Tüp	0.8	0.5	1.2	9	5	15	0.2	0.1	0.2	3	2	4
Kyrgyzstan	Uzgen	0.8	0.5	1.2	25	14	36	0.2	0.1	0.2	6	4	9
Kyrgyzstan	Ysyk-Ata	0.8	0.5	1.3	9	5	15	0.2	0.1	0.2	3	2	4
Kyrgyzstan	Ysyk-Köl	0.8	0.5	1.2	4	3	7	0.2	0.1	0.2	1	1	2
Kyrgyzstan	Ysyk-Köl (lake)	0.8	0.5	1.2	2	1	4	0.2	0.1	0.2	1	0	1
Mongolia	Adaatsag	0.2	0.2	0.2	0	0	0	0.1	0.1	0.1	0	0	0
Mongolia	Airag	0.2	0.2	0.2	0	0	0	0.1	0.1	0.1	0	0	0
Mongolia	Airag	0.1	0.1	0.2	0	0	0	0.1	0.1	0.1	0	0	0
Mongolia	Alag-Erdene	0.2	0.1	0.2	0	0	0	0.1	0.1	0.1	0	0	0
Mongolia	Aldarkhaan	0.2	0.2	0.2	1	1	1	0.1	0.1	0.1	0	0	0
Mongolia	Altai	0.2	0.1	0.2	0	0	0	0.1	0.1	0.1	0	0	0
Mongolia	Altai	0.2	0.2	0.3	0	0	0	0.1	0.1	0.1	0	0	0
Mongolia	Altai	0.2	0.2	0.2	0	0	0	0.1	0.1	0.1	0	0	0
Mongolia	Altanbulag	0.2	0.2	0.2	0	0	1	0.1	0.1	0.1	0	0	0
Mongolia	Altanbulag	0.2	0.1	0.2	0	0	0	0.1	0.1	0.1	0	0	0
Mongolia	Altanshiree	0.2	0.2	0.2	0	0	0	0.1	0.1	0.1	0	0	0
Mongolia	Altantsögts	0.2	0.1	0.2	0	0	0	0.1	0.1	0.1	0	0	0
Mongolia	Arbulag	0.1	0.1	0.2	0	0	0	0.1	0.1	0.1	0	0	0
Mongolia	Argalant	0.2	0.1	0.2	0	0	0	0.1	0.1	0.1	0	0	0
Mongolia	Arkhust	0.2	0.1	0.2	0	0	0	0.1	0.1	0.1	0	0	0

Country	Unit	2000 mortality						2017 mortality					
		mean rate (per 10,000)	lower rate (per 10,000)	upper rate (per 10,000)	mean count	lower count	upper count	mean rate (per 10,000)	lower rate (per 10,000)	upper rate (per 10,000)	mean count	lower count	upper count
Mongolia	Arvaikheer	0.2	0.1	0.2	1	1	1	0.1	0.1	0.1	0	0	0
Mongolia	Asgat	0.2	0.2	0.2	0	0	0	0.1	0.1	0.1	0	0	0
Mongolia	Asgat	0.1	0.1	0.2	0	0	0	0.1	0.1	0.1	0	0	0
Mongolia	Baatsagaan	0.2	0.2	0.2	0	0	0	0.1	0.1	0.1	0	0	0
Mongolia	Baruun Bayan-Ulaan	0.2	0.2	0.2	0	0	0	0.1	0.1	0.1	0	0	0
Mongolia	Baruunbüren	0.2	0.2	0.2	0	0	0	0.1	0.1	0.1	0	0	0
Mongolia	Baruuntutuun	0.2	0.2	0.3	0	0	0	0.1	0.1	0.1	0	0	0
Mongolia	Bat-Ölzii	0.2	0.2	0.2	0	0	0	0.1	0.1	0.1	0	0	0
Mongolia	Batnorov	0.2	0.2	0.2	0	0	0	0.1	0.1	0.1	0	0	0
Mongolia	Batshireet	0.2	0.2	0.2	0	0	0	0.1	0.1	0.1	0	0	0
Mongolia	Batsümber	0.2	0.2	0.2	0	0	0	0.1	0.1	0.1	0	0	0
Mongolia	Battsengel	0.2	0.2	0.2	0	0	0	0.1	0.1	0.1	0	0	0
Mongolia	Bayan	0.2	0.2	0.2	0	0	0	0.1	0.1	0.1	0	0	0
Mongolia	Bayan-Adarga	0.2	0.2	0.3	0	0	0	0.1	0.1	0.1	0	0	0
Mongolia	Bayan-Agt	0.2	0.2	0.2	0	0	0	0.1	0.1	0.1	0	0	0
Mongolia	Bayan-Öndör	0.2	0.2	0.2	1	1	1	0.1	0.0	0.1	1	0	1
Mongolia	Bayan-Öndör	0.2	0.2	0.2	0	0	0	0.1	0.1	0.1	0	0	0
Mongolia	Bayan-Öndör	0.2	0.2	0.2	0	0	0	0.1	0.1	0.1	0	0	0
Mongolia	Bayan-Önjüül	0.2	0.1	0.2	0	0	0	0.1	0.1	0.1	0	0	0
Mongolia	Bayan-Ovoo	0.2	0.2	0.2	0	0	0	0.1	0.1	0.1	0	0	0
Mongolia	Bayan-Ovoo	0.2	0.2	0.2	0	0	0	0.1	0.1	0.1	0	0	0
Mongolia	Bayan-Ovoo	0.2	0.1	0.2	0	0	0	0.1	0.1	0.1	0	0	0
Mongolia	Bayan-Uul	0.2	0.2	0.2	0	0	0	0.1	0.1	0.1	0	0	0
Mongolia	Bayan-Uul	0.2	0.2	0.2	0	0	0	0.1	0.1	0.1	0	0	0
Mongolia	Bayanbulag	0.2	0.2	0.2	0	0	0	0.1	0.1	0.1	0	0	0
Mongolia	Bayanchandmani	0.2	0.2	0.2	0	0	0	0.1	0.1	0.1	0	0	0
Mongolia	Bayandalai	0.2	0.2	0.2	0	0	0	0.1	0.1	0.1	0	0	0
Mongolia	Bayandelger	0.1	0.1	0.2	0	0	0	0.1	0.1	0.1	0	0	0
Mongolia	Bayandelger	0.2	0.1	0.2	0	0	0	0.1	0.1	0.1	0	0	0
Mongolia	Bayandun	0.2	0.2	0.2	0	0	0	0.1	0.1	0.1	0	0	0
Mongolia	Bayangol	0.2	0.2	0.2	0	0	0	0.1	0.1	0.1	0	0	0
Mongolia	Bayangol	0.2	0.2	0.2	0	0	0	0.1	0.1	0.1	0	0	0
Mongolia	Bayangovi	0.2	0.2	0.2	0	0	0	0.1	0.1	0.1	0	0	0
Mongolia	Bayanjargalan	0.2	0.2	0.2	0	0	0	0.1	0.1	0.1	0	0	0
Mongolia	Bayanjargalan	0.2	0.1	0.2	0	0	0	0.1	0.1	0.1	0	0	0
Mongolia	Bayankhairkhan	0.2	0.2	0.2	0	0	0	0.1	0.1	0.1	0	0	0
Mongolia	Bayankhangai	0.2	0.1	0.2	0	0	0	0.1	0.1	0.1	0	0	0
Mongolia	Bayankhongor	0.2	0.2	0.2	1	1	1	0.0	0.0	0.1	0	0	0
Mongolia	Bayankhutag	0.2	0.2	0.2	0	0	0	0.1	0.1	0.1	0	0	0
Mongolia	Bayanlig	0.2	0.2	0.2	0	0	0	0.1	0.1	0.1	0	0	0
Mongolia	Bayanmönkh	0.2	0.2	0.3	0	0	0	0.1	0.1	0.1	0	0	0
Mongolia	Bayannuur	0.2	0.1	0.2	0	0	0	0.1	0.1	0.1	0	0	0
Mongolia	Bayannuur	0.2	0.1	0.2	0	0	0	0.1	0.1	0.1	0	0	0
Mongolia	Bayantal	0.2	0.2	0.2	0	0	0	0.1	0.1	0.1	0	0	0
Mongolia	Bayantes	0.2	0.2	0.2	0	0	0	0.1	0.1	0.1	0	0	0
Mongolia	Bayantsagaan	0.2	0.1	0.2	0	0	0	0.1	0.1	0.1	0	0	0
Mongolia	Bayantsagaan	0.2	0.2	0.2	0	0	0	0.1	0.1	0.1	0	0	0
Mongolia	Bayantsogt	0.2	0.1	0.2	0	0	0	0.1	0.1	0.1	0	0	0
Mongolia	Bayantümen	0.2	0.2	0.2	0	0	0	0.1	0.0	0.1	0	0	0
Mongolia	Bayanzürkh	0.2	0.1	0.2	0	0	0	0.1	0.1	0.1	0	0	0
Mongolia	Biger	0.2	0.2	0.2	0	0	0	0.1	0.1	0.1	0	0	0
Mongolia	Binder	0.2	0.2	0.3	0	0	0	0.1	0.1	0.1	0	0	0
Mongolia	Bogd	0.2	0.2	0.2	0	0	0	0.1	0.1	0.1	0	0	0
Mongolia	Bogd	0.2	0.2	0.2	0	0	0	0.1	0.1	0.1	0	0	0
Mongolia	Bökhmörön	0.2	0.2	0.3	0	0	0	0.1	0.1	0.1	0	0	0
Mongolia	Bömbögör	0.2	0.2	0.2	0	0	0	0.1	0.1	0.1	0	0	0
Mongolia	Bornuur	0.2	0.2	0.2	0	0	0	0.1	0.1	0.1	0	0	0
Mongolia	Bugat	0.2	0.2	0.2	0	0	0	0.1	0.1	0.1	0	0	0
Mongolia	Bugat	0.2	0.1	0.2	0	0	0	0.1	0.1	0.1	0	0	0

Table 3: Diarrhoea Mortality by Unit *(continued)*

Country	Unit	2000 mortality mean rate (per 10,000)	2000 mortality lower rate (per 10,000)	2000 mortality upper rate (per 10,000)	2000 mortality mean count	2000 mortality lower count	2000 mortality upper count	2017 mortality mean rate (per 10,000)	2017 mortality lower rate (per 10,000)	2017 mortality upper rate (per 10,000)	2017 mortality mean count	2017 mortality lower count	2017 mortality upper count
Mongolia	Bugat	0.2	0.2	0.2	0	0	0	0.1	0.1	0.1	0	0	0
Mongolia	Bulgan	0.2	0.2	0.2	0	0	0	0.1	0.1	0.1	0	0	0
Mongolia	Bulgan	0.2	0.2	0.3	0	0	0	0.1	0.1	0.1	0	0	0
Mongolia	Bulgan	0.2	0.2	0.2	0	0	0	0.1	0.1	0.1	0	0	0
Mongolia	Bulgan	0.2	0.1	0.2	0	0	0	0.1	0.1	0.1	0	0	0
Mongolia	Bulgan	0.2	0.1	0.2	0	0	0	0.1	0.1	0.1	0	0	0
Mongolia	Bürd	0.2	0.2	0.2	0	0	0	0.1	0.1	0.1	0	0	0
Mongolia	Büregkhangai	0.2	0.2	0.2	0	0	0	0.1	0.1	0.1	0	0	0
Mongolia	Büren	0.2	0.1	0.2	0	0	0	0.1	0.1	0.1	0	0	0
Mongolia	Bürentogtokh	0.1	0.1	0.2	0	0	0	0.1	0.1	0.1	0	0	0
Mongolia	Buutsagaan	0.2	0.2	0.2	0	0	0	0.1	0.1	0.1	0	0	0
Mongolia	Buyant	0.2	0.2	0.2	0	0	0	0.1	0.0	0.1	0	0	0
Mongolia	Buyant	0.2	0.1	0.2	0	0	0	0.1	0.1	0.1	0	0	0
Mongolia	Chandmani	0.2	0.2	0.3	0	0	0	0.1	0.1	0.1	0	0	0
Mongolia	Chandmani	0.2	0.2	0.2	0	0	0	0.1	0.1	0.1	0	0	0
Mongolia	Chandmani-Öndör	0.2	0.1	0.2	0	0	0	0.1	0.1	0.1	0	0	0
Mongolia	Choibalsan	0.2	0.2	0.2	0	0	0	0.1	0.1	0.1	0	0	0
Mongolia	Chuluunkhoroot	0.2	0.2	0.3	0	0	0	0.1	0.1	0.1	0	0	0
Mongolia	Chuluut	0.2	0.2	0.2	0	0	0	0.1	0.1	0.1	0	0	0
Mongolia	Dadal	0.2	0.2	0.3	0	0	0	0.1	0.1	0.1	0	0	0
Mongolia	Dalanjargalan	0.2	0.2	0.2	0	0	0	0.1	0.1	0.1	0	0	0
Mongolia	Dalanzadgad	0.2	0.2	0.2	0	0	0	0.1	0.1	0.1	0	0	0
Mongolia	Dariganga	0.1	0.1	0.1	0	0	0	0.1	0.1	0.1	0	0	0
Mongolia	Darkhan	0.2	0.1	0.2	1	1	1	0.0	0.0	0.1	0	0	0
Mongolia	Darkhan	0.2	0.2	0.2	0	0	0	0.1	0.1	0.1	0	0	0
Mongolia	Darvi	0.2	0.2	0.3	0	0	0	0.1	0.1	0.1	0	0	0
Mongolia	Darvi	0.2	0.2	0.2	0	0	0	0.1	0.1	0.1	0	0	0
Mongolia	Dashbalbar	0.2	0.2	0.2	0	0	0	0.1	0.1	0.1	0	0	0
Mongolia	Dashinchilen	0.2	0.2	0.2	0	0	0	0.1	0.1	0.1	0	0	0
Mongolia	Davst	0.2	0.2	0.3	0	0	0	0.1	0.1	0.1	0	0	0
Mongolia	Delger	0.2	0.2	0.2	0	0	0	0.1	0.1	0.1	0	0	0
Mongolia	Delgerekh	0.2	0.1	0.2	0	0	0	0.1	0.1	0.1	0	0	0
Mongolia	Delgerkhaan	0.2	0.2	0.2	0	0	0	0.1	0.1	0.1	0	0	0
Mongolia	Delgerkhaan	0.2	0.1	0.2	0	0	0	0.1	0.1	0.1	0	0	0
Mongolia	Delgerkhangai	0.2	0.2	0.2	0	0	0	0.1	0.1	0.1	0	0	0
Mongolia	Delgertsogt	0.2	0.2	0.2	0	0	0	0.1	0.1	0.1	0	0	0
Mongolia	Delüün	0.2	0.1	0.2	0	0	0	0.1	0.1	0.1	0	0	0
Mongolia	Deren	0.2	0.2	0.2	0	0	0	0.1	0.1	0.1	0	0	0
Mongolia	Dörgön	0.2	0.2	0.3	0	0	0	0.1	0.1	0.1	0	0	0
Mongolia	Dörvöljin	0.2	0.2	0.2	0	0	0	0.1	0.1	0.1	0	0	0
Mongolia	Duut	0.2	0.2	0.2	0	0	0	0.1	0.1	0.1	0	0	0
Mongolia	Erdene	0.2	0.2	0.2	0	0	0	0.1	0.1	0.1	0	0	0
Mongolia	Erdene	0.2	0.2	0.2	0	0	0	0.1	0.1	0.1	0	0	0
Mongolia	Erdene	0.2	0.2	0.2	0	0	0	0.1	0.1	0.1	0	0	0
Mongolia	Erdenebulgan	0.2	0.2	0.2	1	0	1	0.0	0.0	0.1	0	0	0
Mongolia	Erdenebulgan	0.2	0.1	0.2	0	0	0	0.1	0.1	0.1	0	0	0
Mongolia	Erdenebüren	0.2	0.2	0.2	0	0	0	0.1	0.1	0.1	0	0	0
Mongolia	Erdenedalai	0.2	0.2	0.3	0	0	0	0.1	0.1	0.1	0	0	0
Mongolia	Erdenekhairkhan	0.2	0.2	0.2	0	0	0	0.1	0.1	0.1	0	0	0
Mongolia	Erdenemandal	0.2	0.2	0.2	0	0	0	0.1	0.1	0.1	0	0	0
Mongolia	Erdenesant	0.2	0.1	0.2	0	0	0	0.1	0.1	0.1	0	0	0
Mongolia	Erdenetsagaan	0.1	0.1	0.2	0	0	0	0.1	0.1	0.1	0	0	0
Mongolia	Erdenetsogt	0.2	0.2	0.2	0	0	0	0.1	0.1	0.1	0	0	0
Mongolia	Galshar	0.2	0.1	0.2	0	0	0	0.1	0.1	0.1	0	0	0
Mongolia	Galt	0.1	0.1	0.2	0	0	0	0.1	0.1	0.1	0	0	0
Mongolia	Galuut	0.2	0.2	0.2	0	0	0	0.1	0.1	0.1	0	0	0
Mongolia	Govi-Ugtaal	0.2	0.2	0.2	0	0	0	0.1	0.1	0.1	0	0	0
Mongolia	Guchin-Us	0.2	0.2	0.2	0	0	0	0.1	0.1	0.1	0	0	0
Mongolia	Gurvan tes	0.2	0.2	0.2	0	0	0	0.1	0.1	0.1	0	0	0

Table 3: Diarrhoea Mortality by Unit *(continued)*

Country	Unit	2000 mortality mean rate (per 10,000)	2000 mortality lower rate (per 10,000)	2000 mortality upper rate (per 10,000)	2000 mortality mean count	2000 mortality lower count	2000 mortality upper count	2017 mortality mean rate (per 10,000)	2017 mortality lower rate (per 10,000)	2017 mortality upper rate (per 10,000)	2017 mortality mean count	2017 mortality lower count	2017 mortality upper count
Mongolia	Gurvanbulag	0.2	0.2	0.2	0	0	0	0.1	0.1	0.1	0	0	0
Mongolia	Gurvanbulag	0.2	0.2	0.2	0	0	0	0.1	0.1	0.1	0	0	0
Mongolia	Gurvansaikhan	0.2	0.2	0.3	0	0	0	0.1	0.1	0.1	0	0	0
Mongolia	Gurvanzagal	0.2	0.2	0.2	0	0	0	0.1	0.1	0.1	0	0	0
Mongolia	Ider	0.2	0.2	0.2	0	0	0	0.1	0.1	0.1	0	0	0
Mongolia	Ikh-Tamir	0.2	0.2	0.2	0	0	0	0.1	0.1	0.1	0	0	0
Mongolia	Ikh-Uul	0.2	0.2	0.2	0	0	0	0.1	0.1	0.1	0	0	0
Mongolia	Ikh-Uul	0.1	0.1	0.2	0	0	0	0.1	0.1	0.1	0	0	0
Mongolia	Ikhkhet	0.2	0.1	0.2	0	0	0	0.1	0.1	0.1	0	0	0
Mongolia	Jargalan	0.2	0.2	0.2	0	0	0	0.1	0.1	0.1	0	0	0
Mongolia	Jargalant	0.2	0.2	0.2	0	0	0	0.1	0.1	0.1	0	0	0
Mongolia	Jargalant	0.2	0.2	0.2	0	0	0	0.1	0.1	0.1	0	0	0
Mongolia	Jargalant	0.2	0.2	0.2	0	0	0	0.1	0.1	0.1	0	0	0
Mongolia	Jargalant	0.2	0.2	0.2	0	0	0	0.1	0.1	0.1	0	0	0
Mongolia	Jargalant	0.2	0.2	0.3	1	1	1	0.1	0.1	0.1	0	0	0
Mongolia	Jargalant	0.2	0.1	0.2	0	0	0	0.1	0.1	0.1	0	0	0
Mongolia	Jargaltkhaan	0.2	0.2	0.2	0	0	0	0.1	0.1	0.1	0	0	0
Mongolia	Javkhlant	0.2	0.2	0.2	0	0	0	0.1	0.1	0.1	0	0	0
Mongolia	Jinst	0.2	0.2	0.2	0	0	0	0.1	0.1	0.1	0	0	0
Mongolia	Khairkhan	0.2	0.2	0.2	0	0	0	0.1	0.1	0.1	0	0	0
Mongolia	Khairkhandulaan	0.2	0.2	0.2	0	0	0	0.1	0.1	0.1	0	0	0
Mongolia	Khaliun	0.2	0.2	0.2	0	0	0	0.1	0.1	0.1	0	0	0
Mongolia	Khalkhgol	0.2	0.2	0.2	0	0	0	0.1	0.1	0.1	0	0	0
Mongolia	Khalzan	0.1	0.1	0.2	0	0	0	0.1	0.1	0.1	0	0	0
Mongolia	Khan khongor	0.2	0.2	0.2	0	0	0	0.1	0.1	0.1	0	0	0
Mongolia	Khanbogd	0.2	0.2	0.2	0	0	0	0.1	0.1	0.1	0	0	0
Mongolia	Khangai	0.2	0.2	0.2	0	0	0	0.1	0.1	0.1	0	0	0
Mongolia	Khangal	0.2	0.2	0.2	0	0	0	0.1	0.1	0.1	0	0	0
Mongolia	Khankh	0.2	0.2	0.2	0	0	0	0.1	0.1	0.1	0	0	0
Mongolia	Kharkhorin	0.2	0.2	0.2	0	0	0	0.1	0.1	0.1	0	0	0
Mongolia	Khashaat	0.2	0.2	0.2	0	0	0	0.1	0.1	0.1	0	0	0
Mongolia	Khatanbulag	0.2	0.2	0.2	0	0	0	0.1	0.1	0.1	0	0	0
Mongolia	Khatgal	0.2	0.1	0.2	0	0	0	0.1	0.1	0.1	0	0	0
Mongolia	Kherlen	0.2	0.2	0.2	1	1	1	0.0	0.0	0.1	0	0	0
Mongolia	Kherlen	0.2	0.2	0.2	0	0	1	0.1	0.1	0.1	0	0	0
Mongolia	Khishig-Öndör	0.2	0.2	0.2	0	0	0	0.1	0.1	0.1	0	0	0
Mongolia	Khökh morit	0.2	0.2	0.3	0	0	0	0.1	0.1	0.1	0	0	0
Mongolia	Khölönbuir	0.2	0.1	0.2	0	0	0	0.1	0.1	0.1	0	0	0
Mongolia	Khongor	0.2	0.2	0.2	0	0	0	0.0	0.0	0.1	0	0	0
Mongolia	Khotont	0.2	0.2	0.2	0	0	0	0.1	0.1	0.1	0	0	0
Mongolia	Khovd	0.2	0.2	0.2	0	0	0	0.1	0.0	0.1	0	0	0
Mongolia	Khovd	0.2	0.2	0.3	0	0	0	0.1	0.1	0.1	0	0	0
Mongolia	Khövsgöl	0.2	0.2	0.2	0	0	0	0.1	0.1	0.1	0	0	0
Mongolia	Khüder	0.2	0.2	0.2	0	0	0	0.1	0.1	0.1	0	0	0
Mongolia	Khujirt	0.2	0.2	0.2	0	0	0	0.1	0.1	0.1	0	0	0
Mongolia	Khuld	0.2	0.2	0.3	0	0	0	0.1	0.1	0.1	0	0	0
Mongolia	Khüreemaral	0.2	0.2	0.2	0	0	0	0.1	0.1	0.1	0	0	0
Mongolia	Khürmen	0.2	0.2	0.2	0	0	0	0.1	0.1	0.1	0	0	0
Mongolia	Khushaat	0.2	0.2	0.2	0	0	0	0.1	0.1	0.1	0	0	0
Mongolia	Khutag-Öndör	0.2	0.2	0.2	0	0	0	0.1	0.1	0.1	0	0	0
Mongolia	Khyargas	0.2	0.2	0.3	0	0	0	0.1	0.1	0.1	0	0	0
Mongolia	Lün	0.2	0.1	0.2	0	0	0	0.1	0.1	0.1	0	0	0
Mongolia	Luus	0.2	0.2	0.3	0	0	0	0.1	0.1	0.1	0	0	0
Mongolia	Malchin	0.2	0.2	0.3	0	0	0	0.1	0.1	0.1	0	0	0
Mongolia	Mandakh	0.2	0.2	0.2	0	0	0	0.1	0.1	0.1	0	0	0
Mongolia	Mandal	0.2	0.2	0.2	0	0	1	0.1	0.1	0.1	0	0	0
Mongolia	Mandal-Ovoo	0.2	0.2	0.2	0	0	0	0.1	0.1	0.1	0	0	0
Mongolia	Mankhan	0.2	0.2	0.3	0	0	0	0.1	0.1	0.1	0	0	0
Mongolia	Manlai	0.2	0.2	0.2	0	0	0	0.1	0.1	0.1	0	0	0
Mongolia	Matad	0.2	0.2	0.2	0	0	0	0.1	0.1	0.1	0	0	0

Table 3: Diarrhoea Mortality by Unit *(continued)*

		2000 mortality						2017 mortality					
Country	Unit	mean rate (per 10,000)	lower rate (per 10,000)	upper rate (per 10,000)	mean count	lower count	upper count	mean rate (per 10,000)	lower rate (per 10,000)	upper rate (per 10,000)	mean count	lower count	upper count
Mongolia	Mogod	0.2	0.2	0.2	0	0	0	0.1	0.1	0.1	0	0	0
Mongolia	Möngönmorit	0.2	0.2	0.2	0	0	0	0.1	0.1	0.1	0	0	0
Mongolia	Mönkhkhairkhan	0.2	0.2	0.2	0	0	0	0.1	0.1	0.1	0	0	0
Mongolia	Mörön	0.1	0.1	0.2	1	0	1	0.1	0.1	0.1	0	0	0
Mongolia	Mörön	0.2	0.2	0.2	0	0	0	0.1	0.1	0.1	0	0	0
Mongolia	Möst	0.2	0.2	0.3	0	0	0	0.1	0.1	0.1	0	0	0
Mongolia	Myangad	0.2	0.2	0.3	0	0	0	0.1	0.1	0.1	0	0	0
Mongolia	Naran	0.1	0.1	0.2	0	0	0	0.1	0.1	0.1	0	0	0
Mongolia	Naranbulag	0.2	0.2	0.3	0	0	0	0.1	0.1	0.1	0	0	0
Mongolia	Nariinteel	0.2	0.2	0.2	0	0	0	0.1	0.1	0.1	0	0	0
Mongolia	Nogoonnuur	0.2	0.2	0.2	0	0	0	0.1	0.1	0.1	0	0	0
Mongolia	Nomgon	0.2	0.2	0.2	0	0	0	0.1	0.1	0.1	0	0	0
Mongolia	Nömrög	0.2	0.2	0.2	0	0	0	0.1	0.1	0.1	0	0	0
Mongolia	Norovlin	0.2	0.2	0.3	0	0	0	0.1	0.1	0.1	0	0	0
Mongolia	Noyon	0.2	0.2	0.2	0	0	0	0.1	0.1	0.1	0	0	0
Mongolia	Ögii nuur	0.2	0.2	0.2	0	0	0	0.1	0.1	0.1	0	0	0
Mongolia	Ölgii	0.2	0.2	0.3	0	0	0	0.1	0.1	0.1	0	0	0
Mongolia	Ölgii (city)	0.2	0.1	0.2	1	1	1	0.1	0.1	0.1	0	0	0
Mongolia	Ölziit	0.2	0.2	0.3	0	0	0	0.1	0.1	0.1	0	0	0
Mongolia	Ölziit	0.2	0.2	0.2	0	0	0	0.1	0.1	0.1	0	0	0
Mongolia	Ölziit	0.2	0.2	0.2	0	0	0	0.1	0.1	0.1	0	0	0
Mongolia	Ölziit	0.2	0.2	0.2	0	0	0	0.1	0.0	0.1	0	0	0
Mongolia	Ömnödelger	0.2	0.2	0.2	0	0	0	0.1	0.1	0.1	0	0	0
Mongolia	Ömnögovi	0.2	0.2	0.3	0	0	0	0.1	0.1	0.1	0	0	0
Mongolia	Öndör-Ulaan	0.2	0.2	0.2	0	0	0	0.1	0.1	0.1	0	0	0
Mongolia	Öndörkhangai	0.2	0.2	0.3	0	0	0	0.1	0.1	0.1	0	0	0
Mongolia	Öndörshil	0.2	0.2	0.3	0	0	0	0.1	0.1	0.1	0	0	0
Mongolia	Öndörshireet	0.2	0.1	0.2	0	0	0	0.1	0.1	0.1	0	0	0
Mongolia	Ongon	0.1	0.1	0.2	0	0	0	0.1	0.1	0.1	0	0	0
Mongolia	Örgön	0.2	0.2	0.2	0	0	0	0.1	0.1	0.1	0	0	0
Mongolia	Orkhon	0.2	0.2	0.2	0	0	0	0.1	0.1	0.1	0	0	0
Mongolia	Orkhon	0.2	0.2	0.2	0	0	0	0.1	0.1	0.1	0	0	0
Mongolia	Orkhon	0.2	0.2	0.2	0	0	0	0.1	0.1	0.1	0	0	0
Mongolia	Orkhontuul	0.2	0.2	0.2	0	0	0	0.1	0.1	0.1	0	0	0
Mongolia	Otgon	0.2	0.2	0.2	0	0	0	0.1	0.1	0.1	0	0	0
Mongolia	Rashaant	0.2	0.2	0.2	0	0	0	0.1	0.1	0.1	0	0	0
Mongolia	Rashaant	0.1	0.1	0.2	0	0	0	0.1	0.1	0.1	0	0	0
Mongolia	Renchinlkhümbe	0.2	0.2	0.2	0	0	0	0.1	0.1	0.1	0	0	0
Mongolia	Sagil	0.2	0.2	0.2	0	0	0	0.1	0.1	0.1	0	0	0
Mongolia	Sagsai	0.2	0.1	0.2	0	0	0	0.1	0.1	0.1	0	0	0
Mongolia	Saikhan	0.2	0.2	0.2	0	0	0	0.1	0.1	0.1	0	0	0
Mongolia	Saikhan-Ovoo	0.2	0.2	0.2	0	0	0	0.1	0.1	0.1	0	0	0
Mongolia	Saikhandulaan	0.2	0.2	0.2	0	0	0	0.1	0.1	0.1	0	0	0
Mongolia	Sainshand	0.2	0.2	0.2	0	0	0	0.1	0.1	0.1	0	0	0
Mongolia	Saintsagaan	0.2	0.2	0.3	0	0	1	0.1	0.1	0.1	0	0	0
Mongolia	Sant	0.2	0.2	0.2	0	0	0	0.1	0.1	0.1	0	0	0
Mongolia	Sant	0.2	0.2	0.2	0	0	0	0.1	0.1	0.1	0	0	0
Mongolia	Santmargats	0.2	0.2	0.3	0	0	0	0.1	0.1	0.1	0	0	0
Mongolia	Saykhan	0.2	0.2	0.2	0	0	0	0.1	0.1	0.1	0	0	0
Mongolia	Selenge	0.2	0.2	0.2	0	0	0	0.1	0.1	0.1	0	0	0
Mongolia	Sergelen	0.2	0.2	0.2	0	0	0	0.1	0.1	0.1	0	0	0
Mongolia	Sergelen	0.2	0.1	0.2	0	0	0	0.1	0.1	0.1	0	0	0
Mongolia	Sevrei	0.2	0.2	0.2	0	0	0	0.1	0.1	0.1	0	0	0
Mongolia	Shaamar	0.2	0.2	0.2	0	0	0	0.1	0.1	0.1	0	0	0
Mongolia	Sharga	0.2	0.2	0.2	0	0	0	0.1	0.1	0.1	0	0	0
Mongolia	Sharyngol	0.2	0.2	0.2	0	0	0	0.1	0.1	0.1	0	0	0
Mongolia	Shilüüstei	0.2	0.2	0.2	0	0	0	0.1	0.1	0.1	0	0	0
Mongolia	Shine-Ider	0.1	0.1	0.2	0	0	0	0.1	0.1	0.1	0	0	0
Mongolia	Shinejinst	0.2	0.2	0.2	0	0	0	0.1	0.1	0.1	0	0	0
Mongolia	Shiveegovi	0.2	0.2	0.2	0	0	0	0.1	0.1	0.1	0	0	0

Country	Unit	2000 mortality mean rate (per 10,000)	2000 mortality lower rate (per 10,000)	2000 mortality upper rate (per 10,000)	2000 mortality mean count	2000 mortality lower count	2000 mortality upper count	2017 mortality mean rate (per 10,000)	2017 mortality lower rate (per 10,000)	2017 mortality upper rate (per 10,000)	2017 mortality mean count	2017 mortality lower count	2017 mortality upper count
Mongolia	Songino	0.2	0.2	0.2	0	0	0	0.1	0.1	0.1	0	0	0
Mongolia	Sükhbaatar	0.1	0.1	0.2	0	0	0	0.0	0.0	0.1	0	0	0
Mongolia	Sümber	0.2	0.2	0.2	0	0	0	0.1	0.1	0.1	0	0	0
Mongolia	Sümber	0.2	0.2	0.2	0	0	0	0.1	0.1	0.1	0	0	0
Mongolia	Taishir	0.2	0.2	0.2	0	0	0	0.1	0.1	0.1	0	0	0
Mongolia	Taragt	0.2	0.2	0.2	0	0	0	0.1	0.1	0.1	0	0	0
Mongolia	Tarialan	0.2	0.2	0.3	0	0	0	0.1	0.1	0.1	0	0	0
Mongolia	Tarialan	0.2	0.1	0.2	0	0	0	0.1	0.1	0.1	0	0	0
Mongolia	Tariat	0.2	0.2	0.2	0	0	0	0.1	0.1	0.1	0	0	0
Mongolia	Telmen	0.2	0.2	0.2	0	0	0	0.1	0.1	0.1	0	0	0
Mongolia	Tes	0.2	0.2	0.3	0	0	0	0.1	0.1	0.1	0	0	0
Mongolia	Tes	0.2	0.2	0.2	0	0	0	0.1	0.1	0.1	0	0	0
Mongolia	Teshig	0.2	0.2	0.2	0	0	0	0.1	0.1	0.1	0	0	0
Mongolia	Tögrög	0.2	0.2	0.2	0	0	0	0.1	0.1	0.1	0	0	0
Mongolia	Tögrög	0.2	0.2	0.2	0	0	0	0.1	0.1	0.1	0	0	0
Mongolia	Tolbo	0.2	0.1	0.2	0	0	0	0.1	0.1	0.1	0	0	0
Mongolia	Tömörbulag	0.1	0.1	0.2	0	0	0	0.1	0.1	0.1	0	0	0
Mongolia	Tonkhil	0.2	0.2	0.3	0	0	0	0.1	0.1	0.1	0	0	0
Mongolia	Tosontsengel	0.2	0.2	0.2	0	0	0	0.1	0.1	0.1	0	0	0
Mongolia	Tosontsengel	0.1	0.1	0.2	0	0	0	0.1	0.1	0.1	0	0	0
Mongolia	Tsagaan-Ovoo	0.2	0.2	0.2	0	0	0	0.1	0.1	0.1	0	0	0
Mongolia	Tsagaan-Uul	0.1	0.1	0.2	0	0	0	0.1	0.1	0.1	0	0	0
Mongolia	Tsagaan-Üür	0.2	0.2	0.2	0	0	0	0.1	0.1	0.1	0	0	0
Mongolia	Tsagaanchuluut	0.2	0.2	0.2	0	0	0	0.1	0.1	0.1	0	0	0
Mongolia	Tsagaandelger	0.2	0.2	0.2	0	0	0	0.1	0.1	0.1	0	0	0
Mongolia	Tsagaanhairhan	0.2	0.2	0.3	0	0	0	0.1	0.1	0.1	0	0	0
Mongolia	Tsagaankhairkhan	0.2	0.2	0.2	0	0	0	0.1	0.1	0.1	0	0	0
Mongolia	Tsagaannuur	0.2	0.2	0.2	0	0	0	0.1	0.1	0.1	0	0	0
Mongolia	Tsagaannuur	0.2	0.2	0.2	0	0	0	0.1	0.1	0.1	0	0	0
Mongolia	Tsagaannuur	0.2	0.1	0.2	0	0	0	0.1	0.1	0.1	0	0	0
Mongolia	Tsakhir	0.2	0.2	0.2	0	0	0	0.1	0.1	0.1	0	0	0
Mongolia	Tseel	0.2	0.2	0.2	0	0	0	0.1	0.1	0.1	0	0	0
Mongolia	Tseel	0.2	0.2	0.2	0	0	0	0.1	0.1	0.1	0	0	0
Mongolia	Tsengel	0.2	0.1	0.2	0	0	0	0.1	0.1	0.1	0	0	0
Mongolia	Tsenkher	0.2	0.2	0.2	0	0	0	0.1	0.1	0.1	0	0	0
Mongolia	Tsenkhermandal	0.2	0.2	0.2	0	0	0	0.1	0.1	0.1	0	0	0
Mongolia	Tsetseg	0.2	0.2	0.3	0	0	0	0.1	0.1	0.1	0	0	0
Mongolia	Tsetsen-Uul	0.2	0.2	0.2	0	0	0	0.1	0.1	0.1	0	0	0
Mongolia	Tsetserleg	0.2	0.2	0.2	0	0	0	0.1	0.1	0.1	0	0	0
Mongolia	Tsetserleg	0.2	0.1	0.2	0	0	0	0.1	0.1	0.1	0	0	0
Mongolia	Tsogt	0.2	0.2	0.2	0	0	0	0.1	0.1	0.1	0	0	0
Mongolia	Tsogt-Ovoo	0.2	0.2	0.2	0	0	0	0.1	0.1	0.1	0	0	0
Mongolia	Tsogttsetsii	0.2	0.2	0.2	0	0	0	0.1	0.1	0.1	0	0	0
Mongolia	Tüdevtei	0.2	0.2	0.2	0	0	0	0.1	0.1	0.1	0	0	0
Mongolia	Tümentsogt	0.1	0.1	0.2	0	0	0	0.1	0.1	0.1	0	0	0
Mongolia	Tünel	0.1	0.1	0.2	0	0	0	0.1	0.1	0.1	0	0	0
Mongolia	Türgen	0.2	0.2	0.2	0	0	0	0.1	0.1	0.1	0	0	0
Mongolia	Tüshig	0.2	0.2	0.2	0	0	0	0.1	0.1	0.1	0	0	0
Mongolia	Tüvshinshiree	0.1	0.1	0.2	0	0	0	0.1	0.1	0.1	0	0	0
Mongolia	Tüvshrüülekh	0.2	0.2	0.2	0	0	0	0.1	0.1	0.1	0	0	0
Mongolia	Ugtaal	0.2	0.2	0.2	0	0	0	0.1	0.1	0.1	0	0	0
Mongolia	Ulaan-Uul	0.2	0.1	0.2	0	0	0	0.1	0.1	0.1	0	0	0
Mongolia	Ulaanbadrakh	0.2	0.2	0.2	0	0	0	0.1	0.1	0.1	0	0	0
Mongolia	Ulaangom	0.2	0.2	0.3	1	1	1	0.0	0.0	0.1	0	0	0
Mongolia	Ulaankhus	0.2	0.1	0.2	0	0	0	0.1	0.1	0.1	0	0	0
Mongolia	Ulan Bator	0.2	0.2	0.2	12	10	13	0.1	0.1	0.1	10	9	12
Mongolia	Urgamal	0.2	0.2	0.3	0	0	0	0.1	0.1	0.1	0	0	0
Mongolia	Uulbayan	0.1	0.1	0.2	0	0	0	0.1	0.1	0.1	0	0	0
Mongolia	Uyanga	0.2	0.2	0.2	0	0	0	0.1	0.1	0.1	0	0	0
Mongolia	Üyench	0.2	0.2	0.3	0	0	0	0.1	0.1	0.1	0	0	0

Table 3: Diarrhoea Mortality by Unit *(continued)*

Country	Unit	2000 mortality mean rate (per 10,000)	lower rate (per 10,000)	upper rate (per 10,000)	mean count	lower count	upper count	2017 mortality mean rate (per 10,000)	lower rate (per 10,000)	upper rate (per 10,000)	mean count	lower count	upper count
Mongolia	Yaruu	0.2	0.2	0.2	0	0	0	0.1	0.1	0.1	0	0	0
Mongolia	Yeröö	0.2	0.2	0.2	0	0	0	0.1	0.1	0.1	0	0	0
Mongolia	Yesönbulag	0.2	0.2	0.2	0	0	1	0.1	0.1	0.1	0	0	0
Mongolia	Yesönzüil	0.2	0.2	0.2	0	0	0	0.1	0.1	0.1	0	0	0
Mongolia	Zaamar	0.2	0.2	0.2	0	0	0	0.1	0.1	0.1	0	0	0
Mongolia	Zag	0.2	0.2	0.2	0	0	0	0.1	0.1	0.1	0	0	0
Mongolia	Zamyn-Üüd	0.2	0.1	0.2	0	0	0	0.1	0.1	0.1	0	0	0
Mongolia	Zavkhan	0.2	0.2	0.3	0	0	0	0.1	0.1	0.1	0	0	0
Mongolia	Zavkhanmandal	0.2	0.2	0.3	0	0	0	0.1	0.1	0.1	0	0	0
Mongolia	Zereg	0.2	0.2	0.3	0	0	0	0.1	0.1	0.1	0	0	0
Mongolia	Züünbayan-Ulaan	0.2	0.2	0.2	0	0	0	0.1	0.1	0.1	0	0	0
Mongolia	Züünbüren	0.2	0.2	0.2	0	0	0	0.1	0.1	0.1	0	0	0
Mongolia	Züüngovi	0.2	0.2	0.3	0	0	0	0.1	0.1	0.1	0	0	0
Mongolia	Züünkhangai	0.2	0.2	0.2	0	0	0	0.1	0.1	0.1	0	0	0
Tajikistan	Asht	2.9	1.7	4.4	46	28	70	0.9	0.6	1.2	18	12	26
Tajikistan	Ayni	2.5	1.6	3.8	23	15	36	1.0	0.7	1.3	10	7	14
Tajikistan	Baljuvon	3.0	1.8	4.5	9	6	14	1.9	1.4	2.5	8	6	11
Tajikistan	Bokhtar	2.9	1.8	4.7	99	62	161	1.4	1.1	1.8	69	54	88
Tajikistan	Danghara	3.0	1.8	4.5	46	27	70	1.9	1.5	2.4	44	34	55
Tajikistan	Darvoz	2.7	1.7	4.0	7	4	10	1.4	1.0	1.9	3	2	4
Tajikistan	Farkhor	3.0	1.9	4.7	53	33	83	2.1	1.6	2.7	51	38	66
Tajikistan	Fayzobod	2.6	1.5	3.9	27	16	40	1.4	1.0	1.8	19	14	25
Tajikistan	Ghafurov	2.8	1.7	4.2	190	116	284	0.8	0.5	1.1	66	44	91
Tajikistan	Ghonchi	2.6	1.7	4.1	45	29	70	0.8	0.6	1.1	19	14	26
Tajikistan	Hissor	2.6	1.5	4.1	77	44	120	1.2	0.9	1.4	47	37	59
Tajikistan	Isfara	2.8	1.6	4.2	69	41	105	0.9	0.6	1.3	29	18	41
Tajikistan	Ishkoshim	2.5	1.5	3.7	6	4	9	1.1	0.7	1.6	3	2	4
Tajikistan	Istaravshan	2.6	1.7	4.1	61	38	94	0.8	0.6	1.1	26	18	36
Tajikistan	Jabor Rasulov	2.7	1.7	4.3	37	23	58	0.8	0.5	1.1	13	9	17
Tajikistan	Jilikul	2.9	1.7	4.6	41	24	66	1.3	1.0	1.7	25	19	32
Tajikistan	Jirgatol	2.8	1.7	4.3	22	14	34	1.1	0.7	1.6	10	7	15
Tajikistan	Jomi	3.0	1.9	4.6	53	34	82	1.5	1.2	1.9	39	31	48
Tajikistan	Khovaling	3.0	1.8	4.6	18	11	28	2.0	1.5	2.7	18	14	25
Tajikistan	Khuroson	2.9	1.9	4.6	37	24	58	1.4	1.1	1.8	26	21	33
Tajikistan	Kolkhozobod	2.9	1.8	4.7	54	34	88	1.4	1.0	1.8	38	28	49
Tajikistan	Konibodom	2.9	1.7	4.4	65	38	101	0.9	0.6	1.3	23	16	33
Tajikistan	Kuhistoni Mastchoh	2.5	1.6	3.7	6	4	9	1.0	0.7	1.3	3	2	5
Tajikistan	Kulob	3.0	1.8	4.8	69	40	109	2.1	1.6	2.8	65	49	86
Tajikistan	Matchin	2.7	1.7	3.9	30	19	43	0.8	0.5	1.1	11	7	16
Tajikistan	Moskva	3.0	1.9	4.7	51	31	79	2.2	1.7	2.8	47	36	60
Tajikistan	Muminobod	3.0	1.8	4.7	29	17	46	2.0	1.4	2.8	27	18	37
Tajikistan	Murghob	2.4	1.6	3.5	5	3	7	1.0	0.7	1.3	1	1	2
Tajikistan	Norak	3.0	1.7	4.4	19	11	29	1.7	1.3	2.2	15	11	19
Tajikistan	Nosir Khusrav	2.9	1.7	4.3	10	6	14	1.2	0.8	1.7	7	5	9
Tajikistan	Nurobod	2.7	1.6	4.3	23	13	36	1.4	1.0	2.0	16	11	22
Tajikistan	Pandjakent	2.4	1.6	3.8	68	44	107	0.9	0.6	1.4	35	24	52
Tajikistan	Panj	2.9	1.8	4.7	35	22	57	1.7	1.2	2.2	25	18	34
Tajikistan	Qabodiyon	2.9	1.7	4.4	50	30	76	1.2	0.9	1.7	30	22	41
Tajikistan	Qumsangir	2.9	1.7	4.4	40	24	61	1.3	0.9	1.8	28	19	37
Tajikistan	Rasht	2.7	1.6	4.1	35	21	52	1.4	1.0	1.9	25	18	34
Tajikistan	Roghun	2.6	1.5	4.0	12	7	19	1.5	1.1	2.0	10	7	14
Tajikistan	Roshtqala	2.5	1.5	3.8	7	4	10	1.2	0.8	1.7	3	2	4
Tajikistan	Rudaki	2.5	1.5	3.8	174	104	265	1.0	0.8	1.3	109	86	140
Tajikistan	Rudaki	2.6	1.6	4.0	116	71	181	1.1	0.9	1.4	87	68	108
Tajikistan	Rushon	2.5	1.6	3.8	5	3	8	1.1	0.8	1.6	2	2	3
Tajikistan	Sarband	2.9	1.8	4.6	33	20	52	1.7	1.3	2.1	22	17	27
Tajikistan	Shahrinav	2.6	1.5	3.9	36	21	55	1.1	0.9	1.4	22	17	28
Tajikistan	Shahriston	2.5	1.5	3.8	12	8	19	0.8	0.6	1.2	5	4	7
Tajikistan	Shahrituz	2.9	1.7	4.4	42	25	64	1.2	0.8	1.6	24	17	32

Table 3: Diarrhoea Mortality by Unit *(continued)*

		2000 mortality						2017 mortality					
Country	Unit	mean rate (per 10,000)	lower rate (per 10,000)	upper rate (per 10,000)	mean count	lower count	upper count	mean rate (per 10,000)	lower rate (per 10,000)	upper rate (per 10,000)	mean count	lower count	upper count
Tajikistan	Shughnon	2.6	1.7	4.0	17	11	25	1.4	0.9	2.0	9	6	12
Tajikistan	Shurobod	3.0	1.9	4.6	20	12	30	2.1	1.6	2.7	18	13	23
Tajikistan	Sovet	2.9	1.7	4.5	22	13	34	2.1	1.6	2.7	22	17	28
Tajikistan	Spitamen	2.7	1.6	4.2	39	22	59	0.8	0.5	1.1	14	10	19
Tajikistan	Tavildara	2.7	1.8	4.0	6	4	9	1.3	1.0	1.8	6	4	8
Tajikistan	Tojikobod	2.7	1.7	4.2	13	8	19	1.3	0.9	1.9	9	6	12
Tajikistan	Tursunzoda	2.5	1.5	3.9	70	42	108	1.1	0.8	1.5	45	33	59
Tajikistan	Vahdat	2.6	1.7	4.0	73	46	112	1.2	0.9	1.5	52	40	65
Tajikistan	Vakhsh	2.9	1.8	4.6	60	37	96	1.6	1.2	2.0	47	36	60
Tajikistan	Vanj	2.6	1.6	3.9	8	5	12	1.1	0.7	1.6	4	3	6
Tajikistan	Varzob	2.6	1.6	4.0	24	15	38	1.1	0.9	1.4	16	13	20
Tajikistan	Vose	3.0	1.9	4.8	72	45	115	2.2	1.7	2.8	67	51	86
Tajikistan	Yovon	3.0	1.8	4.5	63	39	94	1.5	1.2	1.9	50	39	62
Tajikistan	Zafarobod	2.4	1.5	3.7	18	11	27	0.7	0.5	1.1	7	5	10
Turkmenistan	Ahal	1.5	1.1	2.0	111	80	152	0.1	0.1	0.2	13	8	21
Turkmenistan	Aşgabat	1.5	0.9	2.2	50	31	72	0.1	0.1	0.2	6	3	11
Turkmenistan	Balkan	1.5	1.0	2.1	55	38	77	0.1	0.1	0.2	4	3	5
Turkmenistan	Chardzhou	1.4	1.0	2.0	145	99	207	0.1	0.1	0.1	12	8	17
Turkmenistan	Mary	1.5	1.0	2.1	160	109	224	0.1	0.1	0.2	12	8	18
Turkmenistan	Tashauz	1.3	0.8	2.0	115	74	177	0.1	0.1	0.2	11	7	18
Uzbekistan	Amudaryo	0.2	0.1	0.3	5	3	8	0.0	0.0	0.1	1	0	1
Uzbekistan	Andijon	0.2	0.1	0.4	8	4	12	0.0	0.0	0.1	2	1	3
Uzbekistan	Angor	0.2	0.1	0.3	3	2	4	0.0	0.0	0.1	1	0	1
Uzbekistan	Aral Sea	0.2	0.2	0.4	0	0	0	0.1	0.0	0.1	0	0	0
Uzbekistan	Arnasoy	0.2	0.1	0.3	1	0	1	0.0	0.0	0.1	0	0	0
Uzbekistan	Asaka	0.2	0.1	0.4	5	3	8	0.0	0.0	0.1	1	1	2
Uzbekistan	Baliqchi	0.2	0.1	0.4	4	2	6	0.0	0.0	0.1	1	0	1
Uzbekistan	Bandixon	0.2	0.1	0.3	1	0	1	0.1	0.0	0.1	0	0	0
Uzbekistan	Baxmal	0.2	0.1	0.3	0	0	1	0.0	0.0	0.1	0	0	0
Uzbekistan	Bekobod	0.3	0.2	0.5	5	3	8	0.0	0.0	0.1	1	0	1
Uzbekistan	Beruniy	0.2	0.1	0.3	8	5	11	0.0	0.0	0.1	1	1	2
Uzbekistan	Beshariq	0.3	0.2	0.4	9	6	14	0.0	0.0	0.1	1	1	2
Uzbekistan	Bo'ka	0.3	0.2	0.4	3	2	5	0.0	0.0	0.1	0	0	1
Uzbekistan	Bo'stonliq	0.3	0.2	0.4	5	3	8	0.0	0.0	0.1	1	0	1
Uzbekistan	Bo'zsuv	0.2	0.1	0.3	2	1	4	0.0	0.0	0.1	0	0	1
Uzbekistan	Bog'dod	0.2	0.1	0.4	3	2	4	0.0	0.0	0.1	1	0	1
Uzbekistan	Bog'ot	0.2	0.1	0.3	3	2	5	0.0	0.0	0.1	1	0	1
Uzbekistan	Boyovut	0.2	0.1	0.4	2	1	3	0.0	0.0	0.1	0	0	0
Uzbekistan	Boysun	0.2	0.1	0.3	4	3	6	0.1	0.0	0.1	1	1	2
Uzbekistan	Buloqboshi	0.2	0.1	0.4	2	1	4	0.0	0.0	0.1	1	0	1
Uzbekistan	Bulung'ur	0.2	0.1	0.3	4	2	6	0.0	0.0	0.1	1	0	1
Uzbekistan	Buvayda	0.2	0.1	0.4	3	2	5	0.0	0.0	0.1	1	0	1
Uzbekistan	Buxoro	0.2	0.1	0.3	7	4	11	0.1	0.0	0.1	2	1	3
Uzbekistan	Chimboy	0.2	0.1	0.3	2	1	2	0.0	0.0	0.1	0	0	0
Uzbekistan	Chinoz	0.3	0.1	0.4	3	2	5	0.0	0.0	0.1	0	0	1
Uzbekistan	Chiroqchi	0.2	0.1	0.4	7	4	10	0.1	0.0	0.1	2	1	3
Uzbekistan	Chortoq	0.2	0.1	0.3	4	3	7	0.0	0.0	0.1	1	1	2
Uzbekistan	Chust	0.2	0.1	0.4	6	4	9	0.0	0.0	0.1	1	1	2
Uzbekistan	Dang'ara	0.2	0.2	0.4	6	4	9	0.0	0.0	0.1	1	1	2
Uzbekistan	Dehqonobod	0.2	0.1	0.4	3	2	4	0.1	0.0	0.1	1	1	2
Uzbekistan	Denov	0.3	0.2	0.4	8	5	13	0.1	0.0	0.1	2	1	3
Uzbekistan	Do'stlik	0.2	0.1	0.3	1	1	2	0.0	0.0	0.1	0	0	0
Uzbekistan	Ellikqala	0.2	0.1	0.3	1	1	2	0.0	0.0	0.1	0	0	1
Uzbekistan	Farg'ona	0.2	0.1	0.3	12	7	17	0.0	0.0	0.1	3	1	4
Uzbekistan	Forish	0.2	0.1	0.3	3	2	4	0.0	0.0	0.1	1	1	1
Uzbekistan	Furqat	0.3	0.2	0.4	4	3	6	0.0	0.0	0.1	1	0	1
Uzbekistan	G'allaorol	0.2	0.1	0.3	3	2	5	0.0	0.0	0.1	1	1	2
Uzbekistan	G'ijduvon	0.2	0.1	0.3	1	1	2	0.1	0.0	0.1	0	0	1
Uzbekistan	G'uzor	0.2	0.1	0.4	7	4	11	0.1	0.0	0.1	3	2	5
Uzbekistan	Guliston	0.2	0.1	0.4	2	1	3	0.0	0.0	0.1	0	0	1

Table 3: Diarrhoea Mortality by Unit *(continued)*

Country	Unit	2000 mortality mean rate (per 10,000)	lower rate (per 10,000)	upper rate (per 10,000)	mean count	lower count	upper count	2017 mortality mean rate (per 10,000)	lower rate (per 10,000)	upper rate (per 10,000)	mean count	lower count	upper count
Uzbekistan	Gurlan	0.2	0.1	0.3	3	2	5	0.0	0.0	0.1	1	0	1
Uzbekistan	Hazorasp	0.2	0.1	0.3	5	3	7	0.0	0.0	0.1	1	1	2
Uzbekistan	Ishtixon	0.2	0.1	0.3	5	3	8	0.0	0.0	0.1	1	1	2
Uzbekistan	Izboskan	0.2	0.1	0.4	5	3	8	0.0	0.0	0.1	1	1	2
Uzbekistan	Jalolquduq	0.2	0.1	0.4	4	2	6	0.0	0.0	0.1	1	0	2
Uzbekistan	Jarqo'rg'on	0.2	0.1	0.3	5	3	8	0.1	0.0	0.1	1	1	2
Uzbekistan	Jizzax	0.2	0.1	0.4	6	4	10	0.0	0.0	0.1	1	1	2
Uzbekistan	Jomboy	0.2	0.1	0.3	6	4	9	0.0	0.0	0.1	1	1	2
Uzbekistan	Jondor	0.2	0.1	0.3	4	2	7	0.0	0.0	0.1	1	1	2
Uzbekistan	Karmana	0.2	0.1	0.3	2	1	4	0.0	0.0	0.1	1	0	1
Uzbekistan	Kasbi	0.2	0.1	0.3	6	4	10	0.1	0.0	0.1	1	1	2
Uzbekistan	Kattaqo'rg'on	0.2	0.1	0.3	7	4	11	0.0	0.0	0.1	2	1	3
Uzbekistan	Kegeyli	0.2	0.1	0.3	2	1	2	0.0	0.0	0.1	0	0	1
Uzbekistan	Kitob	0.2	0.1	0.3	4	2	6	0.1	0.0	0.1	1	0	1
Uzbekistan	Kogon	0.2	0.1	0.4	4	2	6	0.1	0.0	0.1	1	0	1
Uzbekistan	Konimex	0.2	0.1	0.3	3	2	5	0.1	0.0	0.1	1	0	1
Uzbekistan	Koson	0.2	0.1	0.4	5	3	7	0.1	0.0	0.1	1	1	3
Uzbekistan	Kosonsoy	0.2	0.1	0.4	6	3	9	0.0	0.0	0.1	1	1	2
Uzbekistan	Marhamat	0.2	0.1	0.4	3	2	4	0.0	0.0	0.1	1	0	1
Uzbekistan	Mingbuloq	0.2	0.1	0.3	3	2	4	0.0	0.0	0.1	1	0	1
Uzbekistan	Mirzacho'l	0.2	0.1	0.3	1	0	1	0.0	0.0	0.1	0	0	0
Uzbekistan	Mirzaobod	0.2	0.1	0.3	2	1	3	0.0	0.0	0.1	0	0	1
Uzbekistan	Mo'ynoq	0.2	0.1	0.3	3	2	4	0.0	0.0	0.1	1	0	1
Uzbekistan	Muborak	0.2	0.1	0.4	7	4	11	0.1	0.0	0.1	1	1	2
Uzbekistan	Muzrabot	0.2	0.1	0.3	2	1	3	0.1	0.0	0.1	0	0	1
Uzbekistan	Namangan	0.2	0.1	0.3	5	3	8	0.0	0.0	0.1	1	1	2
Uzbekistan	Narpay	0.2	0.1	0.3	0	0	1	0.0	0.0	0.1	0	0	0
Uzbekistan	Navbahor	0.2	0.1	0.3	3	2	5	0.1	0.0	0.1	1	0	1
Uzbekistan	Nishon	0.2	0.1	0.4	4	2	6	0.1	0.0	0.1	2	1	3
Uzbekistan	Norin	0.2	0.1	0.4	2	1	4	0.0	0.0	0.1	1	0	1
Uzbekistan	Nukus	0.2	0.1	0.3	3	2	5	0.0	0.0	0.1	1	0	1
Uzbekistan	Nurobod	0.2	0.1	0.3	3	2	4	0.0	0.0	0.1	1	0	1
Uzbekistan	Nurota	0.2	0.1	0.3	5	3	7	0.1	0.0	0.1	1	1	2
Uzbekistan	O'rtachirchiq	0.3	0.2	0.4	35	22	53	0.0	0.0	0.1	5	2	8
Uzbekistan	O'zbekiston	0.3	0.2	0.4	7	4	10	0.0	0.0	0.1	1	1	2
Uzbekistan	Ohangaron	0.3	0.2	0.4	10	6	15	0.0	0.0	0.1	1	1	2
Uzbekistan	Olot	0.2	0.1	0.3	2	1	3	0.0	0.0	0.1	1	0	1
Uzbekistan	Oltiariq	0.2	0.1	0.3	5	3	7	0.0	0.0	0.1	1	1	2
Uzbekistan	Oltinko'l	0.2	0.1	0.4	3	2	6	0.0	0.0	0.1	1	0	1
Uzbekistan	Oltinsoy	0.2	0.1	0.4	3	2	4	0.1	0.0	0.1	1	0	1
Uzbekistan	Oqdaryo	0.2	0.1	0.3	4	2	6	0.0	0.0	0.1	1	1	2
Uzbekistan	Oqoltin	0.2	0.1	0.4	1	1	2	0.0	0.0	0.1	0	0	1
Uzbekistan	Oqqo'rg'on	0.3	0.2	0.4	2	1	3	0.0	0.0	0.1	0	0	0
Uzbekistan	Oxunboboev	0.2	0.1	0.3	5	3	7	0.0	0.0	0.1	1	1	2
Uzbekistan	Parkent	0.3	0.2	0.4	3	2	5	0.0	0.0	0.1	1	0	1
Uzbekistan	Pastdarg'om	0.2	0.1	0.3	7	5	11	0.0	0.0	0.1	2	1	3
Uzbekistan	Paxtachi	0.2	0.1	0.3	0	0	0	0.0	0.0	0.1	0	0	0
Uzbekistan	Paxtakor	0.2	0.1	0.4	1	1	2	0.0	0.0	0.1	0	0	0
Uzbekistan	Paxtaobod	0.2	0.1	0.4	3	2	5	0.0	0.0	0.1	1	0	1
Uzbekistan	Payariq	0.2	0.1	0.3	5	3	9	0.0	0.0	0.1	2	1	3
Uzbekistan	Peshku	0.2	0.1	0.3	3	2	4	0.0	0.0	0.1	1	1	1
Uzbekistan	Piskent	0.3	0.2	0.4	6	3	8	0.0	0.0	0.1	1	0	1
Uzbekistan	Pop	0.2	0.1	0.4	7	4	11	0.0	0.0	0.1	1	1	2
Uzbekistan	Qamashi	0.2	0.1	0.4	4	2	7	0.1	0.0	0.1	1	1	2
Uzbekistan	Qanliko'l	0.2	0.1	0.3	1	0	1	0.0	0.0	0.1	0	0	0
Uzbekistan	Qarshi	0.2	0.1	0.4	8	5	13	0.1	0.0	0.1	3	2	5
Uzbekistan	Qibray	0.3	0.2	0.4	10	6	16	0.0	0.0	0.1	2	1	3
Uzbekistan	Qiziltepa	0.2	0.1	0.4	2	1	4	0.1	0.0	0.1	1	0	1
Uzbekistan	Qiziriq	0.2	0.1	0.3	1	1	2	0.0	0.0	0.1	0	0	0
Uzbekistan	Qo'ng'irot	0.2	0.1	0.3	7	4	9	0.0	0.0	0.1	2	1	3

Table 3: Diarrhoea Mortality by Unit *(continued)*

Country	Unit	2000 mortality mean rate (per 10,000)	lower rate (per 10,000)	upper rate (per 10,000)	mean count	lower count	upper count	2017 mortality mean rate (per 10,000)	lower rate (per 10,000)	upper rate (per 10,000)	mean count	lower count	upper count
Uzbekistan	Qo'rg'ontepa	0.2	0.1	0.3	7	4	11	0.0	0.0	0.1	1	1	2
Uzbekistan	Qo'shko'pir	0.2	0.1	0.3	4	3	6	0.0	0.0	0.1	1	0	2
Uzbekistan	Qo'shrabot	0.2	0.1	0.3	2	2	4	0.0	0.0	0.1	1	0	1
Uzbekistan	Qorako'l	0.2	0.1	0.3	2	1	2	0.0	0.0	0.1	1	0	1
Uzbekistan	Qorao'zak	0.2	0.1	0.3	1	1	2	0.0	0.0	0.1	0	0	1
Uzbekistan	Qorovulbozor	0.2	0.1	0.4	4	2	6	0.1	0.0	0.1	1	0	1
Uzbekistan	Qumqo'rg'on	0.2	0.1	0.4	6	4	9	0.1	0.0	0.1	2	1	3
Uzbekistan	Quva	0.2	0.1	0.3	5	3	7	0.0	0.0	0.1	1	1	2
Uzbekistan	Quyichirchiq	0.3	0.2	0.4	4	2	5	0.0	0.0	0.1	0	0	1
Uzbekistan	Rishton	0.2	0.1	0.4	3	2	5	0.0	0.0	0.1	1	0	1
Uzbekistan	Romitan	0.2	0.1	0.3	3	2	4	0.0	0.0	0.1	1	0	1
Uzbekistan	Samarqand	0.2	0.1	0.3	5	3	7	0.0	0.0	0.1	2	1	3
Uzbekistan	Sariosiyo	0.3	0.2	0.4	8	5	12	0.1	0.0	0.1	2	1	3
Uzbekistan	Sayxunobod	0.2	0.1	0.3	1	1	2	0.0	0.0	0.1	0	0	0
Uzbekistan	Shahrisabz	0.2	0.1	0.4	3	2	5	0.1	0.0	0.1	1	1	2
Uzbekistan	Shahrixon	0.2	0.1	0.3	4	3	6	0.0	0.0	0.1	1	0	1
Uzbekistan	Sharof Rashidov	0.2	0.1	0.4	2	1	3	0.0	0.0	0.1	0	0	0
Uzbekistan	Sherobod	0.2	0.1	0.3	4	3	7	0.1	0.0	0.1	1	1	2
Uzbekistan	Sho'rchi	0.2	0.2	0.4	4	2	6	0.1	0.0	0.1	1	0	1
Uzbekistan	Shofirkon	0.2	0.1	0.3	2	1	4	0.1	0.0	0.1	1	0	1
Uzbekistan	Shovot	0.2	0.1	0.3	4	2	6	0.0	0.0	0.1	1	1	2
Uzbekistan	Shumanay	0.2	0.1	0.3	1	0	1	0.0	0.0	0.1	0	0	0
Uzbekistan	Sirdaryo	0.2	0.1	0.3	5	3	7	0.0	0.0	0.1	1	0	1
Uzbekistan	So'x	0.3	0.2	0.5	0	0	1	0.1	0.0	0.1	0	0	0
Uzbekistan	Tashkent City	0.3	0.2	0.4	21	12	32	0.0	0.0	0.1	3	2	6
Uzbekistan	Taxtako'pir	0.2	0.1	0.3	1	1	2	0.0	0.0	0.1	0	0	1
Uzbekistan	Termiz	0.2	0.1	0.3	6	4	9	0.0	0.0	0.1	2	1	3
Uzbekistan	To'raqo'rg'on	0.2	0.1	0.4	4	2	6	0.0	0.0	0.1	1	0	2
Uzbekistan	To'rtko'l	0.2	0.1	0.3	2	1	3	0.0	0.0	0.1	1	0	1
Uzbekistan	Tomdi	0.2	0.2	0.4	5	3	8	0.1	0.0	0.1	1	1	2
Uzbekistan	Toshkent	0.3	0.2	0.4	4	2	7	0.0	0.0	0.1	1	0	1
Uzbekistan	Toshloq	0.2	0.1	0.3	4	2	6	0.0	0.0	0.1	1	1	2
Uzbekistan	Toyloq	0.2	0.1	0.3	2	2	4	0.0	0.0	0.1	0	0	1
Uzbekistan	Uchko'prik	0.2	0.2	0.4	4	2	6	0.0	0.0	0.1	1	0	1
Uzbekistan	Uchqo'rg'on	0.2	0.1	0.4	4	2	5	0.0	0.0	0.1	1	0	1
Uzbekistan	Uchquduq	0.2	0.1	0.3	2	1	2	0.0	0.0	0.1	1	0	1
Uzbekistan	Ulug'nor	0.2	0.1	0.3	4	2	5	0.0	0.0	0.1	1	0	1
Uzbekistan	Urganch	0.2	0.1	0.3	7	4	11	0.0	0.0	0.1	2	1	3
Uzbekistan	Urgut	0.2	0.1	0.3	4	3	6	0.0	0.0	0.1	1	1	2
Uzbekistan	Usmon Yusupov	0.2	0.1	0.3	9	6	14	0.1	0.0	0.1	2	1	3
Uzbekistan	Uychi	0.2	0.1	0.4	5	3	8	0.0	0.0	0.1	1	1	2
Uzbekistan	Uzun	0.3	0.2	0.5	4	2	6	0.1	0.0	0.1	1	1	2
Uzbekistan	Vobkent	0.2	0.1	0.4	7	4	11	0.1	0.0	0.1	2	1	3
Uzbekistan	Xatirchi	0.2	0.1	0.3	7	4	11	0.0	0.0	0.1	2	1	3
Uzbekistan	Xiva	0.2	0.1	0.3	4	3	7	0.0	0.0	0.1	1	1	2
Uzbekistan	Xo'jaobod	0.2	0.1	0.4	5	3	7	0.0	0.0	0.1	1	1	2
Uzbekistan	Xo'jayli	0.2	0.1	0.3	5	3	8	0.0	0.0	0.1	1	1	2
Uzbekistan	Xonqa	0.2	0.1	0.3	3	2	5	0.0	0.0	0.1	1	0	2
Uzbekistan	Xovos	0.3	0.2	0.4	4	3	6	0.0	0.0	0.1	1	0	1
Uzbekistan	Yagiqo'rg'on	0.2	0.1	0.4	6	4	9	0.0	0.0	0.1	1	1	2
Uzbekistan	Yakkabog'	0.2	0.1	0.4	3	2	5	0.1	0.0	0.1	1	1	2
Uzbekistan	Yangiariq	0.2	0.1	0.3	2	1	3	0.0	0.0	0.1	1	0	1
Uzbekistan	Yangibozor	0.2	0.1	0.3	2	1	3	0.0	0.0	0.1	0	0	1
Uzbekistan	Yangiobod	0.5	0.3	0.7	2	1	3	0.1	0.0	0.1	0	0	1
Uzbekistan	Yangiyo'l	0.3	0.2	0.4	6	4	9	0.0	0.0	0.1	1	0	2
Uzbekistan	Yozyovon	0.2	0.1	0.3	3	2	4	0.0	0.0	0.1	1	0	1
Uzbekistan	Yuqorichirchiq	0.3	0.2	0.4	3	2	5	0.0	0.0	0.1	1	0	1
Uzbekistan	Zafarobod	0.2	0.1	0.3	1	1	1	0.0	0.0	0.1	0	0	0
Uzbekistan	Zangiota	0.3	0.2	0.4	18	11	28	0.0	0.0	0.1	3	1	5
Uzbekistan	Zarbdor	0.2	0.1	0.4	1	1	2	0.0	0.0	0.1	0	0	0

Table 3: Diarrhoea Mortality by Unit *(continued)*

Country	Unit	2000 mortality mean rate (per 10,000)	lower rate (per 10,000)	upper rate (per 10,000)	mean count	lower count	upper count	2017 mortality mean rate (per 10,000)	lower rate (per 10,000)	upper rate (per 10,000)	mean count	lower count	upper count
Uzbekistan	Zomin	0.2	0.1	0.4	4	3	7	0.0	0.0	0.1	1	0	2
**Latin America and Caribbean**													
Belize	Belize	0.3	0.2	0.4	2	1	3	0.1	0.1	0.2	1	1	1
Belize	Cayo	0.3	0.2	0.5	2	2	3	0.1	0.1	0.2	1	1	2
Belize	Corozal	0.3	0.2	0.4	1	1	2	0.1	0.1	0.2	1	1	1
Belize	Orange Walk	0.3	0.2	0.4	1	1	2	0.1	0.1	0.2	1	1	1
Belize	Stann Creek	0.3	0.2	0.4	1	1	2	0.1	0.1	0.2	1	1	1
Belize	Toledo	0.4	0.3	0.5	1	1	2	0.2	0.1	0.2	1	1	1
Bolivia	Abel Iturralde	1.4	1.2	1.6	2	2	3	0.2	0.2	0.2	1	0	1
Bolivia	Alonso de Ibáñez	1.4	1.1	1.6	6	5	7	0.2	0.1	0.2	1	1	1
Bolivia	Andrés Ibáñez	1.2	1.0	1.5	232	194	276	0.2	0.1	0.2	41	33	50
Bolivia	Aniceto Arce	1.3	1.0	1.5	10	8	12	0.2	0.1	0.2	1	1	1
Bolivia	Antonio Quijarro	1.2	1.0	1.5	7	6	9	0.2	0.1	0.2	2	1	2
Bolivia	Arani	1.3	1.1	1.5	12	9	14	0.2	0.1	0.2	1	1	2
Bolivia	Aroma	1.2	1.0	1.4	14	12	17	0.2	0.1	0.2	2	2	3
Bolivia	Arque	1.4	1.1	1.6	7	5	8	0.2	0.1	0.2	1	1	1
Bolivia	Atahuallpa	1.2	1.0	1.4	2	1	2	0.2	0.1	0.2	0	0	1
Bolivia	Ayopaya	1.4	1.1	1.6	12	10	14	0.2	0.1	0.2	1	1	1
Bolivia	Bautista Saavedra	1.3	1.1	1.5	2	2	2	0.2	0.1	0.2	0	0	0
Bolivia	Belisario Boeto	1.4	1.2	1.7	3	2	3	0.2	0.1	0.2	0	0	0
Bolivia	Bernardino Bilbao	1.4	1.2	1.7	2	2	3	0.2	0.1	0.2	0	0	0
Bolivia	Burnet O'Connor	1.3	1.1	1.6	4	3	5	0.2	0.1	0.2	1	0	1
Bolivia	Capinota	1.3	1.1	1.5	7	6	8	0.2	0.1	0.2	1	1	1
Bolivia	Carangas	1.2	1.0	1.4	4	4	5	0.2	0.1	0.2	1	0	1
Bolivia	Carrasco	1.4	1.1	1.7	24	20	28	0.2	0.1	0.2	3	3	4
Bolivia	Cercado	1.3	1.1	1.6	18	14	21	0.2	0.1	0.2	3	3	4
Bolivia	Cercado	1.2	1.0	1.4	45	37	52	0.2	0.1	0.2	8	7	9
Bolivia	Cercado	1.2	1.0	1.4	24	20	29	0.1	0.1	0.2	4	3	5
Bolivia	Cercado	1.2	1.0	1.4	94	77	109	0.2	0.1	0.2	15	12	18
Bolivia	Chapare	1.3	1.1	1.5	43	35	50	0.2	0.1	0.2	8	6	9
Bolivia	Charcas	1.4	1.2	1.7	9	8	11	0.2	0.1	0.2	1	1	1
Bolivia	Chayanta	1.4	1.1	1.6	20	17	23	0.2	0.1	0.2	3	2	3
Bolivia	Chiquitos	1.2	1.0	1.5	17	14	21	0.2	0.1	0.2	3	3	4
Bolivia	Cordillera	1.3	1.1	1.6	21	18	25	0.2	0.1	0.2	3	2	4
Bolivia	Cornelio Saavedra	1.4	1.1	1.6	13	11	16	0.2	0.1	0.2	1	1	2
Bolivia	Daniel Campos	1.2	0.9	1.4	1	1	1	0.2	0.1	0.2	0	0	0
Bolivia	Eduardo Avaroa	1.3	1.1	1.5	8	7	9	0.2	0.1	0.2	1	1	1
Bolivia	Eliodoro Camacho	1.3	1.0	1.5	9	8	11	0.2	0.1	0.2	1	1	1
Bolivia	Esteban Arce	1.3	1.1	1.5	7	6	8	0.2	0.1	0.2	1	1	1
Bolivia	Eustaquio Méndez	1.2	1.0	1.5	7	6	8	0.1	0.1	0.2	1	1	1
Bolivia	Federico Román	1.2	1.0	1.5	7	6	9	0.2	0.1	0.2	3	2	3
Bolivia	Florida	1.3	1.1	1.5	5	4	6	0.2	0.1	0.2	1	1	1
Bolivia	Franz Tamayo	1.4	1.1	1.6	3	3	4	0.2	0.2	0.2	1	1	1
Bolivia	Germán Jordán	1.2	1.0	1.4	6	5	6	0.2	0.1	0.2	1	1	1
Bolivia	Gran Chaco	1.2	1.0	1.5	19	15	23	0.1	0.1	0.2	3	2	3
Bolivia	Gualberto Villarroel	1.2	1.0	1.4	3	2	3	0.2	0.1	0.2	0	0	0
Bolivia	Hernando Siles	1.4	1.1	1.7	8	6	9	0.2	0.1	0.2	1	1	1
Bolivia	Ichilo	1.4	1.1	1.6	10	8	11	0.2	0.1	0.2	2	1	2
Bolivia	Ignacio Warnes	1.3	1.1	1.6	11	9	13	0.2	0.1	0.2	3	2	4
Bolivia	Ingavi	1.2	1.0	1.3	18	15	21	0.2	0.1	0.2	4	3	4
Bolivia	Inquisivi	1.3	1.1	1.5	10	9	12	0.2	0.1	0.2	1	1	2
Bolivia	Jaime Zudáñez	1.4	1.2	1.6	7	6	8	0.2	0.1	0.2	1	1	1
Bolivia	José Ballivián	1.4	1.2	1.6	14	12	17	0.2	0.2	0.2	2	2	3
Bolivia	José María Avilés	1.3	1.0	1.5	4	3	5	0.1	0.1	0.2	1	0	1

Table 3: Diarrhoea Mortality by Unit *(continued)*

Country	Unit	2000 mortality						2017 mortality					
		mean rate (per 10,000)	lower rate (per 10,000)	upper rate (per 10,000)	mean count	lower count	upper count	mean rate (per 10,000)	lower rate (per 10,000)	upper rate (per 10,000)	mean count	lower count	upper count
Bolivia	José María Linares	1.4	1.1	1.6	12	10	14	0.2	0.1	0.2	1	1	2
Bolivia	José Miguel de Velasco	1.3	1.0	1.6	13	11	16	0.2	0.1	0.2	2	2	2
Bolivia	Juana Azurduay de Padilla	1.5	1.2	1.7	6	5	7	0.2	0.1	0.2	1	0	1
Bolivia	Ladislao Cabrera	1.3	1.1	1.5	2	2	3	0.2	0.1	0.2	0	0	0
Bolivia	Larecaja	1.3	1.1	1.5	11	10	13	0.2	0.1	0.2	2	2	2
Bolivia	Litoral	1.2	1.0	1.4	1	0	1	0.2	0.1	0.2	0	0	0
Bolivia	Loayza	1.2	1.0	1.4	7	6	8	0.2	0.1	0.2	1	1	1
Bolivia	Los Andes	1.1	1.0	1.3	12	10	14	0.2	0.1	0.2	2	2	2
Bolivia	Luis Calvo	1.4	1.1	1.6	4	4	5	0.2	0.1	0.2	0	0	1
Bolivia	Madre de Dios	1.4	1.2	1.7	3	2	4	0.2	0.2	0.3	1	1	2
Bolivia	Mamoré	1.4	1.1	1.6	3	2	3	0.2	0.1	0.2	0	0	0
Bolivia	Manco Kapac	1.2	1.0	1.3	3	3	4	0.2	0.1	0.2	0	0	1
Bolivia	Manuel María Caballero	1.4	1.1	1.6	4	3	5	0.2	0.1	0.2	1	0	1
Bolivia	Manuripi	1.4	1.1	1.6	2	2	3	0.2	0.2	0.2	1	1	1
Bolivia	Marbán	1.4	1.1	1.7	8	7	10	0.2	0.1	0.2	2	1	2
Bolivia	Mizque	1.4	1.1	1.6	8	7	10	0.2	0.1	0.2	1	1	1
Bolivia	Modesto Omiste	1.3	1.0	1.5	6	5	7	0.2	0.1	0.2	1	1	1
Bolivia	Moxos	1.4	1.2	1.6	6	5	7	0.2	0.2	0.2	1	1	1
Bolivia	Muñecas	1.3	1.1	1.5	5	4	6	0.2	0.1	0.2	1	1	1
Bolivia	Narciso Campero	1.4	1.1	1.6	8	7	9	0.2	0.1	0.2	1	1	1
Bolivia	Nor Chichas	1.3	1.1	1.5	7	6	9	0.2	0.1	0.2	1	1	1
Bolivia	Nor Cinti	1.4	1.1	1.7	15	12	17	0.2	0.1	0.2	2	1	2
Bolivia	Nor Lípez	1.2	0.9	1.4	2	2	3	0.2	0.1	0.2	0	0	1
Bolivia	Nor Yungas	1.3	1.1	1.5	12	10	15	0.2	0.1	0.2	2	2	2
Bolivia	Ñuflo de Chávez	1.4	1.1	1.6	22	18	26	0.2	0.1	0.2	4	3	4
Bolivia	Obispo Santistevan	1.3	1.0	1.5	26	21	31	0.2	0.1	0.2	4	3	5
Bolivia	Omasuyos	1.2	1.0	1.4	17	14	20	0.2	0.1	0.2	2	2	2
Bolivia	Oropeza	1.3	1.1	1.5	46	38	54	0.2	0.1	0.2	7	5	8
Bolivia	Pacajes	1.2	1.0	1.4	9	7	10	0.2	0.1	0.2	1	1	1
Bolivia	Pantaleón Dalence	1.3	1.1	1.5	5	4	5	0.2	0.1	0.2	1	1	1
Bolivia	Pedro Domingo Murillo	1.2	1.0	1.4	224	192	263	0.2	0.1	0.2	32	27	38
Bolivia	Poopó	1.3	1.1	1.5	3	3	4	0.2	0.1	0.2	0	0	1
Bolivia	Punata	1.3	1.0	1.5	8	7	10	0.2	0.1	0.2	1	1	1
Bolivia	Quillacollo	1.3	1.0	1.5	33	27	38	0.2	0.1	0.2	6	5	7
Bolivia	Rafael Bustillo	1.3	1.1	1.6	16	13	19	0.2	0.1	0.2	2	2	3
Bolivia	Sajama	1.2	1.0	1.4	3	2	3	0.2	0.1	0.2	0	0	0
Bolivia	Sara	1.3	1.1	1.6	13	11	16	0.2	0.1	0.2	2	2	2
Bolivia	Saucarí	1.2	1.0	1.4	2	1	2	0.2	0.1	0.2	0	0	0
Bolivia	Sud Chichas	1.2	1.0	1.5	9	7	11	0.2	0.1	0.2	1	1	2
Bolivia	Sud Cinti	1.4	1.1	1.6	5	4	6	0.2	0.1	0.2	1	0	1
Bolivia	Sud Lípez	1.3	1.0	1.5	1	1	1	0.2	0.1	0.2	0	0	0
Bolivia	Sud Yungas	1.3	1.1	1.5	11	9	13	0.2	0.1	0.2	3	2	3
Bolivia	Tapacarí	1.3	1.1	1.6	5	4	6	0.2	0.1	0.2	1	0	1
Bolivia	Tomás Frías	1.3	1.1	1.5	36	30	42	0.2	0.1	0.2	6	5	8
Bolivia	Tomina	1.4	1.2	1.7	8	7	9	0.2	0.1	0.2	1	1	1
Bolivia	Vaca Díez	1.4	1.1	1.6	32	26	38	0.2	0.2	0.2	5	4	6
Bolivia	Vallegrande	1.3	1.1	1.6	6	5	7	0.2	0.1	0.2	1	0	1
Bolivia	Yacuma	1.3	1.1	1.6	7	6	9	0.2	0.1	0.2	1	1	1
Bolivia	Yamparáez	1.4	1.1	1.6	7	5	8	0.2	0.1	0.2	1	1	1
Brazil	Abadia de Goiás	0.2	0.1	0.2	0	0	0	0.1	0.0	0.1	0	0	0
Brazil	Abadia dos Dourados	0.3	0.2	0.4	0	0	0	0.1	0.0	0.1	0	0	0
Brazil	Abadiânia	0.2	0.1	0.2	0	0	0	0.1	0.0	0.1	0	0	0

Table 3: Diarrhoea Mortality by Unit *(continued)*

Country	Unit	2000 mortality mean rate (per 10,000)	2000 mortality lower rate (per 10,000)	2000 mortality upper rate (per 10,000)	2000 mortality mean count	2000 mortality lower count	2000 mortality upper count	2017 mortality mean rate (per 10,000)	2017 mortality lower rate (per 10,000)	2017 mortality upper rate (per 10,000)	2017 mortality mean count	2017 mortality lower count	2017 mortality upper count
Brazil	Abaeté	0.3	0.2	0.4	1	0	1	0.1	0.0	0.1	0	0	0
Brazil	Abaetetuba	0.9	0.7	1.1	12	10	15	0.1	0.1	0.2	2	2	3
Brazil	Abaiara	2.3	1.7	2.9	2	2	3	0.1	0.1	0.2	0	0	0
Brazil	Abaíra	1.7	1.4	2.2	2	1	2	0.2	0.2	0.3	0	0	0
Brazil	Abaré	1.7	1.4	2.2	2	2	3	0.2	0.2	0.3	0	0	1
Brazil	Abatiá	0.3	0.2	0.3	0	0	0	0.1	0.0	0.1	0	0	0
Brazil	Abdon Batista	0.2	0.2	0.3	0	0	0	0.1	0.0	0.1	0	0	0
Brazil	Abel Figueiredo	0.9	0.7	1.1	1	1	1	0.1	0.1	0.2	0	0	0
Brazil	Abelardo Luz	0.2	0.2	0.3	0	0	1	0.1	0.0	0.1	0	0	0
Brazil	Abre Campo	0.3	0.2	0.4	0	0	0	0.1	0.0	0.1	0	0	0
Brazil	Abreu e Lima	2.0	1.6	2.6	15	12	20	0.2	0.2	0.3	2	1	2
Brazil	Abreulândia	0.8	0.7	1.1	0	0	0	0.1	0.1	0.2	0	0	0
Brazil	Acaiaca	0.3	0.2	0.4	0	0	0	0.1	0.0	0.1	0	0	0
Brazil	Açailândia	1.7	1.3	2.2	21	16	27	0.2	0.2	0.3	3	2	4
Brazil	Acajutiba	1.7	1.4	2.2	4	3	5	0.2	0.2	0.3	0	0	1
Brazil	Acará	0.9	0.7	1.1	6	5	8	0.1	0.1	0.2	1	1	1
Brazil	Acarapé	2.3	1.7	2.9	4	3	5	0.1	0.1	0.2	0	0	0
Brazil	Acaraú	2.3	1.7	2.9	14	11	18	0.1	0.1	0.2	1	1	1
Brazil	Acari	1.2	0.9	1.5	1	1	2	0.1	0.1	0.2	0	0	0
Brazil	Acauã	1.4	1.1	1.8	1	1	1	0.2	0.1	0.2	0	0	0
Brazil	Acopiara	2.3	1.7	2.9	11	8	14	0.1	0.1	0.2	1	1	1
Brazil	Acorizal	0.4	0.3	0.5	0	0	0	0.1	0.1	0.1	0	0	0
Brazil	Acrelândia	1.1	0.9	1.3	1	1	1	0.2	0.2	0.3	0	0	0
Brazil	Acreúna	0.2	0.1	0.2	0	0	0	0.1	0.0	0.1	0	0	0
Brazil	Açu	1.2	0.9	1.5	5	4	6	0.1	0.1	0.2	1	0	1
Brazil	Açucena	0.3	0.2	0.4	0	0	0	0.1	0.0	0.1	0	0	0
Brazil	Adamantina	0.2	0.1	0.2	0	0	0	0.0	0.0	0.0	0	0	0
Brazil	Adelândia	0.2	0.1	0.2	0	0	0	0.1	0.0	0.1	0	0	0
Brazil	Adolfo	0.2	0.1	0.2	0	0	0	0.0	0.0	0.0	0	0	0
Brazil	Adrianópolis	0.3	0.2	0.3	0	0	0	0.1	0.0	0.1	0	0	0
Brazil	Adustina	1.7	1.4	2.2	3	3	4	0.2	0.2	0.3	0	0	1
Brazil	Afogados da Ingazeira	2.0	1.6	2.6	8	7	11	0.2	0.2	0.3	1	1	1
Brazil	Afonso Bezerra	1.2	0.9	1.5	1	1	2	0.1	0.1	0.2	0	0	0
Brazil	Afonso Cláudio	0.3	0.2	0.3	1	1	1	0.1	0.1	0.1	0	0	0
Brazil	Afonso Cunha	1.7	1.3	2.2	1	1	2	0.2	0.2	0.3	0	0	0
Brazil	Afrânio	2.0	1.6	2.6	4	3	5	0.2	0.2	0.3	0	0	1
Brazil	Afuá	0.9	0.7	1.1	4	3	5	0.1	0.1	0.2	1	1	1
Brazil	Agrestina	2.0	1.6	2.6	5	4	7	0.2	0.2	0.3	0	0	1
Brazil	Agricolândia	1.4	1.1	1.8	1	0	1	0.2	0.1	0.2	0	0	0
Brazil	Agrolândia	0.2	0.2	0.3	0	0	0	0.1	0.0	0.1	0	0	0
Brazil	Agronômica	0.2	0.2	0.3	0	0	0	0.1	0.0	0.1	0	0	0
Brazil	Água Azul do Norte	0.9	0.7	1.1	2	2	3	0.1	0.1	0.2	0	0	1
Brazil	Água Boa	0.4	0.3	0.5	1	1	1	0.1	0.1	0.1	0	0	0
Brazil	Água Boa	0.3	0.2	0.4	1	0	1	0.1	0.0	0.1	0	0	0
Brazil	Água Branca	2.8	2.2	3.7	8	6	10	0.3	0.2	0.4	1	1	1
Brazil	Água Branca	1.0	0.7	1.2	1	1	1	0.1	0.1	0.1	0	0	0
Brazil	Água Branca	1.4	1.1	1.8	2	2	3	0.2	0.1	0.2	0	0	0
Brazil	Água Clara	0.5	0.3	0.6	0	0	1	0.2	0.1	0.2	0	0	0
Brazil	Água Comprida	0.3	0.2	0.4	0	0	0	0.1	0.0	0.1	0	0	0
Brazil	Água Doce	0.2	0.2	0.3	0	0	0	0.1	0.0	0.1	0	0	0
Brazil	Água Doce do Maranhão	1.7	1.3	2.2	2	2	3	0.2	0.2	0.3	0	0	0
Brazil	Água Doce do Norte	0.3	0.2	0.3	0	0	0	0.1	0.1	0.1	0	0	0
Brazil	Água Fria	1.7	1.4	2.2	3	2	4	0.2	0.2	0.3	0	0	0
Brazil	Água Fria de Goiás	0.2	0.1	0.2	0	0	0	0.1	0.0	0.1	0	0	0
Brazil	Água Limpa	0.2	0.1	0.2	0	0	0	0.1	0.0	0.1	0	0	0

Table 3: Diarrhoea Mortality by Unit *(continued)*

Country	Unit	2000 mortality mean rate (per 10,000)	lower rate (per 10,000)	upper rate (per 10,000)	mean count	lower count	upper count	2017 mortality mean rate (per 10,000)	lower rate (per 10,000)	upper rate (per 10,000)	mean count	lower count	upper count
Brazil	Água Nova	1.2	0.9	1.5	0	0	0	0.1	0.1	0.2	0	0	0
Brazil	Água Preta	2.0	1.6	2.6	15	12	19	0.2	0.2	0.3	1	1	2
Brazil	Água Santa	0.1	0.1	0.2	0	0	0	0.0	0.0	0.0	0	0	0
Brazil	Aguai	0.2	0.1	0.2	0	0	0	0.0	0.0	0.0	0	0	0
Brazil	Aguanil	0.3	0.2	0.4	0	0	0	0.1	0.0	0.1	0	0	0
Brazil	Águas Belas	2.0	1.6	2.6	10	8	14	0.2	0.2	0.3	1	1	1
Brazil	Águas da Prata	0.2	0.1	0.2	0	0	0	0.0	0.0	0.0	0	0	0
Brazil	Águas de Chapecó	0.2	0.2	0.3	0	0	0	0.1	0.0	0.1	0	0	0
Brazil	Águas de Lindóia	0.2	0.1	0.2	0	0	0	0.0	0.0	0.0	0	0	0
Brazil	Águas de Santa Bárbara	0.2	0.1	0.2	0	0	0	0.0	0.0	0.0	0	0	0
Brazil	Águas de São Pedro	0.2	0.1	0.2	0	0	0	0.0	0.0	0.0	0	0	0
Brazil	Águas Formosas	0.3	0.2	0.4	0	0	1	0.1	0.0	0.1	0	0	0
Brazil	Águas Frias	0.2	0.2	0.3	0	0	0	0.1	0.0	0.1	0	0	0
Brazil	Águas Lindas de Goiás	0.2	0.1	0.2	2	2	3	0.1	0.0	0.1	1	1	1
Brazil	Águas Mornas	0.2	0.2	0.3	0	0	0	0.1	0.0	0.1	0	0	0
Brazil	Águas Vermelhas	0.3	0.2	0.4	0	0	1	0.1	0.0	0.1	0	0	0
Brazil	Agudo	0.1	0.1	0.2	0	0	0	0.0	0.0	0.0	0	0	0
Brazil	Agudos	0.2	0.1	0.2	0	0	1	0.0	0.0	0.0	0	0	0
Brazil	Agudos do Sul	0.3	0.2	0.3	0	0	0	0.1	0.0	0.1	0	0	0
Brazil	Aguiar	1.0	0.7	1.2	0	0	0	0.1	0.1	0.1	0	0	0
Brazil	Aguiarnópolis	0.8	0.7	1.1	0	0	0	0.1	0.1	0.2	0	0	0
Brazil	Aimorés	0.3	0.2	0.4	1	0	1	0.1	0.0	0.1	0	0	0
Brazil	Aiquara	1.7	1.4	2.2	2	2	2	0.2	0.2	0.3	0	0	0
Brazil	Aiuaba	2.3	1.7	2.9	4	3	5	0.1	0.1	0.2	0	0	0
Brazil	Aiuruoca	0.3	0.2	0.4	0	0	0	0.1	0.0	0.1	0	0	0
Brazil	Ajuricaba	0.1	0.1	0.2	0	0	0	0.0	0.0	0.0	0	0	0
Brazil	Alagoa	0.3	0.2	0.4	0	0	0	0.1	0.0	0.1	0	0	0
Brazil	Alagoa Grande	1.0	0.7	1.2	3	3	4	0.1	0.1	0.1	0	0	0
Brazil	Alagoa Nova	1.0	0.7	1.2	2	2	3	0.1	0.1	0.1	0	0	0
Brazil	Alagoinha	1.0	0.7	1.2	1	1	2	0.1	0.1	0.1	0	0	0
Brazil	Alagoinha	2.0	1.6	2.6	3	2	3	0.2	0.2	0.3	0	0	0
Brazil	Alagoinha do Piauí	1.4	1.1	1.8	1	1	1	0.2	0.1	0.2	0	0	0
Brazil	Alagoinhas	1.7	1.4	2.2	24	19	31	0.2	0.2	0.3	3	2	4
Brazil	Alambari	0.2	0.1	0.2	0	0	0	0.0	0.0	0.0	0	0	0
Brazil	Albertina	0.3	0.2	0.4	0	0	0	0.1	0.0	0.1	0	0	0
Brazil	Alcântara	1.7	1.3	2.2	5	4	6	0.2	0.2	0.3	1	0	1
Brazil	Alcântaras	2.3	1.7	2.9	3	2	3	0.1	0.1	0.2	0	0	0
Brazil	Alcantil	1.0	0.7	1.2	1	0	1	0.1	0.1	0.1	0	0	0
Brazil	Alcinópolis	0.5	0.3	0.6	0	0	0	0.2	0.1	0.2	0	0	0
Brazil	Alcobaca	1.7	1.4	2.2	3	2	3	0.2	0.2	0.3	0	0	0
Brazil	Aldeias Altas	1.7	1.3	2.2	4	3	5	0.2	0.2	0.3	1	1	1
Brazil	Alecrim	0.1	0.1	0.2	0	0	0	0.0	0.0	0.0	0	0	0
Brazil	Alegre	0.3	0.2	0.3	1	1	1	0.1	0.1	0.1	0	0	0
Brazil	Alegrete	0.1	0.1	0.2	1	1	1	0.0	0.0	0.0	0	0	0
Brazil	Alegrete do Piauí	1.4	1.1	1.8	1	1	1	0.2	0.1	0.2	0	0	0
Brazil	Alegria	0.1	0.1	0.2	0	0	0	0.0	0.0	0.0	0	0	0
Brazil	Além Paraíba	0.3	0.2	0.4	0	0	1	0.1	0.0	0.1	0	0	0
Brazil	Alenquer	0.9	0.7	1.1	4	3	5	0.1	0.1	0.2	1	1	1
Brazil	Alexandria	1.2	0.9	1.5	2	1	2	0.1	0.1	0.2	0	0	0
Brazil	Alexania	0.2	0.1	0.2	0	0	0	0.1	0.0	0.1	0	0	0
Brazil	Alexânia	0.2	0.1	0.2	0	0	0	0.1	0.0	0.1	0	0	0
Brazil	Alfenas	0.3	0.2	0.4	2	1	2	0.1	0.0	0.1	0	0	0
Brazil	Alfredo Chaves	0.3	0.2	0.3	0	0	0	0.1	0.1	0.1	0	0	0
Brazil	Alfredo Marconde	0.2	0.1	0.2	0	0	0	0.0	0.0	0.0	0	0	0
Brazil	Alfredo Vasconcelos	0.3	0.2	0.4	0	0	0	0.1	0.0	0.1	0	0	0

Table 3: Diarrhoea Mortality by Unit *(continued)*

Country	Unit	2000 mortality mean rate (per 10,000)	lower rate (per 10,000)	upper rate (per 10,000)	mean count	lower count	upper count	2017 mortality mean rate (per 10,000)	lower rate (per 10,000)	upper rate (per 10,000)	mean count	lower count	upper count
Brazil	Alfredo Wagner	0.2	0.2	0.3	0	0	0	0.1	0.0	0.1	0	0	0
Brazil	Algodão de Jandaíra	1.0	0.7	1.2	0	0	0	0.1	0.1	0.1	0	0	0
Brazil	Alhandra	1.0	0.7	1.2	2	2	2	0.1	0.1	0.1	0	0	0
Brazil	Aliança	2.0	1.6	2.6	10	8	13	0.2	0.2	0.3	1	1	1
Brazil	Aliança do Tocantins	0.8	0.7	1.1	1	0	1	0.1	0.1	0.2	0	0	0
Brazil	Almadina	1.7	1.4	2.2	1	1	2	0.2	0.2	0.3	0	0	0
Brazil	Almas	0.8	0.7	1.1	1	1	1	0.1	0.1	0.2	0	0	0
Brazil	Almenara	0.3	0.2	0.4	1	1	1	0.1	0.0	0.1	0	0	0
Brazil	Almerim	0.9	0.7	1.1	5	4	7	0.1	0.1	0.2	1	1	1
Brazil	Almino Afonso	1.2	0.9	1.5	1	1	1	0.1	0.1	0.2	0	0	0
Brazil	Almirante Tamandaré	0.3	0.2	0.3	2	2	3	0.1	0.0	0.1	0	0	1
Brazil	Aloândia	0.2	0.1	0.2	0	0	0	0.1	0.0	0.1	0	0	0
Brazil	Alpercata	0.3	0.2	0.4	0	0	0	0.1	0.0	0.1	0	0	0
Brazil	Alpestre	0.1	0.1	0.2	0	0	0	0.0	0.0	0.0	0	0	0
Brazil	Alpinópolis	0.3	0.2	0.4	0	0	1	0.1	0.0	0.1	0	0	0
Brazil	Alta Floresta	0.4	0.3	0.5	2	2	3	0.1	0.1	0.1	0	0	1
Brazil	Alta Floresta d'Oeste	0.4	0.3	0.5	1	1	1	0.1	0.1	0.1	0	0	0
Brazil	Altair	0.2	0.1	0.2	0	0	0	0.0	0.0	0.0	0	0	0
Brazil	Altamira	0.9	0.7	1.1	8	6	10	0.1	0.1	0.2	2	1	2
Brazil	Altamira do Maranhão	1.7	1.3	2.2	2	2	3	0.2	0.2	0.3	0	0	0
Brazil	Altamira do Paran	0.3	0.2	0.3	0	0	0	0.1	0.0	0.1	0	0	0
Brazil	Altaneira	2.3	1.7	2.9	1	1	1	0.1	0.1	0.2	0	0	0
Brazil	Alterosa	0.3	0.2	0.4	0	0	0	0.1	0.0	0.1	0	0	0
Brazil	Altinho	2.0	1.6	2.6	6	5	8	0.2	0.2	0.3	0	0	1
Brazil	Altinópolis	0.2	0.1	0.2	0	0	0	0.0	0.0	0.0	0	0	0
Brazil	Alto Alegre	0.7	0.6	0.9	2	1	2	0.2	0.2	0.3	0	0	0
Brazil	Alto Alegre	0.2	0.1	0.2	0	0	0	0.0	0.0	0.0	0	0	0
Brazil	Alto Alegre	0.1	0.1	0.2	0	0	0	0.0	0.0	0.0	0	0	0
Brazil	Alto Alegre do Maranho	1.7	1.3	2.2	5	4	6	0.2	0.2	0.3	1	1	1
Brazil	Alto Alegre do Pindaré	1.7	1.3	2.2	7	5	9	0.2	0.2	0.3	1	1	1
Brazil	Alto Alegre dos Parecis	0.4	0.3	0.5	1	0	1	0.1	0.1	0.1	0	0	0
Brazil	Alto Araguaia	0.4	0.3	0.5	0	0	1	0.1	0.1	0.1	0	0	0
Brazil	Alto Bela Vista	0.2	0.2	0.3	0	0	0	0.1	0.0	0.1	0	0	0
Brazil	Alto Boa Vista	0.4	0.3	0.5	0	0	0	0.1	0.1	0.1	0	0	0
Brazil	Alto Caparaó	0.3	0.2	0.4	0	0	0	0.1	0.0	0.1	0	0	0
Brazil	Alto do Rodrigues	1.2	0.9	1.5	1	1	2	0.1	0.1	0.2	0	0	0
Brazil	Alto Feliz	0.1	0.1	0.2	0	0	0	0.0	0.0	0.0	0	0	0
Brazil	Alto Garças	0.4	0.3	0.5	0	0	0	0.1	0.1	0.1	0	0	0
Brazil	Alto Horizonte	0.2	0.1	0.2	0	0	0	0.1	0.0	0.1	0	0	0
Brazil	Alto Jequitibá	0.3	0.2	0.4	0	0	0	0.1	0.0	0.1	0	0	0
Brazil	Alto Longá	1.4	1.1	1.8	2	1	2	0.2	0.1	0.2	0	0	0
Brazil	Alto Paraguai	0.4	0.3	0.5	0	0	0	0.1	0.1	0.1	0	0	0
Brazil	Alto Paraíso	0.4	0.3	0.5	1	0	1	0.1	0.1	0.1	0	0	0
Brazil	Alto Paraíso de Goiás	0.2	0.1	0.2	0	0	0	0.1	0.0	0.1	0	0	0
Brazil	Alto Paraná	0.3	0.2	0.3	0	0	0	0.1	0.0	0.1	0	0	0
Brazil	Alto Parnaiba	1.7	1.3	2.2	2	2	3	0.2	0.2	0.3	0	0	0
Brazil	Alto Piquiri	0.3	0.2	0.3	0	0	0	0.1	0.0	0.1	0	0	0
Brazil	Alto Rio doce	0.3	0.2	0.4	0	0	0	0.1	0.0	0.1	0	0	0
Brazil	Alto Rio Novo	0.3	0.2	0.3	0	0	0	0.1	0.1	0.1	0	0	0

Table 3: Diarrhoea Mortality by Unit *(continued)*

Country	Unit	2000 mortality mean rate (per 10,000)	2000 mortality lower rate (per 10,000)	2000 mortality upper rate (per 10,000)	2000 mortality mean count	2000 mortality lower count	2000 mortality upper count	2017 mortality mean rate (per 10,000)	2017 mortality lower rate (per 10,000)	2017 mortality upper rate (per 10,000)	2017 mortality mean count	2017 mortality lower count	2017 mortality upper count
Brazil	Alto Santo	2.3	1.7	2.9	4	3	5	0.1	0.1	0.2	0	0	0
Brazil	Alto Taquari	0.4	0.3	0.5	0	0	0	0.1	0.1	0.1	0	0	0
Brazil	Altônia	0.3	0.2	0.3	0	0	1	0.1	0.0	0.1	0	0	0
Brazil	Altos	1.4	1.1	1.8	7	5	9	0.2	0.1	0.2	1	1	1
Brazil	Alumínio	0.2	0.1	0.2	0	0	0	0.0	0.0	0.0	0	0	0
Brazil	Alvarães	0.8	0.6	0.9	1	1	1	0.2	0.2	0.3	0	0	0
Brazil	Alvarenga	0.3	0.2	0.4	0	0	0	0.1	0.0	0.1	0	0	0
Brazil	Álvares Florence	0.2	0.1	0.2	0	0	0	0.0	0.0	0.0	0	0	0
Brazil	Álvares Machado	0.2	0.1	0.2	0	0	1	0.0	0.0	0.0	0	0	0
Brazil	Álvaro de Carvalho	0.2	0.1	0.2	0	0	0	0.0	0.0	0.0	0	0	0
Brazil	Alvinlândia	0.2	0.1	0.2	0	0	0	0.0	0.0	0.0	0	0	0
Brazil	Alvinópolis	0.3	0.2	0.4	0	0	0	0.1	0.0	0.1	0	0	0
Brazil	Alvorada	0.1	0.1	0.2	2	2	3	0.0	0.0	0.0	0	0	1
Brazil	Alvorada	0.8	0.7	1.1	1	1	1	0.1	0.1	0.2	0	0	0
Brazil	Alvorada d'Oeste	0.4	0.3	0.5	1	1	1	0.1	0.1	0.1	0	0	0
Brazil	Alvorada de Minas	0.3	0.2	0.4	0	0	0	0.1	0.0	0.1	0	0	0
Brazil	Alvorada do Gurguéia	1.4	1.1	1.8	1	1	1	0.2	0.1	0.2	0	0	0
Brazil	Alvorada do Norte	0.2	0.1	0.2	0	0	0	0.1	0.0	0.1	0	0	0
Brazil	Alvorada do Sul	0.3	0.2	0.3	0	0	0	0.1	0.0	0.1	0	0	0
Brazil	Amajari	0.7	0.6	0.9	1	0	1	0.2	0.2	0.3	0	0	0
Brazil	Amambai	0.5	0.3	0.6	2	1	2	0.2	0.1	0.2	1	0	1
Brazil	Amapá	0.2	0.2	0.3	0	0	0	0.1	0.1	0.2	0	0	0
Brazil	Amapá do Maranho	1.7	1.3	2.2	1	1	2	0.2	0.2	0.3	0	0	0
Brazil	Amapora	0.3	0.2	0.3	0	0	0	0.1	0.0	0.1	0	0	0
Brazil	Amaraji	2.0	1.6	2.6	6	5	8	0.2	0.2	0.3	0	0	1
Brazil	Amaral Ferrador	0.1	0.1	0.2	0	0	0	0.0	0.0	0.0	0	0	0
Brazil	Amaralina	0.2	0.1	0.2	0	0	0	0.1	0.0	0.1	0	0	0
Brazil	Amarante	1.4	1.1	1.8	3	3	4	0.2	0.1	0.2	0	0	0
Brazil	Amarante do Maranhão	1.7	1.3	2.2	10	7	13	0.2	0.2	0.3	1	1	2
Brazil	Amargosa	1.7	1.4	2.2	6	4	7	0.2	0.2	0.3	1	0	1
Brazil	Amaturá	0.8	0.6	0.9	1	1	1	0.2	0.2	0.3	0	0	0
Brazil	Amélia Rodrigues	1.7	1.4	2.2	4	3	5	0.2	0.2	0.3	0	0	1
Brazil	América dourada	1.7	1.4	2.2	3	2	4	0.2	0.2	0.3	0	0	0
Brazil	Americana	0.2	0.1	0.2	2	1	2	0.0	0.0	0.0	0	0	1
Brazil	Americano do Brazil	0.2	0.1	0.2	0	0	0	0.1	0.0	0.1	0	0	0
Brazil	Américo Brasiliense	0.2	0.1	0.2	0	0	0	0.0	0.0	0.0	0	0	0
Brazil	Américo de Campos	0.2	0.1	0.2	1	0	1	0.0	0.0	0.0	0	0	0
Brazil	Ametista do Sul	0.1	0.1	0.2	0	0	0	0.0	0.0	0.0	0	0	0
Brazil	Amontada	2.3	1.7	2.9	9	7	12	0.1	0.1	0.2	1	1	1
Brazil	Amorinópolis	0.2	0.1	0.2	0	0	0	0.1	0.0	0.1	0	0	0
Brazil	Amparo	0.2	0.1	0.2	0	0	0	0.0	0.0	0.0	0	0	0
Brazil	Amparo	1.0	0.7	1.2	0	0	0	0.1	0.1	0.1	0	0	0
Brazil	Amparo de São Francisco	1.3	1.0	1.7	0	0	0	0.2	0.1	0.3	0	0	0
Brazil	Amparo do Serra	0.3	0.2	0.4	0	0	0	0.1	0.0	0.1	0	0	0
Brazil	Ampére	0.3	0.2	0.3	0	0	1	0.1	0.0	0.1	0	0	0
Brazil	Anadia	2.8	2.2	3.7	7	5	9	0.3	0.2	0.4	1	0	1
Brazil	Anagé	1.7	1.4	2.2	6	5	7	0.2	0.2	0.3	0	0	1
Brazil	Anahy	0.3	0.2	0.3	0	0	0	0.1	0.0	0.1	0	0	0
Brazil	Anajas	0.9	0.7	1.1	2	2	3	0.1	0.1	0.2	1	0	1
Brazil	Anajatuba	1.7	1.3	2.2	6	5	8	0.2	0.2	0.3	1	1	1

Table 3: Diarrhoea Mortality by Unit *(continued)*

Country	Unit	2000 mortality mean rate (per 10,000)	lower rate (per 10,000)	upper rate (per 10,000)	mean count	lower count	upper count	2017 mortality mean rate (per 10,000)	lower rate (per 10,000)	upper rate (per 10,000)	mean count	lower count	upper count
Brazil	Analandia	0.2	0.1	0.2	0	0	0	0.0	0.0	0.0	0	0	0
Brazil	Anamã	0.8	0.6	0.9	1	0	1	0.2	0.2	0.3	0	0	0
Brazil	Ananás	0.8	0.7	1.1	1	1	2	0.1	0.1	0.2	0	0	0
Brazil	Ananindeua	0.9	0.7	1.1	35	27	43	0.1	0.1	0.2	5	4	7
Brazil	Anápolis	0.2	0.1	0.2	5	4	6	0.1	0.0	0.1	1	1	2
Brazil	Anapu	0.9	0.7	1.1	1	1	1	0.1	0.1	0.2	1	0	1
Brazil	Anapuros	1.7	1.3	2.2	3	2	4	0.2	0.2	0.3	1	0	1
Brazil	Anastácio	0.5	0.3	0.6	1	1	2	0.2	0.1	0.2	0	0	0
Brazil	Anaurilândia	0.5	0.3	0.6	0	0	0	0.2	0.1	0.2	0	0	0
Brazil	Anchieta	0.2	0.2	0.3	0	0	0	0.1	0.0	0.1	0	0	0
Brazil	Anchieta	0.3	0.2	0.3	0	0	0	0.1	0.1	0.1	0	0	0
Brazil	Andarai	1.7	1.4	2.2	3	2	4	0.2	0.2	0.3	0	0	0
Brazil	Andira	0.3	0.2	0.3	0	0	1	0.1	0.0	0.1	0	0	0
Brazil	Andirá	0.3	0.2	0.3	0	0	0	0.1	0.0	0.1	0	0	0
Brazil	Andorinha	1.7	1.4	2.2	3	2	4	0.2	0.2	0.3	0	0	0
Brazil	Andradas	0.3	0.2	0.4	1	1	1	0.1	0.0	0.1	0	0	0
Brazil	Andradina	0.2	0.1	0.2	1	0	1	0.0	0.0	0.0	0	0	0
Brazil	André da Rocha	0.1	0.1	0.2	0	0	0	0.0	0.0	0.0	0	0	0
Brazil	Andrelândia	0.3	0.2	0.4	0	0	0	0.1	0.0	0.1	0	0	0
Brazil	Angatuba	0.2	0.1	0.2	0	0	0	0.0	0.0	0.0	0	0	0
Brazil	Angelândia	0.3	0.2	0.4	0	0	0	0.1	0.0	0.1	0	0	0
Brazil	Angélica	0.5	0.3	0.6	0	0	0	0.2	0.1	0.2	0	0	0
Brazil	Angelim	2.0	1.6	2.6	2	2	3	0.2	0.2	0.3	0	0	0
Brazil	Angelina	0.2	0.2	0.3	0	0	0	0.1	0.0	0.1	0	0	0
Brazil	Angical	1.7	1.4	2.2	3	3	4	0.2	0.2	0.3	0	0	0
Brazil	Angical do Piauí	1.4	1.1	1.8	1	1	2	0.2	0.1	0.2	0	0	0
Brazil	Angico	0.8	0.7	1.1	0	0	0	0.1	0.1	0.2	0	0	0
Brazil	Angicos	1.2	0.9	1.5	2	1	2	0.1	0.1	0.2	0	0	0
Brazil	Angra dos Reis	0.2	0.1	0.3	1	1	2	0.1	0.0	0.1	0	0	1
Brazil	Anguera	1.7	1.4	2.2	1	1	2	0.2	0.2	0.3	0	0	0
Brazil	Ângulo	0.3	0.2	0.3	0	0	0	0.1	0.0	0.1	0	0	0
Brazil	Anhanguera	0.2	0.1	0.2	0	0	0	0.1	0.0	0.1	0	0	0
Brazil	Anhembi	0.2	0.1	0.2	0	0	0	0.0	0.0	0.0	0	0	0
Brazil	Anhumas	0.2	0.1	0.2	0	0	0	0.0	0.0	0.0	0	0	0
Brazil	Anicuns	0.2	0.1	0.2	0	0	0	0.1	0.0	0.1	0	0	0
Brazil	Anísio de Abreu	1.4	1.1	1.8	1	1	1	0.2	0.1	0.2	0	0	0
Brazil	Anita Garibaldi	0.2	0.2	0.3	0	0	0	0.1	0.0	0.1	0	0	0
Brazil	Anitápolis	0.2	0.2	0.3	0	0	0	0.1	0.0	0.1	0	0	0
Brazil	Anori	0.8	0.6	0.9	1	1	1	0.2	0.2	0.3	1	0	1
Brazil	Anta Gorda	0.1	0.1	0.2	0	0	0	0.0	0.0	0.0	0	0	0
Brazil	Antas	1.7	1.4	2.2	4	3	5	0.2	0.2	0.3	1	0	1
Brazil	Antonina	0.3	0.2	0.3	0	0	0	0.1	0.0	0.1	0	0	0
Brazil	Antonina do Norte	2.3	1.7	2.9	2	1	2	0.1	0.1	0.2	0	0	0
Brazil	Antônio Almeida	1.4	1.1	1.8	0	0	1	0.2	0.1	0.2	0	0	0
Brazil	Antônio Cardoso	1.7	1.4	2.2	2	2	3	0.2	0.2	0.3	0	0	0
Brazil	Antônio Carlos	0.2	0.2	0.3	0	0	0	0.1	0.0	0.1	0	0	0
Brazil	Antônio Carlos	0.3	0.2	0.4	0	0	0	0.1	0.0	0.1	0	0	0
Brazil	Antônio Dias	0.3	0.2	0.4	0	0	0	0.1	0.0	0.1	0	0	0
Brazil	Antônio Gonçalves	1.7	1.4	2.2	5	4	6	0.2	0.2	0.3	1	0	1
Brazil	Antônio João	0.5	0.3	0.6	0	0	1	0.2	0.1	0.2	0	0	0
Brazil	Antônio Martins	1.2	0.9	1.5	1	1	1	0.1	0.1	0.2	0	0	0
Brazil	Antonio Olinto	0.3	0.2	0.3	0	0	0	0.1	0.0	0.1	0	0	0
Brazil	Antônio Prado	0.1	0.1	0.2	0	0	0	0.0	0.0	0.0	0	0	0
Brazil	Antonio Prado de Minas	0.3	0.2	0.4	0	0	0	0.1	0.0	0.1	0	0	0
Brazil	Aparecida	1.0	0.7	1.2	1	0	1	0.1	0.1	0.1	0	0	0
Brazil	Aparecida	0.2	0.1	0.2	1	0	1	0.0	0.0	0.0	0	0	0
Brazil	Aparecida de Goiânia	0.2	0.1	0.2	7	5	9	0.1	0.0	0.1	2	2	3

Table 3: Diarrhoea Mortality by Unit *(continued)*

Country	Unit	2000 mortality mean rate (per 10,000)	lower rate (per 10,000)	upper rate (per 10,000)	mean count	lower count	upper count	2017 mortality mean rate (per 10,000)	lower rate (per 10,000)	upper rate (per 10,000)	mean count	lower count	upper count
Brazil	Aparecida do Rio doce	0.2	0.1	0.2	0	0	0	0.1	0.0	0.1	0	0	0
Brazil	Aparecida do Rio Negro	0.8	0.7	1.1	0	0	0	0.1	0.1	0.2	0	0	0
Brazil	Aparecida do Taboado	0.5	0.3	0.6	1	1	1	0.2	0.1	0.2	0	0	0
Brazil	Aparecida doeste	0.2	0.1	0.2	0	0	0	0.0	0.0	0.0	0	0	0
Brazil	Aperibé	0.2	0.1	0.3	0	0	0	0.1	0.0	0.1	0	0	0
Brazil	Apiacá	0.3	0.2	0.3	0	0	0	0.1	0.1	0.1	0	0	0
Brazil	Apiacás	0.4	0.3	0.5	0	0	0	0.1	0.1	0.1	0	0	0
Brazil	Apiaí	0.2	0.1	0.2	0	0	1	0.0	0.0	0.0	0	0	0
Brazil	Apicum-Açu	1.7	1.3	2.2	3	2	4	0.2	0.2	0.3	0	0	1
Brazil	Apiúna	0.2	0.2	0.3	0	0	0	0.1	0.0	0.1	0	0	0
Brazil	Apodi	1.2	0.9	1.5	4	3	6	0.1	0.1	0.2	0	0	1
Brazil	Aporá	1.7	1.4	2.2	4	3	5	0.2	0.2	0.3	0	0	1
Brazil	Aporé	0.2	0.1	0.2	0	0	0	0.1	0.0	0.1	0	0	0
Brazil	Apuarema	1.7	1.4	2.2	2	1	2	0.2	0.2	0.3	0	0	0
Brazil	Apucarana	0.3	0.2	0.3	3	2	3	0.1	0.0	0.1	0	0	1
Brazil	Apuí	0.8	0.6	0.9	1	1	2	0.2	0.2	0.3	1	0	1
Brazil	Apuiarés	2.3	1.7	2.9	4	3	5	0.1	0.1	0.2	0	0	0
Brazil	Aquidabã	1.3	1.0	1.7	3	2	3	0.2	0.1	0.3	0	0	0
Brazil	Aquidauana	0.5	0.3	0.6	2	1	3	0.2	0.1	0.2	1	0	1
Brazil	Aquiraz	2.3	1.7	2.9	19	15	25	0.1	0.1	0.2	2	1	2
Brazil	Arabutã	0.2	0.2	0.3	0	0	0	0.1	0.0	0.1	0	0	0
Brazil	Aracagi	1.0	0.7	1.2	2	1	2	0.1	0.1	0.1	0	0	0
Brazil	Aracai	0.3	0.2	0.4	0	0	0	0.1	0.0	0.1	0	0	0
Brazil	Aracaju	1.3	1.0	1.7	53	39	68	0.2	0.1	0.3	9	6	12
Brazil	Araçariguama	0.2	0.1	0.2	0	0	0	0.0	0.0	0.0	0	0	0
Brazil	Araças	1.7	1.4	2.2	2	1	2	0.2	0.2	0.3	0	0	0
Brazil	Aracati	2.3	1.7	2.9	15	12	19	0.1	0.1	0.2	1	1	1
Brazil	Aracatu	1.7	1.4	2.2	3	2	4	0.2	0.2	0.3	0	0	0
Brazil	Araçatuba	0.2	0.1	0.2	2	1	2	0.0	0.0	0.0	0	0	0
Brazil	Araci	1.7	1.4	2.2	13	10	17	0.2	0.2	0.3	1	1	2
Brazil	Aracitaba	0.3	0.2	0.4	0	0	0	0.1	0.0	0.1	0	0	0
Brazil	Araçoiaba	2.0	1.6	2.6	3	3	4	0.2	0.2	0.3	0	0	0
Brazil	Araçoiaba	2.3	1.7	2.9	7	5	9	0.1	0.1	0.2	0	0	0
Brazil	Araçoiaba da Serra	0.2	0.1	0.2	0	0	0	0.0	0.0	0.0	0	0	0
Brazil	Aracruz	0.3	0.2	0.3	2	1	2	0.1	0.1	0.1	1	1	1
Brazil	Araçu	0.2	0.1	0.2	0	0	0	0.1	0.0	0.1	0	0	0
Brazil	Araçuaí	0.3	0.2	0.4	1	1	1	0.1	0.0	0.1	0	0	0
Brazil	Aragarças	0.2	0.1	0.2	0	0	0	0.1	0.0	0.1	0	0	0
Brazil	Aragoiânia	0.2	0.1	0.2	0	0	0	0.1	0.0	0.1	0	0	0
Brazil	Aragominas	0.8	0.7	1.1	1	0	1	0.1	0.1	0.2	0	0	0
Brazil	Araguacema	0.8	0.7	1.1	1	0	1	0.1	0.1	0.2	0	0	0
Brazil	Araguaçu	0.8	0.7	1.1	1	1	1	0.1	0.1	0.2	0	0	0
Brazil	Araguaiana	0.4	0.3	0.5	0	0	0	0.1	0.1	0.1	0	0	0
Brazil	Araguaína	0.8	0.7	1.1	12	9	15	0.1	0.1	0.2	2	2	3
Brazil	Araguainha	0.4	0.3	0.5	0	0	0	0.1	0.1	0.1	0	0	0
Brazil	Araguanã	0.8	0.7	1.1	0	0	0	0.1	0.1	0.2	0	0	0
Brazil	Araguanã	1.7	1.3	2.2	2	1	3	0.2	0.2	0.3	0	0	1
Brazil	Araguapaz	0.2	0.1	0.2	0	0	0	0.1	0.0	0.1	0	0	0
Brazil	Araguari	0.3	0.2	0.4	2	2	3	0.1	0.0	0.1	0	0	1
Brazil	Araguatins	0.8	0.7	1.1	3	2	4	0.1	0.1	0.2	0	0	1
Brazil	Araioses	1.7	1.3	2.2	9	7	11	0.2	0.2	0.3	1	1	2
Brazil	Aral Moreira	0.5	0.3	0.6	0	0	1	0.2	0.1	0.2	0	0	0
Brazil	Aramari	1.7	1.4	2.2	3	2	3	0.2	0.2	0.3	0	0	0
Brazil	Arambaré	0.1	0.1	0.2	0	0	0	0.0	0.0	0.0	0	0	0
Brazil	Arame	1.7	1.3	2.2	6	4	7	0.2	0.2	0.3	1	1	1
Brazil	Aramina	0.2	0.1	0.2	0	0	0	0.0	0.0	0.0	0	0	0

Table 3: Diarrhoea Mortality by Unit *(continued)*

Country	Unit	2000 mortality mean rate (per 10,000)	lower rate (per 10,000)	upper rate (per 10,000)	mean count	lower count	upper count	2017 mortality mean rate (per 10,000)	lower rate (per 10,000)	upper rate (per 10,000)	mean count	lower count	upper count
Brazil	Arandu	0.2	0.1	0.2	0	0	0	0.0	0.0	0.0	0	0	0
Brazil	Arantina	0.3	0.2	0.4	0	0	0	0.1	0.0	0.1	0	0	0
Brazil	Arapeí	0.2	0.1	0.2	0	0	0	0.0	0.0	0.0	0	0	0
Brazil	Arapiraca	2.8	2.2	3.7	67	52	88	0.3	0.2	0.4	8	6	10
Brazil	Arapoema	0.8	0.7	1.1	1	1	1	0.1	0.1	0.2	0	0	0
Brazil	Araponga	0.3	0.2	0.4	0	0	0	0.1	0.0	0.1	0	0	0
Brazil	Arapongas	0.3	0.2	0.3	2	2	3	0.1	0.0	0.1	0	0	1
Brazil	Araporã	0.2	0.1	0.2	0	0	0	0.1	0.0	0.1	0	0	0
Brazil	Arapoti	0.3	0.2	0.3	1	0	1	0.1	0.0	0.1	0	0	0
Brazil	Arapu	0.3	0.2	0.3	0	0	0	0.1	0.0	0.1	0	0	0
Brazil	Arapuá	0.3	0.2	0.4	0	0	0	0.1	0.0	0.1	0	0	0
Brazil	Araputanga	0.4	0.3	0.5	1	0	1	0.1	0.1	0.1	0	0	0
Brazil	Araquari	0.2	0.2	0.3	1	1	2	0.1	0.0	0.1	0	0	0
Brazil	Arara	1.0	0.7	1.2	1	1	1	0.1	0.1	0.1	0	0	0
Brazil	Araranguá	0.2	0.2	0.3	1	1	2	0.1	0.0	0.1	0	0	0
Brazil	Araraquara	0.2	0.1	0.2	2	1	2	0.0	0.0	0.0	0	0	1
Brazil	Araras	0.2	0.1	0.2	1	1	2	0.0	0.0	0.0	0	0	0
Brazil	Ararendá	2.3	1.7	2.9	3	2	3	0.1	0.1	0.2	0	0	0
Brazil	Arari	1.7	1.3	2.2	6	5	8	0.2	0.2	0.3	1	1	1
Brazil	Araricá	0.1	0.1	0.2	0	0	0	0.0	0.0	0.0	0	0	0
Brazil	Araripe	2.3	1.7	2.9	6	5	8	0.1	0.1	0.2	0	0	1
Brazil	Araripina	2.0	1.6	2.6	23	18	29	0.2	0.2	0.3	2	2	3
Brazil	Araruama	0.2	0.1	0.3	1	1	2	0.1	0.0	0.1	0	0	1
Brazil	Araruna	1.0	0.7	1.2	2	2	3	0.1	0.1	0.1	0	0	0
Brazil	Araruna	0.3	0.2	0.3	0	0	0	0.1	0.0	0.1	0	0	0
Brazil	Arataca	1.7	1.4	2.2	2	2	3	0.2	0.2	0.3	0	0	0
Brazil	Aratiba	0.1	0.1	0.2	0	0	0	0.0	0.0	0.0	0	0	0
Brazil	Aratuba	2.3	1.7	2.9	3	3	4	0.1	0.1	0.2	0	0	0
Brazil	Aratuipe	1.7	1.4	2.2	1	1	1	0.2	0.2	0.3	0	0	0
Brazil	Aratuípe	1.7	1.4	2.2	1	1	1	0.2	0.2	0.3	0	0	0
Brazil	Arauá	1.3	1.0	1.7	2	1	2	0.2	0.1	0.3	0	0	0
Brazil	Araucária	0.3	0.2	0.3	3	2	3	0.1	0.0	0.1	1	0	1
Brazil	Araujos	0.3	0.2	0.4	0	0	0	0.1	0.0	0.1	0	0	0
Brazil	Araújos	0.3	0.2	0.3	0	0	0	0.1	0.0	0.1	0	0	0
Brazil	Araxá	0.3	0.2	0.4	1	1	2	0.1	0.0	0.1	0	0	0
Brazil	Arceburgo	0.3	0.2	0.4	0	0	0	0.1	0.0	0.1	0	0	0
Brazil	Arco-íris	0.2	0.1	0.2	0	0	0	0.0	0.0	0.0	0	0	0
Brazil	Arcos	0.3	0.2	0.4	1	1	1	0.1	0.0	0.1	0	0	0
Brazil	Arcoverde	2.0	1.6	2.6	15	12	19	0.2	0.2	0.3	2	1	2
Brazil	Areado	0.3	0.2	0.4	0	0	0	0.1	0.0	0.1	0	0	0
Brazil	Areal	0.2	0.1	0.3	0	0	0	0.1	0.0	0.1	0	0	0
Brazil	Arealva	0.2	0.1	0.2	0	0	0	0.0	0.0	0.0	0	0	0
Brazil	Areia	1.0	0.7	1.2	4	3	5	0.1	0.1	0.1	0	0	0
Brazil	Areia Branca	1.2	0.9	1.5	2	2	3	0.1	0.1	0.2	0	0	0
Brazil	Areia Branca	1.3	1.0	1.7	2	2	3	0.2	0.1	0.3	0	0	0
Brazil	Areia de Baraúnas	1.0	0.7	1.2	0	0	0	0.1	0.1	0.1	0	0	0
Brazil	Areial	1.0	0.7	1.2	0	0	1	0.1	0.1	0.1	0	0	0
Brazil	Areias	0.2	0.1	0.2	0	0	0	0.0	0.0	0.0	0	0	0
Brazil	Areiópolis	0.2	0.1	0.2	0	0	0	0.0	0.0	0.0	0	0	0
Brazil	Arenápolis	0.4	0.3	0.5	0	0	1	0.1	0.1	0.1	0	0	0
Brazil	Arenópolis	0.2	0.1	0.2	0	0	0	0.1	0.0	0.1	0	0	0
Brazil	Arês	1.2	0.9	1.5	2	1	2	0.1	0.1	0.2	0	0	0
Brazil	Argirita	0.3	0.2	0.4	0	0	0	0.1	0.0	0.1	0	0	0
Brazil	Aricanduva	0.3	0.2	0.4	0	0	0	0.1	0.0	0.1	0	0	0
Brazil	Arinos	0.3	0.2	0.4	1	0	1	0.1	0.0	0.1	0	0	0
Brazil	Aripuanã	0.4	0.3	0.5	1	1	2	0.1	0.1	0.1	1	0	1
Brazil	Ariquemes	0.4	0.3	0.5	3	3	4	0.1	0.1	0.1	1	1	1
Brazil	Ariranha	0.2	0.1	0.2	0	0	0	0.0	0.0	0.0	0	0	0
Brazil	Ariranha do Ivaí	0.3	0.2	0.3	0	0	0	0.1	0.0	0.1	0	0	0

Table 3: Diarrhoea Mortality by Unit *(continued)*

Country	Unit	2000 mortality						2017 mortality					
		mean rate (per 10,000)	lower rate (per 10,000)	upper rate (per 10,000)	mean count	lower count	upper count	mean rate (per 10,000)	lower rate (per 10,000)	upper rate (per 10,000)	mean count	lower count	upper count
Brazil	Armação dos Búzios	0.2	0.1	0.3	0	0	0	0.1	0.0	0.1	0	0	0
Brazil	Armazém	0.2	0.2	0.3	0	0	0	0.1	0.0	0.1	0	0	0
Brazil	Arneiroz	2.3	1.7	2.9	2	2	3	0.1	0.1	0.2	0	0	0
Brazil	Aroazes	1.4	1.1	1.8	1	1	1	0.2	0.1	0.2	0	0	0
Brazil	Aroeiras	1.0	0.7	1.2	2	2	3	0.1	0.1	0.1	0	0	0
Brazil	Arraial	1.4	1.1	1.8	1	1	1	0.2	0.1	0.2	0	0	0
Brazil	Arraial do Cabo	0.2	0.1	0.3	0	0	1	0.1	0.0	0.1	0	0	0
Brazil	Arraias	0.8	0.7	1.1	1	1	1	0.1	0.1	0.2	0	0	0
Brazil	Arroio do Meio	0.1	0.1	0.2	0	0	0	0.0	0.0	0.0	0	0	0
Brazil	Arroio do Sal	0.1	0.1	0.2	0	0	0	0.0	0.0	0.0	0	0	0
Brazil	Arroio do Tigre	0.1	0.1	0.2	0	0	0	0.0	0.0	0.0	0	0	0
Brazil	Arroio dos Ratos	0.1	0.1	0.2	0	0	0	0.0	0.0	0.0	0	0	0
Brazil	Arroio Grande	0.1	0.1	0.2	0	0	0	0.0	0.0	0.0	0	0	0
Brazil	Arroio Trinta	0.2	0.2	0.3	0	0	0	0.1	0.0	0.1	0	0	0
Brazil	Artur Nogueira	0.2	0.1	0.2	0	0	1	0.0	0.0	0.0	0	0	0
Brazil	Aruanã	0.2	0.1	0.2	0	0	0	0.1	0.0	0.1	0	0	0
Brazil	Aruja	0.2	0.1	0.2	1	1	1	0.0	0.0	0.0	0	0	0
Brazil	Arvoredo	0.2	0.2	0.3	0	0	0	0.1	0.0	0.1	0	0	0
Brazil	Arvorezinha	0.1	0.1	0.2	0	0	0	0.0	0.0	0.0	0	0	0
Brazil	Ascurra	0.2	0.2	0.3	0	0	0	0.1	0.0	0.1	0	0	0
Brazil	Aspásia	0.2	0.1	0.2	0	0	0	0.0	0.0	0.0	0	0	0
Brazil	Assaí	0.3	0.2	0.3	0	0	1	0.1	0.0	0.1	0	0	0
Brazil	Assaré	2.3	1.7	2.9	6	4	7	0.1	0.1	0.2	0	0	0
Brazil	Assis	0.2	0.1	0.2	1	1	1	0.0	0.0	0.0	0	0	0
Brazil	Assis Brazil	1.1	0.9	1.3	0	0	0	0.2	0.2	0.3	0	0	0
Brazil	Assis Chateaubri	0.3	0.2	0.3	1	1	1	0.1	0.0	0.1	0	0	0
Brazil	Assunção	1.0	0.7	1.2	0	0	1	0.1	0.1	0.1	0	0	0
Brazil	Assunção do Piauí	1.4	1.1	1.8	1	1	1	0.2	0.1	0.2	0	0	0
Brazil	Astolfo Dutra	0.3	0.2	0.4	0	0	0	0.1	0.0	0.1	0	0	0
Brazil	Astorga	0.3	0.2	0.3	1	0	1	0.1	0.0	0.1	0	0	0
Brazil	Atalaia do Norte	0.8	0.6	0.9	1	1	1	0.2	0.2	0.3	0	0	1
Brazil	Atalanta	0.2	0.2	0.3	0	0	0	0.1	0.0	0.1	0	0	0
Brazil	Ataleia	0.3	0.2	0.4	0	0	1	0.1	0.0	0.1	0	0	0
Brazil	Ataléia	0.3	0.2	0.3	0	0	0	0.1	0.0	0.1	0	0	0
Brazil	Ataléia	2.8	2.2	3.7	17	13	22	0.3	0.2	0.4	2	1	2
Brazil	Atibaia	0.2	0.1	0.2	1	1	2	0.0	0.0	0.0	0	0	0
Brazil	Atilio Vivacqua	0.3	0.2	0.3	0	0	0	0.1	0.1	0.1	0	0	0
Brazil	Augustinópolis	0.8	0.7	1.1	2	1	2	0.1	0.1	0.2	0	0	0
Brazil	Augusto Corrêa	0.9	0.7	1.1	4	3	5	0.1	0.1	0.2	1	1	1
Brazil	Augusto de Lima	0.3	0.2	0.4	0	0	0	0.1	0.0	0.1	0	0	0
Brazil	Augusto Pestana	0.1	0.1	0.2	0	0	0	0.0	0.0	0.0	0	0	0
Brazil	Augusto Severo	1.2	0.9	1.5	1	1	2	0.1	0.1	0.2	0	0	0
Brazil	Aurelino Leal	1.7	1.4	2.2	4	3	5	0.2	0.2	0.3	0	0	0
Brazil	Auriflama	0.2	0.1	0.2	0	0	0	0.0	0.0	0.0	0	0	0
Brazil	Aurilândia	0.2	0.1	0.2	0	0	0	0.1	0.0	0.1	0	0	0
Brazil	Aurora	2.3	1.7	2.9	6	5	8	0.1	0.1	0.2	0	0	0
Brazil	Aurora	0.2	0.2	0.3	0	0	0	0.1	0.0	0.1	0	0	0
Brazil	Aurora do Pará	0.9	0.7	1.1	2	2	3	0.1	0.1	0.2	0	0	1
Brazil	Aurora do Tocantins	0.8	0.7	1.1	0	0	0	0.1	0.1	0.2	0	0	0
Brazil	Autazes	0.8	0.6	0.9	2	2	3	0.2	0.2	0.3	1	1	1
Brazil	Avaí	0.2	0.1	0.2	0	0	0	0.0	0.0	0.0	0	0	0
Brazil	Avanhandava	0.2	0.1	0.2	0	0	0	0.0	0.0	0.0	0	0	0
Brazil	Avare	0.2	0.1	0.2	1	1	1	0.0	0.0	0.0	0	0	0
Brazil	Aveiro	0.9	0.7	1.1	2	1	2	0.1	0.1	0.2	0	0	0
Brazil	Avelino Lopes	1.4	1.1	1.8	2	1	2	0.2	0.1	0.2	0	0	0
Brazil	Avelinópolis	0.2	0.1	0.2	0	0	0	0.1	0.0	0.1	0	0	0
Brazil	Axixá	1.7	1.3	2.2	2	2	3	0.2	0.2	0.3	0	0	0

Table 3: Diarrhoea Mortality by Unit *(continued)*

Country	Unit	2000 mortality mean rate (per 10,000)	2000 lower rate (per 10,000)	2000 upper rate (per 10,000)	2000 mean count	2000 lower count	2000 upper count	2017 mortality mean rate (per 10,000)	2017 lower rate (per 10,000)	2017 upper rate (per 10,000)	2017 mean count	2017 lower count	2017 upper count
Brazil	Axixá do Tocantins	0.8	0.7	1.1	1	1	1	0.1	0.1	0.2	0	0	0
Brazil	Babaçulândia	0.8	0.7	1.1	1	1	1	0.1	0.1	0.2	0	0	0
Brazil	Bacabal	1.7	1.3	2.2	23	17	29	0.2	0.2	0.3	3	2	4
Brazil	Bacabeira	1.7	1.3	2.2	3	2	3	0.2	0.2	0.3	0	0	1
Brazil	Bacuri	1.7	1.3	2.2	4	3	6	0.2	0.2	0.3	1	0	1
Brazil	Bacurituba	1.7	1.3	2.2	2	1	2	0.2	0.2	0.3	0	0	0
Brazil	Bady Bassitt	0.2	0.1	0.2	0	0	0	0.0	0.0	0.0	0	0	0
Brazil	Baependi	0.3	0.2	0.4	0	0	1	0.1	0.0	0.1	0	0	0
Brazil	Bagé	0.9	0.7	1.1	2	2	3	0.1	0.1	0.2	1	1	1
Brazil	Baía da Traição	1.0	0.7	1.2	1	1	1	0.1	0.1	0.1	0	0	0
Brazil	Baía Formosa	1.2	0.9	1.5	1	1	1	0.1	0.1	0.2	0	0	0
Brazil	Baianópolis	1.7	1.4	2.2	2	2	3	0.2	0.2	0.3	0	0	0
Brazil	Baião	0.9	0.7	1.1	3	2	3	0.1	0.1	0.2	1	1	1
Brazil	Baixa Grande	1.7	1.4	2.2	4	3	6	0.2	0.2	0.3	1	0	1
Brazil	Baixa Grande do Ribeiro	1.4	1.1	1.8	1	1	2	0.2	0.1	0.2	0	0	0
Brazil	Baixio	2.3	1.7	2.9	2	1	2	0.1	0.1	0.2	0	0	0
Brazil	Baixo Guandu	0.3	0.2	0.3	1	1	1	0.1	0.1	0.1	0	0	0
Brazil	Baje	0.1	0.1	0.2	1	1	2	0.0	0.0	0.0	0	0	0
Brazil	Balbinos	0.2	0.1	0.2	0	0	0	0.0	0.0	0.0	0	0	0
Brazil	Baldim	0.3	0.2	0.4	0	0	0	0.1	0.0	0.1	0	0	0
Brazil	Baliza	0.2	0.1	0.2	0	0	0	0.1	0.0	0.1	0	0	0
Brazil	Balneário Arroio do Silva	0.2	0.2	0.3	1	1	2	0.1	0.0	0.1	0	0	0
Brazil	Balneário Barra do Sul	0.2	0.2	0.3	0	0	0	0.1	0.0	0.1	0	0	0
Brazil	Balneário Camboriú	0.2	0.2	0.3	0	0	0	0.1	0.0	0.1	0	0	0
Brazil	Balneário Gaivota	0.2	0.2	0.3	0	0	0	0.1	0.0	0.1	0	0	0
Brazil	Balneário Pinhal	0.1	0.1	0.2	0	0	0	0.0	0.0	0.0	0	0	0
Brazil	Balsa Nova	0.3	0.2	0.3	1	1	2	0.1	0.0	0.1	0	0	0
Brazil	Bálsamo	0.2	0.1	0.2	0	0	0	0.0	0.0	0.0	0	0	0
Brazil	Balsas	1.7	1.3	2.2	17	13	22	0.2	0.2	0.3	3	2	4
Brazil	Bambuí	0.3	0.2	0.4	0	0	1	0.1	0.0	0.1	0	0	0
Brazil	Banabuiú	2.3	1.7	2.9	4	3	5	0.1	0.1	0.2	0	0	0
Brazil	Bananal	0.2	0.1	0.2	0	0	0	0.0	0.0	0.0	0	0	0
Brazil	Bananeiras	1.0	0.7	1.2	3	2	4	0.1	0.1	0.1	0	0	0
Brazil	Bandeira	0.3	0.2	0.4	0	0	0	0.1	0.0	0.1	0	0	0
Brazil	Bandeirante	0.2	0.2	0.3	0	0	0	0.1	0.0	0.1	0	0	0
Brazil	Bandeirantes	0.5	0.3	0.6	0	0	0	0.2	0.1	0.2	0	0	0
Brazil	Bandeirantes	0.3	0.2	0.3	1	1	1	0.1	0.0	0.1	0	0	0
Brazil	Bandeirantes do Tocantins	0.8	0.7	1.1	0	0	0	0.1	0.1	0.2	0	0	0
Brazil	Bandiera do Sul	0.3	0.2	0.4	0	0	0	0.1	0.0	0.1	0	0	0
Brazil	Bannach	0.9	0.7	1.1	0	0	0	0.1	0.1	0.2	0	0	0
Brazil	Banzaê	1.7	1.4	2.2	2	2	3	0.2	0.2	0.3	0	0	0
Brazil	Barão de Antonina	0.2	0.1	0.2	0	0	0	0.0	0.0	0.0	0	0	0
Brazil	Barão de Cocais	0.3	0.2	0.4	1	0	1	0.1	0.0	0.1	0	0	0
Brazil	Barao de Cotegipe	0.1	0.1	0.2	0	0	0	0.0	0.0	0.0	0	0	0
Brazil	Barão de Grajaú	1.7	1.3	2.2	4	3	5	0.2	0.2	0.3	0	0	1
Brazil	Barão de Melgaço	0.4	0.3	0.5	0	0	0	0.1	0.1	0.1	0	0	0
Brazil	Barão de Monte Alto	0.3	0.2	0.4	0	0	0	0.1	0.0	0.1	0	0	0
Brazil	Barão do Triunfo	0.1	0.1	0.2	0	0	0	0.0	0.0	0.0	0	0	0
Brazil	Baraúna	1.2	0.9	1.5	3	2	3	0.1	0.1	0.2	0	0	1
Brazil	Baraúna	1.0	0.7	1.2	0	0	0	0.1	0.1	0.1	0	0	0

Table 3: Diarrhoea Mortality by Unit *(continued)*

Country	Unit	2000 mortality mean rate (per 10,000)	lower rate (per 10,000)	upper rate (per 10,000)	mean count	lower count	upper count	2017 mortality mean rate (per 10,000)	lower rate (per 10,000)	upper rate (per 10,000)	mean count	lower count	upper count
Brazil	Barbacena	0.3	0.2	0.4	3	2	3	0.1	0.0	0.1	0	0	1
Brazil	Barbalha	2.3	1.7	2.9	16	12	20	0.1	0.1	0.2	1	1	1
Brazil	Barbosa	0.2	0.1	0.2	0	0	0	0.0	0.0	0.0	0	0	0
Brazil	Barbosa Ferraz	0.3	0.2	0.3	0	0	0	0.1	0.0	0.1	0	0	0
Brazil	Barcarena	0.9	0.7	1.1	6	5	8	0.1	0.1	0.2	2	1	2
Brazil	Barcelona	1.2	0.9	1.5	1	0	1	0.1	0.1	0.2	0	0	0
Brazil	Barcelos	0.8	0.6	0.9	2	2	3	0.2	0.2	0.3	1	1	1
Brazil	Bariri	0.2	0.1	0.2	0	0	0	0.0	0.0	0.0	0	0	0
Brazil	Baro	0.1	0.1	0.2	0	0	0	0.0	0.0	0.0	0	0	0
Brazil	Barra	1.7	1.4	2.2	8	6	10	0.2	0.2	0.3	1	1	1
Brazil	Barra Bonita	0.2	0.2	0.3	0	0	0	0.1	0.0	0.1	0	0	0
Brazil	Barra Bonita	0.2	0.1	0.2	0	0	1	0.0	0.0	0.0	0	0	0
Brazil	Barra d'Alcântara	1.4	1.1	1.8	1	1	1	0.2	0.1	0.2	0	0	0
Brazil	Barra da Choça	1.7	1.4	2.2	8	7	11	0.2	0.2	0.3	1	1	1
Brazil	Barra da Estiva	1.7	1.4	2.2	6	4	7	0.2	0.2	0.3	1	0	1
Brazil	Barra de Guabira	2.0	1.6	2.6	3	3	4	0.2	0.2	0.3	0	0	0
Brazil	Barra de Santa Rosa	1.0	0.7	1.2	2	1	2	0.1	0.1	0.1	0	0	0
Brazil	Barra de Santana	1.0	0.7	1.2	1	1	1	0.1	0.1	0.1	0	0	0
Brazil	Barra de Santo Antônio	2.8	2.2	3.7	4	3	5	0.3	0.2	0.4	0	0	1
Brazil	Barra de São Francisco	0.3	0.2	0.3	1	1	1	0.1	0.1	0.1	0	0	0
Brazil	Barra de São Miguel	1.0	0.7	1.2	0	0	1	0.1	0.1	0.1	0	0	0
Brazil	Barra de São Miguel	2.8	2.2	3.7	0	0	0	0.3	0.2	0.4	0	0	0
Brazil	Barra do Chapéu	0.2	0.1	0.2	0	0	0	0.0	0.0	0.0	0	0	0
Brazil	Barra do Corda	1.7	1.3	2.2	21	16	27	0.2	0.2	0.3	3	2	4
Brazil	Barra do Garças	0.4	0.3	0.5	2	2	2	0.1	0.1	0.1	0	0	1
Brazil	Barra do Guarita	0.1	0.1	0.2	0	0	0	0.0	0.0	0.0	0	0	0
Brazil	Barra do Jacaré	0.3	0.2	0.3	0	0	0	0.1	0.0	0.1	0	0	0
Brazil	Barra do Mendes	1.7	1.4	2.2	2	2	3	0.2	0.2	0.3	0	0	0
Brazil	Barra do Ouro	0.8	0.7	1.1	0	0	1	0.1	0.1	0.2	0	0	0
Brazil	Barra do Piraí	0.2	0.1	0.3	2	1	2	0.1	0.0	0.1	0	0	0
Brazil	Barra do Quaraí	0.1	0.1	0.2	0	0	0	0.0	0.0	0.0	0	0	0
Brazil	Barra do Ribeiro	0.1	0.1	0.2	0	0	0	0.0	0.0	0.0	0	0	0
Brazil	Barra do Rio Azul	0.1	0.1	0.2	0	0	0	0.0	0.0	0.0	0	0	0
Brazil	Barra do Rocha	1.7	1.4	2.2	1	1	1	0.2	0.2	0.3	0	0	0
Brazil	Barra do Turvo	0.2	0.1	0.2	0	0	0	0.0	0.0	0.0	0	0	0
Brazil	Barra dos Bugre	0.4	0.3	0.5	1	1	2	0.1	0.1	0.1	0	0	0
Brazil	Barra dos Coqueiros	1.3	1.0	1.7	4	3	5	0.2	0.1	0.3	1	0	1
Brazil	Barra Funda	0.1	0.1	0.2	0	0	0	0.0	0.0	0.0	0	0	0
Brazil	Barra Longa	0.3	0.2	0.4	0	0	0	0.1	0.0	0.1	0	0	0
Brazil	Barra Mansa	0.2	0.1	0.3	3	2	4	0.1	0.0	0.1	1	1	1
Brazil	Barra Velha	0.2	0.2	0.3	0	0	1	0.1	0.0	0.1	0	0	0
Brazil	Barracão	0.3	0.2	0.3	0	0	0	0.1	0.0	0.1	0	0	0
Brazil	Barracão	0.1	0.1	0.2	0	0	0	0.0	0.0	0.0	0	0	0
Brazil	Barras	1.4	1.1	1.8	7	5	9	0.2	0.1	0.2	1	1	1
Brazil	Barreira	2.3	1.7	2.9	5	4	6	0.1	0.1	0.2	0	0	0
Brazil	Barreiras	1.7	1.4	2.2	31	24	39	0.2	0.2	0.3	7	6	10
Brazil	Barreiras do Piauí	1.4	1.1	1.8	1	0	1	0.2	0.1	0.2	0	0	0
Brazil	Barreirinha	0.8	0.6	0.9	2	2	3	0.2	0.2	0.3	1	1	1
Brazil	Barreirinhas	1.7	1.3	2.2	11	8	14	0.2	0.2	0.3	2	2	3
Brazil	Barreiros	2.0	1.6	2.6	10	8	13	0.2	0.2	0.3	1	1	1
Brazil	Barretos	0.2	0.1	0.2	1	1	2	0.0	0.0	0.0	0	0	0

Table 3: Diarrhoea Mortality by Unit *(continued)*

Country	Unit	2000 mortality mean rate (per 10,000)	lower rate (per 10,000)	upper rate (per 10,000)	mean count	lower count	upper count	2017 mortality mean rate (per 10,000)	lower rate (per 10,000)	upper rate (per 10,000)	mean count	lower count	upper count
Brazil	Barrinha	0.2	0.1	0.2	0	0	0	0.0	0.0	0.0	0	0	0
Brazil	Barro	2.3	1.7	2.9	5	3	6	0.1	0.1	0.2	0	0	0
Brazil	Barro Alto	0.2	0.1	0.2	0	0	0	0.1	0.0	0.1	0	0	0
Brazil	Barro Alto	1.7	1.4	2.2	2	2	3	0.2	0.2	0.3	0	0	0
Brazil	Barro Duro	1.4	1.1	1.8	1	1	1	0.2	0.1	0.2	0	0	0
Brazil	Barro Preto	1.7	1.4	2.2	1	1	1	0.2	0.2	0.3	0	0	0
Brazil	Barrolândia	0.8	0.7	1.1	1	0	1	0.1	0.1	0.2	0	0	0
Brazil	Barroquinha	2.3	1.7	2.9	4	3	5	0.1	0.1	0.2	0	0	0
Brazil	Barros Cassal	0.1	0.1	0.2	0	0	0	0.0	0.0	0.0	0	0	0
Brazil	Barroso	0.3	0.2	0.4	0	0	0	0.1	0.0	0.1	0	0	0
Brazil	Barueri	0.2	0.1	0.2	3	3	4	0.0	0.0	0.0	1	1	1
Brazil	Bastos	0.2	0.1	0.2	0	0	0	0.0	0.0	0.0	0	0	0
Brazil	Bataguassu	0.5	0.3	0.6	1	0	1	0.2	0.1	0.2	0	0	0
Brazil	Bataiporã	0.5	0.3	0.6	1	0	1	0.2	0.1	0.2	0	0	0
Brazil	Batalha	1.4	1.1	1.8	5	4	6	0.2	0.1	0.2	0	0	1
Brazil	Batalha	2.8	2.2	3.7	6	5	8	0.3	0.2	0.4	1	0	1
Brazil	Batatais	0.2	0.1	0.2	1	0	1	0.0	0.0	0.0	0	0	0
Brazil	Baturité	2.3	1.7	2.9	9	7	11	0.1	0.1	0.2	1	0	1
Brazil	Bauru	0.2	0.1	0.2	4	3	5	0.0	0.0	0.0	1	1	1
Brazil	Bayeux	1.0	0.7	1.2	9	7	12	0.1	0.1	0.1	1	1	1
Brazil	Bebedouro	0.2	0.1	0.2	1	1	1	0.0	0.0	0.0	0	0	0
Brazil	Beberibe	2.3	1.7	2.9	10	7	12	0.1	0.1	0.2	1	0	1
Brazil	Bela Cruz	2.3	1.7	2.9	9	7	12	0.1	0.1	0.2	1	0	1
Brazil	Bela Vista	0.5	0.3	0.6	1	1	1	0.2	0.1	0.2	0	0	0
Brazil	Bela Vista da Caroba	0.3	0.2	0.3	0	0	0	0.1	0.0	0.1	0	0	0
Brazil	Bela Vista de Goiás	0.2	0.1	0.2	0	0	0	0.1	0.0	0.1	0	0	0
Brazil	Bela Vista de Minas	0.3	0.2	0.4	0	0	0	0.1	0.0	0.1	0	0	0
Brazil	Bela Vista do Maranhão	1.7	1.3	2.2	2	1	2	0.2	0.2	0.3	0	0	0
Brazil	Bela Vista do Paraíso	0.3	0.2	0.3	0	0	0	0.1	0.0	0.1	0	0	0
Brazil	Bela Vista do Piauí	1.4	1.1	1.8	0	0	1	0.2	0.1	0.2	0	0	0
Brazil	Bela Vista do Toldo	0.2	0.2	0.3	0	0	0	0.1	0.0	0.1	0	0	0
Brazil	Belágua	1.7	1.3	2.2	2	1	2	0.2	0.2	0.3	0	0	0
Brazil	Belém	0.9	0.7	1.1	93	73	116	0.1	0.1	0.2	13	10	17
Brazil	Belém	1.0	0.7	1.2	2	2	3	0.1	0.1	0.1	0	0	0
Brazil	Belém	2.8	2.2	3.7	1	1	2	0.3	0.2	0.4	0	0	0
Brazil	Belém de Maria	2.0	1.6	2.6	2	1	2	0.2	0.2	0.3	0	0	0
Brazil	Belém de São Francisco	2.0	1.6	2.6	7	5	9	0.2	0.2	0.3	1	0	1
Brazil	Belém do Brejo do Cruz	1.0	0.7	1.2	1	1	1	0.1	0.1	0.1	0	0	0
Brazil	Belém do Piauí	1.4	1.1	1.8	0	0	1	0.2	0.1	0.2	0	0	0
Brazil	Belford Roxo	0.2	0.1	0.3	9	7	12	0.1	0.0	0.1	2	2	3
Brazil	Belmiro Braga	0.3	0.2	0.4	0	0	0	0.1	0.0	0.1	0	0	0
Brazil	Belmonte	0.2	0.2	0.3	0	0	0	0.1	0.0	0.1	0	0	0
Brazil	Belmonte	1.7	1.4	2.2	2	2	3	0.2	0.2	0.3	0	0	0
Brazil	Belo Campo	1.7	1.4	2.2	3	2	3	0.2	0.2	0.3	0	0	0
Brazil	Belo Horizonte	0.3	0.2	0.4	49	38	62	0.1	0.0	0.1	8	6	11
Brazil	Belo Jardim	2.0	1.6	2.6	18	14	23	0.2	0.2	0.3	2	1	2
Brazil	Belo Monte	2.8	2.2	3.7	3	2	4	0.3	0.2	0.4	0	0	0
Brazil	Belo Oriente	0.3	0.2	0.4	0	0	1	0.1	0.0	0.1	0	0	0
Brazil	Belo Vale	0.3	0.2	0.4	0	0	0	0.1	0.0	0.1	0	0	0
Brazil	Belterra	0.9	0.7	1.1	2	1	2	0.1	0.1	0.2	0	0	0
Brazil	Beneditinos	1.4	1.1	1.8	1	1	2	0.2	0.1	0.2	0	0	0

Table 3: Diarrhoea Mortality by Unit *(continued)*

Country	Unit	2000 mortality mean rate (per 10,000)	lower rate (per 10,000)	upper rate (per 10,000)	mean count	lower count	upper count	2017 mortality mean rate (per 10,000)	lower rate (per 10,000)	upper rate (per 10,000)	mean count	lower count	upper count
Brazil	Benedito Leite	1.7	1.3	2.2	2	2	3	0.2	0.2	0.3	0	0	0
Brazil	Benedito Novo	0.2	0.2	0.3	0	0	0	0.1	0.0	0.1	0	0	0
Brazil	Benevides	0.9	0.7	1.1	3	2	4	0.1	0.1	0.2	1	1	1
Brazil	Benjamin Constant	0.8	0.6	0.9	2	2	3	0.2	0.2	0.3	1	1	2
Brazil	Benjamin Constant do Sul	0.1	0.1	0.2	0	0	0	0.0	0.0	0.0	0	0	0
Brazil	Bento de Abreu	0.2	0.1	0.2	0	0	0	0.0	0.0	0.0	0	0	0
Brazil	Bento Fernandes	1.2	0.9	1.5	1	1	1	0.1	0.1	0.2	0	0	0
Brazil	Bento Gonçalves	0.1	0.1	0.2	1	0	1	0.0	0.0	0.0	0	0	0
Brazil	Bequimão	1.7	1.3	2.2	4	3	5	0.2	0.2	0.3	1	0	1
Brazil	Berilo	0.3	0.2	0.4	0	0	1	0.1	0.0	0.1	0	0	0
Brazil	Berizal	0.3	0.2	0.4	0	0	0	0.1	0.0	0.1	0	0	0
Brazil	Bernardino Batista	1.0	0.7	1.2	0	0	0	0.1	0.1	0.1	0	0	0
Brazil	Bernardino de Campos	0.2	0.1	0.2	0	0	0	0.0	0.0	0.0	0	0	0
Brazil	Bernardo do Mearim	1.7	1.3	2.2	1	1	2	0.2	0.2	0.3	0	0	0
Brazil	Bernardo Sayão	0.8	0.7	1.1	0	0	1	0.1	0.1	0.2	0	0	0
Brazil	Bertioga	0.2	0.1	0.2	0	0	0	0.0	0.0	0.0	0	0	0
Brazil	Bertolínia	1.4	1.1	1.8	1	1	1	0.2	0.1	0.2	0	0	0
Brazil	Bertópolis	0.3	0.2	0.4	0	0	0	0.1	0.0	0.1	0	0	0
Brazil	Beruri	0.8	0.6	0.9	1	1	1	0.2	0.2	0.3	0	0	1
Brazil	Betânia	2.0	1.6	2.6	3	3	4	0.2	0.2	0.3	0	0	0
Brazil	Betânia do Piauí	1.4	1.1	1.8	2	2	3	0.2	0.1	0.2	0	0	0
Brazil	Betim	0.3	0.2	0.4	9	7	12	0.1	0.0	0.1	2	2	3
Brazil	Bezerros	2.0	1.6	2.6	11	9	15	0.2	0.2	0.3	1	1	1
Brazil	Bias Fortes	0.3	0.2	0.4	0	0	0	0.1	0.0	0.1	0	0	0
Brazil	Bicas	0.3	0.2	0.4	0	0	0	0.1	0.0	0.1	0	0	0
Brazil	Biguaçu	0.2	0.2	0.3	1	1	1	0.1	0.0	0.1	0	0	0
Brazil	Bilac	0.2	0.1	0.2	0	0	0	0.0	0.0	0.0	0	0	0
Brazil	Biquinhas	0.3	0.2	0.4	0	0	0	0.1	0.0	0.1	0	0	0
Brazil	Birigui	0.2	0.1	0.2	1	1	1	0.0	0.0	0.0	0	0	0
Brazil	Biritiba Mirim	0.2	0.1	0.2	0	0	1	0.0	0.0	0.0	0	0	0
Brazil	Biritinga	1.7	1.4	2.2	3	3	4	0.2	0.2	0.3	0	0	0
Brazil	Bituruna	0.3	0.2	0.3	1	0	1	0.1	0.0	0.1	0	0	0
Brazil	Blumenau	0.2	0.2	0.3	5	4	6	0.1	0.0	0.1	1	1	1
Brazil	Boa Esperança	0.3	0.2	0.3	0	0	0	0.1	0.1	0.1	0	0	0
Brazil	Boa Esperança	0.3	0.2	0.3	0	0	0	0.1	0.0	0.1	0	0	0
Brazil	Boa Esperança	0.3	0.2	0.4	1	1	1	0.1	0.0	0.1	0	0	0
Brazil	Boa Esperança do Iguaçu	0.3	0.2	0.3	0	0	0	0.1	0.0	0.1	0	0	0
Brazil	Boa Esperanca do Sul	0.2	0.1	0.2	0	0	0	0.0	0.0	0.0	0	0	0
Brazil	Boa Hora	1.4	1.1	1.8	1	1	1	0.2	0.1	0.2	0	0	0
Brazil	Boa Nova	1.7	1.4	2.2	4	3	6	0.2	0.2	0.3	0	0	0
Brazil	Boa Ventura	1.0	0.7	1.2	1	1	1	0.1	0.1	0.1	0	0	0
Brazil	Boa Ventura de São Roque	0.3	0.2	0.3	0	0	0	0.1	0.0	0.1	0	0	0
Brazil	Boa Viagem	2.3	1.7	2.9	14	11	18	0.1	0.1	0.2	1	1	1
Brazil	Boa Vista	1.0	0.7	1.2	1	0	1	0.1	0.1	0.1	0	0	0
Brazil	Boa Vista	0.7	0.6	0.9	20	16	25	0.2	0.2	0.3	8	6	10
Brazil	Boa Vista da Aparecida	0.3	0.2	0.3	0	0	0	0.1	0.0	0.1	0	0	0
Brazil	Boa Vista das Misses	0.1	0.1	0.2	0	0	0	0.0	0.0	0.0	0	0	0
Brazil	Boa Vista das Missões	0.1	0.1	0.2	0	0	0	0.0	0.0	0.0	0	0	0
Brazil	Boa Vista do Buricá	0.1	0.1	0.2	0	0	0	0.0	0.0	0.0	0	0	0

Table 3: Diarrhoea Mortality by Unit *(continued)*

Country	Unit	2000 mortality mean rate (per 10,000)	2000 lower rate (per 10,000)	2000 upper rate (per 10,000)	2000 mean count	2000 lower count	2000 upper count	2017 mortality mean rate (per 10,000)	2017 lower rate (per 10,000)	2017 upper rate (per 10,000)	2017 mean count	2017 lower count	2017 upper count
Brazil	Boa Vista do Gurupi	1.7	1.3	2.2	1	0	1	0.2	0.2	0.3	0	0	0
Brazil	Boa Vista do Ramos	0.8	0.6	0.9	1	1	1	0.2	0.2	0.3	1	0	1
Brazil	Boa Vista do Sul	0.1	0.1	0.2	0	0	0	0.0	0.0	0.0	0	0	0
Brazil	Boa Vista do Tupim	1.7	1.4	2.2	4	3	6	0.2	0.2	0.3	0	0	1
Brazil	Boca da Mata	2.8	2.2	3.7	8	6	10	0.3	0.2	0.4	1	1	1
Brazil	Boca do Acre	0.8	0.6	0.9	3	2	3	0.2	0.2	0.3	1	1	1
Brazil	Bocaina	1.4	1.1	1.8	1	0	1	0.2	0.1	0.2	0	0	0
Brazil	Bocaina	0.2	0.1	0.2	0	0	0	0.0	0.0	0.0	0	0	0
Brazil	Bocaina de Minas	0.3	0.2	0.4	0	0	0	0.1	0.0	0.1	0	0	0
Brazil	Bocaina do Sul	0.2	0.2	0.3	0	0	0	0.1	0.0	0.1	0	0	0
Brazil	Bocaiúva	0.3	0.2	0.4	1	1	2	0.1	0.0	0.1	0	0	0
Brazil	Bocaiúva do Sul	0.3	0.2	0.3	0	0	0	0.1	0.0	0.1	0	0	0
Brazil	Bodó	1.2	0.9	1.5	0	0	0	0.1	0.1	0.2	0	0	0
Brazil	Bodocó	2.0	1.6	2.6	10	8	13	0.2	0.2	0.3	1	1	1
Brazil	Bodoquena	0.5	0.3	0.6	0	0	1	0.2	0.1	0.2	0	0	0
Brazil	Bofete	0.2	0.1	0.2	0	0	0	0.0	0.0	0.0	0	0	0
Brazil	Boituva	0.2	0.1	0.2	0	0	1	0.0	0.0	0.0	0	0	0
Brazil	Bom Conselho	2.0	1.6	2.6	12	9	15	0.2	0.2	0.3	1	1	1
Brazil	Bom despacho	0.3	0.2	0.4	1	1	1	0.1	0.0	0.1	0	0	0
Brazil	Bom Jardim	0.2	0.1	0.3	0	0	1	0.1	0.0	0.1	0	0	0
Brazil	Bom Jardim	2.0	1.6	2.6	9	7	12	0.2	0.2	0.3	1	1	1
Brazil	Bom Jardim da Serra	0.2	0.2	0.3	0	0	0	0.1	0.0	0.1	0	0	0
Brazil	Bom Jardim de Goiás	0.2	0.1	0.2	0	0	0	0.1	0.0	0.1	0	0	0
Brazil	Bom Jardim de Minas	0.3	0.2	0.4	0	0	0	0.1	0.0	0.1	0	0	0
Brazil	Bom Jardin	1.7	1.3	2.2	11	9	15	0.2	0.2	0.3	2	1	2
Brazil	Bom Jesus	1.2	0.9	1.5	1	1	2	0.1	0.1	0.2	0	0	0
Brazil	Bom Jesus	1.4	1.1	1.8	3	2	4	0.2	0.1	0.2	1	0	1
Brazil	Bom Jesus	1.0	0.7	1.2	0	0	0	0.1	0.1	0.1	0	0	0
Brazil	Bom Jesus	0.2	0.2	0.3	0	0	0	0.1	0.0	0.1	0	0	0
Brazil	Bom Jesus	0.1	0.1	0.2	0	0	0	0.0	0.0	0.0	0	0	0
Brazil	Bom Jesus da Lapa	1.7	1.4	2.2	13	10	16	0.2	0.2	0.3	2	1	2
Brazil	Bom Jesus da Penha	0.3	0.2	0.4	0	0	0	0.1	0.0	0.1	0	0	0
Brazil	Bom Jesus da Serra	1.7	1.4	2.2	2	1	2	0.2	0.2	0.3	0	0	0
Brazil	Bom Jesus das Selvas	1.7	1.3	2.2	4	3	6	0.2	0.2	0.3	1	1	2
Brazil	Bom Jesus de Goiás	0.2	0.1	0.2	0	0	0	0.1	0.0	0.1	0	0	0
Brazil	Bom Jesus do Amparo	0.3	0.2	0.4	0	0	0	0.1	0.0	0.1	0	0	0
Brazil	Bom Jesus do Galho	0.3	0.2	0.4	0	0	0	0.1	0.0	0.1	0	0	0
Brazil	Bom Jesus do Itabapoana	0.2	0.1	0.3	1	0	1	0.1	0.0	0.1	0	0	0
Brazil	Bom Jesus do Norte	0.3	0.2	0.3	0	0	0	0.1	0.1	0.1	0	0	0
Brazil	Bom Jesus do Oeste	0.2	0.2	0.3	0	0	0	0.1	0.0	0.1	0	0	0
Brazil	Bom Jesus do Sul	0.3	0.2	0.3	0	0	0	0.1	0.0	0.1	0	0	0
Brazil	Bom Jesus do Tocantins	0.9	0.7	1.1	2	1	2	0.1	0.1	0.2	0	0	0
Brazil	Bom Jesus do Tocantins	0.8	0.7	1.1	1	0	1	0.1	0.1	0.2	0	0	0

Country	Unit	2000 mortality mean rate (per 10,000)	2000 mortality lower rate (per 10,000)	2000 mortality upper rate (per 10,000)	2000 mortality mean count	2000 mortality lower count	2000 mortality upper count	2017 mortality mean rate (per 10,000)	2017 mortality lower rate (per 10,000)	2017 mortality upper rate (per 10,000)	2017 mortality mean count	2017 mortality lower count	2017 mortality upper count
Brazil	Bom Lugar	1.7	1.3	2.2	3	2	4	0.2	0.2	0.3	0	0	1
Brazil	Bom Princípio	0.1	0.1	0.2	0	0	0	0.0	0.0	0.0	0	0	0
Brazil	Bom Princípio do Piauí	1.4	1.1	1.8	1	1	1	0.2	0.1	0.2	0	0	0
Brazil	Bom Progresso	0.1	0.1	0.2	0	0	0	0.0	0.0	0.0	0	0	0
Brazil	Bom Repouso	0.3	0.2	0.4	0	0	0	0.1	0.0	0.1	0	0	0
Brazil	Bom Retiro	0.2	0.2	0.3	0	0	0	0.1	0.0	0.1	0	0	0
Brazil	Bom Retiro do Sul	0.1	0.1	0.2	0	0	0	0.0	0.0	0.0	0	0	0
Brazil	Bom Sucesso	1.0	0.7	1.2	1	0	1	0.1	0.1	0.1	0	0	0
Brazil	Bom Sucesso	0.3	0.2	0.3	0	0	0	0.1	0.0	0.1	0	0	0
Brazil	Bom Sucesso	0.3	0.2	0.4	0	0	1	0.1	0.0	0.1	0	0	0
Brazil	Bom Sucesso de Itararé	0.2	0.1	0.2	0	0	0	0.0	0.0	0.0	0	0	0
Brazil	Bom Sucesso do Sul	0.3	0.2	0.3	0	0	0	0.1	0.0	0.1	0	0	0
Brazil	Bombinhas	0.2	0.2	0.3	0	0	0	0.1	0.0	0.1	0	0	0
Brazil	Bon Jesus dos Perdoes	0.2	0.1	0.2	0	0	0	0.0	0.0	0.0	0	0	0
Brazil	Bonfim	0.7	0.6	0.9	1	1	1	0.2	0.2	0.3	0	0	0
Brazil	Bonfim	0.3	0.2	0.4	0	0	0	0.1	0.0	0.1	0	0	0
Brazil	Bonfim do Piauí	1.4	1.1	1.8	1	1	1	0.2	0.1	0.2	0	0	0
Brazil	Bonfinópolis	0.2	0.1	0.2	0	0	0	0.1	0.0	0.1	0	0	0
Brazil	Bonfinópolis de Minas	0.3	0.2	0.4	0	0	0	0.1	0.0	0.1	0	0	0
Brazil	Boninal	1.7	1.4	2.2	3	2	3	0.2	0.2	0.3	0	0	0
Brazil	Bonito	0.5	0.3	0.6	1	1	1	0.2	0.1	0.2	0	0	0
Brazil	Bonito	0.9	0.7	1.1	1	1	1	0.1	0.1	0.2	0	0	0
Brazil	Bonito	2.0	1.6	2.6	11	8	14	0.2	0.2	0.3	1	1	1
Brazil	Bonito	1.7	1.4	2.2	3	2	4	0.2	0.2	0.3	0	0	1
Brazil	Bonito de Minas	0.3	0.2	0.4	0	0	0	0.1	0.0	0.1	0	0	0
Brazil	Bonito de Santa Fé	1.0	0.7	1.2	1	1	1	0.1	0.1	0.1	0	0	0
Brazil	Bonópolis	0.2	0.1	0.2	0	0	0	0.1	0.0	0.1	0	0	0
Brazil	Boqueirão	1.0	0.7	1.2	2	1	2	0.1	0.1	0.1	0	0	0
Brazil	Boqueirão do Leão	0.1	0.1	0.2	0	0	0	0.0	0.0	0.0	0	0	0
Brazil	Boqueirão do Piauí	1.4	1.1	1.8	1	1	1	0.2	0.1	0.2	0	0	0
Brazil	Boqueirao dos Cochos	1.0	0.7	1.2	1	1	1	0.1	0.1	0.1	0	0	0
Brazil	Boquira	1.7	1.4	2.2	5	4	6	0.2	0.2	0.3	0	0	1
Brazil	Borá	0.2	0.1	0.2	0	0	0	0.0	0.0	0.0	0	0	0
Brazil	Boracéia	0.2	0.1	0.2	0	0	0	0.0	0.0	0.0	0	0	0
Brazil	Borba	0.8	0.6	0.9	3	2	4	0.2	0.2	0.3	1	1	1
Brazil	Borborema	1.0	0.7	1.2	1	1	1	0.1	0.1	0.1	0	0	0
Brazil	Borborema	0.2	0.1	0.2	0	0	0	0.0	0.0	0.0	0	0	0
Brazil	Borda da Mata	0.3	0.2	0.4	0	0	0	0.1	0.0	0.1	0	0	0
Brazil	Borebi	0.2	0.1	0.2	0	0	0	0.0	0.0	0.0	0	0	0
Brazil	Borrazópolis	0.3	0.2	0.3	0	0	0	0.1	0.0	0.1	0	0	0
Brazil	Bossoroca	0.1	0.1	0.2	0	0	0	0.0	0.0	0.0	0	0	0
Brazil	Botelhos	0.3	0.2	0.4	0	0	0	0.1	0.0	0.1	0	0	0
Brazil	Botucatu	0.2	0.1	0.2	1	1	2	0.0	0.0	0.0	0	0	0
Brazil	Botumirim	0.3	0.2	0.4	0	0	0	0.1	0.0	0.1	0	0	0
Brazil	Botuporã	1.7	1.4	2.2	2	1	2	0.2	0.2	0.3	0	0	0
Brazil	Botuverá	0.2	0.2	0.3	0	0	0	0.1	0.0	0.1	0	0	0
Brazil	Braço do Norte	0.2	0.2	0.3	1	0	1	0.1	0.0	0.1	0	0	0
Brazil	Braço do Trombudo	0.2	0.2	0.3	0	0	0	0.1	0.0	0.1	0	0	0
Brazil	Braga	0.1	0.1	0.2	0	0	0	0.0	0.0	0.0	0	0	0

Table 3: Diarrhoea Mortality by Unit *(continued)*

Country	Unit	2000 mortality mean rate (per 10,000)	lower rate (per 10,000)	upper rate (per 10,000)	mean count	lower count	upper count	2017 mortality mean rate (per 10,000)	lower rate (per 10,000)	upper rate (per 10,000)	mean count	lower count	upper count
Brazil	Bragança Paulista	0.2	0.1	0.2	2	1	2	0.0	0.0	0.0	0	0	0
Brazil	Braganey	0.3	0.2	0.3	0	0	0	0.1	0.0	0.1	0	0	0
Brazil	Braganga	0.9	0.7	1.1	10	8	13	0.1	0.1	0.2	2	1	2
Brazil	Branquinha	2.8	2.2	3.7	4	3	6	0.3	0.2	0.4	0	0	0
Brazil	Bras Pires	0.3	0.2	0.4	0	0	0	0.1	0.0	0.1	0	0	0
Brazil	Brasabrantes	0.2	0.1	0.2	0	0	0	0.1	0.0	0.1	0	0	0
Brazil	Brasilândia	0.5	0.3	0.6	1	1	1	0.2	0.1	0.2	0	0	0
Brazil	Brasilândia de Minas	0.3	0.2	0.4	0	0	0	0.1	0.0	0.1	0	0	0
Brazil	Brasilândia do Sul	0.3	0.2	0.3	0	0	0	0.1	0.0	0.1	0	0	0
Brazil	Brasilândia do Tocantins	0.8	0.7	1.1	0	0	0	0.1	0.1	0.2	0	0	0
Brazil	Brasiléia	1.1	0.9	1.3	2	1	2	0.2	0.2	0.3	0	0	0
Brazil	Brasília	0.2	0.1	0.2	32	25	41	0.0	0.0	0.1	9	7	12
Brazil	Brasília de Minas	0.3	0.2	0.4	1	1	1	0.1	0.0	0.1	0	0	0
Brazil	Brasnorte	0.4	0.3	0.5	0	0	1	0.1	0.1	0.1	0	0	0
Brazil	Brasópolis	0.3	0.2	0.4	1	1	1	0.1	0.0	0.1	0	0	0
Brazil	Brauna	0.2	0.1	0.2	0	0	0	0.0	0.0	0.0	0	0	0
Brazil	Braúnas	0.3	0.2	0.4	0	0	0	0.1	0.0	0.1	0	0	0
Brazil	Brazil Novo	0.9	0.7	1.1	2	1	2	0.1	0.1	0.2	0	0	0
Brazil	Brazileira	1.4	1.1	1.8	1	1	2	0.2	0.1	0.2	0	0	0
Brazil	Brejão	2.0	1.6	2.6	3	2	3	0.2	0.2	0.3	0	0	0
Brazil	Brejetuba	0.3	0.2	0.3	0	0	0	0.1	0.1	0.1	0	0	0
Brazil	Brejinho	1.2	0.9	1.5	1	1	1	0.1	0.1	0.2	0	0	0
Brazil	Brejinho	2.0	1.6	2.6	1	1	2	0.2	0.2	0.3	0	0	0
Brazil	Brejinho de Nazaré	0.8	0.7	1.1	1	0	1	0.1	0.1	0.2	0	0	0
Brazil	Brejo	1.7	1.3	2.2	7	5	9	0.2	0.2	0.3	1	1	1
Brazil	Brejo Alegre	0.2	0.1	0.2	0	0	0	0.0	0.0	0.0	0	0	0
Brazil	Brejo da Madre de deus	2.0	1.6	2.6	14	11	19	0.2	0.2	0.3	2	1	2
Brazil	Brejo de Areia	1.7	1.3	2.2	1	1	2	0.2	0.2	0.3	0	0	0
Brazil	Brejo do Cruz	1.0	0.7	1.2	1	1	2	0.1	0.1	0.1	0	0	0
Brazil	Brejo do Piauí	1.4	1.1	1.8	1	0	1	0.2	0.1	0.2	0	0	0
Brazil	Brejo dos Santos	1.0	0.7	1.2	0	0	1	0.1	0.1	0.1	0	0	0
Brazil	Brejo Grande	1.3	1.0	1.7	1	1	2	0.2	0.1	0.3	0	0	0
Brazil	Brejo Grande do Araguaia	0.9	0.7	1.1	1	1	1	0.1	0.1	0.2	0	0	0
Brazil	Brejo Santo	2.3	1.7	2.9	9	7	12	0.1	0.1	0.2	1	0	1
Brazil	Brejões	1.7	1.4	2.2	3	3	4	0.2	0.2	0.3	0	0	0
Brazil	Brejolândia	1.7	1.4	2.2	2	2	3	0.2	0.2	0.3	0	0	0
Brazil	Breu Branco	0.9	0.7	1.1	6	5	8	0.1	0.1	0.2	2	1	2
Brazil	Breves	0.9	0.7	1.1	11	9	14	0.1	0.1	0.2	2	1	2
Brazil	Brochier	0.1	0.1	0.2	0	0	0	0.0	0.0	0.0	0	0	0
Brazil	Brodosqui	0.2	0.1	0.2	0	0	0	0.0	0.0	0.0	0	0	0
Brazil	Brotas	0.2	0.1	0.2	0	0	0	0.0	0.0	0.0	0	0	0
Brazil	Brotas de Macaúbas	1.7	1.4	2.2	2	2	3	0.2	0.2	0.3	0	0	0
Brazil	Brumadinho	0.3	0.2	0.4	1	1	1	0.1	0.0	0.1	0	0	0
Brazil	Brumado	1.7	1.4	2.2	11	9	14	0.2	0.2	0.3	1	1	2
Brazil	Brunópolis	0.2	0.2	0.3	0	0	0	0.1	0.0	0.1	0	0	0
Brazil	Brusque	0.2	0.2	0.3	1	1	2	0.1	0.0	0.1	0	0	1
Brazil	Bueno Brandão	0.3	0.2	0.4	0	0	0	0.1	0.0	0.1	0	0	0
Brazil	Buenópolis	0.3	0.2	0.4	0	0	0	0.1	0.0	0.1	0	0	0
Brazil	Buenos Aires	2.0	1.6	2.6	3	3	4	0.2	0.2	0.3	0	0	0
Brazil	Buerarema	1.7	1.4	2.2	3	3	4	0.2	0.2	0.3	0	0	0
Brazil	Bugre	0.3	0.2	0.4	0	0	0	0.1	0.0	0.1	0	0	0
Brazil	Buíque	2.0	1.6	2.6	15	12	19	0.2	0.2	0.3	2	1	2

Table 3: Diarrhoea Mortality by Unit *(continued)*

Country	Unit	2000 mortality mean rate (per 10,000)	lower rate (per 10,000)	upper rate (per 10,000)	mean count	lower count	upper count	2017 mortality mean rate (per 10,000)	lower rate (per 10,000)	upper rate (per 10,000)	mean count	lower count	upper count
Brazil	Bujari	1.1	0.9	1.3	1	1	1	0.2	0.2	0.3	0	0	0
Brazil	Bujaru	0.9	0.7	1.1	2	2	3	0.1	0.1	0.2	0	0	1
Brazil	Buquim	1.3	1.0	1.7	4	3	5	0.2	0.1	0.3	1	0	1
Brazil	Buri	0.2	0.1	0.2	0	0	0	0.0	0.0	0.0	0	0	0
Brazil	Buritama	0.2	0.1	0.2	0	0	0	0.0	0.0	0.0	0	0	0
Brazil	Buriti	1.7	1.3	2.2	7	5	9	0.2	0.2	0.3	1	1	1
Brazil	Buriti Alegre	0.2	0.1	0.2	0	0	0	0.1	0.0	0.1	0	0	0
Brazil	Buriti Bravo	1.7	1.3	2.2	5	4	6	0.2	0.2	0.3	1	1	1
Brazil	Buriti de Goiás	0.2	0.1	0.2	0	0	0	0.1	0.0	0.1	0	0	0
Brazil	Buriti do Tocantins	0.8	0.7	1.1	1	1	1	0.1	0.1	0.2	0	0	0
Brazil	Buriti dos Lopes	1.4	1.1	1.8	3	2	4	0.2	0.1	0.2	0	0	0
Brazil	Buriti dos Montes	1.4	1.1	1.8	1	1	2	0.2	0.1	0.2	0	0	0
Brazil	Buriticupu	1.7	1.3	2.2	12	9	15	0.2	0.2	0.3	2	1	3
Brazil	Buritinópolis	0.2	0.1	0.2	0	0	0	0.1	0.0	0.1	0	0	0
Brazil	Buritirama	1.7	1.4	2.2	4	3	5	0.2	0.2	0.3	1	0	1
Brazil	Buritirana	1.7	1.3	2.2	3	2	4	0.2	0.2	0.3	0	0	0
Brazil	Buritis	0.3	0.2	0.4	1	0	1	0.1	0.0	0.1	0	0	0
Brazil	Buritis	0.4	0.3	0.5	1	1	1	0.1	0.1	0.1	0	0	0
Brazil	Buritizal	0.2	0.1	0.2	0	0	0	0.0	0.0	0.0	0	0	0
Brazil	Buritizeiro	0.3	0.2	0.4	1	1	1	0.1	0.0	0.1	0	0	0
Brazil	Butiá	0.1	0.1	0.2	0	0	0	0.0	0.0	0.0	0	0	0
Brazil	Caapiranga	0.8	0.6	0.9	1	1	1	0.2	0.2	0.3	0	0	0
Brazil	Caaporã	1.0	0.7	1.2	3	2	3	0.1	0.1	0.1	0	0	0
Brazil	Caarapó	0.5	0.3	0.6	1	1	1	0.2	0.1	0.2	0	0	0
Brazil	Caatiba	1.7	1.4	2.2	3	2	3	0.2	0.2	0.3	0	0	0
Brazil	Cabaceiras	1.0	0.7	1.2	0	0	1	0.1	0.1	0.1	0	0	0
Brazil	Cabaceiras do Paraguaçu	1.7	1.4	2.2	3	2	3	0.2	0.2	0.3	0	0	0
Brazil	Cabeceira Grande	0.3	0.2	0.4	0	0	0	0.1	0.0	0.1	0	0	0
Brazil	Cabeceiras	0.2	0.1	0.2	0	0	0	0.1	0.0	0.1	0	0	0
Brazil	Cabeceiras do Piauí	1.4	1.1	1.8	1	1	2	0.2	0.1	0.2	0	0	0
Brazil	Cabedelo	1.0	0.7	1.2	2	2	3	0.1	0.1	0.1	0	0	0
Brazil	Cabixi	0.4	0.3	0.5	0	0	0	0.1	0.1	0.1	0	0	0
Brazil	Cabo	2.0	1.6	2.6	32	26	42	0.2	0.2	0.3	4	3	5
Brazil	Cabo Frio	0.2	0.1	0.3	1	1	2	0.1	0.0	0.1	1	0	1
Brazil	Cabo Verde	0.3	0.2	0.4	0	0	0	0.1	0.0	0.1	0	0	0
Brazil	Cabrália Paulista	0.2	0.1	0.2	0	0	0	0.0	0.0	0.0	0	0	0
Brazil	Cabreúva	0.2	0.1	0.2	1	0	1	0.0	0.0	0.0	0	0	0
Brazil	Cabrobó	2.0	1.6	2.6	9	7	12	0.2	0.2	0.3	1	1	1
Brazil	Caçador	0.2	0.2	0.3	2	1	2	0.1	0.0	0.1	0	0	0
Brazil	Caçapava	0.2	0.1	0.2	1	1	1	0.0	0.0	0.0	0	0	0
Brazil	Caçapava do Sul	0.1	0.1	0.2	0	0	0	0.0	0.0	0.0	0	0	0
Brazil	Cacaulândia	0.4	0.3	0.5	0	0	0	0.1	0.1	0.1	0	0	0
Brazil	Cacequi	0.1	0.1	0.2	0	0	0	0.0	0.0	0.0	0	0	0
Brazil	Cáceres	0.4	0.3	0.5	4	3	5	0.1	0.1	0.1	1	1	1
Brazil	Cachoeira	1.7	1.4	2.2	7	6	9	0.2	0.2	0.3	1	1	1
Brazil	Cachoeira Alta	0.2	0.1	0.2	0	0	0	0.1	0.0	0.1	0	0	0
Brazil	Cachoeira da Prata	0.3	0.2	0.4	0	0	0	0.1	0.0	0.1	0	0	0
Brazil	Cachoeira de Goias	0.2	0.1	0.2	0	0	0	0.1	0.0	0.1	0	0	0
Brazil	Cachoeira de Minas	0.3	0.2	0.4	0	0	0	0.1	0.0	0.1	0	0	0
Brazil	Cachoeira de Pajes	0.3	0.2	0.4	0	0	0	0.1	0.0	0.1	0	0	0
Brazil	Cachoeira do Arari	0.9	0.7	1.1	2	1	2	0.1	0.1	0.2	0	0	0
Brazil	Cachoeira do Piriá	0.9	0.7	1.1	2	2	3	0.1	0.1	0.2	1	1	1

Table 3: Diarrhoea Mortality by Unit *(continued)*

Country	Unit	2000 mortality						2017 mortality					
		mean rate (per 10,000)	lower rate (per 10,000)	upper rate (per 10,000)	mean count	lower count	upper count	mean rate (per 10,000)	lower rate (per 10,000)	upper rate (per 10,000)	mean count	lower count	upper count
Brazil	Cachoeira do Sul	0.1	0.1	0.2	1	1	1	0.0	0.0	0.0	0	0	0
Brazil	Cachoeira dos índios	1.0	0.7	1.2	1	1	1	0.1	0.1	0.1	0	0	0
Brazil	Cachoeira dourada	0.3	0.2	0.4	0	0	0	0.1	0.0	0.1	0	0	0
Brazil	Cachoeira dourada	0.2	0.1	0.2	0	0	0	0.1	0.0	0.1	0	0	0
Brazil	Cachoeira Grande	1.7	1.3	2.2	3	2	4	0.2	0.2	0.3	0	0	0
Brazil	Cachoeira Paulista	0.2	0.1	0.2	0	0	0	0.0	0.0	0.0	0	0	0
Brazil	Cachoeiras de Macacu	0.2	0.1	0.3	1	1	1	0.1	0.0	0.1	0	0	0
Brazil	Cachoeirinha	0.8	0.7	1.1	0	0	0	0.1	0.1	0.2	0	0	0
Brazil	Cachoeirinha	0.1	0.1	0.2	1	1	1	0.0	0.0	0.0	0	0	0
Brazil	Cachoeiro de Itapemirim	0.3	0.2	0.3	5	3	6	0.1	0.1	0.1	1	1	1
Brazil	Cachoerinha	2.0	1.6	2.6	4	3	5	0.2	0.2	0.3	0	0	0
Brazil	Cacimba de Areia	1.0	0.7	1.2	1	1	1	0.1	0.1	0.1	0	0	0
Brazil	Cacimba de dentro	1.0	0.7	1.2	2	2	3	0.1	0.1	0.1	0	0	0
Brazil	Cacimbas	1.0	0.7	1.2	1	1	1	0.1	0.1	0.1	0	0	0
Brazil	Cacimbinhas	2.8	2.2	3.7	4	3	5	0.3	0.2	0.4	0	0	0
Brazil	Cacique doble	0.1	0.1	0.2	0	0	0	0.0	0.0	0.0	0	0	0
Brazil	Cacoal	0.4	0.3	0.5	3	2	4	0.1	0.1	0.1	1	0	1
Brazil	Caconde	0.3	0.2	0.4	0	0	1	0.1	0.0	0.1	0	0	0
Brazil	Caçu	0.2	0.1	0.2	0	0	0	0.1	0.0	0.1	0	0	0
Brazil	Caculé	1.7	1.4	2.2	4	3	5	0.2	0.2	0.3	0	0	1
Brazil	Caém	1.7	1.4	2.2	2	2	3	0.2	0.2	0.3	0	0	0
Brazil	Caetanópolis	0.3	0.2	0.4	0	0	0	0.1	0.0	0.1	0	0	0
Brazil	Caetanos	1.7	1.4	2.2	3	2	4	0.2	0.2	0.3	0	0	0
Brazil	Caeté	0.3	0.2	0.4	1	1	1	0.1	0.0	0.1	0	0	0
Brazil	Caetés	2.0	1.6	2.6	5	4	7	0.2	0.2	0.3	1	0	1
Brazil	Caetité	1.7	1.4	2.2	9	7	11	0.2	0.2	0.3	1	1	1
Brazil	Cafarnaum	1.7	1.4	2.2	4	3	5	0.2	0.2	0.3	0	0	1
Brazil	Cafeara	0.3	0.2	0.3	0	0	0	0.1	0.0	0.1	0	0	0
Brazil	Cafelândia	0.3	0.2	0.3	0	0	0	0.1	0.0	0.1	0	0	0
Brazil	Cafelândia	0.2	0.1	0.2	0	0	0	0.0	0.0	0.0	0	0	0
Brazil	Cafezal do Sul	0.3	0.2	0.3	0	0	0	0.1	0.0	0.1	0	0	0
Brazil	Caiabu	0.2	0.1	0.2	0	0	0	0.0	0.0	0.0	0	0	0
Brazil	Caiana	0.3	0.2	0.4	0	0	0	0.1	0.0	0.1	0	0	0
Brazil	Caiapônia	0.2	0.1	0.2	0	0	0	0.1	0.0	0.1	0	0	0
Brazil	Caibaté	0.1	0.1	0.2	0	0	0	0.0	0.0	0.0	0	0	0
Brazil	Caibi	0.2	0.2	0.3	0	0	0	0.1	0.0	0.1	0	0	0
Brazil	Caiçara	1.0	0.7	1.2	0	0	1	0.1	0.1	0.1	0	0	0
Brazil	Caiçara	0.1	0.1	0.2	0	0	0	0.0	0.0	0.0	0	0	0
Brazil	Caiçara do Norte	1.2	0.9	1.5	1	0	1	0.1	0.1	0.2	0	0	0
Brazil	Caiçara do Rio do Vento	1.2	0.9	1.5	7	5	9	0.1	0.1	0.2	1	1	1
Brazil	Caicó	1.2	0.9	1.5	0	0	0	0.1	0.1	0.2	0	0	0
Brazil	Caieiras	0.2	0.1	0.2	1	1	2	0.0	0.0	0.0	0	0	0
Brazil	Cairu	1.7	1.4	2.2	2	2	3	0.2	0.2	0.3	0	0	1
Brazil	Caiuá	0.2	0.1	0.2	0	0	0	0.0	0.0	0.0	0	0	0
Brazil	Cajamar	0.2	0.1	0.2	1	0	1	0.0	0.0	0.0	0	0	0
Brazil	Cajapió	1.7	1.3	2.2	2	1	2	0.2	0.2	0.3	0	0	0
Brazil	Cajari	1.7	1.3	2.2	3	2	3	0.2	0.2	0.3	0	0	1
Brazil	Cajati	0.2	0.1	0.2	0	0	1	0.0	0.0	0.0	0	0	0
Brazil	Cajazeiras	1.0	0.7	1.2	5	4	7	0.1	0.1	0.1	1	0	1
Brazil	Cajazeiras do Piauí	1.4	1.1	1.8	0	0	0	0.2	0.1	0.2	0	0	0
Brazil	Cajazeirinhas	1.0	0.7	1.2	0	0	0	0.1	0.1	0.1	0	0	0

Table 3: Diarrhoea Mortality by Unit *(continued)*

Country	Unit	2000 mortality mean rate (per 10,000)	lower rate (per 10,000)	upper rate (per 10,000)	mean count	lower count	upper count	2017 mortality mean rate (per 10,000)	lower rate (per 10,000)	upper rate (per 10,000)	mean count	lower count	upper count
Brazil	Cajobi	0.2	0.1	0.2	0	0	0	0.0	0.0	0.0	0	0	0
Brazil	Cajueiro	2.8	2.2	3.7	8	6	10	0.3	0.2	0.4	1	1	1
Brazil	Cajueiro da Praia	1.4	1.1	1.8	1	0	1	0.2	0.1	0.2	0	0	0
Brazil	Cajuri	0.3	0.2	0.4	0	0	0	0.1	0.0	0.1	0	0	0
Brazil	Cajuru	0.2	0.1	0.2	0	0	0	0.0	0.0	0.0	0	0	0
Brazil	Calçado	2.0	1.6	2.6	3	2	4	0.2	0.2	0.3	0	0	0
Brazil	Calçoene	0.2	0.2	0.3	0	0	0	0.1	0.1	0.2	0	0	0
Brazil	Caldas	0.3	0.2	0.4	0	0	0	0.1	0.0	0.1	0	0	0
Brazil	Caldas Brandão	1.0	0.7	1.2	0	0	0	0.1	0.1	0.1	0	0	0
Brazil	Caldas Novas	0.2	0.1	0.2	1	1	1	0.1	0.0	0.1	0	0	0
Brazil	Caldazinha	0.2	0.1	0.2	0	0	0	0.1	0.0	0.1	0	0	0
Brazil	Caldeirão Grande	1.7	1.4	2.2	2	2	3	0.2	0.2	0.3	0	0	0
Brazil	Caldeirão Grande do Piauí	1.4	1.1	1.8	1	1	2	0.2	0.1	0.2	0	0	0
Brazil	Califórnia	0.3	0.2	0.3	0	0	0	0.1	0.0	0.1	0	0	0
Brazil	Calmon	0.2	0.2	0.3	0	0	0	0.1	0.0	0.1	0	0	0
Brazil	Calumbi	2.0	1.6	2.6	2	2	3	0.2	0.2	0.3	0	0	0
Brazil	Camacan	1.7	1.4	2.2	6	5	8	0.2	0.2	0.3	1	1	1
Brazil	Camaçari	1.7	1.4	2.2	35	27	45	0.2	0.2	0.3	8	6	11
Brazil	Camacho	0.3	0.2	0.4	0	0	0	0.1	0.0	0.1	0	0	0
Brazil	Camagua	0.1	0.1	0.2	1	0	1	0.0	0.0	0.0	0	0	0
Brazil	Camalaú	1.0	0.7	1.2	1	0	1	0.1	0.1	0.1	0	0	0
Brazil	Camamu	1.7	1.4	2.2	9	7	11	0.2	0.2	0.3	1	1	1
Brazil	Camanducaia	0.3	0.2	0.4	1	0	1	0.1	0.0	0.1	0	0	0
Brazil	Camapuã	0.5	0.3	0.6	1	0	1	0.2	0.1	0.2	0	0	0
Brazil	Camaragibe	2.0	1.6	2.6	24	19	32	0.2	0.2	0.3	2	2	3
Brazil	Camargo	0.1	0.1	0.2	0	0	0	0.0	0.0	0.0	0	0	0
Brazil	Cambará	0.3	0.2	0.3	1	0	1	0.1	0.0	0.1	0	0	0
Brazil	Cambará do Sul	0.1	0.1	0.2	0	0	0	0.0	0.0	0.0	0	0	0
Brazil	Cambé	0.3	0.2	0.3	2	1	2	0.1	0.0	0.1	0	0	0
Brazil	Cambira	0.3	0.2	0.3	0	0	0	0.1	0.0	0.1	0	0	0
Brazil	Camboriú	0.2	0.2	0.3	1	1	1	0.1	0.0	0.1	0	0	0
Brazil	Cambuci	0.2	0.1	0.3	0	0	0	0.1	0.0	0.1	0	0	0
Brazil	Cambuí	0.3	0.2	0.4	0	0	1	0.1	0.0	0.1	0	0	0
Brazil	Cambuquira	0.3	0.2	0.4	0	0	0	0.1	0.0	0.1	0	0	0
Brazil	Cametá	0.9	0.7	1.1	12	9	15	0.1	0.1	0.2	2	2	3
Brazil	Camocim	2.3	1.7	2.9	16	12	21	0.1	0.1	0.2	1	1	1
Brazil	Camocim de São Félix	2.0	1.6	2.6	3	2	4	0.2	0.2	0.3	0	0	0
Brazil	Campanário	0.3	0.2	0.4	0	0	0	0.1	0.0	0.1	0	0	0
Brazil	Campanha	0.3	0.2	0.4	0	0	0	0.1	0.0	0.1	0	0	0
Brazil	Campestre	0.3	0.2	0.4	0	0	1	0.1	0.0	0.1	0	0	0
Brazil	Campestre	2.8	2.2	3.7	3	2	4	0.3	0.2	0.4	0	0	0
Brazil	Campestre da Serra	0.1	0.1	0.2	0	0	0	0.0	0.0	0.0	0	0	0
Brazil	Campestre de Goiás	0.2	0.1	0.2	0	0	0	0.1	0.0	0.1	0	0	0
Brazil	Campestre do Maranhão	1.7	1.3	2.2	3	2	4	0.2	0.2	0.3	0	0	1
Brazil	Campina da Lagoa	0.3	0.2	0.3	0	0	0	0.1	0.0	0.1	0	0	0
Brazil	Campina das Missões	0.1	0.1	0.2	0	0	0	0.0	0.0	0.0	0	0	0
Brazil	Campina do Monte Alegre	0.2	0.1	0.2	0	0	0	0.0	0.0	0.0	0	0	0
Brazil	Campina do Simão	0.3	0.2	0.3	0	0	0	0.1	0.0	0.1	0	0	0
Brazil	Campina Grande	1.0	0.7	1.2	36	28	46	0.1	0.1	0.1	4	3	5
Brazil	Campina Grande do Sul	0.3	0.2	0.3	1	1	1	0.1	0.0	0.1	0	0	0

Table 3: Diarrhoea Mortality by Unit *(continued)*

Country	Unit	2000 mortality: mean rate (per 10,000)	lower rate (per 10,000)	upper rate (per 10,000)	mean count	lower count	upper count	2017 mortality: mean rate (per 10,000)	lower rate (per 10,000)	upper rate (per 10,000)	mean count	lower count	upper count
Brazil	Campina Verde	0.3	0.2	0.4	0	0	0	0.1	0.0	0.1	0	0	0
Brazil	Campinaçu	0.2	0.1	0.2	0	0	0	0.1	0.0	0.1	0	0	0
Brazil	Campinápolis	0.4	0.3	0.5	1	0	1	0.1	0.1	0.1	0	0	0
Brazil	Campinas	0.2	0.1	0.2	12	9	15	0.0	0.0	0.0	3	2	3
Brazil	Campinas do Piauí	1.4	1.1	1.8	1	1	1	0.2	0.1	0.2	0	0	0
Brazil	Campinas do Sul	0.1	0.1	0.2	0	0	0	0.0	0.0	0.0	0	0	0
Brazil	Campinorte	0.2	0.1	0.2	0	0	0	0.1	0.0	0.1	0	0	0
Brazil	Campo	0.3	0.2	0.3	2	2	3	0.1	0.0	0.1	0	0	1
Brazil	Campo Alegre	2.8	2.2	3.7	14	11	18	0.3	0.2	0.4	2	1	2
Brazil	Campo Alegre	0.2	0.2	0.3	0	0	0	0.1	0.0	0.1	0	0	0
Brazil	Campo Alegre de Goiás	0.2	0.1	0.2	0	0	0	0.1	0.0	0.1	0	0	0
Brazil	Campo Alegre de Lourdes	1.7	1.4	2.2	6	5	8	0.2	0.2	0.3	1	1	1
Brazil	Campo Alegre do Fidalgo	1.4	1.1	1.8	1	1	1	0.2	0.1	0.2	0	0	0
Brazil	Campo Azul	0.3	0.2	0.4	0	0	0	0.1	0.0	0.1	0	0	0
Brazil	Campo Belo	0.3	0.2	0.4	1	1	1	0.1	0.0	0.1	0	0	0
Brazil	Campo Belo do Sul	0.2	0.2	0.3	0	0	0	0.1	0.0	0.1	0	0	0
Brazil	Campo Bom	0.1	0.1	0.2	1	1	1	0.0	0.0	0.0	0	0	0
Brazil	Campo Bonito	0.3	0.2	0.3	0	0	0	0.1	0.0	0.1	0	0	0
Brazil	Campo do Brito	1.3	1.0	1.7	3	2	3	0.2	0.1	0.3	0	0	0
Brazil	Campo do Meio	0.3	0.2	0.4	0	0	0	0.1	0.0	0.1	0	0	0
Brazil	Campo do Tenente	0.3	0.2	0.3	0	0	0	0.1	0.0	0.1	0	0	0
Brazil	Campo Erê	0.2	0.2	0.3	0	0	0	0.1	0.0	0.1	0	0	0
Brazil	Campo Florido	0.3	0.2	0.4	0	0	0	0.1	0.0	0.1	0	0	0
Brazil	Campo Formoso	1.7	1.4	2.2	12	9	15	0.2	0.2	0.3	1	1	2
Brazil	Campo Grande	2.8	2.2	3.7	4	3	5	0.3	0.2	0.4	0	0	0
Brazil	Campo Grande	0.5	0.3	0.6	27	21	36	0.2	0.1	0.2	9	7	11
Brazil	Campo Grande do Piauí	1.4	1.1	1.8	1	1	1	0.2	0.1	0.2	0	0	0
Brazil	Campo Largo do Piauí	1.4	1.1	1.8	1	1	1	0.2	0.1	0.2	0	0	0
Brazil	Campo Limpo Paulista	0.2	0.1	0.2	1	1	1	0.0	0.0	0.0	0	0	0
Brazil	Campo Magro	0.3	0.2	0.3	1	0	1	0.1	0.0	0.1	0	0	0
Brazil	Campo Maior	1.4	1.1	1.8	6	5	8	0.2	0.1	0.2	1	0	1
Brazil	Campo Mourão	0.3	0.2	0.3	2	1	2	0.1	0.0	0.1	0	0	0
Brazil	Campo Novo	0.1	0.1	0.2	0	0	0	0.0	0.0	0.0	0	0	0
Brazil	Campo Novo de Rondônia	0.4	0.3	0.5	1	0	1	0.1	0.1	0.1	0	0	0
Brazil	Campo Novo do Parecis	0.4	0.3	0.5	1	1	1	0.1	0.1	0.1	0	0	0
Brazil	Campo Real	0.1	0.1	0.2	0	0	0	0.0	0.0	0.0	0	0	0
Brazil	Campo Redondo	1.2	0.9	1.5	1	1	2	0.1	0.1	0.2	0	0	0
Brazil	Campo Verde	0.4	0.3	0.5	1	1	1	0.1	0.1	0.1	0	0	0
Brazil	Campos	0.2	0.1	0.3	7	5	9	0.1	0.0	0.1	2	2	3
Brazil	Campos Altos	0.3	0.2	0.4	0	0	0	0.1	0.0	0.1	0	0	0
Brazil	Campos Belos	0.2	0.1	0.2	0	0	0	0.1	0.0	0.1	0	0	0
Brazil	Campos Borges	0.1	0.1	0.2	0	0	0	0.0	0.0	0.0	0	0	0
Brazil	Campos de Júlio	0.4	0.3	0.5	0	0	0	0.1	0.1	0.1	0	0	0
Brazil	Campos do Jordão	0.2	0.1	0.2	1	1	1	0.0	0.0	0.0	0	0	0
Brazil	Campos Gerais	0.3	0.2	0.4	1	1	1	0.1	0.0	0.1	0	0	0
Brazil	Campos Lindos	0.8	0.7	1.1	1	1	1	0.1	0.1	0.2	0	0	0
Brazil	Campos Novos	0.2	0.2	0.3	1	0	1	0.1	0.0	0.1	0	0	0
Brazil	Campos Novos Paulista	0.2	0.1	0.2	0	0	0	0.0	0.0	0.0	0	0	0

Table 3: Diarrhoea Mortality by Unit *(continued)*

Country	Unit	2000 mortality						2017 mortality					
		mean rate (per 10,000)	lower rate (per 10,000)	upper rate (per 10,000)	mean count	lower count	upper count	mean rate (per 10,000)	lower rate (per 10,000)	upper rate (per 10,000)	mean count	lower count	upper count
Brazil	Campos Sales	2.3	1.7	2.9	8	6	10	0.1	0.1	0.2	0	0	1
Brazil	Campos Verdes	0.2	0.1	0.2	0	0	0	0.1	0.0	0.1	0	0	0
Brazil	Campos Verdes de Goiás	0.3	0.2	0.4	0	0	0	0.1	0.0	0.1	0	0	0
Brazil	Camutanga	2.0	1.6	2.6	1	1	1	0.2	0.2	0.3	0	0	0
Brazil	Canaã	0.3	0.2	0.4	0	0	0	0.1	0.0	0.1	0	0	0
Brazil	Canaã dos Carajás	0.9	0.7	1.1	1	1	1	0.1	0.1	0.2	1	1	1
Brazil	CanaBrava do Norte	0.4	0.3	0.5	0	0	0	0.1	0.1	0.1	0	0	0
Brazil	Cananéia	0.2	0.1	0.2	0	0	1	0.0	0.0	0.0	0	0	0
Brazil	Canapi	2.8	2.2	3.7	7	5	9	0.3	0.2	0.4	1	0	1
Brazil	Canápolis	0.3	0.2	0.4	0	0	0	0.1	0.0	0.1	0	0	0
Brazil	Canápolis	1.7	1.4	2.2	2	2	3	0.2	0.2	0.3	0	0	0
Brazil	Canarana	0.4	0.3	0.5	1	1	1	0.1	0.1	0.1	0	0	0
Brazil	Canarana	1.7	1.4	2.2	5	4	7	0.2	0.2	0.3	1	1	1
Brazil	Canas	0.2	0.1	0.2	0	0	0	0.0	0.0	0.0	0	0	0
Brazil	Canavieira	1.4	1.1	1.8	1	0	1	0.2	0.1	0.2	0	0	0
Brazil	Canavieiras	1.7	1.4	2.2	2	1	2	0.2	0.2	0.3	0	0	0
Brazil	Candeal	1.7	1.4	2.2	2	2	3	0.2	0.2	0.3	0	0	0
Brazil	Candeias	0.3	0.2	0.4	0	0	0	0.1	0.0	0.1	0	0	0
Brazil	Candeias	1.7	1.4	2.2	14	11	18	0.2	0.2	0.3	2	1	3
Brazil	Candeias do Jamari	0.4	0.3	0.5	1	0	1	0.1	0.1	0.1	0	0	0
Brazil	Candelária	0.1	0.1	0.2	0	0	0	0.0	0.0	0.0	0	0	0
Brazil	Candiba	1.7	1.4	2.2	2	2	3	0.2	0.2	0.3	0	0	0
Brazil	Cândido de Abreu	0.3	0.2	0.3	1	0	1	0.1	0.0	0.1	0	0	0
Brazil	Cândido Godói	0.1	0.1	0.2	0	0	0	0.0	0.0	0.0	0	0	0
Brazil	Cândido Mendes	1.7	1.3	2.2	4	3	5	0.2	0.2	0.3	1	0	1
Brazil	Cândido Mota	0.2	0.1	0.2	0	0	1	0.0	0.0	0.0	0	0	0
Brazil	Cândido Rodrigues	0.2	0.1	0.2	0	0	0	0.0	0.0	0.0	0	0	0
Brazil	Cândido Sales	1.7	1.4	2.2	3	2	4	0.2	0.2	0.3	0	0	0
Brazil	Candiota	0.1	0.1	0.2	0	0	0	0.0	0.0	0.0	0	0	0
Brazil	Candói	0.3	0.2	0.3	0	0	1	0.1	0.0	0.1	0	0	0
Brazil	Canela	0.1	0.1	0.2	0	0	0	0.0	0.0	0.0	0	0	0
Brazil	Canelinha	0.2	0.2	0.3	0	0	0	0.1	0.0	0.1	0	0	0
Brazil	Canguaretama	1.2	0.9	1.5	4	3	6	0.1	0.1	0.2	0	0	1
Brazil	Canguçu	0.1	0.1	0.2	0	0	1	0.0	0.0	0.0	0	0	0
Brazil	Canhoba	1.3	1.0	1.7	1	1	1	0.2	0.1	0.3	0	0	0
Brazil	Canhotinho	2.0	1.6	2.6	7	5	9	0.2	0.2	0.3	1	0	1
Brazil	Canindé	2.3	1.7	2.9	21	16	27	0.1	0.1	0.2	1	1	2
Brazil	Canindé de São Francisco	1.3	1.0	1.7	3	2	4	0.2	0.1	0.3	1	0	1
Brazil	Canitar	0.2	0.1	0.2	0	0	0	0.0	0.0	0.0	0	0	0
Brazil	Canoas	0.1	0.1	0.2	3	2	4	0.0	0.0	0.0	1	1	1
Brazil	Canoinhas	0.2	0.2	0.3	1	1	1	0.1	0.0	0.1	0	0	0
Brazil	Cansanção	1.7	1.4	2.2	8	6	10	0.2	0.2	0.3	1	1	1
Brazil	Cantá	0.7	0.6	0.9	1	1	2	0.2	0.2	0.3	1	0	1
Brazil	Cantagalo	0.3	0.2	0.4	0	0	0	0.1	0.0	0.1	0	0	0
Brazil	Cantagalo	0.2	0.1	0.3	0	0	0	0.1	0.0	0.1	0	0	0
Brazil	Cantagalo	0.3	0.2	0.3	0	0	0	0.1	0.0	0.1	0	0	0
Brazil	Cantanhede	1.7	1.3	2.2	5	4	6	0.2	0.2	0.3	1	0	1
Brazil	Canto do Buriti	1.4	1.1	1.8	3	2	3	0.2	0.1	0.2	0	0	0
Brazil	Canudos	1.7	1.4	2.2	3	2	4	0.2	0.2	0.3	0	0	1
Brazil	Canutama	0.8	0.6	0.9	1	1	2	0.2	0.2	0.3	0	0	1
Brazil	Capanema	0.9	0.7	1.1	5	4	6	0.1	0.1	0.2	1	1	1
Brazil	Capanema	0.3	0.2	0.3	0	0	0	0.1	0.0	0.1	0	0	0
Brazil	Capão Alto	0.2	0.2	0.3	0	0	0	0.1	0.0	0.1	0	0	0

Table 3: Diarrhoea Mortality by Unit *(continued)*

Country	Unit	2000 mortality mean rate (per 10,000)	lower rate (per 10,000)	upper rate (per 10,000)	mean count	lower count	upper count	2017 mortality mean rate (per 10,000)	lower rate (per 10,000)	upper rate (per 10,000)	mean count	lower count	upper count
Brazil	Capão Bonito	0.2	0.1	0.2	1	1	1	0.0	0.0	0.0	0	0	0
Brazil	Capão da Canoa	0.1	0.1	0.2	0	0	0	0.0	0.0	0.0	0	0	0
Brazil	Capão do Leão	0.1	0.1	0.2	0	0	0	0.0	0.0	0.0	0	0	0
Brazil	Caparaó	0.3	0.2	0.4	0	0	0	0.1	0.0	0.1	0	0	0
Brazil	Capela	1.3	1.0	1.7	4	3	5	0.2	0.1	0.3	1	0	1
Brazil	Capela	2.8	2.2	3.7	6	5	8	0.3	0.2	0.4	1	0	1
Brazil	Capela de Santana	0.1	0.1	0.2	0	0	0	0.0	0.0	0.0	0	0	0
Brazil	Capela do Alto	0.2	0.1	0.2	0	0	0	0.0	0.0	0.0	0	0	0
Brazil	Capela do Alto Alegre	1.7	1.4	2.2	2	2	3	0.2	0.2	0.3	0	0	0
Brazil	Capela Nova	0.3	0.2	0.4	0	0	0	0.1	0.0	0.1	0	0	0
Brazil	Capelinha	0.3	0.2	0.4	1	1	1	0.1	0.0	0.1	0	0	0
Brazil	Capetinga	0.3	0.2	0.4	0	0	0	0.1	0.0	0.1	0	0	0
Brazil	Capim	1.0	0.7	1.2	1	0	1	0.1	0.1	0.1	0	0	0
Brazil	Capim Branco	0.3	0.2	0.4	0	0	0	0.1	0.0	0.1	0	0	0
Brazil	Capim Grosso	1.7	1.4	2.2	5	4	7	0.2	0.2	0.3	1	1	1
Brazil	Capinópolis	0.3	0.2	0.4	0	0	0	0.1	0.0	0.1	0	0	0
Brazil	Capinzal	0.2	0.2	0.3	1	0	1	0.1	0.0	0.1	0	0	0
Brazil	Capinzal do Norte	1.7	1.3	2.2	3	2	3	0.2	0.2	0.3	0	0	0
Brazil	Capistrano	2.3	1.7	2.9	4	3	6	0.1	0.1	0.2	0	0	0
Brazil	Capitão	0.1	0.1	0.2	0	0	0	0.0	0.0	0.0	0	0	0
Brazil	Capitão Andrade	0.3	0.2	0.4	0	0	0	0.1	0.0	0.1	0	0	0
Brazil	Capitão de Campos	1.4	1.1	1.8	1	1	2	0.2	0.1	0.2	0	0	0
Brazil	Capitão Enéas	0.3	0.2	0.4	0	0	1	0.1	0.0	0.1	0	0	0
Brazil	Capitão Gervásio Oliveira	1.4	1.1	1.8	1	1	1	0.2	0.1	0.2	0	0	0
Brazil	Capitão Leônidas Marques	0.3	0.2	0.3	0	0	0	0.1	0.0	0.1	0	0	0
Brazil	Capitão Poço	0.9	0.7	1.1	5	4	7	0.1	0.1	0.2	1	1	1
Brazil	Capitólio	0.3	0.2	0.4	0	0	0	0.1	0.0	0.1	0	0	0
Brazil	Capivari	0.2	0.1	0.2	1	0	1	0.0	0.0	0.0	0	0	0
Brazil	Capivari de Baixo	0.2	0.2	0.3	0	0	0	0.1	0.0	0.1	0	0	0
Brazil	Capivari do Sul	0.1	0.1	0.2	0	0	0	0.0	0.0	0.0	0	0	0
Brazil	Capixaba	1.1	0.9	1.3	1	1	1	0.2	0.2	0.3	0	0	0
Brazil	Capoeiras	2.0	1.6	2.6	6	5	8	0.2	0.2	0.3	1	0	1
Brazil	Caputira	0.3	0.2	0.4	0	0	0	0.1	0.0	0.1	0	0	0
Brazil	Caraá	0.1	0.1	0.2	0	0	0	0.0	0.0	0.0	0	0	0
Brazil	Caracaraí	0.7	0.6	0.9	1	1	2	0.2	0.2	0.3	0	0	1
Brazil	Caracol	0.5	0.3	0.6	0	0	0	0.2	0.1	0.2	0	0	0
Brazil	Caracol	1.4	1.1	1.8	1	1	2	0.2	0.1	0.2	0	0	0
Brazil	Caraguatatuba	0.2	0.1	0.2	1	1	1	0.0	0.0	0.0	0	0	0
Brazil	Caraí	0.3	0.2	0.4	1	1	1	0.1	0.0	0.1	0	0	0
Brazil	Caraíbas	1.7	1.4	2.2	4	3	5	0.2	0.2	0.3	0	0	0
Brazil	Carambeí	0.3	0.2	0.3	0	0	1	0.1	0.0	0.1	0	0	0
Brazil	Caranaíba	0.3	0.2	0.4	0	0	0	0.1	0.0	0.1	0	0	0
Brazil	Carandaí	0.3	0.2	0.4	0	0	1	0.1	0.0	0.1	0	0	0
Brazil	Carangola	0.3	0.2	0.4	1	1	1	0.1	0.0	0.1	0	0	0
Brazil	Carapicuíba	0.2	0.1	0.2	4	3	5	0.0	0.0	0.0	1	1	1
Brazil	Caratinga	0.3	0.2	0.4	2	2	3	0.1	0.0	0.1	0	0	1
Brazil	Carauari	0.8	0.6	0.9	3	2	3	0.2	0.2	0.3	1	1	1
Brazil	Caraúbas	1.2	0.9	1.5	3	2	3	0.1	0.1	0.2	0	0	0
Brazil	Caraúbas	1.0	0.7	1.2	0	0	0	0.1	0.1	0.1	0	0	0
Brazil	Caraúbas do Piauí	1.4	1.1	1.8	1	1	1	0.2	0.1	0.2	0	0	0
Brazil	Caravalhopolis	0.3	0.2	0.4	0	0	0	0.1	0.0	0.1	0	0	0
Brazil	Caravelas	1.7	1.4	2.2	4	3	6	0.2	0.2	0.3	1	0	1
Brazil	Carazinho	0.1	0.1	0.2	1	0	1	0.0	0.0	0.0	0	0	0

Table 3: Diarrhoea Mortality by Unit *(continued)*

Country	Unit	2000 mortality mean rate (per 10,000)	lower rate (per 10,000)	upper rate (per 10,000)	mean count	lower count	upper count	2017 mortality mean rate (per 10,000)	lower rate (per 10,000)	upper rate (per 10,000)	mean count	lower count	upper count
Brazil	Carbonita	0.3	0.2	0.4	0	0	0	0.1	0.0	0.1	0	0	0
Brazil	Cardeal da Silva	1.7	1.4	2.2	1	1	2	0.2	0.2	0.3	0	0	0
Brazil	Cardoso	0.2	0.1	0.2	0	0	0	0.0	0.0	0.0	0	0	0
Brazil	Cardoso Moreira	0.2	0.1	0.3	0	0	0	0.1	0.0	0.1	0	0	0
Brazil	Careaçu	0.3	0.2	0.4	0	0	0	0.1	0.0	0.1	0	0	0
Brazil	Careiro	0.8	0.6	0.9	2	2	3	0.2	0.2	0.3	1	1	1
Brazil	Careiro da Várzea	0.8	0.6	0.9	1	1	2	0.2	0.2	0.3	1	0	1
Brazil	Carepebus	0.2	0.1	0.3	0	0	0	0.1	0.0	0.1	0	0	0
Brazil	Cariacica	0.3	0.2	0.3	8	6	10	0.1	0.1	0.1	2	1	2
Brazil	Caridade	2.3	1.7	2.9	5	4	6	0.1	0.1	0.2	0	0	0
Brazil	Caridade do Piauí	1.4	1.1	1.8	1	1	1	0.2	0.1	0.2	0	0	0
Brazil	Carinhanha	1.7	1.4	2.2	6	4	7	0.2	0.2	0.3	1	0	1
Brazil	Carira	1.3	1.0	1.7	3	2	3	0.2	0.1	0.3	0	0	0
Brazil	Cariré	2.3	1.7	2.9	6	5	8	0.1	0.1	0.2	0	0	0
Brazil	Cariri do Tocantins	0.8	0.7	1.1	0	0	0	0.1	0.1	0.2	0	0	0
Brazil	Caririaçú	2.3	1.7	2.9	7	5	9	0.1	0.1	0.2	0	0	1
Brazil	Cariús	2.3	1.7	2.9	5	4	6	0.1	0.1	0.2	0	0	0
Brazil	Carlinda	0.4	0.3	0.5	1	0	1	0.1	0.1	0.1	0	0	0
Brazil	Carlópolis	0.3	0.2	0.3	0	0	0	0.1	0.0	0.1	0	0	0
Brazil	Carlos Barbosa	0.1	0.1	0.2	0	0	0	0.0	0.0	0.0	0	0	0
Brazil	Carlos Chagas	0.3	0.2	0.4	1	0	1	0.1	0.0	0.1	0	0	0
Brazil	Carlos Gomes	0.1	0.1	0.2	0	0	0	0.0	0.0	0.0	0	0	0
Brazil	Carmésia	0.3	0.2	0.4	0	0	0	0.1	0.0	0.1	0	0	0
Brazil	Carmo	0.2	0.1	0.3	0	0	0	0.1	0.0	0.1	0	0	0
Brazil	Carmo da Cachoeira	0.3	0.2	0.4	0	0	0	0.1	0.0	0.1	0	0	0
Brazil	Carmo da Mata	0.3	0.2	0.4	0	0	0	0.1	0.0	0.1	0	0	0
Brazil	Carmo de Minas	0.3	0.2	0.4	0	0	1	0.1	0.0	0.1	0	0	0
Brazil	Carmo do Cajuru	0.3	0.2	0.4	0	0	1	0.1	0.0	0.1	0	0	0
Brazil	Carmo do Paranaiba	0.3	0.2	0.4	1	0	1	0.1	0.0	0.1	0	0	0
Brazil	Carmo do Rio Claro	0.3	0.2	0.4	0	0	1	0.1	0.0	0.1	0	0	0
Brazil	Carmo do Rio Verde	0.2	0.1	0.2	0	0	0	0.1	0.0	0.1	0	0	0
Brazil	Carmolândia	0.8	0.7	1.1	0	0	0	0.1	0.1	0.2	0	0	0
Brazil	Carmópolis	1.3	1.0	1.7	1	1	2	0.2	0.1	0.3	0	0	0
Brazil	Carmópolis de Minas	0.3	0.2	0.4	0	0	0	0.1	0.0	0.1	0	0	0
Brazil	Carnaíba	2.0	1.6	2.6	4	3	6	0.2	0.2	0.3	0	0	1
Brazil	Carnaúba dos Dantas	1.2	0.9	1.5	1	1	1	0.1	0.1	0.2	0	0	0
Brazil	Carnaubais	1.2	0.9	1.5	1	1	2	0.1	0.1	0.2	0	0	0
Brazil	Carnaubal	2.3	1.7	2.9	4	3	5	0.1	0.1	0.2	0	0	0
Brazil	Carnaubeira da Penha	2.0	1.6	2.6	3	2	4	0.2	0.2	0.3	0	0	0
Brazil	Carneirinho	0.3	0.2	0.4	0	0	0	0.1	0.0	0.1	0	0	0
Brazil	Carneiros	2.8	2.2	3.7	4	3	5	0.3	0.2	0.4	0	0	1
Brazil	Caroebe	0.7	0.6	0.9	1	0	1	0.2	0.2	0.3	0	0	0
Brazil	Carolina	1.7	1.3	2.2	6	5	8	0.2	0.2	0.3	1	1	1
Brazil	Carpina	2.0	1.6	2.6	12	9	16	0.2	0.2	0.3	1	1	2
Brazil	Carrancas	0.3	0.2	0.4	0	0	0	0.1	0.0	0.1	0	0	0
Brazil	Carrapateira	1.0	0.7	1.2	0	0	0	0.1	0.1	0.1	0	0	0
Brazil	Carrasco Bonito	0.8	0.7	1.1	0	0	0	0.1	0.1	0.2	0	0	0
Brazil	Caruaru	2.0	1.6	2.6	61	48	79	0.2	0.2	0.3	8	6	11
Brazil	Carutapera	1.7	1.3	2.2	5	4	7	0.2	0.2	0.3	1	1	1
Brazil	Carvalhos	0.3	0.2	0.4	0	0	0	0.1	0.0	0.1	0	0	0
Brazil	Casa Branca	0.2	0.1	0.2	0	0	0	0.0	0.0	0.0	0	0	0

Table 3: Diarrhoea Mortality by Unit *(continued)*

Country	Unit	2000 mortality mean rate (per 10,000)	lower rate (per 10,000)	upper rate (per 10,000)	mean count	lower count	upper count	2017 mortality mean rate (per 10,000)	lower rate (per 10,000)	upper rate (per 10,000)	mean count	lower count	upper count
Brazil	Casa Grande	0.3	0.2	0.4	0	0	0	0.1	0.0	0.1	0	0	0
Brazil	Casa Nova	1.7	1.4	2.2	13	10	16	0.2	0.2	0.3	2	2	3
Brazil	Casca	0.1	0.1	0.2	0	0	0	0.0	0.0	0.0	0	0	0
Brazil	Cascalho Rico	0.3	0.2	0.4	0	0	0	0.1	0.0	0.1	0	0	0
Brazil	Cascavel	2.3	1.7	2.9	15	11	19	0.1	0.1	0.2	1	1	1
Brazil	Cascavel	0.3	0.2	0.3	6	5	8	0.1	0.0	0.1	1	1	2
Brazil	Caseara	0.8	0.7	1.1	0	0	0	0.1	0.1	0.2	0	0	0
Brazil	Caseiros	0.1	0.1	0.2	0	0	0	0.0	0.0	0.0	0	0	0
Brazil	Casimiro de Abreu	0.2	0.1	0.3	0	0	0	0.1	0.0	0.1	0	0	0
Brazil	Casinhas	2.0	1.6	2.6	3	3	4	0.2	0.2	0.3	0	0	0
Brazil	Casserengue	1.0	0.7	1.2	1	0	1	0.1	0.1	0.1	0	0	0
Brazil	Cássia	0.3	0.2	0.4	0	0	1	0.1	0.0	0.1	0	0	0
Brazil	Cássia dos Coqueiros	0.2	0.1	0.2	0	0	0	0.0	0.0	0.0	0	0	0
Brazil	Cassilândia	0.5	0.3	0.6	1	0	1	0.2	0.1	0.2	0	0	0
Brazil	Cassiterita	0.3	0.2	0.4	0	0	0	0.1	0.0	0.1	0	0	0
Brazil	Castanhal	0.9	0.7	1.1	12	10	15	0.1	0.1	0.2	2	2	3
Brazil	Castanheira	0.4	0.3	0.5	0	0	0	0.1	0.1	0.1	0	0	0
Brazil	Castanheiras	0.4	0.3	0.5	0	0	0	0.1	0.1	0.1	0	0	0
Brazil	Castelândia	0.2	0.1	0.2	0	0	0	0.1	0.0	0.1	0	0	0
Brazil	Castelo	0.3	0.2	0.3	1	1	1	0.1	0.1	0.1	0	0	0
Brazil	Castelo do Piauí	1.4	1.1	1.8	3	3	4	0.2	0.1	0.2	0	0	0
Brazil	Castilho	0.2	0.1	0.2	0	0	0	0.0	0.0	0.0	0	0	0
Brazil	Castro	0.3	0.2	0.3	2	1	2	0.1	0.0	0.1	0	0	0
Brazil	Castro Alves	1.7	1.4	2.2	5	4	7	0.2	0.2	0.3	1	0	1
Brazil	Cataguases	0.3	0.2	0.4	1	1	2	0.1	0.0	0.1	0	0	0
Brazil	Catalão	0.2	0.1	0.2	1	1	1	0.1	0.0	0.1	0	0	0
Brazil	Catanduva	0.2	0.1	0.2	1	1	1	0.0	0.0	0.0	0	0	0
Brazil	Catanduvas	0.2	0.2	0.3	0	0	0	0.1	0.0	0.1	0	0	0
Brazil	Catanduvas	0.3	0.2	0.3	0	0	0	0.1	0.0	0.1	0	0	0
Brazil	Catarina	2.3	1.7	2.9	3	2	4	0.1	0.1	0.2	0	0	0
Brazil	Catas Altas	0.3	0.2	0.4	0	0	0	0.1	0.0	0.1	0	0	0
Brazil	Catas Altas da Noruega	0.3	0.2	0.4	0	0	0	0.1	0.0	0.1	0	0	0
Brazil	Catende	2.0	1.6	2.6	9	7	11	0.2	0.2	0.3	1	1	1
Brazil	Catigua	0.2	0.1	0.2	0	0	0	0.0	0.0	0.0	0	0	0
Brazil	Catingueira	1.0	0.7	1.2	1	0	1	0.1	0.1	0.1	0	0	0
Brazil	Catolândia	1.7	1.4	2.2	1	1	1	0.2	0.2	0.3	0	0	0
Brazil	Catolé do Rocha	1.0	0.7	1.2	3	2	4	0.1	0.1	0.1	0	0	0
Brazil	Catu	1.7	1.4	2.2	8	6	10	0.2	0.2	0.3	1	1	1
Brazil	Catuípe	0.1	0.1	0.2	0	0	0	0.0	0.0	0.0	0	0	0
Brazil	Catuji	0.3	0.2	0.4	0	0	0	0.1	0.0	0.1	0	0	0
Brazil	Catunda	2.3	1.7	2.9	3	2	3	0.1	0.1	0.2	0	0	0
Brazil	Caturaí	0.2	0.1	0.2	0	0	0	0.1	0.0	0.1	0	0	0
Brazil	Caturama	1.7	1.4	2.2	2	1	2	0.2	0.2	0.3	0	0	0
Brazil	Caturité	1.0	0.7	1.2	0	0	1	0.1	0.1	0.1	0	0	0
Brazil	Catuti	0.3	0.2	0.4	0	0	0	0.1	0.0	0.1	0	0	0
Brazil	Caucaia	2.3	1.7	2.9	72	55	92	0.1	0.1	0.2	5	4	7
Brazil	Cavalcante	0.2	0.1	0.2	0	0	0	0.1	0.0	0.1	0	0	0
Brazil	Caxambu	0.3	0.2	0.4	0	0	1	0.1	0.0	0.1	0	0	0
Brazil	Caxambu do Sul	0.2	0.2	0.3	0	0	0	0.1	0.0	0.1	0	0	0
Brazil	Caxias	1.7	1.3	2.2	33	26	43	0.2	0.2	0.3	4	3	6
Brazil	Caxias do Sul	0.1	0.1	0.2	3	2	4	0.0	0.0	0.0	1	1	1
Brazil	Caxingó	1.4	1.1	1.8	1	1	1	0.2	0.1	0.2	0	0	0
Brazil	Ceará-Mirim	1.2	0.9	1.5	11	8	14	0.1	0.1	0.2	1	1	2
Brazil	Cedral	0.2	0.1	0.2	0	0	0	0.0	0.0	0.0	0	0	0
Brazil	Cedral	1.7	1.3	2.2	1	1	1	0.2	0.2	0.3	0	0	0
Brazil	Cedro	2.3	1.7	2.9	6	5	8	0.1	0.1	0.2	0	0	0
Brazil	Cedro	2.0	1.6	2.6	3	2	3	0.2	0.2	0.3	0	0	0

Table 3: Diarrhoea Mortality by Unit *(continued)*

Country	Unit	2000 mortality mean rate (per 10,000)	lower rate (per 10,000)	upper rate (per 10,000)	mean count	lower count	upper count	2017 mortality mean rate (per 10,000)	lower rate (per 10,000)	upper rate (per 10,000)	mean count	lower count	upper count
Brazil	Cedro de São João	1.3	1.0	1.7	1	1	1	0.2	0.1	0.3	0	0	0
Brazil	Cedro do Abaeté	0.3	0.2	0.4	0	0	0	0.1	0.0	0.1	0	0	0
Brazil	Celso Ramos	0.2	0.2	0.3	0	0	0	0.1	0.0	0.1	0	0	0
Brazil	Centenário	0.8	0.7	1.1	0	0	0	0.1	0.1	0.2	0	0	0
Brazil	Centenário	0.1	0.1	0.2	0	0	0	0.0	0.0	0.0	0	0	0
Brazil	Centenário do Sul	0.3	0.2	0.3	0	0	0	0.1	0.0	0.1	0	0	0
Brazil	Central	1.7	1.4	2.2	4	3	5	0.2	0.2	0.3	0	0	1
Brazil	Central de Minas	0.3	0.2	0.4	0	0	0	0.1	0.0	0.1	0	0	0
Brazil	Central do Maranhão	1.7	1.3	2.2	2	1	2	0.2	0.2	0.3	0	0	0
Brazil	Centralina	0.3	0.2	0.4	0	0	0	0.1	0.0	0.1	0	0	0
Brazil	Centro do Guilherme	1.7	1.3	2.2	1	1	2	0.2	0.2	0.3	0	0	1
Brazil	Centro Novo do Maranhão	1.7	1.3	2.2	3	2	4	0.2	0.2	0.3	0	0	1
Brazil	Cerejeiras	0.4	0.3	0.5	1	1	1	0.1	0.1	0.1	0	0	0
Brazil	Ceres	0.2	0.1	0.2	0	0	0	0.1	0.0	0.1	0	0	0
Brazil	Cerqueira César	0.2	0.1	0.2	0	0	0	0.0	0.0	0.0	0	0	0
Brazil	Cerquilho	0.2	0.1	0.2	0	0	0	0.0	0.0	0.0	0	0	0
Brazil	Cerrito	0.1	0.1	0.2	0	0	0	0.0	0.0	0.0	0	0	0
Brazil	Cêrro Azul	0.3	0.2	0.3	0	0	1	0.1	0.0	0.1	0	0	0
Brazil	Cerro Branco	0.1	0.1	0.2	0	0	0	0.0	0.0	0.0	0	0	0
Brazil	Cerro Corá	1.2	0.9	1.5	1	1	2	0.1	0.1	0.2	0	0	0
Brazil	Cerro Grande	0.1	0.1	0.2	0	0	0	0.0	0.0	0.0	0	0	0
Brazil	Cerro Grande do Sul	0.1	0.1	0.2	0	0	0	0.0	0.0	0.0	0	0	0
Brazil	Cerro Largo	0.1	0.1	0.2	0	0	0	0.0	0.0	0.0	0	0	0
Brazil	Cerro Negro	0.2	0.2	0.3	0	0	0	0.1	0.0	0.1	0	0	0
Brazil	Cesário Lange	0.2	0.1	0.2	0	0	0	0.0	0.0	0.0	0	0	0
Brazil	Céu Azul	0.3	0.2	0.3	0	0	0	0.1	0.0	0.1	0	0	0
Brazil	Cezarina	0.2	0.1	0.2	0	0	0	0.1	0.0	0.1	0	0	0
Brazil	Chã de Alegria	2.0	1.6	2.6	2	1	2	0.2	0.2	0.3	0	0	0
Brazil	Chã Grande	2.0	1.6	2.6	3	2	4	0.2	0.2	0.3	0	0	0
Brazil	Chã Preta	2.8	2.2	3.7	3	3	4	0.3	0.2	0.4	0	0	0
Brazil	Chácara	0.3	0.2	0.4	0	0	0	0.1	0.0	0.1	0	0	0
Brazil	Chale	0.3	0.2	0.4	0	0	0	0.1	0.0	0.1	0	0	0
Brazil	Chapada	0.1	0.1	0.2	0	0	0	0.0	0.0	0.0	0	0	0
Brazil	Chapada da Natividade	0.8	0.7	1.1	0	0	0	0.1	0.1	0.2	0	0	0
Brazil	Chapada de Areia	0.8	0.7	1.1	0	0	0	0.1	0.1	0.2	0	0	0
Brazil	Chapada do Norte	0.3	0.2	0.4	0	0	1	0.1	0.0	0.1	0	0	0
Brazil	Chapada dos Guimarães	0.4	0.3	0.5	0	0	1	0.1	0.1	0.1	0	0	0
Brazil	Chapada Gaúcha	0.3	0.2	0.4	0	0	0	0.1	0.0	0.1	0	0	0
Brazil	Chapadão do Céu	1.7	1.3	2.2	14	11	18	0.2	0.2	0.3	2	2	3
Brazil	Chapadão do Lageado	0.2	0.2	0.3	0	0	0	0.1	0.0	0.1	0	0	0
Brazil	Chapadão do Sul	0.5	0.3	0.6	0	0	1	0.2	0.1	0.2	0	0	0
Brazil	Chapadinha	0.2	0.1	0.2	0	0	0	0.1	0.0	0.1	0	0	0
Brazil	Chapecó	0.2	0.2	0.3	3	3	4	0.1	0.0	0.1	1	1	1
Brazil	Charqueada	0.2	0.1	0.2	0	0	0	0.0	0.0	0.0	0	0	0
Brazil	Charqueadas	0.1	0.1	0.2	0	0	0	0.0	0.0	0.0	0	0	0
Brazil	Charrua	0.1	0.1	0.2	0	0	0	0.0	0.0	0.0	0	0	0
Brazil	Chaval	2.3	1.7	2.9	3	3	4	0.1	0.1	0.2	0	0	0
Brazil	Chavantes	0.2	0.1	0.2	0	0	0	0.0	0.0	0.0	0	0	0
Brazil	Chaves	0.9	0.7	1.1	2	1	2	0.1	0.1	0.2	0	0	0
Brazil	Chaveslandia	0.3	0.2	0.4	0	0	0	0.1	0.0	0.1	0	0	0
Brazil	Chiador	0.3	0.2	0.4	0	0	0	0.1	0.0	0.1	0	0	0

Table 3: Diarrhoea Mortality by Unit *(continued)*

Country	Unit	2000 mortality mean rate (per 10,000)	2000 mortality lower rate (per 10,000)	2000 mortality upper rate (per 10,000)	2000 mortality mean count	2000 mortality lower count	2000 mortality upper count	2017 mortality mean rate (per 10,000)	2017 mortality lower rate (per 10,000)	2017 mortality upper rate (per 10,000)	2017 mortality mean count	2017 mortality lower count	2017 mortality upper count
Brazil	Chiapeta	0.1	0.1	0.2	0	0	0	0.0	0.0	0.0	0	0	0
Brazil	Chopinzinho	0.3	0.2	0.3	1	0	1	0.1	0.0	0.1	0	0	0
Brazil	Choró	2.3	1.7	2.9	3	3	4	0.1	0.1	0.2	0	0	0
Brazil	Chorozinho	2.3	1.7	2.9	5	4	7	0.1	0.1	0.2	0	0	0
Brazil	Chorrochó	1.7	1.4	2.2	2	2	3	0.2	0.2	0.3	0	0	0
Brazil	Chuí	0.1	0.1	0.2	0	0	0	0.0	0.0	0.0	0	0	0
Brazil	Chupinguaia	0.4	0.3	0.5	0	0	0	0.1	0.1	0.1	0	0	0
Brazil	Chuvisca	0.1	0.1	0.2	0	0	0	0.0	0.0	0.0	0	0	0
Brazil	Cianorte	0.3	0.2	0.3	1	1	2	0.1	0.0	0.1	0	0	0
Brazil	Cícero Dantas	1.7	1.4	2.2	6	5	8	0.2	0.2	0.3	1	1	1
Brazil	Cidade Gaúcha	0.3	0.2	0.3	0	0	0	0.1	0.0	0.1	0	0	0
Brazil	Cidade Ocidental	0.2	0.1	0.2	1	1	1	0.1	0.0	0.1	0	0	0
Brazil	Cidelândia	1.7	1.3	2.2	3	2	4	0.2	0.2	0.3	0	0	1
Brazil	Cidreira	0.1	0.1	0.2	0	0	0	0.0	0.0	0.0	0	0	0
Brazil	Cipó	1.7	1.4	2.2	3	3	4	0.2	0.2	0.3	0	0	1
Brazil	Cipotânea	0.3	0.2	0.4	0	0	0	0.1	0.0	0.1	0	0	0
Brazil	Ciríaco	0.1	0.1	0.2	0	0	0	0.0	0.0	0.0	0	0	0
Brazil	Claraval	0.3	0.2	0.4	0	0	0	0.1	0.0	0.1	0	0	0
Brazil	Claro dos Poções	0.3	0.2	0.4	0	0	0	0.1	0.0	0.1	0	0	0
Brazil	Cláudia	0.4	0.3	0.5	0	0	0	0.1	0.1	0.1	0	0	0
Brazil	Cláudio	0.3	0.2	0.4	1	0	1	0.1	0.0	0.1	0	0	0
Brazil	Clementina	0.2	0.1	0.2	0	0	0	0.0	0.0	0.0	0	0	0
Brazil	Clevelândia	0.3	0.2	0.3	0	0	1	0.1	0.0	0.1	0	0	0
Brazil	Coaraci	1.7	1.4	2.2	4	3	6	0.2	0.2	0.3	0	0	0
Brazil	Coari	0.8	0.6	0.9	7	6	9	0.2	0.2	0.3	2	2	2
Brazil	Cocal	1.4	1.1	1.8	5	4	6	0.2	0.1	0.2	0	0	1
Brazil	Cocal de Telha	1.4	1.1	1.8	1	1	1	0.2	0.1	0.2	0	0	0
Brazil	Cocal do Sul	0.2	0.2	0.3	0	0	0	0.1	0.0	0.1	0	0	0
Brazil	Cocal dos Alves	1.4	1.1	1.8	1	1	1	0.2	0.1	0.2	0	0	0
Brazil	Cocalinho	0.4	0.3	0.5	0	0	0	0.1	0.1	0.1	0	0	0
Brazil	Cocalzinho de Goiás	0.2	0.1	0.2	0	0	0	0.1	0.0	0.1	0	0	0
Brazil	Cocos	1.7	1.4	2.2	4	3	5	0.2	0.2	0.3	0	0	1
Brazil	Codajás	0.8	0.6	0.9	2	1	2	0.2	0.2	0.3	1	0	1
Brazil	Codó	1.7	1.3	2.2	27	21	35	0.2	0.2	0.3	3	3	5
Brazil	Coelho Neto	1.7	1.3	2.2	10	8	13	0.2	0.2	0.3	1	1	2
Brazil	Coimbra	0.3	0.2	0.4	0	0	0	0.1	0.0	0.1	0	0	0
Brazil	Coité do Nóia	2.8	2.2	3.7	4	3	6	0.3	0.2	0.4	0	0	0
Brazil	Coivaras	1.4	1.1	1.8	1	0	1	0.2	0.1	0.2	0	0	0
Brazil	Colares	0.9	0.7	1.1	2	1	2	0.1	0.1	0.2	0	0	0
Brazil	Colatina	0.3	0.2	0.3	3	2	3	0.1	0.1	0.1	1	0	1
Brazil	Colíder	0.4	0.3	0.5	1	1	2	0.1	0.1	0.1	0	0	0
Brazil	Colina	0.2	0.1	0.2	0	0	0	0.0	0.0	0.0	0	0	0
Brazil	Colinas	0.1	0.1	0.2	0	0	0	0.0	0.0	0.0	0	0	0
Brazil	Colinas	1.7	1.3	2.2	9	7	11	0.2	0.2	0.3	1	1	2
Brazil	Colinas do Sul	0.2	0.1	0.2	0	0	0	0.1	0.0	0.1	0	0	0
Brazil	Colinas do Tocantins	0.8	0.7	1.1	2	2	3	0.1	0.1	0.2	0	0	0
Brazil	Colméia	0.8	0.7	1.1	1	1	1	0.1	0.1	0.2	0	0	0
Brazil	Colômbia	0.2	0.1	0.2	0	0	0	0.0	0.0	0.0	0	0	0
Brazil	Colombo	0.3	0.2	0.3	5	4	7	0.1	0.0	0.1	1	1	1
Brazil	Colônia do Gurguéia	1.4	1.1	1.8	1	1	1	0.2	0.1	0.2	0	0	0
Brazil	Colônia do Piauí	1.4	1.1	1.8	1	1	1	0.2	0.1	0.2	0	0	0
Brazil	Colônia Leopoldina	2.8	2.2	3.7	4	3	5	0.3	0.2	0.4	0	0	1
Brazil	Colorado	0.1	0.1	0.2	0	0	0	0.0	0.0	0.0	0	0	0
Brazil	Colorado	0.3	0.2	0.3	0	0	1	0.1	0.0	0.1	0	0	0
Brazil	Colorado do Oeste	0.4	0.3	0.5	1	1	1	0.1	0.1	0.1	0	0	0

Table 3: Diarrhoea Mortality by Unit *(continued)*

Country	Unit	2000 mortality mean rate (per 10,000)	2000 mortality lower rate (per 10,000)	2000 mortality upper rate (per 10,000)	2000 mortality mean count	2000 mortality lower count	2000 mortality upper count	2017 mortality mean rate (per 10,000)	2017 mortality lower rate (per 10,000)	2017 mortality upper rate (per 10,000)	2017 mortality mean count	2017 mortality lower count	2017 mortality upper count
Brazil	Coluna	0.3	0.2	0.4	0	0	0	0.1	0.0	0.1	0	0	0
Brazil	Combinado	0.8	0.7	1.1	0	0	1	0.1	0.1	0.2	0	0	0
Brazil	Comendador Gomes	0.3	0.2	0.4	0	0	0	0.1	0.0	0.1	0	0	0
Brazil	Comendador Levy Gasparian	0.2	0.1	0.3	0	0	0	0.1	0.0	0.1	0	0	0
Brazil	Comercinho	0.3	0.2	0.4	0	0	0	0.1	0.0	0.1	0	0	0
Brazil	Comodoro	0.4	0.3	0.5	1	1	1	0.1	0.1	0.1	0	0	0
Brazil	Conceição	1.0	0.7	1.2	2	2	3	0.1	0.1	0.1	0	0	0
Brazil	Conceição da Aparecida	0.3	0.2	0.4	0	0	0	0.1	0.0	0.1	0	0	0
Brazil	Conceição da Barra	0.3	0.2	0.3	1	1	1	0.1	0.1	0.1	0	0	0
Brazil	Conceição da Feira	1.7	1.4	2.2	3	3	4	0.2	0.2	0.3	1	0	1
Brazil	Conceição das Alagoas	0.3	0.2	0.4	0	0	1	0.1	0.0	0.1	0	0	0
Brazil	Conceição das Pedras	0.3	0.2	0.4	0	0	0	0.1	0.0	0.1	0	0	0
Brazil	Conceição de Ipanema	0.3	0.2	0.4	0	0	0	0.1	0.0	0.1	0	0	0
Brazil	Conceicao do Almeida	1.7	1.4	2.2	0	0	0	0.2	0.2	0.3	0	0	0
Brazil	Conceição do Almeida	1.7	1.4	2.2	2	2	3	0.2	0.2	0.3	0	0	0
Brazil	Conceição do Araguaia	0.9	0.7	1.1	2	2	3	0.1	0.1	0.2	0	0	1
Brazil	Conceição do Canindé	1.4	1.1	1.8	1	1	1	0.2	0.1	0.2	0	0	0
Brazil	Conceição do Castelo	0.3	0.2	0.3	0	0	0	0.1	0.1	0.1	0	0	0
Brazil	Conceição do Coité	1.7	1.4	2.2	12	9	15	0.2	0.2	0.3	2	1	2
Brazil	Conceição do Jacuípe	1.7	1.4	2.2	4	3	5	0.2	0.2	0.3	1	0	1
Brazil	Conceição do Lago-Açu	1.7	1.3	2.2	3	2	4	0.2	0.2	0.3	1	0	1
Brazil	Conceição do Mato Dentro	0.3	0.2	0.4	1	0	1	0.1	0.0	0.1	0	0	0
Brazil	Conceição do Para	0.3	0.2	0.4	0	0	0	0.1	0.0	0.1	0	0	0
Brazil	Conceição do Rio Verde	0.3	0.2	0.4	0	0	0	0.1	0.0	0.1	0	0	0
Brazil	Conceição do Tocantins	0.8	0.7	1.1	0	0	1	0.1	0.1	0.2	0	0	0
Brazil	Conceição dos Ouros	0.3	0.2	0.4	0	0	0	0.1	0.0	0.1	0	0	0
Brazil	Conceicao Macabu	0.2	0.1	0.3	0	0	0	0.1	0.0	0.1	0	0	0
Brazil	Conchal	0.2	0.1	0.2	0	0	0	0.0	0.0	0.0	0	0	0
Brazil	Conchas	0.2	0.1	0.2	0	0	0	0.0	0.0	0.0	0	0	0
Brazil	Concórdia	0.2	0.2	0.3	1	1	2	0.1	0.0	0.1	0	0	0
Brazil	Concórdia do Pará	0.9	0.7	1.1	4	3	5	0.1	0.1	0.2	1	0	1
Brazil	Condado	2.0	1.6	2.6	8	6	10	0.2	0.2	0.3	1	0	1
Brazil	Condado	1.0	0.7	1.2	1	0	1	0.1	0.1	0.1	0	0	0
Brazil	Conde	1.7	1.4	2.2	5	4	6	0.2	0.2	0.3	1	0	1
Brazil	Conde	1.0	0.7	1.2	3	2	4	0.1	0.1	0.1	0	0	1
Brazil	Condeúba	1.7	1.4	2.2	3	3	4	0.2	0.2	0.3	0	0	0
Brazil	Condor	0.1	0.1	0.2	0	0	0	0.0	0.0	0.0	0	0	0

Table 3: Diarrhoea Mortality by Unit *(continued)*

Country	Unit	2000 mortality mean rate (per 10,000)	lower rate (per 10,000)	upper rate (per 10,000)	mean count	lower count	upper count	2017 mortality mean rate (per 10,000)	lower rate (per 10,000)	upper rate (per 10,000)	mean count	lower count	upper count
Brazil	Cônego Marinho	0.3	0.2	0.4	0	0	0	0.1	0.0	0.1	0	0	0
Brazil	Confins	0.3	0.2	0.4	0	0	0	0.1	0.0	0.1	0	0	0
Brazil	Confresa	0.4	0.3	0.5	1	1	1	0.1	0.1	0.1	0	0	0
Brazil	Congo	1.0	0.7	1.2	0	0	0	0.1	0.1	0.1	0	0	0
Brazil	Congonhal	0.3	0.2	0.4	0	0	0	0.1	0.0	0.1	0	0	0
Brazil	Congonhas	0.3	0.2	0.4	1	1	1	0.1	0.0	0.1	0	0	0
Brazil	Congonhas do Norte	0.3	0.2	0.4	0	0	0	0.1	0.0	0.1	0	0	0
Brazil	Congonhinhas	0.3	0.2	0.3	0	0	0	0.1	0.0	0.1	0	0	0
Brazil	Conquista	0.3	0.2	0.4	0	0	0	0.1	0.0	0.1	0	0	0
Brazil	Conselheiro Lafaiete	0.3	0.2	0.4	2	2	3	0.1	0.0	0.1	0	0	1
Brazil	Conselheiro Mayrinck	0.3	0.2	0.3	0	0	0	0.1	0.0	0.1	0	0	0
Brazil	Conselheiro Pena	0.3	0.2	0.4	1	0	1	0.1	0.0	0.1	0	0	0
Brazil	Consolação	0.3	0.2	0.4	0	0	0	0.1	0.0	0.1	0	0	0
Brazil	Constantina	0.1	0.1	0.2	0	0	0	0.0	0.0	0.0	0	0	0
Brazil	Contagem	0.3	0.2	0.4	12	9	15	0.1	0.0	0.1	2	2	3
Brazil	Contenda	0.3	0.2	0.3	0	0	0	0.1	0.0	0.1	0	0	0
Brazil	Contendas do Sincorá	1.7	1.4	2.2	1	0	1	0.2	0.2	0.3	0	0	0
Brazil	Coqueiral	0.3	0.2	0.4	0	0	0	0.1	0.0	0.1	0	0	0
Brazil	Coqueiro Seco	2.8	2.2	3.7	7	6	10	0.3	0.2	0.4	1	1	1
Brazil	Coqueiros do Sul	0.1	0.1	0.2	0	0	0	0.0	0.0	0.0	0	0	0
Brazil	Coração de Jesus	0.3	0.2	0.4	1	1	1	0.1	0.0	0.1	0	0	0
Brazil	Coração de Maria	1.7	1.4	2.2	6	5	8	0.2	0.2	0.3	1	1	1
Brazil	Corbélia	0.3	0.2	0.3	0	0	0	0.1	0.0	0.1	0	0	0
Brazil	Cordeiro	0.2	0.1	0.3	0	0	0	0.1	0.0	0.1	0	0	0
Brazil	Cordeirópolis	0.2	0.1	0.2	0	0	0	0.0	0.0	0.0	0	0	0
Brazil	Cordeiros	1.7	1.4	2.2	2	1	2	0.2	0.2	0.3	0	0	0
Brazil	Cordilheira Alta	0.2	0.2	0.3	0	0	0	0.1	0.0	0.1	0	0	0
Brazil	Cordisburgo	0.3	0.2	0.4	0	0	0	0.1	0.0	0.1	0	0	0
Brazil	Cordislândia	0.3	0.2	0.4	0	0	0	0.1	0.0	0.1	0	0	0
Brazil	Coreaú	2.3	1.7	2.9	7	5	8	0.1	0.1	0.2	0	0	0
Brazil	Coremas	1.0	0.7	1.2	1	1	2	0.1	0.1	0.1	0	0	0
Brazil	Corguinho	0.5	0.3	0.6	0	0	0	0.2	0.1	0.2	0	0	0
Brazil	Coribe	1.7	1.4	2.2	3	2	4	0.2	0.2	0.3	0	0	0
Brazil	Corinto	0.3	0.2	0.4	0	0	1	0.1	0.0	0.1	0	0	0
Brazil	Cornélio Procópio	0.3	0.2	0.3	1	1	1	0.1	0.0	0.1	0	0	0
Brazil	Coroaci	0.3	0.2	0.4	0	0	0	0.1	0.0	0.1	0	0	0
Brazil	Coroados	0.2	0.1	0.2	0	0	0	0.0	0.0	0.0	0	0	0
Brazil	Coroatá	1.7	1.3	2.2	14	11	18	0.2	0.2	0.3	2	1	2
Brazil	Coromandel	0.3	0.2	0.4	1	0	1	0.1	0.0	0.1	0	0	0
Brazil	Coronel Barros	0.1	0.1	0.2	0	0	0	0.0	0.0	0.0	0	0	0
Brazil	Coronel Bicaco	0.1	0.1	0.2	0	0	0	0.0	0.0	0.0	0	0	0
Brazil	Coronel domingos Soares	0.3	0.2	0.3	0	0	0	0.1	0.0	0.1	0	0	0
Brazil	Coronel Ezequiel	1.2	0.9	1.5	1	1	1	0.1	0.1	0.2	0	0	0
Brazil	Coronel Fabriciano	0.3	0.2	0.4	2	1	2	0.1	0.0	0.1	0	0	0
Brazil	Coronel Freitas	0.2	0.2	0.3	0	0	0	0.1	0.0	0.1	0	0	0
Brazil	Coronel João Pessoa	1.2	0.9	1.5	1	1	1	0.1	0.1	0.2	0	0	0
Brazil	Coronel João Sá	1.7	1.4	2.2	5	4	6	0.2	0.2	0.3	0	0	1
Brazil	Coronel José Dias	1.4	1.1	1.8	1	1	1	0.2	0.1	0.2	0	0	0
Brazil	Coronel Macedo	0.2	0.1	0.2	0	0	0	0.0	0.0	0.0	0	0	0
Brazil	Coronel Martins	0.2	0.2	0.3	0	0	0	0.1	0.0	0.1	0	0	0
Brazil	Coronel Murta	0.3	0.2	0.4	0	0	0	0.1	0.0	0.1	0	0	0
Brazil	Coronel Pacheco	0.3	0.2	0.4	0	0	0	0.1	0.0	0.1	0	0	0
Brazil	Coronel Sapucaia	0.5	0.3	0.6	1	0	1	0.2	0.1	0.2	0	0	0

Table 3: Diarrhoea Mortality by Unit *(continued)*

Country	Unit	2000 mortality mean rate (per 10,000)	lower rate (per 10,000)	upper rate (per 10,000)	mean count	lower count	upper count	2017 mortality mean rate (per 10,000)	lower rate (per 10,000)	upper rate (per 10,000)	mean count	lower count	upper count
Brazil	Coronel Vivida	0.3	0.2	0.3	1	0	1	0.1	0.0	0.1	0	0	0
Brazil	Coronel Xavier Chaves	0.3	0.2	0.4	0	0	0	0.1	0.0	0.1	0	0	0
Brazil	Córrego Danta	0.3	0.2	0.4	0	0	0	0.1	0.0	0.1	0	0	0
Brazil	Córrego do Bom Jesus	0.3	0.2	0.4	0	0	0	0.1	0.0	0.1	0	0	0
Brazil	Córrego do Ouro	0.2	0.1	0.2	0	0	0	0.1	0.0	0.1	0	0	0
Brazil	Córrego Fundo	0.3	0.2	0.4	0	0	0	0.1	0.0	0.1	0	0	0
Brazil	Córrego Novo	0.3	0.2	0.4	0	0	0	0.1	0.0	0.1	0	0	0
Brazil	Correia Pinto	0.2	0.2	0.3	0	0	1	0.1	0.0	0.1	0	0	0
Brazil	Corrente	1.4	1.1	1.8	4	3	5	0.2	0.1	0.2	0	0	1
Brazil	Correntes	2.0	1.6	2.6	5	4	6	0.2	0.2	0.3	0	0	0
Brazil	Correntina	1.7	1.4	2.2	6	5	8	0.2	0.2	0.3	1	1	1
Brazil	Cortes	2.0	1.6	2.6	3	2	3	0.2	0.2	0.3	0	0	0
Brazil	Corumbá	0.5	0.3	0.6	0	0	0	0.2	0.1	0.2	0	0	0
Brazil	Corumbá de Goiás	0.2	0.1	0.2	0	0	0	0.1	0.0	0.1	0	0	0
Brazil	Corumbaíba	0.2	0.1	0.2	0	0	0	0.1	0.0	0.1	0	0	0
Brazil	Corumbataí	0.2	0.1	0.2	0	0	0	0.0	0.0	0.0	0	0	0
Brazil	Corumbataí do Sul	0.3	0.2	0.3	0	0	0	0.1	0.0	0.1	0	0	0
Brazil	Corumbiara	0.4	0.3	0.5	0	0	1	0.1	0.1	0.1	0	0	0
Brazil	Corupá	0.2	0.2	0.3	0	0	0	0.1	0.0	0.1	0	0	0
Brazil	Coruripe	2.8	2.2	3.7	19	14	24	0.3	0.2	0.4	2	2	3
Brazil	Cosmópolis	0.2	0.1	0.2	1	0	1	0.0	0.0	0.0	0	0	0
Brazil	Cosmorama	0.2	0.1	0.2	0	0	0	0.0	0.0	0.0	0	0	0
Brazil	Costa Marques	0.4	0.3	0.5	0	0	1	0.1	0.1	0.1	0	0	0
Brazil	Costa Rica	0.5	0.3	0.6	1	1	1	0.2	0.1	0.2	0	0	0
Brazil	Cotegipe	1.7	1.4	2.2	6	5	7	0.2	0.2	0.3	1	0	1
Brazil	Cotia	0.2	0.1	0.2	4	3	5	0.0	0.0	0.0	1	1	1
Brazil	Cotiporã	0.1	0.1	0.2	0	0	0	0.0	0.0	0.0	0	0	0
Brazil	Cotriguaçu	0.4	0.3	0.5	0	0	0	0.1	0.1	0.1	0	0	0
Brazil	Couto de Magalhães	0.3	0.2	0.4	0	0	0	0.1	0.0	0.1	0	0	0
Brazil	Couto Magalhaes	0.8	0.7	1.1	1	1	1	0.1	0.1	0.2	0	0	0
Brazil	Coxilha	0.1	0.1	0.2	0	0	0	0.0	0.0	0.0	0	0	0
Brazil	Coxim	0.5	0.3	0.6	1	1	1	0.2	0.1	0.2	0	0	0
Brazil	Coxixola	1.0	0.7	1.2	0	0	0	0.1	0.1	0.1	0	0	0
Brazil	Craíbas	2.8	2.2	3.7	7	6	9	0.3	0.2	0.4	1	1	1
Brazil	Crateús	2.3	1.7	2.9	18	14	24	0.1	0.1	0.2	1	1	1
Brazil	Crato	2.3	1.7	2.9	28	21	36	0.1	0.1	0.2	2	1	2
Brazil	Cravinhos	0.2	0.1	0.2	0	0	1	0.0	0.0	0.0	0	0	0
Brazil	Cravolândia	1.7	1.4	2.2	1	0	1	0.2	0.2	0.3	0	0	0
Brazil	Criciúma	0.2	0.2	0.3	4	3	5	0.1	0.0	0.1	1	1	1
Brazil	Crisólita	0.3	0.2	0.4	0	0	0	0.1	0.0	0.1	0	0	0
Brazil	Crisópolis	1.7	1.4	2.2	5	4	6	0.2	0.2	0.3	0	0	1
Brazil	Crissiumal	0.1	0.1	0.2	0	0	0	0.0	0.0	0.0	0	0	0
Brazil	Cristais	0.3	0.2	0.4	0	0	0	0.1	0.0	0.1	0	0	0
Brazil	Cristais Paulista	0.2	0.1	0.2	0	0	0	0.0	0.0	0.0	0	0	0
Brazil	Cristal	0.1	0.1	0.2	0	0	0	0.0	0.0	0.0	0	0	0
Brazil	Cristal do Sul	0.1	0.1	0.2	0	0	0	0.0	0.0	0.0	0	0	0
Brazil	Cristalândia	0.8	0.7	1.1	1	1	1	0.1	0.1	0.2	0	0	0
Brazil	Cristalândia do Piauí	1.4	1.1	1.8	1	1	1	0.2	0.1	0.2	0	0	0
Brazil	Cristália	0.3	0.2	0.4	0	0	0	0.1	0.0	0.1	0	0	0
Brazil	Cristalina	0.2	0.1	0.2	1	1	1	0.1	0.0	0.1	0	0	0
Brazil	Cristiano Otoni	0.3	0.2	0.4	0	0	0	0.1	0.0	0.1	0	0	0
Brazil	Cristianópolis	0.2	0.1	0.2	0	0	0	0.1	0.0	0.1	0	0	0
Brazil	Cristina	0.3	0.2	0.4	0	0	0	0.1	0.0	0.1	0	0	0
Brazil	Cristinápolis	1.3	1.0	1.7	3	2	3	0.2	0.1	0.3	0	0	1

Table 3: Diarrhoea Mortality by Unit *(continued)*

Country	Unit	2000 mortality mean rate (per 10,000)	lower rate (per 10,000)	upper rate (per 10,000)	mean count	lower count	upper count	2017 mortality mean rate (per 10,000)	lower rate (per 10,000)	upper rate (per 10,000)	mean count	lower count	upper count
Brazil	Cristino Castro	1.4	1.1	1.8	2	1	2	0.2	0.1	0.2	0	0	0
Brazil	Cristópolis	1.7	1.4	2.2	2	2	3	0.2	0.2	0.3	0	0	0
Brazil	Crixás	0.2	0.1	0.2	0	0	0	0.1	0.0	0.1	0	0	0
Brazil	Crixás do Tocantins	0.8	0.7	1.1	0	0	0	0.1	0.1	0.2	0	0	0
Brazil	Croatá	2.3	1.7	2.9	5	4	7	0.1	0.1	0.2	0	0	0
Brazil	Cromínia	0.2	0.1	0.2	0	0	0	0.1	0.0	0.1	0	0	0
Brazil	Crucilândia	0.3	0.2	0.4	0	0	0	0.1	0.0	0.1	0	0	0
Brazil	Cruz	2.3	1.7	2.9	5	4	7	0.1	0.1	0.2	0	0	1
Brazil	Cruz Alta	0.1	0.1	0.2	1	0	1	0.0	0.0	0.0	0	0	0
Brazil	Cruz das Almas	1.7	1.4	2.2	9	7	11	0.2	0.2	0.3	1	1	1
Brazil	Cruz do Espírito Santo	1.0	0.7	1.2	2	1	2	0.1	0.1	0.1	0	0	0
Brazil	Cruz Machado	0.3	0.2	0.3	1	0	1	0.1	0.0	0.1	0	0	0
Brazil	Cruzália	0.2	0.1	0.2	0	0	0	0.0	0.0	0.0	0	0	0
Brazil	Cruzeiro	0.2	0.1	0.2	1	1	1	0.0	0.0	0.0	0	0	0
Brazil	Cruzeiro da Fortaleza	0.3	0.2	0.4	0	0	0	0.1	0.0	0.1	0	0	0
Brazil	Cruzeiro do Iguaçu	0.3	0.2	0.3	0	0	0	0.1	0.0	0.1	0	0	0
Brazil	Cruzeiro do Oeste	0.3	0.2	0.3	0	0	1	0.1	0.0	0.1	0	0	0
Brazil	Cruzeiro do Sul	0.1	0.1	0.2	0	0	0	0.0	0.0	0.0	0	0	0
Brazil	Cruzeiro do Sul	1.1	0.9	1.3	8	7	10	0.2	0.2	0.3	2	1	2
Brazil	Cruzeiro do Sul	0.3	0.2	0.3	0	0	0	0.1	0.0	0.1	0	0	0
Brazil	Cruzeta	1.2	0.9	1.5	1	1	1	0.1	0.1	0.2	0	0	0
Brazil	Cruzília	0.3	0.2	0.4	0	0	0	0.1	0.0	0.1	0	0	0
Brazil	Cruzmaltina	0.3	0.2	0.3	0	0	0	0.1	0.0	0.1	0	0	0
Brazil	Cubatão	0.2	0.1	0.2	1	1	1	0.0	0.0	0.0	0	0	0
Brazil	Cubati	1.0	0.7	1.2	1	1	1	0.1	0.1	0.1	0	0	0
Brazil	Cuiaba	0.4	0.3	0.5	18	14	22	0.1	0.1	0.1	4	3	5
Brazil	Cuité	1.0	0.7	1.2	3	2	3	0.1	0.1	0.1	0	0	0
Brazil	Cuité de Mamanguape	1.0	0.7	1.2	1	1	1	0.1	0.1	0.1	0	0	0
Brazil	Cuitegi	1.0	0.7	1.2	1	1	1	0.1	0.1	0.1	0	0	0
Brazil	Cujubim	0.4	0.3	0.5	0	0	0	0.1	0.1	0.1	0	0	0
Brazil	Cumari	0.2	0.1	0.2	0	0	0	0.1	0.0	0.1	0	0	0
Brazil	Cumaru	2.0	1.6	2.6	6	4	7	0.2	0.2	0.3	0	0	0
Brazil	Cumaru do Norte	0.9	0.7	1.1	1	0	1	0.1	0.1	0.2	0	0	0
Brazil	Cumbe	1.3	1.0	1.7	1	0	1	0.2	0.1	0.3	0	0	0
Brazil	Cunha	0.2	0.1	0.2	0	0	0	0.0	0.0	0.0	0	0	0
Brazil	Cunha Porã	0.2	0.2	0.3	0	0	0	0.1	0.0	0.1	0	0	0
Brazil	Cunhataí	0.2	0.2	0.3	0	0	0	0.1	0.0	0.1	0	0	0
Brazil	Cuparaque	0.3	0.2	0.4	0	0	0	0.1	0.0	0.1	0	0	0
Brazil	Cupira	2.0	1.6	2.6	6	5	8	0.2	0.2	0.3	0	0	1
Brazil	Curaçá	1.7	1.4	2.2	7	6	9	0.2	0.2	0.3	1	1	1
Brazil	Curimatá	1.4	1.1	1.8	2	1	2	0.2	0.1	0.2	0	0	0
Brazil	Curionópolis	0.9	0.7	1.1	2	2	2	0.1	0.1	0.2	0	0	0
Brazil	Curitiba	0.3	0.2	0.3	32	24	41	0.1	0.0	0.1	6	5	7
Brazil	Curitibanos	0.2	0.2	0.3	1	1	1	0.1	0.0	0.1	0	0	0
Brazil	Curiúva	0.3	0.2	0.3	0	0	0	0.1	0.0	0.1	0	0	0
Brazil	Currais	1.4	1.1	1.8	1	1	1	0.2	0.1	0.2	0	0	0
Brazil	Currais Novos	1.2	0.9	1.5	6	4	7	0.1	0.1	0.2	1	0	1
Brazil	Curral de Cima	1.0	0.7	1.2	1	0	1	0.1	0.1	0.1	0	0	0
Brazil	Curral de dentro	0.3	0.2	0.4	0	0	0	0.1	0.0	0.1	0	0	0
Brazil	Curral Novo do Piauí	1.4	1.1	1.8	1	1	1	0.2	0.1	0.2	0	0	0
Brazil	Curral Velho	1.0	0.7	1.2	0	0	0	0.1	0.1	0.1	0	0	0
Brazil	Curralinho	0.9	0.7	1.1	3	2	3	0.1	0.1	0.2	1	1	1
Brazil	Curralinhos	1.4	1.1	1.8	1	1	1	0.2	0.1	0.2	0	0	0
Brazil	Curuá	0.9	0.7	1.1	1	1	1	0.1	0.1	0.2	0	0	0

Table 3: Diarrhoea Mortality by Unit *(continued)*

Country	Unit	2000 mortality mean rate (per 10,000)	lower rate (per 10,000)	upper rate (per 10,000)	mean count	lower count	upper count	2017 mortality mean rate (per 10,000)	lower rate (per 10,000)	upper rate (per 10,000)	mean count	lower count	upper count
Brazil	Curuçá	0.9	0.7	1.1	3	2	3	0.1	0.1	0.2	1	0	1
Brazil	Cururupu	1.7	1.3	2.2	7	6	10	0.2	0.2	0.3	1	1	1
Brazil	Curvelo	0.3	0.2	0.4	2	1	2	0.1	0.0	0.1	0	0	0
Brazil	Custódia	2.0	1.6	2.6	6	5	8	0.2	0.2	0.3	1	1	1
Brazil	Cutias	0.2	0.2	0.3	0	0	0	0.1	0.1	0.2	0	0	0
Brazil	Damianópolis	0.2	0.1	0.2	0	0	0	0.1	0.0	0.1	0	0	0
Brazil	Damião	1.0	0.7	1.2	0	0	0	0.1	0.1	0.1	0	0	0
Brazil	Darcinópolis	0.8	0.7	1.1	0	0	1	0.1	0.1	0.2	0	0	0
Brazil	Dário Meira	1.7	1.4	2.2	2	2	3	0.2	0.2	0.3	0	0	0
Brazil	Datas	0.3	0.2	0.4	0	0	0	0.1	0.0	0.1	0	0	0
Brazil	David Canabarro	0.1	0.1	0.2	0	0	0	0.0	0.0	0.0	0	0	0
Brazil	Davinópolis	1.7	1.3	2.2	5	4	6	0.2	0.2	0.3	1	0	1
Brazil	Davinópolis	0.2	0.1	0.2	0	0	0	0.1	0.0	0.1	0	0	0
Brazil	Delfim Moreira	0.3	0.2	0.4	0	0	0	0.1	0.0	0.1	0	0	0
Brazil	Delfinópolis	0.3	0.2	0.4	0	0	0	0.1	0.0	0.1	0	0	0
Brazil	Delmiro Gouveia	2.8	2.2	3.7	16	13	22	0.3	0.2	0.4	2	1	3
Brazil	Delta	0.3	0.2	0.4	0	0	0	0.1	0.0	0.1	0	0	0
Brazil	Demerval Lobão	1.4	1.1	1.8	2	2	2	0.2	0.1	0.2	0	0	0
Brazil	Denise	0.4	0.3	0.5	0	0	0	0.1	0.1	0.1	0	0	0
Brazil	Deodápolis	0.5	0.3	0.6	0	0	1	0.2	0.1	0.2	0	0	0
Brazil	Deputado Irapuan Pinheiro	2.3	1.7	2.9	2	2	3	0.1	0.1	0.2	0	0	0
Brazil	Derrubadas	0.1	0.1	0.2	0	0	0	0.0	0.0	0.0	0	0	0
Brazil	Descalvado	0.2	0.1	0.2	0	0	0	0.0	0.0	0.0	0	0	0
Brazil	Descanso	0.2	0.2	0.3	0	0	0	0.1	0.0	0.1	0	0	0
Brazil	Descoberto	0.3	0.2	0.4	0	0	0	0.1	0.0	0.1	0	0	0
Brazil	Desterro	1.0	0.7	1.2	1	1	1	0.1	0.1	0.1	0	0	0
Brazil	Desterro de Entre Rios	0.3	0.2	0.4	0	0	0	0.1	0.0	0.1	0	0	0
Brazil	Desterro de Malta	1.0	0.7	1.2	0	0	0	0.1	0.1	0.1	0	0	0
Brazil	Desterro do Melo	0.3	0.2	0.4	0	0	0	0.1	0.0	0.1	0	0	0
Brazil	Dezesseis de Novembro	0.1	0.1	0.2	0	0	0	0.0	0.0	0.0	0	0	0
Brazil	Diadema	0.2	0.1	0.2	5	4	6	0.0	0.0	0.0	1	1	1
Brazil	Diamante	1.0	0.7	1.2	1	1	1	0.1	0.1	0.1	0	0	0
Brazil	Diamante d'Oeste	0.3	0.2	0.3	0	0	0	0.1	0.0	0.1	0	0	0
Brazil	Diamante do Norte	0.3	0.2	0.3	0	0	0	0.1	0.0	0.1	0	0	0
Brazil	Diamante do Sul	0.3	0.2	0.3	0	0	0	0.1	0.0	0.1	0	0	0
Brazil	Diamantina	0.3	0.2	0.4	1	1	1	0.1	0.0	0.1	0	0	0
Brazil	Diamantino	0.4	0.3	0.5	1	1	1	0.1	0.1	0.1	0	0	0
Brazil	Dianopolis	0.8	0.7	1.1	2	1	2	0.1	0.1	0.2	0	0	0
Brazil	Dias d'vila	1.7	1.4	2.2	7	6	9	0.2	0.2	0.3	2	1	2
Brazil	Dilermano de Aguiar	0.1	0.1	0.2	0	0	0	0.0	0.0	0.0	0	0	0
Brazil	Diogo de Vasconcelos	0.3	0.2	0.4	0	0	0	0.1	0.0	0.1	0	0	0
Brazil	Dionísio	0.3	0.2	0.4	0	0	0	0.1	0.0	0.1	0	0	0
Brazil	Dionísio Cerqueira	0.2	0.2	0.3	0	0	0	0.1	0.0	0.1	0	0	0
Brazil	Diorama	0.2	0.1	0.2	0	0	0	0.1	0.0	0.1	0	0	0
Brazil	Dirce Reis	0.2	0.1	0.2	0	0	0	0.0	0.0	0.0	0	0	0
Brazil	Dirceu Arcoverde	1.4	1.1	1.8	1	1	1	0.2	0.1	0.2	0	0	0
Brazil	Divina Pastora	1.3	1.0	1.7	1	1	1	0.2	0.1	0.3	0	0	0
Brazil	Divinésia	0.3	0.2	0.4	0	0	0	0.1	0.0	0.1	0	0	0
Brazil	Divino	0.3	0.2	0.4	1	0	1	0.1	0.0	0.1	0	0	0
Brazil	Divino das Laranjeiras	0.3	0.2	0.4	0	0	0	0.1	0.0	0.1	0	0	0
Brazil	Divino de São Lourenço	0.3	0.2	0.3	0	0	0	0.1	0.1	0.1	0	0	0

Table 3: Diarrhoea Mortality by Unit *(continued)*

Country	Unit	2000 mortality mean rate (per 10,000)	2000 mortality lower rate (per 10,000)	2000 mortality upper rate (per 10,000)	2000 mortality mean count	2000 mortality lower count	2000 mortality upper count	2017 mortality mean rate (per 10,000)	2017 mortality lower rate (per 10,000)	2017 mortality upper rate (per 10,000)	2017 mortality mean count	2017 mortality lower count	2017 mortality upper count
Brazil	Divinolândia	0.2	0.1	0.2	0	0	0	0.0	0.0	0.0	0	0	0
Brazil	Divinolandia de Minas	0.3	0.2	0.4	0	0	0	0.1	0.0	0.1	0	0	0
Brazil	Divinópolis	0.3	0.2	0.4	4	3	5	0.1	0.0	0.1	1	1	1
Brazil	Divinópolis de Goiás	0.2	0.1	0.2	0	0	0	0.1	0.0	0.1	0	0	0
Brazil	Divinópolis do Tocantins	0.8	0.7	1.1	1	1	2	0.1	0.1	0.2	0	0	0
Brazil	Divisa Alegre	0.3	0.2	0.4	0	0	0	0.1	0.0	0.1	0	0	0
Brazil	Divisa Nova	0.3	0.2	0.4	0	0	0	0.1	0.0	0.1	0	0	0
Brazil	Divisópolis	0.3	0.2	0.4	0	0	0	0.1	0.0	0.1	0	0	0
Brazil	Dobrada	0.2	0.1	0.2	0	0	0	0.0	0.0	0.0	0	0	0
Brazil	Dois Córregos	0.2	0.1	0.2	0	0	0	0.0	0.0	0.0	0	0	0
Brazil	Dois Irmãos	0.1	0.1	0.2	0	0	0	0.0	0.0	0.0	0	0	0
Brazil	Dois Irmãos das Missões	0.1	0.1	0.2	0	0	0	0.0	0.0	0.0	0	0	0
Brazil	Dois Irmãos do Buriti	0.5	0.3	0.6	0	0	1	0.2	0.1	0.2	0	0	0
Brazil	Dois Lajeados	0.1	0.1	0.2	0	0	0	0.0	0.0	0.0	0	0	0
Brazil	Dois Riachos	2.8	2.2	3.7	5	4	6	0.3	0.2	0.4	0	0	1
Brazil	Dois Vizinhos	0.3	0.2	0.3	1	1	1	0.1	0.0	0.1	0	0	0
Brazil	Dom Aquino	0.4	0.3	0.5	0	0	0	0.1	0.1	0.1	0	0	0
Brazil	Dom Basílio	1.7	1.4	2.2	2	1	2	0.2	0.2	0.3	0	0	0
Brazil	Dom Bosco	0.3	0.2	0.4	0	0	0	0.1	0.0	0.1	0	0	0
Brazil	Dom Cavati	0.3	0.2	0.4	0	0	0	0.1	0.0	0.1	0	0	0
Brazil	Dom Eliseu	0.9	0.7	1.1	4	3	5	0.1	0.1	0.2	1	1	1
Brazil	Dom Expedito Lopes	1.4	1.1	1.8	1	1	1	0.2	0.1	0.2	0	0	0
Brazil	Dom Feliciano	0.1	0.1	0.2	0	0	0	0.0	0.0	0.0	0	0	0
Brazil	Dom Inocêncio	1.4	1.1	1.8	1	1	2	0.2	0.1	0.2	0	0	0
Brazil	Dom Joaquim	0.3	0.2	0.4	0	0	0	0.1	0.0	0.1	0	0	0
Brazil	Dom Macedo Costa	1.7	1.4	2.2	1	1	1	0.2	0.2	0.3	0	0	0
Brazil	Dom Pedrito	0.1	0.1	0.2	0	0	1	0.0	0.0	0.0	0	0	0
Brazil	Dom Pedro	1.7	1.3	2.2	5	4	7	0.2	0.2	0.3	1	0	1
Brazil	Dom Pedro de Alcântara	0.1	0.1	0.2	0	0	0	0.0	0.0	0.0	0	0	0
Brazil	Dom Silvério	0.3	0.2	0.4	0	0	0	0.1	0.0	0.1	0	0	0
Brazil	Dom Viçoso	0.3	0.2	0.4	0	0	0	0.1	0.0	0.1	0	0	0
Brazil	Domingos Martins	0.3	0.2	0.3	1	1	1	0.1	0.1	0.1	0	0	0
Brazil	Domingos Mourão	1.4	1.1	1.8	1	1	1	0.2	0.1	0.2	0	0	0
Brazil	Dona Emma	0.2	0.2	0.3	0	0	0	0.1	0.0	0.1	0	0	0
Brazil	Dona Eusébia	0.3	0.2	0.4	0	0	0	0.1	0.0	0.1	0	0	0
Brazil	Dona Francisca	0.1	0.1	0.2	0	0	0	0.0	0.0	0.0	0	0	0
Brazil	Dona Inês	1.0	0.7	1.2	1	1	1	0.1	0.1	0.1	0	0	0
Brazil	Dores de Campos	0.3	0.2	0.4	0	0	0	0.1	0.0	0.1	0	0	0
Brazil	Dores de Guanhães	0.3	0.2	0.4	0	0	0	0.1	0.0	0.1	0	0	0
Brazil	Dores do Indaiá	0.3	0.2	0.4	0	0	0	0.1	0.0	0.1	0	0	0
Brazil	Dores do Rio Preto	0.3	0.2	0.3	0	0	0	0.1	0.1	0.1	0	0	0
Brazil	Dores do Turvo	0.3	0.2	0.4	0	0	0	0.1	0.0	0.1	0	0	0
Brazil	Doresópolis	0.3	0.2	0.4	0	0	0	0.1	0.0	0.1	0	0	0
Brazil	Dormentes	2.0	1.6	2.6	4	3	5	0.2	0.2	0.3	0	0	1
Brazil	Douradina	0.3	0.2	0.3	0	0	0	0.1	0.0	0.1	0	0	0
Brazil	Douradina	0.5	0.3	0.6	0	0	0	0.2	0.1	0.2	0	0	0
Brazil	Dourado	0.2	0.1	0.2	0	0	0	0.0	0.0	0.0	0	0	0
Brazil	Douradoquara	0.3	0.2	0.4	0	0	0	0.1	0.0	0.1	0	0	0

Table 3: Diarrhoea Mortality by Unit *(continued)*

Country	Unit	2000 mortality mean rate (per 10,000)	lower rate (per 10,000)	upper rate (per 10,000)	mean count	lower count	upper count	2017 mortality mean rate (per 10,000)	lower rate (per 10,000)	upper rate (per 10,000)	mean count	lower count	upper count
Brazil	Dourados	0.5	0.3	0.6	8	6	10	0.2	0.1	0.2	2	2	3
Brazil	Doutor Camargo	0.3	0.2	0.3	0	0	0	0.1	0.0	0.1	0	0	0
Brazil	Doutor Maurício Cardoso	0.1	0.1	0.2	0	0	0	0.0	0.0	0.0	0	0	0
Brazil	Doutor Pedrinho	0.2	0.2	0.3	0	0	0	0.1	0.0	0.1	0	0	0
Brazil	Doutor Ricardo	0.1	0.1	0.2	0	0	0	0.0	0.0	0.0	0	0	0
Brazil	Doutor Severiano	1.2	0.9	1.5	1	1	1	0.1	0.1	0.2	0	0	0
Brazil	Doutor Ulysses	0.3	0.2	0.3	0	0	0	0.1	0.0	0.1	0	0	0
Brazil	Doverlândia	0.2	0.1	0.2	0	0	0	0.1	0.0	0.1	0	0	0
Brazil	Dracena	0.2	0.1	0.2	0	0	1	0.0	0.0	0.0	0	0	0
Brazil	Duartina	0.2	0.1	0.2	0	0	0	0.0	0.0	0.0	0	0	0
Brazil	Duas Barras	0.2	0.1	0.3	0	0	0	0.1	0.0	0.1	0	0	0
Brazil	Duas Estradas	1.0	0.7	1.2	0	0	0	0.1	0.1	0.1	0	0	0
Brazil	Dueré	0.8	0.7	1.1	0	0	0	0.1	0.1	0.2	0	0	0
Brazil	Dulcinopolis	0.2	0.1	0.2	0	0	0	0.0	0.0	0.0	0	0	0
Brazil	Dumont	0.2	0.1	0.2	0	0	0	0.0	0.0	0.0	0	0	0
Brazil	Duque Bacelar	1.7	1.3	2.2	2	2	3	0.2	0.2	0.3	0	0	0
Brazil	Duque de Caxias	0.2	0.1	0.3	14	10	18	0.1	0.0	0.1	3	3	4
Brazil	Durandé	0.3	0.2	0.4	0	0	0	0.1	0.0	0.1	0	0	0
Brazil	Echaporã	0.2	0.1	0.2	0	0	0	0.0	0.0	0.0	0	0	0
Brazil	Ecoporanga	0.3	0.2	0.4	1	0	1	0.1	0.0	0.1	0	0	0
Brazil	Edealina	0.2	0.1	0.2	0	0	0	0.1	0.0	0.1	0	0	0
Brazil	Edéia	0.2	0.1	0.2	0	0	0	0.1	0.0	0.1	0	0	0
Brazil	Eirunepé	0.8	0.6	0.9	3	2	3	0.2	0.2	0.3	1	1	1
Brazil	Eldorado	0.2	0.1	0.2	0	0	0	0.0	0.0	0.0	0	0	0
Brazil	Eldorado	0.5	0.3	0.6	1	0	1	0.2	0.1	0.2	0	0	0
Brazil	Eldorado do Sul	0.1	0.1	0.2	0	0	0	0.0	0.0	0.0	0	0	0
Brazil	Eldorado dos Carajás	0.9	0.7	1.1	3	2	4	0.1	0.1	0.2	0	0	1
Brazil	Elesbão Veloso	1.4	1.1	1.8	2	2	3	0.2	0.1	0.2	0	0	0
Brazil	Elias Fausto	0.2	0.1	0.2	0	0	0	0.0	0.0	0.0	0	0	0
Brazil	Eliseu Martins	1.4	1.1	1.8	1	1	1	0.2	0.1	0.2	0	0	0
Brazil	Elisiário	0.2	0.1	0.2	0	0	0	0.0	0.0	0.0	0	0	0
Brazil	Elísio Medrado	1.7	1.4	2.2	2	1	2	0.2	0.2	0.3	0	0	0
Brazil	Elói Mendes	0.3	0.2	0.4	1	0	1	0.1	0.0	0.1	0	0	0
Brazil	Emas	1.0	0.7	1.2	0	0	0	0.1	0.1	0.1	0	0	0
Brazil	Embaúba	0.2	0.1	0.2	0	0	0	0.0	0.0	0.0	0	0	0
Brazil	Embu	0.2	0.1	0.2	3	2	4	0.0	0.0	0.0	1	0	1
Brazil	Embu-Guaçu	0.2	0.1	0.2	1	1	1	0.0	0.0	0.0	0	0	0
Brazil	Emilianópolis	0.2	0.1	0.2	0	0	0	0.0	0.0	0.0	0	0	0
Brazil	Encantado	0.1	0.1	0.2	0	0	0	0.0	0.0	0.0	0	0	0
Brazil	Encanto	1.2	0.9	1.5	1	1	1	0.1	0.1	0.2	0	0	0
Brazil	Encruzilhada	1.7	1.4	2.2	9	7	12	0.2	0.2	0.3	1	1	1
Brazil	Encruzilhada do Sul	0.1	0.1	0.2	0	0	0	0.0	0.0	0.0	0	0	0
Brazil	Enéas Marques	0.3	0.2	0.3	0	0	0	0.1	0.0	0.1	0	0	0
Brazil	Engenheiro Beltrão	0.3	0.2	0.3	0	0	0	0.1	0.0	0.1	0	0	0
Brazil	Engenheiro Caldas	0.3	0.2	0.4	0	0	0	0.1	0.0	0.1	0	0	0
Brazil	Engenheiro Coelho	0.2	0.1	0.2	0	0	0	0.0	0.0	0.0	0	0	0
Brazil	Engenheiro Navarro	0.3	0.2	0.4	0	0	0	0.1	0.0	0.1	0	0	0
Brazil	Engenheiro Paulo de Front	0.2	0.1	0.3	0	0	0	0.1	0.0	0.1	0	0	0
Brazil	Engenho Velho	0.1	0.1	0.2	0	0	0	0.0	0.0	0.0	0	0	0
Brazil	Entre Folhas	0.3	0.2	0.4	0	0	0	0.1	0.0	0.1	0	0	0
Brazil	Entre Rios	1.7	1.4	2.2	9	7	12	0.2	0.2	0.3	1	1	1
Brazil	Entre Rios	0.2	0.2	0.3	0	0	0	0.1	0.0	0.1	0	0	0

Table 3: Diarrhoea Mortality by Unit *(continued)*

Country	Unit	2000 mortality mean rate (per 10,000)	lower rate (per 10,000)	upper rate (per 10,000)	mean count	lower count	upper count	2017 mortality mean rate (per 10,000)	lower rate (per 10,000)	upper rate (per 10,000)	mean count	lower count	upper count
Brazil	Entre Rios de Minas	0.3	0.2	0.4	0	0	0	0.1	0.0	0.1	0	0	0
Brazil	Entre Rios do Oeste	0.3	0.2	0.3	0	0	0	0.1	0.0	0.1	0	0	0
Brazil	Entre Rios do Sul	0.1	0.1	0.2	0	0	0	0.0	0.0	0.0	0	0	0
Brazil	Entre-Ijuís	0.1	0.1	0.2	0	0	0	0.0	0.0	0.0	0	0	0
Brazil	Envira	0.8	0.6	0.9	3	2	3	0.2	0.2	0.3	1	1	1
Brazil	Epitaciolândia	1.1	0.9	1.3	2	1	2	0.2	0.2	0.3	0	0	1
Brazil	Equador	1.2	0.9	1.5	1	1	1	0.1	0.1	0.2	0	0	0
Brazil	Erebango	0.1	0.1	0.2	0	0	0	0.0	0.0	0.0	0	0	0
Brazil	Erechim	0.1	0.1	0.2	1	1	1	0.0	0.0	0.0	0	0	0
Brazil	Ererê	2.3	1.7	2.9	2	1	2	0.1	0.1	0.2	0	0	0
Brazil	Érico Cardoso	1.7	1.4	2.2	2	2	3	0.2	0.2	0.3	0	0	0
Brazil	Ermo	0.2	0.2	0.3	0	0	0	0.1	0.0	0.1	0	0	0
Brazil	Ernestina	0.1	0.1	0.2	0	0	0	0.0	0.0	0.0	0	0	0
Brazil	Erval	0.1	0.1	0.2	0	0	0	0.0	0.0	0.0	0	0	0
Brazil	Erval Grande	0.1	0.1	0.2	0	0	0	0.0	0.0	0.0	0	0	0
Brazil	Erval Seco	0.1	0.1	0.2	0	0	0	0.0	0.0	0.0	0	0	0
Brazil	Erval Velho	0.2	0.2	0.3	0	0	0	0.1	0.0	0.1	0	0	0
Brazil	Ervália	0.3	0.2	0.4	0	0	0	0.1	0.0	0.1	0	0	0
Brazil	Escada	2.0	1.6	2.6	14	11	18	0.2	0.2	0.3	2	1	2
Brazil	Esmeralda	0.1	0.1	0.2	0	0	0	0.0	0.0	0.0	0	0	0
Brazil	Esmeraldas	0.3	0.2	0.4	2	1	2	0.1	0.0	0.1	0	0	0
Brazil	Espera Feliz	0.3	0.2	0.4	1	0	1	0.1	0.0	0.1	0	0	0
Brazil	Esperança	1.0	0.7	1.2	3	2	4	0.1	0.1	0.1	0	0	0
Brazil	Esperança do Sul	0.1	0.1	0.2	0	0	0	0.0	0.0	0.0	0	0	0
Brazil	Esperança Nova	0.3	0.2	0.3	0	0	0	0.1	0.0	0.1	0	0	0
Brazil	Esperantina	0.8	0.7	1.1	1	1	1	0.1	0.1	0.2	0	0	0
Brazil	Esperantina	1.4	1.1	1.8	5	4	7	0.2	0.1	0.2	1	0	1
Brazil	Esperantinópolis	1.7	1.3	2.2	5	4	6	0.2	0.2	0.3	0	0	1
Brazil	Espigão Alto do Iguaçu	0.3	0.2	0.3	0	0	0	0.1	0.0	0.1	0	0	0
Brazil	Espigão d'Oeste	0.4	0.3	0.5	1	1	1	0.1	0.1	0.1	0	0	0
Brazil	Espinosa	0.3	0.2	0.4	1	1	1	0.1	0.0	0.1	0	0	0
Brazil	Espírito Santo	1.2	0.9	1.5	2	2	3	0.1	0.1	0.2	0	0	0
Brazil	Espírito Santo do Dourado	0.3	0.2	0.4	0	0	0	0.1	0.0	0.1	0	0	0
Brazil	Espírito Santo do Pinhal	0.2	0.1	0.2	0	0	1	0.0	0.0	0.0	0	0	0
Brazil	Espírito Santo do Turvo	0.2	0.1	0.2	0	0	0	0.0	0.0	0.0	0	0	0
Brazil	Esplanada	1.7	1.4	2.2	7	6	9	0.2	0.2	0.3	1	1	1
Brazil	Espumoso	0.1	0.1	0.2	0	0	0	0.0	0.0	0.0	0	0	0
Brazil	Estação	0.1	0.1	0.2	0	0	0	0.0	0.0	0.0	0	0	0
Brazil	Estância	1.3	1.0	1.7	9	7	11	0.2	0.1	0.3	1	1	1
Brazil	Estância Velha	0.1	0.1	0.2	0	0	0	0.0	0.0	0.0	0	0	0
Brazil	Esteio	0.1	0.1	0.2	1	0	1	0.0	0.0	0.0	0	0	0
Brazil	Estiva	0.3	0.2	0.4	0	0	0	0.1	0.0	0.1	0	0	0
Brazil	Estiva Gerbi	0.2	0.1	0.2	0	0	0	0.0	0.0	0.0	0	0	0
Brazil	Estreito	1.7	1.3	2.2	6	4	7	0.2	0.2	0.3	1	1	2
Brazil	Estrela	0.1	0.1	0.2	0	0	0	0.0	0.0	0.0	0	0	0
Brazil	Estrela dalva	0.3	0.2	0.4	0	0	0	0.1	0.0	0.1	0	0	0
Brazil	Estrela de Alagoas	2.8	2.2	3.7	5	4	7	0.3	0.2	0.4	1	0	1
Brazil	Estrela do Indaiá	0.3	0.2	0.4	0	0	0	0.1	0.0	0.1	0	0	0
Brazil	Estrela do Norte	0.2	0.1	0.2	0	0	0	0.0	0.0	0.0	0	0	0
Brazil	Estrela do Norte	0.2	0.1	0.2	0	0	0	0.1	0.0	0.1	0	0	0
Brazil	Estrela do Oeste	0.2	0.1	0.2	0	0	0	0.0	0.0	0.0	0	0	0
Brazil	Estrela do Sul	0.3	0.2	0.4	0	0	0	0.1	0.0	0.1	0	0	0
Brazil	Estrela Velha	0.1	0.1	0.2	0	0	0	0.0	0.0	0.0	0	0	0

Table 3: Diarrhoea Mortality by Unit *(continued)*

Country	Unit	2000 mortality mean rate (per 10,000)	lower rate (per 10,000)	upper rate (per 10,000)	mean count	lower count	upper count	2017 mortality mean rate (per 10,000)	lower rate (per 10,000)	upper rate (per 10,000)	mean count	lower count	upper count
Brazil	Euclides da Cunha	1.7	1.4	2.2	12	9	15	0.2	0.2	0.3	1	1	2
Brazil	Euclides da Cunha Paulista	0.2	0.1	0.2	0	0	0	0.0	0.0	0.0	0	0	0
Brazil	Eugênio de Castro	0.1	0.1	0.2	0	0	0	0.0	0.0	0.0	0	0	0
Brazil	Eugenópolis	0.3	0.2	0.4	0	0	0	0.1	0.0	0.1	0	0	0
Brazil	Eunápolis	1.7	1.4	2.2	18	14	24	0.2	0.2	0.3	3	2	4
Brazil	Eusébio	2.3	1.7	2.9	14	11	19	0.1	0.1	0.2	1	1	2
Brazil	Ewbank da Câmara	0.3	0.2	0.4	0	0	0	0.1	0.0	0.1	0	0	0
Brazil	Extrema	0.3	0.2	0.4	0	0	1	0.1	0.0	0.1	0	0	0
Brazil	Extremoz	1.2	0.9	1.5	7	5	9	0.1	0.1	0.2	1	1	1
Brazil	Exu	2.0	1.6	2.6	9	7	12	0.2	0.2	0.3	1	1	1
Brazil	Fagundes	1.0	0.7	1.2	1	1	1	0.1	0.1	0.1	0	0	0
Brazil	Fagundes Varela	0.1	0.1	0.2	0	0	0	0.0	0.0	0.0	0	0	0
Brazil	Faina	0.2	0.1	0.2	0	0	0	0.1	0.0	0.1	0	0	0
Brazil	Fama	0.3	0.2	0.4	0	0	0	0.1	0.0	0.1	0	0	0
Brazil	Faria Lemos	0.3	0.2	0.4	0	0	0	0.1	0.0	0.1	0	0	0
Brazil	Farias Brito	2.3	1.7	2.9	5	4	7	0.1	0.1	0.2	0	0	0
Brazil	Faro	0.9	0.7	1.1	1	1	1	0.1	0.1	0.2	0	0	0
Brazil	Farol	0.3	0.2	0.3	0	0	0	0.1	0.0	0.1	0	0	0
Brazil	Farroupilha	0.1	0.1	0.2	1	0	1	0.0	0.0	0.0	0	0	0
Brazil	Fartura	0.2	0.1	0.2	0	0	0	0.0	0.0	0.0	0	0	0
Brazil	Fartura do Piauí	1.4	1.1	1.8	1	1	1	0.2	0.1	0.2	0	0	0
Brazil	Fátima	1.7	1.4	2.2	4	3	5	0.2	0.2	0.3	0	0	1
Brazil	Fátima	0.8	0.7	1.1	0	0	1	0.1	0.1	0.2	0	0	0
Brazil	Fatima do Sul	0.5	0.3	0.6	1	0	1	0.2	0.1	0.2	0	0	0
Brazil	Faxinal	0.3	0.2	0.3	0	0	0	0.1	0.0	0.1	0	0	0
Brazil	Faxinal do Soturno	0.1	0.1	0.2	0	0	0	0.0	0.0	0.0	0	0	0
Brazil	Faxinal dos Guedes	0.2	0.2	0.3	0	0	0	0.1	0.0	0.1	0	0	0
Brazil	Faxinalzinho	0.1	0.1	0.2	0	0	0	0.0	0.0	0.0	0	0	0
Brazil	Fazenda Nova	0.2	0.1	0.2	0	0	0	0.1	0.0	0.1	0	0	0
Brazil	Fazenda Rio Grande	0.3	0.2	0.3	2	2	3	0.1	0.0	0.1	0	0	1
Brazil	Fazenda Vilanova	0.1	0.1	0.2	0	0	0	0.0	0.0	0.0	0	0	0
Brazil	Feijó	1.1	0.9	1.3	3	2	4	0.2	0.2	0.3	1	1	1
Brazil	Feira da Mata	1.7	1.4	2.2	2	2	3	0.2	0.2	0.3	0	0	0
Brazil	Feira de Santana	1.7	1.4	2.2	95	75	122	0.2	0.2	0.3	14	10	18
Brazil	Feira Grande	2.8	2.2	3.7	7	5	9	0.3	0.2	0.4	1	0	1
Brazil	Feira Nova	1.3	1.0	1.7	1	1	2	0.2	0.1	0.3	0	0	0
Brazil	Feira Nova	2.0	1.6	2.6	5	4	6	0.2	0.2	0.3	0	0	1
Brazil	Feira Nova do Maranhão	1.7	1.3	2.2	2	2	3	0.2	0.2	0.3	0	0	0
Brazil	Felício dos Santos	0.3	0.2	0.4	0	0	0	0.1	0.0	0.1	0	0	0
Brazil	Felipe Guerra	1.2	0.9	1.5	1	0	1	0.1	0.1	0.2	0	0	0
Brazil	Felisberto Caldeira	0.3	0.2	0.4	0	0	0	0.1	0.0	0.1	0	0	0
Brazil	Felisburgo	0.3	0.2	0.4	0	0	0	0.1	0.0	0.1	0	0	0
Brazil	Felixlândia	0.3	0.2	0.4	0	0	0	0.1	0.0	0.1	0	0	0
Brazil	Feliz	0.1	0.1	0.2	0	0	0	0.0	0.0	0.0	0	0	0
Brazil	Feliz Deserto	2.8	2.2	3.7	1	1	2	0.3	0.2	0.4	0	0	0
Brazil	Feliz Natal	0.4	0.3	0.5	0	0	0	0.1	0.1	0.1	0	0	0
Brazil	Fênix	0.3	0.2	0.3	0	0	0	0.1	0.0	0.1	0	0	0
Brazil	Fernandes Pinheiro	0.3	0.2	0.3	0	0	0	0.1	0.0	0.1	0	0	0
Brazil	Fernandes Tourinho	0.3	0.2	0.4	0	0	0	0.1	0.0	0.1	0	0	0

Table 3: Diarrhoea Mortality by Unit *(continued)*

Country	Unit	2000 mortality mean rate (per 10,000)	lower rate (per 10,000)	upper rate (per 10,000)	mean count	lower count	upper count	2017 mortality mean rate (per 10,000)	lower rate (per 10,000)	upper rate (per 10,000)	mean count	lower count	upper count
Brazil	Fernando de Noronha	1.2	0.9	1.5	0	0	0	0.1	0.1	0.2	0	0	0
Brazil	Fernando Falcão	1.7	1.3	2.2	1	1	1	0.2	0.2	0.3	0	0	0
Brazil	Fernando Pedroza	1.2	0.9	1.5	0	0	0	0.1	0.1	0.2	0	0	0
Brazil	Fernando Prestes	0.2	0.1	0.2	0	0	0	0.0	0.0	0.0	0	0	0
Brazil	Fernandópolis	0.2	0.1	0.2	1	0	1	0.0	0.0	0.0	0	0	0
Brazil	Ferno	0.2	0.1	0.2	0	0	0	0.0	0.0	0.0	0	0	0
Brazil	Ferraz de Vascon	0.2	0.1	0.2	3	3	4	0.0	0.0	0.0	1	1	1
Brazil	Ferreira Gomes	0.2	0.2	0.3	0	0	0	0.1	0.1	0.2	0	0	0
Brazil	Ferreiros	2.0	1.6	2.6	2	1	2	0.2	0.2	0.3	0	0	0
Brazil	Ferros	0.3	0.2	0.4	0	0	0	0.1	0.0	0.1	0	0	0
Brazil	Fervedouro	0.3	0.2	0.4	0	0	0	0.1	0.0	0.1	0	0	0
Brazil	Figueira	0.3	0.2	0.3	0	0	0	0.1	0.0	0.1	0	0	0
Brazil	Figueirópolis	0.8	0.7	1.1	1	1	1	0.1	0.1	0.2	0	0	0
Brazil	Figueirópolis d'Oeste	0.4	0.3	0.5	0	0	0	0.1	0.1	0.1	0	0	0
Brazil	Filadélfia	1.7	1.4	2.2	5	4	6	0.2	0.2	0.3	1	0	1
Brazil	Filadélfia	0.8	0.7	1.1	2	1	2	0.1	0.1	0.2	0	0	0
Brazil	Firmino Alves	1.7	1.4	2.2	1	1	1	0.2	0.2	0.3	0	0	0
Brazil	Firminópolis	0.2	0.1	0.2	0	0	0	0.1	0.0	0.1	0	0	0
Brazil	Flexeiras	2.8	2.2	3.7	8	6	11	0.3	0.2	0.4	1	1	1
Brazil	Flor da Serra do Sul	0.3	0.2	0.3	0	0	0	0.1	0.0	0.1	0	0	0
Brazil	Flor do Sertão	0.2	0.2	0.3	0	0	0	0.1	0.0	0.1	0	0	0
Brazil	Flora Rica	0.2	0.1	0.2	0	0	0	0.0	0.0	0.0	0	0	0
Brazil	Floraí	0.3	0.2	0.3	0	0	0	0.1	0.0	0.1	0	0	0
Brazil	Florânia	1.2	0.9	1.5	1	1	2	0.1	0.1	0.2	0	0	0
Brazil	Floreal	0.2	0.1	0.2	0	0	0	0.0	0.0	0.0	0	0	0
Brazil	Flores	2.0	1.6	2.6	5	4	7	0.2	0.2	0.3	0	0	1
Brazil	Flores da Cunha	0.1	0.1	0.2	0	0	0	0.0	0.0	0.0	0	0	0
Brazil	Flores de Goiás	0.2	0.1	0.2	0	0	0	0.1	0.0	0.1	0	0	0
Brazil	Flores do Piauí	1.4	1.1	1.8	1	1	1	0.2	0.1	0.2	0	0	0
Brazil	Floresta	0.3	0.2	0.3	0	0	0	0.1	0.0	0.1	0	0	0
Brazil	Floresta	2.0	1.6	2.6	7	6	9	0.2	0.2	0.3	1	1	1
Brazil	Floresta Azul	1.7	1.4	2.2	2	1	2	0.2	0.2	0.3	0	0	0
Brazil	Floresta do Araguaia	0.9	0.7	1.1	1	1	2	0.1	0.1	0.2	0	0	0
Brazil	Floresta do Piauí	1.4	1.1	1.8	0	0	0	0.2	0.1	0.2	0	0	0
Brazil	Florestal	0.3	0.2	0.4	0	0	0	0.1	0.0	0.1	0	0	0
Brazil	Florestópolis	0.3	0.2	0.3	0	0	0	0.1	0.0	0.1	0	0	0
Brazil	Floriano	1.4	1.1	1.8	8	6	10	0.2	0.1	0.2	1	1	1
Brazil	Floriano Peixoto	0.1	0.1	0.2	0	0	0	0.0	0.0	0.0	0	0	0
Brazil	Florianopolis	0.2	0.2	0.3	5	4	6	0.1	0.0	0.1	1	1	1
Brazil	Flórida	0.3	0.2	0.3	0	0	0	0.1	0.0	0.1	0	0	0
Brazil	Flórida Paulista	0.2	0.1	0.2	0	0	0	0.0	0.0	0.0	0	0	0
Brazil	Florínia	0.2	0.1	0.2	0	0	0	0.0	0.0	0.0	0	0	0
Brazil	Floriniapolis	0.2	0.2	0.3	0	0	0	0.1	0.0	0.1	0	0	0
Brazil	Fonte Boa	0.8	0.6	0.9	3	3	4	0.2	0.2	0.3	1	0	1
Brazil	Fontoura Xavier	0.1	0.1	0.2	0	0	0	0.0	0.0	0.0	0	0	0
Brazil	Formiga	0.3	0.2	0.4	1	1	2	0.1	0.0	0.1	0	0	0
Brazil	Formigueiro	0.1	0.1	0.2	0	0	0	0.0	0.0	0.0	0	0	0
Brazil	Formosa	0.2	0.1	0.2	2	1	2	0.1	0.0	0.1	0	0	1
Brazil	Formosa da Serra Negra	1.7	1.3	2.2	3	3	4	0.2	0.2	0.3	1	0	1
Brazil	Formosa do Oeste	0.3	0.2	0.3	0	0	0	0.1	0.0	0.1	0	0	0
Brazil	Formosa do Rio Preto	1.7	1.4	2.2	5	4	6	0.2	0.2	0.3	1	1	1
Brazil	Formosa do Sul	0.2	0.2	0.3	0	0	0	0.1	0.0	0.1	0	0	0
Brazil	Formoso	0.3	0.2	0.4	0	0	0	0.1	0.0	0.1	0	0	0
Brazil	Formoso	0.2	0.1	0.2	0	0	0	0.1	0.0	0.1	0	0	0

Table 3: Diarrhoea Mortality by Unit *(continued)*

Country	Unit	2000 mortality mean rate (per 10,000)	lower rate (per 10,000)	upper rate (per 10,000)	mean count	lower count	upper count	2017 mortality mean rate (per 10,000)	lower rate (per 10,000)	upper rate (per 10,000)	mean count	lower count	upper count
Brazil	Formoso do Araguaia	0.8	0.7	1.1	0	0	0	0.1	0.1	0.2	0	0	0
Brazil	Forquilha	2.3	1.7	2.9	5	4	6	0.1	0.1	0.2	0	0	0
Brazil	Forquilhinha	0.2	0.2	0.3	0	0	1	0.1	0.0	0.1	0	0	0
Brazil	Fortaleza	2.3	1.7	2.9	477	361	612	0.1	0.1	0.2	31	24	42
Brazil	Fortaleza de Minas	0.3	0.2	0.4	0	0	0	0.1	0.0	0.1	0	0	0
Brazil	Fortaleza do Tabocão	0.8	0.7	1.1	0	0	1	0.1	0.1	0.2	0	0	0
Brazil	Fortaleza dos Nogueiras	1.7	1.3	2.2	3	2	3	0.2	0.2	0.3	0	0	0
Brazil	Fortaleza dos Valos	0.1	0.1	0.2	0	0	0	0.0	0.0	0.0	0	0	0
Brazil	Fortim	2.3	1.7	2.9	2	2	3	0.1	0.1	0.2	0	0	0
Brazil	Fortuna	1.7	1.3	2.2	3	3	5	0.2	0.2	0.3	0	0	1
Brazil	Fortuna de Minas	0.3	0.2	0.4	0	0	0	0.1	0.0	0.1	0	0	0
Brazil	Foz do Iguaçu	0.3	0.2	0.3	7	6	10	0.1	0.0	0.1	1	1	1
Brazil	Foz do Jordão	0.3	0.2	0.3	0	0	0	0.1	0.0	0.1	0	0	0
Brazil	Fraiburgo	0.2	0.2	0.3	1	1	1	0.1	0.0	0.1	0	0	0
Brazil	Franca	0.2	0.1	0.2	4	3	5	0.0	0.0	0.0	1	1	1
Brazil	Francinópolis	1.4	1.1	1.8	1	1	1	0.2	0.1	0.2	0	0	0
Brazil	Francisco Alves	0.3	0.2	0.3	0	0	0	0.1	0.0	0.1	0	0	0
Brazil	Francisco Ayres	1.4	1.1	1.8	1	1	1	0.2	0.1	0.2	0	0	0
Brazil	Francisco Badaró	0.3	0.2	0.4	0	0	0	0.1	0.0	0.1	0	0	0
Brazil	Francisco Beltrão	0.3	0.2	0.3	2	1	2	0.1	0.0	0.1	0	0	0
Brazil	Francisco Dantas	1.2	0.9	1.5	1	1	1	0.1	0.1	0.2	0	0	0
Brazil	Francisco Dumon	0.3	0.2	0.4	0	0	0	0.1	0.0	0.1	0	0	0
Brazil	Francisco Macêdo	1.4	1.1	1.8	0	0	0	0.2	0.1	0.2	0	0	0
Brazil	Francisco Morato	0.2	0.1	0.2	2	1	2	0.0	0.0	0.0	0	0	1
Brazil	Francisco Sá	0.3	0.2	0.4	0	0	0	0.1	0.0	0.1	0	0	0
Brazil	Francisco Santos	1.4	1.1	1.8	1	1	1	0.2	0.1	0.2	0	0	0
Brazil	Franciscópolis	0.3	0.2	0.4	1	0	1	0.1	0.0	0.1	0	0	0
Brazil	Franco da Rocha	0.2	0.1	0.2	2	2	3	0.0	0.0	0.0	1	0	1
Brazil	Frecheirinha	2.3	1.7	2.9	3	2	4	0.1	0.1	0.2	0	0	0
Brazil	Frederico Westphalen	0.1	0.1	0.2	0	0	0	0.0	0.0	0.0	0	0	0
Brazil	Frei Gaspar	0.3	0.2	0.4	0	0	0	0.1	0.0	0.1	0	0	0
Brazil	Frei Inocêncio	0.3	0.2	0.4	0	0	0	0.1	0.0	0.1	0	0	0
Brazil	Frei Lagonegro	0.3	0.2	0.4	0	0	0	0.1	0.0	0.1	0	0	0
Brazil	Frei Martinho	1.0	0.7	1.2	0	0	0	0.1	0.1	0.1	0	0	0
Brazil	Frei Miguelinho	2.0	1.6	2.6	4	3	5	0.2	0.2	0.3	0	0	0
Brazil	Frei Paulo	1.3	1.0	1.7	2	1	2	0.2	0.1	0.3	0	0	0
Brazil	Frei Rogério	0.2	0.2	0.3	0	0	0	0.1	0.0	0.1	0	0	0
Brazil	Fronteira	0.3	0.2	0.4	0	0	0	0.1	0.0	0.1	0	0	0
Brazil	Fronteira dos Vales	0.3	0.2	0.4	0	0	0	0.1	0.0	0.1	0	0	0
Brazil	Fronteiras	1.4	1.1	1.8	2	1	2	0.2	0.1	0.2	0	0	0
Brazil	Fruta de Leite	0.3	0.2	0.4	0	0	0	0.1	0.0	0.1	0	0	0
Brazil	Frutal	0.3	0.2	0.4	1	1	1	0.1	0.0	0.1	0	0	0
Brazil	Frutuoso Gomes	1.2	0.9	1.5	0	0	1	0.1	0.1	0.2	0	0	0
Brazil	Fundão	0.3	0.2	0.3	0	0	0	0.1	0.1	0.1	0	0	0
Brazil	Funilândia	0.3	0.2	0.4	0	0	0	0.1	0.0	0.1	0	0	0
Brazil	Gabriel Monteiro	0.2	0.1	0.2	0	0	0	0.0	0.0	0.0	0	0	0
Brazil	Gado Bravo	1.0	0.7	1.2	1	1	1	0.1	0.1	0.1	0	0	0
Brazil	Gália	0.2	0.1	0.2	0	0	0	0.0	0.0	0.0	0	0	0
Brazil	Galiléia	0.3	0.2	0.4	0	0	0	0.1	0.0	0.1	0	0	0
Brazil	Galinhos	1.2	0.9	1.5	0	0	0	0.1	0.1	0.2	0	0	0
Brazil	Galvão	0.2	0.2	0.3	0	0	0	0.1	0.0	0.1	0	0	0
Brazil	Gameleira	2.0	1.6	2.6	7	6	9	0.2	0.2	0.3	1	0	1
Brazil	Gameleiras	0.3	0.2	0.4	0	0	0	0.1	0.0	0.1	0	0	0

Table 3: Diarrhoea Mortality by Unit *(continued)*

Country	Unit	2000 mortality mean rate (per 10,000)	lower rate (per 10,000)	upper rate (per 10,000)	mean count	lower count	upper count	2017 mortality mean rate (per 10,000)	lower rate (per 10,000)	upper rate (per 10,000)	mean count	lower count	upper count
Brazil	Gandu	1.7	1.4	2.2	5	4	7	0.2	0.2	0.3	1	1	1
Brazil	Garanhuns	2.0	1.6	2.6	29	23	38	0.2	0.2	0.3	3	2	4
Brazil	Gararu	1.3	1.0	1.7	2	2	3	0.2	0.1	0.3	0	0	0
Brazil	Garça	0.2	0.1	0.2	0	0	1	0.0	0.0	0.0	0	0	0
Brazil	Garibaldi	0.1	0.1	0.2	0	0	0	0.0	0.0	0.0	0	0	0
Brazil	Garopaba	0.2	0.2	0.3	0	0	0	0.1	0.0	0.1	0	0	0
Brazil	Garrafão do Norte	0.9	0.7	1.1	3	2	4	0.1	0.1	0.2	0	0	0
Brazil	Garruchos	0.1	0.1	0.2	0	0	0	0.0	0.0	0.0	0	0	0
Brazil	Garuva	0.2	0.2	0.3	0	0	0	0.1	0.0	0.1	0	0	0
Brazil	Gaspar	0.2	0.2	0.3	1	1	2	0.1	0.0	0.1	0	0	0
Brazil	Gastão Vidigal	0.2	0.1	0.2	0	0	0	0.0	0.0	0.0	0	0	0
Brazil	Gaúcha do Norte	0.4	0.3	0.5	0	0	0	0.1	0.1	0.1	0	0	0
Brazil	Gaurama	0.1	0.1	0.2	0	0	0	0.0	0.0	0.0	0	0	0
Brazil	Gavião	1.7	1.4	2.2	1	1	1	0.2	0.2	0.3	0	0	0
Brazil	Gavião Peixoto	0.2	0.1	0.2	0	0	0	0.0	0.0	0.0	0	0	0
Brazil	Geminiano	1.4	1.1	1.8	1	1	2	0.2	0.1	0.2	0	0	0
Brazil	General Câmara	0.1	0.1	0.2	0	0	0	0.0	0.0	0.0	0	0	0
Brazil	General Carneiro	0.3	0.2	0.3	0	0	1	0.1	0.0	0.1	0	0	0
Brazil	General Carneiro	0.4	0.3	0.5	0	0	0	0.1	0.1	0.1	0	0	0
Brazil	General Maynard	1.3	1.0	1.7	0	0	0	0.2	0.1	0.3	0	0	0
Brazil	General Salgado	0.2	0.1	0.2	0	0	0	0.0	0.0	0.0	0	0	0
Brazil	General Sampaio	2.3	1.7	2.9	2	1	2	0.1	0.1	0.2	0	0	0
Brazil	Gentil	0.1	0.1	0.2	0	0	0	0.0	0.0	0.0	0	0	0
Brazil	Gentio do Ouro	1.7	1.4	2.2	3	2	3	0.2	0.2	0.3	0	0	0
Brazil	Getulina	0.2	0.1	0.2	0	0	0	0.0	0.0	0.0	0	0	0
Brazil	Getúlio Vargas	0.1	0.1	0.2	0	0	0	0.0	0.0	0.0	0	0	0
Brazil	Gilbués	1.4	1.1	1.8	2	2	3	0.2	0.1	0.2	0	0	0
Brazil	Girau do Ponciano	2.8	2.2	3.7	11	8	14	0.3	0.2	0.4	1	1	2
Brazil	Giruá	0.1	0.1	0.2	0	0	0	0.0	0.0	0.0	0	0	0
Brazil	Glaucilândia	0.3	0.2	0.4	0	0	0	0.1	0.0	0.1	0	0	0
Brazil	Glicério	0.2	0.1	0.2	0	0	0	0.0	0.0	0.0	0	0	0
Brazil	Glória	1.7	1.4	2.2	3	3	4	0.2	0.2	0.3	0	0	1
Brazil	Glória d'Oeste	0.4	0.3	0.5	0	0	0	0.1	0.1	0.1	0	0	0
Brazil	Glória de Dourados	0.5	0.3	0.6	0	0	0	0.2	0.1	0.2	0	0	0
Brazil	Glória do Goitá	2.0	1.6	2.6	8	6	10	0.2	0.2	0.3	1	1	1
Brazil	Glorinha	0.1	0.1	0.2	0	0	0	0.0	0.0	0.0	0	0	0
Brazil	Godofredo Viana	1.7	1.3	2.2	2	2	3	0.2	0.2	0.3	0	0	1
Brazil	Godoy Moreira	0.3	0.2	0.3	0	0	0	0.1	0.0	0.1	0	0	0
Brazil	Goiabeira	0.3	0.2	0.4	0	0	0	0.1	0.0	0.1	0	0	0
Brazil	Goianá	0.3	0.2	0.4	0	0	0	0.1	0.0	0.1	0	0	0
Brazil	Goianá	2.0	1.6	2.6	15	12	19	0.2	0.2	0.3	1	1	2
Brazil	Goianápolis	0.2	0.1	0.2	0	0	0	0.1	0.0	0.1	0	0	0
Brazil	Goiandira	0.2	0.1	0.2	0	0	0	0.1	0.0	0.1	0	0	0
Brazil	Goianésia	0.2	0.1	0.2	1	1	1	0.1	0.0	0.1	0	0	0
Brazil	Goianésia do Pará	0.9	0.7	1.1	3	2	4	0.1	0.1	0.2	1	0	1
Brazil	Goiania	0.2	0.1	0.2	17	13	22	0.1	0.0	0.1	5	4	6
Brazil	Goianinha	1.2	0.9	1.5	3	2	4	0.1	0.1	0.2	0	0	1
Brazil	Goianira	0.2	0.1	0.2	0	0	0	0.1	0.0	0.1	0	0	0
Brazil	Goianorte	0.8	0.7	1.1	0	0	1	0.1	0.1	0.2	0	0	0
Brazil	Goiás	0.2	0.1	0.2	0	0	1	0.1	0.0	0.1	0	0	0
Brazil	Goiatins	0.8	0.7	1.1	1	1	2	0.1	0.1	0.2	0	0	0
Brazil	Goiatuba	0.2	0.1	0.2	0	0	1	0.1	0.0	0.1	0	0	0
Brazil	Goioerê	0.3	0.2	0.3	1	1	1	0.1	0.0	0.1	0	0	0
Brazil	Goioxim	0.3	0.2	0.3	0	0	0	0.1	0.0	0.1	0	0	0
Brazil	Gonçalves	0.3	0.2	0.4	0	0	0	0.1	0.0	0.1	0	0	0
Brazil	Gonçalves Dias	1.7	1.3	2.2	4	3	5	0.2	0.2	0.3	0	0	1

Table 3: Diarrhoea Mortality by Unit *(continued)*

Country	Unit	2000 mortality mean rate (per 10,000)	lower rate (per 10,000)	upper rate (per 10,000)	mean count	lower count	upper count	2017 mortality mean rate (per 10,000)	lower rate (per 10,000)	upper rate (per 10,000)	mean count	lower count	upper count
Brazil	Gongogi	1.7	1.4	2.2	3	2	4	0.2	0.2	0.3	0	0	0
Brazil	Gonzaga	0.3	0.2	0.4	0	0	0	0.1	0.0	0.1	0	0	0
Brazil	Gouvea	0.3	0.2	0.4	0	0	0	0.1	0.0	0.1	0	0	0
Brazil	Gouvelândia	0.2	0.1	0.2	0	0	0	0.1	0.0	0.1	0	0	0
Brazil	Governador Archer	1.7	1.3	2.2	2	2	3	0.2	0.2	0.3	0	0	0
Brazil	Governador Celso Ramos	0.2	0.2	0.3	0	0	0	0.1	0.0	0.1	0	0	0
Brazil	Governador Dix-Sept Rosad	1.2	0.9	1.5	2	1	2	0.1	0.1	0.2	0	0	0
Brazil	Governador Edison Lobão	1.7	1.3	2.2	2	2	3	0.2	0.2	0.3	0	0	1
Brazil	Governador Eugênio Barros	1.7	1.3	2.2	3	2	4	0.2	0.2	0.3	0	0	1
Brazil	Governador Jorge Teixeira	0.4	0.3	0.5	1	0	1	0.1	0.1	0.1	0	0	0
Brazil	Governador Luiz Rocha	1.7	1.3	2.2	2	1	2	0.2	0.2	0.3	0	0	0
Brazil	Governador Mangabeira	1.7	1.4	2.2	3	2	4	0.2	0.2	0.3	0	0	1
Brazil	Governador Newton Bello	1.7	1.3	2.2	3	3	5	0.2	0.2	0.3	0	0	1
Brazil	Governador Nunes Freire	1.7	1.3	2.2	9	7	12	0.2	0.2	0.3	1	1	1
Brazil	Governador Valadares	0.3	0.2	0.4	6	5	8	0.1	0.0	0.1	1	1	1
Brazil	Graça	2.3	1.7	2.9	8	6	10	0.1	0.1	0.2	0	0	1
Brazil	Graça Aranha	1.7	1.3	2.2	2	1	2	0.2	0.2	0.3	0	0	0
Brazil	Gracho Cardoso	1.3	1.0	1.7	1	1	1	0.2	0.1	0.3	0	0	0
Brazil	Grajaú	1.7	1.3	2.2	12	9	15	0.2	0.2	0.3	2	2	3
Brazil	Gramado	0.1	0.1	0.2	0	0	0	0.0	0.0	0.0	0	0	0
Brazil	Gramado dos Loureiros	0.1	0.1	0.2	0	0	0	0.0	0.0	0.0	0	0	0
Brazil	Gramado Xavier	0.1	0.1	0.2	0	0	0	0.0	0.0	0.0	0	0	0
Brazil	Grandes Rios	0.3	0.2	0.3	0	0	0	0.1	0.0	0.1	0	0	0
Brazil	Granito	2.0	1.6	2.6	1	1	2	0.2	0.2	0.3	0	0	0
Brazil	Granja	2.3	1.7	2.9	17	13	21	0.1	0.1	0.2	1	1	1
Brazil	Granjeiro	2.3	1.7	2.9	1	1	1	0.1	0.1	0.2	0	0	0
Brazil	Grão Mogol	0.3	0.2	0.4	0	0	1	0.1	0.0	0.1	0	0	0
Brazil	Grão Pará	0.2	0.2	0.3	0	0	0	0.1	0.0	0.1	0	0	0
Brazil	Gravatá	2.0	1.6	2.6	15	12	20	0.2	0.2	0.3	2	1	2
Brazil	Gravataí	0.2	0.2	0.3	0	0	0	0.1	0.0	0.1	0	0	0
Brazil	Gravataí	0.1	0.1	0.2	3	2	4	0.0	0.0	0.0	1	1	1
Brazil	Groaíras	1.2	0.9	1.5	1	1	1	0.1	0.1	0.2	0	0	0
Brazil	Groaíras	2.3	1.7	2.9	2	1	2	0.1	0.1	0.2	0	0	0
Brazil	Grupiara	0.3	0.2	0.4	0	0	0	0.1	0.0	0.1	0	0	0
Brazil	Guabiju	0.1	0.1	0.2	0	0	0	0.0	0.0	0.0	0	0	0
Brazil	Guabiruba	0.2	0.2	0.3	0	0	0	0.1	0.0	0.1	0	0	0
Brazil	Guaçuí	0.3	0.2	0.3	1	0	1	0.1	0.1	0.1	0	0	0
Brazil	Guadalupe	1.4	1.1	1.8	1	1	2	0.2	0.1	0.2	0	0	0
Brazil	Guaíba	0.1	0.1	0.2	1	1	1	0.0	0.0	0.0	0	0	0
Brazil	Guaiçara	0.2	0.1	0.2	0	0	0	0.0	0.0	0.0	0	0	0
Brazil	Guaimbê	0.2	0.1	0.2	0	0	0	0.0	0.0	0.0	0	0	0
Brazil	Guaíra	0.3	0.2	0.3	1	0	1	0.1	0.0	0.1	0	0	0
Brazil	Guaíra	0.2	0.1	0.2	0	0	1	0.0	0.0	0.0	0	0	0
Brazil	Guairaçá	0.3	0.2	0.3	0	0	0	0.1	0.0	0.1	0	0	0
Brazil	Guaiúba	2.3	1.7	2.9	6	4	8	0.1	0.1	0.2	0	0	1
Brazil	Guajará	0.8	0.6	0.9	4	3	5	0.2	0.2	0.3	1	1	1
Brazil	Guajará-Mirim	0.4	0.3	0.5	2	1	2	0.1	0.1	0.1	0	0	0
Brazil	Guajeru	1.7	1.4	2.2	2	1	2	0.2	0.2	0.3	0	0	0

Table 3: Diarrhoea Mortality by Unit *(continued)*

Country	Unit	2000 mortality mean rate (per 10,000)	lower rate (per 10,000)	upper rate (per 10,000)	mean count	lower count	upper count	2017 mortality mean rate (per 10,000)	lower rate (per 10,000)	upper rate (per 10,000)	mean count	lower count	upper count
Brazil	Guamaré	1.2	0.9	1.5	0	0	0	0.1	0.1	0.2	0	0	0
Brazil	Guamiranga	0.3	0.2	0.3	0	0	0	0.1	0.0	0.1	0	0	0
Brazil	Guanambi	1.7	1.4	2.2	14	11	17	0.2	0.2	0.3	2	1	2
Brazil	Guanhães	0.3	0.2	0.4	1	1	1	0.1	0.0	0.1	0	0	0
Brazil	Guapé	0.3	0.2	0.4	0	0	0	0.1	0.0	0.1	0	0	0
Brazil	Guapiaçu	0.2	0.1	0.2	0	0	0	0.0	0.0	0.0	0	0	0
Brazil	Guapiara	0.2	0.1	0.2	0	0	0	0.0	0.0	0.0	0	0	0
Brazil	Guapimirim	0.2	0.1	0.3	1	1	2	0.1	0.0	0.1	0	0	0
Brazil	Guapirama	0.3	0.2	0.3	0	0	0	0.1	0.0	0.1	0	0	0
Brazil	Guapó	0.2	0.1	0.2	0	0	0	0.1	0.0	0.1	0	0	0
Brazil	Guaporé	0.1	0.1	0.2	0	0	0	0.0	0.0	0.0	0	0	0
Brazil	Guaporema	0.3	0.2	0.3	0	0	0	0.1	0.0	0.1	0	0	0
Brazil	Guará	0.2	0.1	0.2	0	0	0	0.0	0.0	0.0	0	0	0
Brazil	Guarabira	1.0	0.7	1.2	6	5	8	0.1	0.1	0.1	1	0	1
Brazil	Guaraçaí	0.2	0.1	0.2	0	0	0	0.0	0.0	0.0	0	0	0
Brazil	Guaraci	0.3	0.2	0.3	0	0	0	0.1	0.0	0.1	0	0	0
Brazil	Guaraci	0.2	0.1	0.2	0	0	0	0.0	0.0	0.0	0	0	0
Brazil	Guaraciaba	0.2	0.2	0.3	0	0	0	0.1	0.0	0.1	0	0	0
Brazil	Guaraciaba	0.3	0.2	0.4	0	0	0	0.1	0.0	0.1	0	0	0
Brazil	Guaraciaba do Norte	2.3	1.7	2.9	10	8	13	0.1	0.1	0.2	1	0	1
Brazil	Guaraciama	0.3	0.2	0.4	0	0	0	0.1	0.0	0.1	0	0	0
Brazil	Guaraíta	0.2	0.1	0.2	0	0	0	0.1	0.0	0.1	0	0	0
Brazil	Guaramiranga	2.3	1.7	2.9	1	1	2	0.1	0.1	0.2	0	0	0
Brazil	Guaramirim	0.2	0.2	0.3	1	0	1	0.1	0.0	0.1	0	0	0
Brazil	Guaranesia	0.3	0.2	0.4	0	0	0	0.1	0.0	0.1	0	0	0
Brazil	Guarani	0.3	0.2	0.4	0	0	0	0.1	0.0	0.1	0	0	0
Brazil	Guarani das Missões	0.1	0.1	0.2	0	0	0	0.0	0.0	0.0	0	0	0
Brazil	Guarani de Goiás	0.2	0.1	0.2	0	0	0	0.1	0.0	0.1	0	0	0
Brazil	Guarani do Oeste	0.2	0.1	0.2	0	0	0	0.0	0.0	0.0	0	0	0
Brazil	Guaraniaçu	0.3	0.2	0.3	0	0	1	0.1	0.0	0.1	0	0	0
Brazil	Guarantã	0.2	0.1	0.2	0	0	0	0.0	0.0	0.0	0	0	0
Brazil	Guarantã do Norte	0.4	0.3	0.5	1	1	2	0.1	0.1	0.1	0	0	0
Brazil	Guarapari	0.3	0.2	0.3	2	1	2	0.1	0.1	0.1	0	0	1
Brazil	Guarapuava	0.3	0.2	0.3	5	3	6	0.1	0.0	0.1	1	1	1
Brazil	Guaraqueçaba	0.3	0.2	0.3	0	0	0	0.1	0.0	0.1	0	0	0
Brazil	Guarará	0.3	0.2	0.4	0	0	0	0.1	0.0	0.1	0	0	0
Brazil	Guararapes	0.2	0.1	0.2	2	1	2	0.0	0.0	0.0	0	0	0
Brazil	Guararema	0.2	0.1	0.2	0	0	0	0.0	0.0	0.0	0	0	0
Brazil	Guaratinga	1.7	1.4	2.2	5	4	6	0.2	0.2	0.3	1	0	1
Brazil	Guaratinguetá	0.2	0.1	0.2	0	0	0	0.0	0.0	0.0	0	0	0
Brazil	Guaratuba	0.3	0.2	0.3	0	0	1	0.1	0.0	0.1	0	0	0
Brazil	Guarda-Mor	0.3	0.2	0.4	0	0	0	0.1	0.0	0.1	0	0	0
Brazil	Guareí	0.2	0.1	0.2	0	0	0	0.0	0.0	0.0	0	0	0
Brazil	Guariba	0.2	0.1	0.2	0	0	1	0.0	0.0	0.0	0	0	0
Brazil	Guaribas	1.4	1.1	1.8	1	1	1	0.2	0.1	0.2	0	0	0
Brazil	Guarinos	0.2	0.1	0.2	0	0	0	0.1	0.0	0.1	0	0	0
Brazil	Guarujá	0.2	0.1	0.2	4	3	5	0.0	0.0	0.0	1	1	1
Brazil	Guarujá do Sul	0.2	0.2	0.3	0	0	0	0.1	0.0	0.1	0	0	0
Brazil	Guarulhos	0.2	0.1	0.2	17	13	22	0.0	0.0	0.0	4	3	5
Brazil	Guatambú	0.2	0.2	0.3	0	0	0	0.1	0.0	0.1	0	0	0
Brazil	Guatapará	0.2	0.1	0.2	0	0	0	0.0	0.0	0.0	0	0	0
Brazil	Guaxupé	0.3	0.2	0.4	1	1	1	0.1	0.0	0.1	0	0	0
Brazil	Guia Branca	0.3	0.2	0.3	0	0	0	0.1	0.1	0.1	0	0	0
Brazil	Guia Lopes da Laguna	0.5	0.3	0.6	1	0	1	0.2	0.1	0.2	0	0	0
Brazil	Guidoval	0.3	0.2	0.4	0	0	0	0.1	0.0	0.1	0	0	0
Brazil	Guimarães	1.7	1.3	2.2	2	2	3	0.2	0.2	0.3	0	0	0

Table 3: Diarrhoea Mortality by Unit *(continued)*

Country	Unit	2000 mortality mean rate (per 10,000)	lower rate (per 10,000)	upper rate (per 10,000)	mean count	lower count	upper count	2017 mortality mean rate (per 10,000)	lower rate (per 10,000)	upper rate (per 10,000)	mean count	lower count	upper count
Brazil	Guimarania	0.3	0.2	0.4	0	0	0	0.1	0.0	0.1	0	0	0
Brazil	Guiratinga	0.4	0.3	0.5	0	0	1	0.1	0.1	0.1	0	0	0
Brazil	Guiricema	0.3	0.2	0.4	0	0	0	0.1	0.0	0.1	0	0	0
Brazil	Gurinhatã	0.3	0.2	0.4	0	0	0	0.1	0.0	0.1	0	0	0
Brazil	Gurinhém	1.0	0.7	1.2	2	1	2	0.1	0.1	0.1	0	0	0
Brazil	Gurjão	1.0	0.7	1.2	0	0	0	0.1	0.1	0.1	0	0	0
Brazil	Gurupá	0.9	0.7	1.1	3	3	4	0.1	0.1	0.2	1	0	1
Brazil	Gurupi	0.8	0.7	1.1	6	4	7	0.1	0.1	0.2	1	1	1
Brazil	Guzolandia	0.2	0.1	0.2	0	0	0	0.0	0.0	0.0	0	0	0
Brazil	Harmonia	0.1	0.1	0.2	0	0	0	0.0	0.0	0.0	0	0	0
Brazil	Heitoraí	0.2	0.1	0.2	0	0	0	0.1	0.0	0.1	0	0	0
Brazil	Heliodora	0.3	0.2	0.4	0	0	0	0.1	0.0	0.1	0	0	0
Brazil	Heliópolis	1.7	1.4	2.2	3	2	4	0.2	0.2	0.3	0	0	0
Brazil	Herculândia	0.2	0.1	0.2	0	0	0	0.0	0.0	0.0	0	0	0
Brazil	Herval d'Oeste	0.2	0.2	0.3	0	0	1	0.1	0.0	0.1	0	0	0
Brazil	Herveiras	0.1	0.1	0.2	0	0	0	0.0	0.0	0.0	0	0	0
Brazil	Hidrolândia	0.2	0.1	0.2	0	0	1	0.1	0.0	0.1	0	0	0
Brazil	Hidrolândia	2.3	1.7	2.9	4	3	6	0.1	0.1	0.2	0	0	0
Brazil	Hidrolina	0.2	0.1	0.2	0	0	0	0.1	0.0	0.1	0	0	0
Brazil	Holambra	0.2	0.1	0.2	0	0	0	0.0	0.0	0.0	0	0	0
Brazil	Honório Serpa	0.3	0.2	0.3	0	0	0	0.1	0.0	0.1	0	0	0
Brazil	Horizonte	2.3	1.7	2.9	10	7	12	0.1	0.1	0.2	1	1	1
Brazil	Horizontina	0.1	0.1	0.2	0	0	0	0.0	0.0	0.0	0	0	0
Brazil	Hortolândia	0.2	0.1	0.2	2	1	2	0.0	0.0	0.0	0	0	1
Brazil	Hugo Napoleão	1.4	1.1	1.8	1	0	1	0.2	0.1	0.2	0	0	0
Brazil	Hulha Negra	0.1	0.1	0.2	0	0	0	0.0	0.0	0.0	0	0	0
Brazil	Humaitá	0.1	0.1	0.2	0	0	0	0.0	0.0	0.0	0	0	0
Brazil	Humaitá	0.8	0.6	0.9	3	3	4	0.2	0.2	0.3	1	1	1
Brazil	Humberto Campos	1.7	1.3	2.2	5	4	6	0.2	0.2	0.3	1	1	1
Brazil	Iacanga	0.2	0.1	0.2	0	0	0	0.0	0.0	0.0	0	0	0
Brazil	Iaciara	0.2	0.1	0.2	0	0	0	0.1	0.0	0.1	0	0	0
Brazil	Iacri	0.2	0.1	0.2	0	0	0	0.0	0.0	0.0	0	0	0
Brazil	Iaçu	1.7	1.4	2.2	5	4	7	0.2	0.2	0.3	1	0	1
Brazil	Iaçu	0.3	0.2	0.4	0	0	0	0.1	0.0	0.1	0	0	0
Brazil	Iaras	0.2	0.1	0.2	0	0	0	0.0	0.0	0.0	0	0	0
Brazil	Iati	2.0	1.6	2.6	5	4	7	0.2	0.2	0.3	1	0	1
Brazil	Ibaiti	0.3	0.2	0.3	1	1	1	0.1	0.0	0.1	0	0	0
Brazil	Ibarama	0.1	0.1	0.2	0	0	0	0.0	0.0	0.0	0	0	0
Brazil	Ibaretama	2.3	1.7	2.9	4	3	5	0.1	0.1	0.2	0	0	0
Brazil	Ibaté	0.2	0.1	0.2	0	0	0	0.0	0.0	0.0	0	0	0
Brazil	Ibateguara	2.8	2.2	3.7	6	5	8	0.3	0.2	0.4	1	0	1
Brazil	Ibatiba	0.3	0.2	0.3	1	0	1	0.1	0.1	0.1	0	0	0
Brazil	Ibema	0.3	0.2	0.3	0	0	0	0.1	0.0	0.1	0	0	0
Brazil	Ibertioga	0.3	0.2	0.4	0	0	0	0.1	0.0	0.1	0	0	0
Brazil	Ibiá	0.3	0.2	0.4	0	0	1	0.1	0.0	0.1	0	0	0
Brazil	Ibiaçá	0.1	0.1	0.2	0	0	0	0.0	0.0	0.0	0	0	0
Brazil	Ibiaí	0.3	0.2	0.4	0	0	0	0.1	0.0	0.1	0	0	0
Brazil	Ibiam	0.2	0.2	0.3	0	0	0	0.1	0.0	0.1	0	0	0
Brazil	Ibiapina	2.3	1.7	2.9	7	6	9	0.1	0.1	0.2	0	0	1
Brazil	Ibiara	1.0	0.7	1.2	1	1	1	0.1	0.1	0.1	0	0	0
Brazil	Ibiassucê	1.7	1.4	2.2	2	2	3	0.2	0.2	0.3	0	0	0
Brazil	Ibicaraí	1.7	1.4	2.2	6	5	8	0.2	0.2	0.3	1	0	1
Brazil	Ibicaré	0.2	0.2	0.3	0	0	0	0.1	0.0	0.1	0	0	0
Brazil	Ibicoara	1.7	1.4	2.2	3	2	4	0.2	0.2	0.3	0	0	1
Brazil	Ibicuí	1.7	1.4	2.2	3	3	4	0.2	0.2	0.3	0	0	1
Brazil	Ibicuitinga	2.3	1.7	2.9	2	2	3	0.1	0.1	0.2	0	0	0
Brazil	Ibimirim	2.0	1.6	2.6	8	6	10	0.2	0.2	0.3	1	1	1
Brazil	Ibipeba	1.7	1.4	2.2	3	2	4	0.2	0.2	0.3	0	0	0
Brazil	Ibipitanga	1.7	1.4	2.2	3	2	3	0.2	0.2	0.3	0	0	0

Table 3: Diarrhoea Mortality by Unit *(continued)*

Country	Unit	2000 mortality mean rate (per 10,000)	lower rate (per 10,000)	upper rate (per 10,000)	mean count	lower count	upper count	2017 mortality mean rate (per 10,000)	lower rate (per 10,000)	upper rate (per 10,000)	mean count	lower count	upper count
Brazil	Ibiporã	0.3	0.2	0.3	1	1	2	0.1	0.0	0.1	0	0	0
Brazil	Ibiquera	1.7	1.4	2.2	1	1	1	0.2	0.2	0.3	0	0	0
Brazil	Ibirá	0.2	0.1	0.2	0	0	0	0.0	0.0	0.0	0	0	0
Brazil	Ibiracatu	0.3	0.2	0.4	0	0	0	0.1	0.0	0.1	0	0	0
Brazil	Ibiraci	0.3	0.2	0.4	0	0	0	0.1	0.0	0.1	0	0	0
Brazil	Ibiraçu	0.3	0.2	0.3	0	0	0	0.1	0.1	0.1	0	0	0
Brazil	Ibiraiaras	0.1	0.1	0.2	0	0	0	0.0	0.0	0.0	0	0	0
Brazil	Ibirajuba	2.0	1.6	2.6	2	1	2	0.2	0.2	0.3	0	0	0
Brazil	Ibirama	0.2	0.2	0.3	0	0	0	0.1	0.0	0.1	0	0	0
Brazil	Ibirapitanga	1.7	1.4	2.2	5	4	6	0.2	0.2	0.3	1	0	1
Brazil	Ibirapuã	1.7	1.4	2.2	2	1	2	0.2	0.2	0.3	0	0	0
Brazil	Ibirapuitã	0.1	0.1	0.2	0	0	0	0.0	0.0	0.0	0	0	0
Brazil	Ibirarema	0.2	0.1	0.2	0	0	0	0.0	0.0	0.0	0	0	0
Brazil	Ibirataia	1.7	1.4	2.2	4	3	5	0.2	0.2	0.3	0	0	0
Brazil	Ibirité	0.3	0.2	0.4	4	3	5	0.1	0.0	0.1	1	1	1
Brazil	Ibirubá	0.1	0.1	0.2	0	0	0	0.0	0.0	0.0	0	0	0
Brazil	Ibitiara	1.7	1.4	2.2	2	2	3	0.2	0.2	0.3	0	0	0
Brazil	Ibitinga	0.2	0.1	0.2	1	0	1	0.0	0.0	0.0	0	0	0
Brazil	Ibitirama	0.3	0.2	0.3	0	0	0	0.1	0.1	0.1	0	0	0
Brazil	Ibititá	1.7	1.4	2.2	4	3	5	0.2	0.2	0.3	0	0	1
Brazil	Ibitiúra de Minas	0.3	0.2	0.4	0	0	0	0.1	0.0	0.1	0	0	0
Brazil	Ibituruna	0.3	0.2	0.4	0	0	0	0.1	0.0	0.1	0	0	0
Brazil	Ibiúna	0.2	0.1	0.2	1	1	1	0.0	0.0	0.0	0	0	0
Brazil	Ibotirama	1.7	1.4	2.2	4	3	5	0.2	0.2	0.3	1	0	1
Brazil	Icapuí	2.3	1.7	2.9	3	2	4	0.1	0.1	0.2	0	0	0
Brazil	Içara	0.2	0.2	0.3	1	1	2	0.1	0.0	0.1	0	0	0
Brazil	Icaraí de Minas	0.3	0.2	0.4	0	0	0	0.1	0.0	0.1	0	0	0
Brazil	Icaraíma	0.3	0.2	0.3	0	0	0	0.1	0.0	0.1	0	0	0
Brazil	Icatu	1.7	1.3	2.2	6	5	8	0.2	0.2	0.3	1	1	1
Brazil	Icém	0.2	0.1	0.2	0	0	0	0.0	0.0	0.0	0	0	0
Brazil	Ichu	1.7	1.4	2.2	1	1	1	0.2	0.2	0.3	0	0	0
Brazil	Icó	2.3	1.7	2.9	16	12	21	0.1	0.1	0.2	1	1	1
Brazil	Iconha	0.3	0.2	0.3	0	0	0	0.1	0.1	0.1	0	0	0
Brazil	Ielmo Marinho	1.2	0.9	1.5	2	1	2	0.1	0.1	0.2	0	0	0
Brazil	Iepê	0.2	0.1	0.2	0	0	0	0.0	0.0	0.0	0	0	0
Brazil	Igaci	2.8	2.2	3.7	8	6	11	0.3	0.2	0.4	1	1	1
Brazil	Igaporã	1.7	1.4	2.2	2	2	3	0.2	0.2	0.3	0	0	0
Brazil	Igaracu	2.0	1.6	2.6	21	17	28	0.2	0.2	0.3	2	2	3
Brazil	Igaraçu do Tietê	0.2	0.1	0.2	0	0	0	0.0	0.0	0.0	0	0	0
Brazil	Igarapava	0.2	0.1	0.2	0	0	0	0.0	0.0	0.0	0	0	0
Brazil	Igarapé	0.3	0.2	0.4	1	0	1	0.1	0.0	0.1	0	0	0
Brazil	Igarapé do Meio	1.7	1.3	2.2	2	2	3	0.2	0.2	0.3	0	0	0
Brazil	Igarapé Grande	1.7	1.3	2.2	3	2	4	0.2	0.2	0.3	0	0	1
Brazil	Igarapé-Açu	0.9	0.7	1.1	3	2	4	0.1	0.1	0.2	0	0	1
Brazil	Igarapé-Miri	0.9	0.7	1.1	6	5	8	0.1	0.1	0.2	1	1	1
Brazil	Igaratá	0.2	0.1	0.2	0	0	0	0.0	0.0	0.0	0	0	0
Brazil	Igaratinga	0.3	0.2	0.4	0	0	0	0.1	0.0	0.1	0	0	0
Brazil	Igrapiúna	1.7	1.4	2.2	4	3	5	0.2	0.2	0.3	0	0	1
Brazil	Igreja Nova	2.8	2.2	3.7	7	6	9	0.3	0.2	0.4	1	1	1
Brazil	Igrejinha	0.1	0.1	0.2	0	0	0	0.0	0.0	0.0	0	0	0
Brazil	Iguaba Grande	0.2	0.1	0.3	0	0	0	0.1	0.0	0.1	0	0	0
Brazil	Iguaí	1.7	1.4	2.2	5	4	6	0.2	0.2	0.3	1	0	1
Brazil	Iguape	0.2	0.1	0.2	0	0	0	0.0	0.0	0.0	0	0	0
Brazil	Iguaraci	2.0	1.6	2.6	3	2	4	0.2	0.2	0.3	0	0	0
Brazil	Iguaraçu	0.3	0.2	0.3	0	0	0	0.1	0.0	0.1	0	0	0
Brazil	Iguatama	0.3	0.2	0.4	0	0	0	0.1	0.0	0.1	0	0	0
Brazil	Iguatemi	0.5	0.3	0.6	1	1	1	0.2	0.1	0.2	0	0	0
Brazil	Iguatu	0.3	0.2	0.3	0	0	0	0.1	0.0	0.1	0	0	0
Brazil	Iguatu	2.3	1.7	2.9	19	14	24	0.1	0.1	0.2	1	1	2
Brazil	Ijaci	0.3	0.2	0.4	0	0	0	0.1	0.0	0.1	0	0	0

Table 3: Diarrhoea Mortality by Unit *(continued)*

Country	Unit	2000 mortality mean rate (per 10,000)	lower rate (per 10,000)	upper rate (per 10,000)	mean count	lower count	upper count	2017 mortality mean rate (per 10,000)	lower rate (per 10,000)	upper rate (per 10,000)	mean count	lower count	upper count
Brazil	Ijuí	0.1	0.1	0.2	1	1	1	0.0	0.0	0.0	0	0	0
Brazil	Ilha das Flores	1.3	1.0	1.7	1	1	1	0.2	0.1	0.3	0	0	0
Brazil	Ilha Grande	1.4	1.1	1.8	1	1	2	0.2	0.1	0.2	0	0	0
Brazil	Ilha Solteira	0.2	0.1	0.2	0	0	0	0.0	0.0	0.0	0	0	0
Brazil	Ilha Trindade	0.3	0.2	0.3	0	0	0	0.1	0.1	0.1	0	0	0
Brazil	Ilhabela	0.2	0.1	0.2	0	0	0	0.0	0.0	0.0	0	0	0
Brazil	Ilhas de Martim Vaz	0.3	0.2	0.3	0	0	0	0.1	0.1	0.1	0	0	0
Brazil	Ilhéus	1.7	1.4	2.2	25	20	32	0.2	0.2	0.3	2	2	3
Brazil	Ilhota	0.2	0.2	0.3	0	0	0	0.1	0.0	0.1	0	0	0
Brazil	Ilicínea	0.3	0.2	0.4	0	0	0	0.1	0.0	0.1	0	0	0
Brazil	Ilópolis	0.1	0.1	0.2	0	0	0	0.0	0.0	0.0	0	0	0
Brazil	Imaculada	1.0	0.7	1.2	2	1	2	0.1	0.1	0.1	0	0	0
Brazil	Imaruí	0.2	0.2	0.3	0	0	0	0.1	0.0	0.1	0	0	0
Brazil	Imbaú	0.3	0.2	0.3	0	0	0	0.1	0.0	0.1	0	0	0
Brazil	Imbé	0.1	0.1	0.2	0	0	0	0.0	0.0	0.0	0	0	0
Brazil	Imbé de Minas	0.3	0.2	0.4	0	0	0	0.1	0.0	0.1	0	0	0
Brazil	Imbituba	0.2	0.2	0.3	1	0	1	0.1	0.0	0.1	0	0	0
Brazil	Imbituva	0.3	0.2	0.3	1	0	1	0.1	0.0	0.1	0	0	0
Brazil	Imbuia	0.2	0.2	0.3	0	0	0	0.1	0.0	0.1	0	0	0
Brazil	Imigrante	0.1	0.1	0.2	0	0	0	0.0	0.0	0.0	0	0	0
Brazil	Imperatriz	1.7	1.3	2.2	50	38	64	0.2	0.2	0.3	6	4	8
Brazil	Inácio Martins	0.3	0.2	0.3	0	0	0	0.1	0.0	0.1	0	0	0
Brazil	Inaciolândia	0.2	0.1	0.2	0	0	0	0.1	0.0	0.1	0	0	0
Brazil	Inajá	0.3	0.2	0.3	0	0	0	0.1	0.0	0.1	0	0	0
Brazil	Inajá	2.0	1.6	2.6	3	2	4	0.2	0.2	0.3	0	0	1
Brazil	Inconfidentes	0.3	0.2	0.4	0	0	0	0.1	0.0	0.1	0	0	0
Brazil	Indaiabira	0.3	0.2	0.4	0	0	0	0.1	0.0	0.1	0	0	0
Brazil	Indaial	0.2	0.2	0.3	1	1	1	0.1	0.0	0.1	0	0	0
Brazil	Indaiatuba	0.2	0.1	0.2	2	1	2	0.0	0.0	0.0	1	0	1
Brazil	Independência	0.1	0.1	0.2	0	0	0	0.0	0.0	0.0	0	0	0
Brazil	Independência	2.3	1.7	2.9	7	5	9	0.1	0.1	0.2	0	0	0
Brazil	Indiana	0.2	0.1	0.2	0	0	0	0.0	0.0	0.0	0	0	0
Brazil	Indianópolis	0.3	0.2	0.3	0	0	0	0.1	0.0	0.1	0	0	0
Brazil	Indianópolis	0.3	0.2	0.4	0	0	0	0.1	0.0	0.1	0	0	0
Brazil	Indiaporã	0.2	0.1	0.2	0	0	0	0.0	0.0	0.0	0	0	0
Brazil	Indiara	0.2	0.1	0.2	0	0	0	0.1	0.0	0.1	0	0	0
Brazil	Indiaroba	1.3	1.0	1.7	2	2	3	0.2	0.1	0.3	0	0	0
Brazil	Indiavaí	0.4	0.3	0.5	0	0	0	0.1	0.1	0.1	0	0	0
Brazil	Ingá	1.0	0.7	1.2	2	2	3	0.1	0.1	0.1	0	0	0
Brazil	Ingaí	0.3	0.2	0.4	0	0	0	0.1	0.0	0.1	0	0	0
Brazil	Ingazeira	2.0	1.6	2.6	1	1	1	0.2	0.2	0.3	0	0	0
Brazil	Inhacor	0.1	0.1	0.2	0	0	0	0.0	0.0	0.0	0	0	0
Brazil	Inhambupe	1.7	1.4	2.2	7	6	9	0.2	0.2	0.3	1	1	1
Brazil	Inhangapi	0.9	0.7	1.1	1	1	1	0.1	0.1	0.2	0	0	0
Brazil	Inhapi	2.8	2.2	3.7	8	6	10	0.3	0.2	0.4	1	1	1
Brazil	Inhapim	0.3	0.2	0.4	1	0	1	0.1	0.0	0.1	0	0	0
Brazil	Inhaúma	0.3	0.2	0.4	0	0	0	0.1	0.0	0.1	0	0	0
Brazil	Inhuma	1.4	1.1	1.8	2	2	3	0.2	0.1	0.2	0	0	0
Brazil	Inhumas	0.2	0.1	0.2	1	1	1	0.1	0.0	0.1	0	0	0
Brazil	Inimutaba	0.3	0.2	0.4	0	0	0	0.1	0.0	0.1	0	0	0
Brazil	Inocência	0.5	0.3	0.6	0	0	0	0.2	0.1	0.2	0	0	0
Brazil	Inúbia Paulista	0.2	0.1	0.2	0	0	0	0.0	0.0	0.0	0	0	0
Brazil	Iomerê	0.2	0.2	0.3	0	0	0	0.1	0.0	0.1	0	0	0
Brazil	Ipaba	0.3	0.2	0.4	0	0	0	0.1	0.0	0.1	0	0	0
Brazil	Ipameri	0.2	0.1	0.2	0	0	0	0.1	0.0	0.1	0	0	0
Brazil	Ipanema	0.3	0.2	0.4	0	0	0	0.1	0.0	0.1	0	0	0
Brazil	Ipanguaçu	1.2	0.9	1.5	3	2	4	0.1	0.1	0.2	0	0	1
Brazil	Ipaporanga	2.3	1.7	2.9	3	2	3	0.1	0.1	0.2	0	0	0
Brazil	Ipatinga	0.3	0.2	0.4	5	4	6	0.1	0.0	0.1	1	1	1

Table 3: Diarrhoea Mortality by Unit *(continued)*

Country	Unit	2000 mortality mean rate (per 10,000)	lower rate (per 10,000)	upper rate (per 10,000)	mean count	lower count	upper count	2017 mortality mean rate (per 10,000)	lower rate (per 10,000)	upper rate (per 10,000)	mean count	lower count	upper count
Brazil	Ipaucu	0.2	0.1	0.2	0	0	0	0.0	0.0	0.0	0	0	0
Brazil	Ipaumirim	2.3	1.7	2.9	3	2	3	0.1	0.1	0.2	0	0	0
Brazil	Ipê	0.1	0.1	0.2	0	0	0	0.0	0.0	0.0	0	0	0
Brazil	Ipecaetá	1.7	1.4	2.2	4	3	5	0.2	0.2	0.3	0	0	0
Brazil	Iperó	0.2	0.1	0.2	0	0	0	0.0	0.0	0.0	0	0	0
Brazil	Ipeúna	0.2	0.1	0.2	0	0	0	0.0	0.0	0.0	0	0	0
Brazil	Ipiaçu	0.3	0.2	0.4	0	0	0	0.1	0.0	0.1	0	0	0
Brazil	Ipiaú	1.7	1.4	2.2	5	4	7	0.2	0.2	0.3	1	0	1
Brazil	Ipiguá	0.2	0.1	0.2	0	0	0	0.0	0.0	0.0	0	0	0
Brazil	Ipirá	0.2	0.2	0.3	0	0	0	0.1	0.0	0.1	0	0	0
Brazil	Ipirá	1.7	1.4	2.2	13	10	17	0.2	0.2	0.3	1	1	2
Brazil	Ipiranga	0.3	0.2	0.3	0	0	1	0.1	0.0	0.1	0	0	0
Brazil	Ipiranga do Piauí	1.4	1.1	1.8	1	1	2	0.2	0.1	0.2	0	0	0
Brazil	Ipiranga do Sul	0.1	0.1	0.2	0	0	0	0.0	0.0	0.0	0	0	0
Brazil	Ipixuna	0.8	0.6	0.9	2	1	2	0.2	0.2	0.3	1	1	1
Brazil	Ipixuna do Pará	0.9	0.7	1.1	3	2	3	0.1	0.1	0.2	1	1	1
Brazil	Ipojuca	2.0	1.6	2.6	15	12	20	0.2	0.2	0.3	2	2	3
Brazil	Iporá	0.2	0.1	0.2	0	0	1	0.1	0.0	0.1	0	0	0
Brazil	Iporá	0.3	0.2	0.3	0	0	0	0.1	0.0	0.1	0	0	0
Brazil	Iporã	0.3	0.2	0.3	0	0	0	0.1	0.0	0.1	0	0	0
Brazil	Iporã do Oeste	0.2	0.2	0.3	0	0	0	0.1	0.0	0.1	0	0	0
Brazil	Iporanga	0.2	0.1	0.2	0	0	0	0.0	0.0	0.0	0	0	0
Brazil	Ipú	2.3	1.7	2.9	11	8	14	0.1	0.1	0.2	1	0	1
Brazil	Ipuã	0.2	0.1	0.2	0	0	0	0.0	0.0	0.0	0	0	0
Brazil	Ipuaçu	0.2	0.2	0.3	0	0	0	0.1	0.0	0.1	0	0	0
Brazil	Ipubi	2.0	1.6	2.6	7	6	10	0.2	0.2	0.3	1	1	1
Brazil	Ipueira	1.2	0.9	1.5	0	0	0	0.1	0.1	0.2	0	0	0
Brazil	Ipueiras	0.8	0.7	1.1	0	0	0	0.1	0.1	0.2	0	0	0
Brazil	Ipueiras	2.3	1.7	2.9	11	9	14	0.1	0.1	0.2	1	0	1
Brazil	Ipuiúna	0.3	0.2	0.4	0	0	0	0.1	0.0	0.1	0	0	0
Brazil	Ipumirim	0.2	0.2	0.3	0	0	0	0.1	0.0	0.1	0	0	0
Brazil	Ipupiara	1.7	1.4	2.2	1	1	2	0.2	0.2	0.3	0	0	0
Brazil	Iracema	0.7	0.6	0.9	0	0	0	0.2	0.2	0.3	0	0	0
Brazil	Iracema	2.3	1.7	2.9	3	2	4	0.1	0.1	0.2	0	0	0
Brazil	Iracema do Oeste	0.3	0.2	0.3	0	0	0	0.1	0.0	0.1	0	0	0
Brazil	Iracemápolis	0.2	0.1	0.2	0	0	0	0.0	0.0	0.0	0	0	0
Brazil	Iraceminha	0.2	0.2	0.3	0	0	0	0.1	0.0	0.1	0	0	0
Brazil	Iraí	0.1	0.1	0.2	0	0	0	0.0	0.0	0.0	0	0	0
Brazil	Iraí de Minas	0.3	0.2	0.4	0	0	0	0.1	0.0	0.1	0	0	0
Brazil	Irajuba	1.7	1.4	2.2	1	1	2	0.2	0.2	0.3	0	0	0
Brazil	Iramaia	1.7	1.4	2.2	4	3	5	0.2	0.2	0.3	0	0	0
Brazil	Iranduba	0.8	0.6	0.9	3	2	3	0.2	0.2	0.3	1	1	1
Brazil	Irani	0.2	0.2	0.3	0	0	0	0.1	0.0	0.1	0	0	0
Brazil	Irapuã	0.2	0.1	0.2	0	0	0	0.0	0.0	0.0	0	0	0
Brazil	Irapuru	0.2	0.1	0.2	0	0	0	0.0	0.0	0.0	0	0	0
Brazil	Iraquara	1.7	1.4	2.2	4	3	5	0.2	0.2	0.3	1	0	1
Brazil	Irará	1.7	1.4	2.2	5	4	6	0.2	0.2	0.3	1	0	1
Brazil	Irati	0.2	0.2	0.3	0	0	0	0.1	0.0	0.1	0	0	0
Brazil	Irati	0.3	0.2	0.3	1	1	2	0.1	0.0	0.1	0	0	0
Brazil	Irauçuba	2.3	1.7	2.9	7	5	8	0.1	0.1	0.2	0	0	0
Brazil	Irecê	1.7	1.4	2.2	12	9	15	0.2	0.2	0.3	2	1	2
Brazil	Iretama	0.3	0.2	0.3	0	0	0	0.1	0.0	0.1	0	0	0
Brazil	Irineópolis	0.2	0.2	0.3	0	0	0	0.1	0.0	0.1	0	0	0
Brazil	Irituia	0.9	0.7	1.1	3	3	4	0.1	0.1	0.2	0	0	1
Brazil	Irupi	0.3	0.2	0.3	0	0	0	0.1	0.1	0.1	0	0	0
Brazil	Isaías Coelho	1.4	1.1	1.8	1	1	1	0.2	0.1	0.2	0	0	0
Brazil	Israelândia	0.2	0.1	0.2	0	0	0	0.1	0.0	0.1	0	0	0
Brazil	Itá	0.2	0.2	0.3	0	0	0	0.1	0.0	0.1	0	0	0
Brazil	Itaara	0.1	0.1	0.2	0	0	0	0.0	0.0	0.0	0	0	0
Brazil	Itabaiana	1.3	1.0	1.7	11	8	14	0.2	0.1	0.3	1	1	2

Table 3: Diarrhoea Mortality by Unit *(continued)*

Country	Unit	2000 mortality mean rate (per 10,000)	lower rate (per 10,000)	upper rate (per 10,000)	mean count	lower count	upper count	2017 mortality mean rate (per 10,000)	lower rate (per 10,000)	upper rate (per 10,000)	mean count	lower count	upper count
Brazil	Itabaiana	1.0	0.7	1.2	3	2	4	0.1	0.1	0.1	0	0	0
Brazil	Itabaianinha	1.3	1.0	1.7	6	4	7	0.2	0.1	0.3	1	1	1
Brazil	Itabela	1.7	1.4	2.2	7	5	8	0.2	0.2	0.3	1	1	1
Brazil	Itaberá	0.2	0.1	0.2	0	0	0	0.0	0.0	0.0	0	0	0
Brazil	Itaberaba	1.7	1.4	2.2	12	9	15	0.2	0.2	0.3	2	1	2
Brazil	Itaberaí	0.2	0.1	0.2	0	0	1	0.1	0.0	0.1	0	0	0
Brazil	Itabi	1.3	1.0	1.7	1	1	1	0.2	0.1	0.3	0	0	0
Brazil	Itabira	0.3	0.2	0.4	2	2	3	0.1	0.0	0.1	1	0	1
Brazil	Itabirinha de Mantena	0.3	0.2	0.4	0	0	0	0.1	0.0	0.1	0	0	0
Brazil	Itabirito	0.3	0.2	0.4	1	1	1	0.1	0.0	0.1	0	0	0
Brazil	Itaboraí	0.2	0.1	0.3	4	3	5	0.1	0.0	0.1	1	1	1
Brazil	Itabuna	1.7	1.4	2.2	35	28	45	0.2	0.2	0.3	4	3	6
Brazil	Itacajá	0.8	0.7	1.1	1	0	1	0.1	0.1	0.2	0	0	0
Brazil	Itacarambi	0.3	0.2	0.4	0	0	0	0.1	0.0	0.1	0	0	0
Brazil	Itacarambira	0.3	0.2	0.4	1	1	1	0.1	0.0	0.1	0	0	0
Brazil	Itacaré	1.7	1.4	2.2	3	3	4	0.2	0.2	0.3	0	0	1
Brazil	Itacoatiara	0.8	0.6	0.9	7	5	8	0.2	0.2	0.3	2	2	3
Brazil	Itacuruba	2.0	1.6	2.6	1	1	1	0.2	0.2	0.3	0	0	0
Brazil	Itacurubi	0.1	0.1	0.2	0	0	0	0.0	0.0	0.0	0	0	0
Brazil	Itaeté	1.7	1.4	2.2	2	2	3	0.2	0.2	0.3	0	0	0
Brazil	Itagi	1.7	1.4	2.2	2	2	3	0.2	0.2	0.3	0	0	0
Brazil	Itagibá	1.7	1.4	2.2	7	6	10	0.2	0.2	0.3	1	1	1
Brazil	Itagimirim	1.7	1.4	2.2	2	1	2	0.2	0.2	0.3	0	0	0
Brazil	Itaguaçu	0.3	0.2	0.3	0	0	1	0.1	0.1	0.1	0	0	0
Brazil	Itaguaçu da Bahia	1.7	1.4	2.2	3	2	4	0.2	0.2	0.3	0	0	0
Brazil	Itaguaí	0.2	0.1	0.3	1	1	1	0.1	0.0	0.1	0	0	0
Brazil	Itaguajé	0.3	0.2	0.3	0	0	0	0.1	0.0	0.1	0	0	0
Brazil	Itaguara	0.3	0.2	0.4	0	0	0	0.1	0.0	0.1	0	0	0
Brazil	Itaguari	0.2	0.1	0.2	0	0	0	0.1	0.0	0.1	0	0	0
Brazil	Itaguaru	0.2	0.1	0.2	0	0	0	0.1	0.0	0.1	0	0	0
Brazil	Itaguatins	0.8	0.7	1.1	1	0	1	0.1	0.1	0.2	0	0	0
Brazil	Itaí	0.2	0.1	0.2	0	0	0	0.0	0.0	0.0	0	0	0
Brazil	Itaíba	2.0	1.6	2.6	9	7	11	0.2	0.2	0.3	1	1	1
Brazil	Itaiçaba	2.3	1.7	2.9	2	1	2	0.1	0.1	0.2	0	0	0
Brazil	Itainópolis	1.4	1.1	1.8	2	1	2	0.2	0.1	0.2	0	0	0
Brazil	Itaiópolis	0.2	0.2	0.3	0	0	1	0.1	0.0	0.1	0	0	0
Brazil	Itaipava do Grajaú	1.7	1.3	2.2	3	3	4	0.2	0.2	0.3	0	0	1
Brazil	Itaipé	0.3	0.2	0.4	0	0	0	0.1	0.0	0.1	0	0	0
Brazil	Itaipulândia	0.3	0.2	0.3	0	0	0	0.1	0.0	0.1	0	0	0
Brazil	Itaitinga	2.3	1.7	2.9	8	6	10	0.1	0.1	0.2	1	0	1
Brazil	Itaituba	0.9	0.7	1.1	10	8	12	0.1	0.1	0.2	1	1	2
Brazil	Itajá	0.2	0.1	0.2	0	0	0	0.1	0.0	0.1	0	0	0
Brazil	Itajá	1.2	0.9	1.5	1	1	2	0.1	0.1	0.2	0	0	0
Brazil	Itajaí	0.2	0.2	0.3	3	2	4	0.1	0.0	0.1	1	1	1
Brazil	Itajobi	0.2	0.1	0.2	0	0	0	0.0	0.0	0.0	0	0	0
Brazil	Itaju	0.2	0.1	0.2	0	0	0	0.0	0.0	0.0	0	0	0
Brazil	Itaju do Colônia	1.7	1.4	2.2	2	2	3	0.2	0.2	0.3	0	0	0
Brazil	Itajubá	0.3	0.2	0.4	2	2	3	0.1	0.0	0.1	0	0	0
Brazil	Itajuípe	1.7	1.4	2.2	5	4	6	0.2	0.2	0.3	0	0	1
Brazil	Italva	0.2	0.1	0.3	0	0	0	0.1	0.0	0.1	0	0	0
Brazil	Itamaraju	1.7	1.4	2.2	13	11	17	0.2	0.2	0.3	1	1	2
Brazil	Itamarandiba	0.3	0.2	0.4	1	1	1	0.1	0.0	0.1	0	0	0
Brazil	Itamarati	0.8	0.6	0.9	1	1	1	0.2	0.2	0.3	0	0	0
Brazil	Itamarati de Minas	0.3	0.2	0.4	0	0	0	0.1	0.0	0.1	0	0	0
Brazil	Itamari	1.7	1.4	2.2	2	1	2	0.2	0.2	0.3	0	0	0
Brazil	Itambacuri	0.3	0.2	0.4	0	0	0	0.1	0.0	0.1	0	0	0

Table 3: Diarrhoea Mortality by Unit *(continued)*

Country	Unit	2000 mortality mean rate (per 10,000)	lower rate (per 10,000)	upper rate (per 10,000)	mean count	lower count	upper count	2017 mortality mean rate (per 10,000)	lower rate (per 10,000)	upper rate (per 10,000)	mean count	lower count	upper count
Brazil	Itambaraca	0.3	0.2	0.3	0	0	0	0.1	0.0	0.1	0	0	0
Brazil	Itambaracá	2.0	1.6	2.6	2	2	3	0.2	0.2	0.3	0	0	0
Brazil	Itambé	0.3	0.2	0.3	0	0	0	0.1	0.0	0.1	0	0	0
Brazil	Itambé	1.7	1.4	2.2	6	4	7	0.2	0.2	0.3	0	0	1
Brazil	Itambé do Mato Dentro	0.3	0.2	0.4	1	0	1	0.1	0.0	0.1	0	0	0
Brazil	Itamogi	0.3	0.2	0.4	0	0	0	0.1	0.0	0.1	0	0	0
Brazil	Itamonte	0.3	0.2	0.4	0	0	0	0.1	0.0	0.1	0	0	0
Brazil	Itanagra	1.7	1.4	2.2	1	1	2	0.2	0.2	0.3	0	0	0
Brazil	Itanhaém	0.2	0.1	0.2	1	1	1	0.0	0.0	0.0	0	0	0
Brazil	Itanhandu	0.3	0.2	0.4	0	0	0	0.1	0.0	0.1	0	0	0
Brazil	Itanhém	1.7	1.4	2.2	4	3	5	0.2	0.2	0.3	0	0	1
Brazil	Itanhomi	0.3	0.2	0.4	1	0	1	0.1	0.0	0.1	0	0	0
Brazil	Itaobim	0.3	0.2	0.4	0	0	0	0.1	0.0	0.1	0	0	0
Brazil	Itaóca	0.2	0.1	0.2	0	0	0	0.0	0.0	0.0	0	0	0
Brazil	Itaocara	0.2	0.1	0.3	0	0	0	0.1	0.0	0.1	0	0	0
Brazil	Itapaci	0.2	0.1	0.2	0	0	0	0.1	0.0	0.1	0	0	0
Brazil	Itapagipe	0.3	0.2	0.4	1	0	1	0.1	0.0	0.1	0	0	0
Brazil	Itapajé	2.3	1.7	2.9	13	10	17	0.1	0.1	0.2	1	1	1
Brazil	Itaparica	1.7	1.4	2.2	0	0	0	0.2	0.2	0.3	0	0	0
Brazil	Itapé	1.7	1.4	2.2	3	2	4	0.2	0.2	0.3	0	0	0
Brazil	Itapebi	1.7	1.4	2.2	2	2	3	0.2	0.2	0.3	0	0	0
Brazil	Itapecerica	0.3	0.2	0.4	1	1	1	0.1	0.0	0.1	0	0	0
Brazil	Itapecerica da Serra	0.2	0.1	0.2	2	1	3	0.0	0.0	0.0	0	0	1
Brazil	Itapecuru Mirim	1.7	1.3	2.2	9	7	12	0.2	0.2	0.3	2	1	2
Brazil	Itapejara d'Oeste	0.3	0.2	0.3	0	0	0	0.1	0.0	0.1	0	0	0
Brazil	Itapema	0.2	0.2	0.3	0	0	0	0.1	0.0	0.1	0	0	0
Brazil	Itapemirim	0.3	0.2	0.3	1	0	1	0.1	0.1	0.1	0	0	0
Brazil	Itaperuçu	0.3	0.2	0.3	0	0	0	0.1	0.0	0.1	0	0	0
Brazil	Itaperuna	0.2	0.1	0.3	1	1	2	0.1	0.0	0.1	0	0	0
Brazil	Itapetim	2.0	1.6	2.6	4	3	5	0.2	0.2	0.3	0	0	0
Brazil	Itapetinga	1.7	1.4	2.2	12	9	15	0.2	0.2	0.3	2	1	2
Brazil	Itapetininga	0.2	0.1	0.2	2	1	2	0.0	0.0	0.0	0	0	1
Brazil	Itapeva	0.2	0.1	0.2	1	1	2	0.0	0.0	0.0	0	0	0
Brazil	Itapeva	0.3	0.2	0.4	0	0	0	0.1	0.0	0.1	0	0	0
Brazil	Itapevi	0.2	0.1	0.2	2	2	3	0.0	0.0	0.0	1	0	1
Brazil	Itapicuru	1.7	1.4	2.2	8	6	10	0.2	0.2	0.3	1	1	1
Brazil	Itapipoca	2.3	1.7	2.9	28	21	36	0.1	0.1	0.2	2	2	3
Brazil	Itapira	0.2	0.1	0.2	1	0	1	0.0	0.0	0.0	0	0	0
Brazil	Itapiranga	0.2	0.2	0.3	0	0	1	0.1	0.0	0.1	0	0	0
Brazil	Itapirapuã	0.2	0.1	0.2	0	0	0	0.1	0.0	0.1	0	0	0
Brazil	Itapirapuã Paulista	0.2	0.1	0.2	0	0	0	0.0	0.0	0.0	0	0	0
Brazil	Itapiratins	0.8	0.7	1.1	0	0	0	0.1	0.1	0.2	0	0	0
Brazil	Itapissuma	2.0	1.6	2.6	5	4	7	0.2	0.2	0.3	1	0	1
Brazil	Itapitanga	1.7	1.4	2.2	2	1	2	0.2	0.2	0.3	0	0	0
Brazil	Itapiúna	2.3	1.7	2.9	5	4	6	0.1	0.1	0.2	0	0	0
Brazil	Itapoá	0.2	0.2	0.3	0	0	0	0.1	0.0	0.1	0	0	0
Brazil	Itápolis	0.2	0.1	0.2	0	0	1	0.0	0.0	0.0	0	0	0
Brazil	Itaporã	0.5	0.3	0.6	1	1	1	0.2	0.1	0.2	0	0	0
Brazil	Itaporã do Tocantins	0.8	0.7	1.1	0	0	0	0.1	0.1	0.2	0	0	0
Brazil	Itaporanga	0.2	0.1	0.2	0	0	0	0.0	0.0	0.0	0	0	0
Brazil	Itaporanga	1.0	0.7	1.2	2	2	3	0.1	0.1	0.1	0	0	0
Brazil	Itaporanga dajuda	1.3	1.0	1.7	4	3	5	0.2	0.1	0.3	1	0	1
Brazil	Itapororoca	1.0	0.7	1.2	2	1	2	0.1	0.1	0.1	0	0	0
Brazil	Itapuã do Oeste	0.4	0.3	0.5	0	0	0	0.1	0.1	0.1	0	0	0
Brazil	Itapuca	0.1	0.1	0.2	0	0	0	0.0	0.0	0.0	0	0	0

Table 3: Diarrhoea Mortality by Unit *(continued)*

Country	Unit	2000 mortality mean rate (per 10,000)	lower rate (per 10,000)	upper rate (per 10,000)	mean count	lower count	upper count	2017 mortality mean rate (per 10,000)	lower rate (per 10,000)	upper rate (per 10,000)	mean count	lower count	upper count
Brazil	Itapuí	0.2	0.1	0.2	0	0	0	0.0	0.0	0.0	0	0	0
Brazil	Itapura	0.2	0.1	0.2	0	0	0	0.0	0.0	0.0	0	0	0
Brazil	Itapuranga	0.2	0.1	0.2	0	0	1	0.1	0.0	0.1	0	0	0
Brazil	Itaquaquecetuba	0.2	0.1	0.2	5	4	6	0.0	0.0	0.0	1	1	1
Brazil	Itaquara	1.7	1.4	2.2	1	1	2	0.2	0.2	0.3	0	0	0
Brazil	Itaqui	0.1	0.1	0.2	1	0	1	0.0	0.0	0.0	0	0	0
Brazil	Itaquiraí	0.5	0.3	0.6	1	1	1	0.2	0.1	0.2	0	0	0
Brazil	Itaquitinga	2.0	1.6	2.6	2	1	2	0.2	0.2	0.3	0	0	0
Brazil	Itarana	0.3	0.2	0.3	0	0	0	0.1	0.1	0.1	0	0	0
Brazil	Itarantim	1.7	1.4	2.2	4	3	5	0.2	0.2	0.3	0	0	1
Brazil	Itararé	0.2	0.1	0.2	1	1	1	0.0	0.0	0.0	0	0	0
Brazil	Itariri	0.2	0.1	0.2	0	0	0	0.0	0.0	0.0	0	0	0
Brazil	Itaruma	0.2	0.1	0.2	0	0	0	0.1	0.0	0.1	0	0	0
Brazil	Itarumã	2.3	1.7	2.9	9	7	11	0.1	0.1	0.2	1	1	1
Brazil	Itatiaia	0.2	0.1	0.3	0	0	1	0.1	0.0	0.1	0	0	0
Brazil	Itatiaiuçu	0.3	0.2	0.4	0	0	0	0.1	0.0	0.1	0	0	0
Brazil	Itatiba	0.2	0.1	0.2	1	1	1	0.0	0.0	0.0	0	0	0
Brazil	Itatiba do Sul	0.1	0.1	0.2	0	0	0	0.0	0.0	0.0	0	0	0
Brazil	Itatim	1.7	1.4	2.2	3	2	4	0.2	0.2	0.3	0	0	1
Brazil	Itatinga	0.2	0.1	0.2	0	0	0	0.0	0.0	0.0	0	0	0
Brazil	Itatira	2.3	1.7	2.9	4	3	6	0.1	0.1	0.2	0	0	0
Brazil	Itatuba	1.0	0.7	1.2	1	1	1	0.1	0.1	0.1	0	0	0
Brazil	Itaú	1.2	0.9	1.5	1	1	1	0.1	0.1	0.2	0	0	0
Brazil	Itaú de Minas	0.3	0.2	0.4	0	0	0	0.1	0.0	0.1	0	0	0
Brazil	Itaúba	0.4	0.3	0.5	1	0	1	0.1	0.1	0.1	0	0	0
Brazil	Itaubal	0.2	0.2	0.3	0	0	0	0.1	0.1	0.2	0	0	0
Brazil	Itauçu	0.2	0.1	0.2	0	0	0	0.1	0.0	0.1	0	0	0
Brazil	Itaueira	1.4	1.1	1.8	2	1	2	0.2	0.1	0.2	0	0	0
Brazil	Itaúna	0.3	0.2	0.4	2	1	2	0.1	0.0	0.1	0	0	0
Brazil	Itaúna do Sul	0.3	0.2	0.3	0	0	0	0.1	0.0	0.1	0	0	0
Brazil	Itaverava	0.3	0.2	0.4	0	0	0	0.1	0.0	0.1	0	0	0
Brazil	Itinga	0.3	0.2	0.4	0	0	1	0.1	0.0	0.1	0	0	0
Brazil	Itinga do Maranhão	1.7	1.3	2.2	6	4	7	0.2	0.2	0.3	1	1	1
Brazil	Itiquira	0.4	0.3	0.5	0	0	1	0.1	0.1	0.1	0	0	0
Brazil	Itirapina	0.2	0.1	0.2	0	0	0	0.0	0.0	0.0	0	0	0
Brazil	Itirapuã	0.2	0.1	0.2	0	0	0	0.0	0.0	0.0	0	0	0
Brazil	Itiruçu	1.7	1.4	2.2	3	3	4	0.2	0.2	0.3	0	0	0
Brazil	Itiúba	1.7	1.4	2.2	9	7	11	0.2	0.2	0.3	1	1	1
Brazil	Itobi	0.2	0.1	0.2	0	0	0	0.0	0.0	0.0	0	0	0
Brazil	Itororó	1.7	1.4	2.2	4	3	5	0.2	0.2	0.3	0	0	1
Brazil	Itu	0.2	0.1	0.2	2	1	2	0.0	0.0	0.0	0	0	1
Brazil	Ituaçu	1.7	1.4	2.2	3	3	4	0.2	0.2	0.3	0	0	1
Brazil	Ituberá	1.7	1.4	2.2	5	4	6	0.2	0.2	0.3	1	0	1
Brazil	Itueta	0.3	0.2	0.4	0	0	0	0.1	0.0	0.1	0	0	0
Brazil	Ituiutaba	0.3	0.2	0.4	2	1	2	0.1	0.0	0.1	0	0	1
Brazil	Itumbiara	0.2	0.1	0.2	1	1	2	0.1	0.0	0.1	0	0	0
Brazil	Itumirim	0.3	0.2	0.4	0	0	0	0.1	0.0	0.1	0	0	0
Brazil	Itupeva	0.2	0.1	0.2	0	0	0	0.0	0.0	0.0	0	0	0
Brazil	Itupiranga	0.9	0.7	1.1	4	3	5	0.1	0.1	0.2	1	0	1
Brazil	Ituporanga	0.2	0.2	0.3	0	0	1	0.1	0.0	0.1	0	0	0
Brazil	Iturama	0.3	0.2	0.4	1	0	1	0.1	0.0	0.1	0	0	0
Brazil	Itutinga	0.3	0.2	0.4	0	0	0	0.1	0.0	0.1	0	0	0
Brazil	Ituverava	0.2	0.1	0.2	0	0	1	0.0	0.0	0.0	0	0	0
Brazil	Iuiú	1.7	1.4	2.2	2	2	3	0.2	0.2	0.3	0	0	0
Brazil	Iúna	0.3	0.2	0.3	1	1	1	0.1	0.1	0.1	0	0	0
Brazil	Ivaí	0.3	0.2	0.3	0	0	0	0.1	0.0	0.1	0	0	0
Brazil	Ivaiporã	0.3	0.2	0.3	1	0	1	0.1	0.0	0.1	0	0	0
Brazil	Ivaté	0.3	0.2	0.3	0	0	0	0.1	0.0	0.1	0	0	0
Brazil	Ivatuva	0.3	0.2	0.3	0	0	0	0.1	0.0	0.1	0	0	0

Table 3: Diarrhoea Mortality by Unit *(continued)*

Country	Unit	2000 mortality mean rate (per 10,000)	lower rate (per 10,000)	upper rate (per 10,000)	mean count	lower count	upper count	2017 mortality mean rate (per 10,000)	lower rate (per 10,000)	upper rate (per 10,000)	mean count	lower count	upper count
Brazil	Ivinhema	0.5	0.3	0.6	1	1	1	0.2	0.1	0.2	0	0	0
Brazil	Ivolândia	0.2	0.1	0.2	0	0	0	0.1	0.0	0.1	0	0	0
Brazil	Ivorá	0.1	0.1	0.2	0	0	0	0.0	0.0	0.0	0	0	0
Brazil	Ivoti	0.1	0.1	0.2	0	0	0	0.0	0.0	0.0	0	0	0
Brazil	Jaboatão dos Guararapes	2.0	1.6	2.6	114	90	149	0.2	0.2	0.3	12	9	15
Brazil	Jaborá	0.2	0.2	0.3	0	0	0	0.1	0.0	0.1	0	0	0
Brazil	Jaborandi	1.7	1.4	2.2	2	2	3	0.2	0.2	0.3	0	0	0
Brazil	Jaborandi	0.2	0.1	0.2	0	0	0	0.0	0.0	0.0	0	0	0
Brazil	Jaboticaba	0.1	0.1	0.2	0	0	0	0.0	0.0	0.0	0	0	0
Brazil	Jaboticatubas	0.3	0.2	0.4	0	0	0	0.1	0.0	0.1	0	0	0
Brazil	Jabuti	0.3	0.2	0.3	0	0	0	0.1	0.0	0.1	0	0	0
Brazil	Jabuticabal	0.2	0.1	0.2	1	1	1	0.0	0.0	0.0	0	0	0
Brazil	Jaçanã	1.2	0.9	1.5	1	1	1	0.1	0.1	0.2	0	0	0
Brazil	Jacaraci	1.7	1.4	2.2	2	2	3	0.2	0.2	0.3	0	0	0
Brazil	Jacaraú	1.0	0.7	1.2	2	1	2	0.1	0.1	0.1	0	0	0
Brazil	Jacaré dos Homens	2.8	2.2	3.7	3	2	4	0.3	0.2	0.4	0	0	0
Brazil	Jacareacanga	0.9	0.7	1.1	2	2	3	0.1	0.1	0.2	0	0	0
Brazil	Jacareí	0.2	0.1	0.2	3	2	3	0.0	0.0	0.0	1	0	1
Brazil	Jacarezinho	0.3	0.2	0.3	1	1	1	0.1	0.0	0.1	0	0	0
Brazil	Jaci	0.2	0.1	0.2	0	0	0	0.0	0.0	0.0	0	0	0
Brazil	Jaciara	0.4	0.3	0.5	1	1	1	0.1	0.1	0.1	0	0	0
Brazil	Jacinto	0.3	0.2	0.4	0	0	0	0.1	0.0	0.1	0	0	0
Brazil	Jacinto Machado	0.2	0.2	0.3	0	0	0	0.1	0.0	0.1	0	0	0
Brazil	Jacobina	1.7	1.4	2.2	16	12	20	0.2	0.2	0.3	2	1	2
Brazil	Jacobina do Piauí	1.4	1.1	1.8	1	1	2	0.2	0.1	0.2	0	0	0
Brazil	Jacuí	0.3	0.2	0.4	0	0	0	0.1	0.0	0.1	0	0	0
Brazil	Jacuípe	2.8	2.2	3.7	3	3	4	0.3	0.2	0.4	0	0	0
Brazil	Jacundá	0.9	0.7	1.1	5	4	6	0.1	0.1	0.2	1	1	1
Brazil	Jacupiranga	0.2	0.1	0.2	0	0	0	0.0	0.0	0.0	0	0	0
Brazil	Jacutinga	0.1	0.1	0.2	0	0	0	0.0	0.0	0.0	0	0	0
Brazil	Jacutinga	0.3	0.2	0.4	0	0	1	0.1	0.0	0.1	0	0	0
Brazil	Jaguapitã	0.3	0.2	0.3	0	0	0	0.1	0.0	0.1	0	0	0
Brazil	Jaguaquara	1.7	1.4	2.2	10	8	13	0.2	0.2	0.3	1	1	2
Brazil	Jaguaraçu	0.3	0.2	0.4	0	0	0	0.1	0.0	0.1	0	0	0
Brazil	Jaguarão	0.1	0.1	0.2	0	0	0	0.0	0.0	0.0	0	0	0
Brazil	Jaguarari	1.7	1.4	2.2	5	4	7	0.2	0.2	0.3	1	1	1
Brazil	Jaguaré	0.3	0.2	0.3	1	0	1	0.1	0.1	0.1	0	0	0
Brazil	Jaguaretama	2.3	1.7	2.9	5	4	7	0.1	0.1	0.2	0	0	0
Brazil	Jaguari	0.1	0.1	0.2	0	0	0	0.0	0.0	0.0	0	0	0
Brazil	Jaguariaíva	0.3	0.2	0.3	1	1	1	0.1	0.0	0.1	0	0	0
Brazil	Jaguaribara	2.3	1.7	2.9	2	1	2	0.1	0.1	0.2	0	0	0
Brazil	Jaguaribe	2.3	1.7	2.9	9	7	11	0.1	0.1	0.2	0	0	1
Brazil	Jaguaripe	1.7	1.4	2.2	3	2	4	0.2	0.2	0.3	0	0	1
Brazil	Jaguariúna	0.2	0.1	0.2	0	0	0	0.0	0.0	0.0	0	0	0
Brazil	Jaguaruana	2.3	1.7	2.9	8	6	10	0.1	0.1	0.2	0	0	1
Brazil	Jaguaruna	0.2	0.2	0.3	0	0	0	0.1	0.0	0.1	0	0	0
Brazil	Jaíba	0.3	0.2	0.4	1	1	1	0.1	0.0	0.1	0	0	0
Brazil	Jaicós	1.4	1.1	1.8	3	2	4	0.2	0.1	0.2	0	0	0
Brazil	Jales	0.2	0.1	0.2	1	0	1	0.0	0.0	0.0	0	0	0
Brazil	Jambeiro	0.2	0.1	0.2	0	0	0	0.0	0.0	0.0	0	0	0
Brazil	Jampruca	0.3	0.2	0.4	0	0	0	0.1	0.0	0.1	0	0	0
Brazil	Janaúba	0.3	0.2	0.4	2	1	2	0.1	0.0	0.1	0	0	0
Brazil	Jandaia	0.2	0.1	0.2	0	0	0	0.1	0.0	0.1	0	0	0
Brazil	Jandaia do Sul	0.3	0.2	0.3	0	0	1	0.1	0.0	0.1	0	0	0
Brazil	Jandaíra	1.7	1.4	2.2	3	2	3	0.2	0.2	0.3	0	0	0
Brazil	Jandaíra	1.2	0.9	1.5	1	1	1	0.1	0.1	0.2	0	0	0
Brazil	Jandira	0.2	0.1	0.2	1	1	2	0.0	0.0	0.0	0	0	0
Brazil	Janduís	1.2	0.9	1.5	1	1	1	0.1	0.1	0.2	0	0	0

Table 3: Diarrhoea Mortality by Unit *(continued)*

Country	Unit	2000 mortality mean rate (per 10,000)	lower rate (per 10,000)	upper rate (per 10,000)	mean count	lower count	upper count	2017 mortality mean rate (per 10,000)	lower rate (per 10,000)	upper rate (per 10,000)	mean count	lower count	upper count
Brazil	Jangada	0.4	0.3	0.5	0	0	0	0.1	0.1	0.1	0	0	0
Brazil	Janiópolis	0.3	0.2	0.3	0	0	0	0.1	0.0	0.1	0	0	0
Brazil	Januária	0.3	0.2	0.4	2	1	2	0.1	0.0	0.1	0	0	0
Brazil	Januário Cicco	1.2	0.9	1.5	1	1	1	0.1	0.1	0.2	0	0	0
Brazil	Japaraíba	0.3	0.2	0.4	0	0	0	0.1	0.0	0.1	0	0	0
Brazil	Japaratinga	2.8	2.2	3.7	3	2	4	0.3	0.2	0.4	0	0	0
Brazil	Japaratuba	1.3	1.0	1.7	3	2	4	0.2	0.1	0.3	0	0	1
Brazil	Japeri	0.2	0.1	0.3	1	1	2	0.1	0.0	0.1	0	0	0
Brazil	Japi	1.2	0.9	1.5	1	1	1	0.1	0.1	0.2	0	0	0
Brazil	Japira	0.3	0.2	0.3	0	0	0	0.1	0.0	0.1	0	0	0
Brazil	Japoatã	1.3	1.0	1.7	2	2	3	0.2	0.1	0.3	0	0	0
Brazil	Japonvar	0.3	0.2	0.4	0	0	0	0.1	0.0	0.1	0	0	0
Brazil	Japorã	0.5	0.3	0.6	0	0	0	0.2	0.1	0.2	0	0	0
Brazil	Japurá	0.3	0.2	0.3	0	0	0	0.1	0.0	0.1	0	0	0
Brazil	Japurá	0.8	0.6	0.9	1	1	1	0.2	0.2	0.3	0	0	0
Brazil	Jaqueira	2.0	1.6	2.6	6	4	7	0.2	0.2	0.3	0	0	0
Brazil	Jaquirana	0.1	0.1	0.2	0	0	0	0.0	0.0	0.0	0	0	0
Brazil	Jaraguá	0.2	0.1	0.2	1	0	1	0.1	0.0	0.1	0	0	0
Brazil	Jaraguá do Sul	0.2	0.2	0.3	2	2	3	0.1	0.0	0.1	1	0	1
Brazil	Jaraguari	0.5	0.3	0.6	0	0	0	0.2	0.1	0.2	0	0	0
Brazil	Jaramataia	2.8	2.2	3.7	2	2	3	0.3	0.2	0.4	0	0	0
Brazil	Jardim	0.5	0.3	0.6	1	1	1	0.2	0.1	0.2	0	0	0
Brazil	Jardim	2.3	1.7	2.9	7	5	8	0.1	0.1	0.2	0	0	0
Brazil	Jardim Alegre	0.3	0.2	0.3	0	0	0	0.1	0.0	0.1	0	0	0
Brazil	Jardim de Angicos	1.2	0.9	1.5	0	0	0	0.1	0.1	0.2	0	0	0
Brazil	Jardim do Mulato	1.4	1.1	1.8	1	0	1	0.2	0.1	0.2	0	0	0
Brazil	Jardim do Seridó	1.2	0.9	1.5	1	1	2	0.1	0.1	0.2	0	0	0
Brazil	Jardim Olinda	0.3	0.2	0.3	0	0	0	0.1	0.0	0.1	0	0	0
Brazil	Jardim-Piranhas	1.2	0.9	1.5	1	1	2	0.1	0.1	0.2	0	0	0
Brazil	Jardinópolis	0.2	0.2	0.3	0	0	0	0.1	0.0	0.1	0	0	0
Brazil	Jardinópolis	0.2	0.1	0.2	0	0	1	0.0	0.0	0.0	0	0	0
Brazil	Jari	0.1	0.1	0.2	0	0	0	0.0	0.0	0.0	0	0	0
Brazil	Jarinu	0.2	0.1	0.2	0	0	0	0.0	0.0	0.0	0	0	0
Brazil	Jaru	0.4	0.3	0.5	2	2	3	0.1	0.1	0.1	0	0	1
Brazil	Jataí	0.2	0.1	0.2	1	1	2	0.1	0.0	0.1	0	0	0
Brazil	Jataizinho	0.3	0.2	0.3	0	0	0	0.1	0.0	0.1	0	0	0
Brazil	Jataúba	2.0	1.6	2.6	4	3	6	0.2	0.2	0.3	0	0	1
Brazil	Jateí	0.5	0.3	0.6	0	0	0	0.2	0.1	0.2	0	0	0
Brazil	Jati	2.3	1.7	2.9	2	1	2	0.1	0.1	0.2	0	0	0
Brazil	Jatobá	2.0	1.6	2.6	3	2	4	0.2	0.2	0.3	0	0	0
Brazil	Jatobá	1.7	1.3	2.2	1	1	1	0.2	0.2	0.3	0	0	0
Brazil	Jatobá do Piauí	1.4	1.1	1.8	1	1	1	0.2	0.1	0.2	0	0	0
Brazil	Jaú	0.2	0.1	0.2	1	1	2	0.0	0.0	0.0	0	0	0
Brazil	Jaú do Tocantins	0.8	0.7	1.1	0	0	0	0.1	0.1	0.2	0	0	0
Brazil	Jaupaci	0.2	0.1	0.2	0	0	0	0.1	0.0	0.1	0	0	0
Brazil	Jauru	0.4	0.3	0.5	1	0	1	0.1	0.1	0.1	0	0	0
Brazil	Jeceaba	0.3	0.2	0.4	0	0	0	0.1	0.0	0.1	0	0	0
Brazil	Jenipapo de Minas	0.3	0.2	0.4	0	0	0	0.1	0.0	0.1	0	0	0
Brazil	Jenipapo dos Vieiras	1.7	1.3	2.2	2	2	3	0.2	0.2	0.3	0	0	1
Brazil	Jequeri	0.3	0.2	0.4	0	0	0	0.1	0.0	0.1	0	0	0
Brazil	Jequié	1.7	1.4	2.2	28	22	35	0.2	0.2	0.3	3	3	5
Brazil	Jequitaí	0.3	0.2	0.4	0	0	0	0.1	0.0	0.1	0	0	0
Brazil	Jequitibá	0.3	0.2	0.4	0	0	0	0.1	0.0	0.1	0	0	0
Brazil	Jequitinhonha	0.3	0.2	0.4	1	0	1	0.1	0.0	0.1	0	0	0
Brazil	Jeremoabo	1.7	1.4	2.2	4	3	5	0.2	0.2	0.3	0	0	1
Brazil	Jericó	1.0	0.7	1.2	1	0	1	0.1	0.1	0.1	0	0	0
Brazil	Jeriquara	0.2	0.1	0.2	0	0	0	0.0	0.0	0.0	0	0	0

Table 3: Diarrhoea Mortality by Unit *(continued)*

Country	Unit	2000 mortality mean rate (per 10,000)	lower rate (per 10,000)	upper rate (per 10,000)	mean count	lower count	upper count	2017 mortality mean rate (per 10,000)	lower rate (per 10,000)	upper rate (per 10,000)	mean count	lower count	upper count
Brazil	Jerônimo Monteiro	0.3	0.2	0.3	0	0	0	0.1	0.1	0.1	0	0	0
Brazil	Jerumenha	1.4	1.1	1.8	1	1	1	0.2	0.1	0.2	0	0	0
Brazil	Jesuânia	0.3	0.2	0.4	0	0	0	0.1	0.0	0.1	0	0	0
Brazil	Jesuítas	0.3	0.2	0.3	0	0	0	0.1	0.0	0.1	0	0	0
Brazil	Jesúpolis	0.2	0.1	0.2	0	0	0	0.1	0.0	0.1	0	0	0
Brazil	Ji-Paraná	0.4	0.3	0.5	5	4	6	0.1	0.1	0.1	1	1	1
Brazil	Jijoca de Jericoacoara	2.3	1.7	2.9	3	2	3	0.1	0.1	0.2	0	0	0
Brazil	Jiquiriçá	1.7	1.4	2.2	7	5	9	0.2	0.2	0.3	1	1	1
Brazil	Jitaúna	1.7	1.4	2.2	2	2	3	0.2	0.2	0.3	0	0	0
Brazil	Joaçaba	0.2	0.2	0.3	1	0	1	0.1	0.0	0.1	0	0	0
Brazil	Joaíma	0.3	0.2	0.4	0	0	1	0.1	0.0	0.1	0	0	0
Brazil	Joanésia	0.3	0.2	0.4	0	0	0	0.1	0.0	0.1	0	0	0
Brazil	Joanópolis	0.2	0.1	0.2	0	0	0	0.0	0.0	0.0	0	0	0
Brazil	João Alfredo	2.0	1.6	2.6	7	5	9	0.2	0.2	0.3	1	0	1
Brazil	João Câmara	1.2	0.9	1.5	5	4	6	0.1	0.1	0.2	0	0	1
Brazil	João Costa	1.4	1.1	1.8	1	0	1	0.2	0.1	0.2	0	0	0
Brazil	João Dias	1.2	0.9	1.5	0	0	0	0.1	0.1	0.2	0	0	0
Brazil	João Dourado	1.7	1.4	2.2	5	4	6	0.2	0.2	0.3	1	1	1
Brazil	João Lisboa	1.7	1.3	2.2	6	5	8	0.2	0.2	0.3	1	0	1
Brazil	João Monlevade	0.3	0.2	0.4	1	1	2	0.1	0.0	0.1	0	0	0
Brazil	João Neiva	0.3	0.2	0.3	0	0	0	0.1	0.1	0.1	0	0	0
Brazil	João Pessoa	1.0	0.7	1.2	53	41	67	0.1	0.1	0.1	7	5	9
Brazil	João Pinheiro	0.3	0.2	0.4	1	1	2	0.1	0.0	0.1	0	0	0
Brazil	João Ramalho	0.2	0.1	0.2	0	0	0	0.0	0.0	0.0	0	0	0
Brazil	Joaquim Felício	0.3	0.2	0.4	0	0	0	0.1	0.0	0.1	0	0	0
Brazil	Joaquim Gomes	2.8	2.2	3.7	5	4	7	0.3	0.2	0.4	0	0	1
Brazil	Joaquim Nabuco	2.0	1.6	2.6	3	2	4	0.2	0.2	0.3	0	0	0
Brazil	Joaquim Pires	1.4	1.1	1.8	3	2	4	0.2	0.1	0.2	0	0	0
Brazil	Joaquim Távora	0.3	0.2	0.3	0	0	0	0.1	0.0	0.1	0	0	0
Brazil	Joca Marques	1.4	1.1	1.8	1	1	1	0.2	0.1	0.2	0	0	0
Brazil	Jóia	0.1	0.1	0.2	0	0	0	0.0	0.0	0.0	0	0	0
Brazil	Joinvile	0.2	0.2	0.3	9	6	11	0.1	0.0	0.1	2	1	3
Brazil	Jordânia	0.3	0.2	0.4	0	0	0	0.1	0.0	0.1	0	0	0
Brazil	Jordão	1.1	0.9	1.3	1	1	1	0.2	0.2	0.3	0	0	0
Brazil	José Boiteux	0.2	0.2	0.3	0	0	0	0.1	0.0	0.1	0	0	0
Brazil	José Bonifácio	0.2	0.1	0.2	0	0	0	0.0	0.0	0.0	0	0	0
Brazil	José da Penha	1.2	0.9	1.5	1	1	1	0.1	0.1	0.2	0	0	0
Brazil	José de Freitas	1.4	1.1	1.8	5	4	7	0.2	0.1	0.2	1	0	1
Brazil	José Gonçalves de Minas	0.3	0.2	0.4	0	0	0	0.1	0.0	0.1	0	0	0
Brazil	José Raydan	0.3	0.2	0.4	0	0	0	0.1	0.0	0.1	0	0	0
Brazil	Joselândia	1.7	1.3	2.2	4	3	5	0.2	0.2	0.3	0	0	1
Brazil	Josenópolis	0.3	0.2	0.4	0	0	0	0.1	0.0	0.1	0	0	0
Brazil	Joviânia	0.2	0.1	0.2	0	0	0	0.1	0.0	0.1	0	0	0
Brazil	Juara	0.4	0.3	0.5	1	1	2	0.1	0.1	0.1	0	0	0
Brazil	Juarez Távora	1.0	0.7	1.2	1	0	1	0.1	0.1	0.1	0	0	0
Brazil	Juarina	0.8	0.7	1.1	0	0	0	0.1	0.1	0.2	0	0	0
Brazil	Juatuba	0.3	0.2	0.4	0	0	0	0.1	0.0	0.1	0	0	0
Brazil	Juazeirinho	1.0	0.7	1.2	2	1	2	0.1	0.1	0.1	0	0	0
Brazil	Juazeiro	1.7	1.4	2.2	39	31	50	0.2	0.2	0.3	6	4	8
Brazil	Juazeiro do Norte	2.3	1.7	2.9	57	43	73	0.1	0.1	0.2	4	3	5
Brazil	Juazeiro do Piauí	1.4	1.1	1.8	1	1	1	0.2	0.1	0.2	0	0	0
Brazil	Jucás	2.3	1.7	2.9	6	4	7	0.1	0.1	0.2	0	0	0
Brazil	Jucati	2.0	1.6	2.6	3	2	4	0.2	0.2	0.3	0	0	0
Brazil	Jucuruçu	1.7	1.4	2.2	3	2	4	0.2	0.2	0.3	0	0	0
Brazil	Jucurutu	1.2	0.9	1.5	2	2	3	0.1	0.1	0.2	0	0	0
Brazil	Juína	0.4	0.3	0.5	2	1	2	0.1	0.1	0.1	0	0	0
Brazil	Juiz de Fora	0.3	0.2	0.4	9	7	12	0.1	0.0	0.1	2	1	2

Table 3: Diarrhoea Mortality by Unit *(continued)*

Country	Unit	2000 mortality mean rate (per 10,000)	lower rate (per 10,000)	upper rate (per 10,000)	mean count	lower count	upper count	2017 mortality mean rate (per 10,000)	lower rate (per 10,000)	upper rate (per 10,000)	mean count	lower count	upper count
Brazil	Júlio Borges	1.4	1.1	1.8	1	1	1	0.2	0.1	0.2	0	0	0
Brazil	Júlio de Castilhos	0.1	0.1	0.2	0	0	0	0.0	0.0	0.0	0	0	0
Brazil	Júlio Mesquita	0.2	0.1	0.2	0	0	0	0.0	0.0	0.0	0	0	0
Brazil	Jumirim	0.2	0.1	0.2	0	0	0	0.0	0.0	0.0	0	0	0
Brazil	Junco	1.2	0.9	1.5	0	0	0	0.1	0.1	0.2	0	0	0
Brazil	Junco do Maranhão	1.7	1.3	2.2	1	1	2	0.2	0.2	0.3	0	0	0
Brazil	Junco do Seridó	1.0	0.7	1.2	0	0	0	0.1	0.1	0.1	0	0	0
Brazil	Jundiá	2.8	2.2	3.7	2	2	3	0.3	0.2	0.4	0	0	0
Brazil	Jundiaí	0.2	0.1	0.2	4	3	5	0.0	0.0	0.0	1	1	1
Brazil	Jundiaí do Sul	0.3	0.2	0.3	0	0	0	0.1	0.0	0.1	0	0	0
Brazil	Junqueiro	2.8	2.2	3.7	10	8	13	0.3	0.2	0.4	1	1	1
Brazil	Junqueirópolis	0.2	0.1	0.2	0	0	0	0.0	0.0	0.0	0	0	0
Brazil	Jupiá	0.2	0.2	0.3	0	0	0	0.1	0.0	0.1	0	0	0
Brazil	Jupiá	2.0	1.6	2.6	3	2	4	0.2	0.2	0.3	0	0	0
Brazil	Juquiá	0.2	0.1	0.2	0	0	0	0.0	0.0	0.0	0	0	0
Brazil	Juquitiba	0.2	0.1	0.2	0	0	1	0.0	0.0	0.0	0	0	0
Brazil	Juramento	0.3	0.2	0.4	0	0	0	0.1	0.0	0.1	0	0	0
Brazil	Juranda	0.3	0.2	0.3	0	0	0	0.1	0.0	0.1	0	0	0
Brazil	Jurema	1.4	1.1	1.8	1	0	1	0.2	0.1	0.2	0	0	0
Brazil	Jurema	2.0	1.6	2.6	4	3	5	0.2	0.2	0.3	0	0	0
Brazil	Juripiranga	1.0	0.7	1.2	1	1	1	0.1	0.1	0.1	0	0	0
Brazil	Juru	1.0	0.7	1.2	1	1	2	0.1	0.1	0.1	0	0	0
Brazil	Juruá	0.8	0.6	0.9	1	1	1	0.2	0.2	0.3	0	0	1
Brazil	Juruaia	0.3	0.2	0.4	0	0	0	0.1	0.0	0.1	0	0	0
Brazil	Juruena	0.4	0.3	0.5	0	0	0	0.1	0.1	0.1	0	0	0
Brazil	Juruti	0.9	0.7	1.1	4	3	5	0.1	0.1	0.2	1	1	1
Brazil	Juscimeira	0.4	0.3	0.5	0	0	1	0.1	0.1	0.1	0	0	0
Brazil	Jussara	0.2	0.1	0.2	0	0	0	0.1	0.0	0.1	0	0	0
Brazil	Jussara	0.3	0.2	0.3	0	0	0	0.1	0.0	0.1	0	0	0
Brazil	Jussara	1.7	1.4	2.2	3	3	4	0.2	0.2	0.3	0	0	0
Brazil	Jussari	1.7	1.4	2.2	2	1	2	0.2	0.2	0.3	0	0	0
Brazil	Jussiape	1.7	1.4	2.2	2	1	2	0.2	0.2	0.3	0	0	0
Brazil	Jutaí	0.8	0.6	0.9	2	2	3	0.2	0.2	0.3	0	0	1
Brazil	Juti	0.5	0.3	0.6	0	0	0	0.2	0.1	0.2	0	0	0
Brazil	Juvenília	0.3	0.2	0.4	0	0	0	0.1	0.0	0.1	0	0	0
Brazil	Kaloré	0.3	0.2	0.3	0	0	0	0.1	0.0	0.1	0	0	0
Brazil	Lábrea	0.8	0.6	0.9	3	2	4	0.2	0.2	0.3	1	1	1
Brazil	Lacerdópolis	0.2	0.2	0.3	0	0	0	0.1	0.0	0.1	0	0	0
Brazil	Ladainha	0.3	0.2	0.4	0	0	1	0.1	0.0	0.1	0	0	0
Brazil	Lafaiete Coutinho	1.7	1.4	2.2	1	1	1	0.2	0.2	0.3	0	0	0
Brazil	Lagamar	0.3	0.2	0.4	0	0	0	0.1	0.0	0.1	0	0	0
Brazil	Lagarto	1.3	1.0	1.7	12	9	16	0.2	0.1	0.3	2	1	2
Brazil	Lages	0.2	0.2	0.3	4	3	5	0.1	0.0	0.1	1	0	1
Brazil	Lago da Pedra	1.7	1.3	2.2	11	8	14	0.2	0.2	0.3	1	1	2
Brazil	Lago do Junco	1.7	1.3	2.2	2	2	3	0.2	0.2	0.3	0	0	0
Brazil	Lago dos Rodrigues	1.7	1.3	2.2	2	2	3	0.2	0.2	0.3	0	0	0
Brazil	Lago Verde	1.7	1.3	2.2	3	2	3	0.2	0.2	0.3	0	0	1
Brazil	Lagoa	1.0	0.7	1.2	0	0	1	0.1	0.1	0.1	0	0	0
Brazil	Lagoa Alegre	1.4	1.1	1.8	1	1	2	0.2	0.1	0.2	0	0	0
Brazil	Lagoa da Canoa	2.8	2.2	3.7	9	7	12	0.3	0.2	0.4	1	0	1
Brazil	Lagoa da Confusão	0.8	0.7	1.1	1	1	1	0.1	0.1	0.2	0	0	0
Brazil	Lagoa da Prata	0.3	0.2	0.4	1	1	1	0.1	0.0	0.1	0	0	0
Brazil	Lagoa de Anta	1.2	0.9	1.5	1	1	1	0.1	0.1	0.2	0	0	0
Brazil	Lagoa de dentro	1.0	0.7	1.2	1	1	1	0.1	0.1	0.1	0	0	0
Brazil	Lagoa de Pedras	1.2	0.9	1.5	1	1	1	0.1	0.1	0.2	0	0	0
Brazil	Lagoa de São Francisco	1.4	1.1	1.8	1	1	1	0.2	0.1	0.2	0	0	0

Table 3: Diarrhoea Mortality by Unit *(continued)*

Country	Unit	2000 mortality mean rate (per 10,000)	lower rate (per 10,000)	upper rate (per 10,000)	mean count	lower count	upper count	2017 mortality mean rate (per 10,000)	lower rate (per 10,000)	upper rate (per 10,000)	mean count	lower count	upper count
Brazil	Lagoa do Barro do Piauí	1.4	1.1	1.8	1	1	1	0.2	0.1	0.2	0	0	0
Brazil	Lagoa do Carro	2.0	1.6	2.6	4	3	5	0.2	0.2	0.3	0	0	1
Brazil	Lagoa do Itaenga	2.0	1.6	2.6	4	3	5	0.2	0.2	0.3	0	0	0
Brazil	Lagoa do Mato	1.7	1.3	2.2	2	1	2	0.2	0.2	0.3	0	0	0
Brazil	Lagoa do Ouro	2.0	1.6	2.6	3	2	4	0.2	0.2	0.3	0	0	0
Brazil	Lagoa do Piauí	1.4	1.1	1.8	1	0	1	0.2	0.1	0.2	0	0	0
Brazil	Lagoa do Sítio	1.4	1.1	1.8	1	0	1	0.2	0.1	0.2	0	0	0
Brazil	Lagoa do Tocantins	0.8	0.7	1.1	0	0	0	0.1	0.1	0.2	0	0	0
Brazil	Lagoa dos Gatos	2.0	1.6	2.6	6	5	8	0.2	0.2	0.3	0	0	1
Brazil	Lagoa dos Patos	0.3	0.2	0.4	0	0	0	0.1	0.0	0.1	0	0	0
Brazil	Lagoa dos Três Cantos	0.1	0.1	0.2	0	0	0	0.0	0.0	0.0	0	0	0
Brazil	Lagoa dourada	0.3	0.2	0.4	0	0	0	0.1	0.0	0.1	0	0	0
Brazil	Lagoa Formosa	0.3	0.2	0.4	0	0	0	0.1	0.0	0.1	0	0	0
Brazil	Lagoa Grande	0.3	0.2	0.4	1	1	1	0.1	0.0	0.1	0	0	0
Brazil	Lagoa Grande	2.0	1.6	2.6	5	4	7	0.2	0.2	0.3	1	0	1
Brazil	Lagoa Grande do Maranhão	1.7	1.3	2.2	2	2	3	0.2	0.2	0.3	0	0	0
Brazil	Lagoa Mirim	0.1	0.1	0.2	0	0	0	0.0	0.0	0.0	0	0	0
Brazil	Lagoa Nova	1.2	0.9	1.5	2	1	2	0.1	0.1	0.2	0	0	0
Brazil	Lagoa Real	1.7	1.4	2.2	2	2	3	0.2	0.2	0.3	0	0	0
Brazil	Lagoa Salgada	1.2	0.9	1.5	1	1	1	0.1	0.1	0.2	0	0	0
Brazil	Lagoa Santa	0.3	0.2	0.4	1	1	2	0.1	0.0	0.1	0	0	0
Brazil	Lagoa Seca	1.0	0.7	1.2	3	3	4	0.1	0.1	0.1	0	0	0
Brazil	Lagoa Vermelha	0.1	0.1	0.2	0	0	0	0.0	0.0	0.0	0	0	0
Brazil	Lagoão	0.1	0.1	0.2	0	0	0	0.0	0.0	0.0	0	0	0
Brazil	Lagoas de Velhos	1.2	0.9	1.5	0	0	1	0.1	0.1	0.2	0	0	0
Brazil	Lagoinha	0.2	0.1	0.2	0	0	0	0.0	0.0	0.0	0	0	0
Brazil	Lagoinha do Piauí	1.4	1.1	1.8	1	0	1	0.2	0.1	0.2	0	0	0
Brazil	Laguna	0.2	0.2	0.3	1	1	1	0.1	0.0	0.1	0	0	0
Brazil	Laguna Carapã	0.5	0.3	0.6	0	0	0	0.2	0.1	0.2	0	0	0
Brazil	Laje	1.7	1.4	2.2	4	3	5	0.2	0.2	0.3	1	0	1
Brazil	Laje do Muriaé	0.2	0.1	0.3	0	0	0	0.1	0.0	0.1	0	0	0
Brazil	Lajeado do Bugre	0.1	0.1	0.2	0	0	0	0.0	0.0	0.0	0	0	0
Brazil	Lajeado Grande	0.2	0.2	0.3	0	0	0	0.1	0.0	0.1	0	0	0
Brazil	Lajeado Novo	1.7	1.3	2.2	1	1	2	0.2	0.2	0.3	0	0	0
Brazil	Lajedao	1.7	1.4	2.2	1	1	1	0.2	0.2	0.3	0	0	0
Brazil	Lajedão	0.1	0.1	0.2	1	0	1	0.0	0.0	0.0	0	0	0
Brazil	Lajedão	0.8	0.7	1.1	0	0	0	0.1	0.1	0.2	0	0	0
Brazil	Lajedinho	1.7	1.4	2.2	1	1	1	0.2	0.2	0.3	0	0	0
Brazil	Lajedo	2.0	1.6	2.6	8	6	11	0.2	0.2	0.3	1	1	1
Brazil	Lajedo do Tabocal	1.7	1.4	2.2	1	1	2	0.2	0.2	0.3	0	0	0
Brazil	Lajes	1.2	0.9	1.5	1	1	2	0.1	0.1	0.2	0	0	0
Brazil	Lajes Pintadas	1.2	0.9	1.5	1	1	1	0.1	0.1	0.2	0	0	0
Brazil	Lajinha	0.3	0.2	0.4	1	0	1	0.1	0.0	0.1	0	0	0
Brazil	Lamarão	1.7	1.4	2.2	3	2	4	0.2	0.2	0.3	0	0	0
Brazil	Lambari	0.3	0.2	0.4	0	0	0	0.1	0.0	0.1	0	0	0
Brazil	Lambari d'Oeste	0.4	0.3	0.5	0	0	0	0.1	0.1	0.1	0	0	0
Brazil	Lamim	0.3	0.2	0.4	0	0	0	0.1	0.0	0.1	0	0	0
Brazil	Landri Sales	1.4	1.1	1.8	1	1	1	0.2	0.1	0.2	0	0	0
Brazil	Lapão	1.7	1.4	2.2	5	4	7	0.2	0.2	0.3	1	0	1
Brazil	Laranja da Terra	0.3	0.2	0.3	0	0	0	0.1	0.1	0.1	0	0	0
Brazil	Laranjal	0.3	0.2	0.3	0	0	0	0.1	0.0	0.1	0	0	0
Brazil	Laranjal	0.3	0.2	0.4	0	0	0	0.1	0.0	0.1	0	0	0
Brazil	Laranjal do Jari	0.2	0.2	0.3	0	0	1	0.1	0.1	0.2	0	0	0
Brazil	Laranjal Paulista	0.2	0.1	0.2	0	0	0	0.0	0.0	0.0	0	0	0

Table 3: Diarrhoea Mortality by Unit *(continued)*

Country	Unit	2000 mortality mean rate (per 10,000)	lower rate (per 10,000)	upper rate (per 10,000)	mean count	lower count	upper count	2017 mortality mean rate (per 10,000)	lower rate (per 10,000)	upper rate (per 10,000)	mean count	lower count	upper count
Brazil	Laranjeiras	1.3	1.0	1.7	3	3	4	0.2	0.1	0.3	0	0	1
Brazil	Laranjeiras do Sul	0.3	0.2	0.3	1	1	1	0.1	0.0	0.1	0	0	0
Brazil	Lassance	0.3	0.2	0.4	0	0	0	0.1	0.0	0.1	0	0	0
Brazil	Lastro	1.0	0.7	1.2	0	0	0	0.1	0.1	0.1	0	0	0
Brazil	Laurentino	0.2	0.2	0.3	0	0	0	0.1	0.0	0.1	0	0	0
Brazil	Lauro de Freitas	1.7	1.4	2.2	16	13	21	0.2	0.2	0.3	3	2	4
Brazil	Lauro Muller	0.2	0.2	0.3	0	0	0	0.1	0.0	0.1	0	0	0
Brazil	Lavandeira	0.8	0.7	1.1	0	0	0	0.1	0.1	0.2	0	0	0
Brazil	Lavínia	0.2	0.1	0.2	0	0	0	0.0	0.0	0.0	0	0	0
Brazil	Lavras	0.3	0.2	0.4	2	1	2	0.1	0.0	0.1	0	0	0
Brazil	Lavras da Mangabeira	2.3	1.7	2.9	8	6	10	0.1	0.1	0.2	0	0	1
Brazil	Lavras do Sul	0.1	0.1	0.2	0	0	0	0.0	0.0	0.0	0	0	0
Brazil	Lavrinhas	0.2	0.1	0.2	0	0	0	0.0	0.0	0.0	0	0	0
Brazil	Leandro Ferreira	0.3	0.2	0.4	0	0	0	0.1	0.0	0.1	0	0	0
Brazil	Lebon Régis	0.2	0.2	0.3	0	0	0	0.1	0.0	0.1	0	0	0
Brazil	Leme	0.2	0.1	0.2	1	1	1	0.0	0.0	0.0	0	0	0
Brazil	Leme do Prado	0.3	0.2	0.4	0	0	0	0.1	0.0	0.1	0	0	0
Brazil	Lençóis	1.7	1.4	2.2	2	2	3	0.2	0.2	0.3	0	0	0
Brazil	Lençóis Paulista	0.2	0.1	0.2	1	1	1	0.0	0.0	0.0	0	0	0
Brazil	Leoberto Leal	0.2	0.2	0.3	0	0	0	0.1	0.0	0.1	0	0	0
Brazil	Leopoldina	0.3	0.2	0.4	1	1	1	0.1	0.0	0.1	0	0	0
Brazil	Leopoldo de Bulhões	0.2	0.1	0.2	0	0	0	0.1	0.0	0.1	0	0	0
Brazil	Leópolis	0.3	0.2	0.3	0	0	0	0.1	0.0	0.1	0	0	0
Brazil	Liberato Salzano	0.1	0.1	0.2	0	0	0	0.0	0.0	0.0	0	0	0
Brazil	Liberdade	0.3	0.2	0.4	0	0	0	0.1	0.0	0.1	0	0	0
Brazil	Licínio de Almeida	1.7	1.4	2.2	2	2	3	0.2	0.2	0.3	0	0	0
Brazil	Lidianópolis	0.3	0.2	0.3	0	0	0	0.1	0.0	0.1	0	0	0
Brazil	Lima Campos	1.7	1.3	2.2	3	2	4	0.2	0.2	0.3	0	0	0
Brazil	Lima Duarte	0.3	0.2	0.4	0	0	0	0.1	0.0	0.1	0	0	0
Brazil	Limeira	0.2	0.1	0.2	3	2	4	0.0	0.0	0.0	1	1	1
Brazil	Limeira do Oeste	0.3	0.2	0.4	0	0	0	0.1	0.0	0.1	0	0	0
Brazil	Limoeiro	2.0	1.6	2.6	13	10	17	0.2	0.2	0.3	1	1	2
Brazil	Limoeiro de Anadia	2.8	2.2	3.7	11	8	14	0.3	0.2	0.4	1	1	1
Brazil	Limoeiro do Ajuru	0.9	0.7	1.1	2	2	3	0.1	0.1	0.2	0	0	1
Brazil	Limoeiro do Norte	2.3	1.7	2.9	11	8	14	0.1	0.1	0.2	1	1	1
Brazil	Lindoeste	0.3	0.2	0.3	0	0	0	0.1	0.0	0.1	0	0	0
Brazil	Lindóia	0.2	0.1	0.2	0	0	0	0.0	0.0	0.0	0	0	0
Brazil	Lindóia do Sul	0.2	0.2	0.3	0	0	0	0.1	0.0	0.1	0	0	0
Brazil	Lindolfo Collor	0.1	0.1	0.2	0	0	0	0.0	0.0	0.0	0	0	0
Brazil	Linha Nova	0.1	0.1	0.2	0	0	0	0.0	0.0	0.0	0	0	0
Brazil	Linhares	0.3	0.2	0.3	3	2	4	0.1	0.1	0.1	1	1	1
Brazil	Lins	0.2	0.1	0.2	1	1	1	0.0	0.0	0.0	0	0	0
Brazil	Livramento	1.0	0.7	1.2	1	0	1	0.1	0.1	0.1	0	0	0
Brazil	Livramento do Brumado	1.7	1.4	2.2	7	6	10	0.2	0.2	0.3	1	1	1
Brazil	Lizarda	0.8	0.7	1.1	0	0	1	0.1	0.1	0.2	0	0	0
Brazil	Loanda	0.3	0.2	0.3	0	0	1	0.1	0.0	0.1	0	0	0
Brazil	Lobato	0.3	0.2	0.3	0	0	0	0.1	0.0	0.1	0	0	0
Brazil	Logradouro	1.0	0.7	1.2	1	1	1	0.1	0.1	0.1	0	0	0
Brazil	Londrina	0.3	0.2	0.3	10	8	13	0.1	0.0	0.1	2	1	2
Brazil	Lontra	0.3	0.2	0.4	0	0	0	0.1	0.0	0.1	0	0	0
Brazil	Lontras	0.2	0.2	0.3	0	0	0	0.1	0.0	0.1	0	0	0
Brazil	Lorena	0.2	0.1	0.2	1	1	1	0.0	0.0	0.0	0	0	0

Table 3: Diarrhoea Mortality by Unit *(continued)*

Country	Unit	2000 mortality mean rate (per 10,000)	lower rate (per 10,000)	upper rate (per 10,000)	mean count	lower count	upper count	2017 mortality mean rate (per 10,000)	lower rate (per 10,000)	upper rate (per 10,000)	mean count	lower count	upper count
Brazil	Loreto	1.7	1.3	2.2	3	2	4	0.2	0.2	0.3	0	0	0
Brazil	Lourdes	0.2	0.1	0.2	0	0	0	0.0	0.0	0.0	0	0	0
Brazil	Louveira	0.2	0.1	0.2	0	0	0	0.0	0.0	0.0	0	0	0
Brazil	Lucas do Rio Verde	0.4	0.3	0.5	1	1	1	0.1	0.1	0.1	1	0	1
Brazil	Lucélia	0.2	0.1	0.2	0	0	0	0.0	0.0	0.0	0	0	0
Brazil	Lucena	1.0	0.7	1.2	0	0	0	0.1	0.1	0.1	0	0	0
Brazil	Lucianópolis	0.2	0.1	0.2	0	0	0	0.0	0.0	0.0	0	0	0
Brazil	Luciára	0.4	0.3	0.5	0	0	0	0.1	0.1	0.1	0	0	0
Brazil	Lucrécia	1.2	0.9	1.5	0	0	0	0.1	0.1	0.2	0	0	0
Brazil	Luís Antônio	0.2	0.1	0.2	0	0	0	0.0	0.0	0.0	0	0	0
Brazil	Luís Correia	1.4	1.1	1.8	4	3	5	0.2	0.1	0.2	0	0	1
Brazil	Luís Domingues	1.7	1.3	2.2	1	1	2	0.2	0.2	0.3	0	0	0
Brazil	Luís Gomes	1.2	0.9	1.5	1	1	1	0.1	0.1	0.2	0	0	0
Brazil	Luisburgo	0.3	0.2	0.4	0	0	0	0.1	0.0	0.1	0	0	0
Brazil	Luisiania	0.2	0.1	0.2	0	0	0	0.0	0.0	0.0	0	0	0
Brazil	Luislândia	0.3	0.2	0.4	0	0	0	0.1	0.0	0.1	0	0	0
Brazil	Luiz Alves	0.2	0.2	0.3	0	0	0	0.1	0.0	0.1	0	0	0
Brazil	Luiziânia	0.3	0.2	0.3	0	0	0	0.1	0.0	0.1	0	0	0
Brazil	Luminárias	0.3	0.2	0.4	0	0	0	0.1	0.0	0.1	0	0	0
Brazil	Lunardelli	0.3	0.2	0.3	0	0	0	0.1	0.0	0.1	0	0	0
Brazil	Lupercio	0.2	0.1	0.2	0	0	0	0.0	0.0	0.0	0	0	0
Brazil	Lupionópolis	0.3	0.2	0.3	0	0	0	0.1	0.0	0.1	0	0	0
Brazil	Lutécia	0.2	0.1	0.2	0	0	0	0.0	0.0	0.0	0	0	0
Brazil	Luz	0.3	0.2	0.4	0	0	0	0.1	0.0	0.1	0	0	0
Brazil	Luzerna	0.2	0.2	0.3	0	0	0	0.1	0.0	0.1	0	0	0
Brazil	Luziânia	0.2	0.1	0.2	3	3	4	0.1	0.0	0.1	1	1	1
Brazil	Luzilândia	1.4	1.1	1.8	4	3	5	0.2	0.1	0.2	0	0	1
Brazil	Luzinópolis	0.8	0.7	1.1	0	0	0	0.1	0.1	0.2	0	0	0
Brazil	Macaé	0.2	0.1	0.3	1	1	2	0.1	0.0	0.1	1	1	1
Brazil	Macaíba	1.2	0.9	1.5	8	6	10	0.1	0.1	0.2	1	1	1
Brazil	Macajuba	1.7	1.4	2.2	3	2	4	0.2	0.2	0.3	0	0	0
Brazil	Maçambara	0.1	0.1	0.2	0	0	0	0.0	0.0	0.0	0	0	0
Brazil	Maçambara	1.3	1.0	1.7	1	0	1	0.2	0.1	0.3	0	0	0
Brazil	Macapa	0.2	0.2	0.3	7	6	9	0.1	0.1	0.2	5	4	7
Brazil	Macaparana	2.0	1.6	2.6	6	4	7	0.2	0.2	0.3	0	0	1
Brazil	Macarani	1.7	1.4	2.2	4	3	5	0.2	0.2	0.3	1	0	1
Brazil	Macatuba	0.2	0.1	0.2	0	0	0	0.0	0.0	0.0	0	0	0
Brazil	Macau	1.2	0.9	1.5	3	2	3	0.1	0.1	0.2	0	0	0
Brazil	Macaubal	0.2	0.1	0.2	0	0	0	0.0	0.0	0.0	0	0	0
Brazil	Macaúbas	1.7	1.4	2.2	8	7	11	0.2	0.2	0.3	1	1	1
Brazil	Macedonia	0.2	0.1	0.2	0	0	0	0.0	0.0	0.0	0	0	0
Brazil	Maceió (capital)	2.8	2.2	3.7	215	166	283	0.3	0.2	0.4	26	19	34
Brazil	Machacalis	0.3	0.2	0.4	0	0	0	0.1	0.0	0.1	0	0	0
Brazil	Machadinho	0.1	0.1	0.2	0	0	0	0.0	0.0	0.0	0	0	0
Brazil	Machadinho	0.4	0.3	0.5	1	1	1	0.1	0.1	0.1	0	0	0
Brazil	Machado	0.3	0.2	0.4	1	1	1	0.1	0.0	0.1	0	0	0
Brazil	Machados	2.0	1.6	2.6	2	2	3	0.2	0.2	0.3	0	0	0
Brazil	Macieira	0.2	0.2	0.3	0	0	0	0.1	0.0	0.1	0	0	0
Brazil	Macuco	0.2	0.1	0.3	0	0	0	0.1	0.0	0.1	0	0	0
Brazil	Macururé	1.7	1.4	2.2	2	1	2	0.2	0.2	0.3	0	0	0
Brazil	Madalena	2.3	1.7	2.9	4	3	5	0.1	0.1	0.2	0	0	0
Brazil	Madeiro	1.4	1.1	1.8	1	1	1	0.2	0.1	0.2	0	0	0
Brazil	Madre de deus	1.7	1.4	2.2	0	0	0	0.2	0.2	0.3	0	0	0
Brazil	Madre de deus de Minas	0.3	0.2	0.4	0	0	0	0.1	0.0	0.1	0	0	0
Brazil	Mãe d'Água	1.0	0.7	1.2	0	0	0	0.1	0.1	0.1	0	0	0
Brazil	Maetinga	1.7	1.4	2.2	2	1	2	0.2	0.2	0.3	0	0	0
Brazil	Mafra	0.2	0.2	0.3	1	1	2	0.1	0.0	0.1	0	0	0
Brazil	Magalhães Barata	0.9	0.7	1.1	1	1	1	0.1	0.1	0.2	0	0	0

Table 3: Diarrhoea Mortality by Unit *(continued)*

Country	Unit	2000 mortality mean rate (per 10,000)	lower rate (per 10,000)	upper rate (per 10,000)	mean count	lower count	upper count	2017 mortality mean rate (per 10,000)	lower rate (per 10,000)	upper rate (per 10,000)	mean count	lower count	upper count
Brazil	Magalhães de Almeida	1.7	1.3	2.2	2	2	3	0.2	0.2	0.3	0	0	1
Brazil	Magda	0.2	0.1	0.2	0	0	0	0.0	0.0	0.0	0	0	0
Brazil	Magé	0.2	0.1	0.3	4	3	6	0.1	0.0	0.1	1	1	1
Brazil	Maiquinique	1.7	1.4	2.2	1	1	1	0.2	0.2	0.3	0	0	0
Brazil	Mairi	1.7	1.4	2.2	4	3	6	0.2	0.2	0.3	0	0	1
Brazil	Mairinque	0.2	0.1	0.2	1	0	1	0.0	0.0	0.0	0	0	0
Brazil	Mairiporã	0.2	0.1	0.2	1	1	1	0.0	0.0	0.0	0	0	0
Brazil	Mairipotaba	0.2	0.1	0.2	0	0	0	0.1	0.0	0.1	0	0	0
Brazil	Major Gercino	0.2	0.2	0.3	0	0	0	0.1	0.0	0.1	0	0	0
Brazil	Major Isidoro	2.8	2.2	3.7	6	5	8	0.3	0.2	0.4	1	0	1
Brazil	Major Sales	1.2	0.9	1.5	0	0	0	0.1	0.1	0.2	0	0	0
Brazil	Major Vieira	0.2	0.2	0.3	0	0	0	0.1	0.0	0.1	0	0	0
Brazil	Malacacheta	0.3	0.2	0.4	1	0	1	0.1	0.0	0.1	0	0	0
Brazil	Malhada	1.7	1.4	2.2	5	4	7	0.2	0.2	0.3	1	0	1
Brazil	Malhada de Pedras	1.7	1.4	2.2	1	1	2	0.2	0.2	0.3	0	0	0
Brazil	Malhada dos Bois	1.3	1.0	1.7	0	0	1	0.2	0.1	0.3	0	0	0
Brazil	Malhador	1.3	1.0	1.7	1	1	2	0.2	0.1	0.3	0	0	0
Brazil	Mallet	0.3	0.2	0.3	0	0	0	0.1	0.0	0.1	0	0	0
Brazil	Malta	1.0	0.7	1.2	1	0	1	0.1	0.1	0.1	0	0	0
Brazil	Mamanguape	1.0	0.7	1.2	4	3	5	0.1	0.1	0.1	0	0	1
Brazil	Mambaí	0.2	0.1	0.2	0	0	0	0.1	0.0	0.1	0	0	0
Brazil	Mamborê	0.3	0.2	0.3	0	0	0	0.1	0.0	0.1	0	0	0
Brazil	Mamonas	0.3	0.2	0.4	0	0	0	0.1	0.0	0.1	0	0	0
Brazil	Mampituba	0.1	0.1	0.2	0	0	0	0.0	0.0	0.0	0	0	0
Brazil	Manacapuru	0.8	0.6	0.9	7	6	9	0.2	0.2	0.3	2	2	3
Brazil	Manaíra	1.0	0.7	1.2	1	1	2	0.1	0.1	0.1	0	0	0
Brazil	Manaquiri	0.8	0.6	0.9	1	1	2	0.2	0.2	0.3	1	1	1
Brazil	Manari	2.0	1.6	2.6	4	3	5	0.2	0.2	0.3	1	0	1
Brazil	Mâncio Lima	1.1	0.9	1.3	2	1	2	0.2	0.2	0.3	0	0	1
Brazil	Mandaguaçu	0.3	0.2	0.3	0	0	0	0.1	0.0	0.1	0	0	0
Brazil	Mandaguari	0.3	0.2	0.3	1	0	1	0.1	0.0	0.1	0	0	0
Brazil	Mandirituba	0.3	0.2	0.3	1	0	1	0.1	0.0	0.1	0	0	0
Brazil	Manduri	0.2	0.1	0.2	0	0	0	0.0	0.0	0.0	0	0	0
Brazil	Manfrinópolis	0.3	0.2	0.3	0	0	0	0.1	0.0	0.1	0	0	0
Brazil	Manga	0.3	0.2	0.4	1	0	1	0.1	0.0	0.1	0	0	0
Brazil	Mangaratiba	0.2	0.1	0.2	0	0	0	0.0	0.0	0.0	0	0	0
Brazil	Mangaratiba	0.2	0.1	0.3	0	0	0	0.1	0.0	0.1	0	0	0
Brazil	Mangueirinha	0.3	0.2	0.3	1	0	1	0.1	0.0	0.1	0	0	0
Brazil	Manhuaçu	0.3	0.2	0.4	2	1	2	0.1	0.0	0.1	0	0	1
Brazil	Manhumirim	0.3	0.2	0.4	1	0	1	0.1	0.0	0.1	0	0	0
Brazil	Manicore	0.8	0.6	0.9	4	3	4	0.2	0.2	0.3	1	1	2
Brazil	Manoel Emídio	1.4	1.1	1.8	1	1	1	0.2	0.1	0.2	0	0	0
Brazil	Manoel Ribas	0.3	0.2	0.3	0	0	0	0.1	0.0	0.1	0	0	0
Brazil	Manoel Urbano	1.1	0.9	1.3	1	1	1	0.2	0.2	0.3	0	0	0
Brazil	Manoel Viana	0.1	0.1	0.2	0	0	0	0.0	0.0	0.0	0	0	0
Brazil	Manoel Vitorino	1.7	1.4	2.2	4	3	5	0.2	0.2	0.3	0	0	0
Brazil	Mansidão	1.7	1.4	2.2	3	2	3	0.2	0.2	0.3	0	0	0
Brazil	Mantena	0.3	0.2	0.4	1	1	1	0.1	0.0	0.1	0	0	0
Brazil	Mantenópolis	0.3	0.2	0.3	0	0	0	0.1	0.1	0.1	0	0	0
Brazil	Maquiné	0.1	0.1	0.2	0	0	0	0.0	0.0	0.0	0	0	0
Brazil	Mar de Espanha	0.3	0.2	0.4	0	0	0	0.1	0.0	0.1	0	0	0
Brazil	Mar Vermelho	2.8	2.2	3.7	1	1	1	0.3	0.2	0.4	0	0	0
Brazil	Mara Rosa	0.2	0.1	0.2	0	0	0	0.1	0.0	0.1	0	0	0
Brazil	Maraã	0.8	0.6	0.9	2	2	2	0.2	0.2	0.3	0	0	1
Brazil	Marabá	0.9	0.7	1.1	17	14	22	0.1	0.1	0.2	4	3	5
Brazil	Marabá Paulista	0.2	0.1	0.2	0	0	0	0.0	0.0	0.0	0	0	0
Brazil	Maracaçumé	1.7	1.3	2.2	3	3	4	0.2	0.2	0.3	1	0	1
Brazil	Maracaí	0.2	0.1	0.2	0	0	0	0.0	0.0	0.0	0	0	0

Table 3: Diarrhoea Mortality by Unit *(continued)*

Country	Unit	2000 mortality mean rate (per 10,000)	lower rate (per 10,000)	upper rate (per 10,000)	mean count	lower count	upper count	2017 mortality mean rate (per 10,000)	lower rate (per 10,000)	upper rate (per 10,000)	mean count	lower count	upper count
Brazil	Maracajá	0.2	0.2	0.3	0	0	0	0.1	0.0	0.1	0	0	0
Brazil	Maracaju	0.5	0.3	0.6	1	1	2	0.2	0.1	0.2	1	0	1
Brazil	Maracanã	0.9	0.7	1.1	3	2	4	0.1	0.1	0.2	0	0	0
Brazil	Maracanaú	2.3	1.7	2.9	41	31	52	0.1	0.1	0.2	3	2	4
Brazil	Maracás	1.7	1.4	2.2	6	5	8	0.2	0.2	0.3	0	0	1
Brazil	Maragogi	2.8	2.2	3.7	9	7	12	0.3	0.2	0.4	1	1	1
Brazil	Maragogipe	1.7	1.4	2.2	7	6	10	0.2	0.2	0.3	1	1	1
Brazil	Maraial	2.0	1.6	2.6	4	3	6	0.2	0.2	0.3	0	0	0
Brazil	Marajá do Sena	1.7	1.3	2.2	1	1	2	0.2	0.2	0.3	0	0	0
Brazil	Maranguape	2.3	1.7	2.9	21	16	27	0.1	0.1	0.2	2	1	2
Brazil	Maranhãozinho	1.7	1.3	2.2	3	2	4	0.2	0.2	0.3	1	1	1
Brazil	Marapanim	0.9	0.7	1.1	2	2	3	0.1	0.1	0.2	0	0	0
Brazil	Marapoama	0.2	0.1	0.2	0	0	0	0.0	0.0	0.0	0	0	0
Brazil	Maratá	0.1	0.1	0.2	0	0	0	0.0	0.0	0.0	0	0	0
Brazil	Marataízes	0.3	0.2	0.3	1	1	1	0.1	0.1	0.1	0	0	0
Brazil	Maraú	0.1	0.1	0.2	0	0	0	0.0	0.0	0.0	0	0	0
Brazil	Maraú	1.7	1.4	2.2	4	4	6	0.2	0.2	0.3	1	0	1
Brazil	Maravilha	0.2	0.2	0.3	0	0	0	0.1	0.0	0.1	0	0	0
Brazil	Maravilha	2.8	2.2	3.7	6	4	7	0.3	0.2	0.4	0	0	0
Brazil	Maravilhas	0.3	0.2	0.4	0	0	0	0.1	0.0	0.1	0	0	0
Brazil	Marcação	1.0	0.7	1.2	1	1	1	0.1	0.1	0.1	0	0	0
Brazil	Marcelândia	0.4	0.3	0.5	1	1	1	0.1	0.1	0.1	0	0	0
Brazil	Marcelino Vieira	1.2	0.9	1.5	1	1	1	0.1	0.1	0.2	0	0	0
Brazil	Marcionilio Dias	0.1	0.1	0.2	0	0	0	0.0	0.0	0.0	0	0	0
Brazil	Marcionílio Souza	1.7	1.4	2.2	2	2	3	0.2	0.2	0.3	0	0	0
Brazil	Marco	2.3	1.7	2.9	6	5	8	0.1	0.1	0.2	1	0	1
Brazil	Marcolândia	1.4	1.1	1.8	0	0	0	0.2	0.1	0.2	0	0	0
Brazil	Marcos Parente	1.4	1.1	1.8	1	1	1	0.2	0.1	0.2	0	0	0
Brazil	Marechal Cândido Rondon	0.3	0.2	0.3	1	1	1	0.1	0.0	0.1	0	0	0
Brazil	Marechal deodoro	2.8	2.2	3.7	13	10	17	0.3	0.2	0.4	2	1	2
Brazil	Marechal Floriano	0.3	0.2	0.3	0	0	0	0.1	0.1	0.1	0	0	0
Brazil	Marechal Thaumaturgo	1.1	0.9	1.3	1	1	2	0.2	0.2	0.3	0	0	1
Brazil	Marema	0.2	0.2	0.3	0	0	0	0.1	0.0	0.1	0	0	0
Brazil	Mari	1.0	0.7	1.2	2	2	3	0.1	0.1	0.1	0	0	0
Brazil	Maria da Fé	0.3	0.2	0.4	0	0	0	0.1	0.0	0.1	0	0	0
Brazil	Maria Helena	0.3	0.2	0.3	0	0	0	0.1	0.0	0.1	0	0	0
Brazil	Marialva	0.3	0.2	0.3	1	1	1	0.1	0.0	0.1	0	0	0
Brazil	Mariana	0.3	0.2	0.4	1	1	2	0.1	0.0	0.1	0	0	0
Brazil	Mariana Pimentel	0.1	0.1	0.2	0	0	0	0.0	0.0	0.0	0	0	0
Brazil	Mariano Moro	0.1	0.1	0.2	0	0	0	0.0	0.0	0.0	0	0	0
Brazil	Marianópolis do Tocantins	0.8	0.7	1.1	0	0	0	0.1	0.1	0.2	0	0	0
Brazil	Mariápolis	0.2	0.1	0.2	0	0	0	0.0	0.0	0.0	0	0	0
Brazil	Maribondo	2.8	2.2	3.7	6	4	7	0.3	0.2	0.4	0	0	1
Brazil	Maricá	0.2	0.1	0.3	1	1	1	0.1	0.0	0.1	0	0	1
Brazil	Marilac	0.3	0.2	0.4	0	0	0	0.1	0.0	0.1	0	0	0
Brazil	Marilândia	0.3	0.2	0.3	0	0	0	0.1	0.1	0.1	0	0	0
Brazil	Marilândia do Sul	0.3	0.2	0.3	0	0	0	0.1	0.0	0.1	0	0	0
Brazil	Marilena	0.3	0.2	0.3	0	0	0	0.1	0.0	0.1	0	0	0
Brazil	Marília	0.2	0.1	0.2	2	2	3	0.0	0.0	0.0	0	0	1
Brazil	Mariluz	0.3	0.2	0.3	0	0	0	0.1	0.0	0.1	0	0	0
Brazil	Maringá	0.3	0.2	0.3	6	5	8	0.1	0.0	0.1	1	1	2
Brazil	Marinópolis	0.2	0.1	0.2	0	0	0	0.0	0.0	0.0	0	0	0
Brazil	Mário Campos	0.3	0.2	0.4	0	0	0	0.1	0.0	0.1	0	0	0
Brazil	Marilópolis	0.3	0.2	0.3	0	0	0	0.1	0.0	0.1	0	0	0
Brazil	Maripá	0.3	0.2	0.3	0	0	0	0.1	0.0	0.1	0	0	0
Brazil	Maripá de Minas	0.3	0.2	0.4	0	0	0	0.1	0.0	0.1	0	0	0

Table 3: Diarrhoea Mortality by Unit *(continued)*

Country	Unit	2000 mortality mean rate (per 10,000)	lower rate (per 10,000)	upper rate (per 10,000)	mean count	lower count	upper count	2017 mortality mean rate (per 10,000)	lower rate (per 10,000)	upper rate (per 10,000)	mean count	lower count	upper count
Brazil	Marituba	0.9	0.7	1.1	11	8	13	0.1	0.1	0.2	2	2	3
Brazil	Marizópolis	1.0	0.7	1.2	1	0	1	0.1	0.1	0.1	0	0	0
Brazil	Marliéria	0.3	0.2	0.4	0	0	0	0.1	0.0	0.1	0	0	0
Brazil	Marmeleiro	0.3	0.2	0.3	0	0	1	0.1	0.0	0.1	0	0	0
Brazil	Marmelópolis	0.3	0.2	0.4	0	0	0	0.1	0.0	0.1	0	0	0
Brazil	Marques de Souza	0.1	0.1	0.2	0	0	0	0.0	0.0	0.0	0	0	0
Brazil	Marquinho	0.3	0.2	0.3	0	0	0	0.1	0.0	0.1	0	0	0
Brazil	Martinho Campos	0.3	0.2	0.4	0	0	0	0.1	0.0	0.1	0	0	0
Brazil	Martinópole	2.3	1.7	2.9	2	2	3	0.1	0.1	0.2	0	0	0
Brazil	Martinópolis	0.2	0.1	0.2	0	0	0	0.0	0.0	0.0	0	0	0
Brazil	Martins	1.2	0.9	1.5	1	1	1	0.1	0.1	0.2	0	0	0
Brazil	Martins Soares	0.3	0.2	0.4	0	0	0	0.1	0.0	0.1	0	0	0
Brazil	Maruim	1.3	1.0	1.7	2	1	2	0.2	0.1	0.3	0	0	0
Brazil	Marumbi	0.3	0.2	0.3	0	0	0	0.1	0.0	0.1	0	0	0
Brazil	Marzagão	0.2	0.1	0.2	0	0	0	0.1	0.0	0.1	0	0	0
Brazil	Mascote	1.7	1.4	2.2	4	3	5	0.2	0.2	0.3	0	0	0
Brazil	Massapê	2.3	1.7	2.9	9	7	12	0.1	0.1	0.2	1	0	1
Brazil	Massapê do Piauí	1.4	1.1	1.8	1	1	1	0.2	0.1	0.2	0	0	0
Brazil	Massaranduba	1.0	0.7	1.2	2	1	2	0.1	0.1	0.1	0	0	0
Brazil	Massaranduba	0.2	0.2	0.3	0	0	0	0.1	0.0	0.1	0	0	0
Brazil	Mata	0.1	0.1	0.2	0	0	0	0.0	0.0	0.0	0	0	0
Brazil	Mata de São João	1.7	1.4	2.2	12	9	15	0.2	0.2	0.3	2	2	3
Brazil	Mata Grande	2.8	2.2	3.7	13	10	17	0.3	0.2	0.4	1	1	2
Brazil	Mata Roma	1.7	1.3	2.2	2	2	3	0.2	0.2	0.3	0	0	1
Brazil	Mata Verde	0.3	0.2	0.4	0	0	0	0.1	0.0	0.1	0	0	0
Brazil	Matão	0.2	0.1	0.2	1	1	1	0.0	0.0	0.0	0	0	0
Brazil	Mataraca	1.0	0.7	1.2	0	0	1	0.1	0.1	0.1	0	0	0
Brazil	Mateira	0.2	0.1	0.2	0	0	0	0.1	0.0	0.1	0	0	0
Brazil	Mateiros	0.8	0.7	1.1	0	0	0	0.1	0.1	0.2	0	0	0
Brazil	Matelândia	0.3	0.2	0.3	0	0	0	0.1	0.0	0.1	0	0	0
Brazil	Materlândia	0.3	0.2	0.4	0	0	0	0.1	0.0	0.1	0	0	0
Brazil	Mates do Norte	1.7	1.3	2.2	2	1	2	0.2	0.2	0.3	0	0	1
Brazil	Mateus Leme	0.3	0.2	0.4	1	0	1	0.1	0.0	0.1	0	0	0
Brazil	Mathias Lobato	0.3	0.2	0.4	0	0	0	0.1	0.0	0.1	0	0	0
Brazil	Matias Barbosa	0.3	0.2	0.4	1	0	1	0.1	0.0	0.1	0	0	0
Brazil	Matias Cardoso	0.3	0.2	0.4	0	0	0	0.1	0.0	0.1	0	0	0
Brazil	Matias Olímpio	1.4	1.1	1.8	2	1	2	0.2	0.1	0.2	0	0	0
Brazil	Matina	1.7	1.4	2.2	3	2	3	0.2	0.2	0.3	0	0	0
Brazil	Matinha	1.7	1.3	2.2	5	4	6	0.2	0.2	0.3	1	0	1
Brazil	Matinhas	1.0	0.7	1.2	0	0	1	0.1	0.1	0.1	0	0	0
Brazil	Matinhos	0.3	0.2	0.3	0	0	0	0.1	0.0	0.1	0	0	0
Brazil	Matipó	0.3	0.2	0.4	0	0	1	0.1	0.0	0.1	0	0	0
Brazil	Mato Castelhano	0.1	0.1	0.2	0	0	0	0.0	0.0	0.0	0	0	0
Brazil	Mato Grosso	1.0	0.7	1.2	0	0	0	0.1	0.1	0.1	0	0	0
Brazil	Mato Grosso	0.4	0.3	0.5	1	0	1	0.1	0.1	0.1	0	0	0
Brazil	Mato Leitão	0.1	0.1	0.2	0	0	0	0.0	0.0	0.0	0	0	0
Brazil	Mato Rico	0.3	0.2	0.3	0	0	0	0.1	0.0	0.1	0	0	0
Brazil	Mato Verde	0.3	0.2	0.4	0	0	0	0.1	0.0	0.1	0	0	0
Brazil	Matões	1.7	1.3	2.2	6	4	7	0.2	0.2	0.3	1	1	1
Brazil	Matos Costa	0.2	0.2	0.3	0	0	0	0.1	0.0	0.1	0	0	0
Brazil	Matozinhos	0.3	0.2	0.4	1	1	1	0.1	0.0	0.1	0	0	0
Brazil	Matrinchã	0.2	0.1	0.2	0	0	0	0.1	0.0	0.1	0	0	0
Brazil	Matriz de Camaragibe	2.8	2.2	3.7	11	9	15	0.3	0.2	0.4	1	1	1
Brazil	Matupá	0.4	0.3	0.5	1	0	1	0.1	0.1	0.1	0	0	0
Brazil	Maturéia	1.0	0.7	1.2	1	0	1	0.1	0.1	0.1	0	0	0
Brazil	Matutina	0.3	0.2	0.4	0	0	0	0.1	0.0	0.1	0	0	0
Brazil	Mauá	0.2	0.1	0.2	5	4	7	0.0	0.0	0.0	1	1	1
Brazil	Mauá da Serra	0.3	0.2	0.3	0	0	0	0.1	0.0	0.1	0	0	0

		2000 mortality						2017 mortality					
Country	Unit	mean rate (per 10,000)	lower rate (per 10,000)	upper rate (per 10,000)	mean count	lower count	upper count	mean rate (per 10,000)	lower rate (per 10,000)	upper rate (per 10,000)	mean count	lower count	upper count
Brazil	Maués	0.8	0.6	0.9	110	89	136	0.2	0.2	0.3	39	31	49
Brazil	Maurilândia	0.2	0.1	0.2	0	0	0	0.1	0.0	0.1	0	0	0
Brazil	Maurilândia do Tocantins	0.8	0.7	1.1	0	0	0	0.1	0.1	0.2	0	0	0
Brazil	Maxaranguape	1.2	0.9	1.5	1	1	1	0.1	0.1	0.2	0	0	0
Brazil	Maximiliano de Almaeida	0.1	0.1	0.2	0	0	0	0.0	0.0	0.0	0	0	0
Brazil	Mazagão	0.2	0.2	0.3	0	0	0	0.1	0.1	0.2	0	0	0
Brazil	Me do Rio	0.9	0.7	1.1	2	2	3	0.1	0.1	0.2	0	0	0
Brazil	Medeiros	0.3	0.2	0.4	0	0	0	0.1	0.0	0.1	0	0	0
Brazil	Medeiros Neto	1.7	1.4	2.2	3	3	4	0.2	0.2	0.3	0	0	1
Brazil	Medianeira	0.3	0.2	0.3	1	1	1	0.1	0.0	0.1	0	0	0
Brazil	Medicilândia	0.9	0.7	1.1	2	2	3	0.1	0.1	0.2	0	0	1
Brazil	Medina	0.3	0.2	0.4	1	0	1	0.1	0.0	0.1	0	0	0
Brazil	Meleiro	0.2	0.2	0.3	0	0	0	0.1	0.0	0.1	0	0	0
Brazil	Melgaco	0.9	0.7	1.1	1	0	1	0.1	0.1	0.2	0	0	0
Brazil	Melgaço	0.9	0.7	1.1	2	2	3	0.1	0.1	0.2	0	0	1
Brazil	Mendes	0.2	0.1	0.3	0	0	0	0.1	0.0	0.1	0	0	0
Brazil	Mendes Pimentel	0.3	0.2	0.4	0	0	0	0.1	0.0	0.1	0	0	0
Brazil	Mendonça	0.2	0.1	0.2	0	0	0	0.0	0.0	0.0	0	0	0
Brazil	Mercedes	0.3	0.2	0.3	0	0	0	0.1	0.0	0.1	0	0	0
Brazil	Mercês	0.3	0.2	0.4	0	0	0	0.1	0.0	0.1	0	0	0
Brazil	Meridiano	0.2	0.1	0.2	0	0	0	0.0	0.0	0.0	0	0	0
Brazil	Meruoca	2.3	1.7	2.9	3	2	4	0.1	0.1	0.2	0	0	0
Brazil	Mesópolis	0.2	0.1	0.2	0	0	0	0.0	0.0	0.0	0	0	0
Brazil	Mesquita	0.3	0.2	0.4	0	0	0	0.1	0.0	0.1	0	0	0
Brazil	Messias	2.8	2.2	3.7	3	2	4	0.3	0.2	0.4	0	0	0
Brazil	Miguel Alves	1.4	1.1	1.8	7	5	9	0.2	0.1	0.2	1	1	1
Brazil	Miguel Calmon	1.7	1.4	2.2	6	5	8	0.2	0.2	0.3	1	0	1
Brazil	Miguel Leão	1.4	1.1	1.8	0	0	0	0.2	0.1	0.2	0	0	0
Brazil	Miguel Pereira	0.2	0.1	0.3	0	0	0	0.1	0.0	0.1	0	0	0
Brazil	Miguelópolis	0.2	0.1	0.2	0	0	0	0.0	0.0	0.0	0	0	0
Brazil	Milagres	2.3	1.7	2.9	6	5	8	0.1	0.1	0.2	0	0	1
Brazil	Milagres	1.7	1.4	2.2	2	1	2	0.2	0.2	0.3	0	0	0
Brazil	Milagres do Maranhão	1.7	1.3	2.2	1	1	1	0.2	0.2	0.3	0	0	0
Brazil	Milhã	2.3	1.7	2.9	3	2	4	0.1	0.1	0.2	0	0	0
Brazil	Milton Brandão	1.4	1.1	1.8	1	1	2	0.2	0.1	0.2	0	0	0
Brazil	Mimoso de Goiás	0.2	0.1	0.2	0	0	0	0.1	0.0	0.1	0	0	0
Brazil	Mimoso do Sul	0.3	0.2	0.3	1	0	1	0.1	0.1	0.1	0	0	0
Brazil	Minaçu	0.2	0.1	0.2	1	1	1	0.1	0.0	0.1	0	0	0
Brazil	Minador do Negrão	2.8	2.2	3.7	2	1	2	0.3	0.2	0.4	0	0	0
Brazil	Minas do Leão	0.1	0.1	0.2	0	0	0	0.0	0.0	0.0	0	0	0
Brazil	Minas Novas	0.3	0.2	0.4	1	1	1	0.1	0.0	0.1	0	0	0
Brazil	Minduri	0.3	0.2	0.4	0	0	0	0.1	0.0	0.1	0	0	0
Brazil	Mineiros	0.2	0.1	0.2	1	1	1	0.1	0.0	0.1	0	0	0
Brazil	Mineiros do Tietê	0.2	0.1	0.2	0	0	0	0.0	0.0	0.0	0	0	0
Brazil	Ministro Andreazza	0.4	0.3	0.5	0	0	1	0.1	0.1	0.1	0	0	0
Brazil	Mira Estrela	0.2	0.1	0.2	0	0	0	0.0	0.0	0.0	0	0	0
Brazil	Mirabela	0.3	0.2	0.4	0	0	0	0.1	0.0	0.1	0	0	0
Brazil	Miracatu	0.2	0.1	0.2	0	0	0	0.0	0.0	0.0	0	0	0
Brazil	Miracema	0.2	0.1	0.3	0	0	1	0.1	0.0	0.1	0	0	0
Brazil	Miracema do Tocantins	0.8	0.7	1.1	2	2	3	0.1	0.1	0.2	0	0	0
Brazil	Mirador	1.7	1.3	2.2	5	4	7	0.2	0.2	0.3	1	0	1
Brazil	Mirador	0.3	0.2	0.3	0	0	0	0.1	0.0	0.1	0	0	0
Brazil	Miradouro	0.3	0.2	0.4	0	0	0	0.1	0.0	0.1	0	0	0
Brazil	Miraguaí	0.1	0.1	0.2	0	0	0	0.0	0.0	0.0	0	0	0

Table 3: Diarrhoea Mortality by Unit *(continued)*

Country	Unit	2000 mortality mean rate (per 10,000)	lower rate (per 10,000)	upper rate (per 10,000)	mean count	lower count	upper count	2017 mortality mean rate (per 10,000)	lower rate (per 10,000)	upper rate (per 10,000)	mean count	lower count	upper count
Brazil	Miraí	0.3	0.2	0.4	0	0	0	0.1	0.0	0.1	0	0	0
Brazil	Miraíma	2.3	1.7	2.9	3	2	4	0.1	0.1	0.2	0	0	0
Brazil	Miranda	0.5	0.3	0.6	1	1	2	0.2	0.1	0.2	0	0	0
Brazil	Miranda do Norte	1.7	1.3	2.2	4	3	5	0.2	0.2	0.3	1	1	1
Brazil	Mirandiba	2.0	1.6	2.6	3	3	5	0.2	0.2	0.3	0	0	0
Brazil	Mirandópolis	0.2	0.1	0.2	0	0	0	0.0	0.0	0.0	0	0	0
Brazil	Mirangaba	1.7	1.4	2.2	3	3	4	0.2	0.2	0.3	0	0	1
Brazil	Miranorte	0.8	0.7	1.1	1	1	1	0.1	0.1	0.2	0	0	0
Brazil	Mirante	1.7	1.4	2.2	2	2	3	0.2	0.2	0.3	0	0	0
Brazil	Mirante da Serra	0.4	0.3	0.5	1	0	1	0.1	0.1	0.1	0	0	0
Brazil	Mirante do Paranapanema	0.2	0.1	0.2	0	0	0	0.0	0.0	0.0	0	0	0
Brazil	Miraselva	0.3	0.2	0.3	0	0	0	0.1	0.0	0.1	0	0	0
Brazil	Mirassol	0.2	0.1	0.2	1	0	1	0.0	0.0	0.0	0	0	0
Brazil	Mirassol d'Oeste	0.4	0.3	0.5	1	1	1	0.1	0.1	0.1	0	0	0
Brazil	Mirassolândia	0.2	0.1	0.2	0	0	0	0.0	0.0	0.0	0	0	0
Brazil	Miravânia	0.3	0.2	0.4	0	0	0	0.1	0.0	0.1	0	0	0
Brazil	Mirim doce	0.2	0.2	0.3	0	0	0	0.1	0.0	0.1	0	0	0
Brazil	Mirinzal	1.7	1.3	2.2	3	3	4	0.2	0.2	0.3	0	0	1
Brazil	Missal	0.3	0.2	0.3	0	0	0	0.1	0.0	0.1	0	0	0
Brazil	Misso Velha	2.3	1.7	2.9	9	7	11	0.1	0.1	0.2	1	0	1
Brazil	Mocajuba	0.9	0.7	1.1	2	2	3	0.1	0.1	0.2	0	0	1
Brazil	Mococa	0.2	0.1	0.2	1	1	1	0.0	0.0	0.0	0	0	0
Brazil	Modelo	0.2	0.2	0.3	0	0	0	0.1	0.0	0.1	0	0	0
Brazil	Moeda	0.3	0.2	0.4	0	0	0	0.1	0.0	0.1	0	0	0
Brazil	Moema	0.3	0.2	0.4	0	0	0	0.1	0.0	0.1	0	0	0
Brazil	Mogi das Cruzes	0.2	0.1	0.2	6	4	7	0.0	0.0	0.0	1	1	2
Brazil	Mogi Guaçu	0.2	0.1	0.2	1	1	2	0.0	0.0	0.0	0	0	0
Brazil	Mogi Mirim	0.2	0.1	0.2	1	1	1	0.0	0.0	0.0	0	0	0
Brazil	Moiporá	0.2	0.1	0.2	0	0	0	0.1	0.0	0.1	0	0	0
Brazil	Moita Bonita	1.3	1.0	1.7	2	1	2	0.2	0.1	0.3	0	0	0
Brazil	Moju	0.9	0.7	1.1	6	5	8	0.1	0.1	0.2	1	1	2
Brazil	Mombaça	2.3	1.7	2.9	10	8	13	0.1	0.1	0.2	1	0	1
Brazil	Mombaça	0.2	0.1	0.2	0	0	0	0.0	0.0	0.0	0	0	0
Brazil	Monção	1.7	1.3	2.2	8	6	10	0.2	0.2	0.3	1	1	2
Brazil	Monções	0.2	0.1	0.2	0	0	0	0.0	0.0	0.0	0	0	0
Brazil	Mondaí	0.2	0.2	0.3	0	0	0	0.1	0.0	0.1	0	0	0
Brazil	Mongaguá	0.2	0.1	0.2	0	0	1	0.0	0.0	0.0	0	0	0
Brazil	Mongeiro	1.0	0.7	1.2	2	1	2	0.1	0.1	0.1	0	0	0
Brazil	Monjolos	0.3	0.2	0.4	0	0	0	0.1	0.0	0.1	0	0	0
Brazil	Monsenhor Gil	1.4	1.1	1.8	2	1	2	0.2	0.1	0.2	0	0	0
Brazil	Monsenhor Hipólito	1.4	1.1	1.8	1	1	1	0.2	0.1	0.2	0	0	0
Brazil	Monsenhor Paulo	0.3	0.2	0.4	0	0	0	0.1	0.0	0.1	0	0	0
Brazil	Monsenhor Tabosa	2.3	1.7	2.9	5	3	6	0.1	0.1	0.2	0	0	0
Brazil	Montadas	1.0	0.7	1.2	0	0	0	0.1	0.1	0.1	0	0	0
Brazil	Montalvânia	0.3	0.2	0.4	0	0	0	0.1	0.0	0.1	0	0	0
Brazil	Montanha	0.3	0.2	0.3	0	0	1	0.1	0.1	0.1	0	0	0
Brazil	Montanhas	1.2	0.9	1.5	1	1	2	0.1	0.1	0.2	0	0	0
Brazil	Montauri	0.1	0.1	0.2	0	0	0	0.0	0.0	0.0	0	0	0
Brazil	Monte Alegre	0.9	0.7	1.1	7	6	9	0.1	0.1	0.2	1	1	1
Brazil	Monte Alegre	1.2	0.9	1.5	4	3	5	0.1	0.1	0.2	0	0	0
Brazil	Monte Alegre de Goiás	0.2	0.1	0.2	0	0	0	0.1	0.0	0.1	0	0	0
Brazil	Monte Alegre de Minas	0.3	0.2	0.4	0	0	0	0.1	0.0	0.1	0	0	0
Brazil	Monte Alegre de Sergipe	1.3	1.0	1.7	2	2	3	0.2	0.1	0.3	0	0	0
Brazil	Monte Alegre do Piauí	1.4	1.1	1.8	2	1	2	0.2	0.1	0.2	0	0	0

Table 3: Diarrhoea Mortality by Unit *(continued)*

Country	Unit	2000 mortality mean rate (per 10,000)	lower rate (per 10,000)	upper rate (per 10,000)	mean count	lower count	upper count	2017 mortality mean rate (per 10,000)	lower rate (per 10,000)	upper rate (per 10,000)	mean count	lower count	upper count
Brazil	Monte Alegre do Sul	0.2	0.1	0.2	0	0	0	0.0	0.0	0.0	0	0	0
Brazil	Monte Alegre dos Campos	0.1	0.1	0.2	0	0	0	0.0	0.0	0.0	0	0	0
Brazil	Monte Alto	0.2	0.1	0.2	0	0	1	0.0	0.0	0.0	0	0	0
Brazil	Monte Aprazível	0.2	0.1	0.2	0	0	0	0.0	0.0	0.0	0	0	0
Brazil	Monte Azul	0.3	0.2	0.4	1	1	1	0.1	0.0	0.1	0	0	0
Brazil	Monte Azul Paulista	0.2	0.1	0.2	0	0	0	0.0	0.0	0.0	0	0	0
Brazil	Monte Belo	0.3	0.2	0.4	0	0	0	0.1	0.0	0.1	0	0	0
Brazil	Monte Belo do Sul	0.1	0.1	0.2	0	0	0	0.0	0.0	0.0	0	0	0
Brazil	Monte Carlo	0.2	0.2	0.3	0	0	0	0.1	0.0	0.1	0	0	0
Brazil	Monte Carmelo	0.3	0.2	0.4	1	1	2	0.1	0.0	0.1	0	0	0
Brazil	Monte Castelo	0.2	0.2	0.3	0	0	0	0.1	0.0	0.1	0	0	0
Brazil	Monte Castelo	0.2	0.1	0.2	0	0	0	0.0	0.0	0.0	0	0	0
Brazil	Monte das Gameleiras	1.2	0.9	1.5	0	0	0	0.1	0.1	0.2	0	0	0
Brazil	Monte do Carmo	0.8	0.7	1.1	1	0	1	0.1	0.1	0.2	0	0	0
Brazil	Monte Formoso	0.3	0.2	0.4	0	0	0	0.1	0.0	0.1	0	0	0
Brazil	Monte Horebe	1.0	0.7	1.2	0	0	0	0.1	0.1	0.1	0	0	0
Brazil	Monte Mor	0.2	0.1	0.2	1	0	1	0.0	0.0	0.0	0	0	0
Brazil	Monte Negro	0.4	0.3	0.5	1	0	1	0.1	0.1	0.1	0	0	0
Brazil	Monte Santo	1.7	1.4	2.2	12	10	16	0.2	0.2	0.3	1	1	2
Brazil	Monte Santo de Minas	0.3	0.2	0.4	0	0	1	0.1	0.0	0.1	0	0	0
Brazil	Monte Santo do Tocantins	0.8	0.7	1.1	0	0	0	0.1	0.1	0.2	0	0	0
Brazil	Monte Sião	0.3	0.2	0.4	0	0	0	0.1	0.0	0.1	0	0	0
Brazil	Monteiro	1.0	0.7	1.2	3	2	4	0.1	0.1	0.1	0	0	0
Brazil	Monteiro Lobato	0.2	0.1	0.2	0	0	0	0.0	0.0	0.0	0	0	0
Brazil	Monteirópolis	2.8	2.2	3.7	3	2	3	0.3	0.2	0.4	0	0	0
Brazil	Montenegro	0.1	0.1	0.2	0	0	1	0.0	0.0	0.0	0	0	0
Brazil	Montes Altos	1.7	1.3	2.2	3	2	3	0.2	0.2	0.3	0	0	0
Brazil	Montes Claros	0.3	0.2	0.4	8	6	10	0.1	0.0	0.1	2	1	2
Brazil	Montes Claros de Goiás	0.2	0.1	0.2	0	0	0	0.1	0.0	0.1	0	0	0
Brazil	Montezuma	0.3	0.2	0.4	0	0	0	0.1	0.0	0.1	0	0	0
Brazil	Montividiu	0.2	0.1	0.2	0	0	0	0.1	0.0	0.1	0	0	0
Brazil	Montividiu do Norte	0.2	0.1	0.2	0	0	0	0.1	0.0	0.1	0	0	0
Brazil	Morada Nova	2.3	1.7	2.9	16	12	20	0.1	0.1	0.2	1	1	1
Brazil	Morada Nova de Minas	0.3	0.2	0.4	0	0	0	0.1	0.0	0.1	0	0	0
Brazil	Moraújo	2.3	1.7	2.9	2	2	3	0.1	0.1	0.2	0	0	0
Brazil	Moreira Sales	0.3	0.2	0.3	0	0	0	0.1	0.0	0.1	0	0	0
Brazil	Moreno	2.0	1.6	2.6	9	7	11	0.2	0.2	0.3	1	1	1
Brazil	Mormaço	0.1	0.1	0.2	0	0	0	0.0	0.0	0.0	0	0	0
Brazil	Morpará	1.7	1.4	2.2	1	1	2	0.2	0.2	0.3	0	0	0
Brazil	Morretes	0.3	0.2	0.3	0	0	1	0.1	0.0	0.1	0	0	0
Brazil	Morrinhos	2.3	1.7	2.9	6	5	8	0.1	0.1	0.2	0	0	0
Brazil	Morrinhos	0.2	0.1	0.2	1	0	1	0.1	0.0	0.1	0	0	0
Brazil	Morrinhos do Sul	0.1	0.1	0.2	0	0	0	0.0	0.0	0.0	0	0	0
Brazil	Morro Agudo	0.2	0.1	0.2	0	0	0	0.0	0.0	0.0	0	0	0
Brazil	Morro Agudo de Goiás	0.2	0.1	0.2	0	0	0	0.1	0.0	0.1	0	0	0
Brazil	Morro Cabeça No Tempo	1.4	1.1	1.8	1	1	1	0.2	0.1	0.2	0	0	0
Brazil	Morro da Fumaça	0.2	0.2	0.3	0	0	0	0.1	0.0	0.1	0	0	0
Brazil	Morro da Garça	0.3	0.2	0.4	0	0	0	0.1	0.0	0.1	0	0	0

Table 3: Diarrhoea Mortality by Unit *(continued)*

Country	Unit	2000 mortality						2017 mortality					
		mean rate (per 10,000)	lower rate (per 10,000)	upper rate (per 10,000)	mean count	lower count	upper count	mean rate (per 10,000)	lower rate (per 10,000)	upper rate (per 10,000)	mean count	lower count	upper count
Brazil	Morro do Chapéu	1.7	1.4	2.2	9	7	12	0.2	0.2	0.3	1	1	1
Brazil	Morro do Chapéu do Piauí	1.4	1.1	1.8	1	1	1	0.2	0.1	0.2	0	0	0
Brazil	Morro do Pilar	0.3	0.2	0.4	0	0	0	0.1	0.0	0.1	0	0	0
Brazil	Morro Grande	0.2	0.2	0.3	0	0	0	0.1	0.0	0.1	0	0	0
Brazil	Morro Redondo	0.1	0.1	0.2	0	0	0	0.0	0.0	0.0	0	0	0
Brazil	Morro Reuter	0.1	0.1	0.2	0	0	0	0.0	0.0	0.0	0	0	0
Brazil	Morros	1.7	1.3	2.2	5	4	7	0.2	0.2	0.3	1	1	1
Brazil	Mortugaba	1.7	1.4	2.2	2	2	3	0.2	0.2	0.3	0	0	0
Brazil	Morungaba	0.2	0.1	0.2	0	0	0	0.0	0.0	0.0	0	0	0
Brazil	Mosquito	0.8	0.7	1.1	1	0	1	0.1	0.1	0.2	0	0	0
Brazil	Mossâmedes	0.2	0.1	0.2	0	0	0	0.1	0.0	0.1	0	0	0
Brazil	Mossoró	1.2	0.9	1.5	29	22	38	0.1	0.1	0.2	4	3	6
Brazil	Mostardas	0.1	0.1	0.2	0	0	0	0.0	0.0	0.0	0	0	0
Brazil	Motuca	0.2	0.1	0.2	0	0	0	0.0	0.0	0.0	0	0	0
Brazil	Mozarlândia	0.2	0.1	0.2	0	0	0	0.1	0.0	0.1	0	0	0
Brazil	Muaná	0.9	0.7	1.1	3	2	4	0.1	0.1	0.2	1	0	1
Brazil	Mucajaí	0.7	0.6	0.9	1	1	2	0.2	0.2	0.3	0	0	1
Brazil	Mucambo	2.3	1.7	2.9	4	3	5	0.1	0.1	0.2	0	0	0
Brazil	Mucugê	1.7	1.4	2.2	3	2	4	0.2	0.2	0.3	0	0	0
Brazil	Muçum	0.1	0.1	0.2	0	0	0	0.0	0.0	0.0	0	0	0
Brazil	Mucuri	1.7	1.4	2.2	5	4	7	0.2	0.2	0.3	1	1	1
Brazil	Mucurici	0.3	0.2	0.3	0	0	0	0.1	0.1	0.1	0	0	0
Brazil	Muitos Capões	0.1	0.1	0.2	0	0	0	0.0	0.0	0.0	0	0	0
Brazil	Muliterno	0.1	0.1	0.2	0	0	0	0.0	0.0	0.0	0	0	0
Brazil	Mulungu	2.3	1.7	2.9	2	2	3	0.1	0.1	0.2	0	0	0
Brazil	Mulungu	1.0	0.7	1.2	1	1	1	0.1	0.1	0.1	0	0	0
Brazil	Mulungu do Morro	1.7	1.4	2.2	3	3	4	0.2	0.2	0.3	0	0	0
Brazil	Mundo Novo	0.2	0.1	0.2	0	0	0	0.1	0.0	0.1	0	0	0
Brazil	Mundo Novo	1.7	1.4	2.2	5	4	6	0.2	0.2	0.3	1	1	1
Brazil	Mundo Novo	0.5	0.3	0.6	1	1	1	0.2	0.1	0.2	0	0	0
Brazil	Munhoz	0.3	0.2	0.4	0	0	0	0.1	0.0	0.1	0	0	0
Brazil	Munhoz de Melo	0.3	0.2	0.3	0	0	0	0.1	0.0	0.1	0	0	0
Brazil	Muniz Ferreira	1.7	1.4	2.2	1	1	2	0.2	0.2	0.3	0	0	0
Brazil	Muniz Freire	0.3	0.2	0.3	1	0	1	0.1	0.1	0.1	0	0	0
Brazil	Muquém de São Francisco	1.7	1.4	2.2	3	2	3	0.2	0.2	0.3	0	0	0
Brazil	Muqui	0.3	0.2	0.3	0	0	0	0.1	0.1	0.1	0	0	0
Brazil	Muriaé	0.3	0.2	0.4	2	2	3	0.1	0.0	0.1	0	0	0
Brazil	Muribeca	1.3	1.0	1.7	1	1	1	0.2	0.1	0.3	0	0	0
Brazil	Murici	2.8	2.2	3.7	11	8	14	0.3	0.2	0.4	1	1	2
Brazil	Murici dos Portelas	1.4	1.1	1.8	1	1	2	0.2	0.1	0.2	0	0	0
Brazil	Muricilândia	0.8	0.7	1.1	0	0	0	0.1	0.1	0.2	0	0	0
Brazil	Muritiba	1.7	1.4	2.2	6	5	8	0.2	0.2	0.3	1	1	1
Brazil	Murutinga do Sul	0.2	0.1	0.2	0	0	0	0.0	0.0	0.0	0	0	0
Brazil	Mutuípe	1.7	1.4	2.2	4	3	5	0.2	0.2	0.3	0	0	1
Brazil	Mutum	0.3	0.2	0.4	1	1	1	0.1	0.0	0.1	0	0	0
Brazil	Mutunópolis	0.2	0.1	0.2	0	0	0	0.1	0.0	0.1	0	0	0
Brazil	Muzambinho	0.3	0.2	0.4	0	0	1	0.1	0.0	0.1	0	0	0
Brazil	Nacip Raydan	0.3	0.2	0.4	0	0	0	0.1	0.0	0.1	0	0	0
Brazil	Nantes	0.2	0.1	0.2	0	0	0	0.0	0.0	0.0	0	0	0
Brazil	Nanuque	0.3	0.2	0.4	1	1	1	0.1	0.0	0.1	0	0	0
Brazil	Naque	0.3	0.2	0.4	0	0	0	0.1	0.0	0.1	0	0	0
Brazil	Narandiba	0.2	0.1	0.2	0	0	0	0.0	0.0	0.0	0	0	0
Brazil	Natal	1.2	0.9	1.5	75	57	96	0.1	0.1	0.2	8	6	11
Brazil	Natalândia	0.3	0.2	0.4	0	0	0	0.1	0.0	0.1	0	0	0
Brazil	Natércia	0.3	0.2	0.4	0	0	0	0.1	0.0	0.1	0	0	0
Brazil	Natividade	0.2	0.1	0.3	0	0	0	0.1	0.0	0.1	0	0	0

Table 3: Diarrhoea Mortality by Unit *(continued)*

Country	Unit	2000 mortality mean rate (per 10,000)	lower rate (per 10,000)	upper rate (per 10,000)	mean count	lower count	upper count	2017 mortality mean rate (per 10,000)	lower rate (per 10,000)	upper rate (per 10,000)	mean count	lower count	upper count
Brazil	Natividade	0.8	0.7	1.1	1	1	1	0.1	0.1	0.2	0	0	0
Brazil	Natividade da Serra	0.2	0.1	0.2	0	0	0	0.0	0.0	0.0	0	0	0
Brazil	Natuba	1.0	0.7	1.2	1	1	1	0.1	0.1	0.1	0	0	0
Brazil	Navegantes	0.2	0.2	0.3	1	1	1	0.1	0.0	0.1	0	0	0
Brazil	Naviraí	0.5	0.3	0.6	2	1	2	0.2	0.1	0.2	1	0	1
Brazil	Nazaré	1.7	1.4	2.2	4	3	6	0.2	0.2	0.3	1	0	1
Brazil	Nazaré	0.8	0.7	1.1	0	0	1	0.1	0.1	0.2	0	0	0
Brazil	Nazaré da Mata	2.0	1.6	2.6	6	5	8	0.2	0.2	0.3	1	1	1
Brazil	Nazaré do Piauí	1.4	1.1	1.8	1	1	2	0.2	0.1	0.2	0	0	0
Brazil	Nazaré Paulista	0.2	0.1	0.2	0	0	0	0.0	0.0	0.0	0	0	0
Brazil	Nazareno	0.3	0.2	0.4	0	0	0	0.1	0.0	0.1	0	0	0
Brazil	Nazarezinho	1.0	0.7	1.2	1	1	1	0.1	0.1	0.1	0	0	0
Brazil	Nazário	0.2	0.1	0.2	0	0	0	0.1	0.0	0.1	0	0	0
Brazil	Neópolis	1.3	1.0	1.7	2	2	3	0.2	0.1	0.3	0	0	0
Brazil	Nepomuceno	0.3	0.2	0.4	1	0	1	0.1	0.0	0.1	0	0	0
Brazil	Nerópolis	0.2	0.1	0.2	0	0	0	0.1	0.0	0.1	0	0	0
Brazil	Neves Paulista	0.2	0.1	0.2	0	0	0	0.0	0.0	0.0	0	0	0
Brazil	Nhamundá	0.8	0.6	0.9	2	1	2	0.2	0.2	0.3	0	0	1
Brazil	Nhandeara	0.2	0.1	0.2	0	0	0	0.0	0.0	0.0	0	0	0
Brazil	Nicolau Vergueiro	0.1	0.1	0.2	0	0	0	0.0	0.0	0.0	0	0	0
Brazil	Nilo Peçanha	1.7	1.4	2.2	2	2	3	0.2	0.2	0.3	0	0	0
Brazil	Nilópolis	0.2	0.1	0.3	2	2	3	0.1	0.0	0.1	1	0	1
Brazil	Nina Rodrigues	1.7	1.3	2.2	2	2	3	0.2	0.2	0.3	0	0	1
Brazil	Ninheira	0.3	0.2	0.4	0	0	0	0.1	0.0	0.1	0	0	0
Brazil	Nioaque	0.5	0.3	0.6	1	1	1	0.2	0.1	0.2	0	0	0
Brazil	Nipoã	0.2	0.1	0.2	0	0	0	0.0	0.0	0.0	0	0	0
Brazil	Niquelândia	0.2	0.1	0.2	1	1	1	0.1	0.0	0.1	0	0	0
Brazil	Nísia Floresta	1.2	0.9	1.5	5	4	7	0.1	0.1	0.2	1	0	1
Brazil	Niterói	0.2	0.1	0.3	6	4	8	0.1	0.0	0.1	1	1	2
Brazil	Nobres	0.4	0.3	0.5	1	1	1	0.1	0.1	0.1	0	0	0
Brazil	Nonoai	0.1	0.1	0.2	0	0	0	0.0	0.0	0.0	0	0	0
Brazil	Nordestina	1.7	1.4	2.2	3	2	3	0.2	0.2	0.3	0	0	0
Brazil	Normandia	0.7	0.6	0.9	1	0	1	0.2	0.2	0.3	0	0	0
Brazil	Nortelândia	0.4	0.3	0.5	0	0	0	0.1	0.1	0.1	0	0	0
Brazil	Nossa Senhora Aprecido	1.3	1.0	1.7	1	1	1	0.2	0.1	0.3	0	0	0
Brazil	Nossa Senhora da Glória	1.3	1.0	1.7	4	3	5	0.2	0.1	0.3	1	0	1
Brazil	Nossa Senhora das Dores	1.3	1.0	1.7	3	2	4	0.2	0.1	0.3	0	0	1
Brazil	Nossa Senhora das Graças	0.3	0.2	0.3	0	0	0	0.1	0.0	0.1	0	0	0
Brazil	Nossa Senhora de Lourdes	1.3	1.0	1.7	0	0	0	0.2	0.1	0.3	0	0	0
Brazil	Nossa Senhora de Nazaré	1.4	1.1	1.8	1	0	1	0.2	0.1	0.2	0	0	0
Brazil	Nossa Senhora do Livramento	0.4	0.3	0.5	1	0	1	0.1	0.1	0.1	0	0	0
Brazil	Nossa Senhora do Socorro	1.3	1.0	1.7	20	15	26	0.2	0.1	0.3	3	2	4
Brazil	Nossa Senhora dos Remédios	1.4	1.1	1.8	1	1	2	0.2	0.1	0.2	0	0	0
Brazil	Nova Aliança	0.2	0.1	0.2	0	0	0	0.0	0.0	0.0	0	0	0
Brazil	Nova Aliança do Ivaí	0.3	0.2	0.3	0	0	0	0.1	0.0	0.1	0	0	0
Brazil	Nova Alvorada	0.1	0.1	0.2	0	0	0	0.0	0.0	0.0	0	0	0
Brazil	Nova Alvorada do Sul	0.5	0.3	0.6	1	0	1	0.2	0.1	0.2	0	0	0
Brazil	Nova América	0.2	0.1	0.2	0	0	0	0.1	0.0	0.1	0	0	0

Table 3: Diarrhoea Mortality by Unit *(continued)*

Country	Unit	2000 mortality mean rate (per 10,000)	lower rate (per 10,000)	upper rate (per 10,000)	mean count	lower count	upper count	2017 mortality mean rate (per 10,000)	lower rate (per 10,000)	upper rate (per 10,000)	mean count	lower count	upper count
Brazil	Nova América da Colina	0.3	0.2	0.3	0	0	0	0.1	0.0	0.1	0	0	0
Brazil	Nova Andradina	0.5	0.3	0.6	1	1	2	0.2	0.1	0.2	1	0	1
Brazil	Nova Araçá	0.1	0.1	0.2	0	0	0	0.0	0.0	0.0	0	0	0
Brazil	Nova Aurora	0.2	0.1	0.2	0	0	0	0.1	0.0	0.1	0	0	0
Brazil	Nova Aurora	0.3	0.2	0.3	0	0	0	0.1	0.0	0.1	0	0	0
Brazil	Nova Bandeirantes	0.4	0.3	0.5	0	0	0	0.1	0.1	0.1	0	0	0
Brazil	Nova Bassano	0.1	0.1	0.2	0	0	0	0.0	0.0	0.0	0	0	0
Brazil	Nova Belém	0.3	0.2	0.4	0	0	0	0.1	0.0	0.1	0	0	0
Brazil	Nova Boa Vista	0.1	0.1	0.2	0	0	0	0.0	0.0	0.0	0	0	0
Brazil	Nova Brasilândia	0.4	0.3	0.5	0	0	0	0.1	0.1	0.1	0	0	0
Brazil	Nova Brasilândia d'Oeste	0.4	0.3	0.5	1	1	1	0.1	0.1	0.1	0	0	0
Brazil	Nova Bréscia	0.1	0.1	0.2	0	0	0	0.0	0.0	0.0	0	0	0
Brazil	Nova Campina	0.2	0.1	0.2	0	0	0	0.0	0.0	0.0	0	0	0
Brazil	Nova Canaã	1.7	1.4	2.2	3	3	4	0.2	0.2	0.3	0	0	1
Brazil	Nova Canaã do Norte	0.4	0.3	0.5	1	0	1	0.1	0.1	0.1	0	0	0
Brazil	Nova Canaã Paulista	0.2	0.1	0.2	0	0	0	0.0	0.0	0.0	0	0	0
Brazil	Nova Candelária	0.1	0.1	0.2	0	0	0	0.0	0.0	0.0	0	0	0
Brazil	Nova Cantu	0.3	0.2	0.3	0	0	0	0.1	0.0	0.1	0	0	0
Brazil	Nova Castilho	0.2	0.1	0.2	0	0	0	0.0	0.0	0.0	0	0	0
Brazil	Nova Colinas	1.7	1.3	2.2	1	1	1	0.2	0.2	0.3	0	0	0
Brazil	Nova Crixás	0.2	0.1	0.2	0	0	0	0.1	0.0	0.1	0	0	0
Brazil	Nova Cruz	1.2	0.9	1.5	4	3	5	0.1	0.1	0.2	0	0	1
Brazil	Nova Era	0.3	0.2	0.4	0	0	1	0.1	0.0	0.1	0	0	0
Brazil	Nova Erechim	0.2	0.2	0.3	0	0	0	0.1	0.0	0.1	0	0	0
Brazil	Nova Esperança	0.3	0.2	0.3	1	0	1	0.1	0.0	0.1	0	0	0
Brazil	Nova Esperança do Piriá	0.9	0.7	1.1	2	2	3	0.1	0.1	0.2	0	0	0
Brazil	Nova Esperança do Sudoeste	0.3	0.2	0.3	0	0	0	0.1	0.0	0.1	0	0	0
Brazil	Nova Esperança do Sul	0.1	0.1	0.2	0	0	0	0.0	0.0	0.0	0	0	0
Brazil	Nova Europa	0.2	0.1	0.2	0	0	0	0.0	0.0	0.0	0	0	0
Brazil	Nova Fátima	0.3	0.2	0.3	0	0	0	0.1	0.0	0.1	0	0	0
Brazil	Nova Fátima	1.7	1.4	2.2	2	1	2	0.2	0.2	0.3	0	0	0
Brazil	Nova Floresta	1.0	0.7	1.2	1	1	2	0.1	0.1	0.1	0	0	0
Brazil	Nova Friburgo	0.2	0.1	0.3	3	2	3	0.1	0.0	0.1	1	0	1
Brazil	Nova Glória	0.2	0.1	0.2	0	0	0	0.1	0.0	0.1	0	0	0
Brazil	Nova Granada	0.2	0.1	0.2	0	0	0	0.0	0.0	0.0	0	0	0
Brazil	Nova Guarita	0.4	0.3	0.5	0	0	0	0.1	0.1	0.1	0	0	0
Brazil	Nova Guataporanga	0.2	0.1	0.2	0	0	0	0.0	0.0	0.0	0	0	0
Brazil	Nova Hartz	0.1	0.1	0.2	0	0	0	0.0	0.0	0.0	0	0	0
Brazil	Nova Ibiá	1.7	1.4	2.2	1	1	2	0.2	0.2	0.3	0	0	0
Brazil	Nova Iguaçu	0.2	0.1	0.3	16	12	22	0.1	0.0	0.1	4	3	5
Brazil	Nova Iguaçu de Goiás	0.2	0.1	0.2	0	0	0	0.1	0.0	0.1	0	0	0
Brazil	Nova Independência	0.2	0.1	0.2	0	0	0	0.0	0.0	0.0	0	0	0
Brazil	Nova Iorque	1.7	1.3	2.2	1	1	1	0.2	0.2	0.3	0	0	0
Brazil	Nova Ipixuna	0.9	0.7	1.1	3	2	3	0.1	0.1	0.2	0	0	0
Brazil	Nova Itaberaba	0.2	0.2	0.3	0	0	0	0.1	0.0	0.1	0	0	0
Brazil	Nova Itarana	1.7	1.4	2.2	1	1	1	0.2	0.2	0.3	0	0	0
Brazil	Nova Lacerda	0.4	0.3	0.5	0	0	0	0.1	0.1	0.1	0	0	0
Brazil	Nova Laranjeiras	0.3	0.2	0.3	0	0	0	0.1	0.0	0.1	0	0	0
Brazil	Nova Lima	0.3	0.2	0.4	3	2	4	0.1	0.0	0.1	1	0	1

Table 3: Diarrhoea Mortality by Unit *(continued)*

Country	Unit	2000 mortality						2017 mortality					
		mean rate (per 10,000)	lower rate (per 10,000)	upper rate (per 10,000)	mean count	lower count	upper count	mean rate (per 10,000)	lower rate (per 10,000)	upper rate (per 10,000)	mean count	lower count	upper count
Brazil	Nova Londrina	0.3	0.2	0.3	0	0	0	0.1	0.0	0.1	0	0	0
Brazil	Nova Luzitânia	0.2	0.1	0.2	0	0	0	0.0	0.0	0.0	0	0	0
Brazil	Nova Mamoré	0.4	0.3	0.5	1	0	1	0.1	0.1	0.1	0	0	0
Brazil	Nova Marilândia	0.4	0.3	0.5	0	0	0	0.1	0.1	0.1	0	0	0
Brazil	Nova Maringá	0.4	0.3	0.5	0	0	0	0.1	0.1	0.1	0	0	0
Brazil	Nova Módica	0.3	0.2	0.4	0	0	0	0.1	0.0	0.1	0	0	0
Brazil	Nova Monte Verde	0.4	0.3	0.5	0	0	0	0.1	0.1	0.1	0	0	0
Brazil	Nova Mutum	0.4	0.3	0.5	1	1	1	0.1	0.1	0.1	1	0	1
Brazil	Nova Odessa	0.2	0.1	0.2	1	1	1	0.0	0.0	0.0	0	0	0
Brazil	Nova Olímpia	0.3	0.2	0.3	0	0	0	0.1	0.0	0.1	0	0	0
Brazil	Nova Olímpia	0.4	0.3	0.5	1	1	1	0.1	0.1	0.1	0	0	0
Brazil	Nova Olinda	2.3	1.7	2.9	3	3	4	0.1	0.1	0.2	0	0	0
Brazil	Nova Olinda	1.0	0.7	1.2	1	1	1	0.1	0.1	0.1	0	0	0
Brazil	Nova Olinda	0.8	0.7	1.1	1	1	1	0.1	0.1	0.2	0	0	0
Brazil	Nova Olinda do Maranhão	1.7	1.3	2.2	4	3	5	0.2	0.2	0.3	1	1	1
Brazil	Nova Olinda do Norte	0.8	0.6	0.9	2	2	3	0.2	0.2	0.3	1	1	1
Brazil	Nova Pádua	0.1	0.1	0.2	0	0	0	0.0	0.0	0.0	0	0	0
Brazil	Nova Palma	0.1	0.1	0.2	0	0	0	0.0	0.0	0.0	0	0	0
Brazil	Nova Palmeira	1.0	0.7	1.2	0	0	1	0.1	0.1	0.1	0	0	0
Brazil	Nova Petrópolis	0.1	0.1	0.2	0	0	0	0.0	0.0	0.0	0	0	0
Brazil	Nova Ponte	0.3	0.2	0.4	0	0	0	0.1	0.0	0.1	0	0	0
Brazil	Nova Porteirinha	0.3	0.2	0.4	0	0	1	0.1	0.0	0.1	0	0	0
Brazil	Nova Prata	0.1	0.1	0.2	0	0	0	0.0	0.0	0.0	0	0	0
Brazil	Nova Prata do Iguaçu	0.3	0.2	0.3	0	0	0	0.1	0.0	0.1	0	0	0
Brazil	Nova Ramada	0.1	0.1	0.2	0	0	0	0.0	0.0	0.0	0	0	0
Brazil	Nova Redenção	1.7	1.4	2.2	2	2	3	0.2	0.2	0.3	0	0	0
Brazil	Nova Resende	0.3	0.2	0.4	0	0	0	0.1	0.0	0.1	0	0	0
Brazil	Nova Roma	0.2	0.1	0.2	0	0	0	0.1	0.0	0.1	0	0	0
Brazil	Nova Roma do Sul	0.1	0.1	0.2	0	0	0	0.0	0.0	0.0	0	0	0
Brazil	Nova Rosalândia	0.8	0.7	1.1	0	0	0	0.1	0.1	0.2	0	0	0
Brazil	Nova Russas	2.3	1.7	2.9	7	6	10	0.1	0.1	0.2	0	0	1
Brazil	Nova Santa Bárbara	0.3	0.2	0.3	0	0	0	0.1	0.0	0.1	0	0	0
Brazil	Nova Santa Rita	1.4	1.1	1.8	1	1	1	0.2	0.1	0.2	0	0	0
Brazil	Nova Santa Rita	0.1	0.1	0.2	0	0	0	0.0	0.0	0.0	0	0	0
Brazil	Nova Santa Rosa	0.3	0.2	0.3	0	0	0	0.1	0.0	0.1	0	0	0
Brazil	Nova Serrana	0.3	0.2	0.4	1	1	1	0.1	0.0	0.1	1	0	1
Brazil	Nova Soure	1.7	1.4	2.2	6	4	7	0.2	0.2	0.3	1	0	1
Brazil	Nova Tebas	0.3	0.2	0.3	0	0	0	0.1	0.0	0.1	0	0	0
Brazil	Nova Timboteua	0.9	0.7	1.1	1	1	1	0.1	0.1	0.2	0	0	0
Brazil	Nova Trento	0.2	0.2	0.3	0	0	0	0.1	0.0	0.1	0	0	0
Brazil	Nova Ubiratã	0.4	0.3	0.5	0	0	0	0.1	0.1	0.1	0	0	0
Brazil	Nova União	0.4	0.3	0.5	0	0	0	0.1	0.1	0.1	0	0	0
Brazil	Nova União	0.3	0.2	0.4	0	0	0	0.1	0.0	0.1	0	0	0
Brazil	Nova Venécia	0.3	0.2	0.3	1	1	1	0.1	0.1	0.1	0	0	0
Brazil	Nova Veneza	0.2	0.1	0.2	0	0	0	0.1	0.0	0.1	0	0	0
Brazil	Nova Veneza	0.2	0.2	0.3	0	0	0	0.1	0.0	0.1	0	0	0
Brazil	Nova Viçosa	1.7	1.4	2.2	7	5	8	0.2	0.2	0.3	1	1	1
Brazil	Nova Xavantina	0.4	0.3	0.5	1	1	1	0.1	0.1	0.1	0	0	0
Brazil	Novais	0.2	0.1	0.2	0	0	0	0.0	0.0	0.0	0	0	0
Brazil	Novo Acordo	0.8	0.7	1.1	0	0	0	0.1	0.1	0.2	0	0	0
Brazil	Novo Airão	0.8	0.6	0.9	1	1	1	0.2	0.2	0.3	0	0	1
Brazil	Novo Alegre	0.8	0.7	1.1	0	0	0	0.1	0.1	0.2	0	0	0
Brazil	Novo Aripuanã	0.8	0.6	0.9	2	1	2	0.2	0.2	0.3	1	0	1
Brazil	Novo Barreiro	0.1	0.1	0.2	0	0	0	0.0	0.0	0.0	0	0	0

Table 3: Diarrhoea Mortality by Unit *(continued)*

Country	Unit	2000 mortality mean rate (per 10,000)	lower rate (per 10,000)	upper rate (per 10,000)	mean count	lower count	upper count	2017 mortality mean rate (per 10,000)	lower rate (per 10,000)	upper rate (per 10,000)	mean count	lower count	upper count
Brazil	Novo Brazil	0.2	0.1	0.2	0	0	0	0.1	0.0	0.1	0	0	0
Brazil	Novo Cabrais	0.1	0.1	0.2	0	0	0	0.0	0.0	0.0	0	0	0
Brazil	Novo Cruzeiro	0.3	0.2	0.4	1	1	1	0.1	0.0	0.1	0	0	0
Brazil	Novo Gama	0.2	0.1	0.2	1	1	2	0.1	0.0	0.1	0	0	1
Brazil	Novo Hamburgo	0.1	0.1	0.2	2	2	3	0.0	0.0	0.0	0	0	1
Brazil	Novo Horizonte	0.2	0.2	0.3	0	0	0	0.1	0.0	0.1	0	0	0
Brazil	Novo Horizonte	1.7	1.4	2.2	1	1	2	0.2	0.2	0.3	0	0	0
Brazil	Novo Horizonte	0.2	0.1	0.2	0	0	0	0.0	0.0	0.0	0	0	0
Brazil	Novo Horizonte do Norte	0.4	0.3	0.5	0	0	0	0.1	0.1	0.1	0	0	0
Brazil	Novo Horizonte do Oeste	0.4	0.3	0.5	0	0	1	0.1	0.1	0.1	0	0	0
Brazil	Novo Horizonte do Sul	0.5	0.3	0.6	0	0	0	0.2	0.1	0.2	0	0	0
Brazil	Novo Itacolomi	0.3	0.2	0.3	0	0	0	0.1	0.0	0.1	0	0	0
Brazil	Novo Jardim	0.8	0.7	1.1	0	0	0	0.1	0.1	0.2	0	0	0
Brazil	Novo Lino	2.8	2.2	3.7	5	4	7	0.3	0.2	0.4	1	0	1
Brazil	Novo Machado	0.1	0.1	0.2	0	0	0	0.0	0.0	0.0	0	0	0
Brazil	Novo Mundo	0.4	0.3	0.5	0	0	0	0.1	0.1	0.1	0	0	0
Brazil	Novo Oriente	2.3	1.7	2.9	7	5	9	0.1	0.1	0.2	0	0	0
Brazil	Novo Oriente de Minas	0.3	0.2	0.4	0	0	0	0.1	0.0	0.1	0	0	0
Brazil	Novo Oriente do Piauí	1.4	1.1	1.8	1	1	1	0.2	0.1	0.2	0	0	0
Brazil	Novo Planalto	0.2	0.1	0.2	0	0	0	0.1	0.0	0.1	0	0	0
Brazil	Novo Progresso	0.9	0.7	1.1	2	2	3	0.1	0.1	0.2	0	0	0
Brazil	Novo Repartimento	0.9	0.7	1.1	5	4	6	0.1	0.1	0.2	1	1	2
Brazil	Novo Santo Antônio	1.4	1.1	1.8	1	0	1	0.2	0.1	0.2	0	0	0
Brazil	Novo São Joaquim	0.4	0.3	0.5	0	0	1	0.1	0.1	0.1	0	0	0
Brazil	Novo Tiradentes	0.1	0.1	0.2	0	0	0	0.0	0.0	0.0	0	0	0
Brazil	Novo Triunfo	1.7	1.4	2.2	2	2	3	0.2	0.2	0.3	0	0	0
Brazil	Novorizonte	0.3	0.2	0.4	0	0	0	0.1	0.0	0.1	0	0	0
Brazil	Nuporanga	0.2	0.1	0.2	0	0	0	0.0	0.0	0.0	0	0	0
Brazil	Óbidos	0.9	0.7	1.1	5	4	7	0.1	0.1	0.2	1	1	1
Brazil	Ocara	2.3	1.7	2.9	6	5	8	0.1	0.1	0.2	0	0	0
Brazil	Ocauçu	0.2	0.1	0.2	0	0	0	0.0	0.0	0.0	0	0	0
Brazil	Oeiras	1.4	1.1	1.8	5	4	7	0.2	0.1	0.2	1	0	1
Brazil	Oeiras do Pará	0.9	0.7	1.1	3	2	4	0.1	0.1	0.2	1	1	1
Brazil	Oiapoque	0.2	0.2	0.3	0	0	0	0.1	0.1	0.2	0	0	0
Brazil	Olaria	0.3	0.2	0.4	0	0	0	0.1	0.0	0.1	0	0	0
Brazil	Óleo	0.2	0.1	0.2	0	0	0	0.0	0.0	0.0	0	0	0
Brazil	Olho d'Água	1.0	0.7	1.2	1	1	1	0.1	0.1	0.1	0	0	0
Brazil	Olho d'Água das Cunhãs	1.7	1.3	2.2	3	3	4	0.2	0.2	0.3	1	0	1
Brazil	Olho d'Água das Flores	2.8	2.2	3.7	7	6	10	0.3	0.2	0.4	1	1	1
Brazil	Olho d'Água do Casado	2.8	2.2	3.7	3	2	4	0.3	0.2	0.4	0	0	1
Brazil	Olho d'água do Piauí	1.4	1.1	1.8	1	1	1	0.2	0.1	0.2	0	0	0
Brazil	Olho d'Água Grande	2.8	2.2	3.7	2	1	2	0.3	0.2	0.4	0	0	0
Brazil	Olho-d'Água do Borges	1.2	0.9	1.5	1	0	1	0.1	0.1	0.2	0	0	0
Brazil	Olhos-d'Água	0.3	0.2	0.4	0	0	0	0.1	0.0	0.1	0	0	0
Brazil	Olímpia	0.2	0.1	0.2	0	0	1	0.0	0.0	0.0	0	0	0
Brazil	Olímpio Noronha	0.3	0.2	0.4	0	0	0	0.1	0.0	0.1	0	0	0

Table 3: Diarrhoea Mortality by Unit *(continued)*

Country	Unit	2000 mortality mean rate (per 10,000)	lower rate (per 10,000)	upper rate (per 10,000)	mean count	lower count	upper count	2017 mortality mean rate (per 10,000)	lower rate (per 10,000)	upper rate (per 10,000)	mean count	lower count	upper count
Brazil	Olinda	2.0	1.6	2.6	75	59	98	0.2	0.2	0.3	7	5	9
Brazil	Olinda Nova do Maranhão	1.7	1.3	2.2	2	2	3	0.2	0.2	0.3	0	0	1
Brazil	Olindina	1.7	1.4	2.2	5	4	7	0.2	0.2	0.3	1	0	1
Brazil	Olivedos	1.0	0.7	1.2	0	0	0	0.1	0.1	0.1	0	0	0
Brazil	Oliveira	0.3	0.2	0.4	1	1	1	0.1	0.0	0.1	0	0	0
Brazil	Oliveira de Fátima	0.8	0.7	1.1	0	0	0	0.1	0.1	0.2	0	0	0
Brazil	Oliveira Fortes	0.3	0.2	0.4	0	0	0	0.1	0.0	0.1	0	0	0
Brazil	Olivença	2.8	2.2	3.7	4	3	6	0.3	0.2	0.4	0	0	1
Brazil	Oliveria dos Brejinhos	1.7	1.4	2.2	5	4	6	0.2	0.2	0.3	0	0	1
Brazil	Onça de Pitangui	0.3	0.2	0.4	0	0	0	0.1	0.0	0.1	0	0	0
Brazil	Onda Verde	0.2	0.1	0.2	0	0	0	0.0	0.0	0.0	0	0	0
Brazil	Oratórios	0.3	0.2	0.4	0	0	0	0.1	0.0	0.1	0	0	0
Brazil	Oriente	0.2	0.1	0.2	0	0	0	0.0	0.0	0.0	0	0	0
Brazil	Orindiúva	0.2	0.1	0.2	0	0	0	0.0	0.0	0.0	0	0	0
Brazil	Oriximiná	0.9	0.7	1.1	5	4	7	0.1	0.1	0.2	1	1	2
Brazil	Orizânia	0.3	0.2	0.4	0	0	0	0.1	0.0	0.1	0	0	0
Brazil	Orizona	0.2	0.1	0.2	0	0	0	0.1	0.0	0.1	0	0	0
Brazil	Orlandia	0.2	0.1	0.2	0	0	1	0.0	0.0	0.0	0	0	0
Brazil	Orleaes	0.2	0.2	0.3	0	0	1	0.1	0.0	0.1	0	0	0
Brazil	Orobó	2.0	1.6	2.6	9	8	12	0.2	0.2	0.3	1	1	1
Brazil	Orós	2.3	1.7	2.9	4	3	6	0.1	0.1	0.2	0	0	0
Brazil	Ortigueira	0.3	0.2	0.3	1	1	1	0.1	0.0	0.1	0	0	0
Brazil	Osasco	0.2	0.1	0.2	8	6	10	0.0	0.0	0.0	1	1	2
Brazil	Oscar Bressane	0.2	0.1	0.2	0	0	0	0.0	0.0	0.0	0	0	0
Brazil	Osório	0.1	0.1	0.2	0	0	1	0.0	0.0	0.0	0	0	0
Brazil	Osvaldo Cruz	0.2	0.1	0.2	0	0	0	0.0	0.0	0.0	0	0	0
Brazil	Otacílio Costa	0.2	0.2	0.3	0	0	0	0.1	0.0	0.1	0	0	0
Brazil	Ourém	0.9	0.7	1.1	1	1	1	0.1	0.1	0.2	0	0	0
Brazil	Ouriçangas	1.7	1.4	2.2	1	1	1	0.2	0.2	0.3	0	0	0
Brazil	Ouricuri	2.0	1.6	2.6	18	14	24	0.2	0.2	0.3	2	1	2
Brazil	Ourilândia do Norte	0.9	0.7	1.1	2	1	2	0.1	0.1	0.2	0	0	1
Brazil	Ourinhos	0.2	0.1	0.2	1	1	2	0.0	0.0	0.0	0	0	0
Brazil	Ourizona	0.3	0.2	0.3	0	0	0	0.1	0.0	0.1	0	0	0
Brazil	Ouro	0.2	0.2	0.3	0	0	0	0.1	0.0	0.1	0	0	0
Brazil	Ouro Branco	0.3	0.2	0.4	1	1	1	0.1	0.0	0.1	0	0	0
Brazil	Ouro Branco	2.8	2.2	3.7	4	3	5	0.3	0.2	0.4	0	0	1
Brazil	Ouro Branco	1.2	0.9	1.5	1	0	1	0.1	0.1	0.2	0	0	0
Brazil	Ouro Fino	0.3	0.2	0.4	1	1	1	0.1	0.0	0.1	0	0	0
Brazil	Ouro Preto	0.3	0.2	0.4	1	1	2	0.1	0.0	0.1	0	0	0
Brazil	Ouro Preto do Oeste	0.4	0.3	0.5	2	1	2	0.1	0.1	0.1	0	0	0
Brazil	Ouro Velho	1.0	0.7	1.2	0	0	0	0.1	0.1	0.1	0	0	0
Brazil	Ouro Verde	0.2	0.2	0.3	0	0	0	0.1	0.0	0.1	0	0	0
Brazil	Ouro Verde	0.2	0.1	0.2	0	0	0	0.0	0.0	0.0	0	0	0
Brazil	Ouro Verde de Goiás	0.2	0.1	0.2	0	0	0	0.1	0.0	0.1	0	0	0
Brazil	Ouro Verde de Minas	0.3	0.2	0.4	0	0	0	0.1	0.0	0.1	0	0	0
Brazil	Ouro Verde do Oeste	0.3	0.2	0.3	0	0	0	0.1	0.0	0.1	0	0	0
Brazil	Ouroeste	0.2	0.1	0.2	0	0	0	0.0	0.0	0.0	0	0	0
Brazil	Ourolândia	1.7	1.4	2.2	3	2	4	0.2	0.2	0.3	0	0	1
Brazil	Ouvidor	0.2	0.1	0.2	0	0	0	0.1	0.0	0.1	0	0	0
Brazil	Pacaembu	0.2	0.1	0.2	0	0	0	0.0	0.0	0.0	0	0	0
Brazil	Pacajá	0.9	0.7	1.1	3	3	4	0.1	0.1	0.2	1	1	1
Brazil	Pacajús	2.3	1.7	2.9	13	10	17	0.1	0.1	0.2	1	1	2

Table 3: Diarrhoea Mortality by Unit *(continued)*

Country	Unit	2000 mortality mean rate (per 10,000)	lower rate (per 10,000)	upper rate (per 10,000)	mean count	lower count	upper count	2017 mortality mean rate (per 10,000)	lower rate (per 10,000)	upper rate (per 10,000)	mean count	lower count	upper count
Brazil	Pacaraima	0.7	0.6	0.9	1	0	1	0.2	0.2	0.3	0	0	0
Brazil	Pacatuba	2.3	1.7	2.9	29	22	37	0.1	0.1	0.2	2	2	3
Brazil	Pacatuba	1.3	1.0	1.7	2	1	2	0.2	0.1	0.3	0	0	0
Brazil	Paço do Lumiar	1.7	1.3	2.2	19	15	25	0.2	0.2	0.3	3	2	4
Brazil	Pacoti	2.3	1.7	2.9	3	2	4	0.1	0.1	0.2	0	0	0
Brazil	Pacujá	2.3	1.7	2.9	2	1	2	0.1	0.1	0.2	0	0	0
Brazil	Padre Bernardo	0.2	0.1	0.2	0	0	1	0.1	0.0	0.1	0	0	0
Brazil	Padre Carvalho	0.3	0.2	0.4	0	0	0	0.1	0.0	0.1	0	0	0
Brazil	Padre Marcos	1.4	1.1	1.8	1	1	2	0.2	0.1	0.2	0	0	0
Brazil	Padre Paraíso	0.3	0.2	0.4	1	0	1	0.1	0.0	0.1	0	0	0
Brazil	Paes Landim	1.4	1.1	1.8	1	1	1	0.2	0.1	0.2	0	0	0
Brazil	Pai Pedro	0.3	0.2	0.4	0	0	0	0.1	0.0	0.1	0	0	0
Brazil	Paial	0.2	0.2	0.3	0	0	0	0.1	0.0	0.1	0	0	0
Brazil	Paiçandu	0.3	0.2	0.3	0	0	0	0.1	0.0	0.1	0	0	0
Brazil	Paim Filho	0.1	0.1	0.2	0	0	0	0.0	0.0	0.0	0	0	0
Brazil	Paineiras	0.3	0.2	0.4	0	0	0	0.1	0.0	0.1	0	0	0
Brazil	Painel	0.2	0.2	0.3	0	0	0	0.1	0.0	0.1	0	0	0
Brazil	Pains	0.3	0.2	0.4	0	0	0	0.1	0.0	0.1	0	0	0
Brazil	Paiva	0.3	0.2	0.4	0	0	0	0.1	0.0	0.1	0	0	0
Brazil	Pajeú do Piauí	1.4	1.1	1.8	0	0	1	0.2	0.1	0.2	0	0	0
Brazil	Palestina	0.2	0.1	0.2	0	0	0	0.0	0.0	0.0	0	0	0
Brazil	Palestina	2.8	2.2	3.7	2	2	3	0.3	0.2	0.4	0	0	0
Brazil	Palestina de Goiás	0.2	0.1	0.2	0	0	0	0.1	0.0	0.1	0	0	0
Brazil	Palestina do Pará	0.9	0.7	1.1	1	1	1	0.1	0.1	0.2	0	0	0
Brazil	Palhano	2.3	1.7	2.9	2	1	2	0.1	0.1	0.2	0	0	0
Brazil	Palhoça	0.2	0.2	0.3	2	1	2	0.1	0.0	0.1	0	0	1
Brazil	Palma	0.3	0.2	0.4	0	0	0	0.1	0.0	0.1	0	0	0
Brazil	Palma Sola	0.2	0.2	0.3	0	0	0	0.1	0.0	0.1	0	0	0
Brazil	Palmácia	2.3	1.7	2.9	3	2	3	0.1	0.1	0.2	0	0	0
Brazil	Palmares	2.0	1.6	2.6	13	10	17	0.2	0.2	0.3	1	1	1
Brazil	Palmares do Sul	0.1	0.1	0.2	0	0	0	0.0	0.0	0.0	0	0	0
Brazil	Palmares Paulista	0.2	0.1	0.2	0	0	0	0.0	0.0	0.0	0	0	0
Brazil	Palmas	0.3	0.2	0.3	1	1	1	0.1	0.0	0.1	0	0	0
Brazil	Palmas	0.8	0.7	1.1	14	11	18	0.1	0.1	0.2	3	3	5
Brazil	Palmas de Monte Alto	1.7	1.4	2.2	4	3	5	0.2	0.2	0.3	1	0	1
Brazil	Palmeira	0.2	0.2	0.3	0	0	0	0.1	0.0	0.1	0	0	0
Brazil	Palmeira	0.3	0.2	0.3	1	1	1	0.1	0.0	0.1	0	0	0
Brazil	Palmeira das Missões	0.1	0.1	0.2	0	0	1	0.0	0.0	0.0	0	0	0
Brazil	Palmeira do Oeste	0.2	0.1	0.2	0	0	0	0.0	0.0	0.0	0	0	0
Brazil	Palmeira do Piauí	1.4	1.1	1.8	1	1	1	0.2	0.1	0.2	0	0	0
Brazil	Palmeira dos índios	2.8	2.2	3.7	23	18	30	0.3	0.2	0.4	2	2	3
Brazil	Palmeirais	1.4	1.1	1.8	2	2	3	0.2	0.1	0.2	0	0	0
Brazil	Palmeirândia	1.7	1.3	2.2	4	3	5	0.2	0.2	0.3	1	0	1
Brazil	Palmeirante	0.8	0.7	1.1	1	1	1	0.1	0.1	0.2	0	0	0
Brazil	Palmeiras	1.7	1.4	2.2	2	1	2	0.2	0.2	0.3	0	0	0
Brazil	Palmeiras de Goiás	0.2	0.1	0.2	0	0	0	0.1	0.0	0.1	0	0	0
Brazil	Palmeirina	2.0	1.6	2.6	2	2	3	0.2	0.2	0.3	0	0	0
Brazil	Palmeirópolis	0.8	0.7	1.1	1	0	1	0.1	0.1	0.2	0	0	0
Brazil	Palmelo	0.2	0.1	0.2	0	0	0	0.1	0.0	0.1	0	0	0
Brazil	Palminópolis	0.2	0.1	0.2	0	0	0	0.1	0.0	0.1	0	0	0
Brazil	Palmital	0.3	0.2	0.3	0	0	1	0.1	0.0	0.1	0	0	0
Brazil	Palmital	0.2	0.1	0.2	0	0	0	0.0	0.0	0.0	0	0	0
Brazil	Palmitinhos	0.1	0.1	0.2	0	0	0	0.0	0.0	0.0	0	0	0
Brazil	Palmitos	0.2	0.2	0.3	0	0	0	0.1	0.0	0.1	0	0	0

Table 3: Diarrhoea Mortality by Unit *(continued)*

Country	Unit	2000 mortality mean rate (per 10,000)	lower rate (per 10,000)	upper rate (per 10,000)	mean count	lower count	upper count	2017 mortality mean rate (per 10,000)	lower rate (per 10,000)	upper rate (per 10,000)	mean count	lower count	upper count
Brazil	Palmópolis	0.3	0.2	0.4	0	0	0	0.1	0.0	0.1	0	0	0
Brazil	Palotina	0.3	0.2	0.3	1	0	1	0.1	0.0	0.1	0	0	0
Brazil	Panamá	0.2	0.1	0.2	0	0	0	0.1	0.0	0.1	0	0	0
Brazil	Panambi	0.1	0.1	0.2	0	0	0	0.0	0.0	0.0	0	0	0
Brazil	Pancas	0.3	0.2	0.3	1	0	1	0.1	0.1	0.1	0	0	0
Brazil	Panelas	2.0	1.6	2.6	7	6	10	0.2	0.2	0.3	1	0	1
Brazil	Panorama	0.2	0.1	0.2	0	0	0	0.0	0.0	0.0	0	0	0
Brazil	Pantano Grande	0.1	0.1	0.2	0	0	0	0.0	0.0	0.0	0	0	0
Brazil	Pão de Açúcar	2.8	2.2	3.7	8	6	11	0.3	0.2	0.4	1	0	1
Brazil	Papagaios	0.3	0.2	0.4	0	0	0	0.1	0.0	0.1	0	0	0
Brazil	Papanduva	0.2	0.2	0.3	0	0	1	0.1	0.0	0.1	0	0	0
Brazil	Paquetá	1.4	1.1	1.8	1	0	1	0.2	0.1	0.2	0	0	0
Brazil	Pará de Minas	0.3	0.2	0.4	2	1	2	0.1	0.0	0.1	0	0	0
Brazil	Paracambi	0.2	0.1	0.3	1	0	1	0.1	0.0	0.1	0	0	0
Brazil	Paracatu	0.3	0.2	0.4	2	2	3	0.1	0.0	0.1	0	0	1
Brazil	Paracuru	2.3	1.7	2.9	7	5	9	0.1	0.1	0.2	0	0	1
Brazil	Paragominas	0.9	0.7	1.1	9	7	11	0.1	0.1	0.2	2	1	2
Brazil	Paraguaçu	0.3	0.2	0.4	0	0	1	0.1	0.0	0.1	0	0	0
Brazil	Paraguaçu Paulista	0.2	0.1	0.2	1	0	1	0.0	0.0	0.0	0	0	0
Brazil	Paraí	0.1	0.1	0.2	0	0	0	0.0	0.0	0.0	0	0	0
Brazil	Paraíba do Sul	0.2	0.1	0.3	1	0	1	0.1	0.0	0.1	0	0	0
Brazil	Paraibano	1.7	1.3	2.2	4	3	6	0.2	0.2	0.3	1	0	1
Brazil	Paraibuna	0.2	0.1	0.2	0	0	0	0.0	0.0	0.0	0	0	0
Brazil	Paraipaba	2.3	1.7	2.9	7	6	9	0.1	0.1	0.2	0	0	1
Brazil	Paraíso	0.2	0.2	0.3	0	0	0	0.1	0.0	0.1	0	0	0
Brazil	Paraíso	0.2	0.1	0.2	0	0	0	0.0	0.0	0.0	0	0	0
Brazil	Paraíso do Norte	0.3	0.2	0.3	0	0	0	0.1	0.0	0.1	0	0	0
Brazil	Paraíso do Sul	0.1	0.1	0.2	0	0	0	0.0	0.0	0.0	0	0	0
Brazil	Paraíso do Tocantins	0.8	0.7	1.1	3	3	4	0.1	0.1	0.2	1	0	1
Brazil	Paraisópolis	0.3	0.2	0.4	0	0	1	0.1	0.0	0.1	0	0	0
Brazil	Parambu	2.3	1.7	2.9	9	7	12	0.1	0.1	0.2	0	0	1
Brazil	Paramirim	1.7	1.4	2.2	3	2	4	0.2	0.2	0.3	0	0	1
Brazil	Paramoti	2.3	1.7	2.9	3	2	4	0.1	0.1	0.2	0	0	0
Brazil	Paraná	0.8	0.7	1.1	1	1	2	0.1	0.1	0.2	0	0	0
Brazil	Paraná	1.2	0.9	1.5	0	0	0	0.1	0.1	0.2	0	0	0
Brazil	Paranacity	0.3	0.2	0.3	0	0	0	0.1	0.0	0.1	0	0	0
Brazil	Paranaguá	0.3	0.2	0.3	4	3	5	0.1	0.0	0.1	1	1	1
Brazil	Paranaíba	0.5	0.3	0.6	2	1	2	0.2	0.1	0.2	0	0	0
Brazil	Paranaíta	0.4	0.3	0.5	1	1	1	0.1	0.1	0.1	0	0	0
Brazil	Paranaparema	0.2	0.1	0.2	0	0	0	0.0	0.0	0.0	0	0	0
Brazil	Paranapoema	0.3	0.2	0.3	0	0	0	0.1	0.0	0.1	0	0	0
Brazil	Paranapuã	0.2	0.1	0.2	0	0	0	0.0	0.0	0.0	0	0	0
Brazil	Paranatama	2.0	1.6	2.6	2	2	3	0.2	0.2	0.3	0	0	0
Brazil	Paranatinga	0.4	0.3	0.5	0	0	1	0.1	0.1	0.1	0	0	0
Brazil	Paranavaí	0.3	0.2	0.3	2	1	2	0.1	0.0	0.1	0	0	0
Brazil	Paranhos	0.5	0.3	0.6	0	0	1	0.2	0.1	0.2	0	0	0
Brazil	Paraopeba	0.3	0.2	0.4	1	0	1	0.1	0.0	0.1	0	0	0
Brazil	Parapuã	0.2	0.1	0.2	0	0	0	0.0	0.0	0.0	0	0	0
Brazil	Parari	1.0	0.7	1.2	0	0	0	0.1	0.1	0.1	0	0	0
Brazil	Parati	0.2	0.1	0.3	1	0	1	0.1	0.0	0.1	0	0	0
Brazil	Paratinga	1.7	1.4	2.2	6	5	8	0.2	0.2	0.3	1	1	1
Brazil	Paraú	1.2	0.9	1.5	1	0	1	0.1	0.1	0.2	0	0	0
Brazil	Parauapebas	0.9	0.7	1.1	7	6	9	0.1	0.1	0.2	4	3	5
Brazil	Paraúna	0.2	0.1	0.2	0	0	0	0.1	0.0	0.1	0	0	0
Brazil	Parazinho	1.2	0.9	1.5	0	0	1	0.1	0.1	0.2	0	0	0
Brazil	Pardinho	0.2	0.1	0.2	0	0	0	0.0	0.0	0.0	0	0	0
Brazil	Pareci Novo	0.1	0.1	0.2	0	0	0	0.0	0.0	0.0	0	0	0
Brazil	Parecis	0.4	0.3	0.5	0	0	0	0.1	0.1	0.1	0	0	0

Table 3: Diarrhoea Mortality by Unit *(continued)*

Country	Unit	2000 mortality mean rate (per 10,000)	2000 mortality lower rate (per 10,000)	2000 mortality upper rate (per 10,000)	2000 mortality mean count	2000 mortality lower count	2000 mortality upper count	2017 mortality mean rate (per 10,000)	2017 mortality lower rate (per 10,000)	2017 mortality upper rate (per 10,000)	2017 mortality mean count	2017 mortality lower count	2017 mortality upper count
Brazil	Parelhas	1.2	0.9	1.5	2	2	3	0.1	0.1	0.2	0	0	0
Brazil	Pariconha	2.8	2.2	3.7	4	3	5	0.3	0.2	0.4	0	0	1
Brazil	Parintins	0.8	0.6	0.9	9	7	11	0.2	0.2	0.3	3	2	3
Brazil	Paripiranga	1.7	1.4	2.2	6	4	7	0.2	0.2	0.3	1	0	1
Brazil	Paripueira	2.8	2.2	3.7	2	2	3	0.3	0.2	0.4	0	0	0
Brazil	Pariquera-Açu	0.2	0.1	0.2	0	0	0	0.0	0.0	0.0	0	0	0
Brazil	Parisi	0.2	0.1	0.2	0	0	0	0.0	0.0	0.0	0	0	0
Brazil	Parnaguá	1.4	1.1	1.8	2	2	2	0.2	0.1	0.2	0	0	0
Brazil	Parnaíba	1.4	1.1	1.8	21	16	27	0.2	0.1	0.2	2	2	3
Brazil	Parnamirim	2.0	1.6	2.6	5	4	7	0.2	0.2	0.3	1	0	1
Brazil	Parnamirim	1.2	0.9	1.5	19	15	25	0.1	0.1	0.2	3	2	4
Brazil	Parnarama	1.7	1.3	2.2	8	6	11	0.2	0.2	0.3	1	1	1
Brazil	Parobé	0.1	0.1	0.2	0	0	1	0.0	0.0	0.0	0	0	0
Brazil	Passa e Fica	1.2	0.9	1.5	1	1	1	0.1	0.1	0.2	0	0	0
Brazil	Passa Quatro	0.3	0.2	0.4	0	0	1	0.1	0.0	0.1	0	0	0
Brazil	Passa Sete	0.1	0.1	0.2	0	0	0	0.0	0.0	0.0	0	0	0
Brazil	Passa Tempo	0.3	0.2	0.4	0	0	0	0.1	0.0	0.1	0	0	0
Brazil	Passa Vinte	0.3	0.2	0.4	0	0	0	0.1	0.0	0.1	0	0	0
Brazil	Passabém	1.0	0.7	1.2	0	0	0	0.1	0.1	0.1	0	0	0
Brazil	Passabém	0.3	0.2	0.4	0	0	0	0.1	0.0	0.1	0	0	0
Brazil	Passabém	1.2	0.9	1.5	0	0	0	0.1	0.1	0.2	0	0	0
Brazil	Passagem Franca	1.7	1.3	2.2	3	3	4	0.2	0.2	0.3	0	0	1
Brazil	Passagem Franca do Piauí	1.4	1.1	1.8	1	1	1	0.2	0.1	0.2	0	0	0
Brazil	Passira	2.0	1.6	2.6	7	6	9	0.2	0.2	0.3	1	0	1
Brazil	Passo de Camaragibe	2.8	2.2	3.7	4	3	5	0.3	0.2	0.4	0	0	1
Brazil	Passo de Torres	0.2	0.2	0.3	0	0	0	0.1	0.0	0.1	0	0	0
Brazil	Passo do Sobrado	0.1	0.1	0.2	0	0	0	0.0	0.0	0.0	0	0	0
Brazil	Passo Fundo	0.1	0.1	0.2	2	1	2	0.0	0.0	0.0	0	0	1
Brazil	Passos	0.3	0.2	0.4	2	2	3	0.1	0.0	0.1	0	0	1
Brazil	Passos Maia	0.2	0.2	0.3	0	0	0	0.1	0.0	0.1	0	0	0
Brazil	Pastos Bons	1.7	1.3	2.2	4	3	6	0.2	0.2	0.3	1	0	1
Brazil	Patis	0.3	0.2	0.4	0	0	0	0.1	0.0	0.1	0	0	0
Brazil	Pato Bragado	0.3	0.2	0.3	0	0	0	0.1	0.0	0.1	0	0	0
Brazil	Pato Branco	0.3	0.2	0.3	1	1	2	0.1	0.0	0.1	0	0	0
Brazil	Patos	1.0	0.7	1.2	10	8	13	0.1	0.1	0.1	1	1	1
Brazil	Patos de Minas	0.3	0.2	0.4	3	2	4	0.1	0.0	0.1	1	0	1
Brazil	Patos do Piauí	1.4	1.1	1.8	1	1	1	0.2	0.1	0.2	0	0	0
Brazil	Patrocínio	0.3	0.2	0.4	2	1	2	0.1	0.0	0.1	0	0	0
Brazil	Patrocínio do Muriaé	0.3	0.2	0.4	0	0	0	0.1	0.0	0.1	0	0	0
Brazil	Patrocínio Paulista	0.2	0.1	0.2	0	0	0	0.0	0.0	0.0	0	0	0
Brazil	Patu	1.2	0.9	1.5	1	1	2	0.1	0.1	0.2	0	0	0
Brazil	Paty do Alferes	0.2	0.1	0.3	1	0	1	0.1	0.0	0.1	0	0	0
Brazil	Pau Brazil	1.7	1.4	2.2	3	2	4	0.2	0.2	0.3	0	0	0
Brazil	Pau d'Arco	0.9	0.7	1.1	1	0	1	0.1	0.1	0.2	0	0	0
Brazil	Pau d'Arco	0.8	0.7	1.1	0	0	1	0.1	0.1	0.2	0	0	0
Brazil	Pau dos Ferros	1.2	0.9	1.5	3	3	4	0.1	0.1	0.2	0	0	0
Brazil	Paudalho	2.0	1.6	2.6	15	12	19	0.2	0.2	0.3	2	1	2
Brazil	Pauini	0.8	0.6	0.9	2	1	2	0.2	0.2	0.3	1	0	1
Brazil	Paula Cândido	0.3	0.2	0.4	0	0	0	0.1	0.0	0.1	0	0	0
Brazil	Paula Freitas	0.3	0.2	0.3	0	0	0	0.1	0.0	0.1	0	0	0
Brazil	Paulicéia	0.2	0.1	0.2	0	0	0	0.0	0.0	0.0	0	0	0
Brazil	Paulínia	0.2	0.1	0.2	1	1	1	0.0	0.0	0.0	0	0	0
Brazil	Paulino Neves	1.7	1.3	2.2	3	2	4	0.2	0.2	0.3	1	0	1
Brazil	Paulista	2.0	1.6	2.6	46	36	60	0.2	0.2	0.3	5	3	6
Brazil	Paulista	1.0	0.7	1.2	1	1	2	0.1	0.1	0.1	0	0	0
Brazil	Paulistana	1.4	1.1	1.8	3	2	3	0.2	0.1	0.2	0	0	0

Table 3: Diarrhoea Mortality by Unit *(continued)*

Country	Unit	2000 mortality mean rate (per 10,000)	lower rate (per 10,000)	upper rate (per 10,000)	mean count	lower count	upper count	2017 mortality mean rate (per 10,000)	lower rate (per 10,000)	upper rate (per 10,000)	mean count	lower count	upper count
Brazil	Paulistânia	0.2	0.1	0.2	0	0	0	0.0	0.0	0.0	0	0	0
Brazil	Paulistas	0.3	0.2	0.4	0	0	0	0.1	0.0	0.1	0	0	0
Brazil	Paulo Afonso	1.7	1.4	2.2	18	14	23	0.2	0.2	0.3	3	2	3
Brazil	Paulo de Faria	0.2	0.1	0.2	0	0	0	0.0	0.0	0.0	0	0	0
Brazil	Paulo Frontin	0.3	0.2	0.3	0	0	0	0.1	0.0	0.1	0	0	0
Brazil	Paulo Jacinto	2.8	2.2	3.7	1	1	2	0.3	0.2	0.4	0	0	0
Brazil	Paulo Lopez	0.2	0.2	0.3	0	0	0	0.1	0.0	0.1	0	0	0
Brazil	Paulo Ramos	1.7	1.3	2.2	5	3	6	0.2	0.2	0.3	1	0	1
Brazil	Pavão	0.3	0.2	0.4	0	0	0	0.1	0.0	0.1	0	0	0
Brazil	Paverama	0.1	0.1	0.2	0	0	0	0.0	0.0	0.0	0	0	0
Brazil	Pavussu	1.4	1.1	1.8	1	0	1	0.2	0.1	0.2	0	0	0
Brazil	Pé de Serra	1.7	1.4	2.2	2	2	3	0.2	0.2	0.3	0	0	0
Brazil	Peabiru	0.3	0.2	0.3	0	0	0	0.1	0.0	0.1	0	0	0
Brazil	Peçanha	0.3	0.2	0.4	0	0	1	0.1	0.0	0.1	0	0	0
Brazil	Pederneiras	0.2	0.1	0.2	1	0	1	0.0	0.0	0.0	0	0	0
Brazil	Pedra	2.0	1.6	2.6	5	4	7	0.2	0.2	0.3	1	0	1
Brazil	Pedra Azul	0.3	0.2	0.4	1	1	1	0.1	0.0	0.1	0	0	0
Brazil	Pedra Bela	0.2	0.1	0.2	0	0	0	0.0	0.0	0.0	0	0	0
Brazil	Pedra Bonita	0.3	0.2	0.4	0	0	0	0.1	0.0	0.1	0	0	0
Brazil	Pedra Branca	2.3	1.7	2.9	12	9	15	0.1	0.1	0.2	1	0	1
Brazil	Pedra Branca	1.0	0.7	1.2	0	0	1	0.1	0.1	0.1	0	0	0
Brazil	Pedra Branca do Amaparí	0.2	0.2	0.3	0	0	0	0.1	0.1	0.2	0	0	0
Brazil	Pedra do Anta	0.3	0.2	0.4	0	0	0	0.1	0.0	0.1	0	0	0
Brazil	Pedra do Indaiá	0.3	0.2	0.4	0	0	0	0.1	0.0	0.1	0	0	0
Brazil	Pedra dourada	0.3	0.2	0.4	0	0	0	0.1	0.0	0.1	0	0	0
Brazil	Pedra Grande	1.2	0.9	1.5	1	0	1	0.1	0.1	0.2	0	0	0
Brazil	Pedra Lavadra	1.0	0.7	1.2	1	1	1	0.1	0.1	0.1	0	0	0
Brazil	Pedra Mole	1.3	1.0	1.7	0	0	0	0.2	0.1	0.3	0	0	0
Brazil	Pedra Preta	1.2	0.9	1.5	0	0	1	0.1	0.1	0.2	0	0	0
Brazil	Pedra Preta	0.4	0.3	0.5	0	0	1	0.1	0.1	0.1	0	0	0
Brazil	Pedralva	0.3	0.2	0.4	0	0	0	0.1	0.0	0.1	0	0	0
Brazil	Pedranópolis	0.2	0.1	0.2	0	0	0	0.0	0.0	0.0	0	0	0
Brazil	Pedrão	1.7	1.4	2.2	1	1	2	0.2	0.2	0.3	0	0	0
Brazil	Pedras de Fogo	1.0	0.7	1.2	4	3	5	0.1	0.1	0.1	0	0	0
Brazil	Pedras de Maria da Cruz	0.3	0.2	0.4	1	0	1	0.1	0.0	0.1	0	0	0
Brazil	Pedras Grandes	0.2	0.2	0.3	0	0	0	0.1	0.0	0.1	0	0	0
Brazil	Pedregulho	0.2	0.1	0.2	0	0	0	0.0	0.0	0.0	0	0	0
Brazil	Pedreira	0.2	0.1	0.2	0	0	0	0.0	0.0	0.0	0	0	0
Brazil	Pedreiras	1.7	1.3	2.2	9	7	12	0.2	0.2	0.3	1	1	1
Brazil	Pedrinhas	1.3	1.0	1.7	1	0	1	0.2	0.1	0.3	0	0	0
Brazil	Pedrinhas Paulista	0.2	0.1	0.2	0	0	0	0.0	0.0	0.0	0	0	0
Brazil	Pedrinópolis	0.3	0.2	0.4	0	0	0	0.1	0.0	0.1	0	0	0
Brazil	Pedro Afonso	0.8	0.7	1.1	0	0	1	0.1	0.1	0.2	0	0	0
Brazil	Pedro Alexandre	1.7	1.4	2.2	5	4	6	0.2	0.2	0.3	0	0	1
Brazil	Pedro Avelino	1.2	0.9	1.5	1	1	2	0.1	0.1	0.2	0	0	0
Brazil	Pedro Canário	0.3	0.2	0.3	0	0	0	0.1	0.1	0.1	0	0	0
Brazil	Pedro de Toledo	0.2	0.1	0.2	0	0	0	0.0	0.0	0.0	0	0	0
Brazil	Pedro do Rosário	1.7	1.3	2.2	4	3	6	0.2	0.2	0.3	1	1	1
Brazil	Pedro Gomes	0.5	0.3	0.6	0	0	1	0.2	0.1	0.2	0	0	0
Brazil	Pedro Laurentino	1.4	1.1	1.8	0	0	0	0.2	0.1	0.2	0	0	0
Brazil	Pedro Leopoldo	0.3	0.2	0.4	2	1	2	0.1	0.0	0.1	0	0	0
Brazil	Pedro Li	1.4	1.1	1.8	6	5	8	0.2	0.1	0.2	1	0	1
Brazil	Pedro Osório	0.1	0.1	0.2	0	0	0	0.0	0.0	0.0	0	0	0
Brazil	Pedro Régis	1.0	0.7	1.2	0	0	1	0.1	0.1	0.1	0	0	0
Brazil	Pedro Teixeira	0.3	0.2	0.4	0	0	0	0.1	0.0	0.1	0	0	0
Brazil	Pedro Velho	1.2	0.9	1.5	3	2	4	0.1	0.1	0.2	0	0	0
Brazil	Peixe	0.8	0.7	1.1	1	1	1	0.1	0.1	0.2	0	0	0

Table 3: Diarrhoea Mortality by Unit *(continued)*

Country	Unit	2000 mortality mean rate (per 10,000)	lower rate (per 10,000)	upper rate (per 10,000)	mean count	lower count	upper count	2017 mortality mean rate (per 10,000)	lower rate (per 10,000)	upper rate (per 10,000)	mean count	lower count	upper count
Brazil	Peixe Boi	0.9	0.7	1.1	1	1	2	0.1	0.1	0.2	0	0	0
Brazil	Peixoto de Azevedo	0.4	0.3	0.5	1	1	1	0.1	0.1	0.1	0	0	0
Brazil	Pejuçara	0.1	0.1	0.2	0	0	0	0.0	0.0	0.0	0	0	0
Brazil	Pelotas	0.1	0.1	0.2	3	2	4	0.0	0.0	0.0	1	0	1
Brazil	Penaforte	2.3	1.7	2.9	2	1	2	0.1	0.1	0.2	0	0	0
Brazil	Penalva	1.7	1.3	2.2	8	6	10	0.2	0.2	0.3	1	1	1
Brazil	Penápolis	0.2	0.1	0.2	1	0	1	0.0	0.0	0.0	0	0	0
Brazil	Pendências	1.2	0.9	1.5	1	1	2	0.1	0.1	0.2	0	0	0
Brazil	Penedo	2.8	2.2	3.7	24	19	32	0.3	0.2	0.4	2	2	3
Brazil	Penha	0.2	0.2	0.3	1	0	1	0.1	0.0	0.1	0	0	0
Brazil	Pentecoste	2.3	1.7	2.9	11	8	14	0.1	0.1	0.2	1	0	1
Brazil	Pequeri	0.3	0.2	0.4	0	0	0	0.1	0.0	0.1	0	0	0
Brazil	Pequi	0.3	0.2	0.4	0	0	0	0.1	0.0	0.1	0	0	0
Brazil	Pequizeiro	0.8	0.7	1.1	0	0	1	0.1	0.1	0.2	0	0	0
Brazil	Perdigão	0.3	0.2	0.4	0	0	0	0.1	0.0	0.1	0	0	0
Brazil	Perdizes	0.3	0.2	0.4	0	0	0	0.1	0.0	0.1	0	0	0
Brazil	Perdões	0.3	0.2	0.4	0	0	1	0.1	0.0	0.1	0	0	0
Brazil	Pereira Barreto	0.2	0.1	0.2	0	0	0	0.0	0.0	0.0	0	0	0
Brazil	Pereiras	0.2	0.1	0.2	0	0	0	0.0	0.0	0.0	0	0	0
Brazil	Pereiro	2.3	1.7	2.9	4	3	5	0.1	0.1	0.2	0	0	0
Brazil	Peri-Mirim	1.7	1.3	2.2	3	2	4	0.2	0.2	0.3	0	0	1
Brazil	Periquito	0.3	0.2	0.4	0	0	0	0.1	0.0	0.1	0	0	0
Brazil	Peritiba	0.2	0.2	0.3	0	0	0	0.1	0.0	0.1	0	0	0
Brazil	Peritoró	1.7	1.3	2.2	4	3	6	0.2	0.2	0.3	1	0	1
Brazil	Perobal	0.3	0.2	0.3	0	0	0	0.1	0.0	0.1	0	0	0
Brazil	Pérola	0.3	0.2	0.3	0	0	0	0.1	0.0	0.1	0	0	0
Brazil	Pérola d'Oeste	0.3	0.2	0.3	0	0	0	0.1	0.0	0.1	0	0	0
Brazil	Perolândia	0.2	0.1	0.2	0	0	0	0.1	0.0	0.1	0	0	0
Brazil	Peruíbe	0.2	0.1	0.2	1	0	1	0.0	0.0	0.0	0	0	0
Brazil	Pescador	0.3	0.2	0.4	0	0	0	0.1	0.0	0.1	0	0	0
Brazil	Pesqueira	2.0	1.6	2.6	14	11	18	0.2	0.2	0.3	1	1	2
Brazil	Petrolândia	2.0	1.6	2.6	7	6	9	0.2	0.2	0.3	1	1	1
Brazil	Petrolândia	0.2	0.2	0.3	0	0	0	0.1	0.0	0.1	0	0	0
Brazil	Petrolina	2.0	1.6	2.6	60	47	78	0.2	0.2	0.3	8	6	11
Brazil	Petrolina de Goiás	0.2	0.1	0.2	0	0	0	0.1	0.0	0.1	0	0	0
Brazil	Petrópolis	0.2	0.1	0.3	4	3	6	0.1	0.0	0.1	1	1	1
Brazil	Piaçabuçu	2.8	2.2	3.7	6	4	8	0.3	0.2	0.4	0	0	1
Brazil	Piacatu	0.2	0.1	0.2	0	0	0	0.0	0.0	0.0	0	0	0
Brazil	Piancó	1.0	0.7	1.2	2	1	2	0.1	0.1	0.1	0	0	0
Brazil	Piatã	1.7	1.4	2.2	4	3	5	0.2	0.2	0.3	0	0	1
Brazil	Piau	0.3	0.2	0.4	0	0	0	0.1	0.0	0.1	0	0	0
Brazil	Picada Café	0.1	0.1	0.2	0	0	0	0.0	0.0	0.0	0	0	0
Brazil	Piçarra	0.9	0.7	1.1	1	1	2	0.1	0.1	0.2	0	0	0
Brazil	Piçarras	0.2	0.2	0.3	0	0	0	0.1	0.0	0.1	0	0	0
Brazil	Picos	1.4	1.1	1.8	10	8	13	0.2	0.1	0.2	1	1	1
Brazil	Picuí	1.0	0.7	1.2	2	2	2	0.1	0.1	0.1	0	0	0
Brazil	Piedade	0.2	0.1	0.2	1	1	1	0.0	0.0	0.0	0	0	0
Brazil	Piedade de Caratinga	0.3	0.2	0.4	0	0	0	0.1	0.0	0.1	0	0	0
Brazil	Piedade do Ponte Nova	0.3	0.2	0.4	0	0	0	0.1	0.0	0.1	0	0	0
Brazil	Piedade do Rio Grande	0.3	0.2	0.4	0	0	0	0.1	0.0	0.1	0	0	0
Brazil	Piedade dos Gerais	0.3	0.2	0.4	0	0	0	0.1	0.0	0.1	0	0	0
Brazil	Piên	0.3	0.2	0.3	0	0	0	0.1	0.0	0.1	0	0	0
Brazil	Pilão Arcado	1.7	1.4	2.2	7	5	9	0.2	0.2	0.3	1	1	1
Brazil	Pilar	1.0	0.7	1.2	1	1	2	0.1	0.1	0.1	0	0	0

Table 3: Diarrhoea Mortality by Unit *(continued)*

Country	Unit	2000 mortality mean rate (per 10,000)	lower rate (per 10,000)	upper rate (per 10,000)	mean count	lower count	upper count	2017 mortality mean rate (per 10,000)	lower rate (per 10,000)	upper rate (per 10,000)	mean count	lower count	upper count
Brazil	Pilar	2.8	2.2	3.7	13	10	17	0.3	0.2	0.4	1	1	2
Brazil	Pilar de Goiás	0.2	0.1	0.2	0	0	0	0.1	0.0	0.1	0	0	0
Brazil	Pilar do Sul	0.2	0.1	0.2	0	0	0	0.0	0.0	0.0	0	0	0
Brazil	Pilões	1.2	0.9	1.5	0	0	0	0.1	0.1	0.2	0	0	0
Brazil	Pilões	1.0	0.7	1.2	1	1	1	0.1	0.1	0.1	0	0	0
Brazil	Pilõezinhos	1.0	0.7	1.2	0	0	1	0.1	0.1	0.1	0	0	0
Brazil	Pimenta	0.3	0.2	0.4	0	0	0	0.1	0.0	0.1	0	0	0
Brazil	Pimenta Bueno	0.4	0.3	0.5	1	1	2	0.1	0.1	0.1	0	0	0
Brazil	Pimenteiras	1.4	1.1	1.8	2	1	2	0.2	0.1	0.2	0	0	0
Brazil	Pimenteiras do Oeste	0.4	0.3	0.5	0	0	0	0.1	0.1	0.1	0	0	0
Brazil	Pindaí	1.7	1.4	2.2	3	2	3	0.2	0.2	0.3	0	0	0
Brazil	Pindamonhangaba	0.2	0.1	0.2	2	1	2	0.0	0.0	0.0	0	0	1
Brazil	Pindaré-Mirim	1.7	1.3	2.2	9	7	12	0.2	0.2	0.3	1	1	2
Brazil	Pindoba	2.8	2.2	3.7	1	1	1	0.3	0.2	0.4	0	0	0
Brazil	Pindobaçu	1.7	1.4	2.2	4	3	6	0.2	0.2	0.3	1	0	1
Brazil	Pindorama	0.2	0.1	0.2	0	0	0	0.0	0.0	0.0	0	0	0
Brazil	Pindorama do Tocantins	0.8	0.7	1.1	0	0	1	0.1	0.1	0.2	0	0	0
Brazil	Pindoretama	2.3	1.7	2.9	3	2	3	0.1	0.1	0.2	0	0	0
Brazil	Pingo d'Água	0.3	0.2	0.4	0	0	0	0.1	0.0	0.1	0	0	0
Brazil	Pinhais	0.3	0.2	0.3	4	3	5	0.1	0.0	0.1	1	1	1
Brazil	Pinhal	0.1	0.1	0.2	0	0	0	0.0	0.0	0.0	0	0	0
Brazil	Pinhal	0.2	0.1	0.2	0	0	0	0.0	0.0	0.0	0	0	0
Brazil	Pinhal de São Bento	0.3	0.2	0.3	0	0	0	0.1	0.0	0.1	0	0	0
Brazil	Pinhal Grande	0.1	0.1	0.2	0	0	0	0.0	0.0	0.0	0	0	0
Brazil	Pinhalão	0.3	0.2	0.3	0	0	0	0.1	0.0	0.1	0	0	0
Brazil	Pinhalzinho	0.2	0.2	0.3	0	0	0	0.1	0.0	0.1	0	0	0
Brazil	Pinhalzinho	0.2	0.1	0.2	0	0	0	0.0	0.0	0.0	0	0	0
Brazil	Pinhão	0.3	0.2	0.3	1	1	1	0.1	0.0	0.1	0	0	0
Brazil	Pinhão	1.3	1.0	1.7	1	1	1	0.2	0.1	0.3	0	0	0
Brazil	Pinheiral	0.2	0.1	0.3	0	0	0	0.1	0.0	0.1	0	0	0
Brazil	Pinheirinho do Vale	0.1	0.1	0.2	0	0	0	0.0	0.0	0.0	0	0	0
Brazil	Pinheiro	1.7	1.3	2.2	15	12	20	0.2	0.2	0.3	2	2	3
Brazil	Pinheiro Machado	0.1	0.1	0.2	0	0	0	0.0	0.0	0.0	0	0	0
Brazil	Pinheiro Preto	0.2	0.2	0.3	0	0	0	0.1	0.0	0.1	0	0	0
Brazil	Pinheiros	0.3	0.2	0.3	1	0	1	0.1	0.1	0.1	0	0	0
Brazil	Pintadas	1.7	1.4	2.2	2	2	3	0.2	0.2	0.3	0	0	0
Brazil	Pintópolis	0.3	0.2	0.4	0	0	0	0.1	0.0	0.1	0	0	0
Brazil	Pio IX	1.4	1.1	1.8	3	2	4	0.2	0.1	0.2	0	0	0
Brazil	Pio XII	1.7	1.3	2.2	8	6	10	0.2	0.2	0.3	1	1	1
Brazil	Piquerobi	0.2	0.1	0.2	0	0	0	0.0	0.0	0.0	0	0	0
Brazil	Piquet Carneiro	2.3	1.7	2.9	3	2	4	0.1	0.1	0.2	0	0	0
Brazil	Piquete	0.2	0.1	0.2	0	0	0	0.0	0.0	0.0	0	0	0
Brazil	Piracaia	0.2	0.1	0.2	0	0	0	0.0	0.0	0.0	0	0	0
Brazil	Piracanjuba	0.2	0.1	0.2	0	0	0	0.1	0.0	0.1	0	0	0
Brazil	Piracema	0.3	0.2	0.4	0	0	0	0.1	0.0	0.1	0	0	0
Brazil	Piracicaba	0.2	0.1	0.2	4	3	5	0.0	0.0	0.0	1	1	1
Brazil	Piracununga	0.2	0.1	0.2	1	1	1	0.0	0.0	0.0	0	0	0
Brazil	Piracuruca	1.4	1.1	1.8	4	3	6	0.2	0.1	0.2	0	0	1
Brazil	Piraí	0.2	0.1	0.3	0	0	1	0.1	0.0	0.1	0	0	0
Brazil	Piraí do Norte	1.7	1.4	2.2	3	2	4	0.2	0.2	0.3	0	0	0
Brazil	Piraí do Sul	0.3	0.2	0.3	1	0	1	0.1	0.0	0.1	0	0	0
Brazil	Pirajuba	0.3	0.2	0.4	0	0	0	0.1	0.0	0.1	0	0	0
Brazil	Pirajui	0.2	0.1	0.2	1	0	1	0.0	0.0	0.0	0	0	0
Brazil	Pirajuí	0.2	0.1	0.2	0	0	0	0.0	0.0	0.0	0	0	0
Brazil	Pirambu	1.3	1.0	1.7	1	1	1	0.2	0.1	0.3	0	0	0

Table 3: Diarrhoea Mortality by Unit *(continued)*

Country	Unit	2000 mortality mean rate (per 10,000)	lower rate (per 10,000)	upper rate (per 10,000)	mean count	lower count	upper count	2017 mortality mean rate (per 10,000)	lower rate (per 10,000)	upper rate (per 10,000)	mean count	lower count	upper count
Brazil	Piranga	0.3	0.2	0.4	0	0	1	0.1	0.0	0.1	0	0	0
Brazil	Pirangi	0.2	0.1	0.2	0	0	0	0.0	0.0	0.0	0	0	0
Brazil	Piranguçu	0.3	0.2	0.4	0	0	0	0.1	0.0	0.1	0	0	0
Brazil	Piranguinho	0.3	0.2	0.4	0	0	0	0.1	0.0	0.1	0	0	0
Brazil	Piranhas	0.2	0.1	0.2	0	0	0	0.1	0.0	0.1	0	0	0
Brazil	Piranhas	2.8	2.2	3.7	7	6	10	0.3	0.2	0.4	1	1	1
Brazil	Pirapemas	1.7	1.3	2.2	4	3	5	0.2	0.2	0.3	1	0	1
Brazil	Pirapetinga	0.3	0.2	0.4	0	0	0	0.1	0.0	0.1	0	0	0
Brazil	Pirapó	0.1	0.1	0.2	0	0	0	0.0	0.0	0.0	0	0	0
Brazil	Pirapora	0.3	0.2	0.4	1	1	2	0.1	0.0	0.1	0	0	0
Brazil	Pirapora do Bom Jesus	0.2	0.1	0.2	0	0	0	0.0	0.0	0.0	0	0	0
Brazil	Pirapozinho	0.2	0.1	0.2	0	0	0	0.0	0.0	0.0	0	0	0
Brazil	Piraquara	0.3	0.2	0.3	2	2	3	0.1	0.0	0.1	1	0	1
Brazil	Piraquê	0.8	0.7	1.1	0	0	0	0.1	0.1	0.2	0	0	0
Brazil	Piratini	0.1	0.1	0.2	0	0	0	0.0	0.0	0.0	0	0	0
Brazil	Piratininga	0.2	0.1	0.2	0	0	0	0.0	0.0	0.0	0	0	0
Brazil	Piratuba	0.2	0.2	0.3	0	0	0	0.1	0.0	0.1	0	0	0
Brazil	Pirauba	0.3	0.2	0.4	0	0	0	0.1	0.0	0.1	0	0	0
Brazil	Pirenópolis	0.2	0.1	0.2	0	0	1	0.1	0.0	0.1	0	0	0
Brazil	Pires do Rio	0.2	0.1	0.2	0	0	1	0.1	0.0	0.1	0	0	0
Brazil	Pires Ferreira	2.3	1.7	2.9	3	2	3	0.1	0.1	0.2	0	0	0
Brazil	Piripá	1.7	1.4	2.2	3	2	4	0.2	0.2	0.3	0	0	0
Brazil	Piripiri	1.4	1.1	1.8	10	8	13	0.2	0.1	0.2	1	1	1
Brazil	Piritiba	1.7	1.4	2.2	4	3	5	0.2	0.2	0.3	1	0	1
Brazil	Pirpirituba	1.0	0.7	1.2	1	1	1	0.1	0.1	0.1	0	0	0
Brazil	Pitanga	0.3	0.2	0.3	1	1	1	0.1	0.0	0.1	0	0	0
Brazil	Pitangueiras	0.3	0.2	0.3	0	0	0	0.1	0.0	0.1	0	0	0
Brazil	Pitangueiras	0.2	0.1	0.2	0	0	1	0.0	0.0	0.0	0	0	0
Brazil	Pitangui	0.3	0.2	0.4	1	0	1	0.1	0.0	0.1	0	0	0
Brazil	Pitimbu	1.0	0.7	1.2	1	1	1	0.1	0.1	0.1	0	0	0
Brazil	Piui	0.3	0.2	0.4	1	1	1	0.1	0.0	0.1	0	0	0
Brazil	Pium	0.8	0.7	1.1	1	1	1	0.1	0.1	0.2	0	0	0
Brazil	Piúma	0.3	0.2	0.3	0	0	0	0.1	0.1	0.1	0	0	0
Brazil	Placas	0.9	0.7	1.1	1	1	2	0.1	0.1	0.2	0	0	1
Brazil	Plácido de Castro	1.1	0.9	1.3	2	1	2	0.2	0.2	0.3	0	0	0
Brazil	Planaltina	0.2	0.1	0.2	2	1	2	0.1	0.0	0.1	0	0	0
Brazil	Planaltina do Paraná	0.3	0.2	0.3	0	0	0	0.1	0.0	0.1	0	0	0
Brazil	Planaltino	1.7	1.4	2.2	2	1	2	0.2	0.2	0.3	0	0	0
Brazil	Planalto	0.3	0.2	0.3	0	0	0	0.1	0.0	0.1	0	0	0
Brazil	Planalto	0.1	0.1	0.2	0	0	0	0.0	0.0	0.0	0	0	0
Brazil	Planalto	1.7	1.4	2.2	4	3	5	0.2	0.2	0.3	1	0	1
Brazil	Planalto	0.2	0.1	0.2	0	0	0	0.0	0.0	0.0	0	0	0
Brazil	Planalto Alegre	0.2	0.2	0.3	0	0	0	0.1	0.0	0.1	0	0	0
Brazil	Planalto da Serra	0.4	0.3	0.5	0	0	0	0.1	0.1	0.1	0	0	0
Brazil	Planura	0.3	0.2	0.4	0	0	0	0.1	0.0	0.1	0	0	0
Brazil	Platina	0.2	0.1	0.2	0	0	0	0.0	0.0	0.0	0	0	0
Brazil	Poá	0.2	0.1	0.2	2	1	3	0.0	0.0	0.0	0	0	1
Brazil	Poção	2.0	1.6	2.6	2	2	3	0.2	0.2	0.3	0	0	0
Brazil	Poção de Pedras	1.7	1.3	2.2	5	4	6	0.2	0.2	0.3	0	0	1
Brazil	Pocinhos	1.0	0.7	1.2	2	1	2	0.1	0.1	0.1	0	0	0
Brazil	Poço Branco	1.2	0.9	1.5	1	1	2	0.1	0.1	0.2	0	0	0
Brazil	Poço Dantas	1.2	0.9	1.5	0	0	1	0.1	0.1	0.2	0	0	0
Brazil	Poço das Antas	0.1	0.1	0.2	0	0	0	0.0	0.0	0.0	0	0	0
Brazil	Poço das Trincheiras	2.8	2.2	3.7	5	4	7	0.3	0.2	0.4	0	0	1
Brazil	Poço de José de Moura	1.0	0.7	1.2	3	2	4	0.1	0.1	0.1	0	0	0
Brazil	Poço Fundo	0.3	0.2	0.4	0	0	0	0.1	0.0	0.1	0	0	0

Table 3: Diarrhoea Mortality by Unit *(continued)*

Country	Unit	2000 mortality mean rate (per 10,000)	lower rate (per 10,000)	upper rate (per 10,000)	mean count	lower count	upper count	2017 mortality mean rate (per 10,000)	lower rate (per 10,000)	upper rate (per 10,000)	mean count	lower count	upper count
Brazil	Poço Redondo	1.3	1.0	1.7	5	4	6	0.2	0.1	0.3	1	1	1
Brazil	Poço Verde	1.3	1.0	1.7	3	2	4	0.2	0.1	0.3	0	0	0
Brazil	Poções	1.7	1.4	2.2	9	7	11	0.2	0.2	0.3	1	1	1
Brazil	Poconé	0.4	0.3	0.5	1	1	2	0.1	0.1	0.1	0	0	0
Brazil	Poços de Caldas	0.3	0.2	0.4	3	2	4	0.1	0.0	0.1	1	0	1
Brazil	Pocrane	0.3	0.2	0.4	0	0	0	0.1	0.0	0.1	0	0	0
Brazil	Pojuca	1.7	1.4	2.2	3	2	4	0.2	0.2	0.3	0	0	1
Brazil	Poloni	0.2	0.1	0.2	0	0	0	0.0	0.0	0.0	0	0	0
Brazil	Pombal	1.0	0.7	1.2	1	0	1	0.1	0.1	0.1	0	0	0
Brazil	Pombos	2.0	1.6	2.6	7	5	9	0.2	0.2	0.3	1	0	1
Brazil	Pomerode	0.2	0.2	0.3	0	0	0	0.1	0.0	0.1	0	0	0
Brazil	Pompéia	0.2	0.1	0.2	0	0	0	0.0	0.0	0.0	0	0	0
Brazil	Pompéu	0.3	0.2	0.4	1	1	1	0.1	0.0	0.1	0	0	0
Brazil	Pongaí	0.2	0.1	0.2	0	0	0	0.0	0.0	0.0	0	0	0
Brazil	Ponta Alta	0.2	0.2	0.3	0	0	0	0.1	0.0	0.1	0	0	0
Brazil	Ponta de Pedras	0.9	0.7	1.1	2	1	2	0.1	0.1	0.2	0	0	1
Brazil	Ponta Grossa	0.3	0.2	0.3	7	6	10	0.1	0.0	0.1	1	1	2
Brazil	Ponta Porã	0.5	0.3	0.6	2	2	3	0.2	0.1	0.2	1	1	1
Brazil	Pontal	0.2	0.1	0.2	0	0	1	0.0	0.0	0.0	0	0	0
Brazil	Pontal do Araguaia	0.4	0.3	0.5	1	1	1	0.1	0.1	0.1	0	0	0
Brazil	Pontal do Paraná	0.3	0.2	0.3	0	0	0	0.1	0.0	0.1	0	0	0
Brazil	Pontalina	0.2	0.1	0.2	0	0	0	0.1	0.0	0.1	0	0	0
Brazil	Pontalinda	0.2	0.1	0.2	0	0	0	0.0	0.0	0.0	0	0	0
Brazil	Pontão	0.1	0.1	0.2	0	0	0	0.0	0.0	0.0	0	0	0
Brazil	Ponte Alta do Bom Jesus	0.8	0.7	1.1	0	0	0	0.1	0.1	0.2	0	0	0
Brazil	Ponte Alta do Norte	0.8	0.7	1.1	1	0	1	0.1	0.1	0.2	0	0	0
Brazil	Ponte Alta do Norte	0.2	0.2	0.3	0	0	0	0.1	0.0	0.1	0	0	0
Brazil	Ponte Branca	0.4	0.3	0.5	0	0	0	0.1	0.1	0.1	0	0	0
Brazil	Ponte Nova	0.3	0.2	0.4	1	1	2	0.1	0.0	0.1	0	0	0
Brazil	Ponte Preta	0.1	0.1	0.2	0	0	0	0.0	0.0	0.0	0	0	0
Brazil	Ponte Serrada	0.2	0.2	0.3	0	0	0	0.1	0.0	0.1	0	0	0
Brazil	Pontes e Lacerda	0.4	0.3	0.5	2	2	3	0.1	0.1	0.1	0	0	1
Brazil	Pontes Gestral	0.2	0.1	0.2	0	0	0	0.0	0.0	0.0	0	0	0
Brazil	Ponto Belo	0.3	0.2	0.3	0	0	0	0.1	0.1	0.1	0	0	0
Brazil	Ponto Chique	0.3	0.2	0.4	0	0	0	0.1	0.0	0.1	0	0	0
Brazil	Ponto dos Volantes	0.3	0.2	0.4	0	0	0	0.1	0.0	0.1	0	0	0
Brazil	Ponto Novo	1.7	1.4	2.2	3	2	4	0.2	0.2	0.3	0	0	0
Brazil	Populina	0.2	0.1	0.2	0	0	0	0.0	0.0	0.0	0	0	0
Brazil	Poranga	2.3	1.7	2.9	3	2	4	0.1	0.1	0.2	0	0	0
Brazil	Porangaba	0.2	0.1	0.2	0	0	0	0.0	0.0	0.0	0	0	0
Brazil	Porangatu	0.2	0.1	0.2	1	1	1	0.1	0.0	0.1	0	0	0
Brazil	Porciúncula	0.2	0.1	0.3	0	0	0	0.1	0.0	0.1	0	0	0
Brazil	Porecatu	0.3	0.2	0.3	0	0	0	0.1	0.0	0.1	0	0	0
Brazil	Portalegre	1.2	0.9	1.5	1	0	1	0.1	0.1	0.2	0	0	0
Brazil	Portao	0.1	0.1	0.2	0	0	0	0.0	0.0	0.0	0	0	0
Brazil	Porteiras	2.3	1.7	2.9	4	3	5	0.1	0.1	0.2	0	0	0
Brazil	Porteirinha	0.3	0.2	0.4	1	1	1	0.1	0.0	0.1	0	0	0
Brazil	Porteiro	0.2	0.1	0.2	0	0	0	0.1	0.0	0.1	0	0	0
Brazil	Portel	0.9	0.7	1.1	5	4	6	0.1	0.1	0.2	1	1	1
Brazil	Portelândia	0.2	0.1	0.2	0	0	0	0.1	0.0	0.1	0	0	0
Brazil	Porto	1.4	1.1	1.8	2	2	3	0.2	0.1	0.2	0	0	0
Brazil	Porto Acre	1.1	0.9	1.3	1	1	2	0.2	0.2	0.3	0	0	0
Brazil	Porto Alegre	0.1	0.1	0.2	12	9	16	0.0	0.0	0.0	3	2	3
Brazil	Porto Alegre do Norte	0.4	0.3	0.5	0	0	0	0.1	0.1	0.1	0	0	0

Table 3: Diarrhoea Mortality by Unit *(continued)*

Country	Unit	2000 mortality mean rate (per 10,000)	lower rate (per 10,000)	upper rate (per 10,000)	mean count	lower count	upper count	2017 mortality mean rate (per 10,000)	lower rate (per 10,000)	upper rate (per 10,000)	mean count	lower count	upper count
Brazil	Porto Alegre do Piauí	1.4	1.1	1.8	0	0	1	0.2	0.1	0.2	0	0	0
Brazil	Porto Alegre do Tocantins	0.8	0.7	1.1	0	0	0	0.1	0.1	0.2	0	0	0
Brazil	Porto Amazonas	0.3	0.2	0.3	0	0	0	0.1	0.0	0.1	0	0	0
Brazil	Porto Barreiro	0.3	0.2	0.3	0	0	0	0.1	0.0	0.1	0	0	0
Brazil	Porto Belo	0.2	0.2	0.3	0	0	1	0.1	0.0	0.1	0	0	0
Brazil	Porto Calvo	2.8	2.2	3.7	7	6	10	0.3	0.2	0.4	1	0	1
Brazil	Porto da Folha	1.3	1.0	1.7	6	4	7	0.2	0.1	0.3	1	0	1
Brazil	Porto de Moz	0.9	0.7	1.1	3	2	4	0.1	0.1	0.2	1	1	1
Brazil	Porto de Pedras	2.8	2.2	3.7	8	6	10	0.3	0.2	0.4	1	0	1
Brazil	Porto do Mangue	1.2	0.9	1.5	1	0	1	0.1	0.1	0.2	0	0	0
Brazil	Porto dos Gaúchos	0.4	0.3	0.5	0	0	0	0.1	0.1	0.1	0	0	0
Brazil	Porto Esperidião	0.4	0.3	0.5	0	0	1	0.1	0.1	0.1	0	0	0
Brazil	Porto Estrela	0.4	0.3	0.5	0	0	0	0.1	0.1	0.1	0	0	0
Brazil	Porto Feliz	0.2	0.1	0.2	1	0	1	0.0	0.0	0.0	0	0	0
Brazil	Porto Ferreira	0.2	0.1	0.2	1	0	1	0.0	0.0	0.0	0	0	0
Brazil	Porto Firme	0.3	0.2	0.4	0	0	0	0.1	0.0	0.1	0	0	0
Brazil	Porto Franco	1.7	1.3	2.2	5	4	6	0.2	0.2	0.3	1	1	1
Brazil	Porto Grande	0.2	0.2	0.3	0	0	0	0.1	0.1	0.2	0	0	0
Brazil	Porto Lucena	0.1	0.1	0.2	0	0	0	0.0	0.0	0.0	0	0	0
Brazil	Porto Mauá	0.1	0.1	0.2	0	0	0	0.0	0.0	0.0	0	0	0
Brazil	Porto Murtinho	0.5	0.3	0.6	1	0	1	0.2	0.1	0.2	0	0	0
Brazil	Porto Nacional	0.8	0.7	1.1	5	3	6	0.1	0.1	0.2	1	0	1
Brazil	Porto Real	0.2	0.1	0.3	0	0	0	0.1	0.0	0.1	0	0	0
Brazil	Porto Real do Colégio	2.8	2.2	3.7	8	6	11	0.3	0.2	0.4	1	1	1
Brazil	Porto Rico	0.3	0.2	0.3	0	0	0	0.1	0.0	0.1	0	0	0
Brazil	Porto Rico do Maranhão	1.7	1.3	2.2	1	1	2	0.2	0.2	0.3	0	0	0
Brazil	Porto Seguro	1.7	1.4	2.2	23	18	30	0.2	0.2	0.3	4	3	5
Brazil	Porto União	0.2	0.2	0.3	1	1	1	0.1	0.0	0.1	0	0	0
Brazil	Porto Velho	0.4	0.3	0.5	15	12	19	0.1	0.1	0.1	4	4	5
Brazil	Porto Vera Cruz	0.1	0.1	0.2	0	0	0	0.0	0.0	0.0	0	0	0
Brazil	Porto Vitória	0.3	0.2	0.3	0	0	0	0.1	0.0	0.1	0	0	0
Brazil	Porto Walter	1.1	0.9	1.3	1	1	1	0.2	0.2	0.3	0	0	0
Brazil	Porto Xavier	0.1	0.1	0.2	0	0	0	0.0	0.0	0.0	0	0	0
Brazil	Posse	0.2	0.1	0.2	0	0	1	0.1	0.0	0.1	0	0	0
Brazil	Poté	0.3	0.2	0.4	0	0	1	0.1	0.0	0.1	0	0	0
Brazil	Potengi	2.3	1.7	2.9	3	2	3	0.1	0.1	0.2	0	0	0
Brazil	Potim	0.2	0.1	0.2	0	0	0	0.0	0.0	0.0	0	0	0
Brazil	Potiraguá	1.7	1.4	2.2	3	2	3	0.2	0.2	0.3	0	0	0
Brazil	Potirendaba	0.2	0.1	0.2	0	0	0	0.0	0.0	0.0	0	0	0
Brazil	Potiretama	2.3	1.7	2.9	1	1	2	0.1	0.1	0.2	0	0	0
Brazil	Pouso Alegre	0.3	0.2	0.4	2	2	3	0.1	0.0	0.1	1	0	1
Brazil	Pouso Alto	0.3	0.2	0.4	0	0	0	0.1	0.0	0.1	0	0	0
Brazil	Pouso Novo	0.1	0.1	0.2	0	0	0	0.0	0.0	0.0	0	0	0
Brazil	Pouso Redondo	0.2	0.2	0.3	0	0	0	0.1	0.0	0.1	0	0	0
Brazil	Poxoréo	0.4	0.3	0.5	1	1	1	0.1	0.1	0.1	0	0	0
Brazil	Pracinha	0.2	0.1	0.2	0	0	0	0.0	0.0	0.0	0	0	0
Brazil	Pracuúba	0.2	0.2	0.3	0	0	0	0.1	0.1	0.2	0	0	0
Brazil	Prado	1.7	1.4	2.2	5	4	7	0.2	0.2	0.3	1	0	1
Brazil	Prado Ferreira	0.3	0.2	0.3	0	0	0	0.1	0.0	0.1	0	0	0
Brazil	Pradópolis	0.2	0.1	0.2	0	0	0	0.0	0.0	0.0	0	0	0
Brazil	Prados	0.3	0.2	0.4	0	0	0	0.1	0.0	0.1	0	0	0
Brazil	Praia Grande	0.2	0.2	0.3	0	0	0	0.1	0.0	0.1	0	0	0
Brazil	Praia Grande	0.2	0.1	0.2	1	1	1	0.0	0.0	0.0	0	0	0
Brazil	Praia Norte	0.8	0.7	1.1	1	0	1	0.1	0.1	0.2	0	0	0
Brazil	Prainha	0.9	0.7	1.1	3	2	3	0.1	0.1	0.2	0	0	1

Country	Unit	2000 mortality mean rate (per 10,000)	lower rate (per 10,000)	upper rate (per 10,000)	mean count	lower count	upper count	2017 mortality mean rate (per 10,000)	lower rate (per 10,000)	upper rate (per 10,000)	mean count	lower count	upper count
Brazil	Pranchita	0.3	0.2	0.3	0	0	0	0.1	0.0	0.1	0	0	0
Brazil	Prata	1.0	0.7	1.2	0	0	0	0.1	0.1	0.1	0	0	0
Brazil	Prata	0.3	0.2	0.4	1	0	1	0.1	0.0	0.1	0	0	0
Brazil	Prata do Piauí	1.4	1.1	1.8	0	0	1	0.2	0.1	0.2	0	0	0
Brazil	Pratânia	0.2	0.1	0.2	0	0	0	0.0	0.0	0.0	0	0	0
Brazil	Pratápolis	0.3	0.2	0.4	0	0	0	0.1	0.0	0.1	0	0	0
Brazil	Pratinha	0.3	0.2	0.4	0	0	0	0.1	0.0	0.1	0	0	0
Brazil	Presidente Alves	0.2	0.1	0.2	0	0	0	0.0	0.0	0.0	0	0	0
Brazil	Presidente Bernardes	0.2	0.1	0.2	0	0	0	0.0	0.0	0.0	0	0	0
Brazil	Presidente Bernardes	0.3	0.2	0.4	0	0	0	0.1	0.0	0.1	0	0	0
Brazil	Presidente Castelo Branco	0.2	0.2	0.3	0	0	0	0.1	0.0	0.1	0	0	0
Brazil	Presidente Castelo Branco	0.3	0.2	0.3	0	0	0	0.1	0.0	0.1	0	0	0
Brazil	Presidente Dutra	1.7	1.3	2.2	10	8	13	0.2	0.2	0.3	1	1	2
Brazil	Presidente Dutra	1.7	1.4	2.2	3	2	4	0.2	0.2	0.3	0	0	0
Brazil	Presidente Epitácio	0.2	0.1	0.2	0	0	1	0.0	0.0	0.0	0	0	0
Brazil	Presidente Figueiredo	0.8	0.6	0.9	2	1	2	0.2	0.2	0.3	1	1	1
Brazil	Presidente Getúlio	0.2	0.2	0.3	0	0	0	0.1	0.0	0.1	0	0	0
Brazil	Presidente Jânio Quadros	1.7	1.4	2.2	3	3	4	0.2	0.2	0.3	0	0	0
Brazil	Presidente Juscelino	1.2	0.9	1.5	1	1	1	0.1	0.1	0.2	0	0	0
Brazil	Presidente Juscelino	1.7	1.3	2.2	3	2	4	0.2	0.2	0.3	0	0	0
Brazil	Presidente Juscelino	0.3	0.2	0.4	0	0	0	0.1	0.0	0.1	0	0	0
Brazil	Presidente Kennedy	0.8	0.7	1.1	0	0	1	0.1	0.1	0.2	0	0	0
Brazil	Presidente Kennedy	0.3	0.2	0.3	0	0	0	0.1	0.1	0.1	0	0	0
Brazil	Presidente Kubitschek	0.3	0.2	0.4	0	0	0	0.1	0.0	0.1	0	0	0
Brazil	Presidente Lucena	0.1	0.1	0.2	0	0	0	0.0	0.0	0.0	0	0	0
Brazil	Presidente Médici	1.7	1.3	2.2	1	1	2	0.2	0.2	0.3	0	0	0
Brazil	Presidente Médici	0.4	0.3	0.5	1	1	1	0.1	0.1	0.1	0	0	0
Brazil	Presidente Nereu	0.2	0.2	0.3	0	0	0	0.1	0.0	0.1	0	0	0
Brazil	Presidente Olegário	0.3	0.2	0.4	0	0	1	0.1	0.0	0.1	0	0	0
Brazil	Presidente Prudente	0.2	0.1	0.2	2	2	3	0.0	0.0	0.0	0	0	1
Brazil	Presidente Sarney	1.7	1.3	2.2	3	2	4	0.2	0.2	0.3	0	0	1
Brazil	Presidente Tancredo Neves	1.7	1.4	2.2	4	3	5	0.2	0.2	0.3	1	0	1
Brazil	Presidente Vargas	1.7	1.3	2.2	3	3	4	0.2	0.2	0.3	0	0	1
Brazil	Presidente Venceslau	0.2	0.1	0.2	0	0	0	0.0	0.0	0.0	0	0	0
Brazil	Primavera	2.0	1.6	2.6	2	2	3	0.2	0.2	0.3	0	0	0
Brazil	Primavera	0.9	0.7	1.1	1	1	1	0.1	0.1	0.2	0	0	0
Brazil	Primavera de Rondônia	0.4	0.3	0.5	0	0	0	0.1	0.1	0.1	0	0	0
Brazil	Primavera do Leste	0.4	0.3	0.5	1	1	2	0.1	0.1	0.1	0	0	1
Brazil	Primeira Cruz	1.7	1.3	2.2	4	3	5	0.2	0.2	0.3	1	1	1

Table 3: Diarrhoea Mortality by Unit *(continued)*

Country	Unit	2000 mortality mean rate (per 10,000)	lower rate (per 10,000)	upper rate (per 10,000)	mean count	lower count	upper count	2017 mortality mean rate (per 10,000)	lower rate (per 10,000)	upper rate (per 10,000)	mean count	lower count	upper count
Brazil	Primeiro de Maio	0.3	0.2	0.3	0	0	0	0.1	0.0	0.1	0	0	0
Brazil	Princesa	0.2	0.2	0.3	0	0	0	0.1	0.0	0.1	0	0	0
Brazil	Princesa Isabel	1.0	0.7	1.2	2	1	2	0.1	0.1	0.1	0	0	0
Brazil	Professor Jamil	0.2	0.1	0.2	0	0	0	0.1	0.0	0.1	0	0	0
Brazil	Progresso	0.1	0.1	0.2	0	0	0	0.0	0.0	0.0	0	0	0
Brazil	Promissão	0.2	0.1	0.2	0	0	1	0.0	0.0	0.0	0	0	0
Brazil	Propriá	1.3	1.0	1.7	3	2	4	0.2	0.1	0.3	0	0	1
Brazil	Protásio Alves	0.1	0.1	0.2	0	0	0	0.0	0.0	0.0	0	0	0
Brazil	Prudente de Morais	0.3	0.2	0.4	0	0	0	0.1	0.0	0.1	0	0	0
Brazil	Prudentópolis	0.3	0.2	0.3	1	1	2	0.1	0.0	0.1	0	0	0
Brazil	Pugmil	0.8	0.7	1.1	0	0	0	0.1	0.1	0.2	0	0	0
Brazil	Pureza	1.2	0.9	1.5	1	1	2	0.1	0.1	0.2	0	0	0
Brazil	Putinga	0.1	0.1	0.2	0	0	0	0.0	0.0	0.0	0	0	0
Brazil	Puxinanã	1.0	0.7	1.2	1	1	2	0.1	0.1	0.1	0	0	0
Brazil	Quadra	0.2	0.1	0.2	0	0	0	0.0	0.0	0.0	0	0	0
Brazil	Quaraí	0.1	0.1	0.2	0	0	0	0.0	0.0	0.0	0	0	0
Brazil	Quartel Geral	0.3	0.2	0.4	0	0	0	0.1	0.0	0.1	0	0	0
Brazil	Quarto Centenário	0.3	0.2	0.3	0	0	0	0.1	0.0	0.1	0	0	0
Brazil	Quatá	0.2	0.1	0.2	0	0	0	0.0	0.0	0.0	0	0	0
Brazil	Quatiguá	0.3	0.2	0.3	0	0	0	0.1	0.0	0.1	0	0	0
Brazil	Quatipuru	0.9	0.7	1.1	1	1	1	0.1	0.1	0.2	0	0	0
Brazil	Quatis	0.2	0.1	0.3	0	0	0	0.1	0.0	0.1	0	0	0
Brazil	Quatro Barras	0.3	0.2	0.3	0	0	1	0.1	0.0	0.1	0	0	0
Brazil	Quatro Pontes	0.3	0.2	0.3	0	0	0	0.1	0.0	0.1	0	0	0
Brazil	Quebrangulo	2.8	2.2	3.7	4	3	6	0.3	0.2	0.4	0	0	1
Brazil	Quedas do Iguaçu	0.3	0.2	0.3	1	1	1	0.1	0.0	0.1	0	0	0
Brazil	Queimada Nova	1.4	1.1	1.8	2	1	2	0.2	0.1	0.2	0	0	0
Brazil	Queimadas	1.0	0.7	1.2	5	4	6	0.1	0.1	0.1	0	0	1
Brazil	Queimadas	1.7	1.4	2.2	5	4	7	0.2	0.2	0.3	1	0	1
Brazil	Queimados	0.2	0.1	0.3	2	2	3	0.1	0.0	0.1	1	0	1
Brazil	Queiroz	0.2	0.1	0.2	0	0	0	0.0	0.0	0.0	0	0	0
Brazil	Queluz	0.2	0.1	0.2	0	0	0	0.0	0.0	0.0	0	0	0
Brazil	Queluzita	0.3	0.2	0.4	0	0	0	0.1	0.0	0.1	0	0	0
Brazil	Querência	0.4	0.3	0.5	0	0	0	0.1	0.1	0.1	0	0	0
Brazil	Querência do Norte	0.3	0.2	0.3	0	0	0	0.1	0.0	0.1	0	0	0
Brazil	Quevedos	0.1	0.1	0.2	0	0	0	0.0	0.0	0.0	0	0	0
Brazil	Quijingue	1.7	1.4	2.2	6	5	8	0.2	0.2	0.3	1	0	1
Brazil	Quilombo	0.2	0.2	0.3	0	0	0	0.1	0.0	0.1	0	0	0
Brazil	Quinta do Sol	0.3	0.2	0.3	0	0	0	0.1	0.0	0.1	0	0	0
Brazil	Quinze de Novembro	0.1	0.1	0.2	0	0	0	0.0	0.0	0.0	0	0	0
Brazil	Quipapá	2.0	1.6	2.6	6	5	8	0.2	0.2	0.3	1	0	1
Brazil	Quirinópolis	0.2	0.1	0.2	1	0	1	0.1	0.0	0.1	0	0	0
Brazil	Quissamã	0.2	0.1	0.3	0	0	0	0.1	0.0	0.1	0	0	0
Brazil	Quitana	0.2	0.1	0.2	0	0	0	0.0	0.0	0.0	0	0	0
Brazil	Quitandinha	0.3	0.2	0.3	0	0	1	0.1	0.0	0.1	0	0	0
Brazil	Quiterianópolis	2.3	1.7	2.9	5	4	6	0.1	0.1	0.2	0	0	0
Brazil	Quixabá	2.0	1.6	2.6	3	2	3	0.2	0.2	0.3	0	0	0
Brazil	Quixabá	1.0	0.7	1.2	0	0	0	0.1	0.1	0.1	0	0	0
Brazil	Quixabeira	1.7	1.4	2.2	2	2	3	0.2	0.2	0.3	0	0	0
Brazil	Quixada	2.3	1.7	2.9	21	16	27	0.1	0.1	0.2	1	1	2
Brazil	Quixelô	2.3	1.7	2.9	3	2	3	0.1	0.1	0.2	0	0	0
Brazil	Quixeramobim	2.3	1.7	2.9	17	13	21	0.1	0.1	0.2	1	1	2
Brazil	Quixeré	2.3	1.7	2.9	5	4	6	0.1	0.1	0.2	0	0	0
Brazil	Rafael Fernandes	1.2	0.9	1.5	1	1	1	0.1	0.1	0.2	0	0	0
Brazil	Rafael Godeiro	1.2	0.9	1.5	0	0	0	0.1	0.1	0.2	0	0	0
Brazil	Rafael Jambeiro	1.7	1.4	2.2	4	3	5	0.2	0.2	0.3	0	0	1

Table 3: Diarrhoea Mortality by Unit *(continued)*

Country	Unit	2000 mortality mean rate (per 10,000)	2000 mortality lower rate (per 10,000)	2000 mortality upper rate (per 10,000)	2000 mortality mean count	2000 mortality lower count	2000 mortality upper count	2017 mortality mean rate (per 10,000)	2017 mortality lower rate (per 10,000)	2017 mortality upper rate (per 10,000)	2017 mortality mean count	2017 mortality lower count	2017 mortality upper count
Brazil	Rafard	0.2	0.1	0.2	0	0	0	0.0	0.0	0.0	0	0	0
Brazil	Ramilândia	0.3	0.2	0.3	0	0	0	0.1	0.0	0.1	0	0	0
Brazil	Rancharia	0.2	0.1	0.2	0	0	0	0.0	0.0	0.0	0	0	0
Brazil	Rancho Alegre	0.3	0.2	0.3	0	0	0	0.1	0.0	0.1	0	0	0
Brazil	Rancho Alegre d'Oeste	0.3	0.2	0.3	0	0	0	0.1	0.0	0.1	0	0	0
Brazil	Rancho Queimado	0.2	0.2	0.3	0	0	0	0.1	0.0	0.1	0	0	0
Brazil	Raposa	1.7	1.3	2.2	3	3	5	0.2	0.2	0.3	1	0	1
Brazil	Raposos	0.3	0.2	0.4	0	0	0	0.1	0.0	0.1	0	0	0
Brazil	Raul Soares	0.3	0.2	0.4	1	0	1	0.1	0.0	0.1	0	0	0
Brazil	Realeza	0.3	0.2	0.3	0	0	1	0.1	0.0	0.1	0	0	0
Brazil	Rebouças	0.3	0.2	0.3	0	0	0	0.1	0.0	0.1	0	0	0
Brazil	Recife	2.0	1.6	2.6	244	192	319	0.2	0.2	0.3	23	17	29
Brazil	Recreio	0.3	0.2	0.4	0	0	0	0.1	0.0	0.1	0	0	0
Brazil	Recursolândia	0.8	0.7	1.1	0	0	0	0.1	0.1	0.2	0	0	0
Brazil	Redenção	0.9	0.7	1.1	7	5	8	0.1	0.1	0.2	1	1	1
Brazil	Redenção	2.3	1.7	2.9	6	5	8	0.1	0.1	0.2	0	0	1
Brazil	Redenção da Serra	0.2	0.1	0.2	0	0	0	0.0	0.0	0.0	0	0	0
Brazil	Redenção do Gurguéia	1.4	1.1	1.8	1	1	2	0.2	0.1	0.2	0	0	0
Brazil	Redentora	0.1	0.1	0.2	0	0	0	0.0	0.0	0.0	0	0	0
Brazil	Reduto	0.3	0.2	0.4	0	0	0	0.1	0.0	0.1	0	0	0
Brazil	Regeneração	1.4	1.1	1.8	3	2	3	0.2	0.1	0.2	0	0	0
Brazil	Regente Feijó	0.2	0.1	0.2	0	0	0	0.0	0.0	0.0	0	0	0
Brazil	Reginópolis	0.2	0.1	0.2	0	0	0	0.0	0.0	0.0	0	0	0
Brazil	Registro	0.2	0.1	0.2	1	1	1	0.0	0.0	0.0	0	0	0
Brazil	Relvado	0.1	0.1	0.2	0	0	0	0.0	0.0	0.0	0	0	0
Brazil	Remanso	1.7	1.4	2.2	8	6	10	0.2	0.2	0.3	1	1	1
Brazil	Remígio	1.0	0.7	1.2	1	1	1	0.1	0.1	0.1	0	0	0
Brazil	Renascença	0.3	0.2	0.3	0	0	0	0.1	0.0	0.1	0	0	0
Brazil	Reriutaba	2.3	1.7	2.9	6	5	8	0.1	0.1	0.2	0	0	0
Brazil	Resende	0.2	0.1	0.3	2	1	3	0.1	0.0	0.1	1	0	1
Brazil	Resende Costa	0.3	0.2	0.4	0	0	0	0.1	0.0	0.1	0	0	0
Brazil	Reserva	0.3	0.2	0.3	1	1	1	0.1	0.0	0.1	0	0	0
Brazil	Reserva do Cabaçal	0.4	0.3	0.5	0	0	0	0.1	0.1	0.1	0	0	0
Brazil	Reserva do Iguaçu	0.3	0.2	0.3	0	0	0	0.1	0.0	0.1	0	0	0
Brazil	Resplendor	0.3	0.2	0.4	0	0	0	0.1	0.0	0.1	0	0	0
Brazil	Ressaquinha	0.3	0.2	0.4	0	0	0	0.1	0.0	0.1	0	0	0
Brazil	Restinga	0.2	0.1	0.2	0	0	0	0.0	0.0	0.0	0	0	0
Brazil	Restinga Seca	0.1	0.1	0.2	0	0	0	0.0	0.0	0.0	0	0	0
Brazil	Retirolândia	1.7	1.4	2.2	2	2	3	0.2	0.2	0.3	0	0	0
Brazil	Riachão	1.7	1.3	2.2	5	4	7	0.2	0.2	0.3	0	0	1
Brazil	Riachão das Neves	1.7	1.4	2.2	4	3	6	0.2	0.2	0.3	1	0	1
Brazil	Riachão do Bacamarte	1.0	0.7	1.2	0	0	1	0.1	0.1	0.1	0	0	0
Brazil	Riachao do dantas	1.3	1.0	1.7	3	2	4	0.2	0.1	0.3	0	0	0
Brazil	Riachao do Jacuipe	1.7	1.4	2.2	6	4	7	0.2	0.2	0.3	1	1	1
Brazil	Riachão do Poço	1.0	0.7	1.2	0	0	0	0.1	0.1	0.1	0	0	0
Brazil	Riachinho	0.8	0.7	1.1	0	0	0	0.1	0.1	0.2	0	0	0
Brazil	Riachinho	0.3	0.2	0.4	0	0	0	0.1	0.0	0.1	0	0	0
Brazil	Riacho	1.0	0.7	1.2	0	0	0	0.1	0.1	0.1	0	0	0
Brazil	Riacho da Cruz	1.2	0.9	1.5	1	0	1	0.1	0.1	0.2	0	0	0
Brazil	Riacho das Almas	2.0	1.6	2.6	3	3	5	0.2	0.2	0.3	0	0	0

Table 3: Diarrhoea Mortality by Unit *(continued)*

		2000 mortality						2017 mortality					
Country	Unit	mean rate (per 10,000)	lower rate (per 10,000)	upper rate (per 10,000)	mean count	lower count	upper count	mean rate (per 10,000)	lower rate (per 10,000)	upper rate (per 10,000)	mean count	lower count	upper count
Brazil	Riacho de Santana	1.2	0.9	1.5	1	0	1	0.1	0.1	0.2	0	0	0
Brazil	Riacho de Santana	1.7	1.4	2.2	6	5	8	0.2	0.2	0.3	1	1	1
Brazil	Riacho de Santo Antônio	1.0	0.7	1.2	0	0	0	0.1	0.1	0.1	0	0	0
Brazil	Riacho dos Machados	0.3	0.2	0.4	0	0	0	0.1	0.0	0.1	0	0	0
Brazil	Riacho Frio	1.4	1.1	1.8	1	1	1	0.2	0.1	0.2	0	0	0
Brazil	Riachuelo	1.2	0.9	1.5	1	1	1	0.1	0.1	0.2	0	0	0
Brazil	Riachuelo	1.3	1.0	1.7	1	1	1	0.2	0.1	0.3	0	0	0
Brazil	Rialma	0.2	0.1	0.2	0	0	0	0.1	0.0	0.1	0	0	0
Brazil	Rianápolis	0.2	0.1	0.2	0	0	0	0.1	0.0	0.1	0	0	0
Brazil	Ribamar Fiquene	1.7	1.3	2.2	2	1	2	0.2	0.2	0.3	0	0	0
Brazil	Ribas do Rio Pardo	0.5	0.3	0.6	1	1	1	0.2	0.1	0.2	0	0	0
Brazil	Ribeira	0.2	0.1	0.2	0	0	0	0.0	0.0	0.0	0	0	0
Brazil	Ribeira do Amparo	1.7	1.4	2.2	3	2	4	0.2	0.2	0.3	0	0	0
Brazil	Ribeira do Piauí	1.4	1.1	1.8	1	1	1	0.2	0.1	0.2	0	0	0
Brazil	Ribeira do Pombal	1.7	1.4	2.2	10	8	13	0.2	0.2	0.3	1	1	2
Brazil	Ribeirão	2.0	1.6	2.6	12	9	16	0.2	0.2	0.3	1	1	1
Brazil	Ribeirão Bonito	0.2	0.1	0.2	0	0	0	0.0	0.0	0.0	0	0	0
Brazil	Ribeirão Branco	0.2	0.1	0.2	0	0	0	0.0	0.0	0.0	0	0	0
Brazil	Ribeirão Cascalheira	0.4	0.3	0.5	0	0	1	0.1	0.1	0.1	0	0	0
Brazil	Ribeirão Claro	0.3	0.2	0.3	0	0	0	0.1	0.0	0.1	0	0	0
Brazil	Ribeirão Corrente	0.2	0.1	0.2	0	0	0	0.0	0.0	0.0	0	0	0
Brazil	Ribeirão das Neves	0.3	0.2	0.4	6	4	7	0.1	0.0	0.1	1	1	1
Brazil	Ribeirão do Largo	1.7	1.4	2.2	4	3	6	0.2	0.2	0.3	0	0	0
Brazil	Ribeirão do Pinhal	0.3	0.2	0.3	0	0	0	0.1	0.0	0.1	0	0	0
Brazil	Ribeirão do Sul	0.2	0.1	0.2	0	0	0	0.0	0.0	0.0	0	0	0
Brazil	Ribeirão dos índios	0.2	0.1	0.2	0	0	0	0.0	0.0	0.0	0	0	0
Brazil	Ribeirão Grande	0.2	0.1	0.2	0	0	0	0.0	0.0	0.0	0	0	0
Brazil	Ribeirão Pires	0.2	0.1	0.2	3	2	3	0.0	0.0	0.0	1	0	1
Brazil	Ribeirao Preto	0.2	0.1	0.2	6	4	7	0.0	0.0	0.0	1	1	2
Brazil	Ribeirão Vermelho	0.3	0.2	0.4	0	0	0	0.1	0.0	0.1	0	0	0
Brazil	Ribeirãozinho	0.4	0.3	0.5	0	0	0	0.1	0.1	0.1	0	0	0
Brazil	Ribeiro Gonçalves	1.4	1.1	1.8	1	1	1	0.2	0.1	0.2	0	0	0
Brazil	Ribeirópolis	1.3	1.0	1.7	2	2	3	0.2	0.1	0.3	0	0	0
Brazil	Ricaho dos Cavalos	1.0	0.7	1.2	1	1	1	0.1	0.1	0.1	0	0	0
Brazil	Rifaina	0.2	0.1	0.2	0	0	0	0.0	0.0	0.0	0	0	0
Brazil	Rincão	0.2	0.1	0.2	0	0	0	0.0	0.0	0.0	0	0	0
Brazil	Rinópolis	0.2	0.1	0.2	0	0	0	0.0	0.0	0.0	0	0	0
Brazil	Rio Acima	0.3	0.2	0.4	0	0	0	0.1	0.0	0.1	0	0	0
Brazil	Rio Azul	0.3	0.2	0.3	0	0	0	0.1	0.0	0.1	0	0	0
Brazil	Rio Bananal	0.3	0.2	0.3	0	0	1	0.1	0.1	0.1	0	0	0
Brazil	Rio Bom	0.3	0.2	0.3	0	0	0	0.1	0.0	0.1	0	0	0
Brazil	Rio Bonito	0.2	0.1	0.3	1	1	1	0.1	0.0	0.1	0	0	0
Brazil	Rio Bonito do Iguaçu	0.3	0.2	0.3	0	0	0	0.1	0.0	0.1	0	0	0
Brazil	Rio Branco	0.4	0.3	0.5	0	0	0	0.1	0.1	0.1	0	0	0

Table 3: Diarrhoea Mortality by Unit *(continued)*

Country	Unit	2000 mortality mean rate (per 10,000)	lower rate (per 10,000)	upper rate (per 10,000)	mean count	lower count	upper count	2017 mortality mean rate (per 10,000)	lower rate (per 10,000)	upper rate (per 10,000)	mean count	lower count	upper count
Brazil	Rio Branco	1.1	0.9	1.3	30	25	36	0.2	0.2	0.3	6	5	8
Brazil	Rio Branco do Ivaí	0.3	0.2	0.3	0	0	0	0.1	0.0	0.1	0	0	0
Brazil	Rio Branco do Sul	0.3	0.2	0.3	1	1	1	0.1	0.0	0.1	0	0	0
Brazil	Rio Brilhante	0.5	0.3	0.6	1	1	1	0.2	0.1	0.2	0	0	1
Brazil	Rio Casca	0.3	0.2	0.4	0	0	0	0.1	0.0	0.1	0	0	0
Brazil	Rio Claro	0.2	0.1	0.2	2	1	2	0.0	0.0	0.0	0	0	1
Brazil	Rio Claro	0.2	0.1	0.3	0	0	0	0.1	0.0	0.1	0	0	0
Brazil	Rio Crespo	0.4	0.3	0.5	0	0	0	0.1	0.1	0.1	0	0	0
Brazil	Rio da Conceição	0.8	0.7	1.1	0	0	0	0.1	0.1	0.2	0	0	0
Brazil	Rio das Antas	0.2	0.2	0.3	0	0	0	0.1	0.0	0.1	0	0	0
Brazil	Rio das Flores	0.2	0.1	0.3	0	0	0	0.1	0.0	0.1	0	0	0
Brazil	Rio das Ostras	0.2	0.1	0.3	1	0	1	0.1	0.0	0.1	1	1	1
Brazil	Rio das Pedras	0.2	0.1	0.2	0	0	0	0.0	0.0	0.0	0	0	0
Brazil	Rio de Contas	1.7	1.4	2.2	2	2	3	0.2	0.2	0.3	0	0	0
Brazil	Rio de Janeiro	0.2	0.1	0.3	83	62	111	0.1	0.0	0.1	21	16	27
Brazil	Rio do Antônio	1.7	1.4	2.2	3	2	4	0.2	0.2	0.3	0	0	0
Brazil	Rio do Campo	0.2	0.2	0.3	0	0	0	0.1	0.0	0.1	0	0	0
Brazil	Rio do Fogo	1.2	0.9	1.5	1	1	1	0.1	0.1	0.2	0	0	0
Brazil	Rio do Oeste	0.2	0.2	0.3	0	0	0	0.1	0.0	0.1	0	0	0
Brazil	Rio do Pires	1.7	1.4	2.2	2	1	2	0.2	0.2	0.3	0	0	0
Brazil	Rio do Prado	0.3	0.2	0.4	0	0	0	0.1	0.0	0.1	0	0	0
Brazil	Rio do Sul	0.2	0.2	0.3	1	1	1	0.1	0.0	0.1	0	0	0
Brazil	Rio doce	0.3	0.2	0.4	0	0	0	0.1	0.0	0.1	0	0	0
Brazil	Rio dos Bois	0.8	0.7	1.1	0	0	0	0.1	0.1	0.2	0	0	0
Brazil	Rio dos Cedros	0.2	0.2	0.3	0	0	0	0.1	0.0	0.1	0	0	0
Brazil	Rio dos índios	0.1	0.1	0.2	0	0	0	0.0	0.0	0.0	0	0	0
Brazil	Rio Espera	0.3	0.2	0.4	0	0	0	0.1	0.0	0.1	0	0	0
Brazil	Rio Formoso	2.0	1.6	2.6	5	4	7	0.2	0.2	0.3	1	0	1
Brazil	Rio Fortuna	0.2	0.2	0.3	0	0	0	0.1	0.0	0.1	0	0	0
Brazil	Rio Grande	0.1	0.1	0.2	2	1	2	0.0	0.0	0.0	0	0	0
Brazil	Rio Grande da Serra	0.2	0.1	0.2	1	0	1	0.0	0.0	0.0	0	0	0
Brazil	Rio Grande do Piauí	1.4	1.1	1.8	1	1	1	0.2	0.1	0.2	0	0	0
Brazil	Rio Largo	2.8	2.2	3.7	25	19	33	0.3	0.2	0.4	3	2	3
Brazil	Rio Manso	0.3	0.2	0.4	0	0	0	0.1	0.0	0.1	0	0	0
Brazil	Rio Maria	0.9	0.7	1.1	2	1	2	0.1	0.1	0.2	0	0	0
Brazil	Rio Negrinho	0.2	0.2	0.3	1	1	1	0.1	0.0	0.1	0	0	0
Brazil	Rio Negro	0.3	0.2	0.3	1	1	1	0.1	0.0	0.1	0	0	0
Brazil	Rio Negro	0.5	0.3	0.6	0	0	0	0.2	0.1	0.2	0	0	0
Brazil	Rio Novo	0.3	0.2	0.4	0	0	0	0.1	0.0	0.1	0	0	0
Brazil	Rio Novo do Sul	0.3	0.2	0.3	0	0	0	0.1	0.1	0.1	0	0	0
Brazil	Rio Paranaiba	0.3	0.2	0.4	0	0	0	0.1	0.0	0.1	0	0	0
Brazil	Rio Paranaíba	0.3	0.2	0.4	1	1	1	0.1	0.0	0.1	0	0	0
Brazil	Rio Pardo	0.1	0.1	0.2	0	0	0	0.0	0.0	0.0	0	0	0
Brazil	Rio Pardo de Minas	0.3	0.2	0.4	0	0	0	0.1	0.0	0.1	0	0	0
Brazil	Rio Pomba	0.3	0.2	0.4	0	0	0	0.1	0.0	0.1	0	0	0
Brazil	Rio Preto	0.3	0.2	0.4	0	0	0	0.1	0.0	0.1	0	0	0
Brazil	Rio Preto da Eva	0.8	0.6	0.9	2	1	2	0.2	0.2	0.3	1	1	1
Brazil	Rio Quente	0.2	0.1	0.2	0	0	0	0.1	0.0	0.1	0	0	0
Brazil	Rio Real	1.7	1.4	2.2	8	6	10	0.2	0.2	0.3	1	1	1
Brazil	Rio Rufino	0.2	0.2	0.3	0	0	0	0.1	0.0	0.1	0	0	0
Brazil	Rio Sono	0.8	0.7	1.1	1	0	1	0.1	0.1	0.2	0	0	0
Brazil	Rio Tinto	1.0	0.7	1.2	4	3	5	0.1	0.1	0.1	0	0	0
Brazil	Rio Verde	0.2	0.1	0.2	2	2	3	0.1	0.0	0.1	1	1	1
Brazil	Rio Verde de Mato Grosso	0.5	0.3	0.6	1	1	1	0.2	0.1	0.2	0	0	0

Table 3: Diarrhoea Mortality by Unit *(continued)*

Country	Unit	2000 mortality mean rate (per 10,000)	lower rate (per 10,000)	upper rate (per 10,000)	mean count	lower count	upper count	2017 mortality mean rate (per 10,000)	lower rate (per 10,000)	upper rate (per 10,000)	mean count	lower count	upper count
Brazil	Rio Vermelho	0.3	0.2	0.4	0	0	1	0.1	0.0	0.1	0	0	0
Brazil	Riolândia	0.2	0.1	0.2	0	0	0	0.0	0.0	0.0	0	0	0
Brazil	Riozinho	0.1	0.1	0.2	0	0	0	0.0	0.0	0.0	0	0	0
Brazil	Riqueza	0.2	0.2	0.3	0	0	0	0.1	0.0	0.1	0	0	0
Brazil	Ritápolis	0.3	0.2	0.4	0	0	0	0.1	0.0	0.1	0	0	0
Brazil	Riversul	0.2	0.1	0.2	0	0	0	0.0	0.0	0.0	0	0	0
Brazil	Roca Sales	0.1	0.1	0.2	0	0	0	0.0	0.0	0.0	0	0	0
Brazil	Rochedo	0.5	0.3	0.6	0	0	0	0.2	0.1	0.2	0	0	0
Brazil	Rochedo de Minas	0.3	0.2	0.4	0	0	0	0.1	0.0	0.1	0	0	0
Brazil	Rodeio	0.2	0.2	0.3	0	0	0	0.1	0.0	0.1	0	0	0
Brazil	Rodeio Bonito	0.1	0.1	0.2	0	0	0	0.0	0.0	0.0	0	0	0
Brazil	Rodeiro	0.3	0.2	0.4	0	0	0	0.1	0.0	0.1	0	0	0
Brazil	Rodelas	1.7	1.4	2.2	2	1	2	0.2	0.2	0.3	0	0	0
Brazil	Rodolfo Fernandes	1.2	0.9	1.5	1	0	1	0.1	0.1	0.2	0	0	0
Brazil	Rodrigues Alves	1.1	0.9	1.3	1	1	1	0.2	0.2	0.3	0	0	0
Brazil	Rolândia	0.3	0.2	0.3	1	1	1	0.1	0.0	0.1	0	0	0
Brazil	Rolante	0.1	0.1	0.2	0	0	0	0.0	0.0	0.0	0	0	0
Brazil	Rolim de Moura	0.4	0.3	0.5	2	2	2	0.1	0.1	0.1	0	0	0
Brazil	Romaria	0.3	0.2	0.4	0	0	0	0.1	0.0	0.1	0	0	0
Brazil	Romelândia	0.2	0.2	0.3	0	0	0	0.1	0.0	0.1	0	0	0
Brazil	Roncador	0.3	0.2	0.3	0	0	0	0.1	0.0	0.1	0	0	0
Brazil	Ronda Alta	0.1	0.1	0.2	0	0	0	0.0	0.0	0.0	0	0	0
Brazil	Rondinha	0.1	0.1	0.2	0	0	0	0.0	0.0	0.0	0	0	0
Brazil	Rondon	0.3	0.2	0.3	0	0	0	0.1	0.0	0.1	0	0	0
Brazil	Rondon do Pará	0.9	0.7	1.1	4	3	5	0.1	0.1	0.2	1	1	1
Brazil	Rondonópolis	0.4	0.3	0.5	6	5	7	0.1	0.1	0.1	2	1	2
Brazil	Roque Gonzales	0.1	0.1	0.2	0	0	0	0.0	0.0	0.0	0	0	0
Brazil	Rorainópolis	0.7	0.6	0.9	2	1	2	0.2	0.2	0.3	1	1	1
Brazil	Rosana	0.2	0.1	0.2	0	0	0	0.0	0.0	0.0	0	0	0
Brazil	Rosário	1.7	1.3	2.2	8	6	10	0.2	0.2	0.3	1	1	1
Brazil	Rosário da Limeira	0.3	0.2	0.4	0	0	0	0.1	0.0	0.1	0	0	0
Brazil	Rosário do Catete	1.3	1.0	1.7	2	1	2	0.2	0.1	0.3	0	0	0
Brazil	Rosário do Ivaí	0.3	0.2	0.3	0	0	0	0.1	0.0	0.1	0	0	0
Brazil	Rosário do Sul	0.1	0.1	0.2	0	0	1	0.0	0.0	0.0	0	0	0
Brazil	Rosário Oeste	0.4	0.3	0.5	1	1	1	0.1	0.1	0.1	0	0	0
Brazil	Roseira	0.2	0.1	0.2	0	0	0	0.0	0.0	0.0	0	0	0
Brazil	Roteiro	2.8	2.2	3.7	3	2	4	0.3	0.2	0.4	0	0	0
Brazil	Rubelita	0.3	0.2	0.4	0	0	0	0.1	0.0	0.1	0	0	0
Brazil	Rubiácea	0.2	0.1	0.2	0	0	0	0.0	0.0	0.0	0	0	0
Brazil	Rubiataba	0.2	0.1	0.2	0	0	0	0.1	0.0	0.1	0	0	0
Brazil	Rubim	0.3	0.2	0.4	0	0	0	0.1	0.0	0.1	0	0	0
Brazil	Rubinéia	0.2	0.1	0.2	0	0	0	0.0	0.0	0.0	0	0	0
Brazil	Rurópolis	0.9	0.7	1.1	3	2	3	0.1	0.1	0.2	1	1	1
Brazil	Russas	2.3	1.7	2.9	14	11	18	0.1	0.1	0.2	1	1	1
Brazil	Ruy Barbosa	1.2	0.9	1.5	0	0	1	0.1	0.1	0.2	0	0	0
Brazil	Ruy Barbosa	1.7	1.4	2.2	7	5	8	0.2	0.2	0.3	1	1	1
Brazil	Sabará	0.3	0.2	0.4	4	3	6	0.1	0.0	0.1	1	1	1
Brazil	Sabáudia	0.3	0.2	0.3	0	0	0	0.1	0.0	0.1	0	0	0
Brazil	Sabino	0.2	0.1	0.2	0	0	0	0.0	0.0	0.0	0	0	0
Brazil	Sabinópolis	0.3	0.2	0.4	1	0	1	0.1	0.0	0.1	0	0	0
Brazil	Saboeiro	2.3	1.7	2.9	4	3	6	0.1	0.1	0.2	0	0	0
Brazil	Sacramento	0.3	0.2	0.4	0	0	1	0.1	0.0	0.1	0	0	0
Brazil	Sagrada Família	0.1	0.1	0.2	0	0	0	0.0	0.0	0.0	0	0	0
Brazil	Sagres	0.2	0.1	0.2	0	0	0	0.0	0.0	0.0	0	0	0
Brazil	Sairé	2.0	1.6	2.6	6	5	8	0.2	0.2	0.3	0	0	1
Brazil	Saldanha Marinho	0.1	0.1	0.2	0	0	0	0.0	0.0	0.0	0	0	0

Table 3: Diarrhoea Mortality by Unit *(continued)*

Country	Unit	2000 mortality mean rate (per 10,000)	lower rate (per 10,000)	upper rate (per 10,000)	mean count	lower count	upper count	2017 mortality mean rate (per 10,000)	lower rate (per 10,000)	upper rate (per 10,000)	mean count	lower count	upper count
Brazil	Sales	0.2	0.1	0.2	0	0	0	0.0	0.0	0.0	0	0	0
Brazil	Sales Oliveira	0.2	0.1	0.2	0	0	0	0.0	0.0	0.0	0	0	0
Brazil	Salesópolis	0.2	0.1	0.2	0	0	0	0.0	0.0	0.0	0	0	0
Brazil	Salete	0.2	0.2	0.3	0	0	0	0.1	0.0	0.1	0	0	0
Brazil	Salgadinho	2.0	1.6	2.6	2	1	2	0.2	0.2	0.3	0	0	0
Brazil	Salgadinho	1.0	0.7	1.2	0	0	0	0.1	0.1	0.1	0	0	0
Brazil	Salgado	1.3	1.0	1.7	3	2	4	0.2	0.1	0.3	0	0	0
Brazil	Salgado de São Félix	1.0	0.7	1.2	1	1	1	0.1	0.1	0.1	0	0	0
Brazil	Salgado Filho	0.3	0.2	0.3	0	0	0	0.1	0.0	0.1	0	0	0
Brazil	Salgueiro	2.0	1.6	2.6	14	11	18	0.2	0.2	0.3	1	1	2
Brazil	Salidao	2.0	1.6	2.6	2	1	2	0.2	0.2	0.3	0	0	0
Brazil	Salinas	0.3	0.2	0.4	1	1	1	0.1	0.0	0.1	0	0	0
Brazil	Salinas da Margarida	1.7	1.4	2.2	1	1	2	0.2	0.2	0.3	0	0	0
Brazil	Salinópolis	0.9	0.7	1.1	3	2	3	0.1	0.1	0.2	0	0	1
Brazil	Salitre	2.3	1.7	2.9	4	3	6	0.1	0.1	0.2	0	0	0
Brazil	Salmourão	0.2	0.1	0.2	0	0	0	0.0	0.0	0.0	0	0	0
Brazil	Saloá	2.0	1.6	2.6	4	3	5	0.2	0.2	0.3	0	0	1
Brazil	Saltinho	0.2	0.2	0.3	0	0	0	0.1	0.0	0.1	0	0	0
Brazil	Saltinho	0.2	0.1	0.2	0	0	0	0.0	0.0	0.0	0	0	0
Brazil	Salto	0.2	0.1	0.2	1	1	1	0.0	0.0	0.0	0	0	0
Brazil	Salto da Divisa	0.3	0.2	0.4	0	0	0	0.1	0.0	0.1	0	0	0
Brazil	Salto do Céu	0.4	0.3	0.5	0	0	0	0.1	0.1	0.1	0	0	0
Brazil	Salto do Itararé	0.3	0.2	0.3	0	0	0	0.1	0.0	0.1	0	0	0
Brazil	Salto do Jacuí	0.1	0.1	0.2	0	0	0	0.0	0.0	0.0	0	0	0
Brazil	Salto do Londra	0.3	0.2	0.3	0	0	0	0.1	0.0	0.1	0	0	0
Brazil	Salto do Pirapora	0.2	0.1	0.2	1	0	1	0.0	0.0	0.0	0	0	0
Brazil	Salto Grande	0.2	0.1	0.2	0	0	0	0.0	0.0	0.0	0	0	0
Brazil	Salto Veloso	0.2	0.2	0.3	0	0	0	0.1	0.0	0.1	0	0	0
Brazil	Salvador	1.7	1.4	2.2	354	279	456	0.2	0.2	0.3	45	34	59
Brazil	Salvador das Missões	0.1	0.1	0.2	0	0	0	0.0	0.0	0.0	0	0	0
Brazil	Salvador do Sul	0.1	0.1	0.2	0	0	0	0.0	0.0	0.0	0	0	0
Brazil	Salvaterra	0.9	0.7	1.1	2	2	3	0.1	0.1	0.2	0	0	1
Brazil	Sambaíba	1.7	1.3	2.2	1	1	2	0.2	0.2	0.3	0	0	0
Brazil	Sampaio	0.8	0.7	1.1	0	0	0	0.1	0.1	0.2	0	0	0
Brazil	San Antonio do Itambe	0.3	0.2	0.4	0	0	0	0.1	0.0	0.1	0	0	0
Brazil	San Antonio do Rio Abai	0.3	0.2	0.4	0	0	0	0.1	0.0	0.1	0	0	0
Brazil	Sananduva	0.1	0.1	0.2	0	0	0	0.0	0.0	0.0	0	0	0
Brazil	Sanclerlândia	0.2	0.1	0.2	0	0	0	0.1	0.0	0.1	0	0	0
Brazil	Sandolândia	0.8	0.7	1.1	0	0	0	0.1	0.1	0.2	0	0	0
Brazil	Sandovalina	0.2	0.1	0.2	0	0	0	0.0	0.0	0.0	0	0	0
Brazil	Sangão	0.2	0.2	0.3	0	0	0	0.1	0.0	0.1	0	0	0
Brazil	Sanharó	2.0	1.6	2.6	4	3	5	0.2	0.2	0.3	1	0	1
Brazil	Santa Adélia	0.2	0.1	0.2	0	0	0	0.0	0.0	0.0	0	0	0
Brazil	Santa Albertina	0.2	0.1	0.2	0	0	0	0.0	0.0	0.0	0	0	0
Brazil	Santa Amélia	0.3	0.2	0.3	0	0	0	0.1	0.0	0.1	0	0	0
Brazil	Santa Bárbara	1.7	1.4	2.2	4	3	5	0.2	0.2	0.3	0	0	1
Brazil	Santa Bárbara	0.3	0.2	0.4	1	1	1	0.1	0.0	0.1	0	0	0
Brazil	Santa Bárbara d'Oeste	0.2	0.1	0.2	2	1	2	0.0	0.0	0.0	0	0	0
Brazil	Santa Bárbara de Goiás	0.2	0.1	0.2	0	0	0	0.1	0.0	0.1	0	0	0
Brazil	Santa Bárbara do Leste	0.3	0.2	0.4	0	0	0	0.1	0.0	0.1	0	0	0
Brazil	Santa Bárbara do Monte Verde	0.3	0.2	0.4	0	0	0	0.1	0.0	0.1	0	0	0

Table 3: Diarrhoea Mortality by Unit *(continued)*

Country	Unit	2000 mortality mean rate (per 10,000)	2000 lower rate (per 10,000)	2000 upper rate (per 10,000)	2000 mean count	2000 lower count	2000 upper count	2017 mortality mean rate (per 10,000)	2017 lower rate (per 10,000)	2017 upper rate (per 10,000)	2017 mean count	2017 lower count	2017 upper count
Brazil	Santa Bárbara do Pará	0.9	0.7	1.1	1	1	2	0.1	0.1	0.2	0	0	0
Brazil	Santa Bárbara do Sul	0.1	0.1	0.2	0	0	0	0.0	0.0	0.0	0	0	0
Brazil	Santa Bárbara do Tugúrio	0.3	0.2	0.4	0	0	0	0.1	0.0	0.1	0	0	0
Brazil	Santa Branca	0.2	0.1	0.2	0	0	0	0.0	0.0	0.0	0	0	0
Brazil	Santa Brígida	1.7	1.4	2.2	3	3	4	0.2	0.2	0.3	0	0	0
Brazil	Santa Carmem	0.4	0.3	0.5	0	0	0	0.1	0.1	0.1	0	0	0
Brazil	Santa Cecília	1.0	0.7	1.2	1	1	1	0.1	0.1	0.1	0	0	0
Brazil	Santa Cecília	0.2	0.2	0.3	0	0	1	0.1	0.0	0.1	0	0	0
Brazil	Santa Cecília do Pavão	0.3	0.2	0.3	0	0	0	0.1	0.0	0.1	0	0	0
Brazil	Santa Clara do Oeste	0.2	0.1	0.2	0	0	0	0.0	0.0	0.0	0	0	0
Brazil	Santa Clara do Sul	0.1	0.1	0.2	0	0	0	0.0	0.0	0.0	0	0	0
Brazil	Santa Cruz	1.2	0.9	1.5	5	4	7	0.1	0.1	0.2	1	0	1
Brazil	Santa Cruz	2.0	1.6	2.6	4	3	5	0.2	0.2	0.3	0	0	0
Brazil	Santa Cruz Cabrália	1.7	1.4	2.2	5	4	6	0.2	0.2	0.3	1	0	1
Brazil	Santa Cruz da Baixa Verde	2.0	1.6	2.6	2	2	3	0.2	0.2	0.3	0	0	0
Brazil	Santa Cruz da Conceição	0.2	0.1	0.2	0	0	0	0.0	0.0	0.0	0	0	0
Brazil	Santa Cruz da Esperança	0.2	0.1	0.2	0	0	0	0.0	0.0	0.0	0	0	0
Brazil	Santa Cruz da Vitória	1.7	1.4	2.2	1	1	1	0.2	0.2	0.3	0	0	0
Brazil	Santa Cruz das Palmeiras	0.2	0.1	0.2	0	0	0	0.0	0.0	0.0	0	0	0
Brazil	Santa Cruz de Goiás	0.2	0.1	0.2	0	0	0	0.1	0.0	0.1	0	0	0
Brazil	Santa Cruz de Minas	0.3	0.2	0.4	0	0	0	0.1	0.0	0.1	0	0	0
Brazil	Santa Cruz de Monte Caste	0.3	0.2	0.3	0	0	0	0.1	0.0	0.1	0	0	0
Brazil	Santa Cruz de Salinas	0.3	0.2	0.4	0	0	0	0.1	0.0	0.1	0	0	0
Brazil	Santa Cruz do Arari	0.9	0.7	1.1	0	0	0	0.1	0.1	0.2	0	0	0
Brazil	Santa Cruz do Capibaribe	2.0	1.6	2.6	10	8	13	0.2	0.2	0.3	2	1	2
Brazil	Santa Cruz do Escalvado	0.3	0.2	0.4	0	0	0	0.1	0.0	0.1	0	0	0
Brazil	Santa Cruz do Piaui	1.4	1.1	1.8	1	1	1	0.2	0.1	0.2	0	0	0
Brazil	Santa Cruz do Rio Pardo	0.2	0.1	0.2	1	0	1	0.0	0.0	0.0	0	0	0
Brazil	Santa Cruz do Sul	0.1	0.1	0.2	1	1	1	0.0	0.0	0.0	0	0	0
Brazil	Santa Cruz dos Milagres	1.4	1.1	1.8	0	0	1	0.2	0.1	0.2	0	0	0
Brazil	Santa Efigênia de Minas	0.3	0.2	0.4	0	0	0	0.1	0.0	0.1	0	0	0
Brazil	Santa Ernestina	0.2	0.1	0.2	0	0	0	0.0	0.0	0.0	0	0	0
Brazil	Santa Fé	0.3	0.2	0.3	0	0	0	0.1	0.0	0.1	0	0	0
Brazil	Santa Fé de Goiás	1.7	1.3	2.2	1	1	1	0.2	0.2	0.3	0	0	0
Brazil	Santa Fé de Minas	0.3	0.2	0.4	0	0	0	0.1	0.0	0.1	0	0	0

Table 3: Diarrhoea Mortality by Unit *(continued)*

Country	Unit	2000 mortality mean rate (per 10,000)	lower rate (per 10,000)	upper rate (per 10,000)	mean count	lower count	upper count	2017 mortality mean rate (per 10,000)	lower rate (per 10,000)	upper rate (per 10,000)	mean count	lower count	upper count
Brazil	Santa Fé do Araguaia	0.8	0.7	1.1	1	0	1	0.1	0.1	0.2	0	0	0
Brazil	Santa Fé do Sul	0.2	0.1	0.2	0	0	0	0.0	0.0	0.0	0	0	0
Brazil	Santa Filomena	2.0	1.6	2.6	4	3	5	0.2	0.2	0.3	0	0	0
Brazil	Santa Filomena	1.4	1.1	1.8	2	2	3	0.2	0.1	0.2	0	0	0
Brazil	Santa Filomena do Maranhão	0.2	0.1	0.2	0	0	0	0.1	0.0	0.1	0	0	0
Brazil	Santa Gertrudes	0.2	0.1	0.2	0	0	0	0.0	0.0	0.0	0	0	0
Brazil	Santa Helena	1.7	1.3	2.2	6	5	8	0.2	0.2	0.3	1	1	1
Brazil	Santa Helena	1.0	0.7	1.2	1	0	1	0.1	0.1	0.1	0	0	0
Brazil	Santa Helena	0.2	0.2	0.3	0	0	0	0.1	0.0	0.1	0	0	0
Brazil	Santa Helena	0.3	0.2	0.3	0	0	1	0.1	0.0	0.1	0	0	0
Brazil	Santa Helena de Goiás	0.2	0.1	0.2	1	0	1	0.1	0.0	0.1	0	0	0
Brazil	Santa Helena de Minas	0.3	0.2	0.4	0	0	0	0.1	0.0	0.1	0	0	0
Brazil	Santa Inês	1.7	1.3	2.2	13	10	17	0.2	0.2	0.3	2	1	2
Brazil	Santa Inês	1.0	0.7	1.2	0	0	1	0.1	0.1	0.1	0	0	0
Brazil	Santa Inês	0.3	0.2	0.3	0	0	0	0.1	0.0	0.1	0	0	0
Brazil	Santa Inês	1.7	1.4	2.2	2	2	3	0.2	0.2	0.3	0	0	0
Brazil	Santa Isabel	0.2	0.1	0.2	0	0	0	0.1	0.0	0.1	0	0	0
Brazil	Santa Isabel	0.2	0.1	0.2	1	1	1	0.0	0.0	0.0	0	0	0
Brazil	Santa Isabel do Ivaí	0.3	0.2	0.3	0	0	0	0.1	0.0	0.1	0	0	0
Brazil	Santa Isabel do Oeste	0.3	0.2	0.3	0	0	0	0.1	0.0	0.1	0	0	0
Brazil	Santa Isabel do Pará	0.9	0.7	1.1	5	4	6	0.1	0.1	0.2	1	1	1
Brazil	Santa Isabel do Rio Negro	0.8	0.6	0.9	1	1	1	0.2	0.2	0.3	1	1	1
Brazil	Santa Juliana	0.3	0.2	0.4	0	0	0	0.1	0.0	0.1	0	0	0
Brazil	Santa Leopoldina	0.3	0.2	0.3	0	0	0	0.1	0.1	0.1	0	0	0
Brazil	Santa Lucia	0.2	0.1	0.2	0	0	0	0.0	0.0	0.0	0	0	0
Brazil	Santa Lúcia	0.3	0.2	0.3	0	0	0	0.1	0.0	0.1	0	0	0
Brazil	Santa Luz	1.4	1.1	1.8	1	1	1	0.2	0.1	0.2	0	0	0
Brazil	Santa Luzia	1.7	1.3	2.2	18	14	24	0.2	0.2	0.3	2	2	3
Brazil	Santa Luzia	1.0	0.7	1.2	1	1	2	0.1	0.1	0.1	0	0	0
Brazil	Santa Luzia	1.7	1.4	2.2	4	3	5	0.2	0.2	0.3	0	0	0
Brazil	Santa Luzia	0.3	0.2	0.4	5	4	6	0.1	0.0	0.1	1	1	1
Brazil	Santa Luzia d'Oeste	0.4	0.3	0.5	1	0	1	0.1	0.1	0.1	0	0	0
Brazil	Santa Luzia do Itanhy	1.3	1.0	1.7	2	2	3	0.2	0.1	0.3	0	0	0
Brazil	Santa Luzia do Norte	2.8	2.2	3.7	2	2	3	0.3	0.2	0.4	0	0	0
Brazil	Santa Luzia do Pará	0.9	0.7	1.1	2	2	3	0.1	0.1	0.2	0	0	0
Brazil	Santa Luzia do Paruá	1.7	1.3	2.2	5	4	7	0.2	0.2	0.3	1	1	1
Brazil	Santa Margarida	0.3	0.2	0.4	0	0	1	0.1	0.0	0.1	0	0	0
Brazil	Santa Maria	1.2	0.9	1.5	0	0	1	0.1	0.1	0.2	0	0	0
Brazil	Santa Maria	0.1	0.1	0.2	2	2	3	0.0	0.0	0.0	0	0	1
Brazil	Santa Maria da Boa Vista	2.0	1.6	2.6	11	9	15	0.2	0.2	0.3	1	1	1
Brazil	Santa Maria da Serra	0.2	0.1	0.2	0	0	0	0.0	0.0	0.0	0	0	0
Brazil	Santa Maria da Vitória	1.7	1.4	2.2	7	6	9	0.2	0.2	0.3	1	1	1
Brazil	Santa Maria das Barreiras	0.9	0.7	1.1	1	1	1	0.1	0.1	0.2	0	0	0

Table 3: Diarrhoea Mortality by Unit *(continued)*

Country	Unit	2000 mortality mean rate (per 10,000)	lower rate (per 10,000)	upper rate (per 10,000)	mean count	lower count	upper count	2017 mortality mean rate (per 10,000)	lower rate (per 10,000)	upper rate (per 10,000)	mean count	lower count	upper count
Brazil	Santa Maria de Itabira	0.3	0.2	0.4	0	0	0	0.1	0.0	0.1	0	0	0
Brazil	Santa Maria de Jetibá	0.3	0.2	0.3	1	1	1	0.1	0.1	0.1	0	0	0
Brazil	Santa Maria do Cambucá	2.0	1.6	2.6	3	2	4	0.2	0.2	0.3	0	0	0
Brazil	Santa Maria do Herval	0.1	0.1	0.2	0	0	0	0.0	0.0	0.0	0	0	0
Brazil	Santa Maria do Oeste	0.3	0.2	0.3	0	0	0	0.1	0.0	0.1	0	0	0
Brazil	Santa Maria do Pará	0.9	0.7	1.1	2	1	2	0.1	0.1	0.2	0	0	0
Brazil	Santa Maria do Salto	0.3	0.2	0.4	0	0	0	0.1	0.0	0.1	0	0	0
Brazil	Santa Maria do Suaçuí	0.3	0.2	0.4	0	0	1	0.1	0.0	0.1	0	0	0
Brazil	Santa Maria do Tocantins	0.8	0.7	1.1	0	0	0	0.1	0.1	0.2	0	0	0
Brazil	Santa Maria Madalena	0.2	0.1	0.3	0	0	0	0.1	0.0	0.1	0	0	0
Brazil	Santa Mariana	0.3	0.2	0.3	0	0	0	0.1	0.0	0.1	0	0	0
Brazil	Santa Mercedes	0.2	0.1	0.2	0	0	0	0.0	0.0	0.0	0	0	0
Brazil	Santa Mônica	0.3	0.2	0.3	0	0	0	0.1	0.0	0.1	0	0	0
Brazil	Santa Quitéria	2.3	1.7	2.9	12	9	15	0.1	0.1	0.2	1	0	1
Brazil	Santa Quitéria do Maranhão	1.7	1.3	2.2	9	7	11	0.2	0.2	0.3	1	1	1
Brazil	Santa Rita	1.7	1.3	2.2	7	5	9	0.2	0.2	0.3	1	1	1
Brazil	Santa Rita	1.0	0.7	1.2	13	10	17	0.1	0.1	0.1	1	1	1
Brazil	Santa Rita de Araguaia	0.2	0.1	0.2	0	0	0	0.1	0.0	0.1	0	0	0
Brazil	Santa Rita de Caldas	0.3	0.2	0.4	0	0	0	0.1	0.0	0.1	0	0	0
Brazil	Santa Rita de Cássia	1.7	1.4	2.2	6	4	7	0.2	0.2	0.3	1	0	1
Brazil	Santa Rita de Jacutinga	0.3	0.2	0.4	0	0	0	0.1	0.0	0.1	0	0	0
Brazil	Santa Rita de Minas	0.3	0.2	0.4	0	0	0	0.1	0.0	0.1	0	0	0
Brazil	Santa Rita do Ibitipoca	0.3	0.2	0.4	1	1	1	0.1	0.0	0.1	0	0	0
Brazil	Santa Rita do Novo destino	0.2	0.1	0.2	0	0	0	0.1	0.0	0.1	0	0	0
Brazil	Santa Rita do Oeste	0.2	0.1	0.2	0	0	0	0.0	0.0	0.0	0	0	0
Brazil	Santa Rita do Pardo	0.5	0.3	0.6	0	0	1	0.2	0.1	0.2	0	0	0
Brazil	Santa Rita do Passa Quatro	0.2	0.1	0.2	0	0	0	0.0	0.0	0.0	0	0	0
Brazil	Santa Rita do Sapucaí	0.3	0.2	0.4	0	0	0	0.1	0.0	0.1	0	0	0
Brazil	Santa Rita do Tocantins	0.8	0.7	1.1	0	0	0	0.1	0.1	0.2	0	0	0
Brazil	Santa Rita Itueto	0.3	0.2	0.4	0	0	0	0.1	0.0	0.1	0	0	0
Brazil	Santa Rosa	0.1	0.1	0.2	1	0	1	0.0	0.0	0.0	0	0	0
Brazil	Santa Rosa da Serra	0.3	0.2	0.4	0	0	0	0.1	0.0	0.1	0	0	0
Brazil	Santa Rosa de Goiás	0.2	0.1	0.2	0	0	0	0.1	0.0	0.1	0	0	0
Brazil	Santa Rosa de Lima	0.2	0.2	0.3	0	0	0	0.1	0.0	0.1	0	0	0

Table 3: Diarrhoea Mortality by Unit *(continued)*

Country	Unit	2000 mortality mean rate (per 10,000)	2000 mortality lower rate (per 10,000)	2000 mortality upper rate (per 10,000)	2000 mortality mean count	2000 mortality lower count	2000 mortality upper count	2017 mortality mean rate (per 10,000)	2017 mortality lower rate (per 10,000)	2017 mortality upper rate (per 10,000)	2017 mortality mean count	2017 mortality lower count	2017 mortality upper count
Brazil	Santa Rosa de Lima	1.3	1.0	1.7	0	0	0	0.2	0.1	0.3	0	0	0
Brazil	Santa Rosa de Viterbo	0.2	0.1	0.2	0	0	0	0.0	0.0	0.0	0	0	0
Brazil	Santa Rosa do Piauí	1.4	1.1	1.8	1	1	1	0.2	0.1	0.2	0	0	0
Brazil	Santa Rosa do Purus	1.1	0.9	1.3	0	0	0	0.2	0.2	0.3	0	0	0
Brazil	Santa Rosa do Sul	0.2	0.2	0.3	0	0	0	0.1	0.0	0.1	0	0	0
Brazil	Santa Rosa do Tocantins	0.8	0.7	1.1	0	0	1	0.1	0.1	0.2	0	0	0
Brazil	Santa Salete	0.2	0.1	0.2	0	0	0	0.0	0.0	0.0	0	0	0
Brazil	Santa Teresa	0.3	0.2	0.3	1	0	1	0.1	0.1	0.1	0	0	0
Brazil	Santa Teresinha	1.0	0.7	1.2	1	0	1	0.1	0.1	0.1	0	0	0
Brazil	Santa Teresinha	1.7	1.4	2.2	2	1	2	0.2	0.2	0.3	0	0	0
Brazil	Santa Tereza	0.1	0.1	0.2	0	0	0	0.0	0.0	0.0	0	0	0
Brazil	Santa Tereza de Goiás	0.2	0.1	0.2	0	0	0	0.1	0.0	0.1	0	0	0
Brazil	Santa Tereza do Oeste	0.3	0.2	0.3	0	0	0	0.1	0.0	0.1	0	0	0
Brazil	Santa Tereza do Tocantins	0.8	0.7	1.1	0	0	0	0.1	0.1	0.2	0	0	0
Brazil	Santa Terezinha	0.4	0.3	0.5	0	0	0	0.1	0.1	0.1	0	0	0
Brazil	Santa Terezinha	2.0	1.6	2.6	2	2	3	0.2	0.2	0.3	0	0	0
Brazil	Santa Terezinha	0.2	0.2	0.3	0	0	0	0.1	0.0	0.1	0	0	0
Brazil	Santa Terezinha de Goiás	0.2	0.1	0.2	0	0	0	0.1	0.0	0.1	0	0	0
Brazil	Santa Terezinha de Itaipu	0.3	0.2	0.3	1	0	1	0.1	0.0	0.1	0	0	0
Brazil	Santa Terezinha do Progresso	0.2	0.2	0.3	0	0	0	0.1	0.0	0.1	0	0	0
Brazil	Santa Terezinha do Tocantins	0.8	0.7	1.1	0	0	0	0.1	0.1	0.2	0	0	0
Brazil	Santa Vitória	0.3	0.2	0.4	0	0	0	0.1	0.0	0.1	0	0	0
Brazil	Santa Vitória do Palmar	0.1	0.1	0.2	0	0	0	0.0	0.0	0.0	0	0	0
Brazil	Santaluz	1.7	1.4	2.2	6	5	8	0.2	0.2	0.3	1	1	1
Brazil	Santana	1.2	0.9	1.5	0	0	0	0.1	0.1	0.2	0	0	0
Brazil	Santana	0.2	0.2	0.3	2	1	2	0.1	0.1	0.2	1	1	1
Brazil	Santana	1.7	1.4	2.2	6	4	7	0.2	0.2	0.3	1	1	1
Brazil	Santana da Boa Vista	0.1	0.1	0.2	0	0	0	0.0	0.0	0.0	0	0	0
Brazil	Santana da Ponte Pensa	0.2	0.1	0.2	0	0	0	0.0	0.0	0.0	0	0	0
Brazil	Santana da Vargem	0.3	0.2	0.4	0	0	0	0.1	0.0	0.1	0	0	0
Brazil	Santana de Cataguases	0.3	0.2	0.4	0	0	0	0.1	0.0	0.1	0	0	0
Brazil	Santana de Mangueira	1.0	0.7	1.2	1	1	1	0.1	0.1	0.1	0	0	0
Brazil	Santana de Parnaíba	0.2	0.1	0.2	2	1	2	0.0	0.0	0.0	0	0	1
Brazil	Santana de Pirapama	0.3	0.2	0.4	0	0	0	0.1	0.0	0.1	0	0	0
Brazil	Santana do Acaraú	2.3	1.7	2.9	9	7	11	0.1	0.1	0.2	1	0	1
Brazil	Santana do Araguaia	0.9	0.7	1.1	3	2	4	0.1	0.1	0.2	1	1	1
Brazil	Santana do Cariri	2.3	1.7	2.9	4	3	6	0.1	0.1	0.2	0	0	0

Table 3: Diarrhoea Mortality by Unit *(continued)*

Country	Unit	2000 mortality						2017 mortality					
		mean rate (per 10,000)	lower rate (per 10,000)	upper rate (per 10,000)	mean count	lower count	upper count	mean rate (per 10,000)	lower rate (per 10,000)	upper rate (per 10,000)	mean count	lower count	upper count
Brazil	Santana do deserto	0.3	0.2	0.4	0	0	0	0.1	0.0	0.1	0	0	0
Brazil	Santana do Garambéu	0.3	0.2	0.4	0	0	0	0.1	0.0	0.1	0	0	0
Brazil	Santana do Ipanema	2.8	2.2	3.7	18	14	23	0.3	0.2	0.4	2	1	2
Brazil	Santana do Itararé	0.3	0.2	0.3	0	0	0	0.1	0.0	0.1	0	0	0
Brazil	Santana do Jacaré	0.3	0.2	0.4	0	0	0	0.1	0.0	0.1	0	0	0
Brazil	Santana do Livramento	0.1	0.1	0.2	1	1	1	0.0	0.0	0.0	0	0	0
Brazil	Santana do Manhuaçu	0.3	0.2	0.4	0	0	0	0.1	0.0	0.1	0	0	0
Brazil	Santana do Maranhão	1.7	1.3	2.2	4	3	5	0.2	0.2	0.3	0	0	1
Brazil	Santana do Matos	1.2	0.9	1.5	2	2	3	0.1	0.1	0.2	0	0	0
Brazil	Santana do Mundaú	2.8	2.2	3.7	4	3	5	0.3	0.2	0.4	0	0	0
Brazil	Santana do Paraíso	0.3	0.2	0.4	1	1	1	0.1	0.0	0.1	0	0	0
Brazil	Santana do Piauí	1.4	1.1	1.8	1	1	1	0.2	0.1	0.2	0	0	0
Brazil	Santana do Riacho	0.3	0.2	0.4	0	0	0	0.1	0.0	0.1	0	0	0
Brazil	Santana do São Francisco	1.3	1.0	1.7	1	0	1	0.2	0.1	0.3	0	0	0
Brazil	Santana dos Garrotes	1.0	0.7	1.2	1	1	1	0.1	0.1	0.1	0	0	0
Brazil	Santana dos Montes	0.3	0.2	0.4	0	0	0	0.1	0.0	0.1	0	0	0
Brazil	Santanópolis	1.7	1.4	2.2	2	2	3	0.2	0.2	0.3	0	0	0
Brazil	Santarém	0.9	0.7	1.1	27	21	33	0.1	0.1	0.2	4	3	5
Brazil	Santarém	1.0	0.7	1.2	0	0	0	0.1	0.1	0.1	0	0	0
Brazil	Santarém Novo	0.9	0.7	1.1	1	0	1	0.1	0.1	0.2	0	0	0
Brazil	Santiago	0.1	0.1	0.2	1	0	1	0.0	0.0	0.0	0	0	0
Brazil	Santiago do Sul	0.2	0.2	0.3	0	0	0	0.1	0.0	0.1	0	0	0
Brazil	Santo Afonso	0.4	0.3	0.5	0	0	0	0.1	0.1	0.1	0	0	0
Brazil	Santo Amaro	1.7	1.4	2.2	8	6	10	0.2	0.2	0.3	1	1	1
Brazil	Santo Amaro da Imperatriz	0.2	0.2	0.3	0	0	0	0.1	0.0	0.1	0	0	0
Brazil	Santo Amaro das Brotas	1.3	1.0	1.7	2	2	3	0.2	0.1	0.3	0	0	0
Brazil	Santo Amaro do Maranhão	1.7	1.3	2.2	3	2	4	0.2	0.2	0.3	1	0	1
Brazil	Santo Anastácio	0.2	0.1	0.2	0	0	0	0.0	0.0	0.0	0	0	0
Brazil	Santo André	1.0	0.7	1.2	0	0	0	0.1	0.1	0.1	0	0	0
Brazil	Santo André	0.2	0.1	0.2	8	6	10	0.0	0.0	0.0	2	1	2
Brazil	Santo Ángelo	0.1	0.1	0.2	1	1	1	0.0	0.0	0.0	0	0	0
Brazil	Santo Antônio	1.2	0.9	1.5	4	3	5	0.1	0.1	0.2	0	0	0
Brazil	Santo Antônio da Alegria	0.2	0.1	0.2	0	0	0	0.0	0.0	0.0	0	0	0
Brazil	Santo Antônio da Barra	0.2	0.1	0.2	0	0	0	0.1	0.0	0.1	0	0	0
Brazil	Santo Antônio da Patrulha	0.1	0.1	0.2	0	0	0	0.0	0.0	0.0	0	0	0
Brazil	Santo Antonio da Platina	0.3	0.2	0.3	1	1	1	0.1	0.0	0.1	0	0	0
Brazil	Santo Antônio das Missões	0.1	0.1	0.2	0	0	0	0.0	0.0	0.0	0	0	0
Brazil	Santo Antônio de Goiás	0.2	0.1	0.2	0	0	0	0.1	0.0	0.1	0	0	0

Table 3: Diarrhoea Mortality by Unit *(continued)*

Country	Unit	2000 mortality mean rate (per 10,000)	lower rate (per 10,000)	upper rate (per 10,000)	mean count	lower count	upper count	2017 mortality mean rate (per 10,000)	lower rate (per 10,000)	upper rate (per 10,000)	mean count	lower count	upper count
Brazil	Santo Antônio de Jesus	1.7	1.4	2.2	14	11	18	0.2	0.2	0.3	2	2	3
Brazil	Santo Antônio de Lisboa	1.4	1.1	1.8	0	0	0	0.2	0.1	0.2	0	0	0
Brazil	Santo Antônio de Pádua	0.2	0.1	0.3	1	0	1	0.1	0.0	0.1	0	0	0
Brazil	Santo Antônio de Posse	0.2	0.1	0.2	0	0	0	0.0	0.0	0.0	0	0	0
Brazil	Santo Antônio do Amparo	0.3	0.2	0.4	1	0	1	0.1	0.0	0.1	0	0	0
Brazil	Santo Antônio do Aracanguá	0.2	0.1	0.2	0	0	0	0.0	0.0	0.0	0	0	0
Brazil	Santo Antônio do Aventureiro	0.3	0.2	0.4	0	0	0	0.1	0.0	0.1	0	0	0
Brazil	Santo Antonio do Caiuá	0.3	0.2	0.3	0	0	0	0.1	0.0	0.1	0	0	0
Brazil	Santo Antônio do Descoberto	0.2	0.1	0.2	1	1	1	0.1	0.0	0.1	0	0	0
Brazil	Santo Antônio do Grama	0.3	0.2	0.4	0	0	0	0.1	0.0	0.1	0	0	0
Brazil	Santo Antônio do Içá	0.8	0.6	0.9	3	3	4	0.2	0.2	0.3	1	1	1
Brazil	Santo Antônio do Jacinto	0.3	0.2	0.4	0	0	0	0.1	0.0	0.1	0	0	0
Brazil	Santo Antônio do Leverger	0.4	0.3	0.5	1	1	1	0.1	0.1	0.1	0	0	0
Brazil	Santo Antônio do Monte	0.3	0.2	0.4	0	0	0	0.1	0.0	0.1	0	0	0
Brazil	Santo Antônio do Palma	0.1	0.1	0.2	0	0	0	0.0	0.0	0.0	0	0	0
Brazil	Santo Antonio do Paraíso	0.3	0.2	0.3	0	0	0	0.1	0.0	0.1	0	0	0
Brazil	Santo Antônio do Pinhal	0.2	0.1	0.2	0	0	0	0.0	0.0	0.0	0	0	0
Brazil	Santo Antônio do Planalto	0.1	0.1	0.2	0	0	0	0.0	0.0	0.0	0	0	0
Brazil	Santo Antônio do Retiro	0.3	0.2	0.4	1	0	1	0.1	0.0	0.1	0	0	0
Brazil	Santo Antônio do Tauá	0.9	0.7	1.1	2	1	2	0.1	0.1	0.2	0	0	1
Brazil	Santo Antônio dos Lopes	1.7	1.3	2.2	3	3	4	0.2	0.2	0.3	0	0	1
Brazil	Santo Antônio dos Milagres	1.4	1.1	1.8	1	1	1	0.2	0.1	0.2	0	0	0
Brazil	Santo Augusto	0.1	0.1	0.2	0	0	0	0.0	0.0	0.0	0	0	0
Brazil	Santo Cristo	0.1	0.1	0.2	0	0	0	0.0	0.0	0.0	0	0	0
Brazil	Santo Estêvão	1.7	1.4	2.2	9	7	12	0.2	0.2	0.3	1	1	2
Brazil	Santo Expedito	0.2	0.1	0.2	0	0	0	0.0	0.0	0.0	0	0	0
Brazil	Santo Expedito do Sul	0.1	0.1	0.2	0	0	0	0.0	0.0	0.0	0	0	0
Brazil	Santo Hipólito	0.3	0.2	0.4	0	0	0	0.1	0.0	0.1	0	0	0
Brazil	Santo Inácio	0.3	0.2	0.3	0	0	0	0.1	0.0	0.1	0	0	0
Brazil	Santo Inácio do Piauí	1.4	1.1	1.8	1	0	1	0.2	0.1	0.2	0	0	0
Brazil	Santópolis do Aguapeí	0.2	0.1	0.2	0	0	0	0.0	0.0	0.0	0	0	0
Brazil	Santos	0.2	0.1	0.2	4	3	5	0.0	0.0	0.0	1	1	1
Brazil	Santos Dumont	0.3	0.2	0.4	1	1	1	0.1	0.0	0.1	0	0	0
Brazil	São Antonio de Sudoeste	0.3	0.2	0.3	0	0	1	0.1	0.0	0.1	0	0	0

Table 3: Diarrhoea Mortality by Unit *(continued)*

Country	Unit	2000 mortality mean rate (per 10,000)	lower rate (per 10,000)	upper rate (per 10,000)	mean count	lower count	upper count	2017 mortality mean rate (per 10,000)	lower rate (per 10,000)	upper rate (per 10,000)	mean count	lower count	upper count
Brazil	São Benedito	2.3	1.7	2.9	9	7	12	0.1	0.1	0.2	1	0	1
Brazil	São Benedito do Rio Preto	1.7	1.3	2.2	5	4	6	0.2	0.2	0.3	1	0	1
Brazil	São Benedito do Sul	2.0	1.6	2.6	3	2	4	0.2	0.2	0.3	0	0	0
Brazil	São Bentinho	1.0	0.7	1.2	0	0	0	0.1	0.1	0.1	0	0	0
Brazil	São Bento	1.7	1.3	2.2	6	5	8	0.2	0.2	0.3	1	1	1
Brazil	São Bento	1.0	0.7	1.2	3	2	4	0.1	0.1	0.1	0	0	0
Brazil	São Bento Abade	0.3	0.2	0.4	0	0	0	0.1	0.0	0.1	0	0	0
Brazil	São Bento do Norte	1.2	0.9	1.5	1	1	1	0.1	0.1	0.2	0	0	0
Brazil	São Bento do Sapucaí	0.2	0.1	0.2	0	0	0	0.0	0.0	0.0	0	0	0
Brazil	São Bento do Sul	0.2	0.2	0.3	1	1	2	0.1	0.0	0.1	0	0	0
Brazil	São Bento do Tocantins	0.8	0.7	1.1	0	0	0	0.1	0.1	0.2	0	0	0
Brazil	São Bento do Trairí	1.2	0.9	1.5	1	0	1	0.1	0.1	0.2	0	0	0
Brazil	São Bento do Una	2.0	1.6	2.6	12	10	16	0.2	0.2	0.3	1	1	2
Brazil	São Bernardino	0.2	0.2	0.3	0	0	0	0.1	0.0	0.1	0	0	0
Brazil	São Bernardo	1.7	1.3	2.2	7	5	9	0.2	0.2	0.3	1	1	1
Brazil	São Bernardo do Campo	0.2	0.1	0.2	10	7	12	0.0	0.0	0.0	2	2	3
Brazil	São Bonifácio	0.2	0.2	0.3	0	0	0	0.1	0.0	0.1	0	0	0
Brazil	São Borja	0.1	0.1	0.2	1	1	1	0.0	0.0	0.0	0	0	0
Brazil	São Brás	2.8	2.2	3.7	2	2	3	0.3	0.2	0.4	0	0	0
Brazil	São Brás do Suaçuí	0.3	0.2	0.4	0	0	0	0.1	0.0	0.1	0	0	0
Brazil	São Braz do Piauí	1.4	1.1	1.8	1	1	1	0.2	0.1	0.2	0	0	0
Brazil	São Caetano de Odivelas	0.9	0.7	1.1	2	1	2	0.1	0.1	0.2	0	0	0
Brazil	São Caetano do Sul	0.2	0.1	0.2	2	1	2	0.0	0.0	0.0	0	0	0
Brazil	São Caitano	2.0	1.6	2.6	9	7	12	0.2	0.2	0.3	1	1	1
Brazil	São Carlos	0.2	0.2	0.3	0	0	0	0.1	0.0	0.1	0	0	0
Brazil	São Carlos	0.2	0.1	0.2	2	2	3	0.0	0.0	0.0	1	0	1
Brazil	São Carlos do Ivaí	0.3	0.2	0.3	0	0	0	0.1	0.0	0.1	0	0	0
Brazil	São Cristóvão	1.3	1.0	1.7	12	9	15	0.2	0.1	0.3	2	1	3
Brazil	São Cristóvão do Sul	0.2	0.2	0.3	0	0	0	0.1	0.0	0.1	0	0	0
Brazil	São Desidério	1.7	1.4	2.2	4	3	6	0.2	0.2	0.3	1	1	1
Brazil	São Domingos	0.2	0.1	0.2	0	0	0	0.1	0.0	0.1	0	0	0
Brazil	São Domingos	0.2	0.2	0.3	0	0	0	0.1	0.0	0.1	0	0	0
Brazil	São Domingos	1.7	1.4	2.2	1	1	2	0.2	0.2	0.3	0	0	0
Brazil	São Domingos	1.3	1.0	1.7	1	1	2	0.2	0.1	0.3	0	0	0
Brazil	São Domingos das Dores	0.3	0.2	0.4	0	0	0	0.1	0.0	0.1	0	0	0
Brazil	São Domingos de Pombal	1.0	0.7	1.2	0	0	0	0.1	0.1	0.1	0	0	0
Brazil	São Domingos do Araguaia	0.9	0.7	1.1	2	2	3	0.1	0.1	0.2	0	0	0
Brazil	São Domingos do Azeitão	1.7	1.3	2.2	1	1	2	0.2	0.2	0.3	0	0	0
Brazil	São Domingos do Capim	0.9	0.7	1.1	3	3	4	0.1	0.1	0.2	1	0	1
Brazil	São Domingos do Cariri	1.0	0.7	1.2	0	0	0	0.1	0.1	0.1	0	0	0
Brazil	São Domingos do Maranhão	1.7	1.3	2.2	8	6	10	0.2	0.2	0.3	1	1	1

Table 3: Diarrhoea Mortality by Unit *(continued)*

Country	Unit	2000 mortality						2017 mortality					
		mean rate (per 10,000)	lower rate (per 10,000)	upper rate (per 10,000)	mean count	lower count	upper count	mean rate (per 10,000)	lower rate (per 10,000)	upper rate (per 10,000)	mean count	lower count	upper count
Brazil	São Domingos do Norte	0.3	0.2	0.3	0	0	0	0.1	0.1	0.1	0	0	0
Brazil	São Domingos do Prata	0.3	0.2	0.4	0	0	0	0.1	0.0	0.1	0	0	0
Brazil	São Domingos do Sul	0.1	0.1	0.2	0	0	0	0.0	0.0	0.0	0	0	0
Brazil	São Felipe	1.7	1.4	2.2	3	3	4	0.2	0.2	0.3	0	0	0
Brazil	São Felipe d'Oeste	0.4	0.3	0.5	0	0	0	0.1	0.1	0.1	0	0	0
Brazil	São Félix	1.7	1.4	2.2	3	2	3	0.2	0.2	0.3	0	0	0
Brazil	São Félix de Balsas	1.7	1.3	2.2	1	1	1	0.2	0.2	0.3	0	0	0
Brazil	São Félix de Minas	0.3	0.2	0.4	0	0	0	0.1	0.0	0.1	0	0	0
Brazil	São Félix do Coribe	1.7	1.4	2.2	3	3	4	0.2	0.2	0.3	0	0	1
Brazil	São Félix do Piauí	1.4	1.1	1.8	1	0	1	0.2	0.1	0.2	0	0	0
Brazil	São Félix do Tocantins	0.8	0.7	1.1	0	0	0	0.1	0.1	0.2	0	0	0
Brazil	São Félix do Xingu	0.9	0.7	1.1	4	3	4	0.1	0.1	0.2	2	2	3
Brazil	São Félix Xingu	0.4	0.3	0.5	0	0	1	0.1	0.1	0.1	0	0	0
Brazil	São Fernando	1.2	0.9	1.5	0	0	1	0.1	0.1	0.2	0	0	0
Brazil	São Fidélis	0.2	0.1	0.3	1	0	1	0.1	0.0	0.1	0	0	0
Brazil	São Francisco	1.0	0.7	1.2	0	0	0	0.1	0.1	0.1	0	0	0
Brazil	São Francisco	0.2	0.1	0.2	0	0	0	0.0	0.0	0.0	0	0	0
Brazil	São Francisco	1.3	1.0	1.7	1	0	1	0.2	0.1	0.3	0	0	0
Brazil	São Francisco	0.3	0.2	0.4	2	1	2	0.1	0.0	0.1	0	0	0
Brazil	São Francisco de Assis	0.1	0.1	0.2	0	0	0	0.0	0.0	0.0	0	0	0
Brazil	São Francisco de Assis do Piauí	1.4	1.1	1.8	1	0	1	0.2	0.1	0.2	0	0	0
Brazil	São Francisco de Goias	0.2	0.1	0.2	0	0	0	0.1	0.0	0.1	0	0	0
Brazil	São Francisco de Itabapoana	0.2	0.1	0.3	1	0	1	0.1	0.0	0.1	0	0	0
Brazil	São Francisco de Oliveira	0.3	0.2	0.4	0	0	0	0.1	0.0	0.1	0	0	0
Brazil	São Francisco de Paula	0.1	0.1	0.2	0	0	0	0.0	0.0	0.0	0	0	0
Brazil	São Francisco de Sales	0.3	0.2	0.4	0	0	0	0.1	0.0	0.1	0	0	0
Brazil	São Francisco do Brejão	1.7	1.3	2.2	1	1	2	0.2	0.2	0.3	0	0	0
Brazil	São Francisco do Conde	1.7	1.4	2.2	7	6	9	0.2	0.2	0.3	1	1	1
Brazil	São Francisco do Glória	0.3	0.2	0.4	0	0	0	0.1	0.0	0.1	0	0	0
Brazil	São Francisco do Guaporé	0.4	0.3	0.5	1	1	1	0.1	0.1	0.1	0	0	0
Brazil	São Francisco do Maranhão	1.7	1.3	2.2	3	3	4	0.2	0.2	0.3	0	0	1
Brazil	São Francisco do Oeste	1.2	0.9	1.5	0	0	0	0.1	0.1	0.2	0	0	0
Brazil	São Francisco do Pará	0.9	0.7	1.1	1	1	1	0.1	0.1	0.2	0	0	0
Brazil	São Francisco do Piauí	1.4	1.1	1.8	1	1	1	0.2	0.1	0.2	0	0	0
Brazil	São Francisco do Sul	0.2	0.2	0.3	1	1	1	0.1	0.0	0.1	0	0	0

Table 3: Diarrhoea Mortality by Unit *(continued)*

Country	Unit	2000 mortality mean rate (per 10,000)	lower rate (per 10,000)	upper rate (per 10,000)	mean count	lower count	upper count	2017 mortality mean rate (per 10,000)	lower rate (per 10,000)	upper rate (per 10,000)	mean count	lower count	upper count
Brazil	São Gabriel	1.7	1.4	2.2	4	3	5	0.2	0.2	0.3	0	0	1
Brazil	São Gabriel	0.1	0.1	0.2	1	0	1	0.0	0.0	0.0	0	0	0
Brazil	São Gabriel da Palha	0.3	0.2	0.3	1	0	1	0.1	0.1	0.1	0	0	0
Brazil	São Gabriel de Cahoeira	0.8	0.6	0.9	3	2	4	0.2	0.2	0.3	1	1	1
Brazil	São Gabriel do Oeste	0.5	0.3	0.6	1	1	1	0.2	0.1	0.2	0	0	0
Brazil	São Geraldo	0.3	0.2	0.4	0	0	0	0.1	0.0	0.1	0	0	0
Brazil	São Geraldo da Piedade	0.3	0.2	0.4	0	0	0	0.1	0.0	0.1	0	0	0
Brazil	São Geraldo do Araguaia	0.9	0.7	1.1	3	2	3	0.1	0.1	0.2	0	0	0
Brazil	São Geraldo do Baixio	0.3	0.2	0.4	0	0	0	0.1	0.0	0.1	0	0	0
Brazil	São Gonçalo	0.2	0.1	0.3	14	10	19	0.1	0.0	0.1	3	3	5
Brazil	São Gonçalo do Abaeté	0.3	0.2	0.4	0	0	0	0.1	0.0	0.1	0	0	0
Brazil	São Gonçalo do Amarante	1.2	0.9	1.5	16	12	21	0.1	0.1	0.2	2	1	2
Brazil	São Gonçalo do Amarante	2.3	1.7	2.9	9	7	12	0.1	0.1	0.2	1	1	1
Brazil	São Gonçalo do Gurguéia	1.4	1.1	1.8	1	0	1	0.2	0.1	0.2	0	0	0
Brazil	São Gonçalo do Pará	0.3	0.2	0.4	0	0	0	0.1	0.0	0.1	0	0	0
Brazil	São Gonçalo do Piauí	1.4	1.1	1.8	1	1	1	0.2	0.1	0.2	0	0	0
Brazil	São Gonçalo do Rio Abaixo	0.3	0.2	0.4	0	0	0	0.1	0.0	0.1	0	0	0
Brazil	São Gonçalo do Sapucaí	0.3	0.2	0.4	1	0	1	0.1	0.0	0.1	0	0	0
Brazil	São Gonçalo dos Campos	1.7	1.4	2.2	6	5	8	0.2	0.2	0.3	1	1	1
Brazil	São Gotardo	0.3	0.2	0.4	1	1	1	0.1	0.0	0.1	0	0	0
Brazil	São Jerônimo	0.1	0.1	0.2	0	0	0	0.0	0.0	0.0	0	0	0
Brazil	São Jerônimo da Serra	0.3	0.2	0.3	0	0	0	0.1	0.0	0.1	0	0	0
Brazil	São João	2.0	1.6	2.6	14	11	18	0.2	0.2	0.3	1	1	2
Brazil	São João	0.3	0.2	0.3	0	0	0	0.1	0.0	0.1	0	0	0
Brazil	São João Batista	1.7	1.3	2.2	5	4	6	0.2	0.2	0.3	1	0	1
Brazil	São João Batista	0.2	0.2	0.3	0	0	0	0.1	0.0	0.1	0	0	0
Brazil	São João Batista do Glória	0.3	0.2	0.4	0	0	0	0.1	0.0	0.1	0	0	0
Brazil	São João d'Aliança	0.2	0.1	0.2	0	0	0	0.1	0.0	0.1	0	0	0
Brazil	São João da Baliza	0.7	0.6	0.9	1	0	1	0.2	0.2	0.3	0	0	0
Brazil	São João da Barra	0.2	0.1	0.3	0	0	0	0.1	0.0	0.1	0	0	0
Brazil	São João da Boa Vista	0.2	0.1	0.2	1	1	1	0.0	0.0	0.0	0	0	0
Brazil	São João da Canabrava	1.4	1.1	1.8	1	0	1	0.2	0.1	0.2	0	0	0
Brazil	São João da Fronteira	1.4	1.1	1.8	1	1	1	0.2	0.1	0.2	0	0	0
Brazil	São João da Lagoa	0.3	0.2	0.4	0	0	0	0.1	0.0	0.1	0	0	0
Brazil	São João da Mata	0.3	0.2	0.4	0	0	0	0.1	0.0	0.1	0	0	0
Brazil	São João da Paraúna	0.2	0.1	0.2	0	0	0	0.1	0.0	0.1	0	0	0

Table 3: Diarrhoea Mortality by Unit *(continued)*

Country	Unit	2000 mortality mean rate (per 10,000)	2000 mortality lower rate (per 10,000)	2000 mortality upper rate (per 10,000)	2000 mortality mean count	2000 mortality lower count	2000 mortality upper count	2017 mortality mean rate (per 10,000)	2017 mortality lower rate (per 10,000)	2017 mortality upper rate (per 10,000)	2017 mortality mean count	2017 mortality lower count	2017 mortality upper count
Brazil	São João da Ponta	0.9	0.7	1.1	0	0	1	0.1	0.1	0.2	0	0	0
Brazil	São João da Ponte	0.3	0.2	0.4	1	1	1	0.1	0.0	0.1	0	0	0
Brazil	São João da Serra	1.4	1.1	1.8	1	1	1	0.2	0.1	0.2	0	0	0
Brazil	São João da Urtiga	0.1	0.1	0.2	0	0	0	0.0	0.0	0.0	0	0	0
Brazil	São João da Varjota	1.4	1.1	1.8	1	1	1	0.2	0.1	0.2	0	0	0
Brazil	São João das Duas Ponte	0.2	0.1	0.2	0	0	0	0.0	0.0	0.0	0	0	0
Brazil	São João das Missões	0.3	0.2	0.4	0	0	0	0.1	0.0	0.1	0	0	0
Brazil	São João de Iracema	0.2	0.1	0.2	0	0	0	0.0	0.0	0.0	0	0	0
Brazil	São João de Meriti	0.2	0.1	0.3	7	5	9	0.1	0.0	0.1	1	1	2
Brazil	São João de Pirabas	0.9	0.7	1.1	2	1	2	0.1	0.1	0.2	0	0	0
Brazil	São João del Rei	0.3	0.2	0.4	2	1	2	0.1	0.0	0.1	0	0	0
Brazil	São João do Araguaia	0.9	0.7	1.1	1	1	2	0.1	0.1	0.2	0	0	0
Brazil	São João do Arraial	1.4	1.1	1.8	1	1	1	0.2	0.1	0.2	0	0	0
Brazil	São João do Belm	2.3	1.7	2.9	13	10	17	0.1	0.1	0.2	1	1	1
Brazil	São João do Belmonte	2.0	1.6	2.6	8	6	11	0.2	0.2	0.3	1	1	1
Brazil	São João do Caiuá	0.3	0.2	0.3	0	0	0	0.1	0.0	0.1	0	0	0
Brazil	São João do Cariri	1.0	0.7	1.2	0	0	1	0.1	0.1	0.1	0	0	0
Brazil	São João do Carú	1.7	1.3	2.2	3	2	3	0.2	0.2	0.3	0	0	0
Brazil	São João do Itaperiú	0.2	0.2	0.3	0	0	0	0.1	0.0	0.1	0	0	0
Brazil	São João do Ivaí	0.3	0.2	0.3	0	0	0	0.1	0.0	0.1	0	0	0
Brazil	São João do Jaguaribe	2.3	1.7	2.9	2	2	3	0.1	0.1	0.2	0	0	0
Brazil	São João do Manhuaçu	0.3	0.2	0.4	0	0	0	0.1	0.0	0.1	0	0	0
Brazil	São João do Manteninha	0.3	0.2	0.4	0	0	0	0.1	0.0	0.1	0	0	0
Brazil	São João do Oeste	0.2	0.2	0.3	0	0	0	0.1	0.0	0.1	0	0	0
Brazil	São João do Oriente	0.3	0.2	0.4	0	0	0	0.1	0.0	0.1	0	0	0
Brazil	São João do Pacuí	0.3	0.2	0.4	0	0	0	0.1	0.0	0.1	0	0	0
Brazil	São João do Paraíso	1.7	1.3	2.2	2	2	3	0.2	0.2	0.3	0	0	0
Brazil	São João do Paraíso	0.3	0.2	0.4	1	0	1	0.1	0.0	0.1	0	0	0
Brazil	São João do Pau d'Alho	0.2	0.1	0.2	0	0	0	0.0	0.0	0.0	0	0	0
Brazil	São João do Polêsine	0.1	0.1	0.2	0	0	0	0.0	0.0	0.0	0	0	0
Brazil	São João do Rio do Peixe	1.0	0.7	1.2	2	1	2	0.1	0.1	0.1	0	0	0
Brazil	São João do Sabugi	1.2	0.9	1.5	1	0	1	0.1	0.1	0.2	0	0	0
Brazil	São João do Soter	1.7	1.3	2.2	4	3	5	0.2	0.2	0.3	1	0	1

Table 3: Diarrhoea Mortality by Unit *(continued)*

Country	Unit	2000 mortality mean rate (per 10,000)	lower rate (per 10,000)	upper rate (per 10,000)	mean count	lower count	upper count	2017 mortality mean rate (per 10,000)	lower rate (per 10,000)	upper rate (per 10,000)	mean count	lower count	upper count
Brazil	São João do Sul	0.2	0.2	0.3	0	0	0	0.1	0.0	0.1	0	0	0
Brazil	São João do Tigre	1.0	0.7	1.2	0	0	1	0.1	0.1	0.1	0	0	0
Brazil	São João do Triunfo	0.3	0.2	0.3	0	0	0	0.1	0.0	0.1	0	0	0
Brazil	São João dos Patos	1.7	1.3	2.2	4	3	6	0.2	0.2	0.3	1	0	1
Brazil	São João Evangelista	0.3	0.2	0.4	0	0	1	0.1	0.0	0.1	0	0	0
Brazil	São João Nepomuceno	0.3	0.2	0.4	0	0	1	0.1	0.0	0.1	0	0	0
Brazil	São João Piaui	1.4	1.1	1.8	3	2	4	0.2	0.1	0.2	0	0	0
Brazil	São Joaquim	0.2	0.2	0.3	0	0	1	0.1	0.0	0.1	0	0	0
Brazil	São Joaquim da Barra	0.2	0.1	0.2	0	0	1	0.0	0.0	0.0	0	0	0
Brazil	São Joaquim de Bicas	0.3	0.2	0.4	1	1	1	0.1	0.0	0.1	0	0	0
Brazil	São Joaquin do Monte	2.0	1.6	2.6	6	5	8	0.2	0.2	0.3	0	0	1
Brazil	São Jorge	0.1	0.1	0.2	0	0	0	0.0	0.0	0.0	0	0	0
Brazil	São Jorge d'Oeste	0.3	0.2	0.3	0	0	0	0.1	0.0	0.1	0	0	0
Brazil	São Jorge do Ivaí	0.3	0.2	0.3	0	0	0	0.1	0.0	0.1	0	0	0
Brazil	São Jorge do Patrocínio	0.3	0.2	0.3	0	0	0	0.1	0.0	0.1	0	0	0
Brazil	São José	0.2	0.2	0.3	2	1	2	0.1	0.0	0.1	0	0	1
Brazil	São José da Barra	0.3	0.2	0.4	0	0	0	0.1	0.0	0.1	0	0	0
Brazil	São José da Bela Vista	0.2	0.1	0.2	0	0	0	0.0	0.0	0.0	0	0	0
Brazil	São José da Boa Vista	0.3	0.2	0.3	0	0	0	0.1	0.0	0.1	0	0	0
Brazil	São José da Coroa Grande	2.0	1.6	2.6	3	2	4	0.2	0.2	0.3	0	0	1
Brazil	São José da Lagoa Tapada	1.0	0.7	1.2	1	1	1	0.1	0.1	0.1	0	0	0
Brazil	São José da Laje	2.8	2.2	3.7	9	7	11	0.3	0.2	0.4	1	1	1
Brazil	São José da Lapa	0.3	0.2	0.4	0	0	0	0.1	0.0	0.1	0	0	0
Brazil	São José da Safira	0.3	0.2	0.4	0	0	0	0.1	0.0	0.1	0	0	0
Brazil	São José da Tapera	2.8	2.2	3.7	17	13	22	0.3	0.2	0.4	2	1	2
Brazil	São José da Varginha	0.3	0.2	0.4	0	0	0	0.1	0.0	0.1	0	0	0
Brazil	São José da Vitória	1.7	1.4	2.2	0	0	0	0.2	0.2	0.3	0	0	0
Brazil	São José das Missões	0.1	0.1	0.2	0	0	0	0.0	0.0	0.0	0	0	0
Brazil	São José das Palmeiras	0.3	0.2	0.3	0	0	0	0.1	0.0	0.1	0	0	0
Brazil	São José de Caiana	1.0	0.7	1.2	1	1	1	0.1	0.1	0.1	0	0	0
Brazil	São José de Espinharas	1.0	0.7	1.2	1	1	1	0.1	0.1	0.1	0	0	0
Brazil	São José de Mipibu	1.2	0.9	1.5	5	4	6	0.1	0.1	0.2	0	0	1
Brazil	São José de Piranhas	1.0	0.7	1.2	2	2	3	0.1	0.1	0.1	0	0	0
Brazil	São José de Princesa	1.0	0.7	1.2	1	0	1	0.1	0.1	0.1	0	0	0
Brazil	São José de Ribamar	1.7	1.3	2.2	29	22	38	0.2	0.2	0.3	5	4	6
Brazil	São José de Ubá	0.2	0.1	0.3	0	0	0	0.1	0.0	0.1	0	0	0

Table 3: Diarrhoea Mortality by Unit *(continued)*

Country	Unit	2000 mortality mean rate (per 10,000)	lower rate (per 10,000)	upper rate (per 10,000)	mean count	lower count	upper count	2017 mortality mean rate (per 10,000)	lower rate (per 10,000)	upper rate (per 10,000)	mean count	lower count	upper count
Brazil	São José do Alegre	0.3	0.2	0.4	0	0	0	0.1	0.0	0.1	0	0	0
Brazil	São José do Barreiro	0.2	0.1	0.2	0	0	0	0.0	0.0	0.0	0	0	0
Brazil	São José do Belmonte	1.0	0.7	1.2	0	0	0	0.1	0.1	0.1	0	0	0
Brazil	São José do Bonfim	1.0	0.7	1.2	0	0	1	0.1	0.1	0.1	0	0	0
Brazil	São José do Calçado	0.3	0.2	0.3	0	0	0	0.1	0.1	0.1	0	0	0
Brazil	São José do Campestre	1.2	0.9	1.5	2	2	3	0.1	0.1	0.2	0	0	0
Brazil	São José do Cedro	0.2	0.2	0.3	0	0	0	0.1	0.0	0.1	0	0	0
Brazil	São José do Cerrito	0.2	0.2	0.3	0	0	0	0.1	0.0	0.1	0	0	0
Brazil	São José do Divino	1.4	1.1	1.8	1	1	1	0.2	0.1	0.2	0	0	0
Brazil	São José do Divino	0.3	0.2	0.4	0	0	0	0.1	0.0	0.1	0	0	0
Brazil	São José do Goiabal	0.3	0.2	0.4	0	0	0	0.1	0.0	0.1	0	0	0
Brazil	São José do Herval	0.1	0.1	0.2	0	0	0	0.0	0.0	0.0	0	0	0
Brazil	São José do Hortêncio	0.1	0.1	0.2	0	0	0	0.0	0.0	0.0	0	0	0
Brazil	São José do Inhacorá	0.1	0.1	0.2	0	0	0	0.0	0.0	0.0	0	0	0
Brazil	São José do Jacuípe	1.7	1.4	2.2	1	1	2	0.2	0.2	0.3	0	0	0
Brazil	São José do Jacuri	0.3	0.2	0.4	0	0	0	0.1	0.0	0.1	0	0	0
Brazil	São José do Mantimento	0.3	0.2	0.4	0	0	0	0.1	0.0	0.1	0	0	0
Brazil	São José do Ouro	0.1	0.1	0.2	0	0	0	0.0	0.0	0.0	0	0	0
Brazil	São José do Peixe	1.4	1.1	1.8	1	1	1	0.2	0.1	0.2	0	0	0
Brazil	São José do Piauí	1.4	1.1	1.8	1	1	1	0.2	0.1	0.2	0	0	0
Brazil	São José do Povo	0.4	0.3	0.5	0	0	0	0.1	0.1	0.1	0	0	0
Brazil	São José do Rio Claro	0.4	0.3	0.5	1	0	1	0.1	0.1	0.1	0	0	0
Brazil	São José do Rio Pardo	0.2	0.1	0.2	1	0	1	0.0	0.0	0.0	0	0	0
Brazil	São José do Rio Preto	0.2	0.1	0.2	4	3	5	0.0	0.0	0.0	1	1	1
Brazil	São José do Sabugi	1.0	0.7	1.2	0	0	0	0.1	0.1	0.1	0	0	0
Brazil	São José do Seridó	1.2	0.9	1.5	0	0	0	0.1	0.1	0.2	0	0	0
Brazil	São José do Vale do Rio Preto	0.2	0.1	0.3	0	0	0	0.1	0.0	0.1	0	0	0
Brazil	São José do Xingu	0.4	0.3	0.5	0	0	0	0.1	0.1	0.1	0	0	0
Brazil	São José dos Ausentes	0.1	0.1	0.2	0	0	0	0.0	0.0	0.0	0	0	0
Brazil	São José dos Basílios	1.7	1.3	2.2	2	1	2	0.2	0.2	0.3	0	0	0
Brazil	São José dos Campos	0.2	0.1	0.2	7	5	9	0.0	0.0	0.0	2	1	2
Brazil	São José dos Cordeiros	1.0	0.7	1.2	1	1	1	0.1	0.1	0.1	0	0	0

Table 3: Diarrhoea Mortality by Unit *(continued)*

Country	Unit	2000 mortality mean rate (per 10,000)	lower rate (per 10,000)	upper rate (per 10,000)	mean count	lower count	upper count	2017 mortality mean rate (per 10,000)	lower rate (per 10,000)	upper rate (per 10,000)	mean count	lower count	upper count
Brazil	São José dos Pinhais	0.3	0.2	0.3	7	5	9	0.1	0.0	0.1	2	1	2
Brazil	São José dos Quatro Marcos	0.4	0.3	0.5	1	1	1	0.1	0.1	0.1	0	0	0
Brazil	São José dos Ramos	1.0	0.7	1.2	1	1	1	0.1	0.1	0.1	0	0	0
Brazil	São Juliao	1.4	1.1	1.8	1	1	1	0.2	0.1	0.2	0	0	0
Brazil	São Leopoldo	0.1	0.1	0.2	2	2	3	0.0	0.0	0.0	0	0	1
Brazil	São Lourenço	0.3	0.2	0.4	1	0	1	0.1	0.0	0.1	0	0	0
Brazil	São Lourenço da Mata	2.0	1.6	2.6	19	15	25	0.2	0.2	0.3	2	2	3
Brazil	São Lourenço da Serra	0.2	0.1	0.2	0	0	0	0.0	0.0	0.0	0	0	0
Brazil	São Lourenço do Oeste	0.2	0.2	0.3	0	0	0	0.1	0.0	0.1	0	0	0
Brazil	São Lourenço do Piauí	1.4	1.1	1.8	1	1	1	0.2	0.1	0.2	0	0	0
Brazil	São Lourenço do Sul	0.1	0.1	0.2	0	0	0	0.0	0.0	0.0	0	0	0
Brazil	São Ludgero	0.2	0.2	0.3	0	0	0	0.1	0.0	0.1	0	0	0
Brazil	São Luis	1.7	1.3	2.2	139	107	181	0.2	0.2	0.3	19	15	26
Brazil	São Luís de Montes Belos	0.2	0.1	0.2	0	0	1	0.1	0.0	0.1	0	0	0
Brazil	São Luis do Piauí	1.4	1.1	1.8	0	0	0	0.2	0.1	0.2	0	0	0
Brazil	São Luís do Quitunde	2.8	2.2	3.7	15	12	20	0.3	0.2	0.4	2	1	2
Brazil	São Luis Gonzaga do Maranhao	1.7	1.3	2.2	6	4	7	0.2	0.2	0.3	1	0	1
Brazil	São Luiz	0.7	0.6	0.9	1	0	1	0.2	0.2	0.3	0	0	0
Brazil	São Luiz do Curu	2.3	1.7	2.9	2	2	3	0.1	0.1	0.2	0	0	0
Brazil	São Luiz do Norte	0.2	0.1	0.2	0	0	0	0.1	0.0	0.1	0	0	0
Brazil	São Luiz do Paraitinga	0.2	0.1	0.2	0	0	0	0.0	0.0	0.0	0	0	0
Brazil	São Luiz Gonzaga	0.1	0.1	0.2	0	0	0	0.0	0.0	0.0	0	0	0
Brazil	São Mamede	1.0	0.7	1.2	1	1	1	0.1	0.1	0.1	0	0	0
Brazil	São Manoel do Paraná	0.3	0.2	0.3	0	0	0	0.1	0.0	0.1	0	0	0
Brazil	São Manuel	0.2	0.1	0.2	1	0	1	0.0	0.0	0.0	0	0	0
Brazil	São Marcos	0.1	0.1	0.2	0	0	0	0.0	0.0	0.0	0	0	0
Brazil	São Martinho	0.2	0.2	0.3	0	0	0	0.1	0.0	0.1	0	0	0
Brazil	São Martinho	0.1	0.1	0.2	0	0	0	0.0	0.0	0.0	0	0	0
Brazil	São Martinho da Serra	0.1	0.1	0.2	0	0	0	0.0	0.0	0.0	0	0	0
Brazil	São Mateus	0.3	0.2	0.3	3	2	3	0.1	0.1	0.1	1	1	1
Brazil	São Mateus do Maranhão	1.7	1.3	2.2	8	6	10	0.2	0.2	0.3	1	1	2
Brazil	São Mateus do Sul	0.3	0.2	0.3	2	1	2	0.1	0.0	0.1	0	0	0
Brazil	São Miguel	1.2	0.9	1.5	3	2	4	0.1	0.1	0.2	0	0	0
Brazil	São Miguel Arcanjo	0.2	0.1	0.2	0	0	1	0.0	0.0	0.0	0	0	0
Brazil	São Miguel da Baixa Grande	1.4	1.1	1.8	0	0	0	0.2	0.1	0.2	0	0	0
Brazil	São Miguel da Boa Vista	0.2	0.2	0.3	0	0	0	0.1	0.0	0.1	0	0	0
Brazil	São Miguel das Matas	1.7	1.4	2.2	2	2	3	0.2	0.2	0.3	0	0	0
Brazil	São Miguel das Misses	0.1	0.1	0.2	0	0	0	0.0	0.0	0.0	0	0	0

Table 3: Diarrhoea Mortality by Unit *(continued)*

Country	Unit	2000 mortality mean rate (per 10,000)	2000 mortality lower rate (per 10,000)	2000 mortality upper rate (per 10,000)	2000 mortality mean count	2000 mortality lower count	2000 mortality upper count	2017 mortality mean rate (per 10,000)	2017 mortality lower rate (per 10,000)	2017 mortality upper rate (per 10,000)	2017 mortality mean count	2017 mortality lower count	2017 mortality upper count
Brazil	São Miguel de Touros	1.2	0.9	1.5	1	1	1	0.1	0.1	0.2	0	0	0
Brazil	São Miguel do Aleixo	1.3	1.0	1.7	0	0	1	0.2	0.1	0.3	0	0	0
Brazil	São Miguel do Anta	0.3	0.2	0.4	0	0	0	0.1	0.0	0.1	0	0	0
Brazil	São Miguel do Araguaia	0.2	0.1	0.2	0	0	0	0.1	0.0	0.1	0	0	0
Brazil	São Miguel do Fidalgo	1.4	1.1	1.8	0	0	1	0.2	0.1	0.2	0	0	0
Brazil	São Miguel do Guamá	0.9	0.7	1.1	4	3	5	0.1	0.1	0.2	1	1	1
Brazil	São Miguel do Guaporé	0.4	0.3	0.5	1	1	1	0.1	0.1	0.1	0	0	0
Brazil	São Miguel do Iguaçu	0.3	0.2	0.3	1	0	1	0.1	0.0	0.1	0	0	0
Brazil	São Miguel do Oeste	0.2	0.2	0.3	1	0	1	0.1	0.0	0.1	0	0	0
Brazil	São Miguel do Passa Quatro	0.2	0.1	0.2	0	0	0	0.1	0.0	0.1	0	0	0
Brazil	São Miguel do Tocantins	0.8	0.7	1.1	2	2	3	0.1	0.1	0.2	0	0	0
Brazil	São Miguel dos Campos	2.8	2.2	3.7	22	17	29	0.3	0.2	0.4	3	2	4
Brazil	São Miguel dos Milagres	2.8	2.2	3.7	1	1	1	0.3	0.2	0.4	0	0	0
Brazil	São Miguel Taipu	1.0	0.7	1.2	1	1	1	0.1	0.1	0.1	0	0	0
Brazil	São Miguel Tapuio	1.4	1.1	1.8	3	2	4	0.2	0.1	0.2	0	0	0
Brazil	São Nicolau	0.1	0.1	0.2	0	0	0	0.0	0.0	0.0	0	0	0
Brazil	São Patrício	0.2	0.1	0.2	0	0	0	0.1	0.0	0.1	0	0	0
Brazil	São Paulo	0.2	0.1	0.2	128	96	164	0.0	0.0	0.0	26	20	34
Brazil	São Paulo das Missões	0.1	0.1	0.2	0	0	0	0.0	0.0	0.0	0	0	0
Brazil	São Paulo de Olivença	0.8	0.6	0.9	3	2	3	0.2	0.2	0.3	1	1	1
Brazil	São Paulo do Potengi	1.2	0.9	1.5	2	2	3	0.1	0.1	0.2	0	0	0
Brazil	São Pedro	1.2	0.9	1.5	1	1	1	0.1	0.1	0.2	0	0	0
Brazil	São Pedro	0.2	0.1	0.2	0	0	0	0.0	0.0	0.0	0	0	0
Brazil	São Pedro da Água Branca	1.7	1.3	2.2	1	1	2	0.2	0.2	0.3	0	0	0
Brazil	São Pedro da Aldeia	0.2	0.1	0.3	1	1	1	0.1	0.0	0.1	0	0	0
Brazil	São Pedro da Cipa	0.4	0.3	0.5	0	0	0	0.1	0.1	0.1	0	0	0
Brazil	São Pedro da Serra	0.1	0.1	0.2	0	0	0	0.0	0.0	0.0	0	0	0
Brazil	São Pedro da União	0.3	0.2	0.4	0	0	0	0.1	0.0	0.1	0	0	0
Brazil	São Pedro de Alcântara	0.2	0.2	0.3	0	0	0	0.1	0.0	0.1	0	0	0
Brazil	São Pedro do Butiá	0.1	0.1	0.2	0	0	0	0.0	0.0	0.0	0	0	0
Brazil	São Pedro do Iguaçu	0.3	0.2	0.3	0	0	0	0.1	0.0	0.1	0	0	0
Brazil	São Pedro do Ivaí	0.3	0.2	0.3	0	0	0	0.1	0.0	0.1	0	0	0
Brazil	São Pedro do Paraná	0.3	0.2	0.3	0	0	0	0.1	0.0	0.1	0	0	0
Brazil	São Pedro do Piauí	1.4	1.1	1.8	2	2	3	0.2	0.1	0.2	0	0	0

Table 3: Diarrhoea Mortality by Unit *(continued)*

Country	Unit	2000 mortality mean rate (per 10,000)	2000 mortality lower rate (per 10,000)	2000 mortality upper rate (per 10,000)	2000 mortality mean count	2000 mortality lower count	2000 mortality upper count	2017 mortality mean rate (per 10,000)	2017 mortality lower rate (per 10,000)	2017 mortality upper rate (per 10,000)	2017 mortality mean count	2017 mortality lower count	2017 mortality upper count
Brazil	São Pedro do Suaçuí	0.3	0.2	0.4	0	0	0	0.1	0.0	0.1	0	0	0
Brazil	São Pedro do Sul	0.1	0.1	0.2	0	0	0	0.0	0.0	0.0	0	0	0
Brazil	São Pedro do Turvo	0.2	0.1	0.2	0	0	0	0.0	0.0	0.0	0	0	0
Brazil	São Pedro dos Crentes	1.7	1.3	2.2	1	1	1	0.2	0.2	0.3	0	0	0
Brazil	São Pedro dos Ferros	0.3	0.2	0.4	0	0	0	0.1	0.0	0.1	0	0	0
Brazil	São Rafael	1.2	0.9	1.5	1	1	1	0.1	0.1	0.2	0	0	0
Brazil	São Raimundo das Mangabeiras	1.7	1.3	2.2	4	3	5	0.2	0.2	0.3	1	0	1
Brazil	São Raimundo do Doca Bezerra	1.7	1.3	2.2	1	1	1	0.2	0.2	0.3	0	0	0
Brazil	São Raimundo Nonato	1.4	1.1	1.8	5	4	6	0.2	0.1	0.2	1	0	1
Brazil	São Roberto	1.7	1.3	2.2	1	1	1	0.2	0.2	0.3	0	0	0
Brazil	São Romão	0.3	0.2	0.4	0	0	0	0.1	0.0	0.1	0	0	0
Brazil	São Roque	0.2	0.1	0.2	1	1	1	0.0	0.0	0.0	0	0	0
Brazil	São Roque de Minas	0.3	0.2	0.4	0	0	0	0.1	0.0	0.1	0	0	0
Brazil	São Roque do Canaã	0.3	0.2	0.3	0	0	0	0.1	0.1	0.1	0	0	0
Brazil	São Salvador do Tocantins	0.8	0.7	1.1	0	0	0	0.1	0.1	0.2	0	0	0
Brazil	São Sebastião	0.2	0.1	0.2	1	1	1	0.0	0.0	0.0	0	0	0
Brazil	São Sebastião	2.8	2.2	3.7	12	10	16	0.3	0.2	0.4	1	1	2
Brazil	São Sebastião da Amoreira	0.3	0.2	0.3	0	0	0	0.1	0.0	0.1	0	0	0
Brazil	São Sebastião da Bela Vista	0.3	0.2	0.4	0	0	0	0.1	0.0	0.1	0	0	0
Brazil	São Sebastião da Boa Vista	0.9	0.7	1.1	2	2	3	0.1	0.1	0.2	0	0	1
Brazil	São Sebastião da Grama	0.2	0.1	0.2	0	0	0	0.0	0.0	0.0	0	0	0
Brazil	São Sebastião de Lagoa de Roça	1.0	0.7	1.2	1	1	1	0.1	0.1	0.1	0	0	0
Brazil	São Sebastião do Alto	0.2	0.1	0.3	0	0	0	0.1	0.0	0.1	0	0	0
Brazil	São Sebastião do Anta	0.3	0.2	0.4	0	0	0	0.1	0.0	0.1	0	0	0
Brazil	São Sebastião do Caí	0.1	0.1	0.2	0	0	0	0.0	0.0	0.0	0	0	0
Brazil	São Sebastião do Maranhão	0.3	0.2	0.4	0	0	0	0.1	0.0	0.1	0	0	0
Brazil	São Sebastião do Oeste	0.3	0.2	0.4	0	0	0	0.1	0.0	0.1	0	0	0
Brazil	São Sebastião do Paraíso	0.3	0.2	0.4	1	1	2	0.1	0.0	0.1	0	0	0
Brazil	São Sebastião do Passé	1.7	1.4	2.2	8	6	10	0.2	0.2	0.3	1	1	1
Brazil	São Sebastião do Rio Preto	0.3	0.2	0.4	0	0	0	0.1	0.0	0.1	0	0	0
Brazil	São Sebastião do Rio Verde	0.3	0.2	0.4	0	0	0	0.1	0.0	0.1	0	0	0
Brazil	São Sebastião do Tocantins	0.8	0.7	1.1	1	0	1	0.1	0.1	0.2	0	0	0
Brazil	São Sebastião do Uatumã	0.8	0.6	0.9	3	2	4	0.2	0.2	0.3	1	1	2
Brazil	São Sebastião do Umbuzeiro	1.0	0.7	1.2	0	0	0	0.1	0.1	0.1	0	0	0

Table 3: Diarrhoea Mortality by Unit *(continued)*

Country	Unit	2000 mortality mean rate (per 10,000)	lower rate (per 10,000)	upper rate (per 10,000)	mean count	lower count	upper count	2017 mortality mean rate (per 10,000)	lower rate (per 10,000)	upper rate (per 10,000)	mean count	lower count	upper count
Brazil	São Sebastio da Vargem Alegre	0.3	0.2	0.4	0	0	0	0.1	0.0	0.1	0	0	0
Brazil	São Sepé	0.1	0.1	0.2	0	0	0	0.0	0.0	0.0	0	0	0
Brazil	São Simão	0.2	0.1	0.2	0	0	0	0.1	0.0	0.1	0	0	0
Brazil	São Simão	0.2	0.1	0.2	0	0	0	0.0	0.0	0.0	0	0	0
Brazil	São Thomé das Letras	0.3	0.2	0.4	0	0	0	0.1	0.0	0.1	0	0	0
Brazil	São Tiago	0.3	0.2	0.4	0	0	0	0.1	0.0	0.1	0	0	0
Brazil	São Tomás de Aquino	0.3	0.2	0.4	0	0	0	0.1	0.0	0.1	0	0	0
Brazil	São Tomé	1.2	0.9	1.5	2	1	2	0.1	0.1	0.2	0	0	0
Brazil	São Tomé	0.3	0.2	0.3	0	0	0	0.1	0.0	0.1	0	0	0
Brazil	São Valentim	0.1	0.1	0.2	0	0	0	0.0	0.0	0.0	0	0	0
Brazil	São Valentim do Sul	0.1	0.1	0.2	0	0	0	0.0	0.0	0.0	0	0	0
Brazil	São Valério da Natividade	0.8	0.7	1.1	0	0	1	0.1	0.1	0.2	0	0	0
Brazil	São Valério do Sul	0.1	0.1	0.2	0	0	0	0.0	0.0	0.0	0	0	0
Brazil	São Vendelino	0.1	0.1	0.2	0	0	0	0.0	0.0	0.0	0	0	0
Brazil	São Vicente	1.2	0.9	1.5	1	1	1	0.1	0.1	0.2	0	0	0
Brazil	São Vicente	0.2	0.1	0.2	2	2	3	0.0	0.0	0.0	1	0	1
Brazil	São Vicente de Minas	0.3	0.2	0.4	0	0	0	0.1	0.0	0.1	0	0	0
Brazil	São Vicente Ferrer	2.0	1.6	2.6	5	4	6	0.2	0.2	0.3	0	0	0
Brazil	São Vicente Ferrer	1.7	1.3	2.2	4	3	5	0.2	0.2	0.3	1	0	1
Brazil	Sapé	1.0	0.7	1.2	5	4	6	0.1	0.1	0.1	0	0	1
Brazil	Sapeaçu	1.7	1.4	2.2	3	2	4	0.2	0.2	0.3	0	0	0
Brazil	Sapezal	0.4	0.3	0.5	0	0	0	0.1	0.1	0.1	0	0	0
Brazil	Sapezal	0.5	0.3	0.6	5	4	7	0.2	0.1	0.2	2	1	2
Brazil	Sapiranga	0.1	0.1	0.2	1	1	1	0.0	0.0	0.0	0	0	0
Brazil	Sapopema	0.3	0.2	0.3	0	0	0	0.1	0.0	0.1	0	0	0
Brazil	Sapucaí-Mirim	0.3	0.2	0.4	0	0	0	0.1	0.0	0.1	0	0	0
Brazil	Sapucaia	0.9	0.7	1.1	0	0	0	0.1	0.1	0.2	0	0	0
Brazil	Sapucaia	0.2	0.1	0.3	0	0	1	0.1	0.0	0.1	0	0	0
Brazil	Sapucaia do Sul	0.1	0.1	0.2	1	1	1	0.0	0.0	0.0	0	0	0
Brazil	Saquarema	0.2	0.1	0.3	1	1	1	0.1	0.0	0.1	0	0	0
Brazil	Sarandi	0.3	0.2	0.3	1	1	2	0.1	0.0	0.1	0	0	0
Brazil	Sarandi	0.1	0.1	0.2	0	0	0	0.0	0.0	0.0	0	0	0
Brazil	Sarapuí	0.2	0.1	0.2	0	0	0	0.0	0.0	0.0	0	0	0
Brazil	Sardoá	0.3	0.2	0.4	0	0	0	0.1	0.0	0.1	0	0	0
Brazil	Sarutaiá	0.2	0.1	0.2	0	0	0	0.0	0.0	0.0	0	0	0
Brazil	Sarzedo	0.3	0.2	0.4	1	1	1	0.1	0.0	0.1	0	0	0
Brazil	Sátiro Dias	1.7	1.4	2.2	4	3	5	0.2	0.2	0.3	0	0	1
Brazil	Satuba	2.8	2.2	3.7	4	3	5	0.3	0.2	0.4	0	0	1
Brazil	Satubinha	1.7	1.3	2.2	3	2	4	0.2	0.2	0.3	0	0	1
Brazil	Saubara	1.7	1.4	2.2	1	1	1	0.2	0.2	0.3	0	0	0
Brazil	Saudade do Iguaçu	0.3	0.2	0.3	0	0	0	0.1	0.0	0.1	0	0	0
Brazil	Saudades	0.2	0.2	0.3	0	0	0	0.1	0.0	0.1	0	0	0
Brazil	Saúde	1.7	1.4	2.2	2	2	3	0.2	0.2	0.3	0	0	0
Brazil	Schroeder	0.2	0.2	0.3	0	0	0	0.1	0.0	0.1	0	0	0
Brazil	Seabra	1.7	1.4	2.2	10	8	13	0.2	0.2	0.3	1	1	1
Brazil	Seara	0.2	0.2	0.3	0	0	0	0.1	0.0	0.1	0	0	0
Brazil	Sebastianópolis do Sul	0.2	0.1	0.2	0	0	0	0.0	0.0	0.0	0	0	0
Brazil	Sebastião Barros	1.4	1.1	1.8	1	1	1	0.2	0.1	0.2	0	0	0
Brazil	Sebastião Laranjeiras	1.7	1.4	2.2	2	2	3	0.2	0.2	0.3	0	0	0

Table 3: Diarrhoea Mortality by Unit *(continued)*

Country	Unit	2000 mortality mean rate (per 10,000)	lower rate (per 10,000)	upper rate (per 10,000)	mean count	lower count	upper count	2017 mortality mean rate (per 10,000)	lower rate (per 10,000)	upper rate (per 10,000)	mean count	lower count	upper count
Brazil	Sebastião Leal	1.4	1.1	1.8	1	0	1	0.2	0.1	0.2	0	0	0
Brazil	Seberi	0.1	0.1	0.2	0	0	0	0.0	0.0	0.0	0	0	0
Brazil	Sede Nova	0.1	0.1	0.2	0	0	0	0.0	0.0	0.0	0	0	0
Brazil	Segredo	0.1	0.1	0.2	0	0	0	0.0	0.0	0.0	0	0	0
Brazil	Selbach	0.1	0.1	0.2	0	0	0	0.0	0.0	0.0	0	0	0
Brazil	Selvíria	0.5	0.3	0.6	0	0	0	0.2	0.1	0.2	0	0	0
Brazil	Sem-Peixe	0.3	0.2	0.4	0	0	0	0.1	0.0	0.1	0	0	0
Brazil	Sena Madureira	1.1	0.9	1.3	4	3	5	0.2	0.2	0.3	1	1	1
Brazil	Senador Alexandre Costa	1.7	1.3	2.2	2	2	3	0.2	0.2	0.3	0	0	0
Brazil	Senador Amaral	0.3	0.2	0.4	0	0	0	0.1	0.0	0.1	0	0	0
Brazil	Senador Canedo	0.2	0.1	0.2	1	1	1	0.1	0.0	0.1	0	0	1
Brazil	Senador Cortes	0.3	0.2	0.4	0	0	0	0.1	0.0	0.1	0	0	0
Brazil	Senador Elói de Souza	1.2	0.9	1.5	1	1	1	0.1	0.1	0.2	0	0	0
Brazil	Senador Firmino	0.3	0.2	0.4	0	0	0	0.1	0.0	0.1	0	0	0
Brazil	Senador Georgino Avelino	1.2	0.9	1.5	0	0	0	0.1	0.1	0.2	0	0	0
Brazil	Senador Guiomard	1.1	0.9	1.3	3	3	4	0.2	0.2	0.3	1	0	1
Brazil	Senador José Bento	0.3	0.2	0.4	0	0	0	0.1	0.0	0.1	0	0	0
Brazil	Senador José Porfírio	0.9	0.7	1.1	2	1	2	0.1	0.1	0.2	0	0	0
Brazil	Senador La Rocque	1.7	1.3	2.2	5	4	6	0.2	0.2	0.3	1	0	1
Brazil	Senador Modestino Gonçalves	0.3	0.2	0.4	0	0	0	0.1	0.0	0.1	0	0	0
Brazil	Senador Pompeu	2.3	1.7	2.9	7	5	9	0.1	0.1	0.2	0	0	0
Brazil	Senador Rui Palmeira	2.8	2.2	3.7	4	3	5	0.3	0.2	0.4	0	0	1
Brazil	Senador Sá	2.3	1.7	2.9	2	1	2	0.1	0.1	0.2	0	0	0
Brazil	Senador Salgado Filho	0.1	0.1	0.2	0	0	0	0.0	0.0	0.0	0	0	0
Brazil	Sengés	0.3	0.2	0.3	1	0	1	0.1	0.0	0.1	0	0	0
Brazil	Senhor do Bonfim	1.7	1.4	2.2	13	10	17	0.2	0.2	0.3	2	1	2
Brazil	Senhora de Oliveira	0.3	0.2	0.4	0	0	0	0.1	0.0	0.1	0	0	0
Brazil	Senhora do Porto	0.3	0.2	0.4	0	0	0	0.1	0.0	0.1	0	0	0
Brazil	Senhora dos Remédios	0.3	0.2	0.4	0	0	0	0.1	0.0	0.1	0	0	0
Brazil	Sentinela do Sul	0.1	0.1	0.2	0	0	0	0.0	0.0	0.0	0	0	0
Brazil	Sento Sé	1.7	1.4	2.2	8	7	11	0.2	0.2	0.3	1	1	1
Brazil	Serafina Corrêa	0.1	0.1	0.2	0	0	0	0.0	0.0	0.0	0	0	0
Brazil	Sericita	0.3	0.2	0.4	0	0	0	0.1	0.0	0.1	0	0	0
Brazil	Seridó	1.0	0.7	1.2	1	1	1	0.1	0.1	0.1	0	0	0
Brazil	Seringueiras	0.4	0.3	0.5	0	0	1	0.1	0.1	0.1	0	0	0
Brazil	Sério	0.1	0.1	0.2	0	0	0	0.0	0.0	0.0	0	0	0
Brazil	Seritinga	0.3	0.2	0.4	0	0	0	0.1	0.0	0.1	0	0	0
Brazil	Seropédica	0.2	0.1	0.3	1	1	2	0.1	0.0	0.1	0	0	0
Brazil	Serra	0.3	0.2	0.3	8	6	11	0.1	0.1	0.1	3	2	4
Brazil	Serra Alta	0.2	0.2	0.3	0	0	0	0.1	0.0	0.1	0	0	0
Brazil	Serra Azul	0.2	0.1	0.2	0	0	0	0.0	0.0	0.0	0	0	0
Brazil	Serra Azul de Minas	0.3	0.2	0.4	0	0	0	0.1	0.0	0.1	0	0	0
Brazil	Serra Branca	1.0	0.7	1.2	1	1	2	0.1	0.1	0.1	0	0	0
Brazil	Serra da Raiz	1.0	0.7	1.2	0	0	0	0.1	0.1	0.1	0	0	0
Brazil	Serra da Saudad	0.3	0.2	0.4	0	0	0	0.1	0.0	0.1	0	0	0
Brazil	Serra de São Bento	1.2	0.9	1.5	1	1	1	0.1	0.1	0.2	0	0	0

Table 3: Diarrhoea Mortality by Unit *(continued)*

Country	Unit	2000 mortality						2017 mortality					
		mean rate (per 10,000)	lower rate (per 10,000)	upper rate (per 10,000)	mean count	lower count	upper count	mean rate (per 10,000)	lower rate (per 10,000)	upper rate (per 10,000)	mean count	lower count	upper count
Brazil	Serra do Mel	1.2	0.9	1.5	1	1	1	0.1	0.1	0.2	0	0	0
Brazil	Serra do Navio	0.2	0.2	0.3	0	0	0	0.1	0.1	0.2	0	0	0
Brazil	Serra do Ramalho	1.7	1.4	2.2	8	6	10	0.2	0.2	0.3	1	1	1
Brazil	Serra do Salitre	0.3	0.2	0.4	0	0	0	0.1	0.0	0.1	0	0	0
Brazil	Serra dos Aimorés	0.3	0.2	0.4	0	0	0	0.1	0.0	0.1	0	0	0
Brazil	Serra dourada	1.7	1.4	2.2	4	3	5	0.2	0.2	0.3	0	0	1
Brazil	Serra Grande	1.0	0.7	1.2	0	0	0	0.1	0.1	0.1	0	0	0
Brazil	Serra Negra	0.2	0.1	0.2	0	0	0	0.0	0.0	0.0	0	0	0
Brazil	Serra Negra do Norte	1.2	0.9	1.5	1	1	1	0.1	0.1	0.2	0	0	0
Brazil	Serra Preta	1.7	1.4	2.2	3	2	4	0.2	0.2	0.3	0	0	0
Brazil	Serra Redonda	1.0	0.7	1.2	1	1	1	0.1	0.1	0.1	0	0	0
Brazil	Serra Talhada	2.0	1.6	2.6	18	14	23	0.2	0.2	0.3	2	1	2
Brazil	Serrana	0.2	0.1	0.2	0	0	1	0.0	0.0	0.0	0	0	0
Brazil	Serrania	0.3	0.2	0.4	0	0	0	0.1	0.0	0.1	0	0	0
Brazil	Serrano do Maranhão	1.7	1.3	2.2	2	2	3	0.2	0.2	0.3	0	0	0
Brazil	Serranópolis	0.2	0.1	0.2	0	0	0	0.1	0.0	0.1	0	0	0
Brazil	Serranópolis de Minas	0.3	0.2	0.4	0	0	0	0.1	0.0	0.1	0	0	0
Brazil	Serranópolis do Iguaçu	0.3	0.2	0.3	0	0	0	0.1	0.0	0.1	0	0	0
Brazil	Serranos	0.3	0.2	0.4	0	0	0	0.1	0.0	0.1	0	0	0
Brazil	Serraria	1.0	0.7	1.2	1	1	1	0.1	0.1	0.1	0	0	0
Brazil	Serrinha	1.2	0.9	1.5	1	1	1	0.1	0.1	0.2	0	0	0
Brazil	Serrinha	1.7	1.4	2.2	16	13	21	0.2	0.2	0.3	2	2	3
Brazil	Serrinha dos Pintos	1.2	0.9	1.5	0	0	0	0.1	0.1	0.2	0	0	0
Brazil	Serrita	2.0	1.6	2.6	6	5	8	0.2	0.2	0.3	1	0	1
Brazil	Serro	0.3	0.2	0.4	1	0	1	0.1	0.0	0.1	0	0	0
Brazil	Serrolândia	1.7	1.4	2.2	2	2	3	0.2	0.2	0.3	0	0	0
Brazil	Sertaneja	0.3	0.2	0.3	0	0	0	0.1	0.0	0.1	0	0	0
Brazil	Sertânia	2.0	1.6	2.6	7	6	10	0.2	0.2	0.3	1	1	1
Brazil	Sertanópolis	0.3	0.2	0.3	0	0	0	0.1	0.0	0.1	0	0	0
Brazil	Sertão	0.1	0.1	0.2	0	0	0	0.0	0.0	0.0	0	0	0
Brazil	Sertão Santana	0.1	0.1	0.2	0	0	0	0.0	0.0	0.0	0	0	0
Brazil	Sertaozinho	0.2	0.1	0.2	1	1	2	0.0	0.0	0.0	0	0	0
Brazil	Sertãozinho	1.0	0.7	1.2	0	0	0	0.1	0.1	0.1	0	0	0
Brazil	Sete Barras	0.2	0.1	0.2	0	0	0	0.0	0.0	0.0	0	0	0
Brazil	Sete de Setembro	0.1	0.1	0.2	0	0	0	0.0	0.0	0.0	0	0	0
Brazil	Sete Lagoas	0.3	0.2	0.4	5	4	6	0.1	0.0	0.1	1	1	1
Brazil	Sete Quedas	0.5	0.3	0.6	0	0	0	0.2	0.1	0.2	0	0	0
Brazil	Setubinha	0.3	0.2	0.4	0	0	0	0.1	0.0	0.1	0	0	0
Brazil	Severiano de Almeida	0.1	0.1	0.2	0	0	0	0.0	0.0	0.0	0	0	0
Brazil	Severiano Melo	1.2	0.9	1.5	1	1	2	0.1	0.1	0.2	0	0	0
Brazil	Severínia	0.2	0.1	0.2	0	0	0	0.0	0.0	0.0	0	0	0
Brazil	Sideròpolis	0.2	0.2	0.3	0	0	0	0.1	0.0	0.1	0	0	0
Brazil	Sidrolândia	0.5	0.3	0.6	1	1	2	0.2	0.1	0.2	1	1	1
Brazil	Sigefredo Pacheco	1.4	1.1	1.8	1	1	2	0.2	0.1	0.2	0	0	0
Brazil	Silva Jardim	0.2	0.1	0.3	0	0	1	0.1	0.0	0.1	0	0	0
Brazil	Silvânia	0.2	0.1	0.2	0	0	1	0.1	0.0	0.1	0	0	0
Brazil	Silvanópolis	0.8	0.7	1.1	0	0	1	0.1	0.1	0.2	0	0	0
Brazil	Silveira Martins	0.1	0.1	0.2	0	0	0	0.0	0.0	0.0	0	0	0
Brazil	Silveirânia	0.3	0.2	0.4	0	0	0	0.1	0.0	0.1	0	0	0
Brazil	Silveiras	0.2	0.1	0.2	0	0	0	0.0	0.0	0.0	0	0	0
Brazil	Silves	0.8	0.6	0.9	1	1	1	0.2	0.2	0.3	0	0	0
Brazil	Silvianópolis	0.3	0.2	0.4	0	0	0	0.1	0.0	0.1	0	0	0
Brazil	Simão Dias	1.3	1.0	1.7	5	4	7	0.2	0.1	0.3	1	0	1

Table 3: Diarrhoea Mortality by Unit *(continued)*

Country	Unit	2000 mortality mean rate (per 10,000)	lower rate (per 10,000)	upper rate (per 10,000)	mean count	lower count	upper count	2017 mortality mean rate (per 10,000)	lower rate (per 10,000)	upper rate (per 10,000)	mean count	lower count	upper count
Brazil	Simão Pereira	0.3	0.2	0.4	0	0	0	0.1	0.0	0.1	0	0	0
Brazil	Simões	1.4	1.1	1.8	2	2	3	0.2	0.1	0.2	0	0	0
Brazil	Simões Filho	1.7	1.4	2.2	24	19	31	0.2	0.2	0.3	4	3	5
Brazil	Simolândia	0.2	0.1	0.2	0	0	0	0.1	0.0	0.1	0	0	0
Brazil	Simonésia	0.3	0.2	0.4	0	0	0	0.1	0.0	0.1	0	0	0
Brazil	Simplício Mendes	1.4	1.1	1.8	2	1	2	0.2	0.1	0.2	0	0	0
Brazil	Sinimbu	0.1	0.1	0.2	0	0	0	0.0	0.0	0.0	0	0	0
Brazil	Sinop	0.4	0.3	0.5	3	3	4	0.1	0.1	0.1	1	1	2
Brazil	Siqueira Campos	0.3	0.2	0.3	0	0	0	0.1	0.0	0.1	0	0	0
Brazil	Sirinhaém	2.0	1.6	2.6	10	8	13	0.2	0.2	0.3	1	1	2
Brazil	Siriri	1.3	1.0	1.7	1	1	1	0.2	0.1	0.3	0	0	0
Brazil	Sítio d'Abadia	0.2	0.1	0.2	0	0	0	0.1	0.0	0.1	0	0	0
Brazil	Sítio do Mato	1.7	1.4	2.2	2	2	3	0.2	0.2	0.3	0	0	0
Brazil	Sítio do Quinto	1.7	1.4	2.2	4	3	5	0.2	0.2	0.3	0	0	0
Brazil	Sitio dos Moreiras	2.0	1.6	2.6	4	3	5	0.2	0.2	0.3	0	0	0
Brazil	Sítio Novo	1.2	0.9	1.5	1	1	1	0.1	0.1	0.2	0	0	0
Brazil	Sítio Novo	1.7	1.3	2.2	4	3	5	0.2	0.2	0.3	0	0	1
Brazil	Sítio Novo do Tocantins	0.8	0.7	1.1	1	1	2	0.1	0.1	0.2	0	0	0
Brazil	Sobradinho	1.7	1.4	2.2	5	4	6	0.2	0.2	0.3	1	0	1
Brazil	Sobradinho	0.1	0.1	0.2	0	0	0	0.0	0.0	0.0	0	0	0
Brazil	Sobrado	1.0	0.7	1.2	1	1	1	0.1	0.1	0.1	0	0	0
Brazil	Sobral	2.3	1.7	2.9	43	33	55	0.1	0.1	0.2	3	2	4
Brazil	Sobrália	0.3	0.2	0.4	0	0	0	0.1	0.0	0.1	0	0	0
Brazil	Socorro	0.2	0.1	0.2	0	0	0	0.0	0.0	0.0	0	0	0
Brazil	Socorro do Piauí	1.4	1.1	1.8	1	1	1	0.2	0.1	0.2	0	0	0
Brazil	Solânea	1.0	0.7	1.2	3	2	4	0.1	0.1	0.1	0	0	0
Brazil	Soledade	1.0	0.7	1.2	1	1	2	0.1	0.1	0.1	0	0	0
Brazil	Soledade	0.1	0.1	0.2	0	0	0	0.0	0.0	0.0	0	0	0
Brazil	Soledade de Minas	0.3	0.2	0.4	0	0	0	0.1	0.0	0.1	0	0	0
Brazil	Solonópole	2.3	1.7	2.9	4	3	5	0.1	0.1	0.2	0	0	0
Brazil	Sombrio	0.2	0.2	0.3	0	0	1	0.1	0.0	0.1	0	0	0
Brazil	Sonora	0.5	0.3	0.6	0	0	0	0.2	0.1	0.2	0	0	0
Brazil	Sooretama	0.3	0.2	0.3	0	0	1	0.1	0.1	0.1	0	0	0
Brazil	Sorocaba	0.2	0.1	0.2	6	5	8	0.0	0.0	0.0	1	1	2
Brazil	Sossêgo	1.0	0.7	1.2	0	0	1	0.1	0.1	0.1	0	0	0
Brazil	Soure	0.9	0.7	1.1	1	1	2	0.1	0.1	0.2	0	0	0
Brazil	Sousa	1.0	0.7	1.2	6	5	8	0.1	0.1	0.1	1	0	1
Brazil	Souto Soares	1.7	1.4	2.2	4	3	5	0.2	0.2	0.3	0	0	1
Brazil	Sucupira	0.8	0.7	1.1	0	0	0	0.1	0.1	0.2	0	0	0
Brazil	Sucupira do Norte	1.7	1.3	2.2	2	1	2	0.2	0.2	0.3	0	0	0
Brazil	Sucupira do Riachão	1.7	1.3	2.2	1	1	1	0.2	0.2	0.3	0	0	0
Brazil	Sud Mennucci	0.2	0.1	0.2	0	0	0	0.0	0.0	0.0	0	0	0
Brazil	Sul Brazil	0.2	0.2	0.3	0	0	0	0.1	0.0	0.1	0	0	0
Brazil	Sulina	0.3	0.2	0.3	0	0	0	0.1	0.0	0.1	0	0	0
Brazil	Sumaré	0.2	0.1	0.2	2	2	3	0.0	0.0	0.0	1	1	1
Brazil	Sumé	1.0	0.7	1.2	1	1	2	0.1	0.1	0.1	0	0	0
Brazil	Sumidouro	0.2	0.1	0.3	0	0	0	0.1	0.0	0.1	0	0	0
Brazil	Surubim	2.0	1.6	2.6	12	9	15	0.2	0.2	0.3	1	1	2
Brazil	Sussuapara	1.4	1.1	1.8	1	1	1	0.2	0.1	0.2	0	0	0
Brazil	Suzanápolis	0.2	0.1	0.2	1	1	1	0.0	0.0	0.0	0	0	0
Brazil	Suzano	0.2	0.1	0.2	3	2	4	0.0	0.0	0.0	1	1	1
Brazil	Tabaí	0.1	0.1	0.2	0	0	0	0.0	0.0	0.0	0	0	0
Brazil	Tabaporã	0.4	0.3	0.5	1	0	1	0.1	0.1	0.1	0	0	0
Brazil	Tabapuã	0.2	0.1	0.2	0	0	0	0.0	0.0	0.0	0	0	0
Brazil	Tabatinga	0.8	0.6	0.9	1	1	1	0.2	0.2	0.3	0	0	0

Table 3: Diarrhoea Mortality by Unit *(continued)*

Country	Unit	2000 mortality mean rate (per 10,000)	lower rate (per 10,000)	upper rate (per 10,000)	mean count	lower count	upper count	2017 mortality mean rate (per 10,000)	lower rate (per 10,000)	upper rate (per 10,000)	mean count	lower count	upper count
Brazil	Tabatinga	0.2	0.1	0.2	0	0	0	0.0	0.0	0.0	0	0	0
Brazil	Tabira	2.0	1.6	2.6	6	5	8	0.2	0.2	0.3	1	0	1
Brazil	Taboão da Serra	0.2	0.1	0.2	2	2	3	0.0	0.0	0.0	1	0	1
Brazil	Tabocas do Brejo Velho	1.7	1.4	2.2	3	2	3	0.2	0.2	0.3	0	0	0
Brazil	Taboleiro Grande	1.2	0.9	1.5	0	0	0	0.1	0.1	0.2	0	0	0
Brazil	Tabuleiro	0.3	0.2	0.4	0	0	0	0.1	0.0	0.1	0	0	0
Brazil	Tabuleiro do Norte	2.3	1.7	2.9	7	6	10	0.1	0.1	0.2	0	0	0
Brazil	Tacaimbó	2.0	1.6	2.6	4	3	5	0.2	0.2	0.3	0	0	0
Brazil	Tacaratu	2.0	1.6	2.6	6	5	8	0.2	0.2	0.3	1	1	1
Brazil	Taciba	0.2	0.1	0.2	0	0	0	0.0	0.0	0.0	0	0	0
Brazil	Tacima	1.0	0.7	1.2	2	2	3	0.1	0.1	0.1	0	0	0
Brazil	Tacuru	0.5	0.3	0.6	0	0	1	0.2	0.1	0.2	0	0	0
Brazil	Taguaí	0.2	0.1	0.2	0	0	0	0.0	0.0	0.0	0	0	0
Brazil	Taguatinga	0.8	0.7	1.1	2	1	2	0.1	0.1	0.2	0	0	0
Brazil	Taiaçu	0.2	0.1	0.2	0	0	0	0.0	0.0	0.0	0	0	0
Brazil	Tailândia	0.9	0.7	1.1	5	4	6	0.1	0.1	0.2	2	2	3
Brazil	Taió	0.2	0.2	0.3	0	0	0	0.1	0.0	0.1	0	0	0
Brazil	Taiobeiras	0.3	0.2	0.4	1	1	1	0.1	0.0	0.1	0	0	0
Brazil	Taipas do Tocantins	0.8	0.7	1.1	0	0	0	0.1	0.1	0.2	0	0	0
Brazil	Taipu	1.2	0.9	1.5	2	2	3	0.1	0.1	0.2	0	0	0
Brazil	Taiúva	0.2	0.1	0.2	0	0	0	0.0	0.0	0.0	0	0	0
Brazil	Talismã	0.8	0.7	1.1	0	0	0	0.1	0.1	0.2	0	0	0
Brazil	Tamandaré	2.0	1.6	2.6	2	1	2	0.2	0.2	0.3	0	0	0
Brazil	Tamarana	0.3	0.2	0.3	0	0	0	0.1	0.0	0.1	0	0	0
Brazil	Tambaú	0.2	0.1	0.2	0	0	0	0.0	0.0	0.0	0	0	0
Brazil	Tambe	2.0	1.6	2.6	11	8	14	0.2	0.2	0.3	1	1	1
Brazil	Tamboara	0.3	0.2	0.3	0	0	0	0.1	0.0	0.1	0	0	0
Brazil	Tamboril	2.3	1.7	2.9	7	5	9	0.1	0.1	0.2	0	0	1
Brazil	Tamboril do Piauí	1.4	1.1	1.8	0	0	0	0.2	0.1	0.2	0	0	0
Brazil	Tanabi	0.2	0.1	0.2	0	0	0	0.0	0.0	0.0	0	0	0
Brazil	Tangará	1.2	0.9	1.5	2	1	2	0.1	0.1	0.2	0	0	0
Brazil	Tangará	0.2	0.2	0.3	0	0	0	0.1	0.0	0.1	0	0	0
Brazil	Tangará da Serra	0.4	0.3	0.5	2	2	3	0.1	0.1	0.1	1	1	1
Brazil	Tanguá	0.2	0.1	0.3	0	0	1	0.1	0.0	0.1	0	0	0
Brazil	Tanhaçu	1.7	1.4	2.2	4	3	5	0.2	0.2	0.3	0	0	1
Brazil	Tanque d'Arca	2.8	2.2	3.7	3	2	4	0.3	0.2	0.4	0	0	0
Brazil	Tanque do Piauí	1.4	1.1	1.8	0	0	0	0.2	0.1	0.2	0	0	0
Brazil	Tanque Novo	1.7	1.4	2.2	4	3	5	0.2	0.2	0.3	0	0	1
Brazil	Tanquinho	1.7	1.4	2.2	1	1	2	0.2	0.2	0.3	0	0	0
Brazil	Taparuba	0.3	0.2	0.4	0	0	0	0.1	0.0	0.1	0	0	0
Brazil	Tapauá	0.8	0.6	0.9	2	2	3	0.2	0.2	0.3	0	0	1
Brazil	Tapejara	0.3	0.2	0.3	0	0	0	0.1	0.0	0.1	0	0	0
Brazil	Tapejara	0.1	0.1	0.2	0	0	0	0.0	0.0	0.0	0	0	0
Brazil	Tapera	0.1	0.1	0.2	0	0	0	0.0	0.0	0.0	0	0	0
Brazil	Taperoá	1.0	0.7	1.2	2	1	2	0.1	0.1	0.1	0	0	0
Brazil	Taperoá	1.7	1.4	2.2	4	3	5	0.2	0.2	0.3	1	0	1
Brazil	Tapes	0.1	0.1	0.2	0	0	0	0.0	0.0	0.0	0	0	0
Brazil	Tapira	0.3	0.2	0.3	0	0	0	0.1	0.0	0.1	0	0	0
Brazil	Tapira	0.3	0.2	0.4	0	0	0	0.1	0.0	0.1	0	0	0
Brazil	Tapiraí	0.2	0.1	0.2	0	0	0	0.0	0.0	0.0	0	0	0
Brazil	Tapiraí	0.3	0.2	0.4	0	0	0	0.1	0.0	0.1	0	0	0
Brazil	Tapiramutá	1.7	1.4	2.2	4	3	5	0.2	0.2	0.3	0	0	1
Brazil	Tapiratiba	0.2	0.1	0.2	0	0	0	0.0	0.0	0.0	0	0	0
Brazil	Tapurah	0.4	0.3	0.5	2	2	3	0.1	0.1	0.1	1	1	2
Brazil	Taquara	0.1	0.1	0.2	1	1	1	0.0	0.0	0.0	0	0	0
Brazil	Taquaraçu de Minas	0.3	0.2	0.4	0	0	0	0.1	0.0	0.1	0	0	0

Table 3: Diarrhoea Mortality by Unit *(continued)*

Country	Unit	2000 mortality mean rate (per 10,000)	lower rate (per 10,000)	upper rate (per 10,000)	mean count	lower count	upper count	2017 mortality mean rate (per 10,000)	lower rate (per 10,000)	upper rate (per 10,000)	mean count	lower count	upper count
Brazil	Taquaral	0.2	0.1	0.2	0	0	0	0.0	0.0	0.0	0	0	0
Brazil	Taquaral de Goiás	0.2	0.1	0.2	0	0	0	0.1	0.0	0.1	0	0	0
Brazil	Taquarana	2.8	2.2	3.7	6	5	8	0.3	0.2	0.4	1	0	1
Brazil	Taquari	0.1	0.1	0.2	0	0	0	0.0	0.0	0.0	0	0	0
Brazil	Taquaritinga	0.2	0.1	0.2	1	1	1	0.0	0.0	0.0	0	0	0
Brazil	Taquaritinga do Norte	2.0	1.6	2.6	7	5	9	0.2	0.2	0.3	1	1	1
Brazil	Taquarituba	0.2	0.1	0.2	0	0	0	0.0	0.0	0.0	0	0	0
Brazil	Taquarivaí	0.2	0.1	0.2	0	0	0	0.0	0.0	0.0	0	0	0
Brazil	Taquaruçu do Sul	0.1	0.1	0.2	0	0	0	0.0	0.0	0.0	0	0	0
Brazil	Taquarussu	0.5	0.3	0.6	0	0	0	0.2	0.1	0.2	0	0	0
Brazil	Tarabai	0.2	0.1	0.2	0	0	0	0.0	0.0	0.0	0	0	0
Brazil	Tarauacá	1.1	0.9	1.3	4	3	5	0.2	0.2	0.3	1	1	1
Brazil	Tarrafas	2.3	1.7	2.9	3	2	3	0.1	0.1	0.2	0	0	0
Brazil	Tartarugalzinho	0.2	0.2	0.3	0	0	0	0.1	0.1	0.2	0	0	0
Brazil	Tarumã	0.2	0.1	0.2	0	0	0	0.0	0.0	0.0	0	0	0
Brazil	Tarumirim	0.3	0.2	0.4	0	0	0	0.1	0.0	0.1	0	0	0
Brazil	Tasso Fragoso	1.7	1.3	2.2	1	1	1	0.2	0.2	0.3	0	0	0
Brazil	Tatuí	0.2	0.1	0.2	1	1	2	0.0	0.0	0.0	0	0	0
Brazil	Tauá	2.3	1.7	2.9	14	11	18	0.1	0.1	0.2	1	1	1
Brazil	Taubaté	0.2	0.1	0.2	3	2	4	0.0	0.0	0.0	1	1	1
Brazil	Tavares	1.0	0.7	1.2	1	1	2	0.1	0.1	0.1	0	0	0
Brazil	Tavares	0.1	0.1	0.2	0	0	0	0.0	0.0	0.0	0	0	0
Brazil	Tefé	0.8	0.6	0.9	6	5	8	0.2	0.2	0.3	2	1	2
Brazil	Teixeira	1.0	0.7	1.2	1	1	2	0.1	0.1	0.1	0	0	0
Brazil	Teixeira de Freitas	1.7	1.4	2.2	23	18	30	0.2	0.2	0.3	4	3	5
Brazil	Teixeiras	0.3	0.2	0.4	0	0	0	0.1	0.0	0.1	0	0	0
Brazil	Teixeirópolis	0.4	0.3	0.5	0	0	0	0.1	0.1	0.1	0	0	0
Brazil	Tejuçuoca	2.3	1.7	2.9	4	3	5	0.1	0.1	0.2	0	0	0
Brazil	Tejupa	0.2	0.1	0.2	0	0	0	0.0	0.0	0.0	0	0	0
Brazil	Telêmaco Borba	0.3	0.2	0.3	2	1	2	0.1	0.0	0.1	0	0	0
Brazil	Telha	1.3	1.0	1.7	0	0	1	0.2	0.1	0.3	0	0	0
Brazil	Tenente Ananias	1.2	0.9	1.5	1	1	1	0.1	0.1	0.2	0	0	0
Brazil	Tenente Laurentino Cruz	1.2	0.9	1.5	0	0	0	0.1	0.1	0.2	0	0	0
Brazil	Tenente Portela	0.1	0.1	0.2	0	0	0	0.0	0.0	0.0	0	0	0
Brazil	Tenório	1.0	0.7	1.2	0	0	1	0.1	0.1	0.1	0	0	0
Brazil	Teodoro Sampaio	1.7	1.4	2.2	2	1	2	0.2	0.2	0.3	0	0	0
Brazil	Teodoro Sampaio	0.2	0.1	0.2	0	0	0	0.0	0.0	0.0	0	0	0
Brazil	Teofilândia	1.7	1.4	2.2	4	3	5	0.2	0.2	0.3	1	0	1
Brazil	Teófilo Otoni	0.3	0.2	0.4	3	2	4	0.1	0.0	0.1	1	0	1
Brazil	Teolândia	1.7	1.4	2.2	3	2	3	0.2	0.2	0.3	0	0	0
Brazil	Teotônio Vilela	2.8	2.2	3.7	13	10	18	0.3	0.2	0.4	2	1	2
Brazil	Terenos	0.5	0.3	0.6	1	0	1	0.2	0.1	0.2	0	0	0
Brazil	Teresina	1.4	1.1	1.8	106	83	138	0.2	0.1	0.2	13	10	18
Brazil	Teresina de Goiás	0.2	0.1	0.2	0	0	0	0.1	0.0	0.1	0	0	0
Brazil	Teresópolis	0.2	0.1	0.3	2	2	3	0.1	0.0	0.1	1	0	1
Brazil	Terezinha	2.0	1.6	2.6	2	1	2	0.2	0.2	0.3	0	0	0
Brazil	Terezópolis de Goiás	0.2	0.1	0.2	0	0	0	0.1	0.0	0.1	0	0	0
Brazil	Terra Alta	0.9	0.7	1.1	1	1	1	0.1	0.1	0.2	0	0	0
Brazil	Terra Boa	0.3	0.2	0.3	0	0	0	0.1	0.0	0.1	0	0	0
Brazil	Terra de Areia	0.1	0.1	0.2	0	0	0	0.0	0.0	0.0	0	0	0
Brazil	Terra Nova	2.0	1.6	2.6	1	1	2	0.2	0.2	0.3	0	0	0
Brazil	Terra Nova	1.7	1.4	2.2	3	2	4	0.2	0.2	0.3	0	0	0
Brazil	Terra Nova do Norte	0.4	0.3	0.5	1	1	1	0.1	0.1	0.1	0	0	0
Brazil	Terra Rica	0.3	0.2	0.3	0	0	0	0.1	0.0	0.1	0	0	0

Table 3: Diarrhoea Mortality by Unit *(continued)*

Country	Unit	2000 mortality mean rate (per 10,000)	lower rate (per 10,000)	upper rate (per 10,000)	mean count	lower count	upper count	2017 mortality mean rate (per 10,000)	lower rate (per 10,000)	upper rate (per 10,000)	mean count	lower count	upper count
Brazil	Terra Roxa	0.3	0.2	0.3	0	0	0	0.1	0.0	0.1	0	0	0
Brazil	Terra Roxa	0.2	0.1	0.2	0	0	0	0.0	0.0	0.0	0	0	0
Brazil	Terra Santa	0.9	0.7	1.1	2	1	2	0.1	0.1	0.2	0	0	0
Brazil	Tesouro	0.4	0.3	0.5	0	0	0	0.1	0.1	0.1	0	0	0
Brazil	Teutônia	0.1	0.1	0.2	0	0	0	0.0	0.0	0.0	0	0	0
Brazil	Texeira Soares	0.3	0.2	0.3	0	0	0	0.1	0.0	0.1	0	0	0
Brazil	Theobroma	0.4	0.3	0.5	0	0	1	0.1	0.1	0.1	0	0	0
Brazil	Tianguá	2.3	1.7	2.9	18	13	23	0.1	0.1	0.2	1	1	2
Brazil	Tibaji	0.3	0.2	0.3	1	0	1	0.1	0.0	0.1	0	0	0
Brazil	Tibau	1.2	0.9	1.5	0	0	1	0.1	0.1	0.2	0	0	0
Brazil	Tibau do Sul	1.2	0.9	1.5	1	1	1	0.1	0.1	0.2	0	0	0
Brazil	Tietê	0.2	0.1	0.2	0	0	1	0.0	0.0	0.0	0	0	0
Brazil	Tigrinhos	0.2	0.2	0.3	0	0	0	0.1	0.0	0.1	0	0	0
Brazil	Tijucas	0.2	0.2	0.3	0	0	0	0.1	0.0	0.1	0	0	0
Brazil	Tijucas do Sul	0.3	0.2	0.3	0	0	0	0.1	0.0	0.1	0	0	0
Brazil	Timbaúba	2.0	1.6	2.6	14	11	18	0.2	0.2	0.3	1	1	1
Brazil	Timbaúba dos Batistas	1.2	0.9	1.5	0	0	0	0.1	0.1	0.2	0	0	0
Brazil	Timbé do Sul	0.2	0.2	0.3	0	0	0	0.1	0.0	0.1	0	0	0
Brazil	Timbiras	1.7	1.3	2.2	6	5	8	0.2	0.2	0.3	1	1	1
Brazil	Timbó	0.2	0.2	0.3	1	0	1	0.1	0.0	0.1	0	0	0
Brazil	Timbó Grande	0.2	0.2	0.3	0	0	0	0.1	0.0	0.1	0	0	0
Brazil	Timburi	0.2	0.1	0.2	0	0	0	0.0	0.0	0.0	0	0	0
Brazil	Timon	1.7	1.3	2.2	29	22	38	0.2	0.2	0.3	4	3	5
Brazil	Timóteo	0.3	0.2	0.4	2	1	2	0.1	0.0	0.1	0	0	1
Brazil	Tiradentes	0.3	0.2	0.4	0	0	0	0.1	0.0	0.1	0	0	0
Brazil	Tiradentes do Sul	0.1	0.1	0.2	0	0	0	0.0	0.0	0.0	0	0	0
Brazil	Tiros	0.3	0.2	0.4	0	0	0	0.1	0.0	0.1	0	0	0
Brazil	Tobias Barreto	1.3	1.0	1.7	6	5	8	0.2	0.1	0.3	1	1	1
Brazil	Tocantínia	0.8	0.7	1.1	1	1	1	0.1	0.1	0.2	0	0	0
Brazil	Tocantinópolis	0.8	0.7	1.1	2	2	3	0.1	0.1	0.2	0	0	0
Brazil	Tocantins	0.3	0.2	0.4	0	0	0	0.1	0.0	0.1	0	0	0
Brazil	Tocos do Moji	0.3	0.2	0.4	0	0	0	0.1	0.0	0.1	0	0	0
Brazil	Toledo	0.3	0.2	0.3	2	2	3	0.1	0.0	0.1	0	0	1
Brazil	Toledo	0.3	0.2	0.4	0	0	0	0.1	0.0	0.1	0	0	0
Brazil	Tomar do Geru	1.3	1.0	1.7	2	1	2	0.2	0.1	0.3	0	0	0
Brazil	Tomazina	0.3	0.2	0.3	0	0	0	0.1	0.0	0.1	0	0	0
Brazil	Tombos	0.3	0.2	0.4	0	0	0	0.1	0.0	0.1	0	0	0
Brazil	Tomé-Açu	0.9	0.7	1.1	5	4	7	0.1	0.1	0.2	1	1	1
Brazil	Tonantins	0.8	0.6	0.9	2	1	2	0.2	0.2	0.3	1	0	1
Brazil	Toritama	2.0	1.6	2.6	2	2	3	0.2	0.2	0.3	0	0	1
Brazil	Torixoréu	0.4	0.3	0.5	0	0	0	0.1	0.1	0.1	0	0	0
Brazil	Toropi	0.1	0.1	0.2	0	0	0	0.0	0.0	0.0	0	0	0
Brazil	Torre de Pedra	0.2	0.1	0.2	0	0	0	0.0	0.0	0.0	0	0	0
Brazil	Torres	0.1	0.1	0.2	0	0	0	0.0	0.0	0.0	0	0	0
Brazil	Torrinha	0.2	0.1	0.2	0	0	0	0.0	0.0	0.0	0	0	0
Brazil	Touros	1.2	0.9	1.5	4	3	5	0.1	0.1	0.2	0	0	1
Brazil	Trabiju	0.2	0.1	0.2	0	0	0	0.0	0.0	0.0	0	0	0
Brazil	Tracuateua	0.9	0.7	1.1	3	2	3	0.1	0.1	0.2	0	0	1
Brazil	Tracunhaém	2.0	1.6	2.6	3	3	5	0.2	0.2	0.3	0	0	0
Brazil	Traipu	2.8	2.2	3.7	9	7	12	0.3	0.2	0.4	1	1	1
Brazil	Trairão	0.9	0.7	1.1	1	1	2	0.1	0.1	0.2	0	0	0
Brazil	Trairi	2.3	1.7	2.9	13	10	17	0.1	0.1	0.2	1	1	1
Brazil	Trajano de Morais	0.2	0.1	0.3	0	0	0	0.1	0.0	0.1	0	0	0
Brazil	Tramandaí	0.1	0.1	0.2	0	0	0	0.0	0.0	0.0	0	0	0
Brazil	Travesseiro	0.1	0.1	0.2	0	0	0	0.0	0.0	0.0	0	0	0
Brazil	Tremedal	1.7	1.4	2.2	5	4	7	0.2	0.2	0.3	0	0	1
Brazil	Tremembé	0.2	0.1	0.2	0	0	1	0.0	0.0	0.0	0	0	0
Brazil	Três Arroios	0.1	0.1	0.2	0	0	0	0.0	0.0	0.0	0	0	0

Table 3: Diarrhoea Mortality by Unit *(continued)*

		2000 mortality						2017 mortality					
Country	Unit	mean rate (per 10,000)	lower rate (per 10,000)	upper rate (per 10,000)	mean count	lower count	upper count	mean rate (per 10,000)	lower rate (per 10,000)	upper rate (per 10,000)	mean count	lower count	upper count
Brazil	Três Barras do Paraná	0.3	0.2	0.3	0	0	0	0.1	0.0	0.1	0	0	0
Brazil	Três Cachoeiras	0.1	0.1	0.2	0	0	0	0.0	0.0	0.0	0	0	0
Brazil	Três Corações	0.3	0.2	0.4	2	1	2	0.1	0.0	0.1	0	0	0
Brazil	Três Coroas	0.1	0.1	0.2	0	0	0	0.0	0.0	0.0	0	0	0
Brazil	Três de Maio	0.1	0.1	0.2	0	0	0	0.0	0.0	0.0	0	0	0
Brazil	Três Forquilhas	0.1	0.1	0.2	0	0	0	0.0	0.0	0.0	0	0	0
Brazil	Três Fronteiras	0.2	0.1	0.2	0	0	0	0.0	0.0	0.0	0	0	0
Brazil	Três Lagoas	0.5	0.3	0.6	3	3	4	0.2	0.1	0.2	1	1	2
Brazil	Três Marias	0.3	0.2	0.4	1	0	1	0.1	0.0	0.1	0	0	0
Brazil	Três Palmeiras	0.1	0.1	0.2	0	0	0	0.0	0.0	0.0	0	0	0
Brazil	Três Passos	0.1	0.1	0.2	0	0	0	0.0	0.0	0.0	0	0	0
Brazil	Três Pontas	0.3	0.2	0.4	1	1	2	0.1	0.0	0.1	0	0	0
Brazil	Três Ranchos	0.2	0.1	0.2	0	0	0	0.1	0.0	0.1	0	0	0
Brazil	Três Rios	0.2	0.1	0.3	1	1	2	0.1	0.0	0.1	0	0	0
Brazil	Treviso	0.2	0.2	0.3	0	0	0	0.1	0.0	0.1	0	0	0
Brazil	Treze de Maio	0.2	0.2	0.3	0	0	0	0.1	0.0	0.1	0	0	0
Brazil	Treze Tílias	0.2	0.2	0.3	0	0	0	0.1	0.0	0.1	0	0	0
Brazil	Trindade	2.0	1.6	2.6	8	6	10	0.2	0.2	0.3	1	1	1
Brazil	Trindade	0.2	0.1	0.2	1	1	2	0.1	0.0	0.1	0	0	1
Brazil	Trindade do Sul	0.1	0.1	0.2	0	0	0	0.0	0.0	0.0	0	0	0
Brazil	Triunfo	2.0	1.6	2.6	4	3	5	0.2	0.2	0.3	0	0	0
Brazil	Triunfo	1.0	0.7	1.2	1	1	1	0.1	0.1	0.1	0	0	0
Brazil	Triunfo	0.1	0.1	0.2	0	0	0	0.0	0.0	0.0	0	0	0
Brazil	Triunfo Potiguar	1.2	0.9	1.5	0	0	1	0.1	0.1	0.2	0	0	0
Brazil	Trizidela do Vale	1.7	1.3	2.2	4	3	5	0.2	0.2	0.3	0	0	1
Brazil	Trombas	0.2	0.1	0.2	0	0	0	0.1	0.0	0.1	0	0	0
Brazil	Trombudo Central	0.2	0.2	0.3	0	0	0	0.1	0.0	0.1	0	0	0
Brazil	Tubarão	0.2	0.2	0.3	2	1	2	0.1	0.0	0.1	0	0	0
Brazil	Tucano	1.7	1.4	2.2	12	9	15	0.2	0.2	0.3	1	1	2
Brazil	Tucumã	0.9	0.7	1.1	3	2	3	0.1	0.1	0.2	1	0	1
Brazil	Tucunduva	0.1	0.1	0.2	0	0	0	0.0	0.0	0.0	0	0	0
Brazil	Tucuruí	0.9	0.7	1.1	6	5	8	0.1	0.1	0.2	1	1	2
Brazil	Tufilândia	1.7	1.3	2.2	1	1	2	0.2	0.2	0.3	0	0	0
Brazil	Tuiuti	0.2	0.1	0.2	0	0	0	0.0	0.0	0.0	0	0	0
Brazil	Tumiritinga	0.3	0.2	0.4	0	0	0	0.1	0.0	0.1	0	0	0
Brazil	Tunápolis	0.2	0.2	0.3	0	0	0	0.1	0.0	0.1	0	0	0
Brazil	Tunas	0.1	0.1	0.2	0	0	0	0.0	0.0	0.0	0	0	0
Brazil	Tunas do Paraná	0.3	0.2	0.3	0	0	0	0.1	0.0	0.1	0	0	0
Brazil	Tuneiras do Oeste	0.3	0.2	0.3	0	0	0	0.1	0.0	0.1	0	0	0
Brazil	Tuntum	1.7	1.3	2.2	9	7	11	0.2	0.2	0.3	1	1	2
Brazil	Tupã	0.2	0.1	0.2	1	0	1	0.0	0.0	0.0	0	0	0
Brazil	Tupaciguara	0.3	0.2	0.4	0	0	1	0.1	0.0	0.1	0	0	0
Brazil	Tupanatinga	2.0	1.6	2.6	5	4	7	0.2	0.2	0.3	1	0	1
Brazil	Tupanci do Sul	0.1	0.1	0.2	0	0	0	0.0	0.0	0.0	0	0	0
Brazil	Tupanciretã	0.1	0.1	0.2	0	0	0	0.0	0.0	0.0	0	0	0
Brazil	Tupandi	0.1	0.1	0.2	0	0	0	0.0	0.0	0.0	0	0	0
Brazil	Tuparendi	0.1	0.1	0.2	0	0	0	0.0	0.0	0.0	0	0	0
Brazil	Tuparetama	2.0	1.6	2.6	1	1	1	0.2	0.2	0.3	0	0	0
Brazil	Tupãssi	0.3	0.2	0.3	0	0	0	0.1	0.0	0.1	0	0	0
Brazil	Tupi Paulista	0.2	0.1	0.2	0	0	0	0.0	0.0	0.0	0	0	0
Brazil	Tupirama	0.8	0.7	1.1	2	2	3	0.1	0.1	0.2	0	0	0
Brazil	Tupiratins	0.8	0.7	1.1	0	0	0	0.1	0.1	0.2	0	0	0
Brazil	Turiaçu	1.7	1.3	2.2	8	6	10	0.2	0.2	0.3	1	1	1
Brazil	Turilândia	1.7	1.3	2.2	5	4	7	0.2	0.2	0.3	1	1	1
Brazil	Turiúba	0.2	0.1	0.2	0	0	0	0.0	0.0	0.0	0	0	0
Brazil	Turmalina	0.2	0.1	0.2	0	0	0	0.0	0.0	0.0	0	0	0
Brazil	Turmalina	0.3	0.2	0.4	0	0	1	0.1	0.0	0.1	0	0	0
Brazil	Turuçu	0.1	0.1	0.2	0	0	0	0.0	0.0	0.0	0	0	0

Table 3: Diarrhoea Mortality by Unit *(continued)*

Country	Unit	2000 mortality mean rate (per 10,000)	2000 lower rate (per 10,000)	2000 upper rate (per 10,000)	2000 mean count	2000 lower count	2000 upper count	2017 mortality mean rate (per 10,000)	2017 lower rate (per 10,000)	2017 upper rate (per 10,000)	2017 mean count	2017 lower count	2017 upper count
Brazil	Tururu	2.3	1.7	2.9	3	3	4	0.1	0.1	0.2	0	0	0
Brazil	Turvânia	0.2	0.1	0.2	0	0	0	0.1	0.0	0.1	0	0	0
Brazil	Turvelândia	0.2	0.1	0.2	0	0	0	0.1	0.0	0.1	0	0	0
Brazil	Turvo	0.2	0.2	0.3	0	0	0	0.1	0.0	0.1	0	0	0
Brazil	Turvo	0.3	0.2	0.3	0	0	1	0.1	0.0	0.1	0	0	0
Brazil	Turvolandia	0.3	0.2	0.4	0	0	0	0.1	0.0	0.1	0	0	0
Brazil	Tutoia	1.7	1.3	2.2	3	3	4	0.2	0.2	0.3	1	0	1
Brazil	Tutóia	1.7	1.3	2.2	6	5	8	0.2	0.2	0.3	1	1	2
Brazil	Uarini	0.8	0.6	0.9	1	1	1	0.2	0.2	0.3	0	0	0
Brazil	Uauá	1.7	1.4	2.2	5	4	7	0.2	0.2	0.3	1	0	1
Brazil	Ubá	0.3	0.2	0.4	2	2	3	0.1	0.0	0.1	0	0	1
Brazil	Ubaí	0.3	0.2	0.4	0	0	0	0.1	0.0	0.1	0	0	0
Brazil	Ubaíra	1.7	1.4	2.2	4	3	5	0.2	0.2	0.3	0	0	0
Brazil	Ubaitaba	1.7	1.4	2.2	7	5	9	0.2	0.2	0.3	1	1	1
Brazil	Ubajara	2.3	1.7	2.9	8	6	11	0.1	0.1	0.2	1	0	1
Brazil	Ubaporanga	0.3	0.2	0.4	0	0	0	0.1	0.0	0.1	0	0	0
Brazil	Ubarana	0.2	0.1	0.2	0	0	0	0.0	0.0	0.0	0	0	0
Brazil	Ubatã	1.7	1.4	2.2	2	2	3	0.2	0.2	0.3	0	0	0
Brazil	Ubatuba	0.2	0.1	0.2	1	0	1	0.0	0.0	0.0	0	0	0
Brazil	Uberaba	0.3	0.2	0.4	5	4	7	0.1	0.0	0.1	1	1	2
Brazil	Uberlândia	0.3	0.2	0.4	11	9	14	0.1	0.0	0.1	3	2	3
Brazil	Ubirajara	0.2	0.1	0.2	0	0	0	0.0	0.0	0.0	0	0	0
Brazil	Ubiratã	0.3	0.2	0.3	0	0	1	0.1	0.0	0.1	0	0	0
Brazil	Ubiretama	0.1	0.1	0.2	0	0	0	0.0	0.0	0.0	0	0	0
Brazil	Uchoa	0.2	0.1	0.2	0	0	0	0.0	0.0	0.0	0	0	0
Brazil	Uibaí	1.7	1.4	2.2	3	2	3	0.2	0.2	0.3	0	0	0
Brazil	Uiramutã	0.7	0.6	0.9	1	0	1	0.2	0.2	0.3	0	0	0
Brazil	Uirapuru	0.2	0.1	0.2	0	0	0	0.1	0.0	0.1	0	0	0
Brazil	Uiraúna	1.0	0.7	1.2	2	1	2	0.1	0.1	0.1	0	0	0
Brazil	Ulianópolis	0.9	0.7	1.1	2	2	3	0.1	0.1	0.2	1	1	1
Brazil	Umari	2.3	1.7	2.9	2	1	2	0.1	0.1	0.2	0	0	0
Brazil	Umarizal	1.2	0.9	1.5	1	1	2	0.1	0.1	0.2	0	0	0
Brazil	Umbauba	1.3	1.0	1.7	3	2	4	0.2	0.1	0.3	0	0	1
Brazil	Umburanas	1.7	1.4	2.2	3	2	4	0.2	0.2	0.3	0	0	1
Brazil	Umburatiba	0.3	0.2	0.4	0	0	0	0.1	0.0	0.1	0	0	0
Brazil	Umbuzeiro	1.0	0.7	1.2	1	1	1	0.1	0.1	0.1	0	0	0
Brazil	Umirim	2.3	1.7	2.9	6	4	7	0.1	0.1	0.2	0	0	0
Brazil	Umuarama	0.3	0.2	0.3	2	1	3	0.1	0.0	0.1	0	0	0
Brazil	Una	1.7	1.4	2.2	6	5	8	0.2	0.2	0.3	0	0	1
Brazil	Unaí	0.3	0.2	0.4	2	1	2	0.1	0.0	0.1	0	0	0
Brazil	União	1.4	1.1	1.8	7	5	9	0.2	0.1	0.2	1	1	1
Brazil	União da Serra	0.1	0.1	0.2	0	0	0	0.0	0.0	0.0	0	0	0
Brazil	União da Vitória	0.3	0.2	0.3	1	1	2	0.1	0.0	0.1	0	0	0
Brazil	União de Minas	0.3	0.2	0.4	0	0	0	0.1	0.0	0.1	0	0	0
Brazil	União do Oeste	0.2	0.2	0.3	0	0	0	0.1	0.0	0.1	0	0	0
Brazil	União do Sul	0.4	0.3	0.5	0	0	0	0.1	0.1	0.1	0	0	0
Brazil	União dos Palmares	2.8	2.2	3.7	23	18	31	0.3	0.2	0.4	2	2	3
Brazil	União Paulista	0.2	0.1	0.2	0	0	0	0.0	0.0	0.0	0	0	0
Brazil	Uniflor	0.3	0.2	0.3	0	0	0	0.1	0.0	0.1	0	0	0
Brazil	Unistalda	0.1	0.1	0.2	0	0	0	0.0	0.0	0.0	0	0	0
Brazil	Upanema	1.2	0.9	1.5	1	1	2	0.1	0.1	0.2	0	0	0
Brazil	Uraí	0.3	0.2	0.3	0	0	0	0.1	0.0	0.1	0	0	0
Brazil	Urandi	1.7	1.4	2.2	4	3	5	0.2	0.2	0.3	0	0	0
Brazil	Urânia	0.2	0.1	0.2	0	0	0	0.0	0.0	0.0	0	0	0
Brazil	Urbano Santos	1.7	1.3	2.2	5	4	7	0.2	0.2	0.3	1	1	1
Brazil	urea	0.1	0.1	0.2	0	0	0	0.0	0.0	0.0	0	0	0
Brazil	Uru	0.2	0.1	0.2	0	0	0	0.0	0.0	0.0	0	0	0
Brazil	Uruaçu	0.2	0.1	0.2	1	0	1	0.1	0.0	0.1	0	0	0
Brazil	Uruana	0.2	0.1	0.2	0	0	0	0.1	0.0	0.1	0	0	0

Table 3: Diarrhoea Mortality by Unit *(continued)*

Country	Unit	2000 mortality mean rate (per 10,000)	lower rate (per 10,000)	upper rate (per 10,000)	mean count	lower count	upper count	2017 mortality mean rate (per 10,000)	lower rate (per 10,000)	upper rate (per 10,000)	mean count	lower count	upper count
Brazil	Uruana de Minas	0.3	0.2	0.4	0	0	0	0.1	0.0	0.1	0	0	0
Brazil	Uruará	0.9	0.7	1.1	5	4	6	0.1	0.1	0.2	1	0	1
Brazil	Urubici	0.2	0.2	0.3	0	0	0	0.1	0.0	0.1	0	0	0
Brazil	Uruburetama	2.3	1.7	2.9	4	3	5	0.1	0.1	0.2	0	0	0
Brazil	Urucânia	0.3	0.2	0.4	0	0	0	0.1	0.0	0.1	0	0	0
Brazil	Urucará	0.8	0.6	0.9	2	1	2	0.2	0.2	0.3	0	0	0
Brazil	Uruçuca	1.7	1.4	2.2	3	2	3	0.2	0.2	0.3	0	0	0
Brazil	Uruçuí	1.4	1.1	1.8	2	2	3	0.2	0.1	0.2	0	0	0
Brazil	Urucuia	0.3	0.2	0.4	0	0	0	0.1	0.0	0.1	0	0	0
Brazil	Urucurituba	0.8	0.6	0.9	1	1	1	0.2	0.2	0.3	1	0	1
Brazil	Uruguaiana	0.1	0.1	0.2	2	1	2	0.0	0.0	0.0	0	0	1
Brazil	Uruoca	2.3	1.7	2.9	4	3	5	0.1	0.1	0.2	0	0	0
Brazil	Urupá	0.4	0.3	0.5	1	1	1	0.1	0.1	0.1	0	0	0
Brazil	Urupema	0.2	0.2	0.3	0	0	0	0.1	0.0	0.1	0	0	0
Brazil	Urupês	0.2	0.1	0.2	0	0	0	0.0	0.0	0.0	0	0	0
Brazil	Urussanga	0.2	0.2	0.3	0	0	0	0.1	0.0	0.1	0	0	0
Brazil	Urutaí	0.2	0.1	0.2	0	0	0	0.1	0.0	0.1	0	0	0
Brazil	Utinga	1.7	1.4	2.2	5	4	6	0.2	0.2	0.3	0	0	1
Brazil	Vacaria	0.1	0.1	0.2	1	0	1	0.0	0.0	0.0	0	0	0
Brazil	Vale do Anari	0.4	0.3	0.5	0	0	0	0.1	0.1	0.1	0	0	0
Brazil	Vale do Paraíso	0.4	0.3	0.5	0	0	1	0.1	0.1	0.1	0	0	0
Brazil	Vale do Sol	0.1	0.1	0.2	0	0	0	0.0	0.0	0.0	0	0	0
Brazil	Vale Real	0.1	0.1	0.2	0	0	0	0.0	0.0	0.0	0	0	0
Brazil	Vale Verde	0.1	0.1	0.2	0	0	0	0.0	0.0	0.0	0	0	0
Brazil	Valença	1.7	1.4	2.2	16	13	21	0.2	0.2	0.3	2	2	3
Brazil	Valença do Piauí	1.4	1.1	1.8	3	2	4	0.2	0.1	0.2	0	0	0
Brazil	Valencia	0.2	0.1	0.3	1	1	2	0.1	0.0	0.1	0	0	0
Brazil	Valente	1.7	1.4	2.2	3	3	4	0.2	0.2	0.3	1	0	1
Brazil	Valentim Gentil	0.2	0.1	0.2	0	0	0	0.0	0.0	0.0	0	0	0
Brazil	Valinhos	0.2	0.1	0.2	1	1	1	0.0	0.0	0.0	0	0	0
Brazil	Valparaíso	0.2	0.1	0.2	0	0	0	0.0	0.0	0.0	0	0	0
Brazil	Valparaíso de Goiás	0.2	0.1	0.2	2	2	3	0.1	0.0	0.1	1	1	1
Brazil	Vanini	0.1	0.1	0.2	0	0	0	0.0	0.0	0.0	0	0	0
Brazil	Vargeão	0.2	0.2	0.3	0	0	0	0.1	0.0	0.1	0	0	0
Brazil	Vargem	0.2	0.2	0.3	0	0	0	0.1	0.0	0.1	0	0	0
Brazil	Vargem	0.2	0.1	0.2	0	0	0	0.0	0.0	0.0	0	0	0
Brazil	Vargem Alegre	0.3	0.2	0.4	0	0	0	0.1	0.0	0.1	0	0	0
Brazil	Vargem Alta	0.3	0.2	0.3	0	0	1	0.1	0.1	0.1	0	0	0
Brazil	Vargem Bonita	0.2	0.2	0.3	0	0	0	0.1	0.0	0.1	0	0	0
Brazil	Vargem Bonita	0.3	0.2	0.4	0	0	0	0.1	0.0	0.1	0	0	0
Brazil	Vargem Grande	1.7	1.3	2.2	10	8	14	0.2	0.2	0.3	2	1	2
Brazil	Vargem Grande do Rio Pardo	0.3	0.2	0.4	0	0	0	0.1	0.0	0.1	0	0	0
Brazil	Vargem Grande do Sul	0.2	0.1	0.2	0	0	1	0.0	0.0	0.0	0	0	0
Brazil	Vargem Grande Paulista	0.2	0.1	0.2	0	0	0	0.0	0.0	0.0	0	0	0
Brazil	Varginha	0.3	0.2	0.4	3	2	3	0.1	0.0	0.1	0	0	1
Brazil	Varjao	0.2	0.1	0.2	0	0	0	0.1	0.0	0.1	0	0	0
Brazil	Varjão de Minas	0.3	0.2	0.4	0	0	0	0.1	0.0	0.1	0	0	0
Brazil	Varjota	2.3	1.7	2.9	4	3	6	0.1	0.1	0.2	0	0	0
Brazil	Varre-Sai	0.2	0.1	0.3	0	0	0	0.1	0.0	0.1	0	0	0
Brazil	Várzea	1.2	0.9	1.5	2	1	2	0.1	0.1	0.2	0	0	0
Brazil	Várzea Alegre	2.3	1.7	2.9	9	7	11	0.1	0.1	0.2	1	0	1
Brazil	Várzea Branca	1.0	0.7	1.2	1	1	1	0.1	0.1	0.1	0	0	0
Brazil	Várzea da Palma	0.3	0.2	0.4	1	1	1	0.1	0.0	0.1	0	0	0
Brazil	Várzea da Roça	1.7	1.4	2.2	3	3	4	0.2	0.2	0.3	0	0	0
Brazil	Várzea do Poço	1.7	1.4	2.2	2	1	2	0.2	0.2	0.3	0	0	0
Brazil	Várzea Grande	0.4	0.3	0.5	10	8	13	0.1	0.1	0.1	3	2	3

Table 3: Diarrhoea Mortality by Unit *(continued)*

Country	Unit	2000 mortality mean rate (per 10,000)	lower rate (per 10,000)	upper rate (per 10,000)	mean count	lower count	upper count	2017 mortality mean rate (per 10,000)	lower rate (per 10,000)	upper rate (per 10,000)	mean count	lower count	upper count
Brazil	Várzea Grande	1.4	1.1	1.8	1	1	1	0.2	0.1	0.2	0	0	0
Brazil	Várzea Nova	1.7	1.4	2.2	2	2	3	0.2	0.2	0.3	0	0	0
Brazil	Várzea Paulista	0.2	0.1	0.2	1	1	1	0.0	0.0	0.0	0	0	0
Brazil	Varzedo	1.7	1.4	2.2	2	1	2	0.2	0.2	0.3	0	0	0
Brazil	Varzelândia	0.3	0.2	0.4	1	0	1	0.1	0.0	0.1	0	0	0
Brazil	Vassouras	0.2	0.1	0.3	1	0	1	0.1	0.0	0.1	0	0	0
Brazil	Venâncio Aires	0.1	0.1	0.2	1	0	1	0.0	0.0	0.0	0	0	0
Brazil	Venceslau Bras	0.3	0.2	0.3	0	0	1	0.1	0.0	0.1	0	0	0
Brazil	Venda Nova do Imigrante	0.3	0.2	0.3	0	0	0	0.1	0.1	0.1	0	0	0
Brazil	Venha-Ver	1.2	0.9	1.5	0	0	1	0.1	0.1	0.2	0	0	0
Brazil	Ventania	0.3	0.2	0.3	0	0	0	0.1	0.0	0.1	0	0	0
Brazil	Venturosa	2.0	1.6	2.6	4	3	5	0.2	0.2	0.3	0	0	1
Brazil	Vera	0.4	0.3	0.5	0	0	1	0.1	0.1	0.1	0	0	0
Brazil	Vera Cruz	1.2	0.9	1.5	1	1	1	0.1	0.1	0.2	0	0	0
Brazil	Vera Cruz	1.7	1.4	2.2	3	2	4	0.2	0.2	0.3	0	0	1
Brazil	Vera Cruz	0.1	0.1	0.2	0	0	0	0.0	0.0	0.0	0	0	0
Brazil	Vera Cruz	0.2	0.1	0.2	0	0	0	0.0	0.0	0.0	0	0	0
Brazil	Vera Cruz do Oeste	0.3	0.2	0.3	0	0	0	0.1	0.0	0.1	0	0	0
Brazil	Vera Mendes	1.4	1.1	1.8	0	0	1	0.2	0.1	0.2	0	0	0
Brazil	Veranópolis	0.1	0.1	0.2	0	0	0	0.0	0.0	0.0	0	0	0
Brazil	Verdejante	2.0	1.6	2.6	3	2	4	0.2	0.2	0.3	0	0	0
Brazil	Verdelândia	0.3	0.2	0.4	0	0	0	0.1	0.0	0.1	0	0	0
Brazil	Verê	0.3	0.2	0.3	0	0	0	0.1	0.0	0.1	0	0	0
Brazil	Vereda	1.7	1.4	2.2	1	1	2	0.2	0.2	0.3	0	0	0
Brazil	Veredinha	0.3	0.2	0.4	0	0	0	0.1	0.0	0.1	0	0	0
Brazil	Veríssimo	0.3	0.2	0.4	0	0	0	0.1	0.0	0.1	0	0	0
Brazil	Vermelho Novo	0.3	0.2	0.4	0	0	0	0.1	0.0	0.1	0	0	0
Brazil	Vertente do Lério	2.0	1.6	2.6	2	1	2	0.2	0.2	0.3	0	0	0
Brazil	Vertentes	2.0	1.6	2.6	4	3	5	0.2	0.2	0.3	0	0	0
Brazil	Vespasiano	0.3	0.2	0.4	2	2	3	0.1	0.0	0.1	0	0	1
Brazil	Vespasiano Correa	0.1	0.1	0.2	0	0	0	0.0	0.0	0.0	0	0	0
Brazil	Viadutos	0.1	0.1	0.2	0	0	0	0.0	0.0	0.0	0	0	0
Brazil	Viamão	0.1	0.1	0.2	3	2	4	0.0	0.0	0.0	1	0	1
Brazil	Viana	1.7	1.3	2.2	11	9	15	0.2	0.2	0.3	1	1	2
Brazil	Viana	0.3	0.2	0.3	2	1	2	0.1	0.1	0.1	0	0	1
Brazil	Vianópolis	0.2	0.1	0.2	0	0	0	0.1	0.0	0.1	0	0	0
Brazil	Vicência	2.0	1.6	2.6	7	6	9	0.2	0.2	0.3	1	0	1
Brazil	Vicente Dutra	0.1	0.1	0.2	0	0	0	0.0	0.0	0.0	0	0	0
Brazil	Vicentina	0.5	0.3	0.6	0	0	0	0.2	0.1	0.2	0	0	0
Brazil	Vicentinópolis	0.2	0.1	0.2	0	0	0	0.1	0.0	0.1	0	0	0
Brazil	Viçosa	2.8	2.2	3.7	11	9	15	0.3	0.2	0.4	1	1	1
Brazil	Viçosa	1.2	0.9	1.5	0	0	0	0.1	0.1	0.2	0	0	0
Brazil	Viçosa	0.3	0.2	0.4	1	1	2	0.1	0.0	0.1	0	0	0
Brazil	Viçosa do Ceará	2.3	1.7	2.9	15	12	20	0.1	0.1	0.2	1	1	1
Brazil	Victor Graeff	0.1	0.1	0.2	0	0	0	0.0	0.0	0.0	0	0	0
Brazil	Victorino Freire	1.7	1.3	2.2	6	5	8	0.2	0.2	0.3	1	1	1
Brazil	Vidal Ramos	0.2	0.2	0.3	0	0	0	0.1	0.0	0.1	0	0	0
Brazil	Videira	0.2	0.2	0.3	1	1	1	0.1	0.0	0.1	0	0	0
Brazil	Vieiras	0.3	0.2	0.4	0	0	0	0.1	0.0	0.1	0	0	0
Brazil	Vieirópolis	1.0	0.7	1.2	0	0	1	0.1	0.1	0.1	0	0	0
Brazil	Vigia	0.9	0.7	1.1	3	2	3	0.1	0.1	0.2	0	0	1
Brazil	Vila Alta	0.3	0.2	0.3	0	0	0	0.1	0.0	0.1	0	0	0
Brazil	Vila Boa	0.2	0.1	0.2	0	0	0	0.1	0.0	0.1	0	0	0
Brazil	Vila Flor	1.2	0.9	1.5	0	0	0	0.1	0.1	0.2	0	0	0
Brazil	Vila Flores	0.1	0.1	0.2	0	0	0	0.0	0.0	0.0	0	0	0
Brazil	Vila Lângaro	0.1	0.1	0.2	0	0	0	0.0	0.0	0.0	0	0	0
Brazil	Vila Maria	0.1	0.1	0.2	0	0	0	0.0	0.0	0.0	0	0	0

Table 3: Diarrhoea Mortality by Unit *(continued)*

Country	Unit	2000 mortality mean rate (per 10,000)	lower rate (per 10,000)	upper rate (per 10,000)	mean count	lower count	upper count	2017 mortality mean rate (per 10,000)	lower rate (per 10,000)	upper rate (per 10,000)	mean count	lower count	upper count
Brazil	Vila Nova do Piauí	1.4	1.1	1.8	0	0	1	0.2	0.1	0.2	0	0	0
Brazil	Vila Nova do Sul	0.1	0.1	0.2	0	0	0	0.0	0.0	0.0	0	0	0
Brazil	Vila Nova dos Martírios	1.7	1.3	2.2	2	2	3	0.2	0.2	0.3	0	0	1
Brazil	Vila Pavão	0.3	0.2	0.3	0	0	0	0.1	0.1	0.1	0	0	0
Brazil	Vila Propício	0.2	0.1	0.2	0	0	0	0.1	0.0	0.1	0	0	0
Brazil	Vila Rica	0.4	0.3	0.5	1	1	1	0.1	0.1	0.1	0	0	0
Brazil	Vila Valério	0.3	0.2	0.3	0	0	0	0.1	0.1	0.1	0	0	0
Brazil	Vila Velha	0.3	0.2	0.3	8	6	10	0.1	0.1	0.1	2	2	3
Brazil	Vilhena	0.4	0.3	0.5	2	2	3	0.1	0.1	0.1	1	1	1
Brazil	Vinhedo	0.2	0.1	0.2	0	0	1	0.0	0.0	0.0	0	0	0
Brazil	Viradouro	0.2	0.1	0.2	0	0	0	0.0	0.0	0.0	0	0	0
Brazil	Virgem da Lapa	0.3	0.2	0.4	0	0	0	0.1	0.0	0.1	0	0	0
Brazil	Virgínia	0.3	0.2	0.4	0	0	0	0.1	0.0	0.1	0	0	0
Brazil	Virginópolis	0.3	0.2	0.4	0	0	0	0.1	0.0	0.1	0	0	0
Brazil	Virgolândia	0.3	0.2	0.4	0	0	0	0.1	0.0	0.1	0	0	0
Brazil	Virmond	0.3	0.2	0.3	0	0	0	0.1	0.0	0.1	0	0	0
Brazil	Visconde do Rio Branco	0.3	0.2	0.4	1	1	1	0.1	0.0	0.1	0	0	0
Brazil	Viseu	0.9	0.7	1.1	6	5	8	0.1	0.1	0.2	1	1	1
Brazil	Vista Alegre	0.1	0.1	0.2	0	0	0	0.0	0.0	0.0	0	0	0
Brazil	Vista Alegre do Alto	0.2	0.1	0.2	0	0	0	0.0	0.0	0.0	0	0	0
Brazil	Vista Alegre do Prata	0.1	0.1	0.2	0	0	0	0.0	0.0	0.0	0	0	0
Brazil	Vista Gaúcha	0.1	0.1	0.2	0	0	0	0.0	0.0	0.0	0	0	0
Brazil	Vitor Meireles	0.2	0.2	0.3	0	0	0	0.1	0.0	0.1	0	0	0
Brazil	Vitoria	0.3	0.2	0.3	5	4	6	0.1	0.1	0.1	1	1	2
Brazil	Vitória Brasil	0.2	0.1	0.2	0	0	0	0.0	0.0	0.0	0	0	0
Brazil	Vitória da Conquista	1.7	1.4	2.2	52	41	66	0.2	0.2	0.3	8	6	10
Brazil	Vitória das Misses	0.1	0.1	0.2	0	0	0	0.0	0.0	0.0	0	0	0
Brazil	Vitória de Santo Antão	2.0	1.6	2.6	28	22	37	0.2	0.2	0.3	3	2	4
Brazil	Vitória do Jari	0.2	0.2	0.3	0	0	0	0.1	0.1	0.2	0	0	0
Brazil	Vitória do Mearim	1.7	1.3	2.2	7	5	9	0.2	0.2	0.3	1	1	1
Brazil	Vitória do Xingu	0.9	0.7	1.1	1	1	2	0.1	0.1	0.2	0	0	0
Brazil	Vitorino	0.3	0.2	0.3	0	0	1	0.1	0.0	0.1	0	0	0
Brazil	Volta Grande	0.3	0.2	0.4	0	0	0	0.1	0.0	0.1	0	0	0
Brazil	Volta Redonda	0.2	0.1	0.3	3	3	5	0.1	0.0	0.1	1	1	1
Brazil	Votorantim	0.2	0.1	0.2	1	1	2	0.0	0.0	0.0	0	0	0
Brazil	Votuporanga	0.2	0.1	0.2	1	1	1	0.0	0.0	0.0	0	0	0
Brazil	Wagner	1.7	1.4	2.2	3	2	3	0.2	0.2	0.3	0	0	0
Brazil	Wall Ferraz	1.4	1.1	1.8	1	0	1	0.2	0.1	0.2	0	0	0
Brazil	Wanderlândia	0.8	0.7	1.1	1	1	1	0.1	0.1	0.2	0	0	0
Brazil	Wenceslau Braz	0.3	0.2	0.4	0	0	0	0.1	0.0	0.1	0	0	0
Brazil	Wenceslau Guimarães	1.7	1.4	2.2	5	4	7	0.2	0.2	0.3	1	0	1
Brazil	Witmarsum	0.2	0.2	0.3	0	0	0	0.1	0.0	0.1	0	0	0
Brazil	Xambioá	0.8	0.7	1.1	1	1	2	0.1	0.1	0.2	0	0	0
Brazil	Xambrê	0.3	0.2	0.3	0	0	0	0.1	0.0	0.1	0	0	0
Brazil	Xangri-lá	0.1	0.1	0.2	0	0	0	0.0	0.0	0.0	0	0	0
Brazil	Xanxerê	0.2	0.2	0.3	1	1	1	0.1	0.0	0.1	0	0	0
Brazil	Xapuri	1.1	0.9	1.3	2	1	2	0.2	0.2	0.3	0	0	0
Brazil	Xavantina	0.2	0.2	0.3	0	0	0	0.1	0.0	0.1	0	0	0
Brazil	Xaxim	0.2	0.2	0.3	1	0	1	0.1	0.0	0.1	0	0	0
Brazil	Xexéu	2.0	1.6	2.6	2	2	3	0.2	0.2	0.3	0	0	0

Table 3: Diarrhoea Mortality by Unit *(continued)*

Country	Unit	2000 mortality mean rate (per 10,000)	lower rate (per 10,000)	upper rate (per 10,000)	mean count	lower count	upper count	2017 mortality mean rate (per 10,000)	lower rate (per 10,000)	upper rate (per 10,000)	mean count	lower count	upper count
Brazil	Xinguara	0.9	0.7	1.1	4	3	4	0.1	0.1	0.2	1	0	1
Brazil	Xique-Xique	1.7	1.4	2.2	13	10	17	0.2	0.2	0.3	2	1	2
Brazil	Zabelê	1.0	0.7	1.2	0	0	0	0.1	0.1	0.1	0	0	0
Brazil	Zacarias	0.2	0.1	0.2	0	0	0	0.0	0.0	0.0	0	0	0
Brazil	Zé Doca	1.7	1.3	2.2	12	9	16	0.2	0.2	0.3	2	1	2
Brazil	Zortéa	0.2	0.2	0.3	0	0	0	0.1	0.0	0.1	0	0	0
Colombia	Abejorral	0.3	0.2	0.4	1	1	1	0.1	0.0	0.1	0	0	0
Colombia	Abrego	0.3	0.3	0.4	1	1	2	0.1	0.1	0.1	0	0	0
Colombia	Abriaquí	0.3	0.2	0.4	0	0	0	0.1	0.0	0.1	0	0	0
Colombia	Acacías	0.3	0.3	0.4	2	1	2	0.1	0.1	0.1	0	0	1
Colombia	Acandí	0.4	0.3	0.5	1	1	1	0.1	0.1	0.1	0	0	0
Colombia	Acevedo	0.3	0.3	0.4	1	1	1	0.1	0.1	0.1	0	0	0
Colombia	Achí	0.3	0.3	0.4	1	1	2	0.1	0.1	0.1	0	0	1
Colombia	Agrado	0.3	0.3	0.4	0	0	1	0.1	0.1	0.1	0	0	0
Colombia	Agua de Dios	0.3	0.2	0.4	0	0	0	0.1	0.1	0.1	0	0	0
Colombia	Aguachica	0.3	0.2	0.4	3	3	4	0.1	0.1	0.1	1	1	1
Colombia	Aguada	0.3	0.2	0.4	0	0	0	0.1	0.0	0.1	0	0	0
Colombia	Aguadas	0.3	0.2	0.4	1	1	1	0.1	0.1	0.1	0	0	0
Colombia	Aguazul	0.3	0.3	0.4	1	1	1	0.1	0.1	0.1	0	0	1
Colombia	Agustín Codazzi	0.3	0.2	0.4	2	2	2	0.1	0.1	0.1	0	0	0
Colombia	Aipe	0.3	0.3	0.4	1	1	1	0.1	0.1	0.1	0	0	0
Colombia	Albán	0.4	0.3	0.4	1	0	1	0.1	0.1	0.1	0	0	0
Colombia	Albán	0.3	0.3	0.4	0	0	0	0.1	0.1	0.1	0	0	0
Colombia	Albania	0.4	0.3	0.5	0	0	1	0.1	0.1	0.1	0	0	0
Colombia	Albania	0.3	0.2	0.4	0	0	0	0.1	0.0	0.1	0	0	0
Colombia	Alcalá	0.2	0.2	0.3	0	0	1	0.1	0.0	0.1	0	0	0
Colombia	Aldana	0.4	0.3	0.4	0	0	0	0.1	0.1	0.1	0	0	0
Colombia	Alejandría	0.3	0.2	0.4	0	0	0	0.1	0.0	0.1	0	0	0
Colombia	Algeciras	0.4	0.3	0.4	1	1	1	0.1	0.1	0.1	0	0	0
Colombia	Almaguer	0.4	0.3	0.5	1	1	1	0.1	0.1	0.1	0	0	0
Colombia	Almeida	0.3	0.2	0.4	0	0	0	0.1	0.0	0.1	0	0	0
Colombia	Alpujarra	0.3	0.3	0.4	0	0	0	0.1	0.1	0.1	0	0	0
Colombia	Altamira	0.3	0.3	0.4	0	0	0	0.1	0.1	0.1	0	0	0
Colombia	Alto Baudó	0.4	0.3	0.5	1	1	2	0.1	0.1	0.1	0	0	1
Colombia	Altos del Rosario	0.3	0.3	0.4	0	0	0	0.1	0.1	0.1	0	0	0
Colombia	Alvarado	0.3	0.2	0.4	0	0	1	0.1	0.1	0.1	0	0	0
Colombia	Amagá	0.3	0.3	0.4	1	1	1	0.1	0.1	0.1	0	0	0
Colombia	Amalfi	0.3	0.3	0.4	1	1	1	0.1	0.1	0.1	0	0	0
Colombia	Ambalema	0.3	0.2	0.4	0	0	0	0.1	0.1	0.1	0	0	0
Colombia	Anapoima	0.3	0.3	0.4	0	0	0	0.1	0.1	0.1	0	0	0
Colombia	Ancuyá	0.4	0.3	0.4	1	0	1	0.1	0.1	0.1	0	0	0
Colombia	Andalucía	0.2	0.2	0.3	1	1	2	0.1	0.0	0.1	0	0	0
Colombia	Andes	0.3	0.3	0.4	1	1	2	0.1	0.1	0.1	0	0	0
Colombia	Angelópolis	0.3	0.3	0.4	0	0	0	0.1	0.1	0.1	0	0	0
Colombia	Angostura	0.3	0.2	0.4	0	0	1	0.1	0.0	0.1	0	0	0
Colombia	Anolaima	0.3	0.3	0.4	0	0	1	0.1	0.1	0.1	0	0	0
Colombia	Anorí	0.3	0.3	0.4	1	0	1	0.1	0.1	0.1	0	0	0
Colombia	Anserma	0.3	0.2	0.3	1	1	1	0.1	0.0	0.1	0	0	0
Colombia	Ansermanuevo	0.2	0.2	0.3	1	0	1	0.1	0.0	0.1	0	0	0
Colombia	Anzá	0.3	0.2	0.4	0	0	0	0.1	0.0	0.1	0	0	0
Colombia	Anzoátegui	0.3	0.3	0.4	1	1	1	0.1	0.1	0.1	0	0	0
Colombia	Apartadó	0.3	0.2	0.4	4	3	5	0.1	0.1	0.1	1	1	1
Colombia	Apía	0.3	0.2	0.4	0	0	0	0.1	0.0	0.1	0	0	0
Colombia	Apulo	0.3	0.2	0.4	0	0	0	0.1	0.1	0.1	0	0	0
Colombia	Aquitania	0.3	0.3	0.4	1	1	1	0.1	0.1	0.1	0	0	0
Colombia	Aracataca	0.3	0.2	0.4	3	2	4	0.1	0.1	0.1	1	1	1
Colombia	Aranzazú	0.3	0.2	0.4	0	0	1	0.1	0.0	0.1	0	0	0
Colombia	Aratoca	0.3	0.2	0.4	0	0	0	0.1	0.0	0.1	0	0	0
Colombia	Arauca	0.3	0.3	0.4	2	2	3	0.1	0.1	0.1	1	1	1
Colombia	Arauquita	0.3	0.3	0.4	1	1	1	0.1	0.1	0.1	0	0	0

Table 3: Diarrhoea Mortality by Unit *(continued)*

Country	Unit	2000 mortality						2017 mortality					
		mean rate (per 10,000)	lower rate (per 10,000)	upper rate (per 10,000)	mean count	lower count	upper count	mean rate (per 10,000)	lower rate (per 10,000)	upper rate (per 10,000)	mean count	lower count	upper count
Colombia	Arbeláez	0.3	0.2	0.4	1	0	1	0.1	0.1	0.1	0	0	0
Colombia	Arboleda	0.4	0.3	0.4	0	0	0	0.1	0.1	0.1	0	0	0
Colombia	Arboledas	0.3	0.3	0.4	0	0	1	0.1	0.1	0.1	0	0	0
Colombia	Arboletes	0.3	0.2	0.4	1	0	1	0.1	0.1	0.1	0	0	0
Colombia	Arcabuco	0.3	0.3	0.4	0	0	0	0.1	0.0	0.1	0	0	0
Colombia	Argelia	0.3	0.2	0.4	0	0	0	0.1	0.1	0.1	0	0	0
Colombia	Argelia	0.4	0.3	0.5	1	0	1	0.1	0.1	0.1	0	0	0
Colombia	Argelia	0.2	0.2	0.3	0	0	0	0.1	0.0	0.1	0	0	0
Colombia	Ariguaní	0.3	0.2	0.4	2	1	2	0.1	0.1	0.1	0	0	0
Colombia	Arjona	0.3	0.2	0.4	2	1	2	0.1	0.1	0.1	0	0	1
Colombia	Armenia	0.3	0.2	0.3	5	4	6	0.1	0.0	0.1	1	1	1
Colombia	Armenia	0.3	0.3	0.4	0	0	0	0.1	0.1	0.1	0	0	0
Colombia	Armero	0.3	0.2	0.4	1	1	1	0.1	0.1	0.1	0	0	0
Colombia	Astrea	0.3	0.2	0.3	1	1	1	0.1	0.1	0.1	0	0	0
Colombia	Ataco	0.3	0.3	0.4	1	1	1	0.1	0.1	0.1	0	0	0
Colombia	Ayapel	0.3	0.3	0.4	2	2	3	0.1	0.1	0.1	0	0	1
Colombia	Bagadó	0.4	0.3	0.5	1	1	1	0.1	0.1	0.1	0	0	0
Colombia	Bahía Solano	0.4	0.3	0.5	1	1	1	0.1	0.1	0.1	0	0	0
Colombia	Bajo Baudó	0.4	0.3	0.5	2	1	2	0.1	0.1	0.1	0	0	0
Colombia	Balboa	0.3	0.2	0.3	0	0	0	0.1	0.0	0.1	0	0	0
Colombia	Balboa	0.4	0.3	0.4	1	1	1	0.1	0.1	0.1	0	0	0
Colombia	Baranoa	0.3	0.2	0.3	1	1	2	0.1	0.1	0.1	0	0	0
Colombia	Baraya	0.3	0.3	0.4	0	0	0	0.1	0.1	0.1	0	0	0
Colombia	Barbacoas	0.4	0.3	0.5	1	1	2	0.1	0.1	0.1	0	0	0
Colombia	Barbosa	0.3	0.2	0.4	2	1	2	0.1	0.0	0.1	0	0	0
Colombia	Barbosa	0.3	0.2	0.4	0	0	0	0.1	0.0	0.1	0	0	0
Colombia	Barichara	0.3	0.2	0.3	0	0	0	0.1	0.0	0.1	0	0	0
Colombia	Barranca de Upía	0.3	0.3	0.4	0	0	0	0.1	0.1	0.1	0	0	0
Colombia	Barrancabermeja	0.3	0.3	0.4	7	5	8	0.1	0.1	0.1	1	1	1
Colombia	Barrancas	0.3	0.3	0.4	2	2	3	0.1	0.1	0.1	1	1	1
Colombia	Barranco de Loba	0.3	0.3	0.4	1	0	1	0.1	0.1	0.1	0	0	0
Colombia	Barranco Minas	0.4	0.3	0.5	0	0	0	0.1	0.1	0.1	0	0	0
Colombia	Barranquilla	0.3	0.2	0.3	38	31	47	0.1	0.0	0.1	7	5	10
Colombia	Becerril	0.3	0.2	0.4	1	1	1	0.1	0.1	0.1	0	0	0
Colombia	Belalcázar	0.3	0.2	0.4	0	0	1	0.1	0.0	0.1	0	0	0
Colombia	Belén	0.3	0.2	0.4	0	0	0	0.1	0.1	0.1	0	0	0
Colombia	Belén	0.4	0.3	0.4	0	0	0	0.1	0.1	0.1	0	0	0
Colombia	Belén de los Andaquies	0.4	0.3	0.5	1	1	1	0.1	0.1	0.1	0	0	0
Colombia	Belén de Umbría	0.3	0.2	0.4	1	0	1	0.1	0.0	0.1	0	0	0
Colombia	Bello	0.3	0.3	0.4	12	10	15	0.1	0.0	0.1	2	1	3
Colombia	Belmira	0.3	0.2	0.4	0	0	0	0.1	0.0	0.1	0	0	0
Colombia	Beltrán	0.3	0.2	0.4	0	0	0	0.1	0.1	0.1	0	0	0
Colombia	Berbeo	0.3	0.2	0.4	0	0	0	0.1	0.1	0.1	0	0	0
Colombia	Betania	0.3	0.3	0.4	0	0	0	0.1	0.1	0.1	0	0	0
Colombia	Beteitiva	0.3	0.3	0.4	0	0	0	0.1	0.1	0.1	0	0	0
Colombia	Betulia	0.3	0.2	0.4	0	0	0	0.1	0.0	0.1	0	0	0
Colombia	Betulia	0.3	0.2	0.4	0	0	0	0.1	0.1	0.1	0	0	0
Colombia	Bituima	0.3	0.3	0.4	0	0	0	0.1	0.1	0.1	0	0	0
Colombia	Boavita	0.3	0.2	0.4	0	0	1	0.1	0.0	0.1	0	0	0
Colombia	Bochalema	0.3	0.3	0.4	0	0	0	0.1	0.1	0.1	0	0	0
Colombia	Bojacá	0.3	0.3	0.4	0	0	0	0.1	0.1	0.1	0	0	0
Colombia	Bojayá	0.4	0.3	0.5	1	0	1	0.1	0.1	0.1	0	0	0
Colombia	Bolívar	0.3	0.3	0.4	1	1	1	0.1	0.1	0.1	0	0	0
Colombia	Bolívar	0.4	0.3	0.5	3	2	3	0.1	0.1	0.1	0	0	0
Colombia	Bolívar	0.2	0.2	0.3	0	0	0	0.1	0.0	0.1	0	0	0
Colombia	Bolívar	0.3	0.3	0.4	1	1	1	0.1	0.1	0.1	0	0	0
Colombia	Bosconia	0.3	0.2	0.3	1	1	1	0.1	0.1	0.1	0	0	0
Colombia	Boyacá	0.3	0.2	0.4	0	0	0	0.1	0.0	0.1	0	0	0
Colombia	Briceño	0.3	0.3	0.4	0	0	0	0.1	0.1	0.1	0	0	0

Table 3: Diarrhoea Mortality by Unit *(continued)*

		2000 mortality						2017 mortality					
Country	Unit	mean rate (per 10,000)	lower rate (per 10,000)	upper rate (per 10,000)	mean count	lower count	upper count	mean rate (per 10,000)	lower rate (per 10,000)	upper rate (per 10,000)	mean count	lower count	upper count
Colombia	Briceño	0.3	0.3	0.4	0	0	0	0.1	0.1	0.1	0	0	0
Colombia	Bucaramanga	0.3	0.2	0.4	15	13	20	0.1	0.1	0.1	3	2	4
Colombia	Bucarasica	0.3	0.3	0.4	0	0	0	0.1	0.1	0.1	0	0	0
Colombia	Buenaventura	0.3	0.3	0.4	10	8	12	0.1	0.1	0.1	2	2	3
Colombia	Buenavista	0.3	0.3	0.4	0	0	0	0.1	0.1	0.1	0	0	0
Colombia	Buenavista	0.3	0.2	0.4	0	0	0	0.1	0.1	0.1	0	0	0
Colombia	Buenavista	0.3	0.2	0.4	1	1	1	0.1	0.1	0.1	0	0	0
Colombia	Buenavista	0.3	0.2	0.3	0	0	0	0.1	0.0	0.1	0	0	0
Colombia	Buenos Aires	0.4	0.3	0.4	2	1	2	0.1	0.1	0.1	0	0	0
Colombia	Buesaco	0.4	0.3	0.4	1	1	2	0.1	0.1	0.1	0	0	0
Colombia	Bugalagrande	0.2	0.2	0.3	1	1	1	0.1	0.0	0.1	0	0	0
Colombia	Buriticá	0.3	0.2	0.4	0	0	0	0.1	0.0	0.1	0	0	0
Colombia	Busbanza	0.3	0.3	0.4	0	0	0	0.1	0.1	0.1	0	0	0
Colombia	Cabrera	0.3	0.3	0.4	0	0	0	0.1	0.1	0.1	0	0	0
Colombia	Cabrera	0.3	0.2	0.3	0	0	0	0.1	0.0	0.1	0	0	0
Colombia	Cabuyaro	0.3	0.3	0.4	0	0	0	0.1	0.1	0.1	0	0	0
Colombia	Cacahual	0.4	0.3	0.5	0	0	0	0.1	0.1	0.1	0	0	0
Colombia	Cáceres	0.3	0.3	0.4	1	1	2	0.1	0.1	0.1	0	0	0
Colombia	Cachipay	0.3	0.2	0.4	0	0	0	0.1	0.1	0.1	0	0	0
Colombia	Cáchira	0.3	0.2	0.4	0	0	1	0.1	0.1	0.1	0	0	0
Colombia	Cácota	0.3	0.2	0.4	0	0	0	0.1	0.0	0.1	0	0	0
Colombia	Caicedo	0.3	0.3	0.4	0	0	0	0.1	0.1	0.1	0	0	0
Colombia	Caicedonia	0.2	0.2	0.3	1	0	1	0.1	0.0	0.1	0	0	0
Colombia	Caimito	0.3	0.3	0.4	0	0	1	0.1	0.1	0.1	0	0	0
Colombia	Cajamarca	0.3	0.2	0.4	1	0	1	0.1	0.1	0.1	0	0	0
Colombia	Cajibío	0.4	0.3	0.4	1	1	2	0.1	0.1	0.1	0	0	0
Colombia	Cajicá	0.3	0.3	0.4	1	1	2	0.1	0.1	0.1	0	0	0
Colombia	Calamar	0.4	0.3	0.5	0	0	1	0.1	0.1	0.1	0	0	0
Colombia	Calamar	0.3	0.2	0.4	1	1	1	0.1	0.1	0.1	0	0	0
Colombia	Calarcá	0.3	0.2	0.3	3	2	3	0.1	0.0	0.1	1	0	1
Colombia	Caldas	0.3	0.3	0.4	0	0	0	0.1	0.0	0.1	0	0	0
Colombia	Caldas	0.3	0.3	0.4	2	2	3	0.1	0.1	0.1	0	0	1
Colombia	Caldonó	0.4	0.3	0.4	2	1	2	0.1	0.1	0.1	0	0	0
Colombia	California	0.3	0.2	0.4	0	0	0	0.1	0.0	0.1	0	0	0
Colombia	Calima	0.3	0.2	0.3	0	0	0	0.1	0.0	0.1	0	0	0
Colombia	Caloto	0.3	0.3	0.4	2	2	3	0.1	0.1	0.1	0	0	1
Colombia	Campamento	0.3	0.2	0.4	0	0	0	0.1	0.1	0.1	0	0	0
Colombia	Campo de la Cruz	0.3	0.2	0.4	1	1	1	0.1	0.1	0.1	0	0	0
Colombia	Campoalegre	0.3	0.2	0.4	1	1	1	0.1	0.1	0.1	0	0	0
Colombia	Campohermoso	0.3	0.3	0.4	0	0	0	0.1	0.1	0.1	0	0	0
Colombia	Canalete	0.3	0.2	0.3	1	1	1	0.1	0.0	0.1	0	0	0
Colombia	Cañasgordas	0.3	0.3	0.4	1	0	1	0.1	0.1	0.1	0	0	0
Colombia	Candelaria	0.3	0.2	0.4	8	7	10	0.1	0.0	0.1	2	1	2
Colombia	Candelaria	0.3	0.2	0.3	0	0	1	0.1	0.1	0.1	0	0	0
Colombia	Cantagallo	0.4	0.3	0.4	0	0	0	0.1	0.1	0.1	0	0	0
Colombia	Caparrapí	0.3	0.3	0.4	1	0	1	0.1	0.1	0.1	0	0	0
Colombia	Capitanejo	0.3	0.2	0.4	0	0	0	0.1	0.0	0.1	0	0	0
Colombia	Cáqueza	0.3	0.2	0.4	1	0	1	0.1	0.0	0.1	0	0	0
Colombia	Caracolí	0.3	0.3	0.4	0	0	0	0.1	0.1	0.1	0	0	0
Colombia	Caramanta	0.3	0.2	0.4	0	0	0	0.1	0.0	0.1	0	0	0
Colombia	Carcasí	0.3	0.2	0.4	0	0	0	0.1	0.0	0.1	0	0	0
Colombia	Carepa	0.3	0.2	0.4	1	1	1	0.1	0.1	0.1	0	0	0
Colombia	Carmen de Apicalá	0.3	0.2	0.4	0	0	0	0.1	0.1	0.1	0	0	0
Colombia	Carmen de Carupa	0.3	0.2	0.4	0	0	0	0.1	0.0	0.1	0	0	0
Colombia	Carolina del Principe	0.3	0.2	0.4	0	0	0	0.1	0.0	0.1	0	0	0
Colombia	Cartagena de Indias	0.3	0.2	0.4	32	25	40	0.1	0.1	0.1	7	5	9

Table 3: Diarrhoea Mortality by Unit *(continued)*

Country	Unit	2000 mortality mean rate (per 10,000)	lower rate (per 10,000)	upper rate (per 10,000)	mean count	lower count	upper count	2017 mortality mean rate (per 10,000)	lower rate (per 10,000)	upper rate (per 10,000)	mean count	lower count	upper count
Colombia	Cartagena del Chairá	0.4	0.3	0.5	2	2	2	0.1	0.1	0.1	0	0	0
Colombia	Cartago	0.2	0.2	0.3	3	2	3	0.1	0.0	0.1	0	0	1
Colombia	Carurú	0.5	0.4	0.6	0	0	0	0.1	0.1	0.1	0	0	0
Colombia	Casabianca	0.3	0.3	0.4	0	0	0	0.1	0.1	0.1	0	0	0
Colombia	Castilla la Nueva	0.3	0.2	0.4	0	0	0	0.1	0.1	0.1	0	0	0
Colombia	Caucasia	0.3	0.3	0.4	2	2	3	0.1	0.1	0.1	1	0	1
Colombia	Cepitá	0.3	0.2	0.4	0	0	0	0.1	0.0	0.1	0	0	0
Colombia	Cereté	0.3	0.2	0.4	3	2	4	0.1	0.0	0.1	1	0	1
Colombia	Cerinza	0.3	0.3	0.4	0	0	0	0.1	0.1	0.1	0	0	0
Colombia	Cerrito	0.3	0.2	0.4	0	0	0	0.1	0.0	0.1	0	0	0
Colombia	Cerro de San Antonio	0.3	0.2	0.4	1	1	1	0.1	0.1	0.1	0	0	0
Colombia	Chachagüí	0.4	0.3	0.4	0	0	0	0.1	0.1	0.1	0	0	0
Colombia	Chaguaní	0.3	0.3	0.4	0	0	0	0.1	0.1	0.1	0	0	0
Colombia	Chalán	0.3	0.2	0.4	0	0	0	0.1	0.1	0.1	0	0	0
Colombia	Chámeza	0.3	0.3	0.4	0	0	0	0.1	0.1	0.1	0	0	0
Colombia	Chaparral	0.3	0.3	0.4	2	1	2	0.1	0.1	0.1	0	0	0
Colombia	Charalá	0.3	0.2	0.4	0	0	1	0.1	0.0	0.1	0	0	0
Colombia	Charta	0.3	0.2	0.3	0	0	0	0.1	0.0	0.1	0	0	0
Colombia	Chía	0.3	0.3	0.4	4	3	5	0.1	0.1	0.1	1	1	1
Colombia	Chigorodó	0.3	0.2	0.4	2	2	3	0.1	0.1	0.1	0	0	1
Colombia	Chimá	0.3	0.2	0.4	0	0	0	0.1	0.0	0.1	0	0	0
Colombia	Chimá	0.3	0.2	0.4	1	1	1	0.1	0.1	0.1	0	0	0
Colombia	Chimichagua	0.3	0.3	0.4	2	1	2	0.1	0.1	0.1	0	0	0
Colombia	Chinácota	0.3	0.3	0.4	1	0	1	0.1	0.1	0.1	0	0	0
Colombia	Chinavita	0.3	0.2	0.4	0	0	0	0.1	0.1	0.1	0	0	0
Colombia	Chinchiná	0.3	0.2	0.4	1	1	1	0.1	0.0	0.1	0	0	0
Colombia	Chinú	0.3	0.2	0.4	2	1	2	0.1	0.1	0.1	0	0	1
Colombia	Chipaque	0.3	0.2	0.4	1	1	1	0.1	0.0	0.1	0	0	0
Colombia	Chipatá	0.3	0.2	0.4	0	0	0	0.1	0.0	0.1	0	0	0
Colombia	Chiquinquirá	0.3	0.2	0.4	1	1	1	0.1	0.0	0.1	0	0	0
Colombia	Chíquiza	0.3	0.3	0.4	0	0	0	0.1	0.1	0.1	0	0	0
Colombia	Chiriguaná	0.3	0.2	0.4	1	1	1	0.1	0.1	0.1	0	0	0
Colombia	Chiscas	0.3	0.3	0.4	0	0	0	0.1	0.1	0.1	0	0	0
Colombia	Chita	0.3	0.3	0.4	0	0	1	0.1	0.1	0.1	0	0	0
Colombia	Chitagá	0.3	0.3	0.4	0	0	1	0.1	0.1	0.1	0	0	0
Colombia	Chitaraque	0.3	0.2	0.4	0	0	0	0.1	0.0	0.1	0	0	0
Colombia	Chivatá	0.3	0.2	0.4	0	0	0	0.1	0.0	0.1	0	0	0
Colombia	Chivolo	0.3	0.2	0.4	1	1	1	0.1	0.1	0.1	0	0	0
Colombia	Chivor	0.3	0.3	0.4	0	0	0	0.1	0.1	0.1	0	0	0
Colombia	Choachí	0.3	0.2	0.4	2	1	2	0.1	0.1	0.1	0	0	0
Colombia	Chocontá	0.3	0.2	0.4	1	1	1	0.1	0.0	0.1	0	0	0
Colombia	Cicuco	0.3	0.3	0.4	6	4	7	0.1	0.1	0.1	1	1	1
Colombia	Ciénaga	0.3	0.2	0.4	0	0	0	0.1	0.0	0.1	0	0	0
Colombia	Ciénaga	0.3	0.3	0.4	8	6	9	0.1	0.1	0.1	2	1	2
Colombia	Ciénaga de Oro	0.3	0.2	0.4	2	1	2	0.1	0.1	0.1	0	0	1
Colombia	Cimitarra	0.3	0.3	0.4	1	1	2	0.1	0.1	0.1	0	0	0
Colombia	Circasia	0.3	0.2	0.3	1	0	1	0.1	0.0	0.1	0	0	0
Colombia	Cisneros	0.3	0.3	0.4	0	0	0	0.1	0.1	0.1	0	0	0
Colombia	Cocorná	0.3	0.2	0.4	1	0	1	0.1	0.1	0.1	0	0	0
Colombia	Coello	0.3	0.2	0.4	1	1	1	0.1	0.1	0.1	0	0	0
Colombia	Cogua	0.3	0.2	0.4	1	1	1	0.1	0.0	0.1	0	0	0
Colombia	Colombia	0.3	0.3	0.4	1	0	1	0.1	0.1	0.1	0	0	0
Colombia	Colón	0.4	0.3	0.5	0	0	0	0.1	0.1	0.1	0	0	0
Colombia	Colón	0.4	0.3	0.4	0	0	0	0.1	0.1	0.1	0	0	0
Colombia	Colosó	0.3	0.2	0.4	0	0	0	0.1	0.1	0.1	0	0	0
Colombia	Cómbita	0.3	0.2	0.4	1	1	1	0.1	0.0	0.1	0	0	0
Colombia	Concepción	0.3	0.2	0.4	0	0	1	0.1	0.0	0.1	0	0	0
Colombia	Concepción	0.3	0.2	0.4	0	0	0	0.1	0.0	0.1	0	0	0

Table 3: Diarrhoea Mortality by Unit *(continued)*

Country	Unit	2000 mortality mean rate (per 10,000)	2000 mortality lower rate (per 10,000)	2000 mortality upper rate (per 10,000)	2000 mortality mean count	2000 mortality lower count	2000 mortality upper count	2017 mortality mean rate (per 10,000)	2017 mortality lower rate (per 10,000)	2017 mortality upper rate (per 10,000)	2017 mortality mean count	2017 mortality lower count	2017 mortality upper count
Colombia	Concordia	0.3	0.2	0.4	1	0	1	0.1	0.0	0.1	0	0	0
Colombia	Condoto	0.4	0.3	0.5	1	1	2	0.1	0.1	0.1	0	0	0
Colombia	Confines	0.3	0.2	0.4	0	0	0	0.1	0.0	0.1	0	0	0
Colombia	Consacá	0.4	0.3	0.4	1	1	1	0.1	0.1	0.1	0	0	0
Colombia	Contadero	0.3	0.3	0.4	0	0	0	0.1	0.1	0.1	0	0	0
Colombia	Contratación	0.3	0.2	0.4	0	0	0	0.1	0.0	0.1	0	0	0
Colombia	Convención	0.3	0.3	0.4	1	1	1	0.1	0.1	0.1	0	0	0
Colombia	Copacabana	0.3	0.3	0.4	4	3	5	0.1	0.1	0.1	1	1	1
Colombia	Coper	0.3	0.3	0.4	0	0	0	0.1	0.1	0.1	0	0	0
Colombia	Córdoba	0.4	0.3	0.4	1	1	1	0.1	0.1	0.1	0	0	0
Colombia	Córdoba	0.3	0.2	0.3	0	0	0	0.1	0.0	0.1	0	0	0
Colombia	Córdoba	0.3	0.2	0.4	1	0	1	0.1	0.1	0.1	0	0	0
Colombia	Corinto	0.3	0.3	0.4	1	1	1	0.1	0.1	0.1	0	0	0
Colombia	Coromoro	0.3	0.3	0.4	0	0	0	0.1	0.1	0.1	0	0	0
Colombia	Corozal	0.3	0.2	0.4	2	2	3	0.1	0.1	0.1	0	0	1
Colombia	Corrales	0.3	0.3	0.4	1	1	1	0.1	0.1	0.1	0	0	0
Colombia	Cota	0.3	0.3	0.4	1	1	2	0.1	0.1	0.1	0	0	0
Colombia	Covarachía	0.3	0.2	0.4	0	0	0	0.1	0.0	0.1	0	0	0
Colombia	Coyaima	0.3	0.2	0.4	1	1	1	0.1	0.1	0.1	0	0	0
Colombia	Cravo Norte	0.3	0.3	0.4	0	0	0	0.1	0.1	0.1	0	0	0
Colombia	Cuaspud	0.4	0.3	0.4	1	0	1	0.1	0.1	0.1	0	0	0
Colombia	Cubará	0.4	0.3	0.5	0	0	0	0.1	0.1	0.1	0	0	0
Colombia	Cucaita	0.3	0.2	0.3	0	0	0	0.1	0.0	0.1	0	0	0
Colombia	Cucunubá	0.3	0.2	0.4	0	0	0	0.1	0.0	0.1	0	0	0
Colombia	Cucutilla	0.3	0.3	0.4	0	0	0	0.1	0.1	0.1	0	0	0
Colombia	Cuítiva	0.3	0.3	0.4	0	0	0	0.1	0.1	0.1	0	0	0
Colombia	Cumaral	0.3	0.3	0.4	0	0	0	0.1	0.1	0.1	0	0	0
Colombia	Cumaribo	0.4	0.3	0.5	1	0	1	0.1	0.1	0.1	0	0	0
Colombia	Cumbal	0.4	0.3	0.4	1	1	1	0.1	0.1	0.1	0	0	0
Colombia	Cumbitara	0.4	0.3	0.4	0	0	0	0.1	0.1	0.1	0	0	0
Colombia	Cunday	0.3	0.3	0.4	0	0	1	0.1	0.1	0.1	0	0	0
Colombia	Curillo	0.4	0.3	0.5	1	0	1	0.1	0.1	0.1	0	0	0
Colombia	Curití	0.3	0.2	0.4	0	0	1	0.1	0.0	0.1	0	0	0
Colombia	Curumaní	0.3	0.2	0.4	1	1	2	0.1	0.1	0.1	0	0	0
Colombia	Dabeiba	0.3	0.3	0.4	1	1	1	0.1	0.1	0.1	0	0	0
Colombia	Dagua	0.3	0.2	0.3	1	1	1	0.1	0.0	0.1	0	0	0
Colombia	Dolores	0.3	0.3	0.4	0	0	0	0.1	0.1	0.1	0	0	0
Colombia	Don Matías	0.3	0.2	0.4	1	0	1	0.1	0.0	0.1	0	0	0
Colombia	Dosquebradas	0.3	0.2	0.3	4	3	5	0.1	0.0	0.1	1	1	1
Colombia	Duitama	0.3	0.3	0.4	4	3	5	0.1	0.1	0.1	1	0	1
Colombia	Durania	0.3	0.3	0.4	0	0	0	0.1	0.1	0.1	0	0	0
Colombia	Ebéjico	0.3	0.2	0.4	0	0	0	0.1	0.1	0.1	0	0	0
Colombia	El Águila	0.3	0.2	0.3	0	0	0	0.1	0.0	0.1	0	0	0
Colombia	El Bagre	0.3	0.3	0.4	2	1	2	0.1	0.1	0.1	0	0	0
Colombia	El Banco	0.3	0.3	0.4	3	2	3	0.1	0.1	0.1	0	0	1
Colombia	El Cairo	0.3	0.2	0.4	0	0	0	0.1	0.1	0.1	0	0	0
Colombia	El Calvario	0.3	0.3	0.4	0	0	0	0.1	0.1	0.1	0	0	0
Colombia	El Cantón del San Pablo	0.4	0.3	0.5	1	1	1	0.1	0.1	0.1	0	0	0
Colombia	El Carmen	0.3	0.3	0.4	1	1	1	0.1	0.1	0.1	0	0	0
Colombia	El Carmen de Atrato	0.4	0.3	0.5	1	1	1	0.1	0.1	0.1	0	0	0
Colombia	El Carmen de Bolívar	0.3	0.2	0.4	3	2	3	0.1	0.1	0.1	0	0	1
Colombia	El Carmen de Chucurí	0.3	0.2	0.4	1	0	1	0.1	0.1	0.1	0	0	0
Colombia	El Carmen de Viboral	0.3	0.3	0.4	1	1	2	0.1	0.1	0.1	0	0	0
Colombia	El Castillo	0.3	0.3	0.4	1	0	1	0.1	0.1	0.1	0	0	0
Colombia	El Cerrito	0.3	0.2	0.3	2	1	2	0.1	0.0	0.1	0	0	0

Table 3: Diarrhoea Mortality by Unit *(continued)*

Country	Unit	2000 mortality mean rate (per 10,000)	lower rate (per 10,000)	upper rate (per 10,000)	mean count	lower count	upper count	2017 mortality mean rate (per 10,000)	lower rate (per 10,000)	upper rate (per 10,000)	mean count	lower count	upper count
Colombia	El Charco	0.4	0.3	0.5	1	1	1	0.1	0.1	0.1	0	0	0
Colombia	El Cocuy	0.3	0.3	0.4	0	0	0	0.1	0.1	0.1	0	0	0
Colombia	El Colegio	0.3	0.3	0.4	1	1	1	0.1	0.1	0.1	0	0	0
Colombia	El Copey	0.3	0.2	0.4	1	1	1	0.1	0.1	0.1	0	0	0
Colombia	El Doncello	0.4	0.3	0.4	2	1	2	0.1	0.1	0.1	0	0	0
Colombia	El Dorado	0.3	0.3	0.4	0	0	0	0.1	0.1	0.1	0	0	0
Colombia	El Dovio	0.2	0.2	0.3	0	0	0	0.1	0.0	0.1	0	0	0
Colombia	El Encanto	0.5	0.4	0.7	0	0	0	0.1	0.1	0.1	0	0	0
Colombia	El Espino	0.3	0.3	0.4	0	0	0	0.1	0.0	0.1	0	0	0
Colombia	El Guacamayo	0.3	0.2	0.4	0	0	0	0.1	0.0	0.1	0	0	0
Colombia	El Guamo	0.3	0.2	0.4	0	0	0	0.1	0.1	0.1	0	0	0
Colombia	El Litoral del San Juan	0.4	0.3	0.5	1	1	2	0.1	0.1	0.1	0	0	0
Colombia	El Molino	0.3	0.3	0.4	0	0	0	0.1	0.1	0.1	0	0	0
Colombia	El Paso	0.3	0.2	0.3	1	1	1	0.1	0.1	0.1	0	0	0
Colombia	El Paujíl	0.4	0.3	0.4	1	1	1	0.1	0.1	0.1	0	0	0
Colombia	El Peñon	0.3	0.3	0.4	0	0	0	0.1	0.1	0.1	0	0	0
Colombia	El Peñon	0.3	0.3	0.4	0	0	0	0.1	0.1	0.1	0	0	0
Colombia	El Piñón	0.3	0.2	0.4	1	1	1	0.1	0.1	0.1	0	0	0
Colombia	El Playón	0.3	0.3	0.4	0	0	0	0.1	0.1	0.1	0	0	0
Colombia	El Retorno	0.4	0.3	0.5	1	0	1	0.1	0.1	0.1	0	0	0
Colombia	El Rosario	0.4	0.3	0.4	0	0	0	0.1	0.1	0.1	0	0	0
Colombia	El Santuario	0.3	0.3	0.4	1	1	1	0.1	0.1	0.1	0	0	0
Colombia	El Tablón de Gomez	0.4	0.3	0.5	2	2	2	0.1	0.1	0.1	0	0	0
Colombia	El Tambo	0.4	0.3	0.5	2	2	2	0.1	0.1	0.1	0	0	0
Colombia	El Tambo	0.4	0.3	0.4	1	1	1	0.1	0.1	0.1	0	0	0
Colombia	El Tarra	0.3	0.3	0.4	0	0	1	0.1	0.1	0.1	0	0	0
Colombia	El Zulia	0.3	0.2	0.4	0	0	0	0.1	0.1	0.1	0	0	0
Colombia	Elías	0.3	0.3	0.4	0	0	0	0.1	0.1	0.1	0	0	0
Colombia	Encino	0.3	0.2	0.4	0	0	0	0.1	0.1	0.1	0	0	0
Colombia	Enciso	0.3	0.2	0.4	0	0	1	0.1	0.0	0.1	0	0	0
Colombia	Entrerríos	0.3	0.2	0.4	0	0	0	0.1	0.0	0.1	0	0	0
Colombia	Envigado	0.3	0.3	0.4	8	6	10	0.1	0.1	0.1	1	1	2
Colombia	Espinal	0.3	0.2	0.4	2	2	3	0.1	0.1	0.1	0	0	0
Colombia	Facatativá	0.3	0.2	0.4	3	2	3	0.1	0.1	0.1	1	1	1
Colombia	Falán	0.3	0.3	0.4	1	1	1	0.1	0.1	0.1	0	0	0
Colombia	Filadelfia	0.3	0.2	0.4	0	0	1	0.1	0.0	0.1	0	0	0
Colombia	Filandia	0.3	0.2	0.3	1	1	1	0.1	0.0	0.1	0	0	0
Colombia	Firavitoba	0.3	0.2	0.4	0	0	0	0.1	0.0	0.1	0	0	0
Colombia	Flandes	0.3	0.2	0.4	1	1	1	0.1	0.1	0.1	0	0	0
Colombia	Florencia	0.4	0.3	0.4	0	0	0	0.1	0.1	0.1	0	0	0
Colombia	Florencia	0.4	0.3	0.4	6	5	8	0.1	0.1	0.1	1	1	2
Colombia	Floresta	0.3	0.3	0.4	0	0	1	0.1	0.1	0.1	0	0	0
Colombia	Florián	0.3	0.3	0.4	0	0	0	0.1	0.1	0.1	0	0	0
Colombia	Florida	0.3	0.3	0.4	2	2	3	0.1	0.1	0.1	0	0	1
Colombia	Floridablanca	0.3	0.3	0.4	8	7	10	0.1	0.1	0.1	2	1	2
Colombia	Fómeque	0.3	0.3	0.4	1	0	1	0.1	0.1	0.1	0	0	0
Colombia	Fonseca	0.3	0.3	0.4	2	1	2	0.1	0.1	0.1	0	0	1
Colombia	Fortul	0.4	0.3	0.4	1	1	1	0.1	0.1	0.1	0	0	0
Colombia	Fosca	0.3	0.3	0.4	0	0	0	0.1	0.1	0.1	0	0	0
Colombia	Francisco Pizarro	0.4	0.3	0.4	1	1	1	0.1	0.1	0.1	0	0	0
Colombia	Fredonia	0.3	0.3	0.4	1	1	1	0.1	0.1	0.1	0	0	0
Colombia	Fresno	0.3	0.3	0.4	1	1	1	0.1	0.1	0.1	0	0	0
Colombia	Frontino	0.3	0.3	0.4	1	1	1	0.1	0.1	0.1	0	0	0
Colombia	Fuente de Oro	0.3	0.3	0.4	0	0	1	0.1	0.1	0.1	0	0	0
Colombia	Fundación	0.3	0.2	0.4	2	1	2	0.1	0.1	0.1	0	0	0
Colombia	Funes	0.4	0.3	0.4	1	1	1	0.1	0.1	0.1	0	0	0
Colombia	Funza	0.3	0.3	0.4	2	2	2	0.1	0.1	0.1	1	1	1
Colombia	Fúquene	0.3	0.2	0.4	0	0	0	0.1	0.0	0.1	0	0	0

Table 3: Diarrhoea Mortality by Unit *(continued)*

Country	Unit	2000 mortality mean rate (per 10,000)	lower rate (per 10,000)	upper rate (per 10,000)	mean count	lower count	upper count	2017 mortality mean rate (per 10,000)	lower rate (per 10,000)	upper rate (per 10,000)	mean count	lower count	upper count
Colombia	Fusagasugá	0.3	0.3	0.4	4	3	4	0.1	0.1	0.1	1	1	1
Colombia	Gachalá	0.3	0.3	0.4	0	0	0	0.1	0.1	0.1	0	0	0
Colombia	Gachancipá	0.3	0.3	0.4	0	0	0	0.1	0.1	0.1	0	0	0
Colombia	Gachantivá	0.3	0.2	0.4	0	0	0	0.1	0.0	0.1	0	0	0
Colombia	Gachetá	0.3	0.3	0.4	1	0	1	0.1	0.1	0.1	0	0	0
Colombia	Galán	0.3	0.2	0.4	0	0	0	0.1	0.0	0.1	0	0	0
Colombia	Galapa	0.3	0.2	0.4	3	2	4	0.1	0.1	0.1	1	1	1
Colombia	Galeras	0.3	0.2	0.4	1	0	1	0.1	0.1	0.1	0	0	0
Colombia	Gama	0.3	0.3	0.4	0	0	0	0.1	0.1	0.1	0	0	0
Colombia	Gamarra	0.3	0.2	0.4	1	1	1	0.1	0.1	0.1	0	0	0
Colombia	Gámbita	0.3	0.2	0.4	0	0	0	0.1	0.0	0.1	0	0	0
Colombia	Gámeza	0.3	0.3	0.4	0	0	0	0.1	0.1	0.1	0	0	0
Colombia	Garagoa	0.3	0.2	0.4	1	1	1	0.1	0.0	0.1	0	0	0
Colombia	Garzón	0.3	0.3	0.4	3	2	3	0.1	0.1	0.1	1	1	1
Colombia	Génova	0.3	0.2	0.3	0	0	0	0.1	0.0	0.1	0	0	0
Colombia	Gigante	0.3	0.3	0.4	1	1	1	0.1	0.1	0.1	0	0	0
Colombia	Ginebra	0.3	0.2	0.3	1	0	1	0.1	0.0	0.1	0	0	0
Colombia	Giraldo	0.3	0.2	0.4	0	0	0	0.1	0.0	0.1	0	0	0
Colombia	Girardot	0.3	0.2	0.4	2	2	3	0.1	0.1	0.1	0	0	0
Colombia	Girardota	0.3	0.2	0.4	2	1	2	0.1	0.1	0.1	0	0	0
Colombia	Girón	0.3	0.2	0.4	3	2	3	0.1	0.1	0.1	1	1	1
Colombia	Gómez Plata	0.3	0.2	0.4	0	0	1	0.1	0.0	0.1	0	0	0
Colombia	González	0.3	0.3	0.4	0	0	1	0.1	0.1	0.1	0	0	0
Colombia	Gramalote	0.3	0.3	0.4	0	0	0	0.1	0.1	0.1	0	0	0
Colombia	Granada	0.3	0.3	0.4	1	1	2	0.1	0.1	0.1	0	0	0
Colombia	Granada	0.3	0.2	0.4	0	0	1	0.1	0.0	0.1	0	0	0
Colombia	Guaca	0.3	0.2	0.4	0	0	0	0.1	0.0	0.1	0	0	0
Colombia	Guacamayas	0.3	0.3	0.4	0	0	0	0.1	0.1	0.1	0	0	0
Colombia	Guacarí	0.2	0.2	0.3	1	1	1	0.0	0.0	0.1	0	0	0
Colombia	Guachetá	0.3	0.2	0.4	0	0	1	0.1	0.0	0.1	0	0	0
Colombia	Guachucal	0.4	0.3	0.4	0	0	0	0.1	0.1	0.1	0	0	0
Colombia	Guadalajara de Buga	0.2	0.2	0.3	3	2	3	0.0	0.0	0.1	0	0	1
Colombia	Guadalupe	0.3	0.3	0.4	1	1	1	0.1	0.1	0.1	0	0	0
Colombia	Guadalupe	0.3	0.2	0.4	0	0	0	0.1	0.0	0.1	0	0	0
Colombia	Guadalupe	0.3	0.2	0.4	0	0	0	0.1	0.0	0.1	0	0	0
Colombia	Guaduas	0.3	0.3	0.4	2	1	2	0.1	0.1	0.1	0	0	0
Colombia	Guaitarilla	0.4	0.3	0.4	1	1	1	0.1	0.1	0.1	0	0	0
Colombia	Gualmatán	0.3	0.3	0.4	0	0	0	0.1	0.1	0.1	0	0	0
Colombia	Guamal	0.3	0.3	0.4	0	0	1	0.1	0.1	0.1	0	0	0
Colombia	Guamal	0.3	0.3	0.4	1	1	2	0.1	0.1	0.1	0	0	0
Colombia	Guamo	0.3	0.2	0.4	1	1	2	0.1	0.0	0.1	0	0	0
Colombia	Guapí	0.4	0.3	0.5	1	1	2	0.1	0.1	0.1	0	0	0
Colombia	Guapotá	0.3	0.2	0.4	0	0	0	0.1	0.0	0.1	0	0	0
Colombia	Guarandá	0.3	0.3	0.4	1	0	1	0.1	0.1	0.1	0	0	0
Colombia	Guarne	0.3	0.3	0.4	5	4	6	0.1	0.1	0.1	1	1	1
Colombia	Guasca	0.3	0.3	0.4	0	0	1	0.1	0.1	0.1	0	0	0
Colombia	Guatapé	0.3	0.3	0.4	0	0	0	0.1	0.1	0.1	0	0	0
Colombia	Guataquí	0.3	0.2	0.4	0	0	0	0.1	0.1	0.1	0	0	0
Colombia	Guatavita	0.3	0.2	0.4	0	0	0	0.1	0.1	0.1	0	0	0
Colombia	Guateque	0.3	0.2	0.4	0	0	0	0.1	0.0	0.1	0	0	0
Colombia	Guática	0.3	0.2	0.4	0	0	0	0.1	0.0	0.1	0	0	0
Colombia	Guavatá	0.3	0.2	0.4	0	0	0	0.1	0.0	0.1	0	0	0
Colombia	Guayabal de Síquima	0.3	0.3	0.4	0	0	0	0.1	0.1	0.1	0	0	0
Colombia	Guayabetal	0.3	0.3	0.4	0	0	0	0.1	0.1	0.1	0	0	0
Colombia	Guayatá	0.3	0.2	0.4	0	0	0	0.1	0.0	0.1	0	0	0
Colombia	Güepsa	0.3	0.2	0.4	0	0	0	0.1	0.0	0.1	0	0	0
Colombia	Guicán	0.4	0.3	0.5	0	0	1	0.1	0.1	0.1	0	0	0
Colombia	Gutiérrez	0.3	0.3	0.4	1	0	1	0.1	0.1	0.1	0	0	0

Table 3: Diarrhoea Mortality by Unit *(continued)*

		2000 mortality						2017 mortality					
Country	Unit	mean rate (per 10,000)	lower rate (per 10,000)	upper rate (per 10,000)	mean count	lower count	upper count	mean rate (per 10,000)	lower rate (per 10,000)	upper rate (per 10,000)	mean count	lower count	upper count
Colombia	Hacarí	0.3	0.3	0.4	0	0	0	0.1	0.1	0.1	0	0	0
Colombia	Hatillo de Loba	0.3	0.3	0.4	0	0	0	0.1	0.1	0.1	0	0	0
Colombia	Hato	0.3	0.2	0.4	0	0	0	0.1	0.0	0.1	0	0	0
Colombia	Hato Corozal	0.3	0.3	0.4	1	0	1	0.1	0.1	0.1	0	0	0
Colombia	Heliconia	0.3	0.3	0.4	0	0	0	0.1	0.1	0.1	0	0	0
Colombia	Herrán	0.3	0.3	0.4	0	0	0	0.1	0.1	0.1	0	0	0
Colombia	Herveo	0.3	0.2	0.4	0	0	0	0.1	0.0	0.1	0	0	0
Colombia	Hispania	0.3	0.3	0.4	0	0	0	0.1	0.1	0.1	0	0	0
Colombia	Hobo	0.3	0.3	0.4	0	0	0	0.1	0.1	0.1	0	0	0
Colombia	Honda	0.3	0.3	0.4	1	1	1	0.1	0.1	0.1	0	0	0
Colombia	Ibagué	0.3	0.3	0.4	16	13	20	0.1	0.1	0.1	3	2	4
Colombia	Icononzo	0.3	0.3	0.4	0	0	1	0.1	0.1	0.1	0	0	0
Colombia	Iles	0.4	0.3	0.4	0	0	1	0.1	0.1	0.1	0	0	0
Colombia	Imués	0.4	0.3	0.4	1	1	1	0.1	0.1	0.1	0	0	0
Colombia	Inzá	0.4	0.3	0.4	1	1	1	0.1	0.1	0.1	0	0	0
Colombia	Ipiales	0.4	0.3	0.4	4	3	5	0.1	0.1	0.1	1	1	1
Colombia	Iquira	0.3	0.3	0.4	0	0	1	0.1	0.1	0.1	0	0	0
Colombia	Isnos	0.3	0.3	0.4	1	1	1	0.1	0.1	0.1	0	0	0
Colombia	Istmina	0.4	0.3	0.5	1	1	2	0.1	0.1	0.1	0	0	0
Colombia	Itagüí	0.3	0.3	0.4	5	4	6	0.1	0.0	0.1	1	1	1
Colombia	Ituango	0.3	0.3	0.4	1	1	1	0.1	0.1	0.1	0	0	0
Colombia	Izá	0.3	0.3	0.4	0	0	0	0.1	0.1	0.1	0	0	0
Colombia	Jambaló	0.4	0.3	0.4	1	0	1	0.1	0.1	0.1	0	0	0
Colombia	Jamundí	0.3	0.2	0.4	3	2	4	0.1	0.0	0.1	1	1	1
Colombia	Jardín	0.3	0.3	0.4	1	1	1	0.1	0.1	0.1	0	0	0
Colombia	Jenesano	0.3	0.3	0.4	0	0	0	0.1	0.0	0.1	0	0	0
Colombia	Jericó	0.3	0.3	0.4	0	0	0	0.1	0.1	0.1	0	0	0
Colombia	Jericó	0.3	0.3	0.4	0	0	1	0.1	0.1	0.1	0	0	0
Colombia	Jerusalén	0.3	0.3	0.4	0	0	0	0.1	0.1	0.1	0	0	0
Colombia	Jesús María	0.3	0.2	0.4	0	0	0	0.1	0.0	0.1	0	0	0
Colombia	Jordán	0.3	0.2	0.4	0	0	0	0.1	0.0	0.1	0	0	0
Colombia	Juan de Acosta	0.3	0.2	0.3	0	0	1	0.1	0.1	0.1	0	0	0
Colombia	Junín	0.3	0.3	0.4	0	0	0	0.1	0.1	0.1	0	0	0
Colombia	Juradó	0.4	0.3	0.5	0	0	0	0.1	0.1	0.1	0	0	0
Colombia	La Argentina	0.4	0.3	0.4	0	0	0	0.1	0.1	0.1	0	0	0
Colombia	La Belleza	0.3	0.3	0.4	0	0	0	0.1	0.1	0.1	0	0	0
Colombia	La Calera	0.3	0.3	0.4	13	10	16	0.1	0.1	0.1	2	2	3
Colombia	La Capilla	0.3	0.2	0.4	0	0	0	0.1	0.0	0.1	0	0	0
Colombia	La Ceja	0.3	0.3	0.4	1	1	2	0.1	0.1	0.1	0	0	0
Colombia	La Celia	0.3	0.2	0.4	0	0	0	0.1	0.1	0.1	0	0	0
Colombia	La Chorrera	0.5	0.4	0.7	0	0	1	0.1	0.1	0.1	0	0	0
Colombia	La Cruz	0.4	0.3	0.5	1	1	2	0.1	0.1	0.1	0	0	0
Colombia	La Cumbre	0.3	0.2	0.3	0	0	0	0.1	0.0	0.1	0	0	0
Colombia	La Dorada	0.3	0.3	0.4	1	1	1	0.1	0.1	0.1	0	0	0
Colombia	La Esperanza	0.3	0.3	0.4	0	0	1	0.1	0.1	0.1	0	0	0
Colombia	La Estrella	0.3	0.3	0.4	2	2	3	0.1	0.1	0.1	0	0	0
Colombia	La Florida	0.4	0.3	0.4	1	1	1	0.1	0.1	0.1	0	0	0
Colombia	La Gloria	0.3	0.2	0.4	1	1	1	0.1	0.1	0.1	0	0	0
Colombia	La Guadalupe	0.4	0.3	0.5	0	0	0	0.1	0.1	0.1	0	0	0
Colombia	La Jagua de Ibirico	0.3	0.2	0.4	1	1	1	0.1	0.1	0.1	0	0	0
Colombia	La Llanada	0.4	0.3	0.5	0	0	0	0.1	0.1	0.1	0	0	0
Colombia	La Macarena	0.4	0.3	0.4	1	1	1	0.1	0.1	0.1	0	0	0
Colombia	La Merced	0.3	0.2	0.4	0	0	0	0.1	0.0	0.1	0	0	0
Colombia	La Mesa	0.3	0.3	0.4	1	1	1	0.1	0.1	0.1	0	0	0
Colombia	La Montañita	0.4	0.3	0.4	1	1	1	0.1	0.1	0.1	0	0	0
Colombia	La Palma	0.3	0.3	0.4	0	0	1	0.1	0.1	0.1	0	0	0
Colombia	La Paz	0.3	0.2	0.4	2	2	2	0.1	0.1	0.1	0	0	0
Colombia	La Paz	0.3	0.2	0.4	0	0	0	0.1	0.1	0.1	0	0	0
Colombia	La Pedrera	0.5	0.4	0.6	0	0	0	0.1	0.1	0.1	0	0	0

Table 3: Diarrhoea Mortality by Unit *(continued)*

Country	Unit	2000 mortality mean rate (per 10,000)	lower rate (per 10,000)	upper rate (per 10,000)	mean count	lower count	upper count	2017 mortality mean rate (per 10,000)	lower rate (per 10,000)	upper rate (per 10,000)	mean count	lower count	upper count
Colombia	La Peña	0.3	0.3	0.4	0	0	0	0.1	0.1	0.1	0	0	0
Colombia	La Plata	0.3	0.3	0.4	2	2	2	0.1	0.1	0.1	0	0	1
Colombia	La Playa de Belén	0.3	0.3	0.4	0	0	0	0.1	0.1	0.1	0	0	0
Colombia	La Primavera	0.4	0.3	0.4	1	1	1	0.1	0.1	0.1	0	0	0
Colombia	La Salina	0.3	0.3	0.4	0	0	0	0.1	0.1	0.1	0	0	0
Colombia	La Sierra	0.4	0.3	0.4	1	1	1	0.1	0.1	0.1	0	0	0
Colombia	La Tebaida	0.3	0.2	0.3	1	0	1	0.1	0.0	0.1	0	0	0
Colombia	La Tola	0.4	0.3	0.4	0	0	0	0.1	0.1	0.1	0	0	0
Colombia	La Unión de Sucre	0.2	0.2	0.3	1	0	1	0.0	0.0	0.1	0	0	0
Colombia	La Unión de Sucre	0.4	0.3	0.4	1	1	1	0.1	0.1	0.1	0	0	0
Colombia	La Unión de Sucre	0.3	0.3	0.4	1	1	1	0.1	0.1	0.1	0	0	0
Colombia	La Unión de Sucre	0.3	0.2	0.4	0	0	0	0.1	0.1	0.1	0	0	0
Colombia	La Uribe	0.4	0.3	0.4	0	0	1	0.1	0.1	0.1	0	0	0
Colombia	La Uvita	0.3	0.3	0.4	0	0	0	0.1	0.1	0.1	0	0	0
Colombia	La Vega	0.4	0.3	0.4	1	1	2	0.1	0.1	0.1	0	0	0
Colombia	La Vega	0.3	0.3	0.4	1	0	1	0.1	0.1	0.1	0	0	0
Colombia	La Victoria	0.2	0.2	0.3	1	0	1	0.1	0.0	0.1	0	0	0
Colombia	La Victoria	0.4	0.3	0.4	0	0	0	0.1	0.1	0.1	0	0	0
Colombia	La Virginia	0.3	0.2	0.3	1	0	1	0.1	0.1	0.1	0	0	0
Colombia	Labateca	0.3	0.3	0.4	0	0	0	0.1	0.1	0.1	0	0	0
Colombia	Labranzagrande	0.3	0.3	0.4	0	0	0	0.1	0.1	0.1	0	0	0
Colombia	Landázuri	0.3	0.3	0.4	0	0	1	0.1	0.1	0.1	0	0	0
Colombia	Lebrija	0.3	0.2	0.4	1	0	1	0.1	0.1	0.1	0	0	0
Colombia	Leiva	0.4	0.3	0.4	1	1	1	0.1	0.1	0.1	0	0	0
Colombia	Lejanías	0.3	0.3	0.4	1	0	1	0.1	0.1	0.1	0	0	0
Colombia	Lenguazaque	0.3	0.2	0.4	0	0	0	0.1	0.0	0.1	0	0	0
Colombia	Lérida	0.3	0.2	0.4	1	1	1	0.1	0.1	0.1	0	0	0
Colombia	Leticia	0.5	0.4	0.6	2	2	3	0.1	0.1	0.1	1	1	1
Colombia	Líbano	0.3	0.2	0.4	1	1	1	0.1	0.1	0.1	0	0	0
Colombia	Liborina	0.3	0.2	0.4	0	0	0	0.1	0.1	0.1	0	0	0
Colombia	Linares	0.3	0.3	0.4	1	0	1	0.1	0.1	0.1	0	0	0
Colombia	Lloró	0.4	0.3	0.5	1	1	1	0.1	0.1	0.1	0	0	0
Colombia	López de Micay	0.4	0.3	0.5	1	1	1	0.1	0.1	0.1	0	0	0
Colombia	Los Andes	0.4	0.3	0.5	1	0	1	0.1	0.1	0.1	0	0	0
Colombia	Los Córdobas	0.3	0.2	0.4	1	0	1	0.1	0.1	0.1	0	0	0
Colombia	Los Palmitos	0.3	0.2	0.4	1	1	1	0.1	0.1	0.1	0	0	0
Colombia	Los Patios	0.3	0.3	0.4	0	0	0	0.1	0.1	0.1	0	0	0
Colombia	Los Santos	0.3	0.2	0.4	0	0	0	0.1	0.0	0.1	0	0	0
Colombia	Lourdes	0.3	0.3	0.4	0	0	0	0.1	0.1	0.1	0	0	0
Colombia	Luruaco	0.3	0.2	0.3	1	1	1	0.1	0.1	0.1	0	0	0
Colombia	Macanal	0.3	0.2	0.4	0	0	0	0.1	0.1	0.1	0	0	0
Colombia	Macaravita	0.3	0.2	0.4	0	0	0	0.1	0.0	0.1	0	0	0
Colombia	Maceo	0.3	0.3	0.4	0	0	0	0.1	0.1	0.1	0	0	0
Colombia	Machetá	0.3	0.2	0.4	0	0	0	0.1	0.0	0.1	0	0	0
Colombia	Madrid	0.3	0.3	0.4	2	1	2	0.1	0.1	0.1	0	0	1
Colombia	Magüí	0.4	0.3	0.5	2	1	2	0.1	0.1	0.1	0	0	0
Colombia	Mahates	0.3	0.2	0.4	1	1	1	0.1	0.1	0.1	0	0	0
Colombia	Maicao	0.3	0.3	0.4	8	7	10	0.1	0.1	0.1	3	2	3
Colombia	Majagual	0.3	0.2	0.4	1	1	1	0.1	0.1	0.1	0	0	0
Colombia	Málaga	0.3	0.2	0.4	0	0	0	0.1	0.0	0.1	0	0	0
Colombia	Malambo	0.3	0.2	0.4	4	3	4	0.1	0.1	0.1	1	1	1
Colombia	Mallama	0.4	0.3	0.5	0	0	1	0.1	0.1	0.1	0	0	0
Colombia	Manatí	0.3	0.2	0.3	1	0	1	0.1	0.1	0.1	0	0	0
Colombia	Manaure	0.3	0.2	0.4	0	0	0	0.1	0.1	0.1	0	0	0
Colombia	Manaure	0.3	0.3	0.4	4	3	5	0.1	0.1	0.1	3	2	4

Table 3: Diarrhoea Mortality by Unit *(continued)*

		2000 mortality						2017 mortality					
Country	Unit	mean rate (per 10,000)	lower rate (per 10,000)	upper rate (per 10,000)	mean count	lower count	upper count	mean rate (per 10,000)	lower rate (per 10,000)	upper rate (per 10,000)	mean count	lower count	upper count
Colombia	Maní	0.3	0.3	0.4	0	0	1	0.1	0.1	0.1	0	0	0
Colombia	Manizales	0.3	0.2	0.4	10	8	12	0.1	0.0	0.1	2	1	2
Colombia	Manta	0.3	0.2	0.4	0	0	0	0.1	0.0	0.1	0	0	0
Colombia	Manzanares	0.3	0.2	0.4	1	0	1	0.1	0.0	0.1	0	0	0
Colombia	Mapiripán	0.4	0.3	0.4	1	0	1	0.1	0.1	0.1	0	0	0
Colombia	Margarita	0.3	0.3	0.4	1	0	1	0.1	0.1	0.1	0	0	0
Colombia	María la Baja	0.3	0.2	0.4	2	1	2	0.1	0.1	0.1	0	0	0
Colombia	Marinilla	0.3	0.3	0.4	2	1	2	0.1	0.1	0.1	0	0	0
Colombia	Maripí	0.4	0.3	0.4	0	0	1	0.1	0.1	0.1	0	0	0
Colombia	Marmato	0.3	0.2	0.4	0	0	0	0.1	0.1	0.1	0	0	0
Colombia	Marquetalia	0.3	0.2	0.4	0	0	1	0.1	0.1	0.1	0	0	0
Colombia	Marsella	0.3	0.2	0.3	1	1	1	0.1	0.0	0.1	0	0	0
Colombia	Marulanda	0.3	0.2	0.4	0	0	0	0.1	0.1	0.1	0	0	0
Colombia	Matanza	0.3	0.2	0.4	0	0	1	0.1	0.0	0.1	0	0	0
Colombia	Medellín	0.3	0.2	0.4	60	48	74	0.1	0.0	0.1	10	8	13
Colombia	Medina	0.3	0.3	0.4	1	1	1	0.1	0.1	0.1	0	0	0
Colombia	Melgar	0.3	0.3	0.4	1	1	1	0.1	0.1	0.1	0	0	0
Colombia	Mercaderes	0.4	0.3	0.4	1	1	1	0.1	0.1	0.1	0	0	0
Colombia	Mesetas	0.3	0.3	0.4	0	0	0	0.1	0.1	0.1	0	0	0
Colombia	Milán	0.4	0.3	0.5	1	1	1	0.1	0.1	0.1	0	0	0
Colombia	Miraflores	0.4	0.3	0.5	1	0	1	0.1	0.1	0.1	0	0	0
Colombia	Miraflores	0.3	0.2	0.4	0	0	0	0.1	0.0	0.1	0	0	0
Colombia	Miranda	0.3	0.3	0.4	1	1	1	0.1	0.1	0.1	0	0	0
Colombia	Mirití-Paraná	0.5	0.4	0.6	0	0	0	0.1	0.1	0.1	0	0	0
Colombia	Mistrato	0.3	0.2	0.4	0	0	0	0.1	0.1	0.1	0	0	0
Colombia	Mitú	0.5	0.4	0.6	2	1	2	0.1	0.1	0.1	1	1	1
Colombia	Mogotes	0.3	0.2	0.4	0	0	0	0.1	0.0	0.1	0	0	0
Colombia	Molagavita	0.3	0.2	0.4	0	0	0	0.1	0.0	0.1	0	0	0
Colombia	Momil	0.3	0.2	0.4	0	0	0	0.1	0.1	0.1	0	0	0
Colombia	Mompós	0.3	0.3	0.4	1	0	1	0.1	0.1	0.1	0	0	0
Colombia	Mongua	0.3	0.3	0.4	0	0	0	0.1	0.1	0.1	0	0	0
Colombia	Monguí	0.3	0.3	0.4	0	0	0	0.1	0.1	0.1	0	0	0
Colombia	Moniquirá	0.3	0.3	0.4	1	1	1	0.1	0.1	0.1	0	0	0
Colombia	Moñitos	0.3	0.2	0.4	1	1	1	0.1	0.1	0.1	0	0	0
Colombia	Montebello	0.3	0.2	0.4	0	0	0	0.1	0.0	0.1	0	0	0
Colombia	Montecristo	0.4	0.3	0.4	1	0	1	0.1	0.1	0.1	0	0	0
Colombia	Montelíbano	0.3	0.2	0.4	3	3	4	0.1	0.1	0.1	1	1	1
Colombia	Montenegro	0.3	0.2	0.3	1	1	1	0.1	0.0	0.1	0	0	0
Colombia	Montería	0.3	0.2	0.4	14	11	17	0.1	0.0	0.1	3	2	4
Colombia	Monterrey	0.3	0.3	0.4	0	0	1	0.1	0.1	0.1	0	0	0
Colombia	Morales	0.3	0.2	0.4	2	2	3	0.1	0.1	0.1	1	0	1
Colombia	Morales	0.4	0.3	0.4	1	1	1	0.1	0.1	0.1	0	0	0
Colombia	Morelia	0.4	0.3	0.4	0	0	0	0.1	0.1	0.1	0	0	0
Colombia	Morroa	0.3	0.2	0.4	1	1	1	0.1	0.1	0.1	0	0	0
Colombia	Mosquera	0.4	0.3	0.4	0	0	1	0.1	0.1	0.1	0	0	0
Colombia	Mosquera	0.3	0.3	0.4	2	2	3	0.1	0.1	0.1	1	1	1
Colombia	Motavita	0.3	0.3	0.4	0	0	0	0.1	0.1	0.1	0	0	0
Colombia	Murillo	0.3	0.3	0.4	0	0	0	0.1	0.1	0.1	0	0	0
Colombia	Murindó	0.4	0.3	0.5	0	0	0	0.1	0.1	0.1	0	0	0
Colombia	Mutatá	0.3	0.3	0.4	1	0	1	0.1	0.1	0.1	0	0	0
Colombia	Mutiscua	0.3	0.3	0.4	0	0	0	0.1	0.1	0.1	0	0	0
Colombia	Muzo	0.4	0.3	0.4	1	0	1	0.1	0.1	0.1	0	0	0
Colombia	n.a. (1)	NA	NA	NA	NA	NA	NA	NA	NA	NA	NA	NA	NA
Colombia	Nariño	0.3	0.2	0.4	0	0	0	0.1	0.1	0.1	0	0	0
Colombia	Nariño	0.3	0.3	0.4	0	0	1	0.1	0.1	0.1	0	0	0
Colombia	Nátaga	0.3	0.3	0.4	0	0	0	0.1	0.1	0.1	0	0	0
Colombia	Natagaima	0.3	0.3	0.4	1	1	1	0.1	0.1	0.1	0	0	0
Colombia	Nechí	0.3	0.3	0.4	0	0	0	0.1	0.1	0.1	0	0	0
Colombia	Necoclí	0.3	0.2	0.4	1	1	2	0.1	0.1	0.1	0	0	0
Colombia	Neira	0.3	0.2	0.4	1	1	2	0.1	0.0	0.1	0	0	0

Table 3: Diarrhoea Mortality by Unit *(continued)*

Country	Unit	2000 mortality mean rate (per 10,000)	lower rate (per 10,000)	upper rate (per 10,000)	mean count	lower count	upper count	2017 mortality mean rate (per 10,000)	lower rate (per 10,000)	upper rate (per 10,000)	mean count	lower count	upper count
Colombia	Neiva	0.3	0.3	0.4	12	10	15	0.1	0.1	0.1	2	2	3
Colombia	Nemocón	0.3	0.3	0.4	1	0	1	0.1	0.1	0.1	0	0	0
Colombia	Nilo	0.3	0.3	0.4	0	0	0	0.1	0.1	0.1	0	0	0
Colombia	Nimaima	0.3	0.3	0.4	0	0	0	0.1	0.1	0.1	0	0	0
Colombia	Nobsa	0.3	0.3	0.4	1	1	2	0.1	0.1	0.1	0	0	0
Colombia	Nocaima	0.4	0.3	0.4	0	0	0	0.1	0.1	0.1	0	0	0
Colombia	Novita	0.4	0.3	0.5	1	1	1	0.1	0.1	0.1	0	0	0
Colombia	Nuevo Colón	0.3	0.2	0.4	0	0	0	0.1	0.0	0.1	0	0	0
Colombia	Nunchía	0.3	0.2	0.4	1	0	1	0.1	0.1	0.1	0	0	0
Colombia	Nuquí	0.4	0.3	0.5	0	0	1	0.1	0.1	0.1	0	0	0
Colombia	Obando	0.2	0.2	0.3	0	0	0	0.1	0.0	0.1	0	0	0
Colombia	Ocamonte	0.3	0.2	0.4	0	0	0	0.1	0.0	0.1	0	0	0
Colombia	Ocaña	0.3	0.3	0.4	3	3	4	0.1	0.1	0.1	1	0	1
Colombia	Oiba	0.3	0.2	0.4	0	0	0	0.1	0.0	0.1	0	0	0
Colombia	Oicatá	0.3	0.2	0.4	1	1	1	0.1	0.0	0.1	0	0	0
Colombia	Olaya	0.3	0.2	0.4	0	0	0	0.1	0.0	0.1	0	0	0
Colombia	Olaya Herrera	0.4	0.3	0.4	1	1	2	0.1	0.1	0.1	0	0	0
Colombia	Onzaga	0.3	0.2	0.4	0	0	0	0.1	0.0	0.1	0	0	0
Colombia	Oporapa	0.3	0.3	0.4	1	0	1	0.1	0.1	0.1	0	0	0
Colombia	Orito	0.4	0.3	0.5	3	2	3	0.1	0.1	0.1	1	1	1
Colombia	Orocué	0.3	0.3	0.4	0	0	0	0.1	0.1	0.1	0	0	0
Colombia	Ortega	0.3	0.2	0.4	1	1	1	0.1	0.1	0.1	0	0	0
Colombia	Ospina	0.4	0.3	0.4	1	1	1	0.1	0.1	0.1	0	0	0
Colombia	Otanche	0.4	0.3	0.4	0	0	0	0.1	0.1	0.1	0	0	0
Colombia	Ovejas	0.3	0.2	0.4	1	1	1	0.1	0.1	0.1	0	0	0
Colombia	Pachavita	0.3	0.2	0.4	0	0	0	0.1	0.0	0.1	0	0	0
Colombia	Pacho	0.3	0.2	0.4	1	1	1	0.1	0.0	0.1	0	0	0
Colombia	Pacoa	0.5	0.4	0.6	0	0	0	0.1	0.1	0.1	0	0	0
Colombia	Pácora	0.3	0.2	0.4	1	1	1	0.1	0.1	0.1	0	0	0
Colombia	Padilla	0.3	0.3	0.4	1	1	1	0.1	0.1	0.1	0	0	0
Colombia	Páez	0.4	0.3	0.4	1	1	2	0.1	0.1	0.1	0	0	0
Colombia	Páez	0.3	0.3	0.4	0	0	0	0.1	0.1	0.1	0	0	0
Colombia	Paicol	0.3	0.3	0.4	0	0	0	0.1	0.1	0.1	0	0	0
Colombia	Pailitas	0.3	0.2	0.4	1	1	1	0.1	0.1	0.1	0	0	0
Colombia	Paime	0.3	0.3	0.4	0	0	0	0.1	0.1	0.1	0	0	0
Colombia	Paipa	0.3	0.3	0.4	1	1	1	0.1	0.1	0.1	0	0	0
Colombia	Pajarito	0.3	0.3	0.4	0	0	0	0.1	0.1	0.1	0	0	0
Colombia	Palermo	0.3	0.2	0.4	1	0	1	0.1	0.1	0.1	0	0	0
Colombia	Palestina	0.3	0.2	0.4	0	0	0	0.1	0.0	0.1	0	0	0
Colombia	Palestina	0.4	0.3	0.4	1	0	1	0.1	0.1	0.1	0	0	0
Colombia	Palmar	0.3	0.2	0.4	0	0	0	0.1	0.0	0.1	0	0	0
Colombia	Palmar de Varela	0.3	0.2	0.3	0	0	0	0.1	0.1	0.1	0	0	0
Colombia	Palmas del Socorro	0.3	0.2	0.4	0	0	0	0.1	0.0	0.1	0	0	0
Colombia	Palmira	0.3	0.2	0.4	16	13	19	0.1	0.0	0.1	3	2	4
Colombia	Pamplona	0.3	0.3	0.4	1	1	2	0.1	0.1	0.1	0	0	0
Colombia	Pamplonita	0.3	0.3	0.4	1	0	1	0.1	0.1	0.1	0	0	0
Colombia	Pana Pana	0.4	0.3	0.5	0	0	0	0.1	0.1	0.1	0	0	0
Colombia	Pandi	0.3	0.3	0.4	0	0	0	0.1	0.1	0.1	0	0	0
Colombia	Panqueba	0.3	0.3	0.4	0	0	0	0.1	0.1	0.1	0	0	0
Colombia	Papunahua	0.5	0.4	0.6	0	0	0	0.1	0.1	0.1	0	0	0
Colombia	Páramo	0.3	0.2	0.4	0	0	0	0.1	0.0	0.1	0	0	0
Colombia	Paratebueno	0.3	0.3	0.4	0	0	0	0.1	0.1	0.1	0	0	0
Colombia	Pasca	0.3	0.2	0.4	0	0	0	0.1	0.1	0.1	0	0	0
Colombia	Patía	0.4	0.3	0.4	1	1	2	0.1	0.1	0.1	0	0	0
Colombia	Pauna	0.3	0.3	0.4	1	0	1	0.1	0.1	0.1	0	0	0
Colombia	Paya	0.3	0.3	0.4	0	0	0	0.1	0.1	0.1	0	0	0
Colombia	Paz de Ariporo	0.3	0.3	0.4	1	1	1	0.1	0.1	0.1	0	0	0
Colombia	Paz de Río	0.3	0.2	0.4	0	0	0	0.1	0.0	0.1	0	0	0
Colombia	Pedraza	0.3	0.2	0.4	1	1	1	0.1	0.1	0.1	0	0	0

Table 3: Diarrhoea Mortality by Unit *(continued)*

Country	Unit	2000 mortality mean rate (per 10,000)	lower rate (per 10,000)	upper rate (per 10,000)	mean count	lower count	upper count	2017 mortality mean rate (per 10,000)	lower rate (per 10,000)	upper rate (per 10,000)	mean count	lower count	upper count
Colombia	Pelaya	0.3	0.2	0.4	1	1	1	0.1	0.1	0.1	0	0	0
Colombia	Peñol	0.3	0.3	0.4	1	1	1	0.1	0.1	0.1	0	0	0
Colombia	Pensilvania	0.3	0.2	0.4	1	1	1	0.1	0.1	0.1	0	0	0
Colombia	Pequé	0.3	0.2	0.4	0	0	0	0.1	0.1	0.1	0	0	0
Colombia	Pereira	0.3	0.2	0.3	11	8	13	0.1	0.0	0.1	2	1	2
Colombia	Pesca	0.3	0.3	0.4	0	0	0	0.1	0.1	0.1	0	0	0
Colombia	Piedecuesta	0.3	0.2	0.4	4	3	5	0.1	0.1	0.1	1	1	1
Colombia	Piedras	0.3	0.2	0.4	0	0	0	0.1	0.1	0.1	0	0	0
Colombia	Piendamó	0.4	0.3	0.4	1	1	1	0.1	0.1	0.1	0	0	0
Colombia	Pijao	0.3	0.2	0.3	0	0	0	0.1	0.0	0.1	0	0	0
Colombia	Pinchote	0.3	0.2	0.4	0	0	0	0.1	0.0	0.1	0	0	0
Colombia	Pinillos	0.3	0.3	0.4	1	1	1	0.1	0.1	0.1	2	2	3
Colombia	Piojó	0.3	0.2	0.3	0	0	0	0.1	0.1	0.1	0	0	0
Colombia	Pisba	0.3	0.3	0.4	0	0	0	0.1	0.1	0.1	0	0	0
Colombia	Pital	0.3	0.3	0.4	0	0	0	0.1	0.1	0.1	0	0	0
Colombia	Pitalito	0.3	0.3	0.4	4	3	4	0.1	0.1	0.1	1	1	1
Colombia	Pivijay	0.3	0.2	0.4	2	2	2	0.1	0.1	0.1	0	0	1
Colombia	Planadas	0.3	0.3	0.4	1	1	1	0.1	0.1	0.1	0	0	0
Colombia	Planeta Rica	0.3	0.2	0.4	2	2	3	0.1	0.1	0.1	0	0	1
Colombia	Plato	0.3	0.3	0.4	3	2	3	0.1	0.1	0.1	1	0	1
Colombia	Policarpa	0.4	0.3	0.4	0	0	1	0.1	0.1	0.1	0	0	0
Colombia	Polonuevo	0.3	0.2	0.3	1	0	1	0.1	0.1	0.1	0	0	0
Colombia	Ponedera	0.3	0.2	0.3	1	1	1	0.1	0.1	0.1	0	0	0
Colombia	Popayán	0.4	0.3	0.4	11	9	13	0.1	0.1	0.1	2	2	3
Colombia	Pore	0.3	0.2	0.4	0	0	0	0.1	0.1	0.1	0	0	0
Colombia	Potosí	0.4	0.3	0.5	1	1	2	0.1	0.1	0.1	0	0	0
Colombia	Pradera	0.3	0.2	0.4	2	1	2	0.1	0.1	0.1	0	0	0
Colombia	Prado	0.3	0.2	0.4	0	0	0	0.1	0.1	0.1	0	0	0
Colombia	Providencia	NA	NA	NA	NA	NA	NA	NA	NA	NA	NA	NA	NA
Colombia	Providencia	0.4	0.3	0.4	0	0	1	0.1	0.1	0.1	0	0	0
Colombia	Pueblo Nuevo	0.3	0.2	0.4	1	1	2	0.1	0.1	0.1	0	0	0
Colombia	Pueblo Rico	0.3	0.3	0.4	1	0	1	0.1	0.1	0.1	0	0	0
Colombia	Pueblo Viejo	0.3	0.3	0.4	0	0	0	0.1	0.1	0.1	0	0	0
Colombia	Pueblorrico	0.3	0.3	0.4	0	0	1	0.1	0.1	0.1	0	0	0
Colombia	Puente Nacional	0.3	0.2	0.4	0	0	1	0.1	0.0	0.1	0	0	0
Colombia	Puerres	0.4	0.3	0.4	1	0	1	0.1	0.1	0.1	0	0	0
Colombia	Puerto Asís	0.4	0.3	0.5	3	2	4	0.1	0.1	0.1	1	1	1
Colombia	Puerto Berrío	0.3	0.3	0.4	1	1	1	0.1	0.1	0.1	0	0	0
Colombia	Puerto Boyacá	0.3	0.3	0.4	2	2	2	0.1	0.1	0.1	0	0	1
Colombia	Puerto Caicedo	0.4	0.3	0.5	1	1	2	0.1	0.1	0.1	0	0	0
Colombia	Puerto Carreño	0.3	0.3	0.4	1	1	1	0.1	0.1	0.1	0	0	0
Colombia	Puerto Colombia	0.3	0.2	0.4	1	1	2	0.1	0.1	0.1	0	0	0
Colombia	Puerto Colombia	0.4	0.3	0.5	0	0	0	0.1	0.1	0.1	0	0	0
Colombia	Puerto Concordia	0.4	0.3	0.4	0	0	1	0.1	0.1	0.1	0	0	0
Colombia	Puerto Escondido	0.3	0.2	0.4	1	1	1	0.1	0.1	0.1	0	0	0
Colombia	Puerto Gaitán	0.3	0.3	0.4	1	1	1	0.1	0.1	0.1	0	0	0
Colombia	Puerto Guzmán	0.4	0.3	0.5	1	1	1	0.1	0.1	0.1	0	0	0
Colombia	Puerto Inírida	0.4	0.3	0.5	1	1	1	0.1	0.1	0.1	0	0	1
Colombia	Puerto Leguízamo	0.4	0.4	0.5	1	1	1	0.1	0.1	0.1	0	0	0
Colombia	Puerto Libertador	0.3	0.3	0.4	1	1	1	0.1	0.1	0.1	0	0	0
Colombia	Puerto Lleras	0.4	0.3	0.4	0	0	1	0.1	0.1	0.1	0	0	0
Colombia	Puerto López	0.3	0.3	0.4	1	1	1	0.1	0.1	0.1	0	0	0
Colombia	Puerto Nare	0.3	0.3	0.4	0	0	0	0.1	0.1	0.1	0	0	0
Colombia	Puerto Nariño	0.5	0.4	0.7	1	0	1	0.1	0.1	0.1	0	0	0
Colombia	Puerto Parra	0.3	0.2	0.4	0	0	0	0.1	0.1	0.1	0	0	0
Colombia	Puerto Rico	0.4	0.3	0.4	2	1	2	0.1	0.1	0.1	0	0	0
Colombia	Puerto Rico	0.4	0.3	0.4	1	1	1	0.1	0.1	0.1	0	0	0
Colombia	Puerto Rondón	0.3	0.3	0.4	1	1	1	0.1	0.1	0.1	0	0	0

Table 3: Diarrhoea Mortality by Unit *(continued)*

Country	Unit	2000 mortality mean rate (per 10,000)	2000 mortality lower rate (per 10,000)	2000 mortality upper rate (per 10,000)	2000 mortality mean count	2000 mortality lower count	2000 mortality upper count	2017 mortality mean rate (per 10,000)	2017 mortality lower rate (per 10,000)	2017 mortality upper rate (per 10,000)	2017 mortality mean count	2017 mortality lower count	2017 mortality upper count
Colombia	Puerto Salgar	0.3	0.3	0.4	1	1	2	0.1	0.1	0.1	0	0	0
Colombia	Puerto Santander	0.3	0.2	0.4	0	0	1	0.1	0.1	0.1	0	0	0
Colombia	Puerto Santander	0.5	0.4	0.6	0	0	0	0.1	0.1	0.1	0	0	0
Colombia	Puerto Tejada	0.3	0.3	0.4	2	2	2	0.1	0.1	0.1	0	0	0
Colombia	Puerto Triunfo	0.3	0.3	0.4	0	0	1	0.1	0.1	0.1	0	0	0
Colombia	Puerto Wilches	0.3	0.3	0.4	1	1	2	0.1	0.1	0.1	0	0	0
Colombia	Pulí	0.3	0.3	0.4	0	0	0	0.1	0.1	0.1	0	0	0
Colombia	Pupiales	0.4	0.3	0.4	1	1	1	0.1	0.1	0.1	0	0	0
Colombia	Puracé	0.4	0.3	0.4	1	1	1	0.1	0.1	0.1	0	0	0
Colombia	Purificación	0.3	0.2	0.4	1	1	1	0.1	0.1	0.1	0	0	0
Colombia	Purísima	0.3	0.3	0.4	1	0	1	0.1	0.1	0.1	0	0	0
Colombia	Quebradanegra	0.3	0.3	0.4	0	0	0	0.1	0.1	0.1	0	0	0
Colombia	Quetame	0.3	0.2	0.4	0	0	0	0.1	0.1	0.1	0	0	0
Colombia	Quibdó	0.4	0.3	0.5	9	7	11	0.1	0.1	0.1	2	2	3
Colombia	Quimbaya	0.2	0.2	0.3	0	0	0	0.1	0.0	0.1	0	0	0
Colombia	Quinchía	0.3	0.2	0.4	1	1	1	0.1	0.0	0.1	0	0	0
Colombia	Quípama	0.4	0.3	0.4	0	0	0	0.1	0.1	0.1	0	0	0
Colombia	Quipile	0.3	0.2	0.4	0	0	0	0.1	0.1	0.1	0	0	0
Colombia	Ragonvalia	0.3	0.3	0.4	0	0	0	0.1	0.1	0.1	0	0	0
Colombia	Ramiriquí	0.3	0.3	0.4	0	0	0	0.1	0.1	0.1	0	0	0
Colombia	Ráquira	0.3	0.2	0.4	0	0	0	0.1	0.0	0.1	0	0	0
Colombia	Recetor	0.3	0.2	0.4	0	0	0	0.1	0.1	0.1	0	0	0
Colombia	Remedios	0.3	0.3	0.4	1	1	1	0.1	0.1	0.1	0	0	0
Colombia	Remolino	0.3	0.2	0.4	1	1	1	0.1	0.1	0.1	0	0	0
Colombia	Repelón	0.3	0.2	0.3	1	1	1	0.1	0.1	0.1	0	0	0
Colombia	Restrepo	0.3	0.3	0.4	3	2	4	0.1	0.1	0.1	1	0	1
Colombia	Restrepo	0.2	0.2	0.3	0	0	0	0.1	0.0	0.1	0	0	0
Colombia	Retiro	0.3	0.3	0.4	4	3	5	0.1	0.1	0.1	1	1	1
Colombia	Ricaurte	0.4	0.3	0.5	1	1	1	0.1	0.1	0.1	0	0	0
Colombia	Ricaurte	0.3	0.2	0.4	2	1	2	0.1	0.1	0.1	0	0	0
Colombia	Río de Oro	0.3	0.3	0.4	0	0	1	0.1	0.1	0.1	0	0	0
Colombia	Río Viejo	0.3	0.2	0.4	0	0	0	0.1	0.1	0.1	0	0	0
Colombia	Rioblanco	0.3	0.3	0.4	1	1	1	0.1	0.1	0.1	0	0	0
Colombia	Riofrío	0.2	0.2	0.3	0	0	0	0.1	0.0	0.1	0	0	0
Colombia	Riohacha	0.3	0.3	0.4	9	8	11	0.1	0.1	0.1	4	3	5
Colombia	Rionegro	0.3	0.2	0.4	1	1	1	0.1	0.1	0.1	0	0	0
Colombia	Rionegro	0.3	0.3	0.4	4	3	5	0.1	0.1	0.1	1	1	1
Colombia	Riosucio	0.4	0.3	0.5	2	2	3	0.1	0.1	0.1	0	0	0
Colombia	Riosucio	0.3	0.2	0.4	1	1	1	0.1	0.1	0.1	0	0	0
Colombia	Risaralda	0.3	0.2	0.4	1	1	1	0.1	0.0	0.1	0	0	0
Colombia	Rivera	0.3	0.3	0.4	1	1	1	0.1	0.1	0.1	0	0	0
Colombia	Roberto Payán	0.4	0.3	0.4	1	1	1	0.1	0.1	0.1	0	0	0
Colombia	Roldanillo	0.2	0.2	0.3	1	0	1	0.0	0.0	0.1	0	0	0
Colombia	Roncesvalles	0.3	0.2	0.4	0	0	0	0.1	0.1	0.1	0	0	0
Colombia	Rondón	0.3	0.2	0.4	0	0	0	0.1	0.0	0.1	0	0	0
Colombia	Rosas	0.4	0.3	0.4	0	0	0	0.1	0.1	0.1	0	0	0
Colombia	Rovira	0.3	0.3	0.4	1	1	1	0.1	0.1	0.1	0	0	0
Colombia	Sabana de Torres	0.3	0.2	0.4	1	0	1	0.1	0.1	0.1	0	0	0
Colombia	Sabanagrande	0.3	0.2	0.4	1	1	1	0.1	0.1	0.1	0	0	0
Colombia	Sabanalarga	0.3	0.3	0.4	0	0	1	0.1	0.1	0.1	0	0	0
Colombia	Sabanalarga	0.3	0.2	0.3	3	2	3	0.1	0.1	0.1	1	0	1
Colombia	Sabanalarga	0.3	0.2	0.4	0	0	0	0.1	0.1	0.1	0	0	0
Colombia	Sabaneta	0.3	0.3	0.4	2	2	3	0.1	0.1	0.1	0	0	1
Colombia	Saboyá	0.3	0.2	0.4	1	1	1	0.1	0.0	0.1	0	0	0
Colombia	Sácama	0.4	0.3	0.5	0	0	0	0.1	0.1	0.1	0	0	0
Colombia	Sáchica	0.3	0.2	0.3	0	0	0	0.1	0.0	0.1	0	0	0
Colombia	Saladoblanco	0.3	0.3	0.4	0	0	0	0.1	0.1	0.1	0	0	0
Colombia	Salamina	0.3	0.2	0.4	0	0	0	0.1	0.1	0.1	0	0	0
Colombia	Salamina	0.3	0.2	0.4	1	1	1	0.1	0.0	0.1	0	0	0
Colombia	Salazar de las Palmas	0.3	0.3	0.4	0	0	0	0.1	0.1	0.1	0	0	0

Table 3: Diarrhoea Mortality by Unit *(continued)*

Country	Unit	2000 mortality mean rate (per 10,000)	2000 mortality lower rate (per 10,000)	2000 mortality upper rate (per 10,000)	2000 mortality mean count	2000 mortality lower count	2000 mortality upper count	2017 mortality mean rate (per 10,000)	2017 mortality lower rate (per 10,000)	2017 mortality upper rate (per 10,000)	2017 mortality mean count	2017 mortality lower count	2017 mortality upper count
Colombia	Saldaña	0.3	0.2	0.4	0	0	0	0.1	0.1	0.1	0	0	0
Colombia	Salento	0.3	0.2	0.3	1	1	2	0.1	0.0	0.1	0	0	0
Colombia	Salgar	0.3	0.2	0.4	1	1	1	0.1	0.1	0.1	0	0	0
Colombia	Samacá	0.3	0.2	0.3	0	0	1	0.1	0.0	0.1	0	0	0
Colombia	Samaná	0.3	0.3	0.4	1	1	1	0.1	0.1	0.1	0	0	0
Colombia	Samaniego	0.4	0.3	0.4	2	1	2	0.1	0.1	0.1	0	0	0
Colombia	Sampués	0.3	0.3	0.4	2	1	2	0.1	0.1	0.1	0	0	0
Colombia	San Agustín	0.4	0.3	0.5	1	1	1	0.1	0.1	0.1	0	0	0
Colombia	San Alberto	0.3	0.2	0.4	1	1	1	0.1	0.1	0.1	0	0	0
Colombia	San Andrés	NA	NA	NA	NA	NA	NA	NA	NA	NA	NA	NA	NA
Colombia	San Andrés de Cuerquia	0.3	0.2	0.4	0	0	0	0.1	0.0	0.1	0	0	0
Colombia	San Andrés de Cuerquia	0.3	0.2	0.4	0	0	0	0.1	0.1	0.1	0	0	0
Colombia	San Andrés de Sotavento	0.3	0.2	0.4	2	2	2	0.1	0.1	0.1	0	0	1
Colombia	San Antero	0.3	0.2	0.4	2	1	2	0.1	0.1	0.1	0	0	0
Colombia	San Antonio	0.3	0.3	0.4	1	0	1	0.1	0.1	0.1	0	0	0
Colombia	San Antonio de Palmito	0.3	0.2	0.4	1	1	1	0.1	0.1	0.1	0	0	0
Colombia	San Antonio del Tequendama	0.3	0.3	0.4	0	0	1	0.1	0.1	0.1	0	0	0
Colombia	San Benito	0.3	0.2	0.4	0	0	0	0.1	0.0	0.1	0	0	0
Colombia	San Benito Abad	0.3	0.2	0.4	1	1	1	0.1	0.1	0.1	0	0	0
Colombia	San Bernardino de Sahagún	0.3	0.2	0.4	2	2	3	0.1	0.1	0.1	2	1	2
Colombia	San Bernardo	0.4	0.3	0.4	0	0	0	0.1	0.1	0.1	0	0	0
Colombia	San Bernardo	0.3	0.2	0.4	0	0	0	0.1	0.1	0.1	0	0	0
Colombia	San Bernardo del Viento	0.3	0.2	0.4	1	1	1	0.1	0.1	0.1	0	0	0
Colombia	San Calixto	0.3	0.3	0.4	1	0	1	0.1	0.1	0.1	0	0	0
Colombia	San Carlos	0.3	0.2	0.4	1	1	1	0.1	0.0	0.1	0	0	0
Colombia	San Carlos	0.3	0.3	0.4	1	0	1	0.1	0.1	0.1	0	0	0
Colombia	San Carlos de Guaroa	0.3	0.3	0.4	0	0	0	0.1	0.1	0.1	0	0	0
Colombia	San Cayetano	0.3	0.2	0.4	0	0	0	0.1	0.1	0.1	0	0	0
Colombia	San Cayetano	0.3	0.3	0.4	0	0	0	0.1	0.0	0.1	0	0	0
Colombia	San Diego	0.3	0.2	0.3	0	0	0	0.1	0.1	0.1	0	0	0
Colombia	San Eduardo	0.3	0.3	0.4	0	0	0	0.1	0.1	0.1	0	0	0
Colombia	San Estanislao de Kostka	0.3	0.2	0.4	1	0	1	0.1	0.1	0.1	0	0	0
Colombia	San Felipe	0.4	0.3	0.5	0	0	0	0.1	0.1	0.1	0	0	0
Colombia	San Fernando	0.3	0.3	0.4	1	0	1	0.1	0.1	0.1	0	0	0
Colombia	San Francisco	0.4	0.3	0.5	1	1	1	0.1	0.1	0.1	0	0	0
Colombia	San Francisco	0.3	0.2	0.4	0	0	0	0.1	0.1	0.1	0	0	0
Colombia	San Francisco	0.3	0.3	0.4	0	0	0	0.1	0.1	0.1	0	0	0
Colombia	San Gil	0.3	0.2	0.4	1	1	2	0.1	0.0	0.1	0	0	0
Colombia	San Jacinto	0.3	0.2	0.4	1	1	1	0.1	0.1	0.1	0	0	0
Colombia	San Jerónimo	0.3	0.2	0.4	0	0	0	0.1	0.1	0.1	0	0	0
Colombia	San Joaquín	0.3	0.2	0.4	0	0	0	0.1	0.0	0.1	0	0	0
Colombia	San José de Cúcuta	0.3	0.2	0.4	17	14	22	0.1	0.1	0.1	3	3	5
Colombia	San José de la Montaña	0.3	0.2	0.4	0	0	0	0.1	0.1	0.1	0	0	0
Colombia	San José de Miranda	0.3	0.2	0.4	0	0	0	0.1	0.0	0.1	0	0	0
Colombia	San Jose de Ocune	0.4	0.3	0.5	1	0	1	0.1	0.1	0.1	0	0	0
Colombia	San José de Pare	0.3	0.2	0.4	1	0	1	0.1	0.0	0.1	0	0	0
Colombia	San José del Fragua	0.4	0.3	0.5	0	0	1	0.1	0.1	0.1	0	0	0

Table 3: Diarrhoea Mortality by Unit *(continued)*

Country	Unit	2000 mortality mean rate (per 10,000)	2000 lower rate (per 10,000)	2000 upper rate (per 10,000)	2000 mean count	2000 lower count	2000 upper count	2017 mortality mean rate (per 10,000)	2017 lower rate (per 10,000)	2017 upper rate (per 10,000)	2017 mean count	2017 lower count	2017 upper count
Colombia	San José del Guaviare	0.4	0.3	0.5	3	3	4	0.1	0.1	0.1	1	1	1
Colombia	San José del Palmar	0.3	0.3	0.4	0	0	0	0.1	0.1	0.1	0	0	0
Colombia	San Juan de Arama	0.3	0.3	0.4	0	0	0	0.1	0.1	0.1	0	0	0
Colombia	San Juan de Betulia	0.3	0.2	0.4	1	1	1	0.1	0.1	0.1	0	0	0
Colombia	San Juan de Pasto	0.4	0.3	0.4	18	15	22	0.1	0.1	0.1	3	2	4
Colombia	San Juan de Río Seco	0.3	0.3	0.4	0	0	0	0.1	0.1	0.1	0	0	0
Colombia	San Juan de Urabá	0.3	0.2	0.3	1	0	1	0.1	0.0	0.1	0	0	0
Colombia	San Juan del Cesar	0.3	0.3	0.4	2	2	3	0.1	0.1	0.1	0	0	1
Colombia	San Juan Nepomuceno	0.3	0.3	0.4	1	1	2	0.1	0.1	0.1	0	0	0
Colombia	San Juanito	0.3	0.3	0.4	0	0	0	0.1	0.1	0.1	0	0	0
Colombia	San Lorenzo	0.4	0.3	0.5	1	1	1	0.1	0.1	0.1	0	0	0
Colombia	San Luis	0.3	0.3	0.4	0	0	0	0.1	0.1	0.1	0	0	0
Colombia	San Luís	0.3	0.2	0.4	1	0	1	0.1	0.1	0.1	0	0	0
Colombia	San Luis de Cubarral	0.3	0.3	0.4	1	0	1	0.1	0.1	0.1	0	0	0
Colombia	San Luis de Gaceno	0.3	0.3	0.4	0	0	0	0.1	0.1	0.1	0	0	0
Colombia	San Luis de Palenque	0.3	0.3	0.4	0	0	0	0.1	0.1	0.1	0	0	0
Colombia	San Marcos	0.3	0.2	0.4	2	2	2	0.1	0.1	0.1	0	0	1
Colombia	San Martín	0.3	0.2	0.4	1	1	1	0.1	0.1	0.1	0	0	0
Colombia	San Martín	0.3	0.3	0.4	1	1	2	0.1	0.1	0.1	0	0	0
Colombia	San Martín de Loba	0.3	0.3	0.4	1	1	1	0.1	0.1	0.1	0	0	0
Colombia	San Mateo	0.3	0.3	0.4	0	0	0	0.1	0.1	0.1	0	0	0
Colombia	San Miguel	0.3	0.2	0.4	0	0	0	0.1	0.0	0.1	0	0	0
Colombia	San Miguel de Mocoa	0.4	0.3	0.5	2	2	2	0.1	0.1	0.1	1	0	1
Colombia	San Miguel de Sema	0.3	0.2	0.4	0	0	0	0.1	0.0	0.1	0	0	0
Colombia	San Onofre	0.3	0.2	0.4	2	1	2	0.1	0.1	0.1	0	0	0
Colombia	San Pablo	0.4	0.3	0.4	0	0	1	0.1	0.1	0.1	0	0	0
Colombia	San Pablo	0.4	0.3	0.4	1	1	1	0.1	0.1	0.1	0	0	0
Colombia	San Pablo de Borbur	0.4	0.3	0.4	0	0	1	0.1	0.1	0.1	0	0	0
Colombia	San Pedro	0.3	0.2	0.4	1	1	1	0.1	0.1	0.1	0	0	0
Colombia	San Pedro	0.2	0.2	0.3	1	1	1	0.1	0.0	0.1	0	0	0
Colombia	San Pedro de Cartago	0.4	0.3	0.4	0	0	0	0.1	0.1	0.1	0	0	0
Colombia	San Pedro de los Milagros	0.3	0.2	0.4	1	1	1	0.1	0.1	0.1	0	0	0
Colombia	San Pedro de Urabá	0.3	0.2	0.4	1	1	1	0.1	0.1	0.1	0	0	0
Colombia	San Pelayo	0.3	0.2	0.4	2	1	2	0.1	0.0	0.1	0	0	0
Colombia	San Rafael	0.3	0.3	0.4	0	0	1	0.1	0.1	0.1	0	0	0
Colombia	San Roque	0.3	0.3	0.4	1	0	1	0.1	0.1	0.1	0	0	0
Colombia	San Sebastián	0.4	0.3	0.5	1	1	1	0.1	0.1	0.1	0	0	0
Colombia	San Sebastián de Buenavista	0.3	0.3	0.4	1	1	1	0.1	0.1	0.1	0	0	0
Colombia	San Sebastian de Mariquita	0.3	0.3	0.4	1	1	2	0.1	0.1	0.1	0	0	0

Table 3: Diarrhoea Mortality by Unit *(continued)*

Country	Unit	2000 mortality mean rate (per 10,000)	lower rate (per 10,000)	upper rate (per 10,000)	mean count	lower count	upper count	2017 mortality mean rate (per 10,000)	lower rate (per 10,000)	upper rate (per 10,000)	mean count	lower count	upper count
Colombia	San Vicente	0.3	0.2	0.4	1	0	1	0.1	0.0	0.1	0	0	0
Colombia	San Vicente de Chucurí	0.3	0.2	0.4	1	1	1	0.1	0.1	0.1	0	0	0
Colombia	San Vicente del Caguán	0.4	0.3	0.4	4	3	4	0.1	0.1	0.1	1	1	1
Colombia	San Zenón	0.3	0.3	0.4	1	1	1	0.1	0.1	0.1	0	0	0
Colombia	Sandoná	0.4	0.3	0.4	1	1	1	0.1	0.1	0.1	0	0	0
Colombia	Santa Ana	0.3	0.2	0.4	2	2	3	0.1	0.1	0.1	0	0	1
Colombia	Santa Bárbara	0.3	0.2	0.4	1	1	1	0.1	0.0	0.1	0	0	0
Colombia	Santa Bárbara	0.4	0.3	0.5	1	1	1	0.1	0.1	0.1	0	0	0
Colombia	Santa Bárbara	0.3	0.2	0.4	0	0	0	0.1	0.1	0.1	0	0	0
Colombia	Santa Catalina	0.3	0.2	0.4	1	1	1	0.1	0.1	0.1	0	0	0
Colombia	Santa Cruz	0.4	0.3	0.5	0	0	1	0.1	0.1	0.1	1	0	1
Colombia	Santa Cruz de Lorica	0.3	0.2	0.4	5	4	6	0.1	0.1	0.1	1	1	1
Colombia	Santa Fe de Antioquia	0.3	0.2	0.4	0	0	1	0.1	0.0	0.1	0	0	0
Colombia	Santa Helena del Opón	0.3	0.3	0.4	0	0	0	0.1	0.1	0.1	0	0	0
Colombia	Santa Isabel	0.3	0.3	0.4	0	0	0	0.1	0.1	0.1	0	0	0
Colombia	Santa Lucía	0.3	0.2	0.3	0	0	0	0.1	0.1	0.1	0	0	0
Colombia	Santa María	0.4	0.3	0.4	0	0	0	0.1	0.1	0.1	0	0	0
Colombia	Santa María	0.3	0.3	0.4	0	0	0	0.1	0.1	0.1	0	0	0
Colombia	Santa Marta (Dist. Esp.)	0.3	0.3	0.4	17	14	21	0.1	0.1	0.1	5	3	6
Colombia	Santa Rita	0.4	0.3	0.5	1	1	1	0.1	0.1	0.1	0	0	0
Colombia	Santa Rosa	0.3	0.2	0.4	1	0	1	0.1	0.1	0.1	0	0	0
Colombia	Santa Rosa	0.4	0.3	0.5	1	1	1	0.1	0.1	0.1	0	0	0
Colombia	Santa Rosa de Cabal	0.3	0.2	0.4	4	3	5	0.1	0.1	0.1	1	1	1
Colombia	Santa Rosa de Osos	0.3	0.2	0.4	1	1	1	0.1	0.0	0.1	0	0	0
Colombia	Santa Rosa de Viterbo	0.3	0.3	0.4	1	1	1	0.1	0.1	0.1	0	0	0
Colombia	Santa Rosa del Sur	0.3	0.3	0.4	1	1	1	0.1	0.1	0.1	0	0	0
Colombia	Santa Rosalía	0.4	0.3	0.5	0	0	0	0.1	0.1	0.1	0	0	0
Colombia	Santa Sofía	0.3	0.2	0.4	0	0	0	0.1	0.0	0.1	0	0	0
Colombia	Santafé de Bogotá	0.3	0.2	0.3	155	125	190	0.1	0.0	0.1	31	24	40
Colombia	Santana	0.3	0.2	0.4	0	0	0	0.1	0.1	0.1	0	0	0
Colombia	Santander de Quilichao	0.3	0.3	0.4	3	3	4	0.1	0.1	0.1	1	1	1
Colombia	Santiago	0.3	0.3	0.4	0	0	0	0.1	0.1	0.1	0	0	0
Colombia	Santiago	0.4	0.3	0.5	0	0	0	0.1	0.1	0.1	0	0	0
Colombia	Santiago de Cali	0.3	0.2	0.4	44	36	54	0.1	0.0	0.1	8	6	10
Colombia	Santo Domingo	0.3	0.3	0.4	0	0	1	0.1	0.1	0.1	0	0	0
Colombia	Santo Domingo de Silos	0.3	0.3	0.4	0	0	0	0.1	0.1	0.1	0	0	0
Colombia	Santo Tomás	0.3	0.2	0.4	1	0	1	0.1	0.1	0.1	0	0	0
Colombia	Santuario	0.3	0.2	0.4	0	0	0	0.1	0.0	0.1	0	0	0
Colombia	Sapuyes	0.4	0.3	0.4	1	1	1	0.1	0.1	0.1	0	0	0
Colombia	Saravena	0.3	0.3	0.4	2	1	2	0.1	0.1	0.1	0	0	1
Colombia	Sardinata	0.3	0.3	0.4	1	1	1	0.1	0.1	0.1	0	0	0
Colombia	Sasaima	0.3	0.3	0.4	0	0	1	0.1	0.1	0.1	0	0	0
Colombia	Sativanorte	0.3	0.2	0.4	0	0	0	0.1	0.0	0.1	0	0	0
Colombia	Sativasur	0.3	0.2	0.4	0	0	0	0.1	0.0	0.1	0	0	0
Colombia	Segovia	0.3	0.3	0.4	1	1	1	0.1	0.1	0.1	0	0	0
Colombia	Sesquilé	0.3	0.3	0.4	0	0	0	0.1	0.1	0.1	0	0	0
Colombia	Sevilla	0.3	0.2	0.3	2	1	2	0.1	0.0	0.1	0	0	0

Table 3: Diarrhoea Mortality by Unit *(continued)*

Country	Unit	2000 mortality						2017 mortality					
		mean rate (per 10,000)	lower rate (per 10,000)	upper rate (per 10,000)	mean count	lower count	upper count	mean rate (per 10,000)	lower rate (per 10,000)	upper rate (per 10,000)	mean count	lower count	upper count
Colombia	Siachoque	0.3	0.2	0.4	0	0	0	0.1	0.0	0.1	0	0	0
Colombia	Sibaté	0.3	0.2	0.4	1	1	1	0.1	0.1	0.1	0	0	0
Colombia	Sibundoy	0.4	0.3	0.4	0	0	0	0.1	0.1	0.1	0	0	0
Colombia	Silvania	0.3	0.3	0.4	1	1	1	0.1	0.1	0.1	0	0	0
Colombia	Silvia	0.4	0.3	0.4	2	2	3	0.1	0.1	0.1	0	0	0
Colombia	Simacota	0.3	0.2	0.4	0	0	0	0.1	0.1	0.1	0	0	0
Colombia	Simijaca	0.3	0.2	0.4	0	0	0	0.1	0.0	0.1	0	0	0
Colombia	Simití	0.3	0.3	0.4	1	1	2	0.1	0.1	0.1	0	0	0
Colombia	Sincé	0.3	0.2	0.4	1	1	1	0.1	0.1	0.1	0	0	0
Colombia	Sincelejo	0.3	0.2	0.4	8	7	10	0.1	0.1	0.1	2	1	2
Colombia	Sipí	0.4	0.3	0.5	0	0	0	0.1	0.1	0.1	0	0	0
Colombia	Soacha	0.3	0.3	0.4	8	7	10	0.1	0.1	0.1	2	2	3
Colombia	Soatá	0.3	0.2	0.4	0	0	0	0.1	0.0	0.1	0	0	0
Colombia	Socha	0.3	0.3	0.4	0	0	0	0.1	0.1	0.1	0	0	0
Colombia	Socorro	0.3	0.2	0.4	1	1	1	0.1	0.0	0.1	0	0	0
Colombia	Socotá	0.3	0.3	0.4	1	0	1	0.1	0.1	0.1	0	0	0
Colombia	Sogamoso	0.3	0.3	0.4	3	2	3	0.1	0.1	0.1	0	0	0
Colombia	Solano	0.4	0.3	0.5	2	1	2	0.1	0.1	0.1	1	0	1
Colombia	Soledad	0.3	0.2	0.3	10	8	13	0.1	0.0	0.1	3	2	4
Colombia	Somondoco	0.3	0.2	0.4	0	0	0	0.1	0.0	0.1	0	0	0
Colombia	Sonsón	0.3	0.3	0.4	1	1	2	0.1	0.1	0.1	0	0	0
Colombia	Sopetrán	0.3	0.2	0.4	0	0	0	0.1	0.0	0.1	0	0	0
Colombia	Soplaviento	0.3	0.2	0.4	0	0	0	0.1	0.1	0.1	0	0	0
Colombia	Sopó	0.3	0.3	0.4	2	2	3	0.1	0.1	0.1	1	0	1
Colombia	Sora	0.3	0.2	0.4	0	0	0	0.1	0.0	0.1	0	0	0
Colombia	Soracá	0.3	0.2	0.4	0	0	0	0.1	0.0	0.1	0	0	0
Colombia	Sotaquirá	0.3	0.2	0.4	0	0	0	0.1	0.0	0.1	0	0	0
Colombia	Sotará	0.4	0.3	0.4	1	1	2	0.1	0.1	0.1	0	0	0
Colombia	Suaita	0.3	0.2	0.4	1	0	1	0.1	0.0	0.1	0	0	0
Colombia	Suan	0.3	0.2	0.4	0	0	0	0.1	0.1	0.1	0	0	0
Colombia	Suárez	0.4	0.3	0.4	0	0	0	0.1	0.1	0.1	0	0	0
Colombia	Suárez	0.3	0.2	0.4	0	0	0	0.1	0.1	0.1	0	0	0
Colombia	Suaza	0.3	0.3	0.4	0	0	1	0.1	0.1	0.1	0	0	0
Colombia	Subachoque	0.3	0.2	0.4	1	1	1	0.1	0.1	0.1	0	0	0
Colombia	Sucre	0.3	0.2	0.4	1	1	1	0.1	0.1	0.1	0	0	0
Colombia	Sucre	0.3	0.3	0.4	0	0	0	0.1	0.1	0.1	0	0	0
Colombia	Suesca	0.3	0.3	0.4	1	0	1	0.1	0.1	0.1	0	0	0
Colombia	Supatá	0.3	0.3	0.4	0	0	0	0.1	0.1	0.1	0	0	0
Colombia	Supía	0.3	0.2	0.4	1	1	1	0.1	0.0	0.1	0	0	0
Colombia	Suratá	0.3	0.2	0.4	0	0	0	0.1	0.1	0.1	0	0	0
Colombia	Susa	0.3	0.2	0.4	0	0	0	0.1	0.0	0.1	0	0	0
Colombia	Susacón	0.3	0.2	0.4	0	0	0	0.1	0.0	0.1	0	0	0
Colombia	Sutamarchán	0.3	0.2	0.4	0	0	0	0.1	0.0	0.1	0	0	0
Colombia	Sutatausa	0.3	0.2	0.4	0	0	0	0.1	0.0	0.1	0	0	0
Colombia	Sutatenza	0.3	0.2	0.4	0	0	0	0.1	0.0	0.1	0	0	0
Colombia	Tabio	0.3	0.3	0.4	1	0	1	0.1	0.1	0.1	0	0	0
Colombia	Tadó	0.4	0.3	0.5	1	1	2	0.1	0.1	0.1	0	0	0
Colombia	Talaigua Nuevo	0.3	0.3	0.4	0	0	0	0.1	0.1	0.1	0	0	0
Colombia	Tamalameque	0.3	0.3	0.4	1	1	1	0.1	0.1	0.1	0	0	0
Colombia	Támara	0.3	0.3	0.4	0	0	0	0.1	0.1	0.1	0	0	0
Colombia	Tame	0.3	0.3	0.4	2	1	2	0.1	0.1	0.1	1	0	1
Colombia	Támesis	0.3	0.3	0.4	0	0	1	0.1	0.0	0.1	0	0	0
Colombia	Taminango	0.4	0.3	0.4	1	1	1	0.1	0.1	0.1	0	0	0
Colombia	Tangua	0.4	0.3	0.4	1	1	1	0.1	0.1	0.1	0	0	0
Colombia	Taraira	0.5	0.4	0.6	0	0	0	0.1	0.1	0.1	0	0	0
Colombia	Tarapacá	0.5	0.4	0.6	0	0	1	0.1	0.1	0.1	0	0	0
Colombia	Tarazá	0.3	0.3	0.4	1	1	1	0.1	0.1	0.1	0	0	0
Colombia	Tarquí	0.3	0.3	0.4	1	1	1	0.1	0.1	0.1	0	0	0
Colombia	Tarso	0.3	0.3	0.4	0	0	0	0.1	0.1	0.1	0	0	0
Colombia	Tasco	0.3	0.3	0.4	0	0	0	0.1	0.1	0.1	0	0	0

Table 3: Diarrhoea Mortality by Unit *(continued)*

Country	Unit	2000 mortality mean rate (per 10,000)	lower rate (per 10,000)	upper rate (per 10,000)	mean count	lower count	upper count	2017 mortality mean rate (per 10,000)	lower rate (per 10,000)	upper rate (per 10,000)	mean count	lower count	upper count
Colombia	Tauramena	0.3	0.3	0.4	0	0	0	0.1	0.1	0.1	0	0	0
Colombia	Tausa	0.3	0.2	0.4	0	0	0	0.1	0.0	0.1	0	0	0
Colombia	Tello	0.3	0.3	0.4	1	1	1	0.1	0.1	0.1	0	0	0
Colombia	Tena	0.3	0.2	0.4	0	0	0	0.1	0.1	0.1	0	0	0
Colombia	Tenerife	0.3	0.2	0.4	1	1	2	0.1	0.1	0.1	0	0	0
Colombia	Tenjo	0.3	0.3	0.4	1	1	1	0.1	0.1	0.1	0	0	0
Colombia	Tenza	0.3	0.2	0.4	0	0	0	0.1	0.0	0.1	0	0	0
Colombia	Teorama	0.3	0.3	0.4	1	1	1	0.1	0.1	0.1	0	0	0
Colombia	Teruel	0.3	0.3	0.4	0	0	1	0.1	0.1	0.1	0	0	0
Colombia	Tesalia	0.3	0.3	0.4	1	1	1	0.1	0.1	0.1	0	0	0
Colombia	Tibacuy	0.3	0.3	0.4	0	0	0	0.1	0.1	0.1	0	0	0
Colombia	Tibaná	0.3	0.2	0.4	0	0	0	0.1	0.0	0.1	0	0	0
Colombia	Tibasosa	0.3	0.3	0.4	0	0	1	0.1	0.1	0.1	0	0	0
Colombia	Tibirita	0.3	0.2	0.4	0	0	0	0.1	0.0	0.1	0	0	0
Colombia	Tibú	0.3	0.3	0.4	2	1	2	0.1	0.1	0.1	0	0	0
Colombia	Tierralta	0.3	0.3	0.4	3	3	4	0.1	0.1	0.1	1	1	1
Colombia	Timaná	0.3	0.3	0.4	1	1	2	0.1	0.1	0.1	0	0	0
Colombia	Timbío	0.4	0.3	0.4	1	1	1	0.1	0.1	0.1	0	0	0
Colombia	Timbiquí	0.4	0.3	0.5	1	1	1	0.1	0.1	0.1	0	0	0
Colombia	Tinjacá	0.3	0.2	0.4	0	0	0	0.1	0.0	0.1	0	0	0
Colombia	Tipacoque	0.3	0.2	0.4	0	0	0	0.1	0.0	0.1	0	0	0
Colombia	Titiribí	0.3	0.3	0.4	0	0	1	0.1	0.1	0.1	0	0	0
Colombia	Toca	0.3	0.2	0.4	0	0	1	0.1	0.0	0.1	0	0	0
Colombia	Tocaima	0.3	0.2	0.4	1	1	1	0.1	0.1	0.1	0	0	0
Colombia	Tocancipá	0.3	0.3	0.4	1	1	1	0.1	0.1	0.1	0	0	0
Colombia	Toguí	0.3	0.3	0.4	0	0	0	0.1	0.0	0.1	0	0	0
Colombia	Toledo	0.3	0.3	0.4	0	0	0	0.1	0.1	0.1	0	0	0
Colombia	Toledo	0.3	0.3	0.4	1	1	1	0.1	0.1	0.1	0	0	0
Colombia	Tolú	0.3	0.2	0.4	2	1	2	0.1	0.1	0.1	0	0	0
Colombia	Toluviejo	0.3	0.2	0.4	1	1	1	0.1	0.1	0.1	0	0	0
Colombia	Tona	0.3	0.2	0.4	1	1	1	0.1	0.1	0.1	0	0	0
Colombia	Topagá	0.3	0.3	0.4	0	0	1	0.1	0.1	0.1	0	0	0
Colombia	Topaipí	0.3	0.3	0.4	0	0	0	0.1	0.1	0.1	0	0	0
Colombia	Toribío	0.4	0.3	0.4	1	1	1	0.1	0.1	0.1	0	0	0
Colombia	Toro	0.2	0.2	0.3	0	0	0	0.1	0.0	0.1	0	0	0
Colombia	Tota	0.3	0.3	0.4	0	0	0	0.1	0.1	0.1	0	0	0
Colombia	Totoró	0.4	0.3	0.4	1	1	2	0.1	0.1	0.1	0	0	0
Colombia	Trinidad	0.3	0.3	0.4	0	0	0	0.1	0.1	0.1	0	0	0
Colombia	Trujillo	0.2	0.2	0.3	0	0	0	0.1	0.0	0.1	0	0	0
Colombia	Tubará	0.3	0.2	0.4	0	0	1	0.1	0.1	0.1	0	0	0
Colombia	Tuluá	0.2	0.2	0.3	3	3	4	0.1	0.0	0.1	1	0	1
Colombia	Tumaco	0.4	0.3	0.5	5	4	7	0.1	0.1	0.1	1	1	1
Colombia	Tunja	0.3	0.2	0.4	4	3	5	0.1	0.0	0.1	1	1	1
Colombia	Tunungua	0.3	0.3	0.4	0	0	0	0.1	0.1	0.1	0	0	0
Colombia	Túquerres	0.4	0.3	0.4	1	1	1	0.1	0.1	0.1	0	0	0
Colombia	Turbaco	0.3	0.2	0.4	2	2	2	0.1	0.1	0.1	1	0	1
Colombia	Turbaná	0.3	0.2	0.4	1	1	1	0.1	0.1	0.1	0	0	0
Colombia	Turbo	0.3	0.3	0.4	4	3	5	0.1	0.1	0.1	1	1	1
Colombia	Turmequé	0.3	0.2	0.4	0	0	0	0.1	0.0	0.1	0	0	0
Colombia	Tuta	0.3	0.2	0.4	0	0	0	0.1	0.0	0.1	0	0	0
Colombia	Tutazá	0.3	0.3	0.4	0	0	0	0.1	0.1	0.1	0	0	0
Colombia	Ubalá	0.3	0.3	0.4	0	0	0	0.1	0.1	0.1	0	0	0
Colombia	Ubaque	0.3	0.2	0.4	1	1	2	0.1	0.1	0.1	0	0	0
Colombia	Ulloa	0.3	0.2	0.3	0	0	1	0.1	0.0	0.1	0	0	0
Colombia	Umbita	0.3	0.2	0.4	0	0	0	0.1	0.0	0.1	0	0	0
Colombia	Une	0.3	0.2	0.4	0	0	0	0.1	0.1	0.1	0	0	0
Colombia	Unguía	0.4	0.3	0.5	1	1	1	0.1	0.1	0.1	0	0	0
Colombia	Uramita	0.3	0.2	0.4	0	0	0	0.1	0.1	0.1	0	0	0
Colombia	Uribia	0.3	0.3	0.4	6	5	8	0.1	0.1	0.1	3	2	4
Colombia	Urrao	0.3	0.3	0.4	1	1	2	0.1	0.1	0.1	0	0	0

Table 3: Diarrhoea Mortality by Unit *(continued)*

Country	Unit	2000 mortality mean rate (per 10,000)	lower rate (per 10,000)	upper rate (per 10,000)	mean count	lower count	upper count	2017 mortality mean rate (per 10,000)	lower rate (per 10,000)	upper rate (per 10,000)	mean count	lower count	upper count
Colombia	Urumita	0.3	0.2	0.4	1	1	1	0.1	0.1	0.1	0	0	0
Colombia	Usiacurí	0.3	0.2	0.3	0	0	0	0.1	0.1	0.1	0	0	0
Colombia	Utica	0.3	0.3	0.4	0	0	0	0.1	0.1	0.1	0	0	0
Colombia	Valdivia	0.3	0.3	0.4	1	0	1	0.1	0.1	0.1	0	0	0
Colombia	Valencia	0.3	0.2	0.4	1	1	1	0.1	0.1	0.1	0	0	0
Colombia	Valle de San José	0.3	0.2	0.3	0	0	0	0.1	0.0	0.1	0	0	0
Colombia	Valle de San Juan	0.3	0.2	0.4	0	0	0	0.1	0.1	0.1	0	0	0
Colombia	Valle del Guamuez	0.4	0.3	0.5	3	2	3	0.1	0.1	0.1	1	1	1
Colombia	Valledupar	0.3	0.2	0.3	13	11	16	0.1	0.1	0.1	4	3	5
Colombia	Valparaíso	0.3	0.2	0.4	0	0	0	0.1	0.0	0.1	0	0	0
Colombia	Valparaíso	0.4	0.3	0.5	1	1	2	0.1	0.1	0.1	0	0	0
Colombia	Vegachí	0.3	0.3	0.4	0	0	0	0.1	0.1	0.1	0	0	0
Colombia	Vélez	0.3	0.2	0.4	1	0	1	0.1	0.0	0.1	0	0	0
Colombia	Venadillo	0.3	0.2	0.4	1	0	1	0.1	0.1	0.1	0	0	0
Colombia	Venecia	0.3	0.2	0.4	0	0	0	0.1	0.1	0.1	0	0	0
Colombia	Venecia	0.3	0.3	0.4	0	0	0	0.1	0.1	0.1	0	0	0
Colombia	Ventaquemada	0.3	0.2	0.4	0	0	1	0.1	0.0	0.1	0	0	0
Colombia	Vergara	0.4	0.3	0.4	0	0	0	0.1	0.1	0.1	0	0	0
Colombia	Versalles	0.3	0.2	0.3	0	0	0	0.1	0.0	0.1	0	0	0
Colombia	Vetas	0.3	0.2	0.4	0	0	0	0.1	0.0	0.1	0	0	0
Colombia	Vianí	0.3	0.3	0.4	0	0	0	0.1	0.1	0.1	0	0	0
Colombia	Victoria	0.3	0.3	0.4	0	0	0	0.1	0.1	0.1	0	0	0
Colombia	Vigía del Fuerte	0.4	0.3	0.5	0	0	0	0.1	0.1	0.1	0	0	0
Colombia	Vijes	0.2	0.2	0.3	0	0	0	0.1	0.0	0.1	0	0	0
Colombia	Villa Caro	0.3	0.3	0.4	0	0	0	0.1	0.1	0.1	0	0	0
Colombia	Villa de Leyva	0.3	0.2	0.4	0	0	0	0.1	0.0	0.1	0	0	0
Colombia	Villa de San Diego de Ubaté	0.3	0.2	0.4	1	1	1	0.1	0.0	0.1	0	0	0
Colombia	Villa del Rosario	0.3	0.2	0.4	6	5	8	0.1	0.1	0.1	1	1	2
Colombia	Villagarzón	0.4	0.3	0.5	1	1	1	0.1	0.1	0.1	0	0	0
Colombia	Villagómez	0.3	0.3	0.4	0	0	0	0.1	0.1	0.1	0	0	0
Colombia	Villahermosa	0.3	0.3	0.4	0	0	1	0.1	0.1	0.1	0	0	0
Colombia	Villamaría	0.3	0.2	0.4	2	2	3	0.1	0.1	0.1	0	0	0
Colombia	Villanueva	0.3	0.3	0.4	2	1	2	0.1	0.1	0.1	0	0	1
Colombia	Villanueva	0.3	0.3	0.4	1	1	1	0.1	0.1	0.1	0	0	0
Colombia	Villanueva	0.3	0.2	0.4	1	0	1	0.1	0.1	0.1	0	0	0
Colombia	Villanueva	0.3	0.2	0.4	0	0	0	0.1	0.0	0.1	0	0	0
Colombia	Villapinzón	0.3	0.3	0.4	1	0	1	0.1	0.1	0.1	0	0	0
Colombia	Villarrica	0.3	0.3	0.4	0	0	0	0.1	0.1	0.1	0	0	0
Colombia	Villavicencio	0.3	0.3	0.4	11	9	14	0.1	0.1	0.1	3	2	3
Colombia	Villavieja	0.3	0.2	0.4	0	0	1	0.1	0.1	0.1	0	0	0
Colombia	Villeta	0.3	0.3	0.4	1	1	1	0.1	0.1	0.1	0	0	0
Colombia	Viotá	0.3	0.3	0.4	0	0	1	0.1	0.1	0.1	0	0	0
Colombia	Viracachá	0.3	0.2	0.4	0	0	0	0.1	0.0	0.1	0	0	0
Colombia	Vista Hermosa	0.3	0.3	0.4	1	1	1	0.1	0.1	0.1	0	0	0
Colombia	Viterbo	0.3	0.2	0.3	0	0	1	0.1	0.0	0.1	0	0	0
Colombia	Yacopí	0.3	0.3	0.4	1	1	1	0.1	0.1	0.1	0	0	0
Colombia	Yacuanquer	0.4	0.3	0.4	0	0	0	0.1	0.1	0.1	0	0	0
Colombia	Yaguará	0.3	0.3	0.4	0	0	0	0.1	0.1	0.1	0	0	0
Colombia	Yalí	0.3	0.3	0.4	0	0	0	0.1	0.1	0.1	0	0	0
Colombia	Yarumal	0.3	0.3	0.4	1	1	2	0.1	0.0	0.1	0	0	0
Colombia	Yavaraté	0.5	0.4	0.6	0	0	0	0.1	0.1	0.1	0	0	0
Colombia	Yolombó	0.3	0.3	0.4	1	1	1	0.1	0.1	0.1	0	0	0
Colombia	Yondó	0.3	0.3	0.4	0	0	0	0.1	0.1	0.1	0	0	0
Colombia	Yopal	0.3	0.3	0.4	3	2	4	0.1	0.1	0.1	1	1	2
Colombia	Yotoco	0.2	0.2	0.3	0	0	0	0.0	0.0	0.1	0	0	0
Colombia	Yumbo	0.3	0.2	0.3	4	3	4	0.1	0.0	0.1	1	0	1
Colombia	Zambrano	0.3	0.2	0.4	0	0	0	0.1	0.1	0.1	0	0	0
Colombia	Zapatoca	0.3	0.2	0.4	0	0	0	0.1	0.0	0.1	0	0	0

Table 3: Diarrhoea Mortality by Unit *(continued)*

Country	Unit	2000 mortality mean rate (per 10,000)	lower rate (per 10,000)	upper rate (per 10,000)	mean count	lower count	upper count	2017 mortality mean rate (per 10,000)	lower rate (per 10,000)	upper rate (per 10,000)	mean count	lower count	upper count
Colombia	Zaragoza	0.3	0.3	0.4	1	1	1	0.1	0.1	0.1	0	0	0
Colombia	Zarzal	0.3	0.2	0.3	1	1	2	0.1	0.0	0.1	0	0	0
Colombia	Zetaquirá	0.3	0.3	0.4	0	0	0	0.1	0.1	0.1	0	0	0
Colombia	Zipacón	0.3	0.3	0.4	0	0	0	0.1	0.1	0.1	0	0	0
Colombia	Zipaquirá	0.3	0.2	0.4	2	2	3	0.1	0.1	0.1	1	0	1
Costa Rica	Abangares	0.1	0.1	0.1	0	0	0	0.0	0.0	0.0	0	0	0
Costa Rica	Acosta	0.1	0.1	0.1	0	0	0	0.0	0.0	0.0	0	0	0
Costa Rica	Aguirre	0.1	0.1	0.1	0	0	0	0.0	0.0	0.0	0	0	0
Costa Rica	Alajuela	0.1	0.1	0.1	2	1	3	0.0	0.0	0.0	1	0	1
Costa Rica	Alajuelita	0.1	0.1	0.1	1	1	1	0.0	0.0	0.0	0	0	0
Costa Rica	Alfaro Ruiz	0.1	0.1	0.1	0	0	0	0.0	0.0	0.0	0	0	0
Costa Rica	Alvarado	0.1	0.1	0.1	0	0	0	0.0	0.0	0.0	0	0	0
Costa Rica	Aserrí	0.1	0.1	0.1	1	0	1	0.0	0.0	0.0	0	0	0
Costa Rica	Atenas	0.1	0.1	0.1	0	0	0	0.0	0.0	0.0	0	0	0
Costa Rica	Bagaces	0.1	0.1	0.1	0	0	0	0.0	0.0	0.0	0	0	0
Costa Rica	Barva	0.1	0.1	0.1	0	0	0	0.0	0.0	0.0	0	0	0
Costa Rica	Belén	0.1	0.1	0.1	0	0	0	0.0	0.0	0.0	0	0	0
Costa Rica	Buenos Aires	0.1	0.1	0.1	1	0	1	0.0	0.0	0.0	0	0	0
Costa Rica	Cañas	0.1	0.1	0.1	0	0	0	0.0	0.0	0.0	0	0	0
Costa Rica	Carrillo	0.1	0.1	0.1	0	0	0	0.0	0.0	0.0	0	0	0
Costa Rica	Cartago	0.1	0.1	0.1	1	1	2	0.0	0.0	0.0	0	0	0
Costa Rica	Corredores	0.1	0.1	0.1	0	0	1	0.0	0.0	0.0	0	0	0
Costa Rica	Coto Brus	0.1	0.1	0.1	0	0	1	0.0	0.0	0.0	0	0	0
Costa Rica	Curridabat	0.1	0.1	0.1	0	0	1	0.0	0.0	0.0	0	0	0
Costa Rica	Desamparados	0.1	0.1	0.1	2	1	3	0.0	0.0	0.0	1	0	1
Costa Rica	Dota	0.1	0.1	0.1	0	0	0	0.0	0.0	0.0	0	0	0
Costa Rica	El Guarco	0.1	0.1	0.1	0	0	1	0.0	0.0	0.0	0	0	0
Costa Rica	Escazú	0.1	0.1	0.1	1	0	1	0.0	0.0	0.0	0	0	0
Costa Rica	Esparza	0.1	0.1	0.1	0	0	1	0.0	0.0	0.0	0	0	0
Costa Rica	Flores	0.1	0.1	0.1	0	0	0	0.0	0.0	0.0	0	0	0
Costa Rica	Garabito	0.1	0.1	0.1	0	0	0	0.0	0.0	0.0	0	0	0
Costa Rica	Goicoechea	0.1	0.1	0.1	1	0	1	0.0	0.0	0.0	0	0	0
Costa Rica	Golfito	0.1	0.1	0.1	0	0	1	0.0	0.0	0.0	0	0	0
Costa Rica	Grecia	0.1	0.1	0.1	1	0	1	0.0	0.0	0.0	0	0	0
Costa Rica	Guácimo	0.1	0.1	0.1	0	0	1	0.0	0.0	0.0	0	0	0
Costa Rica	Guatuso	0.1	0.1	0.1	0	0	0	0.0	0.0	0.0	0	0	0
Costa Rica	Heredia	0.1	0.1	0.1	1	0	1	0.0	0.0	0.0	0	0	0
Costa Rica	Hojancha	0.1	0.1	0.1	0	0	0	0.0	0.0	0.0	0	0	0
Costa Rica	Jiménez	0.1	0.1	0.1	0	0	0	0.0	0.0	0.0	0	0	0
Costa Rica	La Cruz	0.1	0.1	0.1	0	0	0	0.0	0.0	0.0	0	0	0
Costa Rica	La Unión	0.1	0.1	0.1	1	0	1	0.0	0.0	0.0	0	0	0
Costa Rica	León Cortés	0.1	0.1	0.1	0	0	0	0.0	0.0	0.0	0	0	0
Costa Rica	Liberia	0.1	0.1	0.1	1	0	1	0.0	0.0	0.0	0	0	0
Costa Rica	Limón	0.1	0.1	0.1	1	1	1	0.0	0.0	0.1	0	0	0
Costa Rica	Los Chiles	0.1	0.1	0.1	0	0	0	0.0	0.0	0.0	0	0	0
Costa Rica	Matina	0.1	0.1	0.1	0	0	1	0.0	0.0	0.0	0	0	0
Costa Rica	Montes de Oca	0.1	0.1	0.1	0	0	1	0.0	0.0	0.0	0	0	0
Costa Rica	Montes de Oro	0.1	0.1	0.1	0	0	0	0.0	0.0	0.0	0	0	0
Costa Rica	Mora	0.1	0.1	0.1	0	0	0	0.0	0.0	0.0	0	0	0
Costa Rica	Moravia	0.1	0.1	0.1	0	0	0	0.0	0.0	0.0	0	0	0
Costa Rica	Nandayure	0.1	0.1	0.1	0	0	0	0.0	0.0	0.0	0	0	0
Costa Rica	Naranjo	0.1	0.1	0.1	0	0	0	0.0	0.0	0.0	0	0	0
Costa Rica	Nicoya	0.1	0.1	0.1	0	0	1	0.0	0.0	0.0	0	0	0
Costa Rica	Oreamuno	0.1	0.1	0.1	0	0	0	0.0	0.0	0.0	0	0	0
Costa Rica	Orotina	0.1	0.1	0.1	0	0	0	0.0	0.0	0.0	0	0	0
Costa Rica	Osa	0.1	0.1	0.1	0	0	0	0.0	0.0	0.0	0	0	0
Costa Rica	Palmares	0.1	0.1	0.1	0	0	0	0.0	0.0	0.0	0	0	0
Costa Rica	Paraíso	0.1	0.1	0.1	0	0	1	0.0	0.0	0.0	0	0	0
Costa Rica	Parrita	0.1	0.1	0.1	0	0	0	0.0	0.0	0.0	0	0	0
Costa Rica	Pérez Zeledón	0.1	0.1	0.1	1	1	2	0.0	0.0	0.0	0	0	0

Table 3: Diarrhoea Mortality by Unit *(continued)*

Country	Unit	2000 mortality mean rate (per 10,000)	2000 mortality lower rate (per 10,000)	2000 mortality upper rate (per 10,000)	2000 mortality mean count	2000 mortality lower count	2000 mortality upper count	2017 mortality mean rate (per 10,000)	2017 mortality lower rate (per 10,000)	2017 mortality upper rate (per 10,000)	2017 mortality mean count	2017 mortality lower count	2017 mortality upper count
Costa Rica	Poás	0.1	0.1	0.1	0	0	0	0.0	0.0	0.0	0	0	0
Costa Rica	Pococí	0.1	0.1	0.1	1	1	2	0.0	0.0	0.0	0	0	1
Costa Rica	Puntarenas	0.1	0.1	0.1	1	1	1	0.0	0.0	0.0	0	0	0
Costa Rica	Puriscal	0.1	0.1	0.1	0	0	0	0.0	0.0	0.0	0	0	0
Costa Rica	San Carlos	0.1	0.1	0.1	1	1	2	0.0	0.0	0.0	0	0	1
Costa Rica	San Isidro	0.1	0.1	0.1	0	0	0	0.0	0.0	0.0	0	0	0
Costa Rica	San José	0.1	0.1	0.1	2	1	2	0.0	0.0	0.0	0	0	1
Costa Rica	San Mateo	0.1	0.1	0.1	0	0	0	0.0	0.0	0.0	0	0	0
Costa Rica	San Pablo	0.1	0.1	0.1	0	0	0	0.0	0.0	0.0	0	0	0
Costa Rica	San Rafael	0.1	0.1	0.1	0	0	0	0.0	0.0	0.0	0	0	0
Costa Rica	San Ramón	0.1	0.1	0.1	1	0	1	0.0	0.0	0.0	0	0	0
Costa Rica	Santa Ana	0.1	0.1	0.1	0	0	1	0.0	0.0	0.0	0	0	0
Costa Rica	Santa Bárbara	0.1	0.1	0.1	0	0	0	0.0	0.0	0.0	0	0	0
Costa Rica	Santa Cruz	0.1	0.1	0.1	0	0	1	0.0	0.0	0.0	0	0	0
Costa Rica	Santo Domingo	0.1	0.1	0.1	0	0	1	0.0	0.0	0.0	0	0	0
Costa Rica	Sarapiquí	0.1	0.1	0.1	0	0	1	0.0	0.0	0.0	0	0	0
Costa Rica	Siquirres	0.1	0.1	0.1	1	1	1	0.0	0.0	0.0	0	0	0
Costa Rica	Talamanca	0.1	0.1	0.1	0	0	0	0.0	0.0	0.0	0	0	0
Costa Rica	Tarrazú	0.1	0.1	0.1	0	0	0	0.0	0.0	0.0	0	0	0
Costa Rica	Tibás	0.1	0.1	0.1	0	0	0	0.0	0.0	0.0	0	0	0
Costa Rica	Tilarán	0.1	0.1	0.1	0	0	0	0.0	0.0	0.0	0	0	0
Costa Rica	Turrialba	0.1	0.1	0.1	1	0	1	0.0	0.0	0.0	0	0	0
Costa Rica	Turrubares	0.1	0.1	0.1	0	0	0	0.0	0.0	0.0	0	0	0
Costa Rica	Upala	0.1	0.1	0.1	0	0	1	0.0	0.0	0.0	0	0	0
Costa Rica	Valverde Vega	0.1	0.1	0.1	0	0	0	0.0	0.0	0.0	0	0	0
Costa Rica	Vásquez de Coronado	0.1	0.1	0.1	0	0	0	0.0	0.0	0.0	0	0	0
Dominican Republic	Altamira	0.7	0.6	0.8	2	2	2	0.2	0.1	0.2	0	0	0
Dominican Republic	Arenoso	0.7	0.6	0.9	1	1	2	0.2	0.1	0.2	0	0	0
Dominican Republic	Azua de Compostela	0.8	0.7	0.9	10	9	11	0.2	0.2	0.2	2	1	2
Dominican Republic	Bajos de Haina	0.6	0.5	0.7	7	6	8	0.2	0.1	0.2	2	2	3
Dominican Republic	Baní	0.7	0.6	0.8	12	11	14	0.2	0.1	0.2	4	3	4
Dominican Republic	Banica	0.9	0.8	1.0	1	1	1	0.2	0.2	0.3	0	0	0
Dominican Republic	Bayaguana	0.7	0.6	0.8	4	3	4	0.2	0.1	0.2	1	0	1
Dominican Republic	Boca Chica	0.7	0.6	0.8	10	9	12	0.2	0.1	0.2	4	3	4
Dominican Republic	Bohechio	0.8	0.7	0.9	1	1	1	0.2	0.2	0.2	0	0	0
Dominican Republic	Bonao	0.7	0.6	0.8	11	10	13	0.2	0.2	0.2	2	2	3
Dominican Republic	Cabral	1.0	0.8	1.1	2	2	2	0.2	0.2	0.3	0	0	0
Dominican Republic	Cabrera	0.8	0.6	0.9	2	1	2	0.2	0.1	0.2	0	0	0
Dominican Republic	Cambita Garabito	0.7	0.6	0.8	4	3	4	0.2	0.1	0.2	1	1	1
Dominican Republic	Castañuela	0.8	0.7	0.9	2	1	2	0.2	0.2	0.2	0	0	0
Dominican Republic	Castillo	0.7	0.6	0.8	1	1	1	0.2	0.2	0.2	0	0	0
Dominican Republic	Cayetano Germosén	0.7	0.6	0.8	1	1	1	0.2	0.1	0.2	0	0	0
Dominican Republic	Cevicos	0.7	0.6	0.9	2	2	2	0.2	0.2	0.2	0	0	0

Table 3: Diarrhoea Mortality by Unit *(continued)*

Country	Unit	2000 mortality mean rate (per 10,000)	lower rate (per 10,000)	upper rate (per 10,000)	mean count	lower count	upper count	2017 mortality mean rate (per 10,000)	lower rate (per 10,000)	upper rate (per 10,000)	mean count	lower count	upper count
Dominican Republic	Comendador	1.0	0.9	1.1	5	4	5	0.2	0.2	0.3	1	1	1
Dominican Republic	Concepción de la Vega	0.7	0.6	0.8	20	17	23	0.2	0.2	0.2	4	3	5
Dominican Republic	Constanza	0.7	0.6	0.8	5	4	5	0.2	0.2	0.2	1	1	1
Dominican Republic	Consuelo	0.6	0.5	0.7	2	2	3	0.2	0.1	0.2	0	0	0
Dominican Republic	Cotuí	0.7	0.6	0.8	7	6	8	0.2	0.2	0.2	1	1	2
Dominican Republic	Cristobal	1.0	0.9	1.1	1	1	1	0.2	0.2	0.3	0	0	0
Dominican Republic	Dajabón	0.7	0.6	0.8	4	3	4	0.2	0.2	0.2	1	1	1
Dominican Republic	Distrito Nacional	0.6	0.5	0.7	65	56	75	0.2	0.1	0.2	14	11	17
Dominican Republic	Duvergé	0.9	0.8	1.0	3	2	3	0.2	0.2	0.2	0	0	0
Dominican Republic	El Cercado	0.9	0.8	1.0	4	3	4	0.2	0.2	0.3	0	0	1
Dominican Republic	El Factor	0.8	0.7	0.9	2	2	2	0.2	0.2	0.3	1	0	1
Dominican Republic	El Llano	1.1	1.0	1.2	2	1	2	0.3	0.2	0.3	0	0	0
Dominican Republic	El Peñón	0.9	0.8	1.0	0	0	0	0.2	0.2	0.2	0	0	0
Dominican Republic	El Pino	0.7	0.7	0.8	1	1	1	0.2	0.2	0.2	0	0	0
Dominican Republic	El Valle	0.7	0.6	0.8	1	1	1	0.2	0.1	0.2	0	0	0
Dominican Republic	Enriquillo	0.9	0.8	1.0	1	1	1	0.2	0.2	0.3	0	0	0
Dominican Republic	Esperalvillo	0.8	0.7	1.0	2	2	2	0.2	0.2	0.3	0	0	0
Dominican Republic	Esperanza	0.7	0.6	0.8	6	5	7	0.2	0.2	0.2	2	1	2
Dominican Republic	Estebania	0.7	0.6	0.8	1	1	1	0.2	0.2	0.2	0	0	0
Dominican Republic	Fantino	0.7	0.6	0.8	2	1	2	0.2	0.1	0.2	0	0	0
Dominican Republic	Fundación	0.9	0.7	1.0	2	2	3	0.2	0.2	0.2	0	0	1
Dominican Republic	Galvan	1.0	0.9	1.1	3	3	4	0.2	0.2	0.3	1	1	1
Dominican Republic	Gaspar Hernández	0.7	0.6	0.8	3	2	3	0.2	0.2	0.2	1	1	1
Dominican Republic	Guananico	0.7	0.6	0.8	0	0	0	0.2	0.1	0.2	0	0	0
Dominican Republic	Guayabal	0.8	0.7	0.9	0	0	0	0.2	0.1	0.2	0	0	0
Dominican Republic	Guayacanes	0.7	0.5	0.8	1	1	1	0.2	0.1	0.2	0	0	0
Dominican Republic	Guaymate	0.7	0.6	0.9	2	2	3	0.2	0.2	0.2	0	0	0
Dominican Republic	Guayubín	0.8	0.7	0.9	3	2	3	0.2	0.2	0.2	1	1	1
Dominican Republic	Guerra	0.7	0.6	0.8	5	5	6	0.2	0.1	0.2	2	1	2
Dominican Republic	Hato Mayor del Rey	0.6	0.5	0.8	4	4	5	0.2	0.1	0.2	1	1	1

Table 3: Diarrhoea Mortality by Unit *(continued)*

Country	Unit	2000 mortality mean rate (per 10,000)	2000 mortality lower rate (per 10,000)	2000 mortality upper rate (per 10,000)	2000 mortality mean count	2000 mortality lower count	2000 mortality upper count	2017 mortality mean rate (per 10,000)	2017 mortality lower rate (per 10,000)	2017 mortality upper rate (per 10,000)	2017 mortality mean count	2017 mortality lower count	2017 mortality upper count
Dominican Republic	Hondo Valle	1.0	0.9	1.1	2	1	2	0.2	0.2	0.3	0	0	0
Dominican Republic	Hostos	0.7	0.6	0.8	1	0	1	0.2	0.2	0.2	0	0	0
Dominican Republic	Imbert	0.7	0.6	0.8	2	2	3	0.2	0.2	0.2	0	0	0
Dominican Republic	Jamao al Norte	0.7	0.6	0.8	1	1	1	0.2	0.2	0.2	0	0	0
Dominican Republic	Janico	0.7	0.6	0.8	2	2	2	0.2	0.1	0.2	0	0	0
Dominican Republic	Jaquimeyes	0.9	0.8	1.0	1	1	1	0.2	0.2	0.2	0	0	0
Dominican Republic	Jarabacoa	0.7	0.6	0.8	6	5	6	0.2	0.1	0.2	1	1	1
Dominican Republic	Jima Abajo	0.8	0.7	0.9	2	2	2	0.2	0.2	0.2	1	1	1
Dominican Republic	Jimaní	0.9	0.8	1.1	3	2	3	0.2	0.2	0.3	1	1	1
Dominican Republic	Juan de Herrera	0.8	0.7	0.9	2	2	2	0.2	0.2	0.2	0	0	0
Dominican Republic	Juan Santiago	1.0	0.9	1.1	1	1	1	0.2	0.2	0.3	0	0	0
Dominican Republic	La Cienaga	0.9	0.8	1.0	1	1	1	0.2	0.2	0.2	0	0	0
Dominican Republic	La Descubierta	1.0	0.9	1.2	1	1	1	0.3	0.2	0.3	0	0	0
Dominican Republic	La Isabela	0.7	0.6	0.8	1	1	2	0.2	0.2	0.2	0	0	0
Dominican Republic	La Laguna de Nisibón	0.7	0.6	0.8	1	1	1	0.2	0.1	0.2	0	0	0
Dominican Republic	La Mata	0.7	0.6	0.8	4	3	4	0.2	0.2	0.2	1	0	1
Dominican Republic	La Romana	0.6	0.5	0.7	5	4	6	0.2	0.1	0.2	1	1	2
Dominican Republic	Laguna Salada	0.7	0.6	0.8	2	2	3	0.2	0.2	0.2	1	1	1
Dominican Republic	Las Charcas	0.7	0.6	0.8	1	1	1	0.2	0.2	0.2	0	0	0
Dominican Republic	Las Guaranas	0.7	0.6	0.9	1	1	2	0.2	0.2	0.2	0	0	0
Dominican Republic	Las Matas de Farfan	0.9	0.8	1.0	6	5	7	0.2	0.2	0.2	1	1	1
Dominican Republic	Las Matas de Santa Cruz	0.8	0.7	0.9	1	1	1	0.2	0.2	0.2	0	0	0
Dominican Republic	Las Salinas	0.9	0.8	1.1	1	1	1	0.2	0.2	0.3	0	0	0
Dominican Republic	Las Terrenas	0.7	0.6	0.9	2	1	2	0.2	0.1	0.2	0	0	1
Dominican Republic	Las Yayas de Viajama	0.8	0.7	0.9	2	2	2	0.2	0.2	0.2	0	0	1
Dominican Republic	Licey al Medio	0.7	0.6	0.8	2	2	2	0.2	0.1	0.2	0	0	0
Dominican Republic	Loma de Cabrera	0.7	0.6	0.8	2	2	2	0.2	0.2	0.2	0	0	0
Dominican Republic	Los Alcarrizos	0.7	0.6	0.8	17	15	20	0.2	0.1	0.2	5	4	7
Dominican Republic	Los Almácigos	0.8	0.7	0.9	1	1	1	0.2	0.2	0.2	0	0	0
Dominican Republic	Los Cacaos	0.7	0.6	0.8	1	1	1	0.2	0.2	0.2	0	0	0

Table 3: Diarrhoea Mortality by Unit *(continued)*

Country	Unit	2000 mortality mean rate (per 10,000)	lower rate (per 10,000)	upper rate (per 10,000)	mean count	lower count	upper count	2017 mortality mean rate (per 10,000)	lower rate (per 10,000)	upper rate (per 10,000)	mean count	lower count	upper count
Dominican Republic	Los Hidalgos	0.7	0.6	0.8	1	1	1	0.2	0.2	0.2	0	0	0
Dominican Republic	Los Llanos	0.7	0.6	0.8	3	2	3	0.2	0.1	0.2	1	0	1
Dominican Republic	Los Rios	1.0	0.8	1.1	1	1	1	0.2	0.2	0.3	0	0	0
Dominican Republic	Luperon	0.7	0.6	0.8	2	1	2	0.2	0.2	0.2	0	0	0
Dominican Republic	Maimón	0.8	0.7	0.9	2	2	2	0.2	0.2	0.3	0	0	0
Dominican Republic	Mao	0.7	0.6	0.8	6	5	6	0.2	0.2	0.2	2	1	2
Dominican Republic	Mella	0.9	0.8	1.1	1	1	1	0.2	0.2	0.3	0	0	0
Dominican Republic	Miches	0.7	0.6	0.8	2	2	3	0.2	0.1	0.2	0	0	0
Dominican Republic	Moca	0.7	0.6	0.8	12	10	14	0.2	0.2	0.2	3	3	4
Dominican Republic	Monción	0.7	0.6	0.8	1	1	1	0.2	0.2	0.2	0	0	0
Dominican Republic	Monte Plata	0.8	0.7	0.9	5	4	6	0.2	0.2	0.2	1	1	1
Dominican Republic	Montellano	0.7	0.6	0.8	2	2	2	0.2	0.2	0.3	0	0	1
Dominican Republic	Nagua	0.7	0.6	0.9	6	5	6	0.2	0.1	0.2	1	1	2
Dominican Republic	Neyba	1.0	0.9	1.1	3	3	4	0.2	0.2	0.3	1	1	1
Dominican Republic	Nigua	0.7	0.6	0.8	3	2	3	0.2	0.1	0.2	1	0	1
Dominican Republic	Nizao	0.7	0.6	0.8	2	1	2	0.2	0.1	0.2	1	1	1
Dominican Republic	Padre Las Casas	0.8	0.7	0.9	3	3	3	0.2	0.2	0.2	0	0	0
Dominican Republic	Paraiso	0.9	0.8	1.0	2	2	2	0.2	0.2	0.3	0	0	0
Dominican Republic	Partido	0.7	0.6	0.8	1	1	1	0.2	0.2	0.2	0	0	0
Dominican Republic	Pedernales	0.9	0.8	1.0	3	3	4	0.2	0.2	0.2	1	1	1
Dominican Republic	Pedro Brand	0.7	0.6	0.8	5	5	6	0.2	0.1	0.2	2	2	2
Dominican Republic	Pedro Santana	1.0	0.9	1.1	1	1	1	0.2	0.2	0.3	0	0	0
Dominican Republic	Pepillo Salcedo	0.8	0.7	0.9	1	1	1	0.2	0.2	0.2	0	0	0
Dominican Republic	Peralta	0.7	0.6	0.8	1	1	1	0.2	0.1	0.2	0	0	0
Dominican Republic	Piedra Blanca	0.7	0.6	0.9	2	2	3	0.2	0.2	0.2	1	0	1
Dominican Republic	Pimentel	0.7	0.6	0.8	2	2	2	0.2	0.2	0.2	0	0	0
Dominican Republic	Polo	0.9	0.8	1.1	1	1	2	0.2	0.2	0.3	0	0	0
Dominican Republic	Postrer Rio	1.0	0.9	1.1	1	1	1	0.3	0.2	0.3	0	0	0
Dominican Republic	Pueblo Viejo	0.8	0.7	0.9	2	2	3	0.2	0.2	0.2	0	0	0
Dominican Republic	Puñal	0.7	0.6	0.8	1	1	1	0.2	0.1	0.2	0	0	0

Table 3: Diarrhoea Mortality by Unit *(continued)*

Country	Unit	2000 mortality mean rate (per 10,000)	lower rate (per 10,000)	upper rate (per 10,000)	mean count	lower count	upper count	2017 mortality mean rate (per 10,000)	lower rate (per 10,000)	upper rate (per 10,000)	mean count	lower count	upper count
Dominican Republic	Quisquella	0.6	0.5	0.7	2	1	2	0.2	0.1	0.2	0	0	0
Dominican Republic	Ramón Santana	0.7	0.6	0.8	1	1	2	0.2	0.1	0.2	0	0	0
Dominican Republic	Rancho Arriba	0.7	0.6	0.8	1	1	1	0.2	0.1	0.2	0	0	0
Dominican Republic	Restauración	0.8	0.8	0.9	1	1	2	0.2	0.2	0.2	0	0	0
Dominican Republic	Rio San Juan	0.8	0.6	0.9	1	1	2	0.2	0.2	0.2	0	0	0
Dominican Republic	Sabana de la Mar	0.7	0.6	0.8	1	1	2	0.2	0.1	0.2	0	0	0
Dominican Republic	Sabana Grande de Boyá	0.8	0.7	0.9	3	3	4	0.2	0.2	0.3	1	1	1
Dominican Republic	Sabana Grande de Palenque	0.7	0.6	0.8	1	1	2	0.2	0.1	0.2	0	0	0
Dominican Republic	Sabana Iglesia	0.7	0.6	0.8	1	1	1	0.2	0.1	0.2	0	0	0
Dominican Republic	Sabana Larga	0.6	0.5	0.7	1	1	1	0.2	0.1	0.2	0	0	0
Dominican Republic	Sabana Yegua	0.8	0.7	1.0	2	2	2	0.2	0.2	0.2	0	0	0
Dominican Republic	Salcedo	0.7	0.6	0.8	3	3	4	0.2	0.1	0.2	1	0	1
Dominican Republic	Salvaleón de Higüey	0.6	0.5	0.7	11	9	13	0.2	0.1	0.2	6	5	8
Dominican Republic	San Cristóbal	0.7	0.6	0.8	24	21	28	0.2	0.1	0.2	5	4	6
Dominican Republic	San Felipe de Puerto Plata	0.7	0.6	0.8	12	10	13	0.2	0.2	0.2	2	2	3
Dominican Republic	San Fernando de Monte Cristi	0.8	0.7	0.9	3	3	4	0.2	0.2	0.2	0	0	1
Dominican Republic	San Francisco de Macorís	0.7	0.6	0.8	15	13	17	0.2	0.2	0.2	3	3	4
Dominican Republic	San Gregorio de Yaguate	0.7	0.6	0.8	4	4	5	0.2	0.1	0.2	1	1	1
Dominican Republic	San Ignacio de Sabaneta	0.8	0.7	0.9	3	3	4	0.2	0.2	0.2	0	0	1
Dominican Republic	San José de Las Matas	0.7	0.7	0.8	4	3	4	0.2	0.2	0.2	1	0	1
Dominican Republic	San José de Ocoa	0.6	0.6	0.7	3	3	4	0.2	0.1	0.2	1	1	1
Dominican Republic	San Juan de la Maguana	0.8	0.7	0.9	14	13	16	0.2	0.2	0.2	2	2	3
Dominican Republic	San Pedro de Macorís	0.6	0.5	0.7	12	10	14	0.2	0.1	0.2	4	3	5
Dominican Republic	San Rafael del Yuma	0.7	0.6	0.8	2	2	3	0.2	0.1	0.2	1	1	1
Dominican Republic	Sánchez	0.7	0.6	0.9	3	2	3	0.2	0.1	0.2	0	0	0
Dominican Republic	Santa Bárbara de Samaná	0.6	0.5	0.8	5	4	5	0.2	0.1	0.2	1	1	1
Dominican Republic	Santa Cruz de Barahona	0.8	0.7	1.0	8	7	9	0.2	0.2	0.2	1	1	2
Dominican Republic	Santa Cruz del Seybo	0.7	0.6	0.8	6	5	7	0.2	0.1	0.2	1	1	1
Dominican Republic	Santiago de los Caballeros	0.7	0.6	0.8	59	50	67	0.2	0.1	0.2	11	9	13
Dominican Republic	Santo Domingo Este	0.6	0.5	0.7	62	53	72	0.2	0.1	0.2	16	13	20

Table 3: Diarrhoea Mortality by Unit *(continued)*

Country	Unit	2000 mortality mean rate (per 10,000)	lower rate (per 10,000)	upper rate (per 10,000)	mean count	lower count	upper count	2017 mortality mean rate (per 10,000)	lower rate (per 10,000)	upper rate (per 10,000)	mean count	lower count	upper count
Dominican Republic	Santo Domingo Norte	0.7	0.6	0.8	32	28	38	0.2	0.1	0.2	13	10	16
Dominican Republic	Santo Domingo Oeste	0.6	0.5	0.7	30	26	34	0.2	0.1	0.2	8	7	10
Dominican Republic	Sosua	0.7	0.6	0.8	5	4	6	0.2	0.2	0.3	1	1	1
Dominican Republic	Tamayo	0.9	0.8	1.0	3	3	4	0.2	0.2	0.2	1	1	1
Dominican Republic	Tamboril	0.7	0.6	0.8	5	5	6	0.2	0.1	0.2	1	1	1
Dominican Republic	Tenares	0.7	0.6	0.8	2	2	3	0.2	0.1	0.2	0	0	1
Dominican Republic	Vallejuelo	0.9	0.8	1.0	2	1	2	0.2	0.2	0.2	0	0	0
Dominican Republic	Vicente Noble	0.9	0.8	1.0	3	3	4	0.2	0.2	0.2	0	0	1
Dominican Republic	Villa Altagracia	0.8	0.7	0.9	9	8	11	0.2	0.2	0.2	2	1	2
Dominican Republic	Villa Bisonó	0.7	0.6	0.8	4	4	5	0.2	0.2	0.2	1	1	1
Dominican Republic	Villa Gonzalez	0.7	0.6	0.7	3	3	4	0.2	0.1	0.2	1	1	1
Dominican Republic	Villa Hermosa	0.7	0.5	0.8	9	7	11	0.2	0.1	0.2	2	2	3
Dominican Republic	Villa Jaragua	1.0	0.9	1.1	2	2	2	0.2	0.2	0.3	0	0	0
Dominican Republic	Villa Rivas	0.7	0.6	0.9	3	2	3	0.2	0.2	0.2	1	1	1
Dominican Republic	Villa Tabara Arriba	0.8	0.7	0.9	1	1	2	0.2	0.2	0.2	0	0	0
Dominican Republic	Villa Tapia	0.7	0.6	0.8	2	2	2	0.2	0.1	0.2	0	0	0
Dominican Republic	Villa Vázquez	0.7	0.6	0.9	2	2	2	0.2	0.1	0.2	0	0	0
Dominican Republic	Yamasá	0.8	0.7	1.0	5	4	5	0.2	0.2	0.3	1	1	2
Ecuador	24 De Mayo	0.5	0.4	0.7	2	1	2	0.1	0.1	0.1	0	0	0
Ecuador	Aguarico	0.8	0.6	0.9	0	0	1	0.1	0.1	0.2	0	0	0
Ecuador	Alausí	0.6	0.5	0.7	4	3	4	0.1	0.1	0.1	1	0	1
Ecuador	Alfredo Baquerizo Moreno	0.5	0.4	0.6	2	1	2	0.1	0.1	0.1	0	0	0
Ecuador	Ambato	0.6	0.5	0.7	19	16	23	0.1	0.1	0.1	3	2	4
Ecuador	Antonio Ante	0.5	0.4	0.6	3	2	3	0.1	0.1	0.1	0	0	1
Ecuador	Arajuno	0.8	0.6	0.9	1	1	1	0.1	0.1	0.2	0	0	0
Ecuador	Archidona	0.7	0.6	0.8	2	2	2	0.1	0.1	0.1	1	0	1
Ecuador	Arenillas	0.4	0.4	0.5	1	1	2	0.1	0.1	0.1	0	0	0
Ecuador	Atacames	0.5	0.4	0.6	2	2	3	0.1	0.1	0.1	0	0	0
Ecuador	Atahualpa	0.5	0.4	0.6	0	0	0	0.1	0.1	0.1	0	0	0
Ecuador	Azogues	0.6	0.5	0.7	5	4	6	0.1	0.1	0.1	1	1	1
Ecuador	Baba	0.5	0.4	0.6	2	2	3	0.1	0.1	0.1	0	0	1
Ecuador	Babahoyo	0.5	0.4	0.6	10	8	12	0.1	0.1	0.1	2	1	2
Ecuador	Balao	0.5	0.4	0.6	1	1	2	0.1	0.1	0.1	0	0	0
Ecuador	Balsas	0.5	0.4	0.6	0	0	0	0.1	0.1	0.1	0	0	0
Ecuador	Balzar	0.5	0.4	0.6	3	3	4	0.1	0.1	0.1	1	1	1
Ecuador	Baños de Agua Santa	0.6	0.5	0.7	1	1	1	0.1	0.1	0.1	0	0	0
Ecuador	Biblián	0.5	0.4	0.6	1	1	1	0.1	0.1	0.1	0	0	0
Ecuador	Bolívar	0.5	0.4	0.6	1	1	1	0.1	0.1	0.1	0	0	0
Ecuador	Bolívar	0.5	0.4	0.6	2	2	3	0.1	0.1	0.1	0	0	0
Ecuador	Buena Fé	0.4	0.3	0.5	3	2	4	0.1	0.1	0.1	1	1	1

Table 3: Diarrhoea Mortality by Unit *(continued)*

		2000 mortality						2017 mortality					
Country	Unit	mean rate (per 10,000)	lower rate (per 10,000)	upper rate (per 10,000)	mean count	lower count	upper count	mean rate (per 10,000)	lower rate (per 10,000)	upper rate (per 10,000)	mean count	lower count	upper count
Ecuador	Caluma	0.5	0.4	0.7	1	1	1	0.1	0.1	0.1	0	0	0
Ecuador	Calvas	0.6	0.5	0.7	2	2	2	0.1	0.1	0.1	0	0	0
Ecuador	Camilo Ponce Enriquez	0.5	0.4	0.6	1	1	1	0.1	0.1	0.1	0	0	0
Ecuador	Cañar	0.5	0.4	0.6	4	3	5	0.1	0.1	0.1	1	1	1
Ecuador	Carlos Julio Arosemena Tola	0.7	0.6	0.9	0	0	0	0.1	0.1	0.2	0	0	0
Ecuador	Cascales	0.6	0.5	0.8	1	1	1	0.1	0.1	0.2	0	0	0
Ecuador	Catamayo	0.5	0.4	0.7	2	2	3	0.1	0.1	0.1	0	0	0
Ecuador	Cayambe	0.5	0.4	0.6	5	4	6	0.1	0.1	0.1	1	1	1
Ecuador	Celica	0.6	0.5	0.7	1	1	1	0.1	0.1	0.1	0	0	0
Ecuador	Centinela del Cóndor	0.6	0.5	0.8	1	0	1	0.1	0.1	0.1	0	0	0
Ecuador	Cevallos	0.6	0.5	0.7	0	0	1	0.1	0.1	0.1	0	0	0
Ecuador	Chaguarpamba	0.6	0.5	0.7	0	0	1	0.1	0.1	0.1	0	0	0
Ecuador	Chambo	0.6	0.5	0.7	1	1	1	0.1	0.1	0.1	0	0	0
Ecuador	Chilla	0.5	0.4	0.6	0	0	0	0.1	0.1	0.1	0	0	0
Ecuador	Chillanes	0.5	0.4	0.7	1	1	1	0.1	0.1	0.1	0	0	0
Ecuador	Chimbo	0.5	0.4	0.6	1	1	1	0.1	0.1	0.1	0	0	0
Ecuador	Chinchipe	0.7	0.5	0.8	1	1	1	0.1	0.1	0.1	0	0	0
Ecuador	Chone	0.5	0.4	0.6	7	6	9	0.1	0.1	0.1	1	1	2
Ecuador	Chordeleg	0.5	0.4	0.6	1	1	1	0.1	0.1	0.1	0	0	0
Ecuador	Chunchi	0.5	0.4	0.6	1	1	1	0.1	0.1	0.1	0	0	0
Ecuador	Colimes	0.5	0.4	0.6	2	1	2	0.1	0.1	0.1	0	0	0
Ecuador	Colta	0.6	0.5	0.7	2	2	3	0.1	0.1	0.1	0	0	0
Ecuador	Coronel Marcelino Maridueña	0.5	0.4	0.6	1	1	1	0.1	0.1	0.1	0	0	0
Ecuador	Cotacachi	0.6	0.4	0.7	4	3	4	0.1	0.1	0.1	1	0	1
Ecuador	Cuenca	0.5	0.4	0.6	25	20	30	0.1	0.1	0.1	5	4	5
Ecuador	Cumanda	0.6	0.5	0.7	1	1	1	0.1	0.1	0.1	0	0	0
Ecuador	Cuyabeno	0.7	0.5	0.8	1	0	1	0.1	0.1	0.2	0	0	0
Ecuador	Daule	0.5	0.4	0.6	9	8	12	0.1	0.1	0.1	2	2	2
Ecuador	Déleg	0.4	0.3	0.5	0	0	0	0.1	0.1	0.1	0	0	0
Ecuador	Durán	0.5	0.4	0.6	12	10	15	0.1	0.1	0.1	2	2	3
Ecuador	Echeandía	0.5	0.4	0.7	1	0	1	0.1	0.1	0.1	0	0	0
Ecuador	El Carmen	0.5	0.4	0.6	7	5	8	0.1	0.1	0.1	1	1	1
Ecuador	El Chaco	0.7	0.5	0.8	1	0	1	0.1	0.1	0.1	0	0	0
Ecuador	El Empalme	0.4	0.4	0.6	4	3	5	0.1	0.1	0.1	1	1	1
Ecuador	El Guabo	0.4	0.3	0.5	2	2	3	0.1	0.1	0.1	0	0	1
Ecuador	El Pan	0.4	0.4	0.5	0	0	0	0.1	0.1	0.1	0	0	0
Ecuador	El Pangui	0.7	0.5	0.8	1	1	1	0.1	0.1	0.1	0	0	0
Ecuador	El Tambo	0.4	0.4	0.5	1	0	1	0.1	0.1	0.1	0	0	0
Ecuador	El Triunfo	0.5	0.4	0.6	3	2	3	0.1	0.1	0.1	0	0	1
Ecuador	Eloy Alfaro	0.6	0.5	0.7	3	3	4	0.1	0.1	0.1	1	1	1
Ecuador	Esmeraldas	0.5	0.4	0.6	7	6	9	0.1	0.1	0.1	1	1	2
Ecuador	Espejo	0.5	0.4	0.6	1	1	1	0.1	0.1	0.1	0	0	0
Ecuador	Espíndola	0.6	0.5	0.8	1	1	1	0.1	0.1	0.1	0	0	0
Ecuador	Flavio Alfaro	0.5	0.4	0.6	2	1	2	0.1	0.1	0.1	0	0	0
Ecuador	General Antonio Elizalde	0.5	0.4	0.7	1	1	1	0.1	0.1	0.1	0	0	0
Ecuador	Girón	0.5	0.4	0.6	1	0	1	0.1	0.1	0.1	0	0	0
Ecuador	Gonzalo Pizarro	0.7	0.5	0.8	1	1	1	0.1	0.1	0.1	0	0	0
Ecuador	Gonzanamá	0.6	0.5	0.7	1	1	1	0.1	0.1	0.1	0	0	0
Ecuador	Guachapala	0.4	0.3	0.5	0	0	0	0.1	0.1	0.1	0	0	0
Ecuador	Gualaceo	0.5	0.4	0.6	2	2	3	0.1	0.1	0.1	0	0	1
Ecuador	Gualaquiza	0.6	0.5	0.7	1	1	2	0.1	0.1	0.1	0	0	0
Ecuador	Guamote	0.6	0.5	0.7	4	3	5	0.1	0.1	0.1	1	1	1
Ecuador	Guano	0.6	0.5	0.7	4	3	5	0.1	0.1	0.1	1	1	1
Ecuador	Guaranda	0.5	0.4	0.6	6	5	7	0.1	0.1	0.1	1	1	1

Table 3: Diarrhoea Mortality by Unit *(continued)*

Country	Unit	2000 mortality mean rate (per 10,000)	lower rate (per 10,000)	upper rate (per 10,000)	mean count	lower count	upper count	2017 mortality mean rate (per 10,000)	lower rate (per 10,000)	upper rate (per 10,000)	mean count	lower count	upper count
Ecuador	Guayaquil	0.5	0.4	0.6	111	89	136	0.1	0.1	0.1	20	17	24
Ecuador	Huamboya	0.8	0.6	0.9	1	1	1	0.1	0.1	0.2	0	0	0
Ecuador	Huaquillas	0.4	0.4	0.5	2	2	3	0.1	0.1	0.1	0	0	0
Ecuador	Ibarra	0.5	0.4	0.6	9	8	12	0.1	0.1	0.1	2	1	2
Ecuador	Isabela	0.2	0.2	0.3	0	0	0	0.1	0.0	0.1	0	0	0
Ecuador	Isidro Ayora	0.5	0.4	0.6	1	1	1	0.1	0.1	0.1	0	0	0
Ecuador	Jama	0.5	0.4	0.6	1	1	2	0.1	0.1	0.1	0	0	0
Ecuador	Jaramijó	0.5	0.4	0.7	1	1	2	0.1	0.1	0.1	0	0	0
Ecuador	Jipijapa	0.5	0.4	0.7	4	3	5	0.1	0.1	0.1	1	1	1
Ecuador	Junín	0.5	0.4	0.6	1	1	1	0.1	0.1	0.1	0	0	0
Ecuador	La Concordia	0.5	0.4	0.6	2	2	3	0.1	0.1	0.1	0	0	1
Ecuador	La Joya de los Sachas	0.6	0.5	0.8	2	2	3	0.1	0.1	0.1	1	1	1
Ecuador	La Libertad	0.5	0.4	0.6	5	4	6	0.1	0.1	0.1	1	1	1
Ecuador	La Maná	0.6	0.4	0.7	3	2	3	0.1	0.1	0.1	0	0	1
Ecuador	La Troncal	0.5	0.4	0.7	4	3	5	0.1	0.1	0.1	1	0	1
Ecuador	Lago Agrio	0.6	0.5	0.7	6	5	7	0.1	0.1	0.1	1	1	2
Ecuador	Las Lajas	0.5	0.4	0.6	0	0	0	0.1	0.1	0.1	0	0	0
Ecuador	Las Naves	0.5	0.4	0.6	0	0	0	0.1	0.1	0.1	0	0	0
Ecuador	Latacunga	0.5	0.4	0.7	11	9	13	0.1	0.1	0.1	2	1	2
Ecuador	Limón Indanza	0.6	0.5	0.8	1	1	1	0.1	0.1	0.1	0	0	0
Ecuador	Logroño	0.7	0.6	0.9	1	1	1	0.1	0.1	0.2	0	0	0
Ecuador	Loja	0.6	0.5	0.7	13	11	16	0.1	0.1	0.1	2	2	3
Ecuador	Lomas de Sargentillo	0.5	0.4	0.6	1	1	1	0.1	0.1	0.1	0	0	0
Ecuador	Loreto	0.7	0.6	0.9	2	2	2	0.1	0.1	0.2	1	0	1
Ecuador	Macará	0.6	0.5	0.7	1	1	1	0.1	0.1	0.1	0	0	0
Ecuador	Machala	0.4	0.3	0.5	10	8	13	0.1	0.1	0.1	2	2	2
Ecuador	Manta	0.5	0.4	0.7	8	6	9	0.1	0.1	0.1	1	1	2
Ecuador	Marcabelí	0.5	0.4	0.6	0	0	0	0.1	0.1	0.1	0	0	0
Ecuador	Mejía	0.7	0.6	0.8	0	0	0	0.1	0.1	0.1	0	0	0
Ecuador	Mejía	0.5	0.4	0.6	5	4	6	0.1	0.1	0.1	1	1	1
Ecuador	Mera	0.7	0.6	0.9	1	0	1	0.1	0.1	0.2	0	0	0
Ecuador	Milagro	0.5	0.4	0.6	8	7	10	0.1	0.1	0.1	2	1	2
Ecuador	Mira	0.5	0.4	0.6	1	1	1	0.1	0.1	0.1	0	0	0
Ecuador	Mocache	0.4	0.4	0.5	2	2	3	0.1	0.1	0.1	0	0	1
Ecuador	Mocha	0.5	0.4	0.7	0	0	0	0.1	0.1	0.1	0	0	0
Ecuador	Montalvo	0.5	0.4	0.6	1	1	2	0.1	0.1	0.1	0	0	0
Ecuador	Montecristi	0.5	0.4	0.7	6	5	7	0.1	0.1	0.1	1	1	1
Ecuador	Montúfar	0.5	0.4	0.6	2	1	2	0.1	0.1	0.1	0	0	0
Ecuador	Morona	0.7	0.6	0.8	4	3	5	0.1	0.1	0.1	1	1	1
Ecuador	Muisne	0.5	0.4	0.6	2	1	2	0.1	0.1	0.1	0	0	0
Ecuador	Nabón	0.5	0.4	0.6	1	1	1	0.1	0.1	0.1	0	0	0
Ecuador	Nangaritza	0.7	0.6	0.9	1	0	1	0.1	0.1	0.2	0	0	0
Ecuador	Naranjal	0.5	0.4	0.6	4	4	5	0.1	0.1	0.1	1	1	1
Ecuador	Naranjito	0.5	0.4	0.6	2	2	2	0.1	0.1	0.1	0	0	0
Ecuador	Nobol	0.5	0.4	0.6	1	1	2	0.1	0.1	0.1	0	0	0
Ecuador	Olmedo	0.5	0.4	0.6	1	1	1	0.1	0.1	0.1	0	0	0
Ecuador	Olmedo	0.6	0.5	0.7	0	0	0	0.1	0.1	0.1	0	0	0
Ecuador	Oña	0.5	0.4	0.6	0	0	0	0.1	0.1	0.1	0	0	0
Ecuador	Orellana	0.7	0.5	0.8	5	4	6	0.1	0.1	0.2	2	1	2
Ecuador	Otavalo	0.5	0.4	0.6	6	5	7	0.1	0.1	0.1	1	1	1
Ecuador	Pablo Sexto	0.8	0.6	0.9	0	0	0	0.1	0.1	0.2	0	0	0
Ecuador	Paján	0.5	0.4	0.7	2	2	3	0.1	0.1	0.1	0	0	1
Ecuador	Palanda	0.7	0.6	0.8	1	1	1	0.1	0.1	0.2	0	0	0
Ecuador	Palenque	0.5	0.4	0.6	1	1	2	0.1	0.1	0.1	0	0	0
Ecuador	Palestina	0.5	0.4	0.6	1	1	1	0.1	0.1	0.1	0	0	0
Ecuador	Pallatanga	0.5	0.4	0.7	1	1	1	0.1	0.1	0.1	0	0	0
Ecuador	Palora	0.7	0.6	0.9	1	1	1	0.1	0.1	0.2	0	0	0
Ecuador	Paltas	0.6	0.5	0.7	2	1	2	0.1	0.1	0.1	0	0	0

Table 3: Diarrhoea Mortality by Unit *(continued)*

Country	Unit	2000 mortality mean rate (per 10,000)	lower rate (per 10,000)	upper rate (per 10,000)	mean count	lower count	upper count	2017 mortality mean rate (per 10,000)	lower rate (per 10,000)	upper rate (per 10,000)	mean count	lower count	upper count
Ecuador	Pangua	0.6	0.5	0.7	2	1	2	0.1	0.1	0.1	0	0	0
Ecuador	Paquisha	0.7	0.6	0.8	0	0	0	0.1	0.1	0.1	0	0	0
Ecuador	Pasaje	0.4	0.3	0.5	3	3	4	0.1	0.1	0.1	1	0	1
Ecuador	Pastaza	0.7	0.6	0.9	5	4	6	0.1	0.1	0.2	1	1	1
Ecuador	Patate	0.5	0.4	0.6	1	1	1	0.1	0.1	0.1	0	0	0
Ecuador	Paute	0.5	0.4	0.6	2	1	2	0.1	0.1	0.1	0	0	0
Ecuador	Pedernales	0.5	0.4	0.6	4	3	5	0.1	0.1	0.1	1	1	1
Ecuador	Pedro Carbo	0.5	0.4	0.7	3	2	4	0.1	0.1	0.1	1	0	1
Ecuador	Pedro Moncayo	0.5	0.4	0.7	2	2	3	0.1	0.1	0.1	0	0	1
Ecuador	Pedro Vicente Maldonado	0.6	0.5	0.7	1	1	1	0.1	0.1	0.1	0	0	0
Ecuador	Penipe	0.6	0.5	0.7	0	0	1	0.1	0.1	0.1	0	0	0
Ecuador	Pichincha	0.5	0.4	0.6	2	2	3	0.1	0.1	0.1	0	0	0
Ecuador	Pimampiro	0.6	0.5	0.7	1	1	1	0.1	0.1	0.1	0	0	0
Ecuador	Piñas	0.5	0.4	0.6	1	1	2	0.1	0.1	0.1	0	0	0
Ecuador	Pindal	0.6	0.5	0.7	1	0	1	0.1	0.1	0.1	0	0	0
Ecuador	Playas	0.5	0.4	0.6	2	2	3	0.1	0.1	0.1	0	0	1
Ecuador	Portovelo	0.5	0.4	0.6	1	0	1	0.1	0.1	0.1	0	0	0
Ecuador	Portoviejo	0.5	0.4	0.6	14	11	18	0.1	0.1	0.1	2	2	3
Ecuador	Pucará	0.5	0.4	0.6	1	1	1	0.1	0.1	0.1	0	0	0
Ecuador	Pueblo Viejo	0.5	0.4	0.6	2	2	3	0.1	0.1	0.1	0	0	1
Ecuador	Puerto López	0.5	0.4	0.7	2	1	2	0.1	0.1	0.1	0	0	0
Ecuador	Puerto Quito	0.5	0.4	0.6	1	1	2	0.1	0.1	0.1	0	0	0
Ecuador	Pujilí	0.6	0.5	0.7	5	4	6	0.1	0.1	0.1	1	1	1
Ecuador	Putumayo	0.7	0.6	0.9	1	1	1	0.1	0.1	0.2	0	0	0
Ecuador	Puyango	0.6	0.5	0.7	1	1	1	0.1	0.1	0.1	0	0	0
Ecuador	Quero	0.5	0.4	0.7	1	1	1	0.1	0.1	0.1	0	0	0
Ecuador	Quevedo	0.4	0.3	0.5	7	6	9	0.1	0.1	0.1	2	1	2
Ecuador	Quijos	0.7	0.5	0.8	0	0	0	0.1	0.1	0.1	0	0	0
Ecuador	Quilanga	0.5	0.4	0.7	0	0	0	0.1	0.1	0.1	0	0	0
Ecuador	Quinindé	0.5	0.4	0.6	8	6	9	0.1	0.1	0.1	2	1	2
Ecuador	Quinsaloma	0.5	0.4	0.6	1	1	1	0.1	0.1	0.1	0	0	0
Ecuador	Quito	0.5	0.4	0.6	114	90	137	0.1	0.1	0.1	20	16	24
Ecuador	Río Verde	0.5	0.4	0.7	2	2	2	0.1	0.1	0.1	0	0	1
Ecuador	Riobamba	0.6	0.5	0.7	13	10	15	0.1	0.1	0.1	2	2	2
Ecuador	Rocafuerte	0.5	0.4	0.6	2	2	3	0.1	0.1	0.1	0	0	0
Ecuador	Rumiñahui	0.5	0.4	0.6	4	3	4	0.1	0.1	0.1	1	1	1
Ecuador	Salcedo	0.6	0.5	0.7	3	3	4	0.1	0.1	0.1	1	0	1
Ecuador	Salinas	0.5	0.4	0.6	3	3	4	0.1	0.1	0.1	1	1	1
Ecuador	Samborondón	0.5	0.4	0.6	5	4	7	0.1	0.1	0.1	1	1	1
Ecuador	San Cristóbal	0.3	0.2	0.4	0	0	0	0.1	0.0	0.1	0	0	0
Ecuador	San Fernando	0.4	0.4	0.5	0	0	0	0.1	0.1	0.1	0	0	0
Ecuador	San Jacinto de Yaguachi	0.5	0.4	0.6	5	4	6	0.1	0.1	0.1	1	1	1
Ecuador	San Juan Bosco	0.6	0.5	0.7	0	0	0	0.1	0.1	0.1	0	0	0
Ecuador	San Lorenzo	0.6	0.5	0.8	4	3	5	0.1	0.1	0.1	1	1	1
Ecuador	San Miguel	0.5	0.4	0.6	2	1	2	0.1	0.1	0.1	0	0	0
Ecuador	San Miguel de los Bancos	0.6	0.5	0.7	1	1	1	0.1	0.1	0.1	0	0	0
Ecuador	San Miguel de Urcuquí	0.5	0.4	0.7	1	1	1	0.1	0.1	0.1	0	0	0
Ecuador	San Pedro de Huaca	0.5	0.4	0.6	0	0	1	0.1	0.1	0.1	0	0	0
Ecuador	San Pedro de Pelileo	0.5	0.4	0.7	3	3	4	0.1	0.1	0.1	1	0	1
Ecuador	San Vicente	0.5	0.4	0.6	1	1	2	0.1	0.1	0.1	0	0	0
Ecuador	Santa Ana	0.5	0.4	0.6	3	2	4	0.1	0.1	0.1	1	0	1
Ecuador	Santa Clara	0.7	0.6	0.8	0	0	0	0.1	0.1	0.1	0	0	0
Ecuador	Santa Cruz	0.3	0.2	0.4	0	0	0	0.1	0.0	0.1	0	0	0
Ecuador	Santa Elena	0.5	0.4	0.6	9	8	12	0.1	0.1	0.1	2	2	2

Table 3: Diarrhoea Mortality by Unit *(continued)*

Country	Unit	2000 mortality mean rate (per 10,000)	2000 lower rate (per 10,000)	2000 upper rate (per 10,000)	2000 mean count	2000 lower count	2000 upper count	2017 mortality mean rate (per 10,000)	2017 lower rate (per 10,000)	2017 upper rate (per 10,000)	2017 mean count	2017 lower count	2017 upper count
Ecuador	Santa Isabel	0.5	0.4	0.6	1	1	1	0.1	0.1	0.1	0	0	0
Ecuador	Santa Lucia	0.5	0.4	0.6	2	2	3	0.1	0.1	0.1	0	0	1
Ecuador	Santa Rosa	0.4	0.3	0.5	3	2	4	0.1	0.1	0.1	0	0	1
Ecuador	Santiago	0.6	0.5	0.8	1	1	1	0.1	0.1	0.1	0	0	0
Ecuador	Santiago de Pillaro	0.5	0.4	0.7	2	2	3	0.1	0.1	0.1	0	0	0
Ecuador	Santo Domingo	0.5	0.4	0.6	22	17	26	0.1	0.1	0.1	5	4	6
Ecuador	Saquisili	0.5	0.4	0.6	0	0	0	0.1	0.1	0.1	0	0	0
Ecuador	Saquisilí	0.5	0.4	0.6	1	1	1	0.1	0.1	0.1	0	0	0
Ecuador	Saraguro	0.5	0.4	0.6	2	2	3	0.1	0.1	0.1	0	0	0
Ecuador	Sevilla de Oro	0.5	0.4	0.6	0	0	0	0.1	0.1	0.1	0	0	0
Ecuador	Shushufindi	0.6	0.5	0.8	3	2	4	0.1	0.1	0.1	1	1	1
Ecuador	Sigchos	0.6	0.5	0.7	2	1	2	0.1	0.1	0.1	0	0	0
Ecuador	Sigsig	0.4	0.3	0.5	1	1	2	0.1	0.1	0.1	0	0	0
Ecuador	Simon Bolivar	0.5	0.4	0.6	2	1	2	0.1	0.1	0.1	0	0	0
Ecuador	Sozoranga	0.6	0.5	0.7	1	0	1	0.1	0.1	0.1	0	0	0
Ecuador	Sucre	0.5	0.4	0.6	3	3	4	0.1	0.1	0.1	1	0	1
Ecuador	Sucúa	0.7	0.5	0.8	2	1	2	0.1	0.1	0.1	0	0	0
Ecuador	Sucumbíos	0.6	0.5	0.8	0	0	0	0.1	0.1	0.1	0	0	0
Ecuador	Suscal	0.5	0.4	0.6	0	0	0	0.1	0.1	0.1	0	0	0
Ecuador	Taisha	0.8	0.7	1.0	3	2	4	0.1	0.1	0.2	1	0	1
Ecuador	Tena	0.7	0.6	0.9	5	4	5	0.1	0.1	0.2	1	1	2
Ecuador	Tisaleo	0.5	0.4	0.7	1	1	1	0.1	0.1	0.1	0	0	0
Ecuador	Tiwintza	0.7	0.6	0.9	1	1	1	0.1	0.1	0.2	0	0	0
Ecuador	Tosagua	0.5	0.4	0.6	2	2	3	0.1	0.1	0.1	0	0	0
Ecuador	Tulcán	0.5	0.4	0.7	5	4	6	0.1	0.1	0.1	1	1	1
Ecuador	Urbina Jado	0.5	0.4	0.6	3	3	4	0.1	0.1	0.1	1	0	1
Ecuador	Urdaneta	0.5	0.4	0.6	2	1	2	0.1	0.1	0.1	0	0	0
Ecuador	Valencia	0.4	0.4	0.5	3	2	3	0.1	0.1	0.1	1	0	1
Ecuador	Ventanas	0.5	0.4	0.6	4	3	5	0.1	0.1	0.1	1	1	1
Ecuador	Vinces	0.5	0.4	0.6	4	3	5	0.1	0.1	0.1	1	1	1
Ecuador	Yacuambi	0.6	0.5	0.8	1	0	1	0.1	0.1	0.1	0	0	0
Ecuador	Yantzaza	0.6	0.5	0.8	2	1	2	0.1	0.1	0.1	0	0	0
Ecuador	Zamora	0.6	0.5	0.8	2	2	3	0.1	0.1	0.1	0	0	0
Ecuador	Zapotillo	0.6	0.5	0.7	1	1	1	0.1	0.1	0.1	0	0	0
Ecuador	Zaruma	0.5	0.4	0.6	1	1	2	0.1	0.1	0.1	0	0	0
El Salvador	Acajutla	0.7	0.6	1.0	5	4	6	0.1	0.1	0.1	0	0	1
El Salvador	Agua Caliente	0.7	0.5	0.9	1	1	1	0.1	0.1	0.1	0	0	0
El Salvador	Aguilares	0.7	0.5	0.9	1	1	1	0.1	0.1	0.1	0	0	0
El Salvador	Ahuachapán	0.8	0.6	1.0	13	9	16	0.1	0.1	0.2	2	1	2
El Salvador	Alegría	0.7	0.5	0.9	2	1	2	0.1	0.1	0.1	0	0	0
El Salvador	Anamorós	0.7	0.6	0.9	1	1	2	0.1	0.1	0.1	0	0	0
El Salvador	Antiguo Cuscatlán	0.7	0.5	0.9	6	4	7	0.1	0.1	0.1	0	0	1
El Salvador	Apaneca	0.8	0.6	1.0	1	1	1	0.1	0.1	0.2	0	0	0
El Salvador	Apastepeque	0.7	0.6	0.9	2	2	3	0.1	0.1	0.1	0	0	0
El Salvador	Apopa	0.7	0.5	0.9	12	9	16	0.1	0.1	0.1	2	1	2
El Salvador	Arambala	0.8	0.6	1.0	0	0	0	0.1	0.1	0.2	0	0	0
El Salvador	Arcatao	0.8	0.6	1.0	0	0	0	0.1	0.1	0.2	0	0	0
El Salvador	Armenia	0.8	0.6	1.0	3	2	4	0.1	0.1	0.2	0	0	1
El Salvador	Atiquizaya	0.8	0.6	1.0	3	3	4	0.1	0.1	0.2	0	0	1
El Salvador	Ayutuxtepeque	0.7	0.5	0.9	4	3	5	0.1	0.1	0.1	0	0	0
El Salvador	Azacualpa	0.7	0.6	1.0	0	0	0	0.1	0.1	0.1	0	0	0
El Salvador	Berlín	0.7	0.5	0.9	2	1	2	0.1	0.1	0.1	0	0	0
El Salvador	Bolívar	0.7	0.6	0.9	1	0	1	0.1	0.1	0.1	0	0	0
El Salvador	Cacaopera	0.8	0.6	1.0	2	1	2	0.1	0.1	0.2	0	0	0
El Salvador	California	0.8	0.6	0.9	0	0	0	0.1	0.1	0.2	0	0	0
El Salvador	Caluco	0.8	0.6	1.0	1	1	1	0.1	0.1	0.2	0	0	0
El Salvador	Candelaria	0.7	0.6	1.0	1	1	1	0.1	0.1	0.2	0	0	0
El Salvador	Candelaria de la Frontera	0.7	0.5	0.9	2	2	3	0.1	0.1	0.1	0	0	0

Table 3: Diarrhoea Mortality by Unit *(continued)*

Country	Unit	2000 mortality mean rate (per 10,000)	2000 mortality lower rate (per 10,000)	2000 mortality upper rate (per 10,000)	2000 mortality mean count	2000 mortality lower count	2000 mortality upper count	2017 mortality mean rate (per 10,000)	2017 mortality lower rate (per 10,000)	2017 mortality upper rate (per 10,000)	2017 mortality mean count	2017 mortality lower count	2017 mortality upper count
El Salvador	Carolina	0.8	0.6	1.0	1	1	1	0.1	0.1	0.2	0	0	0
El Salvador	Chalatenango	0.7	0.6	0.9	2	2	3	0.1	0.1	0.1	0	0	0
El Salvador	Chalchuapa	0.7	0.5	1.0	7	5	9	0.1	0.1	0.1	1	1	1
El Salvador	Chapeltique	0.8	0.6	1.0	1	1	2	0.1	0.1	0.2	0	0	0
El Salvador	Chilanga	0.8	0.6	1.0	1	1	1	0.1	0.1	0.2	0	0	0
El Salvador	Chiltiupán	0.7	0.5	1.0	1	1	1	0.1	0.1	0.2	0	0	0
El Salvador	Chinameca	0.7	0.6	0.9	2	1	2	0.1	0.1	0.2	0	0	0
El Salvador	Chirilagua	0.7	0.6	0.9	2	2	3	0.1	0.1	0.1	0	0	0
El Salvador	Cinquera	0.8	0.6	1.0	0	0	0	0.1	0.1	0.2	0	0	0
El Salvador	Citalá	0.7	0.5	0.9	0	0	0	0.1	0.1	0.1	0	0	0
El Salvador	Ciudad Arce	0.7	0.5	0.9	5	4	7	0.1	0.1	0.1	1	1	1
El Salvador	Ciudad Barrios	0.7	0.6	0.9	3	2	3	0.1	0.1	0.2	0	0	0
El Salvador	Coatepeque	0.7	0.5	1.0	5	3	6	0.1	0.1	0.1	1	0	1
El Salvador	Cojutepeque	0.8	0.6	1.0	3	3	4	0.1	0.1	0.2	0	0	0
El Salvador	Colón	0.7	0.5	0.9	7	5	9	0.1	0.1	0.1	1	1	2
El Salvador	Comacarán	0.8	0.6	1.0	0	0	1	0.1	0.1	0.2	0	0	0
El Salvador	Comalapa	0.8	0.6	1.0	0	0	0	0.1	0.1	0.2	0	0	0
El Salvador	Comasagua	0.7	0.5	0.9	2	1	2	0.1	0.1	0.1	0	0	0
El Salvador	Concepción Batres	0.7	0.6	0.9	1	1	2	0.1	0.1	0.1	0	0	0
El Salvador	Concepción de Ataco	0.8	0.6	1.0	2	1	2	0.1	0.1	0.2	0	0	0
El Salvador	Concepción de Oriente	0.7	0.6	0.9	1	0	1	0.1	0.1	0.1	0	0	0
El Salvador	Concepción Quezaltepeque	0.7	0.6	1.0	1	0	1	0.1	0.1	0.2	0	0	0
El Salvador	Conchagua	0.7	0.5	0.9	5	4	6	0.1	0.1	0.1	0	0	1
El Salvador	Corinto	0.8	0.6	1.0	1	1	1	0.1	0.1	0.2	0	0	0
El Salvador	Cuisnahuat	0.8	0.6	1.0	1	1	1	0.1	0.1	0.2	0	0	0
El Salvador	Cuscatancingo	0.7	0.5	0.9	3	3	5	0.1	0.1	0.1	0	0	0
El Salvador	Cuyultitán	0.7	0.6	1.0	1	0	1	0.1	0.1	0.2	0	0	0
El Salvador	Delgado	0.7	0.5	0.9	10	7	13	0.1	0.1	0.1	1	1	2
El Salvador	Delicias de Concepción	0.8	0.6	1.0	1	1	1	0.1	0.1	0.2	0	0	0
El Salvador	Dolores	0.7	0.6	1.0	1	1	1	0.1	0.1	0.2	0	0	0
El Salvador	Dulce Nombre de María	0.8	0.6	1.0	0	0	0	0.1	0.1	0.2	0	0	0
El Salvador	El Carmen	0.8	0.6	1.0	2	1	2	0.1	0.1	0.2	0	0	0
El Salvador	El Carmen	0.7	0.6	0.9	1	1	2	0.1	0.1	0.1	0	0	0
El Salvador	El Carrizal	0.8	0.6	1.0	0	0	0	0.1	0.1	0.2	0	0	0
El Salvador	El Congo	0.7	0.5	1.0	2	2	3	0.1	0.1	0.1	0	0	0
El Salvador	El Divisadero	0.8	0.6	1.0	1	1	1	0.1	0.1	0.2	0	0	0
El Salvador	El Paisnal	0.7	0.5	0.9	2	1	2	0.1	0.1	0.1	0	0	0
El Salvador	El Paraíso	0.7	0.6	1.0	1	1	1	0.1	0.1	0.1	0	0	0
El Salvador	El Porvenir	0.7	0.5	1.0	1	1	2	0.1	0.1	0.1	0	0	0
El Salvador	El Refugio	0.7	0.5	0.9	1	1	2	0.1	0.1	0.1	0	0	0
El Salvador	El Rosario	0.7	0.5	0.9	1	1	2	0.1	0.1	0.1	0	0	0
El Salvador	El Rosario	0.8	0.6	1.0	0	0	1	0.1	0.1	0.2	0	0	0
El Salvador	El Rosario	0.8	0.6	1.0	0	0	0	0.1	0.1	0.2	0	0	0
El Salvador	El Sauce	0.7	0.5	0.9	1	1	1	0.1	0.1	0.1	0	0	0
El Salvador	El Tránsito	0.7	0.6	0.9	2	1	2	0.1	0.1	0.1	0	0	0
El Salvador	El Triunfo	0.8	0.6	0.9	1	0	1	0.1	0.1	0.2	0	0	0
El Salvador	Embalse Cerron Grande	0.7	0.6	1.0	1	1	2	0.1	0.1	0.1	0	0	0
El Salvador	Ereguayquín	0.7	0.6	0.9	1	1	1	0.1	0.1	0.1	0	0	0
El Salvador	Estanzuelas	0.7	0.6	0.9	1	1	1	0.1	0.1	0.1	0	0	0
El Salvador	Guacotecti	0.8	0.6	1.0	1	1	1	0.1	0.1	0.2	0	0	0
El Salvador	Guadalupe	0.7	0.5	0.9	0	0	1	0.1	0.1	0.2	0	0	0
El Salvador	Gualococti	0.8	0.6	1.0	1	1	1	0.1	0.1	0.2	0	0	0
El Salvador	Guatajiagua	0.8	0.6	1.0	1	1	2	0.1	0.1	0.2	0	0	0

Table 3: Diarrhoea Mortality by Unit *(continued)*

Country	Unit	2000 mortality mean rate (per 10,000)	lower rate (per 10,000)	upper rate (per 10,000)	mean count	lower count	upper count	2017 mortality mean rate (per 10,000)	lower rate (per 10,000)	upper rate (per 10,000)	mean count	lower count	upper count
El Salvador	Guaymango	0.8	0.6	1.0	2	2	3	0.1	0.1	0.2	0	0	0
El Salvador	Guazapa	0.7	0.5	0.9	2	1	2	0.1	0.1	0.1	0	0	0
El Salvador	Huizúcar	0.7	0.5	0.9	2	1	3	0.1	0.1	0.1	0	0	0
El Salvador	Ilobasco	0.8	0.6	1.0	7	5	8	0.1	0.1	0.2	1	1	1
El Salvador	Ilopango	0.6	0.5	0.8	6	4	7	0.1	0.1	0.1	1	0	1
El Salvador	Intipucá	0.7	0.6	0.9	1	1	1	0.1	0.1	0.1	0	0	0
El Salvador	Izalco	0.8	0.6	1.0	7	5	9	0.1	0.1	0.2	1	1	1
El Salvador	Jayaque	0.7	0.5	0.9	1	1	1	0.1	0.1	0.1	0	0	0
El Salvador	Jerusalén	0.7	0.6	1.0	0	0	0	0.1	0.1	0.2	0	0	0
El Salvador	Jicalapa	0.7	0.5	1.0	1	0	1	0.1	0.1	0.1	0	0	0
El Salvador	Jiquilisco	0.7	0.6	0.9	5	4	6	0.1	0.1	0.1	1	0	1
El Salvador	Joateca	0.8	0.7	1.1	1	0	1	0.1	0.1	0.2	0	0	0
El Salvador	Jocoaitique	0.8	0.6	1.0	0	0	0	0.1	0.1	0.2	0	0	0
El Salvador	Jocoro	0.8	0.6	1.0	1	1	2	0.1	0.1	0.2	0	0	0
El Salvador	Juayúa	0.7	0.5	1.0	3	2	4	0.1	0.1	0.2	0	0	0
El Salvador	Jucuapa	0.7	0.6	0.9	2	2	3	0.1	0.1	0.2	0	0	0
El Salvador	Jucuarán	0.7	0.6	0.9	1	1	2	0.1	0.1	0.1	0	0	0
El Salvador	Jujutla	0.8	0.6	1.0	3	3	5	0.1	0.1	0.2	0	0	0
El Salvador	Jutiapa	0.8	0.6	1.0	1	1	1	0.1	0.1	0.2	0	0	0
El Salvador	La Laguna	0.8	0.6	1.0	0	0	0	0.1	0.1	0.2	0	0	0
El Salvador	La Libertad	0.7	0.5	1.0	3	2	4	0.1	0.1	0.1	0	0	0
El Salvador	La Palma	0.7	0.5	0.9	1	1	1	0.1	0.1	0.1	0	0	0
El Salvador	La Reina	0.7	0.6	1.0	1	1	1	0.1	0.1	0.1	0	0	0
El Salvador	La Unión	0.7	0.5	0.9	2	2	3	0.1	0.1	0.1	0	0	0
El Salvador	Lago de Coatepeque	0.7	0.5	1.0	0	0	1	0.1	0.1	0.2	0	0	0
El Salvador	Lago de Guija	0.7	0.5	0.9	0	0	0	0.1	0.1	0.1	0	0	0
El Salvador	Lago de Llopango	0.7	0.5	0.9	1	1	1	0.1	0.1	0.1	0	0	0
El Salvador	Las Vueltas	0.7	0.6	0.9	0	0	0	0.1	0.1	0.1	0	0	0
El Salvador	Lislique	0.7	0.6	1.0	2	1	2	0.1	0.1	0.1	0	0	0
El Salvador	Lolotique	0.8	0.6	1.0	2	1	2	0.1	0.1	0.2	0	0	0
El Salvador	Lolotiquillo	0.8	0.6	1.0	1	1	1	0.1	0.1	0.2	0	0	0
El Salvador	Masahuat	0.7	0.5	0.9	0	0	1	0.1	0.1	0.1	0	0	0
El Salvador	Meanguera	0.8	0.6	1.0	1	1	1	0.1	0.1	0.2	0	0	0
El Salvador	Meanguera del Golfo	0.7	0.5	0.9	0	0	0	0.1	0.1	0.1	0	0	0
El Salvador	Mejicanos	0.6	0.5	0.8	9	7	12	0.1	0.1	0.1	1	1	1
El Salvador	Mercedes La Ceiba	0.7	0.6	0.9	0	0	0	0.1	0.1	0.2	0	0	0
El Salvador	Mercedes Umaña	0.7	0.6	0.9	1	1	2	0.1	0.1	0.2	0	0	0
El Salvador	Metapán	0.7	0.5	0.9	5	4	7	0.1	0.1	0.1	1	0	1
El Salvador	Moncagua	0.8	0.6	0.9	2	2	3	0.1	0.1	0.2	0	0	0
El Salvador	Monte San Juan	0.8	0.6	1.0	2	2	3	0.1	0.1	0.2	0	0	0
El Salvador	Nahuizalco	0.7	0.6	1.0	3	3	5	0.1	0.1	0.2	1	0	1
El Salvador	Nahulingo	0.8	0.6	1.0	2	1	2	0.1	0.1	0.2	0	0	0
El Salvador	Nejapa	0.7	0.5	0.9	6	4	8	0.1	0.1	0.1	1	1	1
El Salvador	Nombre de Jesús	0.8	0.6	1.0	0	0	0	0.1	0.1	0.2	0	0	0
El Salvador	Nueva Concepción	0.7	0.5	0.9	3	2	4	0.1	0.1	0.1	0	0	0
El Salvador	Nueva Esparta	0.7	0.6	0.9	1	1	1	0.1	0.1	0.1	0	0	0
El Salvador	Nueva Granada	0.7	0.6	0.9	1	1	1	0.1	0.1	0.1	0	0	0
El Salvador	Nueva Guadalupe	0.8	0.6	0.9	1	1	1	0.1	0.1	0.2	0	0	0
El Salvador	Nueva San Salvador	0.7	0.5	0.9	11	9	15	0.1	0.1	0.1	1	1	2
El Salvador	Nueva Trinidad	0.8	0.6	1.0	0	0	0	0.1	0.1	0.1	0	0	0
El Salvador	Nuevo Cuscatlán	0.7	0.5	0.9	1	1	1	0.1	0.1	0.1	0	0	0
El Salvador	Nuevo Edén de San Juan	0.7	0.5	0.9	1	1	1	0.1	0.1	0.1	0	0	0
El Salvador	Ojos de Agua	0.8	0.6	1.0	0	0	0	0.1	0.1	0.2	0	0	0
El Salvador	Olocuilta	0.7	0.5	0.9	2	2	3	0.1	0.1	0.1	0	0	0

Table 3: Diarrhoea Mortality by Unit *(continued)*

Country	Unit	2000 mortality mean rate (per 10,000)	lower rate (per 10,000)	upper rate (per 10,000)	mean count	lower count	upper count	2017 mortality mean rate (per 10,000)	lower rate (per 10,000)	upper rate (per 10,000)	mean count	lower count	upper count
El Salvador	Opico	0.7	0.5	0.9	7	5	9	0.1	0.1	0.1	1	1	1
El Salvador	Oratorio de Concepción	0.8	0.6	1.0	0	0	1	0.1	0.1	0.2	0	0	0
El Salvador	Osicala	0.8	0.6	1.0	1	1	1	0.1	0.1	0.2	0	0	0
El Salvador	Ozatlán	0.8	0.6	1.0	1	1	2	0.1	0.1	0.2	0	0	0
El Salvador	Panchimalco	0.7	0.5	0.9	4	3	5	0.1	0.1	0.1	0	0	1
El Salvador	Paraíso de Osorio	0.7	0.6	1.0	0	0	0	0.1	0.1	0.2	0	0	0
El Salvador	Pasaquina	0.7	0.5	0.9	2	2	3	0.1	0.1	0.1	0	0	0
El Salvador	Perquín	0.8	0.6	1.0	0	0	0	0.1	0.1	0.2	0	0	0
El Salvador	Polorós	0.7	0.6	0.9	1	1	1	0.1	0.1	0.1	0	0	0
El Salvador	Potonico	0.7	0.6	0.9	0	0	0	0.1	0.1	0.1	0	0	0
El Salvador	Puerto El Triunfo	0.7	0.6	0.9	1	1	1	0.1	0.1	0.1	0	0	0
El Salvador	Quelepa	0.7	0.6	0.9	1	1	1	0.1	0.1	0.1	0	0	0
El Salvador	Quezaltepeque	0.7	0.5	0.9	5	4	7	0.1	0.1	0.1	1	0	1
El Salvador	Rosario de Mora	0.7	0.5	0.9	1	1	1	0.1	0.1	0.1	0	0	0
El Salvador	Sacacoyo	0.7	0.5	1.0	1	1	2	0.1	0.1	0.1	0	0	0
El Salvador	Salcoatitán	0.8	0.6	1.0	0	0	1	0.1	0.1	0.2	0	0	0
El Salvador	San Agustín	0.7	0.6	0.9	1	0	1	0.1	0.1	0.1	0	0	0
El Salvador	San Alejo	0.7	0.6	0.9	2	2	3	0.1	0.1	0.1	0	0	0
El Salvador	San Antonio	0.8	0.6	1.0	1	0	1	0.1	0.1	0.2	0	0	0
El Salvador	San Antonio de la Cruz	0.7	0.6	0.9	0	0	0	0.1	0.1	0.1	0	0	0
El Salvador	San Antonio del Monte	0.7	0.5	1.0	4	3	6	0.1	0.1	0.2	1	0	1
El Salvador	San Antonio Los Ranchos	0.7	0.6	0.9	0	0	0	0.1	0.1	0.2	0	0	0
El Salvador	San Antonio Masahuat	0.8	0.6	1.0	0	0	1	0.1	0.1	0.2	0	0	0
El Salvador	San Antonio Pajonal	0.7	0.5	0.9	0	0	0	0.1	0.1	0.1	0	0	0
El Salvador	San Bartolomé Perulapía	0.7	0.5	0.9	1	1	1	0.1	0.1	0.1	0	0	0
El Salvador	San Buenaventura	0.8	0.6	1.0	1	1	1	0.1	0.1	0.2	0	0	0
El Salvador	San Carlos	0.8	0.6	1.0	1	0	1	0.1	0.1	0.2	0	0	0
El Salvador	San Cayetano Istepeque	0.7	0.6	0.9	1	1	1	0.1	0.1	0.1	0	0	0
El Salvador	San Cristóbal	0.7	0.6	0.9	1	1	1	0.1	0.1	0.1	0	0	0
El Salvador	San Dionisio	0.7	0.6	0.9	1	1	1	0.1	0.1	0.1	0	0	0
El Salvador	San Emigdio	0.7	0.6	1.0	0	0	0	0.1	0.1	0.2	0	0	0
El Salvador	San Esteban Catarina	0.7	0.6	0.9	1	1	1	0.1	0.1	0.2	0	0	0
El Salvador	San Fernando	0.8	0.7	1.1	0	0	0	0.1	0.1	0.2	0	0	0
El Salvador	San Fernando	0.8	0.6	1.0	0	0	0	0.1	0.1	0.1	0	0	0
El Salvador	San Francisco Chinameca	0.7	0.6	1.0	1	1	1	0.1	0.1	0.1	0	0	0
El Salvador	San Francisco Gotera	0.8	0.6	1.0	2	2	3	0.1	0.1	0.2	0	0	0
El Salvador	San Francisco Javier	0.7	0.6	0.9	1	0	1	0.1	0.1	0.1	0	0	0
El Salvador	San Francisco Lempa	0.7	0.6	1.0	0	0	0	0.1	0.1	0.1	0	0	0
El Salvador	San Francisco Menéndez	0.8	0.6	1.0	5	4	6	0.1	0.1	0.2	1	0	1
El Salvador	San Francisco Morazán	0.7	0.6	0.9	1	0	1	0.1	0.1	0.1	0	0	0
El Salvador	San Gerardo	0.7	0.6	0.9	1	0	1	0.1	0.1	0.1	0	0	0
El Salvador	San Ignacio	0.8	0.6	1.0	0	0	0	0.1	0.1	0.1	0	0	0
El Salvador	San Ildefonso	0.7	0.5	0.9	1	1	1	0.1	0.1	0.1	0	0	0
El Salvador	San Isidro	0.8	0.6	1.0	0	0	0	0.1	0.1	0.2	0	0	0

Table 3: Diarrhoea Mortality by Unit *(continued)*

Country	Unit	2000 mortality mean rate (per 10,000)	lower rate (per 10,000)	upper rate (per 10,000)	mean count	lower count	upper count	2017 mortality mean rate (per 10,000)	lower rate (per 10,000)	upper rate (per 10,000)	mean count	lower count	upper count
El Salvador	San Isidro	0.8	0.6	1.0	1	1	1	0.1	0.1	0.2	0	0	0
El Salvador	San Isidro Labrador	0.7	0.6	0.9	0	0	0	0.1	0.1	0.1	0	0	0
El Salvador	San Jorge	0.7	0.6	0.9	1	1	1	0.1	0.1	0.1	0	0	0
El Salvador	San José	0.7	0.5	0.9	0	0	1	0.1	0.1	0.1	0	0	0
El Salvador	San José Cancasque	0.7	0.6	0.9	0	0	0	0.1	0.1	0.1	0	0	0
El Salvador	San José Guayabal	0.7	0.6	1.0	1	1	1	0.1	0.1	0.2	0	0	0
El Salvador	San José Las Flores	0.7	0.6	0.9	0	0	0	0.1	0.1	0.1	0	0	0
El Salvador	San José Villanueva	0.7	0.5	0.9	1	1	2	0.1	0.1	0.1	0	0	0
El Salvador	San Juan Nonualco	0.7	0.6	0.9	2	1	2	0.1	0.1	0.1	0	0	0
El Salvador	San Juan Talpa	0.7	0.5	0.9	1	0	1	0.1	0.1	0.1	0	0	0
El Salvador	San Juan Tepezontes	0.8	0.6	1.0	0	0	1	0.1	0.1	0.2	0	0	0
El Salvador	San Julián	0.8	0.6	1.0	2	1	3	0.1	0.1	0.2	0	0	0
El Salvador	San Lorenzo	0.7	0.6	1.0	1	0	1	0.1	0.1	0.2	0	0	0
El Salvador	San Lorenzo	0.8	0.6	1.1	1	1	2	0.1	0.1	0.2	0	0	0
El Salvador	San Luis de la Reina	0.8	0.6	1.0	1	1	1	0.1	0.1	0.2	0	0	0
El Salvador	San Luis del Carmen	0.7	0.6	1.0	0	0	0	0.1	0.1	0.1	0	0	0
El Salvador	San Luis La Herradura	0.7	0.5	0.9	2	1	2	0.1	0.1	0.1	0	0	0
El Salvador	San Luis Talpa	0.7	0.5	0.9	2	2	3	0.1	0.1	0.1	0	0	0
El Salvador	San Marcos	0.7	0.5	0.9	3	2	4	0.1	0.1	0.1	0	0	0
El Salvador	San Martín	0.7	0.5	0.9	5	4	7	0.1	0.1	0.1	1	1	1
El Salvador	San Matías	0.7	0.5	0.9	1	1	1	0.1	0.1	0.1	0	0	0
El Salvador	San Miguel	0.7	0.6	0.9	22	17	28	0.1	0.1	0.1	2	2	3
El Salvador	San Miguel de Mercedes	0.7	0.5	0.9	0	0	0	0.1	0.1	0.1	0	0	0
El Salvador	San Miguel Tepezontes	0.7	0.6	1.0	0	0	1	0.1	0.1	0.2	0	0	0
El Salvador	San Pablo Tacachico	0.7	0.5	0.9	2	1	2	0.1	0.1	0.1	0	0	0
El Salvador	San Pedro Masahuat	0.7	0.5	0.9	2	2	3	0.1	0.1	0.1	0	0	0
El Salvador	San Pedro Nonualco	0.7	0.6	0.9	1	1	1	0.1	0.1	0.2	0	0	0
El Salvador	San Pedro Perulapán	0.8	0.6	1.0	4	3	5	0.1	0.1	0.2	1	0	1
El Salvador	San Pedro Puxtla	0.8	0.6	1.0	1	0	1	0.1	0.1	0.2	0	0	0
El Salvador	San Rafael	0.7	0.6	0.9	1	1	1	0.1	0.1	0.1	0	0	0
El Salvador	San Rafael	0.8	0.6	1.0	0	0	1	0.1	0.1	0.2	0	0	0
El Salvador	San Rafael Cedros	0.8	0.6	1.0	1	1	1	0.1	0.1	0.2	0	0	0
El Salvador	San Rafael Obrajuelo	0.7	0.6	0.9	1	1	1	0.1	0.1	0.1	0	0	0
El Salvador	San Ramón	0.7	0.6	0.9	1	1	1	0.1	0.1	0.1	0	0	0
El Salvador	San Salvador	0.7	0.5	0.9	28	21	36	0.1	0.1	0.1	2	2	3
El Salvador	San Sebastián	0.7	0.6	1.0	2	1	2	0.1	0.1	0.2	0	0	0
El Salvador	San Sebastián Salitrillo	0.7	0.5	0.9	2	1	2	0.1	0.1	0.1	0	0	0
El Salvador	San Simón	0.8	0.6	1.0	1	1	1	0.1	0.1	0.2	0	0	0
El Salvador	San Vicente	0.7	0.6	0.9	4	3	5	0.1	0.1	0.1	0	0	1
El Salvador	Santa Ana	0.7	0.5	0.9	22	16	28	0.1	0.1	0.1	2	2	3
El Salvador	Santa Catarina Masahuat	0.8	0.6	1.0	1	1	2	0.1	0.1	0.2	0	0	0

Table 3: Diarrhoea Mortality by Unit *(continued)*

Country	Unit	2000 mortality mean rate (per 10,000)	lower rate (per 10,000)	upper rate (per 10,000)	mean count	lower count	upper count	2017 mortality mean rate (per 10,000)	lower rate (per 10,000)	upper rate (per 10,000)	mean count	lower count	upper count
El Salvador	Santa Clara	0.7	0.6	0.9	1	0	1	0.1	0.1	0.1	0	0	0
El Salvador	Santa Cruz Analquito	0.7	0.6	1.0	0	0	0	0.1	0.1	0.2	0	0	0
El Salvador	Santa Cruz Michapa	0.8	0.6	1.0	2	1	2	0.1	0.1	0.2	0	0	0
El Salvador	Santa Elena	0.7	0.6	0.9	3	2	3	0.1	0.1	0.1	0	0	0
El Salvador	Santa Isabel Ishuatán	0.8	0.6	1.0	1	1	1	0.1	0.1	0.2	0	0	0
El Salvador	Santa María	0.7	0.6	0.9	1	1	1	0.1	0.1	0.1	0	0	0
El Salvador	Santa María Ostuma	0.7	0.6	0.9	1	0	1	0.1	0.1	0.2	0	0	0
El Salvador	Santa Rita	0.8	0.6	1.0	1	1	1	0.1	0.1	0.2	0	0	0
El Salvador	Santa Rosa de Lima	0.7	0.6	0.9	3	2	4	0.1	0.1	0.1	0	0	0
El Salvador	Santa Rosa Guachipilín	0.7	0.5	0.9	0	0	0	0.1	0.1	0.1	0	0	0
El Salvador	Santiago de la Frontera	0.7	0.5	0.9	1	0	1	0.1	0.1	0.1	0	0	0
El Salvador	Santiago de María	0.7	0.5	0.9	1	1	2	0.1	0.1	0.1	0	0	0
El Salvador	Santiago Nonualco	0.7	0.6	0.9	4	3	6	0.1	0.1	0.1	1	0	1
El Salvador	Santiago Texacuangos	0.7	0.5	0.9	1	1	2	0.1	0.1	0.1	0	0	0
El Salvador	Santo Domingo	0.7	0.6	0.9	1	1	1	0.1	0.1	0.2	0	0	0
El Salvador	Santo Domingo	0.8	0.6	1.0	1	1	1	0.1	0.1	0.2	0	0	0
El Salvador	Santo Tomás	0.7	0.5	0.9	2	2	3	0.1	0.1	0.1	0	0	0
El Salvador	Sensembra	0.8	0.6	1.0	0	0	1	0.1	0.1	0.2	0	0	0
El Salvador	Sensuntepeque	0.8	0.6	1.0	4	3	5	0.1	0.1	0.2	0	0	1
El Salvador	Sesori	0.7	0.6	0.9	1	1	2	0.1	0.1	0.1	0	0	0
El Salvador	Sociedad	0.8	0.6	1.0	2	1	2	0.1	0.1	0.2	0	0	0
El Salvador	Sonsonate	0.7	0.6	1.0	7	5	9	0.1	0.1	0.2	1	1	1
El Salvador	Sonzacate	0.7	0.5	1.0	2	1	2	0.1	0.1	0.2	0	0	0
El Salvador	Soyapango	0.6	0.5	0.8	14	11	19	0.1	0.1	0.1	1	1	2
El Salvador	Suchitoto	0.7	0.6	1.0	2	2	3	0.1	0.1	0.2	0	0	0
El Salvador	Tacuba	0.8	0.6	1.0	3	2	4	0.1	0.1	0.2	0	0	1
El Salvador	Talnique	0.7	0.5	0.9	1	1	1	0.1	0.1	0.1	0	0	0
El Salvador	Tamanique	0.7	0.5	1.0	1	1	2	0.1	0.1	0.1	0	0	0
El Salvador	Tapalhuaca	0.8	0.6	1.0	0	0	1	0.1	0.1	0.2	0	0	0
El Salvador	Tecapán	0.8	0.6	1.0	1	1	2	0.1	0.1	0.2	0	0	0
El Salvador	Tecoluca	0.7	0.6	0.9	2	2	3	0.1	0.1	0.1	0	0	0
El Salvador	Tejutepeque	0.8	0.6	1.0	1	1	1	0.1	0.1	0.2	0	0	0
El Salvador	Tejutla	0.8	0.6	1.0	1	1	2	0.1	0.1	0.1	0	0	0
El Salvador	Tenancingo	0.8	0.6	1.0	1	1	1	0.1	0.1	0.2	0	0	0
El Salvador	Teotepeque	0.7	0.5	1.0	1	1	2	0.1	0.1	0.1	0	0	0
El Salvador	Tepecoyo	0.7	0.5	1.0	1	1	2	0.1	0.1	0.2	0	0	0
El Salvador	Tepetitán	0.7	0.6	0.9	0	0	1	0.1	0.1	0.1	0	0	0
El Salvador	Texistepeque	0.7	0.5	0.9	2	1	2	0.1	0.1	0.1	0	0	0
El Salvador	Tonacatepeque	0.7	0.5	0.9	7	5	9	0.1	0.1	0.1	1	1	2
El Salvador	Torola	0.8	0.7	1.1	0	0	0	0.1	0.1	0.2	0	0	0
El Salvador	Turín	0.8	0.6	1.0	1	0	1	0.1	0.1	0.2	0	0	0
El Salvador	Uluazapa	0.8	0.6	0.9	0	0	1	0.1	0.1	0.1	0	0	0
El Salvador	Usulután	0.7	0.6	0.9	6	5	8	0.1	0.1	0.1	1	0	1
El Salvador	Verapaz	0.7	0.6	0.9	1	1	1	0.1	0.1	0.2	0	0	0
El Salvador	Victoria	0.8	0.6	1.0	2	1	2	0.1	0.1	0.2	0	0	0
El Salvador	Yamabal	0.8	0.6	1.0	1	1	1	0.1	0.1	0.2	0	0	0
El Salvador	Yayantique	0.7	0.6	0.9	1	1	1	0.1	0.1	0.1	0	0	0
El Salvador	Yoloaiquín	0.8	0.6	1.0	0	0	0	0.1	0.1	0.2	0	0	0
El Salvador	Yucuaiquín	0.8	0.6	1.0	1	0	1	0.1	0.1	0.2	0	0	0
El Salvador	Zacatecoluca	0.7	0.6	0.9	5	4	7	0.1	0.1	0.1	1	0	1

Table 3: Diarrhoea Mortality by Unit *(continued)*

Country	Unit	2000 mortality mean rate (per 10,000)	2000 mortality lower rate (per 10,000)	2000 mortality upper rate (per 10,000)	2000 mortality mean count	2000 mortality lower count	2000 mortality upper count	2017 mortality mean rate (per 10,000)	2017 mortality lower rate (per 10,000)	2017 mortality upper rate (per 10,000)	2017 mortality mean count	2017 mortality lower count	2017 mortality upper count
El Salvador	Zaragoza	0.7	0.5	1.0	1	1	1	0.1	0.1	0.1	0	0	0
Guatemala	Acatenango	1.9	1.3	2.5	6	4	8	0.7	0.5	0.8	2	2	3
Guatemala	Agua Blanca	1.5	1.1	2.1	3	3	5	0.6	0.5	0.7	1	1	1
Guatemala	Aguacatán	1.8	1.3	2.4	12	9	16	0.6	0.5	0.8	4	3	5
Guatemala	Almolonga	1.4	1.1	1.9	3	2	4	0.6	0.5	0.7	1	1	2
Guatemala	Alotenango	1.8	1.3	2.4	5	4	7	0.6	0.5	0.8	3	2	3
Guatemala	Amatitlán	1.4	1.1	1.9	25	18	34	0.6	0.5	0.7	11	9	14
Guatemala	Antigua Guatemala	1.4	1.1	2.0	12	9	16	0.6	0.5	0.7	4	3	5
Guatemala	Asunción Mita	1.5	1.1	2.1	9	7	13	0.6	0.5	0.7	3	2	4
Guatemala	Atescatempa	1.6	1.2	2.2	3	2	4	0.6	0.5	0.8	1	1	1
Guatemala	Ayutla	1.7	1.3	2.3	8	6	11	0.7	0.6	0.9	4	3	5
Guatemala	Barberena	1.7	1.2	2.2	9	7	12	0.6	0.5	0.8	4	3	5
Guatemala	Cabañas	1.7	1.2	2.2	3	2	4	0.6	0.5	0.8	1	1	1
Guatemala	Cabricán	1.8	1.3	2.4	5	3	6	0.6	0.5	0.8	2	1	2
Guatemala	Cajolá	1.6	1.2	2.1	1	1	2	0.6	0.5	0.8	0	0	1
Guatemala	Camotán	1.9	1.4	2.5	10	7	13	0.7	0.6	0.9	5	4	6
Guatemala	Canillá	2.0	1.5	2.7	3	2	4	0.7	0.6	0.9	1	1	2
Guatemala	Cantel	1.5	1.1	2.0	8	6	10	0.6	0.5	0.7	4	3	5
Guatemala	Casillas	1.7	1.2	2.3	5	3	6	0.6	0.5	0.8	2	2	2
Guatemala	Catarina	2.0	1.5	2.7	9	7	12	0.8	0.6	1.0	5	4	6
Guatemala	Chahal	2.1	1.6	2.8	6	4	8	0.7	0.6	0.9	3	2	4
Guatemala	Chajul	1.9	1.4	2.5	10	8	14	0.6	0.5	0.8	5	4	7
Guatemala	Champerico	1.7	1.3	2.3	7	5	10	0.7	0.6	0.9	4	3	4
Guatemala	Chiantla	1.6	1.2	2.1	17	13	23	0.6	0.4	0.7	8	6	10
Guatemala	Chicacao	2.0	1.5	2.7	15	11	20	0.8	0.6	1.0	6	4	7
Guatemala	Chicaman	2.0	1.5	2.7	11	8	15	0.7	0.6	0.9	5	4	6
Guatemala	Chiché	1.9	1.4	2.5	7	5	10	0.6	0.5	0.8	3	3	4
Guatemala	Chichicastenango	1.7	1.3	2.3	30	22	40	0.6	0.5	0.8	12	10	16
Guatemala	Chimaltenango	1.6	1.1	2.1	11	8	15	0.6	0.5	0.8	6	5	8
Guatemala	Chinautla	1.4	1.0	1.9	13	10	18	0.6	0.5	0.7	5	4	6
Guatemala	Chinique	1.9	1.4	2.5	2	2	3	0.6	0.5	0.8	1	1	1
Guatemala	Chiquimula	1.5	1.1	2.0	18	13	24	0.6	0.5	0.7	7	6	9
Guatemala	Chiquimulilla	1.7	1.2	2.2	10	8	14	0.7	0.5	0.8	4	3	5
Guatemala	Chisec	1.9	1.4	2.6	24	18	33	0.7	0.5	0.9	11	8	13
Guatemala	Chuarrancho	1.7	1.3	2.4	2	2	3	0.6	0.5	0.8	1	1	1
Guatemala	Ciudad Vieja	1.6	1.1	2.1	7	5	9	0.6	0.5	0.8	3	2	4
Guatemala	Coatepeque	1.9	1.4	2.5	29	21	38	0.8	0.6	0.9	14	11	17
Guatemala	Cobán	2.0	1.5	2.6	48	36	66	0.7	0.6	0.9	25	20	32
Guatemala	Colomba	2.0	1.4	2.7	12	9	17	0.8	0.6	1.0	4	4	5
Guatemala	Colotenango	1.6	1.2	2.2	5	3	6	0.6	0.5	0.8	2	1	2
Guatemala	Comalapa	1.7	1.2	2.3	9	7	12	0.6	0.5	0.8	3	3	4
Guatemala	Comapa	1.8	1.4	2.5	6	4	8	0.7	0.5	0.9	2	2	3
Guatemala	Comitancillo	1.7	1.2	2.3	11	8	15	0.6	0.5	0.8	5	4	6
Guatemala	Concepción	1.8	1.3	2.4	2	1	2	0.6	0.5	0.8	1	1	1
Guatemala	Concepción Chiquirichapa	1.6	1.2	2.1	4	3	5	0.6	0.5	0.8	2	1	2
Guatemala	Concepción Huista	1.9	1.4	2.5	6	4	8	0.6	0.5	0.8	2	1	2
Guatemala	Concepción Las Minas	1.6	1.2	2.1	3	2	4	0.6	0.5	0.8	1	1	1
Guatemala	Concepción Tutuapa	1.8	1.3	2.4	13	10	17	0.7	0.5	0.8	5	4	6
Guatemala	Conguaco	1.9	1.3	2.5	4	3	6	0.7	0.6	0.9	2	1	2
Guatemala	Cubulco	1.9	1.4	2.5	13	9	17	0.6	0.5	0.8	6	5	7
Guatemala	Cuilapa	1.7	1.2	2.3	8	6	10	0.6	0.5	0.8	3	3	4
Guatemala	Cuilco	1.9	1.4	2.5	18	13	24	0.7	0.5	0.9	7	5	8
Guatemala	Cunén	1.8	1.3	2.4	8	6	10	0.6	0.4	0.7	3	2	3
Guatemala	Cuyotenango	1.8	1.3	2.4	12	9	16	0.8	0.6	0.9	5	4	6
Guatemala	Dolores	1.9	1.4	2.7	13	9	18	0.6	0.5	0.8	5	4	6

Table 3: Diarrhoea Mortality by Unit *(continued)*

Country	Unit	2000 mortality mean rate (per 10,000)	lower rate (per 10,000)	upper rate (per 10,000)	mean count	lower count	upper count	2017 mortality mean rate (per 10,000)	lower rate (per 10,000)	upper rate (per 10,000)	mean count	lower count	upper count
Guatemala	El Adelanto	1.8	1.3	2.5	1	1	2	0.7	0.5	0.9	0	0	1
Guatemala	El Asintal	2.0	1.5	2.7	9	6	11	0.8	0.6	1.0	4	3	5
Guatemala	El Estor	2.1	1.6	2.8	15	11	19	0.8	0.6	1.0	8	7	11
Guatemala	El Jícaro	1.6	1.2	2.0	3	2	4	0.6	0.5	0.7	1	1	1
Guatemala	El Palmar	1.8	1.3	2.3	6	5	8	0.7	0.6	0.9	3	2	3
Guatemala	El Progreso	1.5	1.1	2.1	4	3	6	0.6	0.5	0.7	2	1	2
Guatemala	El Quetzal	2.0	1.5	2.7	5	4	7	0.8	0.6	1.0	2	2	3
Guatemala	El Rodeo	2.0	1.5	2.7	5	4	7	0.8	0.6	1.0	2	2	3
Guatemala	El Tejar	1.5	1.1	2.1	7	5	10	0.6	0.5	0.7	4	3	5
Guatemala	El Tumbador	2.0	1.4	2.6	12	9	16	0.8	0.6	0.9	5	4	6
Guatemala	Escuintla	1.7	1.2	2.2	30	22	40	0.7	0.5	0.8	12	10	15
Guatemala	Esquipulas	1.6	1.2	2.2	11	8	15	0.6	0.5	0.8	5	4	6
Guatemala	Esquipulas Palo Gordo	1.6	1.2	2.2	4	3	6	0.7	0.5	0.8	2	2	3
Guatemala	Estanzuela	1.5	1.1	1.9	2	2	3	0.6	0.5	0.7	1	1	1
Guatemala	Flores	1.8	1.3	2.4	10	7	13	0.6	0.5	0.8	5	4	6
Guatemala	Flores Costa Cuca	1.9	1.4	2.6	7	5	9	0.8	0.6	0.9	3	2	4
Guatemala	Fraijanes	1.5	1.1	2.0	10	7	14	0.6	0.5	0.7	5	4	6
Guatemala	Fray Bartolomé de las Casas	2.0	1.5	2.8	16	12	21	0.7	0.6	0.9	7	5	9
Guatemala	Génova	2.0	1.4	2.6	10	7	13	0.8	0.6	0.9	4	3	5
Guatemala	Granados	1.7	1.2	2.3	3	2	4	0.6	0.5	0.8	1	1	1
Guatemala	Gualán	1.7	1.3	2.3	9	7	12	0.7	0.5	0.8	3	2	4
Guatemala	Guanagazapa	1.8	1.3	2.4	4	3	6	0.7	0.6	0.9	2	1	2
Guatemala	Guastatoya	1.5	1.1	2.0	5	4	7	0.6	0.5	0.7	2	2	3
Guatemala	Guazacapán	1.7	1.2	2.2	3	2	5	0.7	0.5	0.8	1	1	1
Guatemala	Huehuetenango	1.6	1.2	2.2	18	13	24	0.6	0.5	0.8	8	6	10
Guatemala	Huitán	1.8	1.3	2.4	3	2	3	0.7	0.5	0.8	1	1	2
Guatemala	Huité	1.7	1.3	2.3	2	2	3	0.6	0.5	0.8	1	1	1
Guatemala	Ipala	1.5	1.1	2.1	5	3	6	0.6	0.5	0.7	1	1	2
Guatemala	Ixcán	1.9	1.4	2.6	21	16	28	0.7	0.6	0.9	11	9	13
Guatemala	Ixchiguan	1.7	1.2	2.3	6	4	8	0.6	0.5	0.8	2	2	3
Guatemala	Iztapa	1.6	1.2	2.1	2	2	3	0.7	0.5	0.8	1	1	1
Guatemala	Jacaltenango	1.9	1.4	2.6	9	7	13	0.7	0.5	0.8	3	3	4
Guatemala	Jalapa	1.8	1.3	2.3	30	22	39	0.6	0.5	0.8	13	10	16
Guatemala	Jalpatagua	1.7	1.3	2.3	5	4	7	0.7	0.5	0.8	2	2	3
Guatemala	Jerez	1.6	1.2	2.1	2	1	2	0.6	0.5	0.8	1	0	1
Guatemala	Jocotán	1.7	1.3	2.3	10	7	13	0.6	0.5	0.8	5	4	6
Guatemala	Jocotenango	1.5	1.1	2.0	1	1	2	0.6	0.5	0.7	0	0	1
Guatemala	Joyabaj	2.0	1.5	2.7	19	14	26	0.7	0.5	0.8	9	7	11
Guatemala	Jutiapa	1.7	1.3	2.3	27	20	36	0.6	0.5	0.8	12	9	15
Guatemala	La Democracia	1.9	1.4	2.5	6	5	8	0.7	0.6	0.9	3	2	4
Guatemala	La Democracia	1.9	1.4	2.5	11	8	15	0.7	0.5	0.8	4	3	5
Guatemala	La Esperanza	1.4	1.1	1.9	3	2	4	0.6	0.5	0.8	1	1	2
Guatemala	La Gomera	1.8	1.3	2.3	14	10	18	0.7	0.6	0.9	5	4	7
Guatemala	La Libertad	1.8	1.4	2.5	26	19	35	0.6	0.5	0.8	11	9	14
Guatemala	La Libertad	2.0	1.4	2.6	8	6	10	0.7	0.6	0.9	3	2	4
Guatemala	La Reforma	2.0	1.5	2.7	5	4	7	0.8	0.6	1.0	2	2	3
Guatemala	La Unión	1.9	1.4	2.6	6	4	7	0.7	0.6	0.9	2	2	3
Guatemala	Lanquín	2.0	1.5	2.7	7	5	9	0.7	0.6	0.9	3	2	4
Guatemala	Livingston	2.0	1.5	2.6	11	8	14	0.7	0.6	0.9	4	3	5
Guatemala	Los Amates	1.8	1.4	2.5	16	12	22	0.7	0.6	0.9	6	4	7
Guatemala	Magdalena Milpas Altas	1.5	1.1	2.0	2	1	3	0.6	0.5	0.7	1	1	1
Guatemala	Malacatán	2.0	1.5	2.6	21	16	28	0.8	0.6	0.9	11	9	13
Guatemala	Malacatancito	1.7	1.3	2.3	14	10	18	0.6	0.5	0.8	5	4	6
Guatemala	Masagua	1.9	1.3	2.5	10	7	14	0.7	0.6	0.9	5	4	6
Guatemala	Mataquescuintla	1.7	1.2	2.2	8	6	11	0.6	0.5	0.8	4	3	5
Guatemala	Mazatenango	1.7	1.2	2.2	14	10	19	0.7	0.6	0.9	7	6	9

Table 3: Diarrhoea Mortality by Unit *(continued)*

Country	Unit	2000 mortality mean rate (per 10,000)	lower rate (per 10,000)	upper rate (per 10,000)	mean count	lower count	upper count	2017 mortality mean rate (per 10,000)	lower rate (per 10,000)	upper rate (per 10,000)	mean count	lower count	upper count
Guatemala	Melchor de Mencos	1.7	1.3	2.3	6	4	8	0.6	0.4	0.7	2	1	2
Guatemala	Mixco	1.3	1.0	1.8	68	49	91	0.5	0.4	0.6	21	17	26
Guatemala	Momostenango	1.7	1.2	2.2	23	17	30	0.6	0.5	0.8	11	9	14
Guatemala	Monjas	1.5	1.1	2.0	5	4	7	0.6	0.5	0.7	2	2	2
Guatemala	Morales	1.8	1.3	2.4	25	18	33	0.7	0.5	0.9	10	8	13
Guatemala	Morazán	1.6	1.2	2.1	2	2	3	0.6	0.5	0.8	1	1	1
Guatemala	Moyuta	1.7	1.3	2.3	8	6	11	0.7	0.5	0.8	3	3	4
Guatemala	Nahualá	1.7	1.2	2.2	14	10	18	0.6	0.5	0.8	5	4	7
Guatemala	Nebaj	1.9	1.4	2.5	18	13	23	0.6	0.5	0.8	8	7	11
Guatemala	Nentón	1.9	1.4	2.5	10	8	14	0.7	0.5	0.8	5	4	6
Guatemala	Nueva Concepción	1.9	1.3	2.5	16	12	22	0.7	0.6	0.9	5	4	6
Guatemala	Nueva Santa Rosa	1.7	1.2	2.3	7	5	9	0.6	0.5	0.8	2	2	3
Guatemala	Nuevo Progreso	2.1	1.5	2.7	9	6	12	0.8	0.6	1.0	4	3	5
Guatemala	Nuevo San Carlos	1.9	1.4	2.5	7	5	10	0.8	0.6	1.0	3	2	4
Guatemala	Ocos	1.9	1.4	2.5	9	6	12	0.7	0.6	0.9	4	3	5
Guatemala	Olintepeque	1.5	1.1	1.9	6	4	7	0.6	0.5	0.7	2	2	3
Guatemala	Olopa	1.7	1.2	2.3	4	3	5	0.6	0.5	0.8	2	1	2
Guatemala	Oratorio	1.8	1.3	2.4	6	4	8	0.7	0.5	0.9	2	2	3
Guatemala	Ostuncalco	1.6	1.2	2.1	11	8	14	0.6	0.5	0.8	5	4	6
Guatemala	Pachalúm	1.7	1.2	2.3	2	1	3	0.6	0.5	0.8	1	1	1
Guatemala	Pajapita	2.0	1.5	2.6	7	5	9	0.8	0.6	0.9	3	2	4
Guatemala	Palencia	1.7	1.2	2.3	11	8	15	0.6	0.5	0.8	4	3	5
Guatemala	Palestina de Los Altos	1.7	1.3	2.3	3	2	4	0.7	0.5	0.8	1	1	1
Guatemala	Palín	1.6	1.2	2.1	11	8	14	0.6	0.5	0.8	5	4	7
Guatemala	Panajachel	1.8	1.3	2.4	1	1	2	0.7	0.5	0.8	1	1	1
Guatemala	Panzós	2.1	1.6	2.8	27	20	35	0.8	0.6	1.0	11	9	14
Guatemala	Parramos	1.6	1.2	2.2	3	3	5	0.5	0.4	0.7	2	1	2
Guatemala	Pasaco	1.7	1.3	2.3	2	2	3	0.7	0.5	0.9	1	1	1
Guatemala	Pastores	1.5	1.1	2.0	4	3	5	0.6	0.5	0.8	2	1	2
Guatemala	Patulul	2.0	1.5	2.7	10	8	14	0.8	0.6	0.9	5	4	6
Guatemala	Patzicía	1.7	1.2	2.3	7	5	9	0.7	0.5	0.8	3	2	4
Guatemala	Patzité	1.7	1.3	2.3	3	2	4	0.6	0.5	0.8	1	1	2
Guatemala	Patzún	1.7	1.3	2.3	14	10	19	0.6	0.5	0.8	6	4	7
Guatemala	Petapa	1.4	1.0	1.9	22	16	29	0.6	0.4	0.7	9	7	11
Guatemala	Pochuta	2.0	1.4	2.7	4	3	5	0.8	0.6	0.9	1	1	2
Guatemala	Poptún	1.9	1.4	2.6	14	10	19	0.6	0.5	0.8	6	5	8
Guatemala	Pueblo Nuevo	1.8	1.3	2.3	2	1	3	0.7	0.6	0.9	1	1	1
Guatemala	Pueblo Nuevo Viñas	1.8	1.3	2.4	6	4	7	0.7	0.5	0.8	2	2	3
Guatemala	Puerto Barrios	1.7	1.2	2.3	21	16	29	0.6	0.5	0.8	8	6	11
Guatemala	Purulhá	2.1	1.5	2.8	12	9	16	0.7	0.6	0.9	6	5	7
Guatemala	Quetzaltenango	1.4	1.0	1.9	28	21	37	0.6	0.5	0.7	13	10	15
Guatemala	Quezada	1.7	1.3	2.3	4	3	5	0.6	0.5	0.8	2	1	2
Guatemala	Quezaltepeque	1.7	1.2	2.3	7	5	9	0.6	0.5	0.8	2	2	3
Guatemala	Rabinal	1.8	1.3	2.4	9	6	12	0.6	0.5	0.8	3	3	4
Guatemala	Retalhuleu	1.7	1.2	2.3	22	16	29	0.7	0.6	0.9	10	8	12
Guatemala	Río Blanco	1.7	1.3	2.3	2	1	2	0.6	0.5	0.8	1	0	1
Guatemala	Río Bravo	1.8	1.3	2.5	5	4	7	0.8	0.6	0.9	2	2	3
Guatemala	Río Hondo	1.5	1.1	1.9	4	3	5	0.6	0.5	0.8	1	1	2
Guatemala	Sacapulas	2.0	1.5	2.6	15	11	20	0.7	0.6	0.9	7	5	8
Guatemala	Salamá	1.8	1.3	2.3	15	11	20	0.6	0.5	0.8	6	5	7
Guatemala	Salcajá	1.5	1.1	1.9	4	3	6	0.6	0.5	0.8	2	2	3
Guatemala	Samayac	1.8	1.3	2.4	5	4	7	0.7	0.6	0.9	2	2	3
Guatemala	San Agustín Acasaguastlán	1.7	1.3	2.3	6	5	8	0.6	0.5	0.8	2	2	3
Guatemala	San Andrés	1.9	1.4	2.5	9	7	12	0.6	0.5	0.8	5	4	6

Country	Unit	2000 mortality mean rate (per 10,000)	2000 lower rate (per 10,000)	2000 upper rate (per 10,000)	2000 mean count	2000 lower count	2000 upper count	2017 mortality mean rate (per 10,000)	2017 lower rate (per 10,000)	2017 upper rate (per 10,000)	2017 mean count	2017 lower count	2017 upper count
Guatemala	San Andrés Itzapa	1.7	1.2	2.2	9	7	12	0.6	0.5	0.8	5	4	6
Guatemala	San Andrés Sajcabajá	1.9	1.4	2.5	6	5	8	0.7	0.5	0.8	3	2	3
Guatemala	San Andrés Semetabaj	1.8	1.3	2.3	4	3	5	0.6	0.5	0.8	2	1	2
Guatemala	San Andrés Villa Seca	1.8	1.3	2.3	10	7	13	0.8	0.6	0.9	4	3	5
Guatemala	San Andrés Xecul	1.6	1.2	2.1	4	3	6	0.6	0.5	0.8	2	1	2
Guatemala	San Antonio Aguas Calientes	1.5	1.1	2.0	1	0	1	0.6	0.5	0.8	0	0	0
Guatemala	San Antonio Huista	1.9	1.4	2.5	5	4	7	0.7	0.5	0.8	2	2	3
Guatemala	San Antonio Ilotenango	1.8	1.3	2.4	10	7	13	0.6	0.5	0.8	4	3	5
Guatemala	San Antonio La Paz	1.7	1.3	2.3	4	3	6	0.6	0.5	0.8	1	1	2
Guatemala	San Antonio Palopó	1.8	1.3	2.4	3	2	3	0.7	0.5	0.8	1	1	1
Guatemala	San Antonio Sacatepéquez	1.7	1.3	2.3	4	3	5	0.7	0.5	0.8	2	1	2
Guatemala	San Antonio Suchitepéquez	2.0	1.4	2.6	9	7	12	0.8	0.6	1.0	5	4	6
Guatemala	San Bartolo	1.7	1.3	2.3	2	1	3	0.6	0.5	0.8	1	1	2
Guatemala	San Bartolomé Jocotenango	2.0	1.5	2.6	3	2	4	0.7	0.5	0.8	1	1	2
Guatemala	San Bartolomé Milpas Altas	1.6	1.1	2.1	1	1	2	0.6	0.5	0.8	1	0	1
Guatemala	San Benito	1.8	1.4	2.5	10	7	13	0.6	0.5	0.8	5	4	7
Guatemala	San Bernardino	1.9	1.4	2.5	5	4	6	0.8	0.6	1.0	2	2	3
Guatemala	San Carlos Alzatate	1.7	1.2	2.2	3	2	4	0.6	0.5	0.8	2	1	2
Guatemala	San Carlos Sija	1.6	1.2	2.1	8	6	10	0.6	0.5	0.7	3	2	4
Guatemala	San Cristóbal Acasaguastlán	1.7	1.2	2.2	1	1	2	0.6	0.5	0.8	0	0	0
Guatemala	San Cristobal Cucho	1.7	1.2	2.3	3	2	4	0.7	0.6	0.9	1	1	2
Guatemala	San Cristóbal Totonicapán	1.6	1.2	2.1	8	6	10	0.6	0.5	0.8	3	3	4
Guatemala	San Cristóbal Verapaz	1.9	1.4	2.5	14	11	19	0.7	0.5	0.8	6	5	8
Guatemala	San Diego	1.7	1.2	2.2	1	1	2	0.6	0.5	0.8	0	0	1
Guatemala	San Felipe	1.8	1.3	2.3	4	3	5	0.7	0.6	0.9	2	1	2
Guatemala	San Francisco	2.0	1.5	2.7	4	3	6	0.7	0.5	0.8	2	2	3
Guatemala	San Francisco El Alto	1.7	1.2	2.2	11	8	15	0.6	0.5	0.8	5	4	6
Guatemala	San Francisco La Unión	1.5	1.1	2.0	2	1	2	0.6	0.5	0.7	1	1	1
Guatemala	San Francisco Zapotitlán	1.7	1.3	2.2	2	1	2	0.7	0.6	0.9	1	1	1
Guatemala	San Gabriel	1.7	1.2	2.2	5	4	7	0.7	0.6	0.9	3	2	4
Guatemala	San Gaspar Ixchil	1.9	1.4	2.6	3	2	3	0.7	0.5	0.8	1	1	1
Guatemala	San Ildefonso Ixtahuacán	1.8	1.3	2.4	12	9	16	0.7	0.5	0.8	5	4	6
Guatemala	San Jacinto	1.7	1.2	2.2	3	2	4	0.6	0.5	0.8	1	1	1
Guatemala	San Jerónimo	1.7	1.3	2.3	5	4	7	0.6	0.5	0.8	2	2	3
Guatemala	San José	1.5	1.1	2.0	8	6	11	0.6	0.5	0.8	3	2	4
Guatemala	San José	1.9	1.4	2.6	1	1	2	0.6	0.5	0.8	1	0	1
Guatemala	San José Acatempa	1.6	1.2	2.2	3	2	4	0.6	0.5	0.8	1	1	1

Table 3: Diarrhoea Mortality by Unit *(continued)*

Country	Unit	2000 mortality mean rate (per 10,000)	2000 lower rate (per 10,000)	2000 upper rate (per 10,000)	2000 mean count	2000 lower count	2000 upper count	2017 mortality mean rate (per 10,000)	2017 lower rate (per 10,000)	2017 upper rate (per 10,000)	2017 mean count	2017 lower count	2017 upper count
Guatemala	San José Chacayá	1.7	1.2	2.2	1	1	1	0.6	0.5	0.8	1	0	1
Guatemala	San José del Golfo	1.6	1.2	2.2	1	1	2	0.6	0.5	0.8	0	0	1
Guatemala	San José El Idolo	1.9	1.4	2.5	3	2	4	0.8	0.6	1.0	1	1	2
Guatemala	San José La Arada	1.6	1.2	2.1	2	2	3	0.6	0.5	0.8	1	1	1
Guatemala	San José Ojetenam	1.5	1.1	2.0	4	3	5	0.6	0.5	0.7	2	1	2
Guatemala	San José Pinula	1.6	1.1	2.1	9	6	12	0.6	0.5	0.7	4	3	5
Guatemala	San José Poaquil	1.7	1.2	2.3	5	3	6	0.6	0.5	0.8	2	1	2
Guatemala	San Juan Atitán	1.7	1.3	2.3	4	3	5	0.6	0.5	0.8	1	1	2
Guatemala	San Juan Bautista	2.0	1.4	2.6	2	2	3	0.8	0.6	0.9	1	1	1
Guatemala	San Juan Chamelco	1.9	1.4	2.5	16	12	21	0.7	0.5	0.9	7	6	9
Guatemala	San Juan Cotzal	2.0	1.5	2.7	7	5	10	0.7	0.5	0.8	3	3	4
Guatemala	San Juan Ermita	1.7	1.2	2.3	3	3	5	0.6	0.5	0.8	1	1	2
Guatemala	San Juan Ixcoy	1.7	1.2	2.3	8	6	11	0.6	0.5	0.8	3	3	4
Guatemala	San Juan La Laguna	1.8	1.3	2.3	2	2	3	0.7	0.5	0.8	1	1	1
Guatemala	San Juan Sacatepéquez	1.6	1.2	2.2	26	19	35	0.6	0.5	0.7	11	9	14
Guatemala	San Juan Tecuaco	1.9	1.3	2.5	2	1	3	0.7	0.6	0.9	1	1	1
Guatemala	San Lorenzo	1.8	1.3	2.4	4	3	5	0.8	0.6	0.9	2	1	2
Guatemala	San Lorenzo	1.6	1.2	2.1	3	2	4	0.6	0.5	0.7	1	1	2
Guatemala	San Lucas Sacatepéquez	1.4	1.0	1.9	6	4	8	0.6	0.5	0.7	2	2	3
Guatemala	San Lucas Tolimán	1.9	1.4	2.5	6	5	8	0.7	0.6	0.9	3	2	3
Guatemala	San Luis	1.9	1.4	2.6	19	14	26	0.6	0.5	0.8	7	6	10
Guatemala	San Luis	1.7	1.2	2.2	4	3	5	0.6	0.5	0.8	2	1	2
Guatemala	San Luis Jilotepeque	1.6	1.2	2.1	5	4	7	0.6	0.5	0.7	2	2	3
Guatemala	San Manuel Chaparrón	1.5	1.1	2.0	2	1	3	0.6	0.5	0.7	1	1	1
Guatemala	San Marcos	1.5	1.1	2.0	11	8	15	0.6	0.5	0.8	5	4	6
Guatemala	San Marcos La Laguna	1.8	1.3	2.3	1	1	1	0.7	0.5	0.8	0	0	0
Guatemala	San Martín Jilotepeque	1.8	1.3	2.4	18	13	23	0.6	0.5	0.8	7	5	8
Guatemala	San Martín Sacatepéquez	1.8	1.3	2.4	9	6	11	0.7	0.5	0.9	3	3	4
Guatemala	San Martín Zapotitlán	1.8	1.3	2.3	2	1	2	0.8	0.6	0.9	1	1	1
Guatemala	San Mateo	1.6	1.1	2.1	3	2	4	0.6	0.5	0.8	1	1	2
Guatemala	San Mateo Ixtatán	1.9	1.4	2.5	9	7	13	0.7	0.5	0.8	4	3	4
Guatemala	San Miguel Acatán	1.9	1.4	2.6	7	5	9	0.7	0.5	0.8	2	2	3
Guatemala	San Miguel Chicaj	1.8	1.3	2.5	7	5	10	0.7	0.5	0.8	3	2	4
Guatemala	San Miguel Dueñas	1.6	1.2	2.2	3	2	4	0.6	0.5	0.8	1	1	2
Guatemala	San Miguel Ixtahuacán	1.7	1.3	2.3	9	6	11	0.6	0.5	0.8	3	2	4
Guatemala	San Miguel Panán	2.2	1.6	2.9	3	2	4	0.9	0.7	1.1	1	1	1
Guatemala	San Miguel Sigüilá	1.6	1.2	2.1	2	1	2	0.6	0.5	0.8	1	1	1
Guatemala	San Pablo	1.9	1.4	2.5	10	7	13	0.7	0.6	0.9	5	4	6

Table 3: Diarrhoea Mortality by Unit *(continued)*

Country	Unit	2000 mortality mean rate (per 10,000)	lower rate (per 10,000)	upper rate (per 10,000)	mean count	lower count	upper count	2017 mortality mean rate (per 10,000)	lower rate (per 10,000)	upper rate (per 10,000)	mean count	lower count	upper count
Guatemala	San Pablo Jocopilas	2.0	1.4	2.6	5	3	6	0.8	0.6	0.9	2	1	2
Guatemala	San Pablo La Laguna	1.8	1.3	2.3	1	0	1	0.7	0.5	0.8	0	0	0
Guatemala	San Pedro Ayampuc	1.6	1.2	2.2	8	6	11	0.6	0.5	0.8	4	3	5
Guatemala	San Pedro Carchá	1.9	1.4	2.6	46	34	63	0.7	0.6	0.9	21	17	27
Guatemala	San Pedro Jocopilas	1.9	1.4	2.5	9	6	11	0.6	0.5	0.8	3	3	4
Guatemala	San Pedro La Laguna	1.6	1.2	2.1	2	2	3	0.7	0.5	0.8	1	1	1
Guatemala	San Pedro Necta	1.8	1.3	2.3	9	7	12	0.6	0.5	0.8	4	3	5
Guatemala	San Pedro Pinula	1.9	1.4	2.6	13	10	18	0.7	0.6	0.9	6	5	7
Guatemala	San Pedro Sacatepéquez	1.5	1.1	2.0	10	7	13	0.5	0.4	0.7	3	3	4
Guatemala	San Pedro Sacatepéquez	1.5	1.1	2.0	12	9	15	0.6	0.5	0.8	5	4	6
Guatemala	San Rafael La Independencia	1.8	1.3	2.4	3	2	4	0.6	0.5	0.8	1	1	1
Guatemala	San Rafaél Las Flores	1.7	1.3	2.3	3	2	4	0.6	0.5	0.8	1	1	2
Guatemala	San Rafael Petzal	1.8	1.3	2.3	2	2	3	0.6	0.5	0.8	1	1	1
Guatemala	San Rafaél Pie de la Cuesta	1.9	1.4	2.5	4	3	5	0.7	0.6	0.9	2	1	2
Guatemala	San Raymundo	1.7	1.2	2.3	5	4	7	0.6	0.5	0.8	2	2	3
Guatemala	San Sebastián	1.7	1.3	2.3	4	3	5	0.8	0.6	0.9	2	1	2
Guatemala	San Sebastián Coatán	1.7	1.2	2.3	5	4	7	0.6	0.5	0.8	2	2	3
Guatemala	San Sebastián Huehuetenango	1.7	1.3	2.3	6	4	8	0.6	0.5	0.7	2	2	3
Guatemala	San Sibinal	1.8	1.3	2.4	4	3	6	0.7	0.5	0.8	2	1	2
Guatemala	San Vicente Pacaya	1.6	1.2	2.2	5	4	7	0.6	0.5	0.8	2	2	3
Guatemala	Sanarate	1.5	1.2	2.1	7	5	9	0.6	0.5	0.7	2	2	3
Guatemala	Sansare	1.7	1.2	2.2	2	2	3	0.6	0.5	0.8	1	1	1
Guatemala	Santa Ana	2.0	1.5	2.7	8	6	11	0.7	0.5	0.8	4	3	6
Guatemala	Santa Ana Huista	1.9	1.4	2.6	4	3	5	0.7	0.6	0.9	1	1	2
Guatemala	Santa Apolonia	1.7	1.2	2.3	3	2	4	0.6	0.5	0.8	1	1	1
Guatemala	Santa Bárbara	2.0	1.4	2.6	6	4	8	0.8	0.6	0.9	2	2	3
Guatemala	Santa Bárbara	1.9	1.4	2.5	6	5	8	0.7	0.5	0.8	2	2	2
Guatemala	Santa Catarina Barahona	1.6	1.2	2.2	1	0	1	0.6	0.5	0.8	0	0	0
Guatemala	Santa Catarina Ixtahuacan	1.8	1.3	2.4	13	10	18	0.7	0.5	0.8	5	4	6
Guatemala	Santa Catarina Mita	1.5	1.1	2.0	5	4	7	0.6	0.5	0.7	2	1	2
Guatemala	Santa Catarina Palopó	1.8	1.3	2.3	1	1	1	0.7	0.5	0.8	1	0	1
Guatemala	Santa Catarina Pinula	1.4	1.0	1.9	11	8	15	0.6	0.4	0.7	4	3	5
Guatemala	Santa Clara La Laguna	1.8	1.3	2.3	1	1	2	0.7	0.5	0.8	1	0	1
Guatemala	Santa Cruz Balanyá	1.7	1.3	2.3	2	1	3	0.7	0.5	0.8	1	1	1
Guatemala	Santa Cruz Barillas	2.1	1.5	2.8	27	19	36	0.8	0.6	0.9	16	13	20
Guatemala	Santa Cruz del Quiché	1.7	1.3	2.3	14	10	18	0.6	0.5	0.7	7	5	9
Guatemala	Santa Cruz El Chol	1.6	1.2	2.2	2	2	3	0.6	0.5	0.7	1	1	1

Table 3: Diarrhoea Mortality by Unit *(continued)*

Country	Unit	2000 mortality mean rate (per 10,000)	2000 lower rate (per 10,000)	2000 upper rate (per 10,000)	2000 mean count	2000 lower count	2000 upper count	2017 mortality mean rate (per 10,000)	2017 lower rate (per 10,000)	2017 upper rate (per 10,000)	2017 mean count	2017 lower count	2017 upper count
Guatemala	Santa Cruz La Laguna	1.9	1.4	2.5	1	1	1	0.7	0.5	0.9	1	0	1
Guatemala	Santa Cruz Muluá	1.8	1.3	2.3	7	5	9	0.8	0.6	0.9	3	3	4
Guatemala	Santa Cruz Naranjo	1.7	1.2	2.3	2	2	3	0.6	0.5	0.8	1	1	1
Guatemala	Santa Cruz Verapaz	1.9	1.4	2.5	6	4	8	0.7	0.5	0.8	3	3	4
Guatemala	Santa Eulalia	2.0	1.4	2.6	12	9	16	0.7	0.6	0.9	6	5	7
Guatemala	Santa Lucía Cotzumalguapa	1.8	1.3	2.4	24	17	32	0.7	0.6	0.9	11	9	14
Guatemala	Santa Lucía La Reforma	2.0	1.4	2.6	3	2	4	0.6	0.5	0.8	1	1	2
Guatemala	Santa Lucía Milpas Altas	1.5	1.1	2.0	2	2	3	0.6	0.5	0.7	1	1	1
Guatemala	Santa Lucía Utatlán	1.7	1.2	2.2	5	4	7	0.6	0.5	0.8	2	2	3
Guatemala	Santa María Cahabón	2.1	1.5	2.8	16	11	21	0.8	0.6	1.0	7	5	8
Guatemala	Santa María Chiquimula	1.8	1.3	2.3	11	8	15	0.6	0.5	0.8	5	4	6
Guatemala	Santa María de Jesús	1.6	1.2	2.1	5	4	6	0.6	0.5	0.7	2	1	2
Guatemala	Santa María Ixhuatán	1.7	1.3	2.3	5	4	6	0.7	0.5	0.8	2	2	2
Guatemala	Santa María Visitación	1.8	1.3	2.4	1	1	2	0.7	0.5	0.8	1	0	1
Guatemala	Santa Rosa de Lima	1.7	1.2	2.2	4	3	6	0.6	0.5	0.8	2	1	2
Guatemala	Santiago Atitlán	1.7	1.2	2.2	8	6	10	0.6	0.5	0.8	3	3	4
Guatemala	Santiago Chimaltenango	1.8	1.3	2.3	3	3	5	0.6	0.5	0.8	1	1	2
Guatemala	Santiago Sacatepéquez	1.7	1.2	2.2	5	4	7	0.6	0.5	0.7	2	2	2
Guatemala	Santo Domingo Suchitepequez	1.8	1.3	2.4	12	9	15	0.8	0.6	0.9	5	4	6
Guatemala	Santo Domingo Xenacoj	1.7	1.2	2.2	3	2	3	0.6	0.5	0.8	1	1	1
Guatemala	Santo Tomás La Unión	2.0	1.5	2.7	2	1	2	0.8	0.6	1.0	1	1	1
Guatemala	Sayaxché	2.1	1.5	2.8	24	18	33	0.7	0.6	0.9	13	10	16
Guatemala	Senahú	2.1	1.5	2.7	19	14	25	0.7	0.6	0.9	7	5	9
Guatemala	Sibilia	1.6	1.2	2.2	2	2	3	0.6	0.5	0.8	1	1	1
Guatemala	Sipacapa	1.8	1.3	2.3	4	3	6	0.6	0.5	0.7	2	1	2
Guatemala	Siquinalá	1.8	1.3	2.4	5	4	7	0.7	0.6	0.9	3	2	3
Guatemala	Sololá	1.7	1.2	2.2	17	13	23	0.6	0.5	0.7	10	8	12
Guatemala	Soloma	1.7	1.2	2.2	8	6	11	0.6	0.5	0.8	3	3	4
Guatemala	Sumpango	1.6	1.2	2.2	5	4	7	0.6	0.5	0.8	2	2	3
Guatemala	Tacaná	1.8	1.3	2.4	17	12	22	0.7	0.5	0.8	6	5	8
Guatemala	Tactic	1.8	1.3	2.5	8	6	11	0.7	0.5	0.8	4	3	5
Guatemala	Tajumulco	1.9	1.4	2.5	13	10	17	0.7	0.5	0.9	6	5	7
Guatemala	Tamahú	2.0	1.4	2.6	5	3	6	0.7	0.5	0.9	2	2	2
Guatemala	Taxisco	1.7	1.3	2.3	6	5	9	0.7	0.5	0.8	2	2	3
Guatemala	Tecpán Guatemala	1.7	1.2	2.3	18	13	24	0.6	0.5	0.8	8	6	9
Guatemala	Tectitán	1.9	1.4	2.5	3	2	4	0.7	0.5	0.8	1	1	1
Guatemala	Teculután	1.5	1.1	2.0	3	2	4	0.6	0.5	0.7	1	1	1
Guatemala	Tejutla	1.8	1.3	2.4	9	7	12	0.7	0.5	0.8	3	3	4
Guatemala	Tiquisate	1.7	1.2	2.2	12	9	17	0.7	0.6	0.9	5	4	6
Guatemala	Todos Santos Cuchumatán	1.7	1.2	2.2	8	6	11	0.6	0.5	0.7	3	2	4

Table 3: Diarrhoea Mortality by Unit *(continued)*

Country	Unit	2000 mortality mean rate (per 10,000)	lower rate (per 10,000)	upper rate (per 10,000)	mean count	lower count	upper count	2017 mortality mean rate (per 10,000)	lower rate (per 10,000)	upper rate (per 10,000)	mean count	lower count	upper count
Guatemala	Totonicapán	1.6	1.2	2.1	26	19	34	0.6	0.5	0.7	11	9	14
Guatemala	Tucurú	2.0	1.5	2.7	9	7	13	0.8	0.6	1.0	4	3	5
Guatemala	Uspantán	2.0	1.5	2.7	14	11	19	0.7	0.5	0.9	7	5	8
Guatemala	Usumatlán	1.6	1.2	2.1	2	1	2	0.6	0.5	0.8	1	1	1
Guatemala	Villa Canales	1.5	1.1	2.0	36	27	49	0.6	0.5	0.7	16	13	20
Guatemala	Villa Nueva	1.4	1.0	1.9	67	49	90	0.6	0.5	0.7	28	22	35
Guatemala	Yepocapa	2.0	1.4	2.7	8	6	11	0.7	0.6	0.9	3	3	4
Guatemala	Yupiltepeque	1.8	1.3	2.4	3	2	4	0.7	0.5	0.8	1	1	1
Guatemala	Zacapa	1.6	1.2	2.1	13	10	17	0.6	0.5	0.8	5	4	6
Guatemala	Zacualpa	2.1	1.5	2.8	8	6	11	0.7	0.5	0.8	5	4	6
Guatemala	Zapotitlán	1.8	1.3	2.4	3	2	4	0.7	0.5	0.9	1	1	1
Guatemala	Zaragoza	1.7	1.3	2.3	6	5	8	0.7	0.5	0.8	3	2	3
Guatemala	ZONA 1	1.3	0.9	1.7	7	5	9	0.5	0.4	0.6	2	2	3
Guatemala	ZONA 10	1.2	0.9	1.6	5	4	6	0.5	0.4	0.6	2	1	2
Guatemala	ZONA 11	1.2	0.9	1.7	11	8	15	0.5	0.4	0.7	4	3	5
Guatemala	ZONA 12	1.2	0.9	1.7	11	8	14	0.5	0.4	0.7	4	3	5
Guatemala	ZONA 13	1.2	0.9	1.6	5	4	7	0.5	0.4	0.6	2	1	2
Guatemala	ZONA 14	1.3	0.9	1.7	3	2	4	0.5	0.4	0.6	1	1	1
Guatemala	ZONA 15	1.3	0.9	1.7	5	3	6	0.5	0.4	0.6	1	1	2
Guatemala	ZONA 16	1.2	0.9	1.7	4	3	6	0.5	0.4	0.6	1	1	2
Guatemala	ZONA 17	1.3	0.9	1.7	15	11	21	0.5	0.4	0.6	4	3	6
Guatemala	ZONA 18	1.3	0.9	1.7	20	15	27	0.5	0.4	0.6	7	5	8
Guatemala	ZONA 19	1.3	1.0	1.8	1	1	2	0.5	0.4	0.7	0	0	1
Guatemala	ZONA 2	1.3	0.9	1.7	6	4	8	0.5	0.4	0.7	2	2	2
Guatemala	ZONA 22	1.3	0.9	1.7	3	2	4	0.5	0.4	0.7	1	1	1
Guatemala	ZONA 24	1.3	0.9	1.7	5	4	7	0.5	0.4	0.7	2	1	2
Guatemala	ZONA 25	1.3	1.0	1.8	8	6	11	0.5	0.4	0.7	3	2	3
Guatemala	ZONA 3	1.3	0.9	1.7	4	3	6	0.5	0.4	0.6	1	1	2
Guatemala	ZONA 4	1.2	0.9	1.6	1	1	1	0.5	0.4	0.6	0	0	0
Guatemala	ZONA 5	1.3	0.9	1.7	6	4	8	0.5	0.4	0.6	2	1	2
Guatemala	ZONA 6	1.3	0.9	1.7	6	5	8	0.5	0.4	0.6	2	2	2
Guatemala	ZONA 7	1.3	0.9	1.7	18	13	24	0.5	0.4	0.7	5	4	7
Guatemala	ZONA 8	1.2	0.9	1.6	1	1	2	0.5	0.4	0.6	0	0	1
Guatemala	ZONA 9	1.2	0.9	1.6	2	2	3	0.5	0.4	0.6	1	1	1
Guatemala	Zunil	1.5	1.1	2.0	5	3	6	0.6	0.5	0.8	2	2	2
Guatemala	Zunilito	1.7	1.3	2.3	2	2	3	0.7	0.6	0.9	1	1	1
Guatemala	NA	1.8	1.3	2.4	9	6	11	0.7	0.6	0.9	4	3	5
Guatemala	NA	1.9	1.5	2.6	1	1	2	0.8	0.6	1.0	1	1	1
Guyana	Abary / Mahaicony	0.6	0.5	0.8	0	0	0	0.2	0.2	0.3	0	0	0
Guyana	Agatash	0.7	0.6	0.9	0	0	0	0.3	0.2	0.3	0	0	0
Guyana	Aishalton - Karaudanawa, Achiwib	1.3	1.0	1.7	0	0	1	0.5	0.4	0.6	0	0	0
Guyana	Amsterdam (Demerara River) / Vriesland	0.8	0.6	1.0	1	1	1	0.2	0.1	0.2	0	0	0
Guyana	Anna Regina	0.6	0.5	0.8	1	1	2	0.2	0.2	0.3	0	0	0
Guyana	Arau	1.3	1.0	1.6	0	0	0	0.4	0.3	0.5	0	0	0
Guyana	Barima / Amakura	1.2	0.9	1.5	1	1	2	0.4	0.3	0.4	0	0	0
Guyana	Bartica	0.7	0.5	0.9	0	0	0	0.3	0.2	0.3	0	0	0
Guyana	Bel Air / Woodlands	0.6	0.4	0.8	0	0	0	0.2	0.2	0.3	0	0	0
Guyana	Berbice River Settlements	0.9	0.7	1.1	0	0	0	0.3	0.3	0.4	0	0	0
Guyana	Black Bush Polder land Development Scheme	0.6	0.4	0.8	0	0	0	0.2	0.1	0.2	0	0	0

Table 3: Diarrhoea Mortality by Unit *(continued)*

Country	Unit	2000 mortality mean rate (per 10,000)	2000 mortality lower rate (per 10,000)	2000 mortality upper rate (per 10,000)	2000 mortality mean count	2000 mortality lower count	2000 mortality upper count	2017 mortality mean rate (per 10,000)	2017 mortality lower rate (per 10,000)	2017 mortality upper rate (per 10,000)	2017 mortality mean count	2017 mortality lower count	2017 mortality upper count
Guyana	Blankenburg / Hague	0.8	0.6	1.0	1	0	1	0.2	0.1	0.2	0	0	0
Guyana	Bonasika / Boerasirie	0.8	0.6	1.0	0	0	1	0.2	0.1	0.2	0	0	0
Guyana	Borlam (No.37) / Kintyre	0.6	0.4	0.8	0	0	0	0.2	0.1	0.2	0	0	0
Guyana	Bush Lot / Adventure	0.6	0.4	0.8	0	0	0	0.2	0.1	0.2	0	0	0
Guyana	Canal No. 2 (part) + The Belle + Little Alliance	0.8	0.6	1.1	0	0	0	0.2	0.1	0.2	0	0	0
Guyana	Canals Polder	0.9	0.7	1.1	1	1	1	0.2	0.1	0.2	0	0	0
Guyana	Cane Field / Enterprise	0.6	0.4	0.8	0	0	0	0.2	0.1	0.2	0	0	0
Guyana	Cane Grove Land Development Scheme	0.6	0.5	0.8	0	0	0	0.2	0.1	0.2	0	0	0
Guyana	Chance / Hamlet	0.6	0.5	0.8	0	0	0	0.2	0.2	0.3	0	0	0
Guyana	Charity / Urasara	0.7	0.5	0.9	2	1	2	0.2	0.2	0.3	0	0	0
Guyana	Chenapau River	1.3	1.0	1.6	0	0	0	0.5	0.4	0.6	0	0	0
Guyana	City of Georgetown	0.7	0.5	0.9	9	7	11	0.2	0.1	0.2	2	1	2
Guyana	Coomaka Lands	0.9	0.7	1.1	1	1	1	0.3	0.3	0.4	0	0	0
Guyana	Corentyne River	0.6	0.4	0.8	2	1	2	0.2	0.1	0.2	0	0	0
Guyana	Cornelia Ida / Stewartville	0.8	0.6	1.0	1	1	2	0.2	0.1	0.2	0	0	0
Guyana	Corriverton	0.6	0.4	0.8	1	0	1	0.2	0.1	0.2	0	0	0
Guyana	Demerara Conservancy	0.7	0.5	0.9	0	0	1	0.2	0.1	0.2	0	0	0
Guyana	Diamond / Golden Grove	0.8	0.6	1.0	1	1	1	0.2	0.1	0.2	0	0	0
Guyana	East Bank Berbice	1.0	0.7	1.2	0	0	0	0.4	0.3	0.5	0	0	0
Guyana	Eccles / Ramsburg	0.7	0.6	0.9	3	2	4	0.2	0.1	0.2	1	0	1
Guyana	Enfield / New Doe Park	0.6	0.4	0.8	0	0	1	0.2	0.1	0.2	0	0	0
Guyana	Enmore / Hope	0.7	0.5	0.9	0	0	0	0.2	0.2	0.3	0	0	0
Guyana	Farm / Woodlands	0.7	0.5	0.9	1	0	1	0.2	0.2	0.3	0	0	0
Guyana	Foulis / Buxton	0.7	0.5	0.9	2	2	3	0.2	0.2	0.2	0	0	0
Guyana	Fyrish / Gibraltar	0.6	0.4	0.8	0	0	0	0.2	0.1	0.2	0	0	0
Guyana	Gelderland / No. 3	0.6	0.4	0.8	1	1	1	0.2	0.1	0.2	0	0	0
Guyana	Good Hope / Hydronie	0.7	0.6	0.9	0	0	0	0.2	0.1	0.2	0	0	0
Guyana	Good Hope / Pomona	0.7	0.5	0.9	0	0	0	0.3	0.2	0.3	0	0	0
Guyana	Good Success / Caledonia	0.8	0.6	1.0	1	1	1	0.2	0.1	0.2	0	0	0
Guyana	Grove / Haslington	0.7	0.5	0.9	1	1	2	0.2	0.2	0.3	0	0	0
Guyana	Hampshire / Kilcoy	0.6	0.5	0.8	0	0	0	0.2	0.1	0.2	0	0	0
Guyana	Herstelling / Little Diamond	0.8	0.6	1.0	1	1	1	0.2	0.1	0.2	0	0	0
Guyana	Hogstye / Lancaster	0.6	0.4	0.8	0	0	0	0.2	0.1	0.2	0	0	0

Table 3: Diarrhoea Mortality by Unit *(continued)*

Country	Unit	2000 mortality mean rate (per 10,000)	lower rate (per 10,000)	upper rate (per 10,000)	mean count	lower count	upper count	2017 mortality mean rate (per 10,000)	lower rate (per 10,000)	upper rate (per 10,000)	mean count	lower count	upper count
Guyana	Ireng / Sawariwau (Including St. Ignatius)	1.2	1.0	1.6	1	1	1	0.4	0.3	0.5	0	0	1
Guyana	Ituni	0.9	0.7	1.1	0	0	0	0.3	0.3	0.4	0	0	0
Guyana	Jackson Creek / Crabwood Creek	0.6	0.5	0.8	0	0	1	0.2	0.2	0.2	0	0	0
Guyana	Jawalla, Kubenang River	1.3	1.0	1.6	0	0	0	0.4	0.3	0.5	0	0	0
Guyana	John / Port Mourant	0.6	0.5	0.8	1	1	1	0.2	0.1	0.2	0	0	0
Guyana	Joppa / Macedonia	0.5	0.4	0.7	0	0	0	0.2	0.1	0.2	0	0	0
Guyana	Kaibarupai	1.2	1.0	1.5	0	0	0	0.5	0.3	0.6	0	0	0
Guyana	Kamarang	1.3	1.0	1.7	0	0	0	0.4	0.3	0.5	0	0	0
Guyana	Karambaru to Kukui River + Phillipi	1.3	1.0	1.7	0	0	0	0.4	0.3	0.5	0	0	0
Guyana	Klein Pouderoyen / Best	0.7	0.6	0.9	1	1	1	0.2	0.1	0.2	0	0	0
Guyana	Kopanang, Waipa, Kenepai	1.3	1.0	1.6	0	0	0	0.4	0.3	0.6	0	0	0
Guyana	Kwakwani	0.8	0.6	1.0	1	0	1	0.3	0.2	0.4	0	0	0
Guyana	La Bonne Intention / Better Hope	0.7	0.5	0.9	3	2	3	0.2	0.1	0.2	1	0	1
Guyana	La Reconnaissance / Mon Repos	0.7	0.5	0.9	1	1	2	0.2	0.1	0.2	0	0	0
Guyana	Leguan (Essequibo Islands)	0.7	0.5	0.9	0	0	0	0.2	0.1	0.2	0	0	0
Guyana	Linden	0.9	0.7	1.1	5	4	6	0.3	0.2	0.4	1	1	2
Guyana	Lower West Demerara	0.7	0.6	0.9	0	0	0	0.2	0.2	0.3	0	0	0
Guyana	Mabaruma / Kumaka / Hosororo	1.2	0.9	1.5	1	1	2	0.3	0.3	0.4	0	0	0
Guyana	Mabura Hills	0.9	0.7	1.2	0	0	0	0.4	0.3	0.5	0	0	0
Guyana	Madhia + Kurubrong River + Mona Falls	1.2	1.0	1.5	0	0	1	0.4	0.3	0.5	0	0	0
Guyana	Makouria River	0.7	0.6	0.9	0	0	1	0.3	0.2	0.3	0	0	0
Guyana	Maripari River + Kurukabaru	1.2	1.0	1.5	0	0	0	0.4	0.3	0.6	0	0	0
Guyana	Marudi	1.4	1.1	1.8	0	0	0	0.5	0.4	0.6	0	0	0
Guyana	Matthews Ridge / Arakaka (Matakai) / Port Kaituma	1.3	1.0	1.6	2	1	2	0.4	0.3	0.5	0	0	1
Guyana	Meer Zorgen / Malgre Tout	0.7	0.6	0.9	1	1	1	0.2	0.1	0.2	0	0	0
Guyana	Mocha / Arcadia	0.7	0.6	0.9	0	0	0	0.2	0.1	0.2	0	0	0
Guyana	Monkey Mountain	1.2	1.0	1.5	0	0	0	0.4	0.3	0.6	0	0	0
Guyana	Naarstigheid / Union	0.6	0.5	0.8	1	0	1	0.2	0.2	0.3	0	0	0
Guyana	New Amsterdam	0.6	0.5	0.8	0	0	1	0.2	0.1	0.2	0	0	0
Guyana	Nismes / La Grange	0.7	0.6	0.9	0	0	1	0.2	0.1	0.2	0	0	0

Table 3: Diarrhoea Mortality by Unit *(continued)*

Country	Unit	2000 mortality						2017 mortality					
		mean rate (per 10,000)	lower rate (per 10,000)	upper rate (per 10,000)	mean count	lower count	upper count	mean rate (per 10,000)	lower rate (per 10,000)	upper rate (per 10,000)	mean count	lower count	upper count
Guyana	No. 38 / Ordnance Fortlands	0.6	0.4	0.8	1	1	1	0.2	0.1	0.2	0	0	0
Guyana	No.51 Village / Good Hope	0.6	0.4	0.8	0	0	0	0.2	0.1	0.2	0	0	0
Guyana	No.74 Village / No.52 Village	0.6	0.4	0.8	1	1	1	0.2	0.1	0.2	0	0	0
Guyana	Nouvelle Flanders / La Jalousie	0.8	0.6	1.0	0	0	1	0.2	0.1	0.2	0	0	0
Guyana	Paradise / Evergreen (including Somerset and Berks)	0.6	0.5	0.8	0	0	0	0.2	0.2	0.3	0	0	0
Guyana	Paramakatoi	1.3	1.0	1.6	0	0	0	0.5	0.4	0.6	0	0	0
Guyana	Parika / Mora	0.7	0.6	0.9	0	0	0	0.2	0.1	0.2	0	0	0
Guyana	Paruima	1.3	1.0	1.6	0	0	0	0.4	0.3	0.5	0	0	0
Guyana	Patentia / Toevlugt	0.8	0.6	1.0	1	1	1	0.2	0.1	0.2	0	0	0
Guyana	Plaisance / Industry	0.7	0.5	0.9	2	2	3	0.2	0.1	0.2	0	0	1
Guyana	Rest of Region 1	1.2	1.0	1.5	0	0	0	0.4	0.3	0.5	0	0	0
Guyana	Rest of Region 10	0.9	0.7	1.1	1	0	1	0.3	0.3	0.4	0	0	0
Guyana	Rest of Region 7	1.2	0.9	1.4	1	1	1	0.4	0.3	0.5	0	0	0
Guyana	Rest of Region 8	1.3	1.0	1.6	1	1	1	0.4	0.4	0.6	0	0	0
Guyana	Rest of Region 9	1.2	0.9	1.5	0	0	0	0.4	0.3	0.5	0	0	0
Guyana	Rising Sun / Profit	0.6	0.5	0.8	1	0	1	0.2	0.2	0.3	0	0	0
Guyana	Riverstown / Annandale	0.7	0.5	0.9	1	1	1	0.2	0.2	0.3	0	0	0
Guyana	Rose Hall	0.6	0.5	0.8	0	0	0	0.2	0.1	0.2	0	0	0
Guyana	Rosignol / Zeelust	0.6	0.4	0.8	1	0	1	0.2	0.2	0.2	0	0	0
Guyana	Sand Creek - Dadanawa, Catunarib, Sawariwau	1.3	1.0	1.6	0	0	0	0.4	0.4	0.6	0	0	0
Guyana	Soesdyke-Linden highway (including Timehri)	0.7	0.5	0.9	1	1	1	0.2	0.2	0.3	0	0	0
Guyana	Sparta / Bonasika and Rest of Essequibo Islands	0.8	0.6	1.0	0	0	0	0.2	0.2	0.3	0	0	0
Guyana	St. Cuthberts / Orange Nassau (Mahaica River)	0.6	0.5	0.9	0	0	0	0.2	0.2	0.3	0	0	0
Guyana	St. Francis Mission	0.7	0.5	0.9	0	0	0	0.3	0.2	0.3	0	0	0
Guyana	Supernaam River, Bethany and Mashabo Villages	0.7	0.6	0.9	0	0	0	0.3	0.2	0.3	0	0	0
Guyana	Tarlogie / Maida	0.6	0.4	0.8	0	0	0	0.2	0.1	0.2	0	0	0
Guyana	Te Huist Coverden / Soesdyke	0.7	0.6	1.0	0	0	0	0.2	0.1	0.3	0	0	0
Guyana	Tempe / Seafield	0.6	0.5	0.8	0	0	0	0.2	0.2	0.3	0	0	0
Guyana	Toka - Jakaretinga	1.2	1.0	1.6	0	0	0	0.4	0.3	0.5	0	0	0

Table 3: Diarrhoea Mortality by Unit *(continued)*

Country	Unit	2000 mortality mean rate (per 10,000)	2000 lower rate (per 10,000)	2000 upper rate (per 10,000)	2000 mean count	2000 lower count	2000 upper count	2017 mortality mean rate (per 10,000)	2017 lower rate (per 10,000)	2017 upper rate (per 10,000)	2017 mean count	2017 lower count	2017 upper count
Guyana	Triumph / Beterverwagting	0.7	0.5	0.9	0	0	0	0.2	0.1	0.2	0	0	0
Guyana	Uitvlugt / Tuschen	0.7	0.6	0.9	1	1	2	0.2	0.1	0.2	0	0	0
Guyana	Vereeniging / Unity	0.7	0.5	0.9	1	0	1	0.2	0.1	0.3	0	0	0
Guyana	Vergenoegen / Greenwich Park	0.7	0.6	0.9	0	0	0	0.2	0.1	0.2	0	0	0
Guyana	Waini	1.1	0.9	1.4	2	2	3	0.3	0.2	0.4	1	0	1
Guyana	Wakenaam (Essequibo Islands)	0.7	0.5	0.9	0	0	0	0.2	0.2	0.3	0	0	0
Guyana	Waramadan	1.3	1.0	1.6	0	0	0	0.4	0.3	0.5	0	0	0
Guyana	West bank Berbice	0.6	0.5	0.8	0	0	0	0.2	0.1	0.2	0	0	0
Guyana	Whim / Bloomfield	0.6	0.4	0.8	0	0	0	0.2	0.1	0.2	0	0	0
Guyana	Woodley Park / Bath	0.6	0.5	0.8	0	0	0	0.2	0.2	0.3	0	0	0
Guyana	Yakarinta - Wowetta, Surama	1.3	1.0	1.7	1	0	1	0.5	0.4	0.6	0	0	0
Guyana	Yarong Paru - Good Hope	1.3	1.0	1.6	0	0	1	0.4	0.3	0.6	0	0	0
Guyana	Zorg-en-Vlygt / Aberdeen	0.7	0.5	0.9	0	0	0	0.2	0.2	0.3	0	0	0
Haiti	Anse d'Hainault	3.8	3.3	4.4	44	38	51	2.1	1.8	2.5	30	25	34
Haiti	Aquin	4.4	3.9	5.0	133	118	150	1.8	1.6	2.1	58	50	66
Haiti	Bainet	4.7	4.1	5.3	99	87	112	2.0	1.8	2.3	34	30	38
Haiti	Belle-Anse	4.8	4.3	5.5	101	90	116	2.3	2.0	2.6	61	53	68
Haiti	Borgne	5.1	4.6	5.7	126	113	141	2.4	2.2	2.6	73	66	81
Haiti	Cerca La Source	4.8	4.4	5.5	78	70	88	2.4	2.1	2.6	45	41	50
Haiti	Corail	4.2	3.6	4.8	62	54	71	2.2	1.9	2.5	45	39	51
Haiti	Croix-des-Bouquets	4.5	4.1	5.1	251	227	282	2.1	1.8	2.3	136	121	150
Haiti	Dessalines	4.8	4.3	5.4	208	187	232	2.1	1.9	2.3	116	106	126
Haiti	Fort-Liberté	3.8	3.4	4.3	28	26	32	2.1	1.9	2.4	18	16	20
Haiti	Grande-Rivière du Nord	4.6	4.1	5.1	35	31	39	2.2	2.0	2.4	21	19	23
Haiti	Gros-Morne	4.7	4.2	5.3	119	106	135	2.2	2.0	2.5	75	68	84
Haiti	Hinche	5.8	5.2	6.4	190	169	211	2.4	2.2	2.7	97	88	106
Haiti	Jacmel	4.6	4.0	5.2	152	133	170	2.0	1.8	2.3	89	78	99
Haiti	Jérémie	3.6	3.1	4.2	90	78	105	1.9	1.6	2.1	55	47	63
Haiti	l'Acul-du-Nord	4.7	4.2	5.3	83	74	93	2.3	2.1	2.6	49	44	55
Haiti	l'Anse-à-Veau	4.6	4.0	5.1	97	86	109	1.8	1.6	2.1	48	42	54
Haiti	l'Arcahaie	4.0	3.6	4.5	110	100	123	2.0	1.8	2.2	71	63	77
Haiti	La Gonâve	3.4	3.0	3.8	46	41	51	1.8	1.6	2.0	53	47	59
Haiti	Lascahobas	5.3	4.7	6.0	129	116	146	2.5	2.2	2.7	70	63	77
Haiti	le Cap-Haïtien	4.2	3.8	4.8	138	124	157	2.2	2.0	2.5	85	76	95
Haiti	le Limbé	4.8	4.3	5.4	51	46	58	2.3	2.1	2.6	34	30	37
Haiti	le Trou-du-Nord	4.2	3.8	4.7	61	55	69	2.3	2.1	2.5	39	35	43
Haiti	Léogâne	4.1	3.6	4.6	214	191	240	1.9	1.7	2.1	143	125	159
Haiti	les Cayes	3.8	3.3	4.3	140	123	159	1.9	1.6	2.2	74	65	86
Haiti	les Chardonnières	3.6	3.1	4.1	34	30	39	2.0	1.7	2.3	21	18	24
Haiti	les Côteaux	3.5	3.0	4.0	24	21	27	1.9	1.6	2.2	14	12	16
Haiti	les Gonaïves	4.8	4.4	5.4	219	197	245	2.5	2.2	2.7	147	133	163
Haiti	Marmelade	5.4	4.8	6.0	122	109	136	2.3	2.1	2.6	67	61	73
Haiti	Miragoâne	4.5	4.0	5.1	56	50	63	1.9	1.7	2.1	29	26	33
Haiti	Mirebalais	5.8	5.2	6.5	145	130	161	2.5	2.2	2.7	77	69	84
Haiti	Môle Saint-Nicolas	4.6	4.0	5.2	117	103	132	2.0	1.7	2.2	70	61	78

Table 3: Diarrhoea Mortality by Unit *(continued)*

		2000 mortality						2017 mortality					
Country	Unit	mean rate (per 10,000)	lower rate (per 10,000)	upper rate (per 10,000)	mean count	lower count	upper count	mean rate (per 10,000)	lower rate (per 10,000)	upper rate (per 10,000)	mean count	lower count	upper count
Haiti	Ouanaminthe	4.0	3.6	4.5	72	65	82	2.2	2.0	2.5	47	43	52
Haiti	Plaisance	5.1	4.6	5.7	76	68	85	2.5	2.2	2.8	46	41	51
Haiti	Port-au-Prince	4.0	3.6	4.5	1040	930	1150	2.0	1.8	2.2	634	566	689
Haiti	Port-de-Paix	4.9	4.3	5.6	178	157	203	2.2	2.0	2.5	118	107	134
Haiti	Port-Salut	3.3	2.9	3.8	29	25	33	1.8	1.6	2.1	17	15	20
Haiti	Saint-Louis du Nord	5.1	4.5	5.8	68	60	78	2.5	2.3	2.9	48	43	53
Haiti	Saint-Marc	4.6	4.1	5.1	217	193	242	2.1	1.9	2.3	130	118	142
Haiti	Saint-Raphaël	5.0	4.5	5.6	53	48	60	2.3	2.1	2.5	29	26	32
Haiti	Vallières	4.2	3.8	4.8	43	39	49	2.1	1.9	2.3	25	22	27
Honduras	Aguaqueterique	1.1	0.8	1.4	1	1	1	0.2	0.2	0.3	0	0	0
Honduras	Ahuas	1.2	0.9	1.5	1	1	1	0.3	0.2	0.4	0	0	0
Honduras	Ajuterique	1.1	0.9	1.4	1	1	2	0.2	0.2	0.3	0	0	1
Honduras	Alauca	1.0	0.8	1.2	1	1	1	0.2	0.2	0.3	0	0	0
Honduras	Alianza	1.0	0.7	1.2	1	1	1	0.2	0.2	0.3	0	0	0
Honduras	Alubarén	0.9	0.7	1.1	1	0	1	0.2	0.2	0.3	0	0	0
Honduras	Amapala	1.0	0.8	1.2	1	1	2	0.2	0.2	0.3	0	0	0
Honduras	Apacilagua	1.0	0.8	1.2	2	1	2	0.2	0.2	0.3	0	0	0
Honduras	Arada	1.1	0.8	1.4	1	1	2	0.2	0.2	0.3	0	0	0
Honduras	Aramecina	1.0	0.7	1.2	1	1	1	0.2	0.2	0.3	0	0	0
Honduras	Arenal	1.0	0.8	1.3	1	1	1	0.2	0.2	0.3	0	0	0
Honduras	Arizona	1.1	0.8	1.3	3	2	4	0.2	0.2	0.3	1	1	1
Honduras	Atima	1.1	0.8	1.4	2	2	3	0.2	0.2	0.3	1	1	1
Honduras	Azacualpa	1.1	0.8	1.5	4	3	5	0.2	0.2	0.3	1	1	1
Honduras	Balfate	1.1	0.8	1.3	3	2	3	0.3	0.2	0.3	1	0	1
Honduras	Belen	1.1	0.9	1.4	1	1	1	0.2	0.2	0.3	0	0	0
Honduras	Belén Gualcho	1.0	0.8	1.3	2	1	2	0.2	0.2	0.3	0	0	1
Honduras	Bonito Oriental	1.1	0.8	1.4	4	3	5	0.3	0.2	0.4	1	1	2
Honduras	Brus Laguna	1.2	0.9	1.5	2	2	3	0.3	0.2	0.4	1	0	1
Honduras	Cabañas	1.2	0.9	1.5	2	1	2	0.2	0.2	0.3	1	0	1
Honduras	Cabañas	1.1	0.8	1.4	1	0	1	0.2	0.2	0.3	0	0	0
Honduras	Camasca	1.2	0.9	1.5	1	1	2	0.3	0.2	0.3	0	0	0
Honduras	Campamento	1.0	0.8	1.2	2	2	3	0.2	0.2	0.3	1	1	1
Honduras	Candelaria	1.2	0.9	1.5	1	1	1	0.3	0.2	0.4	0	0	0
Honduras	Cane	1.1	0.8	1.4	1	0	1	0.2	0.2	0.3	0	0	0
Honduras	Caridad	1.0	0.7	1.2	0	0	1	0.2	0.2	0.3	0	0	0
Honduras	Catacamas	1.1	0.8	1.3	14	11	17	0.3	0.2	0.4	5	4	6
Honduras	Cedros	0.9	0.7	1.1	2	2	3	0.2	0.1	0.3	1	0	1
Honduras	Ceguaca	1.1	0.8	1.4	1	1	1	0.2	0.2	0.3	0	0	0
Honduras	Chinacla	1.1	0.9	1.4	1	1	1	0.2	0.2	0.3	0	0	0
Honduras	Chinda	1.1	0.8	1.5	1	1	1	0.3	0.2	0.3	0	0	0
Honduras	Choloma	1.0	0.7	1.3	24	19	32	0.2	0.2	0.3	7	5	9
Honduras	Choluteca	1.0	0.8	1.3	19	15	23	0.2	0.2	0.3	5	3	6
Honduras	Cololaca	1.1	0.8	1.4	1	1	1	0.2	0.2	0.3	0	0	0
Honduras	Colomoncagua	1.2	0.9	1.5	3	2	4	0.3	0.2	0.4	1	1	1
Honduras	Comayagua	1.1	0.8	1.4	15	12	19	0.2	0.2	0.3	5	4	8
Honduras	Concepción	1.1	0.9	1.5	1	1	1	0.2	0.2	0.3	0	0	0
Honduras	Concepción	1.1	0.8	1.4	1	1	1	0.2	0.2	0.3	0	0	0
Honduras	Concepción	1.2	0.9	1.5	2	1	2	0.3	0.2	0.3	0	0	0
Honduras	Concepción de Maria	1.0	0.8	1.3	3	3	4	0.2	0.2	0.3	1	1	1
Honduras	Concepción del Norte	1.1	0.9	1.5	1	1	2	0.3	0.2	0.3	0	0	0
Honduras	Concepción del Sur	1.1	0.8	1.4	1	1	1	0.2	0.2	0.3	0	0	0
Honduras	Concordia	1.0	0.8	1.2	1	1	1	0.2	0.2	0.3	0	0	0
Honduras	Copán Ruinas	1.2	0.9	1.5	6	5	8	0.3	0.2	0.3	2	1	2
Honduras	Corquín	1.2	0.9	1.5	2	2	3	0.2	0.2	0.3	1	1	1
Honduras	Cucuyagua	1.2	0.9	1.5	2	2	3	0.2	0.2	0.3	1	0	1
Honduras	Curarén	0.9	0.7	1.1	2	1	2	0.2	0.2	0.3	0	0	1

Table 3: Diarrhoea Mortality by Unit *(continued)*

Country	Unit	2000 mortality mean rate (per 10,000)	2000 mortality lower rate (per 10,000)	2000 mortality upper rate (per 10,000)	2000 mortality mean count	2000 mortality lower count	2000 mortality upper count	2017 mortality mean rate (per 10,000)	2017 mortality lower rate (per 10,000)	2017 mortality upper rate (per 10,000)	2017 mortality mean count	2017 mortality lower count	2017 mortality upper count
Honduras	Danlí	0.9	0.7	1.1	20	16	25	0.2	0.2	0.3	6	5	9
Honduras	Distrito Central	0.9	0.7	1.1	99	77	126	0.2	0.1	0.3	27	21	37
Honduras	Dolores	1.1	0.8	1.4	1	0	1	0.2	0.2	0.3	0	0	0
Honduras	Dolores	1.1	0.8	1.5	1	1	1	0.2	0.2	0.3	0	0	0
Honduras	Dolores Merendon	1.1	0.8	1.4	0	0	0	0.2	0.2	0.3	0	0	0
Honduras	Dulce Nombre	1.2	0.9	1.5	1	1	1	0.2	0.2	0.3	0	0	0
Honduras	Dulce Nombre de Culmí	1.1	0.8	1.3	4	3	5	0.3	0.2	0.4	1	1	2
Honduras	Duyure	1.0	0.8	1.2	0	0	0	0.2	0.2	0.3	0	0	0
Honduras	El Corpus	1.0	0.8	1.3	3	3	4	0.2	0.2	0.3	1	1	1
Honduras	El Negrito	1.1	0.8	1.4	6	5	8	0.2	0.2	0.3	2	1	2
Honduras	El Nispero	1.1	0.8	1.4	1	1	2	0.2	0.2	0.3	0	0	0
Honduras	El Paraíso	1.0	0.8	1.2	5	4	7	0.2	0.2	0.3	1	1	2
Honduras	El Paraíso	1.2	0.9	1.6	3	3	4	0.3	0.2	0.3	1	1	1
Honduras	El Porvenir	1.0	0.8	1.3	2	1	2	0.2	0.2	0.3	1	0	1
Honduras	El Porvenir	0.9	0.7	1.1	2	1	2	0.2	0.2	0.3	0	0	1
Honduras	El Progreso	1.1	0.8	1.4	24	18	31	0.2	0.2	0.3	6	5	8
Honduras	El Rosario	1.1	0.9	1.4	4	3	5	0.3	0.2	0.4	1	1	2
Honduras	El Rosario	1.0	0.8	1.3	1	1	1	0.3	0.2	0.3	0	0	0
Honduras	El Triunfo	1.0	0.8	1.2	5	4	6	0.2	0.2	0.3	1	1	2
Honduras	Erandique	1.1	0.8	1.4	2	2	3	0.2	0.2	0.3	1	0	1
Honduras	Esparta	1.0	0.8	1.3	3	2	4	0.2	0.2	0.3	1	0	1
Honduras	Esquías	1.1	0.8	1.3	2	2	3	0.3	0.2	0.3	1	1	1
Honduras	Esquipulas del Norte	1.0	0.8	1.2	1	1	1	0.2	0.2	0.3	0	0	0
Honduras	Florida	1.2	0.9	1.5	5	4	7	0.3	0.2	0.3	1	1	1
Honduras	Fraternidad	1.1	0.8	1.4	0	0	0	0.2	0.2	0.3	0	0	0
Honduras	Goascorán	1.0	0.7	1.2	2	2	3	0.2	0.2	0.3	0	0	1
Honduras	Gracias	1.1	0.9	1.5	6	5	8	0.2	0.2	0.3	2	2	3
Honduras	Guaimaca	0.9	0.7	1.1	2	2	3	0.2	0.2	0.3	1	1	1
Honduras	Guajiquiro	1.1	0.8	1.4	2	2	3	0.2	0.2	0.3	0	0	1
Honduras	Gualaco	1.0	0.8	1.2	3	2	4	0.2	0.2	0.3	1	1	1
Honduras	Gualala	1.1	0.8	1.4	1	1	1	0.3	0.2	0.3	0	0	0
Honduras	Gualcince	1.1	0.9	1.5	2	2	3	0.3	0.2	0.3	1	0	1
Honduras	Guanaja	0.9	0.7	1.2	0	0	1	0.2	0.2	0.3	0	0	0
Honduras	Guarita	1.2	0.9	1.5	2	1	2	0.3	0.2	0.3	0	0	0
Honduras	Guarizama	1.0	0.8	1.2	1	1	1	0.2	0.2	0.3	0	0	0
Honduras	Guata	1.0	0.8	1.2	2	1	2	0.2	0.2	0.3	1	0	1
Honduras	Guayape	1.0	0.8	1.2	2	2	3	0.2	0.2	0.3	0	0	1
Honduras	Guinope	0.9	0.7	1.1	1	1	1	0.2	0.2	0.3	0	0	0
Honduras	Humuya	1.1	0.8	1.4	0	0	0	0.3	0.2	0.3	0	0	0
Honduras	Ilama	1.1	0.8	1.4	2	1	2	0.3	0.2	0.3	0	0	0
Honduras	Intibucá	1.1	0.8	1.4	6	5	8	0.2	0.2	0.3	2	2	3
Honduras	Iriona	1.1	0.9	1.4	3	3	4	0.3	0.2	0.4	1	1	1
Honduras	Jacaleapa	0.9	0.7	1.1	1	0	1	0.2	0.2	0.3	0	0	0
Honduras	Jano	1.0	0.8	1.2	0	0	1	0.2	0.2	0.3	0	0	0
Honduras	Jesús de Otoro	1.1	0.9	1.5	4	3	5	0.3	0.2	0.3	1	1	2
Honduras	Jocón	1.0	0.8	1.3	1	1	1	0.2	0.2	0.3	0	0	0
Honduras	José Santos Guardiola	0.9	0.7	1.1	0	0	0	0.2	0.1	0.3	0	0	0
Honduras	Juan Francisco Bulnes	1.1	0.9	1.4	1	1	1	0.3	0.2	0.4	0	0	0
Honduras	Jutiapa	1.0	0.8	1.3	4	3	5	0.2	0.2	0.3	1	1	1
Honduras	Juticalpa	1.0	0.8	1.2	15	12	18	0.2	0.2	0.3	5	4	7
Honduras	La Campa	1.1	0.8	1.4	1	1	1	0.2	0.2	0.3	0	0	0
Honduras	La Ceiba	1.0	0.8	1.2	17	14	21	0.2	0.2	0.3	6	4	8
Honduras	La Encarnación	1.1	0.8	1.4	0	0	1	0.2	0.2	0.3	0	0	0
Honduras	La Esperanza	1.0	0.8	1.3	1	1	2	0.2	0.2	0.3	0	0	1
Honduras	La Iguala	1.1	0.8	1.4	3	2	4	0.2	0.2	0.3	1	1	1

Table 3: Diarrhoea Mortality by Unit *(continued)*

		2000 mortality						2017 mortality					
Country	Unit	mean rate (per 10,000)	lower rate (per 10,000)	upper rate (per 10,000)	mean count	lower count	upper count	mean rate (per 10,000)	lower rate (per 10,000)	upper rate (per 10,000)	mean count	lower count	upper count
Honduras	La Jigua	1.1	0.8	1.5	2	2	3	0.2	0.2	0.3	1	0	1
Honduras	La Labor	1.0	0.8	1.3	1	1	1	0.2	0.2	0.3	0	0	0
Honduras	La Libertad	1.1	0.8	1.4	3	2	4	0.3	0.2	0.4	1	1	2
Honduras	La Libertad	0.9	0.7	1.1	0	0	0	0.2	0.2	0.3	0	0	0
Honduras	La Lima	1.0	0.7	1.2	8	6	11	0.2	0.2	0.3	2	2	3
Honduras	La Masica	1.0	0.8	1.3	4	3	5	0.2	0.2	0.3	1	1	1
Honduras	La Paz	1.1	0.8	1.4	5	4	6	0.2	0.2	0.3	1	1	2
Honduras	La Trinidad	1.1	0.9	1.4	1	1	1	0.3	0.2	0.4	0	0	0
Honduras	La Unión	1.1	0.9	1.5	2	1	2	0.2	0.2	0.3	0	0	1
Honduras	La Unión	1.2	0.9	1.5	2	2	3	0.2	0.2	0.3	1	1	1
Honduras	La Unión	1.0	0.8	1.3	1	1	2	0.3	0.2	0.3	0	0	0
Honduras	La Venta	0.9	0.7	1.1	1	1	1	0.2	0.2	0.3	0	0	0
Honduras	La Virtud	1.2	0.9	1.5	1	1	2	0.3	0.2	0.3	0	0	0
Honduras	Lamaní	1.0	0.8	1.3	1	1	1	0.2	0.2	0.3	0	0	0
Honduras	Langue	1.0	0.7	1.2	2	2	3	0.2	0.2	0.3	1	0	1
Honduras	Las Flores	1.2	0.9	1.5	1	1	2	0.3	0.2	0.3	0	0	0
Honduras	Las Lajas	1.1	0.8	1.4	2	1	2	0.3	0.2	0.4	1	0	1
Honduras	Las Vegas	1.1	0.8	1.4	2	2	3	0.2	0.2	0.3	1	1	1
Honduras	Lauterique	1.0	0.8	1.3	0	0	1	0.2	0.2	0.3	0	0	0
Honduras	Lejamaní	1.1	0.8	1.4	1	1	1	0.2	0.2	0.3	0	0	0
Honduras	Lepaera	1.1	0.8	1.5	6	4	7	0.2	0.2	0.3	2	1	2
Honduras	Lepaterique	0.9	0.7	1.2	2	1	2	0.2	0.2	0.3	1	0	1
Honduras	Limón	1.1	0.8	1.4	2	1	2	0.3	0.2	0.4	1	0	1
Honduras	Liure	1.0	0.8	1.2	1	1	2	0.2	0.2	0.3	0	0	0
Honduras	Lucerna	1.1	0.8	1.4	1	1	1	0.2	0.2	0.3	0	0	0
Honduras	Macuelizo	1.1	0.8	1.5	4	3	5	0.2	0.2	0.3	1	1	1
Honduras	Magdalena	1.2	0.9	1.5	1	1	1	0.3	0.2	0.3	0	0	0
Honduras	Mangulile	1.0	0.8	1.3	1	1	2	0.2	0.2	0.3	0	0	1
Honduras	Manto	1.0	0.8	1.2	2	1	2	0.2	0.2	0.3	0	0	1
Honduras	Mapulaca	1.2	0.9	1.5	0	0	1	0.3	0.2	0.3	0	0	0
Honduras	Maraita	0.8	0.7	1.1	1	1	1	0.2	0.1	0.3	0	0	0
Honduras	Marale	0.9	0.7	1.2	1	1	2	0.2	0.2	0.3	0	0	0
Honduras	Marcala	1.1	0.8	1.4	4	3	4	0.2	0.2	0.3	1	1	1
Honduras	Marcovia	1.0	0.8	1.3	6	5	8	0.2	0.2	0.3	1	1	2
Honduras	Masaguara	1.1	0.9	1.4	2	2	3	0.2	0.2	0.3	1	0	1
Honduras	Meámbar	1.1	0.8	1.4	2	1	2	0.3	0.2	0.4	1	0	1
Honduras	Mercedes	1.0	0.8	1.3	1	1	1	0.2	0.2	0.3	0	0	0
Honduras	Mercedes de Oriente	1.0	0.8	1.3	0	0	0	0.2	0.2	0.3	0	0	0
Honduras	Minas de Oro	1.1	0.8	1.4	2	1	2	0.3	0.2	0.4	1	0	1
Honduras	Morazán	1.1	0.8	1.4	6	4	7	0.2	0.2	0.3	1	1	2
Honduras	Morocelí	0.9	0.7	1.1	2	2	2	0.2	0.2	0.3	1	0	1
Honduras	Morolica	1.0	0.8	1.2	1	1	1	0.2	0.2	0.3	0	0	0
Honduras	Nacaome	1.0	0.8	1.2	8	6	10	0.2	0.2	0.3	2	1	2
Honduras	Namasigue	1.1	0.8	1.3	4	3	5	0.2	0.2	0.3	1	1	1
Honduras	Naranjito	1.1	0.8	1.5	2	1	2	0.2	0.2	0.3	0	0	1
Honduras	Nueva Arcadia	1.1	0.8	1.4	4	3	6	0.2	0.2	0.3	1	1	2
Honduras	Nueva Armenia	0.9	0.7	1.1	0	0	0	0.2	0.2	0.3	0	0	0
Honduras	Nueva Frontera	1.2	0.9	1.6	2	2	3	0.3	0.2	0.3	0	0	1
Honduras	Nuevo Celilac	1.1	0.8	1.4	2	1	2	0.2	0.2	0.3	0	0	1
Honduras	Ocotepeque	1.1	0.8	1.4	2	1	2	0.2	0.2	0.3	0	0	1
Honduras	Ojo de Agua	1.1	0.8	1.4	1	0	1	0.3	0.2	0.4	0	0	0
Honduras	Ojojona	0.8	0.7	1.1	2	1	2	0.2	0.1	0.3	0	0	1
Honduras	Olanchito	1.0	0.8	1.2	14	11	17	0.2	0.2	0.3	4	3	5
Honduras	Omoa	1.0	0.8	1.4	5	4	6	0.2	0.2	0.3	1	1	2
Honduras	Opatoro	1.1	0.9	1.4	2	1	2	0.2	0.2	0.3	0	0	0
Honduras	Orica	0.9	0.7	1.1	1	1	1	0.2	0.2	0.3	0	0	0
Honduras	Orocuina	1.0	0.8	1.3	2	2	3	0.2	0.2	0.3	0	0	1
Honduras	Oropolí	1.0	0.7	1.2	1	1	1	0.2	0.2	0.3	0	0	0
Honduras	Patuca	1.1	0.9	1.4	4	3	5	0.3	0.2	0.4	1	1	1

Table 3: Diarrhoea Mortality by Unit *(continued)*

Country	Unit	2000 mortality mean rate (per 10,000)	lower rate (per 10,000)	upper rate (per 10,000)	mean count	lower count	upper count	2017 mortality mean rate (per 10,000)	lower rate (per 10,000)	upper rate (per 10,000)	mean count	lower count	upper count
Honduras	Pespire	1.0	0.8	1.2	4	3	5	0.2	0.2	0.3	1	1	1
Honduras	Petoa	1.1	0.8	1.5	2	1	2	0.3	0.2	0.3	0	0	1
Honduras	Pimienta	1.0	0.8	1.3	2	2	3	0.2	0.2	0.3	1	1	1
Honduras	Piraera	1.1	0.9	1.5	2	2	3	0.3	0.2	0.3	1	0	1
Honduras	Potrerillos	1.0	0.8	1.3	2	2	3	0.2	0.2	0.3	1	0	1
Honduras	Potrerillos	0.9	0.7	1.1	0	0	0	0.2	0.2	0.3	0	0	0
Honduras	Protección	1.1	0.8	1.4	2	2	3	0.2	0.2	0.3	1	0	1
Honduras	Puerto Cortés	1.0	0.8	1.3	14	11	18	0.2	0.2	0.3	3	3	4
Honduras	Puerto Lempira	1.2	0.9	1.6	5	4	6	0.3	0.2	0.4	2	2	3
Honduras	Quimistán	1.1	0.8	1.4	7	5	9	0.2	0.2	0.3	2	2	3
Honduras	Ramón Villeda Morales	1.2	0.9	1.6	2	1	2	0.3	0.2	0.4	0	0	1
Honduras	Reitoca	0.9	0.7	1.1	1	1	1	0.2	0.2	0.3	0	0	0
Honduras	Roatán	0.9	0.7	1.1	2	1	2	0.2	0.2	0.3	1	1	1
Honduras	Sabá	1.0	0.8	1.3	3	3	4	0.2	0.2	0.3	1	1	1
Honduras	Sabanagrande	0.8	0.7	1.1	2	1	2	0.2	0.1	0.3	0	0	1
Honduras	Salamá	1.0	0.8	1.3	1	1	1	0.2	0.2	0.3	0	0	0
Honduras	San Agustín	1.1	0.9	1.5	1	1	1	0.2	0.2	0.3	0	0	0
Honduras	San Andrés	1.1	0.8	1.4	2	1	2	0.2	0.2	0.3	1	0	1
Honduras	San Antonio	1.1	0.9	1.4	1	1	1	0.3	0.2	0.3	0	0	0
Honduras	San Antonio	1.1	0.8	1.5	2	2	3	0.2	0.2	0.3	0	0	1
Honduras	San Antonio de Cortés	1.0	0.8	1.3	3	2	4	0.2	0.2	0.3	1	1	1
Honduras	San Antonio de Flores	1.0	0.7	1.2	1	0	1	0.2	0.2	0.3	0	0	0
Honduras	San Antonio de Flores	1.0	0.8	1.2	1	1	1	0.2	0.2	0.3	0	0	0
Honduras	San Antonio de Oriente	0.8	0.7	1.1	2	2	3	0.2	0.1	0.3	1	0	1
Honduras	San Antonio del Norte	1.1	0.8	1.4	0	0	1	0.2	0.2	0.3	0	0	0
Honduras	San Buenaventura	0.8	0.6	1.0	0	0	0	0.2	0.1	0.3	0	0	0
Honduras	San Esteban	1.0	0.8	1.2	4	3	4	0.3	0.2	0.4	1	1	1
Honduras	San Fernando	1.1	0.8	1.4	1	1	2	0.2	0.2	0.3	0	0	0
Honduras	San Francisco	1.2	0.9	1.5	2	1	2	0.3	0.2	0.3	0	0	1
Honduras	San Francisco	1.0	0.8	1.3	3	2	3	0.2	0.2	0.3	1	1	1
Honduras	San Francisco de Becerra	1.0	0.8	1.2	1	1	1	0.2	0.2	0.3	0	0	1
Honduras	San Francisco de Coray	0.9	0.7	1.2	1	1	2	0.2	0.2	0.3	0	0	0
Honduras	San Francisco de la Paz	1.0	0.8	1.2	3	2	4	0.2	0.2	0.3	1	1	1
Honduras	San Francisco de Ojuera	1.1	0.8	1.4	1	1	1	0.2	0.2	0.3	0	0	0
Honduras	San Francisco de Opalaca	1.1	0.9	1.4	2	1	2	0.3	0.2	0.3	1	0	1
Honduras	San Francisco de Yojoa	1.1	0.8	1.4	2	2	3	0.2	0.2	0.3	1	1	1
Honduras	San Francisco del Valle	1.0	0.8	1.4	1	1	2	0.2	0.2	0.3	0	0	0
Honduras	San Ignacio	0.9	0.7	1.1	1	1	1	0.2	0.2	0.3	0	0	0
Honduras	San Isidro	1.1	0.8	1.4	1	0	1	0.2	0.2	0.3	0	0	0
Honduras	San Isidro	1.0	0.8	1.2	0	0	1	0.2	0.2	0.3	0	0	0
Honduras	San Jerónimo	1.1	0.9	1.4	3	2	3	0.3	0.2	0.4	1	1	1
Honduras	San Jerónimo	1.1	0.8	1.4	1	1	1	0.2	0.2	0.3	0	0	0
Honduras	San Jorge	1.1	0.8	1.5	1	0	1	0.2	0.2	0.3	0	0	0
Honduras	San José	1.1	0.9	1.4	1	1	2	0.2	0.2	0.3	0	0	0
Honduras	San José	0.9	0.7	1.2	0	0	1	0.2	0.2	0.3	0	0	0
Honduras	San José	1.1	0.8	1.5	1	1	1	0.2	0.2	0.3	0	0	0

Table 3: Diarrhoea Mortality by Unit *(continued)*

Country	Unit	2000 mortality mean rate (per 10,000)	lower rate (per 10,000)	upper rate (per 10,000)	mean count	lower count	upper count	2017 mortality mean rate (per 10,000)	lower rate (per 10,000)	upper rate (per 10,000)	mean count	lower count	upper count
Honduras	San José de Colinas	1.1	0.8	1.4	3	2	4	0.2	0.2	0.3	1	0	1
Honduras	San José de Comayagua	1.1	0.9	1.5	1	1	2	0.3	0.2	0.4	0	0	0
Honduras	San José del Potrero	1.1	0.8	1.4	1	1	1	0.3	0.2	0.3	0	0	0
Honduras	San Juan	1.1	0.8	1.4	1	1	2	0.2	0.2	0.3	1	0	1
Honduras	San Juan	1.1	0.8	1.4	0	0	0	0.2	0.2	0.3	0	0	0
Honduras	San Juan de Flores	0.9	0.7	1.1	2	1	2	0.2	0.1	0.3	1	0	1
Honduras	San Juan de Opoa	1.2	0.9	1.5	1	1	2	0.2	0.2	0.3	0	0	1
Honduras	San Juan Guarita	1.2	0.9	1.5	0	0	1	0.3	0.2	0.3	0	0	0
Honduras	San Lorenzo	1.0	0.8	1.3	4	3	5	0.2	0.2	0.3	1	1	2
Honduras	San Lucas	0.9	0.7	1.2	1	1	1	0.2	0.2	0.3	0	0	0
Honduras	San Luis	1.1	0.8	1.3	1	1	2	0.3	0.2	0.3	0	0	1
Honduras	San Luis	1.1	0.8	1.5	4	3	6	0.3	0.2	0.3	1	1	1
Honduras	San Manuel	1.0	0.7	1.3	5	4	7	0.2	0.2	0.3	2	1	2
Honduras	San Manuel Colohete	1.1	0.8	1.4	2	2	3	0.2	0.2	0.3	1	0	1
Honduras	San Marcos	1.0	0.8	1.4	2	2	3	0.2	0.2	0.3	1	1	1
Honduras	San Marcos	1.1	0.8	1.4	2	1	2	0.2	0.2	0.3	0	0	1
Honduras	San Marcos de Caiquín	1.1	0.8	1.4	1	0	1	0.2	0.2	0.3	0	0	0
Honduras	San Marcos de Colón	0.9	0.7	1.2	3	2	4	0.2	0.2	0.3	1	1	1
Honduras	San Marcos de la Sierra	1.1	0.9	1.4	1	1	1	0.2	0.2	0.3	0	0	0
Honduras	San Matías	1.0	0.7	1.2	0	0	1	0.2	0.2	0.3	0	0	0
Honduras	San Miguelito	1.1	0.8	1.4	1	1	1	0.2	0.2	0.3	0	0	0
Honduras	San Miguelito	0.9	0.7	1.1	0	0	0	0.2	0.2	0.3	0	0	0
Honduras	San Nicolás	1.1	0.8	1.4	1	1	2	0.2	0.2	0.3	0	0	0
Honduras	San Nicolás	1.1	0.8	1.4	1	1	2	0.2	0.2	0.3	0	0	0
Honduras	San Pedro	1.2	0.9	1.5	1	1	1	0.2	0.2	0.3	0	0	0
Honduras	San Pedro de Tutule	1.1	0.8	1.4	1	1	1	0.2	0.2	0.3	0	0	0
Honduras	San Pedro Sula	1.0	0.7	1.3	77	59	102	0.2	0.2	0.3	21	16	28
Honduras	San Pedro Zacapa	1.1	0.8	1.4	1	1	2	0.3	0.2	0.3	0	0	1
Honduras	San Rafael	1.1	0.9	1.5	2	1	2	0.3	0.2	0.3	0	0	1
Honduras	San Sebastian	1.1	0.8	1.4	2	1	2	0.2	0.2	0.3	0	0	1
Honduras	San Sebastián	1.1	0.8	1.4	1	0	1	0.2	0.2	0.3	0	0	0
Honduras	San Vicente Centenario	1.1	0.8	1.4	1	0	1	0.2	0.2	0.3	0	0	0
Honduras	Santa Ana	1.1	0.9	1.4	1	1	2	0.2	0.2	0.3	0	0	0
Honduras	Santa Ana	0.8	0.7	1.1	1	1	2	0.2	0.1	0.3	0	0	1
Honduras	Santa Ana de Yusguare	1.0	0.8	1.3	1	1	2	0.2	0.2	0.3	0	0	1
Honduras	Santa Bárbara	1.1	0.8	1.4	5	4	7	0.2	0.2	0.3	2	1	2
Honduras	Santa Cruz	1.1	0.8	1.4	1	1	1	0.2	0.2	0.3	0	0	0
Honduras	Santa Cruz de Yojoa	1.0	0.8	1.3	9	7	12	0.2	0.2	0.3	3	2	3
Honduras	Santa Elena	1.1	0.8	1.3	1	1	1	0.2	0.2	0.3	0	0	1
Honduras	Santa Fé	1.1	0.8	1.4	1	0	1	0.2	0.2	0.3	0	0	0
Honduras	Santa Fé	1.1	0.8	1.3	1	1	2	0.3	0.2	0.3	0	0	0
Honduras	Santa Lucía	1.1	0.9	1.4	1	1	1	0.3	0.2	0.3	0	0	0
Honduras	Santa Lucía	0.8	0.7	1.1	2	2	3	0.2	0.1	0.3	1	1	1
Honduras	Santa María	1.1	0.8	1.4	2	1	2	0.2	0.2	0.3	0	0	1
Honduras	Santa Maria del Real	1.0	0.8	1.2	2	1	2	0.2	0.2	0.3	1	0	1
Honduras	Santa Rita	1.1	0.8	1.5	4	3	5	0.2	0.2	0.3	1	1	1

Table 3: Diarrhoea Mortality by Unit *(continued)*

Country	Unit	2000 mortality mean rate (per 10,000)	lower rate (per 10,000)	upper rate (per 10,000)	mean count	lower count	upper count	2017 mortality mean rate (per 10,000)	lower rate (per 10,000)	upper rate (per 10,000)	mean count	lower count	upper count
Honduras	Santa Rita	1.1	0.8	1.4	1	0	1	0.2	0.2	0.3	0	0	0
Honduras	Santa Rita	1.1	0.8	1.4	3	2	4	0.2	0.2	0.3	1	1	1
Honduras	Santa Rosa de Aguán	1.1	0.8	1.4	1	1	1	0.3	0.2	0.4	0	0	0
Honduras	Santa Rosa de Copán	1.1	0.9	1.5	7	5	9	0.2	0.2	0.3	2	2	3
Honduras	Santiago de Puringla	1.1	0.8	1.4	2	2	3	0.2	0.2	0.3	1	0	1
Honduras	Sensenti	1.1	0.8	1.4	1	1	2	0.2	0.2	0.3	0	0	0
Honduras	Siguatepeque	1.1	0.8	1.4	11	8	13	0.2	0.2	0.3	4	3	5
Honduras	Silca	1.0	0.8	1.2	1	1	1	0.2	0.2	0.3	0	0	0
Honduras	Sinuapa	1.0	0.8	1.3	2	1	2	0.2	0.2	0.3	1	0	1
Honduras	Soledad	1.0	0.8	1.3	1	1	2	0.2	0.2	0.3	0	0	0
Honduras	Sonaguera	1.1	0.8	1.3	7	5	8	0.3	0.2	0.3	2	1	2
Honduras	Sulaco	1.1	0.8	1.4	2	2	3	0.3	0.2	0.3	1	1	1
Honduras	Talanga	0.8	0.7	1.1	4	3	5	0.2	0.1	0.3	1	1	1
Honduras	Talgua	1.2	0.9	1.6	2	1	2	0.3	0.2	0.3	0	0	1
Honduras	Tambla	1.1	0.9	1.5	1	0	1	0.3	0.2	0.3	0	0	0
Honduras	Tatumbla	0.8	0.6	1.0	1	1	1	0.2	0.1	0.3	0	0	0
Honduras	Taulabe	1.1	0.9	1.5	3	3	5	0.3	0.2	0.4	1	1	1
Honduras	Tela	1.1	0.8	1.4	13	10	17	0.2	0.2	0.3	3	3	5
Honduras	Teupasenti	0.9	0.7	1.1	4	3	5	0.2	0.2	0.3	1	1	2
Honduras	Texiguat	1.0	0.8	1.2	1	1	2	0.2	0.2	0.3	0	0	0
Honduras	Tocoa	1.0	0.8	1.3	10	7	12	0.2	0.2	0.3	3	3	5
Honduras	Tomalá	1.2	0.9	1.5	1	0	1	0.3	0.2	0.3	0	0	0
Honduras	Trinidad	1.1	0.8	1.4	1	1	2	0.2	0.2	0.3	0	0	0
Honduras	Trinidad de Copán	1.1	0.8	1.4	3	2	4	0.2	0.2	0.3	1	1	1
Honduras	Trojes	1.1	0.8	1.3	5	4	6	0.3	0.2	0.4	2	1	2
Honduras	Trujillo	1.1	0.8	1.3	9	7	11	0.3	0.2	0.3	2	2	3
Honduras	Utila	1.0	0.8	1.2	0	0	0	0.2	0.2	0.3	0	0	0
Honduras	Vado Ancho	1.0	0.8	1.2	0	0	1	0.2	0.2	0.3	0	0	0
Honduras	Valladolid	1.2	0.9	1.5	1	1	1	0.3	0.2	0.3	0	0	0
Honduras	Valle de Ángeles	0.8	0.6	1.1	1	1	2	0.2	0.1	0.3	0	0	1
Honduras	Vallecillo	0.9	0.7	1.2	1	1	1	0.2	0.2	0.3	0	0	0
Honduras	Veracruz	1.1	0.9	1.5	1	0	1	0.2	0.2	0.3	0	0	0
Honduras	Victoria	1.1	0.8	1.4	4	3	5	0.3	0.2	0.3	1	1	1
Honduras	Villa de San Antonio	1.1	0.8	1.4	4	3	5	0.2	0.2	0.3	1	1	2
Honduras	Villa de San Francisco	0.9	0.7	1.1	1	1	1	0.2	0.1	0.3	0	0	0
Honduras	Villanueva	1.0	0.7	1.3	13	10	17	0.2	0.2	0.3	5	4	6
Honduras	Virginia	1.1	0.9	1.4	0	0	1	0.3	0.2	0.3	0	0	0
Honduras	Wampusirpi	1.1	0.9	1.4	1	1	1	0.3	0.2	0.4	0	0	0
Honduras	Yamaranguila	1.1	0.8	1.3	3	2	4	0.2	0.2	0.3	1	1	1
Honduras	Yarula	1.1	0.8	1.3	1	1	2	0.2	0.2	0.3	0	0	1
Honduras	Yauyupe	0.9	0.7	1.2	0	0	0	0.2	0.2	0.3	0	0	0
Honduras	Yocón	1.0	0.8	1.3	1	1	2	0.2	0.2	0.3	0	0	1
Honduras	Yorito	1.1	0.8	1.4	2	1	2	0.3	0.2	0.3	1	0	1
Honduras	Yoro	1.1	0.8	1.4	12	10	16	0.2	0.2	0.3	4	3	5
Honduras	Yuscarán	0.9	0.7	1.1	2	1	2	0.2	0.2	0.3	0	0	1
Jamaica	Clarendon	0.1	0.1	0.1	2	1	3	0.0	0.0	0.1	1	1	1
Jamaica	Hanover	0.1	0.1	0.1	1	0	1	0.0	0.0	0.1	0	0	0
Jamaica	Kingston	0.1	0.0	0.1	0	0	0	0.0	0.0	0.1	0	0	0
Jamaica	Manchester	0.1	0.0	0.1	1	1	2	0.0	0.0	0.1	0	0	1
Jamaica	Portland	0.1	0.1	0.1	1	0	1	0.0	0.0	0.1	0	0	0
Jamaica	Saint Andrew	0.1	0.0	0.1	3	2	4	0.0	0.0	0.1	1	1	2
Jamaica	Saint Ann	0.1	0.1	0.1	1	1	2	0.0	0.0	0.1	1	0	1
Jamaica	Saint Catherine	0.1	0.1	0.1	3	3	4	0.0	0.0	0.1	1	1	2
Jamaica	Saint Elizabeth	0.1	0.1	0.1	1	1	2	0.0	0.0	0.1	0	0	1

Table 3: Diarrhoea Mortality by Unit *(continued)*

Country	Unit	2000 mortality mean rate (per 10,000)	2000 mortality lower rate (per 10,000)	2000 mortality upper rate (per 10,000)	2000 mortality mean count	2000 mortality lower count	2000 mortality upper count	2017 mortality mean rate (per 10,000)	2017 mortality lower rate (per 10,000)	2017 mortality upper rate (per 10,000)	2017 mortality mean count	2017 mortality lower count	2017 mortality upper count
Jamaica	Saint James	0.1	0.1	0.1	1	1	2	0.0	0.0	0.1	1	0	1
Jamaica	Saint Mary	0.1	0.1	0.1	1	1	1	0.0	0.0	0.1	0	0	0
Jamaica	Saint Thomas	0.1	0.1	0.1	1	1	1	0.0	0.0	0.1	0	0	0
Jamaica	Trelawny	0.1	0.1	0.1	1	1	1	0.0	0.0	0.1	0	0	0
Jamaica	Westmoreland	0.1	0.1	0.1	1	1	2	0.0	0.0	0.1	0	0	1
Mexico	Abala	0.3	0.2	0.4	0	0	0	0.1	0.1	0.1	0	0	0
Mexico	Abasolo	0.2	0.2	0.3	0	0	0	0.1	0.0	0.1	0	0	0
Mexico	Abasolo	0.4	0.3	0.5	4	3	5	0.1	0.0	0.1	1	0	1
Mexico	Abasolo	0.1	0.1	0.1	0	0	0	0.0	0.0	0.0	0	0	0
Mexico	Abasolo	0.2	0.1	0.2	0	0	0	0.1	0.0	0.1	0	0	0
Mexico	Abejones	0.7	0.5	0.9	0	0	0	0.2	0.2	0.3	0	0	0
Mexico	Acacoyagua	1.2	0.9	1.6	2	2	3	0.3	0.2	0.4	1	1	1
Mexico	Acajete	0.5	0.4	0.6	4	3	5	0.1	0.1	0.2	2	1	2
Mexico	Acajete	0.3	0.2	0.4	0	0	1	0.1	0.0	0.1	0	0	0
Mexico	Acala	1.1	0.8	1.4	3	2	4	0.3	0.2	0.4	1	1	2
Mexico	Acambaro	0.4	0.3	0.5	4	3	5	0.1	0.0	0.1	1	0	1
Mexico	Acambay	0.3	0.2	0.4	2	2	3	0.1	0.1	0.1	1	0	1
Mexico	Acanceh	0.3	0.2	0.4	0	0	0	0.1	0.1	0.1	0	0	0
Mexico	Acapetahua	1.2	0.9	1.6	3	2	4	0.3	0.2	0.4	1	1	2
Mexico	Acaponeta	0.3	0.2	0.4	1	1	2	0.1	0.1	0.1	0	0	0
Mexico	Acapulco De Juarez	1.0	0.8	1.3	95	74	120	0.2	0.1	0.3	18	12	25
Mexico	Acateno	0.5	0.4	0.7	1	0	1	0.2	0.1	0.2	0	0	0
Mexico	Acatepec	1.1	0.8	1.4	6	5	8	0.2	0.1	0.3	1	1	2
Mexico	Acatic	0.2	0.2	0.3	1	0	1	0.1	0.0	0.1	0	0	0
Mexico	Acatlan	0.2	0.1	0.3	1	0	1	0.1	0.0	0.1	0	0	0
Mexico	Acatlan	0.3	0.2	0.4	0	0	0	0.1	0.0	0.1	0	0	0
Mexico	Acatlan	0.6	0.4	0.7	2	2	3	0.2	0.1	0.2	1	0	1
Mexico	Acatlan De Juarez	0.3	0.2	0.4	1	1	1	0.1	0.0	0.1	0	0	0
Mexico	Acatlan De Perez Figueroa	0.8	0.6	1.1	6	4	8	0.3	0.2	0.4	1	1	2
Mexico	Acatzingo	0.5	0.4	0.6	3	2	4	0.1	0.1	0.2	1	1	2
Mexico	Acaxochitlan	0.2	0.2	0.3	1	1	1	0.1	0.0	0.1	0	0	0
Mexico	Acayucan	0.4	0.3	0.5	3	3	5	0.1	0.1	0.1	1	0	1
Mexico	Acolman	0.3	0.2	0.4	3	2	4	0.1	0.1	0.1	2	1	2
Mexico	Aconchi	0.3	0.2	0.4	0	0	0	0.1	0.0	0.1	0	0	0
Mexico	Acteopan	0.5	0.4	0.6	0	0	0	0.1	0.1	0.2	0	0	0
Mexico	Actopan	0.2	0.2	0.3	1	1	1	0.1	0.0	0.1	0	0	0
Mexico	Actopan	0.4	0.3	0.5	1	1	2	0.1	0.1	0.1	0	0	0
Mexico	Acuamanala De Miguel Hidalgo	0.3	0.2	0.4	0	0	0	0.1	0.1	0.1	0	0	0
Mexico	Acuitzio	0.4	0.3	0.5	1	1	1	0.1	0.1	0.2	0	0	0
Mexico	Acula	0.4	0.3	0.6	0	0	0	0.1	0.1	0.1	0	0	0
Mexico	Aculco	0.3	0.2	0.4	2	1	2	0.1	0.1	0.1	0	0	1
Mexico	Acultzingo	0.4	0.3	0.5	1	1	1	0.1	0.1	0.1	0	0	0
Mexico	Acuna	0.2	0.2	0.3	4	3	5	0.1	0.0	0.1	1	1	1
Mexico	Agua Blanca De Iturbide	0.2	0.2	0.3	0	0	0	0.1	0.0	0.1	0	0	0
Mexico	Agua Dulce	0.4	0.3	0.5	2	2	3	0.1	0.1	0.1	0	0	0
Mexico	Agua Prieta	0.3	0.2	0.4	3	2	4	0.1	0.0	0.1	1	0	1
Mexico	Agualeguas	0.1	0.1	0.2	0	0	0	0.0	0.0	0.1	0	0	0
Mexico	Aguascalientes	0.2	0.2	0.3	18	13	23	0.1	0.1	0.1	7	5	10
Mexico	Aguililla	0.5	0.4	0.6	1	1	2	0.1	0.1	0.2	0	0	0
Mexico	Ahome	0.2	0.1	0.2	9	6	11	0.1	0.0	0.1	2	1	3
Mexico	Ahuacatlan	0.2	0.2	0.3	0	0	1	0.1	0.0	0.1	0	0	0
Mexico	Ahuacatlan	0.5	0.4	0.7	1	1	2	0.1	0.1	0.2	0	0	0
Mexico	Ahuacuotzingo	1.1	0.8	1.3	6	5	7	0.2	0.1	0.3	1	1	1
Mexico	Ahualulco	0.4	0.3	0.5	1	1	1	0.1	0.1	0.2	0	0	0
Mexico	Ahualulco De Mercado	0.3	0.2	0.3	1	0	1	0.1	0.0	0.1	0	0	0

Table 3: Diarrhoea Mortality by Unit *(continued)*

Country	Unit	2000 mortality mean rate (per 10,000)	lower rate (per 10,000)	upper rate (per 10,000)	mean count	lower count	upper count	2017 mortality mean rate (per 10,000)	lower rate (per 10,000)	upper rate (per 10,000)	mean count	lower count	upper count
Mexico	Ahuatlan	0.6	0.4	0.7	0	0	0	0.2	0.1	0.2	0	0	0
Mexico	Ahuazotepec	0.5	0.4	0.6	0	0	1	0.1	0.1	0.2	0	0	0
Mexico	Ahuehuetitla	0.6	0.4	0.8	0	0	0	0.2	0.1	0.2	0	0	0
Mexico	Ahumada	0.3	0.2	0.4	0	0	1	0.1	0.1	0.1	0	0	0
Mexico	Ajacuba	0.2	0.2	0.3	1	0	1	0.1	0.0	0.1	0	0	0
Mexico	Ajalpan	0.6	0.4	0.7	4	3	5	0.2	0.1	0.2	1	1	2
Mexico	Ajuchitlan Del Progreso	1.1	0.8	1.3	8	6	10	0.2	0.2	0.3	1	1	2
Mexico	Akil	0.4	0.3	0.5	1	0	1	0.1	0.1	0.2	0	0	0
Mexico	Alamos	0.3	0.2	0.4	1	1	1	0.1	0.1	0.1	0	0	0
Mexico	Alaquines	0.4	0.3	0.5	0	0	1	0.1	0.1	0.2	0	0	0
Mexico	Albino Zertuche	0.6	0.5	0.8	0	0	0	0.2	0.1	0.2	0	0	0
Mexico	Alcozauca De Guerrero	1.0	0.8	1.3	3	2	4	0.2	0.1	0.3	1	1	1
Mexico	Aldama	0.3	0.2	0.4	1	0	1	0.1	0.1	0.1	0	0	0
Mexico	Aldama	0.2	0.1	0.2	0	0	1	0.1	0.0	0.1	0	0	0
Mexico	Aldama	1.1	0.8	1.5	1	1	1	0.3	0.2	0.4	0	0	0
Mexico	Alfajayucan	0.2	0.2	0.3	0	0	1	0.1	0.0	0.1	0	0	0
Mexico	Aljojuca	0.5	0.4	0.7	0	0	1	0.1	0.1	0.2	0	0	0
Mexico	Allende	0.3	0.2	0.4	0	0	0	0.1	0.1	0.1	0	0	0
Mexico	Allende	0.2	0.2	0.3	0	0	1	0.1	0.0	0.1	0	0	0
Mexico	Allende	0.1	0.1	0.2	0	0	0	0.0	0.0	0.0	0	0	0
Mexico	Almoloya	0.2	0.2	0.3	0	0	1	0.1	0.0	0.1	0	0	0
Mexico	Almoloya De Alquisiras	0.4	0.3	0.5	1	1	1	0.1	0.1	0.1	0	0	0
Mexico	Almoloya De Juarez	0.3	0.3	0.4	5	4	6	0.1	0.1	0.1	2	1	2
Mexico	Almoloya Del Rio	0.4	0.3	0.4	0	0	0	0.1	0.1	0.1	0	0	0
Mexico	Alpatlahuac	0.3	0.3	0.5	1	1	1	0.1	0.0	0.1	0	0	0
Mexico	Alpoyeca	1.0	0.8	1.3	1	1	1	0.2	0.1	0.3	0	0	0
Mexico	Altamira	0.2	0.1	0.2	2	2	3	0.1	0.0	0.1	2	1	2
Mexico	Altamirano	1.2	0.8	1.5	4	3	5	0.3	0.2	0.4	1	1	2
Mexico	Altar	0.3	0.2	0.4	0	0	0	0.1	0.0	0.1	0	0	0
Mexico	Altepexi	0.5	0.4	0.7	2	2	3	0.2	0.1	0.2	0	0	1
Mexico	Alto Lucero De Gutierrez Barrios	0.4	0.3	0.5	1	1	1	0.1	0.0	0.1	0	0	0
Mexico	Altotonga	0.3	0.2	0.4	3	2	4	0.1	0.0	0.1	0	0	1
Mexico	Altzayanca	0.3	0.2	0.4	1	0	1	0.1	0.1	0.1	0	0	0
Mexico	Alvarado	0.4	0.3	0.6	2	2	3	0.1	0.1	0.1	0	0	0
Mexico	Alvaro Obregon	0.2	0.1	0.2	10	8	12	0.1	0.0	0.1	3	2	4
Mexico	Alvaro Obregon	0.4	0.3	0.5	1	1	1	0.1	0.1	0.2	0	0	0
Mexico	Amacueca	0.3	0.2	0.4	0	0	0	0.1	0.0	0.1	0	0	0
Mexico	Amacuzac	0.3	0.2	0.4	1	0	1	0.1	0.1	0.1	0	0	0
Mexico	Amanalco	0.4	0.3	0.4	1	1	1	0.1	0.1	0.1	0	0	0
Mexico	Amatan	1.2	0.9	1.6	4	3	5	0.3	0.2	0.4	1	1	1
Mexico	Amatenango De La Frontera	1.3	0.9	1.7	4	3	5	0.3	0.2	0.4	2	1	2
Mexico	Amatenango Del Valle	1.0	0.8	1.4	1	1	2	0.3	0.2	0.4	0	0	1
Mexico	Amatepec	0.4	0.3	0.5	1	1	2	0.1	0.1	0.1	0	0	0
Mexico	Amatitan	0.3	0.2	0.4	0	0	1	0.1	0.0	0.1	0	0	0
Mexico	Amatitlan	0.4	0.3	0.6	0	0	0	0.1	0.1	0.1	0	0	0
Mexico	Amatlan De Canas	0.3	0.2	0.3	0	0	0	0.1	0.0	0.1	0	0	0
Mexico	Amatlan De Los Reyes	0.4	0.3	0.5	3	2	3	0.1	0.1	0.1	0	0	1
Mexico	Amaxac De Guerrero	0.3	0.2	0.4	0	0	0	0.1	0.1	0.1	0	0	0
Mexico	Amealco De Bonfil	0.3	0.2	0.3	2	2	2	0.1	0.1	0.1	1	0	1

Table 3: Diarrhoea Mortality by Unit *(continued)*

Country	Unit	2000 mortality mean rate (per 10,000)	lower rate (per 10,000)	upper rate (per 10,000)	mean count	lower count	upper count	2017 mortality mean rate (per 10,000)	lower rate (per 10,000)	upper rate (per 10,000)	mean count	lower count	upper count
Mexico	Ameca	0.3	0.2	0.3	1	1	2	0.1	0.0	0.1	0	0	0
Mexico	Amecameca	0.3	0.3	0.4	2	2	3	0.1	0.1	0.1	0	0	1
Mexico	Amixtlan	0.5	0.4	0.7	0	0	1	0.1	0.1	0.2	0	0	0
Mexico	Amozoc	0.5	0.4	0.7	6	4	7	0.1	0.1	0.2	2	1	2
Mexico	Anahuac	0.1	0.1	0.2	0	0	0	0.0	0.0	0.0	0	0	0
Mexico	Angamacutiro	0.4	0.3	0.5	1	1	1	0.1	0.1	0.2	0	0	0
Mexico	Angangueo	0.4	0.3	0.5	0	0	0	0.1	0.1	0.2	0	0	0
Mexico	Angel Albino Corzo	1.3	1.0	1.8	6	5	9	0.3	0.3	0.4	1	1	1
Mexico	Angel R. Cabada	0.4	0.3	0.6	2	1	2	0.1	0.1	0.1	0	0	0
Mexico	Angostura	0.2	0.1	0.2	1	1	1	0.1	0.0	0.1	0	0	0
Mexico	Animas Trujano	0.7	0.5	0.9	0	0	0	0.2	0.2	0.3	0	0	0
Mexico	Antiguo Morelos	0.1	0.1	0.2	0	0	0	0.0	0.0	0.1	0	0	0
Mexico	Apan	0.2	0.2	0.3	1	1	1	0.1	0.0	0.1	0	0	0
Mexico	Apaseo El Alto	0.4	0.3	0.5	3	3	4	0.1	0.1	0.1	1	0	1
Mexico	Apaseo El Grande	0.4	0.3	0.5	4	3	5	0.1	0.0	0.1	1	1	1
Mexico	Apatzingan	0.5	0.4	0.6	8	6	10	0.1	0.1	0.2	2	1	3
Mexico	Apaxco	0.3	0.2	0.4	1	1	1	0.1	0.1	0.1	0	0	0
Mexico	Apaxtla	1.0	0.8	1.3	2	2	3	0.2	0.2	0.3	0	0	0
Mexico	Apazapan	0.4	0.3	0.5	0	0	0	0.1	0.1	0.1	0	0	0
Mexico	Apetatitlan De Antonio Carvajal	0.3	0.2	0.4	0	0	1	0.1	0.1	0.1	0	0	0
Mexico	Apizaco	0.3	0.2	0.4	2	2	3	0.1	0.1	0.1	1	0	1
Mexico	Apodaca	0.1	0.1	0.1	5	3	6	0.0	0.0	0.0	2	1	2
Mexico	Aporo	0.4	0.3	0.5	0	0	0	0.1	0.1	0.2	0	0	0
Mexico	Apozol	0.3	0.2	0.4	0	0	0	0.1	0.1	0.1	0	0	0
Mexico	Apulco	0.3	0.2	0.4	0	0	0	0.1	0.0	0.1	0	0	0
Mexico	Aquila	0.3	0.3	0.4	0	0	0	0.1	0.1	0.1	0	0	0
Mexico	Aquila	0.5	0.4	0.6	2	1	2	0.1	0.1	0.2	0	0	1
Mexico	Aquiles Serdan	0.3	0.2	0.4	0	0	0	0.1	0.1	0.1	0	0	0
Mexico	Aquismon	0.5	0.4	0.6	3	2	4	0.1	0.1	0.2	1	0	1
Mexico	Aquixtla	0.5	0.4	0.6	1	0	1	0.1	0.1	0.2	0	0	0
Mexico	Aramberri	0.1	0.1	0.2	0	0	0	0.0	0.0	0.0	0	0	0
Mexico	Arandas	0.2	0.2	0.3	2	2	3	0.1	0.0	0.1	0	0	1
Mexico	Arcelia	1.1	0.8	1.3	6	4	7	0.2	0.2	0.3	1	1	1
Mexico	Ario	0.4	0.3	0.5	2	2	3	0.1	0.1	0.2	1	0	1
Mexico	Arivechi	0.3	0.2	0.4	0	0	0	0.1	0.1	0.1	0	0	0
Mexico	Arizpe	0.3	0.2	0.4	0	0	0	0.1	0.0	0.1	0	0	0
Mexico	Armadillo De Los Infante	0.4	0.3	0.5	0	0	0	0.1	0.1	0.2	0	0	0
Mexico	Armeria	0.3	0.2	0.4	1	1	1	0.1	0.1	0.1	0	0	0
Mexico	Arriaga	1.1	0.8	1.4	4	3	6	0.3	0.2	0.4	1	1	2
Mexico	Arroyo Seco	0.3	0.2	0.4	0	0	1	0.1	0.1	0.1	0	0	0
Mexico	Arteaga	0.5	0.4	0.7	2	1	2	0.1	0.1	0.2	0	0	0
Mexico	Arteaga	0.2	0.1	0.2	1	0	1	0.1	0.0	0.1	0	0	0
Mexico	Ascension	0.3	0.2	0.4	1	1	1	0.1	0.1	0.1	0	0	0
Mexico	Asientos	0.2	0.2	0.3	1	1	2	0.1	0.1	0.1	0	0	1
Mexico	Astacinga	0.4	0.3	0.5	0	0	0	0.1	0.1	0.1	0	0	0
Mexico	Asuncion Cacalotepec	0.7	0.6	1.0	0	0	0	0.2	0.2	0.3	0	0	0
Mexico	Asuncion Cuyotepeji	0.6	0.5	0.8	0	0	0	0.2	0.1	0.3	0	0	0
Mexico	Asuncion Ixtaltepec	0.8	0.6	1.0	1	1	2	0.3	0.2	0.3	0	0	0
Mexico	Asuncion Nochixtlan	0.6	0.5	0.9	1	1	1	0.2	0.1	0.3	0	0	1
Mexico	Asuncion Ocotlan	0.7	0.5	0.9	0	0	0	0.2	0.2	0.3	0	0	0
Mexico	Asuncion Tlacolulita	0.8	0.6	1.1	0	0	0	0.3	0.2	0.3	0	0	0

Table 3: Diarrhoea Mortality by Unit *(continued)*

Country	Unit	2000 mortality						2017 mortality					
		mean rate (per 10,000)	lower rate (per 10,000)	upper rate (per 10,000)	mean count	lower count	upper count	mean rate (per 10,000)	lower rate (per 10,000)	upper rate (per 10,000)	mean count	lower count	upper count
Mexico	Atarjea	0.4	0.3	0.5	0	0	0	0.1	0.1	0.1	0	0	0
Mexico	Atemajac De Brizuela	0.3	0.2	0.4	0	0	0	0.1	0.0	0.1	0	0	0
Mexico	Atempan	0.5	0.4	0.7	2	1	2	0.1	0.1	0.2	1	0	1
Mexico	Atenango Del Rio	1.1	0.8	1.4	1	1	1	0.2	0.2	0.3	0	0	0
Mexico	Atenco	0.3	0.3	0.4	6	5	8	0.1	0.1	0.1	1	1	2
Mexico	Atengo	0.3	0.2	0.4	0	0	0	0.1	0.0	0.1	0	0	0
Mexico	Atenguillo	0.3	0.2	0.4	0	0	0	0.1	0.0	0.1	0	0	0
Mexico	Atexcal	0.6	0.4	0.7	0	0	1	0.2	0.1	0.2	0	0	0
Mexico	Atil	0.2	0.2	0.3	0	0	0	0.1	0.0	0.1	0	0	0
Mexico	Atitalaquia	0.2	0.2	0.3	1	1	1	0.1	0.0	0.1	0	0	0
Mexico	Atizapan	0.3	0.3	0.4	0	0	0	0.1	0.1	0.1	0	0	0
Mexico	Atizapan De Zaragoza	0.3	0.3	0.4	16	12	19	0.1	0.1	0.1	3	2	4
Mexico	Atlacomulco	0.3	0.2	0.4	3	2	3	0.1	0.1	0.1	1	1	1
Mexico	Atlahuilco	0.4	0.3	0.5	1	0	1	0.1	0.0	0.1	0	0	0
Mexico	Atlamajalcingo Del Monte	1.0	0.7	1.2	1	1	1	0.2	0.1	0.3	0	0	0
Mexico	Atlangatepec	0.3	0.2	0.4	0	0	0	0.1	0.1	0.1	0	0	0
Mexico	Atlapexco	0.3	0.2	0.3	1	1	1	0.1	0.0	0.1	0	0	0
Mexico	Atlatlahucan	0.3	0.2	0.3	0	0	1	0.1	0.1	0.1	0	0	0
Mexico	Atlautla	0.4	0.3	0.5	1	1	1	0.1	0.1	0.1	0	0	0
Mexico	Atlequizayan	0.5	0.4	0.7	0	0	0	0.1	0.1	0.2	0	0	0
Mexico	Atlixco	0.5	0.4	0.7	8	6	10	0.2	0.1	0.2	2	1	3
Mexico	Atlixtac	1.0	0.8	1.3	4	3	5	0.2	0.1	0.3	1	1	1
Mexico	Atolinga	0.3	0.2	0.4	0	0	0	0.1	0.0	0.1	0	0	0
Mexico	Atotonilco De Tula	0.2	0.2	0.3	1	1	1	0.1	0.0	0.1	0	0	0
Mexico	Atotonilco El Alto	0.3	0.2	0.3	2	1	2	0.1	0.0	0.1	0	0	0
Mexico	Atotonilco El Grande	0.2	0.2	0.3	1	0	1	0.1	0.0	0.1	0	0	0
Mexico	Atoyac	0.3	0.2	0.4	0	0	0	0.1	0.0	0.1	0	0	0
Mexico	Atoyac	0.4	0.3	0.5	1	1	1	0.1	0.1	0.1	0	0	0
Mexico	Atoyac De Alvarez	1.0	0.8	1.3	9	7	12	0.2	0.1	0.3	1	1	2
Mexico	Atoyatempan	0.5	0.4	0.7	0	0	1	0.1	0.1	0.2	0	0	0
Mexico	Atzacan	0.4	0.3	0.5	1	1	1	0.1	0.0	0.1	0	0	0
Mexico	Atzala	0.6	0.4	0.7	0	0	0	0.2	0.1	0.2	0	0	0
Mexico	Atzalan	0.3	0.3	0.4	3	2	4	0.1	0.1	0.1	1	0	1
Mexico	Atzitzihuacan	0.5	0.4	0.7	1	1	1	0.1	0.1	0.2	0	0	0
Mexico	Atzitzintla	0.5	0.4	0.6	1	0	1	0.1	0.1	0.2	0	0	0
Mexico	Autlan De Navarro	0.3	0.2	0.3	1	1	2	0.1	0.0	0.1	0	0	0
Mexico	Axapusco	0.3	0.2	0.4	1	1	1	0.1	0.1	0.1	0	0	0
Mexico	Axochiapan	0.3	0.3	0.4	1	1	1	0.1	0.1	0.1	0	0	0
Mexico	Axtla De Terrazas	0.5	0.4	0.6	2	2	3	0.1	0.1	0.2	0	0	1
Mexico	Axutla	0.6	0.5	0.8	0	0	0	0.2	0.1	0.2	0	0	0
Mexico	Ayahualulco	0.3	0.2	0.4	1	1	2	0.1	0.0	0.1	0	0	0
Mexico	Ayala	0.3	0.2	0.3	2	2	3	0.1	0.1	0.1	1	0	1
Mexico	Ayapango	0.4	0.3	0.4	0	0	0	0.1	0.1	0.1	0	0	0
Mexico	Ayoquezco De Aldama	0.7	0.5	0.9	0	0	1	0.2	0.2	0.3	0	0	0
Mexico	Ayotlan	0.3	0.2	0.3	1	1	1	0.1	0.0	0.1	0	0	0
Mexico	Ayotoxco De Guerrero	0.6	0.4	0.8	1	1	1	0.2	0.1	0.2	0	0	0
Mexico	Ayotzintepec	0.8	0.6	1.1	1	0	1	0.3	0.2	0.3	0	0	0
Mexico	Ayutla	0.3	0.2	0.4	0	0	1	0.1	0.0	0.1	0	0	0
Mexico	Ayutla De Los Libres	1.1	0.9	1.4	12	9	16	0.2	0.1	0.3	2	1	3

Table 3: Diarrhoea Mortality by Unit *(continued)*

Country	Unit	2000 mortality mean rate (per 10,000)	lower rate (per 10,000)	upper rate (per 10,000)	mean count	lower count	upper count	2017 mortality mean rate (per 10,000)	lower rate (per 10,000)	upper rate (per 10,000)	mean count	lower count	upper count
Mexico	Azcapotzalco	0.2	0.1	0.2	5	4	7	0.1	0.0	0.1	2	1	2
Mexico	Azoyu	1.1	0.8	1.4	2	2	3	0.2	0.1	0.3	0	0	1
Mexico	Baca	0.3	0.2	0.4	0	0	0	0.1	0.1	0.1	0	0	0
Mexico	Bacadehuachi	0.3	0.2	0.4	0	0	0	0.1	0.1	0.1	0	0	0
Mexico	Bacanora	0.3	0.2	0.4	0	0	0	0.1	0.1	0.1	0	0	0
Mexico	Bacerac	0.3	0.2	0.4	0	0	0	0.1	0.0	0.1	0	0	0
Mexico	Bachiniva	0.3	0.2	0.4	0	0	0	0.1	0.1	0.1	0	0	0
Mexico	Bacoachi	0.3	0.2	0.4	0	0	0	0.1	0.0	0.1	0	0	0
Mexico	Bacum	0.4	0.3	0.5	1	1	1	0.1	0.1	0.1	0	0	0
Mexico	Badiraguato	0.2	0.1	0.2	1	1	1	0.1	0.0	0.1	0	0	0
Mexico	Bahia De Banderas	0.3	0.2	0.4	2	2	3	0.1	0.0	0.1	1	1	2
Mexico	Balancan	0.3	0.2	0.4	2	1	3	0.1	0.1	0.2	1	1	1
Mexico	Balleza	0.3	0.2	0.4	1	1	1	0.1	0.1	0.1	0	0	0
Mexico	Banamichi	0.3	0.2	0.4	0	0	0	0.1	0.0	0.1	0	0	0
Mexico	Banderilla	0.3	0.2	0.4	1	1	2	0.1	0.0	0.1	0	0	0
Mexico	Batopilas	0.4	0.3	0.5	1	1	1	0.1	0.1	0.1	0	0	0
Mexico	Baviacora	0.3	0.2	0.4	0	0	0	0.1	0.1	0.1	0	0	0
Mexico	Bavispe	0.3	0.2	0.4	0	0	0	0.1	0.0	0.1	0	0	0
Mexico	Bejucal De Ocampo	1.1	0.8	1.5	1	0	1	0.3	0.2	0.4	0	0	0
Mexico	Bella Vista	1.1	0.8	1.5	3	2	4	0.3	0.2	0.4	1	1	1
Mexico	Benemerito De Las Americas	1.6	1.2	2.1	4	3	5	0.4	0.3	0.5	1	1	2
Mexico	Benito Juarez	0.3	0.2	0.4	19	13	25	0.1	0.1	0.1	7	5	10
Mexico	Benito Juarez	1.0	0.8	1.4	3	2	3	0.2	0.1	0.3	0	0	0
Mexico	Benito Juarez	0.4	0.3	0.5	1	1	1	0.1	0.1	0.1	0	0	0
Mexico	Benito Juarez	0.4	0.3	0.5	1	1	1	0.1	0.1	0.1	0	0	0
Mexico	Benito Juarez	0.1	0.1	0.2	4	3	5	0.1	0.0	0.1	2	1	2
Mexico	Benito Juarez	0.3	0.2	0.4	0	0	0	0.1	0.1	0.1	0	0	0
Mexico	Benito Juarez	0.3	0.2	0.4	0	0	0	0.1	0.1	0.1	0	0	0
Mexico	Benjamin Hill	0.3	0.2	0.4	0	0	0	0.1	0.0	0.1	0	0	0
Mexico	Berriozabal	1.1	0.8	1.4	5	4	6	0.3	0.2	0.4	3	2	3
Mexico	Boca Del Rio	0.4	0.3	0.5	5	4	7	0.1	0.1	0.1	1	1	1
Mexico	Bochil	1.1	0.8	1.5	5	4	6	0.3	0.2	0.4	2	1	2
Mexico	Bocoyna	0.3	0.2	0.4	1	1	2	0.1	0.1	0.1	0	0	0
Mexico	Bokoba	0.3	0.2	0.4	0	0	0	0.1	0.1	0.1	0	0	0
Mexico	Bolanos	0.3	0.2	0.4	0	0	0	0.1	0.0	0.1	0	0	0
Mexico	Brisenas	0.3	0.3	0.4	1	1	1	0.1	0.1	0.2	0	0	0
Mexico	Buctzotz	0.3	0.3	0.5	0	0	0	0.1	0.1	0.1	0	0	0
Mexico	Buenaventura	0.3	0.2	0.4	1	1	1	0.1	0.1	0.1	0	0	0
Mexico	Buenavista	0.5	0.4	0.6	2	2	3	0.1	0.1	0.2	1	1	1
Mexico	Buenavista De Cuellar	1.0	0.7	1.2	2	1	2	0.2	0.1	0.3	0	0	0
Mexico	Burgos	0.1	0.1	0.2	0	0	0	0.0	0.0	0.1	0	0	0
Mexico	Bustamante	0.1	0.1	0.2	0	0	0	0.0	0.0	0.1	0	0	0
Mexico	Bustamante	0.1	0.1	0.2	0	0	0	0.0	0.0	0.1	0	0	0
Mexico	Cabo Corrientes	0.3	0.2	0.4	0	0	0	0.1	0.0	0.1	0	0	0
Mexico	Caborca	0.3	0.2	0.4	2	2	3	0.1	0.1	0.1	1	1	1
Mexico	Cacahoatan	1.3	1.0	1.8	5	4	7	0.3	0.3	0.4	2	1	2
Mexico	Cacalchen	0.3	0.2	0.4	0	0	0	0.1	0.1	0.1	0	0	0
Mexico	Cadereyta De Montes	0.3	0.2	0.3	2	2	3	0.1	0.1	0.1	1	0	1
Mexico	Cadereyta Jimenez	0.1	0.1	0.2	1	1	1	0.0	0.0	0.0	0	0	0
Mexico	Cajeme	0.3	0.2	0.4	12	9	17	0.1	0.1	0.1	3	2	4
Mexico	Calakmul	0.3	0.2	0.4	1	1	1	0.1	0.1	0.1	0	0	0
Mexico	Calcahualco	0.4	0.3	0.5	1	0	1	0.1	0.0	0.1	0	0	0
Mexico	Calera	0.3	0.2	0.4	2	1	2	0.1	0.0	0.1	0	0	0
Mexico	Calihuala	0.7	0.5	0.9	0	0	0	0.2	0.2	0.3	0	0	0

Table 3: Diarrhoea Mortality by Unit *(continued)*

Country	Unit	2000 mortality mean rate (per 10,000)	lower rate (per 10,000)	upper rate (per 10,000)	mean count	lower count	upper count	2017 mortality mean rate (per 10,000)	lower rate (per 10,000)	upper rate (per 10,000)	mean count	lower count	upper count
Mexico	Calimaya	0.3	0.3	0.4	2	1	2	0.1	0.1	0.1	1	0	1
Mexico	Calkini	0.3	0.2	0.4	2	1	2	0.1	0.1	0.1	1	0	1
Mexico	Calnali	0.3	0.2	0.3	1	1	1	0.1	0.0	0.1	0	0	0
Mexico	Calotmul	0.3	0.3	0.5	0	0	0	0.1	0.1	0.2	0	0	0
Mexico	Calpan	0.5	0.4	0.7	1	1	1	0.1	0.1	0.2	0	0	0
Mexico	Calpulalpan	0.3	0.2	0.4	2	1	2	0.1	0.1	0.1	0	0	1
Mexico	Caltepec	0.5	0.4	0.7	0	0	0	0.1	0.1	0.2	0	0	0
Mexico	Calvillo	0.2	0.2	0.3	1	1	2	0.1	0.1	0.1	1	0	1
Mexico	Camargo	0.3	0.2	0.4	1	1	2	0.1	0.1	0.1	0	0	0
Mexico	Camargo	0.1	0.1	0.2	0	0	1	0.0	0.0	0.1	0	0	0
Mexico	Camaron De Tejeda	0.4	0.3	0.5	0	0	0	0.1	0.1	0.1	0	0	0
Mexico	Camerino Z. Mendoza	0.3	0.3	0.4	1	1	1	0.1	0.0	0.1	0	0	0
Mexico	Camocuautla	0.5	0.4	0.7	0	0	0	0.2	0.1	0.2	0	0	0
Mexico	Campeche	0.3	0.2	0.4	7	5	9	0.1	0.1	0.1	2	1	3
Mexico	Canada Morelos	0.5	0.4	0.7	1	1	2	0.2	0.1	0.2	0	0	1
Mexico	Canadas De Obregon	0.2	0.2	0.3	0	0	0	0.1	0.0	0.1	0	0	0
Mexico	Cananea	0.2	0.2	0.3	1	1	1	0.1	0.0	0.1	0	0	0
Mexico	Canatlan	0.2	0.2	0.3	1	1	1	0.1	0.0	0.1	0	0	0
Mexico	Candela	0.2	0.2	0.3	0	0	0	0.1	0.0	0.1	0	0	0
Mexico	Candelaria	0.3	0.2	0.4	2	1	2	0.1	0.1	0.1	0	0	1
Mexico	Candelaria Loxicha	0.8	0.6	1.0	1	1	1	0.2	0.2	0.3	0	0	0
Mexico	Canelas	0.3	0.2	0.4	0	0	0	0.1	0.0	0.1	0	0	0
Mexico	Canitas De Felipe Pescador	0.3	0.2	0.4	0	0	0	0.1	0.0	0.1	0	0	0
Mexico	Cansahcab	0.3	0.2	0.4	0	0	0	0.1	0.1	0.1	0	0	0
Mexico	Cantamayec	0.3	0.3	0.5	0	0	0	0.1	0.1	0.2	0	0	0
Mexico	Capulalpam De Mendez	0.7	0.5	0.9	0	0	0	0.2	0.2	0.3	0	0	0
Mexico	Capulhuac	0.3	0.3	0.4	1	1	1	0.1	0.1	0.1	0	0	0
Mexico	Caracuaro	0.5	0.4	0.6	1	1	1	0.1	0.1	0.2	0	0	0
Mexico	Carbo	0.3	0.2	0.4	0	0	0	0.1	0.0	0.1	0	0	0
Mexico	Cardenas	0.4	0.3	0.5	10	8	14	0.1	0.1	0.2	3	3	5
Mexico	Cardenas	0.4	0.3	0.5	1	1	1	0.1	0.1	0.2	0	0	0
Mexico	Cardonal	0.2	0.2	0.3	1	0	1	0.1	0.0	0.1	0	0	0
Mexico	Carichi	0.3	0.2	0.4	0	0	0	0.1	0.1	0.1	0	0	0
Mexico	Carlos A. Carrillo	0.4	0.3	0.5	1	1	2	0.1	0.1	0.1	0	0	0
Mexico	Carmen	0.3	0.2	0.4	8	5	10	0.1	0.1	0.1	2	2	3
Mexico	Carmen	0.1	0.1	0.1	0	0	0	0.0	0.0	0.0	0	0	0
Mexico	Carrillo Puerto	0.4	0.3	0.5	1	0	1	0.1	0.1	0.1	0	0	0
Mexico	Casas	0.1	0.1	0.2	0	0	0	0.1	0.0	0.1	0	0	0
Mexico	Casas Grandes	0.3	0.2	0.4	0	0	0	0.1	0.1	0.1	0	0	0
Mexico	Casimiro Castillo	0.3	0.2	0.4	1	0	1	0.1	0.0	0.1	0	0	0
Mexico	Castanos	0.2	0.1	0.3	1	0	1	0.1	0.0	0.1	0	0	0
Mexico	Castillo De Teayo	0.3	0.2	0.4	1	1	1	0.1	0.1	0.1	0	0	0
Mexico	Catazaja	1.2	0.8	1.5	2	2	3	0.3	0.2	0.4	1	0	1
Mexico	Catemaco	0.4	0.3	0.5	3	2	4	0.1	0.1	0.1	0	0	1
Mexico	Catorce	0.3	0.3	0.5	0	0	0	0.1	0.1	0.1	0	0	0
Mexico	Caxhuacan	0.6	0.4	0.7	0	0	0	0.2	0.1	0.2	0	0	0
Mexico	Cazones	0.3	0.2	0.4	1	1	1	0.1	0.1	0.1	0	0	0
Mexico	Cedral	0.3	0.3	0.4	1	1	1	0.1	0.1	0.1	0	0	0
Mexico	Celaya	0.4	0.3	0.4	18	14	22	0.1	0.0	0.1	3	2	5
Mexico	Celestun	0.3	0.2	0.4	0	0	0	0.1	0.1	0.2	0	0	0
Mexico	Cenotillo	0.3	0.3	0.4	0	0	0	0.1	0.1	0.1	0	0	0
Mexico	Centla	0.3	0.3	0.5	4	3	5	0.1	0.1	0.2	1	1	2
Mexico	Centro	0.4	0.3	0.5	21	16	28	0.1	0.1	0.2	8	6	10
Mexico	Cerralvo	0.1	0.1	0.2	0	0	0	0.0	0.0	0.1	0	0	0

Table 3: Diarrhoea Mortality by Unit *(continued)*

Country	Unit	2000 mortality mean rate (per 10,000)	lower rate (per 10,000)	upper rate (per 10,000)	mean count	lower count	upper count	2017 mortality mean rate (per 10,000)	lower rate (per 10,000)	upper rate (per 10,000)	mean count	lower count	upper count
Mexico	Cerritos	0.4	0.3	0.5	1	1	1	0.1	0.1	0.2	0	0	0
Mexico	Cerro Azul	0.4	0.3	0.5	1	1	1	0.1	0.1	0.1	0	0	0
Mexico	Cerro De San Pedro	0.4	0.3	0.5	0	0	0	0.1	0.1	0.1	0	0	0
Mexico	Chacaltianguis	0.4	0.3	0.6	1	0	1	0.1	0.1	0.1	0	0	0
Mexico	Chacsinkin	0.4	0.3	0.5	0	0	0	0.1	0.1	0.2	0	0	0
Mexico	Chahuites	0.8	0.6	1.1	0	0	1	0.3	0.2	0.4	0	0	0
Mexico	Chalcatongo De Hidalgo	0.7	0.5	0.8	1	1	1	0.2	0.1	0.3	0	0	0
Mexico	Chalchicomula De Sesma	0.5	0.4	0.6	3	2	4	0.1	0.1	0.2	1	1	1
Mexico	Chalchihuitan	1.2	0.9	1.5	3	2	4	0.3	0.2	0.4	1	1	1
Mexico	Chalchihuites	0.3	0.2	0.4	0	0	1	0.1	0.0	0.1	0	0	0
Mexico	Chalco	0.3	0.3	0.4	13	10	16	0.1	0.1	0.1	4	2	5
Mexico	Chalma	0.4	0.3	0.5	1	1	1	0.1	0.1	0.1	0	0	0
Mexico	Champoton	0.3	0.2	0.4	3	2	4	0.1	0.1	0.1	1	1	1
Mexico	Chamula	1.0	0.8	1.4	11	8	15	0.3	0.2	0.4	5	4	7
Mexico	Chanal	1.1	0.8	1.4	2	1	2	0.3	0.2	0.4	1	1	1
Mexico	Chankom	0.3	0.2	0.4	0	0	0	0.1	0.1	0.1	0	0	0
Mexico	Chapa De Mota	0.3	0.3	0.4	1	1	1	0.1	0.1	0.1	0	0	1
Mexico	Chapab	0.3	0.3	0.5	0	0	0	0.1	0.1	0.2	0	0	0
Mexico	Chapala	0.3	0.2	0.3	2	1	2	0.1	0.0	0.1	0	0	0
Mexico	Chapantongo	0.2	0.2	0.3	0	0	0	0.1	0.0	0.1	0	0	0
Mexico	Chapulco	0.5	0.4	0.7	1	0	1	0.1	0.1	0.2	0	0	0
Mexico	Chapulhuacan	0.3	0.2	0.3	1	1	1	0.1	0.0	0.1	0	0	0
Mexico	Chapultenango	1.2	0.9	1.5	2	1	2	0.3	0.2	0.4	0	0	0
Mexico	Chapultepec	0.3	0.3	0.4	0	0	0	0.1	0.1	0.1	0	0	0
Mexico	Charapan	0.4	0.3	0.5	1	0	1	0.1	0.1	0.2	0	0	0
Mexico	Charcas	0.4	0.3	0.5	1	1	1	0.1	0.1	0.1	0	0	0
Mexico	Charo	0.4	0.3	0.5	1	1	1	0.1	0.1	0.2	0	0	0
Mexico	Chavinda	0.4	0.3	0.5	0	0	1	0.1	0.1	0.2	0	0	0
Mexico	Chemax	0.3	0.2	0.4	1	1	2	0.1	0.1	0.1	1	0	1
Mexico	Chenalho	1.2	0.9	1.5	7	5	9	0.3	0.2	0.4	2	2	3
Mexico	Cheran	0.4	0.3	0.5	1	1	1	0.1	0.1	0.2	0	0	0
Mexico	Chiapa De Corzo	1.1	0.8	1.4	9	6	12	0.3	0.2	0.4	4	3	5
Mexico	Chiapilla	1.2	0.9	1.6	1	1	1	0.3	0.2	0.4	0	0	0
Mexico	Chiautempan	0.3	0.2	0.4	3	2	4	0.1	0.1	0.1	1	0	1
Mexico	Chiautla	0.3	0.2	0.4	1	1	1	0.1	0.1	0.1	0	0	1
Mexico	Chiautla	0.6	0.4	0.7	1	1	2	0.2	0.1	0.2	0	0	0
Mexico	Chiautzingo	0.5	0.4	0.7	1	1	2	0.2	0.1	0.2	0	0	1
Mexico	Chichimila	0.3	0.2	0.4	0	0	0	0.1	0.1	0.1	0	0	0
Mexico	Chichiquila	0.5	0.4	0.7	2	2	3	0.1	0.1	0.2	0	0	1
Mexico	Chicoasen	1.1	0.8	1.4	1	1	1	0.3	0.2	0.4	0	0	0
Mexico	Chicoloapan	0.3	0.3	0.4	8	6	10	0.1	0.1	0.1	2	1	2
Mexico	Chicomuselo	1.2	0.9	1.7	4	3	6	0.3	0.2	0.4	2	1	2
Mexico	Chiconamel	0.4	0.3	0.5	0	0	0	0.1	0.1	0.1	0	0	0
Mexico	Chiconcuac	0.3	0.2	0.4	0	0	1	0.1	0.1	0.1	0	0	0
Mexico	Chiconcuautla	0.5	0.4	0.7	1	1	2	0.1	0.1	0.2	0	0	0
Mexico	Chiconquiaco	0.3	0.2	0.4	1	1	1	0.1	0.0	0.1	0	0	0
Mexico	Chicontepec	0.4	0.3	0.5	2	2	3	0.1	0.1	0.1	0	0	0
Mexico	Chicxulub Pueblo	0.3	0.2	0.4	0	0	0	0.1	0.1	0.1	0	0	0
Mexico	Chietla	0.5	0.4	0.7	3	2	3	0.1	0.1	0.2	1	0	1
Mexico	Chigmecatitlan	0.6	0.4	0.7	0	0	0	0.2	0.1	0.2	0	0	0
Mexico	Chignahuapan	0.5	0.3	0.6	3	2	4	0.1	0.1	0.2	1	1	1
Mexico	Chignautla	0.5	0.4	0.7	2	2	3	0.1	0.1	0.2	1	0	1
Mexico	Chihuahua	0.3	0.2	0.4	21	15	28	0.1	0.1	0.1	7	5	10
Mexico	Chikindzonot	0.3	0.3	0.4	0	0	0	0.1	0.1	0.2	0	0	0
Mexico	Chila	0.6	0.4	0.7	0	0	1	0.1	0.1	0.2	0	0	0
Mexico	Chila De La Sal	0.6	0.5	0.8	0	0	0	0.2	0.1	0.2	0	0	0
Mexico	Chilapa De Alvarez	1.0	0.8	1.2	17	14	22	0.2	0.1	0.3	4	3	5

Table 3: Diarrhoea Mortality by Unit *(continued)*

Country	Unit	2000 mortality mean rate (per 10,000)	lower rate (per 10,000)	upper rate (per 10,000)	mean count	lower count	upper count	2017 mortality mean rate (per 10,000)	lower rate (per 10,000)	upper rate (per 10,000)	mean count	lower count	upper count
Mexico	Chilchota	0.4	0.3	0.5	2	1	2	0.1	0.1	0.2	1	0	1
Mexico	Chilchotla	0.5	0.3	0.6	1	1	2	0.1	0.1	0.2	0	0	1
Mexico	Chilcuautla	0.2	0.2	0.3	1	0	1	0.1	0.0	0.1	0	0	0
Mexico	Chilon	1.2	0.9	1.5	19	14	25	0.3	0.2	0.4	6	5	8
Mexico	Chilpancingo De Los Bravo	1.0	0.7	1.2	26	21	33	0.2	0.1	0.3	6	4	8
Mexico	Chimalhuacan	0.3	0.3	0.4	13	10	17	0.1	0.1	0.1	4	3	6
Mexico	Chimaltitan	0.3	0.2	0.4	0	0	0	0.1	0.0	0.1	0	0	0
Mexico	China	0.1	0.1	0.2	0	0	0	0.0	0.0	0.0	0	0	0
Mexico	Chinameca	0.4	0.3	0.6	1	1	1	0.1	0.1	0.1	0	0	0
Mexico	Chinampa De Gorostiza	0.4	0.3	0.5	1	1	1	0.1	0.1	0.1	0	0	0
Mexico	Chinantla	0.6	0.5	0.8	0	0	0	0.2	0.1	0.2	0	0	0
Mexico	Chinicuila	0.5	0.3	0.6	0	0	1	0.1	0.1	0.2	0	0	0
Mexico	Chinipas	0.4	0.3	0.5	0	0	0	0.1	0.1	0.1	0	0	0
Mexico	Chiquihuitlan De Benito Juarez	0.8	0.6	1.0	0	0	0	0.2	0.2	0.3	0	0	0
Mexico	Chiquilistlan	0.3	0.2	0.4	0	0	0	0.1	0.0	0.1	0	0	0
Mexico	Chocaman	0.4	0.3	0.5	1	1	1	0.1	0.1	0.1	0	0	0
Mexico	Chochola	0.3	0.2	0.4	0	0	0	0.1	0.1	0.1	0	0	0
Mexico	Choix	0.2	0.1	0.2	1	0	1	0.1	0.0	0.1	0	0	0
Mexico	Chontla	0.4	0.3	0.5	1	1	1	0.1	0.1	0.1	0	0	0
Mexico	Chucandiro	0.4	0.3	0.5	0	0	0	0.1	0.1	0.2	0	0	0
Mexico	Chumatlan	0.4	0.3	0.5	0	0	0	0.1	0.1	0.1	0	0	0
Mexico	Chumayel	0.3	0.2	0.5	0	0	0	0.1	0.1	0.2	0	0	0
Mexico	Churintzio	0.4	0.3	0.5	0	0	0	0.1	0.1	0.2	0	0	0
Mexico	Churumuco	0.5	0.4	0.6	1	1	2	0.1	0.1	0.2	0	0	0
Mexico	Cienega De Flores	0.1	0.1	0.1	0	0	0	0.0	0.0	0.0	1	0	1
Mexico	Cienega De Zimatlan	0.7	0.5	0.9	0	0	0	0.2	0.2	0.3	0	0	0
Mexico	Cihuatlan	0.3	0.2	0.4	1	1	1	0.1	0.0	0.1	0	0	0
Mexico	Cintalapa	1.0	0.8	1.3	9	7	12	0.3	0.2	0.4	3	2	4
Mexico	Citlaltepetl	0.4	0.3	0.5	1	1	1	0.1	0.1	0.1	0	0	0
Mexico	Ciudad Del Maiz	0.4	0.3	0.5	2	1	2	0.1	0.1	0.2	0	0	1
Mexico	Ciudad Fernandez	0.4	0.3	0.5	2	1	2	0.1	0.1	0.2	1	0	1
Mexico	Ciudad Ixtepec	0.7	0.5	1.0	2	1	2	0.2	0.2	0.3	1	0	1
Mexico	Ciudad Madero	0.2	0.1	0.2	2	2	3	0.1	0.0	0.1	1	0	1
Mexico	Ciudad Valles	0.5	0.4	0.6	8	6	11	0.1	0.1	0.2	2	1	3
Mexico	Coacalco De Berriozabal	0.3	0.2	0.4	9	7	11	0.1	0.1	0.1	2	1	2
Mexico	Coacoatzintla	0.3	0.3	0.5	0	0	1	0.1	0.0	0.1	0	0	0
Mexico	Coahuayana	0.5	0.4	0.6	1	0	1	0.1	0.1	0.2	0	0	0
Mexico	Coahuayutla De Jose Maria Izazaga	1.1	0.8	1.4	3	2	4	0.2	0.2	0.3	0	0	0
Mexico	Coahuitlan	0.4	0.3	0.5	0	0	0	0.1	0.1	0.1	0	0	0
Mexico	Coalcoman De Vazquez Pallares	0.5	0.3	0.6	2	1	2	0.1	0.1	0.2	0	0	0
Mexico	Coapilla	1.1	0.8	1.5	1	1	2	0.3	0.2	0.4	0	0	1
Mexico	Coatecas Altas	0.7	0.5	0.9	0	0	1	0.2	0.2	0.3	0	0	0
Mexico	Coatepec	0.6	0.4	0.7	0	0	0	0.2	0.1	0.2	0	0	0
Mexico	Coatepec	0.3	0.3	0.5	3	2	4	0.1	0.0	0.1	1	0	1
Mexico	Coatepec Harinas	0.4	0.3	0.4	1	1	2	0.1	0.1	0.1	0	0	1
Mexico	Coatlan Del Rio	0.3	0.2	0.4	0	0	0	0.1	0.1	0.1	0	0	0
Mexico	Coatzacoalcos	0.4	0.3	0.5	11	8	15	0.1	0.1	0.1	2	2	3
Mexico	Coatzingo	0.6	0.4	0.7	0	0	0	0.2	0.1	0.2	0	0	0
Mexico	Coatzintla	0.3	0.3	0.4	2	2	3	0.1	0.0	0.1	0	0	0
Mexico	Cochoapa El Grande	1.0	0.8	1.3	3	3	4	0.2	0.1	0.3	1	1	1

Table 3: Diarrhoea Mortality by Unit *(continued)*

Country	Unit	2000 mortality mean rate (per 10,000)	lower rate (per 10,000)	upper rate (per 10,000)	mean count	lower count	upper count	2017 mortality mean rate (per 10,000)	lower rate (per 10,000)	upper rate (per 10,000)	mean count	lower count	upper count
Mexico	Cocotitlan	0.3	0.2	0.4	1	0	1	0.1	0.1	0.1	0	0	0
Mexico	Cocula	1.1	0.8	1.3	2	1	2	0.2	0.2	0.3	0	0	0
Mexico	Cocula	0.3	0.2	0.3	1	1	1	0.1	0.0	0.1	0	0	0
Mexico	Coeneo	0.4	0.3	0.5	1	1	1	0.1	0.1	0.2	0	0	0
Mexico	Coetzala	0.4	0.3	0.5	0	0	0	0.1	0.1	0.1	0	0	0
Mexico	Cohetzala	0.6	0.5	0.8	0	0	0	0.2	0.1	0.2	0	0	0
Mexico	Cohuecan	0.5	0.4	0.6	0	0	0	0.1	0.1	0.2	0	0	0
Mexico	Coicoyan De Las Flores	0.7	0.5	0.9	1	1	1	0.2	0.2	0.3	0	0	0
Mexico	Cojumatlan De Regules	0.4	0.3	0.5	0	0	1	0.1	0.1	0.2	0	0	0
Mexico	Colima	0.2	0.2	0.3	4	3	5	0.1	0.1	0.1	1	1	1
Mexico	Colipa	0.4	0.3	0.5	0	0	0	0.1	0.1	0.1	0	0	0
Mexico	Colon	0.2	0.2	0.3	2	1	2	0.1	0.1	0.1	1	0	1
Mexico	Colotlan	0.2	0.2	0.3	1	0	1	0.1	0.0	0.1	0	0	0
Mexico	Comala	0.2	0.2	0.3	1	0	1	0.1	0.0	0.1	0	0	0
Mexico	Comalcalco	0.3	0.3	0.5	8	6	10	0.1	0.1	0.2	3	2	3
Mexico	Comapa	0.4	0.3	0.5	1	1	1	0.1	0.1	0.1	0	0	0
Mexico	Comitan De Dominguez	1.0	0.8	1.4	15	11	20	0.3	0.2	0.4	6	4	7
Mexico	Comondu	0.2	0.1	0.3	1	1	2	0.1	0.0	0.1	0	0	1
Mexico	Comonfort	0.4	0.3	0.5	4	3	5	0.1	0.0	0.1	1	0	1
Mexico	Compostela	0.3	0.2	0.4	2	2	3	0.1	0.0	0.1	1	0	1
Mexico	Concepcion Buenavista	0.6	0.5	0.8	0	0	0	0.2	0.1	0.3	0	0	0
Mexico	Concepcion De Buenos Aires	0.3	0.2	0.3	0	0	0	0.1	0.0	0.1	0	0	0
Mexico	Concepcion Del Oro	0.3	0.2	0.4	0	0	1	0.1	0.0	0.1	0	0	0
Mexico	Concepcion Papalo	0.7	0.5	0.9	0	0	0	0.2	0.2	0.3	0	0	0
Mexico	Concordia	0.2	0.1	0.2	1	0	1	0.0	0.0	0.1	0	0	0
Mexico	Coneto De Comonfort	0.3	0.2	0.3	0	0	0	0.1	0.0	0.1	0	0	0
Mexico	Conkal	0.3	0.2	0.4	0	0	0	0.1	0.1	0.1	0	0	0
Mexico	Constancia Del Rosario	0.7	0.6	1.0	1	1	1	0.2	0.2	0.3	0	0	0
Mexico	Contepec	0.4	0.3	0.5	2	1	2	0.1	0.1	0.2	0	0	1
Mexico	Contla De Juan Cuamatzi	0.3	0.2	0.4	1	1	1	0.1	0.1	0.1	0	0	0
Mexico	Copainala	1.1	0.8	1.5	3	2	4	0.3	0.2	0.4	1	1	1
Mexico	Copala	1.1	0.8	1.4	2	2	3	0.2	0.1	0.3	0	0	1
Mexico	Copalillo	1.1	0.8	1.3	3	2	3	0.2	0.1	0.3	0	0	1
Mexico	Copanatoyac	1.0	0.8	1.3	3	2	4	0.2	0.1	0.3	1	0	1
Mexico	Copandaro	0.4	0.3	0.5	1	1	1	0.1	0.1	0.2	0	0	0
Mexico	Coquimatlan	0.3	0.2	0.3	1	1	1	0.1	0.1	0.1	0	0	0
Mexico	Cordoba	0.3	0.3	0.5	6	5	9	0.1	0.1	0.1	1	1	2
Mexico	Coronado	0.3	0.2	0.4	0	0	0	0.1	0.1	0.1	0	0	0
Mexico	Coronango	0.5	0.3	0.6	2	1	2	0.2	0.1	0.2	1	1	1
Mexico	Coroneo	0.4	0.3	0.5	0	0	0	0.1	0.0	0.1	0	0	0
Mexico	Corregidora	0.2	0.2	0.3	4	3	4	0.1	0.1	0.1	2	1	2
Mexico	Cortazar	0.4	0.3	0.5	4	3	5	0.1	0.0	0.1	1	0	1
Mexico	Cosala	0.2	0.1	0.2	1	0	1	0.1	0.0	0.1	0	0	0
Mexico	Cosamaloapan De Carpio	0.4	0.3	0.6	2	2	3	0.1	0.1	0.1	0	0	1
Mexico	Cosautlan De Carvajal	0.4	0.3	0.5	1	0	1	0.1	0.1	0.1	0	0	0
Mexico	Coscomatepec	0.3	0.2	0.4	3	2	4	0.1	0.0	0.1	0	0	1
Mexico	Cosio	0.2	0.2	0.3	0	0	1	0.1	0.1	0.1	0	0	0
Mexico	Cosolapa	0.7	0.6	1.0	1	1	2	0.2	0.2	0.3	0	0	0

Table 3: Diarrhoea Mortality by Unit *(continued)*

Country	Unit	2000 mortality mean rate (per 10,000)	lower rate (per 10,000)	upper rate (per 10,000)	mean count	lower count	upper count	2017 mortality mean rate (per 10,000)	lower rate (per 10,000)	upper rate (per 10,000)	mean count	lower count	upper count
Mexico	Cosoleacaque	0.4	0.3	0.5	7	5	9	0.1	0.1	0.1	1	1	1
Mexico	Cosoltepec	0.6	0.5	0.8	0	0	0	0.2	0.2	0.3	0	0	0
Mexico	Cotaxtla	0.4	0.3	0.5	1	1	1	0.1	0.1	0.1	0	0	0
Mexico	Cotija	0.4	0.3	0.5	1	1	1	0.1	0.1	0.2	0	0	0
Mexico	Coxcatlan	0.5	0.4	0.6	1	1	1	0.1	0.1	0.2	0	0	0
Mexico	Coxcatlan	0.6	0.4	0.8	2	1	2	0.2	0.1	0.2	0	0	0
Mexico	Coxquihui	0.4	0.3	0.5	1	1	1	0.1	0.1	0.1	0	0	0
Mexico	Coyame Del Sotol	0.3	0.2	0.4	0	0	0	0.1	0.1	0.1	0	0	0
Mexico	Coyoacan	0.1	0.1	0.2	8	7	11	0.1	0.0	0.1	3	2	4
Mexico	Coyomeapan	0.6	0.5	0.8	1	1	2	0.2	0.1	0.2	0	0	0
Mexico	Coyotepec	0.3	0.2	0.4	2	1	2	0.1	0.1	0.1	1	0	1
Mexico	Coyotepec	0.6	0.4	0.7	0	0	0	0.2	0.1	0.2	0	0	0
Mexico	Coyuca De Benitez	1.1	0.9	1.4	10	8	13	0.2	0.1	0.3	2	1	3
Mexico	Coyuca De Catalan	1.0	0.8	1.3	9	7	11	0.2	0.1	0.3	1	1	2
Mexico	Coyutla	0.4	0.3	0.5	1	1	1	0.1	0.1	0.1	0	0	0
Mexico	Cozumel	0.3	0.2	0.4	2	2	3	0.1	0.1	0.1	1	0	1
Mexico	Cruillas	0.1	0.1	0.2	0	0	0	0.0	0.0	0.1	0	0	0
Mexico	Cuajimalpa De Morelos	0.2	0.1	0.2	2	2	3	0.1	0.0	0.1	1	1	1
Mexico	Cuajinicuilapa	1.1	0.8	1.4	5	4	6	0.2	0.1	0.3	1	1	1
Mexico	Cualac	1.0	0.8	1.3	1	1	2	0.2	0.1	0.3	0	0	0
Mexico	Cuapiaxtla	0.3	0.2	0.4	0	0	1	0.1	0.1	0.1	0	0	0
Mexico	Cuapiaxtla De Madero	0.5	0.4	0.6	1	1	1	0.1	0.1	0.2	0	0	0
Mexico	Cuatrocienegas	0.2	0.1	0.3	0	0	0	0.1	0.0	0.1	0	0	0
Mexico	Cuauhtemoc	0.3	0.2	0.4	4	3	5	0.1	0.1	0.1	1	1	2
Mexico	Cuauhtemoc	0.2	0.2	0.3	1	1	1	0.1	0.0	0.1	0	0	0
Mexico	Cuauhtemoc	0.1	0.1	0.2	6	5	8	0.1	0.0	0.1	3	2	3
Mexico	Cuauhtemoc	0.3	0.2	0.4	1	0	1	0.1	0.0	0.1	0	0	0
Mexico	Cuautempan	0.5	0.4	0.7	0	0	1	0.1	0.1	0.2	0	0	0
Mexico	Cuautepec	1.1	0.9	1.4	2	2	3	0.2	0.1	0.3	0	0	0
Mexico	Cuautepec De Hinojosa	0.2	0.2	0.3	2	1	2	0.1	0.0	0.1	0	0	1
Mexico	Cuautinchan	0.5	0.4	0.7	1	1	1	0.2	0.1	0.2	0	0	1
Mexico	Cuautitlan	0.3	0.2	0.4	3	2	3	0.1	0.1	0.1	1	1	1
Mexico	Cuautitlan De Garcia Barragan	0.3	0.2	0.4	1	0	1	0.1	0.0	0.1	0	0	0
Mexico	Cuautitlan Izcalli	0.3	0.3	0.4	18	14	23	0.1	0.1	0.1	5	3	7
Mexico	Cuautla	0.3	0.2	0.4	0	0	0	0.1	0.0	0.1	0	0	0
Mexico	Cuautla	0.3	0.2	0.3	4	3	5	0.1	0.1	0.1	1	1	2
Mexico	Cuautlancingo	0.5	0.4	0.6	3	3	4	0.2	0.1	0.2	2	1	3
Mexico	Cuaxomulco	0.3	0.2	0.4	0	0	0	0.1	0.1	0.1	0	0	0
Mexico	Cuayuca De Andrade	0.6	0.4	0.8	0	0	0	0.2	0.1	0.2	0	0	0
Mexico	Cucurpe	0.3	0.2	0.4	0	0	0	0.1	0.0	0.1	0	0	0
Mexico	Cuencame	0.2	0.2	0.3	1	1	1	0.1	0.0	0.1	0	0	0
Mexico	Cueramaro	0.4	0.3	0.5	1	1	2	0.1	0.1	0.1	0	0	0
Mexico	Cuernavaca	0.3	0.2	0.4	9	7	11	0.1	0.1	0.1	2	2	3
Mexico	Cuetzala Del Progreso	1.0	0.8	1.2	2	1	2	0.2	0.1	0.3	0	0	0
Mexico	Cuetzalan Del Progreso	0.6	0.4	0.7	4	3	5	0.1	0.1	0.2	1	1	1
Mexico	Cuichapa	0.4	0.3	0.5	0	0	0	0.1	0.1	0.1	0	0	0
Mexico	Cuilapam De Guerrero	0.6	0.5	0.8	2	1	2	0.2	0.2	0.3	1	0	1
Mexico	Cuitlahuac	0.4	0.3	0.5	1	1	2	0.1	0.1	0.1	0	0	0
Mexico	Cuitzeo	0.4	0.3	0.5	2	1	2	0.1	0.1	0.2	0	0	1
Mexico	Culiacan	0.2	0.1	0.2	16	12	20	0.0	0.0	0.1	4	3	6

Table 3: Diarrhoea Mortality by Unit *(continued)*

Country	Unit	2000 mortality						2017 mortality					
		mean rate (per 10,000)	lower rate (per 10,000)	upper rate (per 10,000)	mean count	lower count	upper count	mean rate (per 10,000)	lower rate (per 10,000)	upper rate (per 10,000)	mean count	lower count	upper count
Mexico	Cumpas	0.3	0.2	0.4	0	0	0	0.1	0.1	0.1	0	0	0
Mexico	Cuncunul	0.3	0.2	0.4	0	0	0	0.1	0.1	0.1	0	0	0
Mexico	Cunduacan	0.4	0.3	0.5	6	4	8	0.1	0.1	0.2	2	1	3
Mexico	Cuquio	0.3	0.2	0.3	0	0	1	0.1	0.0	0.1	0	0	0
Mexico	Cusihuiriachi	0.3	0.2	0.4	0	0	0	0.1	0.1	0.1	0	0	0
Mexico	Cutzamala De Pinzon	1.1	0.8	1.3	4	3	5	0.2	0.2	0.3	1	0	1
Mexico	Cuyamecalco Villa De Zaragoza	0.8	0.6	1.1	1	0	1	0.3	0.2	0.3	0	0	0
Mexico	Cuyoaco	0.5	0.4	0.6	1	1	1	0.1	0.1	0.2	0	0	0
Mexico	Cuzama	0.3	0.2	0.4	0	0	0	0.1	0.1	0.1	0	0	0
Mexico	Degollado	0.2	0.2	0.3	1	0	1	0.1	0.0	0.1	0	0	0
Mexico	Del Nayar	0.3	0.2	0.4	2	1	2	0.1	0.0	0.1	0	0	1
Mexico	Delicias	0.3	0.2	0.4	3	2	4	0.1	0.1	0.1	1	1	2
Mexico	Divisaderos	0.3	0.2	0.4	0	0	0	0.1	0.1	0.1	0	0	0
Mexico	Doctor Mora	0.4	0.3	0.5	1	1	2	0.1	0.0	0.1	0	0	0
Mexico	Dolores Hidalgo Cuna De La Independenc	0.4	0.3	0.5	8	6	10	0.1	0.0	0.1	1	1	2
Mexico	Domingo Arenas	0.5	0.4	0.7	0	0	0	0.2	0.1	0.2	0	0	0
Mexico	Donato Guerra	0.4	0.3	0.4	1	1	2	0.1	0.1	0.1	0	0	0
Mexico	Dr. Arroyo	0.1	0.1	0.1	0	0	1	0.0	0.0	0.0	0	0	0
Mexico	Dr. Belisario Dominguez	0.3	0.2	0.4	0	0	0	0.1	0.1	0.1	0	0	0
Mexico	Dr. Coss	0.1	0.1	0.2	0	0	0	0.0	0.0	0.1	0	0	0
Mexico	Dr. Gonzalez	0.1	0.1	0.2	0	0	0	0.0	0.0	0.0	0	0	0
Mexico	Durango	0.2	0.2	0.3	15	11	19	0.1	0.0	0.1	4	3	6
Mexico	Dzan	0.4	0.3	0.5	0	0	0	0.1	0.1	0.2	0	0	0
Mexico	Dzemul	0.3	0.2	0.4	0	0	0	0.1	0.1	0.1	0	0	0
Mexico	Dzidzantun	0.3	0.2	0.4	0	0	0	0.1	0.1	0.1	0	0	0
Mexico	Dzilam De Bravo	0.3	0.3	0.5	0	0	0	0.1	0.1	0.2	0	0	0
Mexico	Dzilam Gonzalez	0.3	0.2	0.4	0	0	0	0.1	0.1	0.1	0	0	0
Mexico	Dzitas	0.3	0.3	0.4	0	0	0	0.1	0.1	0.1	0	0	0
Mexico	Dzoncauich	0.3	0.2	0.4	0	0	0	0.1	0.1	0.1	0	0	0
Mexico	Ebano	0.5	0.3	0.6	2	1	2	0.1	0.1	0.2	1	0	1
Mexico	Ecatepec De Morelos	0.3	0.3	0.4	53	41	66	0.1	0.1	0.1	10	7	14
Mexico	Ecatzingo	0.3	0.3	0.4	0	0	0	0.1	0.1	0.1	0	0	0
Mexico	Ecuandureo	0.4	0.3	0.5	1	1	1	0.1	0.1	0.2	0	0	0
Mexico	Eduardo Neri	1.0	0.8	1.3	6	5	8	0.2	0.1	0.3	1	1	2
Mexico	Ejutla	0.3	0.2	0.4	0	0	0	0.1	0.0	0.1	0	0	0
Mexico	El Arenal	0.2	0.2	0.3	1	0	1	0.1	0.0	0.1	0	0	0
Mexico	El Arenal	0.3	0.2	0.4	0	0	1	0.1	0.0	0.1	0	0	0
Mexico	El Barrio De La Soledad	0.8	0.6	1.0	2	1	2	0.2	0.2	0.3	0	0	0
Mexico	El Bosque	1.2	0.9	1.6	3	2	3	0.3	0.2	0.4	1	1	2
Mexico	El Carmen Tequexquitla	0.3	0.2	0.4	0	0	1	0.1	0.1	0.1	0	0	0
Mexico	El Espinal	0.8	0.5	1.0	1	1	1	0.3	0.2	0.3	0	0	0
Mexico	El Fuerte	0.2	0.1	0.2	2	1	3	0.1	0.0	0.1	1	0	1
Mexico	El Grullo	0.3	0.2	0.4	1	0	1	0.1	0.0	0.1	0	0	0
Mexico	El Higo	0.4	0.3	0.5	1	1	1	0.1	0.1	0.1	0	0	0
Mexico	El Limon	0.3	0.2	0.4	0	0	0	0.1	0.0	0.1	0	0	0
Mexico	El Llano	0.2	0.2	0.3	0	0	1	0.1	0.1	0.1	0	0	0
Mexico	El Mante	0.1	0.1	0.2	2	1	2	0.1	0.0	0.1	0	0	1
Mexico	El Marques	0.2	0.2	0.3	2	1	2	0.1	0.1	0.1	2	1	2
Mexico	El Naranjo	0.4	0.3	0.6	1	1	1	0.1	0.1	0.2	0	0	0
Mexico	El Oro	0.3	0.3	0.4	1	1	2	0.1	0.1	0.1	0	0	0
Mexico	El Oro	0.3	0.2	0.3	0	0	0	0.1	0.0	0.1	0	0	0
Mexico	El Plateado De Joaquin Amaro	0.3	0.2	0.4	0	0	0	0.1	0.0	0.1	0	0	0

Table 3: Diarrhoea Mortality by Unit *(continued)*

Country	Unit	2000 mortality mean rate (per 10,000)	lower rate (per 10,000)	upper rate (per 10,000)	mean count	lower count	upper count	2017 mortality mean rate (per 10,000)	lower rate (per 10,000)	upper rate (per 10,000)	mean count	lower count	upper count
Mexico	El Porvenir	1.1	0.8	1.5	2	2	3	0.3	0.2	0.4	1	0	1
Mexico	El Salto	0.2	0.2	0.3	3	3	5	0.1	0.0	0.1	1	1	2
Mexico	El Salvador	0.3	0.2	0.4	0	0	0	0.1	0.0	0.1	0	0	0
Mexico	El Tule	0.3	0.2	0.4	0	0	0	0.1	0.1	0.1	0	0	0
Mexico	Elota	0.2	0.1	0.2	1	1	2	0.0	0.0	0.1	0	0	0
Mexico	Eloxochitlan	0.2	0.2	0.3	0	0	0	0.1	0.0	0.1	0	0	0
Mexico	Eloxochitlan	0.6	0.5	0.8	1	1	2	0.1	0.1	0.2	0	0	0
Mexico	Eloxochitlan De Flores Magon	0.7	0.5	1.0	1	1	1	0.2	0.2	0.3	0	0	0
Mexico	Emiliano Zapata	0.3	0.2	0.4	0	0	0	0.1	0.1	0.1	0	0	0
Mexico	Emiliano Zapata	0.2	0.2	0.3	0	0	0	0.1	0.0	0.1	0	0	0
Mexico	Emiliano Zapata	0.4	0.3	0.5	3	2	3	0.1	0.1	0.1	1	0	1
Mexico	Emiliano Zapata	0.4	0.3	0.5	1	1	2	0.1	0.1	0.2	0	0	1
Mexico	Emiliano Zapata	0.3	0.2	0.3	3	2	4	0.1	0.1	0.1	1	1	1
Mexico	Empalme	0.3	0.2	0.4	2	1	3	0.1	0.1	0.1	0	0	0
Mexico	Encarnacion De Diaz	0.2	0.2	0.3	1	1	2	0.0	0.0	0.1	0	0	0
Mexico	Ensenada	0.2	0.1	0.2	6	4	9	0.0	0.0	0.1	2	1	3
Mexico	Epatlan	0.6	0.4	0.8	0	0	0	0.2	0.1	0.2	0	0	0
Mexico	Epazoyucan	0.2	0.2	0.3	0	0	0	0.1	0.0	0.1	0	0	0
Mexico	Epitacio Huerta	0.4	0.3	0.5	1	1	1	0.1	0.1	0.2	0	0	0
Mexico	Erongaricuaro	0.4	0.3	0.5	1	1	1	0.1	0.1	0.2	0	0	0
Mexico	Escarcega	0.3	0.2	0.4	2	1	3	0.1	0.1	0.1	1	0	1
Mexico	Escobedo	0.2	0.2	0.3	0	0	0	0.1	0.0	0.1	0	0	0
Mexico	Escuinapa	0.2	0.1	0.2	1	1	1	0.0	0.0	0.1	0	0	0
Mexico	Escuintla	1.2	0.9	1.6	4	3	6	0.3	0.2	0.4	1	1	2
Mexico	Espanita	0.3	0.2	0.4	0	0	1	0.1	0.1	0.1	0	0	0
Mexico	Esperanza	0.5	0.4	0.7	1	1	1	0.2	0.1	0.2	0	0	0
Mexico	Espinal	0.4	0.3	0.5	1	1	1	0.1	0.1	0.1	0	0	0
Mexico	Espita	0.3	0.3	0.4	1	0	1	0.1	0.1	0.2	0	0	0
Mexico	Etchojoa	0.3	0.3	0.4	2	2	3	0.1	0.1	0.1	1	0	1
Mexico	Etzatlan	0.3	0.2	0.3	1	0	1	0.1	0.0	0.1	0	0	0
Mexico	Ezequiel Montes	0.2	0.2	0.3	1	1	1	0.1	0.1	0.1	0	0	1
Mexico	Felipe Carrillo Puerto	0.3	0.3	0.4	2	2	3	0.1	0.1	0.1	1	1	1
Mexico	Filomeno Mata	0.4	0.3	0.5	1	1	1	0.1	0.1	0.1	0	0	0
Mexico	Florencio Villarreal	1.1	0.8	1.4	3	3	4	0.2	0.1	0.3	1	0	1
Mexico	Fortin	0.4	0.3	0.5	3	2	4	0.1	0.1	0.1	1	0	1
Mexico	Francisco I. Madero	0.2	0.1	0.3	1	1	1	0.1	0.0	0.1	0	0	0
Mexico	Francisco I. Madero	0.2	0.1	0.2	1	1	1	0.1	0.0	0.1	0	0	1
Mexico	Francisco Leon	1.2	0.8	1.5	1	1	2	0.3	0.2	0.4	0	0	0
Mexico	Francisco Z. Mena	0.5	0.4	0.7	1	1	2	0.2	0.1	0.2	0	0	0
Mexico	Fresnillo	0.3	0.2	0.4	8	6	10	0.1	0.0	0.1	2	1	2
Mexico	Fresnillo De Trujano	0.7	0.5	0.9	0	0	0	0.2	0.2	0.3	0	0	0
Mexico	Frontera	0.2	0.2	0.3	1	1	2	0.1	0.0	0.1	0	0	1
Mexico	Frontera Comalapa	1.2	0.9	1.5	8	6	11	0.3	0.2	0.4	3	2	4
Mexico	Frontera Hidalgo	1.1	0.8	1.5	2	1	2	0.3	0.3	0.4	0	0	1
Mexico	Fronteras	0.3	0.2	0.4	0	0	0	0.1	0.0	0.1	0	0	0
Mexico	Gabriel Zamora	0.5	0.4	0.6	1	1	2	0.1	0.1	0.2	0	0	0
Mexico	Galeana	0.1	0.1	0.1	1	0	1	0.0	0.0	0.0	0	0	0
Mexico	Galeana	0.3	0.2	0.4	0	0	0	0.1	0.1	0.1	0	0	0
Mexico	Garcia	0.1	0.1	0.1	0	0	1	0.0	0.0	0.0	2	1	3
Mexico	Genaro Codina	0.3	0.2	0.4	0	0	0	0.1	0.0	0.1	0	0	0
Mexico	General Bravo	0.1	0.1	0.2	0	0	0	0.0	0.0	0.0	0	0	0

Table 3: Diarrhoea Mortality by Unit *(continued)*

Country	Unit	2000 mortality mean rate (per 10,000)	2000 mortality lower rate (per 10,000)	2000 mortality upper rate (per 10,000)	2000 mortality mean count	2000 mortality lower count	2000 mortality upper count	2017 mortality mean rate (per 10,000)	2017 mortality lower rate (per 10,000)	2017 mortality upper rate (per 10,000)	2017 mortality mean count	2017 mortality lower count	2017 mortality upper count
Mexico	General Canuto A. Neri	1.0	0.8	1.3	1	1	1	0.2	0.1	0.3	0	0	0
Mexico	General Cepeda	0.2	0.1	0.3	0	0	0	0.1	0.0	0.1	0	0	0
Mexico	General Enrique Estrada	0.3	0.2	0.4	0	0	0	0.1	0.0	0.1	0	0	0
Mexico	General Felipe Angeles	0.5	0.4	0.7	1	1	1	0.2	0.1	0.2	0	0	1
Mexico	General Francisco R. Murguia	0.3	0.2	0.4	1	1	1	0.1	0.0	0.1	0	0	0
Mexico	General Heliodoro Castillo	1.0	0.8	1.3	7	6	9	0.2	0.1	0.3	1	1	2
Mexico	General Panfilo Natera	0.3	0.2	0.4	1	1	1	0.1	0.0	0.1	0	0	0
Mexico	General Plutarco Elias Calles	0.3	0.2	0.3	0	0	0	0.1	0.0	0.1	0	0	0
Mexico	Gomez Farias	0.1	0.1	0.2	0	0	0	0.0	0.0	0.1	0	0	0
Mexico	Gomez Farias	0.3	0.2	0.4	0	0	0	0.1	0.1	0.1	0	0	0
Mexico	Gomez Farias	0.3	0.2	0.4	0	0	1	0.1	0.0	0.1	0	0	0
Mexico	Gomez Palacio	0.2	0.1	0.3	8	5	10	0.1	0.0	0.1	2	2	3
Mexico	Gonzalez	0.1	0.1	0.2	1	0	1	0.1	0.0	0.1	0	0	0
Mexico	Gral. Escobedo	0.1	0.1	0.1	4	3	5	0.0	0.0	0.0	1	1	2
Mexico	Gral. Simon Bolivar	0.2	0.2	0.3	0	0	0	0.1	0.0	0.1	0	0	0
Mexico	Gral. Teran	0.1	0.1	0.2	0	0	0	0.0	0.0	0.0	0	0	0
Mexico	Gral. Trevino	0.1	0.1	0.2	0	0	0	0.0	0.0	0.1	0	0	0
Mexico	Gral. Zaragoza	0.1	0.1	0.2	0	0	0	0.0	0.0	0.0	0	0	0
Mexico	Gral. Zuazua	0.1	0.1	0.1	0	0	0	0.0	0.0	0.0	3	2	4
Mexico	Gran Morelos	0.3	0.2	0.4	0	0	0	0.1	0.1	0.1	0	0	0
Mexico	Granados	0.3	0.2	0.4	0	0	0	0.1	0.1	0.1	0	0	0
Mexico	Guachinango	0.3	0.2	0.4	0	0	0	0.1	0.0	0.1	0	0	0
Mexico	Guachochi	0.3	0.2	0.4	2	2	3	0.1	0.1	0.1	1	0	1
Mexico	Guadalajara	0.3	0.2	0.3	42	33	55	0.1	0.0	0.1	6	4	8
Mexico	Guadalcazar	0.4	0.3	0.5	1	1	2	0.1	0.1	0.2	0	0	0
Mexico	Guadalupe	0.3	0.2	0.4	5	3	6	0.1	0.0	0.1	1	1	2
Mexico	Guadalupe	0.1	0.1	0.1	8	6	10	0.0	0.0	0.0	1	1	2
Mexico	Guadalupe	0.3	0.2	0.5	1	0	1	0.1	0.1	0.1	0	0	0
Mexico	Guadalupe	0.6	0.5	0.8	0	0	1	0.2	0.1	0.2	0	0	0
Mexico	Guadalupe De Ramirez	0.7	0.5	0.9	0	0	0	0.2	0.2	0.3	0	0	0
Mexico	Guadalupe Etla	0.6	0.5	0.8	0	0	0	0.2	0.2	0.3	0	0	0
Mexico	Guadalupe Victoria	0.5	0.4	0.7	1	1	2	0.1	0.1	0.2	0	0	0
Mexico	Guadalupe Victoria	0.2	0.2	0.3	1	1	1	0.1	0.0	0.1	0	0	0
Mexico	Guadalupe Y Calvo	0.3	0.2	0.4	3	2	4	0.1	0.1	0.1	1	0	1
Mexico	Guanacevi	0.3	0.2	0.3	0	0	0	0.1	0.0	0.1	0	0	0
Mexico	Guanajuato	0.4	0.3	0.5	7	5	8	0.1	0.0	0.1	1	1	2
Mexico	Guasave	0.2	0.1	0.3	7	5	9	0.1	0.0	0.1	2	1	2
Mexico	Guaymas	0.3	0.2	0.4	4	3	6	0.1	0.1	0.1	1	1	2
Mexico	Guazapares	0.4	0.3	0.5	0	0	1	0.1	0.1	0.1	0	0	0
Mexico	Guelatao De Juarez	0.7	0.5	0.9	0	0	0	0.2	0.2	0.3	0	0	0
Mexico	Guemez	0.1	0.1	0.2	0	0	0	0.0	0.0	0.1	0	0	0
Mexico	Guerrero	0.3	0.2	0.3	0	0	0	0.1	0.0	0.1	0	0	0
Mexico	Guerrero	0.1	0.1	0.2	0	0	0	0.0	0.0	0.1	0	0	0
Mexico	Guerrero	0.3	0.2	0.4	1	1	2	0.1	0.1	0.1	0	0	0
Mexico	Guevea De Humboldt	0.8	0.6	1.1	0	0	1	0.2	0.2	0.3	0	0	0

Table 3: Diarrhoea Mortality by Unit *(continued)*

Country	Unit	2000 mortality mean rate (per 10,000)	lower rate (per 10,000)	upper rate (per 10,000)	mean count	lower count	upper count	2017 mortality mean rate (per 10,000)	lower rate (per 10,000)	upper rate (per 10,000)	mean count	lower count	upper count
Mexico	Gustavo A. Madero	0.1	0.1	0.2	16	12	20	0.1	0.0	0.1	5	3	7
Mexico	Gustavo Diaz Ordaz	0.1	0.1	0.2	0	0	0	0.0	0.0	0.1	0	0	0
Mexico	Gutierrez Zamora	0.3	0.2	0.4	1	1	1	0.1	0.1	0.1	0	0	0
Mexico	Halacho	0.4	0.3	0.5	1	1	1	0.1	0.1	0.2	0	0	0
Mexico	Hecelchakan	0.3	0.2	0.4	1	1	1	0.1	0.1	0.1	0	0	0
Mexico	Hermenegildo Galeana	0.6	0.4	0.7	1	0	1	0.2	0.1	0.2	0	0	0
Mexico	Hermosillo	0.3	0.2	0.4	21	15	28	0.1	0.0	0.1	6	4	8
Mexico	Heroica Ciudad De Ejutla De Crespo	0.7	0.5	0.9	2	1	2	0.2	0.2	0.3	1	0	1
Mexico	Heroica Ciudad De Huajuapan De Leon	0.6	0.5	0.8	4	3	5	0.2	0.1	0.3	2	1	3
Mexico	Heroica Ciudad De Tlaxiaco	0.6	0.5	0.8	3	2	4	0.2	0.1	0.3	1	1	1
Mexico	Hidalgo	0.2	0.2	0.3	0	0	0	0.1	0.0	0.1	0	0	0
Mexico	Hidalgo	0.2	0.2	0.3	0	0	0	0.1	0.0	0.1	0	0	0
Mexico	Hidalgo	0.1	0.1	0.2	0	0	0	0.0	0.0	0.1	0	0	0
Mexico	Hidalgo	0.1	0.1	0.1	0	0	0	0.0	0.0	0.0	0	0	0
Mexico	Hidalgo	0.4	0.3	0.5	7	5	8	0.1	0.1	0.2	2	1	2
Mexico	Hidalgo Del Parral	0.3	0.2	0.4	3	2	4	0.1	0.1	0.1	1	1	1
Mexico	Hidalgotitlan	0.4	0.3	0.6	1	1	1	0.1	0.1	0.1	0	0	0
Mexico	Higueras	0.1	0.1	0.2	0	0	0	0.0	0.0	0.0	0	0	0
Mexico	Hocaba	0.3	0.2	0.4	0	0	0	0.1	0.1	0.1	0	0	0
Mexico	Hoctun	0.3	0.2	0.4	0	0	0	0.1	0.1	0.1	0	0	0
Mexico	Homun	0.3	0.2	0.4	0	0	0	0.1	0.1	0.1	0	0	0
Mexico	Honey	0.5	0.3	0.6	0	0	1	0.1	0.1	0.2	0	0	0
Mexico	Hopelchen	0.3	0.2	0.4	1	1	2	0.1	0.1	0.1	0	0	1
Mexico	Hostotipaquillo	0.3	0.2	0.4	0	0	0	0.1	0.0	0.1	0	0	0
Mexico	Huachinera	0.3	0.2	0.4	0	0	0	0.1	0.0	0.1	0	0	0
Mexico	Huajicori	0.3	0.2	0.4	0	0	1	0.1	0.1	0.1	0	0	0
Mexico	Hualahuises	0.1	0.1	0.2	0	0	0	0.0	0.0	0.0	0	0	0
Mexico	Huamantla	0.3	0.2	0.4	3	2	4	0.1	0.1	0.1	1	1	1
Mexico	Huamuxtitlan	1.1	0.8	1.4	2	2	3	0.2	0.1	0.3	0	0	1
Mexico	Huandacareo	0.4	0.3	0.5	0	0	1	0.1	0.1	0.2	0	0	0
Mexico	Huanimaro	0.4	0.3	0.5	1	1	1	0.1	0.0	0.1	0	0	0
Mexico	Huaniqueo	0.4	0.3	0.5	0	0	0	0.1	0.1	0.2	0	0	0
Mexico	Huanusco	0.3	0.2	0.4	0	0	0	0.1	0.1	0.1	0	0	0
Mexico	Huaquechula	0.6	0.4	0.7	2	2	3	0.2	0.1	0.2	0	0	1
Mexico	Huasabas	0.3	0.2	0.4	0	0	0	0.1	0.1	0.1	0	0	0
Mexico	Huasca De Ocampo	0.2	0.2	0.3	0	0	1	0.1	0.0	0.1	0	0	0
Mexico	Huatabampo	0.3	0.2	0.4	3	2	4	0.1	0.1	0.1	0	0	1
Mexico	Huatlatlauca	0.6	0.4	0.7	0	0	1	0.2	0.1	0.2	0	0	0
Mexico	Huatusco	0.4	0.3	0.5	3	2	4	0.1	0.0	0.1	0	0	1
Mexico	Huauchinango	0.5	0.4	0.7	7	5	9	0.1	0.1	0.2	2	1	2
Mexico	Huautepec	0.7	0.5	0.9	1	0	1	0.2	0.2	0.3	0	0	0
Mexico	Huautla	0.3	0.2	0.3	1	1	1	0.1	0.0	0.1	0	0	0
Mexico	Huautla De Jimenez	0.7	0.5	0.9	3	2	4	0.2	0.2	0.3	1	0	1
Mexico	Huayacocotla	0.3	0.2	0.4	1	1	1	0.1	0.1	0.1	0	0	0
Mexico	Huazalingo	0.3	0.2	0.4	1	0	1	0.1	0.0	0.1	0	0	0
Mexico	Huehuetan	1.2	0.9	1.7	5	4	7	0.3	0.3	0.4	2	1	2
Mexico	Huehuetla	0.3	0.2	0.3	1	1	1	0.1	0.0	0.1	0	0	0
Mexico	Huehuetla	0.5	0.4	0.7	1	1	1	0.1	0.1	0.2	0	0	0
Mexico	Huehuetlan	0.5	0.4	0.7	1	1	1	0.1	0.1	0.2	0	0	0

Table 3: Diarrhoea Mortality by Unit *(continued)*

Country	Unit	2000 mortality mean rate (per 10,000)	lower rate (per 10,000)	upper rate (per 10,000)	mean count	lower count	upper count	2017 mortality mean rate (per 10,000)	lower rate (per 10,000)	upper rate (per 10,000)	mean count	lower count	upper count
Mexico	Huehuetlan El Chico	0.6	0.5	0.8	0	0	1	0.2	0.1	0.2	0	0	0
Mexico	Huehuetlan El Grande	0.6	0.4	0.7	0	0	1	0.2	0.1	0.2	0	0	0
Mexico	Huehuetoca	0.3	0.2	0.4	1	1	1	0.1	0.1	0.1	1	1	2
Mexico	Huejotitan	0.3	0.2	0.4	0	0	0	0.1	0.1	0.1	0	0	0
Mexico	Huejotzingo	0.5	0.4	0.6	5	4	6	0.2	0.1	0.2	1	1	2
Mexico	Huejucar	0.3	0.2	0.4	0	0	0	0.1	0.0	0.1	0	0	0
Mexico	Huejuquilla El Alto	0.3	0.2	0.4	0	0	0	0.1	0.0	0.1	0	0	0
Mexico	Huejutla De Reyes	0.3	0.2	0.3	5	4	6	0.1	0.0	0.1	1	1	1
Mexico	Huepac	0.3	0.2	0.4	0	0	0	0.1	0.0	0.1	0	0	0
Mexico	Huetamo	0.5	0.4	0.6	3	2	4	0.1	0.1	0.2	1	0	1
Mexico	Hueyapan	0.5	0.4	0.7	1	1	1	0.1	0.1	0.2	0	0	0
Mexico	Hueyapan De Ocampo	0.4	0.3	0.6	2	2	3	0.1	0.1	0.1	0	0	0
Mexico	Hueyotlipan	0.3	0.2	0.4	0	0	1	0.1	0.1	0.1	0	0	0
Mexico	Hueypoxtla	0.3	0.2	0.4	1	1	1	0.1	0.1	0.1	0	0	1
Mexico	Hueytamalco	0.6	0.4	0.7	2	1	2	0.2	0.1	0.2	0	0	1
Mexico	Hueytlalpan	0.5	0.4	0.7	1	0	1	0.2	0.1	0.2	0	0	0
Mexico	Huhi	0.3	0.2	0.4	0	0	0	0.1	0.1	0.1	0	0	0
Mexico	Huichapan	0.2	0.2	0.3	1	1	1	0.1	0.0	0.1	0	0	0
Mexico	Huiloapan	0.4	0.3	0.5	1	1	2	0.1	0.1	0.1	0	0	0
Mexico	Huimanguillo	0.3	0.3	0.4	9	6	12	0.1	0.1	0.2	3	2	4
Mexico	Huimilpan	0.3	0.2	0.3	1	1	2	0.1	0.1	0.1	0	0	1
Mexico	Huiramba	0.4	0.3	0.5	0	0	1	0.1	0.1	0.2	0	0	0
Mexico	Huitiupan	1.2	0.9	1.6	4	3	5	0.3	0.2	0.4	1	1	2
Mexico	Huitzilac	0.3	0.2	0.4	1	1	1	0.1	0.1	0.1	0	0	0
Mexico	Huitzilan De Serdan	0.5	0.4	0.7	1	1	1	0.1	0.1	0.2	0	0	0
Mexico	Huitziltepec	0.5	0.4	0.7	0	0	0	0.2	0.1	0.2	0	0	0
Mexico	Huitzuco De Los Figueroa	1.0	0.7	1.2	4	3	5	0.2	0.1	0.3	1	1	1
Mexico	Huixquilucan	0.4	0.3	0.4	12	10	15	0.1	0.1	0.1	2	2	3
Mexico	Huixtan	1.1	0.8	1.4	4	3	5	0.3	0.2	0.4	1	1	2
Mexico	Huixtla	1.1	0.8	1.5	7	5	9	0.3	0.2	0.4	2	1	3
Mexico	Hunucma	0.3	0.2	0.4	1	1	1	0.1	0.1	0.1	0	0	0
Mexico	Ignacio De La Llave	0.4	0.3	0.6	1	1	1	0.1	0.1	0.1	0	0	0
Mexico	Ignacio Zaragoza	0.3	0.2	0.4	0	0	0	0.1	0.1	0.1	0	0	0
Mexico	Iguala De La Independencia	1.0	0.7	1.2	16	12	20	0.2	0.1	0.3	4	3	5
Mexico	Igualapa	1.2	0.9	1.5	2	1	3	0.2	0.1	0.3	0	0	0
Mexico	Ilamatlan	0.4	0.3	0.5	1	1	1	0.1	0.1	0.1	0	0	0
Mexico	Iliatenco	1.1	0.8	1.4	2	1	2	0.2	0.1	0.3	0	0	0
Mexico	Imuris	0.3	0.2	0.4	0	0	1	0.1	0.0	0.1	0	0	0
Mexico	Indaparapeo	0.4	0.3	0.5	1	1	1	0.1	0.1	0.2	0	0	0
Mexico	Inde	0.2	0.2	0.3	0	0	0	0.1	0.0	0.1	0	0	0
Mexico	Irapuato	0.4	0.3	0.5	21	16	27	0.1	0.0	0.1	5	3	7
Mexico	Irimbo	0.5	0.4	0.6	1	1	1	0.1	0.1	0.2	0	0	0
Mexico	Isidro Fabela	0.3	0.3	0.4	0	0	0	0.1	0.1	0.1	0	0	0
Mexico	Isla	0.4	0.3	0.5	2	1	3	0.1	0.1	0.1	0	0	1
Mexico	Isla Mujeres	0.3	0.2	0.5	0	0	1	0.1	0.1	0.1	0	0	0
Mexico	Iturbide	0.1	0.1	0.1	0	0	0	0.0	0.0	0.0	0	0	0
Mexico	Ixcamilpa De Guerrero	0.7	0.5	0.8	0	0	1	0.2	0.1	0.2	0	0	0
Mexico	Ixcaquixtla	0.6	0.4	0.7	0	0	1	0.2	0.1	0.2	0	0	0
Mexico	Ixcateopan De Cuauhtemoc	1.0	0.8	1.2	1	1	1	0.2	0.1	0.3	0	0	0

Table 3: Diarrhoea Mortality by Unit *(continued)*

Country	Unit	2000 mortality mean rate (per 10,000)	lower rate (per 10,000)	upper rate (per 10,000)	mean count	lower count	upper count	2017 mortality mean rate (per 10,000)	lower rate (per 10,000)	upper rate (per 10,000)	mean count	lower count	upper count
Mexico	Ixcatepec	0.4	0.3	0.5	1	1	1	0.1	0.1	0.1	0	0	0
Mexico	Ixhuacan De Los Reyes	0.4	0.3	0.5	1	1	1	0.1	0.1	0.1	0	0	0
Mexico	Ixhuatan	1.2	0.9	1.5	1	1	2	0.3	0.2	0.4	1	1	1
Mexico	Ixhuatlan De Madero	0.4	0.3	0.5	3	2	3	0.1	0.1	0.1	0	0	0
Mexico	Ixhuatlan Del Cafe	0.3	0.3	0.5	1	1	1	0.1	0.0	0.1	0	0	0
Mexico	Ixhuatlan Del Sureste	0.4	0.3	0.6	1	1	1	0.1	0.1	0.1	0	0	0
Mexico	Ixhuatlancillo	0.3	0.3	0.5	1	1	1	0.1	0.0	0.1	0	0	0
Mexico	Ixil	0.3	0.2	0.4	0	0	0	0.1	0.1	0.1	0	0	0
Mexico	Ixmatlahuacan	0.4	0.3	0.6	0	0	0	0.1	0.1	0.1	0	0	0
Mexico	Ixmiquilpan	0.2	0.2	0.3	2	1	2	0.1	0.0	0.1	1	0	1
Mexico	Ixpantepec Nieves	0.6	0.5	0.8	0	0	0	0.2	0.1	0.3	0	0	0
Mexico	Ixtacamaxtitlan	0.5	0.4	0.6	2	1	2	0.1	0.1	0.2	0	0	1
Mexico	Ixtacomitan	1.2	0.9	1.6	2	1	2	0.3	0.2	0.4	0	0	1
Mexico	Ixtacuixtla De Mariano Matamoros	0.3	0.2	0.4	1	1	1	0.1	0.1	0.1	0	0	1
Mexico	Ixtaczoquitlan	0.4	0.3	0.5	3	3	5	0.1	0.0	0.1	1	0	1
Mexico	Ixtapa	1.1	0.8	1.5	3	2	4	0.3	0.2	0.4	1	1	1
Mexico	Ixtapaluca	0.3	0.3	0.4	15	12	19	0.1	0.1	0.1	4	3	5
Mexico	Ixtapan De La Sal	0.4	0.3	0.5	1	1	2	0.1	0.1	0.1	0	0	0
Mexico	Ixtapan Del Oro	0.4	0.3	0.4	0	0	0	0.1	0.1	0.1	0	0	0
Mexico	Ixtapangajoya	1.2	0.9	1.6	1	1	1	0.3	0.2	0.4	0	0	0
Mexico	Ixtenco	0.3	0.2	0.4	0	0	0	0.1	0.1	0.1	0	0	0
Mexico	Ixtepec	0.5	0.4	0.7	0	0	1	0.1	0.1	0.2	0	0	0
Mexico	Ixtlahuaca	0.3	0.2	0.4	5	4	6	0.1	0.1	0.1	1	1	2
Mexico	Ixtlahuacan	0.3	0.2	0.4	0	0	0	0.1	0.1	0.1	0	0	0
Mexico	Ixtlahuacan De Los Membrillos	0.3	0.2	0.3	1	0	1	0.1	0.0	0.1	1	0	1
Mexico	Ixtlahuacan Del Rio	0.3	0.2	0.3	1	1	1	0.1	0.0	0.1	0	0	0
Mexico	Ixtlan	0.4	0.3	0.5	1	0	1	0.1	0.1	0.2	0	0	0
Mexico	Ixtlan De Juarez	0.7	0.6	1.0	1	1	1	0.2	0.2	0.3	0	0	0
Mexico	Ixtlan Del Rio	0.2	0.2	0.3	1	1	1	0.1	0.0	0.1	0	0	0
Mexico	Izamal	0.3	0.2	0.4	1	1	1	0.1	0.1	0.1	0	0	0
Mexico	Iztacalco	0.1	0.1	0.2	5	4	7	0.1	0.0	0.1	2	1	2
Mexico	Iztapalapa	0.2	0.1	0.2	28	22	35	0.1	0.0	0.1	9	7	13
Mexico	Izucar De Matamoros	0.5	0.4	0.7	5	4	6	0.2	0.1	0.2	1	1	2
Mexico	Jacala De Ledezma	0.2	0.2	0.3	0	0	1	0.1	0.0	0.1	0	0	0
Mexico	Jacona	0.4	0.3	0.5	5	3	6	0.1	0.1	0.2	1	1	1
Mexico	Jala	0.2	0.2	0.3	1	0	1	0.1	0.0	0.1	0	0	0
Mexico	Jalacingo	0.3	0.2	0.4	3	2	4	0.1	0.1	0.1	1	0	1
Mexico	Jalapa	0.3	0.3	0.5	1	1	2	0.1	0.1	0.2	0	0	1
Mexico	Jalcomulco	0.4	0.3	0.5	0	0	0	0.1	0.1	0.1	0	0	0
Mexico	Jalostotitlan	0.2	0.2	0.3	1	1	1	0.0	0.0	0.1	0	0	0
Mexico	Jalpa	0.3	0.2	0.4	1	1	1	0.1	0.0	0.1	0	0	0
Mexico	Jalpa De Mendez	0.3	0.2	0.4	3	2	4	0.1	0.1	0.2	1	1	2
Mexico	Jalpan	0.6	0.4	0.7	1	1	2	0.2	0.1	0.2	0	0	0
Mexico	Jalpan De Serra	0.3	0.2	0.4	1	1	1	0.1	0.1	0.1	0	0	0
Mexico	Jaltenco	0.3	0.2	0.4	0	0	0	0.1	0.1	0.1	0	0	0
Mexico	Jaltipan	0.4	0.3	0.6	2	1	3	0.1	0.1	0.1	0	0	0
Mexico	Jaltocan	0.3	0.2	0.3	0	0	0	0.1	0.0	0.1	0	0	0
Mexico	Jamapa	0.4	0.3	0.5	0	0	1	0.1	0.1	0.1	0	0	0

Table 3: Diarrhoea Mortality by Unit *(continued)*

Country	Unit	2000 mortality mean rate (per 10,000)	2000 mortality lower rate (per 10,000)	2000 mortality upper rate (per 10,000)	2000 mortality mean count	2000 mortality lower count	2000 mortality upper count	2017 mortality mean rate (per 10,000)	2017 mortality lower rate (per 10,000)	2017 mortality upper rate (per 10,000)	2017 mortality mean count	2017 mortality lower count	2017 mortality upper count
Mexico	Jamay	0.3	0.2	0.3	1	0	1	0.1	0.0	0.1	0	0	0
Mexico	Janos	0.4	0.3	0.5	0	0	0	0.1	0.1	0.1	0	0	0
Mexico	Jantetelco	0.3	0.2	0.4	0	0	1	0.1	0.1	0.1	0	0	0
Mexico	Jaral Del Progreso	0.4	0.3	0.5	2	1	2	0.1	0.0	0.1	0	0	0
Mexico	Jaumave	0.1	0.1	0.2	0	0	0	0.0	0.0	0.1	0	0	0
Mexico	Jerecuaro	0.4	0.3	0.5	2	2	3	0.1	0.1	0.1	0	0	1
Mexico	Jerez	0.3	0.2	0.4	2	1	2	0.1	0.0	0.1	0	0	0
Mexico	Jesus Carranza	0.4	0.3	0.6	1	1	2	0.1	0.1	0.1	0	0	0
Mexico	Jesus Maria	0.3	0.2	0.3	1	0	1	0.1	0.0	0.1	0	0	0
Mexico	Jesus Maria	0.2	0.2	0.3	2	2	3	0.1	0.1	0.1	1	1	2
Mexico	Jilotepec	0.3	0.3	0.4	3	2	3	0.1	0.1	0.1	1	1	1
Mexico	Jilotepec	0.3	0.3	0.4	1	1	1	0.1	0.0	0.1	0	0	0
Mexico	Jilotlan De Los Dolores	0.3	0.2	0.4	0	0	0	0.1	0.0	0.1	0	0	0
Mexico	Jilotzingo	0.3	0.3	0.4	1	0	1	0.1	0.1	0.1	0	0	0
Mexico	Jimenez	0.2	0.2	0.3	0	0	0	0.1	0.0	0.1	0	0	0
Mexico	Jimenez	0.1	0.1	0.2	0	0	0	0.1	0.0	0.1	0	0	0
Mexico	Jimenez	0.3	0.2	0.4	1	1	2	0.1	0.1	0.1	0	0	0
Mexico	Jimenez	0.4	0.3	0.5	1	0	1	0.1	0.1	0.2	0	0	0
Mexico	Jimenez Del Teul	0.3	0.2	0.4	0	0	0	0.1	0.0	0.1	0	0	0
Mexico	Jiquilpan	0.4	0.3	0.5	2	1	2	0.1	0.1	0.2	0	0	1
Mexico	Jiquipilas	1.1	0.8	1.4	4	3	5	0.3	0.2	0.4	1	1	2
Mexico	Jiquipilco	0.3	0.3	0.4	3	2	3	0.1	0.1	0.1	1	0	1
Mexico	Jitotol	1.1	0.8	1.4	3	2	4	0.3	0.2	0.4	1	1	2
Mexico	Jiutepec	0.3	0.2	0.3	5	4	6	0.1	0.1	0.1	1	1	2
Mexico	Jocotepec	0.3	0.2	0.3	1	1	1	0.1	0.0	0.1	0	0	0
Mexico	Jocotitlan	0.3	0.2	0.4	3	2	3	0.1	0.1	0.1	1	0	1
Mexico	Jojutla	0.3	0.2	0.3	2	1	2	0.1	0.1	0.1	0	0	1
Mexico	Jolalpan	0.6	0.5	0.8	1	1	1	0.2	0.1	0.2	0	0	0
Mexico	Jonacatepec	0.3	0.2	0.4	0	0	1	0.1	0.1	0.1	0	0	0
Mexico	Jonotla	0.6	0.4	0.8	0	0	0	0.2	0.1	0.2	0	0	0
Mexico	Jonuta	0.3	0.2	0.4	1	1	2	0.1	0.1	0.2	0	0	0
Mexico	Jopala	0.6	0.4	0.7	2	1	2	0.2	0.1	0.2	0	0	0
Mexico	Joquicingo	0.4	0.3	0.5	1	0	1	0.1	0.1	0.1	0	0	0
Mexico	Jose Azueta	1.0	0.7	1.3	13	10	17	0.2	0.1	0.3	3	2	4
Mexico	Jose Azueta	0.4	0.3	0.6	1	1	2	0.1	0.1	0.1	0	0	0
Mexico	Jose Joaquin De Herrera	1.0	0.8	1.3	2	2	3	0.2	0.1	0.3	0	0	1
Mexico	Jose Maria Morelos	0.4	0.3	0.5	1	1	2	0.1	0.1	0.1	0	0	0
Mexico	Jose Sixto Verduzco	0.4	0.3	0.5	1	1	1	0.1	0.1	0.2	0	0	0
Mexico	Juan Aldama	0.3	0.2	0.4	0	0	1	0.1	0.0	0.1	0	0	0
Mexico	Juan C. Bonilla	0.5	0.4	0.6	1	1	1	0.2	0.1	0.2	0	0	1
Mexico	Juan Galindo	0.5	0.4	0.7	0	0	0	0.1	0.1	0.2	0	0	0
Mexico	Juan N. Mendez	0.6	0.4	0.7	0	0	0	0.2	0.1	0.2	0	0	0
Mexico	Juan R. Escudero	1.1	0.9	1.4	4	3	5	0.2	0.1	0.3	1	0	1
Mexico	Juan Rodriguez Clara	0.4	0.3	0.5	2	1	2	0.1	0.1	0.1	0	0	0
Mexico	Juanacatlan	0.3	0.2	0.3	1	1	1	0.1	0.0	0.1	0	0	0
Mexico	Juarez	0.3	0.2	0.3	0	0	0	0.1	0.0	0.1	0	0	0
Mexico	Juarez	0.1	0.1	0.1	2	2	3	0.0	0.0	0.0	1	1	2
Mexico	Juarez	0.3	0.2	0.4	49	35	67	0.1	0.1	0.1	12	8	17
Mexico	Juarez	0.5	0.4	0.6	1	1	1	0.1	0.1	0.2	0	0	0
Mexico	Juarez	1.1	0.8	1.5	3	2	4	0.3	0.2	0.4	1	1	1
Mexico	Juarez Hidalgo	0.2	0.2	0.3	0	0	0	0.1	0.0	0.1	0	0	0
Mexico	Juchipila	0.3	0.2	0.4	0	0	1	0.1	0.1	0.1	0	0	0
Mexico	Juchique De Ferrer	0.4	0.3	0.5	1	1	1	0.1	0.1	0.1	0	0	0

Table 3: Diarrhoea Mortality by Unit *(continued)*

Country	Unit	2000 mortality mean rate (per 10,000)	lower rate (per 10,000)	upper rate (per 10,000)	mean count	lower count	upper count	2017 mortality mean rate (per 10,000)	lower rate (per 10,000)	upper rate (per 10,000)	mean count	lower count	upper count
Mexico	Juchitan	1.1	0.8	1.4	1	1	1	0.2	0.1	0.3	0	0	0
Mexico	Juchitan De Zaragoza	0.7	0.5	1.0	7	5	9	0.2	0.2	0.3	2	2	3
Mexico	Juchitepec	0.3	0.3	0.4	1	1	1	0.1	0.1	0.1	0	0	0
Mexico	Juchitlan	0.3	0.2	0.4	0	0	0	0.1	0.0	0.1	0	0	0
Mexico	Julimes	0.3	0.2	0.4	0	0	0	0.1	0.1	0.1	0	0	0
Mexico	Jungapeo	0.5	0.3	0.6	1	1	2	0.1	0.1	0.2	0	0	0
Mexico	Kanasin	0.3	0.2	0.4	2	2	3	0.1	0.1	0.1	1	1	2
Mexico	Kantunil	0.3	0.2	0.4	0	0	0	0.1	0.1	0.1	0	0	0
Mexico	Kaua	0.3	0.2	0.4	0	0	0	0.1	0.1	0.2	0	0	0
Mexico	Kinchil	0.3	0.2	0.4	0	0	0	0.1	0.1	0.1	0	0	0
Mexico	Kopoma	0.3	0.2	0.4	0	0	0	0.1	0.1	0.1	0	0	0
Mexico	La Antigua	0.4	0.3	0.5	1	1	1	0.1	0.1	0.1	0	0	0
Mexico	La Barca	0.2	0.2	0.3	1	1	2	0.1	0.0	0.1	0	0	0
Mexico	La Colorada	0.3	0.2	0.4	0	0	0	0.1	0.1	0.1	0	0	0
Mexico	La Compania	0.7	0.5	0.9	0	0	1	0.2	0.2	0.3	0	0	0
Mexico	La Concordia	1.2	0.9	1.7	7	6	10	0.3	0.3	0.4	2	2	3
Mexico	La Cruz	0.3	0.2	0.4	0	0	0	0.1	0.1	0.1	0	0	0
Mexico	La Grandeza	1.1	0.8	1.4	1	1	1	0.3	0.2	0.4	0	0	0
Mexico	La Huacana	0.5	0.4	0.6	3	2	3	0.1	0.1	0.2	1	0	1
Mexico	La Huerta	0.3	0.2	0.4	1	0	1	0.1	0.0	0.1	0	0	0
Mexico	La Independencia	1.1	0.8	1.5	6	4	8	0.3	0.2	0.4	2	1	2
Mexico	La Libertad	1.3	0.9	1.6	1	1	1	0.3	0.2	0.4	0	0	0
Mexico	La Magdalena Contreras	0.2	0.1	0.2	3	2	3	0.1	0.0	0.1	1	1	1
Mexico	La Magdalena Tlaltelulco	0.3	0.2	0.4	1	0	1	0.1	0.1	0.1	0	0	0
Mexico	La Magdalena Tlatlauquitepec	0.6	0.4	0.7	0	0	0	0.2	0.1	0.2	0	0	0
Mexico	La Manzanilla De La Paz	0.3	0.2	0.3	0	0	0	0.1	0.0	0.1	0	0	0
Mexico	La Mision	0.2	0.2	0.3	0	0	0	0.1	0.0	0.1	0	0	0
Mexico	La Paz	0.2	0.1	0.2	4	3	5	0.0	0.0	0.1	1	1	2
Mexico	La Paz	0.4	0.3	0.4	12	10	16	0.1	0.1	0.1	3	2	3
Mexico	La Pe	0.7	0.5	0.9	0	0	0	0.2	0.2	0.3	0	0	0
Mexico	La Perla	0.3	0.3	0.5	1	1	1	0.1	0.0	0.1	0	0	0
Mexico	La Piedad	0.4	0.3	0.4	4	3	5	0.1	0.1	0.2	1	1	1
Mexico	La Reforma	0.8	0.6	1.0	1	0	1	0.3	0.2	0.4	0	0	0
Mexico	La Trinidad Vista Hermosa	0.6	0.5	0.8	0	0	0	0.2	0.1	0.3	0	0	0
Mexico	La Trinitaria	1.2	0.9	1.5	9	6	11	0.3	0.2	0.4	4	3	5
Mexico	La Union De Isidoro Montes De Oca	1.1	0.8	1.4	4	3	5	0.2	0.1	0.3	1	0	1
Mexico	La Yesca	0.3	0.2	0.4	0	0	1	0.1	0.0	0.1	0	0	0
Mexico	Lafragua	0.5	0.4	0.6	1	0	1	0.1	0.1	0.2	0	0	0
Mexico	Lagos De Moreno	0.2	0.2	0.3	4	3	5	0.0	0.0	0.1	1	1	1
Mexico	Lagunillas	0.4	0.3	0.5	0	0	0	0.1	0.1	0.2	0	0	0
Mexico	Lagunillas	0.4	0.3	0.6	0	0	0	0.1	0.1	0.2	0	0	0
Mexico	Lamadrid	0.2	0.1	0.3	0	0	0	0.1	0.0	0.1	0	0	0
Mexico	Lampazos De Naranjo	0.1	0.1	0.2	0	0	0	0.0	0.0	0.1	0	0	0
Mexico	Landa De Matamoros	0.3	0.2	0.4	1	1	1	0.1	0.1	0.1	0	0	0
Mexico	Landero Y Coss	0.3	0.2	0.4	0	0	0	0.1	0.0	0.1	0	0	0
Mexico	Larrainzar	1.1	0.8	1.5	4	3	5	0.3	0.2	0.4	1	1	2
Mexico	Las Choapas	0.4	0.3	0.5	4	3	5	0.1	0.1	0.1	1	1	1
Mexico	Las Margaritas	1.1	0.8	1.5	18	13	24	0.3	0.2	0.4	5	4	7
Mexico	Las Minas	0.3	0.2	0.4	0	0	0	0.1	0.0	0.1	0	0	0
Mexico	Las Rosas	1.1	0.8	1.5	4	3	5	0.3	0.2	0.4	1	1	1

Table 3: Diarrhoea Mortality by Unit *(continued)*

Country	Unit	2000 mortality mean rate (per 10,000)	lower rate (per 10,000)	upper rate (per 10,000)	mean count	lower count	upper count	2017 mortality mean rate (per 10,000)	lower rate (per 10,000)	upper rate (per 10,000)	mean count	lower count	upper count
Mexico	Las Vigas De Ramirez	0.3	0.2	0.4	1	0	1	0.1	0.0	0.1	0	0	0
Mexico	Lazaro Cardenas	0.3	0.2	0.4	0	0	0	0.1	0.1	0.1	0	0	0
Mexico	Lazaro Cardenas	0.5	0.4	0.6	11	8	14	0.1	0.1	0.2	3	2	4
Mexico	Lazaro Cardenas	0.4	0.3	0.5	1	1	1	0.1	0.1	0.1	0	0	0
Mexico	Leon	0.3	0.2	0.4	56	42	72	0.1	0.0	0.1	11	7	15
Mexico	Leonardo Bravo	1.0	0.8	1.2	4	3	5	0.2	0.1	0.3	1	1	1
Mexico	Lerdo	0.2	0.2	0.3	5	4	6	0.1	0.0	0.1	1	1	2
Mexico	Lerdo De Tejada	0.4	0.3	0.6	0	0	1	0.1	0.1	0.1	0	0	0
Mexico	Lerma	0.3	0.3	0.4	5	4	6	0.1	0.1	0.1	2	1	2
Mexico	Libres	0.5	0.4	0.6	2	2	3	0.1	0.1	0.2	1	0	1
Mexico	Linares	0.1	0.1	0.2	1	1	1	0.0	0.0	0.0	0	0	0
Mexico	Llera	0.1	0.1	0.2	0	0	0	0.0	0.0	0.1	0	0	0
Mexico	Lolotla	0.3	0.2	0.3	0	0	1	0.1	0.0	0.1	0	0	0
Mexico	Loma Bonita	0.8	0.6	1.0	4	3	6	0.3	0.2	0.3	1	1	1
Mexico	Lopez	0.3	0.2	0.4	0	0	0	0.1	0.1	0.1	0	0	0
Mexico	Loreto	0.3	0.2	0.4	2	1	2	0.1	0.0	0.1	0	0	1
Mexico	Loreto	0.2	0.1	0.2	0	0	0	0.0	0.0	0.1	0	0	0
Mexico	Los Aldamas	0.1	0.1	0.2	0	0	0	0.0	0.0	0.1	0	0	0
Mexico	Los Cabos	0.1	0.1	0.2	2	2	3	0.0	0.0	0.1	2	1	2
Mexico	Los Herreras	0.1	0.1	0.2	0	0	0	0.0	0.0	0.1	0	0	0
Mexico	Los Ramones	0.1	0.1	0.2	0	0	0	0.0	0.0	0.1	0	0	0
Mexico	Los Reyes	0.4	0.3	0.5	0	0	0	0.1	0.0	0.1	0	0	0
Mexico	Los Reyes	0.4	0.3	0.5	2	1	2	0.1	0.1	0.2	1	1	1
Mexico	Los Reyes De Juarez	0.5	0.4	0.6	1	1	2	0.1	0.1	0.2	0	0	1
Mexico	Luis Moya	0.3	0.2	0.4	0	0	1	0.1	0.0	0.1	0	0	0
Mexico	Luvianos	0.4	0.3	0.5	2	1	2	0.1	0.1	0.1	0	0	0
Mexico	Macuspana	0.3	0.2	0.4	6	5	8	0.1	0.1	0.2	2	1	2
Mexico	Madera	0.3	0.2	0.4	1	1	2	0.1	0.1	0.1	0	0	0
Mexico	Madero	0.4	0.3	0.6	1	1	2	0.1	0.1	0.2	0	0	0
Mexico	Magdalena	0.4	0.3	0.5	0	0	0	0.1	0.1	0.1	0	0	0
Mexico	Magdalena	0.3	0.2	0.4	1	1	1	0.1	0.0	0.1	0	0	0
Mexico	Magdalena	0.3	0.2	0.4	1	1	1	0.1	0.0	0.1	0	0	0
Mexico	Magdalena Apasco	0.6	0.5	0.8	1	0	1	0.2	0.1	0.3	0	0	0
Mexico	Magdalena Jaltepec	0.7	0.5	0.9	0	0	0	0.2	0.2	0.3	0	0	0
Mexico	Magdalena Mixtepec	0.7	0.5	0.9	0	0	0	0.2	0.2	0.3	0	0	0
Mexico	Magdalena Ocotlan	0.7	0.5	0.9	0	0	0	0.2	0.2	0.3	0	0	0
Mexico	Magdalena Penasco	0.7	0.5	0.9	0	0	0	0.2	0.2	0.3	0	0	0
Mexico	Magdalena Teitipac	0.7	0.5	0.9	1	0	1	0.2	0.2	0.3	0	0	0
Mexico	Magdalena Tequisistlan	0.8	0.6	1.1	1	0	1	0.3	0.2	0.3	0	0	0
Mexico	Magdalena Tlacotepec	0.8	0.6	1.1	0	0	0	0.3	0.2	0.3	0	0	0
Mexico	Magdalena Yodocono De Porfirio Diaz	0.6	0.5	0.8	0	0	0	0.2	0.1	0.3	0	0	0
Mexico	Magdalena Zahuatlan	0.7	0.5	0.9	0	0	0	0.2	0.2	0.3	0	0	0
Mexico	Maguarichi	0.4	0.3	0.5	0	0	0	0.1	0.1	0.1	0	0	0
Mexico	Mainero	0.1	0.1	0.2	0	0	0	0.0	0.0	0.1	0	0	0
Mexico	Malinalco	0.4	0.3	0.5	1	1	1	0.1	0.1	0.1	0	0	0
Mexico	Malinaltepec	1.0	0.8	1.3	5	4	7	0.2	0.1	0.3	1	1	1
Mexico	Maltrata	0.3	0.3	0.5	1	1	1	0.1	0.1	0.1	0	0	0

Table 3: Diarrhoea Mortality by Unit *(continued)*

Country	Unit	2000 mortality mean rate (per 10,000)	lower rate (per 10,000)	upper rate (per 10,000)	mean count	lower count	upper count	2017 mortality mean rate (per 10,000)	lower rate (per 10,000)	upper rate (per 10,000)	mean count	lower count	upper count
Mexico	Mama	0.3	0.2	0.4	0	0	0	0.1	0.1	0.1	0	0	0
Mexico	Mani	0.4	0.3	0.5	0	0	0	0.1	0.1	0.2	0	0	0
Mexico	Manlio Fabio Altamirano	0.4	0.3	0.5	1	1	1	0.1	0.1	0.1	0	0	0
Mexico	Manuel Benavides	0.3	0.2	0.4	0	0	0	0.1	0.1	0.1	0	0	0
Mexico	Manuel Doblado	0.4	0.3	0.5	2	1	2	0.1	0.0	0.1	0	0	0
Mexico	Manzanillo	0.3	0.2	0.4	3	2	4	0.1	0.1	0.1	1	1	2
Mexico	Mapastepec	1.1	0.8	1.5	5	4	7	0.3	0.2	0.4	2	2	3
Mexico	Mapimi	0.2	0.2	0.3	1	0	1	0.1	0.0	0.1	0	0	0
Mexico	Maravatio	0.4	0.3	0.5	4	3	5	0.1	0.1	0.2	1	1	2
Mexico	Maravilla Tenejapa	1.5	1.1	1.9	4	3	5	0.4	0.3	0.5	1	1	1
Mexico	Marcos Castellanos	0.4	0.3	0.5	0	0	1	0.1	0.1	0.2	0	0	0
Mexico	Mariano Escobedo	0.3	0.3	0.5	1	1	2	0.1	0.0	0.1	0	0	0
Mexico	Marin	0.1	0.1	0.1	0	0	0	0.0	0.0	0.0	0	0	0
Mexico	Mariscala De Juarez	0.7	0.5	0.8	0	0	0	0.2	0.2	0.3	0	0	0
Mexico	Marquelia	1.1	0.8	1.4	1	1	2	0.2	0.1	0.3	0	0	0
Mexico	Marques De Comillas	1.4	1.1	1.9	2	2	3	0.3	0.3	0.4	1	0	1
Mexico	Martinez De La Torre	0.3	0.2	0.4	4	3	5	0.1	0.1	0.1	1	0	1
Mexico	Martir De Cuilapan	1.1	0.8	1.3	3	2	3	0.2	0.1	0.3	1	0	1
Mexico	Martires De Tacubaya	0.7	0.5	0.9	0	0	0	0.2	0.2	0.3	0	0	0
Mexico	Mascota	0.3	0.2	0.4	0	0	1	0.1	0.0	0.1	0	0	0
Mexico	Matachi	0.3	0.2	0.4	0	0	0	0.1	0.1	0.1	0	0	0
Mexico	Matamoros	0.2	0.1	0.2	3	2	3	0.1	0.0	0.1	1	1	1
Mexico	Matamoros	0.1	0.1	0.2	8	6	10	0.0	0.0	0.1	2	2	3
Mexico	Matamoros	0.3	0.2	0.4	0	0	0	0.1	0.1	0.1	0	0	0
Mexico	Matehuala	0.3	0.3	0.4	3	3	4	0.1	0.1	0.1	1	1	1
Mexico	Matias Romero Avendano	0.8	0.6	1.0	4	3	5	0.3	0.2	0.3	1	1	1
Mexico	Matlapa	0.5	0.4	0.6	2	2	3	0.1	0.1	0.2	0	0	1
Mexico	Maxcanu	0.3	0.2	0.5	1	1	1	0.1	0.1	0.1	0	0	0
Mexico	Mayapan	0.3	0.3	0.5	0	0	0	0.1	0.1	0.2	0	0	0
Mexico	Mazamitla	0.3	0.2	0.3	0	0	0	0.1	0.0	0.1	0	0	0
Mexico	Mazapa De Madero	1.2	0.9	1.6	2	1	2	0.3	0.2	0.4	1	0	1
Mexico	Mazapil	0.3	0.2	0.4	0	0	1	0.1	0.0	0.1	0	0	0
Mexico	Mazapiltepec De Juarez	0.5	0.4	0.7	0	0	0	0.1	0.1	0.2	0	0	0
Mexico	Mazatan	0.3	0.2	0.4	0	0	0	0.1	0.1	0.1	0	0	0
Mexico	Mazatan	1.2	0.9	1.6	3	2	4	0.3	0.3	0.4	1	1	1
Mexico	Mazatecochco De Jose Maria Morelos	0.3	0.2	0.4	0	0	0	0.1	0.1	0.1	0	0	0
Mexico	Mazatepec	0.3	0.2	0.4	0	0	0	0.1	0.1	0.1	0	0	0
Mexico	Mazatlan	0.2	0.1	0.2	7	5	9	0.1	0.0	0.1	2	1	3
Mexico	Mazatlan Villa De Flores	0.8	0.6	1.0	1	1	2	0.3	0.2	0.3	0	0	0
Mexico	Mecatlan	0.4	0.3	0.5	1	1	1	0.1	0.1	0.1	0	0	0
Mexico	Mecayapan	0.4	0.3	0.6	1	1	1	0.1	0.1	0.1	0	0	0
Mexico	Medellin	0.4	0.3	0.5	2	1	2	0.1	0.1	0.1	1	1	1
Mexico	Melchor Ocampo	0.3	0.2	0.4	0	0	0	0.1	0.0	0.1	0	0	0
Mexico	Melchor Ocampo	0.1	0.1	0.2	0	0	0	0.0	0.0	0.1	0	0	0

Table 3: Diarrhoea Mortality by Unit *(continued)*

Country	Unit	2000 mortality mean rate (per 10,000)	lower rate (per 10,000)	upper rate (per 10,000)	mean count	lower count	upper count	2017 mortality mean rate (per 10,000)	lower rate (per 10,000)	upper rate (per 10,000)	mean count	lower count	upper count
Mexico	Melchor Ocampo	0.3	0.2	0.4	1	1	2	0.1	0.1	0.1	1	0	1
Mexico	Mendez	0.1	0.1	0.2	0	0	0	0.1	0.0	0.1	0	0	0
Mexico	Meoqui	0.3	0.2	0.4	2	1	2	0.1	0.1	0.1	0	0	1
Mexico	Merida	0.3	0.2	0.4	21	15	28	0.1	0.1	0.1	7	5	9
Mexico	Mesones Hidalgo	0.8	0.6	1.0	0	0	1	0.3	0.2	0.4	0	0	0
Mexico	Metapa	1.2	0.9	1.6	1	1	1	0.3	0.3	0.5	0	0	0
Mexico	Metepec	0.2	0.2	0.3	0	0	0	0.1	0.0	0.1	0	0	0
Mexico	Metepec	0.3	0.3	0.4	7	5	8	0.1	0.1	0.1	2	1	2
Mexico	Metlatonoc	1.0	0.8	1.3	4	3	5	0.2	0.1	0.3	1	1	1
Mexico	Metztitlan	0.2	0.2	0.3	0	0	1	0.1	0.0	0.1	0	0	0
Mexico	Mexicali	0.2	0.1	0.2	12	8	18	0.0	0.0	0.1	4	3	6
Mexico	Mexicaltzingo	0.3	0.3	0.4	0	0	1	0.1	0.1	0.1	0	0	0
Mexico	Mexquitic De Carmona	0.4	0.3	0.5	3	2	4	0.1	0.1	0.2	1	1	1
Mexico	Mexticacan	0.2	0.2	0.3	0	0	0	0.1	0.0	0.1	0	0	0
Mexico	Mezquital	0.3	0.2	0.4	1	1	1	0.1	0.0	0.1	0	0	1
Mexico	Mezquital Del Oro	0.4	0.3	0.4	0	0	0	0.1	0.1	0.1	0	0	0
Mexico	Mezquitic	0.3	0.2	0.4	1	1	1	0.1	0.0	0.1	0	0	0
Mexico	Miacatlan	0.3	0.2	0.4	1	1	1	0.1	0.1	0.1	0	0	0
Mexico	Miahuatlan	0.3	0.2	0.4	0	0	0	0.1	0.0	0.1	0	0	0
Mexico	Miahuatlan De Porfirio Diaz	0.7	0.5	0.9	3	2	4	0.2	0.2	0.3	1	1	2
Mexico	Mier	0.2	0.1	0.2	0	0	0	0.0	0.0	0.1	0	0	0
Mexico	Mier Y Noriega	0.1	0.1	0.2	0	0	0	0.0	0.0	0.0	0	0	0
Mexico	Miguel Aleman	0.1	0.1	0.2	0	0	1	0.0	0.0	0.1	0	0	0
Mexico	Miguel Auza	0.3	0.2	0.4	1	1	1	0.1	0.0	0.1	0	0	0
Mexico	Miguel Hidalgo	0.2	0.1	0.2	6	5	8	0.1	0.0	0.1	2	1	3
Mexico	Milpa Alta	0.1	0.1	0.2	2	2	3	0.1	0.0	0.1	1	1	1
Mexico	Mina	0.1	0.1	0.1	0	0	0	0.0	0.0	0.0	0	0	0
Mexico	Minatitlan	0.4	0.3	0.5	9	7	12	0.1	0.1	0.1	1	1	2
Mexico	Minatitlan	0.3	0.2	0.3	0	0	0	0.1	0.0	0.1	0	0	0
Mexico	Mineral De La Reforma	0.2	0.2	0.3	2	2	3	0.1	0.0	0.1	1	1	1
Mexico	Mineral Del Chico	0.2	0.2	0.3	0	0	0	0.1	0.0	0.1	0	0	0
Mexico	Mineral Del Monte	0.2	0.1	0.2	0	0	0	0.1	0.0	0.1	0	0	0
Mexico	Miquihuana	0.1	0.1	0.2	0	0	0	0.0	0.0	0.1	0	0	0
Mexico	Misantla	0.3	0.3	0.5	3	2	4	0.1	0.1	0.1	0	0	1
Mexico	Mitontic	1.1	0.8	1.5	1	1	2	0.3	0.2	0.4	1	0	1
Mexico	Mixistlan De La Reforma	0.7	0.6	1.0	0	0	0	0.2	0.2	0.3	0	0	0
Mexico	Mixquiahuala De Juarez	0.2	0.2	0.3	1	1	1	0.1	0.0	0.1	0	0	0
Mexico	Mixtla	0.5	0.4	0.6	0	0	0	0.1	0.1	0.2	0	0	0
Mexico	Mixtla De Altamirano	0.4	0.3	0.5	1	0	1	0.1	0.1	0.1	0	0	0
Mexico	Mixtlan	0.3	0.2	0.3	0	0	0	0.1	0.0	0.1	0	0	0
Mexico	Mochitlan	1.0	0.8	1.3	2	1	2	0.2	0.1	0.3	0	0	0
Mexico	Mococha	0.3	0.2	0.4	0	0	0	0.1	0.1	0.1	0	0	0
Mexico	Mocorito	0.2	0.1	0.2	1	1	1	0.1	0.0	0.1	0	0	0
Mexico	Moctezuma	0.4	0.3	0.5	1	1	1	0.1	0.1	0.1	0	0	0
Mexico	Moctezuma	0.3	0.2	0.4	0	0	0	0.1	0.1	0.1	0	0	0
Mexico	Molango De Escamilla	0.2	0.2	0.3	0	0	0	0.1	0.0	0.1	0	0	0
Mexico	Molcaxac	0.6	0.4	0.7	0	0	1	0.2	0.1	0.2	0	0	0
Mexico	Moloacan	0.4	0.3	0.6	1	1	1	0.1	0.1	0.1	0	0	0
Mexico	Momax	0.3	0.2	0.4	0	0	0	0.1	0.1	0.1	0	0	0
Mexico	Monclova	0.2	0.1	0.3	5	3	6	0.1	0.0	0.1	2	1	2

Table 3: Diarrhoea Mortality by Unit *(continued)*

Country	Unit	2000 mortality mean rate (per 10,000)	lower rate (per 10,000)	upper rate (per 10,000)	mean count	lower count	upper count	2017 mortality mean rate (per 10,000)	lower rate (per 10,000)	upper rate (per 10,000)	mean count	lower count	upper count
Mexico	Monjas	0.7	0.5	0.9	0	0	0	0.2	0.2	0.3	0	0	0
Mexico	Monte Escobedo	0.3	0.2	0.4	0	0	0	0.1	0.0	0.1	0	0	0
Mexico	Montecristo De Guerrero	1.2	0.9	1.7	1	1	2	0.3	0.2	0.4	0	0	0
Mexico	Montemorelos	0.1	0.1	0.1	1	1	1	0.0	0.0	0.0	0	0	0
Mexico	Monterrey	0.1	0.1	0.1	12	9	15	0.0	0.0	0.0	2	1	3
Mexico	Morelia	0.4	0.3	0.5	35	27	43	0.1	0.1	0.2	8	5	11
Mexico	Morelos	0.2	0.2	0.3	0	0	0	0.1	0.0	0.1	0	0	0
Mexico	Morelos	0.3	0.2	0.4	0	0	1	0.1	0.0	0.1	0	0	0
Mexico	Morelos	0.4	0.3	0.5	0	0	0	0.1	0.1	0.1	0	0	0
Mexico	Morelos	0.4	0.3	0.5	0	0	1	0.1	0.1	0.2	0	0	0
Mexico	Morelos	0.3	0.3	0.4	1	1	1	0.1	0.1	0.1	0	0	0
Mexico	Moris	0.4	0.3	0.5	0	0	0	0.1	0.1	0.1	0	0	0
Mexico	Moroleon	0.4	0.3	0.5	2	1	2	0.1	0.1	0.1	0	0	0
Mexico	Motozintla	1.2	0.8	1.6	10	7	13	0.3	0.2	0.4	3	3	4
Mexico	Motul	0.3	0.2	0.4	1	1	1	0.1	0.1	0.1	0	0	0
Mexico	Moyahua De Estrada	0.3	0.3	0.4	0	0	0	0.1	0.1	0.1	0	0	0
Mexico	Mugica	0.5	0.4	0.6	3	2	4	0.1	0.1	0.2	1	1	1
Mexico	Mulege	0.2	0.1	0.3	1	1	1	0.0	0.0	0.1	0	0	0
Mexico	Muna	0.3	0.2	0.4	0	0	1	0.1	0.1	0.1	0	0	0
Mexico	Munoz De Domingo Arenas	0.3	0.2	0.4	0	0	0	0.1	0.1	0.1	0	0	0
Mexico	Muxupip	0.3	0.2	0.4	0	0	0	0.1	0.1	0.1	0	0	0
Mexico	Muzquiz	0.2	0.2	0.3	1	1	2	0.1	0.0	0.1	0	0	1
Mexico	Nacajuca	0.4	0.3	0.5	3	2	4	0.1	0.1	0.2	2	1	3
Mexico	Naco	0.3	0.2	0.4	0	0	0	0.1	0.0	0.1	0	0	0
Mexico	Nacori Chico	0.3	0.2	0.4	0	0	0	0.1	0.1	0.1	0	0	0
Mexico	Nacozari De Garcia	0.3	0.2	0.4	0	0	1	0.1	0.0	0.1	0	0	0
Mexico	Nadadores	0.2	0.1	0.3	0	0	0	0.1	0.0	0.1	0	0	0
Mexico	Nahuatzen	0.4	0.3	0.5	1	1	2	0.1	0.1	0.2	0	0	1
Mexico	Namiquipa	0.3	0.2	0.4	1	0	1	0.1	0.1	0.1	0	0	0
Mexico	Nanacamilpa De Mariano Arista	0.3	0.2	0.4	0	0	0	0.1	0.1	0.1	0	0	0
Mexico	Nanchital De Lazaro Cardenas Del Rio	0.4	0.3	0.5	2	1	2	0.1	0.1	0.1	0	0	0
Mexico	Naolinco	0.3	0.3	0.5	1	1	1	0.1	0.1	0.1	0	0	0
Mexico	Naranjal	0.4	0.3	0.5	0	0	0	0.1	0.1	0.1	0	0	0
Mexico	Naranjos Amatlan	0.4	0.3	0.5	1	1	1	0.1	0.1	0.1	0	0	0
Mexico	Natividad	0.7	0.5	0.9	0	0	0	0.2	0.2	0.3	0	0	0
Mexico	Nativitas	0.3	0.2	0.4	1	1	1	0.1	0.1	0.1	0	0	0
Mexico	Naucalpan De Juarez	0.3	0.3	0.4	27	21	34	0.1	0.1	0.1	5	4	7
Mexico	Naupan	0.5	0.4	0.7	1	1	1	0.1	0.1	0.2	0	0	0
Mexico	Nautla	0.3	0.3	0.4	1	0	1	0.1	0.0	0.1	0	0	0
Mexico	Nauzontla	0.5	0.4	0.7	0	0	0	0.1	0.1	0.2	0	0	0
Mexico	Nava	0.2	0.2	0.3	1	1	1	0.1	0.0	0.1	0	0	0
Mexico	Navojoa	0.3	0.2	0.4	4	3	6	0.1	0.1	0.1	1	1	2
Mexico	Navolato	0.2	0.1	0.2	4	3	5	0.1	0.0	0.1	1	1	1
Mexico	Nazareno Etla	0.6	0.5	0.8	0	0	0	0.2	0.2	0.3	0	0	0
Mexico	Nazas	0.3	0.2	0.3	0	0	0	0.1	0.0	0.1	0	0	0
Mexico	Nealtican	0.5	0.4	0.6	1	0	1	0.2	0.1	0.2	0	0	0
Mexico	Nejapa De Madero	0.8	0.6	1.0	1	1	1	0.3	0.2	0.3	0	0	0
Mexico	Nextlalpan	0.3	0.2	0.4	1	1	2	0.1	0.1	0.1	1	0	1
Mexico	Nezahualcoyotl	0.4	0.3	0.4	36	27	44	0.1	0.1	0.1	7	5	9
Mexico	Nicolas Bravo	0.5	0.4	0.7	0	0	1	0.1	0.1	0.2	0	0	0

Table 3: Diarrhoea Mortality by Unit *(continued)*

Country	Unit	2000 mortality mean rate (per 10,000)	lower rate (per 10,000)	upper rate (per 10,000)	mean count	lower count	upper count	2017 mortality mean rate (per 10,000)	lower rate (per 10,000)	upper rate (per 10,000)	mean count	lower count	upper count
Mexico	Nicolas Flores	0.2	0.2	0.3	0	0	0	0.1	0.0	0.1	0	0	0
Mexico	Nicolas Romero	0.3	0.3	0.4	9	7	11	0.1	0.1	0.1	3	2	4
Mexico	Nicolas Ruiz	1.2	0.9	1.6	0	0	0	0.3	0.2	0.4	0	0	0
Mexico	Nochistlan De Mejia	0.3	0.2	0.4	1	1	1	0.1	0.0	0.1	0	0	0
Mexico	Nocupetaro	0.5	0.4	0.6	1	0	1	0.1	0.1	0.2	0	0	0
Mexico	Nogales	0.3	0.2	0.4	1	1	2	0.1	0.0	0.1	0	0	0
Mexico	Nogales	0.2	0.2	0.3	7	5	9	0.1	0.0	0.1	2	1	2
Mexico	Nombre De Dios	0.2	0.2	0.3	1	0	1	0.1	0.0	0.1	0	0	0
Mexico	Nonoava	0.3	0.2	0.4	0	0	0	0.1	0.1	0.1	0	0	0
Mexico	Nopala De Villagran	0.2	0.2	0.3	1	0	1	0.1	0.0	0.1	0	0	0
Mexico	Nopaltepec	0.3	0.2	0.4	0	0	0	0.1	0.1	0.1	0	0	0
Mexico	Nopalucan	0.5	0.4	0.6	2	2	3	0.1	0.1	0.2	1	1	1
Mexico	Noria De Angeles	0.3	0.2	0.4	1	0	1	0.1	0.0	0.1	0	0	0
Mexico	Nuevo Casas Grandes	0.3	0.2	0.4	2	1	2	0.1	0.1	0.1	1	0	1
Mexico	Nuevo Ideal	0.2	0.2	0.3	1	1	1	0.1	0.0	0.1	0	0	0
Mexico	Nuevo Laredo	0.1	0.1	0.2	6	5	9	0.0	0.0	0.1	2	1	3
Mexico	Nuevo Morelos	0.1	0.1	0.2	0	0	0	0.0	0.0	0.1	0	0	0
Mexico	Nuevo Parangaricutiro	0.4	0.3	0.6	1	1	1	0.1	0.1	0.2	0	0	1
Mexico	Nuevo Urecho	0.5	0.3	0.6	1	0	1	0.1	0.1	0.2	0	0	0
Mexico	Nuevo Zoquiapam	0.7	0.5	0.9	0	0	0	0.2	0.2	0.3	0	0	0
Mexico	Numaran	0.4	0.3	0.5	0	0	1	0.1	0.1	0.2	0	0	0
Mexico	Oaxaca De Juarez	0.6	0.5	0.8	14	11	18	0.2	0.1	0.3	4	3	5
Mexico	Ocampo	0.2	0.2	0.3	0	0	0	0.1	0.0	0.1	0	0	0
Mexico	Ocampo	0.3	0.2	0.4	1	1	1	0.1	0.0	0.1	0	0	0
Mexico	Ocampo	0.2	0.1	0.3	0	0	0	0.1	0.0	0.1	0	0	0
Mexico	Ocampo	0.1	0.1	0.2	0	0	0	0.0	0.0	0.1	0	0	0
Mexico	Ocampo	0.3	0.2	0.4	0	0	0	0.1	0.1	0.1	0	0	0
Mexico	Ocampo	0.4	0.3	0.5	2	1	2	0.1	0.1	0.2	0	0	1
Mexico	Ocosingo	1.2	0.9	1.6	36	27	47	0.3	0.2	0.4	11	8	14
Mexico	Ocotepec	0.5	0.4	0.7	0	0	0	0.1	0.1	0.2	0	0	0
Mexico	Ocotepec	1.1	0.8	1.4	2	1	2	0.3	0.2	0.4	1	0	1
Mexico	Ocotlan	0.2	0.2	0.3	2	2	3	0.1	0.0	0.1	0	0	1
Mexico	Ocotlan De Morelos	0.7	0.5	0.9	2	1	2	0.2	0.2	0.3	0	0	0
Mexico	Ocoyoacac	0.3	0.3	0.4	2	1	2	0.1	0.1	0.1	1	0	1
Mexico	Ocoyucan	0.5	0.4	0.6	3	2	4	0.1	0.1	0.2	1	1	1
Mexico	Ocozocoautla De Espinosa	1.1	0.8	1.5	10	8	14	0.3	0.2	0.4	4	3	5
Mexico	Ocuilan	0.4	0.3	0.5	1	1	1	0.1	0.1	0.1	0	0	1
Mexico	Ocuituco	0.3	0.2	0.4	1	1	1	0.1	0.1	0.1	0	0	0
Mexico	Ojinaga	0.3	0.2	0.4	0	0	1	0.1	0.1	0.1	0	0	0
Mexico	Ojocaliente	0.3	0.2	0.4	2	1	2	0.1	0.0	0.1	0	0	0
Mexico	Ojuelos De Jalisco	0.2	0.2	0.3	1	1	1	0.0	0.0	0.1	0	0	0
Mexico	Olinala	1.0	0.8	1.3	4	3	5	0.2	0.1	0.3	1	1	1
Mexico	Olintla	0.6	0.4	0.7	1	1	1	0.2	0.1	0.2	0	0	0
Mexico	Oluta	0.4	0.3	0.6	2	2	3	0.1	0.1	0.1	0	0	0
Mexico	Omealca	0.4	0.3	0.5	1	1	2	0.1	0.1	0.1	0	0	0
Mexico	Ometepec	1.1	0.8	1.4	12	8	15	0.2	0.1	0.3	2	1	2
Mexico	Omitlan De Juarez	0.2	0.2	0.3	0	0	0	0.1	0.0	0.1	0	0	0
Mexico	Onavas	0.3	0.2	0.4	0	0	0	0.1	0.1	0.1	0	0	0
Mexico	Opichen	0.3	0.2	0.4	0	0	0	0.1	0.1	0.1	0	0	0
Mexico	Opodepe	0.3	0.2	0.4	0	0	0	0.1	0.0	0.1	0	0	0

Table 3: Diarrhoea Mortality by Unit *(continued)*

Country	Unit	2000 mortality mean rate (per 10,000)	lower rate (per 10,000)	upper rate (per 10,000)	mean count	lower count	upper count	2017 mortality mean rate (per 10,000)	lower rate (per 10,000)	upper rate (per 10,000)	mean count	lower count	upper count
Mexico	Oquitoa	0.3	0.2	0.4	0	0	0	0.1	0.0	0.1	0	0	0
Mexico	Oriental	0.5	0.4	0.6	1	1	2	0.1	0.1	0.2	0	0	0
Mexico	Orizaba	0.3	0.2	0.4	4	3	5	0.1	0.0	0.1	1	0	1
Mexico	Ostuacan	1.1	0.8	1.5	3	2	4	0.3	0.2	0.4	1	1	1
Mexico	Osumacinta	1.0	0.8	1.4	1	0	1	0.3	0.2	0.4	0	0	0
Mexico	Otaez	0.2	0.2	0.3	0	0	0	0.1	0.0	0.1	0	0	0
Mexico	Otatitlan	0.5	0.3	0.6	0	0	0	0.1	0.1	0.1	0	0	0
Mexico	Oteapan	0.4	0.3	0.6	1	0	1	0.1	0.1	0.1	0	0	0
Mexico	Othon P. Blanco	0.3	0.2	0.4	7	5	9	0.1	0.1	0.1	2	1	3
Mexico	Otumba	0.3	0.2	0.4	1	1	1	0.1	0.1	0.1	0	0	0
Mexico	Otzoloapan	0.4	0.3	0.5	0	0	0	0.1	0.1	0.1	0	0	0
Mexico	Otzolotepec	0.3	0.3	0.4	2	2	3	0.1	0.1	0.1	1	0	1
Mexico	Oxchuc	1.0	0.8	1.4	6	4	8	0.3	0.2	0.4	2	1	3
Mexico	Oxkutzcab	0.4	0.3	0.5	1	1	2	0.1	0.1	0.2	0	0	1
Mexico	Ozuluama De Mascarenas	0.4	0.3	0.5	1	1	1	0.1	0.1	0.1	0	0	0
Mexico	Ozumba	0.3	0.2	0.4	1	1	1	0.1	0.1	0.1	0	0	0
Mexico	Pabellon De Arteaga	0.2	0.2	0.3	1	1	1	0.1	0.1	0.1	0	0	1
Mexico	Pachuca De Soto	0.2	0.2	0.3	8	6	10	0.1	0.0	0.1	1	1	2
Mexico	Pacula	0.2	0.2	0.3	0	0	0	0.1	0.0	0.1	0	0	0
Mexico	Padilla	0.1	0.1	0.2	0	0	0	0.0	0.0	0.1	0	0	0
Mexico	Pahuatlan	0.5	0.4	0.7	1	1	2	0.1	0.1	0.2	0	0	1
Mexico	Pajacuaran	0.4	0.3	0.5	1	1	1	0.1	0.1	0.2	0	0	0
Mexico	Pajapan	0.4	0.3	0.6	1	1	1	0.1	0.1	0.1	0	0	0
Mexico	Palenque	1.2	0.9	1.6	17	13	23	0.3	0.2	0.4	5	4	7
Mexico	Palizada	0.4	0.3	0.5	0	0	1	0.1	0.1	0.1	0	0	0
Mexico	Palmar De Bravo	0.5	0.4	0.7	4	3	6	0.2	0.1	0.2	1	1	2
Mexico	Palmillas	0.1	0.1	0.2	0	0	0	0.0	0.0	0.1	0	0	0
Mexico	Panaba	0.4	0.3	0.5	0	0	0	0.1	0.1	0.2	0	0	0
Mexico	Panindicuaro	0.4	0.3	0.5	1	1	1	0.1	0.1	0.2	0	0	0
Mexico	Panotla	0.3	0.2	0.4	1	1	1	0.1	0.1	0.1	0	0	0
Mexico	Pantelho	1.2	0.9	1.6	5	3	6	0.3	0.2	0.4	1	1	2
Mexico	Pantepec	0.6	0.4	0.7	2	1	2	0.2	0.1	0.2	0	0	0
Mexico	Pantepec	1.1	0.8	1.4	2	1	2	0.3	0.2	0.4	1	0	1
Mexico	Panuco	0.4	0.3	0.5	4	3	5	0.1	0.1	0.1	1	1	1
Mexico	Panuco	0.3	0.2	0.4	1	0	1	0.1	0.0	0.1	0	0	0
Mexico	Panuco De Coronado	0.2	0.2	0.3	0	0	0	0.1	0.0	0.1	0	0	0
Mexico	Papalotla	0.3	0.2	0.4	0	0	0	0.1	0.1	0.1	0	0	0
Mexico	Papalotla De Xicohtencatl	0.3	0.2	0.4	1	1	1	0.1	0.1	0.1	0	0	0
Mexico	Papantla	0.3	0.2	0.4	6	5	8	0.1	0.1	0.1	1	1	2
Mexico	Paracho	0.4	0.3	0.5	2	1	2	0.1	0.1	0.2	1	0	1
Mexico	Paracuaro	0.5	0.4	0.6	1	1	2	0.1	0.1	0.2	0	0	0
Mexico	Paraiso	0.3	0.2	0.4	3	2	4	0.1	0.1	0.2	1	1	1
Mexico	Paras	0.1	0.1	0.2	0	0	0	0.0	0.0	0.1	0	0	0
Mexico	Parras	0.2	0.1	0.2	1	1	1	0.1	0.0	0.1	0	0	0
Mexico	Paso De Ovejas	0.4	0.3	0.5	2	1	2	0.1	0.1	0.1	0	0	0
Mexico	Paso Del Macho	0.4	0.3	0.5	1	1	2	0.1	0.1	0.1	0	0	0
Mexico	Patzcuaro	0.4	0.3	0.5	5	4	6	0.1	0.1	0.2	1	1	2
Mexico	Pedro Ascencio Alquisiras	1.0	0.8	1.2	1	1	1	0.2	0.1	0.3	0	0	0
Mexico	Pedro Escobedo	0.2	0.2	0.3	2	1	2	0.1	0.1	0.1	1	0	1
Mexico	Penamiller	0.3	0.2	0.3	1	0	1	0.1	0.1	0.1	0	0	0
Mexico	Penjamillo	0.4	0.3	0.5	1	1	1	0.1	0.1	0.2	0	0	0
Mexico	Penjamo	0.4	0.3	0.5	8	6	10	0.1	0.1	0.1	1	1	2
Mexico	Penon Blanco	0.2	0.2	0.3	0	0	0	0.1	0.0	0.1	0	0	0
Mexico	Periban	0.4	0.3	0.5	2	1	2	0.1	0.1	0.2	0	0	1
Mexico	Perote	0.3	0.2	0.4	3	2	4	0.1	0.0	0.1	1	0	1

Table 3: Diarrhoea Mortality by Unit *(continued)*

Country	Unit	2000 mortality mean rate (per 10,000)	lower rate (per 10,000)	upper rate (per 10,000)	mean count	lower count	upper count	2017 mortality mean rate (per 10,000)	lower rate (per 10,000)	upper rate (per 10,000)	mean count	lower count	upper count
Mexico	Pesqueria	0.1	0.1	0.1	0	0	0	0.0	0.0	0.0	0	0	0
Mexico	Petatlan	1.0	0.8	1.3	7	5	9	0.2	0.1	0.3	1	1	1
Mexico	Petlalcingo	0.6	0.4	0.7	1	1	1	0.2	0.1	0.2	0	0	0
Mexico	Peto	0.4	0.3	0.5	1	1	2	0.1	0.1	0.2	0	0	0
Mexico	Piaxtla	0.6	0.5	0.8	0	0	0	0.2	0.1	0.2	0	0	0
Mexico	Pichucalco	1.1	0.9	1.5	5	4	6	0.3	0.2	0.4	1	1	2
Mexico	Piedras Negras	0.2	0.2	0.3	5	3	6	0.1	0.0	0.1	1	1	2
Mexico	Pihuamo	0.3	0.2	0.4	0	0	0	0.1	0.0	0.1	0	0	0
Mexico	Pijijiapan	1.2	0.9	1.6	8	6	10	0.3	0.2	0.4	2	1	3
Mexico	Pilcaya	0.9	0.7	1.1	1	1	2	0.2	0.1	0.2	0	0	0
Mexico	Pinal De Amoles	0.3	0.2	0.3	1	1	1	0.1	0.1	0.1	0	0	0
Mexico	Pinos	0.3	0.2	0.4	3	2	4	0.1	0.0	0.1	0	0	1
Mexico	Pinotepa De Don Luis	0.9	0.6	1.1	1	1	1	0.3	0.2	0.4	0	0	0
Mexico	Pisaflores	0.3	0.2	0.3	1	1	1	0.1	0.0	0.1	0	0	0
Mexico	Pitiquito	0.3	0.2	0.4	0	0	0	0.1	0.1	0.1	0	0	0
Mexico	Platon Sanchez	0.4	0.3	0.5	1	1	1	0.1	0.1	0.1	0	0	0
Mexico	Playa Vicente	0.4	0.3	0.6	2	2	3	0.1	0.1	0.1	0	0	0
Mexico	Playas De Rosarito	0.2	0.1	0.2	1	1	2	0.0	0.0	0.1	0	0	1
Mexico	Pluma Hidalgo	0.8	0.6	1.0	1	1	1	0.2	0.2	0.3	0	0	0
Mexico	Poanas	0.2	0.2	0.3	1	0	1	0.1	0.0	0.1	0	0	0
Mexico	Polotitlan	0.3	0.2	0.4	0	0	1	0.1	0.1	0.1	0	0	0
Mexico	Poncitlan	0.3	0.2	0.3	2	2	3	0.1	0.0	0.1	0	0	1
Mexico	Poza Rica De Hidalgo	0.3	0.2	0.4	6	4	7	0.1	0.0	0.1	1	1	1
Mexico	Praxedis G. Guerrero	0.3	0.2	0.5	1	0	1	0.1	0.1	0.1	0	0	0
Mexico	Progreso	0.2	0.2	0.3	0	0	0	0.1	0.0	0.1	0	0	0
Mexico	Progreso	0.3	0.2	0.4	1	1	1	0.1	0.1	0.1	0	0	1
Mexico	Progreso De Obregon	0.2	0.2	0.3	0	0	0	0.1	0.0	0.1	0	0	0
Mexico	Puebla	0.5	0.4	0.6	86	65	111	0.2	0.1	0.2	23	15	31
Mexico	Pueblo Nuevo	0.4	0.3	0.5	0	0	1	0.1	0.0	0.1	0	0	0
Mexico	Pueblo Nuevo	0.2	0.2	0.3	2	2	3	0.1	0.0	0.1	0	0	1
Mexico	Pueblo Nuevo Solistahuacan	1.1	0.8	1.4	5	4	6	0.3	0.2	0.4	1	1	2
Mexico	Pueblo Viejo	0.4	0.3	0.5	4	3	6	0.1	0.1	0.1	1	0	1
Mexico	Puente De Ixtla	0.3	0.2	0.4	2	2	3	0.1	0.1	0.1	1	0	1
Mexico	Puente Nacional	0.4	0.3	0.5	1	1	1	0.1	0.1	0.1	0	0	0
Mexico	Puerto Penasco	0.3	0.2	0.4	1	1	2	0.1	0.1	0.1	1	0	1
Mexico	Puerto Vallarta	0.3	0.2	0.3	7	5	9	0.1	0.0	0.1	2	1	2
Mexico	Pungarabato	1.0	0.7	1.2	5	4	6	0.2	0.1	0.3	1	0	1
Mexico	Purepero	0.4	0.3	0.5	1	1	1	0.1	0.1	0.2	0	0	0
Mexico	Purisima Del Rincon	0.3	0.2	0.4	2	2	3	0.1	0.0	0.1	1	0	1
Mexico	Puruandiro	0.4	0.3	0.5	4	3	5	0.1	0.1	0.2	1	1	1
Mexico	Putla Villa De Guerrero	0.7	0.6	0.9	3	2	3	0.2	0.2	0.3	1	0	1
Mexico	Quecholac	0.5	0.4	0.7	3	3	4	0.1	0.1	0.2	1	1	1
Mexico	Quechultenango	1.1	0.8	1.4	6	5	8	0.2	0.1	0.3	1	1	1
Mexico	Querendaro	0.4	0.3	0.5	1	1	1	0.1	0.1	0.2	0	0	0
Mexico	Queretaro	0.2	0.2	0.3	19	14	23	0.1	0.1	0.1	7	4	9
Mexico	Quimixtlan	0.5	0.4	0.7	2	1	3	0.1	0.1	0.2	0	0	1
Mexico	Quintana Roo	0.3	0.3	0.4	0	0	0	0.1	0.1	0.1	0	0	0
Mexico	Quiriego	0.3	0.2	0.4	0	0	0	0.1	0.1	0.1	0	0	0
Mexico	Quiroga	0.4	0.3	0.5	1	1	1	0.1	0.1	0.2	0	0	0
Mexico	Quitupan	0.3	0.2	0.4	0	0	0	0.1	0.0	0.1	0	0	0
Mexico	Rafael Delgado	0.4	0.3	0.5	1	1	1	0.1	0.0	0.1	0	0	0
Mexico	Rafael Lara Grajales	0.5	0.3	0.6	0	0	0	0.1	0.1	0.2	0	0	0

Table 3: Diarrhoea Mortality by Unit *(continued)*

Country	Unit	2000 mortality mean rate (per 10,000)	lower rate (per 10,000)	upper rate (per 10,000)	mean count	lower count	upper count	2017 mortality mean rate (per 10,000)	lower rate (per 10,000)	upper rate (per 10,000)	mean count	lower count	upper count
Mexico	Rafael Lucio	0.3	0.3	0.4	0	0	0	0.1	0.0	0.1	0	0	0
Mexico	Ramos Arizpe	0.2	0.1	0.2	1	1	1	0.1	0.0	0.1	1	0	1
Mexico	Rayon	0.4	0.3	0.5	1	1	1	0.1	0.1	0.2	0	0	0
Mexico	Rayon	0.3	0.2	0.4	0	0	0	0.1	0.0	0.1	0	0	0
Mexico	Rayon	0.4	0.3	0.4	1	0	1	0.1	0.1	0.1	0	0	0
Mexico	Rayon	1.1	0.8	1.5	2	1	3	0.3	0.2	0.4	0	0	1
Mexico	Rayones	0.1	0.1	0.2	0	0	0	0.0	0.0	0.1	0	0	0
Mexico	Reforma	1.0	0.8	1.4	5	4	7	0.3	0.2	0.4	2	1	2
Mexico	Reforma De Pineda	0.8	0.6	1.1	0	0	0	0.3	0.2	0.4	0	0	0
Mexico	Reyes Etla	0.6	0.5	0.8	0	0	1	0.2	0.1	0.3	0	0	0
Mexico	Reynosa	0.1	0.1	0.2	9	6	12	0.0	0.0	0.1	4	3	5
Mexico	Rincon De Romos	0.2	0.2	0.3	1	1	2	0.1	0.1	0.1	1	0	1
Mexico	Rio Blanco	0.3	0.2	0.4	0	0	1	0.1	0.0	0.1	0	0	0
Mexico	Rio Bravo	0.2	0.1	0.2	2	2	3	0.1	0.0	0.1	1	1	1
Mexico	Rio Grande	0.3	0.2	0.4	2	2	3	0.1	0.0	0.1	0	0	1
Mexico	Rio Lagartos	0.3	0.3	0.5	0	0	0	0.1	0.1	0.2	0	0	0
Mexico	Rioverde	0.4	0.3	0.5	4	3	5	0.1	0.1	0.2	1	1	2
Mexico	Riva Palacio	0.3	0.2	0.4	0	0	0	0.1	0.1	0.1	0	0	0
Mexico	Rodeo	0.3	0.2	0.3	0	0	0	0.1	0.0	0.1	0	0	0
Mexico	Rojas De Cuauhtemoc	0.7	0.5	0.9	0	0	0	0.2	0.2	0.3	0	0	0
Mexico	Romita	0.4	0.3	0.5	3	2	3	0.1	0.0	0.1	1	0	1
Mexico	Rosales	0.3	0.2	0.4	1	0	1	0.1	0.1	0.1	0	0	0
Mexico	Rosamorada	0.3	0.2	0.4	1	1	2	0.1	0.0	0.1	0	0	0
Mexico	Rosario	0.2	0.1	0.2	1	1	1	0.1	0.0	0.1	0	0	0
Mexico	Rosario	0.3	0.2	0.4	0	0	0	0.1	0.1	0.1	0	0	0
Mexico	Rosario	0.3	0.2	0.4	0	0	0	0.1	0.1	0.1	0	0	0
Mexico	Ruiz	0.3	0.2	0.4	1	1	1	0.1	0.0	0.1	0	0	0
Mexico	Sabanilla	1.2	0.9	1.5	5	3	6	0.3	0.2	0.4	1	1	2
Mexico	Sabinas	0.2	0.2	0.3	1	1	2	0.1	0.0	0.1	0	0	1
Mexico	Sabinas Hidalgo	0.1	0.1	0.1	0	0	0	0.0	0.0	0.0	0	0	0
Mexico	Sacalum	0.3	0.2	0.5	0	0	0	0.1	0.1	0.2	0	0	0
Mexico	Sacramento	0.2	0.2	0.3	0	0	0	0.1	0.0	0.1	0	0	0
Mexico	Sahuaripa	0.3	0.2	0.4	0	0	0	0.1	0.1	0.1	0	0	0
Mexico	Sahuayo	0.4	0.3	0.5	3	2	3	0.1	0.1	0.2	1	1	1
Mexico	Sain Alto	0.3	0.2	0.4	1	1	1	0.1	0.0	0.1	0	0	0
Mexico	Salamanca	0.4	0.3	0.4	10	7	12	0.1	0.0	0.1	2	1	3
Mexico	Salina Cruz	0.8	0.6	1.0	7	5	9	0.3	0.2	0.3	2	1	3
Mexico	Salinas	0.3	0.3	0.4	1	1	2	0.1	0.1	0.1	0	0	1
Mexico	Salinas Victoria	0.1	0.1	0.1	0	0	0	0.0	0.0	0.0	0	0	0
Mexico	Saltabarranca	0.4	0.3	0.6	1	0	1	0.1	0.1	0.1	0	0	0
Mexico	Saltillo	0.2	0.1	0.2	12	9	17	0.1	0.0	0.1	5	3	7
Mexico	Salto De Agua	1.2	0.9	1.6	10	7	13	0.3	0.2	0.4	3	2	3
Mexico	Salvador Alvarado	0.2	0.1	0.2	2	1	2	0.1	0.0	0.1	0	0	1
Mexico	Salvador Escalante	0.4	0.3	0.5	2	2	3	0.1	0.1	0.2	1	1	1
Mexico	Salvatierra	0.4	0.3	0.5	4	3	5	0.1	0.0	0.1	1	0	1
Mexico	Samahil	0.3	0.2	0.4	0	0	0	0.1	0.1	0.1	0	0	0
Mexico	San Agustin Amatengo	0.7	0.5	0.9	0	0	0	0.2	0.2	0.3	0	0	0
Mexico	San Agustin Atenango	0.7	0.5	0.9	0	0	0	0.2	0.2	0.3	0	0	0
Mexico	San Agustin Chayuco	0.8	0.6	1.1	1	1	1	0.3	0.2	0.4	0	0	0
Mexico	San Agustin De Las Juntas	0.6	0.5	0.8	1	1	1	0.2	0.2	0.3	0	0	1
Mexico	San Agustin Etla	0.6	0.5	0.8	0	0	0	0.2	0.1	0.3	0	0	0
Mexico	San Agustin Loxicha	0.8	0.6	1.0	2	2	3	0.2	0.2	0.3	1	1	1

Table 3: Diarrhoea Mortality by Unit *(continued)*

Country	Unit	2000 mortality						2017 mortality					
		mean rate (per 10,000)	lower rate (per 10,000)	upper rate (per 10,000)	mean count	lower count	upper count	mean rate (per 10,000)	lower rate (per 10,000)	upper rate (per 10,000)	mean count	lower count	upper count
Mexico	San Agustin Metzquititlan	0.2	0.2	0.3	0	0	0	0.1	0.0	0.1	0	0	0
Mexico	San Agustin Tlacotepec	0.7	0.5	0.9	0	0	0	0.2	0.2	0.3	0	0	0
Mexico	San Agustin Tlaxiaca	0.2	0.2	0.3	1	1	1	0.1	0.0	0.1	0	0	0
Mexico	San Agustin Yatareni	0.6	0.5	0.8	1	1	2	0.2	0.1	0.3	0	0	0
Mexico	San Andres Cabecera Nueva	0.7	0.6	0.9	0	0	1	0.2	0.2	0.3	0	0	0
Mexico	San Andres Cholula	0.5	0.4	0.6	6	5	8	0.1	0.1	0.2	2	2	3
Mexico	San Andres Dinicuiti	0.6	0.5	0.8	0	0	0	0.2	0.1	0.3	0	0	0
Mexico	San Andres Duraznal	1.2	0.9	1.5	1	0	1	0.3	0.2	0.4	0	0	1
Mexico	San Andres Huaxpaltepec	0.8	0.6	1.0	1	1	1	0.3	0.2	0.4	0	0	0
Mexico	San Andres Huayapam	0.6	0.5	0.8	1	1	1	0.2	0.1	0.3	0	0	0
Mexico	San Andres Ixtlahuaca	0.7	0.5	0.9	0	0	0	0.2	0.2	0.3	0	0	0
Mexico	San Andres Lagunas	0.6	0.5	0.8	0	0	0	0.2	0.1	0.3	0	0	0
Mexico	San Andres Nuxino	0.7	0.5	0.9	0	0	0	0.2	0.2	0.3	0	0	0
Mexico	San Andres Paxtlan	0.8	0.6	1.0	1	0	1	0.2	0.2	0.3	0	0	0
Mexico	San Andres Sinaxtla	0.7	0.5	0.9	0	0	0	0.2	0.2	0.3	0	0	0
Mexico	San Andres Solaga	0.8	0.6	1.0	0	0	0	0.2	0.2	0.3	0	0	0
Mexico	San Andres Tenejapan	0.4	0.3	0.5	0	0	0	0.1	0.0	0.1	0	0	0
Mexico	San Andres Teotilalpam	0.8	0.6	1.0	1	0	1	0.2	0.2	0.3	0	0	0
Mexico	San Andres Tepetlapa	0.7	0.5	0.9	0	0	0	0.2	0.2	0.3	0	0	0
Mexico	San Andres Tuxtla	0.4	0.3	0.5	10	7	13	0.1	0.1	0.1	1	1	2
Mexico	San Andres Yaa	0.7	0.6	1.0	0	0	0	0.2	0.2	0.3	0	0	0
Mexico	San Andres Zabache	0.7	0.5	0.9	0	0	0	0.2	0.2	0.3	0	0	0
Mexico	San Andres Zautla	0.6	0.5	0.8	0	0	1	0.2	0.2	0.3	0	0	0
Mexico	San Antonino Castillo Velasco	0.6	0.5	0.8	0	0	0	0.2	0.1	0.3	0	0	0
Mexico	San Antonino El Alto	0.7	0.5	0.9	0	0	0	0.2	0.2	0.3	0	0	0
Mexico	San Antonino Monte Verde	0.6	0.5	0.8	1	0	1	0.2	0.1	0.3	0	0	0
Mexico	San Antonio	0.5	0.4	0.6	1	1	1	0.1	0.1	0.2	0	0	0
Mexico	San Antonio Acutla	0.6	0.5	0.8	0	0	0	0.2	0.1	0.3	0	0	0
Mexico	San Antonio Canada	0.5	0.4	0.7	0	0	0	0.1	0.1	0.2	0	0	0
Mexico	San Antonio De La Cal	0.6	0.5	0.8	1	1	2	0.2	0.1	0.3	0	0	1
Mexico	San Antonio Huitepec	0.7	0.5	0.9	1	1	1	0.2	0.2	0.3	0	0	0

Table 3: Diarrhoea Mortality by Unit *(continued)*

Country	Unit	2000 mortality						2017 mortality					
		mean rate (per 10,000)	lower rate (per 10,000)	upper rate (per 10,000)	mean count	lower count	upper count	mean rate (per 10,000)	lower rate (per 10,000)	upper rate (per 10,000)	mean count	lower count	upper count
Mexico	San Antonio La Isla	0.4	0.3	0.4	0	0	0	0.1	0.1	0.1	0	0	0
Mexico	San Antonio Nanahuatipam	0.7	0.6	1.0	0	0	0	0.2	0.2	0.3	0	0	0
Mexico	San Antonio Sinicahua	0.7	0.5	0.8	0	0	0	0.2	0.2	0.3	0	0	0
Mexico	San Antonio Tepetlapa	0.8	0.6	1.0	0	0	0	0.3	0.2	0.4	0	0	0
Mexico	San Baltazar Chichicapam	0.7	0.5	0.9	0	0	0	0.2	0.2	0.3	0	0	0
Mexico	San Baltazar Loxicha	0.8	0.6	1.0	0	0	1	0.2	0.2	0.3	0	0	0
Mexico	San Baltazar Yatzachi El Bajo	0.8	0.6	1.0	0	0	0	0.2	0.2	0.3	0	0	0
Mexico	San Bartolo Coyotepec	0.7	0.5	0.9	1	1	2	0.2	0.2	0.3	0	0	1
Mexico	San Bartolo Soyaltepec	0.6	0.5	0.8	0	0	0	0.2	0.1	0.3	0	0	0
Mexico	San Bartolo Tutotepec	0.3	0.2	0.3	1	0	1	0.1	0.0	0.1	0	0	0
Mexico	San Bartolo Yautepec	0.8	0.6	1.0	0	0	0	0.2	0.2	0.3	0	0	0
Mexico	San Bartolome Ayautla	0.8	0.6	1.0	0	0	1	0.2	0.2	0.3	0	0	0
Mexico	San Bartolome Loxicha	0.8	0.6	1.0	0	0	0	0.2	0.2	0.3	0	0	0
Mexico	San Bartolome Quialana	0.7	0.5	0.9	0	0	0	0.2	0.2	0.3	0	0	0
Mexico	San Bartolome Yucuane	0.7	0.5	0.9	0	0	0	0.2	0.2	0.3	0	0	0
Mexico	San Bartolome Zoogocho	0.7	0.6	1.0	0	0	0	0.2	0.2	0.3	0	0	0
Mexico	San Bernardo	0.3	0.2	0.3	0	0	0	0.1	0.0	0.1	0	0	0
Mexico	San Bernardo Mixtepec	0.7	0.5	0.9	0	0	0	0.2	0.2	0.3	0	0	0
Mexico	San Blas	0.3	0.2	0.4	1	1	2	0.1	0.1	0.1	0	0	0
Mexico	San Blas Atempa	0.7	0.6	1.0	1	1	2	0.2	0.2	0.3	0	0	1
Mexico	San Buenaventura	0.2	0.1	0.3	0	0	0	0.1	0.0	0.1	0	0	0
Mexico	San Carlos	0.1	0.1	0.2	0	0	0	0.1	0.0	0.1	0	0	0
Mexico	San Carlos Yautepec	0.8	0.6	1.0	1	1	1	0.2	0.2	0.3	0	0	0
Mexico	San Ciro De Acosta	0.4	0.3	0.5	0	0	1	0.1	0.1	0.2	0	0	0
Mexico	San Cristobal Amatlan	0.7	0.6	0.9	0	0	1	0.2	0.2	0.3	0	0	0
Mexico	San Cristobal Amoltepec	0.7	0.5	0.9	0	0	0	0.2	0.2	0.3	0	0	0
Mexico	San Cristobal De La Barranca	0.3	0.2	0.4	0	0	0	0.1	0.0	0.1	0	0	0
Mexico	San Cristobal De Las Casas	1.0	0.7	1.3	21	16	28	0.3	0.2	0.4	6	5	8
Mexico	San Cristobal Lachirioag	0.8	0.6	1.1	0	0	0	0.2	0.2	0.3	0	0	0
Mexico	San Cristobal Suchixtlahuaca	0.6	0.5	0.8	0	0	0	0.2	0.1	0.3	0	0	0
Mexico	San Damian Texoloc	0.3	0.2	0.4	0	0	0	0.1	0.1	0.1	0	0	0
Mexico	San Diego De Alejandria	0.2	0.2	0.3	0	0	0	0.0	0.0	0.1	0	0	0

Table 3: Diarrhoea Mortality by Unit *(continued)*

Country	Unit	2000 mortality mean rate (per 10,000)	lower rate (per 10,000)	upper rate (per 10,000)	mean count	lower count	upper count	2017 mortality mean rate (per 10,000)	lower rate (per 10,000)	upper rate (per 10,000)	mean count	lower count	upper count
Mexico	San Diego De La Union	0.4	0.3	0.5	2	1	2	0.1	0.0	0.1	0	0	0
Mexico	San Diego La Mesa Tochimiltzingo	0.6	0.4	0.7	0	0	0	0.2	0.1	0.2	0	0	0
Mexico	San Dimas	0.3	0.2	0.3	1	1	1	0.1	0.0	0.1	0	0	0
Mexico	San Dionisio Del Mar	0.8	0.6	1.1	1	0	1	0.3	0.2	0.3	0	0	0
Mexico	San Dionisio Ocotepec	0.7	0.5	0.9	1	1	1	0.2	0.2	0.3	0	0	0
Mexico	San Dionisio Ocotlan	0.7	0.5	0.9	0	0	0	0.2	0.2	0.3	0	0	0
Mexico	San Esteban Atatlahuca	0.6	0.5	0.8	0	0	0	0.2	0.1	0.3	0	0	0
Mexico	San Felipe	0.3	0.3	0.4	5	4	6	0.1	0.0	0.1	1	1	1
Mexico	San Felipe	0.4	0.3	0.5	0	0	0	0.1	0.1	0.2	0	0	0
Mexico	San Felipe De Jesus	0.3	0.2	0.4	0	0	0	0.1	0.0	0.1	0	0	0
Mexico	San Felipe Del Progreso	0.3	0.2	0.4	4	3	6	0.1	0.1	0.1	2	1	2
Mexico	San Felipe Jalapa De Diaz	0.8	0.6	1.0	3	3	4	0.2	0.2	0.3	1	1	1
Mexico	San Felipe Orizatlan	0.3	0.2	0.3	2	1	2	0.1	0.0	0.1	0	0	0
Mexico	San Felipe Tejalapam	0.7	0.5	0.9	1	0	1	0.2	0.2	0.3	0	0	0
Mexico	San Felipe Teotlalcingo	0.5	0.4	0.6	1	1	1	0.2	0.1	0.2	0	0	0
Mexico	San Felipe Tepatlan	0.6	0.4	0.7	0	0	0	0.2	0.1	0.2	0	0	0
Mexico	San Felipe Usila	0.8	0.6	1.1	2	1	2	0.3	0.2	0.3	0	0	0
Mexico	San Fernando	0.1	0.1	0.2	1	1	1	0.0	0.0	0.1	0	0	0
Mexico	San Fernando	1.0	0.8	1.4	5	3	6	0.3	0.2	0.4	2	1	2
Mexico	San Francisco Cahuacua	0.7	0.6	1.0	0	0	0	0.2	0.2	0.3	0	0	0
Mexico	San Francisco Cajonos	0.8	0.6	1.0	0	0	0	0.2	0.2	0.3	0	0	0
Mexico	San Francisco Chapulapa	0.7	0.6	1.0	0	0	0	0.2	0.2	0.3	0	0	0
Mexico	San Francisco Chindua	0.7	0.5	0.9	0	0	0	0.2	0.2	0.3	0	0	0
Mexico	San Francisco De Borja	0.3	0.2	0.4	0	0	0	0.1	0.1	0.1	0	0	0
Mexico	San Francisco De Conchos	0.3	0.2	0.4	0	0	0	0.1	0.1	0.1	0	0	0
Mexico	San Francisco De Los Romo	0.2	0.2	0.3	1	1	1	0.1	0.1	0.1	0	0	1
Mexico	San Francisco Del Mar	0.8	0.6	1.1	1	1	1	0.3	0.2	0.4	0	0	0
Mexico	San Francisco Del Oro	0.3	0.2	0.4	0	0	0	0.1	0.1	0.1	0	0	0
Mexico	San Francisco Del Rincon	0.3	0.2	0.4	5	4	7	0.1	0.0	0.1	1	1	1
Mexico	San Francisco Huehuetlan	0.7	0.5	0.9	0	0	0	0.2	0.2	0.3	0	0	0
Mexico	San Francisco Ixhuatan	0.8	0.6	1.1	1	1	2	0.3	0.2	0.4	0	0	0
Mexico	San Francisco Jaltepetongo	0.7	0.5	0.9	0	0	0	0.2	0.2	0.3	0	0	0
Mexico	San Francisco Lachigolo	0.7	0.5	0.9	0	0	0	0.2	0.2	0.3	0	0	0

Table 3: Diarrhoea Mortality by Unit *(continued)*

Country	Unit	2000 mortality mean rate (per 10,000)	lower rate (per 10,000)	upper rate (per 10,000)	mean count	lower count	upper count	2017 mortality mean rate (per 10,000)	lower rate (per 10,000)	upper rate (per 10,000)	mean count	lower count	upper count
Mexico	San Francisco Logueche	0.7	0.6	1.0	0	0	0	0.2	0.2	0.3	0	0	0
Mexico	San Francisco Nuxano	0.6	0.5	0.8	0	0	0	0.2	0.1	0.3	0	0	0
Mexico	San Francisco Ozolotepec	0.7	0.6	0.9	0	0	0	0.2	0.2	0.3	0	0	0
Mexico	San Francisco Sola	0.7	0.6	0.9	0	0	0	0.2	0.2	0.3	0	0	0
Mexico	San Francisco Telixtlahuaca	0.7	0.5	0.9	0	0	1	0.2	0.2	0.3	0	0	0
Mexico	San Francisco Teopan	0.6	0.5	0.8	0	0	0	0.2	0.1	0.3	0	0	0
Mexico	San Francisco Tetlanohcan	0.3	0.2	0.4	0	0	0	0.1	0.1	0.1	0	0	0
Mexico	San Francisco Tlapancingo	0.7	0.5	0.9	0	0	0	0.2	0.2	0.3	0	0	0
Mexico	San Gabriel	0.3	0.2	0.4	0	0	0	0.1	0.0	0.1	0	0	0
Mexico	San Gabriel Chilac	0.6	0.4	0.7	1	1	1	0.2	0.1	0.2	0	0	0
Mexico	San Gabriel Mixtepec	0.8	0.6	1.0	1	0	1	0.3	0.2	0.4	0	0	0
Mexico	San Gregorio Atzompa	0.5	0.4	0.6	0	0	0	0.2	0.1	0.2	0	0	0
Mexico	San Ignacio	0.2	0.1	0.2	1	0	1	0.0	0.0	0.1	0	0	0
Mexico	San Ignacio Rio Muerto	0.4	0.3	0.5	1	0	1	0.1	0.1	0.1	0	0	0
Mexico	San Ildefonso Amatlan	0.7	0.6	0.9	0	0	0	0.2	0.2	0.3	0	0	0
Mexico	San Ildefonso Sola	0.7	0.6	0.9	0	0	0	0.2	0.2	0.3	0	0	0
Mexico	San Ildefonso Villa Alta	0.8	0.6	1.0	0	0	0	0.2	0.2	0.3	0	0	0
Mexico	San Jacinto Amilpas	0.6	0.5	0.8	1	1	1	0.2	0.1	0.3	0	0	0
Mexico	San Jacinto Tlacotepec	0.8	0.6	1.0	0	0	0	0.2	0.2	0.3	0	0	0
Mexico	San Javier	0.3	0.2	0.4	0	0	0	0.1	0.1	0.1	0	0	0
Mexico	San Jeronimo Coatlan	0.8	0.6	1.0	1	0	1	0.2	0.2	0.3	0	0	0
Mexico	San Jeronimo Silacayoapilla	0.6	0.5	0.8	0	0	0	0.2	0.1	0.3	0	0	0
Mexico	San Jeronimo Sosola	0.7	0.5	0.9	0	0	0	0.2	0.2	0.3	0	0	0
Mexico	San Jeronimo Taviche	0.7	0.5	0.9	0	0	0	0.2	0.2	0.3	0	0	0
Mexico	San Jeronimo Tecoatl	0.7	0.5	1.0	0	0	0	0.2	0.2	0.3	0	0	0
Mexico	San Jeronimo Tecuanipan	0.5	0.4	0.6	1	1	1	0.2	0.1	0.2	0	0	0
Mexico	San Jeronimo Tlacochahuaya	0.7	0.5	0.9	0	0	0	0.2	0.2	0.3	0	0	0
Mexico	San Jeronimo Xayacatlan	0.6	0.4	0.8	0	0	0	0.2	0.1	0.2	0	0	0
Mexico	San Jeronimo Zacualpan	0.3	0.2	0.4	0	0	0	0.1	0.1	0.1	0	0	0
Mexico	San Joaquin	0.3	0.2	0.4	0	0	0	0.1	0.1	0.1	0	0	0
Mexico	San Jorge Nuchita	0.7	0.5	0.9	0	0	0	0.2	0.2	0.3	0	0	0
Mexico	San Jose Ayuquila	0.7	0.5	0.8	0	0	0	0.2	0.2	0.3	0	0	0

Table 3: Diarrhoea Mortality by Unit *(continued)*

Country	Unit	2000 mortality mean rate (per 10,000)	lower rate (per 10,000)	upper rate (per 10,000)	mean count	lower count	upper count	2017 mortality mean rate (per 10,000)	lower rate (per 10,000)	upper rate (per 10,000)	mean count	lower count	upper count
Mexico	San Jose Chiapa	0.5	0.4	0.7	1	1	2	0.1	0.1	0.2	0	0	0
Mexico	San Jose Chiltepec	0.8	0.6	1.1	1	1	2	0.3	0.2	0.3	0	0	0
Mexico	San Jose De Gracia	0.2	0.2	0.3	0	0	0	0.1	0.1	0.1	0	0	0
Mexico	San Jose Del Penasco	0.7	0.6	0.9	0	0	0	0.2	0.2	0.3	0	0	0
Mexico	San Jose Del Progreso	0.7	0.5	0.9	1	1	1	0.2	0.2	0.3	0	0	0
Mexico	San Jose Del Rincon	0.3	0.2	0.4	4	3	5	0.1	0.0	0.1	1	1	2
Mexico	San Jose Estancia Grande	0.8	0.6	1.0	0	0	0	0.3	0.2	0.4	0	0	0
Mexico	San Jose Independencia	0.8	0.6	1.1	1	0	1	0.3	0.2	0.3	0	0	0
Mexico	San Jose Iturbide	0.3	0.3	0.4	3	2	4	0.1	0.0	0.1	1	0	1
Mexico	San Jose Lachiguiri	0.7	0.6	1.0	0	0	0	0.2	0.2	0.3	0	0	0
Mexico	San Jose Miahuatlan	0.6	0.4	0.8	1	1	2	0.2	0.1	0.2	0	0	0
Mexico	San Jose Teacalco	0.3	0.2	0.4	0	0	0	0.1	0.1	0.1	0	0	0
Mexico	San Jose Tenango	0.8	0.6	1.0	2	2	3	0.2	0.2	0.3	0	0	1
Mexico	San Juan Achiutla	0.7	0.5	0.9	0	0	0	0.2	0.2	0.3	0	0	0
Mexico	San Juan Atenco	0.5	0.4	0.7	0	0	0	0.1	0.1	0.2	0	0	0
Mexico	San Juan Atepec	0.7	0.5	0.9	0	0	0	0.2	0.2	0.3	0	0	0
Mexico	San Juan Atzompa	0.5	0.4	0.7	0	0	0	0.1	0.1	0.2	0	0	0
Mexico	San Juan Bautista Atatlahuca	0.7	0.6	1.0	0	0	0	0.2	0.2	0.3	0	0	0
Mexico	San Juan Bautista Coixtlahuaca	0.6	0.5	0.8	0	0	0	0.2	0.1	0.3	0	0	0
Mexico	San Juan Bautista Cuicatlan	0.7	0.6	1.0	1	1	1	0.2	0.2	0.3	0	0	0
Mexico	San Juan Bautista Guelache	0.7	0.5	0.9	0	0	0	0.2	0.2	0.3	0	0	0
Mexico	San Juan Bautista Jayacatlan	0.7	0.5	0.9	0	0	0	0.2	0.2	0.3	0	0	0
Mexico	San Juan Bautista Lo De Soto	0.7	0.5	0.9	0	0	0	0.2	0.2	0.3	0	0	0
Mexico	San Juan Bautista Suchitepec	0.6	0.5	0.8	0	0	0	0.2	0.1	0.3	0	0	0
Mexico	San Juan Bautista Tlachichilco	0.7	0.5	0.9	0	0	0	0.2	0.2	0.3	0	0	0
Mexico	San Juan Bautista Tlacoatzintepec	0.8	0.6	1.0	0	0	0	0.2	0.2	0.3	0	0	0
Mexico	San Juan Bautista Tuxtepec	0.8	0.6	1.0	14	11	19	0.3	0.2	0.3	4	3	5
Mexico	San Juan Bautista Valle Nacional	0.8	0.6	1.0	2	2	3	0.2	0.2	0.3	1	0	1

Table 3: Diarrhoea Mortality by Unit *(continued)*

Country	Unit	2000 mortality						2017 mortality					
		mean rate (per 10,000)	lower rate (per 10,000)	upper rate (per 10,000)	mean count	lower count	upper count	mean rate (per 10,000)	lower rate (per 10,000)	upper rate (per 10,000)	mean count	lower count	upper count
Mexico	San Juan Cacahuatepec	0.8	0.6	1.0	1	1	2	0.3	0.2	0.4	0	0	0
Mexico	San Juan Cancuc	1.2	0.8	1.5	5	3	6	0.3	0.2	0.4	2	1	2
Mexico	San Juan Chicomezuchil	0.7	0.5	0.9	0	0	0	0.2	0.2	0.3	0	0	0
Mexico	San Juan Chilateca	0.6	0.5	0.8	0	0	0	0.2	0.1	0.3	0	0	0
Mexico	San Juan Cieneguilla	0.7	0.5	0.9	0	0	0	0.2	0.2	0.3	0	0	0
Mexico	San Juan Coatzospam	0.7	0.6	1.0	0	0	0	0.2	0.2	0.3	0	0	0
Mexico	San Juan Colorado	0.8	0.6	1.1	1	1	1	0.3	0.2	0.4	0	0	1
Mexico	San Juan Comaltepec	0.8	0.6	1.0	0	0	0	0.2	0.2	0.3	0	0	0
Mexico	San Juan Cotzocon	0.8	0.6	1.1	3	2	4	0.3	0.2	0.3	1	0	1
Mexico	San Juan De Guadalupe	0.2	0.2	0.3	0	0	0	0.1	0.0	0.1	0	0	0
Mexico	San Juan De Los Cues	0.7	0.5	0.9	0	0	0	0.2	0.2	0.3	0	0	0
Mexico	San Juan De Los Lagos	0.2	0.2	0.3	2	1	2	0.1	0.0	0.1	0	0	1
Mexico	San Juan De Sabinas	0.2	0.2	0.3	1	1	1	0.1	0.0	0.1	0	0	0
Mexico	San Juan Del Estado	0.7	0.5	0.9	0	0	0	0.2	0.2	0.3	0	0	0
Mexico	San Juan Del Rio	0.2	0.2	0.3	6	4	7	0.1	0.1	0.1	2	2	3
Mexico	San Juan Del Rio	0.7	0.5	1.0	0	0	0	0.2	0.2	0.3	0	0	0
Mexico	San Juan Del Rio	0.2	0.2	0.3	0	0	0	0.1	0.0	0.1	0	0	0
Mexico	San Juan Diuxi	0.6	0.5	0.8	0	0	0	0.2	0.1	0.3	0	0	0
Mexico	San Juan Evangelista	0.4	0.3	0.5	2	1	2	0.1	0.1	0.1	0	0	0
Mexico	San Juan Evangelista Analco	0.7	0.5	0.9	0	0	0	0.2	0.2	0.3	0	0	0
Mexico	San Juan Guelavia	0.7	0.5	0.9	0	0	0	0.2	0.2	0.3	0	0	0
Mexico	San Juan Guichicovi	0.8	0.6	1.0	3	2	4	0.3	0.2	0.3	1	0	1
Mexico	San Juan Huactzinco	0.3	0.2	0.4	0	0	0	0.1	0.1	0.1	0	0	0
Mexico	San Juan Ihualtepec	0.6	0.5	0.8	0	0	0	0.2	0.2	0.3	0	0	0
Mexico	San Juan Juquila Mixes	0.7	0.6	1.0	0	0	1	0.2	0.2	0.3	0	0	0
Mexico	San Juan Juquila Vijanos	0.8	0.6	1.0	0	0	0	0.2	0.2	0.3	0	0	0
Mexico	San Juan Lachao	0.8	0.6	1.0	1	0	1	0.2	0.2	0.3	0	0	0
Mexico	San Juan Lachigalla	0.7	0.5	0.9	0	0	0	0.2	0.2	0.3	0	0	0
Mexico	San Juan Lajarcia	0.8	0.6	1.0	0	0	0	0.2	0.2	0.3	0	0	0
Mexico	San Juan Lalana	0.8	0.6	1.0	3	2	3	0.2	0.2	0.3	0	0	1
Mexico	San Juan Mazatlan	0.8	0.6	1.1	2	2	3	0.3	0.2	0.3	1	0	1
Mexico	San Juan Mixtepec - Distr. 08 -	0.7	0.5	0.8	1	1	1	0.2	0.2	0.3	0	0	0

Table 3: Diarrhoea Mortality by Unit *(continued)*

Country	Unit	2000 mortality mean rate (per 10,000)	lower rate (per 10,000)	upper rate (per 10,000)	mean count	lower count	upper count	2017 mortality mean rate (per 10,000)	lower rate (per 10,000)	upper rate (per 10,000)	mean count	lower count	upper count
Mexico	San Juan Mixtepec - Distr. 26 -	0.8	0.6	1.0	0	0	0	0.2	0.2	0.3	0	0	0
Mexico	San Juan Numi	0.6	0.5	0.8	0	0	1	0.2	0.1	0.3	0	0	0
Mexico	San Juan Ozolotepec	0.7	0.5	0.9	0	0	1	0.2	0.2	0.3	0	0	0
Mexico	San Juan Petlapa	0.8	0.6	1.0	0	0	1	0.2	0.2	0.3	0	0	0
Mexico	San Juan Quiahije	0.8	0.6	1.0	1	1	1	0.2	0.2	0.3	0	0	0
Mexico	San Juan Quiotepec	0.7	0.5	0.9	0	0	0	0.2	0.2	0.3	0	0	0
Mexico	San Juan Sayultepec	0.7	0.5	0.9	0	0	0	0.2	0.2	0.3	0	0	0
Mexico	San Juan Tabaa	0.8	0.6	1.0	0	0	0	0.2	0.2	0.3	0	0	0
Mexico	San Juan Tamazola	0.7	0.5	0.9	0	0	0	0.2	0.2	0.3	0	0	0
Mexico	San Juan Teita	0.7	0.5	0.9	0	0	0	0.2	0.2	0.3	0	0	0
Mexico	San Juan Teitipac	0.7	0.5	0.9	0	0	0	0.2	0.2	0.3	0	0	0
Mexico	San Juan Tepeuxila	0.7	0.5	0.9	0	0	0	0.2	0.2	0.3	0	0	0
Mexico	San Juan Teposcolula	0.6	0.5	0.8	0	0	0	0.2	0.1	0.3	0	0	0
Mexico	San Juan Yaee	0.8	0.6	1.0	0	0	0	0.2	0.2	0.3	0	0	0
Mexico	San Juan Yatzona	0.8	0.6	1.1	0	0	0	0.2	0.2	0.3	0	0	0
Mexico	San Juan Yucuita	0.7	0.5	0.9	0	0	0	0.2	0.2	0.3	0	0	0
Mexico	San Juanito De Escobedo	0.3	0.2	0.3	0	0	0	0.1	0.0	0.1	0	0	0
Mexico	San Julian	0.2	0.2	0.3	0	0	0	0.0	0.0	0.1	0	0	0
Mexico	San Lorenzo	0.8	0.6	1.1	1	0	1	0.3	0.2	0.4	0	0	0
Mexico	San Lorenzo Albarradas	0.7	0.5	0.9	0	0	0	0.2	0.2	0.3	0	0	0
Mexico	San Lorenzo Axocomanitla	0.3	0.2	0.4	0	0	0	0.1	0.1	0.1	0	0	0
Mexico	San Lorenzo Cacaotepec	0.6	0.5	0.8	1	1	1	0.2	0.2	0.3	0	0	1
Mexico	San Lorenzo Cuaunecuiltitla	0.7	0.5	1.0	0	0	0	0.2	0.2	0.3	0	0	0
Mexico	San Lorenzo Texmelucan	0.8	0.6	1.0	1	1	1	0.2	0.2	0.3	0	0	0
Mexico	San Lorenzo Victoria	0.7	0.5	0.9	0	0	0	0.2	0.2	0.3	0	0	0
Mexico	San Lucas	0.5	0.4	0.6	1	1	1	0.1	0.1	0.2	0	0	0
Mexico	San Lucas	1.2	0.9	1.6	1	1	1	0.3	0.2	0.4	0	0	0
Mexico	San Lucas Camotlan	0.8	0.6	1.0	0	0	0	0.2	0.2	0.3	0	0	0
Mexico	San Lucas Ojitlan	0.8	0.6	1.0	3	2	4	0.2	0.2	0.3	1	0	1
Mexico	San Lucas Quiavini	0.7	0.5	0.9	0	0	0	0.2	0.2	0.3	0	0	0
Mexico	San Lucas Tecopilco	0.3	0.2	0.4	0	0	0	0.1	0.1	0.1	0	0	0
Mexico	San Lucas Zoquiapam	0.7	0.5	1.0	1	1	1	0.2	0.2	0.3	0	0	0
Mexico	San Luis Acatlan	1.2	0.9	1.4	10	8	12	0.2	0.1	0.3	1	1	2
Mexico	San Luis Amatlan	0.7	0.6	0.9	0	0	0	0.2	0.2	0.3	0	0	0
Mexico	San Luis De La Paz	0.4	0.3	0.5	6	4	7	0.1	0.0	0.1	1	1	1
Mexico	San Luis Del Cordero	0.3	0.2	0.3	0	0	0	0.1	0.0	0.1	0	0	0
Mexico	San Luis Potosi	0.4	0.3	0.5	36	27	47	0.1	0.1	0.2	8	5	11
Mexico	San Luis Rio Colorado	0.3	0.2	0.4	6	4	9	0.1	0.1	0.1	2	1	2

Table 3: Diarrhoea Mortality by Unit *(continued)*

Country	Unit	2000 mortality mean rate (per 10,000)	2000 mortality lower rate (per 10,000)	2000 mortality upper rate (per 10,000)	2000 mortality mean count	2000 mortality lower count	2000 mortality upper count	2017 mortality mean rate (per 10,000)	2017 mortality lower rate (per 10,000)	2017 mortality upper rate (per 10,000)	2017 mortality mean count	2017 mortality lower count	2017 mortality upper count
Mexico	San Marcial Ozolotepec	0.8	0.6	1.0	0	0	0	0.2	0.2	0.3	0	0	0
Mexico	San Marcos	1.2	0.9	1.5	8	6	10	0.2	0.1	0.3	1	1	2
Mexico	San Marcos	0.3	0.2	0.3	0	0	0	0.1	0.0	0.1	0	0	0
Mexico	San Marcos Arteaga	0.6	0.5	0.8	0	0	0	0.2	0.1	0.3	0	0	0
Mexico	San Martin Chalchicuautla	0.5	0.4	0.6	1	1	2	0.1	0.1	0.2	0	0	0
Mexico	San Martin De Bolanos	0.3	0.2	0.4	0	0	0	0.1	0.0	0.1	0	0	0
Mexico	San Martin De Las Piramides	0.3	0.2	0.4	1	0	1	0.1	0.1	0.1	0	0	0
Mexico	San Martin De Los Cansecos	0.7	0.5	0.9	0	0	0	0.2	0.2	0.3	0	0	0
Mexico	San Martin Hidalgo	0.3	0.2	0.3	1	0	1	0.1	0.0	0.1	0	0	0
Mexico	San Martin Huamelulpam	0.6	0.5	0.8	0	0	0	0.2	0.1	0.3	0	0	0
Mexico	San Martin Itunyoso	0.6	0.5	0.8	0	0	0	0.2	0.2	0.3	0	0	0
Mexico	San Martin Lachila	0.7	0.5	0.9	0	0	0	0.2	0.2	0.3	0	0	0
Mexico	San Martin Peras	0.7	0.5	0.9	2	2	3	0.2	0.2	0.3	0	0	0
Mexico	San Martin Texmelucan	0.5	0.4	0.6	8	6	9	0.2	0.1	0.2	2	2	3
Mexico	San Martin Tilcajete	0.7	0.5	0.9	0	0	0	0.2	0.2	0.3	0	0	0
Mexico	San Martin Totoltepec	0.6	0.4	0.8	0	0	0	0.2	0.1	0.2	0	0	0
Mexico	San Martin Toxpalan	0.7	0.5	0.9	1	0	1	0.2	0.2	0.3	0	0	0
Mexico	San Martin Zacatepec	0.7	0.5	0.9	0	0	0	0.2	0.1	0.3	0	0	0
Mexico	San Mateo Atenco	0.3	0.3	0.4	2	2	3	0.1	0.1	0.1	1	0	1
Mexico	San Mateo Cajonos	0.8	0.6	1.0	0	0	0	0.2	0.2	0.3	0	0	0
Mexico	San Mateo Del Mar	0.8	0.6	1.1	1	0	1	0.3	0.2	0.4	0	0	0
Mexico	San Mateo Etlatongo	0.7	0.5	0.9	0	0	0	0.2	0.2	0.3	0	0	0
Mexico	San Mateo Nejapam	0.7	0.5	0.9	0	0	0	0.2	0.2	0.3	0	0	0
Mexico	San Mateo Penasco	0.7	0.5	0.9	0	0	0	0.2	0.2	0.3	0	0	0
Mexico	San Mateo Pinas	0.8	0.6	1.0	0	0	1	0.2	0.2	0.3	0	0	0
Mexico	San Mateo Rio Hondo	0.8	0.6	1.0	1	0	1	0.2	0.2	0.3	0	0	0
Mexico	San Mateo Sindihui	0.7	0.6	0.9	0	0	0	0.2	0.2	0.3	0	0	0
Mexico	San Mateo Tlapiltepec	0.6	0.5	0.8	0	0	0	0.2	0.1	0.3	0	0	0
Mexico	San Mateo Yoloxochitlan	0.7	0.5	1.0	0	0	0	0.2	0.2	0.3	0	0	0
Mexico	San Matias Tlalancaleca	0.5	0.4	0.7	1	1	1	0.2	0.1	0.2	0	0	1
Mexico	San Melchor Betaza	0.8	0.6	1.0	0	0	0	0.2	0.2	0.3	0	0	0
Mexico	San Miguel Achiutla	0.7	0.5	0.9	0	0	0	0.2	0.2	0.3	0	0	0

Table 3: Diarrhoea Mortality by Unit *(continued)*

Country	Unit	2000 mortality mean rate (per 10,000)	lower rate (per 10,000)	upper rate (per 10,000)	mean count	lower count	upper count	2017 mortality mean rate (per 10,000)	lower rate (per 10,000)	upper rate (per 10,000)	mean count	lower count	upper count
Mexico	San Miguel Ahuehuetitlan	0.7	0.5	0.9	0	0	0	0.2	0.2	0.3	0	0	0
Mexico	San Miguel Aloapam	0.7	0.5	0.9	0	0	0	0.2	0.2	0.3	0	0	0
Mexico	San Miguel Amatitlan	0.7	0.5	0.9	1	0	1	0.2	0.1	0.3	0	0	0
Mexico	San Miguel Amatlan	0.7	0.5	0.9	0	0	0	0.2	0.2	0.3	0	0	0
Mexico	San Miguel Chicahua	0.6	0.5	0.8	0	0	0	0.2	0.1	0.3	0	0	0
Mexico	San Miguel Chimalapa	0.8	0.6	1.0	1	1	2	0.2	0.2	0.3	0	0	0
Mexico	San Miguel Coatlan	0.8	0.6	1.0	0	0	1	0.2	0.2	0.3	0	0	0
Mexico	San Miguel De Allende	0.4	0.3	0.5	8	6	10	0.1	0.0	0.1	1	1	2
Mexico	San Miguel De Horcasitas	0.3	0.2	0.4	0	0	0	0.1	0.0	0.1	0	0	0
Mexico	San Miguel Del Puerto	0.8	0.6	1.0	1	1	1	0.2	0.2	0.3	0	0	0
Mexico	San Miguel Del Rio	0.7	0.5	0.9	0	0	0	0.2	0.2	0.3	0	0	0
Mexico	San Miguel Ejutla	0.6	0.5	0.8	0	0	0	0.2	0.1	0.3	0	0	0
Mexico	San Miguel El Alto	0.2	0.2	0.3	1	1	1	0.0	0.0	0.1	0	0	0
Mexico	San Miguel El Grande	0.6	0.5	0.8	0	0	0	0.2	0.1	0.3	0	0	0
Mexico	San Miguel Huautla	0.6	0.5	0.8	0	0	0	0.2	0.1	0.3	0	0	0
Mexico	San Miguel Ixitlan	0.6	0.4	0.7	0	0	0	0.1	0.1	0.2	0	0	0
Mexico	San Miguel Mixtepec	0.7	0.5	0.9	0	0	0	0.2	0.2	0.3	0	0	0
Mexico	San Miguel Panixtlahuaca	0.8	0.6	1.0	1	1	1	0.3	0.2	0.4	0	0	0
Mexico	San Miguel Peras	0.7	0.5	0.8	0	0	1	0.2	0.2	0.3	0	0	0
Mexico	San Miguel Piedras	0.7	0.5	0.9	0	0	0	0.2	0.2	0.3	0	0	0
Mexico	San Miguel Quetzaltepec	0.8	0.6	1.0	1	0	1	0.2	0.2	0.3	0	0	0
Mexico	San Miguel Santa Flor	0.7	0.5	0.9	0	0	0	0.2	0.2	0.3	0	0	0
Mexico	San Miguel Soyaltepec	0.8	0.6	1.0	4	3	5	0.3	0.2	0.3	1	1	1
Mexico	San Miguel Suchixtepec	0.7	0.5	0.9	0	0	0	0.2	0.2	0.3	0	0	0
Mexico	San Miguel Tecomatlan	0.7	0.5	0.9	0	0	0	0.2	0.1	0.3	0	0	0
Mexico	San Miguel Tenango	0.8	0.6	1.0	0	0	0	0.2	0.2	0.3	0	0	0
Mexico	San Miguel Tequixtepec	0.6	0.5	0.8	0	0	0	0.2	0.1	0.3	0	0	0
Mexico	San Miguel Tilquiapam	0.7	0.5	0.9	0	0	1	0.2	0.2	0.3	0	0	0
Mexico	San Miguel Tlacamama	0.8	0.6	1.0	0	0	1	0.3	0.2	0.4	0	0	0
Mexico	San Miguel Tlacotepec	0.6	0.5	0.8	0	0	0	0.2	0.1	0.3	0	0	0
Mexico	San Miguel Totolapan	1.1	0.9	1.4	5	4	7	0.2	0.2	0.3	1	1	1

Table 3: Diarrhoea Mortality by Unit *(continued)*

Country	Unit	2000 mortality mean rate (per 10,000)	lower rate (per 10,000)	upper rate (per 10,000)	mean count	lower count	upper count	2017 mortality mean rate (per 10,000)	lower rate (per 10,000)	upper rate (per 10,000)	mean count	lower count	upper count
Mexico	San Miguel Tulancingo	0.7	0.5	0.9	0	0	0	0.2	0.1	0.3	0	0	0
Mexico	San Miguel Xoxtla	0.4	0.3	0.6	0	0	0	0.1	0.1	0.2	0	0	0
Mexico	San Miguel Yotao	0.8	0.6	1.0	0	0	0	0.2	0.2	0.3	0	0	0
Mexico	San Nicolas	0.7	0.5	0.9	0	0	0	0.2	0.2	0.3	0	0	0
Mexico	San Nicolas	0.1	0.1	0.2	0	0	0	0.0	0.0	0.1	0	0	0
Mexico	San Nicolas Buenos Aires	0.5	0.4	0.7	0	0	1	0.1	0.1	0.2	0	0	0
Mexico	San Nicolas De Los Garza	0.1	0.1	0.1	5	4	6	0.0	0.0	0.0	1	1	1
Mexico	San Nicolas De Los Ranchos	0.5	0.4	0.7	1	0	1	0.1	0.1	0.2	0	0	0
Mexico	San Nicolas Hidalgo	0.7	0.5	0.9	0	0	0	0.2	0.2	0.3	0	0	0
Mexico	San Nicolas Tolentino	0.4	0.3	0.5	0	0	0	0.1	0.1	0.2	0	0	0
Mexico	San Pablo Anicano	0.6	0.4	0.8	0	0	0	0.2	0.1	0.2	0	0	0
Mexico	San Pablo Coatlan	0.8	0.6	1.0	0	0	1	0.2	0.2	0.3	0	0	0
Mexico	San Pablo Cuatro Venados	0.7	0.5	0.9	0	0	0	0.2	0.2	0.3	0	0	0
Mexico	San Pablo Del Monte	0.3	0.2	0.4	2	1	2	0.1	0.1	0.1	1	0	1
Mexico	San Pablo Etla	0.6	0.5	0.8	1	0	1	0.2	0.2	0.3	0	0	1
Mexico	San Pablo Huitzo	0.7	0.5	0.9	1	1	1	0.2	0.2	0.3	0	0	0
Mexico	San Pablo Huixtepec	0.7	0.5	0.9	1	0	1	0.2	0.2	0.3	0	0	0
Mexico	San Pablo Macuiltianguis	0.7	0.5	0.9	0	0	0	0.2	0.2	0.3	0	0	0
Mexico	San Pablo Tijaltepec	0.7	0.5	0.9	0	0	0	0.2	0.2	0.3	0	0	0
Mexico	San Pablo Villa De Mitla	0.7	0.5	0.9	1	1	1	0.2	0.2	0.3	0	0	0
Mexico	San Pablo Yaganiza	0.7	0.6	1.0	0	0	0	0.2	0.2	0.3	0	0	0
Mexico	San Pedro	0.2	0.1	0.2	2	1	3	0.1	0.0	0.1	1	1	1
Mexico	San Pedro Amuzgos	0.9	0.6	1.1	1	1	1	0.3	0.2	0.4	0	0	0
Mexico	San Pedro Apostol	0.7	0.5	0.9	0	0	0	0.2	0.2	0.3	0	0	0
Mexico	San Pedro Atoyac	0.8	0.6	1.1	0	0	0	0.3	0.2	0.4	0	0	0
Mexico	San Pedro Cajonos	0.7	0.6	1.0	0	0	0	0.2	0.2	0.3	0	0	0
Mexico	San Pedro Cholula	0.5	0.3	0.6	6	4	8	0.1	0.1	0.2	2	1	3
Mexico	San Pedro Comitancillo	0.8	0.6	1.1	0	0	0	0.3	0.2	0.3	0	0	0
Mexico	San Pedro Coxcaltepec Cantaros	0.7	0.5	0.9	0	0	0	0.2	0.1	0.3	0	0	0
Mexico	San Pedro De La Cueva	0.3	0.2	0.4	0	0	0	0.1	0.1	0.1	0	0	0
Mexico	San Pedro Del Gallo	0.2	0.2	0.3	0	0	0	0.1	0.0	0.1	0	0	0
Mexico	San Pedro El Alto	0.8	0.6	1.0	1	0	1	0.2	0.2	0.3	0	0	0
Mexico	San Pedro Garza Garcia	0.1	0.1	0.1	2	1	2	0.0	0.0	0.0	0	0	0

Country	Unit	2000 mortality mean rate (per 10,000)	lower rate (per 10,000)	upper rate (per 10,000)	mean count	lower count	upper count	2017 mortality mean rate (per 10,000)	lower rate (per 10,000)	upper rate (per 10,000)	mean count	lower count	upper count
Mexico	San Pedro Huamelula	0.8	0.6	1.0	1	1	1	0.2	0.2	0.3	0	0	0
Mexico	San Pedro Huilotepec	0.8	0.6	1.0	0	0	0	0.3	0.2	0.3	0	0	0
Mexico	San Pedro Ixcatlan	0.8	0.6	1.0	2	1	2	0.2	0.2	0.3	0	0	0
Mexico	San Pedro Ixtlahuaca	0.6	0.5	0.8	1	0	1	0.2	0.1	0.3	0	0	0
Mexico	San Pedro Jaltepetongo	0.7	0.5	0.9	0	0	0	0.2	0.2	0.3	0	0	0
Mexico	San Pedro Jicayan	0.8	0.6	1.1	1	1	2	0.3	0.2	0.4	0	0	1
Mexico	San Pedro Jocotipac	0.7	0.5	0.9	0	0	0	0.2	0.1	0.3	0	0	0
Mexico	San Pedro Juchatengo	0.8	0.6	1.0	0	0	0	0.2	0.2	0.3	0	0	0
Mexico	San Pedro Lagunillas	0.3	0.2	0.3	0	0	0	0.1	0.0	0.1	0	0	0
Mexico	San Pedro Martir	0.7	0.5	0.9	0	0	0	0.2	0.2	0.3	0	0	0
Mexico	San Pedro Martir Quiechapa	0.8	0.6	1.0	0	0	0	0.2	0.2	0.3	0	0	0
Mexico	San Pedro Martir Yucuxaco	0.6	0.5	0.8	0	0	0	0.2	0.1	0.3	0	0	0
Mexico	San Pedro Mixtepec - Distr. 22 -	0.8	0.6	1.0	3	2	3	0.2	0.2	0.3	1	1	2
Mexico	San Pedro Mixtepec - Distr. 26 -	0.8	0.6	1.0	0	0	0	0.2	0.2	0.3	0	0	0
Mexico	San Pedro Molinos	0.7	0.5	0.9	0	0	0	0.2	0.2	0.3	0	0	0
Mexico	San Pedro Nopala	0.6	0.5	0.8	0	0	0	0.2	0.1	0.3	0	0	0
Mexico	San Pedro Ocopetatillo	0.7	0.5	0.9	0	0	0	0.2	0.2	0.3	0	0	0
Mexico	San Pedro Ocotepec	0.8	0.6	1.0	0	0	0	0.2	0.2	0.3	0	0	0
Mexico	San Pedro Pochutla	0.8	0.6	1.0	4	3	5	0.2	0.2	0.3	1	1	2
Mexico	San Pedro Quiatoni	0.7	0.6	1.0	1	1	1	0.2	0.2	0.3	0	0	0
Mexico	San Pedro Sochiapam	0.9	0.6	1.1	1	1	1	0.3	0.2	0.4	0	0	0
Mexico	San Pedro Tapanatepec	0.9	0.6	1.2	2	2	3	0.3	0.2	0.4	1	0	1
Mexico	San Pedro Taviche	0.7	0.5	0.9	0	0	0	0.2	0.2	0.3	0	0	0
Mexico	San Pedro Teozacoalco	0.7	0.5	0.9	0	0	0	0.2	0.2	0.3	0	0	0
Mexico	San Pedro Teutila	0.8	0.6	1.0	1	1	1	0.2	0.2	0.3	0	0	0
Mexico	San Pedro Tidaa	0.6	0.5	0.8	0	0	0	0.2	0.1	0.3	0	0	0
Mexico	San Pedro Topiltepec	0.7	0.5	0.9	0	0	0	0.2	0.2	0.3	0	0	0
Mexico	San Pedro Totolapa	0.7	0.6	1.0	0	0	0	0.2	0.2	0.3	0	0	0
Mexico	San Pedro Y San Pablo Ayutla	0.7	0.5	0.9	0	0	0	0.2	0.2	0.3	0	0	0
Mexico	San Pedro Y San Pablo Teposcolula	0.6	0.5	0.8	0	0	0	0.2	0.1	0.3	0	0	0

Country	Unit	2000 mortality mean rate (per 10,000)	2000 mortality lower rate (per 10,000)	2000 mortality upper rate (per 10,000)	2000 mortality mean count	2000 mortality lower count	2000 mortality upper count	2017 mortality mean rate (per 10,000)	2017 mortality lower rate (per 10,000)	2017 mortality upper rate (per 10,000)	2017 mortality mean count	2017 mortality lower count	2017 mortality upper count
Mexico	San Pedro Y San Pablo Tequixtepec	0.6	0.5	0.8	0	0	0	0.2	0.2	0.3	0	0	0
Mexico	San Pedro Yaneri	0.7	0.6	1.0	0	0	0	0.2	0.2	0.3	0	0	0
Mexico	San Pedro Yeloixtlahuaca	0.6	0.5	0.8	0	0	0	0.2	0.1	0.2	0	0	0
Mexico	San Pedro Yolox	0.7	0.5	0.9	0	0	0	0.2	0.2	0.3	0	0	0
Mexico	San Pedro Yucunama	0.6	0.5	0.8	0	0	0	0.2	0.1	0.3	0	0	0
Mexico	San Rafael	0.3	0.2	0.4	1	1	1	0.1	0.0	0.1	0	0	0
Mexico	San Raymundo Jalpan	0.6	0.5	0.8	0	0	0	0.2	0.2	0.3	0	0	0
Mexico	San Salvador	0.2	0.2	0.3	1	1	1	0.1	0.0	0.1	0	0	0
Mexico	San Salvador El Seco	0.5	0.4	0.6	2	1	2	0.1	0.1	0.2	1	0	1
Mexico	San Salvador El Verde	0.5	0.4	0.6	2	2	3	0.1	0.1	0.2	1	0	1
Mexico	San Salvador Huixcolotla	0.5	0.3	0.6	1	1	1	0.1	0.1	0.2	0	0	0
Mexico	San Sebastian Abasolo	0.7	0.5	0.9	0	0	0	0.2	0.2	0.3	0	0	0
Mexico	San Sebastian Coatlan	0.8	0.6	1.0	0	0	0	0.2	0.2	0.3	0	0	0
Mexico	San Sebastian Del Oeste	0.3	0.2	0.4	0	0	0	0.1	0.0	0.1	0	0	0
Mexico	San Sebastian Ixcapa	0.7	0.5	0.9	0	0	0	0.2	0.2	0.3	0	0	0
Mexico	San Sebastian Nicananduta	0.6	0.5	0.8	0	0	0	0.2	0.1	0.3	0	0	0
Mexico	San Sebastian Rio Hondo	0.7	0.5	0.9	0	0	0	0.2	0.2	0.3	0	0	0
Mexico	San Sebastian Tecomaxtlahuaca	0.6	0.5	0.8	1	0	1	0.2	0.1	0.3	0	0	0
Mexico	San Sebastian Teitipac	0.7	0.5	0.9	0	0	0	0.2	0.2	0.3	0	0	0
Mexico	San Sebastian Tlacotepec	0.7	0.5	0.9	2	1	2	0.2	0.1	0.2	0	0	0
Mexico	San Sebastian Tutla	0.6	0.5	0.8	1	1	2	0.2	0.1	0.3	0	0	1
Mexico	San Simon Almolongas	0.7	0.5	0.9	0	0	0	0.2	0.2	0.3	0	0	0
Mexico	San Simon De Guerrero	0.4	0.3	0.5	0	0	0	0.1	0.1	0.1	0	0	0
Mexico	San Simon Zahuatlan	0.6	0.5	0.8	0	0	0	0.2	0.1	0.3	0	0	0
Mexico	San Vicente Coatlan	0.7	0.5	0.9	0	0	1	0.2	0.2	0.3	0	0	0
Mexico	San Vicente Lachixio	0.7	0.5	0.9	1	0	1	0.2	0.2	0.3	0	0	0
Mexico	San Vicente Nunu	0.6	0.5	0.8	0	0	0	0.2	0.1	0.3	0	0	0
Mexico	San Vicente Tancuayalab	0.5	0.4	0.6	1	1	1	0.1	0.1	0.2	0	0	0
Mexico	Sanahcat	0.3	0.2	0.4	0	0	0	0.1	0.1	0.1	0	0	0
Mexico	Sanctorum De Lazaro Cardenas	0.3	0.2	0.4	0	0	1	0.1	0.1	0.1	0	0	0
Mexico	Santa Ana	0.7	0.6	0.9	0	0	0	0.2	0.2	0.3	0	0	0
Mexico	Santa Ana	0.3	0.2	0.4	0	0	1	0.1	0.0	0.1	0	0	0
Mexico	Santa Ana Ateixtlahuaca	0.8	0.6	1.0	0	0	0	0.2	0.2	0.3	0	0	0
Mexico	Santa Ana Cuauhtemoc	0.8	0.6	1.0	0	0	0	0.3	0.2	0.3	0	0	0

Table 3: Diarrhoea Mortality by Unit *(continued)*

Country	Unit	2000 mortality						2017 mortality					
		mean rate (per 10,000)	lower rate (per 10,000)	upper rate (per 10,000)	mean count	lower count	upper count	mean rate (per 10,000)	lower rate (per 10,000)	upper rate (per 10,000)	mean count	lower count	upper count
Mexico	Santa Ana Del Valle	0.6	0.5	0.9	0	0	0	0.2	0.1	0.3	0	0	0
Mexico	Santa Ana Maya	0.4	0.3	0.5	0	0	1	0.1	0.1	0.2	0	0	0
Mexico	Santa Ana Nopalucan	0.3	0.2	0.4	0	0	0	0.1	0.1	0.1	0	0	0
Mexico	Santa Ana Tavela	0.8	0.6	1.1	0	0	0	0.3	0.2	0.4	0	0	0
Mexico	Santa Ana Tlapacoyan	0.7	0.5	0.9	0	0	0	0.2	0.2	0.3	0	0	0
Mexico	Santa Ana Yareni	0.7	0.5	0.9	0	0	0	0.2	0.2	0.3	0	0	0
Mexico	Santa Ana Zegache	0.7	0.5	0.9	0	0	1	0.2	0.2	0.3	0	0	0
Mexico	Santa Apolonia Teacalco	0.3	0.2	0.4	0	0	0	0.1	0.1	0.1	0	0	0
Mexico	Santa Barbara	0.3	0.2	0.4	0	0	0	0.1	0.1	0.1	0	0	0
Mexico	Santa Catalina Quieri	0.7	0.6	0.9	0	0	0	0.2	0.2	0.3	0	0	0
Mexico	Santa Catarina	0.4	0.3	0.5	0	0	0	0.1	0.0	0.1	0	0	0
Mexico	Santa Catarina	0.5	0.3	0.6	1	0	1	0.1	0.1	0.2	0	0	0
Mexico	Santa Catarina	0.1	0.1	0.1	3	2	4	0.0	0.0	0.0	1	0	1
Mexico	Santa Catarina Ayometla	0.3	0.2	0.4	1	0	1	0.1	0.1	0.1	0	0	0
Mexico	Santa Catarina Cuixtla	0.7	0.5	0.9	0	0	0	0.2	0.2	0.3	0	0	0
Mexico	Santa Catarina Ixtepeji	0.7	0.5	0.9	0	0	0	0.2	0.2	0.3	0	0	0
Mexico	Santa Catarina Juquila	0.8	0.6	1.0	2	1	2	0.2	0.2	0.3	0	0	1
Mexico	Santa Catarina Lachatao	0.7	0.5	0.9	0	0	0	0.2	0.2	0.3	0	0	0
Mexico	Santa Catarina Loxicha	0.8	0.6	1.0	1	0	1	0.2	0.2	0.3	0	0	0
Mexico	Santa Catarina Mechoacan	0.8	0.6	1.1	0	0	1	0.3	0.2	0.4	0	0	0
Mexico	Santa Catarina Minas	0.7	0.5	0.9	0	0	0	0.2	0.2	0.3	0	0	0
Mexico	Santa Catarina Quiane	0.7	0.5	0.9	0	0	1	0.2	0.2	0.3	0	0	0
Mexico	Santa Catarina Quioquitani	0.8	0.6	1.0	0	0	0	0.2	0.2	0.3	0	0	0
Mexico	Santa Catarina Tayata	0.7	0.5	0.9	0	0	0	0.2	0.2	0.3	0	0	0
Mexico	Santa Catarina Ticua	0.7	0.5	0.9	0	0	0	0.2	0.2	0.3	0	0	0
Mexico	Santa Catarina Tlaltempan	0.6	0.4	0.7	0	0	0	0.2	0.1	0.2	0	0	0
Mexico	Santa Catarina Yosonotu	0.6	0.5	0.8	0	0	0	0.2	0.1	0.3	0	0	0
Mexico	Santa Catarina Zapoquila	0.6	0.5	0.8	0	0	0	0.2	0.1	0.3	0	0	0
Mexico	Santa Clara	0.2	0.2	0.3	0	0	0	0.1	0.0	0.1	0	0	0
Mexico	Santa Cruz	0.3	0.2	0.3	0	0	0	0.1	0.0	0.1	0	0	0
Mexico	Santa Cruz Acatepec	0.7	0.5	1.0	0	0	0	0.2	0.2	0.3	0	0	0
Mexico	Santa Cruz Amilpas	0.6	0.5	0.8	1	0	1	0.2	0.1	0.3	0	0	0
Mexico	Santa Cruz De Bravo	0.6	0.5	0.8	0	0	0	0.2	0.1	0.3	0	0	0
Mexico	Santa Cruz De Juventino Rosas	0.4	0.3	0.5	4	3	5	0.1	0.0	0.1	1	1	1
Mexico	Santa Cruz Itundujia	0.7	0.6	0.9	1	1	2	0.2	0.2	0.3	0	0	0

Table 3: Diarrhoea Mortality by Unit *(continued)*

Country	Unit	2000 mortality mean rate (per 10,000)	2000 mortality lower rate (per 10,000)	2000 mortality upper rate (per 10,000)	2000 mortality mean count	2000 mortality lower count	2000 mortality upper count	2017 mortality mean rate (per 10,000)	2017 mortality lower rate (per 10,000)	2017 mortality upper rate (per 10,000)	2017 mortality mean count	2017 mortality lower count	2017 mortality upper count
Mexico	Santa Cruz Mixtepec	0.7	0.5	0.9	0	0	0	0.2	0.2	0.3	0	0	0
Mexico	Santa Cruz Nundaco	0.7	0.5	0.9	0	0	0	0.2	0.2	0.3	0	0	0
Mexico	Santa Cruz Papalutla	0.7	0.5	0.9	0	0	0	0.2	0.2	0.3	0	0	0
Mexico	Santa Cruz Quilehtla	0.3	0.2	0.4	0	0	0	0.1	0.1	0.1	0	0	0
Mexico	Santa Cruz Tacache De Mina	0.7	0.5	0.8	0	0	0	0.2	0.2	0.3	0	0	0
Mexico	Santa Cruz Tacahua	0.7	0.5	0.9	0	0	0	0.2	0.2	0.3	0	0	0
Mexico	Santa Cruz Tayata	0.7	0.5	0.8	0	0	0	0.2	0.2	0.3	0	0	0
Mexico	Santa Cruz Tlaxcala	0.3	0.2	0.4	1	1	1	0.1	0.1	0.1	0	0	0
Mexico	Santa Cruz Xitla	0.8	0.6	1.0	0	0	1	0.2	0.2	0.3	0	0	0
Mexico	Santa Cruz Xoxocotlan	0.6	0.5	0.8	6	5	8	0.2	0.1	0.3	2	2	3
Mexico	Santa Cruz Zenzontepec	0.8	0.6	1.0	3	2	4	0.3	0.2	0.4	1	0	1
Mexico	Santa Elena	0.4	0.3	0.5	0	0	0	0.1	0.1	0.2	0	0	0
Mexico	Santa Gertrudis	0.7	0.5	0.9	0	0	0	0.2	0.2	0.3	0	0	0
Mexico	Santa Ines Ahuatempan	0.6	0.4	0.8	0	0	1	0.2	0.1	0.2	0	0	0
Mexico	Santa Ines De Zaragoza	0.7	0.5	0.9	0	0	0	0.2	0.2	0.3	0	0	0
Mexico	Santa Ines Del Monte	0.7	0.5	0.9	0	0	0	0.2	0.2	0.3	0	0	0
Mexico	Santa Ines Yatzeche	0.7	0.5	0.9	0	0	0	0.2	0.2	0.3	0	0	0
Mexico	Santa Isabel	0.3	0.2	0.4	0	0	0	0.1	0.1	0.1	0	0	0
Mexico	Santa Isabel Cholula	0.5	0.4	0.7	1	1	1	0.2	0.1	0.2	0	0	0
Mexico	Santa Isabel Xiloxoxtla	0.3	0.2	0.4	0	0	0	0.1	0.1	0.1	0	0	0
Mexico	Santa Lucia Del Camino	0.6	0.5	0.8	3	2	4	0.2	0.1	0.3	1	0	1
Mexico	Santa Lucia Miahuatlan	0.8	0.6	1.0	0	0	0	0.2	0.2	0.3	0	0	0
Mexico	Santa Lucia Monteverde	0.7	0.5	0.9	1	0	1	0.2	0.1	0.3	0	0	0
Mexico	Santa Lucia Ocotlan	0.7	0.5	0.9	0	0	0	0.2	0.2	0.3	0	0	0
Mexico	Santa Magdalena Jicotlan	0.6	0.5	0.8	0	0	0	0.2	0.1	0.3	0	0	0
Mexico	Santa Maria Alotepec	0.7	0.6	1.0	0	0	0	0.2	0.2	0.3	0	0	0
Mexico	Santa Maria Apazco	0.6	0.5	0.8	0	0	0	0.2	0.1	0.3	0	0	0
Mexico	Santa Maria Atzompa	0.6	0.5	0.8	4	3	5	0.2	0.1	0.3	1	1	2
Mexico	Santa Maria Camotlan	0.6	0.5	0.8	0	0	0	0.2	0.1	0.3	0	0	0
Mexico	Santa Maria Chachoapam	0.7	0.5	0.9	0	0	0	0.2	0.1	0.3	0	0	0
Mexico	Santa Maria Chilchotla	0.8	0.6	1.0	2	2	3	0.3	0.2	0.3	1	0	1
Mexico	Santa Maria Chimalapa	0.8	0.6	1.1	1	1	2	0.3	0.2	0.3	0	0	0

Table 3: Diarrhoea Mortality by Unit *(continued)*

Country	Unit	2000 mortality mean rate (per 10,000)	lower rate (per 10,000)	upper rate (per 10,000)	mean count	lower count	upper count	2017 mortality mean rate (per 10,000)	lower rate (per 10,000)	upper rate (per 10,000)	mean count	lower count	upper count
Mexico	Santa Maria Colotepec	0.8	0.6	1.0	2	2	3	0.2	0.2	0.3	1	1	1
Mexico	Santa Maria Cortijo	0.8	0.6	1.0	0	0	0	0.2	0.2	0.3	0	0	0
Mexico	Santa Maria Coyotepec	0.7	0.5	0.9	0	0	0	0.2	0.2	0.3	0	0	0
Mexico	Santa Maria De La Paz	0.3	0.2	0.4	0	0	0	0.1	0.1	0.1	0	0	0
Mexico	Santa Maria De Los Angeles	0.3	0.2	0.3	0	0	0	0.1	0.0	0.1	0	0	0
Mexico	Santa Maria Del Oro	0.3	0.2	0.3	1	1	1	0.1	0.0	0.1	0	0	0
Mexico	Santa Maria Del Oro	0.3	0.2	0.4	0	0	0	0.1	0.0	0.1	0	0	0
Mexico	Santa Maria Del Rio	0.4	0.3	0.5	2	2	3	0.1	0.1	0.2	1	0	1
Mexico	Santa Maria Del Rosario	0.6	0.5	0.8	0	0	0	0.2	0.1	0.3	0	0	0
Mexico	Santa Maria Del Tule	0.6	0.5	0.8	0	0	0	0.2	0.2	0.3	0	0	0
Mexico	Santa Maria Ecatepec	0.8	0.6	1.0	0	0	1	0.2	0.2	0.3	0	0	0
Mexico	Santa Maria Guelace	0.7	0.5	0.9	0	0	0	0.2	0.2	0.3	0	0	0
Mexico	Santa Maria Guienagati	0.8	0.6	1.1	1	0	1	0.3	0.2	0.3	0	0	0
Mexico	Santa Maria Huatulco	0.7	0.6	1.0	3	2	4	0.2	0.2	0.3	1	1	2
Mexico	Santa Maria Huazolotitlan	0.8	0.6	1.0	1	1	2	0.3	0.2	0.4	0	0	0
Mexico	Santa Maria Ipalapa	0.8	0.6	1.1	1	0	1	0.3	0.2	0.4	0	0	0
Mexico	Santa Maria Ixcatlan	0.7	0.5	0.9	0	0	0	0.2	0.2	0.3	0	0	0
Mexico	Santa Maria Jacatepec	0.8	0.6	1.1	1	1	2	0.3	0.2	0.3	0	0	0
Mexico	Santa Maria Jalapa Del Marques	0.8	0.6	1.0	1	1	2	0.3	0.2	0.3	0	0	0
Mexico	Santa Maria Jaltianguis	0.7	0.5	0.9	0	0	0	0.2	0.2	0.3	0	0	0
Mexico	Santa Maria La Asuncion	0.7	0.5	0.9	0	0	0	0.2	0.1	0.3	0	0	0
Mexico	Santa Maria Lachixio	0.7	0.5	0.9	0	0	0	0.2	0.2	0.3	0	0	0
Mexico	Santa Maria Mixtequilla	0.8	0.6	1.1	0	0	0	0.3	0.2	0.3	0	0	0
Mexico	Santa Maria Nativitas	0.6	0.5	0.8	0	0	0	0.2	0.1	0.3	0	0	0
Mexico	Santa Maria Nduayaco	0.7	0.5	0.8	0	0	0	0.2	0.2	0.3	0	0	0
Mexico	Santa Maria Ozolotepec	0.7	0.6	0.9	1	0	1	0.2	0.2	0.3	0	0	0
Mexico	Santa Maria Papalo	0.7	0.5	0.9	0	0	0	0.2	0.2	0.3	0	0	0
Mexico	Santa Maria Penoles	0.7	0.5	0.9	1	1	1	0.2	0.2	0.3	0	0	0
Mexico	Santa Maria Petapa	0.8	0.6	1.0	2	1	2	0.2	0.2	0.3	1	0	1
Mexico	Santa Maria Quiegolani	0.7	0.6	1.0	0	0	0	0.2	0.2	0.3	0	0	0

Table 3: Diarrhoea Mortality by Unit *(continued)*

Country	Unit	2000 mortality mean rate (per 10,000)	lower rate (per 10,000)	upper rate (per 10,000)	mean count	lower count	upper count	2017 mortality mean rate (per 10,000)	lower rate (per 10,000)	upper rate (per 10,000)	mean count	lower count	upper count
Mexico	Santa Maria Sola	0.7	0.6	0.9	0	0	0	0.2	0.2	0.3	0	0	0
Mexico	Santa Maria Tataltepec	0.7	0.5	0.9	0	0	0	0.2	0.2	0.3	0	0	0
Mexico	Santa Maria Tecomavaca	0.7	0.5	0.9	0	0	0	0.2	0.2	0.3	0	0	0
Mexico	Santa Maria Temaxcalapa	0.8	0.6	1.1	0	0	0	0.2	0.2	0.3	0	0	0
Mexico	Santa Maria Temaxcaltepec	0.8	0.6	1.0	0	0	0	0.3	0.2	0.4	0	0	0
Mexico	Santa Maria Teopoxco	0.7	0.5	0.9	0	0	1	0.2	0.2	0.3	0	0	0
Mexico	Santa Maria Tepantlali	0.7	0.6	1.0	0	0	0	0.2	0.2	0.3	0	0	0
Mexico	Santa Maria Texcatitlan	0.7	0.5	0.9	0	0	0	0.2	0.1	0.3	0	0	0
Mexico	Santa Maria Tlahuitoltepec	0.7	0.5	0.9	1	1	1	0.2	0.2	0.3	0	0	0
Mexico	Santa Maria Tlalixtac	0.7	0.6	1.0	0	0	0	0.2	0.2	0.3	0	0	0
Mexico	Santa Maria Tonameca	0.8	0.6	1.0	3	3	4	0.2	0.2	0.3	1	1	1
Mexico	Santa Maria Totolapilla	0.8	0.6	1.1	0	0	0	0.3	0.2	0.3	0	0	0
Mexico	Santa Maria Xadani	0.8	0.6	1.1	2	1	2	0.3	0.2	0.4	0	0	0
Mexico	Santa Maria Yalina	0.7	0.5	1.0	0	0	0	0.2	0.2	0.3	0	0	0
Mexico	Santa Maria Yavesia	0.7	0.5	0.9	0	0	0	0.2	0.2	0.3	0	0	0
Mexico	Santa Maria Yolotepec	0.7	0.6	0.9	0	0	0	0.2	0.2	0.3	0	0	0
Mexico	Santa Maria Yosoyua	0.7	0.5	0.9	0	0	0	0.2	0.2	0.3	0	0	0
Mexico	Santa Maria Yucuhiti	0.7	0.5	0.9	1	1	1	0.2	0.2	0.3	0	0	0
Mexico	Santa Maria Zacatepec	0.8	0.6	1.0	2	1	2	0.3	0.2	0.4	1	0	1
Mexico	Santa Maria Zaniza	0.8	0.6	1.0	0	0	0	0.3	0.2	0.4	0	0	0
Mexico	Santa Maria Zoquitlan	0.8	0.6	1.0	0	0	1	0.2	0.2	0.3	0	0	0
Mexico	Santiago	0.1	0.1	0.2	0	0	1	0.0	0.0	0.0	0	0	0
Mexico	Santiago Amoltepec	0.8	0.6	1.1	2	1	2	0.3	0.2	0.4	1	0	1
Mexico	Santiago Apoala	0.6	0.5	0.8	0	0	0	0.2	0.1	0.3	0	0	0
Mexico	Santiago Apostol	0.7	0.5	0.9	0	0	1	0.2	0.2	0.3	0	0	0
Mexico	Santiago Astata	0.8	0.6	1.0	0	0	0	0.2	0.2	0.3	0	0	0
Mexico	Santiago Atitlan	0.7	0.6	1.0	0	0	0	0.2	0.2	0.3	0	0	0
Mexico	Santiago Ayuquililla	0.7	0.5	0.8	0	0	0	0.2	0.1	0.3	0	0	0
Mexico	Santiago Cacaloxtepec	0.6	0.5	0.8	0	0	0	0.2	0.1	0.3	0	0	0
Mexico	Santiago Camotlan	0.8	0.6	1.0	0	0	1	0.2	0.2	0.3	0	0	0
Mexico	Santiago Chazumba	0.6	0.5	0.8	0	0	0	0.2	0.1	0.3	0	0	0
Mexico	Santiago Choapam	0.8	0.6	1.1	0	0	1	0.2	0.2	0.3	0	0	0
Mexico	Santiago Comaltepec	0.7	0.6	1.0	0	0	0	0.2	0.2	0.3	0	0	0

Table 3: Diarrhoea Mortality by Unit *(continued)*

Country	Unit	2000 mortality						2017 mortality					
		mean rate (per 10,000)	lower rate (per 10,000)	upper rate (per 10,000)	mean count	lower count	upper count	mean rate (per 10,000)	lower rate (per 10,000)	upper rate (per 10,000)	mean count	lower count	upper count
Mexico	Santiago De Anaya	0.2	0.2	0.3	0	0	0	0.1	0.0	0.1	0	0	0
Mexico	Santiago Del Rio	0.6	0.5	0.8	0	0	0	0.2	0.1	0.3	0	0	0
Mexico	Santiago El Pinar	1.1	0.8	1.5	0	0	1	0.3	0.2	0.4	0	0	0
Mexico	Santiago Huajolotitlan	0.6	0.5	0.8	0	0	0	0.2	0.1	0.3	0	0	0
Mexico	Santiago Huauclilla	0.7	0.5	0.9	0	0	0	0.2	0.2	0.3	0	0	0
Mexico	Santiago Ihuitlan Plumas	0.6	0.5	0.8	0	0	0	0.2	0.1	0.3	0	0	0
Mexico	Santiago Ixcuintepec	0.8	0.6	1.1	0	0	0	0.3	0.2	0.3	0	0	0
Mexico	Santiago Ixcuintla	0.3	0.2	0.4	3	2	4	0.1	0.0	0.1	1	0	1
Mexico	Santiago Ixtayutla	0.9	0.6	1.1	2	1	2	0.3	0.2	0.4	0	0	1
Mexico	Santiago Jamiltepec	0.8	0.6	1.1	2	2	3	0.3	0.2	0.4	1	0	1
Mexico	Santiago Jocotepec	0.8	0.6	1.0	2	1	2	0.2	0.2	0.3	0	0	1
Mexico	Santiago Juxtlahuaca	0.7	0.5	0.9	4	3	5	0.2	0.2	0.3	1	1	1
Mexico	Santiago Lachiguiri	0.8	0.6	1.1	1	0	1	0.3	0.2	0.3	0	0	0
Mexico	Santiago Lalopa	0.8	0.6	1.0	0	0	0	0.2	0.2	0.3	0	0	0
Mexico	Santiago Laollaga	0.8	0.6	1.1	0	0	0	0.3	0.2	0.3	0	0	0
Mexico	Santiago Laxopa	0.7	0.5	0.9	0	0	0	0.2	0.2	0.3	0	0	0
Mexico	Santiago Llano Grande	0.8	0.6	1.0	0	0	0	0.2	0.2	0.3	0	0	0
Mexico	Santiago Maravatio	0.4	0.3	0.5	0	0	0	0.1	0.0	0.1	0	0	0
Mexico	Santiago Matatlan	0.7	0.5	0.9	1	1	1	0.2	0.2	0.3	0	0	0
Mexico	Santiago Miahuatlan	0.5	0.4	0.7	1	1	2	0.2	0.1	0.2	1	0	1
Mexico	Santiago Miltepec	0.6	0.5	0.8	0	0	0	0.2	0.1	0.3	0	0	0
Mexico	Santiago Minas	0.8	0.6	1.0	0	0	0	0.2	0.2	0.3	0	0	0
Mexico	Santiago Nacaltepec	0.7	0.5	0.9	0	0	0	0.2	0.2	0.3	0	0	0
Mexico	Santiago Nejapilla	0.6	0.5	0.9	0	0	0	0.2	0.1	0.3	0	0	0
Mexico	Santiago Niltepec	0.8	0.6	1.0	0	0	0	0.3	0.2	0.3	0	0	0
Mexico	Santiago Nundiche	0.7	0.5	0.8	0	0	0	0.2	0.1	0.3	0	0	0
Mexico	Santiago Nuyoo	0.7	0.5	0.9	0	0	0	0.2	0.1	0.3	0	0	0
Mexico	Santiago Papasquiaro	0.2	0.2	0.3	1	1	2	0.1	0.0	0.1	0	0	0
Mexico	Santiago Pinotepa Nacional	0.8	0.6	1.0	5	4	6	0.3	0.2	0.3	2	1	2
Mexico	Santiago Sochiapa	0.4	0.3	0.6	0	0	0	0.1	0.1	0.1	0	0	0
Mexico	Santiago Suchilquitongo	0.7	0.5	0.8	1	1	1	0.2	0.2	0.3	0	0	0
Mexico	Santiago Tamazola	0.7	0.5	0.8	0	0	1	0.2	0.2	0.3	0	0	0
Mexico	Santiago Tapextla	0.8	0.6	1.0	0	0	0	0.2	0.2	0.3	0	0	0
Mexico	Santiago Tenango	0.7	0.5	0.9	0	0	0	0.2	0.2	0.3	0	0	0
Mexico	Santiago Tepetlapa	0.6	0.5	0.8	0	0	0	0.2	0.1	0.3	0	0	0

Table 3: Diarrhoea Mortality by Unit *(continued)*

Country	Unit	2000 mortality mean rate (per 10,000)	2000 lower rate (per 10,000)	2000 upper rate (per 10,000)	2000 mean count	2000 lower count	2000 upper count	2017 mortality mean rate (per 10,000)	2017 lower rate (per 10,000)	2017 upper rate (per 10,000)	2017 mean count	2017 lower count	2017 upper count
Mexico	Santiago Tetepec	0.8	0.6	1.0	1	0	1	0.3	0.2	0.4	0	0	0
Mexico	Santiago Texcalcingo	0.7	0.5	0.9	0	0	0	0.3	0.2	0.3	0	0	0
Mexico	Santiago Textitlan	0.7	0.5	0.9	1	0	1	0.2	0.2	0.3	0	0	0
Mexico	Santiago Tilantongo	0.7	0.5	0.9	0	0	0	0.2	0.2	0.3	0	0	0
Mexico	Santiago Tillo	0.7	0.5	0.9	0	0	0	0.2	0.2	0.3	0	0	0
Mexico	Santiago Tlazoyaltepec	0.7	0.5	0.8	0	0	1	0.2	0.2	0.3	0	0	0
Mexico	Santiago Tulantepec De Lugo Guerrero	0.2	0.2	0.3	1	1	1	0.1	0.0	0.1	0	0	0
Mexico	Santiago Tuxtla	0.4	0.3	0.6	3	2	4	0.1	0.1	0.1	0	0	1
Mexico	Santiago Xanica	0.8	0.6	1.0	0	0	0	0.2	0.2	0.3	0	0	0
Mexico	Santiago Xiacui	0.7	0.5	0.9	0	0	0	0.2	0.2	0.3	0	0	0
Mexico	Santiago Yaitepec	0.8	0.6	1.0	0	0	0	0.2	0.2	0.3	0	0	0
Mexico	Santiago Yaveo	0.8	0.6	1.1	1	1	1	0.3	0.2	0.3	0	0	0
Mexico	Santiago Yolomecatl	0.6	0.5	0.8	0	0	0	0.2	0.1	0.3	0	0	0
Mexico	Santiago Yosondua	0.7	0.5	0.9	1	1	1	0.2	0.2	0.3	0	0	0
Mexico	Santiago Yucuyachi	0.6	0.5	0.8	0	0	0	0.2	0.2	0.3	0	0	0
Mexico	Santiago Zacatepec	0.8	0.6	1.0	0	0	0	0.2	0.2	0.3	0	0	0
Mexico	Santiago Zoochila	0.7	0.6	1.0	0	0	0	0.2	0.2	0.3	0	0	0
Mexico	Santo Domingo	0.4	0.3	0.5	0	0	1	0.1	0.1	0.2	0	0	0
Mexico	Santo Domingo Albarradas	0.7	0.5	0.9	0	0	0	0.2	0.2	0.3	0	0	0
Mexico	Santo Domingo Armenta	0.8	0.6	1.0	0	0	1	0.3	0.2	0.3	0	0	0
Mexico	Santo Domingo Chihuitan	0.8	0.6	1.1	0	0	0	0.3	0.2	0.3	0	0	0
Mexico	Santo Domingo De Morelos	0.8	0.6	1.0	1	1	1	0.2	0.2	0.3	0	0	1
Mexico	Santo Domingo Ingenio	0.8	0.6	1.0	1	0	1	0.3	0.2	0.3	0	0	0
Mexico	Santo Domingo Ixcatlan	0.7	0.5	0.9	0	0	0	0.2	0.2	0.3	0	0	0
Mexico	Santo Domingo Nuxaa	0.7	0.5	0.9	0	0	1	0.2	0.2	0.3	0	0	0
Mexico	Santo Domingo Ozolotepec	0.7	0.5	0.9	0	0	0	0.2	0.2	0.3	0	0	0
Mexico	Santo Domingo Petapa	0.8	0.6	1.0	1	1	1	0.3	0.2	0.3	0	0	0
Mexico	Santo Domingo Roayaga	0.8	0.6	1.0	0	0	0	0.2	0.2	0.3	0	0	0
Mexico	Santo Domingo Tehuantepec	0.7	0.5	1.0	6	4	7	0.2	0.2	0.3	2	1	2
Mexico	Santo Domingo Teojomulco	0.8	0.6	1.0	0	0	1	0.2	0.2	0.3	0	0	0
Mexico	Santo Domingo Tepuxtepec	0.7	0.5	0.9	0	0	0	0.2	0.2	0.3	0	0	0
Mexico	Santo Domingo Tlatayapam	0.7	0.5	0.9	0	0	0	0.2	0.1	0.3	0	0	0
Mexico	Santo Domingo Tomaltepec	0.7	0.5	0.9	0	0	0	0.2	0.2	0.3	0	0	0
Mexico	Santo Domingo Tonala	0.6	0.5	0.8	1	0	1	0.2	0.1	0.3	0	0	0

Table 3: Diarrhoea Mortality by Unit *(continued)*

Country	Unit	2000 mortality mean rate (per 10,000)	lower rate (per 10,000)	upper rate (per 10,000)	mean count	lower count	upper count	2017 mortality mean rate (per 10,000)	lower rate (per 10,000)	upper rate (per 10,000)	mean count	lower count	upper count
Mexico	Santo Domingo Tonaltepec	0.6	0.5	0.8	0	0	0	0.2	0.1	0.3	0	0	0
Mexico	Santo Domingo Xagacia	0.7	0.6	1.0	0	0	0	0.2	0.2	0.3	0	0	0
Mexico	Santo Domingo Yanhuitlan	0.7	0.5	0.9	0	0	0	0.2	0.1	0.3	0	0	0
Mexico	Santo Domingo Yodohino	0.7	0.5	0.8	0	0	0	0.2	0.1	0.3	0	0	0
Mexico	Santo Domingo Zanatepec	0.8	0.6	1.1	1	1	2	0.3	0.2	0.4	0	0	0
Mexico	Santo Tomas	0.4	0.3	0.4	0	0	1	0.1	0.1	0.1	0	0	0
Mexico	Santo Tomas Hueyotlipan	0.5	0.3	0.6	0	0	1	0.1	0.1	0.2	0	0	0
Mexico	Santo Tomas Jalieza	0.7	0.5	0.9	0	0	0	0.2	0.2	0.3	0	0	0
Mexico	Santo Tomas Mazaltepec	0.7	0.5	0.9	0	0	0	0.2	0.2	0.3	0	0	0
Mexico	Santo Tomas Ocotepec	0.6	0.5	0.8	0	0	1	0.2	0.1	0.3	0	0	0
Mexico	Santo Tomas Tamazulapan	0.7	0.6	0.9	0	0	0	0.2	0.2	0.3	0	0	0
Mexico	Santos Reyes Nopala	0.8	0.6	1.1	2	2	3	0.3	0.2	0.4	1	0	1
Mexico	Santos Reyes Papalo	0.7	0.5	0.9	0	0	0	0.2	0.2	0.3	0	0	0
Mexico	Santos Reyes Tepejillo	0.6	0.5	0.8	0	0	0	0.2	0.1	0.3	0	0	0
Mexico	Santos Reyes Yucuna	0.7	0.5	0.8	0	0	0	0.2	0.1	0.3	0	0	0
Mexico	Saric	0.2	0.2	0.3	0	0	0	0.1	0.0	0.1	0	0	0
Mexico	Satevo	0.3	0.2	0.4	0	0	0	0.1	0.1	0.1	0	0	0
Mexico	Saucillo	0.3	0.2	0.4	1	1	1	0.1	0.1	0.1	0	0	0
Mexico	Sayula	0.3	0.2	0.3	1	1	1	0.1	0.0	0.1	0	0	0
Mexico	Sayula De Aleman	0.4	0.3	0.5	2	1	2	0.1	0.1	0.1	0	0	0
Mexico	Senguio	0.4	0.3	0.5	1	1	1	0.1	0.1	0.2	0	0	0
Mexico	Seye	0.3	0.2	0.4	0	0	0	0.1	0.1	0.1	0	0	0
Mexico	Sierra Mojada	0.2	0.1	0.3	0	0	0	0.1	0.0	0.1	0	0	0
Mexico	Silacayoapam	0.6	0.5	0.8	1	1	1	0.2	0.1	0.3	0	0	0
Mexico	Silao	0.3	0.3	0.4	7	5	9	0.1	0.0	0.1	2	1	2
Mexico	Siltepec	1.2	0.9	1.6	6	5	8	0.3	0.2	0.4	2	1	2
Mexico	Simojovel	1.2	0.9	1.6	6	4	8	0.3	0.2	0.4	3	2	3
Mexico	Sinaloa	0.2	0.1	0.2	2	1	3	0.1	0.0	0.1	0	0	1
Mexico	Sinanche	0.3	0.2	0.4	0	0	0	0.1	0.1	0.1	0	0	0
Mexico	Singuilucan	0.2	0.2	0.3	0	0	1	0.1	0.0	0.1	0	0	0
Mexico	Sitala	1.2	0.9	1.6	2	1	2	0.3	0.2	0.4	1	1	1
Mexico	Sitio De Xitlapehua	0.7	0.6	0.9	0	0	0	0.2	0.2	0.3	0	0	0
Mexico	Sochiapa	0.4	0.3	0.5	0	0	0	0.1	0.0	0.1	0	0	0
Mexico	Socoltenango	1.2	0.9	1.6	3	2	4	0.3	0.2	0.4	1	1	1
Mexico	Soconusco	0.4	0.3	0.5	0	0	1	0.1	0.1	0.1	0	0	0
Mexico	Soledad Atzompa	0.3	0.3	0.5	1	1	2	0.1	0.0	0.1	0	0	0
Mexico	Soledad De Doblado	0.4	0.3	0.5	1	1	2	0.1	0.1	0.1	0	0	0
Mexico	Soledad De Graciano Sanchez	0.4	0.3	0.5	8	6	11	0.1	0.1	0.2	3	2	5
Mexico	Soledad Etla	0.6	0.5	0.8	0	0	0	0.2	0.2	0.3	0	0	0
Mexico	Solidaridad	0.3	0.2	0.4	4	3	5	0.1	0.1	0.1	2	2	3
Mexico	Solosuchiapa	1.2	0.9	1.6	1	1	2	0.3	0.2	0.4	0	0	1
Mexico	Soltepec	0.5	0.4	0.7	1	1	1	0.1	0.1	0.2	0	0	0

Table 3: Diarrhoea Mortality by Unit *(continued)*

Country	Unit	2000 mortality mean rate (per 10,000)	lower rate (per 10,000)	upper rate (per 10,000)	mean count	lower count	upper count	2017 mortality mean rate (per 10,000)	lower rate (per 10,000)	upper rate (per 10,000)	mean count	lower count	upper count
Mexico	Sombrerete	0.3	0.2	0.4	2	2	3	0.1	0.0	0.1	0	0	1
Mexico	Soteapan	0.4	0.3	0.6	2	2	3	0.1	0.1	0.1	0	0	1
Mexico	Soto La Marina	0.2	0.1	0.2	0	0	0	0.1	0.0	0.1	0	0	0
Mexico	Sotuta	0.3	0.2	0.4	0	0	0	0.1	0.1	0.1	0	0	0
Mexico	Soyalo	1.1	0.8	1.5	1	1	2	0.3	0.2	0.4	0	0	1
Mexico	Soyaniquilpan De Juarez	0.3	0.2	0.4	0	0	0	0.1	0.1	0.1	0	0	0
Mexico	Soyopa	0.3	0.2	0.4	0	0	0	0.1	0.1	0.1	0	0	0
Mexico	Suaqui Grande	0.3	0.2	0.4	0	0	0	0.1	0.1	0.1	0	0	0
Mexico	Suchiapa	1.2	0.9	1.5	3	2	3	0.3	0.2	0.4	1	1	1
Mexico	Suchiate	1.4	1.0	1.8	6	4	7	0.4	0.3	0.5	2	1	2
Mexico	Suchil	0.2	0.2	0.3	0	0	0	0.1	0.0	0.1	0	0	0
Mexico	Sucila	0.3	0.3	0.5	0	0	0	0.1	0.1	0.2	0	0	0
Mexico	Sudzal	0.3	0.2	0.4	0	0	0	0.1	0.1	0.1	0	0	0
Mexico	Sultepec	0.4	0.3	0.5	1	1	2	0.1	0.1	0.1	0	0	0
Mexico	Suma	0.3	0.2	0.4	0	0	0	0.1	0.1	0.1	0	0	0
Mexico	Sunuapa	1.2	0.9	1.6	0	0	0	0.3	0.2	0.4	0	0	0
Mexico	Susticacan	0.3	0.2	0.4	0	0	0	0.1	0.0	0.1	0	0	0
Mexico	Susupuato	0.4	0.3	0.5	1	0	1	0.1	0.1	0.2	0	0	0
Mexico	Tabasco	0.3	0.2	0.4	1	0	1	0.1	0.1	0.1	0	0	0
Mexico	Tacambaro	0.4	0.3	0.5	4	3	5	0.1	0.1	0.2	1	1	2
Mexico	Tacotalpa	0.3	0.2	0.4	2	1	3	0.1	0.1	0.2	0	0	1
Mexico	Tahdziu	0.4	0.3	0.5	0	0	0	0.1	0.1	0.2	0	0	0
Mexico	Tahmek	0.3	0.2	0.4	0	0	0	0.1	0.1	0.1	0	0	0
Mexico	Tala	0.3	0.2	0.4	2	1	2	0.1	0.0	0.1	0	0	1
Mexico	Talpa De Allende	0.3	0.2	0.4	0	0	1	0.1	0.0	0.1	0	0	0
Mexico	Tamalin	0.4	0.3	0.5	0	0	1	0.1	0.1	0.1	0	0	0
Mexico	Tamasopo	0.5	0.3	0.6	2	1	2	0.1	0.1	0.2	0	0	1
Mexico	Tamazula	0.3	0.2	0.4	1	1	2	0.1	0.0	0.1	0	0	0
Mexico	Tamazula De Gordiano	0.3	0.2	0.4	1	1	1	0.1	0.0	0.1	0	0	0
Mexico	Tamazulapam Del Espiritu Santo	0.7	0.6	1.0	1	1	1	0.2	0.2	0.3	0	0	0
Mexico	Tamazunchale	0.5	0.4	0.6	7	5	9	0.1	0.1	0.2	1	1	2
Mexico	Tamiahua	0.4	0.3	0.5	1	1	2	0.1	0.1	0.1	0	0	0
Mexico	Tampacan	0.5	0.4	0.6	1	1	1	0.1	0.1	0.2	0	0	0
Mexico	Tampamolon Corona	0.5	0.4	0.6	1	1	1	0.1	0.1	0.2	0	0	0
Mexico	Tampico	0.2	0.1	0.2	5	4	6	0.1	0.0	0.1	1	1	1
Mexico	Tampico Alto	0.4	0.3	0.5	0	0	1	0.1	0.1	0.1	0	0	0
Mexico	Tamuin	0.5	0.3	0.6	2	2	3	0.1	0.1	0.2	1	0	1
Mexico	Tancanhuitz	0.5	0.4	0.6	1	1	2	0.1	0.1	0.2	0	0	0
Mexico	Tancitaro	0.4	0.3	0.5	2	1	2	0.1	0.1	0.2	0	0	1
Mexico	Tancoco	0.4	0.3	0.5	1	0	1	0.1	0.1	0.1	0	0	0
Mexico	Tanetze De Zaragoza	0.8	0.6	1.0	0	0	0	0.2	0.2	0.3	0	0	0
Mexico	Tangamandapio	0.4	0.3	0.5	1	1	2	0.1	0.1	0.2	0	0	1
Mexico	Tangancicuaro	0.4	0.3	0.5	2	1	2	0.1	0.1	0.2	0	0	1
Mexico	Tanhuato	0.4	0.3	0.5	1	1	1	0.1	0.1	0.2	0	0	0
Mexico	Taniche	0.7	0.5	0.9	0	0	0	0.2	0.2	0.3	0	0	0
Mexico	Tanlajas	0.5	0.4	0.6	1	1	1	0.1	0.1	0.2	0	0	0
Mexico	Tanquian De Escobedo	0.5	0.4	0.6	1	1	1	0.1	0.1	0.2	0	0	0
Mexico	Tantima	0.4	0.3	0.5	1	1	1	0.1	0.1	0.1	0	0	0
Mexico	Tantoyuca	0.4	0.3	0.5	6	4	7	0.1	0.1	0.1	1	1	1
Mexico	Tapachula	1.2	0.8	1.5	35	26	47	0.3	0.3	0.4	13	10	17
Mexico	Tapalapa	1.1	0.8	1.5	1	1	1	0.3	0.2	0.4	0	0	0
Mexico	Tapalpa	0.3	0.2	0.3	1	0	1	0.1	0.0	0.1	0	0	0
Mexico	Tapilula	1.1	0.8	1.5	1	1	2	0.3	0.2	0.4	0	0	1

Table 3: Diarrhoea Mortality by Unit *(continued)*

Country	Unit	2000 mortality mean rate (per 10,000)	lower rate (per 10,000)	upper rate (per 10,000)	mean count	lower count	upper count	2017 mortality mean rate (per 10,000)	lower rate (per 10,000)	upper rate (per 10,000)	mean count	lower count	upper count
Mexico	Tarandacuao	0.4	0.3	0.5	1	0	1	0.1	0.1	0.1	0	0	0
Mexico	Taretan	0.5	0.4	0.6	1	1	1	0.1	0.1	0.2	0	0	0
Mexico	Tarimbaro	0.4	0.3	0.5	2	2	3	0.1	0.1	0.2	2	1	2
Mexico	Tarimoro	0.4	0.3	0.5	2	1	2	0.1	0.0	0.1	0	0	0
Mexico	Tasquillo	0.2	0.2	0.3	0	0	0	0.1	0.0	0.1	0	0	0
Mexico	Tatahuicapan De Juarez	0.4	0.3	0.6	1	0	1	0.1	0.1	0.1	0	0	0
Mexico	Tataltepec De Valdes	0.8	0.6	1.0	1	1	1	0.3	0.2	0.4	0	0	0
Mexico	Tatatila	0.3	0.3	0.5	0	0	1	0.1	0.0	0.1	0	0	0
Mexico	Taxco De Alarcon	0.9	0.7	1.2	13	9	16	0.2	0.1	0.3	2	1	3
Mexico	Teabo	0.3	0.2	0.5	0	0	0	0.1	0.1	0.2	0	0	0
Mexico	Teapa	0.3	0.2	0.4	2	2	3	0.1	0.1	0.2	1	0	1
Mexico	Tecali De Herrera	0.5	0.4	0.7	1	1	2	0.1	0.1	0.2	0	0	1
Mexico	Tecalitlan	0.3	0.2	0.4	0	0	1	0.1	0.0	0.1	0	0	0
Mexico	Tecamac	0.3	0.2	0.4	10	8	13	0.1	0.1	0.1	5	3	6
Mexico	Tecamachalco	0.5	0.4	0.6	5	4	6	0.1	0.1	0.2	1	1	2
Mexico	Tecate	0.2	0.1	0.2	2	1	2	0.0	0.0	0.1	0	0	1
Mexico	Techaluta De Montenegro	0.3	0.2	0.4	0	0	0	0.1	0.0	0.1	0	0	0
Mexico	Tecoanapa	1.1	0.9	1.4	7	5	8	0.2	0.1	0.3	1	1	2
Mexico	Tecoh	0.3	0.2	0.4	1	0	1	0.1	0.1	0.1	0	0	0
Mexico	Tecolotlan	0.3	0.2	0.4	0	0	1	0.1	0.0	0.1	0	0	0
Mexico	Tecolutla	0.3	0.2	0.4	1	1	1	0.1	0.0	0.1	0	0	0
Mexico	Tecoman	0.3	0.2	0.4	4	3	5	0.1	0.1	0.1	1	1	2
Mexico	Tecomatlan	0.6	0.5	0.8	0	0	0	0.2	0.1	0.2	0	0	0
Mexico	Tecozautla	0.2	0.2	0.3	1	1	1	0.1	0.0	0.1	0	0	0
Mexico	Tecpan De Galeana	1.0	0.8	1.3	8	6	10	0.2	0.1	0.3	1	1	2
Mexico	Tecpatan	1.1	0.8	1.5	8	6	10	0.3	0.2	0.4	2	1	2
Mexico	Tecuala	0.3	0.2	0.4	1	1	2	0.1	0.0	0.1	0	0	0
Mexico	Tehuacan	0.5	0.4	0.6	18	14	24	0.1	0.1	0.2	5	3	7
Mexico	Tehuipango	0.4	0.3	0.5	1	1	2	0.1	0.1	0.1	0	0	0
Mexico	Tehuitzingo	0.6	0.5	0.8	1	1	1	0.2	0.1	0.2	0	0	0
Mexico	Tejupilco	0.4	0.3	0.5	3	2	4	0.1	0.1	0.1	1	1	1
Mexico	Tekal De Venegas	0.3	0.2	0.4	0	0	0	0.1	0.1	0.1	0	0	0
Mexico	Tekanto	0.3	0.2	0.4	0	0	0	0.1	0.1	0.1	0	0	0
Mexico	Tekax	0.4	0.3	0.5	2	1	2	0.1	0.1	0.2	1	0	1
Mexico	Tekit	0.3	0.2	0.4	0	0	0	0.1	0.1	0.1	0	0	0
Mexico	Tekom	0.3	0.2	0.4	0	0	0	0.1	0.1	0.1	0	0	0
Mexico	Telchac Pueblo	0.3	0.2	0.4	0	0	0	0.1	0.1	0.1	0	0	0
Mexico	Telchac Puerto	0.3	0.2	0.4	0	0	0	0.1	0.1	0.1	0	0	0
Mexico	Teloloapan	1.0	0.8	1.2	8	6	10	0.2	0.1	0.3	1	1	2
Mexico	Temamatla	0.3	0.3	0.4	1	0	1	0.1	0.1	0.1	0	0	0
Mexico	Temapache	0.3	0.2	0.4	4	3	6	0.1	0.1	0.1	1	0	1
Mexico	Temascalapa	0.3	0.2	0.4	1	1	2	0.1	0.1	0.1	0	0	1
Mexico	Temascalcingo	0.3	0.3	0.4	2	2	3	0.1	0.1	0.1	1	0	1
Mexico	Temascaltepec	0.4	0.3	0.4	1	1	2	0.1	0.1	0.1	0	0	0
Mexico	Temax	0.3	0.2	0.4	0	0	1	0.1	0.1	0.1	0	0	0
Mexico	Temixco	0.3	0.2	0.3	3	3	4	0.1	0.1	0.1	1	1	1
Mexico	Temoac	0.3	0.2	0.3	0	0	0	0.1	0.1	0.1	0	0	0
Mexico	Temoaya	0.3	0.3	0.4	3	3	4	0.1	0.1	0.1	1	1	1
Mexico	Temosachi	0.3	0.2	0.4	0	0	0	0.1	0.1	0.1	0	0	0
Mexico	Temozon	0.3	0.2	0.4	1	0	1	0.1	0.1	0.2	0	0	0
Mexico	Tempoal	0.4	0.3	0.5	2	1	2	0.1	0.1	0.1	0	0	0
Mexico	Tenabo	0.3	0.2	0.4	0	0	0	0.1	0.1	0.1	0	0	0
Mexico	Tenamaxtlan	0.3	0.2	0.3	0	0	0	0.1	0.0	0.1	0	0	0
Mexico	Tenampa	0.4	0.3	0.5	0	0	0	0.1	0.1	0.1	0	0	0
Mexico	Tenampulco	0.5	0.4	0.7	1	0	1	0.1	0.1	0.2	0	0	0
Mexico	Tenancingo	0.3	0.2	0.4	0	0	1	0.1	0.1	0.1	0	0	0

Table 3: Diarrhoea Mortality by Unit *(continued)*

Country	Unit	2000 mortality mean rate (per 10,000)	lower rate (per 10,000)	upper rate (per 10,000)	mean count	lower count	upper count	2017 mortality mean rate (per 10,000)	lower rate (per 10,000)	upper rate (per 10,000)	mean count	lower count	upper count
Mexico	Tenancingo	0.4	0.3	0.5	3	2	4	0.1	0.1	0.1	1	1	1
Mexico	Tenango De Doria	0.2	0.2	0.3	1	1	1	0.1	0.0	0.1	0	0	0
Mexico	Tenango Del Aire	0.3	0.3	0.4	0	0	0	0.1	0.1	0.1	0	0	0
Mexico	Tenango Del Valle	0.3	0.3	0.4	3	2	3	0.1	0.1	0.1	1	0	1
Mexico	Tenejapa	1.1	0.8	1.4	7	5	10	0.3	0.2	0.4	2	2	3
Mexico	Tenochtitlan	0.4	0.3	0.5	0	0	0	0.1	0.1	0.1	0	0	0
Mexico	Tenosique	0.3	0.3	0.4	3	2	4	0.1	0.1	0.2	1	1	1
Mexico	Teocaltiche	0.2	0.2	0.3	1	1	1	0.0	0.0	0.1	0	0	0
Mexico	Teocelo	0.4	0.3	0.5	1	1	1	0.1	0.1	0.1	0	0	0
Mexico	Teococuilco De Marcos Perez	0.7	0.5	0.9	0	0	0	0.2	0.2	0.3	0	0	0
Mexico	Teocuitatlan De Corona	0.3	0.2	0.4	0	0	0	0.1	0.0	0.1	0	0	0
Mexico	Teolocholco	0.3	0.2	0.4	1	0	1	0.1	0.1	0.1	0	0	0
Mexico	Teoloyucan	0.3	0.2	0.4	3	2	4	0.1	0.1	0.1	1	0	1
Mexico	Teopantlan	0.6	0.4	0.7	0	0	0	0.2	0.1	0.2	0	0	0
Mexico	Teopisca	1.0	0.8	1.4	6	4	7	0.3	0.2	0.4	2	2	3
Mexico	Teotihuacan	0.3	0.2	0.4	2	1	2	0.1	0.1	0.1	1	0	1
Mexico	Teotitlan De Flores Magon	0.7	0.5	0.9	1	1	1	0.2	0.2	0.3	0	0	0
Mexico	Teotitlan Del Valle	0.7	0.5	0.9	0	0	1	0.2	0.2	0.3	0	0	0
Mexico	Teotlalco	0.6	0.4	0.7	1	0	1	0.2	0.1	0.2	0	0	0
Mexico	Teotongo	0.6	0.5	0.8	0	0	0	0.2	0.1	0.3	0	0	0
Mexico	Tepache	0.3	0.2	0.4	0	0	0	0.1	0.1	0.1	0	0	0
Mexico	Tepakan	0.3	0.2	0.4	0	0	0	0.1	0.1	0.1	0	0	0
Mexico	Tepalcatepec	0.4	0.3	0.6	1	1	2	0.1	0.1	0.2	0	0	0
Mexico	Tepalcingo	0.3	0.2	0.4	1	1	1	0.1	0.1	0.1	0	0	0
Mexico	Tepanco De Lopez	0.5	0.4	0.7	2	1	2	0.2	0.1	0.2	0	0	1
Mexico	Tepango De Rodriguez	0.5	0.4	0.7	0	0	0	0.1	0.1	0.2	0	0	0
Mexico	Tepatitlan De Morelos	0.2	0.2	0.3	4	3	5	0.1	0.0	0.1	1	1	1
Mexico	Tepatlaxco	0.4	0.3	0.5	0	0	1	0.1	0.1	0.1	0	0	0
Mexico	Tepatlaxco De Hidalgo	0.5	0.4	0.6	1	1	1	0.1	0.1	0.2	0	0	0
Mexico	Tepeaca	0.5	0.4	0.7	5	4	7	0.1	0.1	0.2	2	1	2
Mexico	Tepeapulco	0.2	0.2	0.3	1	1	2	0.1	0.0	0.1	0	0	0
Mexico	Tepechitlan	0.3	0.3	0.4	0	0	0	0.1	0.1	0.1	0	0	0
Mexico	Tepecoacuilco De Trujano	1.0	0.8	1.3	4	3	5	0.2	0.2	0.3	1	1	1
Mexico	Tepehuacan De Guerrero	0.3	0.2	0.3	1	1	2	0.1	0.0	0.1	0	0	0
Mexico	Tepehuanes	0.2	0.2	0.3	0	0	0	0.1	0.0	0.1	0	0	0
Mexico	Tepeji Del Rio De Ocampo	0.2	0.2	0.3	2	2	3	0.1	0.0	0.1	1	0	1
Mexico	Tepelmeme Villa De Morelos	0.6	0.5	0.8	0	0	0	0.2	0.1	0.3	0	0	0
Mexico	Tepemaxalco	0.5	0.4	0.7	0	0	0	0.2	0.1	0.2	0	0	0
Mexico	Tepeojuma	0.6	0.4	0.7	1	1	1	0.2	0.1	0.2	0	0	0
Mexico	Tepetitla De Lardizabal	0.3	0.2	0.4	1	1	1	0.1	0.1	0.1	0	0	0
Mexico	Tepetitlan	0.2	0.2	0.3	0	0	0	0.1	0.0	0.1	0	0	0
Mexico	Tepetlan	0.3	0.2	0.4	0	0	0	0.1	0.0	0.1	0	0	0
Mexico	Tepetlaoxtoc	0.3	0.2	0.4	1	1	1	0.1	0.1	0.1	0	0	0
Mexico	Tepetlixpa	0.3	0.2	0.4	1	0	1	0.1	0.1	0.1	0	0	0
Mexico	Tepetongo	0.3	0.2	0.4	0	0	0	0.1	0.0	0.1	0	0	0

Table 3: Diarrhoea Mortality by Unit *(continued)*

Country	Unit	2000 mortality						2017 mortality					
		mean rate (per 10,000)	lower rate (per 10,000)	upper rate (per 10,000)	mean count	lower count	upper count	mean rate (per 10,000)	lower rate (per 10,000)	upper rate (per 10,000)	mean count	lower count	upper count
Mexico	Tepetzintla	0.5	0.4	0.7	1	1	1	0.1	0.1	0.2	0	0	0
Mexico	Tepetzintla	0.4	0.3	0.5	1	1	1	0.1	0.1	0.1	0	0	0
Mexico	Tepexco	0.5	0.4	0.7	1	0	1	0.1	0.1	0.2	0	0	0
Mexico	Tepexi De Rodriguez	0.6	0.4	0.7	1	1	2	0.2	0.1	0.2	0	0	1
Mexico	Tepeyahualco	0.5	0.4	0.7	2	2	3	0.1	0.1	0.2	0	0	0
Mexico	Tepeyahualco De Cuauhtemoc	0.5	0.4	0.7	0	0	0	0.1	0.1	0.2	0	0	0
Mexico	Tepeyanco	0.3	0.2	0.4	1	1	1	0.1	0.1	0.1	0	0	0
Mexico	Tepezala	0.2	0.2	0.3	0	0	1	0.1	0.1	0.1	0	0	0
Mexico	Tepic	0.3	0.2	0.4	11	8	14	0.1	0.0	0.1	3	2	4
Mexico	Tepotzotlan	0.3	0.2	0.4	3	2	4	0.1	0.1	0.1	1	1	2
Mexico	Tepoztlan	0.3	0.2	0.4	2	2	3	0.1	0.1	0.1	1	1	1
Mexico	Tequila	0.4	0.3	0.5	1	1	1	0.1	0.1	0.1	0	0	0
Mexico	Tequila	0.3	0.2	0.4	1	1	2	0.1	0.0	0.1	0	0	0
Mexico	Tequisquiapan	0.2	0.2	0.3	2	1	2	0.1	0.1	0.1	1	0	1
Mexico	Tequixquiac	0.3	0.2	0.4	1	1	1	0.1	0.1	0.1	0	0	1
Mexico	Terrenate	0.3	0.2	0.4	1	0	1	0.1	0.1	0.1	0	0	0
Mexico	Tetecala	0.3	0.2	0.4	0	0	0	0.1	0.1	0.1	0	0	0
Mexico	Tetela De Ocampo	0.5	0.4	0.7	2	1	2	0.1	0.1	0.2	0	0	1
Mexico	Tetela Del Volcan	0.3	0.2	0.4	1	1	1	0.1	0.1	0.1	0	0	0
Mexico	Teteles De Avila Castillo	0.5	0.4	0.7	0	0	1	0.1	0.1	0.2	0	0	0
Mexico	Tetepango	0.2	0.2	0.3	0	0	0	0.1	0.0	0.1	0	0	0
Mexico	Tetipac	0.9	0.7	1.2	2	2	3	0.2	0.1	0.3	0	0	0
Mexico	Tetiz	0.3	0.2	0.4	0	0	0	0.1	0.1	0.1	0	0	0
Mexico	Tetla De La Solidaridad	0.3	0.2	0.4	1	1	1	0.1	0.1	0.1	0	0	1
Mexico	Tetlatlahuca	0.3	0.2	0.4	1	0	1	0.1	0.1	0.1	0	0	0
Mexico	Teuchitlan	0.3	0.2	0.4	0	0	0	0.1	0.0	0.1	0	0	0
Mexico	Teul De Gonzalez Ortega	0.3	0.2	0.4	0	0	0	0.1	0.1	0.1	0	0	0
Mexico	Texcaltitlan	0.3	0.3	0.4	1	1	1	0.1	0.1	0.1	0	0	0
Mexico	Texcalyacac	0.4	0.3	0.4	0	0	0	0.1	0.1	0.1	0	0	0
Mexico	Texcatepec	0.4	0.3	0.5	1	0	1	0.1	0.1	0.1	0	0	0
Mexico	Texcoco	0.3	0.3	0.4	14	10	17	0.1	0.1	0.1	4	3	5
Mexico	Texhuacan	0.4	0.3	0.5	0	0	1	0.1	0.1	0.1	0	0	0
Mexico	Texistepec	0.4	0.3	0.6	1	1	2	0.1	0.1	0.1	0	0	0
Mexico	Teya	0.3	0.2	0.4	0	0	0	0.1	0.1	0.1	0	0	0
Mexico	Teziutlan	0.5	0.4	0.7	5	4	7	0.1	0.1	0.2	1	1	2
Mexico	Tezoatlan De Segura Y Luna	0.6	0.5	0.8	1	1	1	0.2	0.1	0.3	0	0	0
Mexico	Tezonapa	0.4	0.3	0.6	3	2	4	0.1	0.1	0.1	1	0	1
Mexico	Tezontepec De Aldama	0.2	0.2	0.3	1	1	1	0.1	0.0	0.1	0	0	0
Mexico	Tezoyuca	0.3	0.2	0.4	1	1	1	0.1	0.1	0.1	1	0	1
Mexico	Tianguismanalco	0.5	0.4	0.7	1	1	2	0.2	0.1	0.2	0	0	1
Mexico	Tianguistenco	0.3	0.3	0.4	2	2	3	0.1	0.1	0.1	1	1	1
Mexico	Tianguistengo	0.3	0.2	0.3	1	0	1	0.1	0.0	0.1	0	0	0
Mexico	Ticul	0.3	0.2	0.5	1	1	2	0.1	0.1	0.2	0	0	1
Mexico	Tierra Blanca	0.4	0.3	0.5	4	3	5	0.1	0.1	0.1	1	1	1
Mexico	Tierra Blanca	0.4	0.3	0.5	1	1	1	0.1	0.0	0.1	0	0	0
Mexico	Tierra Nueva	0.4	0.3	0.5	1	0	1	0.1	0.1	0.2	0	0	0
Mexico	Tihuatlan	0.3	0.2	0.4	4	3	5	0.1	0.1	0.1	1	1	1
Mexico	Tijuana	0.2	0.1	0.2	24	16	35	0.0	0.0	0.1	6	4	9
Mexico	Tila	1.1	0.8	1.5	11	8	15	0.3	0.2	0.4	3	3	4
Mexico	Tilapa	0.6	0.4	0.7	1	1	1	0.2	0.1	0.2	0	0	0
Mexico	Timilpan	0.3	0.3	0.4	1	0	1	0.1	0.1	0.1	0	0	0
Mexico	Timucuy	0.3	0.2	0.4	0	0	0	0.1	0.1	0.1	0	0	0

Table 3: Diarrhoea Mortality by Unit *(continued)*

Country	Unit	2000 mortality mean rate (per 10,000)	lower rate (per 10,000)	upper rate (per 10,000)	mean count	lower count	upper count	2017 mortality mean rate (per 10,000)	lower rate (per 10,000)	upper rate (per 10,000)	mean count	lower count	upper count
Mexico	Tingambato	0.4	0.3	0.6	1	1	1	0.1	0.1	0.2	0	0	0
Mexico	Tinguindin	0.4	0.3	0.5	0	0	1	0.1	0.1	0.2	0	0	0
Mexico	Tinum	0.3	0.2	0.4	0	0	0	0.1	0.1	0.1	0	0	0
Mexico	Tiquicheo De Nicolas Romero	0.5	0.4	0.6	1	1	1	0.1	0.1	0.2	0	0	0
Mexico	Tixcacalcupul	0.3	0.2	0.4	0	0	0	0.1	0.1	0.1	0	0	0
Mexico	Tixkokob	0.3	0.2	0.4	0	0	1	0.1	0.1	0.1	0	0	0
Mexico	Tixmehuac	0.4	0.3	0.5	0	0	0	0.1	0.1	0.2	0	0	0
Mexico	Tixpehual	0.3	0.2	0.4	0	0	0	0.1	0.1	0.1	0	0	0
Mexico	Tixtla De Guerrero	0.9	0.7	1.2	5	4	7	0.2	0.1	0.3	1	1	1
Mexico	Tizapan El Alto	0.3	0.2	0.3	0	0	1	0.1	0.0	0.1	0	0	0
Mexico	Tizayuca	0.2	0.2	0.3	1	1	1	0.1	0.0	0.1	1	1	1
Mexico	Tizimin	0.3	0.2	0.4	3	2	4	0.1	0.1	0.2	1	1	1
Mexico	Tlachichilco	0.4	0.3	0.5	1	1	1	0.1	0.1	0.1	0	0	0
Mexico	Tlachichuca	0.5	0.3	0.6	2	1	2	0.1	0.1	0.2	0	0	1
Mexico	Tlacoachistlahuaca	1.1	0.8	1.4	4	3	5	0.2	0.1	0.3	1	1	1
Mexico	Tlacoapa	1.0	0.8	1.3	2	2	3	0.2	0.1	0.3	0	0	0
Mexico	Tlacojalpan	0.4	0.3	0.6	0	0	0	0.1	0.1	0.1	0	0	0
Mexico	Tlacolula De Matamoros	0.7	0.5	0.9	1	1	2	0.2	0.2	0.3	0	0	1
Mexico	Tlacolulan	0.3	0.2	0.4	1	0	1	0.1	0.0	0.1	0	0	0
Mexico	Tlacotalpan	0.5	0.3	0.6	1	1	1	0.1	0.1	0.1	0	0	0
Mexico	Tlacotepec De Benito Juarez	0.5	0.4	0.7	4	3	5	0.2	0.1	0.2	1	1	2
Mexico	Tlacotepec De Mejia	0.4	0.3	0.5	0	0	0	0.1	0.1	0.1	0	0	0
Mexico	Tlacotepec Plumas	0.6	0.5	0.8	0	0	0	0.2	0.1	0.3	0	0	0
Mexico	Tlacuilotepec	0.6	0.4	0.7	2	1	2	0.2	0.1	0.2	0	0	0
Mexico	Tlahuac	0.2	0.1	0.2	8	6	10	0.1	0.0	0.1	2	2	3
Mexico	Tlahualilo	0.2	0.1	0.3	0	0	1	0.1	0.0	0.1	0	0	0
Mexico	Tlahuapan	0.5	0.4	0.6	2	2	3	0.1	0.1	0.2	1	1	1
Mexico	Tlahuelilpan	0.2	0.2	0.3	0	0	0	0.1	0.0	0.1	0	0	0
Mexico	Tlahuiltepa	0.3	0.2	0.3	0	0	1	0.1	0.0	0.1	0	0	0
Mexico	Tlajomulco De Zuniga	0.3	0.2	0.3	7	5	9	0.1	0.0	0.1	6	4	8
Mexico	Tlalchapa	1.1	0.8	1.3	2	1	2	0.2	0.2	0.3	0	0	0
Mexico	Tlalixcoyan	0.4	0.3	0.5	2	1	2	0.1	0.1	0.1	0	0	0
Mexico	Tlalixtac De Cabrera	0.6	0.5	0.8	1	1	2	0.2	0.1	0.3	0	0	1
Mexico	Tlalixtaquilla De Maldonado	1.0	0.8	1.3	1	1	1	0.2	0.1	0.3	0	0	0
Mexico	Tlalmanalco	0.3	0.3	0.4	2	1	2	0.1	0.1	0.1	0	0	0
Mexico	Tlalnelhuayocan	0.3	0.2	0.4	1	1	1	0.1	0.0	0.1	0	0	0
Mexico	Tlalnepantla	0.3	0.2	0.3	0	0	0	0.1	0.1	0.1	0	0	0
Mexico	Tlalnepantla De Baz	0.4	0.3	0.4	28	22	35	0.1	0.1	0.1	5	3	6
Mexico	Tlalpan	0.2	0.1	0.2	10	8	12	0.1	0.0	0.1	3	2	5
Mexico	Tlalpujahua	0.4	0.3	0.5	2	2	2	0.1	0.1	0.2	1	0	1
Mexico	Tlaltenango	0.5	0.4	0.6	0	0	1	0.1	0.1	0.2	0	0	0
Mexico	Tlaltenango De Sanchez Roman	0.3	0.2	0.4	1	1	1	0.1	0.1	0.1	0	0	0
Mexico	Tlaltetela	0.4	0.3	0.5	1	1	1	0.1	0.1	0.1	0	0	0
Mexico	Tlaltizapan	0.3	0.2	0.4	1	1	2	0.1	0.1	0.1	0	0	1
Mexico	Tlanalapa	0.2	0.2	0.3	0	0	0	0.1	0.0	0.1	0	0	0
Mexico	Tlanchinol	0.3	0.2	0.3	2	1	2	0.1	0.0	0.1	0	0	0
Mexico	Tlanepantla	0.5	0.4	0.6	0	0	0	0.1	0.1	0.2	0	0	0
Mexico	Tlaola	0.6	0.4	0.7	2	2	3	0.2	0.1	0.2	0	0	1
Mexico	Tlapa De Comonfort	1.0	0.8	1.3	10	8	12	0.2	0.1	0.3	3	2	4

Table 3: Diarrhoea Mortality by Unit *(continued)*

Country	Unit	2000 mortality mean rate (per 10,000)	lower rate (per 10,000)	upper rate (per 10,000)	mean count	lower count	upper count	2017 mortality mean rate (per 10,000)	lower rate (per 10,000)	upper rate (per 10,000)	mean count	lower count	upper count
Mexico	Tlapacoya	0.6	0.4	0.7	1	0	1	0.2	0.1	0.2	0	0	0
Mexico	Tlapacoyan	0.3	0.3	0.4	2	1	2	0.1	0.1	0.1	0	0	1
Mexico	Tlapanala	0.6	0.5	0.8	1	1	1	0.2	0.1	0.2	0	0	0
Mexico	Tlapehuala	1.1	0.8	1.3	3	2	4	0.2	0.2	0.3	1	0	1
Mexico	Tlaquepaque	0.2	0.2	0.3	22	17	29	0.1	0.0	0.1	4	2	5
Mexico	Tlaquilpa	0.4	0.3	0.5	0	0	1	0.1	0.1	0.1	0	0	0
Mexico	Tlaquiltenango	0.3	0.2	0.4	1	1	1	0.1	0.1	0.1	0	0	0
Mexico	Tlatlauquitepec	0.5	0.4	0.7	3	3	5	0.1	0.1	0.2	1	1	1
Mexico	Tlatlaya	0.4	0.3	0.5	1	1	2	0.1	0.1	0.1	0	0	0
Mexico	Tlaxcala	0.3	0.2	0.4	3	2	4	0.1	0.1	0.1	1	1	1
Mexico	Tlaxco	0.6	0.4	0.7	1	0	1	0.1	0.1	0.2	0	0	0
Mexico	Tlaxco	0.3	0.2	0.4	2	1	2	0.1	0.1	0.1	0	0	1
Mexico	Tlaxcoapan	0.2	0.2	0.3	1	1	1	0.1	0.0	0.1	0	0	0
Mexico	Tlayacapan	0.3	0.2	0.3	0	0	1	0.1	0.1	0.1	0	0	0
Mexico	Tlazazalca	0.4	0.3	0.5	0	0	0	0.1	0.1	0.2	0	0	0
Mexico	Tlilapan	0.4	0.3	0.5	0	0	1	0.1	0.0	0.1	0	0	0
Mexico	Tocatlan	0.3	0.2	0.4	0	0	0	0.1	0.1	0.1	0	0	0
Mexico	Tochimilco	0.5	0.4	0.7	1	1	1	0.1	0.1	0.2	0	0	1
Mexico	Tochtepec	0.5	0.4	0.6	2	1	2	0.1	0.1	0.2	0	0	1
Mexico	Tocumbo	0.4	0.3	0.5	1	1	1	0.1	0.1	0.2	0	0	0
Mexico	Tolcayuca	0.2	0.2	0.3	0	0	1	0.1	0.0	0.1	0	0	0
Mexico	Toliman	0.3	0.2	0.3	1	1	1	0.1	0.1	0.1	0	0	0
Mexico	Toliman	0.3	0.2	0.4	0	0	0	0.1	0.0	0.1	0	0	0
Mexico	Toluca	0.3	0.3	0.4	27	21	33	0.1	0.1	0.1	7	5	10
Mexico	Tomatlan	0.3	0.2	0.4	1	1	1	0.1	0.0	0.1	0	0	0
Mexico	Tomatlan	0.4	0.3	0.5	0	0	0	0.1	0.1	0.1	0	0	0
Mexico	Tonala	0.2	0.2	0.3	14	11	18	0.1	0.0	0.1	3	2	4
Mexico	Tonala	1.2	0.9	1.6	11	8	14	0.3	0.3	0.5	4	3	5
Mexico	Tonanitla	0.3	0.2	0.4	1	1	1	0.1	0.1	0.1	0	0	0
Mexico	Tonatico	0.4	0.3	0.5	1	0	1	0.1	0.1	0.1	0	0	0
Mexico	Tonaya	0.3	0.2	0.4	0	0	0	0.1	0.0	0.1	0	0	0
Mexico	Tonayan	0.3	0.2	0.4	0	0	0	0.1	0.0	0.1	0	0	0
Mexico	Tonila	0.3	0.2	0.4	0	0	0	0.1	0.0	0.1	0	0	0
Mexico	Topia	0.3	0.2	0.4	0	0	0	0.1	0.0	0.1	0	0	0
Mexico	Torreon	0.2	0.1	0.2	11	8	14	0.1	0.0	0.1	4	3	6
Mexico	Totatiche	0.3	0.2	0.3	0	0	0	0.1	0.0	0.1	0	0	0
Mexico	Totolac	0.3	0.2	0.4	0	0	1	0.1	0.1	0.1	0	0	0
Mexico	Totolapa	1.2	0.8	1.5	1	1	1	0.3	0.2	0.4	0	0	0
Mexico	Totolapan	0.3	0.2	0.3	0	0	0	0.1	0.1	0.1	0	0	0
Mexico	Totoltepec De Guerrero	0.6	0.5	0.8	0	0	0	0.2	0.1	0.2	0	0	0
Mexico	Totontepec Villa De Morelos	0.7	0.5	0.9	0	0	1	0.2	0.2	0.3	0	0	0
Mexico	Tototlan	0.3	0.2	0.3	1	0	1	0.1	0.0	0.1	0	0	0
Mexico	Totutla	0.4	0.3	0.5	1	0	1	0.1	0.1	0.1	0	0	0
Mexico	Trancoso	0.3	0.2	0.4	1	0	1	0.1	0.0	0.1	0	0	0
Mexico	Tres Valles	0.4	0.3	0.6	2	2	3	0.1	0.1	0.1	0	0	1
Mexico	Trincheras	0.3	0.2	0.3	0	0	0	0.1	0.0	0.1	0	0	0
Mexico	Trinidad Garcia De La Cadena	0.3	0.2	0.4	0	0	0	0.1	0.1	0.1	0	0	0
Mexico	Trinidad Zaachila	0.7	0.5	0.9	0	0	0	0.2	0.2	0.3	0	0	0
Mexico	Tubutama	0.3	0.2	0.4	0	0	0	0.1	0.0	0.1	0	0	0
Mexico	Tula	0.1	0.1	0.2	0	0	1	0.0	0.0	0.1	0	0	0
Mexico	Tula De Allende	0.2	0.2	0.3	2	2	3	0.1	0.0	0.1	1	0	1
Mexico	Tulancingo De Bravo	0.2	0.1	0.3	3	2	4	0.1	0.0	0.1	1	1	1
Mexico	Tulcingo	0.6	0.5	0.8	1	1	1	0.2	0.1	0.2	0	0	0
Mexico	Tultepec	0.3	0.2	0.4	4	3	5	0.1	0.1	0.1	1	1	2
Mexico	Tultitlan	0.3	0.3	0.4	14	11	17	0.1	0.1	0.1	3	2	5
Mexico	Tumbala	1.1	0.8	1.5	4	3	5	0.3	0.2	0.4	2	1	2

Table 3: Diarrhoea Mortality by Unit *(continued)*

Country	Unit	2000 mortality mean rate (per 10,000)	lower rate (per 10,000)	upper rate (per 10,000)	mean count	lower count	upper count	2017 mortality mean rate (per 10,000)	lower rate (per 10,000)	upper rate (per 10,000)	mean count	lower count	upper count
Mexico	Tumbiscatio	0.5	0.4	0.6	1	1	1	0.1	0.1	0.2	0	0	0
Mexico	Tunkas	0.3	0.3	0.4	0	0	0	0.1	0.1	0.1	0	0	0
Mexico	Turicato	0.5	0.4	0.6	2	2	3	0.1	0.1	0.2	0	0	1
Mexico	Tuxcacuesco	0.3	0.2	0.4	0	0	0	0.1	0.0	0.1	0	0	0
Mexico	Tuxcueca	0.3	0.2	0.3	0	0	0	0.1	0.0	0.1	0	0	0
Mexico	Tuxpam	0.3	0.2	0.4	5	4	7	0.1	0.1	0.1	1	1	1
Mexico	Tuxpan	0.3	0.2	0.3	1	1	2	0.1	0.0	0.1	0	0	0
Mexico	Tuxpan	0.3	0.2	0.3	1	1	1	0.1	0.0	0.1	0	0	0
Mexico	Tuxpan	0.4	0.3	0.5	2	1	2	0.1	0.1	0.2	0	0	0
Mexico	Tuxtilla	0.4	0.3	0.6	0	0	0	0.1	0.1	0.1	0	0	0
Mexico	Tuxtla Chico	1.3	0.9	1.7	9	6	11	0.4	0.3	0.5	3	2	4
Mexico	Tuxtla Gutierrez	1.0	0.7	1.3	56	41	74	0.3	0.2	0.4	20	15	27
Mexico	Tuzamapan De Galeana	0.6	0.4	0.8	1	0	1	0.2	0.1	0.2	0	0	0
Mexico	Tuzantan	1.2	0.9	1.6	3	2	4	0.3	0.2	0.4	1	1	2
Mexico	Tuzantla	0.5	0.4	0.6	1	1	1	0.1	0.1	0.2	0	0	0
Mexico	Tzicatlacoyan	0.5	0.4	0.7	0	0	1	0.2	0.1	0.2	0	0	0
Mexico	Tzimol	1.1	0.8	1.5	2	1	2	0.3	0.2	0.4	1	0	1
Mexico	Tzintzuntzan	0.4	0.3	0.5	1	1	1	0.1	0.1	0.2	0	0	0
Mexico	Tzitzio	0.5	0.4	0.6	1	1	1	0.1	0.1	0.2	0	0	0
Mexico	Tzompantepec	0.3	0.2	0.4	1	1	1	0.1	0.1	0.1	0	0	0
Mexico	Tzucacab	0.4	0.3	0.5	1	1	1	0.1	0.1	0.2	0	0	0
Mexico	Uayma	0.3	0.2	0.4	0	0	0	0.1	0.1	0.1	0	0	0
Mexico	Ucu	0.3	0.2	0.4	0	0	0	0.1	0.1	0.1	0	0	0
Mexico	Uman	0.3	0.2	0.4	2	2	3	0.1	0.1	0.1	0	0	1
Mexico	Union De San Antonio	0.2	0.2	0.3	1	0	1	0.0	0.0	0.1	0	0	0
Mexico	Union De Tula	0.3	0.2	0.4	0	0	0	0.1	0.0	0.1	0	0	0
Mexico	Union Hidalgo	0.8	0.6	1.0	1	1	1	0.3	0.2	0.3	0	0	0
Mexico	Union Juarez	1.4	1.1	1.8	2	2	3	0.3	0.3	0.4	1	0	1
Mexico	Ures	0.3	0.2	0.4	0	0	0	0.1	0.0	0.1	0	0	0
Mexico	Uriangato	0.4	0.3	0.5	3	2	3	0.1	0.1	0.1	0	0	1
Mexico	Urique	0.4	0.3	0.5	1	1	1	0.1	0.1	0.1	0	0	0
Mexico	Ursulo Galvan	0.4	0.3	0.5	1	1	1	0.1	0.1	0.1	0	0	0
Mexico	Uruachi	0.4	0.3	0.5	0	0	0	0.1	0.1	0.1	0	0	0
Mexico	Uruapan	0.4	0.3	0.5	15	11	19	0.1	0.1	0.2	4	3	6
Mexico	Uxpanapa	0.5	0.3	0.6	2	1	2	0.1	0.1	0.1	0	0	0
Mexico	Valerio Trujano	0.7	0.5	0.9	0	0	0	0.2	0.2	0.3	0	0	0
Mexico	Valladolid	0.3	0.2	0.4	2	2	3	0.1	0.1	0.1	1	1	1
Mexico	Valle De Bravo	0.4	0.3	0.5	3	2	3	0.1	0.1	0.1	1	0	1
Mexico	Valle De Chalco Solidaridad	0.4	0.3	0.4	14	10	17	0.1	0.1	0.1	3	2	5
Mexico	Valle De Guadalupe	0.2	0.2	0.3	0	0	0	0.1	0.0	0.1	0	0	0
Mexico	Valle De Juarez	0.3	0.2	0.3	0	0	0	0.1	0.0	0.1	0	0	0
Mexico	Valle De Santiago	0.4	0.3	0.5	6	5	8	0.1	0.0	0.1	1	1	1
Mexico	Valle De Zaragoza	0.3	0.2	0.4	0	0	0	0.1	0.1	0.1	0	0	0
Mexico	Valle Hermoso	0.2	0.1	0.2	1	1	2	0.1	0.0	0.1	0	0	0
Mexico	Vallecillo	0.1	0.1	0.2	0	0	0	0.0	0.0	0.1	0	0	0
Mexico	Valparaiso	0.3	0.2	0.4	2	1	2	0.1	0.0	0.1	0	0	0
Mexico	Vanegas	0.4	0.3	0.5	0	0	0	0.1	0.1	0.2	0	0	0
Mexico	Vega De Alatorre	0.4	0.3	0.5	1	1	1	0.1	0.1	0.1	0	0	0
Mexico	Venado	0.4	0.3	0.5	1	1	1	0.1	0.1	0.2	0	0	0
Mexico	Venustiano Carranza	0.4	0.3	0.5	1	1	1	0.1	0.1	0.2	0	0	0
Mexico	Venustiano Carranza	1.2	0.9	1.5	9	7	12	0.3	0.2	0.4	3	2	3
Mexico	Venustiano Carranza	0.1	0.1	0.2	7	5	8	0.1	0.0	0.1	2	2	3

Table 3: Diarrhoea Mortality by Unit *(continued)*

Country	Unit	2000 mortality mean rate (per 10,000)	lower rate (per 10,000)	upper rate (per 10,000)	mean count	lower count	upper count	2017 mortality mean rate (per 10,000)	lower rate (per 10,000)	upper rate (per 10,000)	mean count	lower count	upper count
Mexico	Venustiano Carranza	0.5	0.4	0.7	2	1	2	0.1	0.1	0.2	1	0	1
Mexico	Veracruz	0.4	0.3	0.5	19	14	26	0.1	0.1	0.1	4	3	5
Mexico	Vetagrande	0.3	0.2	0.4	1	0	1	0.1	0.0	0.1	0	0	0
Mexico	Vicente Guerrero	0.5	0.4	0.7	2	1	2	0.1	0.1	0.2	1	0	1
Mexico	Vicente Guerrero	0.2	0.2	0.3	1	0	1	0.1	0.0	0.1	0	0	0
Mexico	Victoria	0.1	0.1	0.2	4	3	5	0.0	0.0	0.1	1	1	2
Mexico	Victoria	0.4	0.3	0.5	1	1	2	0.1	0.0	0.1	0	0	0
Mexico	Viesca	0.2	0.2	0.3	1	0	1	0.1	0.0	0.1	0	0	0
Mexico	Villa Aldama	0.3	0.2	0.4	0	0	1	0.1	0.0	0.1	0	0	0
Mexico	Villa Comaltitlan	1.2	0.9	1.6	4	3	6	0.3	0.2	0.4	1	1	2
Mexico	Villa Corona	0.3	0.2	0.4	1	0	1	0.1	0.0	0.1	0	0	0
Mexico	Villa Corzo	1.1	0.8	1.5	10	7	13	0.3	0.2	0.4	3	2	4
Mexico	Villa De Allende	0.3	0.3	0.4	2	2	2	0.1	0.1	0.1	1	0	1
Mexico	Villa De Alvarez	0.2	0.2	0.3	2	1	2	0.1	0.1	0.1	1	1	1
Mexico	Villa De Arista	0.4	0.3	0.5	1	0	1	0.1	0.1	0.1	0	0	0
Mexico	Villa De Arriaga	0.4	0.3	0.5	1	1	1	0.1	0.1	0.1	0	0	0
Mexico	Villa De Chilapa De Diaz	0.6	0.5	0.8	0	0	0	0.2	0.1	0.3	0	0	0
Mexico	Villa De Cos	0.3	0.2	0.4	1	1	2	0.1	0.0	0.1	0	0	0
Mexico	Villa De Etla	0.6	0.5	0.8	0	0	0	0.2	0.2	0.3	0	0	0
Mexico	Villa De Guadalupe	0.3	0.3	0.4	0	0	1	0.1	0.1	0.2	0	0	0
Mexico	Villa De La Paz	0.4	0.3	0.5	0	0	0	0.1	0.1	0.1	0	0	0
Mexico	Villa De Ramos	0.4	0.3	0.5	2	1	2	0.1	0.1	0.2	1	0	1
Mexico	Villa De Reyes	0.4	0.3	0.5	2	2	3	0.1	0.1	0.1	1	0	1
Mexico	Villa De Tamazulapam Del Progreso	0.6	0.5	0.8	0	0	1	0.2	0.1	0.3	0	0	0
Mexico	Villa De Tezontepec	0.2	0.2	0.3	0	0	0	0.1	0.0	0.1	0	0	0
Mexico	Villa De Tututepec De Melchor Ocampo	0.8	0.6	1.1	5	4	7	0.3	0.2	0.4	1	1	2
Mexico	Villa De Zaachila	0.7	0.5	0.9	2	1	2	0.2	0.2	0.3	1	0	1
Mexico	Villa Del Carbon	0.3	0.3	0.4	1	1	2	0.1	0.1	0.1	0	0	1
Mexico	Villa Diaz Ordaz	0.7	0.5	0.9	0	0	0	0.2	0.2	0.3	0	0	0
Mexico	Villa Garcia	0.3	0.2	0.4	1	1	1	0.1	0.0	0.1	0	0	0
Mexico	Villa Gonzalez Ortega	0.3	0.2	0.4	1	0	1	0.1	0.0	0.1	0	0	0
Mexico	Villa Guerrero	0.3	0.2	0.4	0	0	0	0.1	0.0	0.1	0	0	0
Mexico	Villa Guerrero	0.4	0.3	0.5	3	2	3	0.1	0.1	0.1	1	1	1
Mexico	Villa Hidalgo	0.2	0.2	0.3	1	0	1	0.0	0.0	0.1	0	0	0
Mexico	Villa Hidalgo	0.4	0.3	0.5	1	1	1	0.1	0.1	0.2	0	0	0
Mexico	Villa Hidalgo	0.8	0.6	1.0	0	0	0	0.2	0.2	0.3	0	0	0
Mexico	Villa Hidalgo	0.3	0.2	0.4	1	0	1	0.1	0.0	0.1	0	0	0
Mexico	Villa Hidalgo	0.3	0.2	0.4	0	0	0	0.1	0.1	0.1	0	0	0
Mexico	Villa Juarez	0.4	0.3	0.6	1	0	1	0.1	0.1	0.2	0	0	0
Mexico	Villa Pesqueira	0.3	0.2	0.4	0	0	0	0.1	0.1	0.1	0	0	0
Mexico	Villa Purificacion	0.3	0.2	0.4	0	0	1	0.1	0.0	0.1	0	0	0
Mexico	Villa Sola De Vega	0.7	0.6	0.9	1	1	2	0.2	0.2	0.3	0	0	0
Mexico	Villa Talea De Castro	0.8	0.6	1.0	0	0	0	0.2	0.2	0.3	0	0	0
Mexico	Villa Tejupam De La Union	0.6	0.5	0.8	0	0	0	0.2	0.1	0.3	0	0	0
Mexico	Villa Union	0.2	0.2	0.3	0	0	0	0.1	0.0	0.1	0	0	0
Mexico	Villa Victoria	0.3	0.3	0.4	4	3	5	0.1	0.1	0.1	1	1	2
Mexico	Villaflores	1.0	0.8	1.4	13	9	17	0.3	0.2	0.4	3	2	4
Mexico	Villagran	0.1	0.1	0.2	0	0	0	0.0	0.0	0.1	0	0	0

Table 3: Diarrhoea Mortality by Unit *(continued)*

Country	Unit	2000 mortality mean rate (per 10,000)	lower rate (per 10,000)	upper rate (per 10,000)	mean count	lower count	upper count	2017 mortality mean rate (per 10,000)	lower rate (per 10,000)	upper rate (per 10,000)	mean count	lower count	upper count
Mexico	Villagran	0.4	0.3	0.5	2	2	3	0.1	0.0	0.1	0	0	1
Mexico	Villaldama	0.1	0.1	0.2	0	0	0	0.0	0.0	0.0	0	0	0
Mexico	Villamar	0.4	0.3	0.5	1	1	1	0.1	0.1	0.2	0	0	0
Mexico	Villanueva	0.3	0.2	0.4	1	1	2	0.1	0.0	0.1	0	0	0
Mexico	Vista Hermosa	0.4	0.3	0.5	1	1	1	0.1	0.1	0.2	0	0	0
Mexico	Xalapa	0.3	0.2	0.4	12	9	16	0.1	0.0	0.1	2	2	3
Mexico	Xalatlaco	0.3	0.2	0.4	1	1	1	0.1	0.1	0.1	0	0	0
Mexico	Xalisco	0.3	0.2	0.3	2	1	2	0.1	0.0	0.1	0	0	1
Mexico	Xaloztoc	0.3	0.2	0.4	0	0	1	0.1	0.1	0.1	0	0	0
Mexico	Xalpatlahuac	1.0	0.8	1.3	3	2	3	0.2	0.1	0.3	0	0	1
Mexico	Xaltocan	0.3	0.2	0.4	0	0	1	0.1	0.1	0.1	0	0	0
Mexico	Xayacatlan De Bravo	0.6	0.4	0.8	0	0	0	0.2	0.1	0.2	0	0	0
Mexico	Xichu	0.4	0.3	0.5	1	1	1	0.1	0.0	0.1	0	0	0
Mexico	Xico	0.4	0.3	0.5	2	2	3	0.1	0.0	0.1	0	0	0
Mexico	Xicohtzinco	0.3	0.2	0.4	0	0	1	0.1	0.1	0.1	0	0	0
Mexico	Xicotencatl	0.1	0.1	0.2	0	0	0	0.0	0.0	0.1	0	0	0
Mexico	Xicotepec	0.6	0.4	0.7	6	4	8	0.2	0.1	0.2	2	1	2
Mexico	Xicotlan	0.6	0.5	0.7	0	0	0	0.2	0.1	0.2	0	0	0
Mexico	Xilitla	0.5	0.4	0.6	4	3	5	0.1	0.1	0.2	1	1	1
Mexico	Xiutetelco	0.5	0.4	0.7	3	2	4	0.1	0.1	0.2	1	0	1
Mexico	Xocchel	0.3	0.2	0.4	0	0	0	0.1	0.1	0.1	0	0	0
Mexico	Xochiapulco	0.5	0.4	0.7	1	0	1	0.1	0.1	0.2	0	0	0
Mexico	Xochiatipan	0.3	0.2	0.3	1	1	1	0.1	0.0	0.1	0	0	0
Mexico	Xochicoatlan	0.2	0.2	0.3	0	0	0	0.1	0.0	0.1	0	0	0
Mexico	Xochihuehuetlan	1.1	0.8	1.4	1	1	2	0.2	0.1	0.3	0	0	0
Mexico	Xochiltepec	0.6	0.4	0.8	0	0	0	0.2	0.1	0.2	0	0	0
Mexico	Xochimilco	0.2	0.1	0.2	9	7	12	0.1	0.0	0.1	3	2	4
Mexico	Xochistlahuaca	1.1	0.8	1.5	5	4	7	0.2	0.1	0.3	1	1	1
Mexico	Xochitepec	0.3	0.2	0.3	2	1	2	0.1	0.1	0.1	1	0	1
Mexico	Xochitlan De Vicente Suarez	0.5	0.4	0.7	1	1	1	0.2	0.1	0.2	0	0	0
Mexico	Xochitlan Todos Santos	0.6	0.4	0.7	1	0	1	0.2	0.1	0.2	0	0	0
Mexico	Xonacatlan	0.3	0.3	0.4	2	1	2	0.1	0.1	0.1	0	0	1
Mexico	Xoxocotla	0.3	0.3	0.4	0	0	0	0.1	0.1	0.1	0	0	0
Mexico	Yahualica	0.3	0.2	0.3	1	1	1	0.1	0.0	0.1	0	0	0
Mexico	Yahualica De Gonzalez Gallo	0.2	0.2	0.3	1	0	1	0.0	0.0	0.1	0	0	0
Mexico	Yajalon	1.1	0.8	1.5	5	4	6	0.3	0.2	0.4	2	1	2
Mexico	Yanga	0.4	0.3	0.5	1	1	1	0.1	0.1	0.1	0	0	0
Mexico	Yaonahuac	0.5	0.4	0.7	0	0	1	0.1	0.1	0.2	0	0	0
Mexico	Yauhquemecan	0.3	0.2	0.4	1	1	1	0.1	0.1	0.1	0	0	0
Mexico	Yautepec	0.3	0.2	0.3	3	2	3	0.1	0.1	0.1	1	1	1
Mexico	Yaxcaba	0.3	0.2	0.4	1	0	1	0.1	0.1	0.1	0	0	0
Mexico	Yaxe	0.7	0.5	0.9	0	0	0	0.2	0.2	0.3	0	0	0
Mexico	Yaxkukul	0.3	0.2	0.4	0	0	0	0.1	0.1	0.1	0	0	0
Mexico	Yecapixtla	0.3	0.2	0.3	2	1	2	0.1	0.1	0.1	0	0	1
Mexico	Yecora	0.3	0.2	0.4	0	0	0	0.1	0.0	0.1	0	0	0
Mexico	Yecuatla	0.4	0.3	0.5	1	1	1	0.1	0.1	0.1	0	0	0
Mexico	Yehualtepec	0.5	0.4	0.7	2	1	2	0.2	0.1	0.2	1	0	1
Mexico	Yobain	0.3	0.2	0.4	0	0	0	0.1	0.1	0.1	0	0	0
Mexico	Yogana	0.7	0.5	0.9	0	0	0	0.2	0.2	0.3	0	0	0
Mexico	Yurecuaro	0.4	0.3	0.5	1	1	2	0.1	0.1	0.2	0	0	0
Mexico	Yuriria	0.4	0.3	0.5	3	2	4	0.1	0.1	0.1	1	0	1
Mexico	Yutanduchi De Guerrero	0.7	0.5	0.9	0	0	0	0.2	0.2	0.3	0	0	0
Mexico	Zacapala	0.6	0.4	0.7	0	0	0	0.2	0.1	0.2	0	0	0
Mexico	Zacapoaxtla	0.5	0.4	0.7	3	2	4	0.1	0.1	0.2	1	1	1
Mexico	Zacapu	0.4	0.3	0.5	4	3	5	0.1	0.1	0.2	1	1	1

Table 3: Diarrhoea Mortality by Unit *(continued)*

Country	Unit	2000 mortality mean rate (per 10,000)	lower rate (per 10,000)	upper rate (per 10,000)	mean count	lower count	upper count	2017 mortality mean rate (per 10,000)	lower rate (per 10,000)	upper rate (per 10,000)	mean count	lower count	upper count
Mexico	Zacatecas	0.3	0.2	0.4	4	3	6	0.1	0.0	0.1	1	1	1
Mexico	Zacatelco	0.3	0.2	0.4	1	1	1	0.1	0.1	0.1	0	0	0
Mexico	Zacatepec De Hidalgo	0.3	0.2	0.3	1	0	1	0.1	0.1	0.1	0	0	0
Mexico	Zacatlan	0.5	0.4	0.6	4	3	6	0.1	0.1	0.2	1	1	2
Mexico	Zacazonapan	0.4	0.3	0.5	0	0	0	0.1	0.1	0.1	0	0	0
Mexico	Zacoalco De Torres	0.3	0.2	0.4	1	1	1	0.1	0.0	0.1	0	0	0
Mexico	Zacualpan	0.4	0.3	0.5	1	1	1	0.1	0.1	0.1	0	0	0
Mexico	Zacualpan	0.3	0.3	0.4	0	0	1	0.1	0.1	0.1	0	0	0
Mexico	Zacualpan De Amilpas	0.3	0.2	0.3	0	0	0	0.1	0.1	0.1	0	0	0
Mexico	Zacualtipan De Angeles	0.2	0.2	0.3	1	1	1	0.1	0.0	0.1	0	0	0
Mexico	Zamora	0.4	0.3	0.5	7	5	9	0.1	0.1	0.2	2	1	3
Mexico	Zapopan	0.2	0.2	0.3	33	25	43	0.1	0.0	0.1	6	4	9
Mexico	Zapotiltic	0.3	0.2	0.3	1	1	1	0.1	0.0	0.1	0	0	0
Mexico	Zapotitlan	0.5	0.4	0.7	1	0	1	0.1	0.1	0.2	0	0	0
Mexico	Zapotitlan De Mendez	0.5	0.4	0.7	0	0	0	0.1	0.1	0.2	0	0	0
Mexico	Zapotitlan De Vadillo	0.3	0.2	0.4	0	0	0	0.1	0.0	0.1	0	0	0
Mexico	Zapotitlan Del Rio	0.8	0.6	1.0	1	0	1	0.2	0.2	0.3	0	0	0
Mexico	Zapotitlan Lagunas	0.7	0.5	0.9	0	0	0	0.2	0.2	0.3	0	0	0
Mexico	Zapotitlan Palmas	0.6	0.5	0.8	0	0	0	0.2	0.1	0.3	0	0	0
Mexico	Zapotitlan Tablas	1.0	0.8	1.3	2	2	3	0.2	0.1	0.3	0	0	1
Mexico	Zapotlan De Juarez	0.2	0.2	0.3	1	0	1	0.1	0.0	0.1	0	0	0
Mexico	Zapotlan Del Rey	0.3	0.2	0.3	1	1	1	0.1	0.0	0.1	0	0	0
Mexico	Zapotlan El Grande	0.2	0.2	0.3	2	2	3	0.1	0.0	0.1	0	0	1
Mexico	Zapotlanejo	0.3	0.2	0.3	2	2	3	0.1	0.0	0.1	1	0	1
Mexico	Zaragoza	0.4	0.3	0.6	1	1	1	0.1	0.1	0.1	0	0	0
Mexico	Zaragoza	0.5	0.4	0.7	1	0	1	0.1	0.1	0.2	0	0	0
Mexico	Zaragoza	0.2	0.2	0.3	0	0	1	0.1	0.0	0.1	0	0	0
Mexico	Zaragoza	0.4	0.3	0.5	1	1	2	0.1	0.1	0.2	0	0	1
Mexico	Zautla	0.5	0.4	0.7	1	1	2	0.1	0.1	0.2	0	0	0
Mexico	Zempoala	0.2	0.2	0.3	1	1	1	0.1	0.0	0.1	0	0	0
Mexico	Zentla	0.4	0.3	0.5	1	0	1	0.1	0.1	0.1	0	0	0
Mexico	Zihuateutla	0.6	0.4	0.7	1	1	1	0.2	0.1	0.2	0	0	0
Mexico	Zimapan	0.2	0.2	0.3	1	1	2	0.1	0.0	0.1	0	0	0
Mexico	Zimatlan De Alvarez	0.7	0.5	0.9	2	1	2	0.2	0.2	0.3	0	0	1
Mexico	Zinacantan	1.1	0.8	1.4	5	4	7	0.3	0.2	0.4	2	1	2
Mexico	Zinacantepec	0.3	0.3	0.4	6	5	7	0.1	0.1	0.1	2	1	2
Mexico	Zinacatepec	0.6	0.4	0.7	1	1	1	0.2	0.1	0.2	0	0	0
Mexico	Zinaparo	0.4	0.3	0.5	0	0	0	0.1	0.1	0.2	0	0	0
Mexico	Zinapecuaro	0.4	0.3	0.5	3	2	4	0.1	0.1	0.2	1	0	1
Mexico	Ziracuaretiro	0.5	0.4	0.6	1	1	1	0.1	0.1	0.2	0	0	0
Mexico	Zirandaro	1.0	0.8	1.3	4	3	5	0.2	0.2	0.3	0	0	1
Mexico	Zitacuaro	0.4	0.3	0.5	8	6	10	0.1	0.1	0.2	2	2	3
Mexico	Zitlala	1.0	0.8	1.3	3	2	4	0.2	0.1	0.3	1	0	1
Mexico	Zitlaltepec De Trinidad Sanchez Santos	0.3	0.2	0.4	0	0	0	0.1	0.1	0.1	0	0	0
Mexico	Zongolica	0.4	0.3	0.5	2	2	3	0.1	0.1	0.1	0	0	0
Mexico	Zongozotla	0.6	0.4	0.7	0	0	1	0.2	0.1	0.2	0	0	0

Table 3: Diarrhoea Mortality by Unit *(continued)*

Country	Unit	2000 mortality mean rate (per 10,000)	lower rate (per 10,000)	upper rate (per 10,000)	mean count	lower count	upper count	2017 mortality mean rate (per 10,000)	lower rate (per 10,000)	upper rate (per 10,000)	mean count	lower count	upper count
Mexico	Zontecomatlan De Lopez Y Fuentes	0.4	0.3	0.5	1	1	1	0.1	0.1	0.1	0	0	0
Mexico	Zoquiapan	0.6	0.4	0.8	0	0	0	0.2	0.1	0.2	0	0	0
Mexico	Zoquitlan	0.6	0.5	0.8	2	2	3	0.2	0.1	0.2	0	0	1
Mexico	Zozocolco De Hidalgo	0.4	0.3	0.5	1	0	1	0.1	0.1	0.1	0	0	0
Mexico	Zumpahuacan	0.4	0.3	0.5	1	1	1	0.1	0.1	0.1	0	0	0
Mexico	Zumpango	0.3	0.2	0.4	4	3	5	0.1	0.1	0.1	2	2	3
Nicaragua	Achuapa	1.1	0.8	1.3	1	1	2	0.1	0.1	0.2	0	0	0
Nicaragua	Acoyapa	1.0	0.8	1.2	2	1	2	0.1	0.1	0.2	0	0	0
Nicaragua	Altagracia	0.9	0.7	1.1	1	1	1	0.1	0.1	0.2	0	0	0
Nicaragua	Belén	0.8	0.6	1.0	2	1	2	0.1	0.1	0.2	0	0	0
Nicaragua	Bluefields	1.0	0.8	1.3	7	6	9	0.1	0.1	0.2	1	1	1
Nicaragua	Boaco	1.1	0.9	1.3	6	5	7	0.1	0.1	0.2	1	0	1
Nicaragua	Bocana de Paiwas	1.6	1.3	1.9	3	3	4	0.1	0.1	0.2	0	0	1
Nicaragua	Bonanza	1.4	1.2	1.8	3	2	4	0.1	0.1	0.2	0	0	1
Nicaragua	Buenos Aires	0.7	0.6	0.9	1	1	1	0.1	0.1	0.1	0	0	0
Nicaragua	Camoapa	1.2	1.0	1.4	5	4	6	0.1	0.1	0.2	0	0	1
Nicaragua	Catarina	1.0	0.8	1.3	1	1	1	0.1	0.1	0.2	0	0	0
Nicaragua	Chichigalpa	1.0	0.8	1.3	5	4	6	0.1	0.1	0.2	0	0	1
Nicaragua	Chinandega	1.0	0.8	1.2	19	16	24	0.1	0.1	0.2	2	1	2
Nicaragua	Cinco Pinos	1.1	0.9	1.3	1	1	1	0.1	0.1	0.2	0	0	0
Nicaragua	Ciudad Antigua	1.2	1.0	1.5	1	1	1	0.1	0.1	0.2	0	0	0
Nicaragua	Ciudad Darío	1.0	0.8	1.2	7	6	8	0.1	0.1	0.2	1	1	1
Nicaragua	Ciudad Sandino	1.1	0.9	1.3	4	3	4	0.1	0.1	0.2	0	0	1
Nicaragua	Comalapa	1.0	0.8	1.2	1	1	2	0.1	0.1	0.2	0	0	0
Nicaragua	Condega	1.0	0.8	1.2	3	3	4	0.1	0.1	0.2	0	0	1
Nicaragua	Corinto	0.8	0.6	1.0	1	0	1	0.1	0.1	0.2	0	0	0
Nicaragua	Dipilto	1.1	0.9	1.4	1	1	1	0.1	0.1	0.2	0	0	0
Nicaragua	Diriá	1.0	0.8	1.3	1	1	1	0.1	0.1	0.2	0	0	0
Nicaragua	Diriamba	0.9	0.7	1.1	7	6	8	0.1	0.1	0.1	1	1	1
Nicaragua	Diriomo	1.1	0.9	1.3	2	2	2	0.1	0.1	0.2	0	0	0
Nicaragua	Dolores	0.9	0.7	1.1	1	1	1	0.1	0.1	0.1	0	0	0
Nicaragua	El Almendro	1.1	0.9	1.4	2	2	3	0.1	0.1	0.2	0	0	0
Nicaragua	El Cuá	1.5	1.3	1.9	20	17	25	0.2	0.1	0.2	3	2	4
Nicaragua	El Jicaral	1.0	0.8	1.2	1	1	2	0.1	0.1	0.2	0	0	0
Nicaragua	El Rama	1.4	1.2	1.8	16	13	19	0.1	0.1	0.2	1	1	2
Nicaragua	El Realejo	1.0	0.8	1.3	2	2	3	0.1	0.1	0.2	0	0	0
Nicaragua	El Rosario	1.0	0.8	1.2	1	1	1	0.1	0.1	0.1	0	0	0
Nicaragua	El Sauce	1.1	0.9	1.3	4	3	5	0.1	0.1	0.2	0	0	0
Nicaragua	El Viejo	1.0	0.8	1.2	9	7	11	0.1	0.1	0.2	1	1	1
Nicaragua	Esquipulas	1.2	0.9	1.4	2	2	3	0.1	0.1	0.2	0	0	0
Nicaragua	Estelí	0.9	0.7	1.1	11	9	13	0.1	0.1	0.1	1	1	2
Nicaragua	Granada	1.0	0.8	1.2	10	8	12	0.1	0.1	0.2	1	1	1
Nicaragua	Jalapa	1.2	0.9	1.5	8	7	10	0.1	0.1	0.2	1	1	2
Nicaragua	Jinotega	1.2	1.0	1.5	21	17	26	0.1	0.1	0.2	3	2	4
Nicaragua	Jinotepe	0.9	0.7	1.1	4	3	5	0.1	0.1	0.1	0	0	1
Nicaragua	Juigalpa	1.0	0.8	1.2	7	6	9	0.1	0.1	0.2	1	1	1
Nicaragua	Kukra Hill	1.4	1.1	1.8	2	2	2	0.1	0.1	0.2	0	0	0
Nicaragua	La Concepción	1.0	0.8	1.2	4	3	4	0.1	0.1	0.2	0	0	1
Nicaragua	La Concordia	1.0	0.8	1.2	2	1	2	0.1	0.1	0.2	0	0	0
Nicaragua	La Conquista	0.9	0.7	1.1	1	1	1	0.1	0.1	0.1	0	0	0
Nicaragua	La Cruz de Río Grande	1.8	1.5	2.2	10	8	12	0.1	0.1	0.2	2	1	3
Nicaragua	La Libertad	1.1	0.9	1.3	1	1	2	0.1	0.1	0.2	0	0	0
Nicaragua	La Paz Centro	0.9	0.8	1.1	3	3	4	0.1	0.1	0.2	0	0	0
Nicaragua	La Paz de Carazo	1.0	0.8	1.3	1	0	1	0.1	0.1	0.2	0	0	0
Nicaragua	La Trinidad	0.9	0.7	1.1	2	2	3	0.1	0.1	0.1	0	0	0
Nicaragua	Lago de Managua	0.9	0.8	1.1	13	11	16	0.1	0.1	0.2	1	1	2

Country	Unit	2000 mortality mean rate (per 10,000)	lower rate (per 10,000)	upper rate (per 10,000)	mean count	lower count	upper count	2017 mortality mean rate (per 10,000)	lower rate (per 10,000)	upper rate (per 10,000)	mean count	lower count	upper count
Nicaragua	Lago de Nicaragua	0.9	0.7	1.1	2	2	3	0.1	0.1	0.2	0	0	0
Nicaragua	Laguna de Perlas	1.4	1.1	1.7	3	2	3	0.1	0.1	0.2	0	0	1
Nicaragua	Larreynaga-Malpaisillo	0.8	0.7	1.0	3	2	4	0.1	0.1	0.1	0	0	0
Nicaragua	Las Sabanas	1.0	0.8	1.3	1	1	1	0.1	0.1	0.2	0	0	0
Nicaragua	León	0.9	0.8	1.1	19	15	23	0.1	0.1	0.2	2	1	2
Nicaragua	Macuelizo	1.1	0.8	1.3	1	1	1	0.1	0.1	0.2	0	0	0
Nicaragua	Managua	0.9	0.7	1.1	108	89	130	0.1	0.1	0.2	12	9	16
Nicaragua	Masatepe	1.0	0.8	1.2	5	4	6	0.1	0.1	0.2	1	0	1
Nicaragua	Masaya	1.0	0.8	1.2	16	13	19	0.1	0.1	0.2	2	1	2
Nicaragua	Matagalpa	1.1	0.9	1.3	19	16	23	0.1	0.1	0.2	3	2	3
Nicaragua	Mateare	1.0	0.8	1.2	5	4	6	0.1	0.1	0.2	1	1	1
Nicaragua	Matiguás	1.3	1.1	1.5	8	7	10	0.1	0.1	0.2	1	1	1
Nicaragua	Morrito	1.1	0.9	1.4	1	1	1	0.1	0.1	0.2	0	0	0
Nicaragua	Moyogalpa	0.8	0.6	1.0	1	0	1	0.1	0.1	0.2	0	0	0
Nicaragua	Mozonte	1.1	0.9	1.4	1	1	1	0.1	0.1	0.2	0	0	0
Nicaragua	Muelle de los Bueyes	1.2	1.0	1.5	4	3	5	0.1	0.1	0.2	0	0	0
Nicaragua	Murra	1.4	1.1	1.7	3	2	4	0.2	0.1	0.2	0	0	1
Nicaragua	Muy Muy	1.1	0.9	1.4	3	2	3	0.1	0.1	0.2	0	0	0
Nicaragua	Nagarote	0.9	0.8	1.1	3	3	4	0.1	0.1	0.2	0	0	0
Nicaragua	Nandaime	1.0	0.8	1.2	5	4	6	0.1	0.1	0.2	0	0	1
Nicaragua	Nandasmo	1.1	0.9	1.3	2	1	2	0.1	0.1	0.2	0	0	0
Nicaragua	Nindirí	1.0	0.8	1.2	7	6	8	0.1	0.1	0.1	1	1	1
Nicaragua	Niquinohomo	1.1	0.9	1.3	1	1	1	0.1	0.1	0.2	0	0	0
Nicaragua	Nueva Guinea	1.1	0.9	1.4	14	11	17	0.1	0.1	0.2	1	1	1
Nicaragua	Ocotal	0.9	0.8	1.2	4	4	5	0.1	0.1	0.2	1	0	1
Nicaragua	Palacagüina	1.0	0.8	1.2	2	2	2	0.1	0.1	0.2	0	0	0
Nicaragua	Posoltega	1.0	0.8	1.3	2	2	2	0.1	0.1	0.2	0	0	0
Nicaragua	Potosí	0.8	0.6	0.9	1	1	1	0.1	0.1	0.1	0	0	0
Nicaragua	Prinzapolka	1.8	1.5	2.3	3	2	3	0.2	0.1	0.2	1	1	1
Nicaragua	Pueblo Nuevo	1.0	0.8	1.2	3	2	3	0.1	0.1	0.2	0	0	0
Nicaragua	Puerto Cabezas	1.3	1.0	1.7	8	6	10	0.2	0.1	0.2	2	1	2
Nicaragua	Puerto Morazán	1.0	0.8	1.3	1	1	1	0.1	0.1	0.2	0	0	0
Nicaragua	Quezalguaque	1.0	0.8	1.2	2	1	2	0.1	0.1	0.2	0	0	0
Nicaragua	Quilalí	1.2	1.0	1.5	5	4	7	0.1	0.1	0.2	1	0	1
Nicaragua	Rancho Grande	1.5	1.3	1.8	5	5	7	0.1	0.1	0.2	1	1	1
Nicaragua	Río Blanco	1.6	1.4	2.0	15	12	18	0.1	0.1	0.2	1	1	2
Nicaragua	Rivas	0.7	0.5	0.8	2	2	3	0.1	0.1	0.1	0	0	0
Nicaragua	Rosita	1.6	1.3	2.0	7	5	8	0.1	0.1	0.2	1	1	1
Nicaragua	San Carlos	1.0	0.8	1.2	6	5	8	0.1	0.1	0.2	1	1	2
Nicaragua	San Dionisio	1.2	1.0	1.4	3	2	4	0.1	0.1	0.2	0	0	0
Nicaragua	San Fernando	1.1	0.9	1.4	2	2	3	0.1	0.1	0.2	0	0	0
Nicaragua	San Francisco del Norte	1.0	0.8	1.3	1	1	1	0.1	0.1	0.2	0	0	0
Nicaragua	San Francisco Libre	1.0	0.8	1.2	1	1	1	0.1	0.1	0.2	0	0	0
Nicaragua	San Isidro	1.0	0.8	1.2	1	1	2	0.1	0.1	0.2	0	0	0
Nicaragua	San Jorge	0.7	0.5	0.8	0	0	1	0.1	0.1	0.1	0	0	0
Nicaragua	San José de Cusmapa	1.2	1.0	1.5	1	0	1	0.1	0.1	0.2	0	0	0
Nicaragua	San José de los Remates	1.0	0.9	1.2	1	1	2	0.1	0.1	0.2	0	0	0
Nicaragua	San Juan de Limay	1.0	0.8	1.2	2	2	3	0.1	0.1	0.2	0	0	0
Nicaragua	San Juan de Oriente	1.0	0.8	1.3	0	0	0	0.1	0.1	0.2	0	0	0
Nicaragua	San Juan del Norte	0.9	0.7	1.2	0	0	0	0.1	0.1	0.2	0	0	0

Table 3: Diarrhoea Mortality by Unit *(continued)*

Country	Unit	2000 mortality						2017 mortality					
		mean rate (per 10,000)	lower rate (per 10,000)	upper rate (per 10,000)	mean count	lower count	upper count	mean rate (per 10,000)	lower rate (per 10,000)	upper rate (per 10,000)	mean count	lower count	upper count
Nicaragua	San Juan del Río Coco	1.2	1.0	1.5	3	3	4	0.2	0.1	0.2	0	0	1
Nicaragua	San Lorenzo	1.0	0.8	1.2	4	3	4	0.1	0.1	0.2	0	0	0
Nicaragua	San Lucas	1.1	0.8	1.3	2	1	2	0.1	0.1	0.2	0	0	0
Nicaragua	San Marcos	1.0	0.8	1.2	3	2	4	0.1	0.1	0.2	0	0	0
Nicaragua	San Miguelito	1.1	0.9	1.4	3	2	3	0.1	0.1	0.2	0	0	0
Nicaragua	San Nicolás	1.0	0.8	1.2	1	1	1	0.1	0.1	0.2	0	0	0
Nicaragua	San Pedro de Lóvago	1.0	0.8	1.2	2	2	2	0.1	0.1	0.2	0	0	0
Nicaragua	San Pedro del Norte	1.2	0.9	1.4	2	1	2	0.1	0.1	0.2	0	0	0
Nicaragua	San Rafael del Norte	1.1	0.9	1.4	5	4	6	0.1	0.1	0.2	1	0	1
Nicaragua	San Rafael del Sur	0.9	0.7	1.1	5	4	6	0.1	0.1	0.2	1	0	1
Nicaragua	San Ramón	1.3	1.1	1.6	7	5	8	0.1	0.1	0.2	1	1	1
Nicaragua	San Sebastián de Yalí	1.2	1.0	1.4	4	3	5	0.1	0.1	0.2	1	0	1
Nicaragua	Santa Lucía	1.1	0.9	1.3	1	1	2	0.1	0.1	0.2	0	0	0
Nicaragua	Santa María	1.1	0.9	1.4	1	1	1	0.1	0.1	0.2	0	0	0
Nicaragua	Santa María de Pantasma	1.3	1.1	1.6	8	7	10	0.1	0.1	0.2	1	1	1
Nicaragua	Santa Rosa del Peñón	1.0	0.8	1.3	1	1	1	0.1	0.1	0.2	0	0	0
Nicaragua	Santo Domingo	1.1	0.9	1.3	2	1	2	0.1	0.1	0.2	0	0	0
Nicaragua	Santo Tomás	1.0	0.8	1.2	2	2	3	0.1	0.1	0.2	0	0	0
Nicaragua	Sébaco	1.0	0.8	1.2	4	4	5	0.1	0.1	0.2	1	0	1
Nicaragua	Siuna	1.7	1.4	2.1	16	13	20	0.1	0.1	0.2	2	1	3
Nicaragua	Somotillo	1.0	0.8	1.2	5	4	6	0.1	0.1	0.2	0	0	1
Nicaragua	Somoto	1.0	0.8	1.3	5	4	6	0.1	0.1	0.2	1	0	1
Nicaragua	Telica	0.9	0.8	1.2	3	3	4	0.1	0.1	0.2	0	0	0
Nicaragua	Telpaneca	1.1	0.9	1.4	2	2	3	0.1	0.1	0.2	0	0	0
Nicaragua	Terrabona	1.1	0.9	1.3	2	2	3	0.1	0.1	0.2	0	0	0
Nicaragua	Teustepe	1.0	0.8	1.2	3	3	4	0.1	0.1	0.2	0	0	1
Nicaragua	Ticuantepe	1.0	0.8	1.2	3	2	3	0.1	0.1	0.2	0	0	0
Nicaragua	Tipitapa	1.0	0.8	1.2	16	14	20	0.1	0.1	0.2	2	1	3
Nicaragua	Tisma	1.0	0.8	1.2	2	1	2	0.1	0.1	0.2	0	0	0
Nicaragua	Tola	0.7	0.6	0.9	4	3	4	0.1	0.1	0.2	0	0	1
Nicaragua	Totogalpa	1.1	0.9	1.4	2	1	2	0.1	0.1	0.2	0	0	0
Nicaragua	Tuma-La Dalia	1.5	1.2	1.8	8	6	9	0.2	0.1	0.2	1	1	1
Nicaragua	Villa Carlos Fonseca	0.9	0.8	1.1	4	3	5	0.1	0.1	0.2	0	0	1
Nicaragua	Villa Sandino	1.0	0.8	1.2	2	2	3	0.1	0.1	0.1	0	0	0
Nicaragua	Villanueva	1.0	0.8	1.3	4	3	5	0.1	0.1	0.2	0	0	1
Nicaragua	Waslala	1.7	1.4	2.0	10	9	12	0.1	0.1	0.2	1	1	2
Nicaragua	Waspán	1.3	1.1	1.7	7	6	9	0.2	0.1	0.2	1	1	1
Nicaragua	Wiwilí	1.5	1.2	1.8	16	13	19	0.2	0.1	0.2	2	2	3
Nicaragua	Yalagüina	1.0	0.8	1.2	1	1	1	0.1	0.1	0.2	0	0	0
Panama	Aguadulce	0.3	0.2	0.4	1	1	1	0.2	0.1	0.2	1	0	1
Panama	Alanje	0.3	0.2	0.4	0	0	1	0.2	0.1	0.2	0	0	0
Panama	Antón	0.3	0.2	0.4	1	1	2	0.2	0.1	0.2	1	1	1
Panama	Arraiján	0.3	0.2	0.4	6	4	9	0.2	0.1	0.3	5	4	7
Panama	Atalaya	0.3	0.2	0.4	0	0	0	0.2	0.2	0.3	0	0	0
Panama	Balboa	0.3	0.2	0.4	0	0	0	0.2	0.1	0.3	0	0	0
Panama	Barú	0.3	0.2	0.4	1	1	2	0.2	0.1	0.2	1	1	1
Panama	Besiko	0.4	0.3	0.5	1	1	1	0.2	0.2	0.3	1	1	1
Panama	Bocas del Toro	0.3	0.2	0.5	1	0	1	0.3	0.2	0.4	1	1	1
Panama	Boquerón	0.3	0.2	0.4	0	0	1	0.2	0.1	0.3	0	0	1
Panama	Boquete	0.3	0.2	0.3	1	0	1	0.2	0.1	0.3	0	0	1
Panama	Bugaba	0.3	0.2	0.4	2	1	2	0.2	0.1	0.3	1	1	2

Table 3: Diarrhoea Mortality by Unit *(continued)*

Country	Unit	2000 mortality mean rate (per 10,000)	2000 mortality lower rate (per 10,000)	2000 mortality upper rate (per 10,000)	2000 mortality mean count	2000 mortality lower count	2000 mortality upper count	2017 mortality mean rate (per 10,000)	2017 mortality lower rate (per 10,000)	2017 mortality upper rate (per 10,000)	2017 mortality mean count	2017 mortality lower count	2017 mortality upper count
Panama	Calobre	0.3	0.2	0.4	0	0	1	0.2	0.2	0.3	0	0	0
Panama	Cañazas	0.3	0.2	0.4	1	0	1	0.2	0.2	0.3	0	0	1
Panama	Capira	0.3	0.2	0.4	1	1	2	0.2	0.1	0.3	1	1	1
Panama	Cémaco	0.3	0.2	0.5	0	0	0	0.2	0.2	0.3	0	0	0
Panama	Chagres	0.3	0.2	0.4	0	0	0	0.2	0.2	0.3	0	0	0
Panama	Chame	0.3	0.2	0.4	1	0	1	0.2	0.1	0.2	0	0	1
Panama	Changuinola	0.3	0.2	0.5	3	2	5	0.3	0.2	0.4	4	3	5
Panama	Chepigana	0.3	0.2	0.4	1	1	2	0.2	0.2	0.3	1	1	1
Panama	Chepo	0.3	0.2	0.4	2	1	2	0.2	0.1	0.3	1	1	2
Panama	Chimán	0.3	0.2	0.4	0	0	0	0.2	0.2	0.3	0	0	0
Panama	Chiriquí Grande	0.3	0.2	0.5	0	0	1	0.3	0.2	0.4	1	0	1
Panama	Chitré	0.2	0.2	0.3	1	1	1	0.2	0.1	0.2	0	0	1
Panama	Colón	0.3	0.2	0.4	5	4	7	0.2	0.1	0.3	4	3	6
Panama	David	0.3	0.2	0.3	3	2	5	0.2	0.1	0.3	3	2	4
Panama	Dolega	0.3	0.2	0.4	1	0	1	0.2	0.1	0.3	0	0	1
Panama	Donoso	0.3	0.2	0.4	0	0	1	0.2	0.2	0.3	0	0	1
Panama	Gualaca	0.3	0.2	0.4	0	0	0	0.2	0.1	0.3	0	0	0
Panama	Guararé	0.2	0.2	0.3	0	0	0	0.2	0.1	0.2	0	0	0
Panama	Kankintú	0.3	0.2	0.4	1	1	2	0.2	0.2	0.3	2	1	2
Panama	Kuna Yala	0.3	0.2	0.4	1	1	1	0.2	0.1	0.3	1	0	1
Panama	Kusapín	0.3	0.2	0.4	1	0	1	0.2	0.2	0.3	1	0	1
Panama	La Chorrera	0.3	0.2	0.4	5	3	6	0.2	0.1	0.3	4	3	5
Panama	La Mesa	0.3	0.2	0.4	0	0	1	0.2	0.2	0.3	0	0	0
Panama	La Pintada	0.3	0.2	0.4	1	0	1	0.2	0.1	0.2	1	0	1
Panama	Lago Alajuela	0.3	0.2	0.4	0	0	0	0.2	0.2	0.3	0	0	0
Panama	Lago Bayano	0.3	0.2	0.4	0	0	0	0.2	0.2	0.3	0	0	0
Panama	Lago Gatún	0.3	0.2	0.4	0	0	0	0.2	0.1	0.3	0	0	0
Panama	Lago Gatún	0.3	0.2	0.4	0	0	0	0.2	0.2	0.3	0	0	0
Panama	Las Minas	0.2	0.2	0.3	0	0	0	0.2	0.1	0.2	0	0	0
Panama	Las Palmas	0.3	0.2	0.4	1	0	1	0.2	0.2	0.3	1	0	1
Panama	Las Tablas	0.2	0.2	0.3	0	0	1	0.2	0.1	0.2	0	0	0
Panama	Los Pozos	0.2	0.2	0.3	0	0	0	0.2	0.1	0.2	0	0	0
Panama	Los Santos	0.2	0.2	0.3	1	0	1	0.2	0.1	0.2	0	0	0
Panama	Macaracas	0.2	0.2	0.3	0	0	0	0.2	0.1	0.2	0	0	0
Panama	Mironó	0.4	0.3	0.5	1	0	1	0.2	0.2	0.3	1	0	1
Panama	Montijo	0.3	0.2	0.4	0	0	0	0.2	0.2	0.3	0	0	0
Panama	Müna	0.4	0.3	0.5	2	1	2	0.3	0.2	0.3	2	1	2
Panama	Natá	0.3	0.2	0.4	1	0	1	0.2	0.1	0.2	0	0	1
Panama	Nole Duima	0.4	0.3	0.5	1	0	1	0.3	0.2	0.3	1	0	1
Panama	Ñürüm	0.3	0.2	0.5	0	0	1	0.2	0.2	0.3	0	0	1
Panama	Ocú	0.3	0.2	0.4	0	0	1	0.2	0.1	0.2	0	0	0
Panama	Olá	0.3	0.2	0.4	0	0	0	0.2	0.1	0.3	0	0	0
Panama	Panamá	0.3	0.2	0.4	23	16	31	0.2	0.2	0.3	18	13	25
Panama	Parita	0.3	0.2	0.3	0	0	0	0.2	0.1	0.2	0	0	0
Panama	Pedasí	0.2	0.2	0.3	0	0	0	0.2	0.1	0.2	0	0	0
Panama	Penonomé	0.3	0.2	0.4	2	2	3	0.2	0.1	0.2	2	1	2
Panama	Pesé	0.2	0.2	0.3	0	0	0	0.2	0.1	0.2	0	0	0
Panama	Pinogana	0.3	0.2	0.4	1	1	1	0.2	0.2	0.3	1	0	1
Panama	Pocrí	0.2	0.2	0.3	0	0	0	0.2	0.1	0.2	0	0	0
Panama	Portobelo	0.3	0.2	0.4	0	0	0	0.2	0.2	0.3	0	0	0
Panama	Remedios	0.3	0.2	0.4	0	0	0	0.2	0.2	0.3	0	0	0
Panama	Renacimiento	0.3	0.2	0.4	1	0	1	0.2	0.1	0.2	0	0	1
Panama	Río de Jesús	0.3	0.2	0.4	0	0	0	0.2	0.2	0.3	0	0	0
Panama	Sambú	0.3	0.2	0.5	0	0	0	0.2	0.2	0.3	0	0	0
Panama	San Carlos	0.3	0.2	0.4	1	0	1	0.2	0.1	0.2	0	0	1
Panama	San Félix	0.3	0.2	0.4	0	0	0	0.2	0.1	0.3	0	0	0
Panama	San Francisco	0.3	0.2	0.4	0	0	0	0.2	0.2	0.3	0	0	0
Panama	San Lorenzo	0.3	0.2	0.4	0	0	0	0.2	0.2	0.3	0	0	0
Panama	San Miguelito	0.3	0.2	0.5	7	5	10	0.2	0.2	0.3	6	5	8
Panama	Santa Fe	0.3	0.2	0.4	1	0	1	0.2	0.2	0.3	1	0	1

Table 3: Diarrhoea Mortality by Unit *(continued)*

Country	Unit	2000 mortality mean rate (per 10,000)	lower rate (per 10,000)	upper rate (per 10,000)	mean count	lower count	upper count	2017 mortality mean rate (per 10,000)	lower rate (per 10,000)	upper rate (per 10,000)	mean count	lower count	upper count
Panama	Santa Isabel	0.3	0.2	0.4	0	0	0	0.2	0.2	0.3	0	0	0
Panama	Santa María	0.3	0.2	0.4	0	0	0	0.2	0.1	0.2	0	0	0
Panama	Santiago	0.3	0.2	0.4	2	2	3	0.2	0.2	0.3	2	1	2
Panama	Soná	0.3	0.2	0.4	1	1	1	0.2	0.2	0.3	1	0	1
Panama	Taboga	0.3	0.2	0.5	0	0	0	0.2	0.2	0.3	0	0	0
Panama	Tolé	0.3	0.2	0.4	1	0	1	0.2	0.2	0.3	1	0	1
Panama	Tonosí	0.2	0.2	0.3	0	0	0	0.2	0.1	0.2	0	0	0
Paraguay	25 de Diciembre	0.5	0.4	0.6	1	0	1	0.1	0.1	0.1	0	0	0
Paraguay	3 de Febrero	0.5	0.4	0.6	0	0	1	0.1	0.1	0.1	0	0	0
Paraguay	Abaí	0.4	0.3	0.6	2	1	2	0.1	0.1	0.1	0	0	0
Paraguay	Acahay	0.4	0.3	0.6	1	1	1	0.1	0.1	0.1	0	0	0
Paraguay	Alberdi	0.5	0.4	0.6	2	2	3	0.1	0.1	0.1	0	0	0
Paraguay	Alto Verá	0.5	0.4	0.6	1	1	1	0.1	0.1	0.1	0	0	0
Paraguay	Altos	0.5	0.4	0.6	1	1	1	0.1	0.1	0.1	0	0	0
Paraguay	Antequera	0.5	0.4	0.7	0	0	0	0.1	0.1	0.1	0	0	0
Paraguay	Areguá	0.5	0.4	0.6	3	2	4	0.1	0.1	0.1	1	1	1
Paraguay	Arroyos y Esteros	0.5	0.4	0.6	1	1	2	0.1	0.1	0.1	0	0	0
Paraguay	Asunción	0.5	0.4	0.6	22	17	27	0.1	0.1	0.1	3	3	4
Paraguay	Atyrá	0.5	0.4	0.6	1	1	1	0.1	0.1	0.1	0	0	0
Paraguay	Ayolas	0.5	0.4	0.6	1	1	1	0.1	0.1	0.1	0	0	0
Paraguay	Belén	0.5	0.4	0.6	1	0	1	0.1	0.1	0.1	0	0	0
Paraguay	Bella Vista	0.5	0.4	0.7	1	1	1	0.1	0.1	0.1	0	0	0
Paraguay	Bella Vista	0.5	0.4	0.6	1	0	1	0.1	0.1	0.1	0	0	0
Paraguay	Benjamín Aceval	0.5	0.4	0.6	1	1	1	0.1	0.1	0.1	0	0	0
Paraguay	Borja	0.4	0.3	0.6	0	0	1	0.1	0.1	0.1	0	0	0
Paraguay	Caacupé	0.5	0.4	0.6	2	2	3	0.1	0.1	0.1	0	0	1
Paraguay	Caaguazú	0.5	0.4	0.6	6	5	8	0.1	0.1	0.1	1	1	1
Paraguay	Caapucú	0.5	0.4	0.6	0	0	1	0.1	0.1	0.1	0	0	0
Paraguay	Caazapá	0.4	0.3	0.6	1	1	2	0.1	0.1	0.1	0	0	0
Paraguay	Cambyreta	0.5	0.4	0.6	2	2	3	0.1	0.1	0.1	1	0	1
Paraguay	Capiatá	0.5	0.4	0.6	9	7	11	0.1	0.1	0.1	2	2	2
Paraguay	Capitán Bado	0.5	0.4	0.6	1	1	1	0.1	0.1	0.1	0	0	0
Paraguay	Capitán Mauricio José Troche	0.4	0.3	0.6	0	0	1	0.1	0.1	0.1	0	0	0
Paraguay	Capitán Meza	0.5	0.4	0.6	1	1	2	0.1	0.1	0.1	0	0	0
Paraguay	Capitán Miranda	0.5	0.4	0.6	1	0	1	0.1	0.1	0.1	0	0	0
Paraguay	Caraguatay	0.4	0.3	0.6	1	1	1	0.1	0.1	0.1	0	0	0
Paraguay	Carapeguá	0.4	0.3	0.6	2	1	2	0.1	0.1	0.1	0	0	0
Paraguay	Carayaó	0.4	0.3	0.6	1	1	1	0.1	0.1	0.1	0	0	0
Paraguay	Carlos Antonio López	0.5	0.4	0.6	1	1	1	0.1	0.1	0.1	0	0	0
Paraguay	Carmen del Paraná	0.5	0.4	0.6	0	0	1	0.1	0.1	0.1	0	0	0
Paraguay	Cerrito	0.5	0.4	0.7	0	0	0	0.1	0.1	0.1	0	0	0
Paraguay	Choré	0.5	0.4	0.6	3	2	3	0.1	0.1	0.1	0	0	1
Paraguay	Ciudad del Este	0.5	0.4	0.7	12	9	16	0.1	0.1	0.1	2	2	3
Paraguay	Concepción	0.6	0.4	0.7	6	4	7	0.1	0.1	0.1	1	1	1
Paraguay	Coronel Bogado	0.5	0.4	0.6	1	1	1	0.1	0.1	0.1	0	0	0
Paraguay	Coronel Martínez	0.5	0.4	0.6	0	0	0	0.1	0.1	0.1	0	0	0
Paraguay	Coronel Oviedo	0.4	0.3	0.6	5	4	7	0.1	0.1	0.1	1	1	1
Paraguay	Corpus Christi	0.5	0.4	0.6	1	1	1	0.1	0.1	0.1	0	0	0
Paraguay	Desmochados	0.5	0.4	0.6	0	0	0	0.1	0.1	0.1	0	0	0
Paraguay	Doctor Botrell	0.4	0.3	0.6	0	0	0	0.1	0.1	0.1	0	0	0
Paraguay	Doctor Cecilio Báez	0.4	0.3	0.6	0	0	0	0.1	0.1	0.1	0	0	0
Paraguay	Doctor J. Eulogio Estigarribia	0.5	0.4	0.6	2	1	2	0.1	0.1	0.1	0	0	0
Paraguay	Doctor Juan León Mallorquín	0.5	0.4	0.6	1	1	1	0.1	0.1	0.1	0	0	0
Paraguay	Doctor Juan Manuel Frutos	0.5	0.4	0.6	1	1	2	0.1	0.1	0.1	0	0	0

Table 3: Diarrhoea Mortality by Unit *(continued)*

Country	Unit	2000 mortality mean rate (per 10,000)	lower rate (per 10,000)	upper rate (per 10,000)	mean count	lower count	upper count	2017 mortality mean rate (per 10,000)	lower rate (per 10,000)	upper rate (per 10,000)	mean count	lower count	upper count
Paraguay	Doctor Moisés S. Bertoni	0.4	0.3	0.6	0	0	0	0.1	0.1	0.1	0	0	0
Paraguay	Doctor Pedro P. Peña	0.6	0.5	0.7	1	1	1	0.1	0.1	0.1	0	0	0
Paraguay	Domingo Martínez de Irala	0.5	0.4	0.6	0	0	0	0.1	0.1	0.1	0	0	0
Paraguay	Edelira	0.5	0.4	0.6	1	1	2	0.1	0.1	0.1	0	0	0
Paraguay	Emboscada	0.5	0.4	0.6	1	1	1	0.1	0.1	0.1	0	0	0
Paraguay	Emboscada (Caazapa)	0.4	0.3	0.6	0	0	0	0.1	0.1	0.1	0	0	0
Paraguay	Encarnación	0.5	0.4	0.6	7	6	9	0.1	0.1	0.1	2	1	2
Paraguay	Escobar	0.4	0.4	0.6	1	0	1	0.1	0.1	0.1	0	0	0
Paraguay	Eusebio Ayala	0.4	0.3	0.6	1	1	2	0.1	0.1	0.1	0	0	0
Paraguay	Félix Perez Cardozo	0.5	0.4	0.6	0	0	0	0.1	0.1	0.1	0	0	0
Paraguay	Fernando de la Mora	0.5	0.4	0.6	6	4	7	0.1	0.1	0.1	1	1	1
Paraguay	Fram	0.4	0.3	0.6	0	0	0	0.1	0.1	0.1	0	0	0
Paraguay	Fuerte Olimpo	0.6	0.4	0.7	0	0	0	0.1	0.1	0.1	0	0	0
Paraguay	Fulgencio Yegros	0.4	0.3	0.6	0	0	0	0.1	0.1	0.1	0	0	0
Paraguay	General Artigas	0.5	0.4	0.6	1	1	1	0.1	0.1	0.1	0	0	0
Paraguay	General Bernardino Caballero	0.4	0.3	0.6	0	0	0	0.1	0.1	0.1	0	0	0
Paraguay	General Delgado	0.5	0.4	0.6	0	0	0	0.1	0.1	0.1	0	0	0
Paraguay	General Elizardo Aquino	0.5	0.4	0.6	1	1	2	0.1	0.1	0.1	0	0	0
Paraguay	General Eugenio A. Garay	0.6	0.5	0.7	1	1	1	0.1	0.1	0.1	0	0	0
Paraguay	General Eugenio A. Garay	0.4	0.3	0.6	0	0	1	0.1	0.1	0.1	0	0	0
Paraguay	General Francisco C. Alvarez	0.5	0.4	0.6	3	2	3	0.1	0.1	0.1	1	0	1
Paraguay	General Higinio Morínigo	0.4	0.3	0.6	0	0	0	0.1	0.1	0.1	0	0	0
Paraguay	General Isidoro Resquín	0.5	0.4	0.6	2	1	2	0.1	0.1	0.1	0	0	0
Paraguay	General José Eduvigis Díaz	0.5	0.4	0.6	0	0	0	0.1	0.1	0.1	0	0	0
Paraguay	Guarambaré	0.4	0.3	0.6	1	1	1	0.1	0.1	0.1	0	0	0
Paraguay	Guazú Cuá	0.5	0.4	0.6	0	0	0	0.1	0.1	0.1	0	0	0
Paraguay	Hernandarias	0.5	0.4	0.7	5	4	6	0.1	0.1	0.1	1	1	1
Paraguay	Hohenau	0.5	0.4	0.6	1	0	1	0.1	0.1	0.1	0	0	0
Paraguay	Horqueta	0.5	0.4	0.6	4	3	5	0.1	0.1	0.1	1	1	1
Paraguay	Humaitá	0.5	0.4	0.7	0	0	0	0.1	0.1	0.1	0	0	0
Paraguay	Independencia	0.4	0.3	0.6	2	2	3	0.1	0.1	0.1	0	0	0
Paraguay	Isla Pucú	0.4	0.3	0.6	0	0	0	0.1	0.1	0.1	0	0	0
Paraguay	Isla Umbú	0.5	0.4	0.6	0	0	0	0.1	0.1	0.1	0	0	0
Paraguay	Itá	0.4	0.3	0.6	3	2	4	0.1	0.1	0.1	1	1	1
Paraguay	Itacurubí de la Cordillera	0.4	0.3	0.5	1	0	1	0.1	0.1	0.1	0	0	0
Paraguay	Itacurubí del Rosario	0.5	0.4	0.6	1	1	1	0.1	0.1	0.1	0	0	0
Paraguay	Itakyry	0.5	0.4	0.6	1	1	2	0.1	0.1	0.1	0	0	0
Paraguay	Itanara	0.5	0.4	0.6	0	0	0	0.1	0.1	0.1	0	0	0
Paraguay	Itapé	0.5	0.3	0.6	0	0	1	0.1	0.1	0.1	0	0	0
Paraguay	Itauguá	0.5	0.4	0.6	4	3	4	0.1	0.1	0.1	1	1	1
Paraguay	Iturbe	0.4	0.3	0.6	1	0	1	0.1	0.1	0.1	0	0	0
Paraguay	Jesús	0.5	0.4	0.6	0	0	0	0.1	0.1	0.1	0	0	0
Paraguay	Jose A. Fassardi	0.4	0.3	0.6	1	0	1	0.1	0.1	0.1	0	0	0

Table 3: Diarrhoea Mortality by Unit *(continued)*

Country	Unit	2000 mortality mean rate (per 10,000)	lower rate (per 10,000)	upper rate (per 10,000)	mean count	lower count	upper count	2017 mortality mean rate (per 10,000)	lower rate (per 10,000)	upper rate (per 10,000)	mean count	lower count	upper count
Paraguay	José Domingo Ocampos	0.5	0.4	0.6	1	1	1	0.1	0.1	0.1	0	0	0
Paraguay	José Leandro Oviedo	0.4	0.3	0.6	0	0	0	0.1	0.1	0.1	0	0	0
Paraguay	Juan Augusto Saldívar	0.4	0.4	0.6	2	2	3	0.1	0.1	0.1	0	0	1
Paraguay	Juan de Mena	0.5	0.4	0.6	0	0	0	0.1	0.1	0.1	0	0	0
Paraguay	Juan Emilio O'Leary	0.5	0.4	0.6	1	1	1	0.1	0.1	0.1	0	0	0
Paraguay	La Colmena	0.4	0.3	0.6	0	0	0	0.1	0.1	0.1	0	0	0
Paraguay	La Pastora	0.4	0.3	0.6	0	0	0	0.1	0.1	0.1	0	0	0
Paraguay	La Paz	0.4	0.4	0.6	0	0	0	0.1	0.1	0.1	0	0	0
Paraguay	La Victoria	0.6	0.4	0.7	1	1	1	0.1	0.1	0.1	0	0	0
Paraguay	Lambaré	0.5	0.4	0.7	6	4	7	0.1	0.1	0.1	1	1	1
Paraguay	Laureles	0.5	0.4	0.6	0	0	0	0.1	0.1	0.1	0	0	0
Paraguay	Lima	0.5	0.4	0.6	1	1	1	0.1	0.1	0.1	0	0	0
Paraguay	Limpio	0.5	0.4	0.6	4	3	5	0.1	0.1	0.1	1	1	2
Paraguay	Loma Grande	0.5	0.4	0.6	0	0	0	0.1	0.1	0.1	0	0	0
Paraguay	Loreto	0.5	0.4	0.7	1	1	2	0.1	0.1	0.1	0	0	0
Paraguay	Los Cedrales	0.5	0.4	0.6	1	0	1	0.1	0.1	0.1	0	0	0
Paraguay	Luque	0.5	0.4	0.6	12	10	15	0.1	0.1	0.1	3	2	3
Paraguay	Maciel	0.4	0.3	0.6	0	0	0	0.1	0.1	0.1	0	0	0
Paraguay	Mariano Roque Alonso	0.5	0.4	0.6	3	3	4	0.1	0.1	0.1	1	1	1
Paraguay	Mariscal Francisco Solano López	0.5	0.4	0.6	1	0	1	0.1	0.1	0.1	0	0	0
Paraguay	Mariscal José Félix Estigarribia	0.5	0.4	0.7	1	1	2	0.1	0.1	0.1	0	0	0
Paraguay	Mayor José J. Martinez	0.5	0.4	0.6	0	0	0	0.1	0.1	0.1	0	0	0
Paraguay	Mayor Julio D. Otaño	0.5	0.4	0.6	1	1	1	0.1	0.1	0.1	0	0	0
Paraguay	Mayor Pablo Lagerenza	0.6	0.4	0.7	0	0	0	0.1	0.1	0.1	0	0	0
Paraguay	Mbaracayú	0.5	0.4	0.6	1	0	1	0.1	0.1	0.1	0	0	0
Paraguay	Mbocayaty del Guairá	0.4	0.3	0.6	0	0	1	0.1	0.1	0.1	0	0	0
Paraguay	Mbocayaty del Yhaguy	0.4	0.3	0.6	0	0	0	0.1	0.1	0.1	0	0	0
Paraguay	Mbuyapey	0.4	0.3	0.6	1	1	1	0.1	0.1	0.1	0	0	0
Paraguay	Minga Guazú	0.5	0.4	0.6	3	3	5	0.1	0.1	0.1	1	1	1
Paraguay	Minga Porá	0.5	0.3	0.6	1	1	1	0.1	0.1	0.1	0	0	0
Paraguay	Ñacunday	0.5	0.4	0.6	1	1	1	0.1	0.1	0.1	0	0	0
Paraguay	Naranjal	0.4	0.3	0.5	1	1	1	0.1	0.1	0.1	0	0	0
Paraguay	Natalicio Talavera	0.4	0.3	0.6	0	0	0	0.1	0.1	0.1	0	0	0
Paraguay	Natalio	0.5	0.4	0.6	1	1	1	0.1	0.1	0.1	0	0	0
Paraguay	Ñemby	0.5	0.4	0.6	4	3	5	0.1	0.1	0.1	1	1	1
Paraguay	Nueva Alborada	0.5	0.4	0.6	0	0	1	0.1	0.1	0.1	0	0	0
Paraguay	Nueva Colombia	0.5	0.4	0.6	0	0	0	0.1	0.1	0.1	0	0	0
Paraguay	Nueva Germania	0.5	0.4	0.6	2	1	2	0.1	0.1	0.1	0	0	1
Paraguay	Nueva Italia	0.5	0.4	0.6	0	0	1	0.1	0.1	0.1	0	0	0
Paraguay	Nueva Londres	0.4	0.3	0.6	0	0	0	0.1	0.1	0.1	0	0	0
Paraguay	Ñumí	0.5	0.4	0.6	0	0	0	0.1	0.1	0.1	0	0	0
Paraguay	Obligado	0.5	0.4	0.6	1	1	1	0.1	0.1	0.1	0	0	0
Paraguay	Paraguarí	0.5	0.4	0.6	1	1	2	0.1	0.1	0.1	0	0	0
Paraguay	Paso de Patria	0.5	0.4	0.7	0	0	0	0.1	0.1	0.1	0	0	0
Paraguay	Pedro Juan Caballero	0.5	0.4	0.6	5	4	7	0.1	0.1	0.1	1	1	1

Table 3: Diarrhoea Mortality by Unit *(continued)*

Country	Unit	2000 mortality						2017 mortality					
		mean rate (per 10,000)	lower rate (per 10,000)	upper rate (per 10,000)	mean count	lower count	upper count	mean rate (per 10,000)	lower rate (per 10,000)	upper rate (per 10,000)	mean count	lower count	upper count
Paraguay	Pilar	0.5	0.4	0.7	1	1	2	0.1	0.1	0.1	0	0	0
Paraguay	Pirapó	0.5	0.4	0.6	0	0	1	0.1	0.1	0.1	0	0	0
Paraguay	Pirayú	0.5	0.4	0.6	1	1	1	0.1	0.1	0.1	0	0	0
Paraguay	Piribebuy	0.5	0.4	0.6	1	1	2	0.1	0.1	0.1	0	0	0
Paraguay	Pozo Colorado	0.6	0.4	0.7	2	1	2	0.1	0.1	0.1	0	0	0
Paraguay	Presidente Franco	0.5	0.4	0.6	4	3	5	0.1	0.1	0.1	1	1	1
Paraguay	Primero de Marzo	0.5	0.4	0.6	0	0	0	0.1	0.1	0.1	0	0	0
Paraguay	Puerto Pinasco	0.6	0.5	0.7	0	0	0	0.1	0.1	0.1	0	0	0
Paraguay	Quiíndy	0.4	0.3	0.6	1	1	1	0.1	0.1	0.1	0	0	0
Paraguay	Quyquyhó	0.4	0.3	0.6	0	0	0	0.1	0.1	0.1	0	0	0
Paraguay	R. I. 3 Corrales	0.4	0.3	0.5	0	0	1	0.1	0.1	0.1	0	0	0
Paraguay	Raúl Arsenio Oviedo	0.5	0.4	0.6	2	2	3	0.1	0.1	0.1	0	0	0
Paraguay	Repatriación	0.5	0.4	0.6	2	2	3	0.1	0.1	0.1	0	0	0
Paraguay	Salto del Guairá	0.5	0.4	0.7	0	0	1	0.1	0.1	0.1	0	0	0
Paraguay	San Alberto	0.5	0.3	0.6	1	1	1	0.1	0.1	0.1	0	0	0
Paraguay	San Antonio	0.5	0.4	0.6	2	2	3	0.1	0.1	0.1	1	0	1
Paraguay	San Bernardino	0.5	0.4	0.6	1	0	1	0.1	0.1	0.1	0	0	0
Paraguay	San Carlos	0.6	0.5	0.8	0	0	0	0.1	0.1	0.1	0	0	0
Paraguay	San Cosme y Damián	0.5	0.4	0.6	0	0	1	0.1	0.1	0.1	0	0	0
Paraguay	San Cristóbal	0.4	0.3	0.6	0	0	1	0.1	0.1	0.1	0	0	0
Paraguay	San Estanislao	0.5	0.4	0.6	6	5	8	0.1	0.1	0.1	1	1	2
Paraguay	San Ignacio	0.4	0.3	0.5	1	1	1	0.1	0.1	0.1	0	0	0
Paraguay	San Joaquín	0.5	0.4	0.6	1	1	1	0.1	0.1	0.1	0	0	0
Paraguay	San José de los Arroyos	0.4	0.3	0.6	1	1	1	0.1	0.1	0.1	0	0	0
Paraguay	San José Obrero	0.5	0.4	0.6	0	0	0	0.1	0.1	0.1	0	0	0
Paraguay	San Juan Bautista de las Misiones	0.4	0.3	0.6	1	1	1	0.1	0.1	0.1	0	0	0
Paraguay	San Juan Bautista del Ñeembucu	0.5	0.4	0.6	0	0	0	0.1	0.1	0.1	0	0	0
Paraguay	San Juan del Paraná	0.5	0.4	0.6	0	0	1	0.1	0.1	0.1	0	0	0
Paraguay	San Juan Nepomuceno	0.4	0.3	0.6	1	1	2	0.1	0.1	0.1	0	0	0
Paraguay	San Lázaro	0.6	0.5	0.8	1	1	1	0.1	0.1	0.1	0	0	0
Paraguay	San Lorenzo	0.5	0.4	0.6	12	9	15	0.1	0.1	0.1	2	2	3
Paraguay	San Miguel	0.4	0.3	0.5	0	0	0	0.1	0.1	0.1	0	0	0
Paraguay	San Pablo	0.5	0.4	0.7	0	0	0	0.1	0.1	0.1	0	0	0
Paraguay	San Patricio	0.4	0.3	0.5	0	0	0	0.1	0.1	0.1	0	0	0
Paraguay	San Pedro del Paraná	0.4	0.3	0.6	2	1	2	0.1	0.1	0.1	0	0	0
Paraguay	San Pedro del Ycuamandyyú	0.5	0.4	0.6	2	2	3	0.1	0.1	0.1	0	0	0
Paraguay	San Rafael del Paraná	0.5	0.4	0.6	1	1	2	0.1	0.1	0.1	0	0	0
Paraguay	San Roque González de Santa Cruz	0.4	0.3	0.6	1	0	1	0.1	0.1	0.1	0	0	0
Paraguay	San Salvador	0.4	0.3	0.6	0	0	0	0.1	0.1	0.1	0	0	0
Paraguay	Santa Elena	0.4	0.3	0.5	0	0	0	0.1	0.1	0.1	0	0	0
Paraguay	Santa María	0.4	0.3	0.5	0	0	0	0.1	0.1	0.1	0	0	0
Paraguay	Santa Rita	0.4	0.3	0.5	1	1	1	0.1	0.1	0.1	0	0	0
Paraguay	Santa Rosa	0.4	0.3	0.5	1	1	1	0.1	0.1	0.1	0	0	0
Paraguay	Santa Rosa del Mbutuy	0.4	0.3	0.5	1	1	1	0.1	0.1	0.1	0	0	0
Paraguay	Santa Rosa del Monday	0.4	0.3	0.6	1	1	1	0.1	0.1	0.1	0	0	0

		2000 mortality						2017 mortality					
Country	Unit	mean rate (per 10,000)	lower rate (per 10,000)	upper rate (per 10,000)	mean count	lower count	upper count	mean rate (per 10,000)	lower rate (per 10,000)	upper rate (per 10,000)	mean count	lower count	upper count
Paraguay	Santiago	0.4	0.3	0.5	0	0	0	0.1	0.1	0.1	0	0	0
Paraguay	Sapucaí	0.4	0.3	0.6	0	0	0	0.1	0.1	0.1	0	0	0
Paraguay	Simón Bolívar	0.4	0.3	0.6	0	0	0	0.1	0.1	0.1	0	0	0
Paraguay	Tabaí	0.4	0.4	0.6	1	1	1	0.1	0.1	0.1	0	0	0
Paraguay	Tacuaras	0.5	0.4	0.6	0	0	0	0.1	0.1	0.1	0	0	0
Paraguay	Tacuatí	0.5	0.4	0.6	1	1	1	0.1	0.1	0.1	0	0	0
Paraguay	Tebicuarymí	0.5	0.3	0.6	0	0	0	0.1	0.1	0.1	0	0	0
Paraguay	Tobatí	0.5	0.4	0.6	1	1	2	0.1	0.1	0.1	0	0	0
Paraguay	Tomás Romero Pereira	0.4	0.4	0.6	2	1	2	0.1	0.1	0.1	0	0	0
Paraguay	Trinidad	0.5	0.4	0.6	0	0	1	0.1	0.1	0.1	0	0	0
Paraguay	Unión	0.4	0.4	0.6	0	0	1	0.1	0.1	0.1	0	0	0
Paraguay	Valenzuela	0.4	0.3	0.6	0	0	0	0.1	0.1	0.1	0	0	0
Paraguay	Villa del Rosario	0.5	0.4	0.7	1	1	1	0.1	0.1	0.1	0	0	0
Paraguay	Villa Elisa	0.5	0.4	0.7	3	3	4	0.1	0.1	0.1	1	1	1
Paraguay	Villa Florida	0.5	0.4	0.6	0	0	0	0.1	0.1	0.1	0	0	0
Paraguay	Villa Franca	0.5	0.4	0.6	0	0	0	0.1	0.1	0.1	0	0	0
Paraguay	Villa Hayes	0.5	0.4	0.6	3	3	4	0.1	0.1	0.1	1	1	1
Paraguay	Villa Oliva	0.5	0.4	0.6	0	0	0	0.1	0.1	0.1	0	0	0
Paraguay	Villa San Isidro Curuguaty	0.5	0.4	0.6	4	3	5	0.1	0.1	0.1	1	1	1
Paraguay	Villa Ygatimí	0.5	0.4	0.7	1	1	1	0.1	0.1	0.1	0	0	0
Paraguay	Villalbín	0.5	0.4	0.7	0	0	0	0.1	0.1	0.1	0	0	0
Paraguay	Villarrica	0.5	0.4	0.6	3	2	4	0.1	0.1	0.1	1	0	1
Paraguay	Villeta	0.5	0.4	0.6	1	1	2	0.1	0.1	0.1	0	0	0
Paraguay	Water body	0.5	0.4	0.6	0	0	0	0.1	0.1	0.1	0	0	0
Paraguay	Yabebyry	0.5	0.4	0.6	0	0	0	0.1	0.1	0.1	0	0	0
Paraguay	Yaguarón	0.5	0.4	0.6	2	1	2	0.1	0.1	0.1	0	0	0
Paraguay	Yataity del Guairá	0.5	0.4	0.6	0	0	0	0.1	0.1	0.1	0	0	0
Paraguay	Yataity del Norte	0.4	0.3	0.5	1	1	1	0.1	0.1	0.1	0	0	0
Paraguay	Yatytay	0.5	0.4	0.6	1	1	1	0.1	0.1	0.1	0	0	0
Paraguay	Yby Yaù	0.5	0.4	0.6	1	1	2	0.1	0.1	0.1	0	0	0
Paraguay	Ybycui	0.4	0.3	0.6	1	1	1	0.1	0.1	0.1	0	0	0
Paraguay	Ybytimí	0.4	0.3	0.6	0	0	1	0.1	0.1	0.1	0	0	0
Paraguay	Yguazú	0.5	0.4	0.6	1	1	1	0.1	0.1	0.1	0	0	0
Paraguay	Yhú	0.5	0.4	0.6	3	2	4	0.1	0.1	0.1	0	0	1
Paraguay	Ypacaraí	0.5	0.4	0.6	1	1	1	0.1	0.1	0.1	0	0	0
Paraguay	Ypané	0.5	0.4	0.6	2	1	2	0.1	0.1	0.1	0	0	1
Paraguay	Ypejhú	0.5	0.4	0.6	0	0	1	0.1	0.1	0.1	0	0	0
Paraguay	Yuty	0.4	0.3	0.6	1	1	2	0.1	0.1	0.1	0	0	0
Peru	Abancay	0.5	0.4	0.6	6	5	8	0.1	0.1	0.1	1	1	1
Peru	Acobamba	0.5	0.4	0.6	4	3	4	0.1	0.1	0.1	2	1	2
Peru	Acomayo	0.5	0.4	0.6	2	2	3	0.1	0.1	0.1	0	0	0
Peru	Aija	0.5	0.4	0.6	1	0	1	0.1	0.1	0.1	0	0	0
Peru	Alto Amazonas	0.7	0.6	0.8	18	15	22	0.1	0.1	0.2	5	4	6
Peru	Ambo	0.5	0.4	0.6	4	3	5	0.1	0.1	0.1	1	1	1
Peru	Andahuaylas	0.5	0.4	0.6	10	8	11	0.1	0.1	0.1	2	2	3
Peru	Angaraes	0.5	0.4	0.6	4	3	5	0.1	0.1	0.1	1	1	2
Peru	Anta	0.5	0.4	0.6	3	3	4	0.1	0.1	0.1	1	1	1
Peru	Antabamba	0.5	0.4	0.6	1	1	1	0.1	0.1	0.1	0	0	0
Peru	Antonio Raymondi	0.5	0.4	0.6	1	1	2	0.1	0.1	0.1	0	0	0
Peru	Arequipa	0.3	0.3	0.4	27	21	33	0.1	0.1	0.1	10	8	12
Peru	Ascope	0.3	0.2	0.4	4	3	5	0.1	0.1	0.1	1	1	1
Peru	Asunción	0.5	0.4	0.6	1	1	1	0.1	0.1	0.1	0	0	0
Peru	Atalaya	0.6	0.5	0.7	5	4	6	0.2	0.1	0.2	2	2	2
Peru	Ayabaca	0.5	0.4	0.6	12	9	14	0.1	0.1	0.1	2	2	3
Peru	Aymaraes	0.5	0.4	0.6	2	2	2	0.1	0.1	0.1	0	0	0
Peru	Azángaro	0.4	0.3	0.5	8	6	9	0.1	0.1	0.1	1	1	2

Table 3: Diarrhoea Mortality by Unit *(continued)*

Country	Unit	2000 mortality mean rate (per 10,000)	lower rate (per 10,000)	upper rate (per 10,000)	mean count	lower count	upper count	2017 mortality mean rate (per 10,000)	lower rate (per 10,000)	upper rate (per 10,000)	mean count	lower count	upper count
Peru	Bagua	0.6	0.5	0.7	6	5	7	0.1	0.1	0.1	1	1	1
Peru	Barranca	0.4	0.3	0.4	5	4	6	0.1	0.1	0.1	1	1	1
Peru	Bellavista	0.6	0.5	0.7	4	3	5	0.1	0.1	0.1	1	1	1
Peru	Bolívar	0.5	0.4	0.6	1	1	2	0.1	0.1	0.1	0	0	0
Peru	Bolognesi	0.5	0.4	0.6	2	2	2	0.1	0.1	0.1	0	0	0
Peru	Bongará	0.6	0.5	0.7	2	1	2	0.1	0.1	0.1	0	0	1
Peru	Cajabamba	0.4	0.4	0.5	5	4	6	0.1	0.1	0.1	1	1	1
Peru	Cajamarca	0.4	0.3	0.5	15	13	18	0.1	0.1	0.1	4	4	5
Peru	Cajatambo	0.5	0.4	0.6	1	0	1	0.1	0.1	0.1	0	0	0
Peru	Calca	0.5	0.4	0.6	4	3	5	0.1	0.1	0.1	1	1	1
Peru	Callao	0.3	0.2	0.4	18	14	22	0.1	0.1	0.1	9	7	10
Peru	Camaná	0.3	0.3	0.4	2	1	2	0.1	0.1	0.1	1	1	1
Peru	Canas	0.5	0.4	0.6	3	2	4	0.1	0.1	0.1	1	0	1
Peru	Canchis	0.5	0.4	0.6	6	5	7	0.1	0.1	0.1	1	1	1
Peru	Candarave	0.3	0.3	0.4	0	0	0	0.1	0.1	0.1	0	0	0
Peru	Cañete	0.4	0.3	0.4	8	7	10	0.1	0.1	0.1	2	2	3
Peru	Cangallo	0.5	0.4	0.6	2	2	3	0.1	0.1	0.1	0	0	1
Peru	Canta	0.4	0.3	0.5	1	1	1	0.1	0.1	0.1	0	0	0
Peru	Carabaya	0.5	0.4	0.6	4	3	5	0.1	0.1	0.1	2	1	2
Peru	Caravelí	0.4	0.3	0.5	1	1	2	0.1	0.1	0.1	0	0	1
Peru	Carhuaz	0.4	0.4	0.5	2	2	3	0.1	0.1	0.1	1	0	1
Peru	Carlos Fermin Fitzcarrald	0.5	0.4	0.6	1	1	2	0.1	0.1	0.1	0	0	0
Peru	Casma	0.4	0.3	0.5	2	2	3	0.1	0.1	0.1	1	0	1
Peru	Castilla	0.4	0.3	0.5	2	2	2	0.1	0.1	0.1	1	0	1
Peru	Castrovirreyna	0.5	0.4	0.6	1	1	2	0.1	0.1	0.1	0	0	0
Peru	Caylloma	0.4	0.3	0.5	2	2	3	0.1	0.1	0.1	0	0	0
Peru	Celendín	0.5	0.4	0.5	6	5	7	0.1	0.1	0.1	1	1	1
Peru	Chachapoyas	0.6	0.5	0.7	3	3	4	0.1	0.1	0.1	1	1	1
Peru	Chanchamayo	0.6	0.4	0.7	10	8	12	0.1	0.1	0.1	3	3	4
Peru	Chepén	0.3	0.2	0.4	2	2	3	0.1	0.1	0.1	1	1	1
Peru	Chiclayo	0.3	0.3	0.4	27	22	32	0.1	0.1	0.1	7	5	8
Peru	Chincha	0.4	0.3	0.4	8	7	10	0.1	0.1	0.1	2	2	3
Peru	Chincheros	0.5	0.4	0.6	4	3	5	0.1	0.1	0.1	1	1	1
Peru	Chota	0.4	0.4	0.5	10	8	12	0.1	0.1	0.1	2	2	2
Peru	Chucuíto	0.4	0.3	0.5	5	4	6	0.1	0.1	0.1	2	1	2
Peru	Chumbivilcas	0.5	0.4	0.6	6	4	7	0.1	0.1	0.1	1	1	1
Peru	Chupaca	0.4	0.4	0.5	5	4	6	0.1	0.1	0.1	1	1	1
Peru	Churcampa	0.5	0.4	0.6	3	2	4	0.1	0.1	0.1	1	1	1
Peru	Concepción	0.5	0.4	0.6	4	3	5	0.1	0.1	0.1	1	1	1
Peru	Condesuyos	0.4	0.3	0.5	1	1	1	0.1	0.1	0.1	0	0	0
Peru	Condorcanqui	0.7	0.6	0.8	6	5	7	0.1	0.1	0.2	2	1	2
Peru	Contralmirante Villar	0.4	0.4	0.5	1	1	1	0.1	0.1	0.1	0	0	0
Peru	Contumazá	0.4	0.3	0.5	2	2	2	0.1	0.1	0.1	0	0	0
Peru	Coronel Portillo	0.6	0.5	0.8	29	24	35	0.1	0.1	0.2	8	7	10
Peru	Corongo	0.5	0.4	0.6	1	0	1	0.1	0.1	0.1	0	0	0
Peru	Cotabambas	0.5	0.4	0.6	4	3	4	0.1	0.1	0.1	1	1	1
Peru	Cusco	0.5	0.4	0.6	17	13	20	0.1	0.1	0.1	5	4	6
Peru	Cutervo	0.5	0.4	0.6	10	8	12	0.1	0.1	0.1	2	2	2
Peru	Daniel Alcides Carrión	0.5	0.4	0.6	2	2	3	0.1	0.1	0.1	1	1	1
Peru	Dos de Mayo	0.5	0.4	0.6	3	3	4	0.1	0.1	0.1	1	1	1
Peru	El Collao	0.4	0.3	0.5	3	3	4	0.1	0.1	0.1	1	1	1
Peru	El Dorado	0.6	0.5	0.7	3	2	3	0.1	0.1	0.1	1	1	1
Peru	Espinar	0.5	0.4	0.6	4	3	5	0.1	0.1	0.1	1	1	1
Peru	Ferreñafe	0.4	0.3	0.5	5	4	6	0.1	0.1	0.1	1	1	1
Peru	General Sánchez Cerro	0.4	0.3	0.4	1	1	1	0.1	0.1	0.1	0	0	0
Peru	Gran Chimú	0.4	0.3	0.5	2	1	2	0.1	0.1	0.1	0	0	0

Table 3: Diarrhoea Mortality by Unit *(continued)*

Country	Unit	2000 mortality						2017 mortality					
		mean rate (per 10,000)	lower rate (per 10,000)	upper rate (per 10,000)	mean count	lower count	upper count	mean rate (per 10,000)	lower rate (per 10,000)	upper rate (per 10,000)	mean count	lower count	upper count
Peru	Grau	0.5	0.4	0.6	2	2	2	0.1	0.1	0.1	0	0	0
Peru	Huacaybamba	0.5	0.4	0.6	2	1	2	0.1	0.1	0.1	0	0	0
Peru	Hualgayoc	0.4	0.3	0.5	4	4	5	0.1	0.1	0.1	1	1	1
Peru	Huallaga	0.6	0.5	0.7	2	2	2	0.1	0.1	0.1	0	0	0
Peru	Huamalíes	0.5	0.4	0.6	5	4	6	0.1	0.1	0.1	1	1	2
Peru	Huamanga	0.5	0.4	0.6	14	11	16	0.1	0.1	0.1	4	4	5
Peru	Huanca Sancos	0.5	0.4	0.6	1	1	1	0.1	0.1	0.1	0	0	0
Peru	Huancabamba	0.5	0.4	0.6	9	7	11	0.1	0.1	0.1	2	2	2
Peru	Huancane	0.4	0.4	0.5	4	3	4	0.1	0.1	0.1	1	1	1
Peru	Huancavelica	0.5	0.4	0.6	9	7	11	0.1	0.1	0.1	3	2	3
Peru	Huancayo	0.4	0.4	0.5	21	17	25	0.1	0.1	0.1	6	5	6
Peru	Huanta	0.5	0.4	0.7	7	5	8	0.1	0.1	0.1	2	2	3
Peru	Huaral	0.3	0.3	0.4	6	4	7	0.1	0.1	0.1	1	1	2
Peru	Huaraz	0.4	0.4	0.5	7	6	9	0.1	0.1	0.1	2	1	2
Peru	Huari	0.5	0.4	0.6	4	3	5	0.1	0.1	0.1	1	1	1
Peru	Huarmey	0.4	0.3	0.5	1	1	2	0.1	0.1	0.1	0	0	0
Peru	Huarochiri	0.4	0.3	0.5	3	3	4	0.1	0.1	0.1	1	1	1
Peru	Huaura	0.4	0.3	0.4	7	6	9	0.1	0.1	0.1	2	2	2
Peru	Huaylas	0.4	0.4	0.5	3	3	4	0.1	0.1	0.1	1	1	1
Peru	Huaytara	0.5	0.4	0.6	1	1	2	0.1	0.1	0.1	0	0	0
Peru	Huenuco	0.5	0.4	0.6	17	14	20	0.1	0.1	0.1	4	3	5
Peru	Ica	0.4	0.3	0.5	13	10	16	0.1	0.1	0.1	4	3	4
Peru	Ilo	0.3	0.2	0.4	2	1	2	0.1	0.1	0.1	1	0	1
Peru	Islay	0.3	0.2	0.4	1	1	2	0.1	0.1	0.1	0	0	0
Peru	Jaén	0.5	0.4	0.6	12	10	15	0.1	0.1	0.1	2	2	3
Peru	Jauja	0.5	0.4	0.6	6	5	7	0.1	0.1	0.1	1	1	1
Peru	Jorge Basadre	0.3	0.2	0.4	0	0	0	0.0	0.0	0.0	0	0	0
Peru	Julcan	0.4	0.3	0.5	2	2	3	0.1	0.1	0.1	0	0	0
Peru	Junín	0.6	0.4	0.7	2	2	3	0.1	0.1	0.1	0	0	0
Peru	La Convención	0.5	0.4	0.6	12	10	14	0.1	0.1	0.1	3	2	3
Peru	La Mar	0.5	0.4	0.7	7	6	8	0.1	0.1	0.1	2	2	2
Peru	La Unión	0.5	0.4	0.6	1	1	1	0.1	0.1	0.1	0	0	0
Peru	Lago Titicaca	0.4	0.4	0.5	2	2	3	0.1	0.1	0.1	1	0	1
Peru	Lamas	0.6	0.5	0.7	6	5	8	0.1	0.1	0.1	1	1	2
Peru	Lambayeque	0.4	0.3	0.5	13	11	16	0.1	0.1	0.1	3	3	4
Peru	Lampa	0.4	0.3	0.5	2	2	3	0.1	0.1	0.1	1	1	1
Peru	Lauricocha	0.5	0.4	0.6	3	2	3	0.1	0.1	0.1	1	0	1
Peru	Leoncio Prado	0.6	0.5	0.7	9	7	11	0.1	0.1	0.1	2	2	2
Peru	Lima	0.3	0.3	0.4	226	176	276	0.1	0.1	0.1	74	63	86
Peru	Loreto	0.8	0.6	0.9	9	7	10	0.2	0.1	0.2	2	2	3
Peru	Lucanas	0.4	0.4	0.5	4	3	4	0.1	0.1	0.1	1	1	1
Peru	Luya	0.6	0.5	0.7	4	4	5	0.1	0.1	0.1	1	1	1
Peru	Manu	0.5	0.4	0.6	1	1	2	0.1	0.1	0.1	1	0	1
Peru	Marañón	0.5	0.4	0.6	2	2	3	0.1	0.1	0.1	1	1	1
Peru	Mariscal Cáceres	0.6	0.5	0.7	5	4	5	0.1	0.1	0.1	1	1	1
Peru	Mariscal Luzuriaga	0.5	0.4	0.6	2	1	2	0.1	0.1	0.1	0	0	0
Peru	Mariscal Nieto	0.3	0.3	0.4	2	2	3	0.1	0.1	0.1	1	0	1
Peru	Mariscal Ramón Castilla	0.8	0.6	0.9	8	6	10	0.2	0.1	0.2	3	2	3
Peru	Maynas	0.7	0.6	0.9	54	43	66	0.1	0.1	0.2	12	10	15
Peru	Melgar	0.4	0.3	0.5	4	3	5	0.1	0.1	0.1	1	1	1
Peru	Moho	0.5	0.4	0.6	1	1	2	0.1	0.1	0.1	0	0	0
Peru	Morropón	0.5	0.4	0.6	10	8	12	0.1	0.1	0.1	2	1	2
Peru	Moyobamba	0.6	0.5	0.7	8	6	9	0.1	0.1	0.1	3	2	3
Peru	Nazca	0.4	0.3	0.4	2	2	2	0.1	0.1	0.1	1	0	1
Peru	Ocros	0.5	0.4	0.6	0	0	1	0.1	0.1	0.1	0	0	0
Peru	Otuzco	0.4	0.3	0.5	5	4	6	0.1	0.1	0.1	1	1	1
Peru	Oxapampa	0.6	0.5	0.7	6	5	8	0.1	0.1	0.1	2	2	2
Peru	Oyon	0.5	0.4	0.6	1	1	2	0.1	0.1	0.1	0	0	0

Table 3: Diarrhoea Mortality by Unit *(continued)*

Country	Unit	2000 mortality mean rate (per 10,000)	lower rate (per 10,000)	upper rate (per 10,000)	mean count	lower count	upper count	2017 mortality mean rate (per 10,000)	lower rate (per 10,000)	upper rate (per 10,000)	mean count	lower count	upper count
Peru	Pacasmayo	0.3	0.2	0.4	3	3	4	0.1	0.1	0.1	1	1	1
Peru	Pachitea	0.5	0.4	0.6	4	4	5	0.1	0.1	0.1	1	1	1
Peru	Padre Abad	0.6	0.5	0.8	4	4	5	0.1	0.1	0.2	1	1	2
Peru	Paita	0.5	0.4	0.6	6	5	7	0.1	0.1	0.1	2	2	3
Peru	Pallasca	0.5	0.4	0.6	2	2	3	0.1	0.1	0.1	0	0	1
Peru	Palpa	0.4	0.3	0.4	1	0	1	0.1	0.1	0.1	0	0	0
Peru	Parinacochas	0.5	0.4	0.6	2	1	2	0.1	0.1	0.1	1	0	1
Peru	Paruro	0.5	0.4	0.6	2	2	3	0.1	0.1	0.1	0	0	1
Peru	Pasco	0.5	0.4	0.6	9	7	11	0.1	0.1	0.1	2	2	3
Peru	Pataz	0.5	0.4	0.6	6	5	7	0.1	0.1	0.1	1	1	2
Peru	Paucar del Sara Sara	0.5	0.4	0.6	1	0	1	0.1	0.1	0.1	0	0	0
Peru	Paucartambo	0.5	0.4	0.6	4	3	5	0.1	0.1	0.1	1	1	1
Peru	Picota	0.6	0.5	0.7	3	2	3	0.1	0.1	0.1	1	1	1
Peru	Pisco	0.4	0.3	0.5	6	5	7	0.1	0.1	0.1	2	1	2
Peru	Piura	0.4	0.4	0.5	35	28	42	0.1	0.1	0.1	10	8	12
Peru	Pomabamba	0.5	0.4	0.6	2	2	2	0.1	0.1	0.1	0	0	0
Peru	Puerto Inca	0.6	0.5	0.8	3	3	4	0.1	0.1	0.2	1	1	1
Peru	Puno	0.4	0.3	0.5	9	7	10	0.1	0.1	0.1	2	2	3
Peru	Purús	0.6	0.5	0.7	0	0	0	0.1	0.1	0.2	0	0	0
Peru	Quispicanchi	0.5	0.4	0.6	6	5	7	0.1	0.1	0.1	1	1	2
Peru	Recuay	0.5	0.4	0.6	1	1	1	0.1	0.1	0.1	0	0	0
Peru	Requena	0.8	0.7	1.0	10	8	12	0.2	0.1	0.2	2	2	3
Peru	Rioja	0.5	0.5	0.6	7	6	8	0.1	0.1	0.1	2	2	2
Peru	Rodríguez de Mendoza	0.6	0.5	0.7	2	2	2	0.1	0.1	0.1	1	0	1
Peru	San Antonio de Putina	0.4	0.3	0.5	2	2	3	0.1	0.1	0.1	1	1	1
Peru	San Ignacio	0.6	0.5	0.7	11	9	13	0.1	0.1	0.1	2	2	3
Peru	San Marcos	0.5	0.4	0.5	3	3	4	0.1	0.1	0.1	1	1	1
Peru	San Martín	0.5	0.4	0.6	9	8	11	0.1	0.1	0.1	2	2	3
Peru	San Miguel	0.4	0.3	0.5	3	2	4	0.1	0.1	0.1	1	0	1
Peru	San Pablo	0.4	0.3	0.5	2	1	2	0.1	0.1	0.1	0	0	0
Peru	San Román	0.4	0.4	0.5	11	9	13	0.1	0.1	0.1	4	3	4
Peru	Sánchez Carrión	0.5	0.4	0.5	10	8	12	0.1	0.1	0.1	2	2	3
Peru	Sandia	0.5	0.4	0.5	4	3	4	0.1	0.1	0.1	1	1	1
Peru	Santa	0.4	0.3	0.5	15	12	18	0.1	0.1	0.1	4	3	5
Peru	Santa Cruz	0.4	0.3	0.5	2	2	3	0.1	0.1	0.1	0	0	1
Peru	Santiago de Chuco	0.4	0.4	0.5	4	3	5	0.1	0.1	0.1	1	1	1
Peru	Satipo	0.6	0.5	0.7	13	11	16	0.1	0.1	0.2	7	6	8
Peru	Sechura	0.5	0.4	0.5	5	4	6	0.1	0.1	0.1	1	1	2
Peru	Sihuas	0.5	0.4	0.6	2	2	3	0.1	0.1	0.1	0	0	1
Peru	Sucre	0.5	0.4	0.6	1	1	1	0.1	0.1	0.1	0	0	0
Peru	Sullana	0.4	0.3	0.5	15	12	19	0.1	0.1	0.1	4	3	5
Peru	Tacna	0.3	0.2	0.3	7	5	8	0.1	0.1	0.1	2	2	3
Peru	Tahuamanu	0.5	0.4	0.6	1	0	1	0.1	0.1	0.1	0	0	0
Peru	Talara	0.4	0.3	0.5	7	6	9	0.1	0.1	0.1	2	1	2
Peru	Tambopata	0.5	0.4	0.6	5	4	6	0.1	0.1	0.1	2	1	2
Peru	Tarata	0.3	0.3	0.4	0	0	0	0.1	0.1	0.1	0	0	0
Peru	Tarma	0.5	0.4	0.6	7	5	8	0.1	0.1	0.1	1	1	1
Peru	Tayacaja	0.5	0.4	0.6	8	7	10	0.1	0.1	0.1	2	2	2
Peru	Tocache	0.6	0.5	0.7	5	4	6	0.1	0.1	0.1	1	1	1
Peru	Trujillo	0.3	0.3	0.4	25	20	30	0.1	0.1	0.1	8	7	10
Peru	Tumbes	0.4	0.3	0.5	7	5	8	0.1	0.1	0.1	2	2	2
Peru	Ucayali	0.8	0.6	0.9	8	7	10	0.2	0.1	0.2	2	2	3
Peru	Urubamba	0.5	0.4	0.6	3	3	4	0.1	0.1	0.1	1	1	1
Peru	Utcubamba	0.5	0.5	0.7	9	8	11	0.1	0.1	0.1	2	1	2
Peru	Victor Fajardo	0.5	0.4	0.6	2	1	2	0.1	0.1	0.1	0	0	0
Peru	Vilcas Huamán	0.5	0.4	0.6	2	1	2	0.1	0.1	0.1	0	0	0

Table 3: Diarrhoea Mortality by Unit *(continued)*

Country	Unit	2000 mortality mean rate (per 10,000)	lower rate (per 10,000)	upper rate (per 10,000)	mean count	lower count	upper count	2017 mortality mean rate (per 10,000)	lower rate (per 10,000)	upper rate (per 10,000)	mean count	lower count	upper count
Peru	Viru	0.4	0.3	0.5	3	2	4	0.1	0.1	0.1	2	1	2
Peru	Yarowilca	0.5	0.4	0.6	3	2	3	0.1	0.1	0.1	1	0	1
Peru	Yauli	0.5	0.4	0.6	4	3	5	0.1	0.1	0.1	1	0	1
Peru	Yauyos	0.5	0.4	0.6	2	1	2	0.1	0.1	0.1	0	0	0
Peru	Yungay	0.4	0.4	0.5	3	3	4	0.1	0.1	0.1	1	1	1
Peru	Yunguyo	0.4	0.4	0.5	2	1	2	0.1	0.1	0.1	0	0	0
Peru	Zarumilla	0.4	0.3	0.5	2	2	3	0.1	0.1	0.1	1	1	1
Suriname	Albina	1.2	0.9	1.5	1	0	1	0.3	0.3	0.4	0	0	0
Suriname	Alkmaar	0.8	0.6	1.0	0	0	0	0.3	0.2	0.3	0	0	0
Suriname	Bakki	0.8	0.6	1.1	0	0	0	0.3	0.2	0.3	0	0	0
Suriname	Beekhuizen	0.9	0.7	1.1	2	1	2	0.3	0.2	0.3	0	0	0
Suriname	Bigi Poika	0.9	0.7	1.2	0	0	0	0.3	0.2	0.3	0	0	0
Suriname	Blauwgrond	0.8	0.6	1.0	2	1	2	0.3	0.2	0.3	0	0	1
Suriname	Boven Suriname	1.2	0.9	1.6	3	2	3	0.4	0.3	0.5	1	1	1
Suriname	Brownsweg	1.2	0.9	1.5	0	0	0	0.3	0.3	0.4	0	0	0
Suriname	Bven Saramacca	1.0	0.7	1.3	0	0	0	0.3	0.2	0.4	0	0	0
Suriname	Calcutta	0.8	0.6	1.1	0	0	0	0.3	0.2	0.3	0	0	0
Suriname	Carolina	1.0	0.8	1.3	0	0	0	0.3	0.3	0.4	0	0	0
Suriname	Centrum	1.1	0.8	1.4	0	0	0	0.3	0.3	0.4	0	0	0
Suriname	Centrum	0.8	0.6	1.1	2	1	2	0.3	0.2	0.3	0	0	1
Suriname	Coeroeni	1.2	0.9	1.6	0	0	0	0.4	0.3	0.5	0	0	0
Suriname	Coppename	1.0	0.8	1.2	0	0	0	0.3	0.3	0.4	0	0	0
Suriname	De Nieuwe Grond	0.8	0.6	1.0	2	1	2	0.3	0.2	0.3	1	1	1
Suriname	Domburg	0.8	0.6	1.1	0	0	0	0.3	0.2	0.3	0	0	0
Suriname	Flora	0.8	0.6	1.0	1	0	1	0.3	0.2	0.3	0	0	0
Suriname	Galibi	0.9	0.7	1.2	0	0	0	0.3	0.2	0.3	0	0	0
Suriname	Groningen	0.8	0.6	1.0	0	0	0	0.2	0.2	0.3	0	0	0
Suriname	Groot Henar	0.6	0.4	0.8	0	0	0	0.2	0.1	0.2	0	0	0
Suriname	Houttuin	0.8	0.6	1.1	2	1	2	0.3	0.2	0.3	1	1	1
Suriname	Jarikaba	0.7	0.5	0.9	0	0	0	0.2	0.2	0.3	0	0	0
Suriname	Johanna Maria	0.7	0.5	0.9	0	0	0	0.2	0.2	0.3	0	0	0
Suriname	Kabalebo	1.1	0.8	1.3	0	0	0	0.4	0.3	0.4	0	0	0
Suriname	Kampong Baroe	0.8	0.6	1.0	0	0	0	0.2	0.2	0.3	0	0	0
Suriname	Klaaskreek	1.1	0.8	1.4	0	0	0	0.3	0.3	0.4	0	0	0
Suriname	Koewarasan	0.7	0.5	1.0	1	1	2	0.2	0.2	0.3	1	1	1
Suriname	Kwakoegron	1.1	0.8	1.4	0	0	0	0.3	0.3	0.4	0	0	0
Suriname	Kwatta	0.7	0.5	0.9	0	0	1	0.2	0.2	0.3	0	0	0
Suriname	Latour	0.8	0.6	1.1	1	1	1	0.3	0.2	0.3	0	0	0
Suriname	Lelydorp	0.8	0.6	1.0	1	1	2	0.3	0.2	0.3	1	0	1
Suriname	Livorno	0.8	0.6	1.1	1	1	2	0.3	0.2	0.3	0	0	1
Suriname	Marechalskreek	1.2	0.9	1.5	0	0	0	0.3	0.3	0.4	0	0	0
Suriname	Margaretha	0.8	0.6	1.0	0	0	0	0.3	0.2	0.3	0	0	0
Suriname	Meerzorg	0.8	0.7	1.1	1	1	2	0.3	0.2	0.4	1	0	1
Suriname	Moengo	1.0	0.8	1.3	1	1	2	0.3	0.2	0.4	0	0	0
Suriname	Moengotapoe	1.1	0.9	1.4	0	0	0	0.3	0.3	0.4	0	0	0
Suriname	Munder	0.7	0.6	1.0	1	0	1	0.2	0.2	0.3	0	0	0
Suriname	Noord	0.9	0.7	1.2	1	0	1	0.3	0.2	0.4	0	0	0
Suriname	Nw . Nickerie	0.6	0.4	0.8	0	0	0	0.2	0.1	0.2	0	0	0
Suriname	Nw. Amsterdam	0.8	0.6	1.0	0	0	1	0.3	0.2	0.3	0	0	0
Suriname	Oost	0.9	0.7	1.2	1	1	1	0.3	0.2	0.4	0	0	0
Suriname	Oostelijke Polders	0.6	0.4	0.8	0	0	1	0.2	0.1	0.2	0	0	0
Suriname	Patamacca	1.2	0.9	1.5	0	0	0	0.3	0.3	0.4	0	0	0
Suriname	Pontbuiten	0.8	0.6	1.0	1	1	1	0.3	0.2	0.3	0	0	0
Suriname	Rainville	0.8	0.6	1.0	1	1	1	0.3	0.2	0.3	0	0	0
Suriname	Sarakreek	1.1	0.9	1.4	1	1	1	0.3	0.3	0.4	0	0	0
Suriname	Saramaccapolder	0.7	0.5	1.0	1	0	1	0.2	0.2	0.3	0	0	0
Suriname	Tamanredjo	0.8	0.6	1.1	0	0	0	0.3	0.2	0.3	0	0	0
Suriname	Tammenga	0.7	0.6	1.0	0	0	1	0.2	0.2	0.3	0	0	0
Suriname	Tapanahony	1.3	1.0	1.7	3	2	4	0.4	0.3	0.5	1	1	1
Suriname	Tijgerkreek	0.7	0.6	1.0	0	0	0	0.2	0.2	0.3	0	0	0

Country	Unit	2000 mortality mean rate (per 10,000)	lower rate (per 10,000)	upper rate (per 10,000)	mean count	lower count	upper count	2017 mortality mean rate (per 10,000)	lower rate (per 10,000)	upper rate (per 10,000)	mean count	lower count	upper count
Suriname	Totness	0.8	0.6	1.0	0	0	0	0.3	0.2	0.3	0	0	0
Suriname	Wageningen	0.7	0.5	0.8	0	0	0	0.2	0.2	0.3	0	0	0
Suriname	Wanhatti	1.1	0.8	1.4	0	0	0	0.3	0.3	0.4	0	0	0
Suriname	Wayambo Weg	0.7	0.5	1.0	0	0	0	0.2	0.2	0.3	0	0	0
Suriname	Weg Naar Zee	0.8	0.6	1.0	1	0	1	0.2	0.2	0.3	0	0	0
Suriname	Welgelegen	0.7	0.6	1.0	0	0	0	0.3	0.2	0.3	0	0	0
Suriname	Welgelegen	0.9	0.7	1.2	2	2	3	0.3	0.2	0.4	0	0	1
Suriname	Westelijke Polders	0.6	0.4	0.8	1	1	1	0.2	0.2	0.2	0	0	0
Suriname	Zuid	1.0	0.8	1.3	1	0	1	0.3	0.2	0.4	0	0	0
**North Africa and Middle East**													
Afghanistan	Ab Band	2.4	1.2	4.2	17	9	29	0.7	0.4	1.2	3	2	5
Afghanistan	Ab Kamari	3.5	1.7	5.6	15	7	25	0.7	0.4	1.2	6	3	10
Afghanistan	Achin	3.5	1.8	5.8	56	29	93	1.1	0.6	2.0	29	16	51
Afghanistan	Adraskan	2.8	1.6	4.6	22	12	36	1.0	0.6	1.7	14	8	25
Afghanistan	Ajristan	2.5	1.3	4.5	15	8	27	0.7	0.4	1.2	3	1	5
Afghanistan	Alasay	3.2	1.7	5.9	16	8	29	0.9	0.5	1.5	6	3	10
Afghanistan	Ali abad	3.4	1.8	6.0	40	21	69	1.0	0.6	1.7	11	6	17
Afghanistan	Alingar	3.1	1.6	5.2	51	26	85	1.1	0.6	1.7	30	17	49
Afghanistan	Alishing	3.1	1.6	5.3	34	18	58	1.0	0.6	1.7	19	11	31
Afghanistan	Almar	3.3	1.7	5.4	44	23	73	1.0	0.5	1.6	22	12	37
Afghanistan	Anar Dara	2.8	1.6	4.7	14	8	23	1.0	0.5	1.4	8	5	13
Afghanistan	Andar	2.3	1.2	4.0	61	31	104	0.7	0.4	1.2	12	6	19
Afghanistan	Andarab	3.4	1.7	5.9	37	19	66	0.8	0.4	1.5	12	6	22
Afghanistan	Andkhoy	3.2	1.8	5.3	25	14	41	1.0	0.5	1.7	12	7	21
Afghanistan	Aqcha	3.6	1.8	5.8	54	27	87	0.9	0.5	1.5	14	8	26
Afghanistan	Arghandab	3.0	1.5	4.9	29	15	48	1.4	0.7	2.3	20	11	34
Afghanistan	Arghandab	3.1	1.6	4.8	30	16	47	0.8	0.5	1.4	10	6	18
Afghanistan	Arghistan	3.0	1.7	5.1	16	9	27	1.1	0.6	1.8	10	6	16
Afghanistan	Asad abad	3.0	1.6	5.3	34	18	60	1.0	0.6	1.7	19	11	31
Afghanistan	Atghar	2.9	1.5	5.2	6	3	11	0.9	0.5	1.6	3	1	5
Afghanistan	Aybak	3.1	1.7	5.3	44	24	75	0.9	0.5	1.4	19	11	30
Afghanistan	Azro	3.0	1.6	5.0	9	5	15	1.0	0.5	1.7	5	3	9
Afghanistan	Baghlan City	3.6	1.9	6.6	34	18	63	0.9	0.5	1.6	14	7	24
Afghanistan	Baghlani Jadid	3.5	1.9	6.0	43	23	75	0.9	0.5	1.5	13	7	21
Afghanistan	Bagram	3.4	1.6	5.8	86	41	146	1.0	0.5	1.7	29	16	50
Afghanistan	Bagrami	3.3	1.7	5.8	231	117	401	1.1	0.6	1.9	121	64	211
Afghanistan	Baharak	3.3	1.8	5.8	36	20	64	1.0	0.6	1.7	21	12	35
Afghanistan	Bak	3.6	1.9	6.1	11	6	19	0.7	0.3	1.1	5	3	9
Afghanistan	Bakwa	3.0	1.6	5.4	18	9	32	0.8	0.5	1.4	9	5	14
Afghanistan	Bala Buluk	2.9	1.6	4.9	33	18	57	0.9	0.5	1.5	18	10	31
Afghanistan	Balkh	3.3	1.8	5.5	75	41	125	1.0	0.6	1.8	28	15	48
Afghanistan	Balkhab	3.0	1.4	5.2	24	11	41	1.0	0.6	1.6	11	7	17
Afghanistan	Bamyan City	3.1	1.6	5.6	37	18	66	0.8	0.4	1.4	14	8	25
Afghanistan	Bangi	3.4	1.8	6.1	21	11	38	1.2	0.8	2.1	12	8	21
Afghanistan	Bar Kunar	3.0	1.6	5.2	17	9	29	1.0	0.6	1.8	9	5	16
Afghanistan	Baraki Barak	3.1	1.6	5.5	34	18	60	0.9	0.5	1.5	16	8	25
Afghanistan	Bargi Matal	3.1	1.7	5.4	11	6	20	1.0	0.6	1.8	6	3	10
Afghanistan	Bati Kot	3.6	1.8	6.1	38	19	64	1.3	0.6	2.2	22	11	38
Afghanistan	Bilchiragh	3.2	1.6	5.4	64	32	108	1.1	0.6	1.9	37	21	65
Afghanistan	Bughran	4.1	2.2	6.8	53	29	90	1.1	0.6	1.8	20	10	33
Afghanistan	Burka	3.4	1.9	6.0	24	13	42	1.0	0.6	1.7	10	5	16
Afghanistan	Burmul	3.1	1.6	5.7	20	10	36	0.7	0.4	1.1	6	3	10
Afghanistan	Chaghcharan	2.7	1.4	4.3	83	44	129	1.2	0.8	1.8	62	38	92
Afghanistan	Chah Ab	3.5	1.8	6.1	36	18	62	1.5	0.9	2.3	25	15	38
Afghanistan	Chahar Asyab	3.3	1.7	6.0	42	22	76	1.1	0.6	1.9	25	14	46
Afghanistan	Chaharikar	3.4	1.5	6.1	96	43	170	0.9	0.5	1.7	33	18	58
Afghanistan	Chak	2.8	1.5	4.8	28	15	48	0.9	0.5	1.5	15	7	24
Afghanistan	Chakhansur	2.9	1.6	4.9	13	7	22	0.9	0.5	1.3	5	3	7
Afghanistan	Chal	3.4	1.7	5.8	15	8	25	1.2	0.7	2.0	9	5	15

Country	Unit	2000 mortality mean rate (per 10,000)	lower rate (per 10,000)	upper rate (per 10,000)	mean count	lower count	upper count	2017 mortality mean rate (per 10,000)	lower rate (per 10,000)	upper rate (per 10,000)	mean count	lower count	upper count
Afghanistan	Chamkani	3.1	1.7	5.0	23	12	37	0.8	0.4	1.4	9	5	16
Afghanistan	Chapa Dara	3.1	1.6	4.8	13	7	20	1.0	0.6	1.6	7	4	11
Afghanistan	Chaparhar	3.6	1.9	6.4	29	16	53	1.2	0.7	2.0	16	10	27
Afghanistan	Char Bolak	3.3	1.8	5.6	36	20	60	1.0	0.6	1.7	12	7	22
Afghanistan	Char Burjak	2.8	1.6	4.7	14	8	22	0.9	0.5	1.3	5	3	7
Afghanistan	Char Dara	3.4	1.7	5.4	48	24	77	0.9	0.5	1.6	13	7	22
Afghanistan	Charkh	3.0	1.6	5.6	26	14	49	0.9	0.5	1.5	11	6	19
Afghanistan	Charkint	3.2	1.6	5.2	25	12	41	1.0	0.5	1.7	9	5	15
Afghanistan	Chawkay	3.1	1.6	5.4	20	10	34	1.1	0.6	1.7	11	6	17
Afghanistan	Chimtal	3.3	1.8	5.5	42	23	71	1.0	0.6	1.7	15	9	26
Afghanistan	Chishti Sharif	2.9	1.6	4.9	9	5	16	1.0	0.6	1.6	6	3	10
Afghanistan	Chora	3.1	1.6	5.3	51	26	88	1.0	0.6	1.6	25	15	39
Afghanistan	Dahana-I-Ghori	3.3	1.8	6.1	19	10	36	0.9	0.5	1.5	7	4	11
Afghanistan	Daman	3.0	1.5	5.0	59	30	99	1.3	0.7	2.1	55	30	88
Afghanistan	Dand Wa Patan	3.2	1.8	5.2	11	6	18	0.8	0.4	1.4	4	2	8
Afghanistan	Dangam	2.8	1.5	4.9	13	7	22	1.0	0.5	1.7	7	4	13
Afghanistan	Dara-I-Nur	3.1	1.6	5.3	20	10	34	1.1	0.6	1.8	12	6	19
Afghanistan	Dara-i-Pech	3.1	1.7	5.0	24	13	40	1.0	0.6	1.6	13	8	21
Afghanistan	Dara-i-Suf	3.1	1.6	5.4	61	31	105	1.0	0.5	1.6	27	14	43
Afghanistan	Darqad	3.6	1.8	6.1	20	10	33	1.6	1.0	2.4	15	10	22
Afghanistan	Darwaz	3.3	1.9	5.5	26	15	43	0.9	0.5	1.5	13	8	22
Afghanistan	Darzab	3.4	1.8	5.7	37	19	61	1.1	0.6	1.8	13	8	22
Afghanistan	Dashti Archi	3.4	1.8	5.9	57	29	98	1.2	0.7	2.2	19	12	36
Afghanistan	Dawlat abad	3.3	1.8	5.4	26	15	43	1.1	0.6	1.8	14	8	24
Afghanistan	Dawlat abad	3.3	1.9	5.3	65	37	107	1.0	0.6	1.7	23	13	38
Afghanistan	Dawlat Shah	3.0	1.5	5.2	20	10	34	0.9	0.5	1.4	10	5	16
Afghanistan	Day Kundi	2.9	1.5	5.0	109	55	184	0.9	0.5	1.4	49	29	74
Afghanistan	Daychopan	2.9	1.6	4.9	40	22	67	0.9	0.5	1.3	13	8	21
Afghanistan	Daymirdad	2.7	1.3	5.1	9	4	16	0.9	0.5	1.5	5	2	8
Afghanistan	Dih Bala	3.4	1.9	5.8	25	14	43	1.1	0.6	2.0	13	7	24
Afghanistan	Dih Sabz	3.3	1.7	5.7	417	210	726	1.1	0.6	1.8	203	108	353
Afghanistan	Dihdadi	3.4	1.8	5.7	51	28	87	1.0	0.6	1.8	19	10	32
Afghanistan	Dihrawud	3.2	1.7	5.5	57	31	100	1.2	0.7	1.9	31	18	51
Afghanistan	Dihyak	2.5	1.3	4.6	23	12	43	0.8	0.4	1.3	4	2	7
Afghanistan	Dila	2.8	1.4	4.8	13	6	22	0.8	0.4	1.3	5	3	8
Afghanistan	Disho	4.4	2.5	7.1	13	8	21	0.9	0.5	1.4	4	2	6
Afghanistan	Doshi	3.4	1.8	5.8	32	17	54	0.9	0.5	1.5	11	6	19
Afghanistan	Dur Baba	3.5	1.8	6.2	15	7	26	1.1	0.6	2.0	8	4	14
Afghanistan	Farah City	3.0	1.5	5.3	41	21	74	0.9	0.5	1.4	22	12	36
Afghanistan	Farkhar	3.3	1.7	5.6	27	15	47	1.2	0.7	1.9	16	10	26
Afghanistan	Farsi	2.8	1.4	4.6	10	5	16	1.0	0.6	1.7	7	4	12
Afghanistan	Fayz abad	3.5	1.9	5.8	23	12	38	0.9	0.5	1.7	7	4	12
Afghanistan	Fayzabad	3.3	1.7	6.0	99	52	180	1.2	0.7	1.9	71	40	109
Afghanistan	Gardez	2.9	1.4	5.1	39	19	70	0.9	0.5	1.4	18	10	30
Afghanistan	Garmser	4.4	2.5	7.3	52	30	87	0.7	0.4	1.1	12	7	19
Afghanistan	Gayan	3.1	1.7	6.0	17	9	33	0.6	0.3	1.1	5	3	8
Afghanistan	Gelan	2.5	1.3	4.3	24	12	41	0.7	0.4	1.2	4	3	7
Afghanistan	Ghazni	2.3	1.1	3.9	67	33	116	0.8	0.4	1.3	13	7	22
Afghanistan	Ghorak	3.3	1.7	5.5	5	3	9	1.2	0.7	2.0	3	2	5
Afghanistan	Ghorband	3.3	1.7	5.3	58	29	94	0.9	0.5	1.6	20	10	35
Afghanistan	Ghormach	3.4	1.7	6.3	25	13	48	0.7	0.4	1.1	10	6	16
Afghanistan	Ghoryan	2.9	1.6	4.6	26	15	41	1.1	0.6	1.6	19	10	29
Afghanistan	Giro	2.4	1.2	4.2	16	8	29	0.7	0.4	1.2	3	2	5
Afghanistan	Gizab	3.1	1.5	5.2	33	17	56	0.8	0.5	1.4	12	7	20
Afghanistan	Gomal	3.1	1.7	5.1	5	3	9	0.7	0.4	1.1	2	1	3
Afghanistan	Goshta	3.4	1.7	5.7	11	6	19	1.2	0.7	2.0	7	4	11
Afghanistan	Gul dara	3.3	1.7	5.5	15	8	25	1.0	0.5	1.6	7	4	11
Afghanistan	Gulistan	2.9	1.7	4.5	20	11	30	0.8	0.4	1.3	10	6	16
Afghanistan	Gulran	2.9	1.6	5.0	31	18	54	1.0	0.5	1.6	21	11	33
Afghanistan	Gurbuz	3.7	1.9	6.2	13	7	21	0.6	0.3	1.0	5	3	10

Table 3: Diarrhoea Mortality by Unit *(continued)*

Country	Unit	2000 mortality mean rate (per 10,000)	2000 mortality lower rate (per 10,000)	2000 mortality upper rate (per 10,000)	2000 mortality mean count	2000 mortality lower count	2000 mortality upper count	2017 mortality mean rate (per 10,000)	2017 mortality lower rate (per 10,000)	2017 mortality upper rate (per 10,000)	2017 mortality mean count	2017 mortality lower count	2017 mortality upper count
Afghanistan	Guzara	2.8	1.4	5.1	50	25	88	1.1	0.6	1.8	37	20	62
Afghanistan	Hazrati Imam	3.4	1.8	5.9	133	70	229	1.0	0.6	1.6	37	22	61
Afghanistan	Hazrati Sultan	3.2	1.6	5.7	21	11	37	0.9	0.5	1.4	8	4	13
Afghanistan	Hirat City	2.8	1.3	5.7	31	14	63	1.1	0.6	1.9	19	10	34
Afghanistan	Hisa-i-Awali Bihsud	2.9	1.3	5.6	9	4	17	0.8	0.4	1.4	4	2	7
Afghanistan	Hisa-I-Awali Panjsher	3.3	1.6	6.3	20	9	37	0.7	0.4	1.1	5	3	8
Afghanistan	Hisa-I-Duwum Panjsher	3.3	1.6	5.7	9	4	16	0.7	0.4	1.2	3	1	4
Afghanistan	Hisarak	3.2	1.7	5.4	16	9	28	1.0	0.6	1.8	9	5	15
Afghanistan	Injil	2.8	1.4	5.3	209	102	394	1.1	0.6	1.9	154	82	271
Afghanistan	Ishkamish	3.4	1.8	6.1	32	17	57	1.1	0.6	1.9	16	8	26
Afghanistan	Ishkashim	3.1	1.7	5.4	6	3	10	0.9	0.5	1.4	3	2	5
Afghanistan	Istalif	3.3	1.7	5.3	12	6	20	1.0	0.5	1.6	5	3	9
Afghanistan	Jabalussaraj	3.4	1.7	6.1	51	25	90	0.9	0.5	1.5	16	9	28
Afghanistan	Jadran	3.0	1.5	5.6	15	8	28	0.7	0.4	1.2	5	3	9
Afghanistan	Jaghatu	2.3	1.2	3.9	60	31	102	0.8	0.4	1.3	16	9	27
Afghanistan	Jaghuri	2.3	1.2	4.1	66	35	115	0.7	0.4	1.2	12	7	21
Afghanistan	Jaji	3.0	1.6	4.8	25	14	41	0.9	0.5	1.5	11	6	19
Afghanistan	Jaji Maydan	3.5	2.0	5.9	10	5	17	0.7	0.4	1.3	5	2	8
Afghanistan	Jalal abad	3.5	1.8	6.2	121	62	213	1.3	0.7	2.1	68	37	112
Afghanistan	Jalrez	2.9	1.4	5.0	15	7	26	0.9	0.5	1.5	8	4	13
Afghanistan	Jani Khel	3.2	1.7	5.3	25	14	42	0.7	0.4	1.2	10	5	16
Afghanistan	Jawand	3.2	1.9	5.2	42	25	67	0.9	0.5	1.3	23	14	35
Afghanistan	Jurm	3.2	1.7	5.6	37	19	65	1.1	0.6	1.7	23	13	38
Afghanistan	Kabul City	3.3	1.6	5.7	830	415	1440	1.1	0.6	1.8	432	230	746
Afghanistan	Kahmard	3.2	1.7	5.5	31	17	53	0.9	0.5	1.4	12	7	21
Afghanistan	Kajaki	4.1	2.1	7.0	44	22	74	1.0	0.6	1.8	15	8	25
Afghanistan	Kalafgan	3.4	1.9	5.8	31	17	53	1.3	0.7	2.0	18	10	28
Afghanistan	Kalakan	3.3	1.7	5.6	10	5	17	1.0	0.6	1.7	5	3	9
Afghanistan	Kaldar	3.3	1.8	6.3	14	8	26	0.9	0.5	1.6	6	3	10
Afghanistan	Kama	3.5	1.8	5.8	33	17	55	1.3	0.7	2.1	19	10	31
Afghanistan	Kamdesh	3.1	1.6	5.5	16	8	29	1.1	0.6	1.8	9	5	15
Afghanistan	Kandahar City	3.0	1.6	5.1	223	116	378	1.4	0.7	2.2	153	82	244
Afghanistan	Kang	2.9	1.5	5.0	11	6	20	0.8	0.4	1.3	4	2	7
Afghanistan	Karukh	2.8	1.5	4.7	25	13	40	0.9	0.5	1.6	15	8	26
Afghanistan	Khaki Jabar	3.2	1.7	5.5	6	3	9	1.0	0.6	1.7	3	2	5
Afghanistan	Khaki Safed	2.9	1.5	5.0	10	5	17	0.9	0.5	1.5	5	3	9
Afghanistan	Khakrez	3.0	1.5	5.1	8	4	14	1.3	0.7	2.1	6	3	10
Afghanistan	Khamyab	3.4	2.0	6.0	6	3	11	0.9	0.4	1.4	2	1	3
Afghanistan	Khan Abad	3.4	1.8	6.2	81	42	147	1.1	0.7	1.8	27	16	44
Afghanistan	Khan Char Bagh	3.2	1.8	5.3	16	9	26	1.0	0.5	1.6	7	4	12
Afghanistan	Khas Kunar	3.2	1.7	5.5	15	8	25	1.1	0.6	1.8	8	5	14
Afghanistan	Khas Uruzgan	3.0	1.5	4.9	41	20	67	0.9	0.5	1.4	17	9	27
Afghanistan	Khash Rod	2.8	1.5	4.8	17	9	29	0.8	0.4	1.4	6	3	10
Afghanistan	Khinjan	3.3	1.7	5.6	17	9	29	0.9	0.5	1.5	6	3	11
Afghanistan	Khogyani	3.4	1.8	5.8	59	32	101	1.1	0.6	1.8	30	17	50
Afghanistan	Khost (Matun)	3.7	1.9	6.2	54	28	90	0.6	0.3	1.1	21	11	39
Afghanistan	Khost Wa Firing	3.3	1.7	5.6	40	21	67	1.0	0.6	1.6	16	9	26
Afghanistan	Khulm	3.4	1.8	6.1	45	23	81	0.9	0.5	1.5	15	8	24
Afghanistan	Khuram Wa Sarbagh	3.2	1.7	5.6	26	14	47	0.9	0.5	1.5	10	6	16
Afghanistan	Khushi	3.1	1.6	5.6	10	5	18	1.0	0.5	1.5	4	2	7
Afghanistan	Khwahan	3.3	1.8	5.5	13	7	22	1.1	0.6	1.7	8	5	13
Afghanistan	Khwaja Du Koh	3.5	2.0	5.8	13	7	21	0.9	0.5	1.6	4	2	6
Afghanistan	Khwaja Ghar	3.5	1.8	6.3	43	23	77	1.4	0.9	2.3	27	17	45
Afghanistan	Khwaja Sabz Posh	3.3	1.6	5.5	66	31	111	1.1	0.7	2.0	38	22	69
Afghanistan	Kijran	3.3	1.8	5.7	24	13	42	1.0	0.5	1.6	10	6	17

Table 3: Diarrhoea Mortality by Unit *(continued)*

Country	Unit	2000 mortality mean rate (per 10,000)	lower rate (per 10,000)	upper rate (per 10,000)	mean count	lower count	upper count	2017 mortality mean rate (per 10,000)	lower rate (per 10,000)	upper rate (per 10,000)	mean count	lower count	upper count
Afghanistan	Kishim	3.2	1.7	5.4	51	27	87	1.2	0.7	1.9	36	20	59
Afghanistan	Kishindih	3.2	1.5	5.5	51	25	89	1.1	0.6	1.8	21	12	36
Afghanistan	Kohband	3.5	1.8	6.2	13	7	23	0.8	0.5	1.4	4	2	7
Afghanistan	Kohi Safi	3.4	1.7	5.8	21	11	37	1.0	0.5	1.6	8	4	13
Afghanistan	Kohistan	3.5	1.8	6.1	72	37	124	0.9	0.5	1.4	23	13	39
Afghanistan	Kohistan	3.1	1.6	4.9	27	14	43	1.1	0.6	1.8	16	9	26
Afghanistan	Kohistanat	2.9	1.6	4.6	39	21	61	1.1	0.7	1.7	20	12	30
Afghanistan	Kuhsan	2.9	1.5	4.8	25	13	41	1.0	0.6	1.6	16	9	25
Afghanistan	Kunduz	3.4	1.8	5.7	179	98	303	1.0	0.6	1.7	53	30	88
Afghanistan	Kuran Wa Munjan	3.0	1.6	5.2	3	2	6	0.9	0.6	1.6	2	1	3
Afghanistan	Kushk	2.9	1.5	5.0	44	23	77	0.9	0.5	1.5	27	14	44
Afghanistan	Kushki Kuhna	3.0	1.6	4.9	16	9	27	0.8	0.5	1.4	9	5	16
Afghanistan	Kuz Kunar	3.3	1.8	5.5	26	14	44	1.2	0.7	1.9	15	8	25
Afghanistan	Lal Pur	3.5	2.0	6.3	14	8	25	1.2	0.6	2.1	7	4	13
Afghanistan	Lal Wa Sarjangal	2.8	1.4	4.8	40	20	68	1.0	0.6	1.7	25	14	41
Afghanistan	Lash Wa Juwayn	2.9	1.5	5.0	15	8	26	0.9	0.5	1.5	8	5	13
Afghanistan	Lashkargah	4.4	2.2	7.9	77	39	139	0.6	0.3	1.0	16	9	27
Afghanistan	Lija Mangal	3.0	1.5	4.9	22	12	37	0.8	0.4	1.5	10	5	18
Afghanistan	Mahmud Raqi	3.5	1.8	5.8	33	17	55	0.9	0.5	1.4	12	7	20
Afghanistan	Malistan	2.4	1.3	4.1	27	15	47	0.7	0.4	1.2	4	2	8
Afghanistan	Mando Zayi	3.6	1.9	6.4	26	14	46	0.6	0.3	1.0	11	6	18
Afghanistan	Mandol	3.1	1.7	5.5	18	10	32	0.8	0.4	1.3	7	4	12
Afghanistan	Mardyan	3.6	1.8	5.9	24	12	40	0.9	0.5	1.5	7	4	12
Afghanistan	Markazi Bihsud	2.8	1.3	5.3	34	16	64	0.8	0.4	1.3	14	8	25
Afghanistan	Marmul	3.4	1.6	5.7	4	2	6	1.0	0.5	1.7	1	1	2
Afghanistan	Maruf	2.9	1.6	5.2	16	9	29	0.9	0.5	1.5	8	4	13
Afghanistan	Marwara	3.1	1.6	5.5	10	5	18	1.0	0.6	1.7	5	3	8
Afghanistan	Mata Khan	2.9	1.5	5.4	14	7	25	0.8	0.4	1.2	5	3	8
Afghanistan	Maydan Shahr	3.1	1.5	5.7	13	6	24	1.0	0.5	1.7	8	4	12
Afghanistan	Maymana	3.3	1.6	5.6	22	11	36	1.2	0.6	2.1	13	7	22
Afghanistan	Maywand	3.3	1.7	5.9	29	15	51	0.9	0.5	1.5	13	7	22
Afghanistan	Mazar-i-Sharif	3.4	1.7	6.0	189	96	333	1.0	0.5	1.7	64	34	111
Afghanistan	Mihtarlam	3.2	1.7	5.4	62	32	106	1.1	0.6	1.8	39	22	61
Afghanistan	Mingajik	3.6	2.0	5.7	20	11	32	0.9	0.5	1.3	5	3	8
Afghanistan	Mirbacha Kot	3.3	1.7	5.9	22	11	40	1.0	0.5	1.7	11	6	19
Afghanistan	Mizan	3.1	1.4	5.5	7	3	12	1.0	0.5	1.8	3	2	5
Afghanistan	Muhammad Agha	3.3	1.7	5.8	29	14	50	1.0	0.6	1.7	13	7	22
Afghanistan	Muhmand Dara	3.6	2.0	6.3	22	12	38	1.2	0.6	2.2	12	6	21
Afghanistan	Muqur	2.3	1.3	3.9	22	12	38	0.7	0.4	1.2	4	2	7
Afghanistan	Muqur	3.6	1.8	6.3	10	5	17	0.6	0.3	1.0	3	2	5
Afghanistan	Murghab	3.5	1.9	5.6	50	28	80	0.6	0.4	1.0	18	11	29
Afghanistan	Musa Khel	3.3	1.8	5.5	10	5	16	0.7	0.4	1.1	5	3	8
Afghanistan	Musa Qala	4.4	2.3	7.2	37	19	60	0.9	0.5	1.4	11	5	17
Afghanistan	Musayi	3.3	1.7	5.6	13	7	22	1.0	0.6	1.8	7	4	12
Afghanistan	Nad Ali	4.4	2.4	7.7	65	36	114	0.6	0.3	1.0	13	7	21
Afghanistan	Nadir Shah Kot	3.4	1.8	6.0	22	12	38	0.6	0.3	1.0	10	6	17
Afghanistan	Nahri Sarraj	4.3	2.3	8.2	76	40	144	0.7	0.4	1.1	17	9	27
Afghanistan	Nahri Shahi	3.4	1.7	5.9	81	41	139	1.0	0.6	1.7	30	17	51
Afghanistan	Nahrin	3.4	1.7	5.9	46	23	79	1.0	0.5	1.7	17	9	29
Afghanistan	Nali	3.0	1.6	5.2	14	7	24	1.0	0.6	1.7	8	4	12
Afghanistan	Narang Wa Badil	3.1	1.6	5.5	18	9	32	1.0	0.6	1.7	9	5	15
Afghanistan	Naw Zad	4.2	2.3	7.0	25	13	41	0.8	0.4	1.2	6	4	10
Afghanistan	Nawa	2.6	1.3	4.5	21	11	37	0.8	0.4	1.3	6	3	9
Afghanistan	Nawa-i-Barak Zayi	4.4	2.2	8.1	49	25	89	0.6	0.3	0.9	8	4	13
Afghanistan	Nawur	2.4	1.3	4.2	47	26	81	0.7	0.4	1.2	9	6	16
Afghanistan	Nazyan	3.5	1.8	6.1	8	4	14	1.2	0.6	2.0	4	2	7
Afghanistan	Nesh	3.0	1.6	5.1	10	6	18	1.3	0.7	1.9	6	4	10
Afghanistan	Nijrab	3.4	1.7	6.2	57	29	104	0.8	0.5	1.4	19	10	31

Table 3: Diarrhoea Mortality by Unit *(continued)*

Country	Unit	2000 mortality mean rate (per 10,000)	2000 lower rate (per 10,000)	2000 upper rate (per 10,000)	2000 mean count	2000 lower count	2000 upper count	2017 mortality mean rate (per 10,000)	2017 lower rate (per 10,000)	2017 upper rate (per 10,000)	2017 mean count	2017 lower count	2017 upper count
Afghanistan	Nika	2.9	1.4	5.5	7	3	13	0.7	0.4	1.2	2	1	4
Afghanistan	Nirkh	3.0	1.4	5.5	28	13	52	1.0	0.6	1.6	16	9	25
Afghanistan	Nurgal	3.2	1.5	5.2	20	10	33	1.2	0.6	1.8	12	6	18
Afghanistan	Nuristan	3.2	1.6	5.3	28	15	47	0.9	0.5	1.6	12	7	22
Afghanistan	Obe	2.9	1.5	5.1	27	14	47	0.9	0.5	1.6	16	8	27
Afghanistan	Omna	3.0	1.6	5.3	6	3	11	0.7	0.4	1.3	2	1	4
Afghanistan	Pachir Wa Agam	3.4	1.8	5.9	19	10	33	1.1	0.6	2.0	10	5	17
Afghanistan	Paghman	3.2	1.6	5.9	99	50	178	1.1	0.5	1.8	55	28	92
Afghanistan	Panjab	3.1	1.6	5.6	26	14	48	0.8	0.5	1.3	11	7	19
Afghanistan	Panjsher	3.4	1.7	6.1	23	12	41	0.8	0.4	1.2	6	3	10
Afghanistan	Panjwayi	3.0	1.6	5.2	83	44	143	1.2	0.7	1.9	54	30	84
Afghanistan	Pasaband	2.8	1.5	4.6	38	20	62	1.2	0.7	1.9	26	15	43
Afghanistan	Pashtun Kot	3.2	1.6	5.2	65	33	105	1.1	0.6	2.0	38	22	69
Afghanistan	Pashtun Zarghun	2.9	1.5	4.8	34	17	56	1.0	0.5	1.7	23	11	38
Afghanistan	Puli Alam	3.2	1.7	5.5	39	20	68	1.0	0.5	1.6	18	10	30
Afghanistan	Puli Khumri	3.5	1.8	6.6	103	55	195	0.9	0.5	1.6	37	19	63
Afghanistan	Purchaman	2.9	1.7	4.4	26	15	40	1.0	0.6	1.6	16	9	25
Afghanistan	Pusht Rod	2.9	1.4	5.1	34	16	58	0.9	0.5	1.4	18	9	29
Afghanistan	Qadis	3.6	2.0	6.3	42	23	73	0.7	0.4	1.1	16	9	28
Afghanistan	Qala-I-Kah	2.9	1.6	5.1	20	11	35	0.9	0.5	1.4	11	6	17
Afghanistan	Qala-I-Naw	3.6	1.9	6.2	48	25	84	0.7	0.4	1.2	18	9	31
Afghanistan	Qala-I-Zal	3.4	1.8	5.5	42	22	68	0.9	0.5	1.3	10	6	16
Afghanistan	Qalandar	3.3	1.8	5.4	5	3	9	0.6	0.3	1.1	3	1	4
Afghanistan	Qalat	3.1	1.6	5.5	19	10	34	0.8	0.4	1.4	7	4	12
Afghanistan	Qarabagh	3.4	1.7	5.7	26	13	44	1.0	0.6	1.7	12	7	21
Afghanistan	Qarabagh	2.2	1.2	3.8	69	36	120	0.7	0.4	1.2	13	7	22
Afghanistan	Qaram Qol	3.2	1.9	5.2	26	16	44	1.0	0.6	1.8	16	9	28
Afghanistan	Qarghayi	3.3	1.7	5.6	60	32	103	1.2	0.7	1.9	37	22	61
Afghanistan	Qarqin	3.5	2.0	5.7	14	8	23	0.9	0.5	1.4	4	2	6
Afghanistan	Qaysar	3.2	1.6	5.5	69	35	120	0.9	0.5	1.4	31	18	51
Afghanistan	Ragh	3.3	1.8	6.1	31	16	56	1.2	0.7	2.0	20	13	33
Afghanistan	Reg	3.2	2.0	5.2	5	3	9	0.9	0.5	1.6	3	1	4
Afghanistan	Reg	4.4	2.5	7.8	30	17	52	0.7	0.4	1.2	6	3	11
Afghanistan	Rodat	3.6	1.9	6.1	117	63	201	1.3	0.7	2.1	69	38	117
Afghanistan	Royi Du Ab	3.1	1.6	5.5	20	11	35	1.0	0.5	1.5	9	5	14
Afghanistan	Rustaq	3.5	1.8	6.2	67	36	119	1.4	0.8	2.3	46	27	74
Afghanistan	Sabari	3.6	1.9	5.9	29	15	48	0.6	0.3	1.1	12	7	22
Afghanistan	Saghar	2.8	1.5	4.4	18	9	28	1.1	0.7	1.8	13	7	20
Afghanistan	Salang	3.3	1.7	5.9	15	8	26	0.8	0.5	1.4	4	2	7
Afghanistan	Sangcharak	2.9	1.5	4.9	65	33	109	1.0	0.5	1.6	29	16	47
Afghanistan	Sangin	4.2	2.1	7.2	45	23	78	0.9	0.5	1.5	14	7	24
Afghanistan	Sar Hawza	3.0	1.5	5.2	13	7	23	0.7	0.4	1.2	5	3	7
Afghanistan	Sar-i-Pul City	3.1	1.7	5.0	82	45	134	0.9	0.5	1.6	32	19	53
Afghanistan	Sarobi	3.0	1.5	5.4	8	4	13	0.7	0.4	1.2	2	1	4
Afghanistan	Sayid Abad	2.8	1.5	4.8	41	21	71	0.9	0.5	1.5	23	12	38
Afghanistan	Sayid Karam	2.9	1.5	4.9	43	23	72	0.9	0.5	1.5	20	11	33
Afghanistan	Sayyad	3.1	1.7	5.0	25	14	40	1.0	0.5	1.6	10	6	17
Afghanistan	Shah Wali Kot	3.0	1.7	4.7	25	14	40	1.3	0.7	2.0	18	10	28
Afghanistan	Shahidi Hasas	3.3	1.8	5.9	40	22	73	1.1	0.6	1.7	20	12	32
Afghanistan	Shahjoy	3.0	1.4	5.0	26	12	44	0.8	0.4	1.3	9	5	16
Afghanistan	Shahrak	2.7	1.5	4.6	43	23	72	1.2	0.7	1.9	31	17	49
Afghanistan	Shahri Buzurg	3.4	1.8	5.9	19	10	32	1.4	0.8	2.3	14	8	23
Afghanistan	Shahristan	2.9	1.5	5.0	46	23	78	0.7	0.5	1.2	16	10	26
Afghanistan	Shakar Dara	3.3	1.6	5.7	70	35	122	1.1	0.5	1.7	32	17	53
Afghanistan	Shamul	3.2	1.7	5.8	4	2	7	0.6	0.3	1.1	2	1	3
Afghanistan	Shamul zayi	2.8	1.5	4.7	12	6	20	0.9	0.5	1.4	5	3	8
Afghanistan	Sharan	2.8	1.4	5.4	32	16	61	0.7	0.4	1.2	11	7	19
Afghanistan	Shekh Ali	3.3	1.5	5.8	14	7	26	0.9	0.4	1.5	5	2	8
Afghanistan	Sherzad	3.3	1.7	5.3	39	21	64	1.1	0.6	1.8	21	11	36
Afghanistan	Shib Koh	3.0	1.5	5.4	3	2	6	0.9	0.5	1.5	2	1	4

Table 3: Diarrhoea Mortality by Unit *(continued)*

Country	Unit	2000 mortality mean rate (per 10,000)	lower rate (per 10,000)	upper rate (per 10,000)	mean count	lower count	upper count	2017 mortality mean rate (per 10,000)	lower rate (per 10,000)	upper rate (per 10,000)	mean count	lower count	upper count
Afghanistan	Shibar	3.2	1.6	5.6	12	6	22	0.8	0.4	1.4	4	2	8
Afghanistan	Shibirghan	3.6	2.0	5.6	100	55	157	0.9	0.5	1.5	30	17	50
Afghanistan	Shighnan	3.2	1.7	5.5	14	7	24	0.9	0.5	1.4	8	5	12
Afghanistan	Shindand	2.8	1.6	4.4	66	37	104	0.9	0.5	1.7	42	21	73
Afghanistan	Shinkay	3.0	1.6	5.3	9	5	15	0.9	0.5	1.4	3	2	6
Afghanistan	Shinwar	3.6	1.8	6.2	37	18	64	1.2	0.6	2.1	20	10	34
Afghanistan	Shinwari	3.3	1.7	5.4	13	6	21	0.9	0.5	1.5	4	2	7
Afghanistan	Shirin Tagab	3.3	1.6	5.5	53	25	87	1.1	0.6	1.9	31	17	53
Afghanistan	Sholgara	3.3	1.7	5.7	69	35	119	1.0	0.6	1.8	26	13	44
Afghanistan	Shorabak	3.0	1.6	5.0	5	3	9	1.0	0.5	1.7	3	1	5
Afghanistan	Shortepa	3.2	1.9	5.4	24	14	40	1.0	0.6	1.6	9	5	15
Afghanistan	Shwak	2.9	1.5	5.3	3	2	6	0.7	0.4	1.3	1	1	2
Afghanistan	Sirkanay	3.1	1.6	5.7	18	9	32	1.0	0.6	1.7	10	5	16
Afghanistan	Sozma Qala	3.0	1.5	5.1	25	13	44	1.0	0.5	1.5	10	5	16
Afghanistan	Spera	3.3	1.7	6.0	10	5	19	0.6	0.3	1.0	4	3	7
Afghanistan	Spin Boldak	3.0	1.5	4.9	51	26	84	1.1	0.6	1.9	30	17	53
Afghanistan	Surkh Rod	3.5	1.9	6.3	75	41	135	1.3	0.7	2.1	43	25	71
Afghanistan	Surkhi Parsa	3.2	1.6	5.4	20	10	33	0.9	0.5	1.5	7	4	11
Afghanistan	Surobi	3.3	1.6	5.5	26	13	44	1.0	0.6	1.7	13	7	22
Afghanistan	Tagab	3.3	1.7	5.8	43	22	74	1.0	0.5	1.6	17	9	29
Afghanistan	Tala Wa Barfak	3.3	1.7	5.5	14	7	24	0.9	0.4	1.5	5	2	8
Afghanistan	Taluqan	3.5	1.7	6.7	117	57	225	1.4	0.8	2.3	75	46	126
Afghanistan	Tani	3.5	1.9	6.4	25	13	46	0.6	0.3	1.0	10	5	17
Afghanistan	Tarnak Wa Jaldak	3.1	1.6	5.4	10	5	18	1.0	0.6	1.8	5	3	8
Afghanistan	Taywara	2.8	1.5	5.0	31	16	56	1.2	0.6	2.0	23	12	38
Afghanistan	Tere Zayi	3.7	1.9	6.5	29	15	50	0.6	0.3	1.1	11	6	21
Afghanistan	Tirin Kot	3.1	1.7	5.7	74	40	138	1.2	0.7	2.1	45	26	75
Afghanistan	Tulak	2.7	1.5	4.8	23	12	40	1.1	0.7	1.7	16	9	24
Afghanistan	Urgun	3.0	1.5	5.6	31	16	58	0.7	0.4	1.2	10	5	17
Afghanistan	Wakhan	2.9	1.8	4.4	5	3	8	0.8	0.5	1.3	3	2	4
Afghanistan	Wama	3.1	1.6	5.3	20	10	33	0.9	0.5	1.5	9	5	15
Afghanistan	Waras	3.1	1.6	5.1	47	25	78	0.8	0.4	1.2	17	10	29
Afghanistan	Warsaj	3.1	1.7	5.3	19	10	32	1.0	0.5	1.7	10	5	17
Afghanistan	Washer	3.9	2.3	6.7	11	6	18	0.7	0.4	1.1	3	1	4
Afghanistan	Waygal	3.1	1.7	5.3	15	8	26	1.0	0.6	1.7	8	4	13
Afghanistan	Wazakhwa	3.0	1.6	4.9	13	7	22	0.8	0.4	1.3	5	3	8
Afghanistan	Wolmamay	3.1	1.7	4.9	2	1	3	0.8	0.4	1.3	1	0	1
Afghanistan	Yakawlang	3.2	1.7	5.3	39	21	65	0.9	0.5	1.5	16	10	27
Afghanistan	Yangi Qala	3.6	1.9	6.2	76	40	132	1.5	0.9	2.4	53	32	81
Afghanistan	Zana Khan	2.6	1.3	4.6	6	3	12	0.8	0.5	1.5	1	1	2
Afghanistan	Zaranj	3.0	1.5	5.6	31	16	57	0.8	0.5	1.4	11	6	17
Afghanistan	Zarghun Shahr	2.9	1.5	5.0	36	19	62	0.8	0.4	1.3	13	8	22
Afghanistan	Zebak	3.0	1.6	5.4	1	1	3	0.9	0.5	1.4	1	1	2
Afghanistan	Ziluk	3.1	1.5	5.8	10	5	19	0.7	0.4	1.1	3	2	6
Afghanistan	Zinda Jan	2.9	1.6	5.3	18	10	33	1.1	0.6	1.9	13	7	21
Afghanistan	Zurmat	2.9	1.5	5.2	55	28	98	0.8	0.4	1.3	23	12	38
Algeria	Abadla	0.3	0.2	0.4	0	0	1	0.0	0.0	0.1	0	0	0
Algeria	Abalissa	0.2	0.2	0.3	0	0	0	0.0	0.0	0.0	0	0	0
Algeria	Abi Youcef	0.2	0.1	0.3	0	0	0	0.0	0.0	0.1	0	0	0
Algeria	Abou El Hassen	0.3	0.2	0.4	1	0	1	0.0	0.0	0.0	0	0	0
Algeria	Achaacha	0.3	0.2	0.5	1	1	2	0.0	0.0	0.1	0	0	0
Algeria	Adekar	0.3	0.2	0.4	0	0	0	0.0	0.0	0.1	0	0	0
Algeria	Adrar	0.3	0.2	0.5	2	1	2	0.0	0.0	0.1	0	0	0
Algeria	Afir	0.2	0.1	0.3	0	0	0	0.0	0.0	0.0	0	0	0
Algeria	Aflou	0.4	0.2	0.5	3	2	4	0.0	0.0	0.1	1	1	1
Algeria	Aghbal	0.3	0.2	0.4	0	0	0	0.0	0.0	0.1	0	0	0
Algeria	Aghbalou	0.2	0.1	0.3	0	0	1	0.0	0.0	0.1	0	0	0
Algeria	Aghlal	0.2	0.1	0.4	0	0	0	0.0	0.0	0.1	0	0	0
Algeria	Aghni-Goughrane	0.2	0.1	0.3	0	0	0	0.0	0.0	0.0	0	0	0

Table 3: Diarrhoea Mortality by Unit *(continued)*

Country	Unit	2000 mortality mean rate (per 10,000)	lower rate (per 10,000)	upper rate (per 10,000)	mean count	lower count	upper count	2017 mortality mean rate (per 10,000)	lower rate (per 10,000)	upper rate (per 10,000)	mean count	lower count	upper count
Algeria	Aghrib	0.2	0.1	0.3	0	0	0	0.0	0.0	0.1	0	0	0
Algeria	Ahl El Ksar	0.2	0.2	0.4	0	0	1	0.0	0.0	0.1	0	0	0
Algeria	Ahmed Rachedi	0.3	0.2	0.5	1	0	1	0.0	0.0	0.1	0	0	0
Algeria	Ahmer El Ain	0.2	0.1	0.4	1	1	1	0.0	0.0	0.1	0	0	0
Algeria	Ahnif	0.2	0.1	0.3	0	0	0	0.0	0.0	0.1	0	0	0
Algeria	Ain Abessa	0.3	0.2	0.5	1	0	1	0.0	0.0	0.1	0	0	0
Algeria	Ain Abid	0.3	0.2	0.5	1	1	1	0.0	0.0	0.1	0	0	0
Algeria	Ain Adden	0.3	0.2	0.5	0	0	0	0.0	0.0	0.1	0	0	0
Algeria	Ain Arnat	0.3	0.2	0.4	1	1	2	0.0	0.0	0.1	0	0	0
Algeria	Ain Azel	0.3	0.2	0.4	2	1	3	0.0	0.0	0.1	0	0	0
Algeria	Ain Bebouche	0.3	0.2	0.4	1	0	1	0.0	0.0	0.1	0	0	0
Algeria	Ain Beida	0.3	0.2	0.4	1	1	1	0.0	0.0	0.1	0	0	0
Algeria	Ain Beida	0.3	0.2	0.4	2	1	3	0.0	0.0	0.1	0	0	1
Algeria	Ain Beida Harriche	0.3	0.2	0.5	1	0	1	0.0	0.0	0.1	0	0	0
Algeria	Ain Ben Beida	0.3	0.2	0.4	0	0	0	0.0	0.0	0.1	0	0	0
Algeria	Ain Ben Khelil	0.3	0.2	0.4	0	0	0	0.0	0.0	0.1	0	0	0
Algeria	Ain Benian	0.3	0.2	0.4	0	0	0	0.0	0.0	0.0	0	0	0
Algeria	Ain Benian	0.3	0.2	0.4	1	1	2	0.0	0.0	0.1	0	0	0
Algeria	Ain Biya	0.3	0.2	0.5	1	1	2	0.0	0.0	0.1	0	0	0
Algeria	Ain Bouchekif	0.3	0.2	0.4	1	0	1	0.0	0.0	0.1	0	0	0
Algeria	Ain Boucif	0.3	0.2	0.4	1	0	1	0.0	0.0	0.0	0	0	0
Algeria	Ain Boudinar	0.3	0.2	0.5	0	0	0	0.0	0.0	0.1	0	0	0
Algeria	Ain Bouihi	0.3	0.2	0.4	1	0	1	0.0	0.0	0.1	0	0	0
Algeria	Ain Bouziane	0.3	0.2	0.4	0	0	0	0.0	0.0	0.1	0	0	0
Algeria	Ain Charchar	0.3	0.2	0.4	0	0	1	0.0	0.0	0.1	0	0	0
Algeria	Ain Chouhada	0.4	0.3	0.6	0	0	1	0.0	0.0	0.1	0	0	0
Algeria	Ain Defla	0.3	0.2	0.5	2	1	3	0.0	0.0	0.1	0	0	0
Algeria	Ain Deheb	0.3	0.2	0.5	1	1	2	0.0	0.0	0.1	0	0	0
Algeria	Ain Djasser	0.3	0.2	0.4	1	0	1	0.0	0.0	0.1	0	0	0
Algeria	Ain El Arbaa	0.2	0.1	0.4	0	0	1	0.0	0.0	0.1	0	0	0
Algeria	Ain El Assel	0.3	0.2	0.4	0	0	0	0.0	0.0	0.1	0	0	0
Algeria	Ain El Berd	0.3	0.2	0.4	0	0	1	0.0	0.0	0.1	0	0	0
Algeria	Ain El Berda	0.3	0.2	0.4	1	0	1	0.0	0.0	0.1	0	0	0
Algeria	Ain El Diss	0.3	0.2	0.4	0	0	0	0.0	0.0	0.1	0	0	0
Algeria	Ain El Fakroun	0.3	0.2	0.5	2	1	3	0.0	0.0	0.1	0	0	0
Algeria	Ain El Hadid	0.3	0.2	0.4	1	0	1	0.0	0.0	0.1	0	0	0
Algeria	Ain El Hadjar	0.3	0.2	0.4	1	1	2	0.0	0.0	0.1	0	0	0
Algeria	Ain El Hadjar	0.2	0.2	0.4	0	0	0	0.0	0.0	0.1	0	0	0
Algeria	Ain El Hadjel	0.3	0.2	0.4	1	1	2	0.0	0.0	0.0	0	0	0
Algeria	Ain El Ibel	0.4	0.2	0.6	1	1	2	0.0	0.0	0.1	0	0	0
Algeria	Ain El Kebira	0.3	0.2	0.4	1	1	2	0.0	0.0	0.1	0	0	0
Algeria	Ain El Kercha	0.3	0.2	0.4	1	1	2	0.0	0.0	0.1	0	0	0
Algeria	Ain El Melh	0.3	0.2	0.4	1	1	2	0.0	0.0	0.0	0	0	0
Algeria	Ain El Orak	0.3	0.2	0.5	0	0	0	0.0	0.0	0.1	0	0	0
Algeria	Ain Errich	0.3	0.2	0.4	1	0	1	0.0	0.0	0.0	0	0	0
Algeria	Ain Fares	0.3	0.2	0.5	1	0	1	0.0	0.0	0.1	0	0	0
Algeria	Ain Fares	0.3	0.2	0.4	0	0	0	0.0	0.0	0.0	0	0	0
Algeria	Ain Fekan	0.3	0.2	0.5	0	0	1	0.0	0.0	0.1	0	0	0
Algeria	Ain Fekka	0.4	0.3	0.6	1	1	2	0.0	0.0	0.1	0	0	0
Algeria	Ain Ferah	0.3	0.2	0.4	0	0	0	0.0	0.0	0.1	0	0	0
Algeria	Ain Fettah	0.3	0.2	0.4	0	0	0	0.0	0.0	0.1	0	0	0
Algeria	Ain Fezza	0.2	0.1	0.4	0	0	1	0.0	0.0	0.1	0	0	0
Algeria	Ain Frass	0.3	0.2	0.5	0	0	0	0.0	0.0	0.1	0	0	0
Algeria	Ain Ghoraba	0.3	0.2	0.4	0	0	0	0.0	0.0	0.1	0	0	0
Algeria	Ain Kada	0.2	0.2	0.4	0	0	0	0.0	0.0	0.1	0	0	0
Algeria	Ain Kebira	0.3	0.2	0.4	0	0	0	0.0	0.0	0.1	0	0	0
Algeria	Ain Kechra	0.3	0.2	0.4	1	0	1	0.0	0.0	0.1	0	0	0
Algeria	Ain Kerma	0.3	0.2	0.4	0	0	1	0.0	0.0	0.1	0	0	0
Algeria	Ain Kerma	0.2	0.1	0.4	1	0	1	0.0	0.0	0.1	0	0	0

Table 3: Diarrhoea Mortality by Unit *(continued)*

Country	Unit	2000 mortality mean rate (per 10,000)	lower rate (per 10,000)	upper rate (per 10,000)	mean count	lower count	upper count	2017 mortality mean rate (per 10,000)	lower rate (per 10,000)	upper rate (per 10,000)	mean count	lower count	upper count
Algeria	Ain Kermes	0.3	0.2	0.5	1	0	1	0.0	0.0	0.1	0	0	0
Algeria	Ain Khadra	0.3	0.2	0.4	1	0	1	0.0	0.0	0.0	0	0	0
Algeria	Ain Kihel	0.2	0.1	0.4	0	0	1	0.0	0.0	0.1	0	0	0
Algeria	Ain Lahdjar	0.3	0.2	0.4	1	1	2	0.0	0.0	0.1	0	0	0
Algeria	Ain Laloui	0.2	0.2	0.4	0	0	0	0.0	0.0	0.1	0	0	0
Algeria	Ain Larbi	0.3	0.2	0.4	0	0	0	0.0	0.0	0.1	0	0	0
Algeria	Ain Lechiakh	0.3	0.2	0.5	1	0	1	0.0	0.0	0.1	0	0	0
Algeria	Ain M'Lila	0.3	0.2	0.4	3	2	4	0.0	0.0	0.1	0	0	1
Algeria	Ain Maabed	0.4	0.2	0.5	1	1	1	0.0	0.0	0.1	0	0	0
Algeria	Ain Madhi	0.3	0.2	0.4	0	0	0	0.0	0.0	0.0	0	0	0
Algeria	Ain Makhlouf	0.3	0.2	0.4	0	0	1	0.0	0.0	0.1	0	0	0
Algeria	Ain Mellouk	0.3	0.2	0.5	1	0	1	0.0	0.0	0.1	0	0	0
Algeria	Ain Merrane	0.3	0.2	0.4	1	1	2	0.0	0.0	0.1	0	0	0
Algeria	Ain Naga	0.3	0.2	0.4	0	0	1	0.0	0.0	0.1	0	0	0
Algeria	Ain Nehala	0.2	0.2	0.4	0	0	0	0.0	0.0	0.1	0	0	0
Algeria	Ain Nouissy	0.3	0.2	0.4	0	0	1	0.0	0.0	0.1	0	0	0
Algeria	Ain Ouksir	0.3	0.2	0.4	0	0	0	0.0	0.0	0.0	0	0	0
Algeria	Ain Oulmane	0.3	0.2	0.5	2	2	4	0.0	0.0	0.1	0	0	1
Algeria	Ain Oussera	0.4	0.2	0.6	5	3	8	0.0	0.0	0.1	1	0	1
Algeria	Ain Rahma	0.3	0.2	0.5	1	0	1	0.0	0.0	0.1	0	0	0
Algeria	Ain Rekada	0.3	0.2	0.4	0	0	0	0.0	0.0	0.1	0	0	0
Algeria	Ain Romana	0.2	0.2	0.3	1	0	1	0.0	0.0	0.0	0	0	0
Algeria	Ain Roua	0.3	0.2	0.5	0	0	1	0.0	0.0	0.1	0	0	0
Algeria	Ain Safra	0.3	0.2	0.4	1	1	2	0.0	0.0	0.1	0	0	1
Algeria	Ain Sandel	0.3	0.2	0.4	0	0	0	0.0	0.0	0.1	0	0	0
Algeria	Ain Sekhouna	0.3	0.2	0.4	0	0	0	0.0	0.0	0.1	0	0	0
Algeria	Ain Semara	0.3	0.2	0.4	2	1	2	0.0	0.0	0.1	0	0	1
Algeria	Ain Sidi Ali	0.4	0.2	0.5	0	0	0	0.0	0.0	0.1	0	0	0
Algeria	Ain Sidi Cherif	0.3	0.2	0.5	0	0	1	0.0	0.0	0.1	0	0	0
Algeria	Ain Soltane	0.3	0.2	0.4	0	0	0	0.0	0.0	0.1	0	0	0
Algeria	Ain Soltane	0.3	0.2	0.5	0	0	0	0.0	0.0	0.1	0	0	0
Algeria	Ain Soltane	0.3	0.2	0.5	1	0	1	0.0	0.0	0.1	0	0	0
Algeria	Ain Taghrout	0.3	0.2	0.5	0	0	1	0.0	0.0	0.1	0	0	0
Algeria	Ain Tagourait	0.3	0.2	0.4	1	0	1	0.0	0.0	0.1	0	0	0
Algeria	Ain Tallout	0.3	0.2	0.4	0	0	1	0.0	0.0	0.1	0	0	0
Algeria	Ain Tarek	0.3	0.2	0.4	0	0	1	0.0	0.0	0.1	0	0	0
Algeria	Ain Tedles	0.3	0.2	0.5	1	1	2	0.0	0.0	0.1	0	0	0
Algeria	Ain Temouchent	0.2	0.1	0.4	1	1	2	0.0	0.0	0.1	0	0	0
Algeria	Ain Tesra	0.3	0.2	0.5	0	0	1	0.0	0.0	0.1	0	0	0
Algeria	Ain Thrid	0.2	0.2	0.4	0	0	1	0.0	0.0	0.1	0	0	0
Algeria	Ain Tindamine	0.3	0.2	0.4	0	0	0	0.0	0.0	0.1	0	0	0
Algeria	Ain Tine	0.3	0.2	0.5	0	0	0	0.0	0.0	0.1	0	0	0
Algeria	Ain Tolba	0.2	0.1	0.4	0	0	0	0.0	0.0	0.1	0	0	0
Algeria	Ain Tork	0.3	0.2	0.5	0	0	1	0.0	0.0	0.1	0	0	0
Algeria	Ain Touila	0.3	0.2	0.4	1	0	1	0.0	0.0	0.1	0	0	0
Algeria	Ain Touta	0.3	0.2	0.5	2	1	3	0.0	0.0	0.1	0	0	0
Algeria	Ain Turk	0.2	0.1	0.3	0	0	0	0.0	0.0	0.1	0	0	1
Algeria	Ain Turk	0.2	0.2	0.3	0	0	0	0.0	0.0	0.1	0	0	0
Algeria	Ain Yagout	0.3	0.2	0.4	0	0	1	0.0	0.0	0.1	0	0	0
Algeria	Ain Youcef	0.2	0.1	0.4	0	0	1	0.0	0.0	0.1	0	0	0
Algeria	Ain Zaatout	0.3	0.2	0.4	0	0	0	0.0	0.0	0.1	0	0	0
Algeria	Ain Zana	0.3	0.2	0.4	0	0	0	0.0	0.0	0.1	0	0	0
Algeria	Ain Zarit	0.3	0.2	0.5	0	0	0	0.0	0.0	0.1	0	0	0
Algeria	Ain Zerga	0.2	0.2	0.3	1	0	1	0.0	0.0	0.1	0	0	0
Algeria	Ain Zitoun	0.3	0.2	0.4	0	0	0	0.0	0.0	0.1	0	0	0
Algeria	Ain Zouit	0.3	0.2	0.4	0	0	0	0.0	0.0	0.1	0	0	0
Algeria	Ain-Bessem	0.2	0.2	0.4	1	1	1	0.0	0.0	0.1	0	0	0
Algeria	Ain-El-Hammam	0.2	0.1	0.3	0	0	0	0.0	0.0	0.0	0	0	0
Algeria	Ain-Legradj	0.3	0.2	0.5	1	0	1	0.0	0.0	0.1	0	0	0
Algeria	Ain-Sebt	0.3	0.2	0.5	1	0	1	0.0	0.0	0.1	0	0	0

Table 3: Diarrhoea Mortality by Unit *(continued)*

Country	Unit	2000 mortality mean rate (per 10,000)	lower rate (per 10,000)	upper rate (per 10,000)	mean count	lower count	upper count	2017 mortality mean rate (per 10,000)	lower rate (per 10,000)	upper rate (per 10,000)	mean count	lower count	upper count
Algeria	Ain-Zaouia	0.2	0.1	0.3	0	0	1	0.0	0.0	0.1	0	0	0
Algeria	Aissaouia	0.3	0.2	0.4	0	0	0	0.0	0.0	0.1	0	0	0
Algeria	Ait Aggouacha	0.2	0.1	0.3	0	0	0	0.0	0.0	0.0	0	0	0
Algeria	Ait Aissa Mimoun	0.2	0.1	0.3	0	0	0	0.0	0.0	0.1	0	0	0
Algeria	Ait Bouadou	0.2	0.1	0.3	0	0	0	0.0	0.0	0.0	0	0	0
Algeria	Ait Boumehdi	0.2	0.1	0.3	0	0	0	0.0	0.0	0.1	0	0	0
Algeria	Ait Khelili	0.2	0.1	0.3	0	0	0	0.0	0.0	0.1	0	0	0
Algeria	Ait Laaziz	0.2	0.2	0.3	0	0	0	0.0	0.0	0.1	0	0	0
Algeria	Ait Naoual Mezada	0.3	0.2	0.5	0	0	0	0.0	0.0	0.1	0	0	0
Algeria	Ait Oumalou	0.2	0.1	0.3	0	0	0	0.0	0.0	0.1	0	0	0
Algeria	Ait R'Zine	0.2	0.1	0.3	0	0	0	0.0	0.0	0.1	0	0	0
Algeria	Ait Toudert	0.2	0.1	0.3	0	0	0	0.0	0.0	0.1	0	0	0
Algeria	Ait Yahia Moussa	0.2	0.1	0.3	0	0	1	0.0	0.0	0.1	0	0	0
Algeria	Ait-Chaffaa	0.2	0.2	0.4	0	0	0	0.0	0.0	0.1	0	0	0
Algeria	Ait-Mahmoud	0.2	0.1	0.3	0	0	0	0.0	0.0	0.0	0	0	0
Algeria	Ait-Smail	0.3	0.2	0.4	0	0	0	0.0	0.0	0.1	0	0	0
Algeria	Ait-Tizi	0.3	0.2	0.4	0	0	0	0.0	0.0	0.1	0	0	0
Algeria	Ait-Yahia	0.2	0.1	0.3	0	0	0	0.0	0.0	0.1	0	0	0
Algeria	Akabli	0.2	0.1	0.3	0	0	0	0.0	0.0	0.1	0	0	0
Algeria	Akbil	0.2	0.1	0.3	0	0	0	0.0	0.0	0.0	0	0	0
Algeria	Akbou	0.2	0.1	0.3	1	1	1	0.0	0.0	0.1	0	0	0
Algeria	Akerrou	0.3	0.2	0.4	0	0	0	0.0	0.0	0.1	0	0	0
Algeria	Akfadou	0.3	0.2	0.4	0	0	0	0.0	0.0	0.1	0	0	0
Algeria	Alaimia	0.3	0.2	0.5	0	0	0	0.0	0.0	0.1	0	0	0
Algeria	Amalou	0.2	0.2	0.4	0	0	0	0.0	0.0	0.1	0	0	0
Algeria	Amernas	0.2	0.2	0.4	1	0	1	0.0	0.0	0.1	0	0	0
Algeria	Amieur	0.2	0.1	0.4	0	0	1	0.0	0.0	0.1	0	0	0
Algeria	Amirat Arres	0.3	0.2	0.5	1	0	1	0.0	0.0	0.1	0	0	0
Algeria	Amizour	0.2	0.1	0.4	1	0	1	0.0	0.0	0.1	0	0	0
Algeria	Ammal	0.3	0.2	0.4	0	0	1	0.0	0.0	0.1	0	0	0
Algeria	Ammari	0.3	0.2	0.4	0	0	0	0.0	0.0	0.1	0	0	0
Algeria	Ammi Moussa	0.2	0.1	0.4	1	0	1	0.0	0.0	0.1	0	0	0
Algeria	Amoucha	0.3	0.2	0.5	1	0	1	0.0	0.0	0.1	0	0	0
Algeria	Amourah	0.4	0.2	0.5	0	0	1	0.0	0.0	0.0	0	0	0
Algeria	Annaba	0.3	0.2	0.4	5	3	8	0.0	0.0	0.1	1	0	1
Algeria	Aokas	0.3	0.2	0.4	0	0	0	0.0	0.0	0.1	0	0	0
Algeria	Aomar	0.2	0.1	0.3	1	0	1	0.0	0.0	0.1	0	0	0
Algeria	Aoubellil	0.2	0.2	0.4	0	0	0	0.0	0.0	0.1	0	0	0
Algeria	Aouf	0.3	0.2	0.5	0	0	0	0.0	0.0	0.1	0	0	0
Algeria	Aougrout	0.3	0.2	0.4	0	0	1	0.0	0.0	0.1	0	0	0
Algeria	Aoulef	0.3	0.2	0.4	1	0	1	0.0	0.0	0.1	0	0	0
Algeria	Arbaouat	0.3	0.2	0.5	0	0	0	0.0	0.0	0.1	0	0	0
Algeria	Arib	0.3	0.2	0.5	1	1	1	0.0	0.0	0.1	0	0	0
Algeria	Arris	0.3	0.2	0.4	1	0	1	0.0	0.0	0.1	0	0	0
Algeria	Arzew	0.3	0.2	0.5	2	1	3	0.0	0.0	0.1	0	0	0
Algeria	Asfour	0.3	0.2	0.4	0	0	1	0.0	0.0	0.1	0	0	0
Algeria	Assela	0.3	0.2	0.4	0	0	0	0.0	0.0	0.1	0	0	0
Algeria	Assi Youcef	0.2	0.1	0.3	0	0	0	0.0	0.0	0.0	0	0	0
Algeria	Ath Mansour Taourirt	0.2	0.1	0.3	0	0	0	0.0	0.0	0.1	0	0	0
Algeria	Azails	0.3	0.2	0.4	0	0	0	0.0	0.0	0.1	0	0	0
Algeria	Azazga	0.2	0.1	0.3	1	0	1	0.0	0.0	0.1	0	0	0
Algeria	Azil Abdelkader (Metkouak)	0.2	0.2	0.4	1	0	1	0.0	0.0	0.0	0	0	0
Algeria	Azzaba	0.3	0.2	0.4	1	1	2	0.0	0.0	0.1	0	0	0
Algeria	Azzefoun	0.3	0.2	0.4	0	0	0	0.0	0.0	0.1	0	0	0
Algeria	Azziz	0.3	0.2	0.5	0	0	1	0.0	0.0	0.1	0	0	0
Algeria	Azzizia	0.3	0.2	0.4	0	0	0	0.0	0.0	0.1	0	0	0

Table 3: Diarrhoea Mortality by Unit *(continued)*

Country	Unit	2000 mortality						2017 mortality					
		mean rate (per 10,000)	lower rate (per 10,000)	upper rate (per 10,000)	mean count	lower count	upper count	mean rate (per 10,000)	lower rate (per 10,000)	upper rate (per 10,000)	mean count	lower count	upper count
Algeria	Baata	0.3	0.2	0.4	0	0	0	0.0	0.0	0.1	0	0	0
Algeria	Bab El Assa	0.3	0.2	0.4	0	0	0	0.0	0.0	0.1	0	0	0
Algeria	Babar	0.2	0.2	0.3	1	1	1	0.0	0.0	0.1	0	0	0
Algeria	Babor	0.3	0.2	0.5	1	0	1	0.0	0.0	0.1	0	0	0
Algeria	Badredine El Mokrani	0.2	0.2	0.4	0	0	0	0.0	0.0	0.1	0	0	0
Algeria	Baghai	0.3	0.2	0.4	0	0	0	0.0	0.0	0.1	0	0	0
Algeria	Baghlia	0.2	0.1	0.4	1	0	1	0.0	0.0	0.1	0	0	0
Algeria	Baladiet Amor	0.3	0.2	0.4	0	0	1	0.0	0.0	0.1	0	0	0
Algeria	Baraki	0.3	0.2	0.4	3	2	5	0.0	0.0	0.1	1	0	1
Algeria	Barbouche	0.3	0.2	0.5	0	0	0	0.0	0.0	0.1	0	0	0
Algeria	Barika	0.3	0.1	0.4	3	1	4	0.0	0.0	0.1	0	0	1
Algeria	Bathia	0.3	0.2	0.5	0	0	0	0.0	0.0	0.1	0	0	0
Algeria	Batna	0.3	0.2	0.4	7	4	11	0.0	0.0	0.1	1	1	2
Algeria	Bayadha	0.3	0.2	0.5	1	1	2	0.0	0.0	0.1	0	0	0
Algeria	Bazer-Sakra	0.3	0.2	0.4	2	1	2	0.0	0.0	0.1	0	0	0
Algeria	Bechar	0.3	0.2	0.4	5	3	8	0.0	0.0	0.1	1	1	1
Algeria	Bechloul	0.2	0.1	0.3	0	0	0	0.0	0.0	0.1	0	0	0
Algeria	Bedjene	0.3	0.2	0.4	0	0	0	0.0	0.0	0.1	0	0	0
Algeria	Beidha Bordj	0.3	0.2	0.4	1	1	1	0.0	0.0	0.1	0	0	0
Algeria	Bejaia	0.2	0.1	0.4	3	2	5	0.0	0.0	0.1	1	0	1
Algeria	Bekkaria	0.2	0.2	0.3	0	0	0	0.0	0.0	0.1	0	0	0
Algeria	Bekkouche Lakhdar	0.3	0.2	0.4	0	0	1	0.0	0.0	0.1	0	0	0
Algeria	Belaas	0.3	0.2	0.5	0	0	0	0.0	0.0	0.1	0	0	0
Algeria	Belaassel Bouzagza	0.3	0.2	0.5	1	0	1	0.0	0.0	0.1	0	0	0
Algeria	Belaiba	0.3	0.2	0.4	1	0	1	0.0	0.0	0.1	0	0	0
Algeria	Belala	0.3	0.2	0.4	0	0	0	0.0	0.0	0.1	0	0	0
Algeria	Belarbi	0.3	0.2	0.4	0	0	0	0.0	0.0	0.1	0	0	0
Algeria	Belimour	0.3	0.2	0.5	0	0	1	0.0	0.0	0.1	0	0	0
Algeria	Belkheir	0.3	0.2	0.4	1	0	1	0.0	0.0	0.1	0	0	0
Algeria	Bellaa	0.3	0.2	0.5	1	0	1	0.0	0.0	0.1	0	0	0
Algeria	Ben Allal	0.3	0.2	0.5	1	0	1	0.0	0.0	0.1	0	0	0
Algeria	Ben Azzouz	0.3	0.2	0.4	1	0	1	0.0	0.0	0.1	0	0	0
Algeria	Ben Badis	0.3	0.2	0.4	1	0	1	0.0	0.0	0.1	0	0	0
Algeria	Ben Badis	0.2	0.2	0.4	0	0	1	0.0	0.0	0.1	0	0	0
Algeria	Ben Chicao	0.3	0.2	0.4	0	0	0	0.0	0.0	0.0	0	0	0
Algeria	Ben Choud	0.2	0.1	0.4	0	0	0	0.0	0.0	0.1	0	0	0
Algeria	Ben Daoud	0.3	0.2	0.5	1	0	1	0.0	0.0	0.1	0	0	0
Algeria	Ben Djerrah	0.3	0.2	0.4	1	0	1	0.0	0.0	0.1	0	0	0
Algeria	Ben Freha	0.3	0.2	0.5	1	0	1	0.0	0.0	0.1	0	0	0
Algeria	Ben Guecha	0.3	0.2	0.4	0	0	0	0.0	0.0	0.1	0	0	0
Algeria	Ben M'Hidi	0.3	0.2	0.4	1	1	1	0.0	0.0	0.1	0	0	0
Algeria	Ben Srour	0.3	0.2	0.4	1	0	1	0.0	0.0	0.0	0	0	0
Algeria	Benabdelmalek Ramdane	0.3	0.2	0.4	0	0	1	0.0	0.0	0.1	0	0	0
Algeria	Benaceur	0.3	0.2	0.4	0	0	0	0.0	0.0	0.0	0	0	0
Algeria	Benaicha Chelia	0.3	0.2	0.4	0	0	0	0.0	0.0	0.1	0	0	0
Algeria	Benairia	0.3	0.2	0.4	0	0	1	0.0	0.0	0.0	0	0	0
Algeria	Bendaoud	0.3	0.2	0.4	0	0	1	0.0	0.0	0.1	0	0	0
Algeria	Benhar	0.4	0.2	0.5	1	1	1	0.0	0.0	0.1	0	0	0
Algeria	Beni Abbes	0.2	0.2	0.4	0	0	0	0.0	0.0	0.1	0	0	0
Algeria	Beni Aissi	0.2	0.1	0.3	0	0	0	0.0	0.0	0.1	0	0	0
Algeria	Beni Amrane	0.3	0.2	0.4	1	0	1	0.0	0.0	0.1	0	0	0
Algeria	Beni Bahdel	0.3	0.2	0.4	0	0	0	0.0	0.0	0.1	0	0	0
Algeria	Beni Bechir	0.3	0.2	0.4	0	0	0	0.0	0.0	0.1	0	0	0
Algeria	Beni Bouattab	0.3	0.2	0.4	0	0	0	0.0	0.0	0.1	0	0	0
Algeria	Beni Boussaid	0.3	0.2	0.4	0	0	1	0.0	0.0	0.1	0	0	0
Algeria	Beni Chaib	0.3	0.2	0.4	0	0	0	0.0	0.0	0.1	0	0	0

Table 3: Diarrhoea Mortality by Unit *(continued)*

Country	Unit	2000 mortality mean rate (per 10,000)	lower rate (per 10,000)	upper rate (per 10,000)	mean count	lower count	upper count	2017 mortality mean rate (per 10,000)	lower rate (per 10,000)	upper rate (per 10,000)	mean count	lower count	upper count
Algeria	Beni Chebana	0.3	0.2	0.4	1	0	1	0.0	0.0	0.1	0	0	0
Algeria	Beni Dejllil	0.3	0.2	0.4	0	0	0	0.0	0.0	0.1	0	0	0
Algeria	Beni Dergoun	0.3	0.2	0.4	0	0	1	0.0	0.0	0.1	0	0	0
Algeria	Beni Fouda	0.3	0.2	0.4	1	0	1	0.0	0.0	0.1	0	0	0
Algeria	Beni Foudala El Hakania	0.3	0.2	0.5	0	0	0	0.0	0.0	0.1	0	0	0
Algeria	Beni Hamidane	0.3	0.2	0.4	0	0	0	0.0	0.0	0.1	0	0	0
Algeria	Beni Haoua	0.3	0.2	0.4	0	0	1	0.0	0.0	0.0	0	0	0
Algeria	Beni Ikhlef	0.2	0.1	0.3	0	0	0	0.0	0.0	0.1	0	0	0
Algeria	Beni Ilmane	0.3	0.2	0.4	0	0	0	0.0	0.0	0.1	0	0	0
Algeria	Beni K'Sila	0.3	0.2	0.4	0	0	0	0.0	0.0	0.1	0	0	0
Algeria	Beni Khellad	0.2	0.2	0.4	0	0	1	0.0	0.0	0.1	0	0	0
Algeria	Beni Lahcene	0.3	0.2	0.5	0	0	0	0.0	0.0	0.1	0	0	0
Algeria	Beni Merad	0.3	0.2	0.4	1	1	2	0.0	0.0	0.1	0	0	0
Algeria	Beni Mester	0.3	0.2	0.4	0	0	1	0.0	0.0	0.1	0	0	0
Algeria	Beni Mezline	0.3	0.2	0.4	0	0	0	0.0	0.0	0.1	0	0	0
Algeria	Beni Mileuk	0.3	0.2	0.4	0	0	0	0.0	0.0	0.0	0	0	0
Algeria	Beni Ouarsous	0.3	0.2	0.4	0	0	0	0.0	0.0	0.1	0	0	0
Algeria	Beni Oulbane	0.3	0.2	0.4	1	0	1	0.0	0.0	0.1	0	0	0
Algeria	Beni Ounif	0.3	0.2	0.4	0	0	0	0.0	0.0	0.1	0	0	0
Algeria	Beni Oussine	0.3	0.2	0.4	0	0	1	0.0	0.0	0.1	0	0	0
Algeria	Beni Rached	0.3	0.2	0.4	1	0	1	0.0	0.0	0.0	0	0	0
Algeria	Beni Saf	0.2	0.2	0.4	1	1	2	0.0	0.0	0.1	0	0	0
Algeria	Beni Slimane	0.3	0.2	0.4	1	1	2	0.0	0.0	0.1	0	0	0
Algeria	Beni Smiel	0.3	0.2	0.4	0	0	0	0.0	0.0	0.1	0	0	0
Algeria	Beni Snous	0.3	0.2	0.4	0	0	1	0.0	0.0	0.1	0	0	0
Algeria	Beni Yenni	0.2	0.1	0.3	0	0	0	0.0	0.0	0.1	0	0	0
Algeria	Beni Zentis	0.3	0.2	0.5	0	0	1	0.0	0.0	0.1	0	0	0
Algeria	Beni Zid	0.3	0.2	0.4	1	0	1	0.0	0.0	0.1	0	0	0
Algeria	Beni Zmenzer	0.2	0.1	0.3	0	0	0	0.0	0.0	0.0	0	0	0
Algeria	Beni-Aziz	0.3	0.2	0.4	1	0	1	0.0	0.0	0.1	0	0	0
Algeria	Beni-Douala	0.2	0.1	0.3	0	0	0	0.0	0.0	0.0	0	0	0
Algeria	Beni-Mellikeche	0.2	0.1	0.3	0	0	0	0.0	0.0	0.1	0	0	0
Algeria	Beni-Mouhli	0.3	0.2	0.4	0	0	0	0.0	0.0	0.1	0	0	0
Algeria	Beni-Ouartilane	0.3	0.2	0.5	0	0	1	0.0	0.0	0.1	0	0	0
Algeria	Beni-Tamou	0.3	0.2	0.4	1	1	1	0.0	0.0	0.1	0	0	0
Algeria	Beni-Zikki	0.3	0.1	0.4	0	0	0	0.0	0.0	0.1	0	0	0
Algeria	Benian	0.3	0.2	0.5	0	0	0	0.0	0.0	0.1	0	0	0
Algeria	Benimaouche	0.3	0.2	0.4	0	0	1	0.0	0.0	0.1	0	0	0
Algeria	Benkhelil	0.3	0.2	0.4	1	1	1	0.0	0.0	0.1	0	0	0
Algeria	Bensekrane	0.2	0.1	0.3	0	0	0	0.0	0.0	0.1	0	0	0
Algeria	Benyacoub	0.4	0.3	0.6	0	0	1	0.0	0.0	0.1	0	0	0
Algeria	Benyahia Abderrahmane	0.3	0.2	0.5	0	0	1	0.0	0.0	0.1	0	0	0
Algeria	Benzouh	0.3	0.2	0.4	0	0	0	0.0	0.0	0.0	0	0	0
Algeria	Berbacha	0.3	0.2	0.4	0	0	1	0.0	0.0	0.1	0	0	0
Algeria	Berhoum	0.3	0.2	0.4	1	0	1	0.0	0.0	0.0	0	0	0
Algeria	Berrahal	0.2	0.2	0.3	1	0	1	0.0	0.0	0.1	0	0	0
Algeria	Berriane	0.3	0.2	0.5	1	1	2	0.0	0.0	0.1	0	0	0
Algeria	Berriche	0.3	0.2	0.4	1	1	2	0.0	0.0	0.1	0	0	0
Algeria	Berrihane	0.3	0.2	0.4	0	0	0	0.0	0.0	0.1	0	0	0
Algeria	Berrouaghia	0.3	0.2	0.4	2	1	3	0.0	0.0	0.0	0	0	0
Algeria	Besbes	0.3	0.2	0.4	0	0	0	0.0	0.0	0.1	0	0	0
Algeria	Besbes	0.3	0.2	0.4	1	1	2	0.0	0.0	0.1	0	0	0
Algeria	Bethioua	0.3	0.2	0.5	1	0	1	0.0	0.0	0.1	0	0	0
Algeria	Bhir El Chergui	0.3	0.2	0.4	0	0	0	0.0	0.0	0.1	0	0	0
Algeria	Bin El Ouiden	0.3	0.2	0.4	1	0	1	0.0	0.0	0.1	0	0	0
Algeria	Bir Ben Laabed	0.3	0.2	0.4	0	0	1	0.0	0.0	0.1	0	0	0
Algeria	Bir Bouhouche	0.3	0.2	0.4	0	0	0	0.0	0.0	0.1	0	0	0
Algeria	Bir Chouhada	0.3	0.2	0.4	0	0	0	0.0	0.0	0.1	0	0	0

Table 3: Diarrhoea Mortality by Unit *(continued)*

Country	Unit	2000 mortality mean rate (per 10,000)	2000 mortality lower rate (per 10,000)	2000 mortality upper rate (per 10,000)	2000 mortality mean count	2000 mortality lower count	2000 mortality upper count	2017 mortality mean rate (per 10,000)	2017 mortality lower rate (per 10,000)	2017 mortality upper rate (per 10,000)	2017 mortality mean count	2017 mortality lower count	2017 mortality upper count
Algeria	Bir Dheb	0.2	0.2	0.3	0	0	0	0.0	0.0	0.1	0	0	0
Algeria	Bir El Ater	0.2	0.1	0.2	1	1	2	0.0	0.0	0.1	0	0	1
Algeria	Bir El Djir	0.3	0.2	0.4	3	2	5	0.0	0.0	0.1	1	1	1
Algeria	Bir El Hammam	0.3	0.2	0.4	0	0	0	0.0	0.0	0.1	0	0	0
Algeria	Bir Foda	0.3	0.2	0.5	0	0	0	0.0	0.0	0.0	0	0	0
Algeria	Bir Ghbalou	0.2	0.2	0.4	0	0	0	0.0	0.0	0.1	0	0	0
Algeria	Bir Haddada	0.3	0.2	0.4	1	0	1	0.0	0.0	0.1	0	0	0
Algeria	Bir Kasdali	0.3	0.2	0.5	0	0	1	0.0	0.0	0.1	0	0	0
Algeria	Bir Mokkadem	0.2	0.2	0.3	0	0	1	0.0	0.0	0.1	0	0	0
Algeria	Bir Ould Khelifa	0.3	0.2	0.5	1	0	1	0.0	0.0	0.1	0	0	0
Algeria	Bir-El-Arch	0.3	0.2	0.4	1	1	1	0.0	0.0	0.1	0	0	0
Algeria	Birine	0.3	0.2	0.5	1	1	2	0.0	0.0	0.0	0	0	0
Algeria	Birtouta	0.3	0.2	0.4	1	1	2	0.0	0.0	0.1	0	0	0
Algeria	Biskra	0.3	0.2	0.4	6	4	9	0.0	0.0	0.1	1	1	1
Algeria	Bitam	0.2	0.1	0.4	1	0	1	0.0	0.0	0.0	0	0	0
Algeria	Blida	0.3	0.2	0.4	5	3	7	0.0	0.0	0.1	1	1	1
Algeria	Boghni	0.2	0.1	0.3	0	0	1	0.0	0.0	0.1	0	0	0
Algeria	Bordj Badji Mokhtar	0.2	0.1	0.2	0	0	0	0.0	0.0	0.0	0	0	0
Algeria	Bordj Ben Azzouz	0.3	0.2	0.5	0	0	1	0.0	0.0	0.1	0	0	0
Algeria	Bordj Bou Arreridj	0.3	0.2	0.4	4	2	7	0.0	0.0	0.1	1	0	1
Algeria	Bordj Bounaama	0.3	0.2	0.5	1	0	1	0.0	0.0	0.0	0	0	0
Algeria	Bordj El Emir Abdelkader	0.3	0.2	0.5	0	0	0	0.0	0.0	0.1	0	0	0
Algeria	Bordj El Haouasse	0.2	0.1	0.2	0	0	0	0.0	0.0	0.0	0	0	0
Algeria	Bordj El Kiffan	0.2	0.2	0.4	8	5	13	0.0	0.0	0.0	1	1	2
Algeria	Bordj Emir Khaled	0.3	0.2	0.5	0	0	0	0.0	0.0	0.1	0	0	0
Algeria	Bordj Ghdir	0.3	0.2	0.5	1	1	1	0.0	0.0	0.1	0	0	0
Algeria	Bordj Menaiel	0.3	0.2	0.4	2	1	3	0.0	0.0	0.1	0	0	0
Algeria	Bordj Okhriss	0.2	0.1	0.4	0	0	0	0.0	0.0	0.1	0	0	0
Algeria	Bordj Omar Driss	0.2	0.2	0.3	0	0	0	0.0	0.0	0.0	0	0	0
Algeria	Bordj Sebbat	0.3	0.2	0.4	0	0	0	0.0	0.0	0.1	0	0	0
Algeria	Bordj Tahar	0.3	0.2	0.5	0	0	0	0.0	0.0	0.1	0	0	0
Algeria	Bordj Zemoura	0.3	0.2	0.5	0	0	1	0.0	0.0	0.1	0	0	0
Algeria	Bou Caid	0.3	0.2	0.4	0	0	0	0.0	0.0	0.0	0	0	0
Algeria	Bou Hachana	0.3	0.2	0.4	0	0	0	0.0	0.0	0.1	0	0	0
Algeria	Bou Hamdane	0.3	0.2	0.4	0	0	0	0.0	0.0	0.1	0	0	0
Algeria	Bou Henni	0.3	0.2	0.5	0	0	1	0.0	0.0	0.1	0	0	0
Algeria	Bou Ismail	0.3	0.2	0.4	2	1	3	0.0	0.0	0.1	0	0	0
Algeria	Bou Saada	0.3	0.2	0.4	4	3	6	0.0	0.0	0.0	1	0	1
Algeria	Bou Zedjar	0.2	0.2	0.4	0	0	0	0.0	0.0	0.1	0	0	0
Algeria	Bouaarfa	0.3	0.2	0.4	1	1	2	0.0	0.0	0.1	0	0	0
Algeria	Bouaiche	0.3	0.2	0.4	0	0	1	0.0	0.0	0.0	0	0	0
Algeria	Bouaichoune	0.3	0.2	0.4	0	0	0	0.0	0.0	0.0	0	0	0
Algeria	Boualem	0.3	0.2	0.5	0	0	0	0.0	0.0	0.1	0	0	0
Algeria	Bouandas	0.3	0.2	0.4	1	0	1	0.0	0.0	0.1	0	0	0
Algeria	Bouati Mahmoud	0.3	0.2	0.4	0	0	0	0.0	0.0	0.1	0	0	0
Algeria	Bouchakroune	0.3	0.2	0.4	0	0	1	0.0	0.0	0.1	0	0	0
Algeria	Bouchekouf	0.3	0.2	0.4	1	0	1	0.0	0.0	0.1	0	0	0
Algeria	Boucherahil	0.3	0.2	0.4	1	0	1	0.0	0.0	0.1	0	0	0
Algeria	Bouchetata	0.3	0.2	0.4	0	0	0	0.0	0.0	0.1	0	0	0
Algeria	Bouda	0.4	0.2	0.5	0	0	1	0.0	0.0	0.1	0	0	0
Algeria	Bouderbala	0.2	0.1	0.3	0	0	0	0.0	0.0	0.0	0	0	0
Algeria	Boudjebaa El Bordj	0.3	0.2	0.5	0	0	0	0.0	0.0	0.1	0	0	0
Algeria	Boudjellil	0.2	0.1	0.3	0	0	1	0.0	0.0	0.1	0	0	0

Table 3: Diarrhoea Mortality by Unit *(continued)*

Country	Unit	2000 mortality mean rate (per 10,000)	lower rate (per 10,000)	upper rate (per 10,000)	mean count	lower count	upper count	2017 mortality mean rate (per 10,000)	lower rate (per 10,000)	upper rate (per 10,000)	mean count	lower count	upper count
Algeria	Boudjeriou Messaoud	0.3	0.2	0.4	0	0	0	0.0	0.0	0.1	0	0	0
Algeria	Boudjima	0.2	0.1	0.3	0	0	0	0.0	0.0	0.1	0	0	0
Algeria	Boudouaou	0.3	0.2	0.4	2	1	2	0.0	0.0	0.1	0	0	0
Algeria	Boudouaou El Bahri	0.3	0.2	0.4	0	0	1	0.0	0.0	0.1	0	0	0
Algeria	Boudria Beniyadjis	0.3	0.2	0.4	0	0	0	0.0	0.0	0.1	0	0	0
Algeria	Boufarik	0.3	0.2	0.4	2	1	3	0.0	0.0	0.1	0	0	1
Algeria	Boufatis	0.3	0.2	0.5	0	0	1	0.0	0.0	0.1	0	0	0
Algeria	Bougaa	0.3	0.2	0.4	1	1	1	0.0	0.0	0.1	0	0	0
Algeria	Bougara	0.3	0.2	0.5	0	0	0	0.0	0.0	0.1	0	0	0
Algeria	Bougara	0.3	0.2	0.4	1	1	2	0.0	0.0	0.1	0	0	0
Algeria	Boughar	0.3	0.2	0.4	1	0	1	0.0	0.0	0.1	0	0	0
Algeria	Boughezoul	0.3	0.2	0.4	1	0	1	0.0	0.0	0.1	0	0	0
Algeria	Bougous	0.3	0.2	0.4	0	0	0	0.0	0.0	0.1	0	0	0
Algeria	Bougtoub	0.3	0.2	0.5	1	0	1	0.0	0.0	0.1	0	0	0
Algeria	Bouguirat	0.3	0.2	0.5	1	1	2	0.0	0.0	0.1	0	0	0
Algeria	Bouhadjar	0.3	0.2	0.4	1	0	1	0.0	0.0	0.1	0	0	0
Algeria	Bouhamza	0.2	0.2	0.4	0	0	0	0.0	0.0	0.1	0	0	0
Algeria	Bouhanifia	0.3	0.2	0.5	1	0	1	0.0	0.0	0.1	0	0	0
Algeria	Bouhatem	0.3	0.2	0.5	1	0	1	0.0	0.0	0.1	0	0	0
Algeria	Bouhlou	0.3	0.2	0.4	0	0	0	0.0	0.0	0.1	0	0	0
Algeria	Bouhmama	0.3	0.2	0.4	0	0	0	0.0	0.0	0.1	0	0	0
Algeria	Bouihi	0.3	0.2	0.5	0	0	0	0.0	0.0	0.1	0	0	0
Algeria	Bouinan	0.3	0.2	0.4	1	1	2	0.0	0.0	0.1	0	0	0
Algeria	Bouira	0.2	0.2	0.4	2	1	3	0.0	0.0	0.1	0	0	1
Algeria	Bouira Lahdab	0.4	0.2	0.6	1	0	1	0.0	0.0	0.0	0	0	0
Algeria	Boukadir	0.2	0.2	0.4	1	1	2	0.0	0.0	0.1	0	0	0
Algeria	Boukais	0.3	0.2	0.4	0	0	0	0.0	0.0	0.1	0	0	0
Algeria	Boukhadra	0.3	0.2	0.4	0	0	0	0.0	0.0	0.1	0	0	0
Algeria	Boukhenifis	0.2	0.2	0.4	0	0	0	0.0	0.0	0.1	0	0	0
Algeria	Boukhlifa	0.3	0.2	0.4	0	0	0	0.0	0.0	0.1	0	0	0
Algeria	Boukram	0.2	0.2	0.4	0	0	0	0.0	0.0	0.0	0	0	0
Algeria	Boulhaf Dyr	0.2	0.1	0.3	0	0	1	0.0	0.0	0.1	0	0	0
Algeria	Boulhilat	0.3	0.2	0.4	0	0	0	0.0	0.0	0.1	0	0	0
Algeria	Boumahra Ahmed	0.3	0.2	0.4	0	0	1	0.0	0.0	0.1	0	0	0
Algeria	Boumedfaa	0.3	0.2	0.4	1	0	1	0.0	0.0	0.0	0	0	0
Algeria	Boumegueur	0.3	0.2	0.5	0	0	1	0.0	0.0	0.1	0	0	0
Algeria	Boumerdes	0.3	0.2	0.4	0	0	0	0.0	0.0	0.1	0	0	0
Algeria	Boumia	0.3	0.2	0.4	0	0	0	0.0	0.0	0.1	0	0	0
Algeria	Bounouh	0.2	0.1	0.3	0	0	0	0.0	0.0	0.0	0	0	0
Algeria	Bounoura	0.3	0.2	0.5	3	2	5	0.0	0.0	0.1	0	0	1
Algeria	Bourached	0.3	0.2	0.4	1	1	2	0.0	0.0	0.1	0	0	0
Algeria	Bouraoui Belhadef	0.3	0.2	0.5	0	0	0	0.0	0.0	0.1	0	0	0
Algeria	Bourkika	0.3	0.2	0.4	1	0	1	0.0	0.0	0.1	0	0	0
Algeria	Bousfer	0.2	0.1	0.3	0	0	0	0.0	0.0	0.1	0	0	0
Algeria	Bouskene	0.3	0.2	0.4	1	0	1	0.0	0.0	0.1	0	0	0
Algeria	Bousselam	0.3	0.2	0.4	1	0	1	0.0	0.0	0.1	0	0	0
Algeria	Boussemghoun	0.3	0.2	0.4	0	0	0	0.0	0.0	0.1	0	0	0
Algeria	Boussif Ouled Askeur	0.3	0.2	0.5	0	0	1	0.0	0.0	0.1	0	0	0
Algeria	Boutaleb	0.3	0.2	0.4	0	0	1	0.0	0.0	0.1	0	0	0
Algeria	Bouteldja	0.3	0.2	0.4	0	0	1	0.0	0.0	0.1	0	0	0
Algeria	Bouti Sayeh	0.3	0.2	0.4	0	0	1	0.0	0.0	0.0	0	0	0
Algeria	Boutlelis	0.2	0.1	0.3	0	0	1	0.0	0.0	0.1	0	0	0
Algeria	Bouzareah	0.3	0.2	0.4	16	10	25	0.0	0.0	0.0	2	1	2
Algeria	Bouzeghaia	0.3	0.2	0.4	1	0	1	0.0	0.0	0.1	0	0	0

Table 3: Diarrhoea Mortality by Unit *(continued)*

Country	Unit	2000 mortality mean rate (per 10,000)	lower rate (per 10,000)	upper rate (per 10,000)	mean count	lower count	upper count	2017 mortality mean rate (per 10,000)	lower rate (per 10,000)	upper rate (per 10,000)	mean count	lower count	upper count
Algeria	Bouzeguene	0.2	0.1	0.4	0	0	1	0.0	0.0	0.1	0	0	0
Algeria	Bouzegza Keddara	0.3	0.2	0.4	0	0	0	0.0	0.0	0.1	0	0	0
Algeria	Bouzina	0.3	0.2	0.4	1	0	1	0.0	0.0	0.1	0	0	0
Algeria	Branis	0.3	0.2	0.4	0	0	0	0.0	0.0	0.1	0	0	0
Algeria	Breira	0.3	0.2	0.4	0	0	1	0.0	0.0	0.0	0	0	0
Algeria	Brezina	0.3	0.2	0.4	0	0	1	0.0	0.0	0.0	0	0	0
Algeria	Brida	0.4	0.2	0.5	0	0	0	0.0	0.0	0.1	0	0	0
Algeria	Chaabet El Ham	0.2	0.1	0.4	1	0	1	0.0	0.0	0.1	0	0	0
Algeria	Chabet El Ameur	0.3	0.2	0.4	1	1	1	0.0	0.0	0.1	0	0	0
Algeria	Chahbounia	0.3	0.2	0.5	0	0	1	0.0	0.0	0.1	0	0	0
Algeria	Chahna	0.3	0.2	0.5	0	0	0	0.0	0.0	0.1	0	0	0
Algeria	Chaiba	0.3	0.2	0.4	1	1	1	0.0	0.0	0.1	0	0	0
Algeria	Charef	0.4	0.3	0.7	1	1	2	0.0	0.0	0.1	0	0	0
Algeria	Charouine	0.3	0.2	0.4	0	0	1	0.0	0.0	0.1	0	0	0
Algeria	Chebaita Mokhtar	0.3	0.2	0.4	1	0	1	0.0	0.0	0.1	0	0	0
Algeria	Chebli	0.3	0.2	0.4	1	1	2	0.0	0.0	0.1	0	0	0
Algeria	Chechar	0.2	0.2	0.4	1	0	1	0.0	0.0	0.1	0	0	0
Algeria	Chefia	0.3	0.2	0.4	0	0	0	0.0	0.0	0.1	0	0	0
Algeria	Cheguig	0.3	0.2	0.5	0	0	0	0.0	0.0	0.1	0	0	0
Algeria	Chehaima	0.3	0.2	0.5	0	0	1	0.0	0.0	0.1	0	0	0
Algeria	Chekfa	0.3	0.2	0.4	1	0	1	0.0	0.0	0.1	0	0	0
Algeria	Chelghoum Laid	0.3	0.2	0.5	3	2	4	0.0	0.0	0.1	0	0	1
Algeria	Chelia	0.3	0.2	0.4	0	0	1	0.0	0.0	0.1	0	0	0
Algeria	Chellal	0.3	0.2	0.4	0	0	0	0.0	0.0	0.1	0	0	0
Algeria	Chellala	0.3	0.2	0.4	0	0	0	0.0	0.0	0.1	0	0	0
Algeria	Chellalet Ladhaoura	0.3	0.2	0.4	1	0	1	0.0	0.0	0.0	0	0	0
Algeria	Chellata	0.2	0.2	0.4	0	0	1	0.0	0.0	0.1	0	0	0
Algeria	Chemini	0.3	0.2	0.4	0	0	1	0.0	0.0	0.1	0	0	0
Algeria	Chemora	0.3	0.2	0.4	1	0	1	0.0	0.0	0.1	0	0	0
Algeria	Cheniguel	0.3	0.2	0.4	0	0	0	0.0	0.0	0.0	0	0	0
Algeria	Chentouf	0.2	0.1	0.4	0	0	0	0.0	0.0	0.1	0	0	0
Algeria	Cheraga	0.3	0.2	0.4	3	2	5	0.0	0.0	0.1	1	0	1
Algeria	Cheraga	0.3	0.2	0.5	1	0	1	0.0	0.0	0.1	0	0	0
Algeria	Cheraia	0.3	0.2	0.4	1	0	1	0.0	0.0	0.1	0	0	0
Algeria	Cherchel	0.3	0.2	0.4	1	1	2	0.0	0.0	0.1	0	0	0
Algeria	Chetaibi	0.3	0.2	0.4	0	0	0	0.0	0.0	0.1	0	0	0
Algeria	Chetma	0.3	0.2	0.4	1	0	1	0.0	0.0	0.1	0	0	0
Algeria	Chetouane	0.2	0.1	0.4	1	1	2	0.0	0.0	0.1	0	0	0
Algeria	Chetouane Belaila	0.2	0.2	0.4	0	0	0	0.0	0.0	0.1	0	0	0
Algeria	Chettia	0.2	0.2	0.4	2	1	2	0.0	0.0	0.1	0	0	0
Algeria	Chiffa	0.2	0.2	0.4	1	1	1	0.0	0.0	0.1	0	0	0
Algeria	Chihani	0.3	0.2	0.4	0	0	1	0.0	0.0	0.1	0	0	0
Algeria	Chir	0.3	0.2	0.4	0	0	0	0.0	0.0	0.1	0	0	0
Algeria	Chlef	0.2	0.2	0.3	4	3	7	0.0	0.0	0.0	1	0	1
Algeria	Chorfa	0.3	0.2	0.5	0	0	0	0.0	0.0	0.1	0	0	0
Algeria	Chorfa	0.2	0.1	0.3	0	0	0	0.0	0.0	0.1	0	0	0
Algeria	Chorfa	0.2	0.2	0.4	0	0	1	0.0	0.0	0.1	0	0	0
Algeria	Chouaiba|Ouled Rahma	0.3	0.2	0.4	0	0	0	0.0	0.0	0.0	0	0	0
Algeria	Chrea	0.3	0.2	0.4	0	0	1	0.0	0.0	0.1	0	0	0
Algeria	Chrea	0.2	0.1	0.4	2	1	3	0.0	0.0	0.1	0	0	1
Algeria	Colla	0.3	0.2	0.5	0	0	0	0.0	0.0	0.1	0	0	0
Algeria	Collo	0.3	0.2	0.4	1	0	1	0.0	0.0	0.1	0	0	0
Algeria	Constantine	0.3	0.2	0.4	14	9	22	0.0	0.0	0.1	2	1	3
Algeria	Corso	0.3	0.2	0.4	0	0	1	0.0	0.0	0.1	0	0	0
Algeria	Dahmouni	0.3	0.2	0.4	1	0	1	0.0	0.0	0.1	0	0	0

Country	Unit	2000 mortality mean rate (per 10,000)	lower rate (per 10,000)	upper rate (per 10,000)	mean count	lower count	upper count	2017 mortality mean rate (per 10,000)	lower rate (per 10,000)	upper rate (per 10,000)	mean count	lower count	upper count
Algeria	Dahouara	0.3	0.2	0.4	0	0	0	0.0	0.0	0.1	0	0	0
Algeria	Dahra	0.3	0.2	0.5	1	1	1	0.0	0.0	0.1	0	0	0
Algeria	Damous	0.3	0.2	0.4	0	0	1	0.0	0.0	0.0	0	0	0
Algeria	Daoussen	0.3	0.2	0.4	1	1	1	0.0	0.0	0.1	0	0	0
Algeria	Dar Ben Abdelah	0.3	0.2	0.4	0	0	0	0.0	0.0	0.1	0	0	0
Algeria	Dar Chioukh	0.4	0.2	0.6	2	1	2	0.0	0.0	0.1	0	0	0
Algeria	Dar El Beida	0.2	0.2	0.4	3	2	5	0.0	0.0	0.0	1	0	1
Algeria	Dar Yaghmouracene	0.2	0.2	0.4	0	0	0	0.0	0.0	0.1	0	0	0
Algeria	Darguina	0.2	0.1	0.4	0	0	1	0.0	0.0	0.1	0	0	0
Algeria	Debdeb	0.2	0.2	0.3	0	0	0	0.0	0.0	0.0	0	0	0
Algeria	Debila	0.3	0.2	0.5	1	1	2	0.0	0.0	0.1	0	0	0
Algeria	Dechmia	0.3	0.2	0.4	0	0	0	0.0	0.0	0.1	0	0	0
Algeria	Dehahna	0.3	0.2	0.4	0	0	0	0.0	0.0	0.0	0	0	0
Algeria	Dehamcha	0.3	0.2	0.4	0	0	1	0.0	0.0	0.1	0	0	0
Algeria	Deldoul	0.4	0.3	0.5	1	1	1	0.0	0.0	0.0	0	0	0
Algeria	Deldoul	0.3	0.2	0.4	0	0	0	0.0	0.0	0.1	0	0	0
Algeria	Dellys	0.2	0.1	0.4	1	0	1	0.0	0.0	0.1	0	0	0
Algeria	Derradji Bousselah	0.3	0.2	0.5	0	0	1	0.0	0.0	0.1	0	0	0
Algeria	Derrag	0.3	0.2	0.5	0	0	0	0.0	0.0	0.1	0	0	0
Algeria	Deux Bassins	0.3	0.2	0.4	0	0	0	0.0	0.0	0.0	0	0	0
Algeria	Dhaya	0.3	0.2	0.5	0	0	0	0.0	0.0	0.1	0	0	0
Algeria	Dhayet Bendhahoua	0.3	0.2	0.5	1	0	1	0.0	0.0	0.1	0	0	0
Algeria	Didouche Mourad	0.3	0.2	0.4	1	1	2	0.0	0.0	0.1	0	0	0
Algeria	Dirrah	0.3	0.2	0.4	1	0	1	0.0	0.0	0.1	0	0	0
Algeria	Djaafra	0.3	0.2	0.5	0	0	1	0.0	0.0	0.1	0	0	0
Algeria	Djamaa	0.3	0.2	0.4	2	1	2	0.0	0.0	0.1	0	0	0
Algeria	Djamora	0.2	0.2	0.4	0	0	1	0.0	0.0	0.0	0	0	0
Algeria	Djanet	0.2	0.1	0.2	0	0	0	0.0	0.0	0.0	0	0	0
Algeria	Djasr Kasentina	0.3	0.2	0.4	7	4	10	0.0	0.0	0.1	1	1	1
Algeria	Djebabra	0.3	0.2	0.4	0	0	0	0.0	0.0	0.1	0	0	0
Algeria	Djebahia	0.2	0.1	0.3	0	0	1	0.0	0.0	0.1	0	0	0
Algeria	Djebala	0.3	0.2	0.4	0	0	1	0.0	0.0	0.1	0	0	0
Algeria	Djebala El Khemissi	0.3	0.2	0.4	0	0	0	0.0	0.0	0.1	0	0	0
Algeria	Djebel Messaad	0.3	0.2	0.4	1	0	1	0.0	0.0	0.0	0	0	0
Algeria	Djebilet Rosfa	0.3	0.2	0.5	0	0	0	0.0	0.0	0.1	0	0	0
Algeria	Djelfa	0.4	0.2	0.5	11	7	16	0.0	0.0	0.0	2	1	3
Algeria	Djelida	0.3	0.2	0.4	1	1	2	0.0	0.0	0.1	0	0	0
Algeria	Djellal	0.2	0.1	0.4	0	0	0	0.0	0.0	0.1	0	0	0
Algeria	Djemaa Beni Habibi	0.3	0.2	0.4	0	0	1	0.0	0.0	0.1	0	0	0
Algeria	Djemaa Ouled Cheikh	0.3	0.2	0.4	0	0	0	0.0	0.0	0.0	0	0	0
Algeria	Djemila	0.3	0.2	0.4	1	1	1	0.0	0.0	0.1	0	0	0
Algeria	Djemila	0.3	0.2	0.5	0	0	1	0.0	0.0	0.1	0	0	0
Algeria	Djendel	0.3	0.2	0.5	1	1	2	0.0	0.0	0.1	0	0	0
Algeria	Djendel Saadi Mohamed	0.3	0.2	0.4	0	0	1	0.0	0.0	0.1	0	0	0
Algeria	Djeniane Bourzeg	0.3	0.2	0.4	0	0	0	0.0	0.0	0.1	0	0	0
Algeria	Djerma	0.3	0.2	0.5	0	0	0	0.0	0.0	0.1	0	0	0
Algeria	Djezzar	0.2	0.2	0.4	1	0	1	0.0	0.0	0.0	0	0	0
Algeria	Djidiouia	0.3	0.2	0.4	1	1	2	0.0	0.0	0.1	0	0	0
Algeria	Djillali Ben Ammar	0.3	0.2	0.4	0	0	0	0.0	0.0	0.1	0	0	0
Algeria	Djinet	0.3	0.2	0.4	1	0	1	0.0	0.0	0.1	0	0	0
Algeria	Djouab	0.3	0.2	0.4	0	0	1	0.0	0.0	0.1	0	0	0
Algeria	Douaouda	0.3	0.2	0.4	0	0	0	0.0	0.0	0.1	0	0	0

Table 3: Diarrhoea Mortality by Unit *(continued)*

Country	Unit	2000 mortality mean rate (per 10,000)	lower rate (per 10,000)	upper rate (per 10,000)	mean count	lower count	upper count	2017 mortality mean rate (per 10,000)	lower rate (per 10,000)	upper rate (per 10,000)	mean count	lower count	upper count
Algeria	Douar El Ma	0.3	0.2	0.4	0	0	0	0.0	0.0	0.1	0	0	0
Algeria	Douera	0.3	0.2	0.4	2	1	3	0.0	0.0	0.1	0	0	1
Algeria	Doui Thabet	0.3	0.2	0.4	0	0	0	0.0	0.0	0.1	0	0	0
Algeria	Douis	0.4	0.3	0.6	1	0	1	0.0	0.0	0.1	0	0	0
Algeria	Draa El Caid	0.3	0.2	0.4	1	0	1	0.0	0.0	0.1	0	0	0
Algeria	Draa El Mizan	0.2	0.1	0.3	1	0	1	0.0	0.0	0.1	0	0	0
Algeria	Draa Smar	0.3	0.2	0.4	1	0	1	0.0	0.0	0.0	0	0	0
Algeria	Draa-Ben-Khedda	0.2	0.1	0.3	0	0	1	0.0	0.0	0.1	0	0	0
Algeria	Draa-Kebila	0.3	0.2	0.4	1	0	1	0.0	0.0	0.1	0	0	0
Algeria	Draria	0.3	0.2	0.4	1	1	2	0.0	0.0	0.1	0	0	0
Algeria	Drea	0.3	0.2	0.4	0	0	1	0.0	0.0	0.1	0	0	0
Algeria	Drean	0.3	0.2	0.4	1	1	1	0.0	0.0	0.1	0	0	0
Algeria	Echatt	0.3	0.2	0.4	1	1	1	0.0	0.0	0.1	0	0	0
Algeria	El Abadia	0.3	0.2	0.4	1	1	2	0.0	0.0	0.0	0	0	0
Algeria	El Ach	0.3	0.2	0.4	1	0	1	0.0	0.0	0.0	0	0	0
Algeria	El Achir	0.3	0.2	0.4	1	0	1	0.0	0.0	0.0	0	0	0
Algeria	El Achour	0.3	0.2	0.4	3	2	5	0.0	0.0	0.0	1	0	1
Algeria	El Adjiba	0.2	0.1	0.3	0	0	0	0.0	0.0	0.1	0	0	0
Algeria	El Aioun	0.3	0.2	0.3	0	0	0	0.0	0.0	0.1	0	0	0
Algeria	El Allia	0.3	0.2	0.4	0	0	0	0.0	0.0	0.1	0	0	0
Algeria	El Amiria	0.3	0.2	0.4	0	0	1	0.0	0.0	0.1	0	0	0
Algeria	El Amra	0.3	0.2	0.4	1	1	1	0.0	0.0	0.0	0	0	0
Algeria	El Amria	0.2	0.2	0.4	1	0	1	0.0	0.0	0.1	0	0	0
Algeria	El Ançar	0.2	0.1	0.4	0	0	0	0.0	0.0	0.1	0	0	0
Algeria	El Ancer	0.3	0.2	0.4	1	0	1	0.0	0.0	0.1	0	0	0
Algeria	El Anseur	0.3	0.2	0.4	1	0	1	0.0	0.0	0.1	0	0	0
Algeria	El Aouana	0.3	0.2	0.4	0	0	1	0.0	0.0	0.1	0	0	0
Algeria	El Aricha	0.3	0.2	0.4	0	0	0	0.0	0.0	0.1	0	0	0
Algeria	El Asnam	0.2	0.1	0.3	0	0	1	0.0	0.0	0.1	0	0	0
Algeria	El Assafia	0.3	0.2	0.5	0	0	0	0.0	0.0	0.0	0	0	0
Algeria	El Attaf	0.3	0.2	0.4	1	1	2	0.0	0.0	0.0	0	0	0
Algeria	El Atteuf	0.3	0.2	0.5	1	0	1	0.0	0.0	0.1	0	0	0
Algeria	El Bayadh	0.3	0.2	0.6	1	1	2	0.0	0.0	0.1	0	0	0
Algeria	El Beidha	0.4	0.2	0.5	0	0	1	0.0	0.0	0.1	0	0	0
Algeria	El Biod	0.3	0.2	0.5	0	0	1	0.0	0.0	0.1	0	0	0
Algeria	El Biodh Sidi Cheikh	0.3	0.2	0.4	3	2	4	0.0	0.0	0.1	1	0	1
Algeria	El Bnoud	0.3	0.2	0.4	0	0	0	0.0	0.0	0.0	0	0	0
Algeria	El Bordj	0.4	0.2	0.5	1	1	1	0.0	0.0	0.1	0	0	0
Algeria	El Borma	0.2	0.2	0.3	0	0	0	0.0	0.0	0.0	0	0	0
Algeria	El Bouni	0.3	0.2	0.4	3	2	5	0.0	0.0	0.1	0	0	1
Algeria	El Braya	0.3	0.2	0.5	0	0	0	0.0	0.0	0.1	0	0	0
Algeria	El Dhaala	0.3	0.2	0.4	0	0	0	0.0	0.0	0.1	0	0	0
Algeria	El Djazia	0.3	0.2	0.4	0	0	0	0.0	0.0	0.1	0	0	0
Algeria	El Eulma	0.3	0.2	0.4	4	2	6	0.0	0.0	0.1	1	0	1
Algeria	El Eulma	0.2	0.2	0.4	0	0	0	0.0	0.0	0.1	0	0	1
Algeria	El Fedjoudj	0.3	0.1	0.4	1	0	1	0.0	0.0	0.1	0	0	0
Algeria	El Fedjoudj Boughrara Saoudi	0.3	0.2	0.4	0	0	0	0.0	0.0	0.1	0	0	0
Algeria	El Fehoul	0.2	0.1	0.4	0	0	0	0.0	0.0	0.1	0	0	0
Algeria	El Feidh	0.2	0.2	0.4	0	0	1	0.0	0.0	0.1	0	0	0
Algeria	El Gaada	0.3	0.2	0.5	0	0	0	0.0	0.0	0.1	0	0	0
Algeria	El Ghedir	0.3	0.2	0.4	0	0	0	0.0	0.0	0.1	0	0	0
Algeria	El Ghicha	0.4	0.2	0.5	0	0	1	0.0	0.0	0.1	0	0	0
Algeria	El Ghomri	0.3	0.2	0.5	0	0	1	0.0	0.0	0.1	0	0	0
Algeria	El Ghrous	0.3	0.2	0.4	1	0	1	0.0	0.0	0.1	0	0	0
Algeria	El Gor	0.3	0.2	0.4	0	0	0	0.0	0.0	0.1	0	0	0
Algeria	El Guedid	0.4	0.3	0.7	1	1	1	0.0	0.0	0.1	0	0	0
Algeria	El Guelb El Kebir	0.3	0.2	0.4	1	0	1	0.0	0.0	0.1	0	0	0

Table 3: Diarrhoea Mortality by Unit *(continued)*

Country	Unit	2000 mortality mean rate (per 10,000)	lower rate (per 10,000)	upper rate (per 10,000)	mean count	lower count	upper count	2017 mortality mean rate (per 10,000)	lower rate (per 10,000)	upper rate (per 10,000)	mean count	lower count	upper count
Algeria	El Guerrarra	0.3	0.2	0.5	3	2	4	0.0	0.0	0.1	0	0	1
Algeria	El Guettana	0.3	0.2	0.5	0	0	0	0.0	0.0	0.1	0	0	0
Algeria	El Guettar	0.3	0.2	0.5	1	0	1	0.0	0.0	0.1	0	0	0
Algeria	El H'Madna	0.3	0.2	0.4	1	0	1	0.0	0.0	0.1	0	0	0
Algeria	El Hacaiba	0.3	0.2	0.4	0	0	0	0.0	0.0	0.1	0	0	0
Algeria	El Hachem	0.3	0.2	0.5	1	1	1	0.0	0.0	0.1	0	0	0
Algeria	El Hachimia	0.3	0.2	0.4	0	0	1	0.0	0.0	0.1	0	0	0
Algeria	El Hadaiek	0.3	0.2	0.4	1	0	1	0.0	0.0	0.1	0	0	0
Algeria	El Hadjab	0.3	0.2	0.4	1	1	1	0.0	0.0	0.1	0	0	0
Algeria	El Hadjadj	0.3	0.2	0.4	0	0	0	0.0	0.0	0.1	0	0	0
Algeria	El Hadjar	0.2	0.2	0.4	1	1	2	0.0	0.0	0.1	0	0	0
Algeria	El Hadjira	0.3	0.2	0.4	1	0	1	0.0	0.0	0.1	0	0	0
Algeria	El Hakimia	0.2	0.2	0.4	0	0	0	0.0	0.0	0.1	0	0	0
Algeria	El Hamadia	0.3	0.2	0.4	1	1	1	0.0	0.0	0.1	0	0	0
Algeria	El Hamdania	0.2	0.2	0.4	0	0	0	0.0	0.0	0.0	0	0	0
Algeria	El Hamma	0.3	0.2	0.4	1	0	1	0.0	0.0	0.1	0	0	0
Algeria	El Haouaita	0.3	0.2	0.4	0	0	0	0.0	0.0	0.0	0	0	0
Algeria	El Haouch	0.3	0.2	0.4	0	0	0	0.0	0.0	0.1	0	0	0
Algeria	El Harmilia	0.3	0.2	0.4	0	0	0	0.0	0.0	0.1	0	0	0
Algeria	El Harrouch	0.3	0.2	0.4	1	1	2	0.0	0.0	0.1	0	0	0
Algeria	El Hassania	0.3	0.2	0.5	0	0	0	0.0	0.0	0.1	0	0	0
Algeria	El Hassasna	0.3	0.2	0.5	0	0	1	0.0	0.0	0.1	0	0	0
Algeria	El Hassi	0.3	0.2	0.4	0	0	0	0.0	0.0	0.1	0	0	0
Algeria	El Hassi	0.3	0.2	0.5	0	0	0	0.0	0.0	0.1	0	0	0
Algeria	El Houamed	0.3	0.2	0.4	0	0	0	0.0	0.0	0.0	0	0	0
Algeria	El Houidjbet	0.2	0.1	0.3	0	0	0	0.0	0.0	0.1	0	0	0
Algeria	El Idrissia	0.4	0.3	0.7	2	1	2	0.0	0.0	0.1	0	0	0
Algeria	El Kaf Lakhdar	0.3	0.2	0.4	0	0	0	0.0	0.0	0.0	0	0	0
Algeria	El Kala	0.3	0.2	0.4	1	1	1	0.0	0.0	0.1	0	0	0
Algeria	El Karimia	0.3	0.2	0.4	1	1	1	0.0	0.0	0.0	0	0	0
Algeria	El Kennar Nouchfi	0.3	0.2	0.4	0	0	1	0.0	0.0	0.1	0	0	0
Algeria	El Kentara	0.2	0.2	0.4	0	0	1	0.0	0.0	0.1	0	0	0
Algeria	El Kerma	0.3	0.2	0.4	1	1	1	0.0	0.0	0.1	0	0	0
Algeria	El Keurt	0.3	0.2	0.5	1	0	1	0.0	0.0	0.1	0	0	0
Algeria	El Khabouzia	0.2	0.2	0.4	0	0	0	0.0	0.0	0.1	0	0	0
Algeria	El Kharrouba	0.3	0.2	0.4	0	0	1	0.0	0.0	0.0	0	0	0
Algeria	El Kheither	0.3	0.2	0.5	0	0	1	0.0	0.0	0.1	0	0	0
Algeria	El Khemis	0.4	0.3	0.6	0	0	1	0.0	0.0	0.1	0	0	0
Algeria	El Khroub	0.3	0.2	0.4	3	2	5	0.0	0.0	0.1	1	1	2
Algeria	El Kouif	0.2	0.2	0.3	0	0	1	0.0	0.0	0.1	0	0	0
Algeria	El Kseur	0.2	0.1	0.3	1	0	1	0.0	0.0	0.1	0	0	0
Algeria	El M'Ghair	0.3	0.2	0.4	2	1	3	0.0	0.0	0.1	0	0	1
Algeria	El M'Hir	0.2	0.2	0.4	0	0	1	0.0	0.0	0.0	0	0	0
Algeria	El Madher	0.3	0.2	0.4	0	0	1	0.0	0.0	0.1	0	0	0
Algeria	El Mahmal	0.3	0.2	0.4	1	1	1	0.0	0.0	0.1	0	0	0
Algeria	El Main	0.3	0.2	0.5	0	0	0	0.0	0.0	0.1	0	0	0
Algeria	El Maine	0.3	0.2	0.4	0	0	1	0.0	0.0	0.1	0	0	0
Algeria	El Malabiodh	0.2	0.1	0.3	0	0	0	0.0	0.0	0.1	0	0	0
Algeria	El Malah	0.2	0.1	0.4	0	0	1	0.0	0.0	0.1	0	0	0
Algeria	El Mamounia	0.3	0.2	0.5	1	0	1	0.0	0.0	0.1	0	0	0
Algeria	El Marsa	0.3	0.2	0.4	0	0	0	0.0	0.0	0.1	0	0	0
Algeria	El Marsa	0.3	0.2	0.4	0	0	0	0.0	0.0	0.1	0	0	0
Algeria	El Matmar	0.3	0.2	0.5	1	0	1	0.0	0.0	0.1	0	0	0
Algeria	El Matmor	0.3	0.2	0.5	1	0	1	0.0	0.0	0.1	0	0	0
Algeria	El Mechira	0.3	0.2	0.5	0	0	1	0.0	0.0	0.1	0	0	0
Algeria	El Mehara	0.3	0.2	0.5	0	0	0	0.0	0.0	0.1	0	0	0
Algeria	El Menaouer	0.4	0.2	0.5	0	0	1	0.0	0.0	0.1	0	0	0
Algeria	El Meniaa	0.3	0.2	0.4	1	1	2	0.0	0.0	0.1	0	0	0
Algeria	El Meridj	0.3	0.2	0.3	0	0	0	0.0	0.0	0.1	0	0	0

Table 3: Diarrhoea Mortality by Unit *(continued)*

Country	Unit	2000 mortality mean rate (per 10,000)	lower rate (per 10,000)	upper rate (per 10,000)	mean count	lower count	upper count	2017 mortality mean rate (per 10,000)	lower rate (per 10,000)	upper rate (per 10,000)	mean count	lower count	upper count
Algeria	El Messaid	0.2	0.2	0.4	0	0	0	0.0	0.0	0.1	0	0	0
Algeria	El Mezeraa	0.2	0.2	0.3	0	0	0	0.0	0.0	0.1	0	0	0
Algeria	El Milia	0.3	0.2	0.4	2	1	3	0.0	0.0	0.1	0	0	0
Algeria	El Mokrani|El Madjen	0.2	0.1	0.3	0	0	0	0.0	0.0	0.1	0	0	0
Algeria	El Ogla	0.2	0.2	0.3	0	0	1	0.0	0.0	0.1	0	0	0
Algeria	El Ogla	0.3	0.2	0.4	0	0	0	0.0	0.0	0.1	0	0	0
Algeria	El Ouata	0.2	0.2	0.4	0	0	0	0.0	0.0	0.1	0	0	0
Algeria	El Oued	0.3	0.2	0.5	5	3	7	0.0	0.0	0.1	1	1	1
Algeria	El Oueldja	0.2	0.1	0.3	0	0	0	0.0	0.0	0.1	0	0	0
Algeria	El Oueldja	0.2	0.1	0.4	0	0	0	0.0	0.0	0.1	0	0	0
Algeria	El Oueldja	0.3	0.2	0.4	1	0	1	0.0	0.0	0.1	0	0	0
Algeria	El Ouinet	0.2	0.2	0.4	1	0	1	0.0	0.0	0.1	0	0	0
Algeria	El Ouinet	0.2	0.2	0.4	0	0	0	0.0	0.0	0.0	0	0	0
Algeria	El Ouitaya	0.3	0.2	0.4	0	0	1	0.0	0.0	0.1	0	0	0
Algeria	El Oumaria	0.3	0.2	0.4	1	0	1	0.0	0.0	0.0	0	0	0
Algeria	El Ouricia	0.3	0.2	0.5	1	1	2	0.0	0.0	0.1	0	0	0
Algeria	El Rahia	0.3	0.2	0.4	0	0	0	0.0	0.0	0.1	0	0	0
Algeria	El Tarf	0.3	0.2	0.4	1	0	1	0.0	0.0	0.1	0	0	0
Algeria	El Youssoufia	0.3	0.2	0.5	0	0	0	0.0	0.0	0.0	0	0	0
Algeria	El-Affroun	0.2	0.2	0.4	1	1	1	0.0	0.0	0.1	0	0	0
Algeria	Elayadi Barbes	0.3	0.2	0.5	0	0	1	0.0	0.0	0.1	0	0	0
Algeria	Emir Abdelkader	0.3	0.2	0.4	1	0	1	0.0	0.0	0.1	0	0	0
Algeria	Emir Abdelkader	0.2	0.2	0.4	0	0	0	0.0	0.0	0.1	0	0	0
Algeria	Emjez Edchich	0.3	0.2	0.4	1	0	1	0.0	0.0	0.1	0	0	0
Algeria	Ensigha	0.3	0.2	0.4	1	0	1	0.0	0.0	0.1	0	0	0
Algeria	Erg Ferradj	0.3	0.2	0.4	0	0	0	0.0	0.0	0.1	0	0	0
Algeria	Erraguene	0.3	0.2	0.4	0	0	0	0.0	0.0	0.1	0	0	0
Algeria	Es Sebt	0.3	0.2	0.4	1	0	1	0.0	0.0	0.1	0	0	0
Algeria	Es Senia	0.2	0.2	0.4	3	2	5	0.0	0.0	0.1	1	0	1
Algeria	Faidh El Botma	0.4	0.3	0.6	1	1	2	0.0	0.0	0.1	0	0	0
Algeria	Faidja	0.3	0.2	0.5	0	0	0	0.0	0.0	0.1	0	0	0
Algeria	Fellaoucene	0.3	0.2	0.4	0	0	0	0.0	0.0	0.1	0	0	0
Algeria	Fenoughil	0.4	0.2	0.5	1	0	1	0.0	0.0	0.1	0	0	0
Algeria	Feraoun	0.3	0.2	0.4	0	0	1	0.0	0.0	0.1	0	0	0
Algeria	Ferdjioua	0.3	0.2	0.5	2	1	2	0.0	0.0	0.1	0	0	0
Algeria	Ferkane	0.2	0.1	0.3	0	0	0	0.0	0.0	0.0	0	0	0
Algeria	Ferraguig	0.3	0.2	0.5	0	0	0	0.0	0.0	0.1	0	0	0
Algeria	Fesdis	0.3	0.2	0.5	0	0	0	0.0	0.0	0.1	0	0	0
Algeria	Filfila	0.3	0.2	0.4	1	0	1	0.0	0.0	0.1	0	0	0
Algeria	Fkirina	0.3	0.2	0.4	1	1	1	0.0	0.0	0.1	0	0	0
Algeria	Foggaret Azzouia	0.3	0.2	0.3	0	0	0	0.0	0.0	0.0	0	0	0
Algeria	Fornaka	0.3	0.2	0.4	0	0	1	0.0	0.0	0.1	0	0	0
Algeria	Foughala	0.3	0.2	0.4	0	0	1	0.0	0.0	0.1	0	0	0
Algeria	Fouka	0.3	0.2	0.5	1	1	1	0.0	0.0	0.1	0	0	0
Algeria	Foum Toub	0.3	0.2	0.4	0	0	0	0.0	0.0	0.1	0	0	0
Algeria	Freha	0.2	0.1	0.3	0	0	1	0.0	0.0	0.1	0	0	0
Algeria	Frenda	0.3	0.2	0.4	2	1	3	0.0	0.0	0.1	0	0	0
Algeria	Frikat	0.2	0.1	0.3	0	0	0	0.0	0.0	0.0	0	0	0
Algeria	Froha	0.3	0.2	0.5	1	0	1	0.0	0.0	0.1	0	0	0
Algeria	Gdyel	0.3	0.2	0.5	1	1	2	0.0	0.0	0.1	0	0	0
Algeria	Ghardaia	0.3	0.2	0.6	3	2	5	0.0	0.0	0.1	0	0	1
Algeria	Gharrous	0.4	0.2	0.6	0	0	0	0.0	0.0	0.1	0	0	0
Algeria	Ghassoul	0.3	0.2	0.5	0	0	0	0.0	0.0	0.1	0	0	0
Algeria	Ghazaouet	0.3	0.2	0.4	1	1	1	0.0	0.0	0.1	0	0	0
Algeria	Ghebala	0.3	0.2	0.4	0	0	0	0.0	0.0	0.1	0	0	0
Algeria	Gherouaou	0.3	0.2	0.4	1	1	1	0.0	0.0	0.1	0	0	0
Algeria	Ghessira	0.2	0.1	0.3	0	0	0	0.0	0.0	0.1	0	0	0
Algeria	Ghilassa	0.3	0.2	0.5	0	0	1	0.0	0.0	0.1	0	0	0
Algeria	Ghriss	0.3	0.2	0.5	1	1	1	0.0	0.0	0.1	0	0	0

Table 3: Diarrhoea Mortality by Unit *(continued)*

Country	Unit	2000 mortality mean rate (per 10,000)	2000 mortality lower rate (per 10,000)	2000 mortality upper rate (per 10,000)	2000 mortality mean count	2000 mortality lower count	2000 mortality upper count	2017 mortality mean rate (per 10,000)	2017 mortality lower rate (per 10,000)	2017 mortality upper rate (per 10,000)	2017 mortality mean count	2017 mortality lower count	2017 mortality upper count
Algeria	Gosbat	0.3	0.2	0.5	1	0	1	0.0	0.0	0.1	0	0	0
Algeria	Gouraya	0.3	0.2	0.5	1	0	1	0.0	0.0	0.1	0	0	0
Algeria	Grarem Gouga	0.3	0.2	0.4	1	1	2	0.0	0.0	0.1	0	0	0
Algeria	Guellal	0.3	0.2	0.4	1	0	1	0.0	0.0	0.1	0	0	0
Algeria	Guelma	0.3	0.1	0.4	2	1	3	0.0	0.0	0.1	0	0	1
Algeria	Guelta Zerka	0.3	0.2	0.4	1	1	1	0.0	0.0	0.1	0	0	0
Algeria	Gueltat Sidi Saad	0.4	0.2	0.5	1	0	1	0.0	0.0	0.1	0	0	0
Algeria	Guemar	0.3	0.2	0.5	1	1	2	0.0	0.0	0.1	0	0	0
Algeria	Guenzet Tassameurt	0.3	0.2	0.5	0	0	0	0.0	0.0	0.1	0	0	0
Algeria	Guerdjoum	0.3	0.2	0.5	0	0	0	0.0	0.0	0.1	0	0	0
Algeria	Guernini	0.4	0.3	0.6	0	0	0	0.0	0.0	0.1	0	0	0
Algeria	Guerrouma	0.2	0.1	0.4	0	0	1	0.0	0.0	0.1	0	0	0
Algeria	Guertoufa	0.3	0.2	0.5	0	0	1	0.0	0.0	0.1	0	0	0
Algeria	Guettara	0.3	0.2	0.5	1	0	1	0.0	0.0	0.1	0	0	0
Algeria	Guidjel	0.3	0.2	0.4	1	1	2	0.0	0.0	0.1	0	0	0
Algeria	Guiga	0.3	0.2	0.5	0	0	1	0.0	0.0	0.1	0	0	0
Algeria	Guorriguer	0.3	0.2	0.4	0	0	0	0.0	0.0	0.1	0	0	0
Algeria	Hacine	0.3	0.2	0.5	0	0	1	0.0	0.0	0.1	0	0	0
Algeria	Had Echkalla	0.3	0.2	0.4	0	0	0	0.0	0.0	0.1	0	0	0
Algeria	Had Sahary	0.4	0.2	0.5	1	1	2	0.0	0.0	0.1	0	0	0
Algeria	Haddada	0.3	0.2	0.4	0	0	0	0.0	0.0	0.1	0	0	0
Algeria	Hadj Mechri	0.4	0.2	0.5	0	0	0	0.0	0.0	0.1	0	0	0
Algeria	Hadjadj	0.3	0.2	0.5	1	0	1	0.0	0.0	0.1	0	0	0
Algeria	Hadjera Zerga	0.3	0.2	0.4	0	0	1	0.0	0.0	0.1	0	0	0
Algeria	Hadjout	0.2	0.2	0.4	1	1	2	0.0	0.0	0.1	0	0	0
Algeria	Hadjret Ennous	0.3	0.2	0.4	0	0	0	0.0	0.0	0.1	0	0	0
Algeria	Haizer	0.2	0.1	0.3	0	0	1	0.0	0.0	0.1	0	0	0
Algeria	Hamadi Krouma	0.3	0.2	0.4	1	1	2	0.0	0.0	0.1	0	0	0
Algeria	Hamadia	0.3	0.2	0.5	1	0	1	0.0	0.0	0.1	0	0	0
Algeria	Hamala	0.3	0.2	0.4	0	0	1	0.0	0.0	0.1	0	0	0
Algeria	Hamma	0.3	0.2	0.5	0	0	1	0.0	0.0	0.1	0	0	0
Algeria	Hamma Bouziane	0.3	0.2	0.4	2	1	3	0.0	0.0	0.1	0	0	1
Algeria	Hammadi	0.2	0.2	0.4	1	1	1	0.0	0.0	0.0	0	0	0
Algeria	Hammam Ben Salah	0.3	0.2	0.4	0	0	0	0.0	0.0	0.1	0	0	0
Algeria	Hammam Boughrara	0.3	0.2	0.4	0	0	1	0.0	0.0	0.1	0	0	0
Algeria	Hammam Bouhadjar	0.2	0.1	0.3	1	1	1	0.0	0.0	0.1	0	0	0
Algeria	Hammam Dalaa	0.3	0.2	0.4	1	1	2	0.0	0.0	0.0	0	0	0
Algeria	Hammam Debagh	0.3	0.2	0.4	0	0	1	0.0	0.0	0.1	0	0	0
Algeria	Hammam Guergour	0.3	0.2	0.5	1	0	1	0.0	0.0	0.1	0	0	0
Algeria	Hammam Melouane	0.3	0.2	0.5	0	0	1	0.0	0.0	0.1	0	0	0
Algeria	Hammam N'Bail	0.3	0.2	0.4	1	0	1	0.0	0.0	0.1	0	0	0
Algeria	Hammam Righa	0.3	0.2	0.4	0	0	0	0.0	0.0	0.0	0	0	0
Algeria	Hammam Soukhna	0.3	0.2	0.4	1	0	1	0.0	0.0	0.1	0	0	0
Algeria	Hammamet	0.2	0.1	0.3	0	0	1	0.0	0.0	0.1	0	0	0
Algeria	Hamraia	0.3	0.2	0.5	0	0	0	0.0	0.0	0.1	0	0	0
Algeria	Hamri	0.3	0.2	0.5	0	0	0	0.0	0.0	0.1	0	0	0
Algeria	Hamri	0.3	0.2	0.4	0	0	1	0.0	0.0	0.0	0	0	0
Algeria	Hanchir Toumghani	0.3	0.2	0.4	1	0	1	0.0	0.0	0.1	0	0	0
Algeria	Hanencha	0.3	0.2	0.4	1	1	1	0.0	0.0	0.1	0	0	0
Algeria	Hannacha	0.3	0.2	0.4	0	0	0	0.0	0.0	0.0	0	0	0
Algeria	Haraoua	0.3	0.2	0.4	1	1	2	0.0	0.0	0.0	0	0	0
Algeria	Haraza	0.3	0.2	0.4	0	0	0	0.0	0.0	0.1	0	0	0

Table 3: Diarrhoea Mortality by Unit *(continued)*

Country	Unit	2000 mortality mean rate (per 10,000)	lower rate (per 10,000)	upper rate (per 10,000)	mean count	lower count	upper count	2017 mortality mean rate (per 10,000)	lower rate (per 10,000)	upper rate (per 10,000)	mean count	lower count	upper count
Algeria	Harbil	0.3	0.2	0.5	0	0	0	0.0	0.0	0.1	0	0	0
Algeria	Harchoune	0.3	0.2	0.4	1	0	1	0.0	0.0	0.0	0	0	0
Algeria	Hasnaoua	0.3	0.2	0.5	1	1	1	0.0	0.0	0.1	0	0	0
Algeria	Hassaine|Beni Yahi	0.3	0.2	0.5	0	0	1	0.0	0.0	0.0	0	0	0
Algeria	Hassani Abdelkrim	0.3	0.2	0.5	1	1	1	0.0	0.0	0.1	0	0	0
Algeria	Hassasna	0.2	0.2	0.4	0	0	0	0.0	0.0	0.1	0	0	0
Algeria	Hassi Bahbah	0.4	0.3	0.6	4	3	6	0.0	0.0	0.1	1	0	1
Algeria	Hassi Ben Abdellah	0.3	0.2	0.4	0	0	0	0.0	0.0	0.0	0	0	0
Algeria	Hassi Ben Okba	0.3	0.2	0.5	1	0	1	0.0	0.0	0.1	0	0	0
Algeria	Hassi Bounif	0.3	0.2	0.5	1	1	2	0.0	0.0	0.1	0	0	0
Algeria	Hassi Dahou	0.3	0.2	0.4	0	0	0	0.0	0.0	0.1	0	0	0
Algeria	Hassi Delaa	0.3	0.2	0.4	0	0	1	0.0	0.0	0.0	0	0	0
Algeria	Hassi El Euch	0.3	0.2	0.5	1	0	1	0.0	0.0	0.0	0	0	0
Algeria	Hassi El Ghella	0.2	0.2	0.4	0	0	0	0.0	0.0	0.1	0	0	0
Algeria	Hassi Fedoul	0.4	0.3	0.6	1	1	1	0.0	0.0	0.1	0	0	0
Algeria	Hassi Fehal	0.3	0.2	0.5	0	0	0	0.0	0.0	0.1	0	0	0
Algeria	Hassi Gara	0.3	0.2	0.5	1	1	1	0.0	0.0	0.1	0	0	0
Algeria	Hassi Khalifa	0.3	0.2	0.4	1	1	2	0.0	0.0	0.1	0	0	0
Algeria	Hassi Mameche	0.3	0.2	0.5	1	1	2	0.0	0.0	0.1	0	0	0
Algeria	Hassi Mefsoukh	0.3	0.2	0.5	0	0	0	0.0	0.0	0.1	0	0	0
Algeria	Hassi Messaoud	0.3	0.2	0.4	2	1	2	0.0	0.0	0.0	0	0	0
Algeria	Hassi R'Mel	0.3	0.2	0.5	1	1	1	0.0	0.0	0.0	0	0	0
Algeria	Hassi Zehana	0.2	0.2	0.4	0	0	0	0.0	0.0	0.1	0	0	0
Algeria	Hattatba	0.3	0.2	0.4	1	1	1	0.0	0.0	0.1	0	0	0
Algeria	Helliopolis	0.3	0.1	0.4	1	1	1	0.0	0.0	0.1	0	0	0
Algeria	Hennaya	0.2	0.1	0.4	1	1	2	0.0	0.0	0.1	0	0	0
Algeria	Herenfa	0.2	0.2	0.4	1	0	1	0.0	0.0	0.1	0	0	0
Algeria	Hidoussa	0.3	0.2	0.5	0	0	0	0.0	0.0	0.1	0	0	0
Algeria	Hoceinia	0.3	0.2	0.4	0	0	0	0.0	0.0	0.0	0	0	0
Algeria	Honaine	0.2	0.2	0.4	0	0	0	0.0	0.0	0.1	0	0	0
Algeria	Houari Boumediene	0.3	0.2	0.4	0	0	0	0.0	0.0	0.1	0	0	0
Algeria	Hounet	0.3	0.2	0.4	0	0	0	0.0	0.0	0.1	0	0	0
Algeria	Ibn Ziad	0.3	0.2	0.4	1	0	1	0.0	0.0	0.1	0	0	0
Algeria	Iboudraren	0.2	0.1	0.3	0	0	0	0.0	0.0	0.1	0	0	0
Algeria	Ichmoul	0.3	0.2	0.4	0	0	1	0.0	0.0	0.1	0	0	0
Algeria	Idjeur	0.3	0.1	0.4	0	0	0	0.0	0.0	0.1	0	0	0
Algeria	Idles	0.2	0.2	0.3	0	0	0	0.0	0.0	0.0	0	0	0
Algeria	Ifelain Ilmathen	0.2	0.2	0.3	0	0	0	0.0	0.0	0.1	0	0	0
Algeria	Iferhounene	0.2	0.1	0.4	0	0	0	0.0	0.0	0.1	0	0	0
Algeria	Ifigha	0.2	0.1	0.3	0	0	0	0.0	0.0	0.1	0	0	0
Algeria	Iflissen	0.2	0.1	0.3	0	0	0	0.0	0.0	0.1	0	0	0
Algeria	Ighil-Ali	0.2	0.1	0.3	0	0	0	0.0	0.0	0.1	0	0	0
Algeria	Ighrem	0.2	0.1	0.4	0	0	0	0.0	0.0	0.1	0	0	0
Algeria	Igli	0.3	0.2	0.4	0	0	0	0.0	0.0	0.1	0	0	0
Algeria	Illilten	0.2	0.1	0.4	0	0	0	0.0	0.0	0.1	0	0	0
Algeria	Illizi	0.2	0.1	0.2	0	0	0	0.0	0.0	0.0	0	0	0
Algeria	Illoula Oumalou	0.2	0.1	0.4	0	0	0	0.0	0.0	0.1	0	0	0
Algeria	Imsouhal	0.2	0.1	0.4	0	0	0	0.0	0.0	0.1	0	0	0
Algeria	In Amenas	0.2	0.2	0.3	0	0	0	0.0	0.0	0.0	0	0	0
Algeria	In Ghar	0.3	0.2	0.4	0	0	1	0.0	0.0	0.1	0	0	0
Algeria	In Guezzam	0.2	0.1	0.2	0	0	0	0.0	0.0	0.0	0	0	0
Algeria	In M'Guel	0.2	0.2	0.3	0	0	0	0.0	0.0	0.0	0	0	0
Algeria	In Salah	0.3	0.2	0.4	1	1	2	0.0	0.0	0.1	0	0	0
Algeria	In Zghmir	0.3	0.2	0.5	1	0	1	0.0	0.0	0.1	0	0	0
Algeria	Inoughissen	0.3	0.2	0.5	0	0	0	0.0	0.0	0.1	0	0	0
Algeria	Irdjen	0.2	0.1	0.3	0	0	0	0.0	0.0	0.1	0	0	0

Table 3: Diarrhoea Mortality by Unit *(continued)*

Country	Unit	2000 mortality mean rate (per 10,000)	lower rate (per 10,000)	upper rate (per 10,000)	mean count	lower count	upper count	2017 mortality mean rate (per 10,000)	lower rate (per 10,000)	upper rate (per 10,000)	mean count	lower count	upper count
Algeria	Isser	0.3	0.2	0.4	1	1	1	0.0	0.0	0.1	0	0	0
Algeria	Jijel	0.3	0.2	0.4	3	2	5	0.0	0.0	0.1	1	0	1
Algeria	Kadiria	0.2	0.1	0.3	1	0	1	0.0	0.0	0.1	0	0	0
Algeria	Kais	0.2	0.2	0.4	1	0	1	0.0	0.0	0.1	0	0	0
Algeria	Kalaa	0.3	0.2	0.5	0	0	1	0.0	0.0	0.1	0	0	0
Algeria	Kalaat Bousbaa	0.3	0.2	0.4	0	0	0	0.0	0.0	0.1	0	0	0
Algeria	Kanoua	0.3	0.2	0.5	0	0	0	0.0	0.0	0.1	0	0	0
Algeria	Kasdir	0.3	0.2	0.5	0	0	0	0.0	0.0	0.1	0	0	0
Algeria	Kef El Ahmar	0.3	0.2	0.5	0	0	0	0.0	0.0	0.1	0	0	0
Algeria	Kenadsa	0.3	0.2	0.4	0	0	1	0.0	0.0	0.1	0	0	0
Algeria	Kendira	0.3	0.2	0.4	0	0	0	0.0	0.0	0.1	0	0	0
Algeria	Kerkera	0.3	0.2	0.4	1	0	1	0.0	0.0	0.1	0	0	0
Algeria	Kerzaz	0.2	0.2	0.3	0	0	0	0.0	0.0	0.0	0	0	0
Algeria	Khadra	0.3	0.2	0.5	1	0	1	0.0	0.0	0.1	0	0	0
Algeria	Khalouia	0.3	0.2	0.5	0	0	0	0.0	0.0	0.1	0	0	0
Algeria	Khams Djouamaa	0.3	0.2	0.4	0	0	0	0.0	0.0	0.1	0	0	0
Algeria	Khatouti Sed Eldjir	0.3	0.2	0.4	0	0	0	0.0	0.0	0.1	0	0	0
Algeria	Khedara	0.3	0.2	0.4	0	0	0	0.0	0.0	0.1	0	0	0
Algeria	Kheir Oued Adjoul	0.3	0.2	0.4	0	0	0	0.0	0.0	0.1	0	0	0
Algeria	Kheiredine	0.3	0.2	0.5	1	0	1	0.0	0.0	0.1	0	0	0
Algeria	Khelil	0.3	0.2	0.5	1	1	2	0.0	0.0	0.1	0	0	0
Algeria	Khemis El Khechna	0.3	0.2	0.4	2	1	3	0.0	0.0	0.1	0	0	1
Algeria	Khemis Miliana	0.3	0.2	0.5	2	1	3	0.0	0.0	0.1	0	0	0
Algeria	Khemissa	0.3	0.2	0.4	0	0	0	0.0	0.0	0.1	0	0	0
Algeria	Khemisti	0.3	0.2	0.5	1	0	1	0.0	0.0	0.1	0	0	0
Algeria	Khenchela	0.2	0.2	0.4	2	1	3	0.0	0.0	0.1	0	0	1
Algeria	Kheneg	0.3	0.2	0.4	0	0	1	0.0	0.0	0.0	0	0	0
Algeria	Kheng Maoun	0.3	0.2	0.5	0	0	0	0.0	0.0	0.1	0	0	0
Algeria	Khenguet Sidi Nadji	0.2	0.1	0.3	0	0	0	0.0	0.0	0.1	0	0	0
Algeria	Kherrata	0.3	0.2	0.4	1	0	1	0.0	0.0	0.1	0	0	0
Algeria	Khezzara	0.3	0.2	0.4	0	0	0	0.0	0.0	0.1	0	0	0
Algeria	Khirane	0.2	0.2	0.4	0	0	0	0.0	0.0	0.1	0	0	0
Algeria	Khoubana	0.3	0.2	0.4	0	0	0	0.0	0.0	0.1	0	0	0
Algeria	Khraicia	0.3	0.2	0.4	1	0	1	0.0	0.0	0.1	0	0	0
Algeria	Kimmel	0.3	0.2	0.4	0	0	0	0.0	0.0	0.1	0	0	0
Algeria	Kolea	0.3	0.2	0.4	2	1	2	0.0	0.0	0.1	0	0	0
Algeria	Kouas	0.3	0.2	0.4	1	0	1	0.0	0.0	0.1	0	0	0
Algeria	Kouba	0.3	0.2	0.4	13	8	21	0.0	0.0	0.1	1	1	2
Algeria	Kouinine	0.3	0.2	0.5	1	1	1	0.0	0.0	0.1	0	0	0
Algeria	Krakda	0.3	0.2	0.5	0	0	0	0.0	0.0	0.1	0	0	0
Algeria	Ksabi	0.2	0.2	0.4	0	0	0	0.0	0.0	0.0	0	0	0
Algeria	Ksar Bellezma	0.3	0.2	0.4	0	0	1	0.0	0.0	0.1	0	0	0
Algeria	Ksar Chellala	0.3	0.2	0.5	1	1	2	0.0	0.0	0.1	0	0	0
Algeria	Ksar El Abtal	0.3	0.2	0.4	1	1	1	0.0	0.0	0.1	0	0	0
Algeria	Ksar El Boukhari	0.3	0.2	0.4	1	1	2	0.0	0.0	0.1	0	0	0
Algeria	Ksar El Sbihi	0.3	0.2	0.4	0	0	1	0.0	0.0	0.1	0	0	0
Algeria	Ksar Hirane	0.3	0.2	0.5	1	0	1	0.0	0.0	0.0	0	0	0
Algeria	Ksar Kaddour	0.3	0.2	0.4	0	0	0	0.0	0.0	0.1	0	0	0
Algeria	Ksour	0.3	0.2	0.4	0	0	0	0.0	0.0	0.0	0	0	0
Algeria	Labiod Medjadja	0.3	0.2	0.4	1	0	1	0.0	0.0	0.0	0	0	0
Algeria	Lac Des Oiseaux	0.3	0.2	0.4	0	0	0	0.0	0.0	0.1	0	0	0
Algeria	Laghouat	0.3	0.2	0.4	5	3	7	0.0	0.0	0.0	1	0	1
Algeria	Lahlef	0.3	0.2	0.4	0	0	1	0.0	0.0	0.1	0	0	0
Algeria	Lahmar	0.3	0.2	0.4	0	0	0	0.0	0.0	0.1	0	0	0
Algeria	Lakhdaria	0.2	0.1	0.3	1	1	2	0.0	0.0	0.1	0	0	0
Algeria	Larbaa	0.3	0.2	0.4	2	1	3	0.0	0.0	0.1	1	0	1

Table 3: Diarrhoea Mortality by Unit *(continued)*

Country	Unit	2000 mortality mean rate (per 10,000)	lower rate (per 10,000)	upper rate (per 10,000)	mean count	lower count	upper count	2017 mortality mean rate (per 10,000)	lower rate (per 10,000)	upper rate (per 10,000)	mean count	lower count	upper count
Algeria	Larbaa	0.3	0.2	0.5	0	0	0	0.0	0.0	0.1	0	0	0
Algeria	Larbaa	0.3	0.2	0.4	0	0	0	0.0	0.0	0.1	0	0	0
Algeria	Larbaa-Nath-Irathen	0.2	0.1	0.3	0	0	1	0.0	0.0	0.1	0	0	0
Algeria	Larbatache	0.2	0.2	0.4	1	0	1	0.0	0.0	0.1	0	0	0
Algeria	Lardjem	0.3	0.2	0.4	0	0	0	0.0	0.0	0.0	1	0	1
Algeria	Larhat	0.3	0.2	0.4	0	0	0	0.0	0.0	0.1	0	0	0
Algeria	Layoune	0.3	0.2	0.5	1	0	1	0.0	0.0	0.1	0	0	0
Algeria	Lazharia	0.3	0.2	0.4	0	0	0	0.0	0.0	0.0	0	0	0
Algeria	Lazrou	0.3	0.2	0.4	0	0	0	0.0	0.0	0.1	0	0	0
Algeria	Leghata	0.3	0.2	0.4	0	0	1	0.0	0.0	0.1	0	0	0
Algeria	Lemsane	0.3	0.2	0.5	0	0	0	0.0	0.0	0.1	0	0	0
Algeria	Lemtar	0.2	0.2	0.4	0	0	0	0.0	0.0	0.1	0	0	0
Algeria	Les Eucalyptus	0.3	0.2	0.4	4	2	6	0.0	0.0	0.0	1	1	2
Algeria	Lichana	0.3	0.2	0.4	0	0	1	0.0	0.0	0.1	0	0	0
Algeria	Lioua	0.3	0.2	0.4	1	0	1	0.0	0.0	0.1	0	0	0
Algeria	M__Ziraa	0.2	0.1	0.3	0	0	0	0.0	0.0	0.1	0	0	0
Algeria	M'Chedallah	0.2	0.1	0.3	1	0	1	0.0	0.0	0.1	0	0	0
Algeria	M'Cid	0.3	0.2	0.5	0	0	0	0.0	0.0	0.1	0	0	0
Algeria	M'Cif	0.3	0.2	0.4	0	0	1	0.0	0.0	0.1	0	0	0
Algeria	M'Daourouche	0.3	0.2	0.4	1	1	1	0.0	0.0	0.1	0	0	0
Algeria	M'Doukal	0.3	0.2	0.4	0	0	0	0.0	0.0	0.0	0	0	0
Algeria	M'Kira	0.2	0.1	0.3	0	0	1	0.0	0.0	0.1	0	0	0
Algeria	M'Lili	0.3	0.2	0.4	0	0	0	0.0	0.0	0.1	0	0	0
Algeria	M'Liliha	0.4	0.3	0.6	1	1	1	0.0	0.0	0.1	0	0	0
Algeria	M'Naguer	0.3	0.2	0.4	1	0	1	0.0	0.0	0.1	0	0	0
Algeria	M'Rara	0.3	0.2	0.5	0	0	0	0.0	0.0	0.1	0	0	0
Algeria	M'Sara	0.3	0.2	0.4	0	0	0	0.0	0.0	0.1	0	0	0
Algeria	M'Sila	0.3	0.2	0.4	5	3	7	0.0	0.0	0.1	1	1	1
Algeria	M'Tarfa	0.3	0.2	0.4	1	1	1	0.0	0.0	0.1	0	0	0
Algeria	M'Toussa	0.3	0.2	0.4	0	0	0	0.0	0.0	0.1	0	0	0
Algeria	Maacem	0.3	0.2	0.4	0	0	0	0.0	0.0	0.1	0	0	0
Algeria	Maadid	0.3	0.2	0.4	1	1	1	0.0	0.0	0.0	0	0	0
Algeria	Maafa	0.3	0.2	0.4	0	0	0	0.0	0.0	0.1	0	0	0
Algeria	Maala	0.2	0.1	0.3	0	0	0	0.0	0.0	0.1	0	0	0
Algeria	Maamora	0.2	0.2	0.3	0	0	0	0.0	0.0	0.0	0	0	0
Algeria	Maamora	0.3	0.2	0.5	0	0	0	0.0	0.0	0.1	0	0	0
Algeria	Maaouia	0.3	0.2	0.4	0	0	1	0.0	0.0	0.1	0	0	0
Algeria	Maarif	0.3	0.2	0.4	0	0	1	0.0	0.0	0.1	0	0	0
Algeria	Maatkas	0.2	0.1	0.3	0	0	1	0.0	0.0	0.0	0	0	0
Algeria	Machroha	0.3	0.2	0.4	1	1	1	0.0	0.0	0.1	0	0	0
Algeria	Madna	0.3	0.2	0.5	0	0	0	0.0	0.0	0.1	0	0	0
Algeria	Maghnia	0.3	0.2	0.4	3	2	4	0.0	0.0	0.1	0	0	1
Algeria	Magra	0.3	0.2	0.4	1	1	2	0.0	0.0	0.0	0	0	0
Algeria	Magrane	0.3	0.2	0.4	1	1	1	0.0	0.0	0.1	0	0	0
Algeria	Magtaa Douz	0.3	0.2	0.5	0	0	1	0.0	0.0	0.1	0	0	0
Algeria	Mahdia	0.3	0.2	0.5	1	1	2	0.0	0.0	0.1	0	0	0
Algeria	Mahelma	0.3	0.2	0.4	1	0	1	0.0	0.0	0.1	0	0	0
Algeria	Makhda	0.3	0.2	0.5	0	0	0	0.0	0.0	0.1	0	0	0
Algeria	Makman Ben Amer	0.3	0.2	0.5	0	0	0	0.0	0.0	0.1	0	0	0
Algeria	Makouda	0.2	0.1	0.3	0	0	1	0.0	0.0	0.0	0	0	0
Algeria	Mansoura	0.3	0.2	0.5	0	0	0	0.0	0.0	0.1	0	0	0
Algeria	Mansoura	0.2	0.2	0.4	1	0	1	0.0	0.0	0.0	0	0	0
Algeria	Mansourah	0.3	0.2	0.5	1	0	1	0.0	0.0	0.1	0	0	0
Algeria	Mansourah	0.3	0.2	0.4	1	1	2	0.0	0.0	0.1	0	0	0
Algeria	Maouaklane	0.3	0.2	0.4	1	0	1	0.0	0.0	0.1	0	0	0
Algeria	Maoussa	0.3	0.2	0.5	1	1	1	0.0	0.0	0.1	0	0	0
Algeria	Marsa Ben M'Hidi	0.3	0.2	0.4	0	0	0	0.0	0.0	0.1	0	0	0

Table 3: Diarrhoea Mortality by Unit *(continued)*

Country	Unit	2000 mortality						2017 mortality					
		mean rate (per 10,000)	lower rate (per 10,000)	upper rate (per 10,000)	mean count	lower count	upper count	mean rate (per 10,000)	lower rate (per 10,000)	upper rate (per 10,000)	mean count	lower count	upper count
Algeria	Marsat El Hadjadj	0.3	0.2	0.4	0	0	1	0.0	0.0	0.1	0	0	0
Algeria	Mascara	0.3	0.2	0.5	3	2	5	0.0	0.0	0.1	1	0	1
Algeria	Mazouna	0.3	0.2	0.5	1	0	1	0.0	0.0	0.1	0	0	0
Algeria	Mecheria	0.3	0.2	0.5	2	1	3	0.0	0.0	0.1	0	0	1
Algeria	Mechouneche	0.2	0.2	0.3	0	0	0	0.0	0.0	0.1	0	0	0
Algeria	Mechraa Houari Boumediene	0.3	0.2	0.4	0	0	0	0.0	0.0	0.1	0	0	0
Algeria	Mechraa Safa	0.3	0.2	0.4	1	0	1	0.0	0.0	0.1	0	0	0
Algeria	Mechtrass	0.2	0.1	0.3	0	0	0	0.0	0.0	0.1	0	0	0
Algeria	Medea	0.3	0.2	0.4	3	2	5	0.0	0.0	0.0	0	0	1
Algeria	Mediouna	0.3	0.2	0.5	1	1	2	0.0	0.0	0.1	0	0	0
Algeria	Medjana	0.3	0.2	0.5	1	1	2	0.0	0.0	0.1	0	0	0
Algeria	Medjaz Ammar	0.3	0.2	0.4	0	0	0	0.0	0.0	0.1	0	0	0
Algeria	Medjaz Sfa	0.3	0.2	0.4	0	0	0	0.0	0.0	0.1	0	0	0
Algeria	Medjebar	0.3	0.2	0.4	0	0	0	0.0	0.0	0.0	0	0	0
Algeria	Medjedel	0.3	0.2	0.4	1	0	1	0.0	0.0	0.0	0	0	0
Algeria	Medrissa	0.3	0.2	0.5	1	0	1	0.0	0.0	0.1	0	0	0
Algeria	Medroussa	0.3	0.2	0.4	0	0	1	0.0	0.0	0.1	0	0	0
Algeria	Meftah	0.2	0.2	0.4	2	1	2	0.0	0.0	0.1	0	0	1
Algeria	Meftaha	0.3	0.2	0.4	0	0	0	0.0	0.0	0.0	0	0	0
Algeria	Megarine	0.2	0.2	0.3	0	0	1	0.0	0.0	0.1	0	0	0
Algeria	Megheraoua	0.3	0.2	0.4	0	0	0	0.0	0.0	0.1	0	0	0
Algeria	Meghila	0.3	0.2	0.4	0	0	0	0.0	0.0	0.1	0	0	0
Algeria	Mekhadma	0.3	0.2	0.4	0	0	0	0.0	0.0	0.1	0	0	0
Algeria	Mekhareg	0.3	0.2	0.5	1	0	1	0.0	0.0	0.0	0	0	0
Algeria	Mekhatria	0.3	0.2	0.5	1	0	1	0.0	0.0	0.1	0	0	0
Algeria	Mekkedra	0.3	0.2	0.4	0	0	0	0.0	0.0	0.1	0	0	0
Algeria	Mekla	0.2	0.1	0.3	0	0	1	0.0	0.0	0.0	0	0	0
Algeria	Melaab	0.3	0.2	0.4	0	0	0	0.0	0.0	0.1	0	0	0
Algeria	Melbou	0.2	0.1	0.4	0	0	0	0.0	0.0	0.1	0	0	0
Algeria	Mellakou	0.3	0.2	0.4	1	0	1	0.0	0.0	0.1	0	0	0
Algeria	Menaa	0.3	0.2	0.4	0	0	1	0.0	0.0	0.0	0	0	0
Algeria	Menaa	0.3	0.2	0.4	0	0	1	0.0	0.0	0.1	0	0	0
Algeria	Menaceur	0.3	0.2	0.4	1	1	1	0.0	0.0	0.1	0	0	0
Algeria	Mendes	0.3	0.2	0.4	0	0	0	0.0	0.0	0.1	0	0	0
Algeria	Merad	0.2	0.2	0.4	1	0	1	0.0	0.0	0.0	0	0	0
Algeria	Merahna	0.3	0.2	0.4	0	0	1	0.0	0.0	0.1	0	0	0
Algeria	Merdja Sidi Abed	0.2	0.2	0.4	0	0	0	0.0	0.0	0.1	0	0	0
Algeria	Merhoum	0.3	0.2	0.4	0	0	0	0.0	0.0	0.1	0	0	0
Algeria	Meridja	0.3	0.2	0.4	0	0	0	0.0	0.0	0.1	0	0	0
Algeria	Merine	0.3	0.2	0.4	0	0	0	0.0	0.0	0.1	0	0	0
Algeria	Merouana	0.3	0.2	0.4	1	1	2	0.0	0.0	0.1	0	0	0
Algeria	Mers El Kebir	0.2	0.1	0.3	1	0	1	0.0	0.0	0.1	0	0	0
Algeria	Meskiana	0.3	0.2	0.4	1	1	1	0.0	0.0	0.1	0	0	0
Algeria	Mesra	0.3	0.2	0.5	1	0	1	0.0	0.0	0.1	0	0	0
Algeria	Messaad	0.4	0.2	0.5	5	3	7	0.0	0.0	0.0	1	0	1
Algeria	Messelmoun	0.3	0.2	0.5	0	0	0	0.0	0.0	0.1	0	0	0
Algeria	Metarfa	0.3	0.2	0.4	0	0	0	0.0	0.0	0.1	0	0	0
Algeria	Metlili	0.3	0.2	0.5	2	1	3	0.0	0.0	0.1	0	0	0
Algeria	Mezaourou	0.3	0.2	0.4	0	0	0	0.0	0.0	0.1	0	0	0
Algeria	Mezdour	0.3	0.2	0.4	0	0	0	0.0	0.0	0.1	0	0	0
Algeria	Mezghrane	0.3	0.2	0.4	1	1	1	0.0	0.0	0.1	0	0	0
Algeria	Mezloug	0.3	0.2	0.4	1	1	1	0.0	0.0	0.1	0	0	0
Algeria	Mezrenna	0.3	0.2	0.4	0	0	0	0.0	0.0	0.1	0	0	0
Algeria	Mih Ouansa	0.3	0.2	0.4	1	0	1	0.0	0.0	0.1	0	0	0
Algeria	Mihoub	0.3	0.2	0.4	0	0	1	0.0	0.0	0.1	0	0	0
Algeria	Mila	0.3	0.2	0.5	2	1	4	0.0	0.0	0.1	0	0	1
Algeria	Miliana	0.3	0.2	0.5	2	1	2	0.0	0.0	0.1	0	0	0
Algeria	Minar Zarza	0.3	0.2	0.5	1	0	1	0.0	0.0	0.1	0	0	0

Table 3: Diarrhoea Mortality by Unit *(continued)*

Country	Unit	2000 mortality mean rate (per 10,000)	lower rate (per 10,000)	upper rate (per 10,000)	mean count	lower count	upper count	2017 mortality mean rate (per 10,000)	lower rate (per 10,000)	upper rate (per 10,000)	mean count	lower count	upper count
Algeria	Misserghin	0.2	0.1	0.3	1	1	2	0.0	0.0	0.1	0	0	0
Algeria	Mizrana	0.2	0.1	0.3	0	0	0	0.0	0.0	0.1	0	0	0
Algeria	Mogheul	0.3	0.2	0.4	0	0	0	0.0	0.0	0.1	0	0	0
Algeria	Moghrar	0.3	0.2	0.4	0	0	0	0.0	0.0	0.1	0	0	0
Algeria	Mohamed Boudiaf	0.3	0.2	0.4	0	0	1	0.0	0.0	0.0	0	0	0
Algeria	Mohammadia	0.3	0.2	0.5	3	2	4	0.0	0.0	0.1	0	0	1
Algeria	Morsot	0.2	0.2	0.4	0	0	1	0.0	0.0	0.1	0	0	0
Algeria	Mostaganem	0.3	0.2	0.4	4	2	6	0.0	0.0	0.1	1	0	1
Algeria	Moudjebara	0.4	0.3	0.6	1	1	1	0.0	0.0	0.1	0	0	0
Algeria	Moulay Larbi	0.3	0.2	0.4	0	0	1	0.0	0.0	0.1	0	0	0
Algeria	Moulay Slissen	0.3	0.2	0.4	0	0	0	0.0	0.0	0.1	0	0	0
Algeria	Moussadek	0.3	0.2	0.4	0	0	0	0.0	0.0	0.1	0	0	0
Algeria	Mouzaia	0.2	0.1	0.3	1	1	2	0.0	0.0	0.1	0	0	0
Algeria	Msirda Fouaga	0.3	0.2	0.4	0	0	0	0.0	0.0	0.1	0	0	0
Algeria	Mustafa Ben Brahim	0.3	0.2	0.5	0	0	0	0.0	0.0	0.1	0	0	0
Algeria	N'Gaous	0.3	0.2	0.5	1	1	1	0.0	0.0	0.1	0	0	0
Algeria	N'Goussa	0.3	0.2	0.4	1	0	1	0.0	0.0	0.1	0	0	0
Algeria	Naama	0.3	0.2	0.4	0	0	1	0.0	0.0	0.1	0	0	0
Algeria	Naciria	0.2	0.1	0.4	1	0	1	0.0	0.0	0.1	0	0	0
Algeria	Nador	0.3	0.2	0.4	0	0	0	0.0	0.0	0.1	0	0	0
Algeria	Nadorah	0.3	0.2	0.5	0	0	0	0.0	0.0	0.1	0	0	0
Algeria	Naima	0.3	0.2	0.5	0	0	1	0.0	0.0	0.1	0	0	0
Algeria	Nakhla	0.3	0.2	0.4	0	0	1	0.0	0.0	0.1	0	0	0
Algeria	Nechemaya	0.3	0.2	0.4	0	0	0	0.0	0.0	0.1	0	0	0
Algeria	Nedroma	0.3	0.2	0.4	1	1	1	0.0	0.0	0.1	0	0	0
Algeria	Negrine	0.2	0.1	0.2	0	0	0	0.0	0.0	0.0	0	0	0
Algeria	Nekmaria	0.3	0.2	0.5	0	0	1	0.0	0.0	0.1	0	0	0
Algeria	Nesmoth	0.3	0.2	0.5	0	0	0	0.0	0.0	0.1	0	0	0
Algeria	Nezla	0.3	0.2	0.4	2	1	2	0.0	0.0	0.0	0	0	0
Algeria	Oggaz	0.3	0.2	0.5	1	0	1	0.0	0.0	0.1	0	0	0
Algeria	Ogla Melha	0.2	0.1	0.3	0	0	0	0.0	0.0	0.1	0	0	0
Algeria	Oran	0.2	0.1	0.3	15	9	22	0.0	0.0	0.1	2	1	3
Algeria	Ouacif	0.2	0.1	0.3	0	0	0	0.0	0.0	0.1	0	0	0
Algeria	Ouadhia	0.2	0.1	0.3	0	0	0	0.0	0.0	0.0	0	0	0
Algeria	Ouaguenoun	0.2	0.1	0.3	0	0	0	0.0	0.0	0.0	0	0	0
Algeria	Ouamri	0.3	0.2	0.4	0	0	1	0.0	0.0	0.0	0	0	0
Algeria	Ouanougha	0.3	0.2	0.4	1	0	1	0.0	0.0	0.1	0	0	0
Algeria	Ouargla	0.3	0.2	0.4	5	3	7	0.0	0.0	0.1	1	0	1
Algeria	Ouarizane	0.3	0.2	0.4	1	0	1	0.0	0.0	0.1	0	0	0
Algeria	Oudjana	0.3	0.2	0.4	0	0	1	0.0	0.0	0.1	0	0	0
Algeria	Oued Athmenia	0.3	0.2	0.5	1	1	2	0.0	0.0	0.1	0	0	0
Algeria	Oued Berkeche	0.2	0.1	0.4	0	0	0	0.0	0.0	0.1	0	0	0
Algeria	Oued Chaaba	0.3	0.2	0.4	2	1	3	0.0	0.0	0.1	0	0	1
Algeria	Oued Cheham	0.3	0.2	0.4	0	0	1	0.0	0.0	0.1	0	0	0
Algeria	Oued Chorfa	0.3	0.2	0.5	0	0	1	0.0	0.0	0.0	0	0	0
Algeria	Oued Chouly	0.3	0.2	0.4	0	0	0	0.0	0.0	0.1	0	0	0
Algeria	Oued Djemaa	0.3	0.2	0.5	0	0	1	0.0	0.0	0.1	0	0	0
Algeria	Oued Djer	0.3	0.2	0.4	1	1	2	0.0	0.0	0.1	0	0	0
Algeria	Oued El Abtal	0.3	0.2	0.5	1	0	1	0.0	0.0	0.1	0	0	0
Algeria	Oued El Alenda	0.3	0.2	0.4	1	0	1	0.0	0.0	0.1	0	0	0
Algeria	Oued El Alleug	0.3	0.2	0.4	0	0	1	0.0	0.0	0.1	0	0	0
Algeria	Oued El Aneb	0.3	0.2	0.4	0	0	1	0.0	0.0	0.1	0	0	0
Algeria	Oued El Barad	0.3	0.2	0.4	0	0	0	0.0	0.0	0.1	0	0	0
Algeria	Oued El Berdi	0.3	0.2	0.4	0	0	0	0.0	0.0	0.1	0	0	0
Algeria	Oued El Djemaa	0.3	0.2	0.5	1	0	1	0.0	0.0	0.1	0	0	0
Algeria	Oued El Kheir	0.3	0.2	0.5	1	0	1	0.0	0.0	0.1	0	0	0
Algeria	Oued El Ma	0.3	0.2	0.4	1	0	1	0.0	0.0	0.1	0	0	0
Algeria	Oued Endja	0.3	0.2	0.5	1	0	1	0.0	0.0	0.1	0	0	0

Table 3: Diarrhoea Mortality by Unit *(continued)*

		2000 mortality						2017 mortality					
Country	Unit	mean rate (per 10,000)	lower rate (per 10,000)	upper rate (per 10,000)	mean count	lower count	upper count	mean rate (per 10,000)	lower rate (per 10,000)	upper rate (per 10,000)	mean count	lower count	upper count
Algeria	Oued Essalem	0.3	0.2	0.4	0	0	1	0.0	0.0	0.1	0	0	0
Algeria	Oued Fodda	0.3	0.2	0.4	1	1	2	0.0	0.0	0.0	0	0	0
Algeria	Oued Fragha	0.3	0.2	0.4	0	0	0	0.0	0.0	0.1	0	0	0
Algeria	Oued Ghir	0.2	0.1	0.4	0	0	1	0.0	0.0	0.1	0	0	0
Algeria	Oued Goussine	0.3	0.2	0.4	0	0	0	0.0	0.0	0.0	0	0	0
Algeria	Oued Harbil	0.2	0.2	0.4	0	0	0	0.0	0.0	0.0	0	0	0
Algeria	Oued Kebrit	0.3	0.2	0.4	0	0	0	0.0	0.0	0.1	0	0	0
Algeria	Oued Lilli	0.3	0.2	0.5	1	1	1	0.0	0.0	0.1	0	0	0
Algeria	Oued M'Zi	0.4	0.2	0.5	0	0	0	0.0	0.0	0.1	0	0	0
Algeria	Oued Mora	0.4	0.2	0.6	0	0	0	0.0	0.0	0.1	0	0	0
Algeria	Oued Nini	0.3	0.2	0.4	0	0	0	0.0	0.0	0.1	0	0	0
Algeria	Oued Rhiou	0.2	0.1	0.4	2	1	3	0.0	0.0	0.1	0	0	0
Algeria	Oued Sebaa	0.3	0.2	0.4	0	0	0	0.0	0.0	0.1	0	0	0
Algeria	Oued Sebbah	0.2	0.1	0.3	0	0	0	0.0	0.0	0.1	0	0	0
Algeria	Oued Sefioune	0.3	0.2	0.4	0	0	0	0.0	0.0	0.1	0	0	0
Algeria	Oued Seguen	0.3	0.2	0.4	1	0	1	0.0	0.0	0.1	0	0	0
Algeria	Oued Sly	0.2	0.2	0.4	1	1	2	0.0	0.0	0.1	0	0	0
Algeria	Oued Taga	0.3	0.2	0.4	1	0	1	0.0	0.0	0.1	0	0	0
Algeria	Oued Taourira	0.3	0.2	0.4	0	0	0	0.0	0.0	0.1	0	0	0
Algeria	Oued Taria	0.3	0.2	0.5	1	0	1	0.0	0.0	0.1	0	0	0
Algeria	Oued Tlelat	0.3	0.2	0.5	1	0	1	0.0	0.0	0.1	0	0	0
Algeria	Oued Zenati	0.3	0.2	0.4	1	1	1	0.0	0.0	0.1	0	0	0
Algeria	Oued Zhour	0.3	0.2	0.4	0	0	0	0.0	0.0	0.1	0	0	0
Algeria	Oued Zitoun	0.3	0.2	0.4	0	0	0	0.0	0.0	0.1	0	0	0
Algeria	Ouenza	0.3	0.2	0.4	1	1	2	0.0	0.0	0.1	0	0	0
Algeria	Ouezra	0.2	0.2	0.4	0	0	1	0.0	0.0	0.0	0	0	0
Algeria	Ouillen	0.3	0.2	0.4	0	0	0	0.0	0.0	0.1	0	0	0
Algeria	Ouldja Boulbalout	0.3	0.2	0.4	0	0	0	0.0	0.0	0.1	0	0	0
Algeria	Ouled Abbes	0.3	0.2	0.4	0	0	0	0.0	0.0	0.0	0	0	0
Algeria	Ouled Addi Guebala	0.3	0.2	0.4	1	1	1	0.0	0.0	0.0	0	0	0
Algeria	Ouled Addouane	0.3	0.2	0.5	0	0	1	0.0	0.0	0.1	0	0	0
Algeria	Ouled Ahmed Temmi	0.3	0.2	0.4	1	1	2	0.0	0.0	0.1	0	0	0
Algeria	Ouled Aissa	0.3	0.2	0.4	0	0	0	0.0	0.0	0.1	0	0	0
Algeria	Ouled Aissa	0.3	0.2	0.4	0	0	0	0.0	0.0	0.1	0	0	0
Algeria	Ouled Ammar	0.3	0.2	0.4	0	0	0	0.0	0.0	0.1	0	0	0
Algeria	Ouled Antar	0.3	0.2	0.5	0	0	0	0.0	0.0	0.1	0	0	0
Algeria	Ouled Aouf	0.3	0.2	0.5	0	0	0	0.0	0.0	0.1	0	0	0
Algeria	Ouled Attia	0.3	0.2	0.5	0	0	1	0.1	0.0	0.1	0	0	0
Algeria	Ouled Ben Abdelkader	0.3	0.2	0.4	1	0	1	0.0	0.0	0.1	0	0	0
Algeria	Ouled Bessem	0.3	0.2	0.4	0	0	0	0.0	0.0	0.1	0	0	0
Algeria	Ouled Bouachra	0.3	0.2	0.4	0	0	0	0.0	0.0	0.0	0	0	0
Algeria	Ouled Boudjemaa	0.2	0.2	0.4	0	0	0	0.0	0.0	0.1	0	0	0
Algeria	Ouled Boughalem	0.3	0.2	0.5	0	0	1	0.0	0.0	0.0	0	0	0
Algeria	Ouled Brahem	0.3	0.2	0.5	0	0	1	0.0	0.0	0.1	0	0	0
Algeria	Ouled Brahim	0.3	0.2	0.4	0	0	1	0.0	0.0	0.0	0	0	0
Algeria	Ouled Brahim	0.3	0.2	0.5	1	0	1	0.0	0.0	0.1	0	0	0
Algeria	Ouled Chebel	0.3	0.2	0.4	1	0	1	0.0	0.0	0.1	0	0	0
Algeria	Ouled Dahmane	0.3	0.2	0.5	0	0	1	0.0	0.0	0.1	0	0	0
Algeria	Ouled Daid	0.3	0.2	0.4	0	0	0	0.0	0.0	0.0	0	0	0
Algeria	Ouled Derradj	0.3	0.2	0.4	1	0	1	0.0	0.0	0.0	0	0	0
Algeria	Ouled Djellal	0.3	0.2	0.4	2	1	3	0.0	0.0	0.1	0	0	1
Algeria	Ouled Driss	0.3	0.2	0.4	1	0	1	0.0	0.0	0.1	0	0	0
Algeria	Ouled Fadhel	0.3	0.2	0.4	0	0	0	0.0	0.0	0.1	0	0	0
Algeria	Ouled Fares	0.3	0.2	0.4	1	1	2	0.0	0.0	0.1	0	0	0
Algeria	Ouled Fayet	0.3	0.2	0.4	1	1	1	0.0	0.0	0.1	0	0	0
Algeria	Ouled Gacem	0.3	0.2	0.5	0	0	0	0.0	0.0	0.1	0	0	0

Table 3: Diarrhoea Mortality by Unit *(continued)*

Country	Unit	2000 mortality mean rate (per 10,000)	lower rate (per 10,000)	upper rate (per 10,000)	mean count	lower count	upper count	2017 mortality mean rate (per 10,000)	lower rate (per 10,000)	upper rate (per 10,000)	mean count	lower count	upper count
Algeria	Ouled Hamla	0.3	0.2	0.5	0	0	1	0.0	0.0	0.1	0	0	0
Algeria	Ouled Hebbaba	0.3	0.2	0.4	0	0	0	0.0	0.0	0.1	0	0	0
Algeria	Ouled Hedadj	0.3	0.2	0.4	1	1	1	0.0	0.0	0.1	0	0	0
Algeria	Ouled Hellal	0.3	0.2	0.5	0	0	0	0.0	0.0	0.1	0	0	0
Algeria	Ouled Khaled	0.3	0.2	0.4	1	1	2	0.0	0.0	0.1	0	0	0
Algeria	Ouled Khelouf	0.3	0.2	0.4	0	0	1	0.0	0.0	0.1	0	0	0
Algeria	Ouled Khoudir	0.2	0.2	0.4	0	0	0	0.0	0.0	0.0	0	0	0
Algeria	Ouled Kihel	0.2	0.2	0.4	0	0	0	0.0	0.0	0.1	0	0	0
Algeria	Ouled Maalah	0.3	0.2	0.5	0	0	0	0.0	0.0	0.1	0	0	0
Algeria	Ouled Maaraf	0.2	0.2	0.4	0	0	0	0.0	0.0	0.0	0	0	0
Algeria	Ouled Madhi	0.3	0.2	0.4	0	0	0	0.0	0.0	0.1	0	0	0
Algeria	Ouled Mansour	0.3	0.2	0.4	0	0	0	0.0	0.0	0.1	0	0	0
Algeria	Ouled Mimoun	0.2	0.2	0.4	1	0	1	0.0	0.0	0.1	0	0	0
Algeria	Ouled Moumen	0.3	0.2	0.4	0	0	0	0.0	0.0	0.1	0	0	0
Algeria	Ouled Moussa	0.3	0.2	0.4	1	1	1	0.0	0.0	0.1	0	0	0
Algeria	Ouled Rabah	0.3	0.2	0.5	0	0	1	0.0	0.0	0.1	0	0	0
Algeria	Ouled Rached	0.3	0.2	0.4	0	0	0	0.0	0.0	0.1	0	0	0
Algeria	Ouled Rahmoune	0.3	0.2	0.4	1	1	1	0.0	0.0	0.1	0	0	0
Algeria	Ouled Rechache	0.3	0.2	0.4	1	0	1	0.0	0.0	0.1	0	0	0
Algeria	Ouled Riyah	0.3	0.2	0.4	0	0	0	0.0	0.0	0.1	0	0	0
Algeria	Ouled Saber	0.3	0.2	0.4	0	0	1	0.0	0.0	0.1	0	0	0
Algeria	Ouled Said	0.3	0.2	0.4	0	0	0	0.0	0.0	0.1	0	0	0
Algeria	Ouled Sellem	0.3	0.2	0.4	1	0	1	0.0	0.0	0.1	0	0	0
Algeria	Ouled Si Ahmed	0.3	0.2	0.5	0	0	1	0.0	0.0	0.1	0	0	0
Algeria	Ouled Si Slimane	0.3	0.2	0.5	1	0	1	0.0	0.0	0.1	0	0	0
Algeria	Ouled Sidi Brahim	0.2	0.1	0.4	0	0	0	0.0	0.0	0.1	0	0	0
Algeria	Ouled Sidi Brahim	0.3	0.2	0.4	0	0	1	0.0	0.0	0.0	0	0	0
Algeria	Ouled Sidi Mihoub	0.3	0.2	0.4	0	0	0	0.0	0.0	0.1	0	0	0
Algeria	Ouled Slama	0.3	0.2	0.4	1	1	2	0.0	0.0	0.1	0	0	0
Algeria	Ouled Slimane	0.3	0.2	0.4	0	0	0	0.0	0.0	0.0	0	0	0
Algeria	Ouled Tebben	0.3	0.2	0.5	0	0	1	0.0	0.0	0.1	0	0	0
Algeria	Ouled Yahia Khadrouche	0.3	0.2	0.4	1	0	1	0.0	0.0	0.1	0	0	0
Algeria	Ouled Yaich	0.3	0.2	0.4	1	1	2	0.0	0.0	0.1	0	0	0
Algeria	Ouled Yaich	0.3	0.2	0.4	0	0	0	0.0	0.0	0.1	0	0	0
Algeria	Ouled Zaoui	0.3	0.2	0.4	0	0	0	0.0	0.0	0.1	0	0	0
Algeria	Oulhaca El Gheraba	0.2	0.2	0.4	0	0	1	0.0	0.0	0.1	0	0	0
Algeria	Oultene	0.3	0.2	0.4	0	0	0	0.0	0.0	0.0	0	0	0
Algeria	Oum Ali	0.2	0.1	0.2	0	0	0	0.0	0.0	0.1	0	0	0
Algeria	Oum Drou	0.2	0.2	0.4	1	0	1	0.0	0.0	0.0	0	0	0
Algeria	Oum El Adhaim	0.3	0.2	0.4	0	0	0	0.0	0.0	0.1	0	0	0
Algeria	Oum El Assel	0.3	0.2	0.3	0	0	0	0.0	0.0	0.0	0	0	0
Algeria	Oum El Bouaghi	0.3	0.2	0.4	2	1	4	0.0	0.0	0.1	0	0	1
Algeria	Oum El Djellil	0.3	0.2	0.5	0	0	0	0.0	0.0	0.1	0	0	0
Algeria	Oum Laadham	0.3	0.2	0.5	1	1	1	0.0	0.0	0.1	0	0	0
Algeria	Oum Toub	0.3	0.2	0.4	1	1	2	0.0	0.0	0.1	0	0	0
Algeria	Oum Touyour	0.3	0.2	0.5	1	0	1	0.0	0.0	0.1	0	0	0
Algeria	Oumache	0.3	0.2	0.4	0	0	1	0.0	0.0	0.1	0	0	0
Algeria	Ourlal	0.3	0.2	0.4	0	0	0	0.0	0.0	0.1	0	0	0
Algeria	Ourmes	0.3	0.2	0.4	0	0	0	0.0	0.0	0.1	0	0	0
Algeria	Ouyoun El Assafir	0.3	0.2	0.5	0	0	1	0.0	0.0	0.1	0	0	0
Algeria	Ouzzelaguen	0.2	0.2	0.4	0	0	1	0.0	0.0	0.1	0	0	0
Algeria	Rabta	0.3	0.2	0.4	0	0	0	0.0	0.0	0.1	0	0	0
Algeria	Ragouba	0.3	0.2	0.4	0	0	0	0.0	0.0	0.1	0	0	0
Algeria	Rahouia	0.3	0.2	0.4	1	0	1	0.0	0.0	0.1	0	0	0

Table 3: Diarrhoea Mortality by Unit *(continued)*

Country	Unit	2000 mortality mean rate (per 10,000)	lower rate (per 10,000)	upper rate (per 10,000)	mean count	lower count	upper count	2017 mortality mean rate (per 10,000)	lower rate (per 10,000)	upper rate (per 10,000)	mean count	lower count	upper count
Algeria	Ramdane Djamel	0.3	0.2	0.4	1	0	1	0.0	0.0	0.1	0	0	0
Algeria	Ramka	0.3	0.2	0.4	0	0	0	0.0	0.0	0.1	0	0	0
Algeria	Raml Souk	0.3	0.2	0.3	0	0	0	0.0	0.0	0.1	0	0	0
Algeria	Raouraoua	0.2	0.2	0.4	0	0	0	0.0	0.0	0.1	0	0	0
Algeria	Ras Ain Amirouche	0.3	0.2	0.5	0	0	1	0.0	0.0	0.1	0	0	0
Algeria	Ras El Agba	0.3	0.2	0.4	0	0	0	0.0	0.0	0.1	0	0	0
Algeria	Ras El Aioun	0.3	0.2	0.5	1	0	1	0.0	0.0	0.1	0	0	0
Algeria	Ras El Ma	0.3	0.2	0.5	0	0	1	0.0	0.0	0.1	0	0	0
Algeria	Ras El Oued	0.3	0.2	0.5	2	1	3	0.0	0.0	0.1	0	0	0
Algeria	Ras Miaad|Ouled Sassi	0.3	0.2	0.4	0	0	0	0.0	0.0	0.0	0	0	0
Algeria	Rechaiga	0.3	0.2	0.4	1	1	1	0.0	0.0	0.1	0	0	0
Algeria	Redjem Demouche	0.3	0.2	0.5	0	0	0	0.0	0.0	0.1	0	0	0
Algeria	Reggane	0.2	0.2	0.3	1	0	1	0.0	0.0	0.0	0	0	0
Algeria	Reghaia	0.3	0.2	0.4	2	1	3	0.0	0.0	0.1	0	0	0
Algeria	Reguiba	0.3	0.2	0.5	2	1	2	0.0	0.0	0.1	0	0	0
Algeria	Rehbat	0.3	0.2	0.4	0	0	1	0.0	0.0	0.1	0	0	0
Algeria	Relizane	0.3	0.2	0.5	4	2	6	0.0	0.0	0.1	1	0	1
Algeria	Remchi	0.2	0.2	0.4	1	1	2	0.0	0.0	0.1	0	0	0
Algeria	Remila	0.3	0.2	0.4	0	0	1	0.0	0.0	0.1	0	0	0
Algeria	Ridane	0.3	0.2	0.4	0	0	0	0.0	0.0	0.1	0	0	0
Algeria	Robbah	0.3	0.2	0.4	1	1	2	0.0	0.0	0.1	0	0	0
Algeria	Rogassa	0.3	0.2	0.5	0	0	0	0.0	0.0	0.1	0	0	0
Algeria	Roknia	0.3	0.2	0.4	0	0	0	0.0	0.0	0.1	0	0	0
Algeria	Rosfa	0.3	0.2	0.5	1	0	1	0.0	0.0	0.1	0	0	0
Algeria	Rouached	0.3	0.2	0.5	1	1	1	0.0	0.0	0.1	0	0	0
Algeria	Roubia	0.2	0.2	0.4	0	0	0	0.0	0.0	0.0	0	0	0
Algeria	Rouiba	0.2	0.2	0.4	2	2	4	0.0	0.0	0.0	0	0	1
Algeria	Rouina	0.3	0.2	0.4	1	0	1	0.0	0.0	0.0	0	0	0
Algeria	Rouissat	0.3	0.2	0.4	2	1	3	0.0	0.0	0.1	0	0	1
Algeria	Sabra	0.3	0.2	0.4	1	0	1	0.0	0.0	0.1	0	0	0
Algeria	Safel El Ouiden	0.3	0.2	0.4	0	0	0	0.0	0.0	0.1	0	0	0
Algeria	Safsaf	0.3	0.2	0.5	0	0	1	0.0	0.0	0.1	0	0	0
Algeria	Safsaf El Ouesra	0.2	0.1	0.2	0	0	0	0.0	0.0	0.1	0	0	0
Algeria	Saharidj	0.2	0.1	0.3	0	0	0	0.0	0.0	0.1	0	0	0
Algeria	Saida	0.3	0.2	0.4	4	2	6	0.0	0.0	0.1	1	0	1
Algeria	Salah Bey	0.3	0.2	0.5	1	0	1	0.0	0.0	0.1	0	0	0
Algeria	Salah Bouchaour	0.3	0.2	0.4	1	1	1	0.0	0.0	0.1	0	0	0
Algeria	Sali	0.3	0.2	0.4	1	0	1	0.0	0.0	0.0	0	0	0
Algeria	Saneg	0.3	0.2	0.4	0	0	0	0.0	0.0	0.1	0	0	0
Algeria	Saoula	0.3	0.2	0.4	2	1	3	0.0	0.0	0.1	0	0	1
Algeria	Sayada	0.3	0.2	0.4	2	1	2	0.0	0.0	0.1	0	0	0
Algeria	Sebaa	0.3	0.2	0.5	0	0	0	0.0	0.0	0.0	0	0	0
Algeria	Sebaine	0.3	0.2	0.4	0	0	0	0.0	0.0	0.1	0	0	0
Algeria	Sebbaa Chioukh	0.2	0.2	0.4	0	0	0	0.0	0.0	0.1	0	0	0
Algeria	Sebdou	0.3	0.2	0.4	1	1	2	0.0	0.0	0.1	0	0	0
Algeria	Sebgag	0.3	0.2	0.5	0	0	0	0.0	0.0	0.1	0	0	0
Algeria	Sebseb	0.3	0.2	0.5	0	0	0	0.0	0.0	0.1	0	0	0
Algeria	Sebt	0.3	0.2	0.4	0	0	0	0.0	0.0	0.1	0	0	0
Algeria	Sed Rahal	0.4	0.3	0.5	1	0	1	0.0	0.0	0.0	0	0	0
Algeria	Seddouk	0.2	0.2	0.4	0	0	1	0.0	0.0	0.1	0	0	0
Algeria	Sedjerara	0.3	0.2	0.5	0	0	1	0.0	0.0	0.1	0	0	0
Algeria	Sedrata	0.3	0.2	0.4	2	1	2	0.0	0.0	0.1	0	0	0
Algeria	Sedraya	0.3	0.2	0.4	0	0	0	0.0	0.0	0.1	0	0	0
Algeria	Sefiane	0.3	0.2	0.4	0	0	1	0.0	0.0	0.1	0	0	0
Algeria	Seggana	0.3	0.2	0.4	0	0	1	0.0	0.0	0.1	0	0	0
Algeria	Seghouane	0.2	0.2	0.4	0	0	0	0.0	0.0	0.0	0	0	0
Algeria	Sehailia	0.4	0.2	0.5	0	0	0	0.0	0.0	0.1	0	0	0

Table 3: Diarrhoea Mortality by Unit *(continued)*

Country	Unit	2000 mortality mean rate (per 10,000)	lower rate (per 10,000)	upper rate (per 10,000)	mean count	lower count	upper count	2017 mortality mean rate (per 10,000)	lower rate (per 10,000)	upper rate (per 10,000)	mean count	lower count	upper count
Algeria	Sehala Thaoura	0.2	0.1	0.4	0	0	0	0.0	0.0	0.1	0	0	0
Algeria	Selaoua Announa	0.3	0.2	0.4	0	0	0	0.0	0.0	0.1	0	0	0
Algeria	Selma Benziada	0.3	0.2	0.4	0	0	0	0.0	0.0	0.1	0	0	0
Algeria	Selmana	0.4	0.2	0.5	1	1	1	0.0	0.0	0.0	0	0	0
Algeria	Sendjas	0.3	0.2	0.4	1	1	2	0.0	0.0	0.1	0	0	0
Algeria	Seraidi	0.3	0.2	0.4	1	0	1	0.0	0.0	0.1	0	0	0
Algeria	Serdj-El-Ghoul	0.3	0.2	0.5	0	0	1	0.0	0.0	0.1	0	0	0
Algeria	Serghine	0.3	0.2	0.5	0	0	1	0.0	0.0	0.1	0	0	0
Algeria	Seriana	0.3	0.2	0.4	0	0	1	0.0	0.0	0.1	0	0	0
Algeria	Setif	0.3	0.2	0.4	8	5	12	0.0	0.0	0.1	1	1	2
Algeria	Settara	0.3	0.2	0.4	0	0	1	0.0	0.0	0.1	0	0	0
Algeria	Sfisef	0.3	0.2	0.5	1	1	2	0.0	0.0	0.1	0	0	0
Algeria	Sfissifa	0.3	0.2	0.4	0	0	0	0.0	0.0	0.1	0	0	0
Algeria	Si Abdelghani	0.3	0.2	0.5	0	0	1	0.0	0.0	0.1	0	0	0
Algeria	Si El Mahdjoub	0.3	0.2	0.4	0	0	0	0.0	0.0	0.0	0	0	0
Algeria	Si Mustapha	0.3	0.2	0.4	1	0	1	0.0	0.0	0.1	0	0	0
Algeria	Sidi Abdelaziz	0.3	0.2	0.4	0	0	0	0.0	0.0	0.1	0	0	0
Algeria	Sidi Abdeldjebar	0.3	0.2	0.5	0	0	0	0.0	0.0	0.1	0	0	0
Algeria	Sidi Abdelli	0.2	0.2	0.4	0	0	1	0.0	0.0	0.1	0	0	0
Algeria	Sidi Abdelmoumene	0.3	0.2	0.5	1	0	1	0.0	0.0	0.1	0	0	0
Algeria	Sidi Abderrahmane	0.2	0.2	0.4	0	0	0	0.0	0.0	0.1	0	0	0
Algeria	Sidi Abderrahmane	0.3	0.2	0.5	0	0	1	0.0	0.0	0.1	0	0	0
Algeria	Sidi Abed	0.3	0.2	0.4	0	0	0	0.0	0.0	0.0	0	0	0
Algeria	Sidi Ahmed	0.3	0.2	0.5	1	0	1	0.0	0.0	0.1	0	0	0
Algeria	Sidi Aissa	0.3	0.2	0.4	2	1	3	0.0	0.0	0.1	0	0	0
Algeria	Sidi Akkacha	0.3	0.2	0.4	1	1	1	0.0	0.0	0.0	0	0	0
Algeria	Sidi Ali	0.3	0.2	0.5	1	1	2	0.0	0.0	0.1	0	0	0
Algeria	Sidi Ali Benyoub	0.2	0.2	0.4	0	0	1	0.0	0.0	0.1	0	0	0
Algeria	Sidi Ali Boussidi	0.2	0.2	0.4	0	0	0	0.0	0.0	0.1	0	0	0
Algeria	Sidi Ali Mellal	0.3	0.2	0.4	0	0	0	0.0	0.0	0.1	0	0	0
Algeria	Sidi Amar	0.3	0.2	0.4	1	1	2	0.0	0.0	0.1	0	0	0
Algeria	Sidi Amar	0.3	0.2	0.4	0	0	1	0.0	0.0	0.1	0	0	0
Algeria	Sidi Amar	0.2	0.2	0.4	0	0	1	0.0	0.0	0.0	0	0	0
Algeria	Sidi Ameur	0.3	0.2	0.5	0	0	0	0.0	0.0	0.1	0	0	0
Algeria	Sidi Ameur	0.3	0.2	0.4	1	1	1	0.0	0.0	0.0	0	0	0
Algeria	Sidi Amrane	0.3	0.2	0.4	1	1	2	0.0	0.0	0.1	0	0	0
Algeria	Sidi Aoun	0.3	0.2	0.5	1	0	1	0.0	0.0	0.1	0	0	0
Algeria	Sidi Baizid	0.4	0.2	0.5	1	0	1	0.0	0.0	0.0	0	0	0
Algeria	Sidi Bakhti	0.3	0.2	0.4	0	0	0	0.0	0.0	0.1	0	0	0
Algeria	Sidi Bel Abbes	0.2	0.1	0.4	5	3	7	0.0	0.0	0.1	1	1	1
Algeria	Sidi Belattar	0.3	0.2	0.5	0	0	1	0.0	0.0	0.1	0	0	0
Algeria	Sidi Ben Adda	0.2	0.1	0.4	0	0	1	0.0	0.0	0.1	0	0	0
Algeria	Sidi Ben Yebka	0.3	0.2	0.4	0	0	0	0.0	0.0	0.1	0	0	0
Algeria	Sidi Boubekeur	0.3	0.2	0.4	1	0	1	0.0	0.0	0.1	0	0	0
Algeria	Sidi Boumediene	0.2	0.1	0.4	0	0	0	0.0	0.0	0.1	0	0	0
Algeria	Sidi Boussaid	0.3	0.2	0.5	0	0	0	0.0	0.0	0.1	0	0	0
Algeria	Sidi Boutouchent	0.3	0.2	0.5	0	0	0	0.0	0.0	0.0	0	0	0
Algeria	Sidi Bouzid	0.4	0.2	0.5	0	0	0	0.0	0.0	0.1	0	0	0
Algeria	Sidi Brahim	0.2	0.2	0.4	0	0	0	0.0	0.0	0.1	0	0	0
Algeria	Sidi Chahmi	0.3	0.2	0.4	3	2	5	0.0	0.0	0.1	1	1	1
Algeria	Sidi Chouab	0.3	0.2	0.4	0	0	0	0.0	0.0	0.1	0	0	0
Algeria	Sidi Dahou Zair	0.2	0.2	0.4	0	0	0	0.0	0.0	0.1	0	0	0
Algeria	Sidi Damed	0.3	0.2	0.4	0	0	0	0.0	0.0	0.0	0	0	0
Algeria	Sidi Daoud	0.2	0.1	0.4	0	0	1	0.0	0.0	0.1	0	0	0
Algeria	Sidi Djilali	0.3	0.2	0.5	0	0	0	0.0	0.0	0.1	0	0	0
Algeria	Sidi Embarek	0.3	0.2	0.5	0	0	1	0.0	0.0	0.1	0	0	0
Algeria	Sidi Errabia	0.3	0.2	0.4	0	0	0	0.0	0.0	0.1	0	0	0

Table 3: Diarrhoea Mortality by Unit *(continued)*

Country	Unit	2000 mortality mean rate (per 10,000)	lower rate (per 10,000)	upper rate (per 10,000)	mean count	lower count	upper count	2017 mortality mean rate (per 10,000)	lower rate (per 10,000)	upper rate (per 10,000)	mean count	lower count	upper count
Algeria	Sidi Fredj	0.3	0.2	0.4	0	0	0	0.0	0.0	0.1	0	0	0
Algeria	Sidi Ghiles	0.3	0.2	0.4	0	0	1	0.0	0.0	0.1	0	0	0
Algeria	Sidi Hadjeres	0.3	0.2	0.4	0	0	0	0.0	0.0	0.0	0	0	0
Algeria	Sidi Hamadouche	0.3	0.2	0.4	0	0	0	0.0	0.0	0.1	0	0	0
Algeria	Sidi Hosni	0.3	0.2	0.4	0	0	0	0.0	0.0	0.1	0	0	0
Algeria	Sidi Kada	0.3	0.2	0.5	1	1	1	0.0	0.0	0.1	0	0	0
Algeria	Sidi Khaled	0.3	0.2	0.4	1	1	2	0.0	0.0	0.1	0	0	0
Algeria	Sidi Khaled	0.2	0.2	0.4	0	0	0	0.0	0.0	0.1	0	0	0
Algeria	Sidi Khelifa	0.3	0.2	0.5	0	0	0	0.0	0.0	0.1	0	0	0
Algeria	Sidi Khelil	0.3	0.2	0.5	0	0	0	0.0	0.0	0.1	0	0	0
Algeria	Sidi Khettab	0.3	0.2	0.5	1	0	1	0.0	0.0	0.1	0	0	0
Algeria	Sidi Khouiled	0.3	0.2	0.4	0	0	0	0.0	0.0	0.1	0	0	0
Algeria	Sidi Ladjel	0.4	0.3	0.6	1	1	1	0.0	0.0	0.1	0	0	0
Algeria	Sidi Lahcene	0.2	0.2	0.4	1	0	1	0.0	0.0	0.1	0	0	0
Algeria	Sidi Lakhdar	0.3	0.2	0.4	1	1	2	0.0	0.0	0.1	0	0	0
Algeria	Sidi Lakhdar	0.3	0.2	0.5	1	0	1	0.0	0.0	0.1	0	0	0
Algeria	Sidi Lantri	0.3	0.2	0.4	0	0	0	0.0	0.0	0.1	0	0	0
Algeria	Sidi Lazreg	0.3	0.2	0.4	0	0	0	0.0	0.0	0.1	0	0	0
Algeria	Sidi M'Hamed	0.3	0.2	0.4	0	0	0	0.0	0.0	0.0	0	0	0
Algeria	Sidi M'Hamed Benali	0.3	0.2	0.5	1	1	1	0.0	0.0	0.1	0	0	0
Algeria	Sidi M'Hamed Benaouda	0.3	0.2	0.5	0	0	1	0.0	0.0	0.1	0	0	0
Algeria	Sidi Makhlouf	0.3	0.2	0.4	0	0	1	0.0	0.0	0.0	0	0	0
Algeria	Sidi Marouf	0.3	0.2	0.4	1	0	1	0.0	0.0	0.1	0	0	0
Algeria	Sidi Medjahed	0.3	0.2	0.4	0	0	0	0.0	0.0	0.1	0	0	0
Algeria	Sidi Merouane	0.3	0.2	0.5	1	0	1	0.0	0.0	0.1	0	0	0
Algeria	Sidi Mezghiche	0.3	0.2	0.4	1	0	1	0.0	0.0	0.1	0	0	0
Algeria	Sidi Moussa	0.3	0.2	0.4	2	1	2	0.0	0.0	0.1	0	0	1
Algeria	Sidi Naamane	0.2	0.1	0.3	0	0	0	0.0	0.0	0.0	0	0	0
Algeria	Sidi Naamane	0.3	0.2	0.4	1	0	1	0.0	0.0	0.1	0	0	0
Algeria	Sidi Okba	0.3	0.2	0.4	1	1	1	0.0	0.0	0.1	0	0	0
Algeria	Sidi Ouri-ache|Tadmaya	0.2	0.2	0.4	0	0	0	0.0	0.0	0.1	0	0	0
Algeria	Sidi Rached	0.2	0.2	0.4	0	0	1	0.0	0.0	0.0	0	0	0
Algeria	Sidi Saada	0.3	0.2	0.5	1	0	1	0.0	0.0	0.1	0	0	0
Algeria	Sidi Safi	0.2	0.2	0.4	0	0	0	0.0	0.0	0.1	0	0	0
Algeria	Sidi Said	0.3	0.2	0.4	0	0	0	0.0	0.0	0.1	0	0	0
Algeria	Sidi Semiane	0.3	0.2	0.4	0	0	0	0.0	0.0	0.1	0	0	0
Algeria	Sidi Slimane	0.3	0.2	0.4	0	0	0	0.0	0.0	0.1	0	0	0
Algeria	Sidi Slimane	0.3	0.2	0.5	0	0	0	0.0	0.0	0.1	0	0	0
Algeria	Sidi Slimane	0.3	0.2	0.4	0	0	0	0.0	0.0	0.1	0	0	0
Algeria	Sidi Tifour	0.3	0.2	0.5	0	0	0	0.0	0.0	0.1	0	0	0
Algeria	Sidi Yacoub	0.2	0.2	0.4	0	0	0	0.0	0.0	0.1	0	0	0
Algeria	Sidi Zahar	0.3	0.2	0.4	0	0	0	0.0	0.0	0.1	0	0	0
Algeria	Sidi Ziane	0.3	0.2	0.4	0	0	0	0.0	0.0	0.0	0	0	0
Algeria	Sig	0.3	0.2	0.5	2	1	4	0.0	0.0	0.1	0	0	0
Algeria	Sigous	0.3	0.2	0.4	1	0	1	0.0	0.0	0.1	0	0	0
Algeria	Sirat	0.3	0.2	0.5	1	0	1	0.0	0.0	0.1	0	0	0
Algeria	Skikda	0.3	0.2	0.4	2	1	3	0.0	0.0	0.1	0	0	1
Algeria	Slim	0.3	0.2	0.5	0	0	0	0.0	0.0	0.0	0	0	0
Algeria	Smaoun	0.2	0.2	0.4	0	0	0	0.0	0.0	0.1	0	0	0
Algeria	Sobha	0.3	0.2	0.4	1	1	1	0.0	0.0	0.1	0	0	0
Algeria	Souaflia	0.3	0.2	0.5	1	0	1	0.0	0.0	0.1	0	0	0
Algeria	Souagui	0.3	0.2	0.4	1	0	1	0.0	0.0	0.1	0	0	0
Algeria	Souahlia	0.3	0.2	0.4	1	0	1	0.0	0.0	0.1	0	0	0
Algeria	Souamaa	0.2	0.1	0.3	0	0	0	0.0	0.0	0.1	0	0	0
Algeria	Souani	0.3	0.2	0.4	0	0	0	0.0	0.0	0.1	0	0	0
Algeria	Souarekh	0.3	0.2	0.4	0	0	0	0.0	0.0	0.1	0	0	0
Algeria	Sougueur	0.3	0.2	0.5	3	2	4	0.0	0.0	0.1	0	0	1

Table 3: Diarrhoea Mortality by Unit *(continued)*

Country	Unit	2000 mortality mean rate (per 10,000)	2000 mortality lower rate (per 10,000)	2000 mortality upper rate (per 10,000)	2000 mortality mean count	2000 mortality lower count	2000 mortality upper count	2017 mortality mean rate (per 10,000)	2017 mortality lower rate (per 10,000)	2017 mortality upper rate (per 10,000)	2017 mortality mean count	2017 mortality lower count	2017 mortality upper count
Algeria	Souhan	0.3	0.2	0.4	0	0	0	0.0	0.0	0.0	0	0	0
Algeria	Souidania	0.3	0.2	0.4	0	0	1	0.0	0.0	0.1	0	0	0
Algeria	Souk Ahras	0.3	0.2	0.4	3	2	5	0.0	0.0	0.1	1	0	1
Algeria	Souk El Had	0.3	0.2	0.4	0	0	0	0.0	0.0	0.1	0	0	0
Algeria	Souk El Had	0.3	0.2	0.4	0	0	0	0.0	0.0	0.1	0	0	0
Algeria	Souk El Khemis	0.2	0.1	0.3	0	0	0	0.0	0.0	0.1	0	0	0
Algeria	Souk El Tenine	0.2	0.1	0.4	0	0	0	0.0	0.0	0.1	0	0	0
Algeria	Souk El Tenine	0.2	0.1	0.3	0	0	0	0.0	0.0	0.0	0	0	0
Algeria	Souk Naamane	0.3	0.2	0.4	1	1	1	0.0	0.0	0.1	0	0	0
Algeria	Souk Oufella	0.2	0.2	0.4	1	0	1	0.0	0.0	0.1	0	0	0
Algeria	Souk Tleta	0.3	0.2	0.4	0	0	0	0.0	0.0	0.1	0	0	0
Algeria	Soumaa	0.3	0.2	0.4	1	1	2	0.0	0.0	0.1	0	0	0
Algeria	Soumaa	0.3	0.2	0.4	0	0	0	0.0	0.0	0.1	0	0	0
Algeria	Sour	0.3	0.2	0.5	1	1	1	0.0	0.0	0.1	0	0	0
Algeria	Sour El Ghouzlane	0.2	0.2	0.4	1	1	2	0.0	0.0	0.1	0	0	0
Algeria	Stah Guentis	0.2	0.1	0.3	0	0	0	0.0	0.0	0.0	0	0	0
Algeria	Staoueli	0.3	0.2	0.4	1	1	1	0.0	0.0	0.1	0	0	0
Algeria	Stidia	0.3	0.2	0.4	1	0	1	0.0	0.0	0.1	0	0	0
Algeria	Still	0.3	0.2	0.5	0	0	0	0.0	0.0	0.1	0	0	0
Algeria	Stitten	0.3	0.2	0.6	0	0	0	0.0	0.0	0.1	0	0	0
Algeria	T Kout	0.3	0.2	0.4	0	0	1	0.0	0.0	0.1	0	0	0
Algeria	Tabelbala	0.2	0.2	0.3	0	0	0	0.0	0.0	0.0	0	0	0
Algeria	Tabia	0.2	0.2	0.4	0	0	0	0.0	0.0	0.1	0	0	0
Algeria	Tablat	0.3	0.2	0.4	1	0	1	0.0	0.0	0.1	0	0	0
Algeria	Tacheta Zegagha	0.3	0.2	0.4	1	0	1	0.0	0.0	0.0	0	0	0
Algeria	Tachouda	0.3	0.2	0.5	0	0	1	0.0	0.0	0.1	0	0	0
Algeria	Tadjemout	0.3	0.2	0.4	1	1	1	0.0	0.0	0.0	0	0	0
Algeria	Tadjena	0.3	0.2	0.4	1	0	1	0.0	0.0	0.1	0	0	0
Algeria	Tadjenanet	0.3	0.2	0.4	1	1	2	0.0	0.0	0.1	0	0	0
Algeria	Tadjrouna	0.3	0.2	0.4	0	0	0	0.0	0.0	0.0	0	0	0
Algeria	Tadmait	0.2	0.1	0.3	0	0	1	0.0	0.0	0.1	0	0	0
Algeria	Tadmit	0.4	0.2	0.5	0	0	1	0.0	0.0	0.1	0	0	0
Algeria	Tafissour	0.3	0.2	0.4	0	0	0	0.0	0.0	0.1	0	0	0
Algeria	Tafraoui	0.3	0.2	0.4	0	0	1	0.0	0.0	0.1	0	0	0
Algeria	Tafraout	0.3	0.2	0.4	0	0	0	0.0	0.0	0.0	0	0	0
Algeria	Tafreg	0.3	0.2	0.5	0	0	0	0.0	0.0	0.1	0	0	0
Algeria	Tagdemt	0.3	0.2	0.5	0	0	0	0.0	0.0	0.1	0	0	0
Algeria	Taghit	0.3	0.2	0.4	0	0	0	0.0	0.0	0.1	0	0	0
Algeria	Taghlimet	0.3	0.2	0.4	0	0	0	0.0	0.0	0.1	0	0	0
Algeria	Taghzout	0.3	0.2	0.5	1	1	1	0.0	0.0	0.1	0	0	0
Algeria	Taghzout	0.2	0.2	0.3	0	0	0	0.0	0.0	0.1	0	0	0
Algeria	Taglait	0.3	0.2	0.5	0	0	0	0.0	0.0	0.1	0	0	0
Algeria	Taguedit	0.2	0.2	0.4	0	0	0	0.0	0.0	0.0	0	0	0
Algeria	Taher	0.3	0.2	0.4	2	1	3	0.0	0.0	0.1	0	0	1
Algeria	Taibet	0.3	0.2	0.4	1	0	1	0.0	0.0	0.1	0	0	0
Algeria	Takhemaret	0.3	0.2	0.5	1	1	2	0.0	0.0	0.1	0	0	0
Algeria	Tala Hamza	0.2	0.1	0.4	0	0	1	0.0	0.0	0.1	0	0	0
Algeria	Tala-Ifacene	0.3	0.2	0.4	1	0	1	0.0	0.0	0.1	0	0	0
Algeria	Taleb Larbi	0.3	0.2	0.4	0	0	0	0.0	0.0	0.1	0	0	0
Algeria	Talkhamt	0.3	0.2	0.4	1	0	1	0.0	0.0	0.1	0	0	0
Algeria	Talmine	0.3	0.2	0.4	0	0	1	0.0	0.0	0.1	0	0	0
Algeria	Tamalous	0.3	0.2	0.4	1	1	2	0.0	0.0	0.1	0	0	0
Algeria	Tamantit	0.3	0.2	0.5	0	0	1	0.0	0.0	0.1	0	0	0
Algeria	Tamekten	0.3	0.2	0.4	1	0	1	0.0	0.0	0.1	0	0	0
Algeria	Tamelaht	0.3	0.2	0.4	0	0	0	0.0	0.0	0.0	0	0	0
Algeria	Tamenghasset	0.3	0.2	0.4	4	2	5	0.0	0.0	0.0	1	0	1
Algeria	Tamest	0.3	0.2	0.5	0	0	1	0.0	0.0	0.1	0	0	0
Algeria	Tamezguida	0.3	0.2	0.4	0	0	0	0.0	0.0	0.0	0	0	0
Algeria	Tamlouka	0.3	0.2	0.4	1	0	1	0.0	0.0	0.1	0	0	0

Table 3: Diarrhoea Mortality by Unit *(continued)*

Country	Unit	2000 mortality mean rate (per 10,000)	lower rate (per 10,000)	upper rate (per 10,000)	mean count	lower count	upper count	2017 mortality mean rate (per 10,000)	lower rate (per 10,000)	upper rate (per 10,000)	mean count	lower count	upper count
Algeria	Tamokra	0.3	0.2	0.4	0	0	0	0.0	0.0	0.1	0	0	0
Algeria	Tamridjet	0.3	0.1	0.4	0	0	0	0.0	0.0	0.1	0	0	0
Algeria	Tamsa	0.3	0.2	0.4	0	0	0	0.0	0.0	0.0	0	0	0
Algeria	Tamtert	0.2	0.1	0.4	0	0	0	0.0	0.0	0.1	0	0	0
Algeria	Tamza	0.3	0.2	0.4	0	0	1	0.0	0.0	0.1	0	0	0
Algeria	Tamzoura	0.2	0.1	0.3	0	0	0	0.0	0.0	0.1	0	0	0
Algeria	Taouala	0.3	0.2	0.5	0	0	0	0.0	0.0	0.1	0	0	0
Algeria	Taoudmout	0.3	0.2	0.4	0	0	0	0.0	0.0	0.1	0	0	0
Algeria	Taougrit	0.3	0.2	0.4	1	1	1	0.0	0.0	0.1	0	0	0
Algeria	Taoura	0.3	0.2	0.4	1	0	1	0.0	0.0	0.1	0	0	0
Algeria	Taourga	0.2	0.1	0.3	0	0	0	0.0	0.0	0.1	0	0	0
Algeria	Taourit Ighil	0.3	0.2	0.4	0	0	0	0.0	0.0	0.1	0	0	0
Algeria	Taouzianat	0.3	0.2	0.4	0	0	0	0.0	0.0	0.1	0	0	0
Algeria	Tarik Ibn-Ziad	0.3	0.2	0.5	0	0	1	0.0	0.0	0.1	0	0	0
Algeria	Tarmount	0.3	0.2	0.4	0	0	1	0.0	0.0	0.0	0	0	0
Algeria	Taskriout	0.2	0.1	0.4	0	0	0	0.0	0.0	0.1	0	0	0
Algeria	Tassadane Haddada	0.3	0.2	0.5	1	0	1	0.0	0.0	0.1	0	0	0
Algeria	Tassala	0.2	0.2	0.4	0	0	0	0.0	0.0	0.1	0	0	0
Algeria	Taxlent	0.3	0.2	0.5	0	0	1	0.0	0.0	0.1	0	0	0
Algeria	Taya	0.3	0.2	0.4	0	0	1	0.0	0.0	0.1	0	0	0
Algeria	Tazgait	0.3	0.2	0.5	0	0	1	0.0	0.0	0.1	0	0	0
Algeria	Tazmalt	0.2	0.1	0.3	0	0	1	0.0	0.0	0.1	0	0	0
Algeria	Tazoult	0.3	0.2	0.4	1	0	1	0.0	0.0	0.1	0	0	0
Algeria	Tazrouk	0.2	0.1	0.2	0	0	0	0.0	0.0	0.0	0	0	0
Algeria	Tebesbest	0.3	0.2	0.4	1	1	1	0.0	0.0	0.0	0	0	0
Algeria	Tebessa	0.2	0.1	0.3	4	3	6	0.0	0.0	0.1	1	1	1
Algeria	Telaa	0.3	0.2	0.4	0	0	0	0.0	0.0	0.1	0	0	0
Algeria	Telagh	0.3	0.2	0.4	1	0	1	0.0	0.0	0.1	0	0	0
Algeria	Telassa	0.2	0.2	0.4	0	0	0	0.0	0.0	0.0	0	0	0
Algeria	Teleghma	0.3	0.2	0.5	1	1	2	0.0	0.0	0.1	0	0	0
Algeria	Temacine	0.3	0.2	0.3	1	1	1	0.0	0.0	0.1	0	0	1
Algeria	Tenedla	0.3	0.2	0.5	0	0	1	0.0	0.0	0.1	0	0	0
Algeria	Tenes	0.3	0.2	0.4	0	0	1	0.0	0.0	0.0	0	0	0
Algeria	Teniet El Abed	0.3	0.2	0.4	0	0	1	0.0	0.0	0.1	0	0	0
Algeria	Teniet En Nasr	0.3	0.2	0.4	0	0	0	0.0	0.0	0.1	0	0	0
Algeria	Tenira	0.2	0.2	0.4	0	0	0	0.0	0.0	0.1	0	0	0
Algeria	Terga	0.2	0.2	0.4	0	0	0	0.0	0.0	0.1	0	0	0
Algeria	Terny Beni Hediel	0.3	0.2	0.4	1	1	1	0.0	0.0	0.1	0	0	0
Algeria	Terraguelt	0.3	0.2	0.4	0	0	0	0.0	0.0	0.1	0	0	0
Algeria	Terrai Bainnane	0.3	0.2	0.5	1	0	1	0.0	0.0	0.1	0	0	0
Algeria	Tesmart	0.3	0.2	0.5	0	0	0	0.0	0.0	0.1	0	0	0
Algeria	Tessala Lamtai	0.3	0.2	0.5	1	0	1	0.0	0.0	0.1	0	0	0
Algeria	Tessala-El-Merdja	0.3	0.2	0.4	1	0	1	0.0	0.0	0.1	0	0	0
Algeria	Texenna	0.3	0.2	0.5	1	0	1	0.0	0.0	0.1	0	0	0
Algeria	Thelidjene	0.2	0.1	0.3	0	0	0	0.0	0.0	0.0	0	0	0
Algeria	Thenia	0.3	0.2	0.4	1	0	1	0.0	0.0	0.1	0	0	0
Algeria	Theniet El Had	0.3	0.2	0.5	1	1	2	0.0	0.0	0.0	0	0	0
Algeria	Thleth Douair	0.2	0.2	0.4	0	0	0	0.0	0.0	0.0	0	0	0
Algeria	Tianet	0.3	0.2	0.4	0	0	0	0.0	0.0	0.1	0	0	0
Algeria	Tiaret	0.3	0.2	0.5	7	4	10	0.0	0.0	0.1	1	1	2
Algeria	Tiberguent	0.3	0.2	0.5	0	0	1	0.0	0.0	0.1	0	0	0
Algeria	Tiberkanine	0.3	0.2	0.4	1	0	1	0.0	0.0	0.0	0	0	0
Algeria	Tichy	0.3	0.2	0.4	0	0	1	0.0	0.0	0.1	0	0	0
Algeria	Tidda	0.3	0.2	0.4	0	0	0	0.0	0.0	0.1	0	0	0
Algeria	Tidjelabine	0.3	0.2	0.4	1	0	1	0.0	0.0	0.1	0	0	0
Algeria	Tiffech	0.3	0.2	0.4	0	0	0	0.0	0.0	0.1	0	0	0
Algeria	Tifra	0.3	0.2	0.4	0	0	0	0.0	0.0	0.1	0	0	0
Algeria	Tighanimine	0.3	0.2	0.4	0	0	0	0.0	0.0	0.1	0	0	0

Table 3: Diarrhoea Mortality by Unit *(continued)*

Country	Unit	2000 mortality mean rate (per 10,000)	lower rate (per 10,000)	upper rate (per 10,000)	mean count	lower count	upper count	2017 mortality mean rate (per 10,000)	lower rate (per 10,000)	upper rate (per 10,000)	mean count	lower count	upper count
Algeria	Tigharghar	0.2	0.2	0.4	0	0	0	0.0	0.0	0.1	0	0	0
Algeria	Tighenif	0.3	0.2	0.5	2	2	4	0.0	0.0	0.1	0	0	0
Algeria	Tigzirt	0.2	0.1	0.3	0	0	0	0.0	0.0	0.1	0	0	0
Algeria	Tilatou	0.3	0.2	0.4	0	0	0	0.0	0.0	0.1	0	0	0
Algeria	Tilmouni	0.2	0.2	0.4	0	0	1	0.0	0.0	0.1	0	0	0
Algeria	Timezrit	0.2	0.1	0.3	0	0	0	0.0	0.0	0.1	0	0	0
Algeria	Timezrit	0.2	0.2	0.4	1	0	1	0.0	0.0	0.1	0	0	0
Algeria	Timgad	0.3	0.2	0.4	0	0	1	0.0	0.0	0.1	0	0	0
Algeria	Timiaouine	0.3	0.2	0.3	0	0	0	0.0	0.0	0.0	0	0	0
Algeria	Timizart	0.2	0.1	0.3	0	0	1	0.0	0.0	0.1	0	0	0
Algeria	Timmimoun	0.3	0.2	0.4	1	1	2	0.0	0.0	0.1	0	0	0
Algeria	Timoudi	0.2	0.2	0.4	0	0	0	0.0	0.0	0.0	0	0	0
Algeria	Tin Zaouatine	0.2	0.2	0.3	0	0	0	0.0	0.0	0.0	0	0	0
Algeria	Tindouf	0.3	0.2	0.4	1	1	2	0.0	0.0	0.1	0	0	1
Algeria	Tinedbar	0.2	0.1	0.3	0	0	0	0.0	0.0	0.1	0	0	0
Algeria	Tinerkouk	0.3	0.2	0.4	1	0	1	0.0	0.0	0.1	0	0	0
Algeria	Tiout	0.3	0.2	0.4	0	0	0	0.0	0.0	0.1	0	0	0
Algeria	Tipaza	0.3	0.2	0.4	1	0	1	0.0	0.0	0.1	0	0	0
Algeria	Tircine	0.3	0.2	0.5	0	0	1	0.0	0.0	0.1	0	0	0
Algeria	Tirmitine	0.2	0.1	0.3	0	0	0	0.0	0.0	0.0	0	0	0
Algeria	Tissemsilt	0.3	0.2	0.4	2	1	3	0.0	0.0	0.1	0	0	1
Algeria	Tit	0.2	0.1	0.3	0	0	0	0.0	0.0	0.1	0	0	0
Algeria	Tixter	0.3	0.2	0.5	0	0	1	0.0	0.0	0.1	0	0	0
Algeria	Tizi	0.3	0.2	0.5	1	0	1	0.0	0.0	0.1	0	0	0
Algeria	Tizi Mahdi	0.3	0.2	0.4	0	0	1	0.0	0.0	0.0	0	0	0
Algeria	Tizi N'Bechar	0.3	0.2	0.4	1	0	1	0.0	0.0	0.1	0	0	0
Algeria	Tizi N'Tleta	0.2	0.1	0.3	0	0	0	0.0	0.0	0.0	0	0	0
Algeria	Tizi Ouzou	0.2	0.1	0.3	2	1	3	0.0	0.0	0.1	0	0	1
Algeria	Tizi-Ghenif	0.2	0.1	0.3	0	0	1	0.0	0.0	0.1	0	0	0
Algeria	Tizi-N'Berber	0.3	0.2	0.4	0	0	1	0.0	0.0	0.1	0	0	0
Algeria	Tizi-Rached	0.2	0.1	0.3	0	0	0	0.0	0.0	0.1	0	0	0
Algeria	Tlemcen	0.2	0.1	0.4	3	2	5	0.0	0.0	0.1	1	0	1
Algeria	Tolga	0.3	0.2	0.4	1	1	2	0.0	0.0	0.1	0	0	0
Algeria	Touahria	0.3	0.2	0.5	0	0	0	0.0	0.0	0.1	0	0	0
Algeria	Toudja	0.3	0.2	0.4	0	0	0	0.0	0.0	0.1	0	0	0
Algeria	Touggourt	0.2	0.2	0.3	2	1	2	0.0	0.0	0.0	0	0	0
Algeria	Tousmouline	0.3	0.2	0.5	0	0	0	0.0	0.0	0.1	0	0	0
Algeria	Tousnina	0.3	0.2	0.5	0	0	1	0.0	0.0	0.1	0	0	0
Algeria	Treat	0.3	0.2	0.4	0	0	0	0.0	0.0	0.1	0	0	0
Algeria	Trifaoui	0.3	0.2	0.5	0	0	1	0.0	0.0	0.1	0	0	0
Algeria	Tsabit	0.3	0.2	0.4	1	0	1	0.0	0.0	0.0	0	0	0
Algeria	Yabous	0.3	0.2	0.4	0	0	0	0.0	0.0	0.1	0	0	0
Algeria	Yahia Beniguecha	0.3	0.2	0.5	0	0	1	0.0	0.0	0.1	0	0	0
Algeria	Yakourene	0.2	0.1	0.4	0	0	0	0.0	0.0	0.1	0	0	0
Algeria	Yatafene	0.2	0.1	0.3	0	0	0	0.0	0.0	0.0	0	0	0
Algeria	Yellel	0.3	0.2	0.5	1	1	2	0.0	0.0	0.1	0	0	0
Algeria	Youb	0.3	0.2	0.4	1	0	1	0.0	0.0	0.1	0	0	0
Algeria	Zaafrane	0.4	0.3	0.6	1	1	1	0.0	0.0	0.1	0	0	0
Algeria	Zaarouria	0.3	0.2	0.4	1	0	1	0.0	0.0	0.1	0	0	0
Algeria	Zaccar	0.4	0.3	0.6	0	0	0	0.0	0.0	0.1	0	0	0
Algeria	Zahana	0.3	0.2	0.5	1	0	1	0.0	0.0	0.1	0	0	0
Algeria	Zanet El Beida	0.3	0.2	0.4	0	0	1	0.0	0.0	0.1	0	0	0
Algeria	Zaouia El Abidia	0.2	0.2	0.3	0	0	1	0.0	0.0	0.1	0	0	0
Algeria	Zaouiet Kounta	0.3	0.2	0.5	1	0	1	0.0	0.0	0.1	0	0	0
Algeria	Zarzour	0.3	0.2	0.4	0	0	0	0.0	0.0	0.0	0	0	0
Algeria	Zbarbar|El Isseri	0.2	0.1	0.3	0	0	0	0.0	0.0	0.1	0	0	0
Algeria	Zeboudja	0.3	0.2	0.4	1	1	1	0.0	0.0	0.0	0	0	0
Algeria	Zeddine	0.3	0.2	0.4	0	0	1	0.0	0.0	0.1	0	0	0
Algeria	Zeghaia	0.3	0.2	0.5	1	0	1	0.0	0.0	0.1	0	0	0
Algeria	Zekri	0.3	0.2	0.4	0	0	0	0.0	0.0	0.1	0	0	0

Table 3: Diarrhoea Mortality by Unit *(continued)*

Country	Unit	2000 mortality mean rate (per 10,000)	lower rate (per 10,000)	upper rate (per 10,000)	mean count	lower count	upper count	2017 mortality mean rate (per 10,000)	lower rate (per 10,000)	upper rate (per 10,000)	mean count	lower count	upper count
Algeria	Zelfana	0.3	0.2	0.5	0	0	1	0.0	0.0	0.1	0	0	0
Algeria	Zelmata	0.3	0.2	0.5	0	0	0	0.0	0.0	0.1	0	0	0
Algeria	Zemmoura	0.3	0.2	0.4	1	1	1	0.0	0.0	0.1	0	0	0
Algeria	Zemmouri	0.3	0.2	0.4	0	0	1	0.0	0.0	0.1	0	0	0
Algeria	Zenata	0.2	0.2	0.4	0	0	0	0.0	0.0	0.1	0	0	0
Algeria	Zeralda	0.3	0.2	0.4	1	1	2	0.0	0.0	0.1	0	0	0
Algeria	Zerdeza	0.3	0.2	0.4	0	0	0	0.0	0.0	0.1	0	0	0
Algeria	Zeribet El Oued	0.2	0.1	0.3	1	0	1	0.0	0.0	0.1	0	0	0
Algeria	Zerizer	0.3	0.2	0.4	0	0	0	0.0	0.0	0.1	0	0	0
Algeria	Zerouala	0.3	0.2	0.4	0	0	0	0.0	0.0	0.1	0	0	0
Algeria	Ziama Mansouria	0.3	0.2	0.4	0	0	0	0.0	0.0	0.1	0	0	0
Algeria	Zighoud Youcef	0.3	0.2	0.4	1	1	2	0.0	0.0	0.1	0	0	0
Algeria	Zitouna	0.3	0.2	0.4	0	0	0	0.0	0.0	0.1	0	0	0
Algeria	Zitouna	0.3	0.2	0.5	0	0	0	0.0	0.0	0.1	0	0	0
Algeria	Zmalet El Emir Abdelkader	0.3	0.2	0.5	1	1	1	0.0	0.0	0.1	0	0	0
Algeria	Zorg	0.3	0.2	0.4	0	0	0	0.0	0.0	0.1	0	0	0
Algeria	Zouabi	0.3	0.2	0.4	0	0	0	0.0	0.0	0.1	0	0	0
Algeria	Zoubiria	0.3	0.2	0.4	1	0	1	0.0	0.0	0.1	0	0	0
Egypt	'Abdin	1.4	0.9	2.0	4	3	6	0.5	0.3	0.8	2	1	3
Egypt	'Ain Schams	1.5	1.0	2.0	43	29	58	0.5	0.3	0.8	17	10	26
Egypt	'Ataqah	1.5	1.0	2.1	11	7	15	0.5	0.3	0.8	6	3	8
Egypt	10 Ramadan 1	1.6	1.1	2.3	9	6	13	0.5	0.3	0.8	4	2	7
Egypt	10 Ramadan 2	1.7	1.1	2.4	6	4	8	0.5	0.3	0.8	3	2	4
Egypt	15 Mayu	1.4	1.0	2.1	30	21	43	0.5	0.3	0.8	11	7	17
Egypt	Abnub	3.5	2.6	4.7	134	100	180	0.7	0.4	1.1	39	26	61
Egypt	Abu al-Matamir	1.7	1.1	2.4	77	51	108	0.5	0.3	0.7	31	20	45
Egypt	Abu Hammad	1.9	1.2	2.5	71	47	97	0.5	0.3	0.8	30	20	48
Egypt	Abu Hummus	2.0	1.4	3.0	85	58	128	0.5	0.3	0.8	33	21	49
Egypt	Abu Kabir	1.9	1.3	2.7	66	43	91	0.5	0.3	0.8	26	17	41
Egypt	Abu Qurqas	2.9	2.0	4.1	150	104	215	0.7	0.4	1.0	53	32	80
Egypt	Abu Radis	1.3	0.9	1.9	1	1	2	0.4	0.3	0.6	1	0	1
Egypt	Abu Simbel	NA	NA	NA	NA	NA	NA	NA	NA	NA	NA	NA	NA
Egypt	Abu Tij	3.6	2.5	4.9	111	78	151	0.7	0.4	1.0	32	20	47
Egypt	Abu Tisht	2.4	1.7	3.3	128	88	172	0.6	0.4	0.8	27	18	40
Egypt	Abu Zenima	1.3	0.8	2.0	0	0	0	0.4	0.2	0.6	0	0	0
Egypt	Ad-Dab'ah	2.6	1.7	4.0	9	6	13	0.6	0.4	0.9	4	2	5
Egypt	Ad-Darb al-Ahmar	1.4	1.0	2.1	6	4	9	0.5	0.3	0.7	2	1	4
Egypt	Ad-Dawahy	1.8	1.1	2.6	8	5	12	0.5	0.3	0.7	3	2	4
Egypt	Ad-Dilinat	2.0	1.3	2.7	69	46	94	0.6	0.4	0.9	30	19	46
Egypt	Ad-Dukhaylah	1.6	1.1	2.3	46	31	67	0.4	0.3	0.6	17	11	26
Egypt	Ad-Duqi	1.4	1.0	2.0	21	14	30	0.5	0.3	0.8	13	8	21
Egypt	Aja	1.7	1.1	2.3	69	47	96	0.5	0.3	0.8	31	20	46
Egypt	Akhmim	2.9	1.8	4.3	101	65	152	0.7	0.4	1.0	36	23	53
Egypt	Al-'Ajuzah	1.4	1.0	2.0	18	12	26	0.5	0.3	0.8	13	8	20
Egypt	Al-'Amriyah	1.6	1.1	2.3	132	90	195	0.4	0.3	0.6	47	30	70
Egypt	Al-'Arab	1.8	1.1	2.6	4	3	6	0.5	0.3	0.7	2	1	2
Egypt	Al-'Arish 1	2.0	1.3	3.0	13	8	19	0.6	0.4	1.0	6	4	10
Egypt	Al-'Arish 2	2.1	1.3	3.1	4	3	6	0.6	0.4	1.0	2	1	3
Egypt	Al-'Arish 3	2.1	1.4	3.2	4	3	6	0.6	0.4	1.0	2	1	3
Egypt	Al-'Arish 4	2.1	1.4	2.8	9	6	12	0.7	0.4	1.0	4	2	6
Egypt	Al-'Atarin	1.5	1.0	2.3	6	4	9	0.4	0.3	0.6	2	1	4
Egypt	Al-'Ayyat	1.6	1.0	2.3	57	36	84	0.5	0.3	0.7	31	20	46
Egypt	Al-'Idwah	2.8	1.9	4.1	73	50	107	0.7	0.4	1.1	30	18	45
Egypt	Al-'Ubur	1.7	1.2	2.3	8	5	10	0.5	0.3	0.8	3	2	5
Egypt	Al-'Umraniyah	1.5	1.0	2.0	59	41	82	0.5	0.3	0.8	37	24	58
Egypt	Al-'Usayrat	2.9	2.0	4.0	49	33	66	0.7	0.5	1.0	18	11	26
Egypt	Al-Ahram	1.5	1.0	2.0	38	26	52	0.5	0.3	0.8	24	15	37
Egypt	Al-Arb'in	1.5	0.9	2.3	33	20	51	0.5	0.3	0.8	18	11	27

Table 3: Diarrhoea Mortality by Unit *(continued)*

Country	Unit	2000 mortality mean rate (per 10,000)	lower rate (per 10,000)	upper rate (per 10,000)	mean count	lower count	upper count	2017 mortality mean rate (per 10,000)	lower rate (per 10,000)	upper rate (per 10,000)	mean count	lower count	upper count
Egypt	Al-Azbakiyah	1.4	0.9	2.0	4	3	6	0.5	0.3	0.8	2	1	3
Egypt	Al-Badari	3.3	2.3	4.7	73	51	105	0.7	0.4	1.0	22	14	33
Egypt	Al-Badrashayn	1.5	1.0	2.1	60	41	84	0.5	0.3	0.8	36	22	56
Egypt	Al-Bajur	1.5	1.0	2.2	57	38	82	0.5	0.3	0.8	28	18	43
Egypt	Al-Baliyana	3.1	2.1	4.1	136	91	184	0.7	0.4	1.1	46	30	70
Egypt	Al-Basatin	1.4	1.0	2.0	89	62	127	0.5	0.3	0.7	36	22	56
Egypt	Al-Burulus	1.6	1.1	2.4	31	21	45	0.4	0.3	0.7	12	8	18
Egypt	Al-Fashn	2.7	1.9	3.9	114	79	167	0.7	0.4	1.1	47	29	69
Egypt	Al-Fath	3.6	2.5	4.9	125	87	172	0.7	0.4	1.1	36	23	58
Egypt	Al-Fayyum	2.4	1.7	3.3	200	143	280	0.7	0.4	1.0	92	56	139
Egypt	Al-Fayyum City	2.7	1.9	3.7	1	0	1	0.7	0.5	1.1	0	0	0
Egypt	Al-Ganoub	1.8	1.2	2.6	17	11	24	0.5	0.3	0.7	6	3	8
Egypt	Al-Ganoub 2	1.9	1.2	2.8	4	3	6	0.5	0.3	0.8	2	1	3
Egypt	Al-Ghanayim	3.3	2.2	4.6	30	21	42	0.7	0.4	1.0	10	6	15
Egypt	Al-Ghurdaqah	1.8	1.1	2.7	9	6	14	0.5	0.3	0.7	3	2	5
Egypt	Al-Ghurdaqah 2	1.7	1.2	2.4	2	1	3	0.5	0.3	0.7	1	1	1
Egypt	Al-Hammam	2.5	1.7	3.6	16	10	22	0.6	0.4	0.9	7	4	10
Egypt	Al-Hamul	1.6	1.1	2.3	45	31	65	0.5	0.3	0.7	18	11	26
Egypt	Al-Hasanah	2.3	1.6	3.3	2	1	3	0.7	0.5	1.0	1	1	1
Egypt	Al-Hawamidiyah	1.4	1.0	2.0	21	15	30	0.5	0.3	0.8	13	8	20
Egypt	Al-Husayniyah	1.9	1.3	2.8	102	67	149	0.5	0.3	0.8	40	24	62
Egypt	Al-Ibrahimiyah	1.9	1.3	2.8	23	15	32	0.5	0.3	0.8	9	6	14
Egypt	Al-Jamaliyah	1.8	1.2	2.6	18	12	26	0.5	0.3	0.7	7	4	11
Egypt	Al-Jamaliyah	1.4	0.9	2.1	11	7	16	0.5	0.3	0.8	4	3	7
Egypt	Al-Janayin	1.5	1.0	2.2	19	12	28	0.5	0.3	0.8	9	6	14
Egypt	Al-Jumruk	1.5	1.0	2.3	6	3	8	0.4	0.3	0.6	2	1	3
Egypt	Al-Kawtar	2.8	1.8	4.3	1	1	2	0.7	0.4	1.0	0	0	1
Egypt	Al-Khalifa	1.4	1.0	2.1	63	43	91	0.5	0.3	0.7	24	14	36
Egypt	Al-Khankah	1.6	1.1	2.2	120	83	161	0.5	0.3	0.8	53	34	83
Egypt	Al-Khusus	1.5	1.0	2.1	38	26	51	0.5	0.3	0.8	16	10	24
Egypt	Al-Laban	1.5	1.0	2.3	3	2	5	0.4	0.3	0.6	1	1	2
Egypt	Al-Ma'adi	1.4	1.0	2.0	32	22	46	0.5	0.3	0.7	13	8	20
Egypt	Al-Mahallah al-Kubra	1.6	1.1	2.2	142	99	199	0.5	0.3	0.7	63	41	96
Egypt	Al-Mahallah al-Kubra 1	1.5	1.1	2.1	16	11	22	0.5	0.3	0.7	6	4	10
Egypt	Al-Mahallah al-Kubra 2	1.5	1.0	2.1	24	16	33	0.5	0.3	0.7	10	7	15
Egypt	Al-Mahmudiyah	1.7	1.1	2.4	37	26	55	0.5	0.3	0.7	15	9	23
Egypt	Al-Manakh	1.8	1.1	2.6	13	8	19	0.5	0.3	0.7	4	3	7
Egypt	Al-Manasrah	1.8	1.1	2.6	13	8	19	0.5	0.3	0.7	5	3	7
Egypt	Al-Manshah	2.9	1.9	4.2	99	65	142	0.7	0.4	1.0	36	23	54
Egypt	Al-Manshiyah	1.5	1.0	2.3	1	1	2	0.4	0.3	0.6	0	0	1
Egypt	Al-Mansurah	1.8	1.2	2.5	99	66	136	0.5	0.3	0.7	40	25	60
Egypt	Al-Mansurah 1	1.7	1.1	2.4	15	10	22	0.5	0.3	0.7	6	4	9
Egypt	Al-Mansurah 2	1.7	1.1	2.4	24	16	34	0.5	0.3	0.7	9	6	14
Egypt	Al-Manzilah	1.8	1.2	2.6	49	33	70	0.5	0.3	0.7	18	10	27
Egypt	Al-Maraghah	2.9	1.9	4.2	102	68	146	0.7	0.4	1.0	37	23	54
Egypt	Al-Marj	1.5	1.0	2.0	86	59	118	0.5	0.3	0.8	34	21	53
Egypt	Al-Matariyah	1.5	1.0	2.0	52	35	71	0.5	0.3	0.8	21	13	33
Egypt	Al-Matariyah	1.8	1.2	2.5	19	13	27	0.5	0.3	0.7	8	4	12
Egypt	Al-Minya	2.8	2.0	4.1	183	126	264	0.7	0.4	1.0	67	41	99
Egypt	Al-Minya City	2.8	1.9	4.0	38	26	54	0.7	0.4	1.0	13	8	20
Egypt	Al-Muntazah	1.5	1.0	2.2	124	80	181	0.4	0.2	0.6	42	27	66
Egypt	Al-Muski	1.4	0.9	2.1	3	2	5	0.5	0.3	0.8	1	1	2
Egypt	Al-Qanatir al-Khayriyah	1.5	1.0	2.0	77	52	104	0.5	0.3	0.8	39	25	61
Egypt	Al-Qanayat	1.8	1.3	2.4	10	7	14	0.5	0.3	0.8	4	3	6
Egypt	Al-Qantarah	1.8	1.2	2.7	26	18	39	0.5	0.3	0.8	10	7	15
Egypt	Al-Qantarah ash-Sharqiyah	1.8	1.2	2.6	15	10	21	0.5	0.3	0.7	6	4	9

Table 3: Diarrhoea Mortality by Unit *(continued)*

Country	Unit	2000 mortality mean rate (per 10,000)	lower rate (per 10,000)	upper rate (per 10,000)	mean count	lower count	upper count	2017 mortality mean rate (per 10,000)	lower rate (per 10,000)	upper rate (per 10,000)	mean count	lower count	upper count
Egypt	Al-Qurayn	1.8	1.2	2.5	8	5	10	0.5	0.3	0.8	3	2	5
Egypt	Al-Qusayr	1.8	1.1	2.8	8	5	13	0.5	0.3	0.7	3	2	5
Egypt	Al-Qusiyah	3.2	2.2	4.4	136	91	187	0.7	0.4	1.0	42	26	64
Egypt	Al-Wahat al-Bahariyah	2.1	1.3	3.2	0	0	1	0.5	0.3	0.7	0	0	0
Egypt	Al-Wahat al-Kharijah	2.0	1.5	2.7	33	24	44	0.5	0.4	0.7	11	8	15
Egypt	Al-Waili	1.4	0.9	2.1	19	13	28	0.5	0.3	0.8	8	5	12
Egypt	Al-Waqf	2.3	1.6	3.3	21	15	30	0.5	0.3	0.8	4	3	6
Egypt	Al-Warraq	1.5	1.0	2.0	143	95	198	0.5	0.3	0.8	88	55	136
Egypt	Al-Wasta	2.2	1.4	3.2	100	64	145	0.7	0.4	1.0	44	28	69
Egypt	An-Nuzhah	1.5	1.0	2.0	42	29	58	0.5	0.3	0.7	16	10	25
Egypt	Ancient Cairo	1.4	1.0	2.0	26	18	37	0.5	0.3	0.8	12	8	19
Egypt	Ar-Rahmaniyah	1.6	1.1	2.3	28	19	39	0.5	0.3	0.7	11	7	18
Egypt	Ar-Raml 1	1.5	0.9	2.3	28	17	41	0.4	0.2	0.6	10	6	15
Egypt	Ar-Raml 2	1.5	1.0	2.3	51	32	77	0.4	0.2	0.6	18	11	29
Egypt	Ar-Riyad	1.6	1.1	2.3	25	17	35	0.5	0.3	0.7	11	7	16
Egypt	Armant	2.3	1.5	3.2	34	22	48	0.5	0.3	0.9	14	8	21
Egypt	As-Saff	1.6	1.0	2.2	54	35	77	0.5	0.3	0.7	23	15	34
Egypt	As-Sajil	1.4	1.0	2.0	26	18	37	0.5	0.3	0.8	13	8	19
Egypt	As-Salam	1.5	1.0	2.1	74	51	102	0.5	0.3	0.8	29	18	44
Egypt	As-Salum	1.8	1.1	2.8	2	1	4	0.4	0.3	0.6	1	1	1
Egypt	As-Santah	1.5	1.0	2.1	66	44	93	0.5	0.3	0.7	32	20	48
Egypt	As-Sayidah Zaynab	1.4	1.0	2.0	9	6	12	0.5	0.3	0.8	4	3	6
Egypt	As-Sinbillawayn	1.8	1.2	2.5	82	56	113	0.5	0.3	0.7	33	21	49
Egypt	Ash-Shalatin	2.7	2.0	3.4	6	5	8	0.6	0.5	0.8	2	1	2
Egypt	Ash-Sharabiyah	1.4	0.9	2.0	19	13	27	0.5	0.3	0.7	8	5	12
Egypt	Ash-Sharq	1.8	1.1	2.6	4	3	6	0.5	0.3	0.7	2	1	2
Egypt	Ash-Shruq	1.5	1.1	2.1	8	6	11	0.5	0.3	0.8	3	2	5
Egypt	Ash-Shuhada	1.5	1.0	2.2	47	30	66	0.5	0.3	0.8	22	14	35
Egypt	Ashmun	1.5	1.0	2.2	111	72	160	0.5	0.3	0.8	57	36	92
Egypt	Aswan	2.2	1.4	3.4	46	28	71	0.5	0.3	0.7	13	8	20
Egypt	Aswan City	1.6	1.0	2.4	0	0	0	0.4	0.2	0.5	0	0	0
Egypt	Asyut	3.7	2.7	5.0	191	142	259	0.7	0.4	1.1	53	34	82
Egypt	Asyut 1	3.6	2.6	4.9	47	33	63	0.7	0.4	1.1	12	7	19
Egypt	Asyut 2	3.5	2.4	4.8	56	39	76	0.7	0.4	1.1	17	11	27
Egypt	At-Tall al-Kabir	1.8	1.2	2.5	28	18	38	0.5	0.3	0.8	11	7	18
Egypt	At-Tebin	1.5	1.0	2.1	14	9	20	0.5	0.3	0.8	5	3	7
Egypt	At-Tur	1.2	0.8	1.8	2	1	3	0.3	0.2	0.5	1	0	1
Egypt	Atfih	1.9	1.2	2.7	64	41	91	0.6	0.4	0.9	23	15	36
Egypt	Awlad Saqr	1.9	1.3	2.7	33	22	46	0.5	0.3	0.8	13	8	20
Egypt	Az-Zahir	1.4	0.9	2.1	10	6	14	0.5	0.3	0.8	4	2	6
Egypt	Az-Zaytun	1.4	0.9	2.0	39	26	55	0.5	0.3	0.7	16	10	25
Egypt	Az-Zohur	1.8	1.1	2.6	23	14	34	0.5	0.3	0.7	8	5	13
Egypt	Bab ash-Sha'riyah	1.4	0.9	2.1	3	2	5	0.5	0.3	0.8	1	1	2
Egypt	Bab Sharqi	1.5	1.0	2.2	15	10	22	0.4	0.2	0.6	5	3	8
Egypt	Badr	1.6	1.0	2.2	2	2	3	0.5	0.3	0.8	1	1	2
Egypt	Badr	1.5	1.0	2.1	33	22	45	0.5	0.3	0.7	15	10	23
Egypt	Banha	1.6	1.1	2.1	94	64	129	0.5	0.3	0.8	44	29	67
Egypt	Bani Mazar	2.8	2.0	4.0	153	106	220	0.7	0.4	1.1	58	35	87
Egypt	Bani Suwayf	2.5	1.7	3.7	146	101	218	0.7	0.4	1.1	61	37	92
Egypt	Bani Suwayf City	2.1	1.5	3.1	6	4	8	0.6	0.4	0.9	3	2	4
Egypt	Bani Ubayd	1.7	1.2	2.4	17	12	24	0.5	0.3	0.7	7	4	10
Egypt	Baris Shurtah	2.0	1.3	3.0	0	0	0	0.5	0.3	0.8	0	0	0
Egypt	Basyun	1.5	1.0	2.1	39	27	54	0.5	0.3	0.7	17	11	26
Egypt	Biba	2.7	1.9	3.8	111	77	159	0.7	0.5	1.1	46	30	70
Egypt	Bilbays	1.8	1.3	2.5	114	81	156	0.5	0.3	0.8	49	33	76
Egypt	Bilqas	1.7	1.1	2.3	73	48	99	0.5	0.3	0.7	29	17	43

Table 3: Diarrhoea Mortality by Unit *(continued)*

Country	Unit	2000 mortality						2017 mortality					
		mean rate (per 10,000)	lower rate (per 10,000)	upper rate (per 10,000)	mean count	lower count	upper count	mean rate (per 10,000)	lower rate (per 10,000)	upper rate (per 10,000)	mean count	lower count	upper count
Egypt	Bir al-'Abd	2.2	1.5	3.1	15	10	21	0.7	0.4	1.0	7	5	10
Egypt	Birkat as-Sab'	1.5	1.0	2.1	43	29	59	0.5	0.3	0.7	20	13	30
Egypt	Biyala	1.6	1.1	2.3	40	28	57	0.5	0.3	0.7	17	11	26
Egypt	Bulaq	1.4	0.9	2.0	5	4	8	0.5	0.3	0.8	3	2	4
Egypt	Bulaq al-Dakrur	1.5	1.0	2.0	53	36	74	0.5	0.3	0.8	34	22	54
Egypt	Burj al-'Arab	2.2	1.5	3.2	16	11	24	0.6	0.3	0.8	6	4	9
Egypt	Dahab	1.2	0.7	1.7	0	0	1	0.3	0.2	0.5	0	0	0
Egypt	Damanhur	1.7	1.1	2.4	116	80	167	0.5	0.3	0.7	47	31	71
Egypt	Damietta	1.7	1.1	2.7	59	37	91	0.5	0.3	0.8	23	13	40
Egypt	Damietta 1	1.8	1.1	2.7	7	4	10	0.5	0.3	0.8	3	2	5
Egypt	Damietta 2	1.7	1.0	2.6	9	5	14	0.4	0.2	0.8	3	2	6
Egypt	Dar as-Salam	3.1	2.1	4.1	136	92	182	0.7	0.5	1.0	44	29	65
Egypt	Daraw	2.4	1.6	3.3	17	11	23	0.5	0.3	0.7	5	3	8
Egypt	Dayr Mawas	3.1	2.1	4.5	124	86	179	0.7	0.4	1.0	40	25	59
Egypt	Dayrut	3.1	2.2	4.4	179	124	252	0.7	0.4	1.0	57	36	86
Egypt	Dikirnis	1.9	1.2	2.7	50	33	73	0.5	0.3	0.8	19	12	30
Egypt	Dishna	2.3	1.6	3.3	103	73	149	0.5	0.3	0.8	22	14	31
Egypt	Disuq	1.6	1.1	2.3	78	54	111	0.5	0.3	0.7	33	21	52
Egypt	Diyarb Najm	1.8	1.2	2.5	58	39	80	0.5	0.3	0.8	24	16	35
Egypt	Fa'id	1.7	1.1	2.5	17	11	26	0.5	0.3	0.7	8	5	12
Egypt	Faisal	1.5	1.0	2.2	21	14	31	0.5	0.3	0.8	11	6	16
Egypt	Faqus	1.9	1.2	2.7	114	73	159	0.5	0.3	0.8	45	28	70
Egypt	Faraskur	2.0	1.3	2.8	38	25	54	0.5	0.3	0.8	15	8	22
Egypt	Farshut	2.3	1.6	3.2	46	32	63	0.5	0.3	0.8	9	6	13
Egypt	Fuwah	1.7	1.1	2.4	25	18	37	0.5	0.3	0.7	10	6	16
Egypt	Gamsa	1.8	1.2	2.4	1	0	1	0.5	0.3	0.7	0	0	0
Egypt	Giza	1.5	1.0	2.0	107	74	150	0.5	0.3	0.8	59	38	89
Egypt	Hada'iq al-Qubbah	1.4	0.9	2.0	26	18	38	0.5	0.3	0.7	11	7	17
Egypt	Hawsh 'Isa	1.7	1.1	2.4	34	22	47	0.5	0.3	0.7	14	9	21
Egypt	Heliopolis	1.4	0.9	2.0	28	19	40	0.5	0.3	0.7	11	7	17
Egypt	Helwan	1.4	1.0	2.0	96	65	136	0.5	0.3	0.7	39	24	61
Egypt	Hihya	1.9	1.2	2.6	37	24	50	0.5	0.3	0.8	15	10	24
Egypt	Ibshaway	2.5	1.8	3.7	93	65	135	0.7	0.4	1.0	40	25	60
Egypt	Idfu	2.2	1.5	3.2	50	34	72	0.5	0.3	0.7	17	11	25
Egypt	Idku	1.7	1.2	2.6	24	17	36	0.5	0.3	0.7	10	7	14
Egypt	Ihnasiya	2.7	1.9	3.9	100	70	142	0.8	0.5	1.3	46	29	71
Egypt	Imbabah	1.5	1.0	2.0	96	65	130	0.5	0.3	0.8	60	39	94
Egypt	Ismailia	1.8	1.2	2.6	53	34	77	0.5	0.3	0.7	22	14	33
Egypt	Ismailia 1	1.8	1.1	2.6	11	7	15	0.5	0.3	0.7	5	3	7
Egypt	Ismailia 2	1.8	1.1	2.5	16	10	23	0.5	0.3	0.7	7	4	10
Egypt	Ismailia 3	1.7	1.1	2.5	13	8	19	0.5	0.3	0.7	6	4	9
Egypt	Isna	2.3	1.6	3.2	82	57	115	0.5	0.3	0.9	17	11	29
Egypt	Itsa	2.6	1.8	3.8	152	107	218	0.7	0.4	1.1	64	41	99
Egypt	Ityay al-Barud	1.7	1.2	2.4	74	53	100	0.5	0.3	0.8	33	21	49
Egypt	Jirja	2.9	2.0	3.9	109	76	146	0.7	0.4	1.0	38	25	55
Egypt	Juhaynah al-Gharbiyah	3.0	1.9	4.2	65	42	92	0.7	0.4	1.0	23	15	35
Egypt	Kafr ad-Dawwar	1.7	1.1	2.6	144	95	215	0.5	0.3	0.7	56	37	85
Egypt	Kafr ash-Shaykh	1.6	1.1	2.2	85	58	120	0.5	0.3	0.8	37	23	60
Egypt	Kafr az-Zayyat	1.5	1.0	2.2	70	48	100	0.5	0.3	0.8	30	19	47
Egypt	Kafr Sa'd	2.0	1.4	2.7	56	38	77	0.5	0.3	0.8	24	14	35
Egypt	Kafr Saqr	1.9	1.3	2.8	44	29	63	0.5	0.3	0.8	17	11	27
Egypt	Kafr Shukr	1.6	1.1	2.1	27	19	36	0.5	0.3	0.7	13	8	18
Egypt	Karmuz	1.5	1.0	2.3	10	6	15	0.4	0.3	0.6	4	2	6
Egypt	Kawm Hamadah	1.5	1.1	2.1	68	47	95	0.5	0.3	0.8	32	21	51
Egypt	Kawm Umbu	2.4	1.6	3.4	63	42	90	0.5	0.3	0.7	18	11	26
Egypt	Kirdasah	1.5	1.0	2.0	101	68	140	0.5	0.3	0.8	64	41	101
Egypt	Luxor	2.2	1.5	3.2	45	30	64	0.5	0.3	0.8	95	55	146
Egypt	Maghaghah	2.8	2.0	4.2	147	102	217	0.7	0.4	1.1	57	35	86

Table 3: Diarrhoea Mortality by Unit *(continued)*

Country	Unit	2000 mortality mean rate (per 10,000)	lower rate (per 10,000)	upper rate (per 10,000)	mean count	lower count	upper count	2017 mortality mean rate (per 10,000)	lower rate (per 10,000)	upper rate (per 10,000)	mean count	lower count	upper count
Egypt	Mahalat Dimnah	1.8	1.2	2.6	8	6	12	0.5	0.3	0.7	3	2	5
Egypt	Mallawi	3.0	2.1	4.2	211	148	298	0.7	0.4	1.0	73	45	111
Egypt	Mallawi City	3.0	2.1	4.4	33	23	49	0.7	0.4	1.0	11	7	17
Egypt	Manfalut	3.5	2.5	4.9	191	139	266	0.7	0.4	1.0	56	35	86
Egypt	Marina al-'Alamayn as-Siyahiyah	2.7	1.7	3.8	1	1	2	0.6	0.4	1.0	1	1	1
Egypt	Marsa 'Alam	1.6	1.1	2.3	1	0	1	0.5	0.3	0.7	0	0	0
Egypt	Marsa Matruh	2.6	1.7	3.7	55	37	80	0.6	0.4	0.8	21	14	31
Egypt	Mashtul as-Suq	1.7	1.2	2.3	27	19	37	0.5	0.3	0.8	13	8	21
Egypt	Matay	2.8	2.0	4.1	84	59	121	0.7	0.4	1.1	33	20	48
Egypt	Minuf	1.5	1.0	2.2	65	42	97	0.5	0.3	0.8	32	20	51
Egypt	Minuf City	1.5	0.9	2.2	11	7	17	0.5	0.3	0.8	5	3	8
Egypt	Minya al-Qamh	1.7	1.2	2.3	97	67	131	0.5	0.3	0.8	44	29	67
Egypt	Minyat an-Nasr	1.9	1.2	2.7	41	27	60	0.5	0.3	0.8	16	10	24
Egypt	Mit Ghamr	1.6	1.1	2.2	102	71	140	0.5	0.3	0.7	45	30	65
Egypt	Mit Salsil	1.8	1.2	2.6	14	9	19	0.5	0.3	0.7	5	3	8
Egypt	Monshat Nasr	1.4	1.0	2.1	16	11	23	0.5	0.3	0.7	6	4	9
Egypt	Mubarak - Sharq at-Tafri'tah	1.9	1.3	2.7	3	2	4	0.5	0.3	0.7	1	1	1
Egypt	Muharam Bik	1.5	1.0	2.2	25	16	37	0.4	0.2	0.6	9	6	14
Egypt	Mutubis	1.7	1.2	2.5	48	34	72	0.5	0.3	0.7	20	13	30
Egypt	Nabaruh	1.7	1.1	2.4	39	26	56	0.5	0.3	0.7	16	10	25
Egypt	Naj' Hammadi	2.4	1.7	3.4	134	93	192	0.5	0.3	0.8	26	17	39
Egypt	Nakhl	2.3	1.6	3.2	2	1	2	0.7	0.5	1.0	1	1	1
Egypt	Naqadah	2.3	1.5	3.2	45	30	63	0.5	0.3	0.8	12	7	18
Egypt	Nasir Bush	2.5	1.8	3.7	86	60	124	0.7	0.5	1.1	37	24	56
Egypt	Nasr	2.4	1.6	3.4	18	13	26	0.5	0.3	0.7	6	3	8
Egypt	Nasr City 1	1.5	1.0	2.0	61	43	85	0.5	0.3	0.8	23	14	36
Egypt	Nasr City 2	1.4	1.0	2.0	17	12	25	0.5	0.3	0.7	7	4	11
Egypt	New Akhmim City	1.5	1.0	2.1	0	0	0	0.3	0.2	0.5	0	0	0
Egypt	New Asyut City	3.4	2.4	4.6	0	0	0	0.7	0.4	1.1	0	0	0
Egypt	New Burj al-'Arab City	2.0	1.3	2.9	10	7	14	0.5	0.3	0.8	3	2	5
Egypt	New Cairo 1	1.5	1.0	2.1	7	5	10	0.5	0.3	0.8	3	2	5
Egypt	New Cairo 2	1.5	1.0	2.1	7	5	10	0.5	0.3	0.8	3	2	4
Egypt	New Cairo 3	1.5	1.0	2.1	8	5	11	0.5	0.3	0.8	3	2	5
Egypt	New Damietta City	1.8	1.1	2.5	10	6	15	0.5	0.3	0.7	4	2	6
Egypt	New Minya City	2.7	1.8	3.8	9	6	13	0.7	0.4	1.0	3	2	5
Egypt	New Salhiyah	1.9	1.2	2.6	20	13	28	0.5	0.3	0.8	9	6	14
Egypt	New Sawhaj City	2.5	1.6	3.6	0	0	0	0.6	0.4	0.9	0	0	0
Egypt	New Tushka City	2.2	1.3	3.5	0	0	1	0.6	0.4	0.9	0	0	0
Egypt	Nuweiba'	1.4	0.9	2.0	1	1	2	0.4	0.3	0.6	0	0	1
Egypt	Port al-Basal	1.5	1.0	2.3	14	9	21	0.4	0.3	0.6	5	3	8
Egypt	Port Alexandria Police Department	1.5	1.0	2.3	8	5	11	0.4	0.3	0.6	3	2	4
Egypt	Port Fuad	1.7	1.1	2.6	3	2	5	0.5	0.3	0.7	1	1	2
Egypt	Port Fuad 2	1.8	1.1	2.6	3	2	5	0.5	0.3	0.7	1	1	2
Egypt	Port of Damietta Police Department	1.7	1.0	2.6	2	1	3	0.4	0.3	0.8	1	0	1
Egypt	Port Sa'id Police Department	1.8	1.1	2.6	6	4	9	0.5	0.3	0.7	2	1	3
Egypt	Port Suez Police Department	1.5	0.9	2.3	0	0	0	0.5	0.3	0.8	0	0	0
Egypt	Qaha	1.6	1.1	2.2	7	5	9	0.5	0.3	0.8	3	2	5
Egypt	Qallin	1.6	1.1	2.2	34	23	48	0.5	0.3	0.8	15	10	25

Table 3: Diarrhoea Mortality by Unit *(continued)*

Country	Unit	2000 mortality mean rate (per 10,000)	lower rate (per 10,000)	upper rate (per 10,000)	mean count	lower count	upper count	2017 mortality mean rate (per 10,000)	lower rate (per 10,000)	upper rate (per 10,000)	mean count	lower count	upper count
Egypt	Qalyub	1.5	1.0	2.1	132	89	179	0.5	0.3	0.8	63	41	99
Egypt	Qasr an-Nil	1.4	0.9	2.0	4	3	5	0.5	0.3	0.8	2	1	3
Egypt	Qift	2.2	1.5	3.1	34	22	48	0.5	0.3	0.7	7	4	10
Egypt	Qina	2.2	1.4	3.1	125	81	178	0.5	0.3	0.8	27	17	38
Egypt	Qina City	2.2	1.4	3.2	2	1	3	0.5	0.3	0.7	0	0	1
Egypt	Qus	2.2	1.4	3.1	99	65	139	0.5	0.3	0.8	22	14	33
Egypt	Qutur	1.5	1.1	2.1	47	33	64	0.5	0.3	0.8	21	13	33
Egypt	Quwaysina	1.5	1.1	2.1	73	50	101	0.5	0.3	0.7	34	22	50
Egypt	Rafah	2.7	1.7	4.0	36	22	53	0.7	0.4	1.0	14	9	22
Egypt	Ras Gharib	1.7	1.1	2.4	4	3	6	0.5	0.3	0.7	2	1	2
Egypt	Ras Sidr	1.3	0.9	1.9	2	1	3	0.4	0.3	0.6	1	1	1
Egypt	Rosetta	1.7	1.1	2.6	37	25	56	0.5	0.3	0.7	14	9	22
Egypt	Rud al-Faraj	1.4	0.9	2.0	7	5	11	0.5	0.3	0.8	4	2	6
Egypt	Sadat City	1.6	1.1	2.3	28	18	40	0.5	0.3	0.8	13	8	20
Egypt	Safaja	1.8	1.2	2.6	11	7	16	0.5	0.3	0.7	5	3	7
Egypt	Sahil Salim	3.4	2.5	4.6	53	38	71	0.7	0.4	1.0	15	10	22
Egypt	Samalut	2.8	1.9	4.1	183	125	269	0.7	0.4	1.0	69	43	104
Egypt	Samannud	1.6	1.1	2.2	59	41	83	0.5	0.3	0.7	25	17	38
Egypt	Sant Katrin	1.1	0.7	1.6	1	1	1	0.3	0.2	0.4	0	0	0
Egypt	Saqultah	2.9	1.9	4.2	47	31	67	0.7	0.4	1.0	16	10	23
Egypt	Sawhaj	2.9	1.8	4.3	200	127	301	0.7	0.4	1.1	70	44	107
Egypt	Sawhaj 2	2.9	1.8	4.4	8	5	12	0.7	0.4	1.1	3	2	5
Egypt	Sharm el-Sheikh	1.4	0.9	2.1	5	3	8	0.4	0.2	0.6	2	1	3
Egypt	Sheikh Zawid	2.2	1.4	3.2	13	8	18	0.6	0.4	1.0	6	4	9
Egypt	Sheikh Zayed	1.5	1.0	2.2	3	2	5	0.5	0.3	0.8	2	1	3
Egypt	Shibin al-Kawm	1.5	1.0	2.1	87	60	125	0.5	0.3	0.8	42	27	63
Egypt	Shibin al-Qanatir	1.6	1.1	2.2	90	62	120	0.5	0.3	0.8	42	26	65
Egypt	Shirbin	1.8	1.2	2.6	59	40	84	0.5	0.3	0.8	23	14	36
Egypt	Shubra	1.4	0.9	2.0	5	3	7	0.5	0.3	0.8	2	1	3
Egypt	Shubra al-Khaymah 1	1.5	1.0	2.1	52	35	73	0.5	0.3	0.8	25	16	38
Egypt	Shubra al-Khaymah 2	1.5	1.0	2.1	111	75	153	0.5	0.3	0.8	50	31	77
Egypt	Shubra Khit	1.6	1.1	2.2	39	27	52	0.5	0.3	0.7	17	11	25
Egypt	Shurtah al-Dakhlah	1.9	1.2	2.8	19	13	28	0.5	0.3	0.7	7	4	10
Egypt	Shurtah al-Farafirah	2.1	1.3	3.3	7	4	10	0.5	0.3	0.7	2	1	3
Egypt	Shurtah al-Qasimah	2.3	1.4	3.2	1	1	1	0.7	0.4	1.0	0	0	1
Egypt	Shurtah Rumanah	2.0	1.3	3.1	8	5	12	0.6	0.4	1.0	4	2	6
Egypt	Sidfa	3.3	2.3	4.7	70	49	98	0.7	0.4	1.0	20	12	30
Egypt	Sidi Barrani	2.3	1.4	3.4	7	4	11	0.5	0.3	0.7	3	2	4
Egypt	Sidi Jabir	1.5	1.0	2.2	33	21	49	0.4	0.2	0.6	11	7	18
Egypt	Sidi Salim	1.6	1.1	2.4	53	37	77	0.5	0.3	0.7	23	15	35
Egypt	Sinnuris	2.4	1.7	3.4	131	91	185	0.7	0.4	1.1	59	36	91
Egypt	Sirs al-Layyanah	1.5	0.9	2.2	8	5	12	0.5	0.3	0.8	4	2	6
Egypt	Siwa	2.7	1.8	4.0	7	5	10	0.5	0.4	0.8	2	2	4
Egypt	Sixth of October 1 City	1.6	1.0	2.2	5	3	6	0.5	0.3	0.9	3	2	5
Egypt	Sixth of October 2 City	1.6	0.9	2.3	15	9	22	0.5	0.3	0.9	9	5	15
Egypt	Suez	1.5	0.9	2.3	7	4	10	0.5	0.3	0.8	4	2	5
Egypt	Sumusta al-Waqf	2.7	1.9	3.7	65	45	91	0.8	0.5	1.2	29	18	44
Egypt	Taba	1.9	1.3	2.9	0	0	1	0.5	0.3	0.7	0	0	0
Egypt	Tahta	3.0	2.0	4.2	92	62	127	0.7	0.4	1.0	30	19	46
Egypt	Tahta City	2.9	2.0	4.0	20	13	27	0.7	0.4	1.0	6	4	9
Egypt	Tala	1.6	1.1	2.1	54	37	75	0.5	0.3	0.8	25	16	38
Egypt	Talkha	1.7	1.2	2.5	61	40	86	0.5	0.3	0.7	24	15	36

Table 3: Diarrhoea Mortality by Unit *(continued)*

Country	Unit	2000 mortality mean rate (per 10,000)	2000 mortality lower rate (per 10,000)	2000 mortality upper rate (per 10,000)	2000 mortality mean count	2000 mortality lower count	2000 mortality upper count	2017 mortality mean rate (per 10,000)	2017 mortality lower rate (per 10,000)	2017 mortality upper rate (per 10,000)	2017 mortality mean count	2017 mortality lower count	2017 mortality upper count
Egypt	Tamiyah	2.4	1.7	3.4	88	60	122	0.7	0.4	1.0	41	24	60
Egypt	Tanta	1.5	1.0	2.1	123	85	170	0.5	0.3	0.7	56	37	86
Egypt	Tanta 1	1.4	1.0	2.0	13	9	18	0.5	0.3	0.7	6	4	8
Egypt	Tanta 2	1.4	1.0	2.0	10	7	14	0.5	0.3	0.7	4	3	7
Egypt	Tibah Police Dept.	2.2	1.5	3.2	7	5	11	0.5	0.3	0.8	16	10	25
Egypt	Tima	3.2	2.2	4.4	109	75	151	0.7	0.4	1.0	36	23	54
Egypt	Timay al-Imdid	1.9	1.3	2.6	28	20	40	0.5	0.3	0.8	11	7	17
Egypt	Tukh	1.6	1.1	2.2	93	63	128	0.5	0.3	0.8	46	30	70
Egypt	Turah	1.5	1.0	2.1	20	14	30	0.5	0.3	0.8	8	5	13
Egypt	Unorganized in Al Buhayrah	1.8	1.2	2.4	14	9	19	0.5	0.3	0.8	6	4	10
Egypt	Unorganized in Al Fayoum	2.5	1.9	3.5	41	30	56	0.7	0.5	1.0	18	12	28
Egypt	Unorganized in Al Iskandariyah	1.6	1.1	2.4	0	0	0	0.4	0.3	0.6	0	0	0
Egypt	Unorganized in Al Jizah	1.6	1.2	2.1	81	59	105	0.5	0.4	0.7	43	29	61
Egypt	Unorganized in Al Minya	2.8	2.1	3.9	73	55	101	0.7	0.4	0.9	28	18	38
Egypt	Unorganized in Al Qahirah	1.6	1.1	2.1	10	7	13	0.5	0.3	0.8	4	2	6
Egypt	Unorganized in Al Qalyubiyah	1.7	1.2	2.3	14	10	20	0.5	0.3	0.8	6	4	9
Egypt	Unorganized in Al Uqsur	2.2	1.5	3.2	4	3	6	0.5	0.3	0.8	10	6	15
Egypt	Unorganized in Ash Sharqiyah	1.7	1.2	2.4	20	14	27	0.5	0.3	0.8	8	5	14
Egypt	Unorganized in Aswan	2.0	1.5	2.7	94	68	126	0.5	0.3	0.6	31	22	43
Egypt	Unorganized in Asyut	3.3	2.5	4.4	136	101	179	0.7	0.5	1.0	46	31	68
Egypt	Unorganized in Bani Suwayf	2.5	1.9	3.6	29	21	41	0.7	0.5	1.0	13	8	18
Egypt	Unorganized in Qina	2.3	1.6	3.0	99	71	132	0.5	0.4	0.7	28	18	38
Egypt	Unorganized in Suhaj	2.8	2.0	3.8	78	55	104	0.7	0.4	0.9	30	20	40
Egypt	Wadi Al-Natron	1.8	1.2	2.5	1	1	2	0.5	0.3	0.8	1	0	1
Egypt	West Nubariyah	1.8	1.2	2.5	7	5	11	0.5	0.3	0.7	3	2	5
Egypt	Yusuf as-Sidiq	2.6	1.8	3.9	80	54	117	0.7	0.4	1.0	33	21	50
Egypt	Zamalik	1.4	0.9	2.0	8	5	12	0.5	0.3	0.8	4	2	6
Egypt	Zaqaziq	1.8	1.3	2.5	155	110	211	0.5	0.3	0.8	65	43	99
Egypt	Zaqaziq 1	1.8	1.3	2.5	7	5	9	0.5	0.3	0.8	3	2	4
Egypt	Zaqaziq 2	1.8	1.3	2.5	9	7	13	0.5	0.3	0.8	4	2	6
Egypt	Zarqa	1.9	1.2	2.9	20	13	31	0.5	0.3	0.8	8	5	12
Egypt	Zawiyya Al-Hamra	1.4	0.9	2.0	40	27	57	0.5	0.3	0.7	17	11	27
Egypt	Zifta	1.5	1.0	2.1	82	55	113	0.5	0.3	0.7	38	24	54
Iraq	Abu al Khasib	0.3	0.2	0.4	7	4	10	0.1	0.0	0.2	4	2	6
Iraq	Abu Ghraib	0.2	0.2	0.4	6	4	8	0.1	0.0	0.2	2	1	4
Iraq	Ad Diwaniyah	0.2	0.1	0.3	16	11	24	0.1	0.1	0.2	11	6	19
Iraq	Adhamiya	0.2	0.1	0.3	103	70	144	0.1	0.0	0.2	53	28	90
Iraq	Afak	0.2	0.1	0.3	6	4	10	0.1	0.0	0.2	4	2	6
Iraq	Ain Al Tamur	0.3	0.2	0.4	1	1	1	0.1	0.0	0.2	1	0	1
Iraq	Akre	0.3	0.2	0.5	5	3	7	0.1	0.0	0.2	1	1	2
Iraq	Al Amarah	0.3	0.2	0.4	20	14	28	0.1	0.1	0.2	11	6	19
Iraq	Al Ba'aj	0.2	0.1	0.4	4	2	6	0.1	0.1	0.2	2	1	4
Iraq	Al Door	0.2	0.1	0.3	1	0	1	0.1	0.0	0.2	1	0	1
Iraq	Al Fallujah	0.2	0.2	0.4	24	16	35	0.1	0.0	0.2	11	5	18

Table 3: Diarrhoea Mortality by Unit *(continued)*

Country	Unit	2000 mortality mean rate (per 10,000)	lower rate (per 10,000)	upper rate (per 10,000)	mean count	lower count	upper count	2017 mortality mean rate (per 10,000)	lower rate (per 10,000)	upper rate (per 10,000)	mean count	lower count	upper count
Iraq	Al Faw	0.3	0.2	0.4	2	1	3	0.1	0.0	0.2	1	0	2
Iraq	Al Haditha	0.2	0.1	0.4	3	2	5	0.1	0.0	0.2	2	1	3
Iraq	Al Hamdaniyah	0.3	0.2	0.4	8	6	12	0.1	0.0	0.2	4	2	7
Iraq	Al Hamza	0.2	0.1	0.3	10	7	15	0.1	0.0	0.2	6	3	11
Iraq	Al Hashimiyah	0.2	0.1	0.3	11	8	16	0.1	0.0	0.2	8	4	13
Iraq	Al Hayy	0.3	0.2	0.4	6	4	9	0.1	0.1	0.2	3	2	6
Iraq	Al Hillah	0.2	0.2	0.3	25	18	35	0.1	0.0	0.2	15	7	27
Iraq	Al Jadwal al Gharbi	0.2	0.2	0.3	3	2	5	0.1	0.0	0.2	2	1	4
Iraq	Al Kahla	0.3	0.2	0.4	1	1	2	0.1	0.0	0.2	1	0	1
Iraq	Al Khalis	0.2	0.1	0.3	7	4	11	0.1	0.0	0.2	5	2	8
Iraq	Al Khithir	0.3	0.2	0.4	3	2	4	0.1	0.0	0.2	2	1	3
Iraq	Al Kufa	0.2	0.2	0.3	7	5	11	0.1	0.0	0.2	5	3	10
Iraq	Al Kut	0.3	0.2	0.4	14	9	22	0.1	0.1	0.2	8	4	16
Iraq	Al Madiana	0.3	0.2	0.4	6	4	9	0.1	0.0	0.2	3	2	6
Iraq	Al Mahawil	0.2	0.2	0.3	13	9	18	0.1	0.0	0.2	8	4	15
Iraq	Al Manathera	0.2	0.2	0.3	3	2	5	0.1	0.1	0.2	2	1	4
Iraq	Al Miamona	0.3	0.2	0.4	4	3	6	0.1	0.0	0.2	2	1	4
Iraq	Al Mijar al Kabir	0.3	0.2	0.4	6	4	9	0.1	0.0	0.2	3	1	5
Iraq	Al Miqdadiyah	0.2	0.1	0.3	5	3	8	0.1	0.0	0.2	3	2	6
Iraq	Al Misiab	0.2	0.2	0.4	6	4	9	0.1	0.0	0.2	4	2	7
Iraq	Al Noamania	0.2	0.1	0.3	4	2	5	0.1	0.0	0.2	2	1	4
Iraq	Al Qa'im	0.2	0.1	0.4	5	3	9	0.1	0.0	0.2	2	1	4
Iraq	Al Qurnah	0.3	0.2	0.4	9	6	14	0.1	0.1	0.2	5	2	9
Iraq	Al Shikhan	0.3	0.2	0.4	1	1	2	0.1	0.0	0.2	1	0	1
Iraq	Al Shirkat	0.2	0.1	0.3	3	2	5	0.1	0.0	0.2	2	1	4
Iraq	Al Zubair	0.3	0.2	0.4	13	9	18	0.1	0.1	0.2	7	4	11
Iraq	Al-Faris	0.2	0.1	0.3	36	25	52	0.1	0.0	0.2	20	10	33
Iraq	Al-Mada'in	0.2	0.2	0.3	8	5	12	0.1	0.0	0.2	4	2	7
Iraq	Ali al Gharbi	0.3	0.2	0.4	2	1	4	0.1	0.1	0.2	1	1	2
Iraq	Amedi	0.3	0.2	0.4	6	4	9	0.1	0.1	0.2	5	3	8
Iraq	An Nasiriyah	0.3	0.2	0.4	32	22	45	0.1	0.1	0.2	14	7	23
Iraq	Anah	0.2	0.1	0.4	2	1	3	0.1	0.0	0.2	1	0	1
Iraq	Ar Ramadi	0.2	0.2	0.4	20	13	30	0.1	0.0	0.2	9	5	15
Iraq	Ar Rutbah	0.2	0.2	0.3	1	1	2	0.1	0.1	0.1	1	0	1
Iraq	Arbil	0.4	0.2	0.5	25	16	36	0.1	0.0	0.2	9	4	15
Iraq	As Salman	0.2	0.2	0.3	1	1	1	0.1	0.1	0.2	0	0	1
Iraq	As Samawah	0.2	0.2	0.3	8	6	12	0.1	0.0	0.2	6	3	11
Iraq	As Suwayrah	0.2	0.1	0.3	10	6	14	0.1	0.1	0.2	6	3	11
Iraq	Ba'qubah	0.2	0.1	0.3	24	16	35	0.1	0.0	0.2	15	7	24
Iraq	Badrah	0.2	0.1	0.4	1	0	1	0.1	0.1	0.2	0	0	1
Iraq	Balad	0.2	0.1	0.3	17	12	25	0.1	0.0	0.2	10	5	17
Iraq	Balad Ruz	0.2	0.1	0.3	8	5	12	0.1	0.0	0.2	4	2	7
Iraq	Basrah	0.3	0.2	0.4	45	31	69	0.1	0.1	0.2	23	12	42
Iraq	Bayji	0.2	0.1	0.3	3	2	5	0.1	0.0	0.2	3	1	5
Iraq	Chamchamal	0.2	0.1	0.3	6	4	9	0.1	0.0	0.2	3	1	5
Iraq	Chibayish	0.3	0.2	0.5	4	3	6	0.1	0.1	0.2	2	1	3
Iraq	Choman	0.3	0.2	0.5	5	3	7	0.1	0.0	0.2	1	1	2
Iraq	Dahuk	0.2	0.1	0.3	6	4	9	0.1	0.0	0.2	6	3	11
Iraq	Daquq	0.2	0.1	0.3	1	1	2	0.1	0.0	0.2	1	1	2
Iraq	Darbandokeh	0.2	0.1	0.4	2	1	3	0.1	0.0	0.2	1	0	1
Iraq	Dibis	0.2	0.1	0.4	1	1	1	0.1	0.0	0.2	0	0	1
Iraq	Dukan	0.3	0.2	0.4	3	2	4	0.1	0.0	0.2	1	0	2
Iraq	Halabja	0.2	0.1	0.4	4	2	6	0.1	0.0	0.1	1	1	2
Iraq	Hatra	0.2	0.1	0.3	2	1	3	0.1	0.0	0.2	1	1	2
Iraq	Haweeja	0.2	0.1	0.3	5	3	7	0.1	0.0	0.2	4	2	7
Iraq	Hit	0.2	0.1	0.4	5	3	7	0.1	0.1	0.2	2	1	4
Iraq	Kadhimiya	0.2	0.2	0.3	12	8	17	0.1	0.0	0.2	5	2	8
Iraq	Kalar	0.2	0.1	0.3	3	1	4	0.1	0.0	0.1	1	1	2
Iraq	Karbala	0.3	0.2	0.4	20	14	29	0.1	0.0	0.2	13	6	21

Table 3: Diarrhoea Mortality by Unit *(continued)*

Country	Unit	2000 mortality mean rate (per 10,000)	lower rate (per 10,000)	upper rate (per 10,000)	mean count	lower count	upper count	2017 mortality mean rate (per 10,000)	lower rate (per 10,000)	upper rate (per 10,000)	mean count	lower count	upper count
Iraq	Khanaqin	0.2	0.1	0.3	7	4	10	0.1	0.0	0.1	4	2	6
Iraq	Kifri	0.2	0.1	0.3	1	1	2	0.1	0.0	0.1	1	0	1
Iraq	Kirkuk	0.2	0.1	0.3	19	13	28	0.1	0.0	0.2	16	8	29
Iraq	Koisnjaq	0.3	0.2	0.4	15	9	22	0.1	0.0	0.2	5	2	9
Iraq	Mahmudiya	0.2	0.2	0.4	7	5	10	0.1	0.0	0.2	4	2	7
Iraq	Makhmur	0.3	0.2	0.4	2	1	3	0.1	0.0	0.2	1	0	2
Iraq	Mergasur	0.3	0.2	0.5	7	4	11	0.1	0.0	0.2	2	1	3
Iraq	Mosul	0.2	0.2	0.3	62	43	89	0.1	0.0	0.2	36	18	62
Iraq	Najaf	0.2	0.2	0.3	15	11	22	0.1	0.1	0.2	11	6	18
Iraq	Penjwin	0.2	0.1	0.4	1	0	1	0.1	0.0	0.2	0	0	1
Iraq	Pshdar	0.3	0.2	0.4	3	2	4	0.1	0.0	0.2	1	0	2
Iraq	Qal'at Salih	0.3	0.2	0.4	3	2	5	0.1	0.0	0.2	2	1	3
Iraq	Rania	0.3	0.2	0.5	3	2	5	0.1	0.0	0.2	1	0	2
Iraq	Refai	0.3	0.2	0.5	15	10	22	0.1	0.1	0.2	7	4	11
Iraq	Rumaitha	0.2	0.2	0.3	9	7	14	0.1	0.0	0.2	6	3	12
Iraq	Samarra	0.2	0.1	0.3	8	5	12	0.1	0.0	0.2	5	3	9
Iraq	Shamiya	0.2	0.1	0.3	8	6	12	0.1	0.1	0.2	6	3	9
Iraq	Shaqlawa	0.4	0.2	0.6	15	10	24	0.1	0.0	0.2	5	2	8
Iraq	Sharbazher	0.2	0.1	0.4	5	3	8	0.1	0.0	0.2	2	1	3
Iraq	Shatrah	0.3	0.2	0.5	18	12	26	0.1	0.1	0.2	8	4	12
Iraq	Shatt Al Arab	0.3	0.2	0.4	10	7	15	0.1	0.0	0.2	5	2	10
Iraq	Shekhan	0.3	0.2	0.4	1	1	1	0.1	0.0	0.2	0	0	1
Iraq	Simele	0.2	0.1	0.3	8	6	12	0.1	0.0	0.2	8	4	14
Iraq	Sinjar	0.2	0.1	0.4	7	4	11	0.1	0.0	0.2	4	2	7
Iraq	Soran	0.3	0.2	0.5	14	8	23	0.1	0.0	0.2	3	2	6
Iraq	Sulaymaniya	0.2	0.1	0.4	23	14	34	0.1	0.0	0.2	9	4	16
Iraq	Suq ash Shuyukh	0.3	0.2	0.5	13	8	19	0.1	0.0	0.2	5	3	9
Iraq	Talafar	0.2	0.1	0.3	13	8	19	0.1	0.1	0.2	8	4	13
Iraq	Tikrit	0.2	0.1	0.3	4	3	7	0.1	0.0	0.2	3	1	5
Iraq	Tilkef	0.2	0.1	0.3	4	3	6	0.1	0.0	0.2	3	1	5
Iraq	Touz Hourmato	0.2	0.1	0.3	4	2	6	0.1	0.0	0.2	3	2	6
Iraq	Zakho	0.2	0.2	0.3	9	6	13	0.1	0.0	0.2	9	4	15
Jordan	Aghwar Shamaliyyeh	0.1	0.1	0.2	0	0	1	0.0	0.0	0.0	0	0	0
Jordan	Ajloun	0.1	0.1	0.2	2	1	3	0.0	0.0	0.0	1	0	1
Jordan	Al-Balqa	0.1	0.1	0.2	0	0	0	0.0	0.0	0.0	0	0	0
Jordan	Amman	0.1	0.1	0.2	9	5	17	0.0	0.0	0.0	5	3	8
Jordan	Aqaba	0.1	0.0	0.2	1	1	2	0.0	0.0	0.0	1	0	1
Jordan	Ar-Ruwayshid	0.1	0.1	0.1	0	0	0	0.0	0.0	0.0	0	0	0
Jordan	Ardhah	0.1	0.1	0.2	2	1	3	0.0	0.0	0.0	1	1	2
Jordan	Ayy	0.1	0.1	0.2	0	0	1	0.0	0.0	0.0	0	0	0
Jordan	Azraq	0.1	0.1	0.2	0	0	0	0.0	0.0	0.1	0	0	0
Jordan	Bal'ama	0.1	0.1	0.2	1	0	1	0.0	0.0	0.0	0	0	1
Jordan	Bani Kenanah	0.1	0.1	0.2	0	0	1	0.0	0.0	0.0	0	0	0
Jordan	Bierain	0.1	0.1	0.2	1	1	2	0.0	0.0	0.0	1	0	1
Jordan	Bsaira	0.1	0.1	0.2	0	0	1	0.0	0.0	0.0	0	0	0
Jordan	Dair Alla	0.1	0.1	0.2	1	0	1	0.0	0.0	0.0	0	0	0
Jordan	Dhiban	0.1	0.1	0.2	0	0	0	0.0	0.0	0.0	0	0	0
Jordan	Faqqoo'	0.1	0.1	0.2	0	0	0	0.0	0.0	0.0	0	0	0
Jordan	Ghour El-Mazra'ah	0.1	0.1	0.2	0	0	1	0.0	0.0	0.0	0	0	0
Jordan	Ghour Essafi	0.1	0.1	0.2	0	0	0	0.0	0.0	0.1	0	0	0
Jordan	Hariema	0.1	0.0	0.2	0	0	0	0.0	0.0	0.0	0	0	0
Jordan	Hesa	0.1	0.1	0.2	0	0	0	0.0	0.0	0.0	0	0	0
Jordan	Husseiniyyeh	0.1	0.1	0.2	0	0	0	0.0	0.0	0.0	0	0	0
Jordan	Iel	0.1	0.1	0.2	0	0	0	0.0	0.0	0.1	0	0	0
Jordan	Irbid	0.1	0.0	0.2	1	0	1	0.0	0.0	0.0	0	0	1
Jordan	Jarash	0.1	0.1	0.2	1	1	2	0.0	0.0	0.0	1	0	1
Jordan	Jizeh	0.1	0.1	0.2	0	0	1	0.0	0.0	0.1	0	0	0
Jordan	Karak	0.1	0.1	0.2	0	0	0	0.0	0.0	0.0	0	0	0

Table 3: Diarrhoea Mortality by Unit *(continued)*

Country	Unit	2000 mortality mean rate (per 10,000)	lower rate (per 10,000)	upper rate (per 10,000)	mean count	lower count	upper count	2017 mortality mean rate (per 10,000)	lower rate (per 10,000)	upper rate (per 10,000)	mean count	lower count	upper count
Jordan	Kofranjah	0.1	0.0	0.2	0	0	0	0.0	0.0	0.0	0	0	0
Jordan	Kora	0.1	0.0	0.2	0	0	1	0.0	0.0	0.0	0	0	0
Jordan	Ma'an	0.1	0.1	0.2	1	0	1	0.0	0.0	0.1	0	0	0
Jordan	Madaba	0.1	0.1	0.2	2	1	3	0.0	0.0	0.0	1	1	1
Jordan	Mafraq	0.1	0.0	0.2	1	1	2	0.0	0.0	0.0	1	1	1
Jordan	Mazar Janoobi	0.1	0.1	0.2	1	0	1	0.0	0.0	0.0	0	0	0
Jordan	Mazar Shamali	0.1	0.0	0.2	1	1	2	0.0	0.0	0.0	0	0	1
Jordan	Mowaqqar	0.1	0.1	0.2	0	0	0	0.0	0.0	0.1	0	0	0
Jordan	Na'oor	0.1	0.1	0.2	2	1	3	0.0	0.0	0.1	1	1	1
Jordan	Qasr	0.1	0.1	0.2	1	0	2	0.0	0.0	0.0	0	0	1
Jordan	Quaira	0.1	0.1	0.2	0	0	0	0.0	0.0	0.0	0	0	0
Jordan	Ramtha	0.1	0.1	0.2	0	0	1	0.0	0.0	0.0	0	0	0
Jordan	Sabha	0.1	0.1	0.2	0	0	1	0.0	0.0	0.0	0	0	0
Jordan	Sahab	0.1	0.1	0.2	2	1	4	0.0	0.0	0.0	1	1	2
Jordan	Salt	0.1	0.1	0.2	4	2	7	0.0	0.0	0.0	2	1	3
Jordan	Sama Serhan	0.1	0.0	0.1	0	0	1	0.0	0.0	0.0	0	0	0
Jordan	Shoabak	0.1	0.0	0.2	0	0	0	0.0	0.0	0.0	0	0	0
Jordan	Shooneh Janoobiyyeh	0.1	0.1	0.2	1	1	2	0.0	0.0	0.0	1	0	1
Jordan	Tafileh	0.1	0.1	0.2	1	0	1	0.0	0.0	0.0	0	0	1
Jordan	Tayybeh	0.1	0.1	0.2	5	3	9	0.0	0.0	0.0	2	2	4
Jordan	Um El-Basatien	0.1	0.1	0.2	1	0	2	0.0	0.0	0.0	0	0	1
Jordan	Wadi Arabah	0.1	0.1	0.2	0	0	0	0.0	0.0	0.0	0	0	0
Jordan	Wadi Essier	0.1	0.1	0.2	8	4	14	0.0	0.0	0.0	4	3	6
Jordan	Wadi Musa	0.1	0.0	0.2	0	0	1	0.0	0.0	0.1	0	0	0
Jordan	Wastiyyeh	0.1	0.0	0.2	4	2	6	0.0	0.0	0.0	2	1	3
Jordan	Zarqa	0.1	0.1	0.2	8	4	14	0.0	0.0	0.0	4	2	6
Morocco	Agadir-Ida ou Tanane	1.0	0.7	1.5	61	41	89	0.2	0.1	0.3	12	8	19
Morocco	Al Haouz	1.1	0.7	1.5	67	45	94	0.2	0.1	0.3	12	9	17
Morocco	Al Hoceïma	1.1	0.8	1.6	54	36	77	0.2	0.2	0.4	9	6	13
Morocco	Assa-Zag	0.9	0.6	1.3	6	4	9	0.2	0.1	0.3	0	0	1
Morocco	Azilal	1.2	0.8	1.7	92	64	128	0.3	0.2	0.4	17	12	24
Morocco	Ben Slimane	1.3	0.9	1.9	47	33	69	0.3	0.2	0.4	10	6	14
Morocco	Béni Mellal	1.1	0.8	1.7	125	85	190	0.3	0.2	0.4	24	17	35
Morocco	Berkane Taourirt	1.0	0.7	1.5	52	35	74	0.2	0.1	0.3	8	6	12
Morocco	Boulemane	1.1	0.8	1.5	26	18	35	0.2	0.2	0.3	5	3	6
Morocco	Casablanca	0.9	0.6	1.4	355	234	526	0.2	0.1	0.3	69	45	102
Morocco	Chefchaouen	1.2	0.8	1.8	81	53	119	0.3	0.2	0.4	15	10	22
Morocco	Chichaoua	1.1	0.7	1.6	51	34	74	0.2	0.2	0.3	9	6	12
Morocco	Chtouka-Aït Baha	1.0	0.7	1.5	36	24	52	0.2	0.1	0.3	8	5	11
Morocco	El Hajeb	1.1	0.7	1.6	38	24	55	0.2	0.2	0.4	8	5	11
Morocco	El Jadida	1.0	0.7	1.5	141	93	201	0.2	0.1	0.3	25	17	35
Morocco	El Kelaâ des Sraghna	1.1	0.8	1.5	117	84	158	0.2	0.2	0.3	20	14	29
Morocco	Errachidia	1.0	0.7	1.3	74	53	96	0.2	0.1	0.3	12	9	16
Morocco	Essaouira	1.0	0.6	1.4	57	37	82	0.2	0.1	0.3	8	6	12
Morocco	Fahs Anjra	1.0	0.7	1.6	45	30	69	0.2	0.1	0.3	12	7	18
Morocco	Fès	1.1	0.7	1.6	59	38	87	0.2	0.1	0.4	11	7	18
Morocco	Figuig	1.1	0.8	1.4	21	16	27	0.2	0.2	0.3	3	2	4
Morocco	Guelmim	0.9	0.6	1.3	17	12	24	0.2	0.1	0.3	3	2	4
Morocco	Ifrane	1.3	0.8	2.0	22	14	32	0.3	0.2	0.5	4	3	6
Morocco	Inezgane-Aït Melloul	1.0	0.6	1.5	36	23	55	0.2	0.1	0.3	8	5	13
Morocco	Jerada	1.2	0.8	1.6	21	14	29	0.2	0.1	0.3	3	2	4
Morocco	Kénitra	1.1	0.8	1.5	158	109	219	0.3	0.2	0.3	34	24	47
Morocco	Khémisset	1.0	0.6	1.4	59	38	87	0.2	0.2	0.3	10	7	14
Morocco	Khénifra	1.2	0.8	1.7	66	46	95	0.3	0.2	0.4	12	8	17
Morocco	Khouribga	1.4	0.9	2.1	88	58	125	0.3	0.2	0.4	15	10	22

Table 3: Diarrhoea Mortality by Unit *(continued)*

Country	Unit	2000 mortality mean rate (per 10,000)	lower rate (per 10,000)	upper rate (per 10,000)	mean count	lower count	upper count	2017 mortality mean rate (per 10,000)	lower rate (per 10,000)	upper rate (per 10,000)	mean count	lower count	upper count
Morocco	Laâyoune	0.7	0.5	1.0	1	0	1	0.2	0.1	0.2	0	0	0
Morocco	Larache	1.1	0.7	1.7	64	40	98	0.2	0.2	0.4	11	7	16
Morocco	Marrakech	0.9	0.6	1.4	102	69	155	0.2	0.1	0.2	20	12	27
Morocco	Meknès	1.1	0.7	1.7	77	49	121	0.2	0.1	0.3	15	9	22
Morocco	Mohammedia	0.9	0.6	1.4	14	9	22	0.2	0.1	0.3	3	2	5
Morocco	Nador	1.0	0.6	1.4	82	53	116	0.2	0.1	0.3	13	9	18
Morocco	Ouarzazate	1.1	0.8	1.4	103	74	141	0.2	0.2	0.3	17	13	23
Morocco	Oujda Angad	1.0	0.7	1.6	51	33	80	0.2	0.1	0.3	9	5	13
Morocco	Rabat	0.9	0.6	1.3	60	38	89	0.2	0.1	0.3	10	6	15
Morocco	Safi	1.1	0.7	1.5	114	80	159	0.2	0.1	0.3	18	12	27
Morocco	Salé	1.0	0.6	1.4	67	43	98	0.2	0.1	0.4	16	10	23
Morocco	Sefrou	1.2	0.8	1.7	37	25	55	0.3	0.2	0.4	7	4	10
Morocco	Settat	1.4	1.0	2.0	174	123	243	0.3	0.2	0.4	33	24	44
Morocco	Sidi Kacem	1.1	0.8	1.5	86	58	119	0.2	0.2	0.4	15	10	21
Morocco	Skhirate-Témara	0.9	0.6	1.4	48	31	71	0.2	0.1	0.3	13	8	19
Morocco	Tan-Tan	0.8	0.5	1.2	5	3	8	0.2	0.1	0.3	1	1	2
Morocco	Tanger-Assilah	1.0	0.7	1.6	46	30	71	0.2	0.1	0.3	12	7	18
Morocco	Taounate	1.2	0.8	1.7	98	66	137	0.3	0.2	0.4	16	11	22
Morocco	Taroudannt	1.1	0.8	1.5	106	76	144	0.2	0.1	0.3	16	11	21
Morocco	Tata	1.0	0.7	1.3	14	10	19	0.2	0.1	0.2	2	1	2
Morocco	Taza	1.2	0.8	1.7	103	72	148	0.2	0.2	0.3	16	11	21
Morocco	Tétouan	1.0	0.7	1.5	73	47	106	0.2	0.2	0.4	17	11	25
Morocco	Tiznit	1.0	0.7	1.4	38	27	54	0.2	0.1	0.3	5	3	6
Morocco	Zagora	1.0	0.7	1.4	10	7	15	0.2	0.1	0.2	2	1	2
Morocco	Zouagha-Moulay Yacoub	1.1	0.7	1.6	80	52	115	0.2	0.2	0.4	16	11	26
Sudan	Abu Hamad	3.3	2.4	4.3	62	46	81	1.0	0.6	1.4	22	15	33
Sudan	Abu Jubaiyah	2.8	2.1	3.6	115	86	147	1.0	0.7	1.5	36	23	52
Sudan	Abyei	3.2	2.4	4.2	183	137	245	1.1	0.7	1.4	51	36	70
Sudan	Ad Damazin	4.7	3.7	5.7	246	195	303	1.1	0.7	1.5	86	60	123
Sudan	Ad Damer	3.6	2.6	4.7	68	50	89	1.0	0.7	1.5	21	15	32
Sudan	Ad Dinder	4.1	3.1	5.1	327	248	412	1.1	0.8	1.6	99	69	142
Sudan	Ad Douiem	3.5	2.7	4.4	241	183	302	1.3	0.9	1.8	108	73	154
Sudan	Addabah	3.2	2.4	4.3	60	45	82	1.1	0.7	1.6	24	16	35
Sudan	Al Deain	3.6	2.8	4.7	485	370	629	1.2	0.8	1.6	280	193	378
Sudan	Al Fasher	3.2	2.4	4.1	244	187	316	1.2	0.8	1.7	146	96	214
Sudan	Al Faw	4.2	3.3	5.2	113	88	139	1.3	0.9	1.8	35	24	48
Sudan	Al Fushqa	4.2	3.2	5.4	181	138	230	1.3	0.9	1.8	64	43	90
Sudan	Al Gadaref	4.1	3.0	5.1	261	190	323	1.2	0.8	1.8	78	52	114
Sudan	Al Galabat	4.4	3.3	5.5	281	211	352	1.2	0.8	1.7	75	51	109
Sudan	Al Gash	3.3	2.6	4.3	136	105	175	1.2	0.8	1.6	55	36	77
Sudan	Al Geneina	3.7	2.8	4.7	613	464	773	1.1	0.8	1.6	152	105	215
Sudan	Al Gutaina	3.6	2.8	4.6	234	182	303	1.4	1.0	1.9	117	82	166
Sudan	Al Jabalian	3.7	2.9	4.7	243	189	314	1.2	0.8	1.7	99	66	143
Sudan	Al Kamlin	3.5	2.7	4.5	159	122	205	1.3	0.9	1.9	65	46	94
Sudan	Al Kurumik	4.8	3.8	6.1	67	53	85	1.0	0.7	1.4	21	14	29
Sudan	Al Mahagil	3.3	2.5	4.2	244	185	315	1.2	0.8	1.8	109	71	155
Sudan	Al Matammah	3.6	2.7	4.5	101	77	129	1.1	0.8	1.6	40	27	57
Sudan	Al Rahd	4.2	3.1	5.2	400	301	499	1.2	0.8	1.8	113	77	164
Sudan	Al Roseires	5.2	4.0	6.5	207	160	257	1.1	0.7	1.5	59	40	84
Sudan	As Salam	3.0	2.2	3.9	82	61	109	1.0	0.6	1.5	29	18	42
Sudan	Atbara	3.5	2.6	4.6	125	93	163	1.0	0.7	1.6	45	29	69
Sudan	Bara	3.2	2.4	4.2	276	208	362	1.1	0.7	1.6	120	78	172
Sudan	Baw	5.0	4.0	6.3	112	90	143	1.1	0.7	1.5	34	23	49
Sudan	Berber	3.5	2.5	4.7	50	35	66	1.0	0.6	1.5	18	12	29
Sudan	Buram	3.9	3.0	4.8	267	208	335	1.3	0.9	1.7	150	103	207
Sudan	Dilling	2.5	2.0	3.3	149	114	193	1.0	0.6	1.3	48	32	67
Sudan	Dongola	3.0	2.2	4.0	109	80	145	1.1	0.7	1.5	44	29	64
Sudan	East al Gazera	3.4	2.6	4.3	332	250	420	1.2	0.8	1.6	125	84	176
Sudan	En Nuhud	3.0	2.2	3.9	302	225	393	1.0	0.7	1.5	133	85	194

Country	Unit	2000 mortality mean rate (per 10,000)	lower rate (per 10,000)	upper rate (per 10,000)	mean count	lower count	upper count	2017 mortality mean rate (per 10,000)	lower rate (per 10,000)	upper rate (per 10,000)	mean count	lower count	upper count
Sudan	Geissan	5.4	4.3	6.7	113	90	139	1.0	0.7	1.5	28	19	41
Sudan	Ghebeish	3.1	2.3	4.0	210	155	274	1.1	0.7	1.5	97	65	138
Sudan	Halayeb	3.0	2.0	4.0	13	9	17	0.9	0.6	1.4	7	5	11
Sudan	Hamashkorieb	3.1	2.4	4.1	87	66	115	1.1	0.7	1.6	41	26	58
Sudan	Id El Ghanem	3.9	2.9	4.9	548	410	690	1.3	0.9	1.9	307	214	447
Sudan	Jebrat al Sheikh	3.7	2.9	4.8	116	91	150	1.3	0.9	1.9	54	38	76
Sudan	Kabkabiya	3.4	2.5	4.3	357	267	460	1.1	0.8	1.6	200	136	285
Sudan	Kadugli	2.5	1.9	3.3	60	45	77	1.0	0.6	1.4	19	12	28
Sudan	Karary	3.6	2.8	4.6	159	124	203	1.3	0.9	1.9	78	53	114
Sudan	Kas	3.7	2.7	4.7	45	32	58	1.2	0.8	1.8	27	18	39
Sudan	Kassala	3.4	2.6	4.4	233	180	304	1.2	0.8	1.7	103	69	150
Sudan	Khartoum	3.7	2.8	4.8	366	283	474	1.4	0.9	2.0	170	115	246
Sudan	Khartoum Bahri	3.7	2.9	4.7	518	403	667	1.3	0.9	2.0	235	156	342
Sudan	Kosti	3.5	2.7	4.5	387	298	501	1.2	0.8	1.7	159	107	220
Sudan	Kutum	3.2	2.4	4.2	55	41	72	1.1	0.7	1.6	30	20	44
Sudan	Lagawa	2.8	2.1	3.6	114	85	147	1.0	0.6	1.4	31	20	42
Sudan	Mellit	3.1	2.3	4.1	163	120	213	1.1	0.8	1.7	96	67	141
Sudan	Merawi	3.3	2.5	4.4	83	62	109	1.1	0.7	1.6	31	21	47
Sudan	Mukjar	3.8	2.9	4.8	375	284	479	1.2	0.9	1.8	116	81	168
Sudan	Nahr Atbara	3.4	2.6	4.3	179	138	224	1.2	0.8	1.7	74	49	107
Sudan	North al Gazera	3.3	2.5	4.3	262	200	338	1.2	0.9	1.8	105	73	151
Sudan	Nyala	3.7	2.8	4.7	169	129	215	1.3	0.8	1.9	101	67	148
Sudan	Nyala	3.8	2.9	4.9	332	249	426	1.3	0.9	1.8	184	126	261
Sudan	Omdurman	3.7	2.9	4.7	595	460	759	1.4	0.9	2.0	282	190	408
Sudan	Port Sudan	2.6	1.9	3.7	161	119	227	0.9	0.6	1.3	96	61	143
Sudan	Rashad	2.9	2.2	3.8	165	126	218	1.1	0.7	1.5	52	35	75
Sudan	Sennar	3.9	3.0	4.9	389	300	486	1.2	0.8	1.7	141	95	199
Sudan	Seteet	3.4	2.6	4.3	72	55	90	1.2	0.8	1.7	28	19	40
Sudan	Sharg En Nile	3.7	2.9	4.8	290	225	375	1.4	0.9	2.0	143	96	206
Sudan	Sharq al Gazera	3.4	2.6	4.4	298	224	381	1.2	0.8	1.8	119	80	175
Sudan	Sheikan	3.4	2.5	4.3	195	144	249	1.1	0.7	1.6	85	57	123
Sudan	Shendi	3.5	2.7	4.4	146	112	182	1.1	0.7	1.7	58	38	85
Sudan	Singa	4.0	3.0	5.1	267	201	338	1.1	0.8	1.6	84	57	119
Sudan	Sinkat	2.8	2.1	3.8	114	86	154	0.9	0.6	1.3	68	45	94
Sudan	South al Gazera	3.3	2.5	4.3	224	170	293	1.2	0.8	1.8	87	56	127
Sudan	South Khartoum	3.7	2.9	4.8	432	338	566	1.4	1.0	2.1	207	141	303
Sudan	Sowdari	3.3	2.4	4.2	176	130	227	1.1	0.8	1.6	78	52	110
Sudan	Talodi	2.6	1.9	3.5	57	42	76	1.0	0.6	1.4	18	12	27
Sudan	Tokar	2.7	2.0	3.6	84	63	112	0.9	0.6	1.3	54	34	75
Sudan	Tulus	3.8	2.8	4.8	192	141	244	1.3	0.9	1.9	109	73	161
Sudan	Um Al Gura	3.4	2.6	4.4	226	173	289	1.2	0.8	1.7	85	58	120
Sudan	Um Badda	3.7	2.9	4.8	235	185	306	1.4	1.0	2.0	116	78	168
Sudan	Um Kadada	3.2	2.5	4.2	102	78	134	1.2	0.8	1.6	60	40	85
Sudan	Um Rawaba	3.4	2.6	4.5	419	319	555	1.2	0.8	1.6	185	121	249
Sudan	Wadi Halfa	3.0	2.2	4.0	33	24	44	1.0	0.7	1.4	13	9	18
Sudan	Zallingi	3.6	2.8	4.6	172	132	222	1.2	0.8	1.7	53	36	75
Syria	'Ayn al-'Arab	0.1	0.0	0.1	2	1	3	0.0	0.0	0.0	0	0	0
Syria	A'zaz	0.1	0.0	0.1	3	1	5	0.0	0.0	0.0	0	0	0
Syria	Abu Kamal	0.1	0.0	0.1	3	1	4	0.0	0.0	0.0	0	0	0
Syria	Afrin	0.1	0.0	0.1	2	1	3	0.0	0.0	0.0	0	0	0
Syria	Al Bab	0.1	0.0	0.1	3	1	5	0.0	0.0	0.0	0	0	0
Syria	Al Qamishli	0.1	0.0	0.1	4	2	7	0.0	0.0	0.0	0	0	1
Syria	Al-Haffah	0.1	0.0	0.1	1	0	1	0.0	0.0	0.0	0	0	0
Syria	Al-Hasakah	0.1	0.0	0.1	4	2	7	0.0	0.0	0.0	0	0	1
Syria	Al-Malikiyah	0.1	0.0	0.1	2	1	3	0.0	0.0	0.0	0	0	0
Syria	Al-Mukharram	0.1	0.0	0.1	0	0	1	0.0	0.0	0.0	0	0	0
Syria	Al-Qusayr	0.1	0.0	0.1	1	1	2	0.0	0.0	0.0	0	0	0
Syria	Al-Qutayfah	0.1	0.0	0.1	1	1	2	0.0	0.0	0.0	0	0	0
Syria	An-Nabk	0.1	0.0	0.1	1	0	2	0.0	0.0	0.0	0	0	0
Syria	Ar-Raqqah	0.1	0.0	0.1	6	3	11	0.0	0.0	0.0	1	0	1

Table 3: Diarrhoea Mortality by Unit *(continued)*

Country	Unit	2000 mortality mean rate (per 10,000)	lower rate (per 10,000)	upper rate (per 10,000)	mean count	lower count	upper count	2017 mortality mean rate (per 10,000)	lower rate (per 10,000)	upper rate (per 10,000)	mean count	lower count	upper count
Syria	Ar-Rastan	0.1	0.0	0.1	3	2	5	0.0	0.0	0.0	0	0	1
Syria	Arihah	0.1	0.0	0.1	2	1	3	0.0	0.0	0.0	0	0	0
Syria	As-Safirah	0.1	0.0	0.1	2	1	3	0.0	0.0	0.0	0	0	0
Syria	As-Sanamayn	0.1	0.0	0.1	1	1	2	0.0	0.0	0.0	0	0	0
Syria	As-Suqaylabiyah	0.1	0.0	0.1	2	1	4	0.0	0.0	0.0	0	0	0
Syria	As-Suwayda	0.1	0.0	0.1	2	1	3	0.0	0.0	0.0	0	0	0
Syria	Ash-Shaykh Badr	0.1	0.0	0.1	0	0	1	0.0	0.0	0.0	0	0	0
Syria	At-Tall	0.1	0.0	0.1	1	0	1	0.0	0.0	0.0	0	0	0
Syria	Ath-Thawrah	0.1	0.0	0.1	1	0	1	0.0	0.0	0.0	0	0	0
Syria	Baniyas	0.1	0.0	0.1	1	1	2	0.0	0.0	0.0	0	0	0
Syria	Damascus	0.1	0.0	0.1	6	3	11	0.0	0.0	0.0	1	0	1
Syria	Daraa	0.1	0.0	0.1	4	2	7	0.0	0.0	0.0	0	0	1
Syria	Darayya	0.1	0.0	0.1	1	1	3	0.0	0.0	0.0	0	0	0
Syria	Deir ez-Zor	0.1	0.0	0.1	5	3	9	0.0	0.0	0.0	1	0	1
Syria	Duma	0.1	0.0	0.1	3	2	5	0.0	0.0	0.0	0	0	1
Syria	Duraykish	0.1	0.0	0.1	1	0	1	0.0	0.0	0.0	0	0	0
Syria	Hama	0.1	0.0	0.1	6	3	12	0.0	0.0	0.0	1	0	1
Syria	Harem	0.1	0.0	0.1	2	1	3	0.0	0.0	0.0	0	0	0
Syria	Hims	0.1	0.0	0.1	8	4	15	0.0	0.0	0.0	1	0	1
Syria	Idlib	0.1	0.0	0.1	4	2	6	0.0	0.0	0.0	0	0	1
Syria	Izra'	0.1	0.0	0.1	3	1	5	0.0	0.0	0.0	0	0	0
Syria	Jabal Sam'an	0.1	0.0	0.1	23	12	42	0.0	0.0	0.0	2	1	4
Syria	Jableh	0.1	0.0	0.1	2	1	3	0.0	0.0	0.0	0	0	0
Syria	Jarabulus	0.1	0.0	0.1	1	0	1	0.0	0.0	0.0	0	0	0
Syria	Jisr ash-Shugur	0.1	0.0	0.1	2	1	3	0.0	0.0	0.0	0	0	0
Syria	Latakia	0.1	0.0	0.1	4	2	7	0.0	0.0	0.0	0	0	1
Syria	Ma'arrat al-Numan	0.1	0.0	0.1	4	2	6	0.0	0.0	0.0	0	0	1
Syria	Manbij	0.1	0.0	0.1	4	2	6	0.0	0.0	0.0	0	0	1
Syria	Markaz Rif Dimashq	0.1	0.0	0.1	21	10	39	0.0	0.0	0.0	2	1	4
Syria	Masyaf	0.1	0.0	0.1	1	1	3	0.0	0.0	0.0	0	0	0
Syria	Mayadin	0.1	0.0	0.1	2	1	3	0.0	0.0	0.0	0	0	0
Syria	Muhardeh	0.1	0.0	0.1	1	1	2	0.0	0.0	0.0	0	0	0
Syria	Palmyra	0.1	0.0	0.1	1	0	1	0.0	0.0	0.0	0	0	0
Syria	Qardaha	0.1	0.0	0.1	1	1	2	0.0	0.0	0.0	0	0	0
Syria	Qatana	0.1	0.0	0.1	2	1	3	0.0	0.0	0.0	0	0	0
Syria	Quneitra	0.1	0.0	0.1	1	0	1	0.0	0.0	0.0	0	0	0
Syria	Ra's al-'Ayn	0.1	0.0	0.1	2	1	4	0.0	0.0	0.0	0	0	0
Syria	Safita	0.1	0.0	0.1	1	1	2	0.0	0.0	0.0	0	0	0
Syria	Salamiyah	0.1	0.0	0.1	2	1	3	0.0	0.0	0.0	0	0	0
Syria	Salkhad	0.1	0.0	0.1	0	0	1	0.0	0.0	0.0	0	0	0
Syria	Shahba	0.1	0.0	0.1	0	0	1	0.0	0.0	0.0	0	0	0
Syria	Tal Abyad	0.1	0.0	0.1	1	1	2	0.0	0.0	0.0	0	0	0
Syria	Talkalakh	0.1	0.0	0.1	1	1	2	0.0	0.0	0.0	0	0	0
Syria	Tartus	0.1	0.0	0.1	2	1	4	0.0	0.0	0.0	0	0	0
Syria	Yabrud	0.1	0.0	0.1	0	0	0	0.0	0.0	0.0	0	0	0
Syria	Zabadani	0.1	0.0	0.1	1	0	2	0.0	0.0	0.0	0	0	0
Tunisia	Agareb	0.1	0.0	0.1	0	0	0	0.0	0.0	0.0	0	0	0
Tunisia	Aïn Draham	0.1	0.1	0.1	0	0	0	0.0	0.0	0.0	0	0	0
Tunisia	Akouda	0.1	0.1	0.1	0	0	0	0.0	0.0	0.0	0	0	0
Tunisia	Alaa	0.1	0.1	0.2	1	1	1	0.0	0.0	0.0	0	0	0
Tunisia	Amdoun	0.1	0.1	0.1	0	0	0	0.0	0.0	0.0	0	0	0
Tunisia	Ariana Médina	0.1	0.1	0.1	0	0	0	0.0	0.0	0.0	0	0	0
Tunisia	Ayoun	0.1	0.0	0.1	0	0	0	0.0	0.0	0.0	0	0	0
Tunisia	Bab Bhar	0.1	0.1	0.1	0	0	0	0.0	0.0	0.0	0	0	0
Tunisia	Bab Souika	0.1	0.1	0.1	0	0	0	0.0	0.0	0.0	0	0	0
Tunisia	Balta Bou Aouane	0.1	0.1	0.1	0	0	0	0.0	0.0	0.0	0	0	0
Tunisia	Bardo	0.1	0.1	0.1	1	0	1	0.0	0.0	0.0	0	0	0

Table 3: Diarrhoea Mortality by Unit *(continued)*

Country	Unit	2000 mortality mean rate (per 10,000)	2000 lower rate (per 10,000)	2000 upper rate (per 10,000)	2000 mean count	2000 lower count	2000 upper count	2017 mortality mean rate (per 10,000)	2017 lower rate (per 10,000)	2017 upper rate (per 10,000)	2017 mean count	2017 lower count	2017 upper count
Tunisia	Bargou	0.1	0.1	0.1	0	0	0	0.0	0.0	0.0	0	0	0
Tunisia	Béja Nord	0.1	0.1	0.1	0	0	0	0.0	0.0	0.0	0	0	0
Tunisia	Béja Sud	0.1	0.1	0.1	0	0	0	0.0	0.0	0.0	0	0	0
Tunisia	Bekalta	0.1	0.1	0.1	0	0	0	0.0	0.0	0.0	0	0	0
Tunisia	Belkhir	0.0	0.0	0.0	0	0	0	0.0	0.0	0.0	0	0	0
Tunisia	Bembla	0.1	0.1	0.1	0	0	0	0.0	0.0	0.0	0	0	0
Tunisia	Ben Arous	0.1	0.1	0.1	0	0	0	0.0	0.0	0.0	0	0	0
Tunisia	Ben Guerdane	0.0	0.0	0.0	0	0	0	0.0	0.0	0.0	0	0	0
Tunisia	Beni Hassen	0.1	0.1	0.1	0	0	0	0.0	0.0	0.0	0	0	0
Tunisia	Beni Khalled	0.0	0.0	0.1	0	0	0	0.0	0.0	0.0	0	0	0
Tunisia	Beni Khedache	0.0	0.0	0.0	0	0	0	0.0	0.0	0.0	0	0	0
Tunisia	Beni Khiar	0.0	0.0	0.1	0	0	0	0.0	0.0	0.0	0	0	0
Tunisia	Bir Ali Ben Khélifa	0.1	0.0	0.1	0	0	0	0.0	0.0	0.0	0	0	0
Tunisia	Bir El Hfay	0.0	0.0	0.1	0	0	0	0.0	0.0	0.0	0	0	0
Tunisia	Bir Lahmar	0.0	0.0	0.0	0	0	0	0.0	0.0	0.0	0	0	0
Tunisia	Bir Mchergua	0.1	0.1	0.1	0	0	0	0.0	0.0	0.0	0	0	0
Tunisia	Bizerte Nord	0.1	0.0	0.1	0	0	0	0.0	0.0	0.0	0	0	0
Tunisia	Bizerte Sud	0.1	0.0	0.1	0	0	0	0.0	0.0	0.0	0	0	0
Tunisia	Borj El Amri	0.1	0.0	0.1	0	0	0	0.0	0.0	0.0	0	0	0
Tunisia	Bou Argoub	0.0	0.0	0.1	0	0	0	0.0	0.0	0.0	0	0	0
Tunisia	Bouarada	0.1	0.1	0.1	0	0	0	0.0	0.0	0.0	0	0	0
Tunisia	Bouficha	0.1	0.1	0.1	0	0	0	0.0	0.0	0.0	0	0	0
Tunisia	Bouhaira	0.1	0.0	0.1	0	0	0	0.0	0.0	0.0	0	0	0
Tunisia	Bouhajla	0.1	0.1	0.1	1	1	1	0.0	0.0	0.0	0	0	0
Tunisia	Boumerdès	0.1	0.1	0.1	0	0	0	0.0	0.0	0.0	0	0	0
Tunisia	Boumhel	0.1	0.1	0.1	0	0	0	0.0	0.0	0.0	0	0	0
Tunisia	Bourouis	0.1	0.1	0.1	0	0	0	0.0	0.0	0.0	0	0	0
Tunisia	Bousalem	0.1	0.1	0.1	0	0	0	0.0	0.0	0.0	0	0	0
Tunisia	Carthage	0.1	0.0	0.1	0	0	0	0.0	0.0	0.0	0	0	0
Tunisia	Chebba	0.1	0.0	0.1	0	0	0	0.0	0.0	0.0	0	0	0
Tunisia	Chebika	0.1	0.1	0.2	1	0	1	0.0	0.0	0.0	0	0	0
Tunisia	Chorbane	0.1	0.1	0.1	0	0	0	0.0	0.0	0.0	0	0	0
Tunisia	Chrarda	0.1	0.1	0.1	0	0	0	0.0	0.0	0.0	0	0	0
Tunisia	Cité El Khadra	0.1	0.1	0.1	1	0	1	0.0	0.0	0.0	0	0	0
Tunisia	Dahmani	0.1	0.1	0.1	0	0	0	0.0	0.0	0.0	0	0	0
Tunisia	Dar Chaabane El Fehri	0.0	0.0	0.1	0	0	0	0.0	0.0	0.0	0	0	0
Tunisia	Degueche	0.0	0.0	0.1	0	0	0	0.0	0.0	0.0	0	0	0
Tunisia	Dhiba	0.0	0.0	0.0	0	0	0	0.0	0.0	0.0	0	0	0
Tunisia	Djerba Ajim	0.0	0.0	0.1	0	0	0	0.0	0.0	0.0	0	0	0
Tunisia	Djerba Midoun	0.1	0.0	0.1	0	0	0	0.0	0.0	0.0	0	0	0
Tunisia	Douar Hicher	0.1	0.1	0.1	0	0	0	0.0	0.0	0.0	0	0	0
Tunisia	Douz	0.0	0.0	0.1	0	0	0	0.0	0.0	0.0	0	0	0
Tunisia	El Alia	0.1	0.0	0.1	0	0	0	0.0	0.0	0.0	0	0	0
Tunisia	El Amra	0.0	0.0	0.1	0	0	0	0.0	0.0	0.0	0	0	0
Tunisia	El Battan	0.1	0.0	0.1	0	0	0	0.0	0.0	0.0	0	0	0
Tunisia	El Ghraiba	0.0	0.0	0.1	0	0	0	0.0	0.0	0.0	0	0	0
Tunisia	El Jem	0.1	0.1	0.1	0	0	0	0.0	0.0	0.0	0	0	0
Tunisia	El Krib	0.1	0.1	0.1	0	0	0	0.0	0.0	0.0	0	0	0
Tunisia	El Menzah	0.1	0.1	0.1	0	0	0	0.0	0.0	0.0	0	0	0
Tunisia	El Mida	0.0	0.0	0.1	0	0	0	0.0	0.0	0.0	0	0	0
Tunisia	El Mourouj	0.1	0.1	0.1	0	0	0	0.0	0.0	0.0	0	0	0
Tunisia	El Ouardia	0.1	0.1	0.1	0	0	1	0.0	0.0	0.0	0	0	0
Tunisia	El Tahrir	0.1	0.0	0.1	0	0	0	0.0	0.0	0.0	0	0	0
Tunisia	Enfidha	0.1	0.1	0.1	0	0	0	0.0	0.0	0.0	0	0	0
Tunisia	Es Sers	0.1	0.1	0.1	0	0	0	0.0	0.0	0.0	0	0	0
Tunisia	Ettadhamen	0.1	0.1	0.1	0	0	0	0.0	0.0	0.0	0	0	0
Tunisia	Ezzahra	0.1	0.1	0.1	0	0	0	0.0	0.0	0.0	0	0	0
Tunisia	Ezzouhour	0.1	0.1	0.1	0	0	0	0.0	0.0	0.0	0	0	0

Table 3: Diarrhoea Mortality by Unit *(continued)*

Country	Unit	2000 mortality mean rate (per 10,000)	lower rate (per 10,000)	upper rate (per 10,000)	mean count	lower count	upper count	2017 mortality mean rate (per 10,000)	lower rate (per 10,000)	upper rate (per 10,000)	mean count	lower count	upper count
Tunisia	Ezzouhour	0.0	0.0	0.1	0	0	0	0.0	0.0	0.0	0	0	0
Tunisia	Fahs	0.1	0.1	0.1	0	0	1	0.0	0.0	0.0	0	0	0
Tunisia	Faouar	0.0	0.0	0.1	0	0	0	0.0	0.0	0.0	0	0	0
Tunisia	Feriana	0.0	0.0	0.1	0	0	0	0.0	0.0	0.0	0	0	0
Tunisia	Fernana	0.1	0.1	0.1	0	0	0	0.0	0.0	0.0	0	0	0
Tunisia	Fouchana	0.1	0.1	0.1	1	1	1	0.0	0.0	0.0	0	0	0
Tunisia	Foussana	0.0	0.0	0.1	0	0	0	0.0	0.0	0.0	0	0	0
Tunisia	Gaafour	0.1	0.1	0.1	0	0	0	0.0	0.0	0.0	0	0	0
Tunisia	Gabès Médina	0.1	0.0	0.1	0	0	0	0.0	0.0	0.0	0	0	0
Tunisia	Gabès Ouest	0.1	0.0	0.1	0	0	0	0.0	0.0	0.0	0	0	0
Tunisia	Gabès Sud	0.1	0.0	0.1	0	0	0	0.0	0.0	0.0	0	0	0
Tunisia	Gafsa Nord	0.0	0.0	0.0	0	0	0	0.0	0.0	0.0	0	0	0
Tunisia	Gafsa Sud	0.0	0.0	0.0	0	0	0	0.0	0.0	0.0	0	0	0
Tunisia	Ghannouch	0.1	0.0	0.1	0	0	0	0.0	0.0	0.0	0	0	0
Tunisia	Ghar El Melh	0.1	0.0	0.1	0	0	0	0.0	0.0	0.0	0	0	0
Tunisia	Ghardimaou	0.1	0.1	0.1	0	0	1	0.0	0.0	0.0	0	0	0
Tunisia	Ghazala	0.1	0.0	0.1	0	0	0	0.0	0.0	0.0	0	0	0
Tunisia	Ghomrassen	0.0	0.0	0.0	0	0	0	0.0	0.0	0.0	0	0	0
Tunisia	Goubellat	0.1	0.1	0.1	0	0	0	0.0	0.0	0.0	0	0	0
Tunisia	Grombalia	0.1	0.0	0.1	0	0	0	0.0	0.0	0.0	0	0	0
Tunisia	Guetar	0.0	0.0	0.0	0	0	0	0.0	0.0	0.0	0	0	0
Tunisia	Haffouz	0.1	0.1	0.2	0	0	0	0.0	0.0	0.0	0	0	0
Tunisia	Hajeb El Ayoun	0.1	0.1	0.1	0	0	1	0.0	0.0	0.0	0	0	0
Tunisia	Hamma	0.0	0.0	0.1	0	0	0	0.0	0.0	0.0	0	0	0
Tunisia	Hammam Chott	0.1	0.1	0.1	0	0	0	0.0	0.0	0.0	0	0	0
Tunisia	Hammam Ghezaz	0.0	0.0	0.1	0	0	0	0.0	0.0	0.0	0	0	0
Tunisia	Hammam Lif	0.1	0.1	0.1	0	0	0	0.0	0.0	0.0	0	0	0
Tunisia	Hammam Sousse	0.1	0.1	0.1	0	0	0	0.0	0.0	0.0	0	0	0
Tunisia	Hammamet	0.1	0.0	0.1	1	0	1	0.0	0.0	0.0	0	0	0
Tunisia	Haouaria	0.0	0.0	0.1	0	0	0	0.0	0.0	0.0	0	0	0
Tunisia	Hassi El Ferid	0.0	0.0	0.0	0	0	0	0.0	0.0	0.0	0	0	0
Tunisia	Hazoua	0.1	0.0	0.1	0	0	0	0.0	0.0	0.0	0	0	0
Tunisia	Hbira	0.1	0.1	0.1	0	0	0	0.0	0.0	0.0	0	0	0
Tunisia	Hencha	0.1	0.0	0.1	0	0	0	0.0	0.0	0.0	0	0	0
Tunisia	Hergla	0.1	0.1	0.1	0	0	0	0.0	0.0	0.0	0	0	0
Tunisia	Hidra	0.1	0.0	0.1	0	0	0	0.0	0.0	0.0	0	0	0
Tunisia	Houmt Souk	0.0	0.0	0.1	0	0	0	0.0	0.0	0.0	0	0	0
Tunisia	Hrairia	0.1	0.1	0.1	0	0	1	0.0	0.0	0.0	0	0	0
Tunisia	Jammel	0.1	0.1	0.1	0	0	0	0.0	0.0	0.0	0	0	0
Tunisia	Jebel Jelloud	0.1	0.1	0.1	0	0	0	0.0	0.0	0.0	0	0	0
Tunisia	Jebeniana	0.0	0.0	0.1	0	0	0	0.0	0.0	0.0	0	0	0
Tunisia	Jedaida	0.1	0.0	0.1	0	0	0	0.0	0.0	0.0	0	0	0
Tunisia	Jedeliane	0.1	0.0	0.1	0	0	0	0.0	0.0	0.0	0	0	0
Tunisia	Jelma	0.1	0.1	0.1	0	0	0	0.0	0.0	0.0	0	0	0
Tunisia	Jendouba Nord	0.1	0.1	0.1	0	0	0	0.0	0.0	0.0	0	0	0
Tunisia	Jendouba Sud	0.1	0.0	0.1	0	0	0	0.0	0.0	0.0	0	0	0
Tunisia	Jerissa	0.1	0.0	0.1	0	0	0	0.0	0.0	0.0	0	0	0
Tunisia	Joumine	0.1	0.0	0.1	0	0	0	0.0	0.0	0.0	0	0	0
Tunisia	Kabaria	0.1	0.1	0.1	0	0	0	0.0	0.0	0.0	0	0	0
Tunisia	Kairouan Nord	0.1	0.1	0.2	1	1	1	0.0	0.0	0.0	0	0	0
Tunisia	Kairouan Sud	0.1	0.1	0.2	1	1	2	0.0	0.0	0.0	0	0	0
Tunisia	Kalaa Kebira	0.1	0.1	0.1	0	0	1	0.0	0.0	0.0	0	0	0
Tunisia	Kalaa Khesba	0.1	0.0	0.1	0	0	0	0.0	0.0	0.0	0	0	0
Tunisia	Kalaa Sghira	0.1	0.1	0.1	0	0	0	0.0	0.0	0.0	0	0	0
Tunisia	Kalaat El Andalous	0.1	0.0	0.1	0	0	0	0.0	0.0	0.0	0	0	0
Tunisia	Kalaat Senan	0.1	0.0	0.1	0	0	0	0.0	0.0	0.0	0	0	0
Tunisia	Kasserine Nord	0.0	0.0	0.1	0	0	0	0.0	0.0	0.0	0	0	0
Tunisia	Kasserine Sud	0.0	0.0	0.1	0	0	0	0.0	0.0	0.0	0	0	0
Tunisia	Kebili Nord	0.0	0.0	0.1	0	0	0	0.0	0.0	0.0	0	0	0

Table 3: Diarrhoea Mortality by Unit *(continued)*

Country	Unit	2000 mortality mean rate (per 10,000)	lower rate (per 10,000)	upper rate (per 10,000)	mean count	lower count	upper count	2017 mortality mean rate (per 10,000)	lower rate (per 10,000)	upper rate (per 10,000)	mean count	lower count	upper count
Tunisia	Kebili Sud	0.0	0.0	0.1	0	0	0	0.0	0.0	0.0	0	0	0
Tunisia	Kef Est	0.1	0.1	0.1	0	0	0	0.0	0.0	0.0	0	0	0
Tunisia	Kef Ouest	0.1	0.1	0.1	0	0	0	0.0	0.0	0.0	0	0	0
Tunisia	Kelibia	0.0	0.0	0.1	0	0	0	0.0	0.0	0.0	0	0	0
Tunisia	Kerkennah	0.0	0.0	0.1	0	0	0	0.0	0.0	0.0	0	0	0
Tunisia	Kesra	0.1	0.1	0.1	0	0	0	0.0	0.0	0.0	0	0	0
Tunisia	Kondar	0.1	0.1	0.1	0	0	0	0.0	0.0	0.0	0	0	0
Tunisia	Korba	0.0	0.0	0.1	0	0	0	0.0	0.0	0.0	0	0	0
Tunisia	Ksar	0.0	0.0	0.0	0	0	0	0.0	0.0	0.0	0	0	0
Tunisia	Ksar Hellal	0.1	0.1	0.1	0	0	0	0.0	0.0	0.0	0	0	0
Tunisia	Ksibet El Mediouni	0.1	0.1	0.1	0	0	0	0.0	0.0	0.0	0	0	0
Tunisia	Ksour	0.1	0.0	0.1	0	0	0	0.0	0.0	0.0	0	0	0
Tunisia	Ksour Essef	0.1	0.0	0.1	0	0	0	0.0	0.0	0.0	0	0	0
Tunisia	La Goulette	0.1	0.0	0.1	0	0	0	0.0	0.0	0.0	0	0	0
Tunisia	La Marsa	0.1	0.0	0.1	0	0	0	0.0	0.0	0.0	0	0	0
Tunisia	Lake Ichkeul	0.1	0.1	0.1	0	0	0	0.0	0.0	0.0	0	0	0
Tunisia	Laroussa	0.1	0.1	0.1	0	0	0	0.0	0.0	0.0	0	0	0
Tunisia	M'Hamdia	0.1	0.1	0.1	0	0	0	0.0	0.0	0.0	0	0	0
Tunisia	M'Saken	0.1	0.1	0.1	1	1	1	0.0	0.0	0.0	0	0	0
Tunisia	Mahdia	0.1	0.1	0.1	0	0	0	0.0	0.0	0.0	0	0	0
Tunisia	Mahres	0.1	0.0	0.1	0	0	0	0.0	0.0	0.0	0	0	0
Tunisia	Majel Belabbes	0.0	0.0	0.0	0	0	0	0.0	0.0	0.0	0	0	0
Tunisia	Makthar	0.1	0.1	0.1	0	0	0	0.0	0.0	0.0	0	0	0
Tunisia	Manouba	0.1	0.1	0.1	0	0	0	0.0	0.0	0.0	0	0	0
Tunisia	Mareth	0.1	0.0	0.1	0	0	0	0.0	0.0	0.0	0	0	0
Tunisia	Mateur	0.1	0.1	0.1	0	0	0	0.0	0.0	0.0	0	0	0
Tunisia	Matmata	0.0	0.0	0.1	0	0	0	0.0	0.0	0.0	0	0	0
Tunisia	Matmata Nouvelle	0.0	0.0	0.1	0	0	0	0.0	0.0	0.0	0	0	0
Tunisia	Mazzouna	0.1	0.0	0.1	0	0	0	0.0	0.0	0.0	0	0	0
Tunisia	Mdhilla	0.0	0.0	0.0	0	0	0	0.0	0.0	0.0	0	0	0
Tunisia	Médenine Nord	0.0	0.0	0.1	0	0	0	0.0	0.0	0.0	0	0	0
Tunisia	Médenine Sud	0.0	0.0	0.1	0	0	0	0.0	0.0	0.0	0	0	0
Tunisia	Médina	0.1	0.1	0.1	0	0	0	0.0	0.0	0.0	0	0	0
Tunisia	Mégrine	0.1	0.1	0.1	0	0	0	0.0	0.0	0.0	0	0	0
Tunisia	Mejez El Bab	0.1	0.0	0.1	0	0	0	0.0	0.0	0.0	0	0	0
Tunisia	Meknassi	0.0	0.0	0.1	0	0	0	0.0	0.0	0.0	0	0	0
Tunisia	Melloulech	0.1	0.0	0.1	0	0	0	0.0	0.0	0.0	0	0	0
Tunisia	Menzel Bourguiba	0.1	0.1	0.1	0	0	0	0.0	0.0	0.0	0	0	0
Tunisia	Menzel Bouzaiene	0.0	0.0	0.0	0	0	0	0.0	0.0	0.0	0	0	0
Tunisia	Menzel Bouzelfa	0.0	0.0	0.1	0	0	0	0.0	0.0	0.0	0	0	0
Tunisia	Menzel Chaker	0.1	0.0	0.1	0	0	0	0.0	0.0	0.0	0	0	0
Tunisia	Menzel Habib	0.0	0.0	0.1	0	0	0	0.0	0.0	0.0	0	0	0
Tunisia	Menzel Jemil	0.1	0.0	0.1	0	0	0	0.0	0.0	0.0	0	0	0
Tunisia	Menzel Temime	0.0	0.0	0.0	0	0	0	0.0	0.0	0.0	0	0	0
Tunisia	Metlaoui	0.0	0.0	0.0	0	0	0	0.0	0.0	0.0	0	0	0
Tunisia	Metouia	0.1	0.0	0.1	0	0	0	0.0	0.0	0.0	0	0	0
Tunisia	Mnihla	0.1	0.1	0.1	0	0	1	0.0	0.0	0.0	0	0	0
Tunisia	Moknine	0.1	0.1	0.1	1	0	1	0.0	0.0	0.0	0	0	0
Tunisia	Monastir	0.1	0.1	0.1	1	1	1	0.0	0.0	0.0	0	0	0
Tunisia	Mornag	0.1	0.1	0.1	1	0	1	0.0	0.0	0.0	0	0	0
Tunisia	Mornaguia	0.1	0.0	0.1	0	0	0	0.0	0.0	0.0	0	0	0
Tunisia	Nabeul	0.0	0.0	0.1	0	0	0	0.0	0.0	0.0	0	0	0
Tunisia	Nadhour	0.1	0.1	0.1	0	0	0	0.0	0.0	0.0	0	0	0
Tunisia	Nasrallah	0.1	0.1	0.1	1	0	1	0.0	0.0	0.0	0	0	0
Tunisia	Nebeur	0.1	0.1	0.1	0	0	0	0.0	0.0	0.0	0	0	0
Tunisia	Nefta	0.0	0.0	0.1	0	0	0	0.0	0.0	0.0	0	0	0
Tunisia	Nefza	0.1	0.0	0.1	0	0	0	0.0	0.0	0.0	0	0	0

Table 3: Diarrhoea Mortality by Unit *(continued)*

Country	Unit	2000 mortality mean rate (per 10,000)	lower rate (per 10,000)	upper rate (per 10,000)	mean count	lower count	upper count	2017 mortality mean rate (per 10,000)	lower rate (per 10,000)	upper rate (per 10,000)	mean count	lower count	upper count
Tunisia	Nouvelle Médina	0.1	0.1	0.1	0	0	0	0.0	0.0	0.0	0	0	0
Tunisia	Omrane	0.1	0.1	0.1	0	0	0	0.0	0.0	0.0	0	0	0
Tunisia	Omrane Supérieur	0.1	0.1	0.1	0	0	0	0.0	0.0	0.0	0	0	0
Tunisia	Oued Ellil	0.1	0.0	0.1	0	0	0	0.0	0.0	0.0	0	0	0
Tunisia	Oued Mliz	0.1	0.1	0.1	0	0	0	0.0	0.0	0.0	0	0	0
Tunisia	Ouerdanine	0.1	0.1	0.1	0	0	0	0.0	0.0	0.0	0	0	0
Tunisia	Oueslatia	0.1	0.1	0.2	1	0	1	0.0	0.0	0.0	0	0	0
Tunisia	Ouled Chamekh	0.1	0.1	0.1	0	0	0	0.0	0.0	0.0	0	0	0
Tunisia	Ouled Haffouz	0.1	0.1	0.1	0	0	0	0.0	0.0	0.0	0	0	0
Tunisia	Oum Larais	0.0	0.0	0.0	0	0	0	0.0	0.0	0.0	0	0	0
Tunisia	Radès	0.1	0.1	0.1	0	0	0	0.0	0.0	0.0	0	0	0
Tunisia	Raoued	0.1	0.0	0.1	0	0	0	0.0	0.0	0.0	0	0	0
Tunisia	Ras Jebel	0.1	0.0	0.1	0	0	0	0.0	0.0	0.0	0	0	0
Tunisia	Redeyef	0.0	0.0	0.0	0	0	0	0.0	0.0	0.0	0	0	0
Tunisia	Regueb	0.1	0.0	0.1	0	0	0	0.0	0.0	0.0	0	0	0
Tunisia	Remada	0.0	0.0	0.0	0	0	0	0.0	0.0	0.0	0	0	0
Tunisia	Rouhia	0.1	0.1	0.1	0	0	0	0.0	0.0	0.0	0	0	0
Tunisia	Sabalat Ouled Asker	0.1	0.0	0.1	0	0	0	0.0	0.0	0.0	0	0	0
Tunisia	Sabkhet Sijoumi	0.1	0.1	0.1	0	0	0	0.0	0.0	0.0	0	0	0
Tunisia	Sahline	0.1	0.1	0.1	0	0	0	0.0	0.0	0.0	0	0	0
Tunisia	Sakiet Eddaier	0.0	0.0	0.1	1	0	1	0.0	0.0	0.0	0	0	0
Tunisia	Sakiet Ezzit	0.0	0.0	0.1	0	0	0	0.0	0.0	0.0	0	0	0
Tunisia	Sakiet Sidi Youssef	0.1	0.1	0.1	0	0	0	0.0	0.0	0.0	0	0	0
Tunisia	Samar	0.0	0.0	0.0	0	0	0	0.0	0.0	0.0	0	0	0
Tunisia	Saouaf	0.1	0.1	0.1	0	0	0	0.0	0.0	0.0	0	0	0
Tunisia	Sayada-Lamta-Bou Hjar	0.1	0.1	0.1	0	0	0	0.0	0.0	0.0	0	0	0
Tunisia	Sbeitla	0.0	0.0	0.1	0	0	0	0.0	0.0	0.0	0	0	0
Tunisia	Sbiba	0.1	0.1	0.1	0	0	0	0.0	0.0	0.0	0	0	0
Tunisia	Sbikha	0.1	0.1	0.2	1	1	1	0.0	0.0	0.0	0	0	0
Tunisia	Sebkhat Sidi El Hani	0.1	0.1	0.1	0	0	0	0.0	0.0	0.0	0	0	0
Tunisia	Sebkhet Ariana	0.1	0.0	0.1	0	0	0	0.0	0.0	0.0	0	0	0
Tunisia	Sebkhet El Moknine	0.1	0.1	0.1	0	0	0	0.0	0.0	0.0	0	0	0
Tunisia	Sebkhit El Kabla	0.1	0.1	0.1	0	0	0	0.0	0.0	0.0	0	0	0
Tunisia	Sejnane	0.1	0.0	0.1	0	0	0	0.0	0.0	0.0	0	0	0
Tunisia	Sened	0.0	0.0	0.0	0	0	0	0.0	0.0	0.0	0	0	0
Tunisia	Sfax Médina	0.0	0.0	0.1	1	0	1	0.0	0.0	0.0	0	0	0
Tunisia	Sfax Ouest	0.0	0.0	0.1	0	0	1	0.0	0.0	0.0	0	0	0
Tunisia	Sfax Sud	0.1	0.0	0.1	1	1	1	0.0	0.0	0.0	0	0	1
Tunisia	Sidi Aich	0.0	0.0	0.0	0	0	0	0.0	0.0	0.0	0	0	0
Tunisia	Sidi Ali Ben Aoun	0.0	0.0	0.0	0	0	0	0.0	0.0	0.0	0	0	0
Tunisia	Sidi Alouane	0.1	0.1	0.1	0	0	0	0.0	0.0	0.0	0	0	0
Tunisia	Sidi Bou Ali	0.1	0.1	0.1	0	0	0	0.0	0.0	0.0	0	0	0
Tunisia	Sidi Bouzid Est	0.1	0.1	0.1	1	0	1	0.0	0.0	0.0	0	0	0
Tunisia	Sidi Bouzid Ouest	0.1	0.0	0.1	0	0	0	0.0	0.0	0.0	0	0	0
Tunisia	Sidi El Béchir	0.1	0.1	0.1	0	0	0	0.0	0.0	0.0	0	0	0
Tunisia	Sidi El Heni	0.1	0.1	0.1	0	0	0	0.0	0.0	0.0	0	0	0
Tunisia	Sidi Hassine	0.1	0.1	0.1	1	0	1	0.0	0.0	0.0	0	0	0
Tunisia	Sidi Makhlouf	0.0	0.0	0.1	0	0	0	0.0	0.0	0.0	0	0	0
Tunisia	Sidi Thabet	0.1	0.0	0.1	0	0	0	0.0	0.0	0.0	0	0	0
Tunisia	Sijoumi	0.1	0.1	0.1	0	0	0	0.0	0.0	0.0	0	0	0
Tunisia	Siliana Nord	0.1	0.1	0.1	0	0	0	0.0	0.0	0.0	0	0	0
Tunisia	Siliana Sud	0.1	0.1	0.1	0	0	0	0.0	0.0	0.0	0	0	0

Country	Unit	2000 mortality mean rate (per 10,000)	lower rate (per 10,000)	upper rate (per 10,000)	mean count	lower count	upper count	2017 mortality mean rate (per 10,000)	lower rate (per 10,000)	upper rate (per 10,000)	mean count	lower count	upper count
Tunisia	Skhira	0.0	0.0	0.1	0	0	0	0.0	0.0	0.0	0	0	0
Tunisia	Soliman	0.0	0.0	0.1	0	0	0	0.0	0.0	0.0	0	0	0
Tunisia	Souassi	0.1	0.1	0.1	0	0	0	0.0	0.0	0.0	0	0	0
Tunisia	Souk El Ahed	0.0	0.0	0.1	0	0	0	0.0	0.0	0.0	0	0	0
Tunisia	Souk Jedid	0.1	0.0	0.1	0	0	0	0.0	0.0	0.0	0	0	0
Tunisia	Soukra	0.1	0.1	0.1	0	0	1	0.0	0.0	0.0	0	0	0
Tunisia	Sousse Jaouhara	0.1	0.1	0.1	0	0	0	0.0	0.0	0.0	0	0	0
Tunisia	Sousse Médina	0.1	0.1	0.1	0	0	0	0.0	0.0	0.0	0	0	0
Tunisia	Sousse Riadh	0.1	0.1	0.1	1	1	1	0.0	0.0	0.0	0	0	0
Tunisia	Sousse Sidi Abdelhamid	0.1	0.1	0.1	0	0	0	0.0	0.0	0.0	0	0	0
Tunisia	Tabarka	0.1	0.1	0.1	0	0	0	0.0	0.0	0.0	0	0	0
Tunisia	Tajerouine	0.1	0.0	0.1	0	0	0	0.0	0.0	0.0	0	0	0
Tunisia	Takelsa	0.0	0.0	0.1	0	0	0	0.0	0.0	0.0	0	0	0
Tunisia	Tamaghza	0.0	0.0	0.0	0	0	0	0.0	0.0	0.0	0	0	0
Tunisia	Tataouine Nord	0.0	0.0	0.0	0	0	0	0.0	0.0	0.0	0	0	0
Tunisia	Tataouine Sud	0.0	0.0	0.0	0	0	0	0.0	0.0	0.0	0	0	0
Tunisia	Teboulba	0.1	0.1	0.1	0	0	0	0.0	0.0	0.0	0	0	0
Tunisia	Tebourba	0.1	0.0	0.1	0	0	0	0.0	0.0	0.0	0	0	0
Tunisia	Téboursouk	0.1	0.1	0.1	0	0	0	0.0	0.0	0.0	0	0	0
Tunisia	Testour	0.1	0.0	0.1	0	0	0	0.0	0.0	0.0	0	0	0
Tunisia	Thala	0.1	0.0	0.1	0	0	0	0.0	0.0	0.0	0	0	0
Tunisia	Thibar	0.1	0.1	0.1	0	0	0	0.0	0.0	0.0	0	0	0
Tunisia	Tinja	0.1	0.1	0.1	0	0	0	0.0	0.0	0.0	0	0	0
Tunisia	Tozeur	0.0	0.0	0.1	0	0	0	0.0	0.0	0.0	0	0	0
Tunisia	Unknown	0.1	0.0	0.1	0	0	0	0.0	0.0	0.0	0	0	0
Tunisia	Unknown1	NA	NA	NA	NA	NA	NA	NA	NA	NA	NA	NA	NA
Tunisia	Utique	0.1	0.0	0.1	0	0	0	0.0	0.0	0.0	0	0	0
Tunisia	Zaghouan	0.1	0.1	0.1	0	0	0	0.0	0.0	0.0	0	0	0
Tunisia	Zarzis	0.0	0.0	0.1	0	0	0	0.0	0.0	0.0	0	0	0
Tunisia	Zeramdine	0.1	0.1	0.1	0	0	0	0.0	0.0	0.0	0	0	0
Tunisia	Zriba	0.1	0.1	0.1	0	0	0	0.0	0.0	0.0	0	0	0
Yemen	Abs	5.5	3.6	8.0	152	99	218	1.5	1.2	2.0	58	46	75
Yemen	Ad Dahi	5.5	3.7	8.0	52	35	75	1.6	1.2	2.0	20	16	26
Yemen	Ad Dhale'e	5.1	3.5	7.2	77	54	109	1.6	1.2	1.9	32	25	40
Yemen	Ad Dis	3.0	1.8	4.5	11	7	17	0.9	0.6	1.2	4	3	6
Yemen	Ad Durayhimi	5.7	3.8	8.1	86	57	123	1.5	1.1	1.9	31	23	39
Yemen	Adh Dhlia'ah	3.2	2.0	4.6	10	6	15	1.0	0.7	1.3	4	3	6
Yemen	Aflah Al Yaman	5.4	3.5	8.0	46	30	67	1.7	1.4	2.2	22	17	27
Yemen	Aflah Ash Shawm	5.4	3.5	7.9	44	29	65	1.7	1.3	2.2	20	15	25
Yemen	Ahwar	4.2	2.9	6.4	16	11	24	1.1	0.9	1.5	6	4	8
Yemen	Ain	4.3	2.8	6.5	20	13	30	1.3	1.0	1.6	9	7	11
Yemen	Al Hawtah	4.0	2.7	5.7	6	4	9	1.3	1.0	1.6	2	2	3
Yemen	Al Mukha	4.9	3.3	6.9	21	15	30	1.3	1.0	1.7	8	6	10
Yemen	Al A'rsh	5.1	3.5	7.0	48	33	66	1.6	1.2	2.0	21	16	26
Yemen	Al Abdiyah	4.6	3.1	6.6	11	7	16	1.4	1.1	1.8	5	4	6
Yemen	Al Abr	3.3	2.1	4.7	2	1	3	1.0	0.7	1.3	1	1	1
Yemen	Al Ashah	4.7	3.1	7.0	42	28	62	1.7	1.3	2.1	21	16	26
Yemen	Al Azariq	5.2	3.6	7.4	42	29	60	1.6	1.2	1.9	18	14	21
Yemen	Al Bayda	4.7	3.4	6.4	47	34	65	1.4	1.1	1.7	20	16	25
Yemen	Al Bayda City	4.7	3.3	6.5	14	10	20	1.4	1.1	1.7	5	4	6
Yemen	Al Buraiqeh	3.7	2.4	5.2	20	13	28	1.2	0.9	1.6	9	7	12
Yemen	Al Dhaher	4.9	3.2	7.0	22	14	31	1.6	1.3	2.1	10	8	13
Yemen	Al Dhihar	5.6	3.9	8.0	100	70	141	1.7	1.3	2.1	40	31	50
Yemen	Al Garrahi	5.5	3.7	8.0	95	63	138	1.5	1.1	1.9	35	27	46
Yemen	Al Ghaydah	3.3	2.0	5.1	15	9	23	0.9	0.6	1.3	6	4	8
Yemen	Al Ghayl	4.6	2.9	6.6	6	4	9	1.7	1.3	2.1	3	2	4
Yemen	Al Had	4.9	3.5	6.8	37	27	52	1.5	1.2	1.8	15	12	19
Yemen	Al Hada	5.3	3.5	7.6	153	99	219	1.6	1.2	1.9	63	50	78
Yemen	Al Hajjaylah	5.4	3.6	8.0	11	7	16	1.7	1.3	2.2	5	4	6

Table 3: Diarrhoea Mortality by Unit *(continued)*

Country	Unit	2000 mortality mean rate (per 10,000)	lower rate (per 10,000)	upper rate (per 10,000)	mean count	lower count	upper count	2017 mortality mean rate (per 10,000)	lower rate (per 10,000)	upper rate (per 10,000)	mean count	lower count	upper count
Yemen	Al Hali	5.6	3.8	8.1	168	112	241	1.5	1.1	1.9	61	45	77
Yemen	Al Hashwah	4.6	3.0	6.7	12	8	17	1.7	1.2	2.1	6	4	8
Yemen	Al Hawak	5.6	3.7	8.1	162	109	234	1.5	1.1	1.9	59	44	76
Yemen	Al Haymah Ad Dakhiliyah	5.0	3.4	7.3	73	49	105	1.7	1.3	2.1	34	26	42
Yemen	Al Haymah Al Kharijiyah	5.3	3.5	7.6	58	39	84	1.7	1.3	2.1	26	20	32
Yemen	Al Hazm	4.6	2.9	6.6	21	14	31	1.6	1.3	2.1	10	8	13
Yemen	Al Humaydat	4.9	3.1	7.1	16	10	23	1.7	1.3	2.2	7	6	9
Yemen	Al Husha	5.3	3.7	7.6	64	45	92	1.6	1.3	2.0	28	21	34
Yemen	Al Husn	5.1	3.3	7.1	27	17	38	1.6	1.2	2.0	11	9	15
Yemen	Al Hussein	5.1	3.6	7.0	36	25	49	1.6	1.2	1.9	16	12	20
Yemen	Al Jabin	5.5	3.7	8.1	74	50	109	1.7	1.3	2.1	31	25	39
Yemen	Al Jafariyah	5.6	3.8	8.1	78	54	113	1.6	1.3	2.1	30	24	39
Yemen	Al Jamimah	5.2	3.4	7.7	46	30	68	1.7	1.4	2.2	20	15	25
Yemen	Al Jubah	4.3	2.8	6.3	16	10	23	1.3	1.0	1.7	7	5	9
Yemen	Al Khabt	5.5	3.6	7.9	62	41	90	1.7	1.4	2.2	27	21	34
Yemen	Al Khalq	4.5	2.9	6.5	7	5	11	1.6	1.3	2.1	4	3	5
Yemen	Al Khawkhah	5.2	3.5	7.3	32	22	45	1.4	1.0	1.8	12	9	15
Yemen	Al Ma'afer	5.1	3.5	7.1	99	69	140	1.5	1.1	1.8	39	30	49
Yemen	Al Madan	4.9	3.3	7.2	24	16	35	1.7	1.3	2.1	11	9	14
Yemen	Al Madaribah Wa Al Arah	4.4	3.0	6.1	36	25	50	1.3	0.9	1.6	14	10	18
Yemen	Al Maflahy	4.9	3.5	6.9	27	19	38	1.4	1.1	1.8	11	9	14
Yemen	Al Maghrabah	5.3	3.5	7.9	67	44	101	1.8	1.4	2.2	31	24	38
Yemen	Al Mahabishah	5.4	3.5	8.0	54	35	80	1.8	1.4	2.2	25	20	32
Yemen	Al Mahfad	4.1	2.8	6.0	18	13	27	1.1	0.9	1.5	7	5	9
Yemen	Al Mahwait	5.3	3.5	7.6	63	42	91	1.7	1.4	2.2	29	23	37
Yemen	Al Mahwait City	5.2	3.5	7.6	11	8	17	1.7	1.4	2.2	5	4	6
Yemen	Al Makhadir	5.6	3.9	8.1	117	81	170	1.7	1.3	2.2	51	40	64
Yemen	Al Malagim	4.7	3.3	6.6	24	17	34	1.4	1.1	1.8	10	8	13
Yemen	Al Manar	5.6	3.7	8.0	58	38	83	1.7	1.3	2.1	23	18	30
Yemen	Al Mansura	3.7	2.5	5.3	58	39	83	1.2	0.9	1.6	24	18	32
Yemen	Al Mansuriyah	5.6	3.8	8.4	43	29	64	1.6	1.2	2.0	17	13	21
Yemen	Al Maqatirah	4.8	3.4	6.7	47	33	65	1.4	1.1	1.8	18	14	23
Yemen	Al Marawi'ah	5.6	3.7	8.1	134	89	193	1.5	1.2	1.9	50	38	63
Yemen	Al Mashannah	5.6	3.8	8.0	81	56	116	1.7	1.3	2.1	35	28	44
Yemen	Al Masilah	3.1	1.9	4.5	5	3	8	0.9	0.6	1.3	2	1	3
Yemen	Al Maslub	4.8	3.0	6.9	8	5	11	1.7	1.4	2.2	4	3	5
Yemen	Al Matammah	4.7	3.0	6.9	21	13	30	1.7	1.3	2.2	10	8	13
Yemen	Al Maton	4.8	3.0	6.8	19	12	27	1.7	1.3	2.2	9	7	11
Yemen	Al Mawasit	5.0	3.5	6.9	110	77	154	1.4	1.1	1.8	44	34	55
Yemen	Al Miftah	5.4	3.5	7.9	37	24	55	1.8	1.4	2.2	17	14	22
Yemen	Al Mighlaf	5.6	3.8	8.1	37	25	54	1.6	1.3	2.0	15	12	19
Yemen	Al Milah	4.6	3.1	6.4	24	16	34	1.4	1.1	1.7	10	8	13
Yemen	Al Mina	5.4	3.6	7.8	44	30	64	1.5	1.1	1.9	18	13	22
Yemen	Al Misrakh	5.1	3.5	7.2	86	59	122	1.5	1.2	1.9	36	27	44
Yemen	Al Mualla	3.6	2.4	5.2	13	8	18	1.1	0.9	1.5	5	4	7
Yemen	Al Mudhaffar	5.2	3.5	7.4	67	45	95	1.5	1.2	1.9	25	20	31
Yemen	Al Mukalla	3.0	1.9	4.5	10	6	16	0.9	0.7	1.3	4	3	6
Yemen	Al Mukalla City	3.0	1.9	4.6	59	37	90	0.9	0.6	1.3	23	16	33
Yemen	Al Munirah	5.5	3.7	8.1	37	25	55	1.5	1.2	1.9	13	11	17
Yemen	Al Musaymir	4.9	3.4	6.8	23	16	33	1.5	1.2	1.8	10	8	12
Yemen	Al Qabbaytah	4.8	3.3	6.8	78	53	109	1.4	1.1	1.8	32	24	39
Yemen	Al Qaf	3.4	2.2	4.9	2	1	2	1.0	0.7	1.4	1	0	1
Yemen	Al Qaflah	4.8	3.2	7.1	43	29	64	1.7	1.3	2.1	21	17	27
Yemen	Al Qafr	5.7	3.8	8.3	112	76	164	1.7	1.3	2.1	44	33	54
Yemen	Al Qahirah	5.3	3.5	7.5	63	42	90	1.5	1.2	1.9	24	19	30
Yemen	Al Qanawis	5.6	3.8	8.2	75	50	109	1.6	1.2	2.0	30	23	37
Yemen	Al Qatn	3.1	2.1	4.7	33	22	49	1.0	0.7	1.3	14	10	19

Country	Unit	2000 mortality mean rate (per 10,000)	lower rate (per 10,000)	upper rate (per 10,000)	mean count	lower count	upper count	2017 mortality mean rate (per 10,000)	lower rate (per 10,000)	upper rate (per 10,000)	mean count	lower count	upper count
Yemen	Al Quraishyah	5.1	3.4	7.1	30	20	41	1.5	1.2	1.9	12	9	15
Yemen	Al Talh	3.5	2.2	4.9	6	4	9	1.1	0.8	1.3	3	2	4
Yemen	Al Udayn	5.6	3.8	8.1	149	102	216	1.7	1.3	2.2	66	51	82
Yemen	Al Wade'a	4.8	3.3	6.9	18	12	26	1.2	0.9	1.6	7	5	8
Yemen	Al Wahdah	4.5	3.0	6.3	65	43	92	1.4	1.1	1.9	30	23	38
Yemen	Al Wazi'iyah	4.8	3.3	6.7	59	40	81	1.4	1.0	1.8	23	17	29
Yemen	Alluheyah	5.6	3.8	8.3	104	71	154	1.5	1.2	1.9	38	29	47
Yemen	Amd	3.2	2.0	4.7	11	7	16	1.0	0.7	1.3	5	3	6
Yemen	Amran	4.8	3.2	7.4	76	50	116	1.7	1.3	2.1	34	27	43
Yemen	An Nadirah	5.4	3.7	7.9	78	54	114	1.7	1.3	2.1	35	27	43
Yemen	Anss	5.4	3.6	7.9	168	110	245	1.6	1.3	2.0	72	56	90
Yemen	Ar Radmah	5.3	3.7	7.8	74	51	108	1.6	1.3	2.1	32	24	39
Yemen	Ar Rawdah	3.6	2.3	5.1	18	12	25	1.1	0.8	1.4	8	6	10
Yemen	Ar Raydah Wa Qusayar	3.1	1.9	4.4	19	12	28	0.9	0.6	1.2	7	5	10
Yemen	Ar Rujum	5.2	3.5	7.7	87	58	129	1.7	1.4	2.2	40	32	50
Yemen	Ar Ryashyyah	5.2	3.6	7.3	20	14	29	1.6	1.2	2.0	9	7	11
Yemen	Arhab	4.6	2.9	6.7	81	51	119	1.6	1.3	2.0	41	32	52
Yemen	Arma	3.5	2.2	5.0	6	4	9	1.1	0.8	1.4	3	2	3
Yemen	As Sabain	4.5	3.0	6.3	203	133	285	1.5	1.1	1.9	88	68	112
Yemen	As Sabrah	5.4	3.8	7.9	81	56	116	1.7	1.3	2.1	36	28	44
Yemen	As Saddah	5.4	3.7	7.9	82	56	119	1.7	1.3	2.1	38	29	47
Yemen	As Safra	4.7	3.0	6.7	76	49	109	1.7	1.3	2.2	38	29	49
Yemen	As Said	3.8	2.5	5.6	23	16	35	1.2	0.8	1.5	10	7	13
Yemen	As Salafiyah	5.5	3.7	8.1	85	58	126	1.7	1.3	2.1	36	28	46
Yemen	As Salif	5.1	3.4	7.5	4	3	6	1.4	1.1	1.9	2	1	2
Yemen	As Sawadiyah	4.8	3.3	6.8	22	15	31	1.5	1.2	1.8	10	8	12
Yemen	As Sawd	5.0	3.3	7.7	27	18	42	1.7	1.3	2.2	13	10	17
Yemen	As Sawm	3.2	2.0	4.8	7	5	11	0.9	0.6	1.4	3	2	4
Yemen	As Sawma'ah	4.7	3.3	6.4	36	25	49	1.4	1.0	1.7	14	11	18
Yemen	As Sayyani	5.4	3.7	7.7	129	88	183	1.7	1.3	2.1	56	44	69
Yemen	As Silw	5.0	3.4	7.1	104	71	147	1.5	1.1	1.8	42	32	53
Yemen	As Sudah	4.9	3.2	7.4	33	22	50	1.7	1.4	2.2	16	13	21
Yemen	As Sukhnah	5.6	3.7	8.1	60	40	87	1.6	1.2	2.0	24	18	30
Yemen	Ash Sha'ir	5.4	3.7	7.8	44	30	63	1.7	1.3	2.1	18	14	23
Yemen	Ash Shaghadirah	5.4	3.5	8.0	56	36	83	1.8	1.4	2.2	24	19	31
Yemen	Ash Shahil	5.6	3.7	8.2	40	26	58	1.8	1.4	2.3	16	13	21
Yemen	Ash Shaikh Outhman	3.7	2.5	5.4	35	23	50	1.2	0.9	1.5	15	11	20
Yemen	Ash Shamayatayn	4.9	3.4	6.8	41	29	57	1.4	1.1	1.8	16	12	20
Yemen	Ash Sharyah	5.1	3.5	7.1	32	22	44	1.5	1.2	1.9	13	10	16
Yemen	Ash Shihr	3.0	1.9	4.4	30	19	44	0.9	0.6	1.3	12	8	17
Yemen	Ash Shu'ayb	5.1	3.6	7.0	34	24	47	1.6	1.2	1.9	15	11	18
Yemen	Aslem	5.6	3.7	8.1	59	39	85	1.7	1.3	2.2	24	18	30
Yemen	Assafi'yah	4.3	2.7	6.0	65	41	91	1.4	1.0	1.8	29	22	38
Yemen	At Ta'iziyah	5.3	3.7	7.6	225	154	320	1.6	1.2	2.0	89	70	111
Yemen	At Taffah	4.9	3.5	6.9	29	21	41	1.5	1.2	1.8	12	9	15
Yemen	At Tahrir	4.5	3.0	6.3	24	16	35	1.4	1.1	1.8	11	9	14
Yemen	At Tawilah	5.1	3.4	7.6	58	39	86	1.7	1.4	2.1	27	21	33
Yemen	At Tuhayat	5.5	3.8	7.9	71	48	102	1.4	1.1	1.9	25	19	33
Yemen	Ataq	3.7	2.5	5.5	25	16	37	1.2	0.8	1.4	11	8	13
Yemen	Ath'thaorah	4.4	2.9	6.3	97	63	137	1.5	1.2	1.9	45	35	56
Yemen	Attawahi	3.6	2.4	5.2	13	9	19	1.1	0.9	1.5	6	4	8
Yemen	Attyal	4.6	2.9	6.6	29	18	42	1.5	1.2	2.0	13	10	17
Yemen	Az Zahir	4.8	3.4	6.6	21	15	29	1.4	1.1	1.8	8	7	11
Yemen	Az Zahir	4.8	3.0	6.8	14	9	20	1.7	1.3	2.2	7	5	9
Yemen	Az Zaydiyah	5.6	3.7	8.2	92	61	136	1.5	1.2	1.9	34	26	43
Yemen	Az Zuhrah	5.7	3.8	8.3	140	94	205	1.6	1.2	1.9	54	42	67
Yemen	Az'zal	4.3	2.7	6.0	82	52	115	1.4	1.0	1.8	37	28	48
Yemen	Ba'dan	5.5	3.8	7.8	116	81	165	1.7	1.3	2.1	49	38	61

Table 3: Diarrhoea Mortality by Unit *(continued)*

Country	Unit	2000 mortality mean rate (per 10,000)	lower rate (per 10,000)	upper rate (per 10,000)	mean count	lower count	upper count	2017 mortality mean rate (per 10,000)	lower rate (per 10,000)	upper rate (per 10,000)	mean count	lower count	upper count
Yemen	Bajil	5.6	3.8	8.1	186	125	270	1.6	1.2	2.0	72	55	90
Yemen	Bakil Al Mir	5.1	3.4	7.4	26	17	37	1.6	1.3	2.0	11	8	13
Yemen	Bani Al Awam	5.3	3.5	7.9	54	36	81	1.7	1.4	2.2	25	20	32
Yemen	Bani Al Harith	4.5	2.9	6.4	128	82	183	1.5	1.2	1.9	60	46	75
Yemen	Bani Dhabyan	4.8	3.1	6.9	16	10	23	1.5	1.2	1.9	7	5	8
Yemen	Bani Hushaysh	4.4	2.8	6.3	100	64	142	1.5	1.1	1.9	47	36	60
Yemen	Bani Matar	4.7	3.2	6.5	140	95	194	1.6	1.2	2.0	66	51	83
Yemen	Bani Qa'is	5.7	3.6	8.2	71	45	103	1.7	1.3	2.2	30	23	38
Yemen	Bani Sa'd	5.4	3.6	7.7	62	41	89	1.7	1.3	2.2	27	21	34
Yemen	Bani Suraim	4.7	3.1	7.0	30	20	45	1.7	1.3	2.1	15	12	19
Yemen	Baqim	4.5	3.0	6.4	25	17	35	1.6	1.2	2.0	12	9	15
Yemen	Bart Al Anan	4.7	3.1	6.7	40	26	57	1.7	1.2	2.1	19	14	25
Yemen	Bayhan	4.3	2.8	6.4	38	25	56	1.3	1.0	1.6	16	12	19
Yemen	Bayt Al Faqiah	5.7	3.9	8.2	242	167	351	1.5	1.2	2.0	89	70	114
Yemen	Bidbadah	4.6	2.9	6.6	12	8	17	1.6	1.2	2.0	5	4	7
Yemen	Bilad Ar Rus	5.1	3.3	7.0	30	20	42	1.6	1.3	2.1	13	10	17
Yemen	Bilad At Ta'am	5.5	3.6	7.9	37	25	53	1.7	1.3	2.2	15	12	20
Yemen	Brom Mayfa	3.1	2.0	4.6	9	6	14	1.0	0.7	1.3	4	3	5
Yemen	Bura	5.5	3.7	8.0	43	29	62	1.7	1.3	2.1	19	14	24
Yemen	Craiter	3.6	2.4	5.2	27	18	39	1.1	0.9	1.5	11	8	15
Yemen	Damt	5.3	3.7	7.5	62	44	88	1.6	1.3	2.0	27	21	33
Yemen	Dar Sad	3.8	2.5	5.5	33	22	47	1.2	0.9	1.6	14	11	19
Yemen	Daw'an	3.1	2.0	4.8	23	14	35	1.0	0.7	1.3	10	7	14
Yemen	Dawran Aness	5.3	3.5	7.6	126	83	180	1.6	1.3	2.0	53	42	66
Yemen	Dhamar City	5.4	3.5	7.7	155	101	222	1.6	1.2	2.0	60	46	74
Yemen	Dhar	3.4	2.0	4.8	6	3	8	1.0	0.7	1.3	2	2	3
Yemen	Dhi As Sufal	5.4	3.7	7.7	140	96	200	1.7	1.3	2.1	62	48	77
Yemen	Dhi Bin	4.6	3.0	6.8	28	18	41	1.7	1.3	2.1	14	11	18
Yemen	Dhi Na'im	4.7	3.4	6.6	20	14	28	1.4	1.1	1.8	9	7	11
Yemen	Dhubab	4.6	3.2	6.5	31	21	43	1.3	0.9	1.6	12	9	15
Yemen	Dimnat Khadir	5.1	3.5	7.2	65	45	93	1.5	1.1	1.8	27	20	33
Yemen	Far Al Udayn	5.6	3.9	8.2	89	61	129	1.7	1.3	2.1	36	28	45
Yemen	Ghamr	4.8	3.2	6.6	23	15	32	1.7	1.3	2.2	11	9	14
Yemen	Ghayl Ba Wazir	3.0	1.8	4.5	23	14	35	0.9	0.6	1.3	10	7	14
Yemen	Ghayl Bin Yamin	3.1	1.9	4.7	15	9	22	0.9	0.7	1.3	6	5	9
Yemen	Habban	3.7	2.5	5.3	20	13	29	1.2	0.9	1.5	9	6	11
Yemen	Habil Jabr	4.8	3.3	6.7	36	25	51	1.4	1.1	1.8	15	12	19
Yemen	Habur Zulaymah	4.9	3.2	7.4	38	25	57	1.7	1.3	2.2	18	14	23
Yemen	Hagr As Sai'ar	3.4	2.1	4.9	1	1	2	1.0	0.7	1.4	1	0	1
Yemen	Hajjah	5.4	3.6	8.0	34	23	51	1.8	1.4	2.2	16	13	21
Yemen	Hajjah City	5.3	3.5	7.9	55	36	82	1.8	1.4	2.2	24	19	31
Yemen	Hajr	3.2	2.1	4.8	14	9	21	1.0	0.8	1.4	6	5	8
Yemen	Halimayn	4.9	3.5	6.8	25	18	34	1.5	1.2	1.9	10	8	13
Yemen	Hamdan	4.6	3.0	6.5	93	62	133	1.6	1.2	2.0	48	37	60
Yemen	Harad	5.1	3.3	7.3	99	63	142	1.5	1.2	1.9	40	31	50
Yemen	Harf Sufyan	4.7	3.1	6.8	48	31	69	1.6	1.3	2.0	23	18	28
Yemen	Harib	4.3	2.9	6.4	22	15	33	1.3	1.0	1.7	9	7	11
Yemen	Harib Al Qaramish	4.6	2.8	6.6	7	4	10	1.5	1.2	2.0	3	2	4
Yemen	Hat	3.5	1.9	5.6	2	1	3	0.9	0.6	1.4	1	0	1
Yemen	Hatib	4.2	2.9	6.0	10	7	15	1.2	0.9	1.5	4	3	5
Yemen	Hawf	3.5	2.0	5.6	3	2	5	0.9	0.6	1.3	1	1	1
Yemen	Haydan	5.0	3.3	7.1	57	37	80	1.7	1.4	2.2	27	21	34
Yemen	Hayfan	4.9	3.5	6.9	134	94	189	1.5	1.1	1.8	54	41	68
Yemen	Hayran	5.3	3.4	7.5	16	10	22	1.5	1.2	1.9	6	5	8
Yemen	Hays	5.4	3.6	7.9	42	28	61	1.5	1.2	2.0	16	12	21
Yemen	Hazm Al Udayn	5.7	3.9	8.2	83	57	119	1.7	1.4	2.2	34	26	43
Yemen	Hidaybu	3.4	2.0	5.6	19	11	31	0.8	0.5	1.1	6	4	8
Yemen	Hubaysh	5.7	4.0	8.1	97	68	140	1.8	1.3	2.2	41	31	52
Yemen	Hufash	5.4	3.5	7.6	40	26	56	1.7	1.3	2.2	18	14	22

Table 3: Diarrhoea Mortality by Unit *(continued)*

Country	Unit	2000 mortality mean rate (per 10,000)	lower rate (per 10,000)	upper rate (per 10,000)	mean count	lower count	upper count	2017 mortality mean rate (per 10,000)	lower rate (per 10,000)	upper rate (per 10,000)	mean count	lower count	upper count
Yemen	Huraidhah	3.1	2.0	4.5	9	6	14	0.9	0.7	1.3	4	3	6
Yemen	Huswain	3.3	2.0	4.9	6	4	9	0.9	0.6	1.2	2	1	3
Yemen	Huth	4.7	3.0	6.8	22	14	33	1.6	1.3	2.1	11	9	14
Yemen	Ibb	5.6	3.9	8.0	182	127	259	1.7	1.3	2.1	75	58	93
Yemen	Iyal Surayh	4.7	3.2	7.1	67	45	100	1.7	1.3	2.1	36	28	46
Yemen	Jabal Ash sharq	5.4	3.7	7.9	68	46	98	1.7	1.3	2.1	29	22	37
Yemen	Jabal Habashy	5.1	3.6	7.3	30	21	43	1.5	1.1	1.9	12	9	15
Yemen	Jabal Iyal Yazid	4.9	3.2	7.5	91	60	140	1.7	1.3	2.2	44	34	56
Yemen	Jabal Murad	4.4	2.9	6.5	6	4	9	1.4	1.1	1.7	3	2	3
Yemen	Jabal Ra’s	5.6	3.8	8.1	52	35	75	1.6	1.3	2.1	21	16	26
Yemen	Jahaf	5.2	3.6	7.3	19	13	27	1.6	1.3	2.0	8	7	11
Yemen	Jahran	5.4	3.6	7.8	94	62	135	1.6	1.2	2.0	37	29	47
Yemen	Jardan	3.6	2.3	5.3	11	7	16	1.1	0.8	1.4	5	3	6
Yemen	Jayshan	4.4	3.0	6.2	10	7	14	1.3	0.9	1.6	4	3	5
Yemen	Jiblah	5.5	3.8	7.9	154	108	222	1.7	1.3	2.1	69	54	86
Yemen	Jihanah	4.8	3.0	6.7	42	27	60	1.5	1.2	2.0	20	15	25
Yemen	Juban	5.2	3.7	7.2	48	34	66	1.6	1.2	1.9	20	16	24
Yemen	Kamaran	5.4	3.6	8.0	2	1	2	1.5	1.1	2.0	1	0	1
Yemen	Khabb wa ash Sha’af	4.6	2.9	6.7	54	34	78	1.6	1.2	2.1	25	19	33
Yemen	Khamir	4.8	3.1	7.2	73	49	111	1.7	1.3	2.2	36	28	46
Yemen	Khanfir	4.3	3.0	6.0	71	49	100	1.2	1.0	1.5	27	21	34
Yemen	Kharab Al Marashi	4.7	3.1	6.8	38	25	54	1.7	1.3	2.1	19	15	23
Yemen	Kharif	4.6	3.0	6.9	46	30	69	1.6	1.3	2.1	22	18	28
Yemen	Khayran Al Muharraq	5.5	3.6	8.0	77	50	112	1.7	1.3	2.2	33	26	42
Yemen	Khur Maksar	3.7	2.5	5.3	17	11	25	1.1	0.9	1.5	8	6	11
Yemen	Khwlan	4.6	3.0	6.6	27	17	38	1.5	1.2	1.9	12	9	15
Yemen	Kitaf wa Al Boqe’e	4.6	3.0	6.7	46	31	68	1.6	1.2	2.1	22	17	28
Yemen	Ku’aydinah	5.7	3.7	8.3	87	56	125	1.7	1.4	2.2	35	28	45
Yemen	Kuhlan Affar	5.1	3.4	7.8	38	25	58	1.7	1.4	2.2	18	14	23
Yemen	Kuhlan Ash Sharaf	5.3	3.4	7.7	50	33	74	1.7	1.4	2.2	24	19	30
Yemen	Kushar	5.2	3.4	7.5	85	56	124	1.7	1.3	2.2	40	32	51
Yemen	Kusmah	5.5	3.7	8.1	83	56	123	1.7	1.3	2.1	36	28	46
Yemen	Lawdar	4.9	3.4	6.8	67	47	94	1.3	1.0	1.7	24	19	31
Yemen	Ma’ain	4.5	3.0	6.3	124	82	175	1.5	1.2	1.9	57	44	73
Yemen	Mabyan	5.4	3.5	8.2	66	43	100	1.8	1.4	2.3	31	24	39
Yemen	Maghirib Ans	5.6	3.8	8.2	60	40	88	1.7	1.3	2.1	25	19	31
Yemen	Mahliyah	4.7	3.2	6.8	8	6	12	1.4	1.1	1.8	3	3	4
Yemen	Majz	4.8	3.2	6.7	67	44	93	1.7	1.3	2.2	33	26	42
Yemen	Majzar	4.5	2.7	6.4	9	6	13	1.5	1.2	2.0	4	3	5
Yemen	Man’ar	3.4	2.0	5.1	3	2	5	0.9	0.6	1.3	1	1	2
Yemen	Manakhah	5.3	3.6	7.9	81	55	120	1.7	1.3	2.2	36	28	45
Yemen	Maqbanah	5.2	3.6	7.3	69	47	96	1.5	1.1	1.9	27	21	34
Yemen	Marib	4.2	2.6	6.0	32	20	46	1.4	1.0	1.7	15	11	18
Yemen	Marib City	4.2	2.6	6.1	20	13	30	1.4	1.0	1.7	9	7	11
Yemen	Mashra’a Wa Hadnan	5.2	3.5	7.4	47	32	67	1.5	1.2	1.9	19	15	24
Yemen	Maswar	5.2	3.5	7.9	44	30	67	1.7	1.4	2.2	21	16	26
Yemen	Maswarah	4.7	3.2	6.7	7	5	11	1.4	1.1	1.7	3	2	4
Yemen	Mawiyah	5.2	3.6	7.5	111	77	160	1.6	1.2	2.0	47	36	58
Yemen	Mawza	4.8	3.3	6.7	87	59	122	1.4	1.0	1.7	33	25	43
Yemen	Mayfa’a	3.5	2.3	4.9	26	17	36	1.1	0.8	1.4	11	8	15
Yemen	Mayfa’at Anss	5.3	3.5	7.5	66	44	95	1.6	1.2	2.0	28	22	35
Yemen	Mazhar	5.5	3.7	8.2	74	50	110	1.7	1.3	2.1	31	24	39
Yemen	Medghal	4.3	2.7	6.1	8	5	11	1.4	1.1	1.8	4	3	4
Yemen	Merkhah Al Ulya	4.4	3.0	6.3	25	17	35	1.3	1.0	1.6	10	8	13

Table 3: Diarrhoea Mortality by Unit *(continued)*

Country	Unit	2000 mortality mean rate (per 10,000)	lower rate (per 10,000)	upper rate (per 10,000)	mean count	lower count	upper count	2017 mortality mean rate (per 10,000)	lower rate (per 10,000)	upper rate (per 10,000)	mean count	lower count	upper count
Yemen	Merkhah As Sufla	4.1	2.8	6.0	31	21	45	1.2	0.9	1.5	13	10	16
Yemen	Midi	5.2	3.4	7.6	16	11	24	1.5	1.1	1.9	6	5	8
Yemen	Milhan	5.5	3.6	7.7	99	66	140	1.7	1.3	2.2	44	34	56
Yemen	Monabbih	4.8	3.2	6.6	47	31	65	1.6	1.3	2.1	22	17	28
Yemen	Mudhaykhirah	5.4	3.6	7.8	79	52	113	1.7	1.3	2.1	33	26	42
Yemen	Mudiyah	4.7	3.1	6.6	26	17	36	1.2	0.9	1.6	9	7	12
Yemen	Mukayras	4.8	3.4	6.6	36	26	50	1.4	1.1	1.7	15	12	19
Yemen	Mustaba	5.4	3.5	7.8	43	29	62	1.6	1.3	2.1	18	14	23
Yemen	Na'man	4.6	3.2	6.6	7	5	10	1.4	1.1	1.7	3	2	3
Yemen	Najrah	5.4	3.5	8.0	27	18	41	1.8	1.4	2.3	13	10	16
Yemen	Nati'	4.6	3.1	6.7	11	8	16	1.4	1.1	1.7	5	4	6
Yemen	Nihm	4.5	2.7	6.5	36	22	52	1.6	1.2	2.0	17	14	22
Yemen	Nisab	4.0	2.7	5.7	30	20	43	1.2	0.9	1.5	12	9	15
Yemen	Old City	4.3	2.7	6.0	20	13	28	1.4	1.0	1.8	9	7	12
Yemen	Qa'atabah	5.3	3.7	7.4	97	67	136	1.7	1.3	2.0	42	32	51
Yemen	Qafl Shamer	5.6	3.6	8.1	53	34	77	1.7	1.4	2.2	25	19	32
Yemen	Qarah	5.1	3.3	7.5	34	22	50	1.7	1.3	2.1	16	12	20
Yemen	Qatabir	4.7	3.2	6.6	18	12	25	1.6	1.3	2.1	9	7	11
Yemen	Qishn	3.3	1.9	4.8	5	3	8	0.9	0.6	1.2	2	1	3
Yemen	Qulensya Wa Abd Al Kuri	3.3	1.9	5.4	5	3	8	0.8	0.5	1.1	1	1	2
Yemen	Rada'	5.1	3.4	7.1	42	29	59	1.6	1.2	2.0	19	15	24
Yemen	Radfan	4.8	3.2	6.6	35	24	48	1.5	1.2	1.8	15	11	18
Yemen	Radman Al Awad	4.8	3.3	6.7	16	11	22	1.5	1.1	1.8	7	5	8
Yemen	Raghwan	4.4	2.7	6.2	3	2	5	1.5	1.2	1.9	2	1	2
Yemen	Rahabah	4.6	3.1	6.8	7	5	11	1.4	1.1	1.8	3	3	4
Yemen	Rajuzah	4.8	3.2	6.9	48	31	69	1.7	1.3	2.2	23	18	29
Yemen	Rakhyah	3.3	2.1	4.8	5	3	7	1.0	0.7	1.3	2	1	2
Yemen	Rasad	4.8	3.4	6.8	38	27	54	1.4	1.1	1.7	16	13	20
Yemen	Raydah	4.7	3.1	7.0	37	24	56	1.7	1.3	2.1	19	15	24
Yemen	Razih	5.0	3.3	7.0	57	37	79	1.8	1.4	2.3	28	22	36
Yemen	Rudum	3.4	2.2	4.9	14	9	20	1.0	0.8	1.4	6	4	8
Yemen	Rumah	3.6	2.1	5.5	4	2	6	1.0	0.6	1.4	1	1	2
Yemen	Sa'adah	4.7	3.0	6.8	33	21	48	1.7	1.3	2.2	16	13	21
Yemen	Sa'fan	5.4	3.6	7.9	31	21	45	1.7	1.3	2.2	14	10	17
Yemen	Sabah	5.3	3.6	7.5	27	19	39	1.6	1.3	2.0	12	9	14
Yemen	Sabir Al Mawadim	5.2	3.5	7.4	286	193	408	1.5	1.2	1.9	119	92	146
Yemen	Sah	3.1	2.0	4.6	13	8	19	0.9	0.7	1.3	5	4	7
Yemen	Sahar	4.8	3.1	6.8	117	77	169	1.7	1.3	2.2	61	47	77
Yemen	Salh	5.2	3.5	7.5	95	64	136	1.5	1.2	1.9	37	29	46
Yemen	Sama	5.0	3.5	7.1	29	20	41	1.5	1.1	1.8	11	9	14
Yemen	Sanhan	4.6	3.0	6.4	148	97	205	1.5	1.2	1.9	69	53	87
Yemen	Saqayn	4.9	3.2	6.9	56	37	78	1.8	1.4	2.3	28	22	35
Yemen	Sarar	4.7	3.3	6.6	13	9	18	1.4	1.1	1.7	5	4	6
Yemen	Sayhut	3.1	1.9	4.7	5	3	7	0.9	0.6	1.2	2	1	2
Yemen	Sayun	3.1	2.1	4.8	53	36	81	1.0	0.7	1.3	23	16	32
Yemen	Shada'a	5.1	3.3	7.0	8	5	10	1.7	1.4	2.3	3	2	4
Yemen	Shahan	3.6	2.0	5.9	2	1	3	0.9	0.6	1.4	1	0	1
Yemen	Shaharah	4.9	3.2	7.2	39	26	58	1.7	1.3	2.1	19	15	24
Yemen	Shara'b Ar Rawnah	5.3	3.6	7.6	159	108	225	1.6	1.3	2.0	65	52	81
Yemen	Shara'b As Salam	5.4	3.6	7.8	123	82	176	1.6	1.3	2.1	52	41	65
Yemen	Sharas	5.2	3.4	7.9	22	14	33	1.7	1.4	2.2	10	8	13
Yemen	Shibam	3.1	2.1	4.7	26	18	39	1.0	0.7	1.3	11	8	15
Yemen	Shibam Kawkaban	5.0	3.3	7.3	38	26	56	1.7	1.3	2.1	19	15	24
Yemen	Shu'aub	4.3	2.8	6.0	100	64	140	1.4	1.1	1.8	44	34	57
Yemen	Sibah	4.9	3.5	6.9	18	13	25	1.4	1.1	1.8	7	6	9
Yemen	Sirwah	4.3	2.8	6.2	16	10	23	1.4	1.1	1.8	7	6	9

Table 3: Diarrhoea Mortality by Unit *(continued)*

Country	Unit	2000 mortality mean rate (per 10,000)	lower rate (per 10,000)	upper rate (per 10,000)	mean count	lower count	upper count	2017 mortality mean rate (per 10,000)	lower rate (per 10,000)	upper rate (per 10,000)	mean count	lower count	upper count
Yemen	Suwayr	5.1	3.3	7.5	24	16	36	1.7	1.4	2.2	11	9	14
Yemen	Tarim	3.2	2.1	4.9	54	35	83	0.9	0.7	1.4	22	15	31
Yemen	Thamud	3.4	2.2	5.0	2	2	4	1.0	0.6	1.4	1	1	1
Yemen	Thula	5.0	3.3	7.6	42	28	64	1.7	1.3	2.2	19	15	24
Yemen	Tuban	4.0	2.7	5.7	68	45	97	1.3	0.9	1.6	30	22	38
Yemen	Tur Al Bahah	4.6	3.2	6.4	42	29	58	1.4	1.0	1.7	18	14	23
Yemen	Usaylan	4.1	2.6	6.2	23	15	35	1.2	0.9	1.5	10	7	12
Yemen	Utmah	5.6	3.8	8.3	159	107	236	1.7	1.3	2.1	67	52	84
Yemen	Wadhrah	5.6	3.7	8.4	8	5	12	1.8	1.4	2.3	3	3	4
Yemen	Wadi Al Ayn	3.1	2.1	4.5	15	10	22	1.0	0.7	1.3	6	5	9
Yemen	Wald Rabi'	5.1	3.4	7.1	19	13	26	1.5	1.2	1.9	8	6	9
Yemen	Washhah	5.4	3.7	8.0	65	44	96	1.7	1.3	2.2	28	22	35
Yemen	Wusab Al Ali	5.7	3.9	8.4	187	129	274	1.7	1.3	2.2	78	60	100
Yemen	Wusab As Safil	5.7	3.9	8.2	172	118	246	1.6	1.2	2.1	69	53	89
Yemen	Yabuth	3.4	2.2	4.7	5	4	8	1.1	0.8	1.4	2	2	3
Yemen	Yafa'a	4.9	3.5	6.9	60	43	84	1.4	1.2	1.8	23	19	29
Yemen	Yahr	4.8	3.4	6.7	33	24	46	1.4	1.1	1.8	13	10	16
Yemen	Yarim	5.4	3.7	7.9	173	116	251	1.7	1.3	2.0	71	55	87
Yemen	Zabid	5.6	3.8	8.2	156	105	227	1.5	1.2	1.9	58	44	74
Yemen	Zamakh wa Manwakh	3.5	2.3	4.9	1	1	2	1.0	0.7	1.5	0	0	1
Yemen	Zingibar	4.3	2.9	6.3	13	9	20	1.3	0.9	1.6	5	4	6
**South Asia**													
Bangladesh	Bagerhat	1.2	0.7	2.2	254	136	445	0.2	0.1	0.3	23	12	38
Bangladesh	Bandarban	1.9	1.1	2.9	114	66	178	0.2	0.1	0.4	15	8	24
Bangladesh	Barguna	1.5	0.8	2.3	172	88	270	0.2	0.1	0.4	17	10	29
Bangladesh	Barisal	1.5	0.8	2.5	476	261	815	0.2	0.1	0.4	44	26	72
Bangladesh	Bhola	1.5	0.8	2.5	419	229	683	0.2	0.1	0.4	42	23	68
Bangladesh	Bogra	1.2	0.7	2.1	502	283	846	0.2	0.1	0.3	60	32	102
Bangladesh	Brahamanbaria	1.6	0.9	2.6	686	382	1140	0.2	0.1	0.4	88	46	155
Bangladesh	Chandpur	1.5	0.8	2.6	517	269	896	0.2	0.1	0.4	56	29	92
Bangladesh	Chittagong	1.6	0.8	2.6	1420	740	2330	0.2	0.1	0.4	165	94	262
Bangladesh	Chuadanga	1.2	0.7	2.1	154	86	260	0.2	0.1	0.3	19	10	32
Bangladesh	Comilla	1.5	0.8	2.6	1100	611	1860	0.2	0.1	0.4	139	76	227
Bangladesh	Cox'S Bazar	1.7	1.0	2.7	500	292	770	0.2	0.1	0.4	68	36	113
Bangladesh	Dhaka	1.3	0.7	2.2	1270	670	2070	0.2	0.1	0.4	219	116	384
Bangladesh	Dinajpur	1.2	0.7	2.1	457	258	773	0.2	0.1	0.3	58	33	95
Bangladesh	Faridpur	1.2	0.7	2.0	319	176	520	0.2	0.1	0.3	37	19	61
Bangladesh	Feni	1.5	0.8	2.5	267	137	436	0.2	0.1	0.4	32	18	55
Bangladesh	Gaibandha	1.3	0.7	2.3	421	232	757	0.2	0.1	0.3	49	27	79
Bangladesh	Gazipur	1.3	0.7	2.2	373	208	616	0.2	0.1	0.4	80	44	155
Bangladesh	Gopalganj	1.3	0.7	2.2	218	116	379	0.2	0.1	0.3	21	12	37
Bangladesh	Habiganj	1.6	0.9	2.6	514	282	812	0.2	0.1	0.4	64	35	115
Bangladesh	Jamalpur	1.3	0.8	2.3	426	253	754	0.2	0.1	0.3	46	25	78
Bangladesh	Jessore	1.2	0.7	1.9	381	225	615	0.2	0.1	0.3	46	25	78
Bangladesh	Jhalokati	1.4	0.7	2.5	138	71	239	0.2	0.1	0.4	13	7	21
Bangladesh	Jhenaidah	1.2	0.7	1.9	240	136	380	0.2	0.1	0.3	29	16	51
Bangladesh	Joypurhat	1.2	0.6	2.0	122	66	201	0.2	0.1	0.3	14	8	24
Bangladesh	Khagrachhari	1.7	0.9	2.9	145	75	249	0.2	0.1	0.4	17	10	31
Bangladesh	Khulna	1.2	0.7	2.0	355	200	589	0.2	0.1	0.3	34	18	55
Bangladesh	Kishoreganj	1.4	0.8	2.2	632	364	1020	0.2	0.1	0.3	72	38	125
Bangladesh	Kurigram	1.3	0.7	2.2	348	189	618	0.2	0.1	0.3	43	24	74
Bangladesh	Kushtia	1.2	0.7	2.0	262	149	430	0.2	0.1	0.3	32	17	55
Bangladesh	Lakshmipur	1.5	0.8	2.8	374	203	690	0.2	0.1	0.4	47	26	74
Bangladesh	Lalmonirhat	1.3	0.7	2.2	210	119	365	0.2	0.1	0.3	26	14	43
Bangladesh	Madaripur	1.3	0.7	2.2	220	112	378	0.2	0.1	0.3	22	12	38
Bangladesh	Magura	1.2	0.7	1.9	137	75	223	0.2	0.1	0.3	17	9	29
Bangladesh	Manikganj	1.3	0.7	2.0	222	115	348	0.2	0.1	0.3	24	13	42
Bangladesh	Maulvibazar	1.6	0.9	2.7	388	216	654	0.2	0.1	0.4	49	28	88
Bangladesh	Meherpur	1.2	0.7	2.1	82	46	138	0.2	0.1	0.3	9	5	16

Table 3: Diarrhoea Mortality by Unit *(continued)*

Country	Unit	2000 mortality mean rate (per 10,000)	lower rate (per 10,000)	upper rate (per 10,000)	mean count	lower count	upper count	2017 mortality mean rate (per 10,000)	lower rate (per 10,000)	upper rate (per 10,000)	mean count	lower count	upper count
Bangladesh	Munshiganj	1.3	0.7	2.2	224	116	382	0.2	0.1	0.3	26	14	46
Bangladesh	Mymensingh	1.3	0.7	2.0	979	548	1540	0.2	0.1	0.4	119	65	215
Bangladesh	Naogaon	1.2	0.7	2.1	365	208	626	0.2	0.1	0.3	41	25	68
Bangladesh	Narail	1.2	0.6	2.1	129	67	218	0.2	0.1	0.3	13	7	22
Bangladesh	Narayanganj	1.3	0.7	2.2	383	199	640	0.2	0.1	0.3	61	30	107
Bangladesh	Narsingdi	1.3	0.8	2.2	392	224	638	0.2	0.1	0.4	49	27	91
Bangladesh	Natore	1.2	0.7	2.0	242	137	399	0.2	0.1	0.3	30	17	50
Bangladesh	Nawabganj	1.3	0.7	2.2	249	137	436	0.2	0.1	0.3	31	16	51
Bangladesh	Netrakona	1.3	0.7	2.2	457	245	753	0.2	0.1	0.3	53	29	94
Bangladesh	Nilphamari	1.2	0.7	2.1	293	159	501	0.2	0.1	0.4	40	20	68
Bangladesh	Noakhali	1.6	0.8	2.8	632	337	1110	0.2	0.1	0.4	82	46	134
Bangladesh	Pabna	1.3	0.7	2.0	396	234	620	0.2	0.1	0.3	49	28	84
Bangladesh	Panchagarh	1.3	0.7	2.2	164	84	277	0.2	0.1	0.4	22	11	39
Bangladesh	Patuakhali	1.5	0.8	2.6	316	166	535	0.2	0.1	0.4	31	19	51
Bangladesh	Pirojpur	1.4	0.8	2.5	215	116	371	0.2	0.1	0.4	21	12	34
Bangladesh	Rajbari	1.2	0.7	2.0	171	94	270	0.2	0.1	0.3	19	11	33
Bangladesh	Rajshahi	1.2	0.7	2.0	335	192	556	0.2	0.1	0.3	41	23	68
Bangladesh	Rangamati	1.7	0.9	2.9	137	72	229	0.2	0.1	0.4	16	9	29
Bangladesh	Rangpur	1.3	0.7	2.3	451	255	812	0.2	0.1	0.3	56	30	91
Bangladesh	Satkhira	1.2	0.7	1.9	267	160	430	0.2	0.1	0.3	29	16	47
Bangladesh	Shariatpur	1.3	0.7	2.3	216	117	381	0.2	0.1	0.3	23	12	40
Bangladesh	Sherpur	1.3	0.7	2.3	269	151	469	0.2	0.1	0.3	29	15	49
Bangladesh	Sirajganj	1.3	0.7	2.0	547	308	852	0.2	0.1	0.3	65	35	109
Bangladesh	Sunamganj	1.6	0.9	2.6	628	355	1020	0.2	0.1	0.4	84	48	136
Bangladesh	Sylhet	1.6	0.8	2.7	651	345	1120	0.2	0.1	0.4	104	57	178
Bangladesh	Tangail	1.3	0.7	2.1	586	315	928	0.2	0.1	0.3	64	36	114
Bangladesh	Thakurgaon	1.3	0.6	2.1	221	113	364	0.2	0.1	0.4	28	15	50
Bhutan	Athang	1.0	0.5	1.5	0	0	0	0.1	0.0	0.1	0	0	0
Bhutan	Balam	1.0	0.5	1.9	0	0	0	0.1	0.0	0.2	0	0	0
Bhutan	Bapisa	0.9	0.4	1.5	0	0	0	0.1	0.0	0.1	0	0	0
Bhutan	Bara	1.0	0.5	1.8	0	0	0	0.1	0.0	0.1	0	0	0
Bhutan	Bardo	1.1	0.5	1.8	0	0	0	0.1	0.1	0.2	0	0	0
Bhutan	Bartsham	1.0	0.6	1.9	0	0	0	0.1	0.0	0.2	0	0	0
Bhutan	Barzhong	0.9	0.4	1.5	0	0	0	0.1	0.0	0.1	0	0	0
Bhutan	Beteni	0.9	0.5	1.6	0	0	0	0.1	0.0	0.1	0	0	0
Bhutan	Bhur	0.8	0.4	1.4	0	0	1	0.1	0.0	0.1	0	0	0
Bhutan	Bidung	1.0	0.6	1.8	0	0	0	0.1	0.0	0.2	0	0	0
Bhutan	Biru	0.9	0.5	1.6	1	0	1	0.1	0.0	0.1	0	0	0
Bhutan	Bjachho	0.9	0.5	1.5	1	0	1	0.1	0.0	0.1	0	0	0
Bhutan	Bjena	1.0	0.5	1.6	0	0	0	0.1	0.0	0.1	0	0	0
Bhutan	Bji	0.9	0.5	1.5	0	0	0	0.1	0.0	0.1	0	0	0
Bhutan	Bjoka	1.0	0.5	1.8	0	0	0	0.1	0.0	0.2	0	0	0
Bhutan	Bongo	0.9	0.5	1.4	1	1	2	0.1	0.0	0.1	0	0	0
Bhutan	Bumdeling	1.2	0.6	2.0	0	0	0	0.1	0.1	0.2	0	0	0
Bhutan	Chang	0.9	0.5	1.5	6	3	10	0.1	0.0	0.1	1	0	1
Bhutan	Chapchha	0.9	0.5	1.6	0	0	1	0.1	0.0	0.1	0	0	0
Bhutan	Chargharay	0.8	0.4	1.4	0	0	1	0.1	0.0	0.1	0	0	0
Bhutan	Chaskhar	1.0	0.5	1.9	0	0	0	0.1	0.0	0.2	0	0	0
Bhutan	Chengmari	1.0	0.5	1.6	0	0	1	0.1	0.0	0.1	0	0	0
Bhutan	Chhali	1.0	0.5	1.8	0	0	0	0.1	0.0	0.2	0	0	0
Bhutan	Chhimung	0.9	0.5	1.7	0	0	0	0.1	0.0	0.2	0	0	0
Bhutan	Chhoekhor	1.0	0.5	1.6	1	0	1	0.1	0.0	0.1	0	0	0
Bhutan	Chhubu	0.9	0.4	1.5	0	0	0	0.1	0.0	0.1	0	0	0
Bhutan	Chhume	1.0	0.5	1.7	0	0	1	0.1	0.0	0.1	0	0	0
Bhutan	Chhuzagang	0.9	0.4	1.5	0	0	0	0.1	0.0	0.1	0	0	0
Bhutan	Chokhorling	1.0	0.5	1.8	0	0	0	0.1	0.0	0.2	0	0	0
Bhutan	Chongshing	0.9	0.4	1.7	0	0	0	0.1	0.0	0.2	0	0	0
Bhutan	Daga	1.0	0.5	1.6	0	0	0	0.1	0.0	0.1	0	0	0
Bhutan	Dagala	0.9	0.5	1.6	1	1	2	0.1	0.0	0.1	0	0	0
Bhutan	Dala	0.9	0.5	1.4	1	0	1	0.1	0.0	0.1	0	0	0

Table 3: Diarrhoea Mortality by Unit *(continued)*

Country	Unit	2000 mortality mean rate (per 10,000)	lower rate (per 10,000)	upper rate (per 10,000)	mean count	lower count	upper count	2017 mortality mean rate (per 10,000)	lower rate (per 10,000)	upper rate (per 10,000)	mean count	lower count	upper count
Bhutan	Dangchhu	1.0	0.5	1.6	0	0	0	0.1	0.0	0.1	0	0	0
Bhutan	Dechhenling	1.0	0.5	1.8	0	0	0	0.1	0.0	0.2	0	0	0
Bhutan	Dekiling	1.0	0.5	1.7	0	0	1	0.1	0.0	0.1	0	0	0
Bhutan	Denchhukha	0.9	0.5	1.5	0	0	0	0.1	0.0	0.1	0	0	0
Bhutan	Deorali	1.0	0.5	1.6	0	0	0	0.1	0.0	0.1	0	0	0
Bhutan	Dewathang	1.0	0.5	1.8	1	1	2	0.1	0.0	0.1	0	0	0
Bhutan	Doban	0.9	0.5	1.4	0	0	0	0.1	0.0	0.1	0	0	0
Bhutan	Doga	0.9	0.5	1.5	0	0	0	0.1	0.0	0.1	0	0	0
Bhutan	Dopshari	0.9	0.5	1.5	0	0	0	0.1	0.0	0.1	0	0	0
Bhutan	Dorokha	0.9	0.5	1.5	0	0	1	0.1	0.0	0.1	0	0	0
Bhutan	Dorona	1.1	0.6	1.8	0	0	0	0.1	0.1	0.2	0	0	0
Bhutan	Doteng	0.9	0.5	1.5	0	0	0	0.1	0.0	0.1	0	0	0
Bhutan	Dragteng	1.0	0.5	1.7	0	0	0	0.1	0.0	0.1	0	0	0
Bhutan	Drametse	1.0	0.5	1.9	0	0	0	0.1	0.0	0.2	0	0	0
Bhutan	Drepung	1.0	0.5	1.9	0	0	1	0.1	0.0	0.2	0	0	0
Bhutan	Drugyelgang	1.0	0.5	1.6	0	0	1	0.1	0.1	0.2	0	0	0
Bhutan	Dunglegang	0.9	0.4	1.5	0	0	0	0.1	0.0	0.1	0	0	0
Bhutan	Dungmin	0.9	0.5	1.7	0	0	0	0.1	0.0	0.2	0	0	0
Bhutan	Dungna	0.9	0.5	1.5	0	0	0	0.1	0.0	0.1	0	0	0
Bhutan	Dungtoe	0.9	0.5	1.5	0	0	0	0.1	0.0	0.1	0	0	0
Bhutan	Dzoma	0.9	0.4	1.5	0	0	0	0.1	0.0	0.1	0	0	0
Bhutan	Gakiling	0.9	0.5	1.6	0	0	0	0.1	0.0	0.1	0	0	0
Bhutan	Gangte	1.0	0.5	1.5	0	0	0	0.1	0.0	0.1	0	0	0
Bhutan	Gangzur	1.1	0.6	2.0	0	0	1	0.1	0.1	0.2	0	0	0
Bhutan	Gasetsho Gom	0.9	0.5	1.6	0	0	0	0.1	0.0	0.1	0	0	0
Bhutan	Gasetsho Om	0.9	0.5	1.7	0	0	1	0.1	0.0	0.1	0	0	0
Bhutan	Gelephu	0.9	0.4	1.5	1	0	1	0.1	0.0	0.1	0	0	0
Bhutan	Geling	0.9	0.4	1.5	1	0	2	0.1	0.0	0.1	0	0	0
Bhutan	Genye	0.9	0.5	1.6	0	0	0	0.1	0.0	0.1	0	0	0
Bhutan	Gesarling	1.1	0.6	1.7	0	0	0	0.1	0.1	0.2	0	0	0
Bhutan	Getana	1.0	0.5	1.6	0	0	0	0.1	0.0	0.1	0	0	0
Bhutan	Goenkhame	1.0	0.5	1.8	0	0	0	0.1	0.0	0.1	0	0	0
Bhutan	Goenkhatoe	1.1	0.5	2.0	0	0	0	0.1	0.0	0.2	0	0	0
Bhutan	Goenshari	0.9	0.4	1.5	0	0	0	0.1	0.0	0.1	0	0	0
Bhutan	Gomdar	0.9	0.5	1.7	0	0	0	0.1	0.0	0.2	0	0	0
Bhutan	Gongdue	1.0	0.5	1.7	0	0	0	0.1	0.0	0.2	0	0	0
Bhutan	Gosarling	0.9	0.4	1.5	0	0	0	0.1	0.0	0.1	0	0	0
Bhutan	Gozhi	1.0	0.5	1.6	0	0	0	0.1	0.1	0.2	0	0	0
Bhutan	Gozhing	1.0	0.5	1.9	0	0	0	0.1	0.1	0.2	0	0	0
Bhutan	Guma	0.9	0.4	1.5	0	0	0	0.1	0.0	0.1	0	0	0
Bhutan	Hiley	0.9	0.4	1.4	0	0	1	0.1	0.0	0.1	0	0	0
Bhutan	Hungrel	0.9	0.5	1.5	0	0	0	0.1	0.0	0.1	0	0	0
Bhutan	Jamkhar	1.1	0.6	2.0	0	0	0	0.1	0.0	0.2	0	0	0
Bhutan	Jangchhubling	0.9	0.5	1.7	0	0	1	0.1	0.0	0.2	0	0	0
Bhutan	Jaray	1.1	0.6	2.1	0	0	0	0.1	0.1	0.2	0	0	0
Bhutan	Jigmichhoeling	0.9	0.4	1.4	0	0	1	0.1	0.0	0.1	0	0	0
Bhutan	Jurmey	0.9	0.5	1.8	0	0	0	0.1	0.0	0.2	0	0	0
Bhutan	Kabjisa	0.9	0.5	1.5	0	0	0	0.1	0.0	0.1	0	0	0
Bhutan	Kalidzingkha	1.1	0.6	1.7	0	0	0	0.1	0.1	0.2	0	0	0
Bhutan	Kanglung	1.0	0.5	1.8	1	0	1	0.1	0.0	0.2	0	0	0
Bhutan	Kangpara	1.0	0.6	1.8	0	0	1	0.1	0.0	0.2	0	0	0
Bhutan	Katsho	0.9	0.5	1.6	0	0	0	0.1	0.0	0.1	0	0	0
Bhutan	Kawang	0.9	0.5	1.5	0	0	0	0.1	0.0	0.1	0	0	0
Bhutan	Kazhi	0.9	0.5	1.5	0	0	0	0.1	0.0	0.1	0	0	0
Bhutan	Kengkhar	1.0	0.5	1.9	0	0	0	0.1	0.0	0.2	0	0	0
Bhutan	Khaling	1.0	0.5	1.9	1	0	1	0.1	0.0	0.2	0	0	0
Bhutan	Khamdang	1.1	0.6	2.0	0	0	1	0.1	0.0	0.2	0	0	0
Bhutan	Khar	0.9	0.5	1.7	0	0	0	0.1	0.0	0.2	0	0	0
Bhutan	Khipisa	1.1	0.5	1.7	0	0	0	0.1	0.1	0.2	0	0	0
Bhutan	Khoma	1.1	0.6	2.0	0	0	0	0.1	0.1	0.2	0	0	0

Table 3: Diarrhoea Mortality by Unit *(continued)*

Country	Unit	2000 mortality mean rate (per 10,000)	lower rate (per 10,000)	upper rate (per 10,000)	mean count	lower count	upper count	2017 mortality mean rate (per 10,000)	lower rate (per 10,000)	upper rate (per 10,000)	mean count	lower count	upper count
Bhutan	Kikorthang	0.9	0.4	1.5	0	0	0	0.1	0.0	0.1	0	0	0
Bhutan	Korphu	0.9	0.5	1.4	0	0	0	0.1	0.0	0.1	0	0	0
Bhutan	Kurtoe	1.1	0.6	1.8	0	0	0	0.1	0.1	0.2	0	0	0
Bhutan	Lajab	1.1	0.6	1.8	0	0	0	0.1	0.1	0.2	0	0	0
Bhutan	Lamgong	0.9	0.4	1.5	0	0	1	0.1	0.0	0.1	0	0	0
Bhutan	Langchhenphu	0.8	0.5	1.5	1	0	1	0.1	0.0	0.1	0	0	0
Bhutan	Langthil	1.0	0.5	1.6	0	0	1	0.1	0.0	0.1	0	0	0
Bhutan	Lauri	1.0	0.5	1.7	0	0	1	0.1	0.0	0.2	0	0	0
Bhutan	Laya	1.1	0.5	1.9	0	0	0	0.1	0.0	0.2	0	0	0
Bhutan	Lhamoizingkha	0.9	0.5	1.6	0	0	0	0.1	0.0	0.1	0	0	0
Bhutan	Lingmukha	0.9	0.4	1.5	0	0	0	0.1	0.0	0.1	0	0	0
Bhutan	Lingzhi	1.1	0.5	1.8	0	0	0	0.1	0.0	0.1	0	0	0
Bhutan	Logchina	0.9	0.4	1.5	0	0	1	0.1	0.0	0.1	0	0	0
Bhutan	Lumang	1.0	0.5	1.7	1	0	1	0.1	0.0	0.2	0	0	0
Bhutan	Lunana	1.0	0.5	1.7	0	0	0	0.1	0.0	0.1	0	0	0
Bhutan	Lungnyi	0.9	0.5	1.5	1	0	1	0.1	0.0	0.1	0	0	0
Bhutan	Martshala	0.9	0.5	1.6	0	0	1	0.1	0.0	0.2	0	0	0
Bhutan	Menbi	1.1	0.6	2.2	0	0	1	0.1	0.1	0.2	0	0	0
Bhutan	Mendrelgang	0.9	0.5	1.5	0	0	0	0.1	0.0	0.1	0	0	0
Bhutan	Merak	1.0	0.6	1.7	0	0	0	0.1	0.0	0.2	0	0	0
Bhutan	Metap	0.9	0.5	1.6	0	0	0	0.1	0.0	0.1	0	0	0
Bhutan	Metsho	1.1	0.6	2.1	0	0	0	0.1	0.1	0.2	0	0	0
Bhutan	Mewang	0.9	0.5	1.5	1	1	2	0.1	0.0	0.1	0	0	0
Bhutan	Minjay	1.1	0.6	2.1	0	0	0	0.1	0.1	0.2	0	0	0
Bhutan	Mongar	1.0	0.5	1.9	1	0	1	0.1	0.0	0.2	0	0	0
Bhutan	Nahi	0.9	0.5	1.6	0	0	1	0.1	0.0	0.1	0	0	0
Bhutan	Naja	0.9	0.5	1.5	0	0	0	0.1	0.0	0.1	0	0	0
Bhutan	Namgyel Chhoeling	1.0	0.5	1.6	1	0	1	0.1	0.0	0.1	0	0	0
Bhutan	Nangkor	1.0	0.5	1.8	0	0	1	0.1	0.1	0.2	0	0	0
Bhutan	Nanong	0.9	0.5	1.7	0	0	1	0.1	0.0	0.2	0	0	0
Bhutan	Narang	1.0	0.5	1.9	0	0	1	0.1	0.0	0.2	0	0	0
Bhutan	Naro	1.0	0.5	1.6	0	0	0	0.1	0.0	0.1	0	0	0
Bhutan	Ngangla	1.0	0.5	1.7	0	0	0	0.1	0.0	0.2	0	0	0
Bhutan	Ngatshang	1.0	0.5	1.9	0	0	0	0.1	0.0	0.2	0	0	0
Bhutan	Nichula	1.0	0.5	1.7	0	0	0	0.1	0.0	0.1	0	0	0
Bhutan	Norbugang	1.0	0.5	1.7	0	0	1	0.1	0.0	0.2	0	0	0
Bhutan	Nubi	1.0	0.5	1.7	0	0	1	0.1	0.0	0.1	0	0	0
Bhutan	Nyisho	1.0	0.5	1.5	0	0	0	0.1	0.0	0.1	0	0	0
Bhutan	Pagli	0.8	0.5	1.3	1	0	1	0.1	0.0	0.1	0	0	0
Bhutan	Pangkhar	1.0	0.5	1.7	0	0	0	0.1	0.0	0.2	0	0	0
Bhutan	Patakla	0.9	0.5	1.6	0	0	0	0.1	0.0	0.2	0	0	0
Bhutan	Pemathang	0.9	0.4	1.5	0	0	0	0.1	0.0	0.1	0	0	0
Bhutan	Phangyuel	0.9	0.5	1.5	0	0	0	0.1	0.0	0.1	0	0	0
Bhutan	Phobji	1.0	0.5	1.5	0	0	0	0.1	0.0	0.1	0	0	0
Bhutan	Phongme	1.0	0.6	1.9	0	0	0	0.1	0.0	0.2	0	0	0
Bhutan	Phuentenchhu	0.9	0.4	1.5	0	0	0	0.1	0.0	0.1	0	0	0
Bhutan	Phuentsholing	0.8	0.4	1.3	2	1	4	0.1	0.0	0.1	0	0	1
Bhutan	Phuntsthothang	0.9	0.5	1.7	1	0	1	0.1	0.0	0.1	0	0	0
Bhutan	Radi	1.0	0.5	1.7	0	0	0	0.1	0.0	0.2	0	0	0
Bhutan	Ramjar	1.1	0.6	2.0	0	0	0	0.1	0.1	0.2	0	0	0
Bhutan	Rangthangling	0.9	0.5	1.6	0	0	0	0.1	0.0	0.1	0	0	0
Bhutan	Ruepisa	0.9	0.5	1.6	1	0	1	0.1	0.0	0.1	0	0	0
Bhutan	Sakteng	1.0	0.6	1.8	0	0	0	0.1	0.0	0.2	0	0	0
Bhutan	Saleng	1.0	0.5	1.9	1	0	1	0.1	0.0	0.2	0	0	0
Bhutan	Sama	0.9	0.5	1.6	0	0	0	0.1	0.0	0.2	0	0	0
Bhutan	Samkhar	1.0	0.5	1.8	1	0	1	0.1	0.0	0.2	0	0	0
Bhutan	Samphelling	0.9	0.4	1.5	1	0	1	0.1	0.0	0.1	0	0	0
Bhutan	Samrang	0.9	0.5	1.6	0	0	0	0.1	0.0	0.2	0	0	0
Bhutan	Samtse	0.9	0.5	1.4	1	0	1	0.1	0.0	0.1	0	0	0

Table 3: Diarrhoea Mortality by Unit *(continued)*

Country	Unit	2000 mortality mean rate (per 10,000)	lower rate (per 10,000)	upper rate (per 10,000)	mean count	lower count	upper count	2017 mortality mean rate (per 10,000)	lower rate (per 10,000)	upper rate (per 10,000)	mean count	lower count	upper count
Bhutan	Senge	0.9	0.5	1.5	0	0	0	0.1	0.0	0.1	0	0	0
Bhutan	Sephu	1.0	0.5	1.5	0	0	0	0.1	0.0	0.1	0	0	0
Bhutan	Serthig	0.9	0.5	1.7	0	0	0	0.1	0.0	0.2	0	0	0
Bhutan	Shapa	0.9	0.5	1.5	1	0	1	0.1	0.0	0.1	0	0	0
Bhutan	Shemjong	0.9	0.4	1.5	0	0	0	0.1	0.0	0.1	0	0	0
Bhutan	Shengabjimi	0.9	0.5	1.5	0	0	0	0.1	0.0	0.1	0	0	0
Bhutan	Shermung	1.0	0.5	1.9	0	0	1	0.1	0.0	0.2	0	0	0
Bhutan	Sherzhong	0.8	0.4	1.4	0	0	0	0.1	0.0	0.1	0	0	0
Bhutan	Shingkhar	1.1	0.5	1.9	0	0	0	0.1	0.1	0.2	0	0	0
Bhutan	Shompangkha	0.8	0.4	1.3	0	0	0	0.1	0.0	0.1	0	0	0
Bhutan	Shongphu	1.0	0.6	1.8	1	0	1	0.1	0.0	0.2	0	0	0
Bhutan	Shumer	0.9	0.5	1.8	0	0	1	0.1	0.0	0.2	0	0	0
Bhutan	Silambi	1.0	0.5	1.8	0	0	0	0.1	0.0	0.2	0	0	0
Bhutan	Sipsu	0.9	0.5	1.5	0	0	1	0.1	0.0	0.1	0	0	0
Bhutan	Soe	1.0	0.4	1.5	0	0	0	0.1	0.0	0.1	0	0	0
Bhutan	Sombey	1.0	0.5	1.6	0	0	0	0.1	0.0	0.1	0	0	0
Bhutan	Tading	0.8	0.4	1.3	1	1	2	0.1	0.0	0.1	0	0	0
Bhutan	Taklai	0.9	0.4	1.5	0	0	0	0.1	0.0	0.1	0	0	0
Bhutan	Talo	0.9	0.4	1.5	0	0	0	0.1	0.0	0.1	0	0	0
Bhutan	Tang	1.0	0.5	1.8	0	0	0	0.1	0.0	0.1	0	0	0
Bhutan	Tangsibji	1.0	0.5	1.6	0	0	1	0.1	0.0	0.1	0	0	0
Bhutan	Tendu	1.0	0.5	1.7	0	0	1	0.1	0.0	0.1	0	0	0
Bhutan	Thangrong	1.0	0.5	1.9	0	0	0	0.1	0.0	0.2	0	0	0
Bhutan	Thedtsho	0.9	0.4	1.4	0	0	1	0.1	0.0	0.1	0	0	0
Bhutan	Thrimshing	1.0	0.5	1.8	0	0	0	0.1	0.0	0.2	0	0	0
Bhutan	Toepisa	0.9	0.5	1.5	0	0	1	0.1	0.0	0.1	0	0	0
Bhutan	Toetsho	1.1	0.6	2.0	0	0	0	0.1	0.0	0.2	0	0	0
Bhutan	Toewang	0.9	0.5	1.6	0	0	0	0.1	0.0	0.1	0	0	0
Bhutan	Tomzhangtshen	1.1	0.6	2.0	0	0	1	0.1	0.0	0.2	0	0	0
Bhutan	Trashiding	1.0	0.5	1.7	0	0	0	0.1	0.1	0.2	0	0	0
Bhutan	Trashiyangtse	1.1	0.6	2.0	1	0	1	0.1	0.1	0.2	0	0	0
Bhutan	Trong	1.0	0.5	1.6	1	0	1	0.1	0.0	0.2	0	0	0
Bhutan	Tsakaling	1.0	0.5	1.8	0	0	0	0.1	0.0	0.2	0	0	0
Bhutan	Tsamang	1.0	0.5	1.9	0	0	0	0.1	0.1	0.2	0	0	0
Bhutan	Tsangkha	1.0	0.5	1.7	0	0	0	0.1	0.1	0.2	0	0	0
Bhutan	Tsendagang	1.1	0.6	1.7	0	0	0	0.1	0.1	0.2	0	0	0
Bhutan	Tsenkhar	1.1	0.6	2.1	0	0	0	0.1	0.1	0.2	0	0	0
Bhutan	Tsento	0.9	0.5	1.6	0	0	1	0.1	0.0	0.1	0	0	0
Bhutan	Tseza	1.1	0.6	1.8	0	0	1	0.1	0.1	0.2	0	0	0
Bhutan	Tsholingkhor	0.9	0.5	1.5	0	0	0	0.1	0.0	0.1	0	0	0
Bhutan	Tsirangtoe	0.9	0.4	1.5	0	0	0	0.1	0.0	0.1	0	0	0
Bhutan	Udzorong	1.0	0.5	1.8	0	0	1	0.1	0.0	0.2	0	0	0
Bhutan	Uesu	0.9	0.5	1.6	0	0	1	0.1	0.0	0.1	0	0	0
Bhutan	Ugentse	1.0	0.5	1.6	0	0	0	0.1	0.0	0.1	0	0	0
Bhutan	Umling	0.9	0.4	1.5	0	0	0	0.1	0.0	0.1	0	0	0
Bhutan	Ura	1.0	0.5	1.7	0	0	0	0.1	0.0	0.1	0	0	0
Bhutan	Wangchang	0.9	0.5	1.5	0	0	1	0.1	0.0	0.1	0	0	0
Bhutan	Wangphu	0.9	0.5	1.6	0	0	0	0.1	0.0	0.2	0	0	0
Bhutan	Yalang	1.1	0.6	2.0	0	0	0	0.1	0.1	0.2	0	0	0
Bhutan	Yangnyer	1.0	0.5	1.9	0	0	1	0.1	0.0	0.2	0	0	0
Bhutan	Yoeseltse	0.9	0.4	1.4	0	0	0	0.1	0.0	0.1	0	0	0
Bhutan	Yurung	0.9	0.5	1.7	0	0	0	0.1	0.0	0.2	0	0	0
Bhutan	Zobel	0.9	0.5	1.7	0	0	0	0.1	0.0	0.2	0	0	0
India	Adilabad	1.7	1.1	2.7	150	97	232	0.3	0.2	0.5	23	13	35
India	Agar Malwa	3.2	2.0	5.0	212	130	334	0.6	0.3	0.9	39	23	63
India	Agra	4.5	2.6	7.2	2580	1500	4090	1.6	1.0	2.5	919	593	1440
India	Ahmadnagar	0.9	0.6	1.3	418	276	628	0.2	0.1	0.3	81	49	126
India	Ahmedabad	1.4	0.9	2.3	983	602	1550	0.3	0.2	0.5	235	149	364
India	Aizawl	0.7	0.5	1.1	6	4	10	0.2	0.1	0.4	2	1	3
India	Ajmer	2.6	1.6	4.1	805	484	1260	0.6	0.3	0.9	172	107	265

Table 3: Diarrhoea Mortality by Unit *(continued)*

Country	Unit	2000 mortality mean rate (per 10,000)	lower rate (per 10,000)	upper rate (per 10,000)	mean count	lower count	upper count	2017 mortality mean rate (per 10,000)	lower rate (per 10,000)	upper rate (per 10,000)	mean count	lower count	upper count
India	Akola	0.9	0.6	1.5	171	104	276	0.2	0.1	0.3	31	18	48
India	Alappuzha	0.1	0.0	0.1	10	6	16	0.0	0.0	0.0	1	1	2
India	Aligarh	4.5	2.7	7.2	2200	1310	3520	1.6	1.0	2.7	820	485	1340
India	Alipurduar	1.1	0.7	1.7	133	85	203	0.2	0.1	0.3	23	14	35
India	Alirajpur	2.5	1.6	4.0	302	189	478	0.6	0.4	0.9	86	49	130
India	Allahabad	4.4	2.8	7.0	2670	1680	4210	1.5	0.9	2.3	879	527	1360
India	Almora	2.0	1.2	3.2	164	96	270	0.3	0.2	0.4	14	8	21
India	Alwar	2.6	1.6	3.9	1220	736	1820	0.6	0.4	0.9	309	195	455
India	Ambala	1.9	1.1	3.0	233	138	357	0.4	0.2	0.6	41	24	62
India	Ambedkar Nagar	4.6	2.8	7.1	1370	833	2100	1.6	0.9	2.4	444	269	669
India	Amethi	4.4	2.8	7.0	1480	919	2330	1.4	0.9	2.3	454	278	733
India	Amravati	0.9	0.6	1.5	251	162	389	0.2	0.1	0.3	43	26	63
India	Amreli	1.6	1.0	2.6	245	147	395	0.3	0.2	0.5	43	25	64
India	Amritsar	2.1	1.2	3.1	465	259	697	0.5	0.3	0.8	102	61	165
India	Amroha	4.4	2.6	6.9	1120	675	1770	1.4	0.8	2.4	388	221	650
India	Anand	1.4	0.9	2.4	376	237	631	0.4	0.2	0.6	87	51	137
India	Anantapur	2.2	1.4	3.3	867	554	1280	0.5	0.3	0.7	167	110	245
India	Anantnag	1.2	0.7	1.9	113	67	182	0.3	0.2	0.4	29	16	48
India	Angul	3.2	2.1	4.9	417	272	630	0.8	0.5	1.2	96	57	147
India	Anjaw	2.4	1.4	3.6	6	4	9	0.4	0.2	0.6	1	1	2
India	Anuppur	2.9	1.8	4.5	277	169	428	0.5	0.3	0.8	53	33	81
India	Araria	3.6	2.2	5.4	1350	839	2030	1.5	0.9	2.4	684	409	1050
India	Ariyalur	1.0	0.6	1.6	67	40	110	0.1	0.1	0.2	8	5	12
India	Arvalli	1.5	0.9	2.3	198	121	304	0.3	0.2	0.5	43	26	69
India	Arwal	4.0	2.4	6.2	282	168	437	1.6	1.0	2.6	119	75	186
India	Ashoknagar	3.3	1.9	5.0	367	214	559	0.6	0.4	1.0	76	46	126
India	Auraiya	4.5	2.6	7.1	848	488	1320	1.5	0.9	2.3	255	151	397
India	Aurangabad	3.5	2.1	5.5	1060	636	1660	1.6	0.9	2.5	533	317	829
India	Aurangabad	0.9	0.5	1.4	333	194	517	0.2	0.1	0.3	83	50	127
India	Azamgarh	4.4	2.7	7.1	2610	1640	4280	1.6	1.0	2.4	888	555	1360
India	Badgam	1.1	0.6	1.9	103	55	179	0.3	0.1	0.4	22	12	35
India	Bagalkot	1.1	0.7	1.7	222	140	349	0.2	0.1	0.3	43	27	64
India	Bageshwar	2.1	1.2	3.4	69	40	113	0.3	0.2	0.4	7	4	10
India	Baghpat	4.5	2.5	6.7	757	426	1120	1.5	0.9	2.3	219	131	339
India	Bahraich	4.6	2.6	7.1	1480	820	2280	2.1	1.3	3.2	735	461	1140
India	Baksa	3.4	2.0	5.4	399	235	638	1.1	0.7	1.6	124	79	189
India	Balaghat	2.8	1.8	4.2	503	312	746	0.5	0.3	0.8	91	54	138
India	Balangir	3.2	1.9	5.0	565	339	872	0.8	0.5	1.3	158	93	244
India	Baleshwar	2.7	1.7	3.9	604	379	863	0.7	0.5	1.2	162	102	261
India	Ballary	1.1	0.7	1.6	283	182	430	0.2	0.2	0.4	65	41	102
India	Ballia	4.1	2.4	6.5	1640	967	2600	1.4	0.9	2.1	536	328	791
India	Balod	1.9	1.2	2.9	167	106	254	0.5	0.3	0.8	46	29	76
India	Baloda Bazar	2.1	1.3	3.1	275	170	407	0.5	0.3	0.8	91	55	136
India	Balrampur	4.6	2.9	7.3	1350	852	2160	2.1	1.2	3.2	676	404	1040
India	Balrampur	2.1	1.3	3.1	189	117	286	0.6	0.3	0.9	57	34	91
India	Banaskantha	1.5	0.9	2.3	561	336	829	0.3	0.2	0.5	138	87	208
India	Banda	4.7	3.1	6.9	1180	774	1740	1.4	0.8	2.2	338	206	530
India	Bandipore	1.2	0.7	2.0	39	22	63	0.3	0.2	0.4	8	5	13
India	Bangalore	0.9	0.5	1.5	586	355	966	0.2	0.1	0.4	202	114	337
India	Bangalore Rural	0.9	0.6	1.4	85	53	133	0.2	0.1	0.4	23	13	38
India	Banka	3.4	2.1	5.3	982	612	1520	1.6	1.0	2.5	507	311	783
India	Bankura	1.2	0.8	1.8	438	273	653	0.3	0.2	0.4	78	47	121
India	Banswara	2.2	1.3	3.4	542	329	834	0.6	0.3	1.0	172	97	279
India	Barabanki	4.5	2.8	6.9	2270	1380	3480	1.6	0.9	2.5	787	469	1250
India	Baramulla	1.2	0.7	1.9	133	77	218	0.3	0.2	0.4	26	15	39
India	Baran	2.8	1.7	4.1	394	245	587	0.6	0.4	0.9	86	54	135
India	Bareilly	4.6	2.8	7.0	2570	1570	3910	1.6	1.0	2.7	964	563	1560
India	Bargarh	3.4	2.1	5.1	475	289	723	0.8	0.5	1.3	106	68	165
India	Barmer	2.5	1.6	3.5	895	575	1260	0.6	0.4	0.9	273	171	400
India	Barnala	2.1	1.2	3.3	125	71	191	0.5	0.3	0.8	26	15	43

		2000 mortality						2017 mortality					
Country	Unit	mean rate (per 10,000)	lower rate (per 10,000)	upper rate (per 10,000)	mean count	lower count	upper count	mean rate (per 10,000)	lower rate (per 10,000)	upper rate (per 10,000)	mean count	lower count	upper count
India	Barpeta	3.5	2.1	5.4	662	392	1010	1.1	0.7	1.8	238	144	391
India	Barwani	2.5	1.5	3.9	498	300	756	0.6	0.4	0.9	150	95	228
India	Bastar	1.9	1.2	3.0	181	114	293	0.5	0.3	0.8	52	33	79
India	Basti	4.5	2.7	7.2	1300	771	2070	1.8	1.1	2.9	504	312	803
India	Bathinda	2.2	1.3	3.3	290	170	440	0.5	0.3	0.8	63	37	101
India	Begusarai	3.6	2.1	5.9	1390	812	2280	1.6	0.9	2.4	686	393	1030
India	Belagavi	1.0	0.6	1.5	508	317	791	0.2	0.1	0.3	97	59	159
India	Bemetara	2.0	1.3	2.9	163	103	240	0.5	0.3	0.8	45	27	69
India	Betul	2.6	1.7	4.0	469	300	717	0.5	0.3	0.8	92	54	139
India	Bhadradri Kothagudem	1.9	1.2	2.9	259	159	392	0.3	0.2	0.4	29	18	43
India	Bhadrak	2.8	1.7	4.0	415	249	600	0.7	0.4	1.2	107	63	169
India	Bhagalpur	3.3	2.1	4.8	1140	710	1650	1.5	0.9	2.3	581	349	886
India	Bhandara	1.0	0.6	1.6	128	81	204	0.2	0.1	0.3	19	12	29
India	Bharatpur	2.6	1.5	3.9	865	517	1320	0.7	0.4	1.1	238	152	389
India	Bharuch	1.4	0.9	2.3	213	135	340	0.3	0.2	0.5	46	28	70
India	Bhavnagar	1.5	0.9	2.4	354	217	563	0.3	0.2	0.5	77	44	115
India	Bhilwara	2.5	1.6	3.9	741	460	1140	0.6	0.3	0.9	164	99	253
India	Bhind	3.1	1.9	5.0	594	363	961	0.6	0.4	1.0	129	78	203
India	Bhiwani	2.6	1.6	3.9	331	212	508	0.5	0.3	0.8	61	38	95
India	Bhojpur	3.8	2.3	6.1	1180	698	1890	1.6	1.0	2.4	516	320	770
India	Bhopal	2.9	1.8	4.3	630	384	949	0.6	0.3	0.9	153	94	244
India	Bid	0.9	0.5	1.4	245	145	376	0.2	0.1	0.3	52	31	78
India	Bidar	0.9	0.5	1.3	154	90	234	0.2	0.1	0.4	37	22	56
India	Bijapur	1.9	1.3	3.1	68	44	108	0.5	0.3	0.8	18	11	27
India	Bijnor	4.3	2.5	6.4	2160	1270	3180	1.5	0.9	2.4	704	416	1150
India	Bikaner	2.5	1.7	3.8	657	437	993	0.6	0.4	0.8	203	135	297
India	Bilaspur	1.4	0.8	2.2	46	28	75	0.3	0.2	0.4	9	5	13
India	Bilaspur	2.1	1.3	3.1	530	324	788	0.5	0.3	0.8	127	83	197
India	Birbhum	1.2	0.7	1.8	428	262	656	0.3	0.2	0.4	87	56	139
India	Bishnupur	1.7	1.0	2.6	41	25	62	0.4	0.3	0.7	10	6	15
India	Biswanath	3.3	2.0	5.0	204	123	309	1.1	0.7	1.7	71	44	109
India	Bokaro	2.4	1.5	3.7	560	341	865	0.8	0.5	1.2	171	97	263
India	Bongaigaon	3.2	1.9	5.0	218	128	339	1.1	0.6	1.7	80	48	130
India	Botad	1.6	1.0	2.6	92	58	151	0.3	0.2	0.5	20	12	30
India	Boudh	3.5	2.1	5.3	181	108	273	0.8	0.5	1.3	45	28	71
India	Budaun	4.8	2.9	7.2	2240	1370	3380	1.6	1.0	2.5	777	479	1190
India	Bulandshahr	4.5	2.7	7.0	2270	1370	3540	1.6	1.0	2.4	740	455	1160
India	Buldana	0.9	0.6	1.5	246	157	385	0.2	0.1	0.3	49	30	76
India	Bundi	2.6	1.6	3.8	360	216	520	0.6	0.3	0.9	74	41	119
India	Burhanpur	2.5	1.5	4.0	255	154	402	0.6	0.4	0.9	68	40	103
India	Buxar	4.5	2.7	7.3	889	545	1450	1.6	0.9	2.5	337	194	514
India	Cachar	3.0	1.9	4.7	577	356	898	1.1	0.7	1.8	235	147	381
India	Central	1.5	0.9	2.3	67	38	100	0.4	0.2	0.6	12	7	19
India	Chamba	1.5	0.9	2.4	93	57	147	0.3	0.2	0.5	15	9	24
India	Chamoli	2.0	1.3	3.2	102	65	159	0.3	0.2	0.5	10	6	16
India	Champawat	2.1	1.3	3.3	55	33	87	0.3	0.2	0.4	6	3	9
India	Champhai	0.8	0.5	1.3	11	7	17	0.3	0.1	0.4	4	2	5
India	Chamrajnagar	1.0	0.7	1.6	94	60	146	0.2	0.1	0.4	16	9	26
India	Chandauli	4.9	2.8	7.5	1270	735	1950	1.5	0.9	2.3	377	234	586
India	Chandel	1.9	1.2	2.8	20	12	30	0.5	0.3	0.8	5	3	9
India	Chandigarh	0.6	0.3	0.9	55	32	85	0.2	0.1	0.3	14	8	21
India	Chandrapur	0.9	0.6	1.4	192	121	298	0.2	0.1	0.3	29	18	47
India	Changlang	2.3	1.4	3.4	46	28	68	0.4	0.2	0.7	9	5	15
India	Charaideo	2.9	1.8	4.4	94	57	141	1.1	0.6	1.7	31	18	50
India	Charkhi Dadri	2.5	1.6	3.8	130	82	199	0.5	0.3	0.7	24	14	37
India	Chatra	2.9	1.7	4.3	394	239	595	1.0	0.6	1.5	145	89	225
India	Chennai	0.9	0.5	1.4	324	195	522	0.1	0.1	0.2	42	25	68
India	Chhatarpur	3.3	2.1	4.7	744	486	1070	0.6	0.4	1.0	160	102	246
India	Chhindwara	2.6	1.6	4.0	618	384	946	0.5	0.3	0.8	120	74	196

Table 3: Diarrhoea Mortality by Unit *(continued)*

Country	Unit	2000 mortality mean rate (per 10,000)	lower rate (per 10,000)	upper rate (per 10,000)	mean count	lower count	upper count	2017 mortality mean rate (per 10,000)	lower rate (per 10,000)	upper rate (per 10,000)	mean count	lower count	upper count
India	Chhotaudepur	1.4	0.9	2.2	156	97	242	0.4	0.2	0.5	36	22	55
India	Chikballapura	0.9	0.6	1.4	97	60	151	0.2	0.1	0.4	20	12	33
India	Chikmagalur	1.1	0.7	1.7	113	74	172	0.2	0.1	0.3	15	10	23
India	Chirang	3.4	2.0	5.0	261	156	391	1.1	0.7	1.8	85	51	143
India	Chitradurga	1.1	0.7	1.7	172	106	261	0.2	0.1	0.3	30	20	45
India	Chitrakoot	4.3	2.8	6.4	662	426	985	1.4	0.8	2.2	211	129	339
India	Chittaurgarh	2.5	1.6	3.7	460	291	677	0.6	0.3	0.9	100	60	154
India	Chittoor	2.0	1.3	3.0	804	511	1230	0.4	0.3	0.7	159	102	233
India	Churachandpur	1.9	1.2	2.8	47	30	73	0.5	0.3	0.7	13	8	20
India	Churu	2.6	1.6	4.1	727	455	1150	0.6	0.4	0.9	141	87	217
India	Coimbatore	1.0	0.6	1.5	282	168	443	0.1	0.1	0.2	34	21	52
India	Cuddalore	1.0	0.6	1.6	246	156	407	0.1	0.1	0.2	31	19	48
India	Cuttack	3.1	2.0	4.6	779	496	1160	0.8	0.4	1.2	182	107	280
India	Dadra and Nagar Haveli	0.7	0.4	1.1	33	20	49	0.4	0.2	0.6	20	12	31
India	Dakshin Dinajpur	1.1	0.7	1.8	174	109	281	0.2	0.1	0.4	32	20	51
India	Dakshina Kannada	0.9	0.6	1.4	164	99	255	0.2	0.1	0.3	26	17	41
India	Daman	0.7	0.4	1.1	7	4	11	0.3	0.2	0.5	4	3	6
India	Damoh	3.1	2.0	4.7	484	316	731	0.6	0.4	1.0	102	64	163
India	Dang	1.2	0.8	1.9	37	23	58	0.3	0.2	0.5	10	6	17
India	Dantewada	1.9	1.2	3.0	62	39	98	0.5	0.3	0.8	17	10	27
India	Darbhanga	3.8	2.3	6.7	1860	1100	3240	1.6	1.0	2.5	821	514	1260
India	Darjiling	1.5	0.9	2.2	199	125	300	0.3	0.2	0.4	31	19	47
India	Darrang	3.6	2.0	5.7	479	269	746	1.2	0.7	1.9	173	106	277
India	Datia	3.2	1.9	4.8	274	167	416	0.6	0.4	0.9	57	35	88
India	Dausa	2.5	1.7	3.8	561	366	848	0.6	0.4	1.0	149	88	233
India	Davanagere	1.2	0.7	1.9	222	139	350	0.2	0.1	0.3	36	24	56
India	Dehradun	1.9	1.2	3.1	285	176	464	0.3	0.2	0.4	41	23	61
India	Deogarh	3.3	2.0	5.0	107	66	162	0.8	0.5	1.3	26	16	42
India	Deoghar	2.4	1.4	3.8	446	266	718	0.9	0.5	1.4	177	106	289
India	Deoria	4.5	2.8	6.9	1760	1070	2680	1.4	0.8	2.2	506	294	795
India	Devbhumi Dwarka	1.5	0.9	2.3	121	72	189	0.3	0.2	0.4	22	13	33
India	Dewas	3.0	1.8	4.3	526	319	761	0.6	0.4	0.9	114	70	177
India	Dhalai	1.1	0.7	1.8	42	25	66	0.5	0.3	0.8	22	13	33
India	Dhamtari	2.0	1.2	3.0	173	109	262	0.5	0.3	0.8	45	26	73
India	Dhanbad	2.6	1.4	4.1	807	448	1290	0.8	0.5	1.3	230	130	363
India	Dhar	2.9	1.8	4.5	773	475	1210	0.6	0.4	0.9	200	125	308
India	Dharmapuri	1.1	0.6	1.6	151	90	232	0.1	0.1	0.2	18	11	29
India	Dharwad	1.1	0.7	1.7	185	119	292	0.2	0.1	0.3	34	20	55
India	Dhaulpur	2.7	1.6	4.2	470	276	734	0.7	0.4	1.0	124	75	195
India	Dhemaji	2.7	1.6	4.1	179	108	269	1.0	0.6	1.6	74	46	115
India	Dhenkanal	3.2	2.0	4.8	406	262	619	0.7	0.4	1.1	90	49	132
India	Dhubri	3.0	1.8	4.6	397	245	616	1.1	0.6	1.6	172	105	265
India	Dhule	1.0	0.6	1.5	208	124	311	0.2	0.1	0.3	41	25	59
India	Dibang Valley	2.8	1.7	4.2	3	2	4	0.5	0.3	0.7	0	0	1
India	Dibrugarh	3.0	1.7	4.7	404	235	633	1.1	0.6	1.6	134	79	208
India	Dima Hasao	4.0	2.5	6.0	128	79	191	1.3	0.8	2.0	42	26	65
India	Dimapur	1.1	0.6	1.7	36	22	55	0.4	0.2	0.6	14	8	22
India	Dindigul	1.0	0.6	1.7	205	121	329	0.1	0.1	0.2	24	14	37
India	Dindori	2.8	1.7	4.2	292	176	441	0.6	0.4	0.9	64	41	98
India	Diu	0.9	0.5	1.5	2	1	4	0.3	0.2	0.5	1	0	1
India	Doda	1.2	0.7	1.9	59	36	92	0.3	0.2	0.5	14	9	22
India	Dohad	1.5	0.9	2.4	404	242	640	0.4	0.2	0.6	116	67	187
India	Dumka	2.3	1.4	3.5	376	226	580	0.8	0.5	1.4	130	77	217
India	Dungarpur	2.1	1.2	3.3	381	222	598	0.6	0.3	0.9	119	67	190
India	Durg	2.0	1.2	3.0	351	218	540	0.5	0.3	0.8	100	61	158
India	East	1.6	0.9	2.5	167	95	258	0.4	0.2	0.6	39	23	64
India	East Garo Hills	1.9	1.1	2.8	41	24	61	0.6	0.4	1.1	14	10	25

Table 3: Diarrhoea Mortality by Unit *(continued)*

Country	Unit	2000 mortality mean rate (per 10,000)	lower rate (per 10,000)	upper rate (per 10,000)	mean count	lower count	upper count	2017 mortality mean rate (per 10,000)	lower rate (per 10,000)	upper rate (per 10,000)	mean count	lower count	upper count
India	East Godavari	1.8	1.1	3.0	916	546	1480	0.4	0.3	0.6	166	103	245
India	East Jaintia Hills	1.8	1.1	2.9	31	18	48	0.7	0.4	1.1	13	8	20
India	East Kameng	2.9	1.7	4.6	19	11	30	0.4	0.2	0.6	4	2	6
India	East Khasi Hills	1.7	1.0	2.7	137	83	221	0.7	0.4	1.0	54	30	84
India	East Nimar	2.7	1.6	4.1	421	248	634	0.6	0.4	0.9	106	64	159
India	East Siang	2.1	1.2	3.2	15	9	23	0.4	0.2	0.6	3	2	5
India	East Sikkim	1.1	0.7	1.7	27	17	41	0.2	0.2	0.4	5	3	8
India	Ernakulam	0.1	0.0	0.1	15	8	23	0.0	0.0	0.0	2	1	4
India	Erode	1.1	0.7	1.7	164	100	250	0.1	0.1	0.2	17	10	27
India	Etah	4.4	2.5	7.1	1120	639	1820	1.6	0.9	2.5	382	216	600
India	Etawah	4.7	2.8	7.4	909	542	1440	1.5	0.9	2.5	289	162	469
India	Faizabad	4.4	2.6	6.7	1170	705	1790	1.5	0.9	2.3	397	244	596
India	Faridabad	2.5	1.5	3.8	587	353	876	0.5	0.3	0.9	149	89	239
India	Faridkot	2.1	1.2	3.1	122	71	182	0.5	0.3	0.8	25	15	40
India	Farrukhabad	4.9	2.8	7.8	1250	719	1970	1.7	1.0	2.6	412	250	637
India	Fatehabad	2.1	1.3	3.3	195	119	303	0.4	0.3	0.7	38	24	61
India	Fatehgarh Sahib	1.7	1.0	2.6	96	56	150	0.4	0.2	0.6	20	12	29
India	Fatehpur	4.6	2.9	7.1	1620	1040	2500	1.4	0.9	2.3	456	276	734
India	Fazilka	2.2	1.4	3.4	210	129	317	0.5	0.3	0.8	47	29	74
India	Firozabad	4.7	2.7	7.6	1500	880	2450	1.7	0.9	2.6	530	301	829
India	Firozpur	2.2	1.3	3.3	219	127	332	0.5	0.3	0.8	47	30	74
India	Gadag	1.1	0.7	1.8	129	88	205	0.2	0.1	0.3	24	15	35
India	Gadchiroli	1.0	0.6	1.5	97	63	148	0.2	0.1	0.3	16	10	24
India	Gajapati	3.2	2.1	5.1	231	149	367	0.9	0.6	1.5	64	39	107
India	Ganderbal	1.1	0.6	1.9	60	35	104	0.3	0.1	0.4	13	7	19
India	Gandhinagar	1.4	0.8	2.2	167	100	261	0.3	0.2	0.5	35	21	53
India	Ganganagar	2.8	1.8	4.1	645	421	941	0.6	0.4	0.9	124	81	186
India	Ganjam	3.1	1.9	5.1	1100	664	1790	0.8	0.5	1.3	272	157	432
India	Garhwa	3.4	2.1	5.1	575	356	849	0.9	0.5	1.5	168	95	278
India	Gariaband	2.0	1.3	3.0	124	80	187	0.5	0.3	0.8	43	26	65
India	Gautam Buddha Nagar	4.2	2.5	6.4	1290	759	1950	1.3	0.8	2.1	529	320	852
India	Gaya	3.6	2.3	5.8	1920	1190	3040	1.7	1.0	2.6	991	609	1540
India	Ghaziabad	4.4	2.4	6.5	1310	720	1940	1.3	0.8	2.1	489	294	787
India	Ghazipur	4.6	2.7	7.3	2120	1260	3380	1.5	0.9	2.2	656	406	1000
India	Gir Somnath	1.5	0.9	2.4	164	96	263	0.3	0.2	0.5	30	18	46
India	Giridih	2.7	1.5	4.5	828	461	1390	0.9	0.6	1.4	310	194	490
India	Goalpara	3.4	2.0	5.2	365	219	561	1.1	0.7	1.8	140	88	221
India	Godda	2.7	1.7	4.0	453	287	675	0.9	0.5	1.4	150	85	240
India	Golaghat	3.2	2.0	4.8	349	217	527	1.1	0.7	1.7	120	73	182
India	Gomati	1.1	0.6	1.8	46	26	75	0.5	0.3	0.8	21	13	32
India	Gonda	4.6	2.8	7.4	2080	1240	3290	2.0	1.2	3.0	947	561	1450
India	Gondiya	1.0	0.6	1.5	103	63	160	0.2	0.1	0.3	16	10	27
India	Gopalganj	4.8	2.7	7.7	1430	816	2320	1.7	1.0	2.7	512	298	813
India	Gorakhpur	4.5	2.6	6.9	2190	1270	3400	1.6	1.0	2.8	770	459	1290
India	Gumla	2.7	1.7	4.1	345	210	520	0.8	0.5	1.3	106	62	168
India	Guna	3.2	2.0	4.8	455	280	677	0.6	0.4	0.9	102	65	156
India	Guntur	1.9	1.2	3.0	903	560	1390	0.4	0.3	0.7	174	106	282
India	Gurdaspur	2.1	1.2	3.2	330	184	491	0.5	0.3	0.8	66	42	102
India	Gurugram	2.4	1.4	3.7	273	162	416	0.5	0.3	0.8	103	62	160
India	Gwalior	3.1	1.8	4.7	614	354	946	0.6	0.3	0.9	140	82	208
India	Hailakandi	3.0	1.8	4.9	232	140	374	1.1	0.7	1.8	97	61	157
India	Hamirpur	1.5	0.9	2.3	63	37	97	0.3	0.2	0.4	9	6	14
India	Hamirpur	4.5	3.0	6.7	692	455	1030	1.4	0.9	2.2	183	115	288
India	Hanumangarh	2.8	1.7	4.1	525	319	779	0.6	0.4	0.8	108	69	156
India	Haora	1.2	0.7	1.8	432	246	646	0.3	0.1	0.4	79	47	123
India	Hapur	4.6	2.7	7.1	698	410	1070	1.5	0.9	2.5	295	169	475
India	Harda	3.0	1.8	4.6	196	120	304	0.6	0.4	0.9	45	28	67
India	Hardoi	4.7	2.8	7.1	2600	1570	3960	1.7	1.0	2.6	936	555	1460
India	Hardwar	2.1	1.2	3.1	470	275	701	0.4	0.2	0.5	77	45	117

Table 3: Diarrhoea Mortality by Unit *(continued)*

Country	Unit	2000 mortality mean rate (per 10,000)	lower rate (per 10,000)	upper rate (per 10,000)	mean count	lower count	upper count	2017 mortality mean rate (per 10,000)	lower rate (per 10,000)	upper rate (per 10,000)	mean count	lower count	upper count
India	Hassan	1.1	0.7	1.6	167	105	250	0.2	0.1	0.3	24	14	37
India	Hathras	4.2	2.4	6.8	892	507	1450	1.5	0.9	2.5	313	182	506
India	Haveri	1.1	0.7	1.7	160	98	245	0.2	0.1	0.4	29	18	46
India	Hazaribagh	2.7	1.6	4.3	586	359	936	0.9	0.6	1.4	211	131	325
India	Hingoli	0.9	0.5	1.4	121	71	183	0.2	0.1	0.3	26	16	40
India	Hisar	2.5	1.5	3.7	444	276	662	0.5	0.3	0.8	82	53	127
India	Hojai	3.2	2.0	4.9	118	72	183	1.2	0.7	1.9	52	31	82
India	Hoshangabad	3.0	1.9	4.5	403	252	618	0.6	0.4	0.9	80	50	126
India	Hoshiarpur	2.0	1.2	3.2	319	183	512	0.5	0.3	0.7	60	39	90
India	Hugli	1.2	0.7	1.7	529	298	755	0.2	0.1	0.4	88	52	137
India	Hyderabad	2.1	1.1	3.3	1000	551	1590	0.3	0.2	0.5	112	59	169
India	Idukki	0.1	0.0	0.1	6	4	10	0.0	0.0	0.0	1	1	1
India	Imphal East	1.6	1.0	2.5	54	33	82	0.4	0.3	0.7	15	9	23
India	Imphal West	1.7	1.0	2.5	87	53	133	0.4	0.3	0.7	23	14	37
India	Indore	2.8	1.7	4.2	856	507	1270	0.6	0.4	0.9	231	138	357
India	Jabalpur	2.9	1.8	4.3	738	467	1110	0.5	0.3	0.9	140	86	226
India	Jagatsinghapur	3.0	1.8	4.4	300	182	436	0.7	0.4	1.1	60	37	96
India	Jagitial	1.9	1.2	2.9	173	110	266	0.3	0.2	0.5	22	12	33
India	Jaipur	2.7	1.6	4.1	1900	1160	2930	0.6	0.4	0.9	455	291	692
India	Jaisalmer	3.0	2.0	4.4	284	183	408	0.6	0.4	0.8	68	43	97
India	Jajapur	2.9	1.7	4.1	532	321	750	0.7	0.4	1.1	128	74	198
India	Jalandhar	2.0	1.1	3.3	394	222	632	0.5	0.3	0.7	81	49	122
India	Jalaun	4.6	3.0	7.2	946	604	1470	1.4	0.9	2.3	262	164	431
India	Jalgaon	1.0	0.6	1.5	422	247	662	0.2	0.1	0.3	80	50	122
India	Jalna	0.9	0.5	1.4	195	117	307	0.2	0.1	0.3	46	26	70
India	Jalor	2.3	1.4	3.3	540	331	785	0.6	0.4	1.0	170	103	269
India	Jalpaiguri	1.1	0.7	1.7	268	166	415	0.2	0.1	0.3	48	30	71
India	Jammu	1.2	0.7	1.9	141	85	214	0.3	0.2	0.4	24	14	37
India	Jamnagar	1.5	0.9	2.4	211	123	331	0.3	0.2	0.5	37	23	58
India	Jamtara	2.3	1.4	3.7	220	132	352	0.8	0.5	1.4	80	46	134
India	Jamui	3.9	2.3	6.2	835	503	1350	1.7	1.0	2.7	406	244	639
India	Jangoan	1.9	1.1	2.9	109	65	166	0.3	0.2	0.5	12	7	19
India	Janjgir-Champa	2.2	1.3	3.5	384	232	620	0.5	0.3	0.8	104	65	163
India	Jashpur	2.0	1.3	3.0	199	130	298	0.5	0.3	0.8	53	30	81
India	Jaunpur	4.3	2.6	6.7	2530	1540	3880	1.5	1.0	2.3	799	518	1210
India	Jayashankar Bhupalapal	1.9	1.1	2.9	126	73	199	0.3	0.2	0.5	15	9	22
India	Jehanabad	3.4	2.1	5.4	481	300	770	1.6	0.9	2.4	235	141	365
India	Jhabua	2.7	1.6	4.3	391	233	630	0.6	0.4	1.0	119	68	192
India	Jhajjar	2.5	1.5	3.8	267	161	399	0.5	0.3	0.8	48	28	77
India	Jhalawar	2.7	1.7	4.0	438	274	656	0.6	0.3	0.9	97	58	147
India	Jhansi	4.2	2.6	6.3	988	599	1470	1.3	0.8	2.0	276	171	421
India	Jhargram	1.4	0.8	2.0	147	87	219	0.3	0.2	0.4	26	16	42
India	Jharsuguda	3.1	1.9	4.8	173	107	264	0.8	0.5	1.3	42	25	67
India	Jhunjhunun	2.6	1.6	4.0	672	414	1030	0.6	0.3	0.9	131	77	202
India	Jind	2.5	1.6	4.0	360	222	561	0.5	0.3	0.8	63	39	100
India	Jiribam	1.6	1.0	2.6	7	4	12	0.4	0.3	0.7	2	1	3
India	Jodhpur	2.7	1.7	3.9	1200	765	1760	0.6	0.4	0.9	314	191	462
India	Jogulamba Gadwa	2.2	1.4	3.3	173	111	256	0.3	0.2	0.6	22	14	36
India	Jorhat	2.9	1.7	4.3	247	146	362	1.0	0.6	1.6	78	49	122
India	Junagadh	1.5	0.9	2.3	205	122	310	0.3	0.2	0.4	36	23	53
India	Kabeerdham	2.1	1.3	3.1	198	127	295	0.5	0.3	0.9	74	42	116
India	Kachchh	1.6	1.1	2.3	354	237	503	0.3	0.2	0.5	89	57	131
India	Kaimur	4.5	2.7	6.9	848	513	1300	1.6	0.9	2.5	343	199	528
India	Kaithal	2.2	1.3	3.5	265	159	419	0.4	0.3	0.7	48	28	75
India	Kakching	1.8	1.1	2.8	27	17	42	0.5	0.3	0.7	6	4	10
India	Kalaburgi	1.0	0.6	1.4	264	162	392	0.2	0.1	0.4	66	39	104
India	Kalahandi	3.3	2.0	5.5	601	371	1000	0.9	0.5	1.3	166	99	253
India	Kalimpong	1.3	0.8	2.0	23	14	33	0.2	0.2	0.4	4	2	5

Table 3: Diarrhoea Mortality by Unit *(continued)*

Country	Unit	2000 mortality mean rate (per 10,000)	lower rate (per 10,000)	upper rate (per 10,000)	mean count	lower count	upper count	2017 mortality mean rate (per 10,000)	lower rate (per 10,000)	upper rate (per 10,000)	mean count	lower count	upper count
India	Kamareddy	1.9	1.2	2.7	218	136	315	0.3	0.2	0.5	27	16	43
India	Kamjong	1.8	1.1	2.7	5	3	8	0.5	0.3	0.7	2	1	3
India	Kamle	2.5	1.5	3.8	7	5	11	0.4	0.2	0.6	2	1	3
India	Kamrup	3.6	2.2	5.7	534	322	833	1.2	0.7	1.8	175	108	275
India	Kamrup Metropolitan	3.6	2.1	5.7	314	184	505	1.3	0.7	2.0	117	68	189
India	Kancheepuram	0.9	0.6	1.6	299	176	495	0.1	0.1	0.2	54	32	83
India	Kandhamal	3.3	2.0	5.0	296	177	448	0.9	0.5	1.4	76	48	121
India	Kangpokpi	1.6	1.0	2.5	14	8	21	0.4	0.3	0.7	5	3	8
India	Kangra	1.5	0.9	2.3	226	134	344	0.3	0.2	0.4	33	20	51
India	Kannauj	4.8	2.8	7.5	1050	610	1640	1.6	1.0	2.5	355	211	539
India	Kanniyakumari	1.0	0.6	1.6	165	101	247	0.1	0.1	0.2	19	11	29
India	Kannur	0.1	0.0	0.1	15	9	23	0.0	0.0	0.0	2	1	4
India	Kanpur Dehat	4.6	2.8	7.4	1030	627	1670	1.4	0.8	2.2	281	169	448
India	Kanpur Nagar	4.6	2.8	7.1	2160	1320	3330	1.4	0.8	2.3	561	330	887
India	Kapurthala	2.1	1.1	3.2	159	87	242	0.5	0.3	0.7	31	19	47
India	Karaikal	0.7	0.4	1.2	15	9	26	0.3	0.2	0.5	5	3	8
India	Karauli	2.6	1.5	3.9	487	291	735	0.6	0.4	1.0	128	76	196
India	Karbi Anglong	3.7	2.3	5.5	301	187	442	1.2	0.8	1.9	109	68	163
India	Kargil	1.2	0.8	1.9	16	10	25	0.3	0.2	0.4	3	2	5
India	Karimganj	2.9	1.8	4.5	418	256	662	1.2	0.7	1.8	193	116	308
India	Karimnagar	1.8	1.1	2.8	174	106	272	0.3	0.2	0.5	20	12	31
India	Karnal	2.5	1.5	3.8	409	245	619	0.5	0.3	0.7	76	44	120
India	Karur	1.0	0.6	1.8	93	57	161	0.1	0.1	0.2	11	6	16
India	Kasaragod	0.1	0.0	0.1	8	5	12	0.0	0.0	0.0	1	1	2
India	Kasganj	4.6	2.6	7.1	989	565	1540	1.7	1.0	2.6	352	198	544
India	Kathua	1.2	0.7	1.9	61	36	96	0.3	0.2	0.4	11	7	17
India	Katihar	3.4	2.2	5.2	1370	891	2080	1.5	0.9	2.3	689	430	1030
India	Katni	3.1	2.0	4.6	453	286	662	0.6	0.4	0.9	96	61	150
India	Kaushambi	4.7	3.0	7.4	1900	1200	2970	1.4	0.8	2.2	589	354	898
India	Kendrapara	2.9	1.8	4.3	443	280	652	0.7	0.4	1.1	99	61	153
India	Kendujhar	3.1	2.0	4.6	684	440	998	0.9	0.5	1.4	199	112	317
India	Khagaria	3.4	2.0	5.3	723	424	1120	1.5	0.9	2.3	385	237	580
India	Khammam	1.9	1.1	2.9	278	170	432	0.3	0.2	0.5	33	21	49
India	Khargone	2.6	1.5	3.7	577	335	835	0.6	0.4	0.9	162	103	239
India	Kheda	1.5	0.9	2.3	312	196	492	0.3	0.2	0.6	68	42	109
India	Khordha	3.0	1.9	4.7	498	314	772	0.8	0.5	1.1	128	77	193
India	Khowai	1.1	0.7	1.8	27	16	42	0.5	0.3	0.8	12	7	18
India	Khunti	2.7	1.7	3.9	175	111	256	0.8	0.4	1.3	52	28	83
India	Kinnaur	1.7	1.0	2.6	12	7	19	0.3	0.2	0.5	2	1	3
India	Kiphire	1.1	0.7	1.5	17	11	25	0.4	0.2	0.6	3	2	4
India	Kishanganj	3.2	2.0	4.9	717	446	1090	1.5	0.9	2.3	387	243	604
India	Kishtwar	1.2	0.7	1.9	35	22	57	0.3	0.2	0.4	7	5	11
India	Koch Bihar	1.0	0.6	1.5	288	186	435	0.2	0.1	0.3	58	36	87
India	Kodagu	1.0	0.6	1.5	53	32	82	0.2	0.1	0.3	7	5	11
India	Koderma	2.8	1.6	4.5	186	110	301	0.9	0.6	1.4	72	43	109
India	Kohima	1.1	0.7	1.6	25	16	37	0.4	0.2	0.6	9	6	15
India	Kokrajhar	3.1	1.9	4.8	493	298	756	1.1	0.6	1.7	162	98	257
India	Kolar	0.9	0.5	1.4	118	74	189	0.2	0.1	0.3	25	14	42
India	Kolasib	0.7	0.4	1.1	23	14	36	0.2	0.1	0.4	8	5	13
India	Kolhapur	0.9	0.6	1.4	321	193	483	0.2	0.1	0.3	53	31	94
India	Kolkata	1.3	0.8	1.9	694	418	1050	0.3	0.2	0.4	119	70	186
India	Kollam	0.1	0.0	0.1	13	7	21	0.0	0.0	0.0	2	1	3
India	Kondagaon	1.9	1.2	3.2	129	79	214	0.5	0.3	0.8	38	22	58
India	Koppal	1.1	0.7	1.7	167	105	263	0.2	0.2	0.4	36	23	54
India	Koraput	2.9	1.9	4.7	527	342	851	0.8	0.5	1.4	162	99	262
India	Korba	2.1	1.2	3.2	281	170	441	0.5	0.3	0.8	73	46	115
India	Koriya	2.0	1.3	3.1	172	106	265	0.5	0.3	0.9	43	26	72
India	Kota	2.6	1.5	3.8	508	302	743	0.5	0.3	0.9	116	69	189
India	Kottayam	0.1	0.0	0.1	9	5	16	0.0	0.0	0.0	1	1	2

Table 3: Diarrhoea Mortality by Unit *(continued)*

Country	Unit	2000 mortality mean rate (per 10,000)	lower rate (per 10,000)	upper rate (per 10,000)	mean count	lower count	upper count	2017 mortality mean rate (per 10,000)	lower rate (per 10,000)	upper rate (per 10,000)	mean count	lower count	upper count
India	Kozhikode	0.1	0.0	0.1	16	10	26	0.0	0.0	0.0	3	2	5
India	Kra Daddi	2.3	1.3	3.7	6	3	10	0.4	0.2	0.6	3	2	5
India	Krishna	1.9	1.2	2.8	716	468	1060	0.4	0.3	0.6	130	80	200
India	Krishnagiri	1.1	0.7	1.7	190	119	298	0.1	0.1	0.2	24	14	37
India	Kulgam	1.2	0.7	2.0	79	48	129	0.3	0.1	0.4	14	8	23
India	Kullu	1.6	1.0	2.5	63	37	98	0.3	0.2	0.5	10	6	15
India	Kumuram Bheem Asifabad	1.8	1.1	2.9	107	64	171	0.3	0.2	0.5	14	8	24
India	Kupwara	1.2	0.7	1.9	117	69	195	0.3	0.2	0.4	30	17	47
India	Kurnool	2.2	1.4	3.1	924	616	1330	0.5	0.3	0.7	192	125	298
India	Kurukshetra	2.0	1.2	3.1	208	124	320	0.4	0.2	0.6	37	23	57
India	Kurung Kumey	2.6	1.5	4.1	7	4	10	0.4	0.2	0.7	4	2	6
India	Kushinagar	4.6	2.7	7.1	2130	1270	3290	1.6	0.9	2.6	763	456	1260
India	Lahul & Spiti	1.6	1.0	2.5	5	3	8	0.3	0.2	0.5	1	0	1
India	Lakhimpur	2.9	1.8	4.3	312	192	460	1.0	0.6	1.6	117	72	179
India	Lakhimpur Kheri	4.4	2.8	6.7	2280	1430	3440	1.6	1.0	2.4	911	584	1340
India	Lakhisarai	3.3	2.0	5.6	411	246	683	1.6	0.9	2.4	215	121	322
India	Lakshadweep	1.0	0.6	1.4	1	1	2	NA	NA	NA	NA	NA	NA
India	Lalitpur	4.2	2.5	6.7	747	438	1180	1.4	0.9	2.2	259	165	416
India	Latehar	2.9	1.8	4.4	289	175	434	0.9	0.6	1.4	100	61	155
India	Latur	0.9	0.6	1.4	222	136	326	0.2	0.1	0.3	45	28	74
India	Lawangtlai	0.8	0.5	1.2	9	6	13	0.2	0.1	0.4	3	2	5
India	Leh (Ladakh)	1.2	0.8	1.8	10	7	15	0.3	0.2	0.4	2	1	3
India	Lohardaga	2.8	1.7	4.3	146	89	228	0.8	0.5	1.3	48	28	77
India	Lohit	2.1	1.3	3.2	12	7	18	0.4	0.2	0.6	2	1	4
India	Longding	2.4	1.5	3.6	18	11	27	0.4	0.3	0.7	3	2	5
India	Longleng	1.1	0.6	1.6	20	12	30	0.4	0.2	0.6	2	1	3
India	Lower Dibang Valley	2.2	1.3	3.2	14	8	20	0.4	0.2	0.6	2	1	4
India	Lower Siang	2.0	1.2	3.2	4	3	7	0.4	0.2	0.6	1	1	1
India	Lower Subansiri	2.5	1.5	3.9	11	7	18	0.4	0.3	0.7	4	2	6
India	Lucknow	4.3	2.6	6.5	1950	1180	2950	1.4	0.9	2.2	676	413	1040
India	Ludhiana	1.7	1.0	2.7	531	316	845	0.4	0.3	0.6	119	72	180
India	Lunglei	0.8	0.5	1.2	12	8	18	0.2	0.1	0.3	3	2	5
India	Madhepura	4.1	2.5	6.3	1030	642	1590	1.6	1.0	2.6	501	305	782
India	Madhubani	4.1	2.4	6.9	2040	1200	3430	1.7	1.0	2.6	908	559	1420
India	Madurai	1.0	0.6	1.6	265	145	406	0.1	0.1	0.2	35	20	55
India	Maharajganj	4.4	2.7	6.9	1720	1060	2660	1.7	1.0	2.8	694	412	1110
India	Mahasamund	2.1	1.3	3.1	219	134	330	0.5	0.3	0.8	60	37	94
India	Mahbubnagar	2.1	1.3	3.2	392	246	594	0.3	0.2	0.5	53	32	85
India	Mahe	0.6	0.4	0.9	13	8	20	0.2	0.1	0.4	3	2	4
India	Mahendragarh	2.5	1.5	3.7	216	128	315	0.5	0.3	0.8	40	24	61
India	Mahesana	1.5	0.9	2.4	301	184	474	0.3	0.2	0.5	58	35	91
India	Mahisagar	1.5	0.9	2.4	195	113	311	0.3	0.2	0.5	44	26	69
India	Mahoba	4.3	2.7	6.5	485	308	729	1.4	0.8	2.0	143	87	210
India	Mahuababad	1.9	1.2	3.1	146	88	230	0.3	0.2	0.5	17	10	26
India	Mainpuri	4.5	2.5	7.3	1160	651	1880	1.6	1.0	2.6	390	236	620
India	Majuli	3.1	1.9	4.6	70	42	103	1.1	0.7	1.7	22	14	34
India	Malappuram	0.1	0.0	0.1	27	17	45	0.0	0.0	0.0	5	3	8
India	Maldah	1.3	0.8	2.0	534	347	847	0.3	0.2	0.4	112	71	176
India	Malkangiri	2.9	1.8	4.2	241	153	353	0.8	0.5	1.2	76	51	115
India	Mamit	0.8	0.5	1.2	8	5	12	0.2	0.1	0.4	3	2	4
India	Mancherial	1.8	1.1	2.9	141	83	225	0.3	0.2	0.5	18	10	28
India	Mandi	1.5	1.0	2.3	152	97	237	0.3	0.2	0.4	22	14	35
India	Mandla	2.9	1.8	4.6	301	191	475	0.5	0.3	0.8	58	36	90
India	Mandsaur	3.0	1.7	4.6	425	241	651	0.6	0.4	0.9	80	51	131
India	Mandya	1.1	0.6	1.7	162	96	260	0.2	0.1	0.4	26	15	42
India	Mansa	2.1	1.2	3.3	136	81	210	0.5	0.3	0.8	28	17	44
India	Mathura	4.4	2.6	6.8	1600	942	2450	1.6	1.0	2.5	577	365	922
India	Mau	4.4	2.8	6.9	1160	718	1800	1.5	0.9	2.2	372	224	574

Table 3: Diarrhoea Mortality by Unit *(continued)*

Country	Unit	2000 mortality mean rate (per 10,000)	lower rate (per 10,000)	upper rate (per 10,000)	mean count	lower count	upper count	2017 mortality mean rate (per 10,000)	lower rate (per 10,000)	upper rate (per 10,000)	mean count	lower count	upper count
India	Mayurbhanj	3.0	1.9	4.2	884	564	1240	0.9	0.5	1.5	255	155	422
India	Medak	2.0	1.3	3.1	186	116	280	0.3	0.2	0.5	24	13	37
India	Medchal Malkajgiri	2.0	1.1	3.3	366	206	591	0.3	0.2	0.5	69	36	107
India	Meerut	4.7	2.7	7.1	2140	1250	3260	1.5	0.8	2.5	639	363	1070
India	Mewat	2.5	1.5	3.9	339	209	540	0.6	0.4	0.9	115	71	175
India	Mirzapur	4.5	2.6	7.0	1710	981	2680	1.4	0.9	2.3	547	348	868
India	Moga	2.1	1.2	3.3	191	110	295	0.5	0.3	0.8	39	25	61
India	Mokokchung	1.1	0.6	1.7	23	13	36	0.4	0.2	0.6	4	3	7
India	Mon	1.1	0.7	1.7	36	22	53	0.4	0.2	0.6	8	5	13
India	Moradabad	4.3	2.5	6.4	1920	1110	2840	1.6	0.9	2.6	785	444	1250
India	Morbi	1.6	1.0	2.3	133	83	196	0.3	0.2	0.5	27	17	42
India	Morena	3.1	1.8	4.9	701	412	1100	0.6	0.4	1.0	168	102	255
India	Morigaon	3.6	2.1	5.8	437	256	704	1.3	0.7	2.1	178	104	292
India	Muktsar	2.2	1.4	3.3	202	127	308	0.5	0.3	0.8	45	26	71
India	Mumbai City	0.7	0.4	1.1	158	89	244	0.2	0.1	0.3	23	14	36
India	Mumbai Suburban	0.7	0.4	1.1	610	356	916	0.2	0.1	0.3	123	77	196
India	Mungeli	2.0	1.3	3.0	187	117	277	0.5	0.3	0.8	47	29	73
India	Munger	3.4	2.0	5.4	572	337	899	1.5	0.9	2.3	259	154	390
India	Murshidabad	1.2	0.7	1.7	919	558	1310	0.2	0.2	0.4	187	124	289
India	Muzaffarnagar	4.6	2.6	6.7	1830	1020	2690	1.5	0.9	2.5	592	348	946
India	Muzaffarpur	3.7	2.1	5.9	2110	1220	3390	1.7	1.0	2.5	1090	650	1620
India	Mysuru	1.1	0.7	1.7	278	172	450	0.2	0.1	0.4	50	31	81
India	Nabarangapur	3.0	1.9	5.0	468	299	763	0.9	0.5	1.3	143	87	220
India	Nadia	1.2	0.7	1.7	513	312	753	0.2	0.1	0.4	91	56	141
India	Nagaon	3.2	1.9	4.8	914	538	1370	1.2	0.7	1.8	387	238	604
India	Nagappattinam	1.0	0.6	1.6	139	88	231	0.1	0.1	0.2	16	10	25
India	Nagarkurnool	2.1	1.2	3.2	217	124	342	0.3	0.2	0.5	29	17	47
India	Nagaur	2.6	1.6	3.9	1030	642	1570	0.6	0.4	0.9	245	154	362
India	Nagpur	0.9	0.6	1.4	374	223	578	0.2	0.1	0.3	67	40	104
India	Nainital	2.0	1.2	3.2	197	116	316	0.3	0.2	0.5	27	16	43
India	Nalanda	3.5	2.1	5.8	1290	779	2160	1.6	1.0	2.5	624	378	951
India	Nalbari	3.2	1.9	5.2	274	160	444	1.1	0.7	1.6	90	55	137
India	Nalgonda	2.0	1.2	3.2	376	220	592	0.3	0.2	0.5	44	26	68
India	Namakkal	0.9	0.5	1.4	127	75	200	0.1	0.1	0.2	15	9	23
India	Namsai	2.2	1.3	3.2	24	14	35	0.4	0.2	0.6	5	3	8
India	Nanded	0.9	0.5	1.3	315	193	456	0.2	0.1	0.3	65	39	106
India	Nandurbar	1.0	0.6	1.4	170	107	248	0.2	0.1	0.3	37	23	56
India	Narayanpur	1.8	1.1	3.2	26	16	44	0.5	0.3	0.8	8	5	13
India	Narmada	1.4	0.9	2.1	95	60	140	0.3	0.2	0.5	21	13	33
India	Narsimhapur	3.0	1.7	4.7	388	217	595	0.6	0.4	1.0	75	47	131
India	Nashik	0.9	0.5	1.4	553	325	840	0.2	0.1	0.3	124	76	183
India	Navsari	1.2	0.7	1.7	143	89	211	0.3	0.2	0.4	30	18	45
India	Nawada	3.8	2.4	6.0	1020	642	1630	1.7	1.0	2.6	495	295	740
India	Nayagarh	3.2	1.9	5.1	272	165	439	0.8	0.4	1.1	61	35	92
India	Neemuch	3.0	1.7	4.5	250	145	375	0.6	0.3	0.9	46	28	75
India	Nellore	2.0	1.2	3.0	531	320	805	0.5	0.3	0.7	112	69	172
India	New Delhi	1.5	0.8	2.2	52	29	78	0.4	0.2	0.6	9	6	15
India	Nicobars	0.8	0.6	1.4	5	3	7	0.3	0.2	0.5	1	0	1
India	Nirmal	1.8	1.2	2.7	143	93	215	0.3	0.2	0.5	21	12	33
India	Niwari	3.1	1.8	4.6	161	91	241	0.6	0.4	0.9	34	21	54
India	Nizamabad	1.8	1.2	2.7	337	215	497	0.3	0.2	0.5	45	26	70
India	Noney	1.8	1.0	2.8	8	4	12	0.5	0.3	0.7	3	2	5
India	North	1.6	0.9	2.4	136	78	209	0.4	0.2	0.6	29	18	47
India	North & Middle Andaman	0.8	0.5	1.2	12	7	18	0.3	0.2	0.4	2	1	4
India	North 24 Parganas	1.3	0.8	1.9	1000	587	1480	0.3	0.2	0.4	171	102	269
India	North East	1.7	0.9	2.6	189	106	289	0.4	0.2	0.6	49	30	80

Table 3: Diarrhoea Mortality by Unit *(continued)*

Country	Unit	2000 mortality mean rate (per 10,000)	lower rate (per 10,000)	upper rate (per 10,000)	mean count	lower count	upper count	2017 mortality mean rate (per 10,000)	lower rate (per 10,000)	upper rate (per 10,000)	mean count	lower count	upper count
India	North Garo Hills	1.8	1.0	2.7	16	10	25	0.6	0.4	1.0	6	4	10
India	North Goa	0.4	0.2	0.7	21	12	33	0.2	0.1	0.3	6	3	9
India	North Sikkim	1.3	0.8	1.9	6	4	9	0.3	0.2	0.4	1	1	2
India	North Tripura	1.0	0.6	1.6	35	21	55	0.5	0.3	0.8	19	12	29
India	North West	1.5	0.9	2.4	482	278	736	0.4	0.2	0.6	122	75	195
India	Nuapada	3.3	2.1	5.1	239	148	364	0.9	0.5	1.4	63	38	100
India	Osmanabad	0.9	0.6	1.4	172	110	255	0.2	0.1	0.3	32	19	49
India	Pakke Kessang	2.7	1.6	4.3	6	3	9	0.4	0.3	0.7	1	1	2
India	Pakur	2.6	1.6	4.1	306	186	477	0.8	0.5	1.3	106	66	168
India	Palakkad	0.1	0.0	0.1	18	10	28	0.0	0.0	0.0	3	2	5
India	Palamu	3.1	2.0	4.6	694	437	1000	0.9	0.5	1.5	217	120	348
India	Palghar	0.8	0.5	1.2	187	118	281	0.2	0.1	0.3	51	32	79
India	Pali	2.6	1.6	3.8	651	399	974	0.6	0.4	1.0	141	85	224
India	Palwal	2.7	1.7	4.3	257	158	406	0.6	0.4	1.0	68	41	107
India	Panch Mahals	1.5	0.9	2.2	293	182	443	0.3	0.2	0.5	68	40	105
India	Panchkula	1.7	1.0	2.6	79	46	120	0.4	0.2	0.5	17	10	25
India	Panipat	2.6	1.4	4.1	327	180	504	0.6	0.3	0.9	74	45	116
India	Panna	3.3	2.2	4.9	438	291	643	0.6	0.4	1.0	90	58	138
India	Papum Pare	2.4	1.4	3.7	33	20	52	0.4	0.2	0.7	10	6	16
India	Parbhani	0.9	0.6	1.4	172	104	258	0.2	0.1	0.3	37	23	61
India	Paschimi Barddhama	1.3	0.8	2.0	390	237	617	0.3	0.2	0.4	69	39	114
India	Pashchim Champaran	4.4	2.7	7.3	2190	1310	3590	1.7	1.0	2.8	950	544	1570
India	Pashchim Medinipur	1.3	0.7	1.8	594	350	861	0.3	0.2	0.4	105	66	163
India	Pashchimi Singhbhum	3.0	1.9	4.4	569	353	835	1.0	0.6	1.5	180	106	290
India	Patan	1.6	1.0	2.4	234	144	356	0.3	0.2	0.5	47	28	72
India	Pathanamthitta	0.1	0.0	0.1	7	4	11	0.0	0.0	0.0	1	1	1
India	Pathankot	1.8	1.0	2.9	112	61	174	0.5	0.3	0.8	25	16	40
India	Patiala	1.7	1.0	2.7	306	180	481	0.4	0.2	0.6	70	41	107
India	Patna	3.7	2.3	5.9	2300	1420	3660	1.6	1.0	2.4	1060	649	1590
India	Pauri Garhwal	2.0	1.2	3.0	192	116	297	0.3	0.2	0.5	18	11	28
India	Peddapalli	1.9	1.1	3.0	153	90	241	0.3	0.2	0.5	18	11	29
India	Perambalur	1.0	0.6	1.7	48	29	80	0.1	0.1	0.2	6	3	10
India	Peren	1.2	0.7	1.9	14	8	21	0.4	0.2	0.6	4	2	6
India	Phek	1.1	0.6	1.5	19	12	28	0.4	0.2	0.6	6	4	9
India	Pherzawl	2.0	1.2	3.1	7	5	12	0.5	0.3	0.8	2	1	3
India	Pilibhit	4.2	2.7	6.1	1170	741	1710	1.5	0.9	2.3	411	244	605
India	Pithoragarh	2.2	1.4	3.3	138	86	206	0.3	0.2	0.5	12	7	19
India	Poonch	1.2	0.6	1.9	72	40	119	0.3	0.2	0.4	16	10	26
India	Porbandar	1.4	0.8	2.2	81	47	123	0.3	0.2	0.4	13	8	19
India	Prakasam	1.9	1.1	2.8	628	380	920	0.4	0.3	0.7	130	83	204
India	Pratapgarh	2.5	1.4	3.7	303	166	457	0.6	0.3	1.0	78	46	126
India	Pratapgarh	4.3	2.7	6.6	1720	1080	2660	1.5	0.9	2.3	561	351	865
India	Puducherry	0.6	0.4	1.0	41	25	65	0.3	0.2	0.5	16	9	26
India	Pudukkottai	1.0	0.6	1.7	162	95	286	0.1	0.1	0.2	19	11	29
India	Pulwama	1.1	0.6	1.8	85	49	143	0.2	0.1	0.4	17	9	29
India	Pune	0.8	0.5	1.3	654	393	1010	0.2	0.1	0.3	164	99	275
India	Purba Barddhaman	1.1	0.6	1.5	445	251	624	0.2	0.1	0.3	79	49	121
India	Purba Champaran	4.2	2.4	6.9	2650	1540	4360	1.7	1.0	2.7	1260	730	1960
India	Purba Medinipur	1.0	0.5	1.4	458	258	680	0.2	0.1	0.3	85	53	133
India	Purbi Singhbhum	2.6	1.6	3.9	636	391	961	0.8	0.5	1.3	179	103	286
India	Puri	2.9	1.8	4.6	417	261	662	0.7	0.4	1.0	94	59	143
India	Purnia	3.4	2.2	5.1	1520	968	2260	1.5	1.0	2.3	795	504	1160
India	Puruliya	1.2	0.7	1.9	398	246	631	0.2	0.1	0.4	76	43	119
India	Rae Bareli	4.5	2.9	6.8	1350	865	2020	1.4	0.9	2.3	411	247	655

Table 3: Diarrhoea Mortality by Unit *(continued)*

Country	Unit	2000 mortality mean rate (per 10,000)	lower rate (per 10,000)	upper rate (per 10,000)	mean count	lower count	upper count	2017 mortality mean rate (per 10,000)	lower rate (per 10,000)	upper rate (per 10,000)	mean count	lower count	upper count
India	Raichur	1.0	0.7	1.5	225	144	332	0.2	0.2	0.4	50	32	76
India	Raigad	0.7	0.4	1.0	161	95	232	0.2	0.1	0.3	36	22	60
India	Raigarh	2.0	1.2	3.2	306	187	479	0.5	0.3	0.8	83	49	130
India	Raipur	2.0	1.3	2.9	432	280	634	0.5	0.3	0.8	150	91	228
India	Raisen	3.1	1.8	4.8	526	310	815	0.6	0.4	1.0	112	67	185
India	Rajanna Sircilla	1.7	1.1	2.6	92	56	140	0.3	0.2	0.5	11	6	17
India	Rajgarh	3.1	2.0	5.0	513	325	819	0.6	0.3	0.9	108	66	169
India	Rajkot	1.5	0.9	2.3	417	242	640	0.3	0.2	0.5	82	52	129
India	Rajnandgaon	1.9	1.3	2.8	323	220	469	0.5	0.3	0.8	92	58	144
India	Rajouri	1.2	0.7	2.0	62	36	101	0.3	0.2	0.5	15	9	24
India	Rajsamand	2.5	1.5	3.9	352	214	548	0.6	0.3	0.9	80	48	121
India	Ramanagara	1.0	0.6	1.5	100	62	154	0.2	0.1	0.4	17	10	28
India	Ramanathapuram	1.0	0.6	1.6	120	69	186	0.1	0.1	0.2	14	8	22
India	Ramban	1.2	0.7	1.9	32	19	51	0.3	0.2	0.5	8	5	13
India	Ramgarh	2.4	1.5	3.7	256	158	399	0.8	0.4	1.2	73	41	116
India	Rampur	4.3	2.5	6.3	1380	802	2040	1.6	0.9	2.5	536	318	853
India	Ranchi	2.6	1.7	4.0	774	489	1170	0.8	0.4	1.2	232	127	359
India	Ranga Reddy	2.0	1.2	3.2	669	387	1050	0.3	0.2	0.5	108	59	161
India	Ratlam	2.9	1.7	4.5	495	285	758	0.6	0.4	0.9	109	68	174
India	Ratnagiri	0.8	0.5	1.2	122	77	192	0.2	0.1	0.3	16	9	26
India	Rayagada	3.2	2.0	5.0	382	243	600	0.9	0.5	1.5	113	65	183
India	Reasi	1.2	0.7	1.9	40	23	64	0.3	0.2	0.4	8	5	13
India	Rewa	3.1	2.0	4.8	844	531	1320	0.6	0.4	1.0	178	112	290
India	Rewari	2.6	1.5	3.8	193	114	285	0.5	0.3	0.8	39	23	59
India	Ri Bhoi	1.8	1.1	2.8	62	37	97	0.7	0.4	1.0	27	15	41
India	Rohtak	2.6	1.5	4.1	285	168	442	0.5	0.3	0.9	53	32	85
India	Rohtas	4.0	2.5	6.1	1440	918	2200	1.6	1.0	2.5	594	352	926
India	Rudraprayag	2.0	1.3	3.2	59	37	92	0.3	0.2	0.5	6	4	9
India	Rupnagar	1.7	1.0	2.8	111	66	180	0.4	0.3	0.6	22	14	33
India	Sabar Kantha	1.5	0.9	2.2	241	147	361	0.3	0.2	0.5	54	33	83
India	Sagar	3.1	1.9	4.9	898	548	1430	0.6	0.4	1.0	196	124	310
India	Saharanpur	4.0	2.3	5.9	1800	1060	2710	1.3	0.7	1.9	577	337	877
India	Saharsa	3.8	2.3	5.7	1020	609	1550	1.6	1.0	2.4	486	291	716
India	Sahibganj	2.8	1.8	4.2	589	382	890	0.9	0.5	1.4	191	116	309
India	Sahibzada Ajit Singh Nagar	1.4	0.8	2.1	151	91	230	0.3	0.2	0.5	42	25	62
India	Saiha	0.8	0.5	1.2	5	3	7	0.2	0.1	0.4	1	1	2
India	Salem	1.0	0.6	1.5	296	173	449	0.1	0.1	0.2	37	22	61
India	Samastipur	3.5	2.1	5.9	2080	1240	3470	1.6	1.0	2.4	1020	649	1520
India	Samba	1.2	0.7	2.0	34	20	53	0.3	0.2	0.4	6	4	9
India	Sambalpur	3.3	2.1	5.1	369	232	563	0.8	0.5	1.4	87	52	148
India	Sambhal	4.5	2.7	7.1	1420	850	2240	1.6	0.9	2.6	540	315	848
India	Sangareddy	2.1	1.3	3.2	381	235	584	0.3	0.2	0.5	49	28	76
India	Sangli	0.9	0.6	1.4	259	164	401	0.2	0.1	0.3	43	26	75
India	Sangrur	1.8	1.0	2.8	277	161	427	0.4	0.3	0.7	57	35	91
India	Sant Kabir Nagar	4.4	2.6	6.9	833	494	1330	1.8	1.1	3.0	336	199	559
India	Sant Ravi Das Nagar	4.5	2.7	7.4	958	573	1580	1.5	0.9	2.3	306	187	463
India	Saraikela Kharsawan	2.7	1.6	4.2	299	177	454	0.8	0.5	1.3	92	51	148
India	Saran	3.9	2.4	6.6	1770	1080	2970	1.7	1.0	2.6	802	496	1230
India	Satara	0.9	0.6	1.3	250	155	376	0.2	0.1	0.3	41	25	72
India	Satna	3.1	2.0	4.5	805	512	1150	0.6	0.4	0.9	169	112	257
India	Sawai Madhopur	2.5	1.6	3.9	387	241	591	0.6	0.4	1.0	95	57	161
India	Sehore	3.0	1.9	4.8	466	291	735	0.6	0.4	0.9	102	66	160
India	Senapati	1.7	1.0	2.5	52	32	77	0.5	0.3	0.7	24	16	39
India	Seoni	2.9	1.8	4.4	419	264	639	0.5	0.3	0.9	78	50	135
India	Serchhip	0.8	0.5	1.2	3	2	4	0.2	0.1	0.4	1	0	1
India	Shahdara	1.7	1.0	2.6	177	102	272	0.4	0.2	0.7	44	26	73
India	Shahdol	3.0	2.0	4.5	342	221	510	0.6	0.4	0.9	72	44	109

Table 3: Diarrhoea Mortality by Unit *(continued)*

Country	Unit	2000 mortality mean rate (per 10,000)	lower rate (per 10,000)	upper rate (per 10,000)	mean count	lower count	upper count	2017 mortality mean rate (per 10,000)	lower rate (per 10,000)	upper rate (per 10,000)	mean count	lower count	upper count
India	Shahid Bhagat Singh Nagar	1.8	1.0	3.0	105	62	178	0.4	0.2	0.6	19	11	28
India	Shahjahanpur	4.5	2.7	6.9	1920	1150	2940	1.7	1.0	2.6	722	428	1120
India	Shajapur	3.2	2.0	5.0	350	218	545	0.6	0.4	0.9	65	43	99
India	Shamli	4.6	2.6	6.8	773	436	1150	1.5	0.9	2.3	240	141	370
India	Sheikhpura	3.3	1.9	5.3	314	182	507	1.6	0.9	2.5	161	93	243
India	Sheohar	3.6	2.0	5.7	323	179	517	1.7	1.0	2.6	172	99	269
India	Sheopur	3.2	2.0	4.8	305	196	455	0.6	0.4	1.0	67	42	107
India	Shi Yomi	2.3	1.4	3.6	15	9	24	0.4	0.2	0.6	3	2	4
India	Shimla	1.5	0.9	2.5	107	63	176	0.3	0.2	0.4	16	10	23
India	Shivamogga	1.1	0.7	1.7	184	111	283	0.2	0.1	0.3	29	18	46
India	Shivpuri	3.3	1.9	4.8	705	412	1030	0.6	0.4	0.9	149	93	220
India	Shravasti	4.6	2.6	7.4	1460	814	2330	2.1	1.3	3.4	782	483	1230
India	Shupiyan	1.1	0.6	1.9	33	19	56	0.3	0.1	0.4	7	4	12
India	Siang	2.2	1.3	3.4	9	5	13	0.4	0.2	0.6	1	1	2
India	Siddharth Nagar	4.6	2.9	7.5	2300	1440	3730	1.9	1.1	3.0	1020	597	1620
India	Siddipet	1.9	1.2	2.8	211	135	314	0.3	0.2	0.5	26	14	42
India	Sidhi	3.0	1.8	4.6	418	253	638	0.6	0.4	1.0	99	59	156
India	Sikar	2.6	1.5	4.0	785	470	1230	0.6	0.3	0.9	173	104	264
India	Simdega	3.0	1.9	4.5	215	137	323	0.8	0.5	1.3	53	30	83
India	Sindhudurg	0.8	0.5	1.3	57	35	88	0.2	0.1	0.3	7	4	12
India	Singrauli	3.0	1.8	4.5	482	291	729	0.6	0.3	1.0	119	70	200
India	Sipahijala	1.1	0.7	1.8	46	27	73	0.5	0.3	0.8	20	12	31
India	Sirmaur	1.5	0.9	2.4	79	46	124	0.3	0.2	0.4	12	7	18
India	Sirohi	2.3	1.5	3.6	322	202	494	0.6	0.4	1.0	91	53	150
India	Sirsa	2.6	1.7	3.9	337	217	508	0.5	0.3	0.8	66	40	101
India	Sitamarhi	4.2	2.5	6.4	1750	1040	2660	1.8	1.0	2.7	836	479	1300
India	Sitapur	4.6	2.7	6.9	2780	1650	4170	1.8	1.2	2.8	1170	734	1780
India	Sivaganga	1.0	0.6	1.6	124	71	196	0.1	0.1	0.2	16	9	24
India	Sivasagar	2.8	1.7	4.1	234	145	348	1.0	0.6	1.7	79	49	129
India	Siwan	4.5	2.6	7.3	1640	926	2650	1.7	1.0	2.7	654	385	1040
India	Solan	1.5	0.9	2.4	88	53	141	0.3	0.2	0.4	13	8	19
India	Solapur	0.9	0.6	1.4	420	263	624	0.2	0.1	0.3	76	46	118
India	Sonbhadra	4.1	2.4	6.1	1070	629	1600	1.3	0.8	2.2	367	223	598
India	Sonepur	3.5	2.2	5.4	227	139	347	0.8	0.5	1.4	51	32	86
India	Sonipat	2.7	1.5	4.1	459	255	693	0.6	0.3	0.9	89	52	137
India	Sonitpur	3.3	1.8	5.1	438	246	687	1.1	0.7	1.8	157	96	255
India	South	1.4	0.8	2.2	193	111	298	0.4	0.2	0.6	51	31	82
India	South 24 Parganas	1.1	0.7	1.7	999	591	1490	0.2	0.1	0.4	190	119	298
India	South Andaman	0.8	0.5	1.3	18	11	30	0.3	0.2	0.4	5	3	8
India	South East	1.5	0.8	2.2	172	98	263	0.4	0.2	0.6	44	26	70
India	South Garo Hills	2.1	1.2	3.2	35	20	53	0.6	0.4	0.9	10	6	16
India	South Goa	0.4	0.2	0.7	29	16	47	0.1	0.1	0.3	8	4	13
India	South Salmara Mancachar	3.4	2.0	5.2	178	107	273	1.1	0.7	1.6	67	42	98
India	South Sikkim	1.2	0.7	1.8	15	10	24	0.2	0.1	0.4	3	2	4
India	South Tripura	1.1	0.7	1.8	45	28	74	0.5	0.3	0.8	21	13	34
India	South West	1.4	0.8	2.1	232	131	355	0.3	0.2	0.6	65	38	103
India	South West Garo Hills	1.7	1.0	2.5	17	10	25	0.6	0.4	0.9	6	4	9
India	South West Khasi Hills	1.9	1.2	2.9	28	17	42	0.5	0.3	0.8	8	5	12
India	Srikakulam	2.1	1.4	3.3	505	327	798	0.4	0.3	0.7	86	51	138
India	Srinagar	1.1	0.6	1.9	64	36	112	0.3	0.1	0.4	14	7	22
India	Sukma	1.9	1.3	2.9	57	37	86	0.5	0.3	0.7	15	9	22
India	Sultanpur	4.5	2.8	6.9	1210	759	1870	1.4	0.8	2.2	373	219	574
India	Sundargarh	3.2	2.0	5.0	693	433	1090	0.8	0.5	1.3	177	103	278
India	Supaul	4.1	2.5	6.1	1190	726	1750	1.6	1.0	2.5	537	334	839
India	Surajpur	2.0	1.2	3.2	195	117	311	0.5	0.3	0.9	54	34	92

Table 3: Diarrhoea Mortality by Unit *(continued)*

Country	Unit	2000 mortality mean rate (per 10,000)	2000 mortality lower rate (per 10,000)	2000 mortality upper rate (per 10,000)	2000 mortality mean count	2000 mortality lower count	2000 mortality upper count	2017 mortality mean rate (per 10,000)	2017 mortality lower rate (per 10,000)	2017 mortality upper rate (per 10,000)	2017 mortality mean count	2017 mortality lower count	2017 mortality upper count
India	Surat	1.3	0.8	2.0	613	364	972	0.3	0.2	0.5	207	121	320
India	Surendranagar	1.6	1.0	2.4	276	178	412	0.3	0.2	0.5	56	33	85
India	Surguja	2.0	1.2	3.0	203	124	308	0.5	0.3	0.8	57	34	90
India	Suryapet	1.9	1.2	3.0	230	137	357	0.3	0.2	0.5	28	16	43
India	Tamenglong	1.8	1.0	2.7	18	10	27	0.5	0.3	0.7	5	3	8
India	Tapi	1.3	0.8	2.0	105	68	160	0.3	0.2	0.5	24	14	35
India	Tarn Taran	2.2	1.2	3.2	225	128	329	0.5	0.3	0.8	52	32	83
India	Tawang	2.7	1.5	4.4	10	5	16	0.5	0.3	0.8	2	1	4
India	Tehri Garhwal	2.0	1.2	3.1	166	100	260	0.3	0.2	0.4	15	9	23
India	Tengnoupal	1.8	1.1	2.8	7	5	11	0.5	0.3	0.8	2	1	3
India	Thane	0.7	0.4	1.1	394	236	601	0.2	0.1	0.3	114	72	182
India	Thanjavur	1.0	0.6	1.6	210	125	342	0.1	0.1	0.2	24	14	37
India	The Nilgiris	1.1	0.7	1.7	76	45	119	0.1	0.1	0.2	6	4	11
India	Theni	1.0	0.6	1.6	107	59	169	0.1	0.1	0.2	13	8	21
India	Thiruvallur	1.0	0.6	1.5	327	205	511	0.1	0.1	0.2	55	33	86
India	Thiruvananthapuram	0.1	0.0	0.1	16	9	26	0.0	0.0	0.0	2	1	4
India	Thiruvarur	1.0	0.6	1.6	129	76	206	0.1	0.1	0.2	15	9	23
India	Thoubal	1.7	1.0	2.6	51	30	77	0.5	0.3	0.7	14	9	22
India	Thrissur	0.1	0.0	0.1	17	10	29	0.0	0.0	0.0	3	2	4
India	Tikamgarh	3.1	1.8	4.8	385	225	588	0.6	0.4	0.9	87	58	130
India	Tinsukia	3.4	2.0	5.1	409	246	612	1.1	0.6	1.7	137	78	220
India	Tirap	2.4	1.5	3.6	19	11	28	0.4	0.3	0.7	4	2	6
India	Tiruchirappalli	1.0	0.6	1.7	248	146	417	0.1	0.1	0.2	31	18	46
India	Tirunelveli	1.0	0.6	1.5	289	169	423	0.1	0.1	0.2	36	22	58
India	Tiruppur	1.0	0.6	1.6	189	115	306	0.1	0.1	0.2	26	15	39
India	Tiruvannamalai	1.0	0.6	1.5	245	148	378	0.1	0.1	0.2	30	18	45
India	Tonk	2.5	1.5	3.8	421	249	626	0.6	0.3	0.8	91	52	138
India	Tuensang	1.1	0.7	1.7	28	17	41	0.4	0.2	0.6	8	5	13
India	Tumakuru	1.0	0.6	1.7	251	156	403	0.2	0.1	0.3	41	25	63
India	Tuticorin	1.0	0.6	1.4	150	87	220	0.1	0.1	0.2	18	11	29
India	Udaipur	2.3	1.4	3.5	892	532	1320	0.6	0.4	1.0	247	150	407
India	Udalguri	3.6	2.0	5.8	224	126	361	1.2	0.7	1.8	70	43	110
India	Udham Singh Nagar	2.1	1.3	3.3	377	227	588	0.4	0.2	0.5	61	36	91
India	Udhampur	1.2	0.7	1.8	71	41	108	0.3	0.2	0.4	14	8	21
India	Udupi	0.9	0.5	1.5	79	46	128	0.2	0.1	0.2	10	6	16
India	Ujjain	3.0	1.8	4.6	658	396	1000	0.6	0.4	0.9	132	83	202
India	Ukhrul	1.6	0.9	2.3	22	13	33	0.5	0.3	0.8	8	5	13
India	Umaria	3.0	1.9	4.3	266	169	379	0.6	0.4	0.9	61	38	95
India	Una	1.6	0.9	2.6	72	41	116	0.3	0.2	0.4	11	7	16
India	Unnao	4.6	2.9	6.8	1880	1210	2820	1.5	0.9	2.3	544	345	847
India	Unokoti	1.1	0.7	1.8	33	20	51	0.5	0.3	0.8	16	10	24
India	Upper Siang	2.5	1.5	3.8	9	6	15	0.4	0.3	0.6	2	1	2
India	Upper Subansiri	2.6	1.5	3.9	11	7	17	0.4	0.3	0.7	3	2	5
India	Uttar Bastar Kanker	1.9	1.2	2.9	160	103	245	0.5	0.3	0.8	44	26	70
India	Uttar Dinajpur	1.3	0.8	2.0	416	260	651	0.3	0.2	0.4	92	59	138
India	Uttara Kannada	1.0	0.6	1.4	132	83	191	0.2	0.1	0.3	20	12	30
India	Uttarkashi	2.0	1.3	3.1	90	57	143	0.3	0.2	0.4	10	6	15
India	Vadodara	1.4	0.9	2.3	439	273	715	0.3	0.2	0.5	99	58	153
India	Vaishali	3.7	2.2	6.2	1620	967	2700	1.6	1.0	2.5	810	488	1230
India	Valsad	1.1	0.7	1.6	194	118	287	0.3	0.2	0.4	53	32	79
India	Varanasi	4.4	2.5	6.9	1900	1090	2990	1.4	0.9	2.2	568	363	914
India	Vellore	1.0	0.6	1.5	365	219	551	0.1	0.1	0.2	44	26	67
India	Vidisha	3.3	2.0	5.0	587	356	905	0.6	0.4	1.1	131	82	222
India	Vijaypura	1.0	0.6	1.6	245	144	383	0.2	0.1	0.4	56	34	87
India	Vikarabad	2.1	1.3	3.5	201	125	333	0.3	0.2	0.5	36	21	56
India	Viluppuram	1.0	0.6	1.6	356	222	582	0.1	0.1	0.2	46	29	71
India	Virudunagar	1.0	0.6	1.5	177	102	264	0.1	0.1	0.2	22	13	36
India	Visakhapatnam	2.0	1.2	2.9	783	477	1160	0.4	0.3	0.6	156	97	225

Table 3: Diarrhoea Mortality by Unit *(continued)*

Country	Unit	2000 mortality mean rate (per 10,000)	lower rate (per 10,000)	upper rate (per 10,000)	mean count	lower count	upper count	2017 mortality mean rate (per 10,000)	lower rate (per 10,000)	upper rate (per 10,000)	mean count	lower count	upper count
India	Vizianagaram	2.0	1.3	3.0	476	300	702	0.5	0.3	0.8	83	50	139
India	Wanaparthy	2.1	1.3	3.2	159	94	242	0.3	0.2	0.6	21	12	34
India	Warangal Rural	1.9	1.1	3.2	137	80	232	0.3	0.2	0.5	16	10	25
India	Warangal Urban	1.8	1.1	3.0	196	118	323	0.3	0.2	0.5	24	15	37
India	Wardha	1.0	0.6	1.4	112	70	168	0.2	0.1	0.3	17	9	25
India	Washim	0.9	0.6	1.4	110	66	168	0.2	0.1	0.3	22	13	33
India	Wayanad	0.1	0.0	0.1	6	3	9	0.0	0.0	0.0	1	1	1
India	West	1.6	0.9	2.4	375	213	572	0.4	0.2	0.6	89	53	141
India	West Garo Hills	1.7	1.0	2.6	112	67	168	0.6	0.4	0.9	40	26	61
India	West Godavari	1.9	1.1	2.8	677	414	1030	0.4	0.3	0.6	117	74	173
India	West Jaintia Hills	1.8	1.1	2.8	63	38	98	0.7	0.4	1.1	28	16	44
India	West Kameng	3.0	1.6	4.7	27	15	42	0.5	0.3	0.8	5	3	7
India	West Karbi Anglong	3.7	2.3	5.8	139	86	216	1.3	0.8	2.0	55	33	82
India	West Khasi Hills	2.0	1.3	3.2	71	46	113	0.5	0.3	0.7	18	11	27
India	West Siang	2.7	1.6	4.1	4	2	6	0.5	0.3	0.8	1	0	1
India	West Sikkim	1.3	0.8	1.9	17	10	26	0.2	0.2	0.4	3	2	4
India	West Tripura	1.1	0.6	1.7	84	49	136	0.5	0.3	0.8	36	22	55
India	Wokha	1.1	0.7	1.7	18	11	27	0.4	0.2	0.6	5	3	8
India	Y.S.R.	2.1	1.2	3.1	597	353	912	0.5	0.3	0.7	118	73	180
India	Yadadri Bhuvanagiri	2.1	1.2	3.1	166	98	250	0.3	0.2	0.5	18	10	27
India	Yadgir	1.0	0.6	1.5	134	82	199	0.2	0.1	0.4	34	21	54
India	Yamunanagar	2.2	1.3	3.3	217	124	325	0.4	0.2	0.7	39	22	62
India	Yanam	0.8	0.5	1.3	1	1	2	0.3	0.2	0.4	1	0	1
India	Yavatmal	0.9	0.6	1.4	260	164	396	0.2	0.1	0.3	47	29	69
India	Zunheboto	1.1	0.7	1.6	20	13	30	0.4	0.2	0.7	5	3	8
Nepal	Bagmati	1.8	1.0	3.1	708	415	1230	0.2	0.1	0.4	113	64	203
Nepal	Bheri	1.9	1.1	3.2	479	271	797	0.3	0.2	0.4	43	25	72
Nepal	Dhaualagiri	1.7	1.0	2.9	310	186	520	0.2	0.1	0.4	15	8	24
Nepal	Gandaki	1.7	0.9	2.6	560	318	876	0.2	0.1	0.4	51	28	81
Nepal	Janakpur	2.0	1.2	3.1	708	443	1110	0.2	0.1	0.4	66	34	111
Nepal	Karnali	2.1	1.1	3.6	342	181	586	0.3	0.2	0.5	11	6	19
Nepal	Koshi	1.7	1.0	2.7	344	200	547	0.2	0.1	0.4	72	42	117
Nepal	Lumbini	1.7	1.0	2.9	698	417	1170	0.2	0.1	0.4	83	47	127
Nepal	Mahakali	2.1	1.1	3.3	152	82	248	0.3	0.1	0.4	31	17	46
Nepal	Mechi	1.6	0.9	2.6	226	121	357	0.2	0.1	0.4	24	14	40
Nepal	Narayani	1.8	1.1	3.1	595	366	1030	0.3	0.1	0.4	106	60	171
Nepal	Rapti	2.0	1.2	3.2	397	235	650	0.3	0.1	0.4	41	23	64
Nepal	Sagarmatha	1.9	1.2	3.1	657	405	1040	0.2	0.1	0.4	57	31	91
Nepal	Seti	2.0	1.2	3.4	270	160	462	0.3	0.1	0.4	41	24	65
Pakistan	Azad Kashmir	2.0	1.2	3.1	888	549	1400	0.9	0.5	1.4	408	236	637
Pakistan	Bahawalpur	2.5	1.8	3.6	3520	2500	5100	1.1	0.7	1.4	1770	1170	2410
Pakistan	Bannu	3.3	1.9	5.4	757	432	1220	1.1	0.6	1.9	313	163	536
Pakistan	Dera Ghazi Khan	2.6	1.7	4.0	3560	2290	5480	1.1	0.7	1.6	1790	1150	2710
Pakistan	Dera Ismail Khan	3.5	1.9	5.8	681	362	1120	1.2	0.6	1.9	279	143	452
Pakistan	F.A.T.A. 1	2.2	1.4	3.3	1160	734	1710	0.9	0.6	1.4	537	320	821
Pakistan	F.A.T.A. 2	2.2	1.3	3.6	71	42	116	0.9	0.5	1.5	31	18	51
Pakistan	Faisalabad	2.6	1.5	3.9	4140	2430	6340	1.0	0.6	1.5	1970	1180	2880
Pakistan	Gujranwala	2.4	1.5	3.9	4740	2930	7610	1.0	0.6	1.6	2330	1380	3670
Pakistan	Hazara	3.1	1.8	4.8	1620	941	2550	1.1	0.6	1.8	713	385	1170
Pakistan	Hyderabad	2.6	1.7	4.0	3230	2030	4920	1.1	0.7	1.7	1530	951	2450
Pakistan	Islamabad	2.0	1.1	3.3	373	205	602	0.9	0.5	1.6	243	129	429
Pakistan	Kalat	3.4	2.4	4.8	834	573	1170	1.3	0.9	1.8	290	209	412
Pakistan	Karachi	2.4	1.4	3.6	3200	1850	4860	1.1	0.6	1.8	1700	922	2820
Pakistan	Kohat	3.2	1.9	5.4	754	440	1270	1.1	0.6	1.7	318	184	499
Pakistan	Lahore	2.5	1.4	3.9	5950	3350	9240	1.0	0.6	1.6	2900	1760	4630
Pakistan	Larkana	2.7	1.6	4.4	2400	1390	3880	1.1	0.7	1.6	1080	674	1630
Pakistan	Makran	3.5	2.2	5.1	536	345	793	1.2	0.7	1.7	169	109	252
Pakistan	Malakand	3.2	1.9	5.1	2300	1340	3680	1.1	0.7	1.8	1030	603	1600

Country	Unit	2000 mortality mean rate (per 10,000)	lower rate (per 10,000)	upper rate (per 10,000)	mean count	lower count	upper count	2017 mortality mean rate (per 10,000)	lower rate (per 10,000)	upper rate (per 10,000)	mean count	lower count	upper count
Pakistan	Mardan	3.1	1.8	5.1	1590	901	2600	1.1	0.6	1.9	701	390	1200
Pakistan	Mirpur Khas	2.7	1.7	4.0	1920	1190	2830	1.1	0.6	1.8	905	518	1460
Pakistan	Multan	2.5	1.6	3.8	5160	3190	7740	1.1	0.7	1.6	2560	1610	3820
Pakistan	Nasirabad	3.5	2.1	5.6	577	341	927	1.1	0.7	1.6	168	103	240
Pakistan	Northern Areas	2.1	1.4	3.0	267	184	378	1.0	0.7	1.4	135	91	190
Pakistan	Peshawar	3.2	1.7	5.5	2080	1130	3570	1.1	0.6	1.8	890	495	1450
Pakistan	Quetta	3.2	1.9	5.3	923	549	1520	1.2	0.7	1.9	322	195	502
Pakistan	Rann of Kutch	3.0	1.5	5.0	19	10	32	1.2	0.6	2.0	9	5	15
Pakistan	Rawalpindi	2.4	1.6	3.5	2270	1500	3340	1.0	0.6	1.6	1110	670	1750
Pakistan	Sargodha	2.5	1.7	3.8	2410	1600	3630	1.0	0.6	1.6	1130	725	1760
Pakistan	Sibi	3.9	2.5	5.7	299	193	442	1.3	0.8	1.8	90	56	127
Pakistan	Sukkur	2.6	1.7	4.2	2730	1720	4370	1.1	0.7	1.6	1270	784	1960
Pakistan	Zhob	3.7	2.4	5.5	615	398	904	1.3	0.8	1.8	195	130	285
**Southeast Asia, East Asia, and Oceania**													
Cambodia	Aek Phnum	1.3	0.8	1.9	13	8	19	0.2	0.1	0.3	1	1	2
Cambodia	Andoung Meas	1.3	0.8	1.9	2	1	3	0.2	0.1	0.3	0	0	0
Cambodia	Angk Snuol	1.3	0.8	1.8	18	12	26	0.2	0.1	0.3	3	2	5
Cambodia	Angkor Borei	1.2	0.8	1.7	9	6	13	0.2	0.1	0.3	1	1	2
Cambodia	Angkor Chey	1.1	0.7	1.5	12	8	17	0.2	0.1	0.3	1	1	2
Cambodia	Angkor Chum	1.2	0.8	1.8	10	6	14	0.2	0.1	0.3	1	1	2
Cambodia	Angkor Thum	1.2	0.8	1.8	4	3	6	0.2	0.1	0.3	1	0	1
Cambodia	Anlong Veaeng	1.3	0.9	1.9	2	1	3	0.2	0.1	0.3	8	5	12
Cambodia	Aoral	1.2	0.8	1.7	3	2	5	0.2	0.1	0.2	1	1	1
Cambodia	Ba Phnum	1.2	0.8	1.8	14	9	20	0.2	0.1	0.3	1	1	2
Cambodia	Bakan	1.4	0.9	2.0	28	18	39	0.2	0.1	0.3	3	2	5
Cambodia	Ban Lung	1.4	0.9	2.1	5	3	7	0.2	0.1	0.3	1	1	2
Cambodia	Banan	1.2	0.8	1.8	17	11	25	0.2	0.1	0.3	2	1	3
Cambodia	Banteay Ampil	1.2	0.7	1.7	6	4	9	0.2	0.1	0.2	1	1	2
Cambodia	Banteay Meas	1.1	0.7	1.5	13	9	19	0.2	0.1	0.3	1	1	2
Cambodia	Banteay Srei	1.2	0.8	1.7	7	4	10	0.2	0.1	0.2	1	1	2
Cambodia	Bar Kaev	1.4	0.9	2.1	3	2	5	0.2	0.1	0.3	1	1	1
Cambodia	Baray	1.4	0.9	2.2	36	23	56	0.2	0.1	0.3	3	2	5
Cambodia	Baribour	1.5	1.0	2.2	12	8	18	0.2	0.1	0.3	1	1	2
Cambodia	Basedth	1.1	0.8	1.6	18	13	26	0.2	0.1	0.3	2	1	4
Cambodia	Bat Dambang	1.3	0.8	1.9	23	15	34	0.2	0.1	0.3	2	1	3
Cambodia	Batheay	1.4	0.9	2.1	20	13	30	0.2	0.1	0.3	2	1	3
Cambodia	Bati	1.2	0.8	1.7	20	14	29	0.2	0.1	0.3	3	2	4
Cambodia	Bavel	1.2	0.8	1.8	16	10	23	0.2	0.1	0.3	3	2	4
Cambodia	Botum Sakor	1.0	0.6	1.5	3	2	5	0.1	0.1	0.2	0	0	1
Cambodia	Bourei Cholsar	0.9	0.7	1.3	6	4	8	0.1	0.1	0.2	1	0	1
Cambodia	Chamkar Leu	1.4	0.9	2.2	27	17	41	0.2	0.1	0.3	2	1	3
Cambodia	Chantrea	1.1	0.8	1.7	8	6	12	0.1	0.1	0.2	1	1	1
Cambodia	Chbar Mon	1.2	0.8	1.7	6	4	9	0.2	0.1	0.3	1	0	1
Cambodia	Cheung Prey	1.4	0.9	2.1	18	11	27	0.2	0.1	0.3	2	1	3
Cambodia	Chey Saen	1.3	0.8	2.0	4	3	6	0.2	0.1	0.3	1	0	1
Cambodia	Chhaeb	1.2	0.8	1.8	3	2	5	0.2	0.1	0.3	1	0	1
Cambodia	Chhloung	1.5	1.0	2.2	13	8	19	0.2	0.1	0.3	1	1	2
Cambodia	Chhuk	1.0	0.7	1.5	11	8	16	0.2	0.1	0.3	2	1	3
Cambodia	Chi Kraeng	1.4	0.9	2.0	26	16	38	0.2	0.1	0.3	3	2	5
Cambodia	Choam Khsant	1.2	0.8	1.9	4	3	6	0.2	0.1	0.3	1	1	2
Cambodia	Chol Kiri	1.5	1.0	2.2	7	5	10	0.2	0.1	0.3	1	0	1
Cambodia	Chong Kal	1.3	0.8	1.8	4	3	6	0.2	0.1	0.3	1	0	1
Cambodia	Chum Kiri	1.0	0.7	1.5	7	5	10	0.2	0.1	0.3	2	1	3
Cambodia	Dambae	1.4	0.9	2.0	12	8	18	0.2	0.1	0.3	3	2	5
Cambodia	Dang Tong	1.0	0.7	1.5	8	6	12	0.2	0.1	0.3	1	1	2
Cambodia	Dangkao	1.3	0.9	1.9	22	15	32	0.2	0.1	0.3	7	4	11
Cambodia	Doun Kaev	1.1	0.8	1.7	7	5	10	0.2	0.1	0.3	1	0	1
Cambodia	Kaeb	1.0	0.7	1.5	5	3	7	0.2	0.1	0.3	1	0	1
Cambodia	Kaev Seima	1.3	0.8	1.8	3	2	4	0.2	0.1	0.2	1	1	1
Cambodia	Kamchay Mear	1.2	0.8	1.8	13	9	20	0.2	0.1	0.3	1	1	2

Table 3: Diarrhoea Mortality by Unit *(continued)*

Country	Unit	2000 mortality mean rate (per 10,000)	lower rate (per 10,000)	upper rate (per 10,000)	mean count	lower count	upper count	2017 mortality mean rate (per 10,000)	lower rate (per 10,000)	upper rate (per 10,000)	mean count	lower count	upper count
Cambodia	Kampong Bay	1.1	0.7	1.6	4	3	7	0.1	0.1	0.2	1	0	1
Cambodia	Kampong Cham	1.4	0.9	2.1	6	4	9	0.2	0.1	0.3	1	0	1
Cambodia	Kampong Chhnang	1.6	1.1	2.3	7	5	11	0.2	0.1	0.3	1	0	1
Cambodia	Kampong Leaeng	1.6	1.0	2.3	11	7	16	0.2	0.1	0.3	1	1	2
Cambodia	Kampong Leav	1.3	0.9	1.9	11	7	16	0.2	0.1	0.3	1	1	2
Cambodia	Kampong Rou	1.2	0.8	1.8	11	7	16	0.2	0.1	0.3	1	1	2
Cambodia	Kampong Seila	1.1	0.7	1.6	2	2	4	0.1	0.1	0.2	0	0	1
Cambodia	Kampong Siem	1.5	1.0	2.2	2	1	3	0.2	0.1	0.3	0	0	0
Cambodia	Kampong Siem	1.4	0.9	2.1	22	14	32	0.2	0.1	0.3	2	1	3
Cambodia	Kampong Svay	1.5	1.0	2.1	19	13	28	0.2	0.1	0.3	2	1	3
Cambodia	Kampong Trabaek	1.2	0.8	1.8	19	13	29	0.2	0.1	0.3	2	1	3
Cambodia	Kampong Trach	1.0	0.6	1.4	12	8	17	0.1	0.1	0.3	1	1	2
Cambodia	Kampong Tralach	1.5	1.0	2.1	18	12	25	0.2	0.1	0.3	2	1	3
Cambodia	Kampot	1.0	0.7	1.5	15	10	22	0.2	0.1	0.2	2	1	4
Cambodia	Kandal Stueng	1.2	0.8	1.8	15	10	22	0.2	0.1	0.3	2	1	4
Cambodia	Kandieng	1.5	1.0	2.1	12	8	17	0.2	0.1	0.3	1	1	2
Cambodia	Kang Meas	1.4	0.9	2.1	20	13	29	0.2	0.1	0.3	2	1	3
Cambodia	Kanhchriech	1.2	0.8	1.8	10	7	15	0.2	0.1	0.3	1	1	2
Cambodia	Kaoh Andaet	1.1	0.8	1.7	8	5	12	0.2	0.1	0.3	1	1	1
Cambodia	Kaoh Kong	1.1	0.7	1.6	1	1	2	0.1	0.1	0.2	0	0	0
Cambodia	Kaoh Nheaek	1.5	0.9	2.1	3	2	4	0.2	0.1	0.3	1	0	1
Cambodia	Kaoh Soutin	1.4	0.9	2.0	15	10	21	0.2	0.1	0.3	1	1	2
Cambodia	Kaoh Thum	1.2	0.8	1.8	24	16	36	0.2	0.1	0.3	2	1	4
Cambodia	Khsach Kandal	1.4	0.9	2.0	24	16	34	0.2	0.1	0.3	2	1	4
Cambodia	Kien Svay	1.3	0.9	1.8	28	19	39	0.2	0.1	0.3	3	2	5
Cambodia	Kiri Sakor	1.0	0.6	1.5	1	0	1	0.1	0.1	0.2	0	0	0
Cambodia	Kiri Vong	1.1	0.7	1.6	16	11	22	0.2	0.1	0.3	2	1	3
Cambodia	Kong Pisei	1.2	0.8	1.6	18	12	25	0.2	0.1	0.3	2	1	4
Cambodia	Koun Mom	1.4	0.9	2.1	3	2	4	0.2	0.1	0.3	1	0	1
Cambodia	Kracheh	1.5	1.0	2.2	20	13	29	0.2	0.1	0.3	2	1	3
Cambodia	Krakor	1.5	1.0	2.1	18	12	26	0.2	0.1	0.3	2	1	3
Cambodia	Kralanh	1.3	0.8	1.9	12	8	17	0.2	0.1	0.3	1	1	2
Cambodia	Krouch Chhmar	1.5	1.0	2.2	23	15	34	0.2	0.1	0.3	2	1	3
Cambodia	Kuleaen	1.2	0.8	1.8	3	2	5	0.2	0.1	0.2	2	1	2
Cambodia	Leuk Daek	1.2	0.8	1.8	12	8	18	0.2	0.1	0.3	1	1	2
Cambodia	Lumphat	1.5	0.9	2.2	3	2	5	0.2	0.1	0.3	1	1	1
Cambodia	Lvea Aem	1.3	0.9	1.9	16	11	24	0.2	0.1	0.3	1	1	2
Cambodia	Malai	1.1	0.7	1.7	4	3	6	0.2	0.1	0.3	1	1	2
Cambodia	Me Sang	1.2	0.8	1.8	16	10	24	0.2	0.1	0.3	1	1	2
Cambodia	Mean Chey	1.3	0.9	1.9	44	29	64	0.2	0.1	0.3	6	4	9
Cambodia	Memot	1.2	0.8	1.9	21	14	32	0.2	0.1	0.3	3	2	5
Cambodia	Mittakpheap	1.0	0.6	1.6	10	6	15	0.1	0.1	0.2	2	1	2
Cambodia	Mondol Seima	1.0	0.7	1.6	3	2	4	0.1	0.1	0.2	0	0	1
Cambodia	Mongkol Borei	1.3	0.8	1.9	28	18	40	0.2	0.1	0.3	3	2	4
Cambodia	Moung Ruessei	1.3	0.8	1.9	28	18	41	0.2	0.1	0.3	5	3	8
Cambodia	Mukh Kampul	1.4	0.9	2.0	15	10	22	0.2	0.1	0.3	2	1	3
Cambodia	Odongk	1.3	0.9	1.9	21	14	30	0.2	0.1	0.3	2	1	4
Cambodia	Ou Chrov	1.1	0.7	1.5	15	9	22	0.1	0.1	0.2	3	2	5
Cambodia	Ou Chum	1.3	0.9	2.0	3	2	5	0.2	0.1	0.3	1	0	1
Cambodia	Ou Reang	1.3	0.8	1.8	1	1	2	0.2	0.1	0.2	0	0	0
Cambodia	Ou Reang Ov	1.3	0.9	1.9	17	11	25	0.2	0.1	0.3	1	1	2
Cambodia	Ou Ya Dav	1.4	0.9	2.1	4	2	5	0.2	0.1	0.3	1	0	1
Cambodia	Pailin	1.1	0.7	1.7	3	2	5	0.2	0.1	0.3	2	1	3
Cambodia	Pea Reang	1.3	0.8	1.9	21	14	31	0.2	0.1	0.3	2	1	3
Cambodia	Peam Chor	1.2	0.8	1.8	13	8	19	0.2	0.1	0.3	1	1	2
Cambodia	Peam Ro	1.3	0.8	1.9	10	7	15	0.2	0.1	0.3	1	1	1
Cambodia	Pechr Chenda	1.5	0.9	2.1	2	1	3	0.2	0.1	0.3	1	0	1
Cambodia	Phnom Penh	1.3	0.9	1.9	46	31	68	0.2	0.1	0.3	5	3	7

Table 3: Diarrhoea Mortality by Unit *(continued)*

Country	Unit	2000 mortality mean rate (per 10,000)	lower rate (per 10,000)	upper rate (per 10,000)	mean count	lower count	upper count	2017 mortality mean rate (per 10,000)	lower rate (per 10,000)	upper rate (per 10,000)	mean count	lower count	upper count
Cambodia	Phnum Kravanh	1.4	0.9	2.1	13	9	19	0.2	0.1	0.3	1	1	2
Cambodia	Phnum Proek	1.1	0.7	1.7	8	5	12	0.2	0.1	0.2	7	4	11
Cambodia	Phnum Srok	1.3	0.9	1.9	9	6	13	0.2	0.1	0.3	1	1	1
Cambodia	Phnum Sruoch	1.1	0.8	1.6	14	9	20	0.2	0.1	0.3	2	1	4
Cambodia	Ponhea Kraek	1.3	0.8	1.9	25	17	36	0.2	0.1	0.3	3	2	4
Cambodia	Ponhea Lueu	1.4	0.9	2.0	18	12	26	0.2	0.1	0.3	2	1	3
Cambodia	Prasat Bakong	1.3	0.9	1.9	13	9	19	0.2	0.1	0.3	2	1	3
Cambodia	Prasat Balangk	1.4	0.9	2.0	9	6	14	0.2	0.1	0.3	1	1	2
Cambodia	Prasat Sambour	1.4	0.9	2.0	8	5	12	0.2	0.1	0.3	1	0	1
Cambodia	Preaek Prasab	1.5	1.0	2.2	15	10	23	0.2	0.1	0.3	1	1	2
Cambodia	Preah Netr Preah	1.3	0.9	1.9	15	10	22	0.2	0.1	0.3	2	1	3
Cambodia	Preah Sdach	1.2	0.8	1.8	19	12	28	0.2	0.1	0.3	2	1	3
Cambodia	Prey Chhor	1.4	0.9	2.1	28	18	41	0.2	0.1	0.3	2	1	4
Cambodia	Prey Kabbas	1.2	0.8	1.8	15	10	22	0.2	0.1	0.3	2	1	3
Cambodia	Prey Nob	1.0	0.6	1.6	12	7	19	0.1	0.1	0.2	2	1	3
Cambodia	Prey Veaeng	1.2	0.8	1.8	16	10	24	0.2	0.1	0.3	2	1	3
Cambodia	Puok	1.3	0.8	1.8	25	16	36	0.2	0.1	0.3	3	2	5
Cambodia	Rolea B'ier	1.5	1.0	2.2	22	15	32	0.2	0.1	0.3	2	1	3
Cambodia	Romeas Haek	1.2	0.8	1.8	19	13	29	0.2	0.1	0.3	2	1	3
Cambodia	Rotanak Mondol	1.1	0.7	1.7	5	3	8	0.2	0.1	0.3	1	1	2
Cambodia	Rovieng	1.3	0.8	1.9	7	4	10	0.2	0.1	0.3	1	1	2
Cambodia	Ruessei Kaev	1.3	0.9	1.9	38	25	55	0.2	0.1	0.3	5	3	9
Cambodia	Rumduol	1.2	0.8	1.8	8	6	12	0.2	0.1	0.3	1	0	1
Cambodia	S'ang	1.3	0.9	1.9	32	22	47	0.2	0.1	0.3	4	2	6
Cambodia	Saen Monourom	1.4	0.9	2.1	2	1	3	0.2	0.1	0.3	0	0	1
Cambodia	Sala Krau	1.1	0.7	1.7	2	1	3	0.2	0.1	0.3	3	2	4
Cambodia	Sambour	1.4	0.9	2.1	11	7	16	0.2	0.1	0.3	2	1	2
Cambodia	Sameakki Mean Chey	1.4	0.9	2.0	13	9	19	0.2	0.1	0.3	1	1	2
Cambodia	Samlout	1.2	0.7	1.8	4	2	6	0.2	0.1	0.3	1	1	2
Cambodia	Sampov Meas	1.5	1.0	2.2	14	10	21	0.2	0.1	0.3	1	1	2
Cambodia	Samraong	1.2	0.8	1.7	5	3	8	0.2	0.1	0.2	2	1	3
Cambodia	Samraong	1.2	0.8	1.7	18	12	26	0.2	0.1	0.3	2	1	3
Cambodia	Samraong Tong	1.2	0.8	1.7	25	17	36	0.2	0.1	0.3	3	2	5
Cambodia	Sandan	1.3	0.8	2.0	9	5	13	0.2	0.1	0.3	1	1	2
Cambodia	Sangkae	1.3	0.8	1.8	24	15	35	0.2	0.1	0.3	2	2	4
Cambodia	Sangkom Thmei	1.2	0.8	1.9	3	2	5	0.2	0.1	0.2	0	0	1
Cambodia	Santuk	1.4	0.9	2.1	14	9	21	0.2	0.1	0.3	2	1	3
Cambodia	Serei Saophoan	1.3	0.8	1.9	19	12	28	0.2	0.1	0.3	2	1	3
Cambodia	Sesan	1.4	0.9	2.1	3	2	5	0.2	0.1	0.3	1	0	1
Cambodia	Siem Bouk	1.4	0.9	2.1	3	2	4	0.2	0.1	0.3	1	0	1
Cambodia	Siem Pang	1.3	0.9	2.0	4	2	6	0.2	0.1	0.3	1	0	1
Cambodia	Siem Reab	1.3	0.8	1.9	29	18	42	0.2	0.1	0.3	7	4	10
Cambodia	Sithor Kandal	1.3	0.9	1.9	12	8	18	0.2	0.1	0.3	1	1	1
Cambodia	Smach Mean Chey	1.2	0.8	1.8	5	3	7	0.2	0.1	0.3	0	0	1
Cambodia	Snuol	1.3	0.9	2.0	9	6	14	0.2	0.1	0.3	2	1	3
Cambodia	Soutr Nikom	1.3	0.9	1.9	21	13	30	0.2	0.1	0.3	2	1	4
Cambodia	Srae Ambel	1.0	0.6	1.6	7	4	11	0.1	0.1	0.2	1	0	1
Cambodia	Srei Santhor	1.4	0.9	2.0	20	13	28	0.2	0.1	0.3	2	1	2
Cambodia	Srei Snam	1.3	0.9	1.8	6	4	9	0.2	0.1	0.3	1	1	1
Cambodia	Stoung	1.4	0.9	2.1	22	14	31	0.2	0.1	0.3	2	1	3
Cambodia	Stueng hav	1.0	0.6	1.6	1	1	2	0.1	0.1	0.2	0	0	0
Cambodia	Stueng Saen	1.5	1.0	2.2	16	10	23	0.2	0.1	0.3	1	1	2
Cambodia	Stueng Traeng	1.5	1.0	2.1	7	4	10	0.2	0.1	0.3	1	1	1
Cambodia	Stueng Trang	1.5	1.0	2.2	24	15	35	0.2	0.1	0.3	2	2	4
Cambodia	Svay Chek	1.3	0.8	1.8	10	6	14	0.2	0.1	0.3	1	1	2
Cambodia	Svay Chrum	1.2	0.8	1.7	23	16	35	0.2	0.1	0.3	2	1	4
Cambodia	Svay Leu	1.2	0.8	1.8	4	2	5	0.2	0.1	0.3	3	2	5
Cambodia	Svay Pao	1.3	0.8	1.9	24	16	36	0.2	0.1	0.3	3	2	4

Table 3: Diarrhoea Mortality by Unit *(continued)*

Country	Unit	2000 mortality						2017 mortality					
		mean rate (per 10,000)	lower rate (per 10,000)	upper rate (per 10,000)	mean count	lower count	upper count	mean rate (per 10,000)	lower rate (per 10,000)	upper rate (per 10,000)	mean count	lower count	upper count
Cambodia	Svay Rieng	1.2	0.8	1.8	2	1	3	0.2	0.1	0.3	0	0	0
Cambodia	Svay Teab	1.2	0.8	1.8	10	6	15	0.2	0.1	0.3	1	1	2
Cambodia	Ta Khmau	1.3	0.9	1.9	9	6	13	0.2	0.1	0.3	1	1	2
Cambodia	Ta Veaeng	1.3	0.8	1.9	1	1	2	0.2	0.1	0.3	0	0	0
Cambodia	Tbaeng Mean chey	1.2	0.8	1.8	5	3	8	0.2	0.1	0.2	1	1	1
Cambodia	Tboung Khmum	1.4	0.9	2.1	49	33	72	0.2	0.1	0.3	4	3	7
Cambodia	Thala Barivat	1.4	0.9	2.0	6	4	9	0.2	0.1	0.3	1	1	1
Cambodia	Thma Bang	1.1	0.7	1.6	1	0	1	0.2	0.1	0.2	0	0	0
Cambodia	Thma Puok	1.3	0.8	1.8	10	7	15	0.2	0.1	0.3	1	1	2
Cambodia	Thpong	1.3	0.9	1.9	9	6	12	0.2	0.1	0.3	1	1	2
Cambodia	Tram Kak	1.1	0.7	1.6	24	16	34	0.2	0.1	0.3	3	1	4
Cambodia	Treang	1.1	0.7	1.6	16	10	23	0.2	0.1	0.3	2	1	3
Cambodia	Tuek Phos	1.4	1.0	2.1	12	8	17	0.2	0.1	0.3	1	1	2
Cambodia	Varin	1.2	0.8	1.8	5	3	7	0.2	0.1	0.3	2	1	3
Cambodia	Veal Veaeng	1.3	0.8	1.9	2	1	3	0.2	0.1	0.3	0	0	1
Cambodia	Veun Sai	1.3	0.8	1.9	3	2	5	0.2	0.1	0.3	1	0	1
Indonesia	Aceh Barat	1.2	1.0	1.5	22	18	29	0.4	0.4	0.6	6	5	8
Indonesia	Aceh Barat Daya	1.2	1.0	1.5	15	12	18	0.4	0.4	0.5	5	4	6
Indonesia	Aceh Besar	1.1	0.8	1.3	49	39	61	0.4	0.3	0.5	23	18	29
Indonesia	Aceh Jaya	1.1	0.9	1.4	11	9	14	0.4	0.3	0.6	3	3	4
Indonesia	Aceh Selatan	1.2	1.0	1.4	29	24	35	0.4	0.4	0.5	10	8	12
Indonesia	Aceh Singkil	1.0	0.8	1.3	9	7	12	0.5	0.4	0.6	7	6	9
Indonesia	Aceh Tamiang	1.2	0.9	1.4	35	28	43	0.4	0.3	0.5	10	8	13
Indonesia	Aceh Tengah	1.3	1.1	1.6	26	22	32	0.5	0.4	0.6	11	8	13
Indonesia	Aceh Tenggara	1.2	1.0	1.5	21	17	25	0.5	0.4	0.6	10	8	12
Indonesia	Aceh Timur	1.3	1.0	1.5	57	46	69	0.4	0.3	0.6	17	14	21
Indonesia	Aceh Utara	1.3	1.1	1.6	88	71	107	0.5	0.4	0.6	29	23	35
Indonesia	Agam	1.1	0.9	1.3	62	50	76	0.5	0.4	0.7	26	20	32
Indonesia	Alor	1.4	1.1	1.7	30	24	36	0.7	0.6	0.9	15	12	18
Indonesia	Ambon	1.4	1.0	1.8	41	31	52	0.8	0.6	1.0	36	27	45
Indonesia	Asahan	1.3	1.0	1.5	107	83	130	0.5	0.4	0.6	38	31	45
Indonesia	Asmat	1.5	1.2	1.9	7	6	9	1.6	1.3	2.0	12	10	15
Indonesia	Badung	0.6	0.5	0.7	21	17	25	0.4	0.3	0.4	15	11	18
Indonesia	Balangan	1.6	1.3	1.9	15	12	18	0.6	0.5	0.8	6	5	8
Indonesia	Balikpapan	0.9	0.7	1.1	40	31	51	0.5	0.4	0.6	23	18	30
Indonesia	Banda Aceh	1.1	0.9	1.4	14	11	17	0.4	0.3	0.5	4	3	5
Indonesia	Bandar Lampung	1.1	0.9	1.3	61	49	77	0.4	0.3	0.5	29	23	36
Indonesia	Bandung	1.8	1.5	2.2	437	360	530	0.5	0.4	0.6	149	122	181
Indonesia	Bandung Barat	1.7	1.4	2.1	224	184	268	0.5	0.4	0.6	62	51	76
Indonesia	Banggai	2.2	1.7	2.7	68	53	85	0.8	0.6	1.0	23	18	28
Indonesia	Banggai Kepulauan	2.0	1.6	2.5	32	25	41	0.8	0.6	1.0	11	9	14
Indonesia	Bangka	0.9	0.7	1.1	18	14	23	0.3	0.2	0.4	8	6	9
Indonesia	Bangka Barat	0.8	0.7	1.0	13	10	16	0.3	0.3	0.4	5	4	7
Indonesia	Bangka Selatan	0.9	0.7	1.1	13	10	16	0.3	0.3	0.4	5	4	7
Indonesia	Bangka Tengah	0.9	0.7	1.1	20	15	24	0.3	0.3	0.4	8	6	10
Indonesia	Bangkalan	1.3	1.0	1.6	87	69	105	0.4	0.3	0.5	24	19	29
Indonesia	Bangli	0.6	0.5	0.7	12	10	15	0.3	0.3	0.4	5	4	6
Indonesia	Banjar	1.7	1.3	2.1	26	20	32	0.4	0.3	0.5	6	4	7
Indonesia	Banjar	1.8	1.4	2.1	106	86	127	0.7	0.5	0.8	37	29	46
Indonesia	Banjar Baru	1.7	1.4	2.1	23	18	27	0.6	0.5	0.8	13	10	17
Indonesia	Banjarmasin	1.9	1.5	2.3	87	68	105	0.8	0.6	1.0	28	21	38
Indonesia	Banjarnegara	0.9	0.8	1.1	73	60	86	0.4	0.3	0.5	23	19	29
Indonesia	Bantaeng	1.1	0.9	1.4	17	13	21	0.5	0.4	0.6	6	5	8
Indonesia	Bantul	0.5	0.4	0.6	35	28	42	0.2	0.2	0.3	15	12	19
Indonesia	Banyu Asin	1.3	1.1	1.6	77	63	95	0.4	0.3	0.5	23	18	28
Indonesia	Banyumas	0.9	0.8	1.2	128	104	156	0.4	0.3	0.5	45	36	57
Indonesia	Banyuwangi	1.8	1.4	2.2	220	175	267	0.5	0.4	0.6	47	35	58
Indonesia	Barito Kuala	1.8	1.4	2.1	61	49	72	0.7	0.5	0.9	20	15	25

Country	Unit	2000 mortality mean rate (per 10,000)	lower rate (per 10,000)	upper rate (per 10,000)	mean count	lower count	upper count	2017 mortality mean rate (per 10,000)	lower rate (per 10,000)	upper rate (per 10,000)	mean count	lower count	upper count
Indonesia	Barito Selatan	1.1	0.9	1.3	16	13	20	0.4	0.4	0.5	6	5	8
Indonesia	Barito Timur	1.1	0.9	1.4	8	7	10	0.4	0.4	0.5	4	3	5
Indonesia	Barito Utara	1.3	1.0	1.6	15	12	19	0.5	0.4	0.6	5	4	6
Indonesia	Barru	1.2	0.9	1.5	24	19	30	0.5	0.4	0.6	8	6	10
Indonesia	Batam	0.9	0.7	1.1	66	52	82	0.4	0.4	0.6	62	48	80
Indonesia	Batang	0.9	0.7	1.1	65	53	80	0.4	0.3	0.5	24	19	29
Indonesia	Batang Hari	1.2	0.9	1.4	21	17	26	0.3	0.3	0.4	6	5	8
Indonesia	Batu	1.5	1.2	1.8	21	17	25	0.5	0.4	0.6	5	4	7
Indonesia	Batu Bara	1.3	1.0	1.6	60	48	73	0.5	0.4	0.6	20	16	24
Indonesia	Bau-Bau	1.8	1.4	2.3	23	18	30	0.9	0.7	1.1	13	10	16
Indonesia	Bekasi	1.5	1.2	1.8	269	223	329	0.5	0.4	0.6	136	109	168
Indonesia	Belitung	0.9	0.7	1.1	13	10	17	0.3	0.3	0.4	5	4	6
Indonesia	Belitung Timur	0.9	0.7	1.2	8	6	10	0.3	0.3	0.4	3	2	4
Indonesia	Belu	1.4	1.1	1.7	54	45	66	0.8	0.7	1.0	33	28	40
Indonesia	Bener Meriah	1.3	1.1	1.6	15	12	18	0.5	0.4	0.6	6	5	8
Indonesia	Bengkalis	1.3	1.0	1.6	58	45	72	0.3	0.2	0.4	18	14	22
Indonesia	Bengkayang	1.6	1.3	2.0	37	30	46	0.6	0.4	0.7	14	11	17
Indonesia	Bengkulu	1.4	1.1	1.7	43	33	53	0.4	0.3	0.5	11	8	14
Indonesia	Bengkulu Selatan	1.3	1.0	1.7	17	13	21	0.3	0.3	0.4	5	4	6
Indonesia	Bengkulu Tengah	1.4	1.1	1.7	13	10	16	0.4	0.3	0.4	3	2	4
Indonesia	Bengkulu Utara	1.4	1.1	1.7	35	28	42	0.4	0.3	0.4	8	7	10
Indonesia	Berau	0.9	0.7	1.1	12	10	16	0.4	0.3	0.6	8	6	10
Indonesia	Biak Numfor	1.2	0.9	1.6	19	14	24	1.4	1.2	1.9	17	13	22
Indonesia	Bima	2.1	1.7	2.6	98	77	120	0.7	0.5	0.9	33	25	40
Indonesia	Bintan	0.9	0.7	1.1	29	23	36	0.4	0.3	0.6	13	10	16
Indonesia	Bireuen	1.3	1.1	1.7	55	45	71	0.5	0.4	0.5	21	17	26
Indonesia	Bitung	0.8	0.7	1.1	8	6	10	0.6	0.4	0.7	4	3	6
Indonesia	Blitar	1.3	1.1	1.6	129	106	156	0.4	0.3	0.5	31	25	39
Indonesia	Blora	0.8	0.6	1.0	59	47	71	0.4	0.3	0.5	20	16	26
Indonesia	Boalemo	2.4	1.9	3.0	28	23	35	0.9	0.7	1.2	13	10	16
Indonesia	Bogor	1.4	1.1	1.6	496	405	601	0.4	0.3	0.5	211	168	261
Indonesia	Bojonegoro	1.4	1.2	1.7	156	128	190	0.4	0.4	0.6	37	29	46
Indonesia	Bolaang Mongondow	0.9	0.7	1.1	22	18	27	0.6	0.5	0.8	10	8	13
Indonesia	Bolaang Mongondow Selatan	1.0	0.8	1.2	5	4	6	0.6	0.5	0.8	2	2	3
Indonesia	Bolaang Mongondow Timur	0.9	0.7	1.1	6	5	7	0.6	0.5	0.8	3	2	3
Indonesia	Bolaang Mongondow Utara	1.0	0.8	1.2	7	6	9	0.6	0.5	0.8	3	2	4
Indonesia	Bombana	1.7	1.4	2.1	20	16	24	0.9	0.7	1.0	17	14	20
Indonesia	Bondowoso	1.7	1.3	2.1	103	79	123	0.5	0.4	0.7	23	18	30
Indonesia	Bone	1.1	0.9	1.4	94	75	115	0.5	0.4	0.6	33	25	42
Indonesia	Bone Bolango	2.4	1.9	3.0	61	49	76	0.9	0.7	1.2	29	23	37
Indonesia	Bontang	0.9	0.7	1.1	6	4	7	0.5	0.4	0.6	4	3	5
Indonesia	Boven Digoel	1.5	1.2	1.9	8	6	10	1.7	1.4	2.1	14	11	17
Indonesia	Boyolali	0.7	0.6	0.9	62	51	76	0.3	0.3	0.4	22	18	26
Indonesia	Brebes	1.0	0.8	1.2	166	137	200	0.5	0.4	0.5	55	45	66
Indonesia	Bukittinggi	1.1	0.9	1.4	6	5	8	0.6	0.4	0.7	3	2	3
Indonesia	Buleleng	0.6	0.5	0.7	33	27	39	0.4	0.3	0.5	15	11	18
Indonesia	Bulukumba	1.1	0.9	1.4	51	40	63	0.5	0.4	0.6	18	14	23
Indonesia	Bulungan	0.7	0.6	0.9	11	9	14	0.4	0.3	0.5	6	5	8
Indonesia	Bungo	1.2	0.9	1.5	30	24	39	0.4	0.3	0.5	11	9	14
Indonesia	Buol	2.2	1.8	2.7	28	22	34	0.8	0.6	1.0	11	9	14
Indonesia	Buru	1.4	1.1	1.8	17	13	21	0.8	0.6	1.0	11	9	15
Indonesia	Buru Selatan	1.4	1.0	1.7	9	7	12	0.7	0.6	0.9	6	4	7
Indonesia	Buton	1.9	1.5	2.4	50	39	63	0.9	0.7	1.1	26	21	32

Table 3: Diarrhoea Mortality by Unit *(continued)*

Country	Unit	2000 mortality mean rate (per 10,000)	2000 lower rate (per 10,000)	2000 upper rate (per 10,000)	2000 mean count	2000 lower count	2000 upper count	2017 mortality mean rate (per 10,000)	2017 lower rate (per 10,000)	2017 upper rate (per 10,000)	2017 mean count	2017 lower count	2017 upper count
Indonesia	Buton Utara	1.8	1.4	2.2	8	6	10	0.9	0.7	1.1	5	4	6
Indonesia	Ciamis	1.8	1.4	2.1	236	190	286	0.4	0.3	0.5	50	40	61
Indonesia	Cianjur	1.7	1.3	2.0	338	272	411	0.5	0.4	0.6	93	77	112
Indonesia	Cilacap	0.9	0.7	1.1	136	111	166	0.4	0.3	0.5	44	36	55
Indonesia	Cilegon	1.0	0.8	1.3	40	31	49	0.4	0.3	0.5	13	11	16
Indonesia	Cimahi	2.0	1.7	2.4	67	56	81	0.6	0.5	0.7	23	19	29
Indonesia	Cirebon	1.7	1.4	2.1	330	268	400	0.5	0.4	0.6	86	68	103
Indonesia	Dairi	1.3	1.1	1.6	45	36	56	0.5	0.4	0.7	16	13	20
Indonesia	Danau	1.1	0.9	1.3	1	1	1	0.5	0.4	0.6	0	0	1
Indonesia	Danau Limboto	2.5	2.0	3.1	3	2	4	0.9	0.7	1.2	1	1	2
Indonesia	Deiyai	1.5	1.1	1.9	6	5	8	1.6	1.2	2.0	22	17	27
Indonesia	Deli Serdang	1.3	1.1	1.6	323	262	391	0.5	0.4	0.6	131	104	160
Indonesia	Demak	0.8	0.6	1.0	73	59	89	0.3	0.3	0.4	28	22	34
Indonesia	Denpasar	0.6	0.5	0.8	30	25	36	0.4	0.3	0.5	21	16	26
Indonesia	Depok	1.6	1.3	1.9	185	153	223	0.5	0.4	0.6	82	65	102
Indonesia	Dharmasraya	1.0	0.8	1.3	19	16	25	0.5	0.4	0.6	10	8	12
Indonesia	Dogiyai	1.5	1.1	1.9	12	9	15	1.6	1.2	2.0	19	15	25
Indonesia	Dompu	2.1	1.7	2.6	45	36	56	0.7	0.5	0.9	16	13	20
Indonesia	Donggala	2.2	1.8	2.6	53	44	63	0.9	0.7	1.1	23	18	28
Indonesia	Dumai	1.3	1.0	1.7	34	26	45	0.3	0.2	0.4	13	10	17
Indonesia	Empat Lawang	1.4	1.1	1.7	28	22	34	0.5	0.4	0.6	10	8	12
Indonesia	Ende	1.3	1.1	1.6	35	28	44	0.7	0.6	0.9	18	14	22
Indonesia	Enrekang	1.3	1.0	1.6	26	21	33	0.5	0.4	0.7	10	8	12
Indonesia	Fakfak	0.6	0.5	0.8	7	5	8	0.4	0.3	0.5	2	2	3
Indonesia	Flores Timur	1.2	1.0	1.5	31	24	38	0.7	0.6	0.9	17	13	21
Indonesia	Garut	1.8	1.4	2.1	377	309	452	0.5	0.4	0.6	105	84	126
Indonesia	Gayo Lues	1.2	1.0	1.5	10	8	11	0.5	0.4	0.6	5	4	6
Indonesia	Gianyar	0.6	0.5	0.7	22	19	27	0.3	0.3	0.4	11	9	13
Indonesia	Gorontalo	2.4	2.0	3.0	48	39	59	0.9	0.7	1.2	20	16	24
Indonesia	Gorontalo Utara	2.4	1.9	2.9	24	20	30	0.9	0.7	1.1	11	9	13
Indonesia	Gowa	1.3	1.0	1.6	105	86	132	0.5	0.4	0.6	45	35	56
Indonesia	Gresik	1.4	1.1	1.7	108	89	131	0.4	0.4	0.5	32	26	39
Indonesia	Grobogan	0.8	0.7	0.9	97	81	116	0.3	0.3	0.4	33	26	40
Indonesia	Gunung Kidul	0.5	0.4	0.6	25	21	30	0.2	0.2	0.3	9	8	12
Indonesia	Gunung Mas	1.4	1.1	1.7	16	12	20	0.5	0.4	0.6	7	5	8
Indonesia	Gunungsitoli	1.0	0.7	1.3	10	8	13	0.5	0.4	0.7	5	4	7
Indonesia	Halmahera Barat	0.9	0.6	1.1	9	7	12	0.7	0.5	0.9	9	7	12
Indonesia	Halmahera Selatan	0.9	0.7	1.2	16	13	21	0.7	0.5	0.8	14	11	17
Indonesia	Halmahera Tengah	0.9	0.7	1.1	4	3	4	0.7	0.5	0.8	2	2	3
Indonesia	Halmahera Timur	0.9	0.6	1.1	8	6	10	0.7	0.6	0.9	6	5	7
Indonesia	Halmahera Utara	0.9	0.6	1.1	13	10	16	0.7	0.5	0.9	12	9	15
Indonesia	Hulu Sungai Selatan	1.7	1.3	2.0	38	31	46	0.7	0.5	0.8	12	9	14
Indonesia	Hulu Sungai Tengah	1.6	1.3	2.0	44	35	53	0.6	0.5	0.8	13	10	16
Indonesia	Hulu Sungai Utara	1.5	1.2	1.8	35	29	43	0.6	0.5	0.8	10	8	13
Indonesia	Humbang Hasundutan	1.1	0.8	1.3	25	19	31	0.5	0.4	0.7	11	9	14
Indonesia	Indragiri Hilir	1.3	1.0	1.6	82	64	99	0.3	0.2	0.4	24	18	30
Indonesia	Indragiri Hulu	1.3	1.0	1.7	38	30	48	0.3	0.2	0.4	15	12	19
Indonesia	Indramayu	1.5	1.2	1.9	253	206	310	0.5	0.4	0.6	73	58	90
Indonesia	Intan Jaya	1.5	1.2	1.9	4	3	4	1.5	1.2	1.9	12	9	15
Indonesia	Jakarta Barat	0.7	0.6	0.8	127	107	151	0.4	0.3	0.5	75	61	95
Indonesia	Jakarta Pusat	0.7	0.6	0.8	56	47	67	0.4	0.4	0.6	29	23	36
Indonesia	Jakarta Selatan	0.7	0.6	0.8	130	109	156	0.4	0.3	0.5	73	58	91
Indonesia	Jakarta Timur	0.7	0.6	0.8	168	142	204	0.4	0.3	0.5	95	76	119
Indonesia	Jakarta Utara	0.7	0.6	0.8	103	86	123	0.4	0.3	0.5	54	43	67

Table 3: Diarrhoea Mortality by Unit *(continued)*

		2000 mortality						2017 mortality					
Country	Unit	mean rate (per 10,000)	lower rate (per 10,000)	upper rate (per 10,000)	mean count	lower count	upper count	mean rate (per 10,000)	lower rate (per 10,000)	upper rate (per 10,000)	mean count	lower count	upper count
Indonesia	Jambi	1.1	0.9	1.4	49	38	61	0.3	0.3	0.4	15	12	19
Indonesia	Jayapura	1.3	1.0	1.6	19	15	24	1.5	1.2	2.0	27	22	35
Indonesia	Jayawijaya	1.6	1.2	2.0	10	7	12	1.7	1.4	2.2	19	15	24
Indonesia	Jember	1.7	1.3	2.0	310	243	376	0.5	0.4	0.6	73	56	93
Indonesia	Jembrana	0.6	0.5	0.7	14	11	17	0.4	0.3	0.5	6	5	7
Indonesia	Jeneponto	1.3	1.0	1.6	51	41	63	0.5	0.4	0.7	17	13	22
Indonesia	Jepara	0.8	0.6	1.0	67	53	81	0.4	0.3	0.5	27	22	33
Indonesia	Jombang	1.4	1.2	1.7	137	115	167	0.4	0.4	0.5	33	27	41
Indonesia	Kaimana	0.6	0.5	0.8	4	3	5	0.4	0.3	0.5	2	2	3
Indonesia	Kampar	1.3	1.1	1.6	79	64	96	0.3	0.2	0.4	37	29	44
Indonesia	Kapuas	1.2	1.1	1.5	43	36	50	0.4	0.4	0.5	11	9	14
Indonesia	Kapuas Hulu	1.8	1.4	2.2	41	32	50	0.6	0.5	0.8	14	11	18
Indonesia	Karanganyar	0.6	0.5	0.8	43	35	52	0.3	0.2	0.4	15	12	19
Indonesia	Karangasem	0.6	0.5	0.7	20	17	26	0.3	0.3	0.4	8	6	10
Indonesia	Karawang	1.3	1.1	1.5	219	178	262	0.4	0.3	0.5	80	63	99
Indonesia	Karimun	0.9	0.7	1.1	16	12	20	0.5	0.3	0.6	6	5	8
Indonesia	Karo	1.4	1.1	1.7	51	42	63	0.5	0.4	0.6	22	17	26
Indonesia	Katingan	1.4	1.1	1.6	18	15	21	0.4	0.3	0.5	6	5	8
Indonesia	Kaur	1.3	1.0	1.6	12	10	16	0.3	0.2	0.4	3	2	4
Indonesia	Kayong Utara	1.7	1.4	2.1	15	12	18	0.6	0.4	0.7	6	4	7
Indonesia	Kebumen	0.8	0.7	1.0	87	70	105	0.4	0.3	0.4	27	21	33
Indonesia	Kediri	1.4	1.1	1.6	170	141	205	0.4	0.3	0.5	42	33	50
Indonesia	Keerom	1.3	1.0	1.7	6	5	8	1.5	1.2	1.9	8	7	11
Indonesia	Kendal	0.8	0.6	0.9	63	50	75	0.4	0.3	0.5	24	19	30
Indonesia	Kendari	1.7	1.3	2.1	48	37	59	0.9	0.7	1.1	34	27	43
Indonesia	Kepahiang	1.3	1.1	1.6	20	16	24	0.4	0.3	0.4	5	4	6
Indonesia	Kepulauan Anambas	0.9	0.7	1.2	1	1	2	0.5	0.3	0.6	1	0	1
Indonesia	Kepulauan Aru	1.3	1.0	1.7	13	10	17	0.7	0.6	0.9	7	6	9
Indonesia	Kepulauan Mentawai	1.1	0.8	1.3	8	6	10	0.5	0.4	0.6	3	2	4
Indonesia	Kepulauan Meranti	1.3	1.0	1.7	20	15	27	0.3	0.2	0.4	6	5	8
Indonesia	Kepulauan Sangihe	0.9	0.7	1.2	7	6	9	0.6	0.5	0.8	3	2	3
Indonesia	Kepulauan Selayar	1.2	1.0	1.5	14	11	17	0.5	0.4	0.6	5	4	6
Indonesia	Kepulauan Seribu	0.7	0.6	0.8	0	0	0	0.3	0.3	0.4	0	0	0
Indonesia	Kepulauan Sula	0.9	0.7	1.1	11	9	14	0.7	0.5	0.8	10	8	12
Indonesia	Kepulauan Talaud	0.9	0.7	1.1	11	8	13	0.6	0.5	0.7	4	3	4
Indonesia	Kepulauan Yapen	1.3	1.0	1.7	8	6	10	1.4	1.1	1.8	11	8	14
Indonesia	Kerinci	1.4	1.1	1.7	35	28	43	0.4	0.3	0.5	8	6	10
Indonesia	Ketapang	1.7	1.4	2.0	70	57	83	0.6	0.5	0.7	26	21	33
Indonesia	Klaten	0.6	0.5	0.8	62	52	75	0.3	0.2	0.4	21	17	25
Indonesia	Klungkung	0.6	0.5	0.7	10	8	12	0.3	0.3	0.4	4	3	5
Indonesia	Kolaka	1.7	1.3	2.1	55	43	67	0.9	0.7	1.1	35	28	46
Indonesia	Kolaka Utara	1.9	1.5	2.3	19	16	24	0.9	0.7	1.1	16	13	20
Indonesia	Konawe	1.7	1.4	2.1	40	32	47	0.9	0.7	1.1	20	16	25
Indonesia	Konawe Selatan	1.7	1.3	2.0	55	43	66	0.9	0.7	1.1	32	26	40
Indonesia	Konawe Utara	1.8	1.4	2.3	7	6	9	0.9	0.7	1.1	4	3	5
Indonesia	Kota Bandung	2.1	1.7	2.5	549	457	660	0.6	0.5	0.7	161	129	201
Indonesia	Kota Baru	1.8	1.4	2.1	48	39	58	0.6	0.5	0.8	18	14	22
Indonesia	Kota Bekasi	1.8	1.5	2.2	280	234	340	0.5	0.4	0.7	113	90	143
Indonesia	Kota Bima	2.3	1.8	2.8	27	22	34	0.7	0.5	0.9	9	7	11
Indonesia	Kota Binjai	1.4	1.1	1.7	40	33	49	0.5	0.4	0.7	16	12	19
Indonesia	Kota Blitar	1.3	1.1	1.6	9	8	12	0.4	0.3	0.5	2	2	3
Indonesia	Kota Bogor	1.6	1.3	2.0	106	86	129	0.5	0.4	0.7	40	31	52
Indonesia	Kota Cirebon	1.9	1.6	2.4	66	54	80	0.6	0.4	0.7	18	14	22
Indonesia	Kota Gorontalo	2.5	2.0	3.1	31	25	38	1.0	0.8	1.2	13	10	16

Table 3: Diarrhoea Mortality by Unit *(continued)*

Country	Unit	2000 mortality mean rate (per 10,000)	2000 mortality lower rate (per 10,000)	2000 mortality upper rate (per 10,000)	2000 mortality mean count	2000 mortality lower count	2000 mortality upper count	2017 mortality mean rate (per 10,000)	2017 mortality lower rate (per 10,000)	2017 mortality upper rate (per 10,000)	2017 mortality mean count	2017 mortality lower count	2017 mortality upper count
Indonesia	Kota Jayapura	1.3	1.0	1.7	34	26	44	1.5	1.2	2.0	41	33	55
Indonesia	Kota Kediri	1.4	1.1	1.7	26	21	31	0.4	0.4	0.5	7	5	8
Indonesia	Kota Kupang	1.3	1.0	1.6	42	33	52	0.7	0.6	0.9	31	25	40
Indonesia	Kota Madiun	1.3	1.1	1.6	15	12	18	0.4	0.3	0.5	4	3	5
Indonesia	Kota Magelang	0.7	0.6	0.9	6	5	7	0.3	0.3	0.4	2	2	2
Indonesia	Kota Malang	1.5	1.3	1.9	105	86	127	0.5	0.4	0.7	29	23	38
Indonesia	Kota Medan	1.4	1.1	1.7	298	240	361	0.6	0.5	0.7	111	88	135
Indonesia	Kota Mojokerto	1.4	1.1	1.7	11	9	13	0.5	0.4	0.6	3	2	4
Indonesia	Kota Pasuruan	1.6	1.3	2.0	18	14	23	0.5	0.4	0.7	5	4	6
Indonesia	Kota Pekalongan	0.9	0.8	1.2	15	12	18	0.4	0.3	0.5	5	4	7
Indonesia	Kota Pontianak	1.6	1.2	1.9	56	44	71	0.6	0.4	0.7	19	14	25
Indonesia	Kota Probolinggo	1.7	1.3	2.1	22	18	28	0.6	0.4	0.7	6	5	8
Indonesia	Kota Semarang	0.8	0.7	1.0	106	85	128	0.4	0.3	0.5	46	37	58
Indonesia	Kota Serang	1.0	0.7	1.2	70	55	89	0.4	0.3	0.5	22	18	27
Indonesia	Kota Solok	1.0	0.8	1.3	4	3	5	0.5	0.4	0.6	2	1	2
Indonesia	Kota Sorong	0.7	0.5	0.9	11	8	14	0.4	0.3	0.5	5	4	7
Indonesia	Kota Sukabumi	1.8	1.4	2.2	40	32	48	0.5	0.4	0.6	11	9	14
Indonesia	Kota Tangerang	1.3	1.1	1.6	231	192	278	0.5	0.4	0.6	76	62	96
Indonesia	Kota Tanjungbalai	1.3	1.0	1.7	18	14	22	0.5	0.4	0.6	6	5	8
Indonesia	Kota Tasikmalaya	1.8	1.4	2.2	92	73	112	0.5	0.4	0.6	24	18	29
Indonesia	Kota Tegal	1.1	0.9	1.3	16	13	20	0.5	0.4	0.6	6	5	7
Indonesia	Kota Yogyakarta	0.6	0.5	0.7	12	10	15	0.3	0.2	0.4	5	4	6
Indonesia	Kotamobagu	0.9	0.7	1.1	7	6	9	0.6	0.5	0.8	3	3	4
Indonesia	Kotawaringin Barat	1.4	1.1	1.7	23	18	29	0.4	0.3	0.5	9	7	12
Indonesia	Kotawaringin Timur	1.4	1.1	1.7	48	39	59	0.4	0.3	0.5	16	12	20
Indonesia	Kuantan Singingi	1.4	1.1	1.7	31	25	40	0.3	0.3	0.4	10	8	13
Indonesia	Kubu Raya	1.5	1.2	1.9	112	89	138	0.6	0.4	0.7	43	33	55
Indonesia	Kudus	0.8	0.6	0.9	52	42	62	0.4	0.3	0.5	21	16	26
Indonesia	Kulon Progo	0.5	0.4	0.7	16	13	19	0.3	0.2	0.3	6	5	7
Indonesia	Kuningan	1.8	1.4	2.1	160	128	194	0.4	0.3	0.5	34	27	42
Indonesia	Kupang	1.3	1.1	1.6	50	41	61	0.7	0.6	0.9	31	25	39
Indonesia	Kutai Barat	1.0	0.8	1.2	15	12	19	0.5	0.4	0.6	7	5	8
Indonesia	Kutai Kartanegara	0.9	0.7	1.1	47	38	57	0.5	0.4	0.6	29	24	35
Indonesia	Kutai Timur	0.9	0.7	1.1	19	16	24	0.5	0.4	0.6	15	12	18
Indonesia	Labuhanbatu	1.0	0.8	1.3	38	29	47	0.5	0.4	0.6	20	16	24
Indonesia	Labuhanbatu Selatan	0.9	0.7	1.2	26	20	33	0.5	0.4	0.6	15	12	19
Indonesia	Labuhanbatu Utara	1.1	0.9	1.4	52	40	64	0.5	0.4	0.6	24	19	29
Indonesia	Lahat	1.3	1.1	1.7	45	36	55	0.5	0.4	0.6	15	12	19
Indonesia	Lake Toba	1.2	1.0	1.5	5	4	6	0.5	0.4	0.6	2	2	2
Indonesia	Lamandau	1.5	1.2	1.9	11	8	14	0.4	0.3	0.6	4	3	5
Indonesia	Lamongan	1.4	1.1	1.7	139	114	169	0.4	0.4	0.5	31	24	37
Indonesia	Lampung Barat	1.0	0.8	1.3	42	35	53	0.4	0.3	0.4	13	11	16
Indonesia	Lampung Selatan	1.0	0.8	1.2	98	79	118	0.4	0.3	0.5	33	26	41
Indonesia	Lampung Tengah	0.9	0.8	1.2	132	112	161	0.3	0.3	0.4	42	34	52
Indonesia	Lampung Timur	1.0	0.8	1.2	100	81	122	0.4	0.3	0.4	29	24	36
Indonesia	Lampung Utara	0.9	0.8	1.2	61	50	75	0.3	0.3	0.4	18	14	22
Indonesia	Landak	1.6	1.3	1.9	53	42	65	0.6	0.4	0.7	19	15	23
Indonesia	Langkat	1.4	1.1	1.6	168	140	201	0.5	0.4	0.7	57	45	70
Indonesia	Langsa	1.3	1.0	1.6	15	12	19	0.4	0.3	0.5	7	6	9
Indonesia	Lanny Jaya	1.7	1.4	2.1	14	12	18	1.7	1.3	2.1	34	27	42
Indonesia	Lebak	1.0	0.8	1.2	156	126	192	0.4	0.3	0.5	50	39	61
Indonesia	Lebong	1.4	1.1	1.7	14	11	17	0.3	0.3	0.4	3	3	4
Indonesia	Lembata	1.3	1.0	1.6	14	11	18	0.7	0.6	0.9	9	7	12
Indonesia	Lhokseumawe	1.4	1.1	1.7	16	13	20	0.5	0.4	0.6	6	5	8

Table 3: Diarrhoea Mortality by Unit *(continued)*

Country	Unit	2000 mortality mean rate (per 10,000)	lower rate (per 10,000)	upper rate (per 10,000)	mean count	lower count	upper count	2017 mortality mean rate (per 10,000)	lower rate (per 10,000)	upper rate (per 10,000)	mean count	lower count	upper count
Indonesia	Lima Puluh Kota	1.1	0.8	1.3	43	34	54	0.5	0.4	0.7	20	16	25
Indonesia	Lingga	0.8	0.7	1.1	11	9	14	0.4	0.3	0.6	3	3	4
Indonesia	Lombok Barat	2.4	2.0	3.0	147	119	181	0.6	0.5	0.8	42	33	53
Indonesia	Lombok Tengah	2.5	2.0	3.0	215	176	266	0.6	0.5	0.8	57	45	70
Indonesia	Lombok Timur	2.6	2.1	3.2	309	248	387	0.7	0.5	0.8	77	59	96
Indonesia	Lombok Utara	2.5	2.0	3.1	47	38	59	0.6	0.5	0.8	13	10	17
Indonesia	Lubuklinggau	1.3	1.0	1.6	19	15	24	0.5	0.4	0.6	7	5	9
Indonesia	Lumajang	1.6	1.3	1.9	129	104	160	0.5	0.4	0.6	30	24	38
Indonesia	Luwu	1.3	1.1	1.6	43	36	53	0.5	0.4	0.6	17	13	21
Indonesia	Luwu Timur	1.4	1.1	1.7	39	32	47	0.6	0.4	0.7	16	12	19
Indonesia	Luwu Utara	1.4	1.1	1.7	42	34	52	0.6	0.4	0.7	17	13	21
Indonesia	Madiun	1.3	1.1	1.6	85	70	103	0.4	0.3	0.5	20	16	25
Indonesia	Magelang	0.7	0.6	0.9	75	61	91	0.3	0.3	0.4	27	22	33
Indonesia	Magetan	1.1	0.9	1.3	55	45	68	0.3	0.3	0.4	12	10	15
Indonesia	Majalengka	1.7	1.4	2.1	204	167	247	0.5	0.4	0.6	48	38	57
Indonesia	Majene	2.4	1.8	3.0	37	28	46	0.7	0.5	0.8	11	9	14
Indonesia	Makassar	1.5	1.2	1.8	153	124	190	0.6	0.5	0.7	58	46	73
Indonesia	Malang	1.4	1.1	1.6	262	213	314	0.5	0.4	0.6	70	57	89
Indonesia	Malinau	0.8	0.7	1.0	6	5	8	0.4	0.3	0.5	4	3	5
Indonesia	Maluku Barat Daya	1.5	1.2	1.8	14	11	16	0.9	0.8	1.1	7	5	8
Indonesia	Maluku Tengah	1.3	1.0	1.5	59	48	71	0.7	0.6	0.9	34	27	42
Indonesia	Maluku Tenggara	1.4	1.0	1.7	22	17	28	0.8	0.6	1.0	6	4	7
Indonesia	Maluku Tenggara Barat	1.5	1.1	1.9	19	15	25	0.8	0.7	1.1	8	6	11
Indonesia	Mamasa	2.5	1.9	3.2	43	33	55	0.7	0.5	0.8	12	10	16
Indonesia	Mamberamo Raya	1.3	1.1	1.6	4	3	5	1.4	1.2	1.7	6	5	7
Indonesia	Mamberamo Tengah	1.4	1.1	1.8	16	12	20	1.5	1.2	1.9	31	25	40
Indonesia	Mamuju	2.4	1.9	3.0	85	67	106	0.7	0.6	0.8	33	26	40
Indonesia	Mamuju Utara	2.1	1.7	2.7	21	16	26	0.6	0.5	0.8	13	10	16
Indonesia	Manado	0.9	0.7	1.1	29	23	37	0.6	0.5	0.7	11	9	14
Indonesia	Mandailing Natal	1.1	0.8	1.4	52	40	66	0.5	0.4	0.7	24	19	30
Indonesia	Manggarai	1.5	1.2	1.9	47	38	57	0.8	0.7	1.0	28	22	34
Indonesia	Manggarai Barat	1.6	1.2	1.8	37	30	44	0.9	0.7	1.0	23	18	27
Indonesia	Manggarai Timur	1.4	1.2	1.7	40	32	49	0.8	0.6	1.0	25	20	31
Indonesia	Manokwari	0.6	0.5	0.8	12	9	15	0.4	0.3	0.4	8	7	11
Indonesia	Mappi	1.4	1.1	1.8	12	9	15	1.5	1.2	1.8	20	16	25
Indonesia	Maros	1.2	1.0	1.5	47	38	58	0.5	0.4	0.6	18	14	22
Indonesia	Mataram	2.6	2.1	3.2	82	66	101	0.7	0.5	0.9	26	20	33
Indonesia	Maybrat	0.7	0.5	0.8	2	1	2	0.4	0.3	0.5	2	1	2
Indonesia	Melawi	1.7	1.4	2.2	37	30	47	0.6	0.5	0.8	13	10	16
Indonesia	Merangin	1.3	1.0	1.5	38	30	46	0.4	0.3	0.5	12	10	15
Indonesia	Merauke	1.4	1.1	1.7	25	20	30	1.6	1.3	2.0	44	36	54
Indonesia	Mesuji	1.0	0.8	1.2	13	11	17	0.3	0.3	0.4	4	3	5
Indonesia	Metro	0.9	0.8	1.2	11	9	14	0.3	0.3	0.4	4	3	5
Indonesia	Mimika	1.4	1.1	1.8	12	9	15	1.6	1.2	2.0	72	56	91
Indonesia	Minahasa	0.8	0.7	1.0	31	24	39	0.6	0.4	0.7	17	13	21
Indonesia	Minahasa Selatan	0.8	0.7	1.0	14	11	18	0.6	0.5	0.7	9	7	11
Indonesia	Minahasa Tenggara	0.8	0.7	1.0	7	5	8	0.6	0.5	0.7	4	4	6
Indonesia	Minahasa Utara	0.8	0.7	1.1	15	12	20	0.6	0.4	0.7	9	7	11
Indonesia	Mojokerto	1.3	1.1	1.6	108	91	130	0.4	0.3	0.5	30	24	38
Indonesia	Morowali	1.9	1.6	2.3	40	33	48	0.8	0.6	1.0	17	14	21
Indonesia	Muara Enim	1.3	1.1	1.5	90	73	107	0.4	0.3	0.5	33	27	43
Indonesia	Muaro Jambi	1.1	0.9	1.4	43	35	54	0.3	0.3	0.4	15	12	19
Indonesia	Mukomuko	1.5	1.2	1.9	25	20	31	0.4	0.3	0.5	6	5	7
Indonesia	Muna	1.7	1.3	2.2	55	42	68	0.8	0.7	1.0	25	20	30
Indonesia	Murung Raya	1.4	1.1	1.8	13	10	15	0.5	0.4	0.6	5	4	6

Table 3: Diarrhoea Mortality by Unit *(continued)*

Country	Unit	2000 mortality mean rate (per 10,000)	2000 lower rate (per 10,000)	2000 upper rate (per 10,000)	2000 mean count	2000 lower count	2000 upper count	2017 mortality mean rate (per 10,000)	2017 lower rate (per 10,000)	2017 upper rate (per 10,000)	2017 mean count	2017 lower count	2017 upper count
Indonesia	Musi Banyuasin	1.3	1.0	1.5	71	56	86	0.4	0.3	0.5	22	17	27
Indonesia	Musi Rawas	1.3	1.1	1.6	71	58	86	0.5	0.4	0.6	26	20	33
Indonesia	Nabire	1.3	1.0	1.7	16	12	21	1.6	1.2	2.0	26	20	33
Indonesia	Nagan Raya	1.2	1.0	1.5	22	18	27	0.4	0.4	0.6	7	6	9
Indonesia	Nagekeo	1.3	1.1	1.6	22	18	27	0.8	0.6	1.0	13	10	16
Indonesia	Natuna	0.9	0.7	1.1	6	4	7	0.4	0.3	0.6	2	2	3
Indonesia	Nduga	1.6	1.2	2.0	10	8	12	1.7	1.3	2.1	25	20	31
Indonesia	Ngada	1.4	1.1	1.7	23	19	29	0.8	0.6	1.0	13	11	17
Indonesia	Nganjuk	1.4	1.1	1.7	113	93	138	0.4	0.3	0.5	26	20	32
Indonesia	Ngawi	1.3	1.1	1.5	94	78	113	0.4	0.3	0.5	21	17	26
Indonesia	Nias	1.0	0.7	1.3	18	14	24	0.5	0.4	0.6	9	7	11
Indonesia	Nias Barat	1.0	0.7	1.2	7	5	9	0.5	0.4	0.6	3	3	4
Indonesia	Nias Selatan	1.0	0.8	1.3	33	26	43	0.5	0.4	0.7	15	12	18
Indonesia	Nias Utara	1.0	0.8	1.3	12	9	16	0.5	0.4	0.7	6	5	8
Indonesia	Nunukan	0.7	0.6	0.9	11	8	13	0.4	0.3	0.4	7	6	9
Indonesia	Ogan Ilir	1.3	1.1	1.6	57	46	70	0.4	0.3	0.5	18	15	22
Indonesia	Ogan Komering Ilir	1.3	1.0	1.5	85	70	102	0.4	0.3	0.5	27	22	34
Indonesia	Ogan Komering Ulu	1.3	1.0	1.6	35	28	44	0.4	0.3	0.5	11	8	13
Indonesia	Ogan Komering Ulu Selatan	1.3	1.1	1.6	38	30	47	0.5	0.3	0.6	13	10	15
Indonesia	Ogan Komering Ulu Timur	1.3	1.0	1.6	85	67	105	0.4	0.3	0.5	27	21	35
Indonesia	Pacitan	1.1	0.9	1.3	46	37	57	0.3	0.3	0.4	10	8	13
Indonesia	Padang	1.2	0.9	1.4	112	90	140	0.6	0.5	0.7	50	39	63
Indonesia	Padang Lawas	0.9	0.7	1.2	17	14	22	0.5	0.4	0.6	10	8	12
Indonesia	Padang Lawas Utara	0.9	0.7	1.2	29	22	36	0.5	0.4	0.6	18	14	22
Indonesia	Padang Panjang	1.1	0.9	1.4	3	2	4	0.5	0.4	0.7	1	1	1
Indonesia	Padang Pariaman	1.1	0.9	1.4	39	32	48	0.5	0.4	0.7	16	12	20
Indonesia	Padangsidimpuan	1.0	0.7	1.2	12	9	15	0.5	0.4	0.7	6	4	7
Indonesia	Pagar Alam	1.4	1.1	1.8	19	15	24	0.5	0.4	0.6	7	5	8
Indonesia	Pakpak Barat	1.2	0.9	1.4	5	4	6	0.5	0.4	0.7	2	1	2
Indonesia	Palangka Raya	1.2	1.0	1.5	20	16	24	0.4	0.3	0.5	7	6	9
Indonesia	Palembang	1.3	1.0	1.6	207	164	255	0.5	0.4	0.6	62	49	78
Indonesia	Palopo	1.3	1.1	1.7	21	16	26	0.5	0.4	0.7	8	6	10
Indonesia	Palu	2.2	1.8	2.7	56	46	69	0.9	0.7	1.2	23	18	29
Indonesia	Pamekasan	1.5	1.2	1.8	85	67	103	0.5	0.4	0.6	25	19	32
Indonesia	Pandeglang	1.0	0.8	1.2	135	107	171	0.4	0.3	0.5	42	33	52
Indonesia	Pangkajene Dan Kepulauan	1.2	1.0	1.6	41	33	51	0.5	0.4	0.6	14	11	19
Indonesia	Pangkalpinang	0.9	0.7	1.1	10	8	13	0.3	0.2	0.4	4	3	5
Indonesia	Paniai	1.6	1.3	2.0	15	12	19	1.6	1.2	2.0	44	33	55
Indonesia	Parepare	1.2	1.0	1.5	16	12	19	0.5	0.4	0.6	6	5	7
Indonesia	Pariaman	1.2	0.9	1.4	9	7	11	0.6	0.4	0.7	3	2	4
Indonesia	Parigi Moutong	2.2	1.8	2.6	85	70	100	0.8	0.7	1.0	31	25	37
Indonesia	Pasaman	1.1	0.9	1.3	31	25	38	0.5	0.4	0.6	14	11	17
Indonesia	Pasaman Barat	1.1	0.9	1.4	37	30	47	0.5	0.4	0.6	17	14	21
Indonesia	Paser	0.9	0.8	1.2	18	14	22	0.5	0.4	0.6	10	8	12
Indonesia	Pasuruan	1.5	1.2	1.8	179	146	221	0.5	0.4	0.6	48	37	60
Indonesia	Pati	0.8	0.6	1.0	86	70	103	0.4	0.3	0.5	31	25	38
Indonesia	Payakumbuh	1.1	0.8	1.3	12	9	15	0.6	0.4	0.7	5	4	6
Indonesia	Pegunungan Bintang	1.7	1.3	2.1	9	7	11	1.7	1.3	2.2	20	16	26
Indonesia	Pekalongan	0.9	0.8	1.1	73	59	88	0.4	0.3	0.5	25	20	31
Indonesia	Pekanbaru	1.3	1.0	1.6	71	56	87	0.3	0.2	0.4	29	22	35
Indonesia	Pelalawan	1.3	1.1	1.6	25	20	31	0.3	0.2	0.4	16	13	20
Indonesia	Pemalang	1.0	0.8	1.2	117	96	141	0.4	0.3	0.5	37	30	45
Indonesia	Pematangsiantar	1.4	1.1	1.7	44	36	54	0.5	0.4	0.7	12	9	15

Table 3: Diarrhoea Mortality by Unit *(continued)*

Country	Unit	2000 mortality mean rate (per 10,000)	lower rate (per 10,000)	upper rate (per 10,000)	mean count	lower count	upper count	2017 mortality mean rate (per 10,000)	lower rate (per 10,000)	upper rate (per 10,000)	mean count	lower count	upper count
Indonesia	Penajam Paser Utara	0.9	0.7	1.1	11	8	14	0.5	0.4	0.6	6	5	8
Indonesia	Pesawaran	1.0	0.8	1.2	52	42	64	0.3	0.3	0.4	16	13	20
Indonesia	Pesisir Selatan	1.1	0.9	1.3	51	41	62	0.5	0.4	0.7	22	17	27
Indonesia	Pidie	1.2	0.9	1.5	48	39	62	0.4	0.3	0.5	17	14	22
Indonesia	Pidie Jaya	1.2	1.0	1.6	19	15	25	0.4	0.3	0.5	7	5	9
Indonesia	Pinrang	1.2	0.9	1.5	49	38	61	0.5	0.4	0.6	18	14	22
Indonesia	Pohuwato	2.2	1.7	2.7	24	18	29	0.9	0.7	1.1	11	8	14
Indonesia	Polewali Mandar	2.4	1.9	3.0	113	88	140	0.7	0.5	0.8	29	24	37
Indonesia	Ponorogo	1.2	1.0	1.5	88	70	109	0.4	0.3	0.5	20	15	25
Indonesia	Pontianak	1.4	1.1	1.8	32	25	40	0.6	0.4	0.7	12	10	16
Indonesia	Poso	2.1	1.7	2.5	40	33	48	0.8	0.7	1.0	22	17	27
Indonesia	Prabumulih	1.3	1.0	1.6	14	11	18	0.4	0.3	0.5	6	5	8
Indonesia	Pringsewu	1.0	0.8	1.2	31	25	39	0.3	0.3	0.4	9	8	11
Indonesia	Probolinggo	1.6	1.3	2.0	139	111	172	0.5	0.4	0.6	36	29	45
Indonesia	Pulang Pisau	1.2	1.0	1.5	15	12	18	0.4	0.3	0.5	6	5	8
Indonesia	Pulau Morotai	0.9	0.6	1.1	4	3	5	0.7	0.5	0.9	4	3	5
Indonesia	Puncak	1.6	1.3	2.0	11	9	13	1.6	1.2	2.0	29	23	37
Indonesia	Puncak Jaya	1.5	1.2	1.9	11	9	14	1.5	1.1	1.9	23	18	29
Indonesia	Purbalingga	1.0	0.8	1.1	70	56	83	0.4	0.3	0.5	24	20	30
Indonesia	Purwakarta	1.5	1.2	1.8	111	89	135	0.5	0.4	0.6	37	30	46
Indonesia	Purworejo	0.8	0.6	0.9	51	41	61	0.3	0.3	0.4	16	13	19
Indonesia	Raja Ampat	0.4	0.3	0.5	3	2	3	0.2	0.2	0.3	1	1	1
Indonesia	Rejang Lebong	1.3	1.0	1.6	32	26	40	0.3	0.3	0.4	8	6	10
Indonesia	Rembang	0.8	0.7	1.0	41	34	51	0.4	0.3	0.5	16	12	20
Indonesia	Rokan Hilir	1.0	0.8	1.3	33	25	40	0.3	0.3	0.4	21	17	26
Indonesia	Rokan Hulu	1.2	0.9	1.5	34	27	43	0.3	0.3	0.4	21	17	26
Indonesia	Rote Ndao	1.3	1.0	1.6	16	13	20	0.7	0.6	0.9	10	8	12
Indonesia	Sabang	1.1	0.9	1.4	2	1	2	0.4	0.3	0.5	1	1	1
Indonesia	Sabu Raijua	1.4	1.1	1.8	10	7	13	0.8	0.6	1.1	6	5	8
Indonesia	Salatiga	0.7	0.6	0.9	7	6	9	0.3	0.3	0.4	3	2	4
Indonesia	Samarinda	0.9	0.7	1.1	55	44	68	0.5	0.4	0.6	36	29	45
Indonesia	Sambas	1.6	1.3	2.0	61	48	75	0.5	0.4	0.7	32	24	40
Indonesia	Samosir	1.2	0.9	1.4	14	11	17	0.5	0.4	0.6	4	3	5
Indonesia	Sampang	1.3	1.1	1.6	79	64	95	0.4	0.3	0.6	23	18	29
Indonesia	Sanggau	1.7	1.4	2.1	72	58	87	0.6	0.5	0.7	24	19	30
Indonesia	Sarmi	1.3	1.1	1.7	4	4	5	1.4	1.1	1.7	6	5	7
Indonesia	Sarolangun	1.3	1.0	1.6	24	19	30	0.4	0.3	0.5	8	6	10
Indonesia	Sawahlunto	1.1	0.8	1.3	9	7	12	0.5	0.4	0.7	3	3	4
Indonesia	Sekadau	1.7	1.4	2.1	34	27	41	0.6	0.5	0.8	11	9	14
Indonesia	Seluma	1.4	1.1	1.7	23	18	28	0.4	0.3	0.5	6	5	8
Indonesia	Semarang	0.7	0.6	0.9	62	51	75	0.3	0.3	0.4	24	20	29
Indonesia	Seram Bagian Barat	1.2	1.0	1.5	24	19	29	0.7	0.5	0.9	13	10	17
Indonesia	Seram Bagian Timur	1.3	1.0	1.6	15	12	19	0.7	0.6	0.9	8	7	11
Indonesia	Serang	1.0	0.8	1.2	165	131	205	0.4	0.3	0.5	52	43	64
Indonesia	Serdang Bedagai	1.3	1.1	1.6	88	71	106	0.5	0.4	0.6	35	28	42
Indonesia	Seruyan	1.4	1.1	1.7	17	13	21	0.4	0.3	0.5	8	6	10
Indonesia	Siak	1.3	1.0	1.6	42	33	50	0.3	0.2	0.4	16	12	20
Indonesia	Siau Tagulandang Biaro	0.9	0.7	1.2	5	4	6	0.6	0.5	0.8	1	1	2
Indonesia	Sibolga	1.2	0.9	1.5	1	1	1	0.6	0.5	0.8	0	0	1
Indonesia	Sidenreng Rappang	1.2	1.0	1.5	39	32	48	0.5	0.4	0.6	14	11	17
Indonesia	Sidoarjo	1.5	1.2	1.8	206	170	248	0.5	0.4	0.6	65	50	82
Indonesia	Sigi	2.1	1.7	2.6	80	66	96	0.9	0.7	1.1	34	27	42
Indonesia	Sijunjung	1.1	0.9	1.3	20	16	24	0.5	0.4	0.7	11	9	14
Indonesia	Sikka	1.3	1.0	1.6	47	38	59	0.7	0.6	0.9	24	19	30
Indonesia	Simalungun	1.3	1.0	1.5	143	117	171	0.5	0.4	0.6	40	32	48

Table 3: Diarrhoea Mortality by Unit *(continued)*

Country	Unit	2000 mortality mean rate (per 10,000)	2000 lower rate (per 10,000)	2000 upper rate (per 10,000)	2000 mean count	2000 lower count	2000 upper count	2017 mortality mean rate (per 10,000)	2017 lower rate (per 10,000)	2017 upper rate (per 10,000)	2017 mean count	2017 lower count	2017 upper count
Indonesia	Simeulue	1.2	0.9	1.4	7	5	8	0.4	0.3	0.5	4	3	5
Indonesia	Singkawang	1.5	1.2	2.0	22	17	29	0.6	0.4	0.7	12	9	15
Indonesia	Sinjai	1.1	0.9	1.4	27	21	33	0.5	0.3	0.6	10	7	12
Indonesia	Sintang	1.8	1.4	2.1	92	73	110	0.6	0.5	0.8	16	12	21
Indonesia	Situbondo	1.9	1.5	2.3	99	77	121	0.6	0.4	0.7	23	17	29
Indonesia	Sleman	0.5	0.4	0.7	41	34	50	0.3	0.2	0.3	20	17	24
Indonesia	Solok	1.1	0.9	1.3	43	34	53	0.5	0.4	0.7	19	15	23
Indonesia	Solok Selatan	1.1	0.8	1.3	17	14	21	0.5	0.4	0.6	7	6	9
Indonesia	Soppeng	1.2	1.0	1.5	34	28	42	0.5	0.4	0.6	10	8	13
Indonesia	Sorong	0.7	0.5	0.8	10	7	13	0.4	0.3	0.5	8	6	9
Indonesia	Sorong Selatan	0.6	0.5	0.8	2	1	2	0.4	0.3	0.5	2	2	3
Indonesia	Sragen	0.7	0.6	0.8	48	39	58	0.3	0.2	0.4	16	12	20
Indonesia	Subang	1.4	1.2	1.8	205	167	251	0.5	0.4	0.6	63	52	78
Indonesia	Subulussalam	1.1	0.9	1.4	5	4	6	0.5	0.4	0.6	4	3	4
Indonesia	Sukabumi	1.6	1.3	2.0	371	295	458	0.5	0.4	0.6	109	87	133
Indonesia	Sukamara	1.4	1.1	1.7	4	3	5	0.4	0.3	0.5	2	1	2
Indonesia	Sukoharjo	0.6	0.5	0.8	47	39	58	0.3	0.2	0.4	17	13	21
Indonesia	Sumba Barat	1.5	1.2	1.8	19	15	23	0.8	0.6	1.0	11	9	14
Indonesia	Sumba Barat Daya	1.4	1.1	1.7	44	35	53	0.8	0.6	1.0	29	23	35
Indonesia	Sumba Tengah	1.5	1.2	1.8	11	9	13	0.8	0.7	1.0	7	5	8
Indonesia	Sumba Timur	1.6	1.3	1.9	41	33	51	0.9	0.7	1.1	24	19	29
Indonesia	Sumbawa	2.5	2.0	3.1	100	82	126	0.7	0.5	0.9	29	22	37
Indonesia	Sumbawa Barat	2.6	2.0	3.2	27	21	34	0.7	0.5	0.8	8	6	9
Indonesia	Sumedang	1.7	1.4	2.1	176	147	220	0.5	0.4	0.6	47	38	57
Indonesia	Sumenep	1.6	1.2	1.9	115	91	142	0.5	0.4	0.6	27	21	33
Indonesia	Sungai Penuh	1.4	1.1	1.8	13	10	17	0.4	0.3	0.5	3	2	4
Indonesia	Supiori	1.2	0.9	1.6	2	1	2	1.4	1.1	1.8	2	1	2
Indonesia	Surabaya	1.6	1.3	1.9	322	264	393	0.5	0.4	0.7	84	64	105
Indonesia	Surakarta	0.7	0.6	0.8	16	13	19	0.3	0.2	0.4	6	4	7
Indonesia	Tabalong	1.5	1.2	1.9	28	22	34	0.6	0.5	0.8	13	10	16
Indonesia	Tabanan	0.6	0.5	0.7	21	18	25	0.4	0.3	0.4	9	7	11
Indonesia	Takalar	1.3	1.0	1.6	38	30	47	0.5	0.4	0.6	13	10	16
Indonesia	Tambrauw	0.6	0.5	0.8	1	0	1	0.4	0.3	0.4	1	0	1
Indonesia	Tana Tidung	0.7	0.6	0.9	1	1	2	0.4	0.3	0.5	1	1	1
Indonesia	Tana Toraja	1.4	1.1	1.7	35	28	43	0.5	0.4	0.7	12	9	15
Indonesia	Tanah Bumbu	1.8	1.4	2.2	29	23	36	0.6	0.5	0.8	17	13	22
Indonesia	Tanah Datar	1.1	0.9	1.4	46	36	58	0.6	0.4	0.7	18	14	22
Indonesia	Tanah Laut	1.7	1.3	2.1	42	33	52	0.6	0.5	0.8	17	14	22
Indonesia	Tangerang	1.1	0.9	1.3	302	251	364	0.4	0.3	0.5	117	97	146
Indonesia	Tangerang Selatan	1.3	1.1	1.5	147	122	175	0.4	0.4	0.5	53	43	66
Indonesia	Tanggamus	1.0	0.8	1.2	49	40	62	0.3	0.3	0.4	15	12	18
Indonesia	Tanjung Jabung B	1.3	1.0	1.6	32	25	40	0.4	0.3	0.5	10	8	13
Indonesia	Tanjung Jabung T	1.2	0.9	1.4	25	20	31	0.3	0.3	0.4	6	5	8
Indonesia	Tanjungpinang	0.9	0.7	1.1	7	5	9	0.4	0.3	0.5	2	2	3
Indonesia	Tapanuli Selatan	0.9	0.7	1.2	34	27	42	0.5	0.4	0.7	22	18	28
Indonesia	Tapanuli Tengah	1.1	0.8	1.3	39	30	48	0.6	0.4	0.7	23	18	29
Indonesia	Tapanuli Utara	1.0	0.8	1.3	35	27	43	0.5	0.4	0.7	16	12	20
Indonesia	Tapin	1.7	1.3	2.1	27	21	33	0.7	0.5	0.8	11	9	14
Indonesia	Tarakan	0.9	0.7	1.2	10	8	13	0.5	0.4	0.7	8	6	11
Indonesia	Tasikmalaya	1.7	1.4	2.1	242	197	296	0.4	0.3	0.5	57	45	69
Indonesia	Tebingtinggi	1.4	1.1	1.7	15	12	19	0.5	0.4	0.6	6	5	7
Indonesia	Tebo	1.2	0.9	1.5	30	24	38	0.4	0.3	0.5	10	8	13
Indonesia	Tegal	1.0	0.9	1.2	144	120	171	0.5	0.4	0.6	48	40	59
Indonesia	Teluk Bintuni	0.6	0.5	0.7	6	5	8	0.4	0.3	0.5	2	2	3
Indonesia	Teluk Wondama	0.6	0.5	0.8	2	1	2	0.4	0.3	0.5	1	1	2
Indonesia	Temanggung	0.8	0.7	1.0	51	42	60	0.3	0.3	0.4	17	14	21

Table 3: Diarrhoea Mortality by Unit *(continued)*

Country	Unit	2000 mortality mean rate (per 10,000)	lower rate (per 10,000)	upper rate (per 10,000)	mean count	lower count	upper count	2017 mortality mean rate (per 10,000)	lower rate (per 10,000)	upper rate (per 10,000)	mean count	lower count	upper count
Indonesia	Ternate	0.9	0.6	1.2	23	16	30	0.7	0.5	0.9	11	8	14
Indonesia	Tidore Kepulauan	0.9	0.6	1.1	9	7	11	0.7	0.5	0.8	7	5	8
Indonesia	Timor Tengah Selatan	1.3	1.1	1.7	75	61	93	0.7	0.6	0.9	38	31	46
Indonesia	Timor Tengah Utara	1.5	1.2	1.8	40	33	49	0.8	0.7	1.0	22	18	26
Indonesia	Toba Samosir	1.2	0.9	1.4	26	21	33	0.5	0.4	0.6	9	7	11
Indonesia	Tojo Una-Una	2.1	1.7	2.5	25	20	30	0.8	0.7	1.0	13	10	16
Indonesia	Toli-Toli	2.1	1.7	2.6	48	38	60	0.8	0.6	1.0	17	13	21
Indonesia	Tolikara	1.5	1.2	2.0	12	10	16	1.6	1.2	2.0	27	20	34
Indonesia	Tomohon	0.8	0.7	1.1	7	5	9	0.6	0.4	0.7	4	3	5
Indonesia	Toraja Utara	1.4	1.1	1.8	34	27	42	0.6	0.4	0.7	11	9	14
Indonesia	Trenggalek	1.3	1.0	1.5	69	54	83	0.4	0.3	0.5	15	12	19
Indonesia	Tual	1.3	1.0	1.7	15	11	19	0.8	0.6	1.0	4	3	5
Indonesia	Tuban	1.4	1.2	1.7	135	111	163	0.5	0.4	0.6	33	26	42
Indonesia	Tulang Bawang Barat	1.0	0.8	1.2	28	23	34	0.3	0.3	0.4	6	5	8
Indonesia	Tulangbawang	1.0	0.8	1.2	33	26	40	0.3	0.3	0.4	7	5	8
Indonesia	Tulungagung	1.3	1.1	1.6	106	85	127	0.4	0.3	0.5	26	20	32
Indonesia	Waduk Cirata	1.6	1.3	2.0	3	3	4	0.5	0.4	0.6	1	1	1
Indonesia	Waduk Kedungombo	0.7	0.6	0.9	1	0	1	0.3	0.2	0.4	0	0	0
Indonesia	Wajo	1.2	1.0	1.5	57	45	71	0.5	0.4	0.6	18	14	23
Indonesia	Wakatobi	2.0	1.5	2.5	22	17	27	0.9	0.7	1.1	6	5	8
Indonesia	Waropen	1.3	1.0	1.8	4	3	5	1.5	1.2	1.9	5	4	7
Indonesia	Way Kanan	1.0	0.8	1.2	36	29	44	0.3	0.3	0.4	12	10	15
Indonesia	Wonogiri	0.6	0.5	0.8	54	44	66	0.3	0.2	0.4	16	13	21
Indonesia	Wonosobo	0.9	0.7	1.0	61	51	72	0.4	0.3	0.4	20	16	24
Indonesia	Yahukimo	1.6	1.3	2.0	18	15	23	1.7	1.3	2.2	42	33	53
Indonesia	Yalimo	1.4	1.2	1.8	14	11	17	1.5	1.2	2.0	35	28	47
Laos	Atsaphangthong	3.0	1.8	5.3	19	11	33	1.0	0.6	1.6	3	2	5
Laos	Atsaphone	3.0	1.8	5.1	22	13	38	1.0	0.6	1.6	8	5	12
Laos	Bachiangchaleunsook	3.1	1.9	5.2	23	13	37	1.1	0.6	1.7	10	6	16
Laos	Beng	3.4	1.9	5.5	16	9	26	1.4	0.9	2.1	7	4	11
Laos	Bolikhanh	3.2	1.8	5.5	10	6	17	1.1	0.7	1.8	10	6	17
Laos	Boon Neua	3.5	2.0	5.7	8	5	14	1.5	0.9	2.4	4	2	7
Laos	Boontai	3.5	2.1	5.7	10	6	16	1.5	0.9	2.4	5	3	8
Laos	Botene	3.5	1.9	6.3	6	3	11	1.1	0.6	1.9	1	1	2
Laos	Bualapha	2.9	1.7	5.0	14	8	24	1.0	0.5	1.6	5	3	8
Laos	Champassack	3.2	1.9	5.4	23	14	39	1.1	0.6	1.8	7	4	12
Laos	Champhone	3.1	1.8	5.4	40	24	71	1.0	0.6	1.7	13	7	21
Laos	Chanthabuly	3.4	1.8	6.0	32	17	55	1.1	0.6	1.8	10	6	17
Laos	Chomphet	3.6	2.1	6.2	24	14	41	1.4	0.9	2.1	10	6	14
Laos	Dakcheung	3.6	2.1	5.7	14	8	23	1.2	0.7	1.9	4	2	7
Laos	Feuang	3.4	1.8	5.8	15	8	26	1.2	0.7	1.9	7	4	12
Laos	Hadxaifong	3.2	1.8	5.5	22	13	39	1.0	0.6	1.6	6	4	10
Laos	Hinboon	3.4	1.9	5.7	29	17	49	1.1	0.6	1.9	10	6	16
Laos	Hinhurp	3.3	1.8	5.6	13	7	22	1.2	0.7	1.8	5	3	8
Laos	Hom	3.2	1.8	5.4	7	4	12	1.2	0.7	1.9	2	1	4
Laos	Hongsa	3.8	2.1	6.2	14	8	24	1.4	0.9	2.2	5	3	7
Laos	Hoon	3.6	2.0	5.7	37	21	60	1.5	0.9	2.1	18	11	26
Laos	Houixai	3.8	2.2	5.8	24	14	37	1.5	0.9	2.3	10	6	16
Laos	Huameuang	3.1	1.7	5.4	17	9	29	1.2	0.7	1.9	6	4	10
Laos	Kaleum	3.7	2.1	5.9	11	6	18	1.2	0.7	2.0	3	2	4
Laos	Kasy	3.3	1.8	5.8	17	9	29	1.3	0.8	2.0	4	3	7
Laos	Kenethao	3.7	2.0	6.5	15	8	26	1.1	0.6	2.0	4	2	7
Laos	Keo Oudom	3.4	1.9	6.0	7	4	12	1.2	0.7	1.8	2	1	2
Laos	Kham	3.1	1.7	5.4	23	12	39	1.2	0.7	1.9	9	5	14
Laos	Khamkheuth	3.1	1.7	5.5	35	20	63	1.0	0.6	1.6	15	8	23

Table 3: Diarrhoea Mortality by Unit *(continued)*

Country	Unit	2000 mortality mean rate (per 10,000)	lower rate (per 10,000)	upper rate (per 10,000)	mean count	lower count	upper count	2017 mortality mean rate (per 10,000)	lower rate (per 10,000)	upper rate (per 10,000)	mean count	lower count	upper count
Laos	Khanthabouly	3.2	1.8	5.3	41	24	70	1.0	0.6	1.7	8	5	14
Laos	Khong	3.4	2.1	5.7	44	28	73	1.1	0.7	1.8	14	8	22
Laos	Khongxedone	3.2	1.8	5.5	22	12	37	1.1	0.6	1.8	7	4	12
Laos	Khop	3.9	2.1	6.5	10	5	16	1.4	0.9	2.3	3	2	5
Laos	Khoune	3.0	1.7	5.2	16	9	28	1.2	0.7	1.8	5	3	8
Laos	Khua	3.6	2.2	6.0	13	8	22	1.5	0.9	2.4	4	2	6
Laos	La	3.4	2.0	5.7	8	4	13	1.5	0.9	2.3	3	2	4
Laos	Lakhonepheng	3.2	1.8	5.6	16	9	28	1.1	0.6	1.7	6	3	9
Laos	Lamarm	3.7	2.1	5.8	16	9	25	1.2	0.7	2.0	7	4	12
Laos	Lao Ngarm	3.1	1.8	5.1	30	18	50	1.1	0.6	1.7	13	8	22
Laos	Long	3.7	2.2	6.0	15	9	24	1.5	0.9	2.4	8	5	12
Laos	Longsane	3.3	1.8	5.6	10	5	17	1.2	0.7	1.9	3	2	5
Laos	Louangphrabang	3.6	2.0	6.2	23	13	39	1.4	0.9	2.1	9	6	14
Laos	Mad	3.6	2.0	6.0	9	5	15	1.2	0.7	2.0	4	2	6
Laos	Mahaxay	3.3	1.9	5.8	17	10	30	1.1	0.6	1.8	7	4	11
Laos	May	3.5	2.1	5.5	15	9	24	1.4	0.8	2.4	5	3	8
Laos	Mayparkngum	3.5	1.8	6.0	19	10	33	1.1	0.7	1.9	7	4	12
Laos	Meung	3.9	2.2	6.0	7	4	11	1.5	0.9	2.3	2	1	3
Laos	Moonlapamok	3.3	2.0	5.6	18	11	30	1.1	0.7	1.9	6	4	10
Laos	Morkmay	3.0	1.7	4.8	7	4	11	1.1	0.6	1.9	3	2	5
Laos	Muang Et	2.6	1.4	4.6	7	4	11	1.0	0.6	1.6	2	1	4
Laos	Nakai	3.2	1.8	5.6	11	7	20	1.0	0.6	1.7	4	2	7
Laos	Nalae	3.3	1.9	5.3	12	7	19	1.4	0.9	2.1	4	3	6
Laos	Nam You	3.8	2.2	6.2	14	8	22	1.4	0.9	2.2	6	4	9
Laos	Nambak	3.6	2.1	5.8	30	18	50	1.5	0.9	2.4	14	8	22
Laos	Namor	3.3	1.9	5.3	16	9	27	1.4	0.9	2.2	8	5	13
Laos	Namtha	3.4	1.9	5.5	17	10	28	1.5	0.9	2.2	8	5	12
Laos	Nan	3.6	2.1	6.3	15	8	26	1.4	0.9	2.1	4	3	6
Laos	Naxaithong	3.5	1.9	5.8	24	13	41	1.1	0.6	1.8	9	5	14
Laos	Nga	3.6	2.0	5.9	17	10	29	1.5	0.9	2.2	7	4	10
Laos	Ngeun	3.9	2.1	6.3	8	5	14	1.5	0.9	2.3	3	2	5
Laos	Ngoi	3.6	2.1	5.9	24	15	40	1.5	0.9	2.5	8	5	13
Laos	Nhommalath	3.3	1.8	6.0	17	9	30	1.1	0.6	1.7	6	3	10
Laos	Nhot Ou	3.6	2.1	5.8	15	9	25	1.5	0.8	2.4	6	3	9
Laos	Nong	3.1	1.7	4.9	12	7	19	1.0	0.6	1.8	5	3	8
Laos	Nongbok	3.3	2.0	5.6	17	10	29	1.1	0.6	1.8	5	3	8
Laos	Nonghed	3.1	1.8	5.2	21	12	35	1.2	0.7	2.0	7	4	12
Laos	Outhoomphone	3.1	1.8	5.5	34	20	60	1.0	0.6	1.8	10	6	18
Laos	Pak Xeng	3.4	1.9	6.0	18	10	31	1.4	0.8	2.2	5	3	7
Laos	Pakbeng	3.9	2.2	6.4	17	10	28	1.5	0.9	2.3	7	4	10
Laos	Pakkading	3.4	1.9	5.6	18	10	29	1.1	0.7	1.9	8	5	12
Laos	Paksane	3.4	1.9	5.9	17	9	29	1.2	0.7	1.9	6	4	10
Laos	Paksong	3.2	1.9	5.1	33	19	53	1.1	0.6	1.8	15	9	25
Laos	Paktha	4.0	2.2	6.5	12	7	19	1.5	0.9	2.4	4	2	6
Laos	Pakxe	3.3	1.9	5.3	25	15	41	1.1	0.6	1.8	8	5	13
Laos	Park Ou	3.6	2.0	6.2	14	8	24	1.5	0.9	2.3	5	3	8
Laos	Parklai	3.8	2.1	6.4	27	15	45	1.2	0.7	2.1	7	4	11
Laos	Pathoomphone	3.2	1.9	5.3	32	19	52	1.1	0.6	1.8	11	6	17
Laos	Pek	3.3	1.8	5.6	29	16	49	1.3	0.8	1.9	11	7	17
Laos	Pha Oudom	3.8	2.1	6.2	23	12	37	1.5	0.9	2.3	11	6	17
Laos	Phaxay	3.2	1.7	5.5	6	3	11	1.2	0.8	1.9	1	1	2
Laos	Phiang	3.7	2.1	6.3	25	14	42	1.3	0.8	2.1	9	5	14
Laos	Phine	2.7	1.6	4.5	24	14	39	0.9	0.6	1.6	9	5	15
Laos	Phongsaly	3.6	2.2	5.8	13	8	21	1.5	0.9	2.5	4	2	7
Laos	Phonhong	3.4	1.8	5.9	22	12	38	1.2	0.7	1.8	5	3	8
Laos	Phonthong	3.2	1.9	5.2	34	20	55	1.1	0.6	1.8	10	6	17
Laos	Phonxay	3.3	1.9	5.8	18	11	32	1.3	0.8	2.0	7	5	11
Laos	Phookood	3.3	1.9	5.5	12	7	20	1.3	0.8	2.0	5	3	7
Laos	Phoukhoune	3.3	1.8	5.6	12	7	21	1.3	0.8	2.0	5	3	8
Laos	Phouvong	3.5	2.1	5.6	8	5	12	1.1	0.7	1.9	2	1	4

Table 3: Diarrhoea Mortality by Unit *(continued)*

Country	Unit	2000 mortality mean rate (per 10,000)	lower rate (per 10,000)	upper rate (per 10,000)	mean count	lower count	upper count	2017 mortality mean rate (per 10,000)	lower rate (per 10,000)	upper rate (per 10,000)	mean count	lower count	upper count
Laos	Phun	3.3	1.9	6.0	4	3	8	1.2	0.7	1.9	2	1	3
Laos	Samakkhixay	3.6	2.1	5.9	10	6	17	1.1	0.7	1.9	5	3	9
Laos	Samphanh	3.6	2.2	5.9	16	10	27	1.5	0.9	2.5	6	3	9
Laos	Samuoi	3.5	2.0	5.6	6	4	10	1.2	0.6	1.9	3	2	5
Laos	Sanamxay	3.4	2.0	5.7	15	9	25	1.1	0.6	1.8	6	4	10
Laos	Sanasomboon	3.2	1.9	5.3	27	16	45	1.1	0.7	1.8	8	5	13
Laos	Sangthong	3.4	1.9	5.6	9	5	15	1.1	0.6	1.8	4	2	6
Laos	Sanxay	3.6	2.1	5.9	8	5	14	1.2	0.7	1.9	3	2	6
Laos	Saravane	3.3	1.9	5.4	45	26	74	1.1	0.7	1.8	18	11	29
Laos	Sepone	2.7	1.6	4.4	21	12	34	0.9	0.5	1.6	7	4	12
Laos	Sikhottabong	3.5	1.9	6.1	26	14	45	1.1	0.6	1.8	9	5	14
Laos	Sing	3.6	2.1	5.8	15	9	24	1.5	0.9	2.3	8	5	12
Laos	Sisattanak	3.3	1.8	5.8	20	11	34	1.0	0.6	1.7	6	3	9
Laos	Songkhone	3.1	1.8	5.3	38	21	64	1.0	0.6	1.7	10	6	16
Laos	Sopbao	2.9	1.7	5.3	13	7	23	1.1	0.6	1.7	4	2	6
Laos	Sukhuma	3.2	1.9	5.4	22	13	37	1.1	0.6	1.7	8	5	14
Laos	Ta Oi	3.4	1.9	5.5	18	10	29	1.2	0.7	2.0	6	3	10
Laos	Thakhek	3.5	2.0	6.0	36	20	61	1.1	0.6	1.9	11	6	19
Laos	Thapangthong	3.0	1.7	5.1	16	9	28	1.0	0.6	1.7	7	4	11
Laos	Thaphabath	3.3	1.9	5.4	11	6	18	1.2	0.7	1.8	3	2	5
Laos	Thaphalanxay	2.8	1.7	5.1	14	8	25	1.0	0.6	1.6	5	3	9
Laos	Thateng	3.4	2.0	5.4	14	8	23	1.1	0.7	1.9	10	6	16
Laos	Thathom	3.0	1.7	5.1	5	3	8	1.1	0.6	1.8	3	2	4
Laos	Thongmyxay	3.8	2.1	6.6	4	2	8	1.2	0.7	2.1	1	1	2
Laos	Thoulakhom	3.5	1.8	5.9	22	12	38	1.2	0.7	1.9	5	3	8
Laos	Tonpheung	3.9	2.3	6.0	2	1	3	1.5	1.0	2.3	1	1	1
Laos	Toomlarn	3.1	1.8	5.0	11	6	18	1.1	0.6	1.8	5	3	8
Laos	Vangvieng	3.2	1.8	5.8	23	13	41	1.2	0.7	1.8	7	4	11
Laos	Vapy	3.2	1.8	5.5	16	9	27	1.1	0.7	1.8	5	3	9
Laos	Viengkham	3.2	1.9	5.5	28	16	47	1.3	0.8	2.2	9	5	15
Laos	Viengkham	3.5	1.8	5.9	6	3	11	1.2	0.7	1.9	2	1	3
Laos	Viengphoukha	3.6	2.1	5.8	12	7	19	1.5	0.9	2.2	5	3	8
Laos	Viengthong	3.0	1.7	5.3	14	8	24	1.2	0.7	2.0	6	3	10
Laos	Viengthong	3.1	1.7	5.0	13	7	21	1.1	0.6	1.8	6	3	10
Laos	Viengxay	2.7	1.5	4.9	14	8	24	1.0	0.6	1.6	4	3	7
Laos	Vilabuly	2.7	1.6	4.7	14	8	24	0.9	0.5	1.5	5	3	8
Laos	Xamneua	2.8	1.5	5.0	23	12	40	1.1	0.6	1.7	8	5	13
Laos	Xamtay	2.9	1.6	4.8	31	17	51	1.1	0.6	1.8	12	7	20
Laos	Xanakharm	3.5	1.9	5.9	25	13	43	1.1	0.6	1.9	8	5	14
Laos	Xay	3.4	2.0	5.4	31	18	50	1.5	0.8	2.3	17	10	27
Laos	Xayabury	3.8	2.1	6.3	38	22	64	1.4	0.8	2.1	14	9	22
Laos	Xaybuathong	3.1	1.8	5.3	11	6	19	1.0	0.6	1.7	5	3	8
Laos	Xaybuly	3.3	1.9	5.5	26	15	43	1.1	0.6	1.8	9	5	14
Laos	Xayphoothong	3.1	1.8	5.2	11	7	19	1.0	0.6	1.7	4	2	6
Laos	Xaysetha	3.6	2.2	6.0	21	12	35	1.2	0.7	2.0	7	4	12
Laos	Xaysetha	3.5	1.9	6.2	19	10	34	1.1	0.6	1.8	7	4	11
Laos	Xaysomboun	3.2	1.8	5.6	11	6	19	1.2	0.7	1.9	5	3	8
Laos	Xaythany	3.5	1.8	5.9	35	18	59	1.1	0.6	1.8	16	9	26
Laos	Xebangfay	3.3	1.9	5.8	12	7	21	1.1	0.6	1.8	4	2	7
Laos	Xieng Ngeun	3.4	2.0	5.8	20	12	34	1.3	0.8	2.1	6	4	9
Laos	Xienghone	4.0	2.2	6.7	15	8	25	1.5	0.9	2.4	5	3	8
Laos	Xiengkhor	3.0	1.6	5.6	24	13	45	1.1	0.7	1.8	3	2	5
Laos	Xonbuly	2.9	1.7	5.2	19	11	34	1.0	0.6	1.7	9	5	16
Myanmar	Bassein	1.6	0.9	2.5	241	141	376	0.5	0.3	0.8	62	36	98
Myanmar	Bawlake	1.8	1.0	2.9	9	5	14	0.5	0.3	0.8	3	1	4
Myanmar	Bhamo	1.7	1.0	2.6	43	25	66	0.5	0.3	0.9	18	10	31
Myanmar	Buthidaung	1.6	0.9	2.4	80	44	122	0.5	0.3	0.8	18	10	28
Myanmar	Dawei	1.4	0.8	2.2	67	38	109	0.4	0.2	0.6	20	12	33
Myanmar	Hinthada	1.6	0.9	2.4	198	113	300	0.5	0.3	0.7	50	30	79
Myanmar	Hkamti	1.5	0.9	2.3	60	36	90	0.5	0.3	0.8	19	12	30

Table 3: Diarrhoea Mortality by Unit *(continued)*

Country	Unit	2000 mortality mean rate (per 10,000)	2000 lower rate (per 10,000)	2000 upper rate (per 10,000)	2000 mean count	2000 lower count	2000 upper count	2017 mortality mean rate (per 10,000)	2017 lower rate (per 10,000)	2017 upper rate (per 10,000)	2017 mean count	2017 lower count	2017 upper count
Myanmar	Hpa-an	1.6	0.9	2.4	83	49	125	0.4	0.3	0.7	24	14	39
Myanmar	Kalemyo	1.9	1.1	2.8	78	44	115	0.5	0.3	0.9	22	13	36
Myanmar	Katha	1.6	1.0	2.5	130	77	197	0.5	0.3	0.8	38	20	60
Myanmar	Kawkareik	1.7	1.0	2.5	162	98	240	0.5	0.3	0.8	45	26	72
Myanmar	Kawthoung	1.3	0.7	2.0	23	13	34	0.4	0.3	0.7	8	5	13
Myanmar	Kengtung	1.4	0.8	2.2	40	24	64	0.5	0.3	0.8	19	11	32
Myanmar	Kunlong	1.6	0.9	2.7	22	13	37	0.5	0.3	0.9	9	5	15
Myanmar	Kyaukme	1.5	0.9	2.5	97	56	156	0.5	0.3	0.7	38	21	62
Myanmar	Kyaukse	1.6	0.9	2.6	109	62	178	0.4	0.2	0.7	24	13	38
Myanmar	Kyaunkpyu	1.2	0.7	2.0	63	36	104	0.4	0.2	0.6	14	8	22
Myanmar	Lasho	1.5	0.9	2.4	128	73	201	0.5	0.3	0.8	53	31	90
Myanmar	Lauking	1.6	0.9	2.6	7	4	12	0.5	0.3	0.8	3	2	5
Myanmar	Loikaw	1.8	1.0	2.8	47	27	75	0.5	0.3	0.7	13	8	21
Myanmar	Loilen	1.5	0.9	2.4	92	53	144	0.4	0.3	0.7	36	21	58
Myanmar	Magwe Minbu	1.6	0.9	2.7	212	115	354	0.4	0.3	0.7	43	26	69
Myanmar	Mandalay	1.6	0.9	2.5	286	164	450	0.4	0.2	0.7	64	35	106
Myanmar	Maubin	1.6	0.9	2.4	174	101	268	0.5	0.3	0.7	44	26	70
Myanmar	Maungtaw	1.6	1.0	2.5	11	7	17	0.5	0.3	0.8	3	2	5
Myanmar	Mawlamyine	1.6	1.0	2.3	237	144	351	0.4	0.2	0.7	48	27	79
Myanmar	Mawleik	1.7	1.0	2.7	25	15	40	0.5	0.3	0.9	8	4	13
Myanmar	Meiktila	1.4	0.8	2.4	131	75	220	0.4	0.2	0.6	29	17	48
Myanmar	Mergui	1.3	0.7	1.9	85	48	128	0.4	0.2	0.6	27	16	43
Myanmar	Minbu	1.7	0.9	2.7	122	65	194	0.5	0.3	0.7	26	15	41
Myanmar	Mindat	1.9	1.0	2.8	56	31	83	0.5	0.3	0.8	17	11	26
Myanmar	Mongphat	1.6	1.0	2.4	14	9	22	0.6	0.3	1.0	8	4	13
Myanmar	Mongsat	1.5	0.9	2.3	30	18	45	0.5	0.3	0.8	13	7	21
Myanmar	Monywa	1.7	0.9	2.6	206	110	311	0.5	0.3	0.8	53	31	88
Myanmar	Muse	1.5	0.9	2.4	51	29	82	0.5	0.2	0.8	21	11	36
Myanmar	Myawady	1.5	0.9	2.5	30	18	49	0.4	0.2	0.7	9	5	14
Myanmar	Myingyan	1.6	0.9	2.5	188	101	293	0.4	0.3	0.7	41	26	65
Myanmar	Myitkyina	1.8	1.1	2.7	153	92	229	0.5	0.3	0.8	63	37	99
Myanmar	Myoungmya	1.6	0.9	2.5	243	138	390	0.5	0.3	0.8	64	37	100
Myanmar	Naypyitaw	1.7	1.0	2.8	140	81	232	0.4	0.2	0.7	3	2	5
Myanmar	Pakokku	1.7	0.9	2.6	225	121	339	0.5	0.3	0.7	47	28	72
Myanmar	Palam	1.9	1.1	2.8	86	49	127	0.5	0.3	0.8	24	14	38
Myanmar	Pegu	1.6	0.9	2.4	266	151	395	0.4	0.2	0.7	65	37	111
Myanmar	Pharpon	1.5	0.9	2.4	167	97	264	0.5	0.3	0.7	43	26	68
Myanmar	Putao	1.7	1.0	2.5	11	7	16	0.5	0.3	0.9	5	3	8
Myanmar	Pyay	1.6	0.9	2.6	137	77	215	0.4	0.3	0.7	35	21	58
Myanmar	Pyin-Oo-Lwin	1.6	0.9	2.5	102	57	160	0.4	0.3	0.7	24	14	40
Myanmar	Sagaing	1.7	0.9	2.6	117	64	184	0.4	0.3	0.7	29	17	47
Myanmar	Shwebo	1.6	0.9	2.5	215	123	334	0.4	0.3	0.7	57	32	95
Myanmar	Sittwe	1.6	0.9	2.3	146	80	213	0.5	0.3	0.8	32	20	50
Myanmar	Tamu	1.6	0.9	2.5	14	8	22	0.5	0.3	0.9	4	3	7
Myanmar	Tarchilaik	1.6	1.0	2.3	21	13	31	0.6	0.3	0.9	9	5	16
Myanmar	Taunggye	1.6	0.9	2.7	220	129	371	0.4	0.3	0.7	77	45	128
Myanmar	Taungoo	1.7	1.0	2.7	193	111	301	0.4	0.3	0.7	43	25	71
Myanmar	Thandwe	1.4	0.7	2.3	56	31	95	0.4	0.3	0.7	12	7	19
Myanmar	Thaton	1.7	1.0	2.5	159	94	239	0.5	0.3	0.7	31	18	51
Myanmar	Thayarwady	1.6	1.0	2.5	160	95	247	0.4	0.3	0.7	41	24	68
Myanmar	Thayetmyo	1.6	0.9	2.7	123	68	203	0.4	0.3	0.7	26	15	40
Myanmar	Yamethin	1.7	1.0	2.8	1190	698	1950	0.4	0.2	0.7	31	18	51
Myanmar	Yangon-E	1.5	0.9	2.4	123	75	196	0.4	0.3	0.7	51	30	83
Myanmar	Yangon-N	1.6	0.9	2.4	223	133	349	0.4	0.3	0.7	87	52	140
Myanmar	Yangon-S	1.6	0.9	2.4	144	87	225	0.4	0.3	0.7	57	35	89
Myanmar	Yangon-W	1.5	0.9	2.4	121	73	193	0.4	0.3	0.7	50	29	80
Papua New Guinea	Abau	1.5	1.2	2.0	10	7	12	0.9	0.7	1.1	7	6	9
Papua New Guinea	Aitape-Lumi	1.5	1.2	1.9	14	11	18	0.8	0.6	1.0	10	8	12

Table 3: Diarrhoea Mortality by Unit *(continued)*

Country	Unit	2000 mortality mean rate (per 10,000)	lower rate (per 10,000)	upper rate (per 10,000)	mean count	lower count	upper count	2017 mortality mean rate (per 10,000)	lower rate (per 10,000)	upper rate (per 10,000)	mean count	lower count	upper count
Papua New Guinea	Alotau	1.5	1.2	1.9	18	14	23	0.9	0.7	1.1	14	11	18
Papua New Guinea	Ambunti-Dreikikir	1.5	1.2	2.0	15	12	20	0.9	0.7	1.1	12	9	15
Papua New Guinea	Anglimp-South Waghi	1.7	1.3	2.2	30	23	38	1.0	0.8	1.2	46	35	57
Papua New Guinea	Angoram	1.5	1.2	1.9	18	14	23	0.9	0.7	1.1	15	12	20
Papua New Guinea	Bogia	1.5	1.2	2.0	15	12	19	0.8	0.7	1.1	13	10	17
Papua New Guinea	Bulolo	1.7	1.3	2.1	23	18	29	1.0	0.7	1.2	19	14	24
Papua New Guinea	Central Bougainville	1.7	1.2	2.2	12	9	16	1.0	0.8	1.2	12	9	15
Papua New Guinea	Chuave	1.7	1.3	2.2	11	8	14	1.0	0.7	1.3	7	5	9
Papua New Guinea	Daulo	1.7	1.3	2.1	9	7	12	1.0	0.8	1.3	10	8	12
Papua New Guinea	Dei	1.7	1.2	2.2	14	10	18	1.0	0.7	1.2	16	12	20
Papua New Guinea	Esa'ala	1.6	1.2	2.1	11	8	13	0.9	0.7	1.2	8	7	11
Papua New Guinea	Finschhafen	1.7	1.3	2.1	12	10	15	0.9	0.7	1.1	8	6	10
Papua New Guinea	Gazelle	1.6	1.2	2.0	25	19	32	0.9	0.7	1.1	17	13	21
Papua New Guinea	Goilala	1.7	1.3	2.1	8	6	10	1.0	0.7	1.2	5	4	7
Papua New Guinea	Goroka	1.7	1.3	2.2	19	14	25	1.0	0.8	1.3	17	13	23
Papua New Guinea	Gumine	1.7	1.3	2.2	12	9	15	1.0	0.8	1.3	15	12	19
Papua New Guinea	Henganofi	1.7	1.3	2.2	16	12	21	1.0	0.8	1.3	11	9	15
Papua New Guinea	Huon	1.7	1.3	2.1	19	15	24	0.9	0.7	1.2	17	13	21
Papua New Guinea	Ialibu-Pangia	1.8	1.4	2.4	16	12	22	1.1	0.9	1.4	12	9	16
Papua New Guinea	Ijivitari	1.5	1.2	1.9	18	13	22	0.9	0.7	1.1	15	12	18
Papua New Guinea	Imbonggu	1.7	1.3	2.3	19	14	25	1.1	0.8	1.4	14	11	19
Papua New Guinea	Jimi	1.7	1.3	2.2	11	8	14	1.0	0.8	1.3	16	13	21
Papua New Guinea	Kabwum	1.6	1.3	2.1	12	9	15	0.9	0.8	1.2	7	5	9
Papua New Guinea	Kagua-Erave	1.8	1.4	2.4	18	14	24	1.1	0.8	1.4	20	15	25
Papua New Guinea	Kainantu	1.7	1.3	2.2	27	21	36	1.0	0.7	1.3	25	19	34
Papua New Guinea	Kairuku-Hiri	1.6	1.3	2.1	25	19	32	0.9	0.7	1.2	22	17	27
Papua New Guinea	Kandep	1.7	1.3	2.3	15	11	19	1.0	0.8	1.3	17	13	21
Papua New Guinea	Kandrian-Gloucester	1.6	1.3	2.1	15	12	19	0.9	0.7	1.1	11	9	14
Papua New Guinea	Karimui-Nomane	1.7	1.3	2.2	12	9	15	1.0	0.8	1.3	12	9	15
Papua New Guinea	Kavieng	1.6	1.2	1.9	12	9	15	0.9	0.7	1.1	10	8	12

Table 3: Diarrhoea Mortality by Unit *(continued)*

Country	Unit	2000 mortality						2017 mortality					
		mean rate (per 10,000)	lower rate (per 10,000)	upper rate (per 10,000)	mean count	lower count	upper count	mean rate (per 10,000)	lower rate (per 10,000)	upper rate (per 10,000)	mean count	lower count	upper count
Papua New Guinea	Kerema	1.6	1.3	2.0	18	14	22	1.0	0.8	1.2	13	10	16
Papua New Guinea	Kerowagi	1.7	1.3	2.2	16	12	20	1.0	0.8	1.2	23	18	28
Papua New Guinea	Kikori	1.8	1.4	2.3	13	11	17	1.1	0.9	1.3	8	6	10
Papua New Guinea	Kiriwina-Goodenough	1.6	1.2	1.9	12	9	14	0.9	0.7	1.1	9	7	11
Papua New Guinea	Kokopo	1.6	1.2	2.0	14	11	18	0.9	0.6	1.1	10	8	13
Papua New Guinea	Komo-Magarima	1.8	1.3	2.3	20	15	27	1.0	0.8	1.4	34	27	45
Papua New Guinea	Kompiam-Ambum	1.6	1.2	2.1	13	10	17	1.0	0.7	1.3	10	8	13
Papua New Guinea	Koroba-Kopiago	1.8	1.4	2.4	22	16	29	1.1	0.9	1.5	36	28	47
Papua New Guinea	Kundiawa-Gembogl	1.7	1.3	2.1	16	12	20	1.0	0.8	1.3	15	12	19
Papua New Guinea	Lae	1.8	1.3	2.4	33	24	42	1.1	0.8	1.4	19	15	24
Papua New Guinea	Lagaip-Porgera	1.8	1.3	2.3	29	21	38	1.1	0.8	1.4	43	32	56
Papua New Guinea	Lufa	1.7	1.3	2.2	14	10	18	1.0	0.8	1.3	12	9	16
Papua New Guinea	Madang	1.7	1.3	2.1	23	17	29	0.9	0.7	1.2	18	14	24
Papua New Guinea	Manus	1.6	1.2	2.0	10	7	12	0.9	0.7	1.1	6	5	8
Papua New Guinea	Maprik	1.5	1.1	2.0	14	11	19	0.8	0.6	1.1	11	8	14
Papua New Guinea	Markham	1.7	1.3	2.1	15	11	19	1.0	0.7	1.3	13	10	17
Papua New Guinea	Mendi-Munihu	1.7	1.3	2.2	27	20	35	1.0	0.8	1.4	30	22	40
Papua New Guinea	Menyamya	1.7	1.3	2.2	19	15	25	1.0	0.7	1.2	14	10	17
Papua New Guinea	Middle Fly	1.6	1.3	2.0	16	13	19	1.0	0.8	1.2	12	9	14
Papua New Guinea	Middle Ramu	1.6	1.3	2.0	16	12	20	0.9	0.7	1.2	14	11	18
Papua New Guinea	Mount Hagen	1.7	1.2	2.2	24	17	31	1.0	0.8	1.3	22	17	29
Papua New Guinea	Mul-Baiyer	1.6	1.2	2.1	16	12	20	1.0	0.8	1.2	17	13	21
Papua New Guinea	Namatanai	1.6	1.3	2.0	17	13	21	0.9	0.7	1.1	15	12	19
Papua New Guinea	National Capital District	1.7	1.3	2.2	63	47	83	1.0	0.8	1.3	51	39	66
Papua New Guinea	Nawae	1.7	1.3	2.1	11	8	14	0.9	0.7	1.2	8	6	10
Papua New Guinea	Nipa-Kutubu	1.8	1.4	2.4	31	24	41	1.1	0.8	1.4	35	27	44
Papua New Guinea	North Bougainville	1.6	1.3	2.1	18	14	24	0.9	0.7	1.1	15	12	19
Papua New Guinea	North Fly	1.8	1.4	2.4	16	12	21	1.1	0.8	1.3	12	9	15
Papua New Guinea	North Waghi	1.7	1.3	2.2	14	11	18	1.0	0.7	1.2	15	12	20
Papua New Guinea	Nuku	1.5	1.1	1.9	11	9	15	0.8	0.6	1.0	9	7	11

Table 3: Diarrhoea Mortality by Unit *(continued)*

Country	Unit	2000 mortality						2017 mortality					
		mean rate (per 10,000)	lower rate (per 10,000)	upper rate (per 10,000)	mean count	lower count	upper count	mean rate (per 10,000)	lower rate (per 10,000)	upper rate (per 10,000)	mean count	lower count	upper count
Papua New Guinea	Obura-Wonenara	1.7	1.3	2.1	9	7	12	1.0	0.7	1.3	8	6	10
Papua New Guinea	Okapa	1.7	1.3	2.2	18	14	24	1.0	0.7	1.3	13	10	17
Papua New Guinea	Pomio	1.7	1.3	2.1	14	11	17	0.9	0.8	1.2	11	9	13
Papua New Guinea	Rabaul	1.6	1.2	2.0	6	4	7	0.9	0.6	1.1	4	3	5
Papua New Guinea	Rai Coast	1.6	1.3	2.0	15	12	19	0.9	0.7	1.2	12	10	16
Papua New Guinea	Rigo	1.6	1.2	2.1	11	8	14	0.9	0.7	1.2	8	6	11
Papua New Guinea	Samarai-Murua	1.6	1.4	2.0	9	8	11	0.9	0.8	1.1	7	6	9
Papua New Guinea	Sina Sina-Yonggomugl	1.7	1.3	2.2	11	9	15	1.0	0.8	1.2	13	10	16
Papua New Guinea	Sohe	1.6	1.2	2.0	18	13	22	0.9	0.7	1.1	15	12	18
Papua New Guinea	South Bougainville	1.7	1.3	2.3	18	13	24	1.0	0.8	1.3	14	10	18
Papua New Guinea	South Fly	1.6	1.2	1.9	12	9	15	0.9	0.7	1.1	9	7	11
Papua New Guinea	Sumkar	1.6	1.2	2.1	18	14	24	0.9	0.7	1.2	14	10	17
Papua New Guinea	Talasea	1.7	1.3	2.1	36	28	46	0.9	0.7	1.2	30	24	38
Papua New Guinea	Tambul-Nebilyer	1.7	1.3	2.2	19	14	25	1.0	0.8	1.3	15	12	19
Papua New Guinea	Tari-Pori	1.7	1.3	2.3	15	11	20	1.0	0.8	1.4	18	14	24
Papua New Guinea	Telefomin	1.8	1.4	2.2	11	9	14	1.1	0.9	1.3	8	6	10
Papua New Guinea	Tewae-Siassi	1.6	1.3	2.0	11	9	14	0.9	0.7	1.1	8	7	10
Papua New Guinea	Unggai-Bena	1.7	1.3	2.2	14	11	19	1.0	0.8	1.3	14	11	19
Papua New Guinea	Usino-Bundi	1.7	1.3	2.1	13	10	16	1.0	0.8	1.2	13	11	17
Papua New Guinea	Vanimo-Green River	1.5	1.2	1.9	13	10	17	0.8	0.6	1.0	9	7	11
Papua New Guinea	Wabag	1.7	1.3	2.2	15	12	20	1.0	0.8	1.3	12	9	16
Papua New Guinea	Wapenamanda	1.7	1.2	2.2	15	11	20	1.0	0.8	1.3	17	13	21
Papua New Guinea	Wewak	1.5	1.2	1.9	16	12	20	0.9	0.6	1.1	12	9	16
Papua New Guinea	Wosera-Gawi	1.5	1.1	1.9	13	10	16	0.8	0.6	1.1	10	7	13
Papua New Guinea	Yangoro-Saussia	1.5	1.2	2.0	13	10	16	0.8	0.6	1.1	9	6	12
Philippines	Aborlan	0.6	0.4	0.7	2	1	2	0.3	0.2	0.4	2	1	3
Philippines	Abra de Ilog	0.5	0.4	0.7	2	1	2	0.3	0.2	0.4	2	1	2
Philippines	Abucay	0.6	0.4	0.7	3	2	4	0.3	0.2	0.4	1	1	2
Philippines	Abulug	0.7	0.5	0.8	2	2	3	0.3	0.3	0.4	1	1	2
Philippines	Abuyog	0.6	0.5	0.7	4	3	5	0.3	0.3	0.4	2	2	3
Philippines	Adams	0.6	0.5	0.8	0	0	0	0.3	0.3	0.4	0	0	0
Philippines	Agdangan	0.5	0.4	0.6	0	0	0	0.3	0.2	0.3	0	0	0
Philippines	Aglipay	0.5	0.4	0.6	1	1	2	0.3	0.2	0.4	1	1	1
Philippines	Agno	0.5	0.4	0.7	2	2	2	0.3	0.2	0.4	1	1	1
Philippines	Agoncillo	0.5	0.4	0.7	2	2	2	0.3	0.2	0.4	1	1	1

Table 3: Diarrhoea Mortality by Unit *(continued)*

Country	Unit	2000 mortality: mean rate (per 10,000)	lower rate (per 10,000)	upper rate (per 10,000)	mean count	lower count	upper count	2017 mortality: mean rate (per 10,000)	lower rate (per 10,000)	upper rate (per 10,000)	mean count	lower count	upper count
Philippines	Agoo	0.6	0.5	0.8	4	3	5	0.3	0.3	0.4	2	2	3
Philippines	Aguilar	0.6	0.4	0.7	3	2	3	0.3	0.2	0.4	2	1	2
Philippines	Aguinaldo	0.6	0.5	0.8	1	1	2	0.3	0.3	0.4	1	1	1
Philippines	Agutaya	NA	NA	NA	NA	NA	NA	NA	NA	NA	NA	NA	NA
Philippines	Ajuy	0.5	0.4	0.7	2	2	3	0.3	0.2	0.4	1	1	1
Philippines	Akbar	0.7	0.5	0.8	4	3	5	0.3	0.3	0.4	0	0	0
Philippines	Al-Barka	0.6	0.5	0.8	6	5	8	0.3	0.2	0.4	0	0	1
Philippines	Alabat	0.5	0.4	0.6	1	1	1	0.3	0.2	0.3	1	0	1
Philippines	Alabel	0.7	0.6	0.9	8	7	11	0.4	0.3	0.5	5	4	6
Philippines	Alamada	0.6	0.5	0.8	4	3	5	0.3	0.2	0.4	3	2	3
Philippines	Alaminos	0.5	0.4	0.6	2	2	3	0.3	0.2	0.3	2	1	2
Philippines	Alaminos City	0.5	0.4	0.7	5	4	7	0.3	0.2	0.4	3	3	4
Philippines	Alangalang	0.6	0.5	0.7	4	3	5	0.3	0.2	0.4	2	2	3
Philippines	Albuera	0.6	0.5	0.8	2	2	3	0.3	0.2	0.4	1	1	2
Philippines	Albuquerque	0.6	0.4	0.7	0	0	0	0.3	0.2	0.4	0	0	0
Philippines	Alcala	0.7	0.5	0.9	3	2	4	0.4	0.3	0.4	2	1	2
Philippines	Alcala	0.6	0.5	0.7	3	2	3	0.3	0.3	0.4	2	1	2
Philippines	Alcantara	0.5	0.4	0.7	1	1	1	0.3	0.2	0.4	0	0	1
Philippines	Alcantara	0.6	0.4	0.7	1	1	2	0.3	0.3	0.4	1	0	1
Philippines	Alcoy	0.5	0.4	0.6	1	1	1	0.3	0.2	0.4	0	0	1
Philippines	Alegria	0.7	0.5	0.9	1	1	1	0.4	0.3	0.5	1	0	1
Philippines	Alegria	0.5	0.4	0.7	2	1	2	0.3	0.2	0.4	1	1	1
Philippines	Aleosan	0.7	0.5	0.8	3	2	3	0.3	0.3	0.4	2	1	2
Philippines	Alfonso	0.5	0.4	0.7	3	2	4	0.3	0.2	0.4	2	1	2
Philippines	Alfonso Castaneda	0.5	0.4	0.7	0	0	0	0.3	0.2	0.4	0	0	0
Philippines	Alfonso Lista	0.6	0.5	0.8	3	2	3	0.3	0.3	0.4	2	2	3
Philippines	Aliaga	0.5	0.4	0.7	5	4	7	0.3	0.2	0.4	2	2	3
Philippines	Alicia	0.6	0.5	0.8	5	4	6	0.3	0.3	0.4	3	2	4
Philippines	Alicia	0.6	0.5	0.8	3	2	4	0.3	0.3	0.4	2	2	3
Philippines	Alicia	0.5	0.4	0.7	2	2	3	0.3	0.2	0.4	1	1	1
Philippines	Alilem	0.5	0.4	0.7	1	0	1	0.3	0.2	0.4	0	0	0
Philippines	Alimodian	0.6	0.5	0.8	2	2	3	0.3	0.2	0.4	2	1	2
Philippines	Alitagtag	0.5	0.4	0.6	1	1	1	0.3	0.2	0.3	1	1	1
Philippines	Allacapan	0.7	0.5	0.8	3	2	3	0.3	0.3	0.4	2	1	2
Philippines	Allen	0.5	0.4	0.7	1	1	1	0.3	0.2	0.4	0	0	1
Philippines	Almagro	0.5	0.4	0.7	0	0	0	0.3	0.2	0.4	0	0	0
Philippines	Almeria	0.6	0.4	0.7	1	1	1	0.3	0.2	0.4	1	1	1
Philippines	Aloguinsan	0.6	0.4	0.7	2	2	3	0.3	0.2	0.4	1	1	2
Philippines	Aloran	0.6	0.4	0.7	1	1	2	0.3	0.2	0.4	1	1	1
Philippines	Altavas	0.6	0.4	0.7	2	1	2	0.3	0.2	0.4	1	1	1
Philippines	Alubijid	0.5	0.4	0.7	1	1	2	0.3	0.2	0.4	1	1	1
Philippines	Amadeo	0.6	0.4	0.7	2	2	3	0.3	0.2	0.4	1	1	2
Philippines	Ambaguio	0.6	0.5	0.7	1	1	1	0.3	0.3	0.4	1	1	1
Philippines	Amlan	0.6	0.4	0.7	1	1	1	0.3	0.2	0.4	1	0	1
Philippines	Ampatuan	0.7	0.6	0.9	6	5	8	0.4	0.3	0.4	1	1	1
Philippines	Amulung	0.7	0.5	0.9	4	3	5	0.4	0.3	0.4	2	2	3
Philippines	Anahawan	0.6	0.5	0.8	0	0	0	0.3	0.3	0.4	0	0	0
Philippines	Anao	0.5	0.4	0.7	1	1	1	0.3	0.2	0.4	1	0	1
Philippines	Anda	0.5	0.4	0.7	0	0	1	0.3	0.2	0.4	0	0	0
Philippines	Anda	0.5	0.4	0.7	1	1	2	0.3	0.2	0.4	1	1	1
Philippines	Angadanan	0.6	0.5	0.7	3	2	4	0.3	0.3	0.4	2	1	2
Philippines	Angat	0.5	0.4	0.6	3	3	4	0.3	0.2	0.4	2	2	2
Philippines	Angeles City	0.6	0.5	0.7	17	14	22	0.3	0.2	0.4	9	7	11
Philippines	Angono	0.6	0.5	0.7	13	10	16	0.3	0.3	0.4	9	7	11
Philippines	Anilao	0.6	0.5	0.8	2	1	2	0.3	0.2	0.4	1	1	2
Philippines	Anini-Y	0.6	0.4	0.7	1	1	1	0.3	0.2	0.4	1	0	1
Philippines	Antequera	0.6	0.4	0.7	1	1	1	0.3	0.2	0.4	0	0	0
Philippines	Antipas	0.6	0.5	0.8	2	1	2	0.3	0.3	0.4	1	1	2
Philippines	Antipolo City	0.6	0.5	0.7	45	36	55	0.3	0.3	0.4	31	24	38

Country	Unit	2000 mortality mean rate (per 10,000)	lower rate (per 10,000)	upper rate (per 10,000)	mean count	lower count	upper count	2017 mortality mean rate (per 10,000)	lower rate (per 10,000)	upper rate (per 10,000)	mean count	lower count	upper count
Philippines	Apalit	0.5	0.4	0.7	7	5	9	0.3	0.2	0.4	4	3	5
Philippines	Aparri	0.6	0.5	0.8	5	4	7	0.3	0.3	0.4	2	2	3
Philippines	Araceli	0.6	0.4	0.7	1	1	1	0.3	0.2	0.4	1	0	1
Philippines	Arakan	0.6	0.5	0.8	3	2	3	0.3	0.3	0.4	2	2	3
Philippines	Arayat	0.5	0.4	0.7	9	7	11	0.3	0.2	0.4	5	4	6
Philippines	Argao	0.6	0.4	0.7	4	3	4	0.3	0.2	0.4	3	2	4
Philippines	Aringay	0.6	0.4	0.7	3	3	4	0.3	0.3	0.4	2	2	2
Philippines	Aritao	0.6	0.5	0.7	3	2	3	0.3	0.3	0.4	2	1	2
Philippines	Aroroy	0.6	0.5	0.8	4	3	6	0.4	0.3	0.5	7	5	8
Philippines	Arteche	0.6	0.5	0.8	1	1	2	0.3	0.3	0.4	1	1	1
Philippines	Asingan	0.6	0.5	0.7	4	3	4	0.3	0.3	0.4	2	2	2
Philippines	Asipulo	0.6	0.5	0.7	1	1	1	0.3	0.3	0.4	1	1	1
Philippines	Asturias	0.6	0.4	0.7	3	2	3	0.3	0.2	0.4	2	2	2
Philippines	Asuncion	0.5	0.4	0.7	4	3	5	0.3	0.2	0.4	3	2	4
Philippines	Atimonan	0.5	0.4	0.6	3	3	4	0.3	0.2	0.3	2	2	2
Philippines	Atok	0.6	0.5	0.7	1	1	1	0.3	0.3	0.4	1	1	1
Philippines	Aurora	0.6	0.5	0.8	4	3	5	0.3	0.3	0.4	2	2	3
Philippines	Aurora	0.6	0.5	0.8	2	2	3	0.3	0.3	0.4	1	1	2
Philippines	Ayungon	0.6	0.4	0.7	3	2	4	0.3	0.2	0.4	2	2	3
Philippines	Baao	0.6	0.4	0.7	4	3	6	0.3	0.2	0.4	3	2	4
Philippines	Babatngon	0.6	0.4	0.7	2	2	3	0.3	0.2	0.4	1	1	2
Philippines	Bacacay	0.6	0.4	0.7	4	3	5	0.3	0.3	0.4	3	2	3
Philippines	Bacarra	0.6	0.4	0.8	2	1	3	0.3	0.2	0.4	1	1	1
Philippines	Baclayon	0.6	0.5	0.7	1	0	1	0.3	0.2	0.4	0	0	0
Philippines	Bacnotan	0.6	0.4	0.7	2	2	3	0.3	0.2	0.3	1	1	2
Philippines	Baco	0.5	0.4	0.7	3	2	4	0.3	0.2	0.4	2	1	2
Philippines	Bacolod	0.6	0.4	0.7	1	1	2	0.3	0.2	0.4	1	1	1
Philippines	Bacolod City	0.6	0.5	0.8	34	26	45	0.3	0.3	0.4	18	13	22
Philippines	Bacolod Kalawi	0.6	0.5	0.9	1	1	1	0.3	0.2	0.4	0	0	0
Philippines	Bacolor	0.5	0.4	0.7	6	5	8	0.3	0.2	0.4	4	3	5
Philippines	Bacong	0.6	0.4	0.7	2	1	2	0.3	0.2	0.4	1	1	2
Philippines	Bacoor	0.7	0.5	0.8	34	27	42	0.4	0.3	0.4	30	23	37
Philippines	Bacuag	0.7	0.5	0.9	1	1	2	0.4	0.3	0.5	1	1	1
Philippines	Bacungan	0.6	0.5	0.8	2	1	2	0.3	0.2	0.4	2	1	2
Philippines	Badian	0.5	0.4	0.7	3	2	3	0.3	0.2	0.4	2	1	2
Philippines	Badiangan	0.6	0.4	0.7	2	1	2	0.3	0.2	0.4	1	1	1
Philippines	Badoc	0.6	0.4	0.7	2	1	2	0.3	0.2	0.4	1	1	1
Philippines	Bagabag	0.6	0.5	0.7	2	2	3	0.3	0.3	0.4	2	1	2
Philippines	Bagac	0.6	0.4	0.7	2	1	2	0.3	0.2	0.4	1	1	1
Philippines	Bagamanoc	0.7	0.5	0.8	1	0	1	0.4	0.3	0.5	1	0	1
Philippines	Baganga	0.6	0.4	0.7	4	3	5	0.3	0.2	0.4	3	2	4
Philippines	Baggao	0.6	0.5	0.8	6	5	8	0.3	0.3	0.4	4	3	5
Philippines	Bago City	0.6	0.4	0.7	11	9	15	0.3	0.2	0.4	6	4	7
Philippines	Baguio City	0.7	0.6	0.9	20	15	24	0.4	0.3	0.5	13	10	16
Philippines	Bagulin	0.5	0.4	0.7	1	1	1	0.3	0.2	0.4	0	0	1
Philippines	Bagumbayan	0.7	0.5	0.9	5	4	6	0.4	0.3	0.5	4	3	5
Philippines	Bais City	0.5	0.4	0.7	6	4	7	0.3	0.2	0.4	3	2	3
Philippines	Bakun	0.6	0.5	0.7	1	1	1	0.3	0.3	0.4	1	1	1
Philippines	Balabac	0.6	0.4	0.7	1	1	1	0.3	0.2	0.4	1	1	2
Philippines	Balabagan	0.6	0.5	0.8	5	4	6	0.3	0.2	0.4	0	0	1
Philippines	Balagtas	0.6	0.5	0.7	5	4	6	0.3	0.2	0.4	3	2	3
Philippines	Balamban	0.6	0.4	0.8	4	3	6	0.3	0.2	0.4	3	2	4
Philippines	Balanga City	0.6	0.4	0.7	4	3	5	0.3	0.2	0.4	2	2	2
Philippines	Balangiga	0.6	0.5	0.7	0	0	1	0.3	0.3	0.4	0	0	0
Philippines	Balangkayan	0.6	0.5	0.8	1	1	1	0.3	0.3	0.4	1	0	1
Philippines	Balaoan	0.5	0.4	0.7	2	2	3	0.3	0.2	0.3	1	1	1
Philippines	Balasan	0.5	0.4	0.7	2	1	2	0.3	0.2	0.4	1	1	2
Philippines	Balatan	0.6	0.5	0.7	2	2	3	0.3	0.2	0.4	2	1	2
Philippines	Balayan	0.5	0.4	0.7	5	4	7	0.3	0.2	0.4	3	2	3
Philippines	Balbalan	0.7	0.6	0.9	1	1	2	0.4	0.3	0.5	1	0	1

Table 3: Diarrhoea Mortality by Unit *(continued)*

Country	Unit	2000 mortality mean rate (per 10,000)	lower rate (per 10,000)	upper rate (per 10,000)	mean count	lower count	upper count	2017 mortality mean rate (per 10,000)	lower rate (per 10,000)	upper rate (per 10,000)	mean count	lower count	upper count
Philippines	Baleno	0.6	0.5	0.8	2	1	2	0.3	0.3	0.4	2	1	2
Philippines	Baler	0.5	0.4	0.6	2	2	3	0.3	0.2	0.3	1	1	2
Philippines	Balete	0.6	0.5	0.8	2	1	2	0.3	0.2	0.4	1	1	1
Philippines	Balete	0.5	0.4	0.6	1	1	2	0.3	0.2	0.3	1	1	1
Philippines	Baliangao	0.6	0.4	0.7	1	1	1	0.3	0.2	0.4	1	1	1
Philippines	Baliguian	0.6	0.5	0.8	2	2	3	0.3	0.3	0.4	1	1	1
Philippines	Balilihan	0.5	0.4	0.7	1	1	2	0.3	0.2	0.4	1	0	1
Philippines	Balindong	0.6	0.5	0.8	2	2	3	0.3	0.2	0.4	0	0	1
Philippines	Balingasag	0.6	0.4	0.7	2	2	2	0.3	0.2	0.4	2	1	2
Philippines	Balingoan	0.5	0.4	0.7	1	0	1	0.3	0.2	0.4	1	0	1
Philippines	Baliuag	0.5	0.4	0.7	7	6	9	0.3	0.2	0.4	4	3	5
Philippines	Ballesteros	0.6	0.5	0.8	2	2	3	0.3	0.2	0.4	1	1	2
Philippines	Baloi	0.6	0.4	0.7	3	2	4	0.3	0.2	0.4	3	2	3
Philippines	Balud	0.6	0.5	0.8	2	1	2	0.4	0.3	0.5	2	2	2
Philippines	Balungao	0.6	0.5	0.7	2	2	3	0.3	0.3	0.4	1	1	2
Philippines	Bamban	0.5	0.4	0.7	5	4	6	0.3	0.2	0.3	3	2	3
Philippines	Bambang	0.6	0.5	0.7	3	3	4	0.3	0.3	0.4	2	2	2
Philippines	Banate	0.6	0.5	0.8	1	1	2	0.3	0.2	0.4	1	1	1
Philippines	Banaue	0.6	0.5	0.8	2	1	2	0.3	0.3	0.4	1	1	1
Philippines	Banaybanay	0.6	0.4	0.7	2	2	3	0.3	0.3	0.4	2	1	2
Philippines	Banayoyo	0.6	0.4	0.7	0	0	0	0.3	0.2	0.4	0	0	0
Philippines	Banga	0.7	0.5	0.9	6	5	8	0.4	0.3	0.5	4	3	5
Philippines	Banga	0.6	0.5	0.8	2	2	3	0.3	0.2	0.4	2	1	2
Philippines	Bangar	0.6	0.4	0.7	2	1	2	0.3	0.2	0.4	1	1	1
Philippines	Bangued	0.6	0.5	0.9	4	3	5	0.3	0.3	0.4	1	1	2
Philippines	Bangui	0.6	0.4	0.7	1	1	1	0.3	0.2	0.4	0	0	1
Philippines	Bani	0.5	0.4	0.7	3	3	4	0.3	0.2	0.4	2	1	2
Philippines	Banisilan	0.6	0.5	0.8	4	3	6	0.3	0.3	0.4	3	2	3
Philippines	Banna	0.6	0.4	0.8	1	1	2	0.3	0.2	0.4	1	1	1
Philippines	Bansalan	0.6	0.5	0.8	3	3	4	0.3	0.3	0.4	2	2	3
Philippines	Bansud	0.5	0.4	0.7	2	2	3	0.3	0.2	0.4	2	1	2
Philippines	Bantay	0.6	0.4	0.8	2	1	3	0.3	0.2	0.4	1	1	1
Philippines	Bantayan	0.6	0.4	0.7	3	2	4	0.3	0.2	0.4	2	1	3
Philippines	Banton	0.5	0.4	0.7	0	0	0	0.3	0.2	0.4	0	0	0
Philippines	Baras	0.6	0.5	0.8	0	0	1	0.4	0.3	0.5	0	0	0
Philippines	Baras	0.5	0.4	0.7	2	2	2	0.3	0.2	0.4	1	1	1
Philippines	Barbaza	0.6	0.4	0.7	1	1	2	0.3	0.2	0.4	1	1	1
Philippines	Barcelona	0.6	0.4	0.7	2	1	2	0.3	0.3	0.4	1	1	1
Philippines	Barili	0.6	0.4	0.7	4	3	6	0.3	0.2	0.4	3	2	4
Philippines	Barira	0.6	0.5	0.8	6	4	7	0.3	0.2	0.4	1	0	1
Philippines	Barlig	0.7	0.6	0.9	1	1	1	0.4	0.3	0.5	0	0	0
Philippines	Barobo	0.7	0.5	0.9	3	3	4	0.4	0.3	0.5	2	2	3
Philippines	Barotac Nuevo	0.6	0.5	0.8	3	2	4	0.3	0.2	0.4	2	1	2
Philippines	Barotac Viejo	0.6	0.5	0.8	3	2	3	0.3	0.2	0.4	2	1	2
Philippines	Baroy	0.6	0.4	0.7	2	1	2	0.3	0.2	0.4	1	1	1
Philippines	Barugo	0.6	0.5	0.7	2	2	3	0.3	0.2	0.4	2	1	2
Philippines	Basay	0.6	0.4	0.7	1	1	2	0.3	0.2	0.4	1	1	1
Philippines	Basco	NA	NA	NA	NA	NA	NA	NA	NA	NA	NA	NA	NA
Philippines	Basey	0.6	0.5	0.7	3	2	3	0.3	0.2	0.4	2	1	2
Philippines	Basilisa	0.7	0.5	0.8	1	1	1	0.4	0.3	0.5	1	1	1
Philippines	Basista	0.6	0.5	0.7	2	2	2	0.3	0.2	0.4	1	1	2
Philippines	Basud	0.6	0.5	0.8	4	3	5	0.3	0.3	0.4	2	2	3
Philippines	Batac City	0.6	0.4	0.7	3	2	3	0.3	0.2	0.4	2	1	2
Philippines	Batad	0.5	0.4	0.7	1	1	2	0.3	0.2	0.4	1	1	1
Philippines	Batan	0.6	0.5	0.7	2	1	2	0.3	0.2	0.4	1	1	1
Philippines	Batangas City	0.5	0.4	0.7	19	15	25	0.3	0.2	0.4	11	9	14
Philippines	Bataraza	0.6	0.4	0.7	3	2	4	0.3	0.2	0.4	4	3	5
Philippines	Bato	0.6	0.5	0.8	1	1	1	0.4	0.3	0.5	1	1	1
Philippines	Bato	0.6	0.5	0.7	2	2	3	0.3	0.2	0.4	2	1	2
Philippines	Bato	0.6	0.4	0.7	1	1	1	0.3	0.2	0.4	1	0	1

Table 3: Diarrhoea Mortality by Unit *(continued)*

Country	Unit	2000 mortality						2017 mortality					
		mean rate (per 10,000)	lower rate (per 10,000)	upper rate (per 10,000)	mean count	lower count	upper count	mean rate (per 10,000)	lower rate (per 10,000)	upper rate (per 10,000)	mean count	lower count	upper count
Philippines	Bato Lake	0.6	0.5	0.7	0	0	0	0.3	0.2	0.4	0	0	0
Philippines	Bato Lake	0.6	0.5	0.7	1	1	1	0.3	0.2	0.4	1	1	1
Philippines	Batuan	0.6	0.5	0.8	1	1	1	0.3	0.3	0.4	1	0	1
Philippines	Batuan	0.5	0.4	0.7	1	1	1	0.3	0.2	0.4	0	0	1
Philippines	Bauan	0.5	0.4	0.7	3	3	4	0.3	0.2	0.4	2	1	2
Philippines	Bauang	0.6	0.4	0.7	6	4	7	0.3	0.2	0.4	3	2	3
Philippines	Bauko	0.7	0.5	0.8	2	2	3	0.3	0.3	0.4	1	1	2
Philippines	Baungon	0.5	0.4	0.7	2	2	3	0.3	0.2	0.4	2	1	2
Philippines	Bautista	0.6	0.5	0.7	2	2	3	0.3	0.2	0.4	1	1	2
Philippines	Bay	0.5	0.4	0.7	2	2	3	0.3	0.2	0.4	2	2	2
Philippines	Bayabas	0.7	0.5	0.9	1	1	1	0.4	0.3	0.5	1	0	1
Philippines	Bayambang	0.6	0.5	0.7	7	6	9	0.3	0.2	0.4	5	4	6
Philippines	Bayang	0.6	0.5	0.8	2	2	3	0.2	0.2	0.3	0	0	0
Philippines	Bayawan City	0.5	0.4	0.7	7	5	9	0.3	0.2	0.4	4	3	5
Philippines	Baybay City	0.6	0.5	0.7	7	6	9	0.3	0.3	0.4	4	3	4
Philippines	Bayog	0.6	0.5	0.8	2	2	3	0.3	0.2	0.4	1	1	2
Philippines	Bayombong	0.6	0.5	0.7	4	3	5	0.3	0.3	0.4	3	2	3
Philippines	Bayugan City	0.6	0.5	0.8	7	6	9	0.3	0.3	0.4	4	3	6
Philippines	Belison	0.6	0.4	0.7	1	0	1	0.3	0.2	0.4	0	0	0
Philippines	Benito Soliven	0.6	0.4	0.7	2	2	3	0.3	0.2	0.4	2	1	2
Philippines	Besao	0.6	0.5	0.8	1	0	1	0.3	0.3	0.4	0	0	0
Philippines	Bien Unido	0.6	0.4	0.7	1	1	1	0.3	0.2	0.4	1	0	1
Philippines	Bilar	0.5	0.4	0.7	1	1	2	0.3	0.2	0.4	1	0	1
Philippines	Biliran	0.6	0.4	0.7	1	1	2	0.3	0.2	0.4	1	1	1
Philippines	Binalbagan	0.6	0.4	0.7	5	4	6	0.3	0.2	0.4	2	2	3
Philippines	Binalonan	0.6	0.5	0.7	4	3	5	0.3	0.3	0.4	2	1	2
Philippines	Biñan	0.6	0.5	0.8	21	17	26	0.3	0.3	0.4	14	11	17
Philippines	Binangonan	0.6	0.5	0.7	11	9	14	0.3	0.3	0.4	7	5	8
Philippines	Bindoy	0.5	0.4	0.7	3	2	3	0.3	0.2	0.4	2	1	2
Philippines	Bingawan	0.6	0.4	0.7	1	0	1	0.3	0.2	0.4	0	0	1
Philippines	Binidayan	0.6	0.5	0.8	3	2	4	0.2	0.2	0.3	0	0	1
Philippines	Binmaley	0.6	0.5	0.8	7	6	9	0.3	0.3	0.4	4	3	5
Philippines	Binuangan	0.6	0.4	0.7	0	0	0	0.3	0.2	0.4	0	0	0
Philippines	Biri	NA	NA	NA	NA	NA	NA	NA	NA	NA	NA	NA	NA
Philippines	Bislig City	0.7	0.5	0.9	11	8	14	0.4	0.3	0.5	4	3	5
Philippines	Boac	0.5	0.4	0.7	3	3	4	0.3	0.2	0.4	2	2	3
Philippines	Bobon	0.6	0.4	0.7	2	1	2	0.3	0.3	0.4	1	1	2
Philippines	Bocaue	0.6	0.5	0.7	6	5	8	0.3	0.3	0.4	4	3	5
Philippines	Bogo City	0.5	0.4	0.7	3	2	4	0.3	0.2	0.4	2	1	2
Philippines	Bokod	0.6	0.5	0.7	1	1	2	0.3	0.3	0.4	1	0	1
Philippines	Bolinao	0.5	0.4	0.7	5	4	6	0.3	0.2	0.4	3	2	4
Philippines	Boliney	0.7	0.5	0.8	0	0	0	0.3	0.3	0.4	0	0	0
Philippines	Boljoon	0.5	0.4	0.7	1	1	1	0.3	0.2	0.4	1	0	1
Philippines	Bombon	0.6	0.4	0.7	2	1	2	0.3	0.2	0.4	1	1	1
Philippines	Bongabon	0.5	0.4	0.6	5	4	6	0.3	0.2	0.4	2	1	2
Philippines	Bongabong	0.5	0.4	0.7	4	3	5	0.3	0.2	0.4	3	3	4
Philippines	Bongao	0.7	0.5	0.9	11	9	15	0.3	0.2	0.4	2	1	2
Philippines	Bonifacio	0.6	0.4	0.7	2	1	2	0.3	0.2	0.4	1	1	1
Philippines	Bontoc	0.6	0.4	0.7	2	1	2	0.3	0.2	0.4	1	1	1
Philippines	Bontoc	0.6	0.5	0.8	2	2	3	0.3	0.3	0.4	1	1	1
Philippines	Borbon	0.5	0.4	0.7	2	2	3	0.3	0.2	0.4	1	1	1
Philippines	Borongan City	0.6	0.5	0.8	5	4	6	0.4	0.3	0.4	4	3	5
Philippines	Boston	0.6	0.4	0.8	1	1	1	0.3	0.2	0.4	1	1	1
Philippines	Botolan	0.5	0.4	0.7	3	3	4	0.3	0.2	0.4	2	2	3
Philippines	Braulio E. Dujali	0.6	0.4	0.7	2	1	2	0.3	0.2	0.4	2	1	2
Philippines	Brooke's Point	0.6	0.4	0.7	3	3	4	0.3	0.2	0.4	3	2	3
Philippines	Buadiposo-Buntong	0.6	0.4	0.8	4	3	5	0.3	0.2	0.4	1	0	1
Philippines	Bubong	0.6	0.5	0.8	3	2	4	0.3	0.2	0.4	1	0	1
Philippines	Bucay	0.6	0.5	0.8	1	1	2	0.3	0.3	0.4	1	1	1

Country	Unit	2000 mortality mean rate (per 10,000)	lower rate (per 10,000)	upper rate (per 10,000)	mean count	lower count	upper count	2017 mortality mean rate (per 10,000)	lower rate (per 10,000)	upper rate (per 10,000)	mean count	lower count	upper count
Philippines	Bucloc	0.6	0.5	0.8	0	0	0	0.3	0.3	0.4	0	0	0
Philippines	Buenavista	0.5	0.4	0.7	2	1	2	0.3	0.2	0.4	1	1	1
Philippines	Buenavista	0.6	0.4	0.7	4	3	4	0.3	0.2	0.4	2	2	3
Philippines	Buenavista	0.6	0.5	0.8	2	2	3	0.3	0.3	0.4	1	1	2
Philippines	Buenavista	0.5	0.4	0.7	1	1	2	0.3	0.2	0.4	1	1	1
Philippines	Buenavista	0.5	0.4	0.6	1	1	1	0.3	0.2	0.3	1	1	2
Philippines	Bugallon	0.6	0.4	0.7	5	4	6	0.3	0.2	0.4	3	2	3
Philippines	Bugasong	0.6	0.4	0.7	2	2	3	0.3	0.2	0.4	1	1	2
Philippines	Buguey	0.6	0.5	0.8	2	2	3	0.3	0.2	0.4	1	1	1
Philippines	Buguias	0.6	0.5	0.8	2	2	3	0.3	0.3	0.4	2	2	3
Philippines	Buhi	0.5	0.4	0.7	5	4	6	0.3	0.2	0.4	3	2	4
Philippines	Buhi Lake	0.5	0.4	0.7	1	1	1	0.3	0.2	0.4	0	0	1
Philippines	Bula	0.6	0.5	0.7	5	4	6	0.3	0.2	0.4	4	3	5
Philippines	Bulacan	0.6	0.4	0.7	8	6	10	0.3	0.2	0.4	4	3	5
Philippines	Bulalacao	0.6	0.4	0.7	2	1	2	0.3	0.3	0.4	2	1	2
Philippines	Bulan	0.6	0.5	0.7	6	4	7	0.3	0.3	0.4	3	2	4
Philippines	Buldon	0.6	0.5	0.8	5	3	6	0.3	0.2	0.4	1	1	1
Philippines	Buluan	0.7	0.6	0.9	1	1	2	0.4	0.3	0.5	1	1	2
Philippines	Buluan Lake	0.7	0.5	0.9	1	0	1	0.4	0.3	0.5	0	0	0
Philippines	Buluan Lake	0.7	0.6	0.9	1	1	2	0.4	0.3	0.5	0	0	1
Philippines	Bulusan	0.6	0.5	0.7	2	1	2	0.3	0.3	0.4	1	1	1
Philippines	Bumbaran	0.6	0.5	0.8	1	1	2	0.3	0.2	0.4	0	0	0
Philippines	Bunawan	0.7	0.5	0.8	3	2	4	0.4	0.3	0.5	2	1	2
Philippines	Burauen	0.6	0.4	0.7	4	3	5	0.3	0.2	0.4	2	1	2
Philippines	Burdeos	0.5	0.4	0.6	1	1	1	0.3	0.2	0.4	1	0	1
Philippines	Burgos	0.5	0.4	0.7	2	1	2	0.3	0.2	0.4	1	0	1
Philippines	Burgos	0.7	0.5	0.9	0	0	0	0.4	0.3	0.5	0	0	0
Philippines	Burgos	0.6	0.4	0.7	1	1	1	0.3	0.2	0.4	0	0	0
Philippines	Burgos	0.6	0.5	0.8	2	1	2	0.3	0.3	0.4	1	1	1
Philippines	Burgos	0.6	0.4	0.7	1	0	1	0.3	0.2	0.4	0	0	1
Philippines	Burgos	0.6	0.4	0.7	1	1	1	0.3	0.2	0.4	0	0	0
Philippines	Buruanga	0.6	0.5	0.8	1	1	2	0.4	0.3	0.5	1	1	1
Philippines	Bustos	0.5	0.4	0.7	6	4	7	0.3	0.2	0.4	3	3	4
Philippines	Busuanga	0.5	0.4	0.7	1	1	1	0.3	0.2	0.4	1	1	1
Philippines	Butig	0.6	0.5	0.8	3	3	4	0.2	0.2	0.3	0	0	0
Philippines	Butuan City	0.6	0.5	0.7	26	20	32	0.3	0.2	0.4	15	11	19
Philippines	Buug	0.6	0.5	0.8	3	2	3	0.3	0.2	0.4	2	1	2
Philippines	Caba	0.6	0.4	0.7	2	1	2	0.3	0.3	0.4	1	1	1
Philippines	Cabadbaran City	0.6	0.5	0.8	3	2	4	0.3	0.3	0.4	2	2	3
Philippines	Cabagan	0.7	0.5	0.9	4	3	5	0.4	0.3	0.5	2	2	3
Philippines	Cabanatuan City	0.6	0.4	0.7	18	14	23	0.3	0.2	0.4	11	9	14
Philippines	Cabangan	0.5	0.4	0.7	1	1	2	0.3	0.2	0.4	1	1	1
Philippines	Cabanglasan	0.5	0.4	0.7	3	2	3	0.3	0.2	0.3	1	1	2
Philippines	Cabarroguis	0.5	0.4	0.7	2	2	3	0.3	0.2	0.4	1	1	2
Philippines	Cabatuan	0.6	0.4	0.8	3	2	4	0.3	0.2	0.4	2	2	3
Philippines	Cabatuan	0.6	0.5	0.8	2	2	3	0.3	0.3	0.4	2	1	2
Philippines	Cabiao	0.5	0.4	0.7	4	3	5	0.3	0.2	0.4	3	2	3
Philippines	Cabucgayan	0.6	0.4	0.7	1	1	1	0.3	0.2	0.4	1	1	1
Philippines	Cabugao	0.6	0.4	0.8	2	1	2	0.3	0.2	0.4	1	1	1
Philippines	Cabusao	0.6	0.4	0.7	1	1	2	0.3	0.2	0.4	1	1	1
Philippines	Cabuyao	0.6	0.5	0.8	12	9	15	0.3	0.3	0.4	12	9	15
Philippines	Cadiz City	0.5	0.4	0.6	11	8	13	0.3	0.2	0.4	5	4	7
Philippines	Cagayan de Oro City	0.6	0.5	0.7	36	27	44	0.3	0.2	0.4	27	20	34
Philippines	Cagayancillo	NA	NA	NA	NA	NA	NA	NA	NA	NA	NA	NA	NA
Philippines	Cagdianao	0.7	0.5	0.9	2	1	2	0.4	0.3	0.5	1	1	1
Philippines	Cagwait	0.7	0.5	0.9	2	2	3	0.4	0.3	0.5	1	1	1
Philippines	Caibiran	0.5	0.4	0.7	1	1	2	0.3	0.2	0.4	1	1	1
Philippines	Cainta	0.5	0.4	0.6	17	13	21	0.3	0.2	0.3	10	8	12
Philippines	Cajidiocan	0.5	0.4	0.6	2	1	2	0.3	0.2	0.4	1	1	1

Table 3: Diarrhoea Mortality by Unit *(continued)*

Country	Unit	2000 mortality						2017 mortality					
		mean rate (per 10,000)	lower rate (per 10,000)	upper rate (per 10,000)	mean count	lower count	upper count	mean rate (per 10,000)	lower rate (per 10,000)	upper rate (per 10,000)	mean count	lower count	upper count
Philippines	Calabanga	0.5	0.4	0.7	5	4	6	0.3	0.2	0.4	3	3	4
Philippines	Calaca	0.5	0.4	0.7	4	3	6	0.3	0.2	0.4	3	2	4
Philippines	Calamba	0.6	0.4	0.7	1	1	2	0.3	0.2	0.4	1	1	1
Philippines	Calamba City	0.6	0.5	0.7	23	18	29	0.3	0.3	0.4	18	14	23
Philippines	Calanasan	0.7	0.5	0.9	1	1	1	0.4	0.3	0.5	1	1	1
Philippines	Calanogas	0.6	0.5	0.7	2	2	3	0.2	0.2	0.3	0	0	0
Philippines	Calapan City	0.5	0.4	0.7	7	5	9	0.3	0.2	0.4	5	4	6
Philippines	Calape	0.6	0.4	0.7	2	1	2	0.3	0.2	0.4	1	1	1
Philippines	Calasiao	0.6	0.5	0.8	7	5	8	0.3	0.3	0.4	4	3	5
Philippines	Calatagan	0.5	0.4	0.7	4	3	5	0.3	0.2	0.4	2	1	2
Philippines	Calatrava	0.5	0.4	0.7	1	1	1	0.3	0.2	0.4	0	0	0
Philippines	Calatrava	0.6	0.4	0.7	6	5	8	0.3	0.2	0.4	3	2	4
Philippines	Calauag	0.5	0.4	0.6	4	3	5	0.3	0.2	0.3	2	2	3
Philippines	Calauan	0.5	0.4	0.7	2	2	3	0.3	0.2	0.3	4	3	5
Philippines	Calayan	0.6	0.5	0.8	1	1	1	0.3	0.2	0.4	1	0	1
Philippines	Calbayog City	0.6	0.4	0.7	7	6	9	0.3	0.3	0.4	5	4	6
Philippines	Calbiga	0.6	0.4	0.7	2	1	2	0.3	0.2	0.4	1	1	1
Philippines	Calinog	0.6	0.4	0.7	3	2	4	0.3	0.2	0.4	2	1	2
Philippines	Calintaan	0.6	0.4	0.7	2	1	2	0.3	0.2	0.4	1	1	2
Philippines	Calubian	0.5	0.4	0.7	2	2	3	0.3	0.2	0.4	1	1	1
Philippines	Calumpit	0.6	0.4	0.7	7	6	9	0.3	0.2	0.4	4	3	5
Philippines	Caluya	0.6	0.5	0.8	1	1	1	0.4	0.3	0.5	1	1	1
Philippines	Camalaniugan	0.7	0.5	0.8	2	1	2	0.3	0.3	0.4	1	1	1
Philippines	Camalig	0.6	0.4	0.7	5	4	6	0.3	0.3	0.4	3	2	4
Philippines	Camaligan	0.6	0.5	0.7	1	1	2	0.3	0.3	0.4	1	1	1
Philippines	Camiling	0.6	0.4	0.7	5	4	7	0.3	0.2	0.4	2	2	3
Philippines	Can-Avid	0.6	0.4	0.7	2	1	2	0.3	0.3	0.4	1	1	2
Philippines	Canaman	0.6	0.5	0.7	3	2	4	0.3	0.3	0.4	2	1	2
Philippines	Candaba	0.5	0.4	0.7	7	6	9	0.3	0.2	0.3	4	3	6
Philippines	Candelaria	0.5	0.4	0.7	2	1	2	0.3	0.2	0.4	1	1	1
Philippines	Candelaria	0.5	0.4	0.6	7	5	9	0.3	0.2	0.4	5	4	6
Philippines	Candijay	0.5	0.4	0.7	2	2	3	0.3	0.2	0.4	1	1	1
Philippines	Candon City	0.6	0.4	0.7	3	2	4	0.3	0.2	0.4	2	1	2
Philippines	Candoni	0.5	0.4	0.7	2	2	3	0.3	0.2	0.4	1	1	1
Philippines	Canlaon City	0.6	0.4	0.7	4	3	5	0.3	0.2	0.4	2	2	3
Philippines	Cantilan	0.7	0.5	0.9	2	1	2	0.4	0.3	0.5	1	1	1
Philippines	Caoayan	0.6	0.5	0.8	1	1	1	0.3	0.2	0.4	0	0	1
Philippines	Capalonga	0.6	0.4	0.8	3	2	4	0.3	0.3	0.4	2	1	2
Philippines	Capas	0.5	0.4	0.7	9	7	11	0.3	0.2	0.3	5	4	6
Philippines	Capoocan	0.6	0.4	0.7	3	2	3	0.3	0.2	0.4	2	1	2
Philippines	Capul	0.6	0.4	0.7	0	0	0	0.3	0.2	0.4	0	0	0
Philippines	Caraga	0.5	0.4	0.7	2	2	3	0.3	0.2	0.4	2	1	2
Philippines	Caramoan	0.6	0.4	0.7	2	2	3	0.3	0.3	0.4	2	2	2
Philippines	Caramoran	0.7	0.5	0.8	2	2	3	0.4	0.3	0.5	2	1	2
Philippines	Carasi	0.6	0.5	0.8	0	0	0	0.3	0.3	0.4	0	0	0
Philippines	Carcar	0.6	0.4	0.8	6	4	8	0.3	0.2	0.4	4	3	5
Philippines	Cardona	0.5	0.4	0.7	2	1	2	0.3	0.2	0.4	1	1	1
Philippines	Carigara	0.6	0.5	0.7	4	3	5	0.3	0.2	0.4	2	2	3
Philippines	Carles	0.5	0.4	0.7	3	2	4	0.3	0.2	0.4	3	2	3
Philippines	Carmen	0.5	0.4	0.7	3	3	4	0.3	0.2	0.4	2	1	2
Philippines	Carmen	0.5	0.4	0.7	2	2	3	0.3	0.2	0.4	2	1	2
Philippines	Carmen	0.6	0.4	0.7	5	4	7	0.3	0.2	0.4	5	4	6
Philippines	Carmen	0.6	0.4	0.7	1	1	1	0.3	0.2	0.4	1	1	1
Philippines	Carmen	0.7	0.5	0.8	4	3	5	0.3	0.3	0.4	6	5	8
Philippines	Carmen	0.7	0.5	0.8	1	1	1	0.4	0.3	0.5	1	0	1
Philippines	Carmona	0.6	0.5	0.8	9	7	11	0.3	0.3	0.4	6	4	7
Philippines	Carranglan	0.5	0.4	0.6	2	2	3	0.3	0.2	0.4	2	1	2
Philippines	Carrascal	0.7	0.5	0.9	1	1	1	0.4	0.3	0.5	1	1	1
Philippines	Casiguran	0.5	0.3	0.6	1	1	2	0.3	0.2	0.3	1	1	1
Philippines	Casiguran	0.6	0.5	0.7	2	2	3	0.3	0.3	0.4	1	1	2

Table 3: Diarrhoea Mortality by Unit *(continued)*

Country	Unit	2000 mortality mean rate (per 10,000)	lower rate (per 10,000)	upper rate (per 10,000)	mean count	lower count	upper count	2017 mortality mean rate (per 10,000)	lower rate (per 10,000)	upper rate (per 10,000)	mean count	lower count	upper count
Philippines	Castilla	0.6	0.5	0.7	3	3	4	0.3	0.3	0.4	2	2	3
Philippines	Castillejos	0.5	0.4	0.7	2	2	3	0.3	0.2	0.4	2	1	2
Philippines	Cataingan	0.6	0.4	0.8	4	3	5	0.3	0.3	0.4	2	2	3
Philippines	Catanauan	0.5	0.4	0.6	5	4	6	0.3	0.2	0.4	2	2	3
Philippines	Catarman	0.6	0.4	0.7	1	1	1	0.3	0.2	0.4	0	0	1
Philippines	Catarman	0.6	0.4	0.7	8	6	10	0.3	0.3	0.4	5	4	7
Philippines	Catbalogan City	0.6	0.4	0.7	7	5	8	0.3	0.3	0.4	4	3	5
Philippines	Cateel	0.6	0.4	0.7	2	1	2	0.3	0.2	0.4	2	2	3
Philippines	Catigbian	0.5	0.4	0.7	1	1	2	0.3	0.2	0.4	1	0	1
Philippines	Catmon	0.5	0.4	0.7	1	1	2	0.3	0.2	0.4	1	1	1
Philippines	Catubig	0.6	0.4	0.8	3	2	4	0.3	0.3	0.4	2	2	3
Philippines	Cauayan	0.5	0.4	0.7	7	5	9	0.3	0.2	0.4	4	3	5
Philippines	Cauayan City	0.6	0.5	0.8	8	6	10	0.3	0.3	0.4	6	4	7
Philippines	Cavinti	0.5	0.4	0.6	1	1	1	0.3	0.2	0.3	0	0	1
Philippines	Cavite City	0.6	0.5	0.8	10	8	12	0.3	0.3	0.4	4	3	5
Philippines	Cawayan	0.6	0.4	0.8	4	3	5	0.3	0.3	0.4	2	2	3
Philippines	Cebu City	0.6	0.4	0.7	41	30	53	0.4	0.3	0.5	33	25	43
Philippines	Cervantes	0.5	0.4	0.7	1	1	1	0.3	0.2	0.4	1	1	1
Philippines	Clarin	0.5	0.4	0.7	1	1	2	0.3	0.2	0.4	1	1	1
Philippines	Clarin	0.6	0.4	0.7	2	2	3	0.3	0.2	0.4	2	1	2
Philippines	Claver	0.7	0.5	0.9	1	1	2	0.4	0.3	0.5	2	1	2
Philippines	Claveria	0.6	0.5	0.8	3	2	3	0.3	0.3	0.4	1	1	1
Philippines	Claveria	0.5	0.4	0.7	3	3	4	0.3	0.2	0.4	2	1	2
Philippines	Claveria	0.6	0.5	0.8	3	2	4	0.3	0.3	0.4	2	1	2
Philippines	Columbio	0.7	0.5	0.9	2	2	3	0.4	0.3	0.5	2	2	3
Philippines	Compostela	0.5	0.4	0.7	3	3	4	0.3	0.2	0.4	4	3	5
Philippines	Compostela	0.5	0.4	0.7	2	1	2	0.3	0.2	0.4	1	1	2
Philippines	Concepcion	0.5	0.4	0.7	1	1	1	0.3	0.2	0.4	1	1	1
Philippines	Concepcion	0.5	0.4	0.7	0	0	0	0.3	0.2	0.4	0	0	0
Philippines	Concepcion	0.5	0.4	0.7	10	8	13	0.3	0.2	0.3	5	4	7
Philippines	Concepcion	0.6	0.5	0.7	1	0	1	0.3	0.2	0.4	0	0	0
Philippines	Conner	0.7	0.5	0.9	2	2	3	0.3	0.3	0.4	2	1	2
Philippines	Consolacion	0.6	0.4	0.7	5	4	7	0.3	0.2	0.4	5	4	7
Philippines	Corcuera	0.5	0.4	0.7	0	0	1	0.3	0.2	0.4	0	0	0
Philippines	Cordoba	0.6	0.4	0.7	1	0	1	0.3	0.3	0.4	1	1	1
Philippines	Cordon	0.6	0.5	0.8	3	2	4	0.3	0.3	0.4	2	2	3
Philippines	Corella	0.6	0.4	0.7	1	1	1	0.3	0.2	0.4	0	0	0
Philippines	Coron	0.6	0.4	0.7	3	2	4	0.3	0.2	0.4	2	2	3
Philippines	Cortes	0.6	0.4	0.7	1	0	1	0.3	0.2	0.4	0	0	0
Philippines	Cortes	0.7	0.5	0.9	2	1	2	0.4	0.3	0.5	1	1	1
Philippines	Cotabato City	0.8	0.6	1.0	23	17	29	0.4	0.3	0.5	10	8	13
Philippines	Cuartero	0.6	0.4	0.7	2	1	2	0.3	0.2	0.4	1	1	1
Philippines	Cuenca	0.5	0.4	0.6	1	1	2	0.3	0.2	0.3	1	1	1
Philippines	Culaba	0.6	0.4	0.7	1	1	1	0.3	0.2	0.4	1	1	1
Philippines	Culasi	0.6	0.5	0.8	2	2	3	0.3	0.2	0.4	1	1	2
Philippines	Culion	0.6	0.4	0.7	1	1	1	0.3	0.2	0.4	1	1	1
Philippines	Currimao	0.6	0.4	0.7	1	1	1	0.3	0.2	0.4	0	0	1
Philippines	Cuyapo	0.6	0.5	0.7	3	3	4	0.3	0.2	0.4	2	2	3
Philippines	Cuyo	0.6	0.5	0.8	1	0	1	0.3	0.2	0.5	0	0	1
Philippines	Daanbantayan	0.5	0.4	0.7	3	2	3	0.3	0.2	0.4	2	1	2
Philippines	Daet	0.6	0.5	0.8	7	5	9	0.4	0.3	0.5	4	3	5
Philippines	Dagami	0.6	0.5	0.7	3	2	3	0.3	0.2	0.4	1	1	2
Philippines	Dagohoy	0.5	0.4	0.7	1	1	1	0.3	0.2	0.4	1	0	1
Philippines	Daguioman	0.7	0.5	0.9	0	0	0	0.4	0.3	0.4	0	0	0
Philippines	Dagupan City	0.6	0.5	0.8	8	7	10	0.3	0.3	0.4	6	5	7
Philippines	Dalaguete	0.5	0.4	0.7	4	3	5	0.3	0.2	0.4	2	2	3
Philippines	Damulog	0.6	0.5	0.7	1	1	2	0.3	0.3	0.4	2	1	2
Philippines	Danao	0.5	0.4	0.7	1	1	2	0.3	0.2	0.4	1	1	1
Philippines	Danao City	0.5	0.4	0.7	7	5	9	0.3	0.2	0.4	6	4	7
Philippines	Danao Lake	0.6	0.5	0.8	0	0	0	0.4	0.3	0.4	0	0	0

Table 3: Diarrhoea Mortality by Unit *(continued)*

Country	Unit	2000 mortality mean rate (per 10,000)	2000 mortality lower rate (per 10,000)	2000 mortality upper rate (per 10,000)	2000 mortality mean count	2000 mortality lower count	2000 mortality upper count	2017 mortality mean rate (per 10,000)	2017 mortality lower rate (per 10,000)	2017 mortality upper rate (per 10,000)	2017 mortality mean count	2017 mortality lower count	2017 mortality upper count
Philippines	Dangcagan	0.5	0.4	0.7	1	1	2	0.3	0.2	0.4	1	1	1
Philippines	Danglas	0.6	0.5	0.8	1	1	1	0.3	0.3	0.4	0	0	0
Philippines	Dao	0.6	0.4	0.7	2	1	2	0.3	0.2	0.4	1	1	1
Philippines	Dapa	0.7	0.6	0.9	2	1	2	0.4	0.3	0.5	1	1	1
Philippines	Dapao Lake	0.6	0.5	0.8	0	0	1	0.3	0.2	0.3	0	0	0
Philippines	Dapitan City	0.6	0.5	0.8	5	4	7	0.3	0.3	0.4	3	3	4
Philippines	Daraga	0.6	0.5	0.7	8	7	10	0.3	0.3	0.4	5	4	6
Philippines	Daram	0.6	0.4	0.7	2	2	3	0.3	0.3	0.4	2	1	2
Philippines	Dasmariñas	0.6	0.5	0.8	51	42	63	0.3	0.3	0.4	31	24	37
Philippines	Dasol	0.5	0.4	0.7	2	2	3	0.3	0.2	0.4	1	1	1
Philippines	Datu Abdullah Sanki	0.7	0.6	0.9	6	5	7	0.4	0.3	0.5	1	1	1
Philippines	Datu Anggal Midtimbang	0.7	0.6	0.9	3	2	3	0.3	0.3	0.4	0	0	0
Philippines	Datu Blah T. Sinsuat	0.7	0.5	0.9	5	4	6	0.3	0.3	0.4	1	0	1
Philippines	Datu Odin Sinsuat	0.7	0.6	0.9	23	18	28	0.4	0.3	0.5	3	3	4
Philippines	Datu Paglas	0.7	0.6	0.9	5	4	6	0.4	0.3	0.5	1	1	1
Philippines	Datu Piang	0.7	0.6	0.9	8	6	10	0.3	0.3	0.4	1	1	1
Philippines	Datu Saudi-Ampatuan	0.7	0.5	0.9	5	4	7	0.3	0.3	0.4	0	0	1
Philippines	Datu Unsay	0.7	0.6	0.9	2	2	3	0.3	0.3	0.4	0	0	0
Philippines	Dauin	0.5	0.4	0.7	1	1	1	0.3	0.2	0.4	1	1	1
Philippines	Dauis	0.6	0.4	0.7	3	2	4	0.3	0.2	0.4	2	2	3
Philippines	Davao City	0.6	0.5	0.8	100	78	125	0.3	0.3	0.4	67	53	83
Philippines	Del Carmen	0.7	0.6	0.9	1	1	2	0.4	0.3	0.5	1	1	1
Philippines	Del Gallego	0.6	0.4	0.7	1	1	2	0.3	0.3	0.4	1	1	1
Philippines	Delfin Albano	0.7	0.5	0.8	3	2	3	0.3	0.3	0.4	1	1	2
Philippines	Diadi	0.6	0.5	0.7	1	1	1	0.3	0.3	0.4	1	1	1
Philippines	Diffun	0.6	0.4	0.7	3	2	4	0.3	0.3	0.4	3	2	3
Philippines	Digos City	0.6	0.5	0.8	9	7	12	0.3	0.3	0.4	5	4	6
Philippines	Dilasag	0.5	0.4	0.6	1	1	1	0.3	0.2	0.4	1	0	1
Philippines	Dimasalang	0.6	0.5	0.8	2	2	3	0.3	0.3	0.4	2	1	2
Philippines	Dimataling	0.6	0.5	0.8	2	2	3	0.3	0.3	0.4	1	1	2
Philippines	Dimiao	0.6	0.4	0.7	1	1	1	0.3	0.2	0.4	0	0	1
Philippines	Dinagat	0.7	0.5	0.8	0	0	1	0.4	0.3	0.5	0	0	0
Philippines	Dinalungan	0.5	0.3	0.6	1	0	1	0.3	0.2	0.3	0	0	1
Philippines	Dinalupihan	0.6	0.4	0.7	5	4	6	0.3	0.2	0.4	3	2	3
Philippines	Dinapigue	0.6	0.4	0.7	0	0	0	0.3	0.3	0.4	0	0	0
Philippines	Dinas	0.6	0.5	0.8	3	2	4	0.3	0.2	0.4	2	1	2
Philippines	Dingalan	0.5	0.4	0.6	1	1	2	0.3	0.2	0.4	1	1	1
Philippines	Dingle	0.6	0.5	0.8	2	2	3	0.3	0.2	0.4	2	1	2
Philippines	Dingras	0.6	0.4	0.7	2	2	3	0.3	0.2	0.4	1	1	1
Philippines	Dipaculao	0.5	0.4	0.6	1	1	1	0.3	0.2	0.3	1	1	1
Philippines	Diplahan	0.6	0.5	0.8	2	2	2	0.3	0.3	0.4	2	1	2
Philippines	Dipolog City	0.6	0.5	0.8	8	6	10	0.3	0.3	0.4	5	4	6
Philippines	Ditsaan-Ramain	0.6	0.5	0.8	2	1	2	0.3	0.2	0.4	0	0	0
Philippines	Divilacan	0.6	0.5	0.8	0	0	0	0.3	0.3	0.4	0	0	0
Philippines	Dolores	0.6	0.5	0.7	2	2	3	0.3	0.3	0.4	2	2	2
Philippines	Dolores	0.6	0.5	0.8	1	1	1	0.3	0.3	0.4	0	0	1
Philippines	Dolores	0.5	0.4	0.6	2	1	2	0.3	0.2	0.3	1	1	1
Philippines	Don Carlos	0.5	0.4	0.7	4	3	5	0.3	0.2	0.4	3	2	3
Philippines	Don Marcelino	0.6	0.5	0.8	2	2	3	0.3	0.3	0.4	3	2	4
Philippines	Don Victoriano Chiongbian	0.6	0.4	0.7	1	1	2	0.3	0.2	0.4	0	0	1
Philippines	Doña Remedios Trinidad	0.5	0.4	0.6	2	1	2	0.3	0.2	0.4	1	1	1
Philippines	Donsol	0.6	0.4	0.7	2	1	2	0.3	0.3	0.4	1	1	1
Philippines	Duenas	0.6	0.4	0.7	2	2	3	0.3	0.2	0.4	1	1	2

Country	Unit	2000 mortality mean rate (per 10,000)	lower rate (per 10,000)	upper rate (per 10,000)	mean count	lower count	upper count	2017 mortality mean rate (per 10,000)	lower rate (per 10,000)	upper rate (per 10,000)	mean count	lower count	upper count
Philippines	Duero	0.5	0.4	0.7	1	1	2	0.3	0.2	0.4	1	0	1
Philippines	Dulag	0.6	0.5	0.7	3	2	4	0.3	0.3	0.4	2	1	2
Philippines	Dumaguete City	0.5	0.4	0.7	5	4	6	0.3	0.2	0.4	3	2	4
Philippines	Dumalag	0.6	0.4	0.7	2	2	3	0.3	0.2	0.4	1	1	1
Philippines	Dumalinao	0.6	0.5	0.8	3	2	4	0.3	0.3	0.4	2	1	2
Philippines	Dumalneg	0.6	0.4	0.7	0	0	0	0.3	0.2	0.4	0	0	0
Philippines	Dumangas	0.6	0.5	0.8	4	3	5	0.3	0.3	0.4	2	2	3
Philippines	Dumanjug	0.5	0.4	0.7	2	1	2	0.3	0.2	0.4	1	1	1
Philippines	Dumaran	0.6	0.4	0.7	1	1	1	0.3	0.2	0.4	1	1	1
Philippines	Dumarao	0.6	0.4	0.7	3	2	4	0.3	0.2	0.4	2	1	2
Philippines	Dumingag	0.6	0.5	0.8	4	3	5	0.3	0.2	0.4	2	2	3
Philippines	Dupax Del Norte	0.5	0.4	0.7	1	1	2	0.3	0.2	0.4	1	1	1
Philippines	Dupax Del Sur	0.6	0.4	0.7	1	1	1	0.3	0.2	0.4	1	1	1
Philippines	Echague	0.6	0.4	0.7	5	4	6	0.3	0.2	0.4	3	3	4
Philippines	El Nido	0.6	0.4	0.7	2	1	2	0.3	0.2	0.4	2	2	3
Philippines	El Salvador City	0.6	0.4	0.7	3	2	4	0.3	0.2	0.4	2	1	2
Philippines	Enrile	0.7	0.5	0.9	4	3	5	0.4	0.3	0.5	3	2	4
Philippines	Enrique B. Magalona	0.6	0.4	0.7	4	3	6	0.3	0.2	0.4	2	2	3
Philippines	Enrique Villanueva	0.6	0.4	0.7	0	0	0	0.3	0.2	0.4	0	0	0
Philippines	Escalante City	0.6	0.4	0.7	7	5	8	0.3	0.2	0.4	3	2	4
Philippines	Esperanza	0.6	0.5	0.8	5	4	6	0.3	0.3	0.4	4	3	4
Philippines	Esperanza	0.7	0.5	0.9	3	3	4	0.4	0.3	0.5	3	3	4
Philippines	Esperanza	0.6	0.4	0.8	1	1	1	0.3	0.3	0.4	0	0	1
Philippines	Estancia	0.5	0.4	0.7	2	2	3	0.3	0.2	0.4	2	1	2
Philippines	Famy	0.5	0.4	0.6	1	1	1	0.3	0.2	0.3	1	0	1
Philippines	Ferrol	0.6	0.4	0.7	0	0	1	0.3	0.2	0.4	0	0	0
Philippines	Flora	0.6	0.5	0.8	2	1	2	0.3	0.3	0.4	1	1	1
Philippines	Floridablanca	0.5	0.4	0.7	6	5	8	0.3	0.2	0.4	4	3	5
Philippines	Gabaldon	0.5	0.4	0.6	1	1	2	0.3	0.2	0.3	1	1	1
Philippines	Gainza	0.5	0.4	0.7	1	0	1	0.3	0.2	0.4	0	0	1
Philippines	Galimuyod	0.6	0.4	0.7	1	0	1	0.3	0.2	0.4	0	0	0
Philippines	Gamay	0.6	0.5	0.8	1	1	2	0.4	0.3	0.5	1	1	1
Philippines	Gamu	0.6	0.5	0.8	2	2	3	0.3	0.3	0.4	1	1	2
Philippines	Ganassi	0.6	0.4	0.8	3	3	5	0.3	0.2	0.3	0	0	0
Philippines	Gandara	0.6	0.4	0.7	3	2	4	0.3	0.3	0.4	2	1	2
Philippines	Gapan City	0.5	0.4	0.7	5	4	7	0.3	0.2	0.4	3	2	4
Philippines	Garchitorena	0.6	0.5	0.7	2	2	3	0.3	0.3	0.4	1	1	1
Philippines	Garcia Hernandez	0.6	0.4	0.7	1	1	1	0.3	0.2	0.4	1	0	1
Philippines	Gasan	0.5	0.4	0.7	2	2	3	0.3	0.2	0.4	1	1	1
Philippines	Gattaran	0.7	0.5	0.9	4	3	5	0.4	0.3	0.4	3	2	3
Philippines	Gen. S. K. Pendatun	0.7	0.6	0.9	6	4	7	0.4	0.3	0.5	1	1	1
Philippines	General Emilio Aguinaldo	0.6	0.4	0.7	1	1	1	0.3	0.2	0.4	1	0	1
Philippines	General Luna	0.5	0.4	0.6	1	1	2	0.3	0.2	0.4	1	1	1
Philippines	General Luna	0.7	0.6	1.0	1	0	1	0.4	0.3	0.5	0	0	1
Philippines	General Macarthur	0.6	0.5	0.8	1	1	1	0.3	0.3	0.4	0	0	1
Philippines	General Mamerto Natividad	0.5	0.4	0.7	2	2	3	0.3	0.2	0.4	2	1	2
Philippines	General Mariano Alvarez	0.6	0.5	0.8	6	5	7	0.3	0.3	0.4	3	2	4
Philippines	General Nakar	0.5	0.4	0.6	2	2	3	0.3	0.2	0.4	2	1	2
Philippines	General Santos City	0.8	0.6	1.0	47	36	61	0.4	0.4	0.5	25	20	31
Philippines	General Tinio	0.5	0.4	0.6	2	2	3	0.3	0.2	0.4	2	1	2
Philippines	General Trias	0.6	0.5	0.8	22	18	27	0.3	0.2	0.4	16	12	20
Philippines	Gerona	0.6	0.4	0.7	6	5	7	0.3	0.2	0.4	3	2	4

Table 3: Diarrhoea Mortality by Unit *(continued)*

Country	Unit	2000 mortality mean rate (per 10,000)	lower rate (per 10,000)	upper rate (per 10,000)	mean count	lower count	upper count	2017 mortality mean rate (per 10,000)	lower rate (per 10,000)	upper rate (per 10,000)	mean count	lower count	upper count
Philippines	Gigaquit	0.7	0.5	0.8	2	1	2	0.4	0.3	0.5	1	1	1
Philippines	Gigmoto	0.6	0.5	0.8	1	1	1	0.4	0.3	0.5	0	0	1
Philippines	Ginatilan	0.5	0.4	0.7	1	0	1	0.3	0.2	0.4	1	0	1
Philippines	Gingoog City	0.6	0.4	0.7	8	6	10	0.3	0.2	0.4	5	4	6
Philippines	Giporlos	0.6	0.5	0.7	0	0	0	0.3	0.3	0.4	0	0	0
Philippines	Gitagum	0.5	0.4	0.7	1	1	1	0.3	0.2	0.4	1	1	1
Philippines	Glan	0.7	0.5	0.9	9	7	12	0.4	0.3	0.5	6	5	8
Philippines	Gloria	0.5	0.4	0.7	3	2	4	0.3	0.2	0.4	2	2	3
Philippines	Goa	0.5	0.4	0.7	4	3	6	0.3	0.2	0.4	3	3	4
Philippines	Godod	0.6	0.5	0.8	2	1	2	0.3	0.3	0.4	1	1	1
Philippines	Gonzaga	0.6	0.5	0.8	3	2	3	0.3	0.2	0.4	1	1	2
Philippines	Governor Generoso	0.6	0.5	0.7	2	2	3	0.3	0.3	0.4	2	1	2
Philippines	Gregorio Del Pilar	0.5	0.4	0.7	0	0	0	0.3	0.2	0.4	0	0	0
Philippines	Guagua	0.5	0.4	0.7	6	4	7	0.3	0.2	0.4	4	3	4
Philippines	Gubat	0.6	0.5	0.7	4	3	5	0.3	0.3	0.4	2	2	3
Philippines	Guiguinto	0.6	0.5	0.7	5	4	7	0.3	0.2	0.4	3	2	3
Philippines	Guihulngan City	0.6	0.4	0.7	6	4	8	0.3	0.2	0.4	3	2	4
Philippines	Guimba	0.6	0.4	0.7	6	5	7	0.3	0.2	0.4	4	3	5
Philippines	Guimbal	0.6	0.4	0.8	1	1	1	0.3	0.2	0.4	1	0	1
Philippines	Guinayangan	0.5	0.4	0.6	2	2	3	0.3	0.2	0.3	2	1	2
Philippines	Guindulman	0.5	0.4	0.7	2	2	3	0.3	0.2	0.4	1	1	1
Philippines	Guindulungan	0.7	0.5	0.9	3	2	4	0.3	0.3	0.4	0	0	0
Philippines	Guinobatan	0.6	0.4	0.7	6	5	7	0.3	0.3	0.4	3	2	4
Philippines	Guinsiliban	0.6	0.4	0.7	1	0	1	0.3	0.2	0.4	0	0	0
Philippines	Guipos	0.6	0.5	0.8	2	1	2	0.3	0.2	0.4	1	1	1
Philippines	Guiuan	0.6	0.5	0.8	1	1	1	0.4	0.3	0.5	1	1	1
Philippines	Gumaca	0.5	0.4	0.6	3	3	4	0.3	0.2	0.3	3	2	3
Philippines	Gutalac	0.6	0.5	0.8	3	2	3	0.3	0.3	0.4	2	1	2
Philippines	Hadji Mohammad Ajul	0.7	0.5	0.9	4	3	5	0.4	0.3	0.4	0	0	0
Philippines	Hadji Panglima Tahil	0.6	0.5	0.8	0	0	0	0.3	0.2	0.4	0	0	0
Philippines	Hagonoy	0.6	0.4	0.7	9	7	11	0.3	0.2	0.4	4	3	5
Philippines	Hagonoy	0.6	0.5	0.8	5	4	6	0.3	0.3	0.4	3	2	4
Philippines	Hamtic	0.5	0.4	0.7	3	2	3	0.3	0.2	0.4	2	1	3
Philippines	Hermosa	0.6	0.4	0.7	6	5	7	0.3	0.2	0.4	4	3	5
Philippines	Hernani	0.6	0.5	0.8	1	0	1	0.3	0.3	0.4	0	0	0
Philippines	Hilongos	0.6	0.4	0.7	2	2	3	0.3	0.2	0.4	1	1	2
Philippines	Himamaylan City	0.6	0.4	0.7	6	4	8	0.3	0.2	0.4	3	2	4
Philippines	Hinabangan	0.6	0.4	0.7	1	1	1	0.3	0.3	0.4	1	1	1
Philippines	Hinatuan	0.7	0.5	0.9	3	2	4	0.4	0.3	0.5	2	1	2
Philippines	Hindang	0.6	0.4	0.7	2	1	2	0.3	0.2	0.4	1	1	1
Philippines	Hingyon	0.6	0.5	0.7	1	1	1	0.3	0.3	0.4	0	0	1
Philippines	Hinigaran	0.6	0.4	0.7	6	4	7	0.3	0.2	0.4	3	2	3
Philippines	Hinoba-An	0.5	0.4	0.7	4	3	5	0.3	0.2	0.4	2	2	3
Philippines	Hinunangan	NA	NA	NA	NA	NA	NA	NA	NA	NA	NA	NA	NA
Philippines	Hinunangan	0.6	0.4	0.7	1	1	2	0.3	0.3	0.4	1	1	1
Philippines	Hinundayan	0.6	0.5	0.8	1	1	1	0.3	0.3	0.4	0	0	1
Philippines	Hungduan	0.6	0.5	0.8	1	1	1	0.3	0.3	0.4	1	0	1
Philippines	Iba	0.5	0.4	0.7	3	2	4	0.3	0.2	0.4	2	1	2
Philippines	Ibaan	0.5	0.4	0.6	3	3	4	0.3	0.2	0.3	2	2	3
Philippines	Ibajay	0.7	0.5	0.8	3	2	4	0.4	0.3	0.5	2	1	3
Philippines	Igbaras	0.6	0.4	0.8	2	1	2	0.3	0.2	0.4	1	1	1
Philippines	Iguig	0.7	0.5	0.9	2	2	3	0.4	0.3	0.5	1	1	1
Philippines	Ilagan	0.6	0.5	0.8	10	8	12	0.3	0.3	0.4	5	4	7
Philippines	Iligan City	0.6	0.4	0.7	21	17	28	0.3	0.2	0.4	13	10	17
Philippines	Ilog	0.6	0.4	0.8	5	3	6	0.3	0.2	0.4	2	2	3
Philippines	Iloilo City	0.7	0.5	0.9	18	13	23	0.4	0.3	0.5	9	7	11

Table 3: Diarrhoea Mortality by Unit *(continued)*

Country	Unit	2000 mortality mean rate (per 10,000)	lower rate (per 10,000)	upper rate (per 10,000)	mean count	lower count	upper count	2017 mortality mean rate (per 10,000)	lower rate (per 10,000)	upper rate (per 10,000)	mean count	lower count	upper count
Philippines	Imelda	0.6	0.5	0.8	1	1	2	0.3	0.3	0.4	1	1	2
Philippines	Impasug-Ong	0.5	0.4	0.7	2	2	3	0.3	0.2	0.4	2	2	3
Philippines	Imus	0.7	0.5	0.8	23	18	28	0.4	0.3	0.4	23	17	28
Philippines	Inabanga	0.5	0.4	0.7	3	2	4	0.3	0.2	0.4	1	1	2
Philippines	Indanan	0.6	0.5	0.8	15	12	19	0.3	0.2	0.4	2	1	2
Philippines	Indang	0.6	0.4	0.7	5	4	7	0.3	0.2	0.4	3	2	4
Philippines	Infanta	0.4	0.4	0.6	3	2	3	0.3	0.2	0.4	2	2	3
Philippines	Infanta	0.5	0.4	0.7	2	1	2	0.3	0.2	0.4	1	1	1
Philippines	Initao	0.5	0.4	0.7	2	1	2	0.3	0.2	0.4	1	1	1
Philippines	Inopacan	0.6	0.4	0.7	2	1	2	0.3	0.2	0.4	1	1	1
Philippines	Ipil	0.6	0.5	0.8	4	3	6	0.3	0.3	0.4	3	2	4
Philippines	Iriga City	0.6	0.4	0.7	6	4	7	0.3	0.2	0.4	4	3	5
Philippines	Irosin	0.6	0.4	0.7	4	3	5	0.3	0.3	0.4	3	2	3
Philippines	Isabel	0.6	0.4	0.7	2	2	3	0.3	0.3	0.4	2	1	2
Philippines	Isabela	0.6	0.4	0.7	5	3	6	0.3	0.2	0.4	2	2	3
Philippines	Isabela City	0.7	0.5	0.8	7	5	9	0.3	0.2	0.4	5	4	6
Philippines	Isulan	0.7	0.5	0.9	6	5	8	0.4	0.3	0.5	5	4	6
Philippines	Itbayat	NA	NA	NA	NA	NA	NA	NA	NA	NA	NA	NA	NA
Philippines	Itogon	0.6	0.5	0.7	3	2	4	0.3	0.3	0.4	3	2	3
Philippines	Ivana	NA	NA	NA	NA	NA	NA	NA	NA	NA	NA	NA	NA
Philippines	Ivisan	0.5	0.4	0.7	2	1	2	0.3	0.2	0.4	1	1	1
Philippines	Jabonga	0.6	0.5	0.8	2	1	2	0.3	0.3	0.4	1	1	1
Philippines	Jaen	0.5	0.4	0.7	5	4	6	0.3	0.2	0.4	3	2	4
Philippines	Jagna	0.5	0.4	0.7	2	2	3	0.3	0.2	0.4	1	1	1
Philippines	Jala-Jala	0.5	0.4	0.6	1	1	1	0.3	0.2	0.3	1	0	1
Philippines	Jamindan	0.6	0.4	0.7	3	2	4	0.3	0.2	0.4	2	1	2
Philippines	Janiuay	0.6	0.4	0.7	3	3	4	0.3	0.2	0.4	2	2	3
Philippines	Jaro	0.6	0.5	0.7	4	3	4	0.3	0.2	0.4	2	1	2
Philippines	Jasaan	0.5	0.4	0.7	3	3	4	0.3	0.2	0.4	3	2	3
Philippines	Javier	0.6	0.5	0.7	2	2	3	0.3	0.3	0.4	1	1	2
Philippines	Jetafe	0.5	0.4	0.7	1	1	2	0.3	0.2	0.4	1	1	1
Philippines	Jiabong	0.6	0.4	0.7	1	1	1	0.3	0.3	0.4	1	1	1
Philippines	Jimalalud	0.6	0.4	0.7	2	1	2	0.3	0.2	0.4	1	1	2
Philippines	Jimenez	0.6	0.4	0.7	1	1	2	0.3	0.2	0.4	1	1	1
Philippines	Jipapad	0.6	0.4	0.7	1	1	1	0.3	0.3	0.4	1	0	1
Philippines	Jolo	0.7	0.5	0.8	5	4	7	0.4	0.3	0.5	1	1	1
Philippines	Jomalig	0.5	0.4	0.7	0	0	1	0.3	0.2	0.4	0	0	0
Philippines	Jones	0.5	0.4	0.7	2	2	3	0.3	0.2	0.4	2	1	2
Philippines	Jordan	0.6	0.4	0.8	2	2	3	0.3	0.2	0.4	1	1	2
Philippines	Jose Abad Santos	0.6	0.5	0.8	4	3	5	0.4	0.3	0.5	4	3	5
Philippines	Jose Dalman	0.6	0.5	0.8	2	1	2	0.3	0.3	0.4	1	1	1
Philippines	Jose Panganiban	0.6	0.4	0.8	4	3	5	0.3	0.3	0.4	4	3	5
Philippines	Josefina	0.6	0.5	0.8	1	1	1	0.3	0.2	0.4	0	0	0
Philippines	Jovellar	0.5	0.4	0.7	2	1	2	0.3	0.3	0.4	1	1	1
Philippines	Juban	0.6	0.5	0.7	3	2	4	0.3	0.3	0.4	2	1	2
Philippines	Julita	0.6	0.4	0.7	1	1	2	0.3	0.2	0.4	1	1	1
Philippines	Kabacan	0.7	0.5	0.9	6	4	8	0.4	0.3	0.4	5	4	6
Philippines	Kabankalan City	0.6	0.4	0.7	14	10	18	0.3	0.2	0.4	7	5	8
Philippines	Kabasalan	0.6	0.5	0.8	3	2	4	0.3	0.3	0.4	2	1	2
Philippines	Kabayan	0.6	0.5	0.8	1	1	1	0.3	0.3	0.4	1	1	1
Philippines	Kabugao	0.7	0.5	0.8	1	1	1	0.3	0.3	0.4	1	1	1
Philippines	Kabuntalan	0.7	0.6	0.9	4	3	5	0.4	0.3	0.4	0	0	1
Philippines	Kadingilan	0.6	0.4	0.7	2	2	3	0.3	0.2	0.4	2	1	2
Philippines	Kalamansig	0.7	0.5	0.8	5	4	6	0.4	0.3	0.4	3	2	3
Philippines	Kalawit	0.6	0.5	0.8	3	2	3	0.3	0.3	0.4	1	1	2
Philippines	Kalayaan	0.5	0.4	0.6	1	1	2	0.3	0.2	0.3	1	0	1
Philippines	Kalibato Lake	0.5	0.4	0.6	0	0	0	0.3	0.2	0.3	0	0	0
Philippines	Kalibo	0.6	0.5	0.8	3	2	4	0.4	0.3	0.5	2	1	3
Philippines	Kalilangan	0.6	0.4	0.7	3	2	4	0.3	0.2	0.4	2	1	2
Philippines	Kalingalan Caluang	0.7	0.5	0.8	1	1	1	0.3	0.2	0.3	0	0	0

Table 3: Diarrhoea Mortality by Unit *(continued)*

Country	Unit	2000 mortality mean rate (per 10,000)	lower rate (per 10,000)	upper rate (per 10,000)	mean count	lower count	upper count	2017 mortality mean rate (per 10,000)	lower rate (per 10,000)	upper rate (per 10,000)	mean count	lower count	upper count
Philippines	Kalookan City	0.5	0.4	0.6	61	48	75	0.3	0.2	0.4	38	30	48
Philippines	Kananga	0.5	0.4	0.7	4	3	5	0.3	0.2	0.4	2	2	3
Philippines	Kapai	0.6	0.4	0.7	2	2	3	0.3	0.2	0.4	1	1	1
Philippines	Kapalong	0.5	0.4	0.7	4	3	5	0.3	0.2	0.4	3	2	4
Philippines	Kapangan	0.6	0.5	0.7	1	1	1	0.3	0.3	0.4	1	1	1
Philippines	Kapatagan	0.6	0.5	0.8	3	2	4	0.3	0.2	0.4	0	0	0
Philippines	Kapatagan	0.6	0.4	0.8	4	3	5	0.3	0.2	0.4	3	2	4
Philippines	Kasibu	0.5	0.4	0.7	2	2	3	0.3	0.2	0.4	1	1	2
Philippines	Katipunan	0.6	0.5	0.8	3	2	4	0.3	0.2	0.4	2	2	3
Philippines	Kauswagan	0.6	0.4	0.7	1	1	2	0.3	0.2	0.4	1	1	1
Philippines	Kawayan	0.6	0.4	0.7	1	1	1	0.3	0.2	0.4	1	1	1
Philippines	Kawit	0.7	0.5	0.8	4	3	5	0.3	0.3	0.4	3	2	4
Philippines	Kayapa	0.6	0.5	0.7	2	1	2	0.3	0.3	0.4	1	1	1
Philippines	Kiamba	0.7	0.5	0.9	3	2	4	0.4	0.3	0.5	2	1	2
Philippines	Kiangan	0.6	0.5	0.7	1	1	2	0.3	0.3	0.4	1	1	1
Philippines	Kibawe	0.6	0.4	0.7	3	2	4	0.3	0.2	0.4	2	1	2
Philippines	Kiblawan	0.6	0.5	0.8	2	2	3	0.3	0.3	0.4	1	1	2
Philippines	Kibungan	0.6	0.5	0.7	1	1	1	0.3	0.3	0.4	1	1	1
Philippines	Kidapawan City	0.6	0.5	0.8	8	6	11	0.4	0.3	0.4	6	4	7
Philippines	Kinoguitan	0.6	0.4	0.7	1	0	1	0.3	0.2	0.4	0	0	0
Philippines	Kitaotao	0.5	0.4	0.7	3	2	4	0.3	0.2	0.4	3	2	3
Philippines	Kitcharao	0.7	0.5	0.9	2	2	3	0.4	0.3	0.5	0	0	1
Philippines	Kolambugan	0.6	0.4	0.7	2	1	2	0.3	0.2	0.4	1	1	1
Philippines	Koronadal City	0.7	0.6	0.9	12	9	15	0.4	0.3	0.5	8	6	10
Philippines	Kumalarang	0.6	0.5	0.8	3	2	3	0.3	0.2	0.4	1	1	2
Philippines	La Carlota City	0.6	0.4	0.7	4	3	5	0.3	0.2	0.4	2	1	2
Philippines	La Castellana	0.6	0.4	0.7	6	4	7	0.3	0.2	0.4	3	2	3
Philippines	La Libertad	0.6	0.5	0.8	1	1	1	0.3	0.3	0.4	0	0	0
Philippines	La Libertad	0.6	0.4	0.7	2	2	3	0.3	0.2	0.4	1	1	2
Philippines	La Paz	0.6	0.4	0.7	2	1	2	0.3	0.2	0.4	1	1	1
Philippines	La Paz	0.6	0.5	0.8	2	2	2	0.3	0.3	0.4	2	1	2
Philippines	La Paz	0.5	0.4	0.7	5	4	6	0.3	0.2	0.4	2	2	3
Philippines	La Paz	0.6	0.5	0.8	1	1	1	0.3	0.3	0.4	0	0	1
Philippines	La Trinidad	0.7	0.5	0.9	10	8	12	0.4	0.3	0.5	7	6	9
Philippines	Laak	0.5	0.4	0.7	5	4	6	0.3	0.2	0.4	3	2	4
Philippines	Labangan	0.6	0.5	0.8	3	3	4	0.3	0.2	0.4	2	2	3
Philippines	Labason	0.6	0.5	0.8	4	3	5	0.3	0.3	0.4	2	1	2
Philippines	Labo	0.6	0.5	0.8	7	5	9	0.4	0.3	0.4	5	4	6
Philippines	Labrador	0.6	0.4	0.7	2	2	3	0.3	0.2	0.4	1	1	1
Philippines	Lacub	0.7	0.5	0.9	0	0	0	0.4	0.3	0.5	0	0	0
Philippines	Lagangilang	0.6	0.5	0.8	1	1	1	0.3	0.3	0.4	0	0	1
Philippines	Lagawe	0.6	0.5	0.7	1	1	1	0.3	0.3	0.4	1	1	1
Philippines	Lagayan	0.6	0.5	0.8	0	0	0	0.3	0.3	0.4	0	0	0
Philippines	Lagonglong	0.6	0.4	0.7	1	0	1	0.3	0.2	0.4	0	0	1
Philippines	Lagonoy	0.5	0.4	0.7	3	2	4	0.3	0.2	0.4	3	2	3
Philippines	Laguindingan	0.6	0.4	0.7	1	1	1	0.3	0.2	0.4	1	1	1
Philippines	Laguna lake	0.6	0.5	0.7	61	49	76	0.3	0.2	0.4	36	28	45
Philippines	Lake Sebu	0.7	0.5	0.9	4	3	6	0.4	0.3	0.5	7	6	9
Philippines	Lakewood	0.6	0.5	0.8	1	1	2	0.3	0.3	0.4	1	1	1
Philippines	Lakewood Lake	0.6	0.5	0.8	0	0	0	0.3	0.3	0.4	0	0	0
Philippines	Lal-Lo	0.7	0.5	0.9	4	3	5	0.3	0.3	0.4	2	2	3
Philippines	Lala	0.6	0.4	0.8	4	3	5	0.3	0.2	0.4	3	2	4
Philippines	Lambayong	0.7	0.5	0.9	6	5	7	0.4	0.3	0.5	4	3	4
Philippines	Lambunao	0.6	0.4	0.7	4	3	5	0.3	0.2	0.4	3	2	4
Philippines	Lamitan City	0.7	0.5	0.8	13	10	16	0.3	0.2	0.4	2	1	2
Philippines	Lamut	0.6	0.5	0.7	1	1	2	0.3	0.3	0.4	1	1	1
Philippines	Lanao Lake	0.6	0.5	0.8	22	17	29	0.3	0.2	0.4	6	5	8
Philippines	Langiden	0.6	0.5	0.8	1	0	1	0.3	0.3	0.4	0	0	0
Philippines	Languyan	0.6	0.5	0.8	6	4	7	0.3	0.2	0.4	1	1	1
Philippines	Lantapan	0.5	0.4	0.7	4	3	5	0.3	0.2	0.4	3	2	3

Table 3: Diarrhoea Mortality by Unit *(continued)*

Country	Unit	2000 mortality mean rate (per 10,000)	lower rate (per 10,000)	upper rate (per 10,000)	mean count	lower count	upper count	2017 mortality mean rate (per 10,000)	lower rate (per 10,000)	upper rate (per 10,000)	mean count	lower count	upper count
Philippines	Lantawan	0.7	0.5	0.8	7	6	9	0.3	0.2	0.4	1	0	1
Philippines	Lanuza	0.7	0.5	0.9	1	1	1	0.4	0.3	0.5	1	0	1
Philippines	Laoac	0.6	0.5	0.7	3	2	3	0.3	0.3	0.4	1	1	2
Philippines	Laoag City	0.6	0.4	0.8	7	5	9	0.3	0.2	0.4	3	3	4
Philippines	Laoang	0.6	0.5	0.8	5	3	6	0.4	0.3	0.5	3	2	4
Philippines	Lapinig	0.6	0.5	0.8	1	1	1	0.3	0.3	0.5	1	0	1
Philippines	Lapu-Lapu City	0.6	0.4	0.7	10	8	13	0.3	0.3	0.4	14	11	18
Philippines	Lapuyan	0.6	0.5	0.8	2	2	3	0.3	0.2	0.4	1	1	1
Philippines	Larena	0.6	0.4	0.7	1	1	1	0.3	0.2	0.4	0	0	0
Philippines	Las Navas	0.6	0.4	0.7	3	2	3	0.3	0.3	0.4	3	2	3
Philippines	Las Nieves	0.6	0.5	0.7	2	2	3	0.3	0.2	0.4	2	1	2
Philippines	Las Piñas	0.6	0.5	0.7	35	28	43	0.3	0.2	0.4	21	16	27
Philippines	Lasam	0.7	0.5	0.9	3	3	4	0.4	0.3	0.5	2	1	2
Philippines	Laua-An	0.6	0.5	0.7	1	1	2	0.3	0.2	0.4	1	1	1
Philippines	Laur	0.5	0.4	0.6	2	2	3	0.3	0.2	0.4	1	1	2
Philippines	Laurel	0.5	0.4	0.7	3	2	3	0.3	0.2	0.4	1	1	2
Philippines	Lavezares	0.5	0.4	0.7	2	1	2	0.3	0.2	0.4	1	1	2
Philippines	Lawaan	0.6	0.5	0.7	1	1	1	0.3	0.3	0.4	0	0	1
Philippines	Lazi	0.6	0.4	0.7	1	1	1	0.3	0.2	0.4	1	0	1
Philippines	Lebak	0.7	0.5	0.8	7	5	9	0.3	0.3	0.4	5	4	6
Philippines	Leganes	0.6	0.5	0.8	2	1	2	0.3	0.3	0.4	1	1	2
Philippines	Legazpi City	0.6	0.5	0.7	10	8	12	0.3	0.3	0.4	6	5	7
Philippines	Lemery	0.5	0.4	0.7	4	3	5	0.3	0.2	0.3	3	2	3
Philippines	Lemery	0.6	0.4	0.7	2	1	2	0.3	0.2	0.4	1	1	1
Philippines	Leon	0.6	0.5	0.8	3	2	4	0.3	0.2	0.4	2	1	2
Philippines	Leyte	0.6	0.4	0.7	3	2	4	0.3	0.2	0.4	2	2	3
Philippines	Lezo	0.6	0.4	0.8	1	1	2	0.3	0.3	0.4	1	1	1
Philippines	Lian	0.6	0.4	0.7	4	3	5	0.3	0.2	0.4	2	2	3
Philippines	Lianga	0.7	0.5	0.9	2	2	3	0.4	0.3	0.5	1	1	2
Philippines	Libacao	0.6	0.5	0.8	2	2	3	0.4	0.3	0.5	1	1	2
Philippines	Libagon	0.6	0.5	0.8	1	1	2	0.3	0.3	0.4	1	1	1
Philippines	Libertad	0.5	0.4	0.7	1	1	1	0.3	0.2	0.4	0	0	1
Philippines	Libertad	0.7	0.5	0.8	1	1	1	0.4	0.3	0.5	1	1	1
Philippines	Libjo	0.7	0.5	0.8	1	1	1	0.4	0.3	0.5	0	0	1
Philippines	Libmanan	0.6	0.4	0.7	7	5	9	0.3	0.2	0.4	5	4	6
Philippines	Libon	0.6	0.5	0.7	6	5	8	0.3	0.2	0.4	4	3	4
Philippines	Libona	0.5	0.4	0.7	2	2	3	0.3	0.2	0.4	2	1	2
Philippines	Libungan	0.6	0.5	0.8	4	3	5	0.3	0.3	0.4	2	2	3
Philippines	Licab	0.6	0.5	0.7	1	1	2	0.3	0.2	0.4	1	1	1
Philippines	Licuan-Baay	0.7	0.5	0.9	0	0	0	0.4	0.3	0.4	0	0	0
Philippines	Lidlidda	0.6	0.4	0.7	0	0	0	0.3	0.2	0.4	0	0	0
Philippines	Ligao City	0.6	0.4	0.7	8	6	10	0.3	0.2	0.4	5	4	6
Philippines	Lila	0.6	0.4	0.7	1	0	1	0.3	0.2	0.4	0	0	1
Philippines	Liliw	0.5	0.4	0.6	2	1	2	0.3	0.2	0.3	1	1	1
Philippines	Liloan	0.5	0.4	0.7	4	3	6	0.3	0.2	0.4	4	3	5
Philippines	Liloan	0.6	0.5	0.8	1	1	1	0.3	0.3	0.4	1	0	1
Philippines	Liloy	0.6	0.5	0.8	3	2	4	0.3	0.3	0.4	2	1	2
Philippines	Limasawa	NA	NA	NA	NA	NA	NA	NA	NA	NA	NA	NA	NA
Philippines	Limay	0.5	0.4	0.7	2	2	3	0.3	0.2	0.4	1	1	2
Philippines	Linamon	0.6	0.4	0.7	1	1	1	0.3	0.2	0.4	1	1	1
Philippines	Linapacan	0.6	0.4	0.7	0	0	0	0.3	0.3	0.5	0	0	1
Philippines	Lingayen	0.6	0.5	0.7	7	6	9	0.3	0.3	0.4	4	3	5
Philippines	Lingig	0.7	0.5	0.8	2	2	3	0.4	0.3	0.5	2	1	2
Philippines	Lipa City	0.5	0.4	0.7	17	13	22	0.3	0.2	0.4	13	10	15
Philippines	Llanera	0.5	0.4	0.7	2	2	3	0.3	0.2	0.4	2	1	2
Philippines	Llorente	0.6	0.5	0.8	1	1	2	0.3	0.3	0.4	1	1	1
Philippines	Loay	0.5	0.4	0.7	1	1	1	0.3	0.2	0.4	0	0	1
Philippines	Lobo	0.5	0.4	0.7	3	2	3	0.3	0.2	0.4	1	1	1
Philippines	Loboc	0.6	0.4	0.7	1	1	1	0.3	0.2	0.4	0	0	1
Philippines	Looc	0.5	0.4	0.6	1	1	1	0.3	0.2	0.4	0	0	0

Table 3: Diarrhoea Mortality by Unit *(continued)*

Country	Unit	2000 mortality mean rate (per 10,000)	lower rate (per 10,000)	upper rate (per 10,000)	mean count	lower count	upper count	2017 mortality mean rate (per 10,000)	lower rate (per 10,000)	upper rate (per 10,000)	mean count	lower count	upper count
Philippines	Looc	0.6	0.4	0.7	1	1	2	0.3	0.2	0.4	1	1	1
Philippines	Loon	0.6	0.4	0.7	2	2	3	0.3	0.2	0.4	1	1	1
Philippines	Lope de Vega	0.6	0.4	0.7	1	1	1	0.3	0.3	0.4	1	1	1
Philippines	Lopez	0.5	0.4	0.6	6	4	7	0.3	0.2	0.3	4	3	5
Philippines	Lopez Jaena	0.6	0.4	0.7	1	1	1	0.3	0.2	0.4	1	1	1
Philippines	Loreto	0.6	0.5	0.8	3	3	4	0.3	0.3	0.4	3	2	4
Philippines	Loreto	0.7	0.5	0.8	1	1	1	0.4	0.3	0.5	0	0	0
Philippines	Los Baños	0.6	0.4	0.7	4	3	6	0.3	0.2	0.4	3	2	3
Philippines	Luba	0.6	0.5	0.8	0	0	1	0.3	0.3	0.4	0	0	0
Philippines	Lubang	0.5	0.4	0.7	2	2	3	0.3	0.2	0.4	0	0	0
Philippines	Lubao	0.5	0.4	0.7	10	8	12	0.3	0.2	0.4	6	5	7
Philippines	Lubuagan	0.7	0.5	0.8	1	1	2	0.4	0.3	0.4	0	0	1
Philippines	Lucban	0.5	0.4	0.6	3	2	3	0.3	0.2	0.3	1	1	2
Philippines	Lucena City	0.5	0.4	0.7	13	10	17	0.3	0.2	0.4	8	7	10
Philippines	Lugait	0.6	0.4	0.7	1	1	1	0.3	0.2	0.4	1	0	1
Philippines	Lugus	0.6	0.5	0.8	2	1	2	0.3	0.2	0.4	0	0	1
Philippines	Luisiana	0.5	0.4	0.6	1	1	2	0.3	0.2	0.3	1	1	1
Philippines	Lumba-Bayabao	0.6	0.5	0.8	10	8	13	0.3	0.2	0.3	1	1	1
Philippines	Lumbaca Unayan	0.6	0.4	0.7	1	1	1	0.2	0.2	0.3	0	0	0
Philippines	Lumban	0.5	0.4	0.6	2	1	2	0.3	0.2	0.3	1	1	1
Philippines	Lumbatan	0.6	0.5	0.8	2	2	3	0.3	0.2	0.3	0	0	0
Philippines	Lumbayanague	0.6	0.5	0.8	2	2	3	0.3	0.2	0.3	0	0	0
Philippines	Luna	0.5	0.4	0.7	2	1	2	0.3	0.2	0.3	1	1	1
Philippines	Luna	0.6	0.5	0.8	1	1	2	0.3	0.3	0.4	1	1	1
Philippines	Luna	0.6	0.5	0.8	1	1	2	0.3	0.3	0.4	1	1	1
Philippines	Lupao	0.6	0.4	0.7	2	2	3	0.3	0.2	0.4	2	1	2
Philippines	Lupi	0.6	0.4	0.7	2	2	3	0.3	0.2	0.4	2	1	2
Philippines	Lupon	0.6	0.5	0.7	4	3	6	0.3	0.3	0.4	2	2	3
Philippines	Lutayan	0.7	0.5	0.9	5	4	6	0.4	0.3	0.5	4	3	5
Philippines	Luuk	0.6	0.5	0.8	4	3	5	0.3	0.2	0.3	0	0	1
Philippines	M'Lang	0.7	0.5	0.9	7	5	10	0.4	0.3	0.5	4	3	5
Philippines	Ma-Ayon	0.6	0.4	0.7	3	2	3	0.3	0.2	0.4	1	1	2
Philippines	Maasim	0.7	0.6	0.9	3	2	4	0.4	0.3	0.5	2	2	3
Philippines	Maasin	0.6	0.4	0.7	2	2	3	0.3	0.2	0.4	2	1	2
Philippines	Maasin City	0.6	0.5	0.8	5	3	6	0.3	0.2	0.4	2	2	3
Philippines	Mabalacat	0.5	0.4	0.7	15	12	20	0.3	0.2	0.4	10	8	12
Philippines	Mabinay	0.5	0.4	0.7	6	4	7	0.3	0.2	0.4	3	3	4
Philippines	Mabini	0.5	0.4	0.6	1	1	1	0.3	0.2	0.3	1	1	1
Philippines	Mabini	0.5	0.4	0.7	2	1	2	0.3	0.2	0.4	1	1	1
Philippines	Mabini	0.5	0.4	0.7	3	3	4	0.3	0.2	0.4	2	1	2
Philippines	Mabini	0.5	0.4	0.7	2	1	2	0.3	0.2	0.4	1	1	1
Philippines	Mabitac	0.5	0.4	0.6	3	2	3	0.3	0.2	0.3	2	1	2
Philippines	Mabuhay	0.6	0.5	0.8	2	1	2	0.3	0.3	0.4	1	1	2
Philippines	Macabebe	0.6	0.4	0.7	5	4	6	0.3	0.2	0.4	2	2	3
Philippines	Macalelon	0.5	0.3	0.6	1	1	2	0.3	0.2	0.3	1	1	1
Philippines	Macarthur	0.6	0.4	0.7	1	1	2	0.3	0.3	0.4	1	1	1
Philippines	Maco	0.5	0.4	0.7	5	4	6	0.3	0.2	0.4	3	2	3
Philippines	Maconacon	0.6	0.5	0.8	0	0	1	0.3	0.3	0.4	0	0	0
Philippines	Macrohon	0.6	0.5	0.8	1	0	1	0.3	0.2	0.4	0	0	0
Philippines	Madalag	0.6	0.5	0.8	1	1	2	0.4	0.3	0.5	1	1	1
Philippines	Madalum	0.6	0.5	0.8	2	2	3	0.3	0.2	0.3	0	0	0
Philippines	Madamba	0.6	0.5	0.8	3	2	4	0.3	0.2	0.3	0	0	1
Philippines	Maddela	0.5	0.4	0.6	2	2	3	0.3	0.2	0.4	2	1	2
Philippines	Madrid	0.7	0.5	0.8	1	1	1	0.4	0.3	0.5	1	0	1
Philippines	Madridejos	0.5	0.4	0.7	1	1	2	0.3	0.2	0.4	1	1	2
Philippines	Magalang	0.5	0.4	0.7	7	6	9	0.3	0.2	0.3	4	3	5
Philippines	Magallanes	0.6	0.5	0.7	2	2	3	0.3	0.3	0.4	1	1	2
Philippines	Magallanes	0.6	0.4	0.7	1	1	2	0.3	0.2	0.4	1	1	1
Philippines	Magallanes	0.6	0.5	0.8	1	1	2	0.3	0.2	0.4	1	0	1
Philippines	Magarao	0.6	0.5	0.7	3	2	4	0.3	0.3	0.4	2	2	3

Table 3: Diarrhoea Mortality by Unit *(continued)*

Country	Unit	2000 mortality mean rate (per 10,000)	lower rate (per 10,000)	upper rate (per 10,000)	mean count	lower count	upper count	2017 mortality mean rate (per 10,000)	lower rate (per 10,000)	upper rate (per 10,000)	mean count	lower count	upper count
Philippines	Magdalena	0.5	0.4	0.6	1	1	1	0.3	0.2	0.3	1	1	1
Philippines	Magdiwang	0.5	0.4	0.6	1	1	1	0.3	0.2	0.4	1	1	1
Philippines	Magpet	0.6	0.5	0.8	5	3	6	0.3	0.3	0.4	3	2	3
Philippines	Magsaysay	0.6	0.5	0.7	1	1	1	0.3	0.2	0.4	1	1	2
Philippines	Magsaysay	0.6	0.5	0.8	1	1	1	0.3	0.2	0.4	1	0	1
Philippines	Magsaysay	0.6	0.4	0.7	2	1	2	0.3	0.2	0.4	2	1	2
Philippines	Magsaysay	0.6	0.5	0.8	2	2	3	0.4	0.3	0.5	2	1	2
Philippines	Magsaysay	0.6	0.5	0.8	3	2	4	0.3	0.3	0.4	2	2	3
Philippines	Magsingal	0.6	0.4	0.8	2	1	3	0.3	0.2	0.4	1	1	1
Philippines	Maguing	0.6	0.5	0.8	5	4	6	0.3	0.2	0.3	0	0	1
Philippines	Mahaplag	0.6	0.5	0.7	2	2	3	0.3	0.3	0.4	1	1	1
Philippines	Mahatao	NA	NA	NA	NA	NA	NA	NA	NA	NA	NA	NA	NA
Philippines	Mahayag	0.6	0.5	0.8	5	4	6	0.3	0.2	0.4	2	2	3
Philippines	Mahinog	0.6	0.4	0.7	1	1	1	0.3	0.2	0.4	0	0	0
Philippines	Maigo	0.6	0.4	0.7	2	1	2	0.3	0.2	0.4	1	1	1
Philippines	Maimbung	0.6	0.5	0.8	3	2	4	0.3	0.2	0.4	0	0	0
Philippines	Mainit	0.7	0.5	0.9	1	1	2	0.4	0.3	0.5	1	1	1
Philippines	Mainit Lake	0.7	0.5	0.9	1	1	1	0.4	0.3	0.5	0	0	0
Philippines	Mainit Lake	0.7	0.5	0.8	1	1	1	0.4	0.3	0.5	1	1	1
Philippines	Maitum	0.7	0.5	0.9	2	2	3	0.4	0.3	0.5	2	2	3
Philippines	Majayjay	0.5	0.4	0.6	2	1	2	0.3	0.2	0.3	1	1	2
Philippines	Makati City	0.5	0.4	0.6	49	39	61	0.3	0.2	0.3	22	18	28
Philippines	Makato	0.6	0.5	0.8	2	1	2	0.4	0.3	0.5	1	1	1
Philippines	Makilala	0.7	0.5	0.8	6	5	8	0.4	0.3	0.4	5	4	6
Philippines	Malabang	0.6	0.5	0.8	5	4	6	0.3	0.2	0.3	1	1	1
Philippines	Malabon	0.6	0.5	0.7	19	16	24	0.3	0.2	0.4	10	8	12
Philippines	Malabuyoc	0.5	0.4	0.7	1	1	1	0.3	0.2	0.4	0	0	1
Philippines	Malalag	0.6	0.5	0.8	4	3	5	0.3	0.3	0.4	2	2	3
Philippines	Malangas	0.6	0.5	0.8	2	2	3	0.3	0.3	0.4	1	1	2
Philippines	Malapatan	0.7	0.5	0.9	6	5	8	0.4	0.3	0.5	5	4	7
Philippines	Malasiqui	0.6	0.5	0.7	9	7	12	0.3	0.2	0.4	5	4	6
Philippines	Malay	0.6	0.5	0.8	2	2	3	0.3	0.2	0.4	2	1	2
Philippines	Malaybalay City	0.5	0.4	0.7	10	8	13	0.3	0.2	0.3	7	5	8
Philippines	Malibcong	0.7	0.5	0.9	0	0	0	0.4	0.3	0.5	0	0	0
Philippines	Malilipot	0.6	0.4	0.7	3	2	4	0.3	0.3	0.4	2	1	2
Philippines	Malimono	0.7	0.5	0.8	1	1	2	0.4	0.3	0.5	1	1	1
Philippines	Malinao	0.5	0.4	0.7	4	3	5	0.3	0.3	0.4	3	2	3
Philippines	Malinao	0.6	0.5	0.8	2	1	3	0.3	0.3	0.4	1	1	2
Philippines	Malita	0.6	0.5	0.8	10	8	12	0.3	0.3	0.4	6	5	7
Philippines	Malitbog	0.6	0.4	0.7	1	1	1	0.3	0.2	0.4	1	0	1
Philippines	Malitbog	0.5	0.4	0.7	2	1	2	0.3	0.2	0.4	1	1	1
Philippines	Mallig	0.7	0.5	0.8	2	2	3	0.4	0.3	0.5	1	1	2
Philippines	Malolos City	0.6	0.5	0.7	15	12	19	0.3	0.2	0.4	8	6	10
Philippines	Malungon	0.7	0.5	0.9	9	7	12	0.4	0.3	0.5	4	3	5
Philippines	Maluso	0.7	0.5	0.8	5	4	6	0.3	0.2	0.4	0	0	0
Philippines	Malvar	0.5	0.4	0.6	3	3	4	0.3	0.2	0.3	2	2	3
Philippines	Mamasapano	0.7	0.6	0.9	6	5	8	0.3	0.3	0.4	0	0	1
Philippines	Mambajao	0.5	0.4	0.7	2	2	3	0.3	0.2	0.4	1	1	1
Philippines	Mamburao	0.5	0.4	0.7	2	2	3	0.3	0.2	0.4	2	2	3
Philippines	Mambusao	0.6	0.4	0.7	3	2	4	0.3	0.2	0.4	1	1	2
Philippines	Manabo	0.6	0.5	0.8	1	1	1	0.3	0.3	0.4	0	0	0
Philippines	Manaoag	0.6	0.5	0.8	4	3	5	0.3	0.3	0.4	2	2	3
Philippines	Manapla	0.5	0.4	0.7	4	3	5	0.3	0.2	0.4	2	1	2
Philippines	Manay	0.5	0.4	0.7	2	2	3	0.3	0.2	0.4	2	1	2
Philippines	Mandaluyong	0.5	0.4	0.6	16	13	20	0.3	0.2	0.3	7	6	9
Philippines	Mandaon	0.6	0.5	0.8	3	2	3	0.3	0.3	0.4	3	2	3
Philippines	Mandaue City	0.6	0.4	0.8	13	10	17	0.4	0.3	0.5	10	8	13
Philippines	Mangaldan	0.6	0.5	0.8	7	5	9	0.3	0.3	0.4	5	4	6
Philippines	Mangatarem	0.6	0.4	0.7	5	4	7	0.3	0.2	0.4	4	3	4
Philippines	Mangudadatu	0.7	0.6	0.9	2	1	2	0.4	0.3	0.5	0	0	1

Table 3: Diarrhoea Mortality by Unit *(continued)*

Country	Unit	2000 mortality mean rate (per 10,000)	lower rate (per 10,000)	upper rate (per 10,000)	mean count	lower count	upper count	2017 mortality mean rate (per 10,000)	lower rate (per 10,000)	upper rate (per 10,000)	mean count	lower count	upper count
Philippines	Manila	0.5	0.4	0.6	88	71	109	0.3	0.2	0.3	40	32	50
Philippines	Manito	0.6	0.4	0.7	2	1	2	0.3	0.3	0.4	1	1	1
Philippines	Manjuyod	0.6	0.4	0.7	3	2	4	0.3	0.2	0.4	2	1	2
Philippines	Mankayan	0.6	0.4	0.7	3	2	3	0.3	0.2	0.4	2	1	2
Philippines	Manolo Fortich	0.5	0.4	0.7	4	3	5	0.3	0.2	0.3	3	3	4
Philippines	Mansalay	0.5	0.4	0.7	3	2	4	0.3	0.2	0.4	3	3	4
Philippines	Manticao	0.5	0.4	0.7	1	1	1	0.3	0.2	0.4	1	0	1
Philippines	Manukan	0.6	0.5	0.8	3	2	3	0.3	0.3	0.4	2	1	2
Philippines	Mapanas	0.6	0.5	0.8	1	1	2	0.4	0.3	0.5	1	1	1
Philippines	Mapandan	0.6	0.5	0.7	2	2	3	0.3	0.3	0.4	1	1	2
Philippines	Mapun	0.6	0.5	0.8	3	2	3	0.3	0.2	0.4	0	0	0
Philippines	Marabut	0.6	0.5	0.8	1	1	1	0.3	0.3	0.4	1	1	1
Philippines	Maragondon	0.6	0.5	0.7	3	3	4	0.3	0.2	0.4	2	1	2
Philippines	Maragusan	0.5	0.4	0.7	3	3	4	0.3	0.2	0.4	3	2	3
Philippines	Maramag	0.5	0.4	0.7	6	4	7	0.3	0.2	0.4	4	3	5
Philippines	Marantao	0.6	0.4	0.8	4	3	5	0.3	0.2	0.4	1	1	1
Philippines	Marawi City	0.6	0.5	0.8	9	7	11	0.3	0.2	0.4	5	4	6
Philippines	Marcos	0.6	0.4	0.7	1	1	2	0.3	0.2	0.4	1	1	1
Philippines	Margosatubig	0.6	0.5	0.8	3	2	4	0.3	0.3	0.4	1	1	2
Philippines	Maria	0.6	0.4	0.7	1	1	1	0.3	0.2	0.4	0	0	0
Philippines	Maria Aurora	0.5	0.4	0.6	2	2	2	0.3	0.2	0.4	1	1	2
Philippines	Maribojoc	0.6	0.4	0.7	0	0	1	0.3	0.2	0.4	0	0	0
Philippines	Marihatag	0.7	0.5	0.9	1	1	2	0.4	0.3	0.5	1	1	1
Philippines	Marikina	0.5	0.4	0.6	24	19	30	0.3	0.2	0.3	12	9	15
Philippines	Marilao	0.6	0.5	0.7	12	10	15	0.3	0.3	0.4	9	8	12
Philippines	Maripipi	0.5	0.4	0.7	0	0	0	0.3	0.2	0.4	0	0	0
Philippines	Mariveles	0.5	0.4	0.7	6	5	7	0.3	0.2	0.4	5	4	6
Philippines	Marogong	0.6	0.5	0.7	3	3	4	0.2	0.2	0.3	0	0	1
Philippines	Masantol	0.6	0.4	0.7	5	4	6	0.3	0.2	0.4	2	2	3
Philippines	Masbate City	0.6	0.5	0.8	7	6	9	0.4	0.3	0.4	4	3	6
Philippines	Masinloc	0.5	0.4	0.7	2	2	3	0.3	0.2	0.4	2	1	2
Philippines	Masiu	0.6	0.5	0.8	3	2	4	0.3	0.2	0.3	0	0	1
Philippines	Maslog	0.6	0.5	0.7	0	0	1	0.3	0.3	0.4	0	0	0
Philippines	Mataas Na Kahoy	0.5	0.4	0.6	1	1	1	0.3	0.2	0.3	1	1	1
Philippines	Matag-Ob	0.5	0.4	0.7	1	1	2	0.3	0.2	0.4	1	1	1
Philippines	Matalam	0.6	0.5	0.8	5	4	7	0.3	0.3	0.4	4	3	6
Philippines	Matalom	0.6	0.4	0.7	2	1	2	0.3	0.2	0.4	1	1	1
Philippines	Matanao	0.6	0.5	0.8	4	3	6	0.3	0.3	0.4	2	2	3
Philippines	Matanog	0.6	0.5	0.8	6	4	7	0.3	0.2	0.4	0	0	1
Philippines	Mati City	0.6	0.4	0.7	9	7	11	0.3	0.3	0.4	5	4	6
Philippines	Matnog	0.6	0.4	0.7	2	2	3	0.3	0.3	0.4	2	1	2
Philippines	Matuguinao	0.6	0.5	0.7	1	1	1	0.3	0.3	0.4	0	0	1
Philippines	Matungao	0.6	0.4	0.7	1	1	1	0.3	0.2	0.4	1	1	1
Philippines	Mauban	0.4	0.3	0.6	3	2	4	0.3	0.2	0.3	2	2	3
Philippines	Mawab	0.5	0.4	0.7	2	2	3	0.3	0.2	0.4	1	1	2
Philippines	Mayantoc	0.5	0.4	0.7	2	1	2	0.3	0.2	0.3	1	1	1
Philippines	Maydolong	0.6	0.5	0.8	1	1	1	0.3	0.3	0.4	1	0	1
Philippines	Mayorga	0.6	0.4	0.7	1	1	1	0.3	0.3	0.4	0	0	1
Philippines	Mayoyao	0.6	0.5	0.8	2	1	2	0.3	0.3	0.4	1	1	1
Philippines	Medellin	0.5	0.4	0.7	2	1	2	0.3	0.2	0.4	2	1	2
Philippines	Medina	0.6	0.4	0.7	2	1	2	0.3	0.2	0.4	1	1	2
Philippines	Mendez	0.6	0.4	0.7	1	1	1	0.3	0.2	0.4	1	0	1
Philippines	Mercedes	0.6	0.5	0.8	0	0	0	0.4	0.3	0.4	0	0	0
Philippines	Mercedes	0.6	0.5	0.8	2	2	3	0.4	0.3	0.5	2	1	2
Philippines	Merida	0.6	0.4	0.7	2	1	2	0.3	0.2	0.4	1	1	1
Philippines	Mexico	0.5	0.4	0.7	13	11	17	0.3	0.2	0.4	8	6	9
Philippines	Meycauayan City	0.6	0.5	0.7	19	15	23	0.3	0.3	0.4	11	9	14
Philippines	Miagao	0.6	0.4	0.7	3	2	4	0.3	0.2	0.4	2	1	2
Philippines	Midsalip	0.6	0.5	0.8	3	2	4	0.3	0.2	0.4	2	1	2
Philippines	Midsayap	0.7	0.5	0.9	11	8	13	0.3	0.3	0.4	6	5	8

Table 3: Diarrhoea Mortality by Unit *(continued)*

Country	Unit	2000 mortality mean rate (per 10,000)	lower rate (per 10,000)	upper rate (per 10,000)	mean count	lower count	upper count	2017 mortality mean rate (per 10,000)	lower rate (per 10,000)	upper rate (per 10,000)	mean count	lower count	upper count
Philippines	Milagros	0.6	0.5	0.8	4	3	5	0.4	0.3	0.4	3	2	4
Philippines	Milaor	0.6	0.4	0.7	5	4	6	0.3	0.3	0.4	3	3	4
Philippines	Mina	0.6	0.5	0.8	1	1	2	0.3	0.2	0.4	1	1	1
Philippines	Minalabac	0.6	0.4	0.7	4	3	4	0.3	0.2	0.4	3	2	3
Philippines	Minalin	0.5	0.4	0.7	3	3	4	0.3	0.2	0.4	2	2	2
Philippines	Minglanilla	0.6	0.4	0.7	3	2	4	0.3	0.3	0.4	3	2	3
Philippines	Moalboal	0.5	0.4	0.7	2	2	3	0.3	0.2	0.4	1	1	1
Philippines	Mobo	0.6	0.5	0.8	4	3	5	0.3	0.3	0.4	3	2	3
Philippines	Mogpog	0.5	0.4	0.7	2	2	3	0.3	0.2	0.4	1	1	1
Philippines	Moises Padilla	0.6	0.4	0.7	4	3	5	0.3	0.2	0.4	2	1	2
Philippines	Molave	0.6	0.5	0.8	3	2	3	0.3	0.2	0.4	1	1	2
Philippines	Moncada	0.6	0.4	0.7	4	3	5	0.3	0.2	0.4	2	2	3
Philippines	Mondragon	0.6	0.5	0.7	3	3	4	0.4	0.3	0.4	3	2	3
Philippines	Monkayo	0.5	0.4	0.7	7	5	9	0.3	0.2	0.4	4	3	5
Philippines	Monreal	0.6	0.5	0.8	1	1	2	0.3	0.3	0.4	1	1	2
Philippines	Montevista	0.5	0.4	0.7	2	1	3	0.3	0.2	0.4	2	1	2
Philippines	Morong	0.6	0.5	0.7	3	2	4	0.3	0.2	0.4	1	1	1
Philippines	Morong	0.6	0.4	0.7	4	3	5	0.3	0.2	0.4	2	2	3
Philippines	Motiong	0.6	0.4	0.7	1	1	1	0.3	0.3	0.4	1	1	1
Philippines	Mulanay	0.5	0.4	0.6	3	2	4	0.3	0.2	0.3	2	2	3
Philippines	Mulondo	0.6	0.5	0.8	2	2	3	0.3	0.2	0.4	0	0	0
Philippines	Munai	0.6	0.5	0.8	2	2	3	0.3	0.2	0.3	2	1	2
Philippines	Muñoz City	0.6	0.4	0.7	5	4	6	0.3	0.2	0.4	3	2	4
Philippines	Muntinlupa	0.5	0.4	0.7	22	17	27	0.3	0.2	0.4	11	9	14
Philippines	Murcia	0.6	0.4	0.7	6	5	8	0.3	0.2	0.4	4	3	4
Philippines	Mutia	0.6	0.5	0.8	1	1	1	0.3	0.2	0.4	0	0	1
Philippines	Naawan	0.5	0.4	0.7	1	1	1	0.3	0.2	0.4	0	0	1
Philippines	Nabas	0.7	0.5	0.8	2	1	2	0.4	0.3	0.4	1	1	2
Philippines	Nabua	0.6	0.5	0.7	6	5	8	0.3	0.2	0.4	4	3	5
Philippines	Nabunturan	0.5	0.4	0.7	4	3	5	0.3	0.2	0.4	3	2	3
Philippines	Naga	0.6	0.5	0.8	3	2	4	0.3	0.3	0.4	1	1	2
Philippines	Naga City	0.6	0.5	0.7	6	5	8	0.3	0.3	0.4	5	4	6
Philippines	Naga City	0.6	0.4	0.8	7	6	9	0.3	0.3	0.4	5	4	7
Philippines	Nagbukel	0.6	0.4	0.8	0	0	0	0.3	0.2	0.4	0	0	0
Philippines	Nagcarlan	0.5	0.4	0.6	3	3	4	0.3	0.2	0.3	2	2	3
Philippines	Nagtipunan	0.5	0.4	0.6	1	1	2	0.3	0.2	0.4	1	1	1
Philippines	Naguilian	0.6	0.4	0.7	2	2	3	0.3	0.2	0.4	1	1	2
Philippines	Naguilian	0.6	0.5	0.8	2	1	2	0.3	0.2	0.4	1	1	1
Philippines	Naic	0.6	0.5	0.7	7	6	9	0.3	0.2	0.4	4	3	5
Philippines	Nampicuan	0.6	0.4	0.7	1	1	1	0.3	0.2	0.4	1	1	1
Philippines	Narra	0.6	0.4	0.7	5	4	6	0.3	0.2	0.4	3	2	4
Philippines	Narvacan	0.6	0.4	0.7	2	2	3	0.3	0.2	0.4	1	1	1
Philippines	Nasipit	0.6	0.4	0.7	3	2	4	0.3	0.2	0.4	2	2	3
Philippines	Nasugbu	0.6	0.4	0.7	7	5	8	0.3	0.2	0.4	4	3	5
Philippines	Natividad	0.6	0.4	0.7	1	1	2	0.3	0.2	0.4	1	1	1
Philippines	Natonin	0.6	0.5	0.8	1	1	1	0.3	0.3	0.4	1	1	1
Philippines	Naujan	0.5	0.4	0.7	6	5	8	0.3	0.2	0.4	4	3	5
Philippines	Naujan Lake	0.5	0.4	0.7	0	0	1	0.3	0.2	0.4	0	0	0
Philippines	Naval	0.5	0.4	0.7	3	2	4	0.3	0.2	0.4	2	2	3
Philippines	Navotas	0.5	0.4	0.7	9	8	12	0.3	0.2	0.4	5	4	6
Philippines	New Bataan	0.5	0.4	0.7	3	2	4	0.3	0.2	0.4	2	2	3
Philippines	New Corella	0.5	0.4	0.7	3	2	4	0.3	0.2	0.4	2	2	3
Philippines	New Lucena	0.6	0.5	0.8	1	1	2	0.3	0.2	0.4	1	1	1
Philippines	New Washington	0.6	0.4	0.7	3	2	4	0.3	0.2	0.4	2	1	2
Philippines	Norala	0.7	0.5	0.9	4	3	5	0.4	0.3	0.5	2	2	3
Philippines	Northern Kabuntalan	0.7	0.5	0.9	5	4	6	0.3	0.3	0.4	1	1	1
Philippines	Norzagaray	0.5	0.4	0.6	13	11	16	0.3	0.2	0.4	6	5	8
Philippines	Noveleta	0.7	0.5	0.8	3	2	4	0.3	0.3	0.4	2	1	2
Philippines	Nueva Era	0.6	0.4	0.8	1	1	1	0.3	0.2	0.4	1	0	1

Country	Unit	2000 mortality mean rate (per 10,000)	lower rate (per 10,000)	upper rate (per 10,000)	mean count	lower count	upper count	2017 mortality mean rate (per 10,000)	lower rate (per 10,000)	upper rate (per 10,000)	mean count	lower count	upper count
Philippines	Nueva Valencia	0.6	0.4	0.7	2	1	2	0.3	0.2	0.4	1	1	1
Philippines	Numancia	0.6	0.5	0.8	2	1	2	0.4	0.3	0.5	1	1	2
Philippines	Nunungan	0.6	0.5	0.8	2	2	3	0.3	0.2	0.3	1	1	2
Philippines	Oas	0.6	0.5	0.7	6	5	7	0.3	0.2	0.4	3	3	4
Philippines	Obando	0.6	0.5	0.8	14	11	17	0.3	0.3	0.4	6	5	8
Philippines	Ocampo	0.5	0.4	0.7	3	2	3	0.3	0.2	0.4	2	2	3
Philippines	Odiongan	0.6	0.4	0.7	3	2	4	0.3	0.2	0.4	2	1	2
Philippines	Old Panamao	0.6	0.5	0.8	3	2	3	0.3	0.2	0.3	0	0	0
Philippines	Olongapo City	0.6	0.5	0.8	13	11	17	0.3	0.3	0.4	6	5	7
Philippines	Olutanga	0.6	0.5	0.8	2	2	3	0.3	0.3	0.4	1	1	2
Philippines	Opol	0.6	0.4	0.7	3	3	4	0.3	0.2	0.4	3	2	3
Philippines	Orani	0.6	0.5	0.7	2	2	3	0.3	0.2	0.4	1	1	1
Philippines	Oras	0.6	0.4	0.7	2	2	3	0.3	0.3	0.4	2	2	2
Philippines	Orion	0.5	0.4	0.7	4	3	5	0.3	0.2	0.4	2	2	3
Philippines	Ormoc City	0.6	0.4	0.7	12	10	16	0.3	0.2	0.4	9	7	11
Philippines	Oroquieta City	0.6	0.5	0.8	4	3	5	0.3	0.2	0.4	2	2	3
Philippines	Oslob	0.5	0.4	0.7	1	1	1	0.3	0.2	0.4	1	1	1
Philippines	Oton	0.6	0.5	0.8	5	4	6	0.3	0.2	0.4	3	2	3
Philippines	Ozamis City	0.6	0.4	0.7	5	4	7	0.3	0.2	0.4	3	3	4
Philippines	Padada	0.6	0.5	0.8	2	1	2	0.3	0.3	0.4	1	1	1
Philippines	Padre Burgos	0.6	0.5	0.8	1	0	1	0.3	0.2	0.4	0	0	0
Philippines	Padre Burgos	0.5	0.4	0.6	1	1	1	0.3	0.2	0.3	0	0	1
Philippines	Padre Garcia	0.5	0.4	0.7	2	2	3	0.3	0.2	0.3	2	1	2
Philippines	Paete	0.5	0.4	0.6	2	1	2	0.3	0.2	0.3	1	1	1
Philippines	Pagadian City	0.6	0.5	0.8	10	8	13	0.3	0.3	0.4	10	8	13
Philippines	Pagagawan	0.7	0.5	0.9	3	2	4	0.4	0.3	0.4	1	1	2
Philippines	Pagalungan	0.7	0.6	0.9	4	3	5	0.3	0.3	0.4	2	2	3
Philippines	Pagayawan	0.6	0.4	0.7	2	1	2	0.2	0.2	0.3	0	0	0
Philippines	Pagbilao	0.5	0.4	0.6	4	3	5	0.3	0.2	0.3	3	2	3
Philippines	Paglat	0.7	0.6	0.9	1	1	2	0.4	0.3	0.5	0	0	0
Philippines	Pagsanghan	0.6	0.4	0.7	1	0	1	0.3	0.3	0.4	0	0	0
Philippines	Pagsanjan	0.5	0.4	0.6	2	1	2	0.3	0.2	0.3	1	1	2
Philippines	Pagudpud	0.6	0.4	0.7	1	1	1	0.3	0.2	0.4	1	1	1
Philippines	Pakil	0.5	0.4	0.6	1	1	1	0.3	0.2	0.3	0	0	1
Philippines	Palakpakin Lake	0.5	0.4	0.7	0	0	0	0.3	0.2	0.3	0	0	0
Philippines	Palanan	0.6	0.4	0.7	1	1	2	0.3	0.3	0.4	1	1	1
Philippines	Palanas	0.6	0.5	0.8	2	2	3	0.3	0.3	0.4	1	1	2
Philippines	Palapag	0.6	0.5	0.8	3	2	3	0.4	0.3	0.5	2	1	2
Philippines	Palauig	0.5	0.4	0.7	2	1	2	0.3	0.2	0.4	1	1	2
Philippines	Palayan City	0.5	0.4	0.6	2	1	2	0.3	0.2	0.4	2	1	2
Philippines	Palimbang	0.7	0.5	0.8	6	4	7	0.4	0.3	0.5	4	3	5
Philippines	Palo	0.6	0.5	0.8	3	3	4	0.3	0.2	0.4	3	2	3
Philippines	Palompon	0.6	0.4	0.7	2	2	3	0.3	0.2	0.4	1	1	2
Philippines	Paluan	0.5	0.4	0.6	1	1	1	0.3	0.2	0.4	1	1	1
Philippines	Pambujan	0.6	0.5	0.8	3	2	3	0.4	0.3	0.5	2	2	3
Philippines	Pamplona	0.5	0.4	0.7	3	2	3	0.3	0.2	0.4	1	1	2
Philippines	Pamplona	0.6	0.5	0.8	2	1	3	0.3	0.3	0.4	1	1	1
Philippines	Pamplona	0.5	0.4	0.7	3	2	3	0.3	0.2	0.4	2	1	2
Philippines	Panabo City	0.6	0.5	0.7	8	6	9	0.3	0.3	0.4	7	5	9
Philippines	Panaon	0.6	0.4	0.7	1	1	1	0.3	0.2	0.4	1	0	1
Philippines	Panay	0.5	0.4	0.7	3	3	4	0.3	0.2	0.4	2	1	2
Philippines	Pandag	0.7	0.6	0.9	2	1	2	0.4	0.3	0.5	1	1	2
Philippines	Pandami	0.7	0.5	0.8	0	0	0	0.3	0.2	0.4	0	0	0
Philippines	Pandan	0.7	0.5	0.8	2	1	2	0.4	0.3	0.5	1	1	2
Philippines	Pandan	0.7	0.5	0.8	2	1	2	0.4	0.3	0.5	1	1	1
Philippines	Pandi	0.5	0.4	0.6	4	3	5	0.3	0.2	0.3	3	2	4
Philippines	Panganiban	0.6	0.5	0.8	1	1	1	0.4	0.3	0.5	0	0	1
Philippines	Pangantucan	0.6	0.4	0.7	4	3	5	0.3	0.2	0.4	2	2	3
Philippines	Pangil	0.5	0.4	0.6	1	1	2	0.3	0.2	0.3	1	1	1
Philippines	Panglao	0.6	0.4	0.7	1	1	1	0.3	0.2	0.4	1	1	1

Table 3: Diarrhoea Mortality by Unit *(continued)*

Country	Unit	2000 mortality mean rate (per 10,000)	lower rate (per 10,000)	upper rate (per 10,000)	mean count	lower count	upper count	2017 mortality mean rate (per 10,000)	lower rate (per 10,000)	upper rate (per 10,000)	mean count	lower count	upper count
Philippines	Panglima Estino	0.6	0.5	0.8	2	1	2	0.3	0.2	0.3	0	0	0
Philippines	Panglima Sugala	0.6	0.5	0.8	3	2	4	0.3	0.2	0.4	0	0	0
Philippines	Pangutaran	0.6	0.5	0.8	1	1	2	0.3	0.2	0.4	1	0	1
Philippines	Paniqui	0.6	0.4	0.7	5	4	7	0.3	0.2	0.4	3	2	4
Philippines	Panitan	0.5	0.4	0.7	3	2	4	0.3	0.2	0.4	1	1	2
Philippines	Pantabangan	0.5	0.4	0.6	2	1	2	0.3	0.2	0.4	1	1	2
Philippines	Pantao Ragat	0.6	0.4	0.7	1	1	2	0.3	0.2	0.4	1	1	1
Philippines	Pantar	0.6	0.4	0.7	2	2	3	0.3	0.2	0.3	1	1	2
Philippines	Pantukan	0.6	0.4	0.7	4	3	5	0.3	0.2	0.4	4	3	5
Philippines	Panukulan	0.5	0.4	0.6	1	1	1	0.3	0.2	0.3	1	0	1
Philippines	Paoay	0.6	0.4	0.8	2	1	2	0.3	0.2	0.4	1	1	1
Philippines	Paoay Lake	0.6	0.4	0.8	0	0	0	0.3	0.2	0.4	0	0	0
Philippines	Paombong	0.6	0.4	0.7	4	3	6	0.3	0.2	0.4	2	2	3
Philippines	Paracale	0.6	0.5	0.8	4	3	5	0.4	0.3	0.5	4	3	5
Philippines	Paracelis	0.6	0.5	0.8	2	2	3	0.3	0.3	0.4	2	1	2
Philippines	Parañaque	0.5	0.4	0.7	41	32	52	0.3	0.2	0.4	24	18	30
Philippines	Paranas	0.6	0.4	0.7	2	2	3	0.3	0.3	0.4	2	1	2
Philippines	Parang	0.6	0.5	0.7	4	3	5	0.3	0.2	0.4	1	1	1
Philippines	Parang	0.6	0.5	0.8	21	16	27	0.3	0.2	0.4	2	1	2
Philippines	Pasacao	0.6	0.4	0.7	3	2	4	0.3	0.2	0.4	3	2	3
Philippines	Pasay City	0.5	0.4	0.6	26	21	32	0.3	0.2	0.3	11	9	14
Philippines	Pasig City	0.5	0.4	0.6	37	29	46	0.3	0.2	0.3	20	16	25
Philippines	Pasil	0.7	0.5	0.8	1	1	1	0.4	0.3	0.4	0	0	0
Philippines	Passi City	0.6	0.4	0.7	5	4	7	0.3	0.2	0.4	3	2	4
Philippines	Pastrana	0.6	0.4	0.7	2	1	2	0.3	0.2	0.4	1	1	1
Philippines	Pasuquin	0.6	0.4	0.7	2	1	2	0.3	0.2	0.4	1	1	1
Philippines	Pata	0.6	0.5	0.8	2	1	2	0.3	0.2	0.3	0	0	0
Philippines	Pateros	0.5	0.4	0.6	3	2	4	0.3	0.2	0.3	1	1	2
Philippines	Patikul	0.7	0.5	0.8	16	13	20	0.3	0.3	0.4	2	2	3
Philippines	Patnanungan	0.5	0.4	0.7	1	1	1	0.3	0.2	0.4	1	0	1
Philippines	Patnongon	0.6	0.4	0.7	2	2	3	0.3	0.2	0.4	1	1	2
Philippines	Pavia	0.7	0.5	0.9	3	2	4	0.3	0.3	0.4	2	1	2
Philippines	Payao	0.6	0.5	0.8	3	2	3	0.3	0.3	0.4	2	1	2
Philippines	Peñablanca	0.6	0.5	0.8	3	2	4	0.3	0.3	0.4	2	2	2
Philippines	Peñaranda	0.5	0.4	0.7	2	1	2	0.3	0.2	0.4	1	1	1
Philippines	Peñarrubia	0.6	0.5	0.8	1	0	1	0.3	0.3	0.4	0	0	0
Philippines	Perez	0.5	0.4	0.6	1	0	1	0.3	0.2	0.3	0	0	1
Philippines	Piagapo	0.6	0.4	0.8	1	1	1	0.3	0.2	0.4	0	0	0
Philippines	Piat	0.7	0.5	0.9	2	1	2	0.4	0.3	0.4	1	1	1
Philippines	Picong	0.6	0.5	0.8	2	2	2	0.3	0.2	0.4	0	0	0
Philippines	Piddig	0.6	0.4	0.7	1	1	1	0.3	0.2	0.4	1	0	1
Philippines	Pidigan	0.6	0.5	0.8	1	1	1	0.3	0.3	0.4	0	0	1
Philippines	Pigkawayan	0.7	0.5	0.8	5	4	7	0.3	0.3	0.4	2	2	3
Philippines	Pikit	0.7	0.5	0.9	8	6	10	0.3	0.3	0.4	7	5	8
Philippines	Pila	0.5	0.4	0.7	2	2	3	0.3	0.2	0.3	1	1	2
Philippines	Pilar	0.5	0.4	0.7	2	1	2	0.3	0.2	0.4	1	1	1
Philippines	Pilar	0.5	0.4	0.7	3	2	4	0.3	0.2	0.4	2	1	2
Philippines	Pilar	0.6	0.4	0.7	5	4	6	0.3	0.3	0.4	3	3	4
Philippines	Pilar	0.7	0.6	0.9	1	0	1	0.4	0.3	0.5	1	1	1
Philippines	Pilar	0.6	0.5	0.8	1	1	1	0.3	0.3	0.4	0	0	0
Philippines	Pilar	0.5	0.4	0.7	2	2	3	0.3	0.2	0.4	1	1	1
Philippines	Pilar	0.6	0.4	0.7	0	0	0	0.3	0.3	0.4	0	0	0
Philippines	Pili	0.5	0.4	0.7	6	5	8	0.3	0.2	0.4	4	3	5
Philippines	Pililla	0.5	0.4	0.6	3	2	4	0.3	0.2	0.3	2	1	2
Philippines	Pinabacdao	0.5	0.4	0.7	1	1	1	0.3	0.2	0.4	1	1	1
Philippines	Pinamalayan	0.5	0.4	0.7	5	4	6	0.3	0.2	0.4	3	2	4
Philippines	Pinamungahan	0.6	0.4	0.7	3	3	4	0.3	0.3	0.4	2	2	3
Philippines	Pinan	0.6	0.5	0.8	1	1	2	0.3	0.2	0.4	1	1	1
Philippines	Pinili	0.6	0.4	0.8	1	1	1	0.3	0.2	0.4	1	0	1
Philippines	Pintuyan	0.6	0.5	0.8	1	1	1	0.3	0.3	0.4	0	0	0

Table 3: Diarrhoea Mortality by Unit *(continued)*

Country	Unit	2000 mortality mean rate (per 10,000)	lower rate (per 10,000)	upper rate (per 10,000)	mean count	lower count	upper count	2017 mortality mean rate (per 10,000)	lower rate (per 10,000)	upper rate (per 10,000)	mean count	lower count	upper count
Philippines	Pinukpuk	0.7	0.5	0.8	3	2	4	0.3	0.3	0.4	2	2	2
Philippines	Pio Duran	0.6	0.5	0.7	2	2	3	0.3	0.3	0.4	1	1	1
Philippines	Pio V. Corpuz	0.6	0.4	0.8	1	1	1	0.3	0.3	0.4	1	1	1
Philippines	Pitogo	0.6	0.5	0.8	1	1	1	0.3	0.3	0.4	1	0	1
Philippines	Pitogo	0.5	0.4	0.6	1	1	2	0.3	0.2	0.3	1	1	1
Philippines	Placer	0.7	0.5	0.8	2	2	2	0.4	0.3	0.5	1	1	2
Philippines	Placer	0.6	0.4	0.8	3	2	3	0.3	0.3	0.4	2	2	3
Philippines	Plaridel	0.6	0.4	0.7	2	1	3	0.3	0.2	0.4	1	1	2
Philippines	Plaridel	0.5	0.4	0.7	7	6	9	0.3	0.2	0.4	4	3	5
Philippines	Plaridel	0.5	0.4	0.6	0	0	1	0.3	0.2	0.3	0	0	0
Philippines	Pola	0.5	0.4	0.7	2	2	3	0.3	0.2	0.4	1	1	2
Philippines	Polanco	0.6	0.5	0.8	2	2	3	0.3	0.2	0.4	1	1	2
Philippines	Polangui	0.6	0.5	0.7	5	4	7	0.3	0.2	0.4	3	3	4
Philippines	Polillo	0.5	0.4	0.6	2	1	2	0.3	0.2	0.4	1	1	1
Philippines	Polomolok	0.7	0.6	0.9	13	10	16	0.4	0.3	0.5	8	6	10
Philippines	Pontevedra	0.5	0.4	0.7	2	2	3	0.3	0.2	0.4	1	1	2
Philippines	Pontevedra	0.6	0.4	0.7	2	2	3	0.3	0.2	0.4	1	1	1
Philippines	Poona Bayabao	0.6	0.5	0.8	4	3	5	0.3	0.2	0.3	0	0	1
Philippines	Poona Piagapo	0.6	0.4	0.7	1	1	2	0.3	0.2	0.4	1	1	2
Philippines	Porac	0.5	0.4	0.7	10	8	13	0.3	0.2	0.4	6	5	8
Philippines	Poro	0.6	0.5	0.8	1	1	2	0.4	0.3	0.4	1	1	1
Philippines	Pototan	0.6	0.5	0.8	4	3	6	0.3	0.3	0.4	3	2	3
Philippines	Pozzorubio	0.6	0.5	0.8	4	3	5	0.3	0.3	0.4	2	2	3
Philippines	Pres. Carlos P. Garcia	0.5	0.4	0.7	2	1	2	0.3	0.2	0.4	1	0	1
Philippines	Pres. Manuel A. Roxas	0.6	0.5	0.8	3	2	4	0.3	0.2	0.4	2	1	2
Philippines	Presentacion	0.6	0.4	0.7	1	1	1	0.3	0.3	0.4	1	0	1
Philippines	President Quirino	0.7	0.6	0.9	3	2	3	0.4	0.3	0.5	2	2	3
Philippines	President Roxas	0.5	0.4	0.7	2	1	2	0.3	0.2	0.4	1	1	1
Philippines	President Roxas	0.6	0.5	0.8	4	3	5	0.3	0.3	0.4	2	2	3
Philippines	Prieto Diaz	0.6	0.5	0.7	1	1	2	0.3	0.3	0.4	1	1	1
Philippines	Prosperidad	0.6	0.5	0.8	7	5	8	0.3	0.3	0.4	3	3	4
Philippines	Pualas	0.6	0.5	0.8	1	1	2	0.3	0.2	0.3	0	0	0
Philippines	Pudtol	0.6	0.5	0.8	1	1	1	0.3	0.3	0.4	1	1	1
Philippines	Puerto Galera	0.5	0.4	0.7	1	1	2	0.3	0.2	0.3	2	1	2
Philippines	Puerto Princesa City	0.6	0.5	0.7	13	10	16	0.3	0.2	0.4	8	6	11
Philippines	Pugo	0.6	0.5	0.8	1	1	2	0.3	0.3	0.4	1	0	1
Philippines	Pulilan	0.5	0.4	0.7	5	4	7	0.3	0.2	0.4	3	2	3
Philippines	Pulupandan	0.6	0.4	0.8	3	2	3	0.3	0.2	0.4	1	1	1
Philippines	Pura	0.6	0.4	0.7	1	1	1	0.3	0.2	0.4	1	1	1
Philippines	Quezon	0.6	0.4	0.7	1	1	2	0.3	0.2	0.4	1	1	1
Philippines	Quezon	0.5	0.4	0.7	8	6	10	0.3	0.2	0.4	5	4	6
Philippines	Quezon	0.6	0.4	0.7	4	3	5	0.3	0.2	0.4	3	2	4
Philippines	Quezon	0.7	0.5	0.8	2	1	2	0.4	0.3	0.5	1	1	2
Philippines	Quezon	0.5	0.4	0.7	2	2	3	0.3	0.2	0.4	2	1	2
Philippines	Quezon	0.5	0.4	0.6	0	0	0	0.3	0.2	0.3	0	0	0
Philippines	Quezon City	0.5	0.4	0.6	174	139	214	0.3	0.2	0.3	96	77	120
Philippines	Quinapondan	0.6	0.5	0.8	1	1	1	0.3	0.3	0.4	1	1	1
Philippines	Quirino	0.6	0.4	0.7	0	0	1	0.3	0.2	0.4	0	0	0
Philippines	Quirino	0.7	0.5	0.8	2	1	2	0.4	0.3	0.5	1	1	1
Philippines	Ragay	0.6	0.4	0.8	3	2	4	0.3	0.3	0.4	2	2	3
Philippines	Rajah Buayan	0.7	0.6	0.9	4	3	5	0.3	0.3	0.4	1	0	1
Philippines	Ramon	0.6	0.5	0.8	3	2	3	0.3	0.3	0.4	2	2	3
Philippines	Ramon Magsaysay	0.6	0.5	0.8	2	2	3	0.3	0.2	0.4	1	1	1
Philippines	Ramos	0.6	0.4	0.7	1	1	1	0.3	0.2	0.4	1	0	1
Philippines	Rapu-Rapu	0.6	0.5	0.7	2	1	2	0.3	0.3	0.4	1	1	2
Philippines	Real	0.4	0.4	0.6	2	2	3	0.3	0.2	0.3	2	1	2

Table 3: Diarrhoea Mortality by Unit *(continued)*

Country	Unit	2000 mortality mean rate (per 10,000)	lower rate (per 10,000)	upper rate (per 10,000)	mean count	lower count	upper count	2017 mortality mean rate (per 10,000)	lower rate (per 10,000)	upper rate (per 10,000)	mean count	lower count	upper count
Philippines	Reina Mercedes	0.6	0.5	0.8	1	1	2	0.3	0.3	0.4	1	1	1
Philippines	Remedios T. Romualdez	0.6	0.5	0.8	1	1	1	0.3	0.3	0.4	1	0	1
Philippines	Rizal	0.6	0.5	0.8	1	1	1	0.3	0.2	0.4	0	0	1
Philippines	Rizal	0.6	0.4	0.7	2	2	3	0.3	0.2	0.4	3	2	4
Philippines	Rizal	0.7	0.5	0.8	1	1	2	0.4	0.3	0.4	1	1	1
Philippines	Rizal	0.5	0.4	0.6	3	2	4	0.3	0.2	0.4	2	2	3
Philippines	Rizal	0.5	0.4	0.6	1	1	2	0.3	0.2	0.3	1	1	1
Philippines	Rizal	0.7	0.5	0.9	1	1	2	0.4	0.3	0.4	1	1	1
Philippines	Rizal	0.6	0.4	0.7	3	2	4	0.3	0.3	0.4	2	2	3
Philippines	Rodriguez	0.6	0.5	0.7	11	9	13	0.3	0.3	0.4	17	13	21
Philippines	Romblon	0.5	0.4	0.7	2	2	3	0.3	0.2	0.4	1	1	2
Philippines	Ronda	0.5	0.4	0.7	1	1	1	0.3	0.2	0.4	1	1	1
Philippines	Rosales	0.6	0.5	0.7	3	3	4	0.3	0.3	0.4	2	2	2
Philippines	Rosario	0.6	0.5	0.8	3	3	4	0.3	0.3	0.4	2	2	2
Philippines	Rosario	0.6	0.4	0.7	1	1	1	0.3	0.2	0.4	1	0	1
Philippines	Rosario	0.7	0.5	0.8	4	3	5	0.3	0.3	0.4	2	2	3
Philippines	Rosario	0.5	0.4	0.7	6	5	8	0.3	0.2	0.3	4	3	6
Philippines	Rosario	0.7	0.5	0.8	2	2	3	0.4	0.3	0.5	3	2	3
Philippines	Roseller Lim	0.6	0.5	0.8	3	2	4	0.3	0.3	0.4	2	1	2
Philippines	Roxas	0.5	0.4	0.7	3	2	3	0.3	0.3	0.4	2	2	3
Philippines	Roxas	0.6	0.4	0.7	2	2	3	0.3	0.2	0.4	3	2	3
Philippines	Roxas	0.7	0.5	0.8	4	3	5	0.4	0.3	0.5	3	2	3
Philippines	Roxas City	0.5	0.4	0.7	9	7	11	0.3	0.2	0.4	6	4	7
Philippines	Sabangan	0.7	0.5	0.8	1	1	1	0.4	0.3	0.4	0	0	1
Philippines	Sablan	0.6	0.5	0.8	1	1	2	0.3	0.3	0.4	1	1	1
Philippines	Sablayan	0.6	0.4	0.7	6	5	7	0.3	0.2	0.4	4	3	6
Philippines	Sabtang	NA	NA	NA	NA	NA	NA	NA	NA	NA	NA	NA	NA
Philippines	Sadanga	0.7	0.5	0.8	1	1	1	0.4	0.3	0.4	0	0	0
Philippines	Sagada	0.7	0.5	0.8	1	1	1	0.4	0.3	0.4	0	0	1
Philippines	Sagay	0.6	0.4	0.7	1	1	1	0.3	0.2	0.4	0	0	0
Philippines	Sagay City	0.5	0.4	0.7	9	7	12	0.3	0.2	0.4	5	4	6
Philippines	Sagbayan	0.5	0.4	0.7	1	1	2	0.3	0.2	0.4	1	1	1
Philippines	Sagnay	0.5	0.4	0.7	2	2	3	0.3	0.2	0.4	2	1	2
Philippines	Saguday	0.6	0.4	0.7	1	1	1	0.3	0.2	0.4	1	0	1
Philippines	Saguiaran	0.6	0.5	0.8	2	2	3	0.3	0.2	0.4	1	1	2
Philippines	Saint Bernard	0.6	0.5	0.8	1	1	1	0.3	0.3	0.4	0	0	1
Philippines	Salay	0.6	0.4	0.7	0	0	1	0.3	0.2	0.4	1	0	1
Philippines	Salcedo	0.6	0.4	0.7	1	0	1	0.3	0.2	0.3	0	0	0
Philippines	Salcedo	0.6	0.5	0.8	1	1	1	0.3	0.3	0.4	1	1	1
Philippines	Sallapadan	0.6	0.5	0.8	1	0	1	0.3	0.3	0.4	0	0	0
Philippines	Salug	0.6	0.5	0.8	2	2	3	0.3	0.3	0.4	1	1	2
Philippines	Salvador	0.6	0.4	0.7	1	1	1	0.3	0.2	0.4	1	1	1
Philippines	Salvador Benedicto	0.6	0.4	0.7	2	1	2	0.3	0.2	0.4	1	1	1
Philippines	Samal	0.6	0.4	0.7	3	2	3	0.3	0.2	0.4	1	1	2
Philippines	Samal City	0.6	0.5	0.7	5	4	7	0.3	0.3	0.4	4	3	4
Philippines	Samboan	0.5	0.4	0.7	1	1	2	0.3	0.2	0.4	1	1	1
Philippines	Sampaloc	0.4	0.3	0.6	1	1	1	0.3	0.2	0.3	0	0	0
Philippines	Sampaloc Lake	0.5	0.4	0.7	0	0	0	0.3	0.2	0.4	0	0	0
Philippines	San Agustin	0.5	0.4	0.7	2	1	2	0.3	0.2	0.4	1	1	1
Philippines	San Agustin	0.7	0.5	0.9	1	1	2	0.4	0.3	0.5	1	1	1
Philippines	San Agustin	0.5	0.4	0.6	1	1	2	0.3	0.2	0.4	1	1	1
Philippines	San Andres	0.5	0.4	0.7	1	1	1	0.3	0.2	0.4	1	1	1
Philippines	San Andres	0.5	0.4	0.6	1	1	1	0.3	0.2	0.3	1	1	1
Philippines	San Andres	0.6	0.4	0.7	3	2	3	0.3	0.3	0.4	2	1	2
Philippines	San Antonio	0.5	0.4	0.7	0	0	0	0.3	0.2	0.4	0	0	0
Philippines	San Antonio	0.5	0.4	0.7	5	4	6	0.3	0.2	0.4	3	3	4
Philippines	San Antonio	0.5	0.4	0.6	2	2	3	0.3	0.2	0.3	1	1	2
Philippines	San Antonio	0.5	0.4	0.7	2	1	2	0.3	0.2	0.4	1	1	1

Table 3: Diarrhoea Mortality by Unit *(continued)*

Country	Unit	2000 mortality mean rate (per 10,000)	lower rate (per 10,000)	upper rate (per 10,000)	mean count	lower count	upper count	2017 mortality mean rate (per 10,000)	lower rate (per 10,000)	upper rate (per 10,000)	mean count	lower count	upper count
Philippines	San Benito	0.7	0.6	0.9	1	0	1	0.4	0.3	0.5	0	0	0
Philippines	San Carlos City	0.6	0.4	0.7	9	7	11	0.3	0.2	0.4	4	3	5
Philippines	San Carlos City	0.6	0.5	0.7	12	9	15	0.3	0.2	0.4	8	6	10
Philippines	San Clemente	0.5	0.4	0.7	1	1	2	0.3	0.2	0.4	1	0	1
Philippines	San Dionisio	0.5	0.4	0.7	2	1	2	0.3	0.2	0.4	1	1	1
Philippines	San Emilio	0.6	0.4	0.7	0	0	0	0.3	0.2	0.4	0	0	0
Philippines	San Enrique	0.6	0.4	0.7	1	1	1	0.3	0.2	0.4	0	0	0
Philippines	San Enrique	0.6	0.4	0.8	2	1	2	0.3	0.2	0.4	1	1	1
Philippines	San Esteban	0.5	0.4	0.7	1	0	1	0.3	0.2	0.3	0	0	0
Philippines	San Fabian	0.6	0.5	0.8	4	3	5	0.3	0.3	0.4	2	2	3
Philippines	San Felipe	0.5	0.4	0.7	1	1	2	0.3	0.2	0.4	1	1	1
Philippines	San Fernando	0.5	0.4	0.6	2	1	2	0.3	0.2	0.4	1	1	1
Philippines	San Fernando	0.6	0.5	0.8	1	1	1	0.3	0.3	0.4	1	1	1
Philippines	San Fernando	0.5	0.4	0.7	3	2	4	0.3	0.2	0.4	3	2	3
Philippines	San Fernando	0.5	0.4	0.7	2	1	2	0.3	0.2	0.4	1	1	2
Philippines	San Fernando	0.6	0.4	0.7	4	3	5	0.3	0.3	0.4	3	3	4
Philippines	San Fernando City	0.6	0.4	0.7	8	6	10	0.3	0.2	0.4	4	3	4
Philippines	San Fernando City	0.6	0.4	0.7	13	10	17	0.3	0.2	0.4	8	6	10
Philippines	San Francisco	0.6	0.5	0.8	1	1	1	0.3	0.3	0.4	0	0	1
Philippines	San Francisco	0.7	0.5	0.8	1	1	1	0.4	0.3	0.5	1	1	1
Philippines	San Francisco	0.5	0.4	0.6	4	3	5	0.3	0.2	0.4	3	2	4
Philippines	San Francisco	0.6	0.5	0.8	3	2	4	0.4	0.3	0.4	2	2	2
Philippines	San Francisco	0.7	0.5	0.8	6	4	7	0.4	0.3	0.4	5	4	6
Philippines	San Gabriel	0.5	0.4	0.7	1	1	1	0.3	0.2	0.4	1	0	1
Philippines	San Guillermo	0.6	0.4	0.7	1	1	2	0.3	0.3	0.4	1	1	1
Philippines	San Ildefonso	0.6	0.4	0.8	1	0	1	0.3	0.2	0.4	0	0	0
Philippines	San Ildefonso	0.5	0.4	0.6	6	5	7	0.3	0.2	0.3	3	2	4
Philippines	San Isidro	0.6	0.5	0.7	2	1	2	0.3	0.3	0.4	1	1	1
Philippines	San Isidro	0.7	0.5	0.9	1	0	1	0.4	0.3	0.5	0	0	1
Philippines	San Isidro	0.6	0.4	0.7	2	1	2	0.3	0.2	0.4	1	1	2
Philippines	San Isidro	0.6	0.5	0.8	0	0	0	0.3	0.3	0.4	0	0	0
Philippines	San Isidro	0.5	0.4	0.7	3	2	3	0.3	0.2	0.4	2	1	2
Philippines	San Isidro	0.6	0.5	0.8	1	1	2	0.3	0.3	0.4	1	1	1
Philippines	San Isidro	0.5	0.4	0.7	2	2	3	0.3	0.2	0.4	1	1	1
Philippines	San Isidro	0.5	0.4	0.7	1	1	1	0.3	0.2	0.4	0	0	0
Philippines	San Isidro	0.5	0.4	0.7	2	1	2	0.3	0.2	0.4	1	1	1
Philippines	San Jacinto	0.6	0.5	0.8	2	2	3	0.3	0.3	0.4	1	1	2
Philippines	San Jacinto	0.6	0.5	0.8	3	2	3	0.3	0.3	0.4	2	1	2
Philippines	San Joaquin	0.6	0.4	0.7	3	2	3	0.3	0.2	0.4	1	1	2
Philippines	San Jorge	0.6	0.4	0.7	1	1	1	0.3	0.3	0.4	1	1	1
Philippines	San Jose	0.6	0.4	0.7	6	5	8	0.3	0.3	0.4	6	4	7
Philippines	San Jose	0.6	0.4	0.7	2	1	2	0.3	0.2	0.4	1	1	1
Philippines	San Jose	0.5	0.4	0.7	3	2	4	0.3	0.2	0.4	2	1	2
Philippines	San Jose	0.6	0.5	0.8	0	0	1	0.4	0.3	0.4	0	0	0
Philippines	San Jose	0.6	0.4	0.7	1	1	1	0.3	0.2	0.4	1	1	1
Philippines	San Jose	0.5	0.4	0.7	1	1	2	0.3	0.2	0.4	1	1	1
Philippines	San Jose	0.5	0.4	0.6	4	3	5	0.3	0.2	0.3	3	2	4
Philippines	San Jose	0.5	0.4	0.7	2	2	3	0.3	0.2	0.4	2	1	2
Philippines	San Jose	0.7	0.5	0.9	1	1	2	0.4	0.3	0.5	1	1	1
Philippines	San Jose City	0.6	0.4	0.7	8	6	10	0.3	0.2	0.4	5	4	6
Philippines	San Jose de Buan	0.6	0.5	0.7	1	1	1	0.3	0.3	0.4	0	0	0
Philippines	San Jose del Monte City	0.5	0.4	0.7	38	30	46	0.3	0.2	0.4	24	19	30
Philippines	San Juan	0.5	0.4	0.6	9	7	11	0.3	0.2	0.3	4	3	5
Philippines	San Juan	0.6	0.5	0.8	1	1	1	0.3	0.3	0.4	0	0	0
Philippines	San Juan	0.6	0.4	0.7	2	1	2	0.3	0.2	0.3	1	1	1
Philippines	San Juan	0.6	0.4	0.7	1	1	2	0.3	0.2	0.4	1	1	1
Philippines	San Juan	0.6	0.5	0.8	1	0	1	0.3	0.3	0.4	0	0	0

Table 3: Diarrhoea Mortality by Unit *(continued)*

Country	Unit	2000 mortality mean rate (per 10,000)	lower rate (per 10,000)	upper rate (per 10,000)	mean count	lower count	upper count	2017 mortality mean rate (per 10,000)	lower rate (per 10,000)	upper rate (per 10,000)	mean count	lower count	upper count
Philippines	San Juan	0.5	0.4	0.6	5	4	7	0.3	0.2	0.3	4	3	5
Philippines	San Juan	0.6	0.4	0.7	1	0	1	0.3	0.2	0.4	0	0	0
Philippines	San Julian	0.6	0.5	0.8	1	1	1	0.4	0.3	0.5	0	0	1
Philippines	San Leonardo	0.5	0.4	0.7	3	3	4	0.3	0.2	0.4	2	2	3
Philippines	San Lorenzo	0.6	0.5	0.8	2	1	2	0.3	0.2	0.4	1	1	1
Philippines	San Lorenzo Ruiz	0.6	0.5	0.8	2	1	2	0.4	0.3	0.4	1	1	1
Philippines	San Luis	0.5	0.4	0.7	3	2	4	0.3	0.2	0.3	2	1	2
Philippines	San Luis	0.5	0.4	0.6	2	2	3	0.3	0.2	0.3	1	1	1
Philippines	San Luis	0.5	0.4	0.6	1	1	2	0.3	0.2	0.4	1	1	1
Philippines	San Luis	0.6	0.5	0.8	3	2	4	0.3	0.3	0.4	2	2	3
Philippines	San Manuel	0.6	0.5	0.7	2	1	2	0.3	0.2	0.4	1	1	1
Philippines	San Manuel	0.6	0.5	0.8	2	2	3	0.3	0.3	0.4	1	1	2
Philippines	San Manuel	0.6	0.5	0.7	3	3	4	0.3	0.2	0.4	1	1	2
Philippines	San Marcelino	0.5	0.4	0.7	2	1	2	0.3	0.2	0.4	1	1	1
Philippines	San Mariano	0.6	0.4	0.7	3	2	3	0.3	0.2	0.4	3	2	4
Philippines	San Mateo	0.5	0.4	0.6	16	13	20	0.3	0.2	0.4	11	9	15
Philippines	San Mateo	0.6	0.5	0.8	4	3	4	0.3	0.3	0.4	2	2	3
Philippines	San Miguel	0.7	0.5	0.8	3	2	4	0.4	0.3	0.5	2	2	3
Philippines	San Miguel	0.6	0.5	0.8	2	1	2	0.3	0.2	0.4	1	1	1
Philippines	San Miguel	0.5	0.4	0.7	9	7	11	0.3	0.2	0.4	5	4	6
Philippines	San Miguel	0.6	0.5	0.7	1	1	1	0.3	0.2	0.4	1	1	1
Philippines	San Miguel	0.6	0.5	0.8	1	1	2	0.3	0.2	0.4	1	1	1
Philippines	San Miguel	0.5	0.4	0.7	1	1	2	0.3	0.2	0.4	1	1	1
Philippines	San Miguel	0.6	0.5	0.8	1	1	1	0.4	0.3	0.4	1	1	1
Philippines	San Narciso	0.5	0.4	0.7	1	1	2	0.3	0.2	0.4	1	1	1
Philippines	San Narciso	0.5	0.4	0.6	2	2	3	0.3	0.2	0.3	2	2	3
Philippines	San Nicolas	0.6	0.4	0.8	2	1	3	0.3	0.2	0.4	1	1	1
Philippines	San Nicolas	0.5	0.4	0.7	1	1	1	0.3	0.2	0.4	0	0	0
Philippines	San Nicolas	0.6	0.4	0.7	2	2	3	0.3	0.2	0.4	1	1	1
Philippines	San Pablo	0.6	0.5	0.8	2	1	2	0.3	0.3	0.4	1	1	1
Philippines	San Pablo	0.7	0.5	0.8	2	1	2	0.4	0.3	0.5	1	1	1
Philippines	San Pablo City	0.5	0.4	0.7	14	11	18	0.3	0.2	0.4	9	7	10
Philippines	San Pascual	0.6	0.4	0.7	3	3	4	0.3	0.3	0.4	2	2	3
Philippines	San Pascual	0.5	0.4	0.7	4	3	5	0.3	0.2	0.4	2	2	3
Philippines	San Pedro	0.6	0.5	0.8	15	12	19	0.3	0.3	0.4	9	7	11
Philippines	San Policarpo	0.6	0.5	0.8	1	1	2	0.4	0.3	0.5	1	1	1
Philippines	San Quintin	0.6	0.4	0.8	1	0	1	0.3	0.2	0.4	0	0	0
Philippines	San Quintin	0.6	0.4	0.7	2	2	3	0.3	0.2	0.4	1	1	2
Philippines	San Rafael	0.5	0.4	0.6	6	5	7	0.3	0.2	0.4	3	2	4
Philippines	San Rafael	0.6	0.4	0.7	1	1	1	0.3	0.2	0.4	1	0	1
Philippines	San Remigio	0.6	0.4	0.7	2	2	3	0.3	0.2	0.4	1	1	2
Philippines	San Remigio	0.5	0.4	0.7	3	2	4	0.3	0.2	0.4	2	2	3
Philippines	San Ricardo	0.6	0.5	0.8	0	0	1	0.3	0.3	0.4	0	0	0
Philippines	San Roque	0.6	0.5	0.8	2	2	3	0.4	0.3	0.4	2	2	3
Philippines	San Sebastian	0.6	0.4	0.7	0	0	1	0.3	0.3	0.4	0	0	0
Philippines	San Simon	0.5	0.4	0.6	4	3	5	0.3	0.2	0.3	2	2	3
Philippines	San Teodoro	0.5	0.4	0.7	1	1	2	0.3	0.2	0.4	1	1	1
Philippines	San Vicente	0.6	0.4	0.8	1	0	1	0.3	0.2	0.4	0	0	0
Philippines	San Vicente	0.6	0.5	0.7	1	1	2	0.3	0.2	0.4	2	1	2
Philippines	San Vicente	0.6	0.5	0.8	0	0	0	0.3	0.3	0.4	0	0	0
Philippines	San Vicente	0.6	0.5	0.8	1	1	1	0.4	0.3	0.4	1	0	1
Philippines	Sanchez-Mira	0.6	0.5	0.8	2	1	2	0.3	0.2	0.4	1	1	1
Philippines	Santa	0.6	0.4	0.8	1	1	1	0.3	0.2	0.4	0	0	1
Philippines	Santa Ana	0.5	0.4	0.7	4	3	5	0.3	0.2	0.4	2	2	3
Philippines	Santa Ana	0.6	0.5	0.8	1	1	1	0.3	0.2	0.4	1	1	2
Philippines	Santa Barbara	0.6	0.5	0.7	6	5	8	0.3	0.3	0.4	4	3	5
Philippines	Santa Barbara	0.6	0.5	0.8	4	3	5	0.3	0.2	0.4	2	2	3
Philippines	Santa Catalina	0.5	0.4	0.7	5	4	6	0.3	0.2	0.4	2	2	3
Philippines	Santa Catalina	0.6	0.4	0.8	1	1	1	0.3	0.2	0.4	0	0	0
Philippines	Santa Cruz	0.6	0.5	0.7	3	3	4	0.3	0.3	0.4	2	2	3

Table 3: Diarrhoea Mortality by Unit *(continued)*

Country	Unit	2000 mortality mean rate (per 10,000)	lower rate (per 10,000)	upper rate (per 10,000)	mean count	lower count	upper count	2017 mortality mean rate (per 10,000)	lower rate (per 10,000)	upper rate (per 10,000)	mean count	lower count	upper count
Philippines	Santa Cruz	0.6	0.4	0.7	2	2	3	0.3	0.2	0.4	2	2	2
Philippines	Santa Cruz	0.5	0.4	0.7	4	3	5	0.3	0.2	0.4	2	1	2
Philippines	Santa Cruz	0.5	0.4	0.7	2	2	3	0.3	0.2	0.3	1	1	2
Philippines	Santa Cruz	0.5	0.4	0.7	5	4	6	0.3	0.2	0.4	4	3	4
Philippines	Santa Cruz	0.5	0.4	0.6	4	3	6	0.3	0.2	0.4	1	1	2
Philippines	Santa Elena	0.6	0.5	0.7	3	2	4	0.3	0.3	0.4	2	1	2
Philippines	Santa Fe	0.6	0.4	0.7	1	1	1	0.3	0.2	0.4	1	1	1
Philippines	Santa Fe	0.6	0.4	0.7	1	1	1	0.3	0.2	0.4	0	0	1
Philippines	Santa Fe	0.6	0.5	0.7	2	2	3	0.3	0.2	0.4	2	1	2
Philippines	Santa Fe	0.5	0.4	0.7	1	0	1	0.3	0.2	0.4	0	0	0
Philippines	Santa Ignacia	0.5	0.4	0.7	3	2	4	0.3	0.2	0.4	2	1	2
Philippines	Santa Josefa	0.6	0.4	0.8	3	2	4	0.3	0.2	0.4	2	1	2
Philippines	Santa Lucia	0.5	0.4	0.7	2	1	2	0.3	0.2	0.3	1	1	1
Philippines	Santa Magdalena	0.6	0.4	0.7	1	1	1	0.3	0.3	0.4	1	1	1
Philippines	Santa Marcela	0.6	0.5	0.8	1	1	1	0.3	0.3	0.4	0	0	1
Philippines	Santa Margarita	0.6	0.4	0.7	1	1	2	0.3	0.3	0.4	1	1	1
Philippines	Santa Maria	0.5	0.4	0.7	0	0	0	0.3	0.2	0.4	0	0	0
Philippines	Santa Maria	0.5	0.4	0.7	20	16	25	0.3	0.2	0.4	13	10	16
Philippines	Santa Maria	0.6	0.4	0.7	2	1	2	0.3	0.2	0.4	1	1	1
Philippines	Santa Maria	0.5	0.4	0.6	2	2	3	0.3	0.2	0.3	1	1	2
Philippines	Santa Maria	0.7	0.5	0.9	2	2	3	0.4	0.3	0.5	2	1	2
Philippines	Santa Maria	0.6	0.5	0.8	2	2	3	0.3	0.3	0.4	1	1	1
Philippines	Santa Maria	0.6	0.5	0.8	4	3	5	0.3	0.3	0.4	2	2	3
Philippines	Santa Monica	0.7	0.5	0.9	1	0	1	0.4	0.3	0.5	0	0	1
Philippines	Santa Praxedes	0.7	0.5	0.8	0	0	0	0.3	0.3	0.4	0	0	0
Philippines	Santa Rita	0.5	0.4	0.7	2	1	2	0.3	0.2	0.4	1	1	2
Philippines	Santa Rita	0.6	0.4	0.7	3	2	3	0.3	0.2	0.4	2	2	3
Philippines	Santa Rosa	0.5	0.4	0.7	4	3	5	0.3	0.2	0.4	3	2	3
Philippines	Santa Rosa City	0.6	0.5	0.8	18	14	22	0.3	0.3	0.4	13	10	16
Philippines	Santa Teresita	0.6	0.5	0.8	1	1	2	0.3	0.2	0.4	1	1	1
Philippines	Santa Teresita	0.5	0.4	0.6	1	1	1	0.3	0.2	0.3	0	0	0
Philippines	Santander	0.5	0.4	0.7	1	1	1	0.3	0.2	0.4	0	0	1
Philippines	Santiago	0.6	0.5	0.8	1	1	2	0.3	0.3	0.4	1	1	1
Philippines	Santiago	0.5	0.4	0.7	1	1	1	0.3	0.2	0.3	1	1	1
Philippines	Santiago City	0.6	0.5	0.8	10	7	12	0.3	0.3	0.4	6	5	8
Philippines	Santo Domingo	0.5	0.4	0.7	2	2	3	0.3	0.3	0.4	2	1	2
Philippines	Santo Domingo	0.6	0.4	0.8	1	1	2	0.3	0.2	0.4	1	1	1
Philippines	Santo Domingo	0.5	0.4	0.7	4	3	5	0.3	0.2	0.4	2	2	3
Philippines	Santo Nino	0.6	0.4	0.7	0	0	0	0.3	0.3	0.4	0	0	0
Philippines	Santo Nino	0.7	0.5	0.9	3	2	3	0.4	0.3	0.5	2	1	2
Philippines	Santo Niño	0.7	0.5	0.9	3	2	4	0.4	0.3	0.4	1	1	2
Philippines	Santo Tomas	0.6	0.5	0.8	3	2	3	0.3	0.3	0.4	2	1	2
Philippines	Santo Tomas	0.5	0.4	0.7	2	1	2	0.3	0.2	0.4	1	1	1
Philippines	Santo Tomas	0.5	0.4	0.7	6	5	8	0.3	0.2	0.3	5	4	6
Philippines	Santo Tomas	0.7	0.5	0.8	2	2	3	0.4	0.3	0.5	1	1	1
Philippines	Santo Tomas	0.6	0.5	0.7	0	0	1	0.3	0.3	0.4	0	0	0
Philippines	Santo Tomas	0.6	0.4	0.7	5	4	6	0.3	0.2	0.4	5	3	6
Philippines	Santol	0.5	0.4	0.7	1	1	1	0.3	0.2	0.3	0	0	1
Philippines	Sapa-Sapa	0.6	0.5	0.8	1	1	2	0.3	0.2	0.4	0	0	0
Philippines	Sapad	0.6	0.4	0.7	1	1	1	0.3	0.2	0.4	1	1	1
Philippines	Sapang Dalaga	0.6	0.4	0.7	1	1	1	0.3	0.2	0.4	1	1	1
Philippines	Sapi-An	0.6	0.4	0.7	1	1	2	0.3	0.2	0.4	1	1	1
Philippines	Sara	0.5	0.4	0.7	3	2	4	0.3	0.2	0.4	2	1	2
Philippines	Sarangani	0.6	0.5	0.8	1	1	1	0.3	0.3	0.5	1	1	1
Philippines	Sariaya	0.5	0.4	0.6	7	5	9	0.3	0.2	0.3	5	4	6
Philippines	Sarrat	0.6	0.4	0.7	2	1	2	0.3	0.2	0.4	1	1	1
Philippines	Sasmuan	0.6	0.4	0.7	3	2	4	0.3	0.2	0.4	2	1	2
Philippines	Sebaste	0.7	0.5	0.9	1	1	1	0.4	0.3	0.5	1	1	1
Philippines	Sen. Ninoy Aquino	0.7	0.5	0.8	2	1	2	0.4	0.3	0.5	3	2	4

Table 3: Diarrhoea Mortality by Unit *(continued)*

Country	Unit	2000 mortality mean rate (per 10,000)	2000 lower rate (per 10,000)	2000 upper rate (per 10,000)	2000 mean count	2000 lower count	2000 upper count	2017 mortality mean rate (per 10,000)	2017 lower rate (per 10,000)	2017 upper rate (per 10,000)	2017 mean count	2017 lower count	2017 upper count
Philippines	Sergio Osmena Sr.	0.6	0.4	0.8	3	2	4	0.3	0.2	0.4	1	1	2
Philippines	Sevilla	0.5	0.4	0.7	1	1	1	0.3	0.2	0.4	0	0	0
Philippines	Shariff Aguak	0.7	0.6	0.9	5	4	6	0.3	0.3	0.4	0	0	0
Philippines	Siasi	0.7	0.5	0.8	1	1	2	0.3	0.2	0.4	1	0	1
Philippines	Siaton	0.6	0.4	0.7	4	3	6	0.3	0.2	0.4	3	2	4
Philippines	Siay	0.6	0.5	0.8	3	2	3	0.3	0.3	0.4	2	2	3
Philippines	Siayan	0.6	0.5	0.8	4	3	5	0.3	0.2	0.4	2	1	2
Philippines	Sibagat	0.6	0.5	0.8	3	2	4	0.3	0.3	0.4	2	1	2
Philippines	Sibalom	0.6	0.4	0.7	4	3	5	0.3	0.2	0.4	2	2	3
Philippines	Sibonga	0.6	0.4	0.7	3	2	3	0.3	0.2	0.4	2	1	2
Philippines	Sibuco	0.7	0.5	0.8	2	1	2	0.3	0.3	0.4	2	1	2
Philippines	Sibulan	0.6	0.4	0.7	3	2	4	0.3	0.2	0.4	2	2	3
Philippines	Sibunag	0.6	0.4	0.8	1	1	1	0.3	0.2	0.4	1	1	1
Philippines	Sibutad	0.6	0.5	0.8	1	1	2	0.3	0.3	0.4	1	0	1
Philippines	Sibutu	0.6	0.5	0.8	3	2	3	0.3	0.2	0.4	0	0	0
Philippines	Sierra Bullones	0.5	0.4	0.7	2	1	3	0.3	0.2	0.4	1	0	1
Philippines	Sigay	0.6	0.4	0.7	0	0	0	0.3	0.2	0.4	0	0	0
Philippines	Sigma	0.6	0.4	0.7	2	2	3	0.3	0.2	0.4	1	1	2
Philippines	Sikatuna	0.6	0.4	0.7	0	0	0	0.3	0.2	0.4	0	0	0
Philippines	Silago	0.6	0.5	0.7	1	1	1	0.3	0.3	0.4	1	0	1
Philippines	Silang	0.6	0.5	0.7	15	12	19	0.3	0.2	0.4	10	7	11
Philippines	Silay City	0.6	0.4	0.7	8	6	10	0.3	0.2	0.4	4	3	5
Philippines	Silvino Lobos	0.6	0.4	0.7	1	1	2	0.3	0.3	0.4	1	1	1
Philippines	Simunul	0.6	0.5	0.8	2	1	2	0.3	0.2	0.4	0	0	0
Philippines	Sinacaban	0.6	0.4	0.7	1	1	2	0.3	0.2	0.4	1	1	1
Philippines	Sinait	0.6	0.4	0.7	2	1	2	0.3	0.2	0.4	1	1	1
Philippines	Sindangan	0.6	0.5	0.8	5	4	6	0.3	0.2	0.4	3	3	4
Philippines	Siniloan	0.5	0.4	0.6	1	1	1	0.3	0.2	0.3	0	0	0
Philippines	Siocon	0.6	0.5	0.8	4	3	5	0.3	0.3	0.4	2	1	2
Philippines	Sipalay City	0.5	0.4	0.7	5	4	7	0.3	0.2	0.4	3	2	4
Philippines	Sipocot	0.6	0.4	0.7	4	3	5	0.3	0.2	0.4	3	2	4
Philippines	Siquijor	0.6	0.4	0.7	1	1	2	0.3	0.2	0.4	1	1	1
Philippines	Sirawai	0.7	0.5	0.8	1	1	1	0.3	0.3	0.4	2	1	2
Philippines	Siruma	0.6	0.5	0.8	1	1	2	0.3	0.3	0.4	1	1	1
Philippines	Sison	0.7	0.5	0.8	1	1	1	0.4	0.3	0.5	1	0	1
Philippines	Sison	0.6	0.5	0.7	3	3	4	0.3	0.3	0.4	2	1	2
Philippines	Sitangkai	0.6	0.5	0.8	5	4	6	0.3	0.2	0.4	0	0	0
Philippines	Socorro	0.5	0.4	0.7	3	2	4	0.3	0.2	0.4	2	1	2
Philippines	Socorro	0.7	0.6	0.9	1	1	1	0.4	0.3	0.5	1	1	1
Philippines	Sofronio Espanola	0.6	0.4	0.7	2	2	3	0.3	0.2	0.4	1	1	2
Philippines	Sogod	0.6	0.4	0.7	3	2	4	0.3	0.3	0.4	2	1	2
Philippines	Sogod	0.5	0.4	0.7	1	1	1	0.3	0.2	0.4	1	0	1
Philippines	Solana	0.7	0.5	0.9	7	5	9	0.4	0.3	0.5	4	3	5
Philippines	Solano	0.6	0.5	0.7	4	3	5	0.3	0.3	0.4	2	1	2
Philippines	Solsona	0.6	0.4	0.8	1	1	2	0.3	0.2	0.4	1	1	1
Philippines	Sominot	0.6	0.5	0.8	2	1	2	0.3	0.2	0.4	1	1	1
Philippines	Sorsogon City	0.6	0.5	0.7	13	10	17	0.3	0.3	0.4	7	6	9
Philippines	South Ubian	0.7	0.5	0.8	0	0	0	0.3	0.2	0.4	0	0	0
Philippines	South Upi	0.7	0.5	0.9	2	2	3	0.3	0.3	0.4	2	2	2
Philippines	Sual	0.6	0.4	0.7	3	2	3	0.3	0.2	0.4	1	1	2
Philippines	Subic	0.6	0.4	0.7	5	4	7	0.3	0.2	0.4	5	4	6
Philippines	Sudipen	0.5	0.4	0.7	1	1	1	0.3	0.2	0.3	1	0	1
Philippines	Sugbongcogon	0.6	0.4	0.7	0	0	0	0.3	0.2	0.4	0	0	0
Philippines	Sugpon	0.5	0.4	0.7	0	0	0	0.3	0.2	0.3	0	0	0
Philippines	Sulat	0.6	0.5	0.8	2	2	3	0.4	0.3	0.5	0	0	0
Philippines	Sulop	0.6	0.5	0.8	2	1	2	0.3	0.3	0.4	1	1	2
Philippines	Sultan Dumalondong	0.6	0.5	0.8	2	2	3	0.2	0.2	0.3	0	0	0
Philippines	Sultan Kudarat	0.7	0.5	0.9	25	19	32	0.4	0.3	0.5	5	4	6

Table 3: Diarrhoea Mortality by Unit *(continued)*

Country	Unit	2000 mortality mean rate (per 10,000)	lower rate (per 10,000)	upper rate (per 10,000)	mean count	lower count	upper count	2017 mortality mean rate (per 10,000)	lower rate (per 10,000)	upper rate (per 10,000)	mean count	lower count	upper count
Philippines	Sultan Mastura	0.7	0.5	0.9	4	3	5	0.3	0.3	0.4	0	0	1
Philippines	Sultan Naga Dimaporo	0.6	0.5	0.8	1	1	2	0.3	0.2	0.4	1	1	1
Philippines	Sultan Sa Barongis	0.7	0.6	0.9	3	3	4	0.4	0.3	0.5	1	1	1
Philippines	Sumilao	0.5	0.4	0.7	1	1	2	0.3	0.2	0.3	1	1	2
Philippines	Sumisip	0.7	0.5	0.8	10	8	13	0.3	0.2	0.4	1	0	1
Philippines	Surallah	0.7	0.5	0.9	7	5	8	0.4	0.3	0.5	5	4	6
Philippines	Surigao City	0.7	0.5	0.9	11	8	13	0.4	0.3	0.5	7	5	9
Philippines	Suyo	0.5	0.4	0.7	1	1	1	0.3	0.2	0.3	0	0	0
Philippines	T'Boli	0.7	0.5	0.8	7	6	9	0.4	0.3	0.5	7	5	8
Philippines	Taal	0.5	0.4	0.7	3	2	4	0.3	0.2	0.4	1	1	2
Philippines	Taal lake	0.5	0.4	0.7	3	3	4	0.3	0.2	0.3	2	2	2
Philippines	Tabaco City	0.6	0.5	0.7	9	7	11	0.3	0.3	0.4	5	4	6
Philippines	Tabango	0.6	0.4	0.7	2	2	3	0.3	0.2	0.4	1	1	2
Philippines	Tabina	0.6	0.5	0.8	2	2	3	0.3	0.3	0.4	1	1	1
Philippines	Tabogon	0.5	0.4	0.7	2	2	3	0.3	0.2	0.4	1	1	2
Philippines	Tabontabon	0.6	0.4	0.7	1	0	1	0.3	0.2	0.4	0	0	0
Philippines	Tabuelan	0.5	0.4	0.7	1	1	1	0.3	0.2	0.4	1	1	1
Philippines	Tabuk City	0.6	0.5	0.8	6	5	8	0.3	0.3	0.4	7	5	9
Philippines	Tacloban City	0.6	0.5	0.8	9	7	12	0.3	0.2	0.4	5	4	6
Philippines	Tacurong City	0.7	0.5	0.9	7	5	8	0.4	0.3	0.5	5	4	6
Philippines	Tadian	0.6	0.5	0.8	1	1	2	0.3	0.3	0.4	1	1	1
Philippines	Taft	0.6	0.5	0.7	1	1	1	0.3	0.3	0.4	1	1	1
Philippines	Tagana-An	0.7	0.5	0.9	1	1	1	0.4	0.3	0.5	1	0	1
Philippines	Tagapul-An	0.6	0.4	0.7	1	0	1	0.3	0.3	0.4	0	0	0
Philippines	Tagaytay City	0.5	0.4	0.7	5	4	6	0.3	0.2	0.4	2	2	3
Philippines	Tagbilaran City	0.6	0.5	0.7	3	3	4	0.3	0.2	0.4	2	2	3
Philippines	Tagbina	0.7	0.5	0.9	4	3	5	0.4	0.3	0.5	1	1	2
Philippines	Tagkawayan	0.5	0.4	0.6	3	2	3	0.3	0.2	0.3	2	2	3
Philippines	Tago	0.7	0.5	0.9	3	2	3	0.4	0.3	0.5	2	1	2
Philippines	Tagoloan	0.5	0.4	0.7	0	0	1	0.3	0.2	0.3	0	0	1
Philippines	Tagoloan	0.5	0.4	0.7	4	3	5	0.3	0.2	0.4	3	3	4
Philippines	Tagoloan II	0.6	0.5	0.8	2	1	2	0.3	0.2	0.4	0	0	0
Philippines	Tagudin	0.5	0.4	0.7	2	2	2	0.3	0.2	0.3	1	1	1
Philippines	Taguig	0.5	0.4	0.6	29	23	37	0.3	0.2	0.3	16	12	20
Philippines	Tagum City	0.6	0.5	0.7	12	9	15	0.3	0.2	0.4	11	8	13
Philippines	Talacogon	0.6	0.5	0.8	2	2	3	0.3	0.3	0.4	2	1	2
Philippines	Talaingod	0.5	0.4	0.7	1	1	1	0.3	0.2	0.4	2	1	2
Philippines	Talakag	0.6	0.4	0.7	3	3	4	0.3	0.2	0.4	5	4	6
Philippines	Talalora	0.6	0.4	0.7	0	0	0	0.3	0.2	0.4	0	0	0
Philippines	Talavera	0.5	0.4	0.7	5	4	6	0.3	0.2	0.4	3	3	4
Philippines	Talayan	0.7	0.5	0.9	5	4	7	0.3	0.3	0.4	0	0	0
Philippines	Talibon	0.5	0.4	0.7	3	3	4	0.3	0.2	0.4	2	2	3
Philippines	Talipao	0.6	0.5	0.8	7	5	9	0.3	0.2	0.4	1	1	1
Philippines	Talisay	0.6	0.5	0.8	2	2	3	0.4	0.3	0.5	1	1	2
Philippines	Talisay	0.5	0.4	0.7	3	2	3	0.3	0.2	0.3	1	1	2
Philippines	Talisay City	0.6	0.4	0.7	7	6	10	0.3	0.2	0.4	4	3	5
Philippines	Talisay City	0.6	0.4	0.8	10	7	12	0.4	0.3	0.5	9	7	11
Philippines	Talisayan	0.5	0.4	0.7	1	1	1	0.3	0.2	0.3	1	1	1
Philippines	Talitay	0.7	0.6	0.9	5	4	6	0.3	0.3	0.4	0	0	0
Philippines	Talugtug	0.6	0.4	0.7	1	1	2	0.3	0.2	0.4	1	1	1
Philippines	Talusan	0.6	0.5	0.8	2	1	2	0.3	0.3	0.4	1	1	2
Philippines	Tambulig	0.6	0.5	0.7	4	3	4	0.3	0.2	0.4	2	1	2
Philippines	Tampakan	0.7	0.5	0.9	3	2	4	0.4	0.3	0.5	2	2	2
Philippines	Tamparan	0.6	0.5	0.8	2	1	2	0.3	0.2	0.4	0	0	0
Philippines	Tampilisan	0.6	0.5	0.8	1	1	2	0.3	0.3	0.4	1	1	1
Philippines	Tanauan	0.6	0.4	0.7	3	3	4	0.3	0.2	0.4	2	2	3
Philippines	Tanauan City	0.5	0.4	0.6	8	6	10	0.3	0.2	0.3	6	4	7
Philippines	Tanay	0.5	0.4	0.6	5	4	6	0.3	0.2	0.4	3	2	4

Country	Unit	2000 mortality mean rate (per 10,000)	lower rate (per 10,000)	upper rate (per 10,000)	mean count	lower count	upper count	2017 mortality mean rate (per 10,000)	lower rate (per 10,000)	upper rate (per 10,000)	mean count	lower count	upper count
Philippines	Tandag City	0.7	0.5	0.9	5	4	6	0.4	0.3	0.5	3	2	3
Philippines	Tandubas	0.6	0.5	0.8	1	1	2	0.3	0.2	0.4	0	0	0
Philippines	Tangalan	0.6	0.5	0.8	1	1	2	0.4	0.3	0.5	1	1	1
Philippines	Tangcal	0.6	0.4	0.8	1	1	1	0.3	0.2	0.3	1	1	2
Philippines	Tangub City	0.6	0.4	0.7	5	4	6	0.3	0.2	0.4	3	2	4
Philippines	Tanjay City	0.6	0.4	0.7	5	4	7	0.3	0.2	0.4	3	2	3
Philippines	Tantangan	0.7	0.5	0.9	3	2	4	0.4	0.3	0.5	3	2	3
Philippines	Tanudan	0.6	0.5	0.8	1	1	1	0.3	0.3	0.4	0	0	1
Philippines	Tanza	0.6	0.5	0.8	15	12	18	0.3	0.2	0.4	10	8	12
Philippines	Tapaz	0.6	0.4	0.7	4	3	4	0.3	0.2	0.4	2	1	2
Philippines	Tapul	0.6	0.5	0.8	0	0	1	0.3	0.2	0.4	0	0	0
Philippines	Taraka	0.6	0.5	0.8	3	3	4	0.3	0.2	0.4	0	0	0
Philippines	Tarangnan	0.6	0.4	0.7	1	1	1	0.3	0.3	0.4	0	0	1
Philippines	Tarlac City	0.6	0.4	0.7	23	19	30	0.3	0.2	0.4	11	9	14
Philippines	Tarragona	0.6	0.4	0.7	2	2	2	0.3	0.2	0.4	1	1	2
Philippines	Tayabas City	0.5	0.4	0.6	6	4	7	0.3	0.2	0.3	4	3	4
Philippines	Tayasan	0.6	0.4	0.7	2	1	2	0.3	0.2	0.4	1	1	1
Philippines	Taysan	0.5	0.4	0.6	2	1	2	0.3	0.2	0.3	1	1	2
Philippines	Taytay	0.5	0.4	0.7	20	16	25	0.3	0.2	0.4	14	11	17
Philippines	Taytay	0.6	0.4	0.7	4	3	5	0.3	0.2	0.4	4	3	5
Philippines	Tayug	0.6	0.5	0.7	3	2	3	0.3	0.3	0.4	2	1	2
Philippines	Tayum	0.6	0.5	0.8	1	1	2	0.3	0.3	0.4	1	0	1
Philippines	Teresa	0.6	0.5	0.7	7	5	8	0.3	0.3	0.4	4	3	5
Philippines	Ternate	0.6	0.5	0.7	1	1	2	0.3	0.2	0.4	1	0	1
Philippines	Tiaong	0.5	0.4	0.6	5	4	7	0.3	0.2	0.3	3	3	4
Philippines	Tibiao	0.6	0.4	0.8	1	1	2	0.3	0.2	0.4	1	1	1
Philippines	Tigaon	0.5	0.4	0.7	3	3	4	0.3	0.2	0.4	2	2	3
Philippines	Tigbao	0.6	0.5	0.8	2	1	2	0.3	0.2	0.4	1	1	1
Philippines	Tigbauan	0.6	0.5	0.8	4	3	5	0.3	0.2	0.4	2	2	3
Philippines	Tinambac	0.5	0.4	0.7	3	3	5	0.3	0.2	0.4	2	2	3
Philippines	Tineg	0.7	0.5	0.9	0	0	1	0.4	0.3	0.5	0	0	0
Philippines	Tinglayan	0.7	0.5	0.8	1	1	1	0.4	0.3	0.4	1	0	1
Philippines	Tingloy	0.5	0.4	0.7	1	1	1	0.3	0.2	0.4	0	0	0
Philippines	Tinoc	0.6	0.5	0.8	1	1	1	0.3	0.3	0.4	1	1	1
Philippines	Tipo-Tipo	0.6	0.5	0.8	4	3	5	0.3	0.2	0.4	0	0	0
Philippines	Titay	0.6	0.5	0.8	4	3	5	0.3	0.3	0.4	2	2	3
Philippines	Tiwi	0.5	0.4	0.7	4	3	5	0.3	0.2	0.4	2	2	3
Philippines	Tobias Fornier	0.6	0.4	0.7	1	1	2	0.3	0.2	0.4	1	1	1
Philippines	Toboso	0.6	0.4	0.7	3	2	4	0.3	0.2	0.4	2	1	2
Philippines	Toledo City	0.6	0.4	0.8	13	9	16	0.3	0.3	0.4	7	5	9
Philippines	Tolosa	0.6	0.4	0.7	1	1	1	0.3	0.2	0.4	1	1	1
Philippines	Tomas Oppus	0.6	0.4	0.7	1	1	1	0.3	0.2	0.4	0	0	1
Philippines	Tongkil	0.7	0.5	0.8	1	1	1	0.3	0.2	0.4	0	0	0
Philippines	Torrijos	0.5	0.4	0.7	2	2	3	0.3	0.2	0.4	1	1	1
Philippines	Trece Martires City	0.6	0.5	0.7	6	5	8	0.3	0.2	0.4	6	4	7
Philippines	Trento	0.6	0.5	0.8	4	3	5	0.3	0.3	0.5	2	1	2
Philippines	Trinidad	0.5	0.4	0.7	2	1	2	0.3	0.2	0.4	1	1	1
Philippines	Tuao	0.7	0.5	0.9	5	4	6	0.4	0.3	0.4	2	2	3
Philippines	Tuba	0.7	0.5	0.8	6	5	8	0.4	0.3	0.4	4	3	5
Philippines	Tubajon	0.7	0.5	0.8	1	1	1	0.4	0.3	0.5	0	0	0
Philippines	Tubao	0.6	0.5	0.8	2	1	2	0.3	0.3	0.4	1	1	1
Philippines	Tubaran	0.6	0.4	0.7	2	2	3	0.2	0.2	0.3	0	0	0
Philippines	Tubay	0.6	0.5	0.8	1	1	1	0.3	0.3	0.4	1	1	1
Philippines	Tubigon	0.5	0.4	0.7	3	2	3	0.3	0.2	0.4	1	1	2
Philippines	Tublay	0.6	0.5	0.8	1	1	1	0.3	0.3	0.4	1	1	1
Philippines	Tubo	0.6	0.5	0.8	0	0	1	0.3	0.3	0.4	0	0	0
Philippines	Tubod	0.7	0.5	0.9	1	1	1	0.4	0.3	0.5	1	0	1
Philippines	Tubod	0.6	0.4	0.7	2	2	3	0.3	0.2	0.4	2	1	2
Philippines	Tubungan	0.6	0.4	0.8	1	1	2	0.3	0.2	0.4	1	1	1

Table 3: Diarrhoea Mortality by Unit *(continued)*

Country	Unit	2000 mortality mean rate (per 10,000)	lower rate (per 10,000)	upper rate (per 10,000)	mean count	lower count	upper count	2017 mortality mean rate (per 10,000)	lower rate (per 10,000)	upper rate (per 10,000)	mean count	lower count	upper count
Philippines	Tuburan	0.7	0.5	0.8	5	4	6	0.3	0.2	0.4	0	0	0
Philippines	Tuburan	0.6	0.4	0.7	2	2	3	0.3	0.2	0.4	2	1	2
Philippines	Tudela	0.6	0.4	0.7	1	1	2	0.3	0.2	0.4	1	1	1
Philippines	Tudela	0.6	0.4	0.8	1	1	1	0.4	0.3	0.5	0	0	0
Philippines	Tugaya	0.7	0.5	0.9	2	2	3	0.3	0.2	0.4	0	0	1
Philippines	Tuguegarao City	0.7	0.5	0.9	8	6	11	0.4	0.3	0.5	5	4	7
Philippines	Tukuran	0.6	0.5	0.8	3	2	4	0.3	0.3	0.4	2	1	2
Philippines	Tulunan	0.7	0.5	0.9	5	4	6	0.4	0.3	0.5	4	3	5
Philippines	Tumauini	0.6	0.5	0.8	4	3	6	0.3	0.3	0.4	3	2	4
Philippines	Tunga	0.6	0.5	0.7	0	0	0	0.3	0.2	0.4	0	0	0
Philippines	Tungawan	0.6	0.5	0.8	3	3	4	0.3	0.3	0.4	2	2	3
Philippines	Tupi	0.7	0.5	0.9	5	4	6	0.4	0.3	0.5	4	3	4
Philippines	Turtle Islands	NA	NA	NA	NA	NA	NA	NA	NA	NA	NA	NA	NA
Philippines	Tuy	0.5	0.4	0.7	3	2	3	0.3	0.2	0.3	1	1	2
Philippines	Ubay	0.5	0.4	0.7	5	4	6	0.3	0.2	0.4	3	2	4
Philippines	Umingan	0.6	0.5	0.7	4	3	5	0.3	0.3	0.4	3	2	4
Philippines	Ungkaya Pukan	0.7	0.5	0.8	4	3	5	0.3	0.2	0.4	0	0	0
Philippines	Unisan	0.5	0.4	0.6	1	1	2	0.3	0.2	0.3	1	1	1
Philippines	Upi	0.7	0.5	0.9	4	3	5	0.3	0.3	0.4	2	2	3
Philippines	Urbiztondo	0.6	0.5	0.7	4	3	5	0.3	0.2	0.4	3	2	3
Philippines	Urdaneta City	0.6	0.5	0.8	9	7	11	0.3	0.3	0.4	5	4	6
Philippines	Uson	0.6	0.5	0.8	5	4	6	0.4	0.3	0.4	3	3	4
Philippines	Uyugan	NA	NA	NA	NA	NA	NA	NA	NA	NA	NA	NA	NA
Philippines	Valderrama	0.6	0.5	0.8	2	1	2	0.3	0.2	0.4	1	1	1
Philippines	Valencia	0.5	0.4	0.7	2	2	3	0.3	0.2	0.4	2	1	2
Philippines	Valencia	0.5	0.4	0.7	1	1	2	0.3	0.2	0.4	1	0	1
Philippines	Valencia City	0.5	0.4	0.7	11	8	14	0.3	0.2	0.4	9	7	12
Philippines	Valenzuela	0.6	0.5	0.7	44	35	54	0.3	0.2	0.4	22	18	27
Philippines	Valladolid	0.6	0.4	0.8	2	1	2	0.3	0.2	0.4	1	1	1
Philippines	Vallehermoso	0.6	0.4	0.7	3	2	4	0.3	0.2	0.4	2	1	2
Philippines	Veruela	0.6	0.5	0.8	3	3	4	0.3	0.3	0.4	3	2	3
Philippines	Victoria	0.5	0.4	0.7	3	2	4	0.3	0.2	0.4	2	2	3
Philippines	Victoria	0.6	0.4	0.7	4	3	5	0.3	0.2	0.4	2	2	3
Philippines	Victoria	0.5	0.4	0.7	1	1	1	0.3	0.2	0.4	1	0	1
Philippines	Victoria	0.5	0.4	0.7	2	1	2	0.3	0.2	0.3	2	1	2
Philippines	Victorias City	0.6	0.4	0.7	6	4	7	0.3	0.2	0.4	3	2	3
Philippines	Viga	0.6	0.5	0.8	1	1	2	0.4	0.3	0.5	1	1	1
Philippines	Vigan City	0.6	0.4	0.8	2	2	3	0.3	0.2	0.4	1	1	2
Philippines	Villaba	0.5	0.4	0.7	2	1	2	0.3	0.2	0.4	1	1	1
Philippines	Villanueva	0.5	0.4	0.7	2	1	2	0.3	0.2	0.4	1	1	2
Philippines	Villareal	0.6	0.4	0.7	2	2	3	0.3	0.2	0.4	2	1	2
Philippines	Villasis	0.6	0.5	0.7	4	3	5	0.3	0.3	0.4	2	2	3
Philippines	Villaverde	0.6	0.5	0.7	2	1	2	0.3	0.3	0.4	1	1	1
Philippines	Villaviciosa	0.6	0.5	0.8	0	0	1	0.3	0.3	0.4	0	0	0
Philippines	Vincenzo A. Sagun	0.6	0.5	0.8	2	1	2	0.3	0.3	0.4	1	1	1
Philippines	Vintar	0.6	0.4	0.8	2	1	2	0.3	0.2	0.4	1	1	1
Philippines	Vinzons	0.6	0.5	0.8	3	2	3	0.4	0.3	0.5	2	1	2
Philippines	Virac	0.6	0.4	0.7	3	3	4	0.3	0.3	0.4	2	2	2
Philippines	Wao	0.6	0.5	0.8	3	2	3	0.3	0.2	0.4	1	1	2
Philippines	Waterbody	0.5	0.4	0.7	0	0	0	0.3	0.2	0.3	0	0	0
Philippines	Zamboanga City	0.7	0.6	0.9	56	44	70	0.4	0.3	0.5	32	25	40
Philippines	Zamboanguita	0.5	0.4	0.7	2	1	2	0.3	0.2	0.4	1	1	1
Philippines	Zaragoza	0.5	0.4	0.7	3	2	3	0.3	0.2	0.4	2	1	2
Philippines	Zarraga	0.6	0.5	0.8	1	1	2	0.3	0.3	0.4	1	1	1
Philippines	Zumarraga	0.6	0.4	0.7	1	1	2	0.3	0.3	0.4	1	0	1
Sri Lanka	Addalachchenai	0.0	0.0	0.1	0	0	0	0.0	0.0	0.0	0	0	0
Sri Lanka	Agalawatta	0.0	0.0	0.1	0	0	0	0.0	0.0	0.0	0	0	0
Sri Lanka	Akkaraipattu	0.0	0.0	0.1	0	0	0	0.0	0.0	0.0	0	0	0
Sri Lanka	Akmeemana	0.0	0.0	0.1	0	0	0	0.0	0.0	0.0	0	0	0

Table 3: Diarrhoea Mortality by Unit *(continued)*

Country	Unit	2000 mortality mean rate (per 10,000)	lower rate (per 10,000)	upper rate (per 10,000)	mean count	lower count	upper count	2017 mortality mean rate (per 10,000)	lower rate (per 10,000)	upper rate (per 10,000)	mean count	lower count	upper count
Sri Lanka	Akurana	0.1	0.0	0.1	0	0	0	0.0	0.0	0.0	0	0	0
Sri Lanka	Akuressa	0.0	0.0	0.1	0	0	0	0.0	0.0	0.0	0	0	0
Sri Lanka	Alawwa	0.1	0.0	0.1	0	0	0	0.0	0.0	0.0	0	0	0
Sri Lanka	Alayadiwembu	0.0	0.0	0.1	0	0	0	0.0	0.0	0.0	0	0	0
Sri Lanka	Ambagamuwa	0.1	0.0	0.1	1	1	2	0.0	0.0	0.0	0	0	0
Sri Lanka	Ambalangoda	0.0	0.0	0.1	0	0	1	0.0	0.0	0.0	0	0	0
Sri Lanka	Ambalanthota	0.0	0.0	0.1	0	0	0	0.0	0.0	0.0	0	0	0
Sri Lanka	Ambanganga Korale	0.0	0.0	0.1	0	0	0	0.0	0.0	0.0	0	0	0
Sri Lanka	Ambanpola	0.0	0.0	0.1	0	0	0	0.0	0.0	0.0	0	0	0
Sri Lanka	Ampara	0.0	0.0	0.1	0	0	0	0.0	0.0	0.0	0	0	0
Sri Lanka	Anamaduwa	0.1	0.0	0.1	0	0	0	0.0	0.0	0.0	0	0	0
Sri Lanka	Angunakolapelessa	0.0	0.0	0.1	0	0	0	0.0	0.0	0.0	0	0	0
Sri Lanka	Arachchikattuwa PS	0.0	0.0	0.1	0	0	0	0.0	0.0	0.0	0	0	0
Sri Lanka	Aranayaka	0.0	0.0	0.1	0	0	1	0.0	0.0	0.0	0	0	0
Sri Lanka	Athuraliya	0.0	0.0	0.1	0	0	0	0.0	0.0	0.0	0	0	0
Sri Lanka	Attanagalla	0.1	0.0	0.1	1	0	1	0.0	0.0	0.0	0	0	0
Sri Lanka	Ayagama	0.0	0.0	0.1	0	0	0	0.0	0.0	0.0	0	0	0
Sri Lanka	Badalkumbura	0.0	0.0	0.1	0	0	0	0.0	0.0	0.0	0	0	0
Sri Lanka	Baddegama	0.0	0.0	0.1	0	0	1	0.0	0.0	0.0	0	0	0
Sri Lanka	Badulla	0.0	0.0	0.1	0	0	0	0.0	0.0	0.0	0	0	0
Sri Lanka	Balangoda	0.0	0.0	0.1	0	0	1	0.0	0.0	0.0	0	0	0
Sri Lanka	Balapitiya	0.0	0.0	0.1	0	0	0	0.0	0.0	0.0	0	0	0
Sri Lanka	Bamunakotuwa	0.1	0.0	0.1	0	0	0	0.0	0.0	0.0	0	0	0
Sri Lanka	Bandaragama	0.0	0.0	0.1	0	0	1	0.0	0.0	0.0	0	0	0
Sri Lanka	Bandarawela	0.1	0.0	0.1	0	0	0	0.0	0.0	0.0	0	0	0
Sri Lanka	Beliatta	0.0	0.0	0.1	0	0	0	0.0	0.0	0.0	0	0	0
Sri Lanka	Bentota	0.0	0.0	0.1	0	0	0	0.0	0.0	0.0	0	0	0
Sri Lanka	Beruwala	0.0	0.0	0.1	0	0	1	0.0	0.0	0.0	0	0	0
Sri Lanka	Bibile	0.0	0.0	0.1	0	0	0	0.0	0.0	0.0	0	0	0
Sri Lanka	Bingiriya	0.0	0.0	0.1	0	0	0	0.0	0.0	0.0	0	0	0
Sri Lanka	Biyagama	0.0	0.0	0.1	1	0	1	0.0	0.0	0.0	0	0	0
Sri Lanka	Bope-Poddala	0.0	0.0	0.1	0	0	0	0.0	0.0	0.0	0	0	0
Sri Lanka	Bulathkohupitiya	0.0	0.0	0.1	0	0	0	0.0	0.0	0.0	0	0	0
Sri Lanka	Bulathsinhala	0.0	0.0	0.1	0	0	0	0.0	0.0	0.0	0	0	0
Sri Lanka	Buttala	0.0	0.0	0.1	0	0	0	0.0	0.0	0.0	0	0	0
Sri Lanka	Chilaw	0.0	0.0	0.1	0	0	0	0.0	0.0	0.0	0	0	0
Sri Lanka	Colombo	0.0	0.0	0.1	1	1	3	0.0	0.0	0.0	0	0	0
Sri Lanka	Damana	0.0	0.0	0.1	0	0	0	0.0	0.0	0.0	0	0	0
Sri Lanka	Dambulla	0.0	0.0	0.1	0	0	1	0.0	0.0	0.0	0	0	0
Sri Lanka	Dankotuwa	0.0	0.0	0.1	0	0	0	0.0	0.0	0.0	0	0	0
Sri Lanka	Dehiattakandiya	0.0	0.0	0.1	0	0	1	0.0	0.0	0.0	0	0	0
Sri Lanka	Dehiovita	0.0	0.0	0.1	0	0	1	0.0	0.0	0.0	0	0	0
Sri Lanka	Dehiwala-Mount Lavinia	0.0	0.0	0.1	1	0	1	0.0	0.0	0.0	0	0	0
Sri Lanka	Delft	0.0	0.0	0.1	0	0	0	0.0	0.0	0.0	0	0	0
Sri Lanka	Delthota	0.1	0.0	0.1	0	0	0	0.0	0.0	0.0	0	0	0
Sri Lanka	Deraniyagala	0.0	0.0	0.1	0	0	0	0.0	0.0	0.0	0	0	0
Sri Lanka	Devinuwara	0.0	0.0	0.1	0	0	0	0.0	0.0	0.0	0	0	0
Sri Lanka	Dickwella	0.0	0.0	0.1	0	0	0	0.0	0.0	0.0	0	0	0
Sri Lanka	Dimbulagala	0.0	0.0	0.1	0	0	0	0.0	0.0	0.0	0	0	0
Sri Lanka	Divulapitiya	0.0	0.0	0.1	0	0	1	0.0	0.0	0.0	0	0	0
Sri Lanka	Dodangoda	0.1	0.0	0.1	0	0	1	0.0	0.0	0.0	0	0	0
Sri Lanka	Doluwa	0.1	0.0	0.1	0	0	1	0.0	0.0	0.0	0	0	0
Sri Lanka	Dompe	0.1	0.0	0.1	1	0	1	0.0	0.0	0.0	0	0	0
Sri Lanka	Eheliyagoda	0.0	0.0	0.1	0	0	0	0.0	0.0	0.0	0	0	0
Sri Lanka	Ehetuwewa	0.1	0.0	0.1	0	0	0	0.0	0.0	0.0	0	0	0
Sri Lanka	Elahera	0.0	0.0	0.1	0	0	0	0.0	0.0	0.0	0	0	0
Sri Lanka	Elapatha	0.1	0.0	0.1	0	0	0	0.0	0.0	0.0	0	0	0

Table 3: Diarrhoea Mortality by Unit *(continued)*

Country	Unit	2000 mortality mean rate (per 10,000)	lower rate (per 10,000)	upper rate (per 10,000)	mean count	lower count	upper count	2017 mortality mean rate (per 10,000)	lower rate (per 10,000)	upper rate (per 10,000)	mean count	lower count	upper count
Sri Lanka	Ella	0.0	0.0	0.1	0	0	0	0.0	0.0	0.0	0	0	0
Sri Lanka	Elpitiya	0.0	0.0	0.1	0	0	0	0.0	0.0	0.0	0	0	0
Sri Lanka	Embilipitiya	0.1	0.0	0.1	1	0	1	0.0	0.0	0.0	0	0	0
Sri Lanka	Eragama	0.0	0.0	0.1	0	0	0	0.0	0.0	0.0	0	0	0
Sri Lanka	Eravur Pattu	0.0	0.0	0.1	0	0	1	0.0	0.0	0.0	0	0	0
Sri Lanka	Eravur Town	0.0	0.0	0.1	0	0	0	0.0	0.0	0.0	0	0	0
Sri Lanka	Galenbindunuwewa	0.1	0.0	0.1	0	0	0	0.0	0.0	0.0	0	0	0
Sri Lanka	Galewela	0.0	0.0	0.1	0	0	1	0.0	0.0	0.0	0	0	0
Sri Lanka	Galgamuwa	0.1	0.0	0.1	0	0	0	0.0	0.0	0.0	0	0	0
Sri Lanka	Galigamuwa	0.0	0.0	0.1	0	0	1	0.0	0.0	0.0	0	0	0
Sri Lanka	Galle Four Gravets	0.0	0.0	0.1	0	0	0	0.0	0.0	0.0	0	0	0
Sri Lanka	Galnewa	0.1	0.0	0.1	0	0	0	0.0	0.0	0.0	0	0	0
Sri Lanka	Gampaha	0.0	0.0	0.1	1	0	1	0.0	0.0	0.0	0	0	0
Sri Lanka	Ganewatta	0.0	0.0	0.1	0	0	0	0.0	0.0	0.0	0	0	0
Sri Lanka	Ganga Ihala Korale	0.1	0.0	0.1	0	0	1	0.0	0.0	0.0	0	0	0
Sri Lanka	Giribawa	0.1	0.0	0.1	0	0	0	0.0	0.0	0.0	0	0	0
Sri Lanka	Godakawela	0.0	0.0	0.1	0	0	1	0.0	0.0	0.0	0	0	0
Sri Lanka	Gomarankadawala	0.0	0.0	0.1	0	0	0	0.0	0.0	0.0	0	0	0
Sri Lanka	Habaraduwa	0.0	0.0	0.1	0	0	0	0.0	0.0	0.0	0	0	0
Sri Lanka	Hakmana	0.0	0.0	0.1	0	0	0	0.0	0.0	0.0	0	0	0
Sri Lanka	Hali-Ela	0.0	0.0	0.1	0	0	1	0.0	0.0	0.0	0	0	0
Sri Lanka	Hambantota	0.0	0.0	0.1	0	0	0	0.0	0.0	0.0	0	0	0
Sri Lanka	Hanguranketha	0.1	0.0	0.1	0	0	1	0.0	0.0	0.0	0	0	0
Sri Lanka	Hanwella	0.1	0.0	0.1	0	0	1	0.0	0.0	0.0	0	0	0
Sri Lanka	Haputale	0.1	0.0	0.1	0	0	0	0.0	0.0	0.0	0	0	0
Sri Lanka	Harispattuwa	0.1	0.0	0.1	0	0	1	0.0	0.0	0.0	0	0	0
Sri Lanka	Hatharaliyadda	0.0	0.0	0.1	0	0	0	0.0	0.0	0.0	0	0	0
Sri Lanka	Hikkaduwa	0.0	0.0	0.1	0	0	1	0.0	0.0	0.0	0	0	0
Sri Lanka	Hildummulla	0.1	0.0	0.1	0	0	0	0.0	0.0	0.0	0	0	0
Sri Lanka	Hingurakgoda	0.0	0.0	0.1	0	0	1	0.0	0.0	0.0	0	0	0
Sri Lanka	Homagama	0.1	0.0	0.1	1	0	1	0.0	0.0	0.0	0	0	0
Sri Lanka	Horana	0.1	0.0	0.1	0	0	1	0.0	0.0	0.0	0	0	0
Sri Lanka	Horowpothana	0.0	0.0	0.1	0	0	0	0.0	0.0	0.0	0	0	0
Sri Lanka	Ibbagamuwa	0.0	0.0	0.1	0	0	1	0.0	0.0	0.0	0	0	0
Sri Lanka	Imaduwa	0.0	0.0	0.1	0	0	0	0.0	0.0	0.0	0	0	0
Sri Lanka	Imbulpe	0.1	0.0	0.1	0	0	0	0.0	0.0	0.0	0	0	0
Sri Lanka	Ingiriya	0.1	0.0	0.1	0	0	0	0.0	0.0	0.0	0	0	0
Sri Lanka	Ipalogama	0.1	0.0	0.1	0	0	0	0.0	0.0	0.0	0	0	0
Sri Lanka	Island South (Velanai)	0.0	0.0	0.1	0	0	0	0.0	0.0	0.0	0	0	0
Sri Lanka	Islands North (Kayts)	0.0	0.0	0.1	0	0	0	0.0	0.0	0.0	0	0	0
Sri Lanka	Ja-Ela	0.0	0.0	0.1	1	0	1	0.0	0.0	0.0	0	0	0
Sri Lanka	Jaffna	0.0	0.0	0.1	0	0	0	0.0	0.0	0.0	0	0	0
Sri Lanka	K.F.G. & G. Korale	0.1	0.0	0.1	1	0	1	0.0	0.0	0.0	0	0	0
Sri Lanka	Kaduwela	0.1	0.0	0.1	1	1	2	0.0	0.0	0.0	0	0	0
Sri Lanka	Kahatagasdigiliya	0.1	0.0	0.1	0	0	0	0.0	0.0	0.0	0	0	0
Sri Lanka	Kahawatta	0.0	0.0	0.1	0	0	0	0.0	0.0	0.0	0	0	0
Sri Lanka	Kalawana	0.0	0.0	0.1	0	0	0	0.0	0.0	0.0	0	0	0
Sri Lanka	Kalmunai	0.0	0.0	0.1	0	0	0	0.0	0.0	0.0	0	0	0
Sri Lanka	Kalpitiya	0.0	0.0	0.1	0	0	1	0.0	0.0	0.0	0	0	0
Sri Lanka	Kalutara	0.0	0.0	0.1	1	0	1	0.0	0.0	0.0	0	0	0
Sri Lanka	Kamburupitiya	0.0	0.0	0.1	0	0	0	0.0	0.0	0.0	0	0	0
Sri Lanka	Kandaketiya	0.0	0.0	0.1	0	0	0	0.0	0.0	0.0	0	0	0
Sri Lanka	Kandawali	0.0	0.0	0.1	0	0	1	0.0	0.0	0.0	0	0	0
Sri Lanka	Kantalai	0.0	0.0	0.1	0	0	0	0.0	0.0	0.0	0	0	0
Sri Lanka	Karachchi	0.0	0.0	0.1	1	0	1	0.0	0.0	0.0	0	0	0

Table 3: Diarrhoea Mortality by Unit *(continued)*

Country	Unit	2000 mortality mean rate (per 10,000)	lower rate (per 10,000)	upper rate (per 10,000)	mean count	lower count	upper count	2017 mortality mean rate (per 10,000)	lower rate (per 10,000)	upper rate (per 10,000)	mean count	lower count	upper count
Sri Lanka	Karandeniya	0.0	0.0	0.1	0	0	0	0.0	0.0	0.0	0	0	0
Sri Lanka	Karativu	0.0	0.0	0.1	0	0	0	0.0	0.0	0.0	0	0	0
Sri Lanka	Karuwalagaswewa	0.0	0.0	0.1	0	0	0	0.0	0.0	0.0	0	0	0
Sri Lanka	Katana	0.0	0.0	0.1	1	1	2	0.0	0.0	0.0	0	0	0
Sri Lanka	Katharagama	0.0	0.0	0.1	0	0	0	0.0	0.0	0.0	0	0	0
Sri Lanka	Kattankudy	0.0	0.0	0.1	0	0	0	0.0	0.0	0.0	0	0	0
Sri Lanka	Katupotha	0.1	0.0	0.1	0	0	0	0.0	0.0	0.0	0	0	0
Sri Lanka	Katuwana	0.0	0.0	0.1	0	0	0	0.0	0.0	0.0	0	0	0
Sri Lanka	Kebithigollewa	0.0	0.0	0.1	0	0	0	0.0	0.0	0.0	0	0	0
Sri Lanka	Kegalle	0.0	0.0	0.1	0	0	1	0.0	0.0	0.0	0	0	0
Sri Lanka	Kekirawa	0.1	0.0	0.1	0	0	1	0.0	0.0	0.0	0	0	0
Sri Lanka	Kelaniya	0.0	0.0	0.1	1	0	1	0.0	0.0	0.0	0	0	0
Sri Lanka	Kesbewa	0.0	0.0	0.1	1	1	2	0.0	0.0	0.0	0	0	0
Sri Lanka	Kinniya	0.0	0.0	0.1	0	0	1	0.0	0.0	0.0	0	0	0
Sri Lanka	Kiriella	0.0	0.0	0.1	0	0	0	0.0	0.0	0.0	0	0	0
Sri Lanka	Kirinda-Puhulwella	0.0	0.0	0.1	0	0	0	0.0	0.0	0.0	0	0	0
Sri Lanka	Kobeigane	0.0	0.0	0.1	0	0	0	0.0	0.0	0.0	0	0	0
Sri Lanka	Kolonna	0.0	0.0	0.1	0	0	0	0.0	0.0	0.0	0	0	0
Sri Lanka	Kolonnawa	0.0	0.0	0.1	1	0	1	0.0	0.0	0.0	0	0	0
Sri Lanka	Koralai Pattu (Valachchenai)	0.0	0.0	0.1	0	0	1	0.0	0.0	0.0	0	0	0
Sri Lanka	Koralai Pattu North	0.0	0.0	0.1	0	0	0	0.0	0.0	0.0	0	0	0
Sri Lanka	Koralai Pattu West (Oddamavadi)	0.0	0.0	0.1	0	0	0	0.0	0.0	0.0	0	0	0
Sri Lanka	Kotapola	0.0	0.0	0.1	0	0	0	0.0	0.0	0.0	0	0	0
Sri Lanka	Kotavehera	0.0	0.0	0.1	0	0	0	0.0	0.0	0.0	0	0	0
Sri Lanka	Kothmale	0.0	0.0	0.1	0	0	1	0.0	0.0	0.0	0	0	0
Sri Lanka	Kuchchaveli	0.0	0.0	0.1	0	0	0	0.0	0.0	0.0	0	0	0
Sri Lanka	Kuliyapitiya East	0.1	0.0	0.1	0	0	0	0.0	0.0	0.0	0	0	0
Sri Lanka	Kuliyapitiya West	0.0	0.0	0.1	0	0	1	0.0	0.0	0.0	0	0	0
Sri Lanka	Kundasale	0.1	0.0	0.1	1	0	1	0.0	0.0	0.0	0	0	0
Sri Lanka	Kurunegala	0.1	0.0	0.1	0	0	1	0.0	0.0	0.0	0	0	0
Sri Lanka	Kuruvita	0.1	0.0	0.1	0	0	1	0.0	0.0	0.0	0	0	0
Sri Lanka	Laggala-Pallegama	0.0	0.0	0.1	0	0	0	0.0	0.0	0.0	0	0	0
Sri Lanka	Lahugala	0.0	0.0	0.1	0	0	0	0.0	0.0	0.0	0	0	0
Sri Lanka	Lankapura	0.0	0.0	0.1	0	0	0	0.0	0.0	0.0	0	0	0
Sri Lanka	Lunugala	0.0	0.0	0.1	0	0	0	0.0	0.0	0.0	0	0	0
Sri Lanka	Lunugamvehera	0.0	0.0	0.1	0	0	0	0.0	0.0	0.0	0	0	0
Sri Lanka	Madampe	0.0	0.0	0.1	0	0	0	0.0	0.0	0.0	0	0	0
Sri Lanka	Madhu	0.0	0.0	0.1	0	0	0	0.0	0.0	0.0	0	0	0
Sri Lanka	Madulla	0.1	0.0	0.1	0	0	0	0.0	0.0	0.0	0	0	0
Sri Lanka	Madurawala	0.1	0.0	0.1	0	0	0	0.0	0.0	0.0	0	0	0
Sri Lanka	Maha Vilachchiya	0.0	0.0	0.1	0	0	0	0.0	0.0	0.0	0	0	0
Sri Lanka	Mahakumbukkadawala	0.0	0.0	0.1	0	0	0	0.0	0.0	0.0	0	0	0
Sri Lanka	Mahaoya	0.0	0.0	0.1	0	0	0	0.0	0.0	0.0	0	0	0
Sri Lanka	Mahara	0.0	0.0	0.1	1	0	1	0.0	0.0	0.0	0	0	0
Sri Lanka	Maharagama	0.1	0.0	0.1	1	0	1	0.0	0.0	0.0	0	0	0
Sri Lanka	Mahawa	0.0	0.0	0.1	0	0	0	0.0	0.0	0.0	0	0	0
Sri Lanka	Mahawewa	0.0	0.0	0.1	0	0	0	0.0	0.0	0.0	0	0	0
Sri Lanka	Mahiyanganaya	0.0	0.0	0.1	0	0	1	0.0	0.0	0.0	0	0	0
Sri Lanka	Malimbada	0.0	0.0	0.1	0	0	0	0.0	0.0	0.0	0	0	0
Sri Lanka	Mallawapitiya	0.1	0.0	0.1	0	0	0	0.0	0.0	0.0	0	0	0
Sri Lanka	Manmunai North	0.0	0.0	0.1	0	0	0	0.0	0.0	0.0	0	0	0
Sri Lanka	Manmunai Pattu (Araipattai)	0.0	0.0	0.1	0	0	0	0.0	0.0	0.0	0	0	0
Sri Lanka	Manmunai South and Eruvilpattu	0.0	0.0	0.1	0	0	0	0.0	0.0	0.0	0	0	0

Table 3: Diarrhoea Mortality by Unit *(continued)*

Country	Unit	2000 mortality mean rate (per 10,000)	lower rate (per 10,000)	upper rate (per 10,000)	mean count	lower count	upper count	2017 mortality mean rate (per 10,000)	lower rate (per 10,000)	upper rate (per 10,000)	mean count	lower count	upper count
Sri Lanka	Manmunai South-West	0.0	0.0	0.1	0	0	0	0.0	0.0	0.0	0	0	0
Sri Lanka	Manmunai West	0.0	0.0	0.1	0	0	0	0.0	0.0	0.0	0	0	0
Sri Lanka	Mannar Town	0.0	0.0	0.1	0	0	0	0.0	0.0	0.0	0	0	0
Sri Lanka	Manthai East	0.0	0.0	0.1	0	0	0	0.0	0.0	0.0	0	0	0
Sri Lanka	Manthai West	0.0	0.0	0.1	0	0	0	0.0	0.0	0.0	0	0	0
Sri Lanka	Maritimepattu	0.0	0.0	0.1	0	0	0	0.0	0.0	0.0	0	0	0
Sri Lanka	Maspotha	0.0	0.0	0.1	0	0	0	0.0	0.0	0.0	0	0	0
Sri Lanka	Matale	0.0	0.0	0.1	0	0	0	0.0	0.0	0.0	0	0	0
Sri Lanka	Matara Four Gravets	0.0	0.0	0.1	0	0	1	0.0	0.0	0.0	0	0	0
Sri Lanka	Mathugama	0.0	0.0	0.1	0	0	1	0.0	0.0	0.0	0	0	0
Sri Lanka	Mawanella	0.0	0.0	0.1	0	0	1	0.0	0.0	0.0	0	0	0
Sri Lanka	Mawathagama	0.0	0.0	0.1	0	0	0	0.0	0.0	0.0	0	0	0
Sri Lanka	Medadumbara	0.0	0.0	0.1	0	0	1	0.0	0.0	0.0	0	0	0
Sri Lanka	Medagama	0.0	0.0	0.1	0	0	0	0.0	0.0	0.0	0	0	0
Sri Lanka	Medawachchiya	0.1	0.0	0.1	0	0	0	0.0	0.0	0.0	0	0	0
Sri Lanka	Medirigiriya	0.0	0.0	0.1	0	0	1	0.0	0.0	0.0	0	0	0
Sri Lanka	Meegahakivula	0.0	0.0	0.1	0	0	0	0.0	0.0	0.0	0	0	0
Sri Lanka	Mihinthale	0.1	0.0	0.1	0	0	0	0.0	0.0	0.0	0	0	0
Sri Lanka	Millaniya	0.0	0.0	0.1	0	0	0	0.0	0.0	0.0	0	0	0
Sri Lanka	Minipe	0.0	0.0	0.1	0	0	0	0.0	0.0	0.0	0	0	0
Sri Lanka	Minuwangoda	0.0	0.0	0.1	1	0	1	0.0	0.0	0.0	0	0	0
Sri Lanka	Mirigama	0.1	0.0	0.1	1	0	1	0.0	0.0	0.0	0	0	0
Sri Lanka	Moneragala	0.0	0.0	0.1	0	0	0	0.0	0.0	0.0	0	0	0
Sri Lanka	Moratuwa	0.0	0.0	0.1	0	0	1	0.0	0.0	0.0	0	0	0
Sri Lanka	Morawewa	0.0	0.0	0.1	0	0	0	0.0	0.0	0.0	0	0	0
Sri Lanka	Mulatiyana	0.0	0.0	0.1	0	0	0	0.0	0.0	0.0	0	0	0
Sri Lanka	Mundalama	0.0	0.0	0.1	0	0	0	0.0	0.0	0.0	0	0	0
Sri Lanka	Musali	0.0	0.0	0.1	0	0	0	0.0	0.0	0.0	0	0	0
Sri Lanka	Muttur	0.0	0.0	0.1	0	0	0	0.0	0.0	0.0	0	0	0
Sri Lanka	N. Palatha Central	0.1	0.0	0.1	0	0	1	0.0	0.0	0.0	0	0	0
Sri Lanka	N. Palatha East	0.1	0.0	0.1	0	0	0	0.0	0.0	0.0	0	0	0
Sri Lanka	Nachchadoowa	0.1	0.0	0.1	0	0	0	0.0	0.0	0.0	0	0	0
Sri Lanka	Nagoda	0.0	0.0	0.1	0	0	0	0.0	0.0	0.0	0	0	0
Sri Lanka	Nallur	0.0	0.0	0.1	0	0	0	0.0	0.0	0.0	0	0	0
Sri Lanka	Nanaddan	0.0	0.0	0.1	0	0	0	0.0	0.0	0.0	0	0	0
Sri Lanka	Narammala	0.1	0.0	0.1	0	0	0	0.0	0.0	0.0	0	0	0
Sri Lanka	Nattandiya	0.0	0.0	0.1	0	0	0	0.0	0.0	0.0	0	0	0
Sri Lanka	Naula	0.0	0.0	0.1	0	0	0	0.0	0.0	0.0	0	0	0
Sri Lanka	Navithanveli	0.0	0.0	0.1	0	0	0	0.0	0.0	0.0	0	0	0
Sri Lanka	Nawagattegama	0.1	0.0	0.1	0	0	0	0.0	0.0	0.0	0	0	0
Sri Lanka	Negombo	0.0	0.0	0.1	0	0	1	0.0	0.0	0.0	0	0	0
Sri Lanka	Neluwa	0.0	0.0	0.1	0	0	0	0.0	0.0	0.0	0	0	0
Sri Lanka	Nikaweratiya	0.0	0.0	0.1	0	0	0	0.0	0.0	0.0	0	0	0
Sri Lanka	Ninthavur	0.0	0.0	0.1	0	0	0	0.0	0.0	0.0	0	0	0
Sri Lanka	Nivithigala	0.0	0.0	0.1	0	0	0	0.0	0.0	0.0	0	0	0
Sri Lanka	Niyagama	0.0	0.0	0.1	0	0	0	0.0	0.0	0.0	0	0	0
Sri Lanka	Nochchiyagama	0.1	0.0	0.1	0	0	0	0.0	0.0	0.0	0	0	0
Sri Lanka	Nuwara Eliya	0.1	0.0	0.1	1	1	2	0.0	0.0	0.0	0	0	0
Sri Lanka	Oddusuddan	0.0	0.0	0.1	0	0	1	0.0	0.0	0.0	0	0	0
Sri Lanka	Okewela	0.0	0.0	0.1	0	0	0	0.0	0.0	0.0	0	0	0
Sri Lanka	Opanayaka	0.1	0.0	0.1	0	0	0	0.0	0.0	0.0	0	0	0
Sri Lanka	Pachchilaipalli	0.0	0.0	0.1	0	0	0	0.0	0.0	0.0	0	0	0
Sri Lanka	Padavi Sri Pura	0.0	0.0	0.1	0	0	0	0.0	0.0	0.0	0	0	0
Sri Lanka	Padaviya	0.0	0.0	0.1	0	0	0	0.0	0.0	0.0	0	0	0
Sri Lanka	Padiyathalawa	0.0	0.0	0.1	0	0	0	0.0	0.0	0.0	0	0	0
Sri Lanka	Padukka	0.1	0.0	0.1	0	0	0	0.0	0.0	0.0	0	0	0
Sri Lanka	Palagala	0.0	0.0	0.1	0	0	0	0.0	0.0	0.0	0	0	0

Table 3: Diarrhoea Mortality by Unit *(continued)*

Country	Unit	2000 mortality mean rate (per 10,000)	lower rate (per 10,000)	upper rate (per 10,000)	mean count	lower count	upper count	2017 mortality mean rate (per 10,000)	lower rate (per 10,000)	upper rate (per 10,000)	mean count	lower count	upper count
Sri Lanka	Palindanuwara	0.0	0.0	0.1	0	0	0	0.0	0.0	0.0	0	0	0
Sri Lanka	Pallama	0.0	0.0	0.1	0	0	0	0.0	0.0	0.0	0	0	0
Sri Lanka	Pallepola	0.0	0.0	0.1	0	0	0	0.0	0.0	0.0	0	0	0
Sri Lanka	Palugaswewa	0.0	0.0	0.1	0	0	0	0.0	0.0	0.0	0	0	0
Sri Lanka	Panadura	0.0	0.0	0.1	0	0	1	0.0	0.0	0.0	0	0	0
Sri Lanka	Panduwasnuwara	0.0	0.0	0.1	0	0	1	0.0	0.0	0.0	0	0	0
Sri Lanka	Pannala	0.0	0.0	0.1	0	0	1	0.0	0.0	0.0	0	0	0
Sri Lanka	Panvila	0.0	0.0	0.1	0	0	0	0.0	0.0	0.0	0	0	0
Sri Lanka	Pasbage Korale	0.0	0.0	0.1	0	0	1	0.0	0.0	0.0	0	0	0
Sri Lanka	Pasgoda	0.0	0.0	0.1	0	0	0	0.0	0.0	0.0	0	0	0
Sri Lanka	Passara	0.0	0.0	0.1	0	0	1	0.0	0.0	0.0	0	0	0
Sri Lanka	Pathadumbara	0.1	0.0	0.1	0	0	1	0.0	0.0	0.0	0	0	0
Sri Lanka	Pathahewaheta	0.1	0.0	0.1	0	0	1	0.0	0.0	0.0	0	0	0
Sri Lanka	Pelmadulla	0.0	0.0	0.1	0	0	1	0.0	0.0	0.0	0	0	0
Sri Lanka	Pitabeddara	0.0	0.0	0.1	0	0	0	0.0	0.0	0.0	0	0	0
Sri Lanka	Polgahawela	0.1	0.0	0.1	0	0	0	0.0	0.0	0.0	0	0	0
Sri Lanka	Polpithigama	0.0	0.0	0.1	0	0	1	0.0	0.0	0.0	0	0	0
Sri Lanka	Poojapitiya	0.0	0.0	0.1	0	0	0	0.0	0.0	0.0	0	0	0
Sri Lanka	Poonakary	0.0	0.0	0.1	0	0	0	0.0	0.0	0.0	0	0	0
Sri Lanka	Porativu Pattu	0.0	0.0	0.1	0	0	0	0.0	0.0	0.0	0	0	0
Sri Lanka	Pothuvil	0.0	0.0	0.1	0	0	0	0.0	0.0	0.0	0	0	0
Sri Lanka	Puthukudiyiruppu	0.0	0.0	0.1	0	0	1	0.0	0.0	0.0	0	0	0
Sri Lanka	Puttalam	0.0	0.0	0.1	0	0	1	0.0	0.0	0.0	0	0	0
Sri Lanka	Rajanganaya	0.1	0.0	0.1	0	0	0	0.0	0.0	0.0	0	0	0
Sri Lanka	Rambewa	0.1	0.0	0.1	0	0	0	0.0	0.0	0.0	0	0	0
Sri Lanka	Rambukkana	0.0	0.0	0.1	0	0	1	0.0	0.0	0.0	0	0	0
Sri Lanka	Rasnayakapura	0.0	0.0	0.1	0	0	0	0.0	0.0	0.0	0	0	0
Sri Lanka	Ratnapura	0.1	0.0	0.1	1	0	1	0.0	0.0	0.0	0	0	0
Sri Lanka	Rattota	0.0	0.0	0.1	0	0	1	0.0	0.0	0.0	0	0	0
Sri Lanka	Rideegama	0.0	0.0	0.1	0	0	1	0.0	0.0	0.0	0	0	0
Sri Lanka	Rideemaliyadda	0.0	0.0	0.1	0	0	0	0.0	0.0	0.0	0	0	0
Sri Lanka	Ruwanwella	0.0	0.0	0.1	0	0	0	0.0	0.0	0.0	0	0	0
Sri Lanka	Sainthamarathu	0.0	0.0	0.1	0	0	1	0.0	0.0	0.0	0	0	0
Sri Lanka	Samanthurai	0.0	0.0	0.1	0	0	1	0.0	0.0	0.0	0	0	0
Sri Lanka	Seruvila	0.0	0.0	0.1	0	0	0	0.0	0.0	0.0	0	0	0
Sri Lanka	Sevanagala	0.1	0.0	0.1	0	0	0	0.0	0.0	0.0	0	0	0
Sri Lanka	Siyambalanduwa	0.0	0.0	0.1	0	0	0	0.0	0.0	0.0	0	0	0
Sri Lanka	Sooriyawewa	0.0	0.0	0.1	0	0	0	0.0	0.0	0.0	0	0	0
Sri Lanka	Soranathota	0.0	0.0	0.1	0	0	0	0.0	0.0	0.0	0	0	0
Sri Lanka	Sri Jayawardanapura Kotte	0.0	0.0	0.1	0	0	1	0.0	0.0	0.0	0	0	0
Sri Lanka	Tangalle	0.0	0.0	0.1	0	0	0	0.0	0.0	0.0	0	0	0
Sri Lanka	Thalawa	0.1	0.0	0.1	0	0	0	0.0	0.0	0.0	0	0	0
Sri Lanka	Thamankaduwa	0.0	0.0	0.1	0	0	1	0.0	0.0	0.0	0	0	0
Sri Lanka	Thambuttegama	0.1	0.0	0.1	0	0	0	0.0	0.0	0.0	0	0	0
Sri Lanka	Thampalakamam	0.0	0.0	0.1	0	0	0	0.0	0.0	0.0	0	0	0
Sri Lanka	Thanamalvila	0.0	0.0	0.1	0	0	0	0.0	0.0	0.0	0	0	0
Sri Lanka	Thawalama	0.0	0.0	0.1	0	0	0	0.0	0.0	0.0	0	0	0
Sri Lanka	Thenmaradchy (Chavakachcheri)	0.0	0.0	0.1	0	0	0	0.0	0.0	0.0	0	0	0
Sri Lanka	Thihagoda	0.0	0.0	0.1	0	0	0	0.0	0.0	0.0	0	0	0
Sri Lanka	Thimbirigasyaya	0.0	0.0	0.1	1	0	1	0.0	0.0	0.0	0	0	0
Sri Lanka	Thirappane	0.1	0.0	0.1	0	0	0	0.0	0.0	0.0	0	0	0
Sri Lanka	Thirukkovil	0.0	0.0	0.1	0	0	0	0.0	0.0	0.0	0	0	0
Sri Lanka	Thissamaharama	0.0	0.0	0.1	0	0	0	0.0	0.0	0.0	0	0	0
Sri Lanka	Thumpane	0.0	0.0	0.1	0	0	0	0.0	0.0	0.0	0	0	0
Sri Lanka	Thunukkai	0.0	0.0	0.1	0	0	0	0.0	0.0	0.0	0	0	0
Sri Lanka	Trincomalee Town and Gravets	0.0	0.0	0.1	0	0	0	0.0	0.0	0.0	0	0	0

Table 3: Diarrhoea Mortality by Unit *(continued)*

Country	Unit	2000 mortality mean rate (per 10,000)	lower rate (per 10,000)	upper rate (per 10,000)	mean count	lower count	upper count	2017 mortality mean rate (per 10,000)	lower rate (per 10,000)	upper rate (per 10,000)	mean count	lower count	upper count
Sri Lanka	Udadumbara	0.0	0.0	0.1	0	0	0	0.0	0.0	0.0	0	0	0
Sri Lanka	Udapalatha	0.1	0.0	0.1	0	0	1	0.0	0.0	0.0	0	0	0
Sri Lanka	Udubaddawa	0.0	0.0	0.1	0	0	0	0.0	0.0	0.0	0	0	0
Sri Lanka	Udunuwara	0.1	0.0	0.1	0	0	1	0.0	0.0	0.0	0	0	0
Sri Lanka	Uhana	0.0	0.0	0.1	0	0	1	0.0	0.0	0.0	0	0	0
Sri Lanka	Ukuwela	0.1	0.0	0.1	0	0	1	0.0	0.0	0.0	0	0	0
Sri Lanka	Uva Paranagama	0.1	0.0	0.1	0	0	1	0.0	0.0	0.0	0	0	0
Sri Lanka	Vadamaradchi South-West	0.0	0.0	0.1	0	0	0	0.0	0.0	0.0	0	0	0
Sri Lanka	Vadamaradchy East	0.0	0.0	0.1	0	0	0	0.0	0.0	0.0	0	0	0
Sri Lanka	Vadamaradchy North	0.0	0.0	0.1	0	0	0	0.0	0.0	0.0	0	0	0
Sri Lanka	Valikamam East	0.0	0.0	0.1	0	0	0	0.0	0.0	0.0	0	0	0
Sri Lanka	Valikamam North	0.0	0.0	0.1	0	0	0	0.0	0.0	0.0	0	0	0
Sri Lanka	Valikamam South	0.0	0.0	0.1	0	0	0	0.0	0.0	0.0	0	0	0
Sri Lanka	Valikamam South-West	0.0	0.0	0.1	0	0	0	0.0	0.0	0.0	0	0	0
Sri Lanka	Valikamam West	0.0	0.0	0.1	0	0	0	0.0	0.0	0.0	0	0	0
Sri Lanka	Vanathavilluwa	0.0	0.0	0.1	0	0	0	0.0	0.0	0.0	0	0	0
Sri Lanka	Vavuniya	0.1	0.0	0.1	1	0	1	0.0	0.0	0.0	0	0	0
Sri Lanka	Vavuniya North	0.0	0.0	0.1	0	0	0	0.0	0.0	0.0	0	0	0
Sri Lanka	Vavuniya South	0.1	0.0	0.1	0	0	0	0.0	0.0	0.0	0	0	0
Sri Lanka	Vengalacheddiculam	0.0	0.0	0.1	0	0	0	0.0	0.0	0.0	0	0	0
Sri Lanka	Verugal	0.0	0.0	0.1	0	0	0	0.0	0.0	0.0	0	0	0
Sri Lanka	Walallawita	0.0	0.0	0.1	0	0	0	0.0	0.0	0.0	0	0	0
Sri Lanka	Walapane	0.0	0.0	0.1	1	0	1	0.0	0.0	0.0	0	0	0
Sri Lanka	Warakapola	0.1	0.0	0.1	0	0	1	0.0	0.0	0.0	0	0	0
Sri Lanka	Wariyapola	0.1	0.0	0.1	0	0	0	0.0	0.0	0.0	0	0	0
Sri Lanka	Wattala	0.0	0.0	0.1	1	0	1	0.0	0.0	0.0	0	0	0
Sri Lanka	Weeraketiya	0.0	0.0	0.1	0	0	0	0.0	0.0	0.0	0	0	0
Sri Lanka	Weerambugedara	0.1	0.0	0.1	0	0	0	0.0	0.0	0.0	0	0	0
Sri Lanka	Weligama	0.0	0.0	0.1	0	0	1	0.0	0.0	0.0	0	0	0
Sri Lanka	Weligepola	0.0	0.0	0.1	0	0	0	0.0	0.0	0.0	0	0	0
Sri Lanka	Welikanda	0.0	0.0	0.1	0	0	0	0.0	0.0	0.0	0	0	0
Sri Lanka	Welimada	0.1	0.0	0.1	0	0	1	0.0	0.0	0.0	0	0	0
Sri Lanka	Welipitiya	0.0	0.0	0.1	0	0	0	0.0	0.0	0.0	0	0	0
Sri Lanka	Welivitiya-Divithura	0.0	0.0	0.1	0	0	0	0.0	0.0	0.0	0	0	0
Sri Lanka	Wellawaya	0.1	0.0	0.1	0	0	0	0.0	0.0	0.0	0	0	0
Sri Lanka	Wennappuwa	0.0	0.0	0.1	0	0	0	0.0	0.0	0.0	0	0	0
Sri Lanka	Wilgamuwa	0.0	0.0	0.1	0	0	0	0.0	0.0	0.0	0	0	0
Sri Lanka	Yakkalamulla	0.0	0.0	0.1	0	0	0	0.0	0.0	0.0	0	0	0
Sri Lanka	Yatawatta	0.0	0.0	0.1	0	0	0	0.0	0.0	0.0	0	0	0
Sri Lanka	Yatinuwara	0.0	0.0	0.1	0	0	1	0.0	0.0	0.0	0	0	0
Sri Lanka	Yatiyanthota	0.0	0.0	0.1	0	0	0	0.0	0.0	0.0	0	0	0
Thailand	Akat Amnuai	0.1	0.1	0.2	1	0	1	0.0	0.0	0.1	0	0	0
Thailand	Amphawa	0.1	0.1	0.2	0	0	1	0.0	0.0	0.1	0	0	0
Thailand	Amphoe Muang Yasothon	0.1	0.1	0.2	1	1	2	0.0	0.0	0.1	0	0	0
Thailand	Amphoe Sai Mun	0.1	0.1	0.2	0	0	0	0.0	0.0	0.1	0	0	0
Thailand	Ao Luk	0.1	0.0	0.1	0	0	1	0.0	0.0	0.1	0	0	0
Thailand	Aranyaprathet	0.1	0.1	0.2	1	1	1	0.0	0.0	0.1	0	0	1
Thailand	At Samat	0.1	0.1	0.2	1	0	1	0.0	0.0	0.1	0	0	0
Thailand	Bacho	0.1	0.0	0.2	0	0	1	0.0	0.0	0.1	0	0	0
Thailand	Bamnet Narong	0.1	0.1	0.2	0	0	1	0.0	0.0	0.1	0	0	0
Thailand	Ban Bung	0.1	0.0	0.1	1	0	1	0.0	0.0	0.1	0	0	0
Thailand	Ban Chang	0.1	0.0	0.1	0	0	1	0.0	0.0	0.1	0	0	0
Thailand	Ban Dan Lan Hoi	0.1	0.1	0.2	0	0	1	0.0	0.0	0.1	0	0	0
Thailand	Ban Dung	0.1	0.1	0.2	1	1	2	0.0	0.0	0.1	0	0	0

Table 3: Diarrhoea Mortality by Unit *(continued)*

Country	Unit	2000 mortality mean rate (per 10,000)	lower rate (per 10,000)	upper rate (per 10,000)	mean count	lower count	upper count	2017 mortality mean rate (per 10,000)	lower rate (per 10,000)	upper rate (per 10,000)	mean count	lower count	upper count
Thailand	Ban Fang	0.1	0.1	0.2	0	0	1	0.0	0.0	0.1	0	0	0
Thailand	Ban Hong	0.1	0.1	0.2	0	0	1	0.0	0.0	0.1	0	0	0
Thailand	Ban Khai	0.1	0.0	0.1	0	0	1	0.0	0.0	0.1	0	0	0
Thailand	Ban Khok	0.1	0.1	0.2	0	0	0	0.0	0.0	0.1	0	0	0
Thailand	Ban Khwao	0.1	0.1	0.2	0	0	1	0.0	0.0	0.1	0	0	0
Thailand	Ban Kruat	0.1	0.0	0.1	1	0	1	0.0	0.0	0.1	0	0	0
Thailand	Ban Laem	0.1	0.1	0.2	0	0	1	0.0	0.0	0.1	0	0	0
Thailand	Ban Lat	0.1	0.1	0.2	0	0	1	0.0	0.0	0.1	0	0	0
Thailand	Ban Luam	0.1	0.1	0.2	0	0	0	0.0	0.0	0.1	0	0	0
Thailand	Ban Luang	0.1	0.1	0.2	0	0	0	0.0	0.0	0.1	0	0	0
Thailand	Ban Mai Chai Pho	0.1	0.1	0.2	0	0	0	0.0	0.0	0.1	0	0	0
Thailand	Ban Mi	0.1	0.1	0.2	1	0	1	0.0	0.0	0.1	0	0	0
Thailand	Ban Mo	0.1	0.1	0.2	0	0	0	0.0	0.0	0.1	0	0	0
Thailand	Ban Muang	0.1	0.1	0.2	1	0	1	0.0	0.0	0.1	0	0	0
Thailand	Ban Na	0.1	0.1	0.2	0	0	1	0.0	0.0	0.1	0	0	0
Thailand	Ban Na Doem	0.1	0.1	0.2	0	0	0	0.0	0.0	0.1	0	0	0
Thailand	Ban Na San	0.1	0.1	0.2	1	0	1	0.0	0.0	0.1	0	0	0
Thailand	Ban Phaeng	0.1	0.1	0.2	0	0	1	0.0	0.0	0.1	0	0	0
Thailand	Ban Phaeo	0.1	0.1	0.2	1	0	1	0.0	0.0	0.1	0	0	1
Thailand	Ban Phai	0.1	0.1	0.2	1	0	2	0.0	0.0	0.1	0	0	0
Thailand	Ban Pho	0.1	0.1	0.2	0	0	1	0.0	0.0	0.1	0	0	0
Thailand	Ban Phraek	0.1	0.1	0.2	0	0	0	0.0	0.0	0.1	0	0	0
Thailand	Ban Phu	0.1	0.1	0.2	1	1	2	0.0	0.0	0.1	0	0	0
Thailand	Ban Pong	0.1	0.1	0.2	1	1	2	0.0	0.0	0.1	0	0	0
Thailand	Ban Rai	0.1	0.1	0.2	1	0	1	0.0	0.0	0.1	0	0	0
Thailand	Ban Sang	0.1	0.1	0.2	0	0	0	0.0	0.0	0.1	0	0	0
Thailand	Ban Ta Khun	0.1	0.1	0.2	0	0	0	0.0	0.0	0.1	0	0	0
Thailand	Ban Tak	0.1	0.1	0.2	0	0	1	0.0	0.0	0.1	0	0	0
Thailand	Ban Thaen	0.1	0.1	0.2	0	0	1	0.0	0.0	0.1	0	0	0
Thailand	Ban Thi	0.1	0.1	0.2	0	0	0	0.0	0.0	0.1	0	0	0
Thailand	Bang Ban	0.1	0.1	0.2	0	0	1	0.0	0.0	0.1	0	0	0
Thailand	Bang Bo	0.1	0.1	0.2	1	0	1	0.0	0.0	0.1	0	0	0
Thailand	Bang Bon	0.1	0.1	0.2	1	0	1	0.0	0.0	0.1	0	0	0
Thailand	Bang Bua Thong	0.1	0.1	0.2	1	1	2	0.0	0.0	0.1	1	0	1
Thailand	Bang Kaeo	0.1	0.1	0.2	0	0	0	0.0	0.0	0.1	0	0	0
Thailand	Bang Kapi	0.1	0.1	0.2	1	1	2	0.0	0.0	0.1	0	0	1
Thailand	Bang Khae	0.1	0.1	0.2	1	1	2	0.0	0.0	0.1	0	0	1
Thailand	Bang Khan	0.1	0.1	0.2	0	0	1	0.0	0.0	0.1	0	0	0
Thailand	Bang Khen	0.1	0.1	0.2	1	1	2	0.0	0.0	0.1	0	0	1
Thailand	Bang Khla	0.1	0.1	0.1	0	0	0	0.0	0.0	0.1	0	0	0
Thailand	Bang Kho Laem	0.1	0.1	0.2	0	0	1	0.0	0.0	0.1	0	0	0
Thailand	Bang Khon Ti	0.1	0.1	0.2	0	0	0	0.0	0.0	0.1	0	0	0
Thailand	Bang Klam	0.1	0.1	0.2	0	0	1	0.0	0.0	0.1	0	0	0
Thailand	Bang Krathum	0.1	0.1	0.2	0	0	1	0.0	0.0	0.1	0	0	0
Thailand	Bang Kruai	0.1	0.1	0.2	1	0	1	0.0	0.0	0.1	0	0	0
Thailand	Bang Lamung	0.1	0.0	0.1	1	0	2	0.0	0.0	0.0	0	0	1
Thailand	Bang Len	0.1	0.1	0.2	1	0	1	0.0	0.0	0.1	0	0	0
Thailand	Bang Mun Nak	0.1	0.1	0.2	0	0	1	0.0	0.0	0.1	0	0	0
Thailand	Bang Na	0.1	0.1	0.2	1	0	1	0.0	0.0	0.1	0	0	0
Thailand	Bang Nam Prieo	0.1	0.1	0.2	1	0	1	0.0	0.0	0.1	0	0	0
Thailand	Bang Pa-In	0.1	0.1	0.2	1	0	1	0.0	0.0	0.1	0	0	0
Thailand	Bang Pahan	0.1	0.1	0.2	0	0	1	0.0	0.0	0.1	0	0	0
Thailand	Bang Pakong	0.1	0.1	0.1	1	0	1	0.0	0.0	0.1	0	0	0
Thailand	Bang Phae	0.1	0.1	0.2	0	0	1	0.0	0.0	0.1	0	0	0
Thailand	Bang Pla Ma	0.1	0.1	0.2	1	0	1	0.0	0.0	0.1	0	0	0
Thailand	Bang Plad	0.1	0.1	0.2	0	0	1	0.0	0.0	0.1	0	0	0
Thailand	Bang Plee	0.1	0.1	0.2	1	1	2	0.0	0.0	0.1	1	0	1
Thailand	Bang Rachan	0.1	0.1	0.2	0	0	1	0.0	0.0	0.1	0	0	0
Thailand	Bang Rak	0.1	0.1	0.2	0	0	0	0.0	0.0	0.1	0	0	0

Table 3: Diarrhoea Mortality by Unit *(continued)*

Country	Unit	2000 mortality mean rate (per 10,000)	lower rate (per 10,000)	upper rate (per 10,000)	mean count	lower count	upper count	2017 mortality mean rate (per 10,000)	lower rate (per 10,000)	upper rate (per 10,000)	mean count	lower count	upper count
Thailand	Bang Rakam	0.1	0.1	0.2	1	1	2	0.0	0.0	0.1	0	0	0
Thailand	Bang Sai	0.1	0.1	0.2	1	0	1	0.0	0.0	0.1	0	0	0
Thailand	Bang Saphan	0.1	0.0	0.1	0	0	1	0.0	0.0	0.0	0	0	0
Thailand	Bang Saphan Noi	0.1	0.0	0.1	0	0	0	0.0	0.0	0.1	0	0	0
Thailand	Bang Su	0.1	0.1	0.2	0	0	1	0.0	0.0	0.1	0	0	0
Thailand	Bang Yai	0.1	0.1	0.2	0	0	1	0.0	0.0	0.1	0	0	0
Thailand	Bangkhuntien	0.1	0.1	0.2	1	1	2	0.0	0.0	0.1	1	0	1
Thailand	Bangkok Noi	0.1	0.1	0.2	1	0	1	0.0	0.0	0.1	0	0	0
Thailand	Bangkok Yai	0.1	0.1	0.2	0	0	1	0.0	0.0	0.1	0	0	0
Thailand	Bannang Star	0.1	0.1	0.2	1	0	1	0.0	0.0	0.1	0	0	0
Thailand	Banphot Phisai	0.1	0.1	0.2	1	0	1	0.0	0.0	0.1	0	0	0
Thailand	Batong	0.1	0.1	0.2	0	0	1	0.0	0.0	0.1	0	0	0
Thailand	Benchalak	0.1	0.1	0.2	0	0	0	0.0	0.0	0.1	0	0	0
Thailand	Bo Klue	0.1	0.1	0.2	0	0	0	0.1	0.0	0.1	0	0	0
Thailand	Bo Phloi	0.1	0.1	0.2	0	0	1	0.0	0.0	0.1	0	0	0
Thailand	Bo Rai	0.1	0.1	0.2	0	0	1	0.0	0.0	0.1	0	0	0
Thailand	Bo Thong	0.1	0.0	0.1	0	0	0	0.0	0.0	0.0	0	0	0
Thailand	Borabu	0.1	0.1	0.2	1	0	2	0.0	0.0	0.1	0	0	0
Thailand	Bua Chet	0.1	0.1	0.1	0	0	1	0.0	0.0	0.1	0	0	0
Thailand	Bua Yai	0.1	0.1	0.2	1	0	1	0.0	0.0	0.1	0	0	0
Thailand	Buang Sam Phan	0.1	0.1	0.2	1	0	1	0.0	0.0	0.1	0	0	0
Thailand	Bung Bun	0.1	0.1	0.2	0	0	0	0.0	0.0	0.1	0	0	0
Thailand	Bung Kan	0.1	0.1	0.2	1	1	1	0.0	0.0	0.1	0	0	0
Thailand	Bung Khong Long	0.1	0.1	0.2	0	0	1	0.0	0.0	0.1	0	0	0
Thailand	Bung Kum	0.1	0.1	0.2	1	0	1	0.0	0.0	0.1	0	0	0
Thailand	Buntharik	0.1	0.1	0.2	1	1	1	0.0	0.0	0.1	0	0	0
Thailand	Cha-Am	0.1	0.1	0.2	0	0	1	0.0	0.0	0.1	0	0	0
Thailand	Cha-uat	0.1	0.1	0.2	1	0	1	0.0	0.0	0.1	0	0	0
Thailand	Chae Hom	0.1	0.1	0.2	0	0	1	0.0	0.0	0.1	0	0	0
Thailand	Chai Badan	0.1	0.1	0.2	1	0	1	0.0	0.0	0.1	0	0	0
Thailand	Chai Prakarn	0.1	0.1	0.2	0	0	1	0.0	0.0	0.1	0	0	0
Thailand	Chai Wan	0.1	0.1	0.2	0	0	1	0.0	0.0	0.1	0	0	0
Thailand	Chaiburi	0.1	0.1	0.1	0	0	0	0.0	0.0	0.1	0	0	0
Thailand	Chaiya	0.1	0.1	0.2	0	0	1	0.0	0.0	0.1	0	0	0
Thailand	Chaiyo	0.1	0.1	0.2	0	0	0	0.0	0.0	0.1	0	0	0
Thailand	Chakkarat	0.1	0.1	0.2	1	0	1	0.0	0.0	0.1	0	0	0
Thailand	Chalermphrakiet	0.1	0.1	0.2	0	0	0	0.0	0.0	0.1	0	0	0
Thailand	Chalermphrakiet	0.1	0.0	0.1	0	0	0	0.0	0.0	0.1	0	0	0
Thailand	Chalermphrakiet	0.1	0.1	0.2	0	0	1	0.0	0.0	0.1	0	0	0
Thailand	Chalermphrakiet	0.1	0.1	0.2	0	0	1	0.1	0.0	0.1	0	0	0
Thailand	Chalermphrakiet	0.1	0.1	0.2	0	0	0	0.0	0.0	0.1	0	0	0
Thailand	Cham Ni	0.1	0.0	0.1	0	0	0	0.0	0.0	0.1	0	0	0
Thailand	Chana	0.1	0.1	0.2	1	0	2	0.0	0.0	0.1	0	0	0
Thailand	Changhan	0.1	0.1	0.2	0	0	1	0.0	0.0	0.1	0	0	0
Thailand	Chanuman	0.1	0.1	0.2	0	0	1	0.0	0.0	0.1	0	0	0
Thailand	Chareon Silp	0.1	0.1	0.2	0	0	1	0.0	0.0	0.1	0	0	0
Thailand	Chat Trakan	0.1	0.1	0.2	0	0	1	0.0	0.0	0.1	0	0	0
Thailand	Chatturat	0.1	0.1	0.2	1	0	1	0.0	0.0	0.1	0	0	0
Thailand	Chatuchak	0.1	0.1	0.2	1	1	2	0.0	0.0	0.1	0	0	1
Thailand	Chaturaphak Phim	0.1	0.1	0.2	1	0	1	0.0	0.0	0.1	0	0	0
Thailand	Chawang	0.1	0.0	0.1	0	0	1	0.0	0.0	0.1	0	0	0
Thailand	Chian Yai	0.1	0.1	0.2	0	0	1	0.0	0.0	0.1	0	0	0
Thailand	Chiang Dao	0.1	0.1	0.2	1	0	1	0.0	0.0	0.1	0	0	0
Thailand	Chiang Kham	0.1	0.1	0.2	1	0	1	0.0	0.0	0.1	0	0	0
Thailand	Chiang Khan	0.1	0.1	0.2	0	0	1	0.0	0.0	0.1	0	0	0
Thailand	Chiang Khong	0.1	0.1	0.2	1	0	1	0.1	0.0	0.1	0	0	0
Thailand	Chiang Klang	0.1	0.1	0.2	0	0	0	0.1	0.0	0.1	0	0	0
Thailand	Chiang Muan	0.1	0.1	0.2	0	0	0	0.0	0.0	0.1	0	0	0

Country	Unit	2000 mortality mean rate (per 10,000)	lower rate (per 10,000)	upper rate (per 10,000)	mean count	lower count	upper count	2017 mortality mean rate (per 10,000)	lower rate (per 10,000)	upper rate (per 10,000)	mean count	lower count	upper count
Thailand	Chiang Saen	0.1	0.1	0.2	1	0	1	0.1	0.0	0.1	0	0	0
Thailand	Chiang Yun	0.1	0.1	0.2	1	0	1	0.0	0.0	0.1	0	0	0
Thailand	Cho-I-rong	0.1	0.0	0.2	0	0	1	0.0	0.0	0.1	0	0	0
Thailand	Chok Chai	0.1	0.1	0.2	1	0	1	0.0	0.0	0.1	0	0	0
Thailand	Chom Bung	0.1	0.1	0.2	1	0	1	0.0	0.0	0.1	0	0	0
Thailand	Chom Phra	0.1	0.1	0.2	1	0	1	0.0	0.0	0.1	0	0	0
Thailand	Chom Thong	0.1	0.1	0.2	1	1	2	0.0	0.0	0.1	0	0	1
Thailand	Chom Thong	0.1	0.1	0.2	1	0	1	0.0	0.0	0.1	0	0	0
Thailand	Chon Daen	0.1	0.1	0.2	1	0	1	0.0	0.0	0.1	0	0	0
Thailand	Chonnabot	0.1	0.1	0.2	0	0	1	0.0	0.0	0.1	0	0	0
Thailand	Chulaphon	0.1	0.1	0.2	1	0	1	0.0	0.0	0.1	0	0	0
Thailand	Chum Phae	0.1	0.1	0.2	1	1	2	0.0	0.0	0.1	0	0	0
Thailand	Chum Phuang	0.1	0.1	0.2	1	0	1	0.0	0.0	0.1	0	0	0
Thailand	Chumphon Buri	0.1	0.1	0.2	1	0	1	0.0	0.0	0.1	0	0	0
Thailand	Chumsaeng	0.1	0.1	0.2	1	0	1	0.0	0.0	0.1	0	0	0
Thailand	Chun	0.1	0.1	0.2	0	0	1	0.0	0.0	0.1	0	0	0
Thailand	Damnoen Saduak	0.1	0.1	0.2	1	0	1	0.0	0.0	0.1	0	0	0
Thailand	Dan Chang	0.1	0.1	0.2	1	0	1	0.0	0.0	0.1	0	0	0
Thailand	Dan Khun Thot	0.1	0.1	0.2	1	1	2	0.0	0.0	0.1	0	0	0
Thailand	Dan Makham Tia	0.1	0.1	0.2	0	0	0	0.0	0.0	0.1	0	0	0
Thailand	Dan Sai	0.1	0.1	0.2	0	0	1	0.0	0.0	0.1	0	0	0
Thailand	Den Chai	0.1	0.1	0.2	0	0	0	0.0	0.0	0.1	0	0	0
Thailand	Det Udom	0.1	0.1	0.2	1	1	3	0.0	0.0	0.1	0	0	1
Thailand	Din Dang	0.1	0.1	0.2	0	0	1	0.0	0.0	0.1	0	0	0
Thailand	Doembang Nangbua	0.1	0.1	0.2	1	0	1	0.0	0.0	0.1	0	0	0
Thailand	Doi Saket	0.1	0.1	0.2	0	0	1	0.0	0.0	0.1	0	0	0
Thailand	Doi Tao	0.1	0.1	0.2	0	0	0	0.0	0.0	0.1	0	0	0
Thailand	Dok Kham Tai	0.1	0.1	0.2	1	0	1	0.0	0.0	0.1	0	0	0
Thailand	Don Chedi	0.1	0.1	0.2	0	0	1	0.0	0.0	0.1	0	0	0
Thailand	Don Muang	0.1	0.1	0.2	1	1	2	0.0	0.0	0.1	0	0	1
Thailand	Don Phut	0.1	0.1	0.2	0	0	0	0.0	0.0	0.1	0	0	0
Thailand	Don Sak	0.1	0.0	0.1	0	0	0	0.0	0.0	0.1	0	0	0
Thailand	Don Tan	0.1	0.1	0.2	0	0	1	0.0	0.0	0.1	0	0	0
Thailand	Don Tum	0.1	0.1	0.2	0	0	1	0.0	0.0	0.1	0	0	0
Thailand	Dong Luang	0.1	0.1	0.2	0	0	1	0.0	0.0	0.1	0	0	0
Thailand	Donmotdaeng	0.1	0.1	0.2	0	0	0	0.0	0.0	0.1	0	0	0
Thailand	Dusit	0.1	0.1	0.2	1	0	1	0.0	0.0	0.1	0	0	0
Thailand	Fak Tha	0.1	0.1	0.2	0	0	0	0.0	0.0	0.1	0	0	0
Thailand	Fang	0.1	0.1	0.2	1	0	1	0.0	0.0	0.1	0	0	0
Thailand	Han Kha	0.1	0.1	0.2	0	0	1	0.0	0.0	0.1	0	0	0
Thailand	Hang Chat	0.1	0.1	0.2	0	0	1	0.0	0.0	0.1	0	0	0
Thailand	Hang Dong	0.1	0.1	0.2	1	0	1	0.0	0.0	0.1	0	0	0
Thailand	Hat Yai	0.1	0.1	0.2	3	1	5	0.0	0.0	0.1	1	1	1
Thailand	Hot	0.1	0.1	0.2	0	0	1	0.0	0.0	0.1	0	0	0
Thailand	Hua Hin	0.1	0.1	0.2	1	0	1	0.0	0.0	0.1	0	0	0
Thailand	Hua Sai	0.1	0.1	0.2	1	0	1	0.0	0.0	0.1	0	0	0
Thailand	Hua Taphan	0.1	0.1	0.2	0	0	1	0.0	0.0	0.1	0	0	0
Thailand	Huai Khot	0.1	0.1	0.2	0	0	0	0.0	0.0	0.1	0	0	0
Thailand	Huai Kra Chao	0.1	0.1	0.2	0	0	0	0.0	0.0	0.1	0	0	0
Thailand	Huai Kwang	0.1	0.1	0.2	1	0	1	0.0	0.0	0.1	0	0	0
Thailand	Huai Mek	0.1	0.1	0.2	0	0	1	0.0	0.0	0.1	0	0	0
Thailand	Huai Phung	0.1	0.1	0.2	0	0	0	0.0	0.0	0.1	0	0	0
Thailand	Huai Rat	0.1	0.0	0.1	0	0	0	0.0	0.0	0.1	0	0	0
Thailand	Huai Thalang	0.1	0.1	0.2	1	0	1	0.0	0.0	0.1	0	0	0
Thailand	Huai Thap Than	0.1	0.1	0.1	0	0	1	0.0	0.0	0.1	0	0	0
Thailand	Huai Yot	0.1	0.1	0.2	1	0	1	0.0	0.0	0.1	0	0	0
Thailand	In Buri	0.1	0.1	0.2	1	0	1	0.0	0.0	0.1	0	0	0
Thailand	Ja-Nae	0.1	0.0	0.2	0	0	1	0.0	0.0	0.1	0	0	0
Thailand	K. Ban Dan	0.1	0.1	0.2	0	0	0	0.0	0.0	0.1	0	0	0

Table 3: Diarrhoea Mortality by Unit *(continued)*

		2000 mortality						2017 mortality					
Country	Unit	mean rate (per 10,000)	lower rate (per 10,000)	upper rate (per 10,000)	mean count	lower count	upper count	mean rate (per 10,000)	lower rate (per 10,000)	upper rate (per 10,000)	mean count	lower count	upper count
Thailand	K. Ban Haet	0.1	0.1	0.2	0	0	0	0.0	0.0	0.1	0	0	0
Thailand	K. Ban Kha	0.1	0.1	0.2	0	0	0	0.0	0.0	0.1	0	0	0
Thailand	K. Bang Sao Thon	0.1	0.1	0.2	0	0	1	0.0	0.0	0.1	0	0	0
Thailand	K. Bua Lai	0.1	0.1	0.2	0	0	0	0.0	0.0	0.1	0	0	0
Thailand	K. Bung Khla	0.1	0.1	0.2	0	0	0	0.0	0.0	0.1	0	0	0
Thailand	K. Bung Narang	0.1	0.1	0.2	0	0	0	0.0	0.0	0.1	0	0	0
Thailand	K. Bung Samakki	0.1	0.1	0.2	0	0	0	0.0	0.0	0.1	0	0	0
Thailand	K. Chang Klang	0.1	0.0	0.1	0	0	0	0.0	0.0	0.1	0	0	0
Thailand	K. Chiang Kwan	0.1	0.1	0.2	0	0	0	0.0	0.0	0.1	0	0	0
Thailand	K. Chum Ta Bong	0.1	0.1	0.2	0	0	0	0.0	0.0	0.1	0	0	0
Thailand	K. Chun Chom	0.1	0.1	0.2	0	0	0	0.0	0.0	0.1	0	0	0
Thailand	K. Daen Kong	0.1	0.1	0.2	0	0	1	0.0	0.0	0.1	0	0	0
Thailand	K. Doi Lo	0.1	0.1	0.2	0	0	0	0.0	0.0	0.1	0	0	0
Thailand	K. Doi Luang	0.1	0.1	0.2	0	0	0	0.1	0.0	0.1	0	0	0
Thailand	K. Don Chan	0.1	0.1	0.2	0	0	0	0.0	0.0	0.1	0	0	0
Thailand	K. Dong Charoen	0.1	0.1	0.2	0	0	0	0.0	0.0	0.1	0	0	0
Thailand	K. Erawan	0.1	0.1	0.2	0	0	0	0.0	0.0	0.1	0	0	0
Thailand	K. Fao Rai	0.1	0.1	0.2	0	0	1	0.0	0.0	0.1	0	0	0
Thailand	K. Hat Samran	0.1	0.1	0.2	0	0	0	0.0	0.0	0.1	0	0	0
Thailand	K. Kao Kichakut	0.1	0.0	0.1	0	0	0	0.0	0.0	0.1	0	0	0
Thailand	K. Khao Chamao	0.1	0.0	0.1	0	0	0	0.0	0.0	0.1	0	0	0
Thailand	K. Khlong Khuan	0.1	0.1	0.2	0	0	0	0.0	0.0	0.1	0	0	0
Thailand	K. Khok Pho Cha	0.1	0.1	0.2	0	0	0	0.0	0.0	0.1	0	0	0
Thailand	K. Ko Chan	0.1	0.0	0.1	0	0	0	0.0	0.0	0.0	0	0	0
Thailand	K. Ko Chang	0.1	0.1	0.1	0	0	0	0.0	0.0	0.1	0	0	0
Thailand	K. Ko Kut	0.1	0.0	0.1	0	0	0	0.0	0.0	0.1	0	0	0
Thailand	K. Ko Sam Pi Nakhon	0.1	0.1	0.2	0	0	0	0.0	0.0	0.1	0	0	0
Thailand	K. Kok Sung	0.1	0.1	0.2	0	0	0	0.0	0.0	0.1	0	0	0
Thailand	K. Kong Chai	0.1	0.1	0.2	0	0	0	0.0	0.0	0.1	0	0	0
Thailand	K. Krong Pi Nung	0.1	0.1	0.2	0	0	0	0.0	0.0	0.1	0	0	0
Thailand	K. Ku Kaeo	0.1	0.1	0.2	0	0	0	0.0	0.0	0.1	0	0	0
Thailand	K. Kut Rang	0.1	0.1	0.2	0	0	1	0.0	0.0	0.1	0	0	0
Thailand	K. Kwao Si Narin	0.1	0.1	0.1	0	0	0	0.0	0.0	0.1	0	0	0
Thailand	K. Lam Tha Men Chai	0.1	0.1	0.2	0	0	1	0.0	0.0	0.1	0	0	0
Thailand	K. Lao Sua Kok	0.1	0.1	0.2	0	0	0	0.0	0.0	0.1	0	0	0
Thailand	K. Ma Nang	0.1	0.1	0.2	0	0	0	0.0	0.0	0.1	0	0	0
Thailand	K. Mae On	0.1	0.1	0.2	0	0	0	0.0	0.0	0.1	0	0	0
Thailand	K. Mae Poen	0.1	0.1	0.2	0	0	0	0.0	0.0	0.1	0	0	0
Thailand	K. Muang Yang	0.1	0.1	0.2	0	0	0	0.0	0.0	0.1	0	0	0
Thailand	K. Na Du	0.1	0.1	0.2	0	0	0	0.0	0.0	0.1	0	0	0
Thailand	K. Na Tan	0.1	0.1	0.2	0	0	1	0.0	0.0	0.1	0	0	0
Thailand	K. Na Yai Am	0.1	0.1	0.1	0	0	0	0.0	0.0	0.1	0	0	0
Thailand	K. Na Yia	0.1	0.1	0.2	0	0	0	0.0	0.0	0.1	0	0	0
Thailand	K. Nam Khun	0.1	0.1	0.2	0	0	0	0.0	0.0	0.1	0	0	0
Thailand	K. Nikhom Pattan	0.1	0.0	0.1	0	0	0	0.0	0.0	0.1	0	0	0
Thailand	K. Noen Kham	0.1	0.1	0.2	0	0	0	0.0	0.0	0.1	0	0	0
Thailand	K. Non Narai	0.1	0.1	0.2	0	0	1	0.0	0.0	0.1	0	0	0
Thailand	K. Non Sila	0.1	0.1	0.2	0	0	0	0.0	0.0	0.1	0	0	0
Thailand	K. Nong Hi	0.1	0.1	0.2	0	0	0	0.0	0.0	0.1	0	0	0
Thailand	K. Nong Hin	0.1	0.1	0.2	0	0	0	0.0	0.0	0.1	0	0	0
Thailand	K. Nong Ma Mong	0.1	0.1	0.2	0	0	0	0.0	0.0	0.1	0	0	0
Thailand	K. Nong Na Kham	0.1	0.1	0.2	0	0	0	0.0	0.0	0.1	0	0	0

Table 3: Diarrhoea Mortality by Unit *(continued)*

Country	Unit	2000 mortality mean rate (per 10,000)	lower rate (per 10,000)	upper rate (per 10,000)	mean count	lower count	upper count	2017 mortality mean rate (per 10,000)	lower rate (per 10,000)	upper rate (per 10,000)	mean count	lower count	upper count
Thailand	K. Nophi Tam	0.1	0.0	0.2	0	0	0	0.0	0.0	0.1	0	0	0
Thailand	K. Phanom Dong Rak	0.1	0.0	0.1	0	0	1	0.0	0.0	0.1	0	0	0
Thailand	K. Pho Si Suwan	0.1	0.1	0.2	0	0	0	0.0	0.0	0.1	0	0	0
Thailand	K. Pho Tak	0.1	0.1	0.2	0	0	0	0.0	0.0	0.1	0	0	0
Thailand	K. Phra Thong Kham	0.1	0.1	0.2	0	0	1	0.0	0.0	0.1	0	0	0
Thailand	K. Phu Kam Yao	0.1	0.1	0.2	0	0	0	0.0	0.0	0.1	0	0	0
Thailand	K. Phu Pieng	0.1	0.1	0.2	0	0	0	0.0	0.0	0.1	0	0	0
Thailand	K. Phu Sang	0.1	0.1	0.2	0	0	0	0.1	0.0	0.1	0	0	0
Thailand	K. Prachak Silapakhom	0.1	0.1	0.2	0	0	0	0.0	0.0	0.1	0	0	0
Thailand	K. Rattana Wapi	0.1	0.1	0.2	0	0	1	0.0	0.0	0.1	0	0	0
Thailand	K. Sa Khrai	0.1	0.1	0.2	0	0	0	0.0	0.0	0.1	0	0	0
Thailand	K. Sak Lek	0.1	0.1	0.2	0	0	0	0.0	0.0	0.1	0	0	0
Thailand	K. Sam Chai	0.1	0.1	0.2	0	0	0	0.0	0.0	0.1	0	0	0
Thailand	K. Sam Roi Yot	0.1	0.1	0.2	0	0	1	0.0	0.0	0.1	0	0	0
Thailand	K. Sam Sung	0.1	0.1	0.2	0	0	0	0.0	0.0	0.1	0	0	0
Thailand	K. Sap Yai	0.1	0.1	0.2	0	0	0	0.0	0.0	0.1	0	0	0
Thailand	K. Sawang Weeraw	0.1	0.1	0.2	0	0	0	0.0	0.0	0.1	0	0	0
Thailand	K. Sida	0.1	0.1	0.2	0	0	0	0.0	0.0	0.1	0	0	0
Thailand	K. Sila Lat	0.1	0.1	0.2	0	0	0	0.0	0.0	0.1	0	0	0
Thailand	K. Sri Nakarin	0.1	0.1	0.2	0	0	0	0.0	0.0	0.1	0	0	0
Thailand	K. Sri Narong	0.1	0.0	0.1	0	0	1	0.0	0.0	0.1	0	0	0
Thailand	K. Suk Samran	0.1	0.1	0.2	0	0	0	0.0	0.0	0.1	0	0	0
Thailand	K. The Pha Rak	0.1	0.1	0.2	0	0	0	0.0	0.0	0.1	0	0	0
Thailand	K. Thung Kao Lua	0.1	0.1	0.2	0	0	0	0.0	0.0	0.1	0	0	0
Thailand	K. Wang Chao	0.1	0.1	0.2	0	0	1	0.0	0.0	0.1	0	0	0
Thailand	K. Wang Sombun	0.1	0.0	0.1	0	0	0	0.0	0.0	0.1	0	0	0
Thailand	K. Wang Yang	0.1	0.1	0.2	0	0	0	0.0	0.0	0.1	0	0	0
Thailand	K. Wiang Nong Long	0.1	0.1	0.2	0	0	0	0.0	0.0	0.1	0	0	0
Thailand	K. Wieng Chiang	0.1	0.1	0.2	0	0	0	0.1	0.0	0.1	0	0	0
Thailand	K. Wipawadi	0.1	0.1	0.2	0	0	0	0.0	0.0	0.1	0	0	0
Thailand	Ka Bang	0.1	0.1	0.2	0	0	0	0.0	0.0	0.1	0	0	0
Thailand	Ka Pho	0.1	0.0	0.2	0	0	0	0.0	0.0	0.1	0	0	0
Thailand	Kabin Buri	0.1	0.0	0.1	1	1	2	0.0	0.0	0.1	0	0	1
Thailand	Kae Dam	0.1	0.1	0.2	0	0	0	0.0	0.0	0.1	0	0	0
Thailand	Kaeng Khlo	0.1	0.1	0.2	1	0	1	0.0	0.0	0.1	0	0	0
Thailand	Kaeng Khoi	0.1	0.1	0.2	1	0	1	0.0	0.0	0.1	0	0	0
Thailand	Kaeng Krachan	0.1	0.1	0.2	0	0	0	0.0	0.0	0.1	0	0	0
Thailand	Kaeng Sanam Nang	0.1	0.1	0.2	0	0	1	0.0	0.0	0.1	0	0	0
Thailand	Kamalasai	0.1	0.1	0.2	1	0	1	0.0	0.0	0.1	0	0	0
Thailand	Kamphaeng Saen	0.1	0.1	0.2	1	1	1	0.0	0.0	0.1	0	0	0
Thailand	Kanchanadit	0.1	0.1	0.2	1	0	1	0.0	0.0	0.1	0	0	0
Thailand	Kang Hang Maeo	0.1	0.0	0.1	0	0	0	0.0	0.0	0.1	0	0	0
Thailand	Kantharalak	0.1	0.1	0.2	2	1	3	0.0	0.0	0.1	0	0	0
Thailand	Kanthararom	0.1	0.1	0.2	1	0	1	0.0	0.0	0.1	0	0	0
Thailand	Kantharawichai	0.1	0.1	0.2	1	0	1	0.0	0.0	0.1	0	0	0
Thailand	Kantrang	0.1	0.1	0.2	1	0	1	0.0	0.0	0.1	0	0	0
Thailand	Kao Cha Kan	0.1	0.0	0.1	0	0	1	0.0	0.0	0.1	0	0	0
Thailand	Kao Lieo	0.1	0.1	0.2	0	0	1	0.0	0.0	0.1	0	0	0
Thailand	Kap Choeng	0.1	0.0	0.1	1	0	1	0.0	0.0	0.1	0	0	0
Thailand	Kapoe	0.1	0.1	0.2	0	0	0	0.0	0.0	0.1	0	0	0
Thailand	Kapong	0.1	0.1	0.2	0	0	0	0.0	0.0	0.1	0	0	0
Thailand	Kaset Sombon	0.1	0.1	0.2	1	1	2	0.0	0.0	0.1	0	0	0
Thailand	Kaset Wisai	0.1	0.1	0.2	1	0	2	0.0	0.0	0.1	0	0	0

Table 3: Diarrhoea Mortality by Unit *(continued)*

Country	Unit	2000 mortality						2017 mortality					
		mean rate (per 10,000)	lower rate (per 10,000)	upper rate (per 10,000)	mean count	lower count	upper count	mean rate (per 10,000)	lower rate (per 10,000)	upper rate (per 10,000)	mean count	lower count	upper count
Thailand	Kathu	0.1	0.0	0.1	0	0	0	0.0	0.0	0.1	0	0	0
Thailand	Khai Bang Rachan	0.1	0.1	0.2	0	0	0	0.0	0.0	0.1	0	0	0
Thailand	Kham Khuan Kaeo	0.1	0.1	0.2	1	0	1	0.0	0.0	0.1	0	0	0
Thailand	Kham Muang	0.1	0.1	0.2	0	0	1	0.0	0.0	0.1	0	0	0
Thailand	Kham Sakae Saeng	0.1	0.1	0.2	0	0	1	0.0	0.0	0.1	0	0	0
Thailand	Kham Ta Kla	0.1	0.1	0.2	0	0	1	0.0	0.0	0.1	0	0	0
Thailand	Kham Thala So	0.1	0.1	0.2	0	0	0	0.0	0.0	0.1	0	0	0
Thailand	Khamcha-i	0.1	0.1	0.2	0	0	1	0.0	0.0	0.1	0	0	0
Thailand	Khan Na Yao	0.1	0.1	0.2	1	0	1	0.0	0.0	0.1	0	0	0
Thailand	Khanom	0.1	0.0	0.1	0	0	0	0.0	0.0	0.1	0	0	0
Thailand	Khanu Woralaksaburi	0.1	0.1	0.2	1	1	2	0.0	0.0	0.1	0	0	0
Thailand	Khao Chaison	0.1	0.1	0.2	0	0	1	0.0	0.0	0.1	0	0	0
Thailand	Khao Kho	0.1	0.1	0.2	0	0	1	0.0	0.0	0.1	0	0	0
Thailand	Khao Phanom	0.1	0.0	0.1	0	0	1	0.0	0.0	0.1	0	0	0
Thailand	Khao Saming	0.1	0.1	0.2	0	0	1	0.0	0.0	0.1	0	0	0
Thailand	Khao Suan Kwang	0.1	0.1	0.2	0	0	1	0.0	0.0	0.1	0	0	0
Thailand	Khao Wong	0.1	0.1	0.2	0	0	0	0.0	0.0	0.1	0	0	0
Thailand	Khao Yoi	0.1	0.1	0.2	0	0	0	0.0	0.0	0.1	0	0	0
Thailand	Khemarat	0.1	0.1	0.2	1	0	1	0.0	0.0	0.1	0	0	0
Thailand	Khian Sa	0.1	0.1	0.2	0	0	1	0.0	0.0	0.1	0	0	0
Thailand	Khiri Mat	0.1	0.1	0.2	1	0	1	0.0	0.0	0.1	0	0	0
Thailand	Khiri Ratthanikhom	0.1	0.1	0.2	0	0	1	0.0	0.0	0.1	0	0	0
Thailand	Khlong Hat	0.1	0.0	0.1	0	0	0	0.0	0.0	0.1	0	0	0
Thailand	Khlong Hoi Kong	0.1	0.1	0.2	0	0	0	0.0	0.0	0.1	0	0	0
Thailand	Khlong Luang	0.1	0.1	0.2	1	1	2	0.0	0.0	0.1	1	0	1
Thailand	Khlong Sam Wa	0.1	0.1	0.2	1	1	2	0.0	0.0	0.1	1	0	1
Thailand	Khlong San	0.1	0.1	0.2	0	0	1	0.0	0.0	0.1	0	0	0
Thailand	Khlong Thom	0.1	0.0	0.1	1	0	1	0.0	0.0	0.1	0	0	0
Thailand	Khlong Toey	0.1	0.1	0.2	1	0	1	0.0	0.0	0.1	0	0	0
Thailand	Khlong Yai	0.1	0.1	0.2	0	0	0	0.0	0.0	0.1	0	0	0
Thailand	Khlung	0.1	0.1	0.2	0	0	1	0.0	0.0	0.1	0	0	0
Thailand	Kho Wang	0.1	0.1	0.2	0	0	0	0.0	0.0	0.1	0	0	0
Thailand	Khok Charoen	0.1	0.1	0.2	0	0	0	0.0	0.0	0.1	0	0	0
Thailand	Khok Pho	0.1	0.0	0.2	1	0	1	0.0	0.0	0.1	0	0	0
Thailand	Khok Samrong	0.1	0.1	0.2	1	0	1	0.0	0.0	0.1	0	0	0
Thailand	Khok Sri Supan	0.1	0.1	0.2	0	0	0	0.0	0.0	0.1	0	0	0
Thailand	Khon Buri	0.1	0.1	0.2	1	0	1	0.0	0.0	0.1	0	0	0
Thailand	Khon San	0.1	0.1	0.2	1	0	1	0.0	0.0	0.1	0	0	0
Thailand	Khon Sawan	0.1	0.1	0.2	0	0	1	0.0	0.0	0.1	0	0	0
Thailand	Khong	0.1	0.1	0.2	1	0	1	0.0	0.0	0.1	0	0	0
Thailand	Khong Chiam	0.1	0.1	0.2	0	0	1	0.0	0.0	0.1	0	0	0
Thailand	Khu Muang	0.1	0.1	0.2	1	0	1	0.0	0.0	0.1	0	0	0
Thailand	Khuan Don	0.1	0.0	0.2	0	0	0	0.0	0.0	0.1	0	0	0
Thailand	Khuan Ka Long	0.1	0.1	0.2	0	0	1	0.0	0.0	0.1	0	0	0
Thailand	Khuan Khanun	0.1	0.1	0.2	1	0	1	0.0	0.0	0.1	0	0	0
Thailand	Khuan Niang	0.1	0.1	0.2	0	0	1	0.0	0.0	0.1	0	0	0
Thailand	Khuang Nai	0.1	0.1	0.2	1	1	2	0.0	0.0	0.1	0	0	0
Thailand	Khukhan	0.1	0.1	0.1	1	1	2	0.0	0.0	0.1	0	0	0
Thailand	Khun Han	0.1	0.1	0.2	1	0	1	0.0	0.0	0.1	0	0	0
Thailand	Khun Tan	0.1	0.1	0.2	0	0	0	0.1	0.0	0.1	0	0	0
Thailand	Khun Yuam	0.1	0.1	0.2	0	0	0	0.0	0.0	0.1	0	0	0
Thailand	Khura Buri	0.1	0.1	0.2	0	0	0	0.0	0.0	0.1	0	0	0
Thailand	Klaeng	0.1	0.0	0.1	1	0	1	0.0	0.0	0.1	0	0	1
Thailand	Klong Khlung	0.1	0.1	0.2	1	0	1	0.0	0.0	0.1	0	0	0

Table 3: Diarrhoea Mortality by Unit *(continued)*

Country	Unit	2000 mortality mean rate (per 10,000)	lower rate (per 10,000)	upper rate (per 10,000)	mean count	lower count	upper count	2017 mortality mean rate (per 10,000)	lower rate (per 10,000)	upper rate (per 10,000)	mean count	lower count	upper count
Thailand	Klong Lan	0.1	0.1	0.2	1	0	1	0.0	0.0	0.1	0	0	0
Thailand	Ko Kha	0.1	0.1	0.2	0	0	1	0.0	0.0	0.1	0	0	0
Thailand	Ko Lanta	0.1	0.0	0.2	0	0	0	0.0	0.0	0.1	0	0	0
Thailand	Ko Phangan	0.1	0.0	0.1	0	0	0	0.0	0.0	0.1	0	0	0
Thailand	Ko Samui	0.1	0.0	0.1	0	0	0	0.0	0.0	0.1	0	0	0
Thailand	Ko Sichang	0.1	0.0	0.1	0	0	0	0.0	0.0	0.1	0	0	0
Thailand	Ko Yao	0.1	0.0	0.1	0	0	0	0.0	0.0	0.1	0	0	0
Thailand	Kong Krailat	0.1	0.1	0.2	1	0	1	0.0	0.0	0.1	0	0	0
Thailand	Kong Ra	0.1	0.1	0.2	0	0	1	0.0	0.0	0.1	0	0	0
Thailand	Kosum Phisai	0.1	0.1	0.2	1	1	2	0.0	0.0	0.1	0	0	0
Thailand	Kra Buri	0.1	0.1	0.2	0	0	1	0.0	0.0	0.1	0	0	0
Thailand	Kranuan	0.1	0.1	0.2	1	0	1	0.0	0.0	0.1	0	0	0
Thailand	Krasae Sinthu	0.1	0.1	0.2	0	0	0	0.0	0.0	0.1	0	0	0
Thailand	Krasang	0.1	0.0	0.1	1	1	2	0.0	0.0	0.1	0	0	0
Thailand	Krathum Baen	0.1	0.1	0.2	1	1	2	0.0	0.0	0.1	1	0	1
Thailand	Krok Phra	0.1	0.1	0.2	0	0	0	0.0	0.0	0.1	0	0	0
Thailand	Kuchinarai	0.1	0.1	0.2	1	0	1	0.0	0.0	0.1	0	0	0
Thailand	Kui Buri	0.1	0.1	0.2	0	0	0	0.0	0.0	0.1	0	0	0
Thailand	Kumphawapi	0.1	0.1	0.2	1	1	2	0.0	0.0	0.1	0	0	0
Thailand	Kusuman	0.1	0.1	0.2	0	0	1	0.0	0.0	0.1	0	0	0
Thailand	Kut Bak	0.1	0.1	0.2	0	0	0	0.0	0.0	0.1	0	0	0
Thailand	Kut Chap	0.1	0.1	0.2	1	0	1	0.0	0.0	0.1	0	0	0
Thailand	Kut Chum	0.1	0.1	0.2	1	0	1	0.0	0.0	0.1	0	0	0
Thailand	Kut Khao Pun	0.1	0.1	0.2	0	0	1	0.0	0.0	0.1	0	0	0
Thailand	La-Un	0.1	0.1	0.2	0	0	0	0.0	0.0	0.1	0	0	0
Thailand	Laem Ngop	0.1	0.1	0.2	0	0	0	0.0	0.0	0.1	0	0	0
Thailand	Laem Sing	0.1	0.1	0.2	0	0	0	0.0	0.0	0.1	0	0	0
Thailand	Lahan Sai	0.1	0.0	0.1	1	0	1	0.0	0.0	0.1	0	0	0
Thailand	Lak Si	0.1	0.1	0.2	1	0	1	0.0	0.0	0.1	0	0	0
Thailand	Lam Luk Ka	0.1	0.1	0.2	1	1	2	0.0	0.0	0.1	1	0	1
Thailand	Lam Plai Mat	0.1	0.0	0.1	1	1	2	0.0	0.0	0.1	0	0	0
Thailand	Lam Son Thi	0.1	0.1	0.2	0	0	0	0.0	0.0	0.1	0	0	0
Thailand	Lam Thap	0.1	0.0	0.1	0	0	0	0.0	0.0	0.1	0	0	0
Thailand	Lamae	0.1	0.0	0.2	0	0	0	0.0	0.0	0.1	0	0	0
Thailand	Lamduan	0.1	0.0	0.1	0	0	0	0.0	0.0	0.1	0	0	0
Thailand	Lan Krabu	0.1	0.1	0.2	0	0	1	0.0	0.0	0.1	0	0	0
Thailand	Lan Sak	0.1	0.1	0.2	1	0	1	0.0	0.0	0.1	0	0	0
Thailand	Lan Saka	0.1	0.0	0.1	0	0	0	0.0	0.0	0.1	0	0	0
Thailand	Lang Suan	0.1	0.1	0.2	1	0	1	0.0	0.0	0.1	0	0	0
Thailand	Langu	0.1	0.0	0.2	1	0	1	0.0	0.0	0.1	0	0	0
Thailand	Lao Khwan	0.1	0.1	0.2	0	0	1	0.0	0.0	0.1	0	0	0
Thailand	Laplae	0.1	0.1	0.2	1	0	1	0.0	0.0	0.1	0	0	0
Thailand	Lat Bua Luang	0.1	0.1	0.2	0	0	1	0.0	0.0	0.1	0	0	0
Thailand	Lat Krabang	0.1	0.1	0.2	1	1	2	0.0	0.0	0.1	1	0	1
Thailand	Lat Lum Kaeo	0.1	0.1	0.2	0	0	1	0.0	0.0	0.1	0	0	0
Thailand	Lat Phrao	0.1	0.1	0.2	1	0	1	0.0	0.0	0.1	0	0	0
Thailand	Lat Yao	0.1	0.1	0.2	1	0	1	0.0	0.0	0.1	0	0	0
Thailand	Li	0.1	0.1	0.2	1	0	1	0.0	0.0	0.1	0	0	0
Thailand	Loeng Nok Tha	0.1	0.1	0.2	1	1	1	0.0	0.0	0.1	0	0	0
Thailand	Lom Kao	0.1	0.1	0.2	0	0	1	0.0	0.0	0.1	0	0	0
Thailand	Lom Sak	0.1	0.1	0.2	1	1	2	0.0	0.0	0.1	0	0	0
Thailand	Long	0.1	0.1	0.2	0	0	1	0.0	0.0	0.1	0	0	0
Thailand	Lu Amnat	0.1	0.1	0.2	0	0	0	0.0	0.0	0.1	0	0	0
Thailand	Mae Ai	0.1	0.1	0.2	1	0	1	0.0	0.0	0.1	0	0	0
Thailand	Mae Chaem	0.1	0.1	0.2	1	0	1	0.0	0.0	0.1	0	0	0
Thailand	Mae Chai	0.1	0.1	0.2	0	0	0	0.0	0.0	0.1	0	0	0
Thailand	Mae Chan	0.1	0.1	0.2	1	1	1	0.1	0.0	0.1	0	0	0
Thailand	Mae Charim	0.1	0.1	0.2	0	0	0	0.0	0.0	0.1	0	0	0
Thailand	Mae Fa Luang	0.1	0.1	0.2	1	0	1	0.0	0.0	0.1	0	0	0
Thailand	Mae La Noi	0.1	0.1	0.2	0	0	1	0.0	0.0	0.1	0	0	0

Country	Unit	2000 mortality						2017 mortality					
		mean rate (per 10,000)	lower rate (per 10,000)	upper rate (per 10,000)	mean count	lower count	upper count	mean rate (per 10,000)	lower rate (per 10,000)	upper rate (per 10,000)	mean count	lower count	upper count
Thailand	Mae Lan	0.1	0.0	0.2	0	0	0	0.0	0.0	0.1	0	0	0
Thailand	Mae Lao	0.1	0.1	0.2	0	0	0	0.0	0.0	0.1	0	0	0
Thailand	Mae Mo	0.1	0.1	0.2	0	0	0	0.0	0.0	0.1	0	0	0
Thailand	Mae Phrik	0.1	0.1	0.2	0	0	0	0.0	0.0	0.1	0	0	0
Thailand	Mae Ramat	0.1	0.1	0.2	1	0	1	0.0	0.0	0.1	0	0	0
Thailand	Mae Rim	0.1	0.1	0.2	1	0	1	0.0	0.0	0.1	0	0	0
Thailand	Mae Sai	0.1	0.1	0.2	1	0	1	0.1	0.0	0.1	0	0	0
Thailand	Mae Sariang	0.1	0.1	0.2	1	0	1	0.0	0.0	0.1	0	0	0
Thailand	Mae Sot	0.1	0.1	0.2	2	1	3	0.0	0.0	0.1	0	0	1
Thailand	Mae Suai	0.1	0.1	0.2	1	0	1	0.0	0.0	0.1	0	0	0
Thailand	Mae Taeng	0.1	0.1	0.2	1	0	1	0.0	0.0	0.1	0	0	0
Thailand	Mae Tha	0.1	0.1	0.2	0	0	1	0.0	0.0	0.1	0	0	0
Thailand	Mae Tha	0.1	0.1	0.2	0	0	1	0.0	0.0	0.1	0	0	0
Thailand	Mae Wang	0.1	0.1	0.2	0	0	0	0.0	0.0	0.1	0	0	0
Thailand	Mae Wong	0.1	0.1	0.2	0	0	1	0.0	0.0	0.1	0	0	0
Thailand	Maha Chana Chai	0.1	0.1	0.2	1	0	1	0.0	0.0	0.1	0	0	0
Thailand	Maha Rat	0.1	0.1	0.2	0	0	0	0.0	0.0	0.1	0	0	0
Thailand	Mai Kaen	0.1	0.0	0.2	0	0	0	0.0	0.0	0.1	0	0	0
Thailand	Makham	0.1	0.1	0.1	0	0	0	0.0	0.0	0.1	0	0	0
Thailand	Mancha Khiri	0.1	0.1	0.2	1	0	1	0.0	0.0	0.1	0	0	0
Thailand	Manorom	0.1	0.1	0.2	0	0	0	0.0	0.0	0.1	0	0	0
Thailand	Mayo	0.1	0.0	0.2	1	0	1	0.0	0.0	0.1	0	0	0
Thailand	Min Buri	0.1	0.1	0.1	1	1	2	0.0	0.0	0.1	0	0	1
Thailand	Moei Wadi	0.1	0.1	0.2	0	0	0	0.0	0.0	0.1	0	0	0
Thailand	Muak Lek	0.1	0.1	0.2	0	0	1	0.0	0.0	0.1	0	0	0
Thailand	Muang Amnat Charoen	0.1	0.1	0.2	1	1	2	0.0	0.0	0.1	0	0	0
Thailand	Muang Ang Thong	0.1	0.1	0.2	0	0	1	0.0	0.0	0.1	0	0	0
Thailand	Muang Buri Ram	0.1	0.0	0.1	2	1	3	0.0	0.0	0.1	0	0	0
Thailand	Muang Chachoengsao	0.1	0.1	0.2	1	0	1	0.0	0.0	0.1	0	0	0
Thailand	Muang Chai Nat	0.1	0.1	0.2	1	0	1	0.0	0.0	0.1	0	0	0
Thailand	Muang Chaiyaphum	0.1	0.1	0.2	1	1	3	0.0	0.0	0.1	0	0	0
Thailand	Muang Chan	0.1	0.1	0.2	0	0	0	0.0	0.0	0.1	0	0	0
Thailand	Muang Chanthaburi	0.1	0.1	0.2	1	0	1	0.0	0.0	0.1	0	0	0
Thailand	Muang Chiang Mai	0.1	0.1	0.2	1	1	2	0.0	0.0	0.1	0	0	1
Thailand	Muang Chiang Rai	0.1	0.1	0.2	2	1	3	0.0	0.0	0.1	0	0	1
Thailand	Muang Chon Buri	0.1	0.0	0.1	1	1	2	0.0	0.0	0.1	1	0	1
Thailand	Muang Chumphon	0.1	0.1	0.2	1	1	2	0.0	0.0	0.1	0	0	0
Thailand	Muang Kalasin	0.1	0.1	0.2	1	1	2	0.0	0.0	0.1	0	0	0
Thailand	Muang Kanchanaburi	0.1	0.1	0.2	1	1	2	0.0	0.0	0.1	0	0	0
Thailand	Muang Khon Kaen	0.1	0.1	0.2	3	1	5	0.0	0.0	0.1	1	0	1
Thailand	Muang Krabi	0.1	0.0	0.1	1	0	1	0.0	0.0	0.1	0	0	0
Thailand	Muang Lampang	0.1	0.1	0.2	2	1	3	0.0	0.0	0.1	0	0	0
Thailand	Muang Lamphun	0.1	0.1	0.2	1	1	2	0.0	0.0	0.1	0	0	0
Thailand	Muang Loei	0.1	0.1	0.2	1	0	2	0.0	0.0	0.1	0	0	0
Thailand	Muang Lop Buri	0.1	0.1	0.2	2	1	3	0.0	0.0	0.1	0	0	1
Thailand	Muang Mae Hong Son	0.1	0.1	0.2	0	0	1	0.0	0.0	0.1	0	0	0
Thailand	Muang Maha Sarakam	0.1	0.1	0.2	1	1	2	0.0	0.0	0.1	0	0	0

Table 3: Diarrhoea Mortality by Unit *(continued)*

Country	Unit	2000 mortality mean rate (per 10,000)	lower rate (per 10,000)	upper rate (per 10,000)	mean count	lower count	upper count	2017 mortality mean rate (per 10,000)	lower rate (per 10,000)	upper rate (per 10,000)	mean count	lower count	upper count
Thailand	Muang Mukdahan	0.1	0.1	0.2	1	1	2	0.0	0.0	0.1	0	0	1
Thailand	Muang Nakhon Nayok	0.1	0.1	0.2	1	0	1	0.0	0.0	0.1	0	0	0
Thailand	Muang Nakhon Pathom	0.1	0.1	0.2	2	1	3	0.0	0.0	0.1	1	0	1
Thailand	Muang Nakhon Phanom	0.1	0.1	0.2	2	1	3	0.0	0.0	0.1	0	0	0
Thailand	Muang Nakhon Ratchasima	0.1	0.1	0.2	3	2	5	0.0	0.0	0.1	1	0	1
Thailand	Muang Nakhon Sawan	0.1	0.1	0.2	2	1	3	0.0	0.0	0.1	0	0	1
Thailand	Muang Nakhon Si Thammarat	0.1	0.0	0.1	2	1	3	0.0	0.0	0.1	0	0	1
Thailand	Muang Nan	0.1	0.1	0.2	1	0	1	0.0	0.0	0.1	0	0	0
Thailand	Muang Narathiwat	0.1	0.0	0.2	1	0	2	0.0	0.0	0.0	0	0	0
Thailand	Muang Nong Bua Lam Phu	0.1	0.1	0.2	1	1	2	0.0	0.0	0.1	0	0	0
Thailand	Muang Nong Khai	0.1	0.1	0.2	1	1	2	0.0	0.0	0.1	0	0	0
Thailand	Muang Nonthaburi	0.1	0.1	0.2	2	1	3	0.0	0.0	0.1	1	0	1
Thailand	Muang Pathum Thani	0.1	0.1	0.2	1	1	2	0.0	0.0	0.1	1	0	1
Thailand	Muang Pattani	0.1	0.0	0.2	1	0	1	0.0	0.0	0.1	0	0	0
Thailand	Muang Phangnga	0.1	0.0	0.2	0	0	1	0.0	0.0	0.1	0	0	0
Thailand	Muang Phatthalung	0.1	0.1	0.2	1	1	2	0.0	0.0	0.1	0	0	0
Thailand	Muang Phayao	0.1	0.1	0.2	1	1	2	0.0	0.0	0.1	0	0	0
Thailand	Muang Phetchabun	0.1	0.1	0.2	2	1	3	0.0	0.0	0.1	0	0	0
Thailand	Muang Phetchaburi	0.1	0.1	0.2	1	0	1	0.0	0.0	0.1	0	0	0
Thailand	Muang Phichit	0.1	0.1	0.2	1	1	2	0.0	0.0	0.1	0	0	0
Thailand	Muang Phitsanulok	0.1	0.1	0.2	2	1	4	0.0	0.0	0.1	1	0	1
Thailand	Muang Phrae	0.1	0.1	0.2	1	0	1	0.0	0.0	0.1	0	0	0
Thailand	Muang Phuket	0.1	0.0	0.1	1	0	1	0.0	0.0	0.1	1	0	1
Thailand	Muang Prachin Buri	0.1	0.0	0.2	1	0	1	0.0	0.0	0.1	0	0	0
Thailand	Muang Prachuap Khiri Khan	0.1	0.0	0.1	1	0	1	0.0	0.0	0.1	0	0	0
Thailand	Muang Ranong	0.1	0.1	0.2	1	0	1	0.0	0.0	0.1	0	0	1
Thailand	Muang Ratchaburi	0.1	0.1	0.2	1	1	2	0.0	0.0	0.1	0	0	0
Thailand	Muang Rayong	0.1	0.0	0.1	1	1	2	0.0	0.0	0.1	1	0	1
Thailand	Muang Roi Et	0.1	0.1	0.2	1	1	2	0.0	0.0	0.1	0	0	0
Thailand	Muang Sa Kaeo	0.1	0.0	0.1	1	0	1	0.0	0.0	0.1	0	0	0
Thailand	Muang Sakon Nakhon	0.1	0.1	0.2	1	1	2	0.0	0.0	0.1	0	0	0
Thailand	Muang Samsip	0.1	0.1	0.2	1	0	1	0.0	0.0	0.1	0	0	0
Thailand	Muang Samut Prakan	0.1	0.1	0.2	2	1	4	0.0	0.0	0.1	1	1	2
Thailand	Muang Samut Sakhon	0.1	0.1	0.2	1	1	2	0.0	0.0	0.1	1	1	1
Thailand	Muang Samut Songkhram	0.1	0.1	0.2	1	0	1	0.0	0.0	0.1	0	0	0
Thailand	Muang Saraburi	0.1	0.1	0.2	1	0	1	0.0	0.0	0.1	0	0	0
Thailand	Muang Satun	0.1	0.0	0.2	1	0	1	0.0	0.0	0.1	0	0	0

Table 3: Diarrhoea Mortality by Unit *(continued)*

Country	Unit	2000 mortality mean rate (per 10,000)	lower rate (per 10,000)	upper rate (per 10,000)	mean count	lower count	upper count	2017 mortality mean rate (per 10,000)	lower rate (per 10,000)	upper rate (per 10,000)	mean count	lower count	upper count
Thailand	Muang Si Sa Ket	0.1	0.1	0.2	1	1	2	0.0	0.0	0.1	0	0	0
Thailand	Muang Sing Buri	0.1	0.1	0.2	0	0	1	0.0	0.0	0.1	0	0	0
Thailand	Muang Songkhla	0.1	0.1	0.2	1	1	2	0.0	0.0	0.1	0	0	1
Thailand	Muang Suang	0.1	0.1	0.2	0	0	0	0.0	0.0	0.1	0	0	0
Thailand	Muang Sukhothai	0.1	0.1	0.2	1	1	2	0.0	0.0	0.1	0	0	0
Thailand	Muang Suphanburi	0.1	0.1	0.2	1	1	2	0.0	0.0	0.1	0	0	0
Thailand	Muang Surat Thani	0.1	0.1	0.2	1	1	2	0.0	0.0	0.1	0	0	1
Thailand	Muang Surin	0.1	0.0	0.1	2	1	3	0.0	0.0	0.1	0	0	1
Thailand	Muang Tak	0.1	0.1	0.2	1	1	2	0.0	0.0	0.1	0	0	0
Thailand	Muang Trang	0.1	0.1	0.2	1	1	2	0.0	0.0	0.1	0	0	1
Thailand	Muang Trat	0.1	0.1	0.2	1	0	1	0.0	0.0	0.1	0	0	0
Thailand	Muang Ubon Ratchatani	0.1	0.1	0.2	2	1	3	0.0	0.0	0.1	0	0	1
Thailand	Muang Udon Thani	0.1	0.1	0.2	3	2	5	0.0	0.0	0.1	1	0	1
Thailand	Muang Uthai Thani	0.1	0.1	0.2	0	0	1	0.0	0.0	0.1	0	0	0
Thailand	Muang Uttaradit	0.1	0.1	0.2	1	1	2	0.0	0.0	0.1	0	0	0
Thailand	Muang Yala	0.1	0.0	0.2	1	1	2	0.0	0.0	0.1	0	0	1
Thailand	Mueang Kamphaeng Phet	0.1	0.1	0.2	2	1	4	0.0	0.0	0.1	1	0	1
Thailand	Mueang Pan	0.1	0.1	0.2	0	0	0	0.0	0.0	0.1	0	0	0
Thailand	Na Bon	0.1	0.0	0.1	0	0	0	0.0	0.0	0.1	0	0	0
Thailand	Na Chaluai	0.1	0.1	0.2	1	0	1	0.0	0.0	0.1	0	0	0
Thailand	Na Chuak	0.1	0.0	0.2	1	0	1	0.0	0.0	0.1	0	0	0
Thailand	Na Di	0.1	0.1	0.2	0	0	1	0.0	0.0	0.1	0	0	0
Thailand	Na Duang	0.1	0.1	0.2	0	0	0	0.0	0.0	0.1	0	0	0
Thailand	Na Dun	0.1	0.0	0.2	0	0	1	0.0	0.0	0.1	0	0	0
Thailand	Na Haeo	0.1	0.1	0.2	0	0	0	0.0	0.0	0.1	0	0	0
Thailand	Na Kae	0.1	0.1	0.2	1	0	1	0.0	0.0	0.1	0	0	0
Thailand	Na Klang	0.1	0.1	0.2	1	0	1	0.0	0.0	0.1	0	0	0
Thailand	Na Mom	0.1	0.1	0.2	0	0	0	0.0	0.0	0.1	0	0	0
Thailand	Na Mon	0.1	0.1	0.2	0	0	1	0.0	0.0	0.1	0	0	0
Thailand	Na Mun	0.1	0.1	0.2	0	0	0	0.0	0.0	0.1	0	0	0
Thailand	Na Noi	0.1	0.1	0.2	0	0	0	0.0	0.0	0.1	0	0	0
Thailand	Na Pho	0.1	0.1	0.2	0	0	0	0.0	0.0	0.1	0	0	0
Thailand	Na Thawi	0.1	0.0	0.2	0	0	1	0.0	0.0	0.1	0	0	0
Thailand	Na Thom	0.1	0.1	0.2	0	0	0	0.0	0.0	0.1	0	0	0
Thailand	Na Wa	0.1	0.1	0.2	0	0	1	0.0	0.0	0.1	0	0	0
Thailand	Na Wang	0.1	0.1	0.2	0	0	1	0.0	0.0	0.1	0	0	0
Thailand	Na Yong	0.1	0.1	0.2	0	0	1	0.0	0.0	0.1	0	0	0
Thailand	Na Yung	0.1	0.1	0.2	0	0	0	0.0	0.0	0.1	0	0	0
Thailand	Nakhon Chaisi	0.1	0.1	0.2	1	0	1	0.0	0.0	0.1	0	0	0
Thailand	Nakhon Luang	0.1	0.1	0.2	0	0	1	0.0	0.0	0.1	0	0	0
Thailand	Nakhon Thai	0.1	0.1	0.2	1	0	1	0.0	0.0	0.1	0	0	0
Thailand	Nam Kliang	0.1	0.1	0.2	0	0	1	0.0	0.0	0.1	0	0	0
Thailand	Nam Nao	0.1	0.1	0.2	0	0	0	0.0	0.0	0.1	0	0	0
Thailand	Nam Pat	0.1	0.1	0.2	0	0	1	0.0	0.0	0.1	0	0	0
Thailand	Nam Phong	0.1	0.1	0.2	1	1	2	0.0	0.0	0.1	0	0	0
Thailand	Nam Som	0.1	0.1	0.2	1	0	1	0.0	0.0	0.1	0	0	0
Thailand	Nam Yun	0.1	0.1	0.2	1	0	1	0.0	0.0	0.1	0	0	0
Thailand	Nang Rong	0.1	0.0	0.1	1	0	1	0.0	0.0	0.1	0	0	0
Thailand	Ngao	0.1	0.1	0.2	0	0	1	0.0	0.0	0.1	0	0	0
Thailand	Nikhom Kham Soi	0.1	0.1	0.2	0	0	1	0.0	0.0	0.1	0	0	0
Thailand	Nikhom Nam Un	0.1	0.1	0.2	0	0	0	0.0	0.0	0.1	0	0	0
Thailand	Noen Maprang	0.1	0.1	0.2	1	0	1	0.0	0.0	0.1	0	0	0
Thailand	Noen Sa-Nga	0.1	0.1	0.2	0	0	0	0.0	0.0	0.1	0	0	0

Table 3: Diarrhoea Mortality by Unit *(continued)*

Country	Unit	2000 mortality mean rate (per 10,000)	lower rate (per 10,000)	upper rate (per 10,000)	mean count	lower count	upper count	2017 mortality mean rate (per 10,000)	lower rate (per 10,000)	upper rate (per 10,000)	mean count	lower count	upper count
Thailand	Non Daeng	0.1	0.1	0.2	0	0	0	0.0	0.0	0.1	0	0	0
Thailand	Non Din Daeng	0.1	0.0	0.1	0	0	0	0.0	0.0	0.1	0	0	0
Thailand	Non Khun	0.1	0.1	0.2	0	0	1	0.0	0.0	0.1	0	0	0
Thailand	Non Sa-at	0.1	0.1	0.2	0	0	1	0.0	0.0	0.1	0	0	0
Thailand	Non Sang	0.1	0.1	0.2	1	0	1	0.0	0.0	0.1	0	0	0
Thailand	Non Sung	0.1	0.1	0.2	1	1	2	0.0	0.0	0.1	0	0	0
Thailand	Non Suwan	0.1	0.0	0.1	0	0	0	0.0	0.0	0.1	0	0	0
Thailand	Non Thai	0.1	0.1	0.2	1	0	1	0.0	0.0	0.1	0	0	0
Thailand	Nong Bua	0.1	0.1	0.2	1	0	1	0.0	0.0	0.1	0	0	0
Thailand	Nong Bua Daeng	0.1	0.1	0.2	1	1	2	0.0	0.0	0.1	0	0	0
Thailand	Nong Bua Rawae	0.1	0.1	0.2	0	0	1	0.0	0.0	0.1	0	0	0
Thailand	Nong Bunnak	0.1	0.1	0.2	1	0	1	0.0	0.0	0.1	0	0	0
Thailand	Nong Chang	0.1	0.1	0.2	0	0	1	0.0	0.0	0.1	0	0	0
Thailand	Nong Chik	0.1	0.0	0.2	1	0	2	0.0	0.0	0.1	0	0	0
Thailand	Nong Chok	0.1	0.1	0.2	1	1	2	0.0	0.0	0.1	0	0	1
Thailand	Nong Don	0.1	0.1	0.2	0	0	0	0.0	0.0	0.1	0	0	0
Thailand	Nong Han	0.1	0.1	0.2	1	1	2	0.0	0.0	0.1	0	0	0
Thailand	Nong Hong	0.1	0.1	0.1	0	0	1	0.0	0.0	0.1	0	0	0
Thailand	Nong Khae	0.1	0.1	0.2	1	0	1	0.0	0.0	0.1	0	0	0
Thailand	Nong Khayang	0.1	0.1	0.2	0	0	0	0.0	0.0	0.1	0	0	0
Thailand	Nong Ki	0.1	0.0	0.1	1	0	1	0.0	0.0	0.1	0	0	0
Thailand	Nong Kung Si	0.1	0.1	0.2	1	0	1	0.0	0.0	0.1	0	0	0
Thailand	Nong Muang	0.1	0.1	0.2	0	0	0	0.0	0.0	0.1	0	0	0
Thailand	Nong Muang Kai	0.1	0.1	0.2	0	0	0	0.0	0.0	0.1	0	0	0
Thailand	Nong Phai	0.1	0.1	0.2	1	1	2	0.0	0.0	0.1	0	0	0
Thailand	Nong Phok	0.1	0.1	0.2	1	0	1	0.0	0.0	0.1	0	0	0
Thailand	Nong Prue	0.1	0.1	0.2	0	0	0	0.0	0.0	0.1	0	0	0
Thailand	Nong Rua	0.1	0.1	0.2	1	0	1	0.0	0.0	0.1	0	0	0
Thailand	Nong Saeng	0.1	0.1	0.2	0	0	0	0.0	0.0	0.1	0	0	0
Thailand	Nong Saeng	0.1	0.1	0.2	0	0	0	0.0	0.0	0.1	0	0	0
Thailand	Nong Song Hong	0.1	0.1	0.2	1	0	1	0.0	0.0	0.1	0	0	0
Thailand	Nong Sua	0.1	0.1	0.2	0	0	1	0.0	0.0	0.1	0	0	0
Thailand	Nong Sung	0.1	0.1	0.2	0	0	0	0.0	0.0	0.1	0	0	0
Thailand	Nong Wua So	0.1	0.1	0.2	1	0	1	0.0	0.0	0.1	0	0	0
Thailand	Nong Ya Plong	0.1	0.1	0.2	0	0	0	0.0	0.0	0.1	0	0	0
Thailand	Nong Ya Sai	0.1	0.1	0.2	0	0	1	0.0	0.0	0.1	0	0	0
Thailand	Nong Yai	0.1	0.0	0.1	0	0	0	0.0	0.0	0.0	0	0	0
Thailand	Nongkheam	0.1	0.1	0.2	1	1	2	0.0	0.0	0.1	0	0	1
Thailand	Nua Khlong	0.1	0.0	0.1	0	0	1	0.0	0.0	0.1	0	0	0
Thailand	Omkoi	0.1	0.1	0.2	1	0	1	0.0	0.0	0.1	0	0	0
Thailand	Ongkharak	0.1	0.1	0.2	0	0	1	0.0	0.0	0.1	0	0	0
Thailand	Pa Bon	0.1	0.1	0.2	0	0	1	0.0	0.0	0.1	0	0	0
Thailand	Pa Daet	0.1	0.1	0.2	0	0	0	0.0	0.0	0.1	0	0	0
Thailand	Pa Kham	0.1	0.0	0.1	0	0	1	0.0	0.0	0.1	0	0	0
Thailand	Pa Mok	0.1	0.1	0.2	0	0	0	0.0	0.0	0.1	0	0	0
Thailand	Pa Payom	0.1	0.1	0.2	0	0	1	0.0	0.0	0.1	0	0	0
Thailand	Pa Sang	0.1	0.1	0.2	0	0	1	0.0	0.0	0.1	0	0	0
Thailand	Pa Tiu	0.1	0.1	0.2	0	0	1	0.0	0.0	0.1	0	0	0
Thailand	Pai	0.1	0.1	0.2	0	0	0	0.0	0.0	0.1	0	0	0
Thailand	Pak Chom	0.1	0.1	0.2	0	0	1	0.0	0.0	0.1	0	0	0
Thailand	Pak Chong	0.1	0.1	0.2	1	1	3	0.0	0.0	0.1	0	0	1
Thailand	Pak Khat	0.1	0.1	0.2	0	0	1	0.0	0.0	0.1	0	0	0
Thailand	Pak Kret	0.1	0.1	0.2	1	1	2	0.0	0.0	0.1	1	0	1
Thailand	Pak Phanang	0.1	0.0	0.2	1	0	1	0.0	0.0	0.1	0	0	0
Thailand	Pak Phayun	0.1	0.1	0.2	0	0	1	0.0	0.0	0.1	0	0	0
Thailand	Pak Phli	0.1	0.1	0.2	0	0	0	0.0	0.0	0.1	0	0	0
Thailand	Pak Tho	0.1	0.1	0.2	1	0	1	0.0	0.0	0.1	0	0	0
Thailand	Pak Thong Chai	0.1	0.1	0.2	1	1	2	0.0	0.0	0.1	0	0	0
Thailand	Palian	0.1	0.1	0.2	1	0	1	0.0	0.0	0.1	0	0	0
Thailand	Panare	0.1	0.0	0.2	0	0	1	0.0	0.0	0.1	0	0	0

Table 3: Diarrhoea Mortality by Unit *(continued)*

		2000 mortality						2017 mortality					
Country	Unit	mean rate (per 10,000)	lower rate (per 10,000)	upper rate (per 10,000)	mean count	lower count	upper count	mean rate (per 10,000)	lower rate (per 10,000)	upper rate (per 10,000)	mean count	lower count	upper count
Thailand	Pang Ma Pha	0.1	0.1	0.2	0	0	0	0.0	0.0	0.1	0	0	0
Thailand	Pang Sila Thong	0.1	0.1	0.2	0	0	1	0.0	0.0	0.1	0	0	0
Thailand	Panom Phrai	0.1	0.1	0.2	1	0	1	0.0	0.0	0.1	0	0	0
Thailand	Pathiu	0.1	0.0	0.1	0	0	1	0.0	0.0	0.1	0	0	0
Thailand	Pathum Rat	0.1	0.0	0.2	0	0	1	0.0	0.0	0.1	0	0	0
Thailand	Pathum Ratwongsa	0.1	0.1	0.2	0	0	1	0.0	0.0	0.1	0	0	0
Thailand	Pathum Wan	0.1	0.1	0.2	0	0	1	0.0	0.0	0.1	0	0	0
Thailand	Pha Khao	0.1	0.1	0.2	0	0	1	0.0	0.0	0.1	0	0	0
Thailand	Phachi	0.1	0.1	0.2	0	0	0	0.0	0.0	0.1	0	0	0
Thailand	Phaisali	0.1	0.1	0.2	1	0	1	0.0	0.0	0.1	0	0	0
Thailand	Phak Hai	0.1	0.1	0.2	0	0	1	0.0	0.0	0.1	0	0	0
Thailand	Phakdi Chumphol	0.1	0.1	0.2	0	0	0	0.0	0.0	0.1	0	0	0
Thailand	Phan	0.1	0.1	0.2	1	1	1	0.0	0.0	0.1	0	0	0
Thailand	Phan Thong	0.1	0.0	0.1	0	0	1	0.0	0.0	0.1	0	0	0
Thailand	Phana	0.1	0.1	0.2	0	0	0	0.0	0.0	0.1	0	0	0
Thailand	Phanat Nikhom	0.1	0.0	0.1	1	0	1	0.0	0.0	0.1	0	0	0
Thailand	Phang Khon	0.1	0.1	0.2	0	0	1	0.0	0.0	0.1	0	0	0
Thailand	Phanna Nikhom	0.1	0.1	0.2	1	0	1	0.0	0.0	0.1	0	0	0
Thailand	Phanom	0.1	0.1	0.2	0	0	0	0.0	0.0	0.1	0	0	0
Thailand	Phanom Sarakham	0.1	0.0	0.1	1	0	1	0.0	0.0	0.0	0	0	0
Thailand	Phanom Thuan	0.1	0.1	0.2	0	0	1	0.0	0.0	0.1	0	0	0
Thailand	Phasi Charoen	0.1	0.1	0.2	1	0	1	0.0	0.0	0.1	0	0	0
Thailand	Phato	0.1	0.1	0.2	0	0	0	0.0	0.0	0.1	0	0	0
Thailand	Phatthana Nikhom	0.1	0.1	0.2	0	0	1	0.0	0.0	0.1	0	0	0
Thailand	Phaya Mengrai	0.1	0.1	0.2	0	0	1	0.1	0.0	0.1	0	0	0
Thailand	Phaya Thai	0.1	0.1	0.2	0	0	1	0.0	0.0	0.1	0	0	0
Thailand	Phayakkhaphum Phisai	0.1	0.1	0.2	1	0	2	0.0	0.0	0.1	0	0	0
Thailand	Phayu	0.1	0.1	0.2	0	0	0	0.0	0.0	0.1	0	0	0
Thailand	Phayuha Khiri	0.1	0.1	0.2	1	0	1	0.0	0.0	0.1	0	0	0
Thailand	Phen	0.1	0.1	0.2	1	1	2	0.0	0.0	0.1	0	0	0
Thailand	Phi Pun	0.1	0.0	0.1	0	0	0	0.0	0.0	0.1	0	0	0
Thailand	Phibun Mangsahan	0.1	0.1	0.2	1	1	2	0.0	0.0	0.1	0	0	0
Thailand	Phibun Rak	0.1	0.1	0.2	0	0	0	0.0	0.0	0.1	0	0	0
Thailand	Phichai	0.1	0.1	0.2	1	0	1	0.0	0.0	0.1	0	0	0
Thailand	Phimai	0.1	0.1	0.2	1	1	2	0.0	0.0	0.1	0	0	0
Thailand	Phlapphlachai	0.1	0.0	0.1	0	0	1	0.0	0.0	0.0	0	0	0
Thailand	Pho Chai	0.1	0.1	0.2	0	0	1	0.0	0.0	0.1	0	0	0
Thailand	Pho Prathap Chan	0.1	0.1	0.2	0	0	1	0.0	0.0	0.1	0	0	0
Thailand	Pho Si	0.1	0.1	0.2	0	0	1	0.0	0.0	0.1	0	0	0
Thailand	Pho Thale	0.1	0.1	0.2	1	0	1	0.0	0.0	0.1	0	0	0
Thailand	Pho Thong	0.1	0.1	0.2	0	0	1	0.0	0.0	0.1	0	0	0
Thailand	Phon	0.1	0.1	0.2	1	0	1	0.0	0.0	0.1	0	0	0
Thailand	Phon Charoen	0.1	0.1	0.2	0	0	1	0.0	0.0	0.1	0	0	0
Thailand	Phon Na Kaeo	0.1	0.1	0.2	0	0	1	0.0	0.0	0.1	0	0	0
Thailand	Phon Phisai	0.1	0.1	0.2	1	1	2	0.0	0.0	0.1	0	0	0
Thailand	Phon Sai	0.1	0.1	0.2	0	0	0	0.0	0.0	0.1	0	0	0
Thailand	Phon Sawan	0.1	0.1	0.2	1	0	1	0.0	0.0	0.1	0	0	0
Thailand	Phon Thong	0.1	0.1	0.2	1	1	2	0.0	0.0	0.1	0	0	0
Thailand	Phop Phra	0.1	0.1	0.2	1	0	1	0.0	0.0	0.1	0	0	0
Thailand	Photharam	0.1	0.1	0.2	1	1	2	0.0	0.0	0.1	0	0	0
Thailand	Phra Nakhon	0.1	0.1	0.2	0	0	0	0.0	0.0	0.1	0	0	0
Thailand	Phra Nakhon Si Ayutthaya	0.1	0.1	0.2	1	1	2	0.0	0.0	0.1	0	0	0

Table 3: Diarrhoea Mortality by Unit *(continued)*

Country	Unit	2000 mortality mean rate (per 10,000)	lower rate (per 10,000)	upper rate (per 10,000)	mean count	lower count	upper count	2017 mortality mean rate (per 10,000)	lower rate (per 10,000)	upper rate (per 10,000)	mean count	lower count	upper count
Thailand	Phra Phrom	0.1	0.0	0.1	0	0	1	0.0	0.0	0.1	0	0	0
Thailand	Phra Phutthabat	0.1	0.1	0.2	1	0	1	0.0	0.0	0.1	0	0	0
Thailand	Phra Pra Daeng	0.1	0.1	0.2	1	1	2	0.0	0.0	0.1	1	0	1
Thailand	Phra Samut Jadee	0.1	0.1	0.2	1	0	1	0.0	0.0	0.1	0	0	1
Thailand	Phra Yun	0.1	0.1	0.2	0	0	1	0.0	0.0	0.1	0	0	0
Thailand	Phrai Bung	0.1	0.1	0.1	0	0	1	0.0	0.0	0.1	0	0	0
Thailand	Phran Kratai	0.1	0.1	0.2	1	0	1	0.0	0.0	0.1	0	0	0
Thailand	Phrao	0.1	0.1	0.2	0	0	1	0.0	0.0	0.1	0	0	0
Thailand	Phrasat	0.1	0.0	0.1	1	1	2	0.0	0.0	0.0	0	0	0
Thailand	Phrom Buri	0.1	0.1	0.2	0	0	0	0.0	0.0	0.1	0	0	0
Thailand	Phrom Phiram	0.1	0.1	0.2	1	0	1	0.0	0.0	0.1	0	0	0
Thailand	Phrommakhiri	0.1	0.0	0.1	0	0	0	0.0	0.0	0.1	0	0	0
Thailand	Phu Khieo	0.1	0.1	0.2	1	1	2	0.0	0.0	0.1	0	0	0
Thailand	Phu Kradung	0.1	0.1	0.2	0	0	1	0.0	0.0	0.1	0	0	0
Thailand	Phu Luang	0.1	0.1	0.2	0	0	0	0.0	0.0	0.1	0	0	0
Thailand	Phu Phan	0.1	0.1	0.2	0	0	1	0.0	0.0	0.1	0	0	0
Thailand	Phu Rua	0.1	0.1	0.2	0	0	0	0.0	0.0	0.1	0	0	0
Thailand	Phu Sing	0.1	0.1	0.1	0	0	1	0.0	0.0	0.1	0	0	0
Thailand	Phu Wiang	0.1	0.1	0.2	1	0	1	0.0	0.0	0.1	0	0	0
Thailand	Phunphin	0.1	0.1	0.2	1	0	1	0.0	0.0	0.1	0	0	0
Thailand	Phupa Man	0.1	0.1	0.2	0	0	0	0.0	0.0	0.1	0	0	0
Thailand	Phuttha Mon Thon	0.1	0.1	0.2	0	0	0	0.0	0.0	0.1	0	0	0
Thailand	Phutthaisong	0.1	0.1	0.2	0	0	1	0.0	0.0	0.1	0	0	0
Thailand	Pla Pak	0.1	0.1	0.2	1	0	1	0.0	0.0	0.1	0	0	0
Thailand	Plaeng Yao	0.1	0.0	0.1	0	0	0	0.0	0.0	0.0	0	0	0
Thailand	Plai Phraya	0.1	0.1	0.2	0	0	0	0.0	0.0	0.1	0	0	0
Thailand	Pluak Daeng	0.1	0.0	0.1	0	0	0	0.0	0.0	0.0	0	0	0
Thailand	Pom Pram Sattru	0.1	0.1	0.2	0	0	0	0.0	0.0	0.1	0	0	0
Thailand	Pong	0.1	0.1	0.2	1	0	1	0.0	0.0	0.1	0	0	0
Thailand	Pong Nam Ron	0.1	0.1	0.1	0	0	0	0.0	0.0	0.1	0	0	0
Thailand	Pra Thai	0.1	0.1	0.2	1	0	1	0.0	0.0	0.1	0	0	0
Thailand	Prachantakham	0.1	0.1	0.2	0	0	1	0.0	0.0	0.1	0	0	0
Thailand	Prakanong	0.1	0.1	0.2	1	0	1	0.0	0.0	0.1	0	0	0
Thailand	Prakhon Chai	0.1	0.0	0.1	1	1	2	0.0	0.0	0.1	0	0	0
Thailand	Pran Buri	0.1	0.1	0.2	1	0	1	0.0	0.0	0.1	0	0	0
Thailand	Prang Ku	0.1	0.1	0.1	1	0	1	0.0	0.0	0.1	0	0	0
Thailand	Prasaeng	0.1	0.1	0.2	1	0	1	0.0	0.0	0.1	0	0	0
Thailand	Prawet	0.1	0.1	0.2	1	1	2	0.0	0.0	0.1	0	0	1
Thailand	Pua	0.1	0.1	0.2	1	0	1	0.1	0.0	0.1	0	0	0
Thailand	Puai Noi	0.1	0.1	0.2	0	0	0	0.0	0.0	0.1	0	0	0
Thailand	Raman	0.1	0.0	0.2	1	0	1	0.0	0.0	0.1	0	0	0
Thailand	Rangae	0.1	0.0	0.2	1	0	1	0.0	0.0	0.1	0	0	0
Thailand	Ranot	0.1	0.1	0.2	1	0	1	0.0	0.0	0.1	0	0	0
Thailand	Rasada	0.1	0.1	0.2	0	0	0	0.0	0.0	0.1	0	0	0
Thailand	Rasi Salai	0.1	0.1	0.2	1	0	1	0.0	0.0	0.1	0	0	0
Thailand	Rat Burana	0.1	0.1	0.2	1	0	1	0.0	0.0	0.1	0	0	0
Thailand	Ratchasan	0.1	0.1	0.1	0	0	0	0.0	0.0	0.1	0	0	0
Thailand	Ratchathewi	0.1	0.1	0.2	0	0	1	0.0	0.0	0.1	0	0	0
Thailand	Rattana Buri	0.1	0.1	0.2	1	1	2	0.0	0.0	0.1	0	0	0
Thailand	Rattaphum	0.1	0.1	0.2	1	0	1	0.0	0.0	0.1	0	0	0
Thailand	Renu Nakhon	0.1	0.1	0.2	0	0	1	0.0	0.0	0.1	0	0	0
Thailand	Ron Phi Pun	0.1	0.1	0.2	1	0	1	0.0	0.0	0.1	0	0	0
Thailand	Rong Kham	0.1	0.1	0.2	0	0	0	0.0	0.0	0.1	0	0	0
Thailand	Rong Kwang	0.1	0.1	0.2	0	0	1	0.0	0.0	0.1	0	0	0
Thailand	Ruso	0.1	0.0	0.2	1	0	1	0.0	0.0	0.1	0	0	0
Thailand	Saba Yoi	0.1	0.1	0.2	1	0	1	0.0	0.0	0.1	0	0	0
Thailand	Sadao	0.1	0.0	0.2	1	0	2	0.0	0.0	0.1	0	0	0
Thailand	Sahatsakhan	0.1	0.1	0.2	0	0	1	0.0	0.0	0.1	0	0	0

Table 3: Diarrhoea Mortality by Unit *(continued)*

Country	Unit	2000 mortality						2017 mortality					
		mean rate (per 10,000)	lower rate (per 10,000)	upper rate (per 10,000)	mean count	lower count	upper count	mean rate (per 10,000)	lower rate (per 10,000)	upper rate (per 10,000)	mean count	lower count	upper count
Thailand	Sai Buri	0.1	0.0	0.2	0	0	1	0.0	0.0	0.1	0	0	0
Thailand	Sai Mai	0.1	0.1	0.2	1	0	1	0.0	0.0	0.1	0	0	1
Thailand	Sai Ngam	0.1	0.1	0.2	0	0	1	0.0	0.0	0.1	0	0	0
Thailand	Sai Noi	0.1	0.1	0.2	0	0	0	0.0	0.0	0.1	0	0	0
Thailand	Sai Thong Watthana	0.1	0.1	0.2	0	0	0	0.0	0.0	0.1	0	0	0
Thailand	Sai Yok	0.1	0.1	0.2	0	0	1	0.0	0.0	0.1	0	0	0
Thailand	Sam Chuk	0.1	0.1	0.2	0	0	1	0.0	0.0	0.1	0	0	0
Thailand	Sam Khok	0.1	0.1	0.2	0	0	1	0.0	0.0	0.1	0	0	0
Thailand	Sam Ngam	0.1	0.1	0.2	0	0	1	0.0	0.0	0.1	0	0	0
Thailand	Sam Ngao	0.1	0.1	0.2	0	0	1	0.0	0.0	0.1	0	0	0
Thailand	Sam Phran	0.1	0.1	0.2	1	1	2	0.0	0.0	0.1	0	0	1
Thailand	Samko	0.1	0.1	0.2	0	0	0	0.0	0.0	0.1	0	0	0
Thailand	Samoeng	0.1	0.1	0.2	0	0	0	0.0	0.0	0.1	0	0	0
Thailand	Samphantawong	0.1	0.1	0.2	0	0	0	0.0	0.0	0.1	0	0	0
Thailand	Samrong	0.1	0.1	0.2	0	0	1	0.0	0.0	0.1	0	0	0
Thailand	Samrong Thap	0.1	0.1	0.1	1	0	1	0.0	0.0	0.1	0	0	0
Thailand	San Kamphaeng	0.1	0.1	0.2	0	0	1	0.0	0.0	0.1	0	0	0
Thailand	San Pa Tong	0.1	0.1	0.2	0	0	1	0.0	0.0	0.1	0	0	0
Thailand	San Sai	0.1	0.1	0.2	1	0	1	0.0	0.0	0.1	0	0	0
Thailand	Sanam Chaikhet	0.1	0.0	0.1	1	0	1	0.0	0.0	0.0	0	0	0
Thailand	Sang Khom	0.1	0.1	0.2	0	0	0	0.0	0.0	0.1	0	0	0
Thailand	Sang Khom	0.1	0.1	0.2	0	0	0	0.0	0.0	0.1	0	0	0
Thailand	Sangkha	0.1	0.0	0.1	1	1	2	0.0	0.0	0.1	0	0	0
Thailand	Sangkhla Buri	0.1	0.1	0.2	0	0	1	0.0	0.0	0.1	0	0	0
Thailand	Sankha Buri	0.1	0.1	0.2	1	0	1	0.0	0.0	0.1	0	0	0
Thailand	Sanom	0.1	0.1	0.2	0	0	1	0.0	0.0	0.1	0	0	0
Thailand	Sanphaya	0.1	0.1	0.2	0	0	1	0.0	0.0	0.1	0	0	0
Thailand	Santi Suk	0.1	0.1	0.2	0	0	0	0.1	0.0	0.1	0	0	0
Thailand	Sao Hai	0.1	0.1	0.2	0	0	0	0.0	0.0	0.1	0	0	0
Thailand	Saphan Sung	0.1	0.1	0.2	1	0	1	0.0	0.0	0.1	0	0	0
Thailand	Saraphi	0.1	0.1	0.2	0	0	1	0.0	0.0	0.1	0	0	0
Thailand	Sathing Phra	0.1	0.1	0.2	0	0	1	0.0	0.0	0.1	0	0	0
Thailand	Sathorn	0.1	0.1	0.2	0	0	1	0.0	0.0	0.1	0	0	0
Thailand	Sattahip	0.1	0.0	0.1	0	0	1	0.0	0.0	0.1	0	0	0
Thailand	Satuk	0.1	0.1	0.2	1	1	2	0.0	0.0	0.1	0	0	0
Thailand	Sawaengha	0.1	0.1	0.2	0	0	0	0.0	0.0	0.1	0	0	0
Thailand	Sawang Arom	0.1	0.1	0.2	0	0	0	0.0	0.0	0.1	0	0	0
Thailand	Sawang Daen Din	0.1	0.1	0.2	1	1	2	0.0	0.0	0.1	0	0	0
Thailand	Sawankhalok	0.1	0.1	0.2	1	0	1	0.0	0.0	0.1	0	0	0
Thailand	Sawi	0.1	0.1	0.2	1	0	1	0.0	0.0	0.1	0	0	0
Thailand	Seka	0.1	0.1	0.2	1	0	1	0.0	0.0	0.1	0	0	0
Thailand	Selaphum	0.1	0.1	0.2	1	1	2	0.0	0.0	0.1	0	0	0
Thailand	Sena	0.1	0.1	0.2	1	0	1	0.0	0.0	0.1	0	0	0
Thailand	Senangkhanikhom	0.1	0.1	0.2	0	0	1	0.0	0.0	0.1	0	0	0
Thailand	Si Banphot	0.1	0.1	0.2	0	0	0	0.0	0.0	0.1	0	0	0
Thailand	Si Bun Ruang	0.1	0.1	0.2	1	1	2	0.0	0.0	0.1	0	0	0
Thailand	Si Chiang Mai	0.1	0.1	0.2	0	0	1	0.0	0.0	0.1	0	0	0
Thailand	Si Chomphu	0.1	0.1	0.2	1	0	1	0.0	0.0	0.1	0	0	0
Thailand	Si Mahosot	0.1	0.0	0.1	0	0	0	0.0	0.0	0.0	0	0	0
Thailand	Si Muang Mai	0.1	0.1	0.2	1	0	1	0.0	0.0	0.1	0	0	0
Thailand	Si Nakhon	0.1	0.1	0.2	0	0	0	0.0	0.0	0.1	0	0	0
Thailand	Si Prachan	0.1	0.1	0.2	0	0	1	0.0	0.0	0.1	0	0	0
Thailand	Si Racha	0.1	0.0	0.1	1	1	2	0.0	0.0	0.0	0	0	1
Thailand	Si Rin Ton	0.1	0.1	0.2	1	0	1	0.0	0.0	0.1	0	0	0
Thailand	Si Sakhon	0.1	0.0	0.2	0	0	1	0.0	0.0	0.1	0	0	0
Thailand	Si Sam Rong	0.1	0.1	0.2	1	0	1	0.0	0.0	0.1	0	0	0
Thailand	Si Satchanalai	0.1	0.1	0.2	1	1	2	0.0	0.0	0.1	0	0	0
Thailand	Si Sawat	0.1	0.1	0.2	0	0	0	0.0	0.0	0.1	0	0	0
Thailand	Si Somdet	0.1	0.1	0.2	0	0	1	0.0	0.0	0.1	0	0	0

Table 3: Diarrhoea Mortality by Unit *(continued)*

Country	Unit	2000 mortality mean rate (per 10,000)	lower rate (per 10,000)	upper rate (per 10,000)	mean count	lower count	upper count	2017 mortality mean rate (per 10,000)	lower rate (per 10,000)	upper rate (per 10,000)	mean count	lower count	upper count
Thailand	Si Songkhram	0.1	0.1	0.2	1	0	1	0.0	0.0	0.1	0	0	0
Thailand	Si That	0.1	0.1	0.2	0	0	1	0.0	0.0	0.1	0	0	0
Thailand	Si Thep	0.1	0.1	0.2	1	0	1	0.0	0.0	0.1	0	0	0
Thailand	Si Wilai	0.1	0.1	0.2	0	0	1	0.0	0.0	0.1	0	0	0
Thailand	Sichon	0.1	0.0	0.1	1	0	1	0.0	0.0	0.1	0	0	0
Thailand	Sikao	0.1	0.1	0.2	0	0	1	0.0	0.0	0.1	0	0	0
Thailand	Sikhiu	0.1	0.1	0.2	1	1	2	0.0	0.0	0.1	0	0	0
Thailand	Sikhoraphum	0.1	0.0	0.1	1	1	2	0.0	0.0	0.1	0	0	0
Thailand	Singha Nakhon	0.1	0.1	0.2	1	0	1	0.0	0.0	0.1	0	0	0
Thailand	So Phisai	0.1	0.1	0.2	1	0	1	0.0	0.0	0.1	0	0	0
Thailand	Soem Ngam	0.1	0.1	0.2	0	0	0	0.0	0.0	0.1	0	0	0
Thailand	Soeng Sang	0.1	0.0	0.1	1	0	1	0.0	0.0	0.1	0	0	0
Thailand	Somdet	0.1	0.1	0.2	0	0	1	0.0	0.0	0.1	0	0	0
Thailand	Song	0.1	0.1	0.2	0	0	1	0.0	0.0	0.1	0	0	0
Thailand	Song Dao	0.1	0.1	0.2	0	0	1	0.0	0.0	0.1	0	0	0
Thailand	Song Kwae	0.1	0.1	0.2	0	0	0	0.1	0.0	0.1	0	0	0
Thailand	Song Phi Nong	0.1	0.1	0.2	1	1	2	0.0	0.0	0.1	0	0	0
Thailand	Songkhla Lake	0.1	0.1	0.2	0	0	0	0.0	0.0	0.1	0	0	0
Thailand	Songkhla Lake	0.1	0.1	0.2	0	0	0	0.0	0.0	0.1	0	0	0
Thailand	Sop Moei	0.1	0.1	0.2	1	0	1	0.0	0.0	0.1	0	0	0
Thailand	Sop Prap	0.1	0.1	0.2	0	0	0	0.0	0.0	0.1	0	0	0
Thailand	Soydow	0.1	0.1	0.1	0	0	1	0.1	0.0	0.1	0	0	0
Thailand	Sra Both	0.1	0.1	0.2	0	0	0	0.0	0.0	0.1	0	0	0
Thailand	Sri Mahar Pho	0.1	0.0	0.1	0	0	1	0.0	0.0	0.0	0	0	0
Thailand	Sri Ratana	0.1	0.1	0.1	0	0	1	0.0	0.0	0.1	0	0	0
Thailand	Su-ngai Ko Lok	0.1	0.1	0.2	1	0	1	0.0	0.0	0.1	0	0	0
Thailand	Suan Luang	0.1	0.1	0.2	1	1	2	0.0	0.0	0.1	0	0	1
Thailand	Suan Phung	0.1	0.1	0.2	0	0	1	0.0	0.0	0.1	0	0	0
Thailand	Sukhirin	0.1	0.1	0.2	0	0	0	0.0	0.0	0.1	0	0	0
Thailand	Sung Men	0.1	0.1	0.2	0	0	1	0.0	0.0	0.1	0	0	0
Thailand	Sung Noen	0.1	0.1	0.2	1	0	1	0.0	0.0	0.1	0	0	0
Thailand	Sungai Padi	0.1	0.0	0.2	1	0	1	0.0	0.0	0.1	0	0	0
Thailand	Suwan Khuha	0.1	0.1	0.2	1	0	1	0.0	0.0	0.1	0	0	0
Thailand	Suwannaphum	0.1	0.1	0.2	1	1	2	0.0	0.0	0.1	0	0	0
Thailand	Ta Phraya	0.1	0.1	0.2	0	0	1	0.0	0.0	0.1	0	0	0
Thailand	Tak Bai	0.1	0.0	0.2	1	0	1	0.0	0.0	0.1	0	0	0
Thailand	Tak Fa	0.1	0.1	0.2	0	0	1	0.0	0.0	0.1	0	0	0
Thailand	Takhli	0.1	0.1	0.2	1	1	2	0.0	0.0	0.1	0	0	0
Thailand	Takua Pa	0.1	0.1	0.2	0	0	1	0.0	0.0	0.1	0	0	0
Thailand	Takua Thung	0.1	0.0	0.2	0	0	0	0.0	0.0	0.1	0	0	0
Thailand	Taling Chan	0.1	0.1	0.2	1	0	1	0.0	0.0	0.1	0	0	0
Thailand	Tamot	0.1	0.1	0.2	0	0	0	0.0	0.0	0.1	0	0	0
Thailand	Tan Sum	0.1	0.1	0.2	0	0	0	0.0	0.0	0.1	0	0	0
Thailand	Tao Ngoi	0.1	0.1	0.2	0	0	0	0.0	0.0	0.1	0	0	0
Thailand	Taphan Hin	0.1	0.1	0.2	1	0	1	0.0	0.0	0.1	0	0	0
Thailand	Tha Bo	0.1	0.1	0.2	1	1	2	0.0	0.0	0.1	0	0	0
Thailand	Tha Chana	0.1	0.1	0.2	0	0	1	0.0	0.0	0.1	0	0	0
Thailand	Tha Chang	0.1	0.1	0.2	0	0	0	0.0	0.0	0.1	0	0	0
Thailand	Tha Chang	0.1	0.1	0.2	0	0	0	0.0	0.0	0.1	0	0	0
Thailand	Tha Khantho	0.1	0.1	0.2	0	0	1	0.0	0.0	0.1	0	0	0
Thailand	Tha Li	0.1	0.1	0.2	0	0	0	0.0	0.0	0.1	0	0	0
Thailand	Tha Luang	0.1	0.1	0.2	0	0	0	0.0	0.0	0.1	0	0	0
Thailand	Tha Mai	0.1	0.1	0.1	0	0	1	0.0	0.0	0.1	0	0	0
Thailand	Tha Maka	0.1	0.1	0.2	1	1	2	0.0	0.0	0.1	0	0	0
Thailand	Tha Muang	0.1	0.1	0.2	1	0	1	0.0	0.0	0.1	0	0	0
Thailand	Tha Phae	0.1	0.0	0.2	0	0	0	0.0	0.0	0.1	0	0	0
Thailand	Tha Pla	0.1	0.1	0.2	0	0	1	0.0	0.0	0.1	0	0	0
Thailand	Tha Rua	0.1	0.1	0.2	0	0	1	0.0	0.0	0.1	0	0	0
Thailand	Tha Sae	0.1	0.1	0.2	1	0	1	0.0	0.0	0.1	0	0	0
Thailand	Tha Sala	0.1	0.0	0.1	1	0	1	0.0	0.0	0.1	0	0	0

Table 3: Diarrhoea Mortality by Unit *(continued)*

Country	Unit	2000 mortality mean rate (per 10,000)	lower rate (per 10,000)	upper rate (per 10,000)	mean count	lower count	upper count	2017 mortality mean rate (per 10,000)	lower rate (per 10,000)	upper rate (per 10,000)	mean count	lower count	upper count
Thailand	Tha Song Yang	0.1	0.1	0.2	1	1	2	0.0	0.0	0.1	0	0	0
Thailand	Tha Ta Kieb	0.1	0.0	0.1	0	0	1	0.0	0.0	0.0	0	0	0
Thailand	Tha Tako	0.1	0.1	0.2	1	0	1	0.0	0.0	0.1	0	0	0
Thailand	Tha Tum	0.1	0.1	0.2	1	1	2	0.0	0.0	0.1	0	0	0
Thailand	Tha Uthen	0.1	0.1	0.2	1	0	1	0.0	0.0	0.1	0	0	0
Thailand	Tha Wang Pha	0.1	0.1	0.2	1	0	1	0.1	0.0	0.1	0	0	0
Thailand	Tha Wung	0.1	0.1	0.2	0	0	1	0.0	0.0	0.1	0	0	0
Thailand	Tha Yang	0.1	0.1	0.2	1	0	1	0.0	0.0	0.1	0	0	0
Thailand	Thai Charoen	0.1	0.1	0.2	0	0	0	0.0	0.0	0.1	0	0	0
Thailand	Thai Muang	0.1	0.0	0.2	0	0	1	0.0	0.0	0.1	0	0	0
Thailand	Thalang	0.1	0.0	0.1	0	0	1	0.0	0.0	0.1	0	0	1
Thailand	Tham Phannara	0.1	0.0	0.1	0	0	0	0.0	0.0	0.1	0	0	0
Thailand	Than To	0.1	0.1	0.2	0	0	0	0.0	0.0	0.1	0	0	0
Thailand	Thanyaburi	0.1	0.1	0.2	1	0	1	0.0	0.0	0.1	0	0	1
Thailand	Thap Khlo	0.1	0.1	0.2	0	0	1	0.0	0.0	0.1	0	0	0
Thailand	Thap Put	0.1	0.1	0.2	0	0	0	0.0	0.0	0.1	0	0	0
Thailand	Thap Sakae	0.1	0.0	0.1	0	0	1	0.0	0.0	0.1	0	0	0
Thailand	Thap Than	0.1	0.1	0.2	0	0	0	0.0	0.0	0.1	0	0	0
Thailand	That Phanom	0.1	0.1	0.2	1	0	1	0.0	0.0	0.1	0	0	0
Thailand	Thawatchaburi	0.1	0.1	0.2	1	0	1	0.0	0.0	0.1	0	0	0
Thailand	Thawi Wattana	0.1	0.1	0.2	1	0	1	0.0	0.0	0.1	0	0	0
Thailand	Thep Sathit	0.1	0.1	0.2	1	0	1	0.0	0.0	0.1	0	0	0
Thailand	Thepha	0.1	0.0	0.2	1	0	1	0.0	0.0	0.1	0	0	0
Thailand	Thoen	0.1	0.1	0.2	1	0	1	0.0	0.0	0.1	0	0	0
Thailand	Thoeng	0.1	0.1	0.2	1	0	1	0.1	0.0	0.1	0	0	0
Thailand	Thon Buri	0.1	0.1	0.2	0	0	1	0.0	0.0	0.1	0	0	0
Thailand	Thong Pha Phum	0.1	0.1	0.2	0	0	1	0.0	0.0	0.1	0	0	0
Thailand	Thong Saen Khan	0.1	0.1	0.2	0	0	1	0.0	0.0	0.1	0	0	0
Thailand	Thung Chang	0.1	0.1	0.2	0	0	0	0.1	0.0	0.1	0	0	0
Thailand	Thung Fon	0.1	0.1	0.2	0	0	0	0.0	0.0	0.1	0	0	0
Thailand	Thung Hua Chang	0.1	0.1	0.2	0	0	0	0.0	0.0	0.1	0	0	0
Thailand	Thung Saliam	0.1	0.1	0.2	0	0	1	0.0	0.0	0.1	0	0	0
Thailand	Thung Si Udom	0.1	0.1	0.2	0	0	0	0.0	0.0	0.1	0	0	0
Thailand	Thung Song	0.1	0.1	0.2	1	1	2	0.0	0.0	0.1	0	0	1
Thailand	Thung Tako	0.1	0.1	0.2	0	0	0	0.0	0.0	0.1	0	0	0
Thailand	Thung Wa	0.1	0.1	0.2	0	0	0	0.0	0.0	0.1	0	0	0
Thailand	Thung Yai	0.1	0.0	0.1	1	0	1	0.0	0.0	0.1	0	0	0
Thailand	Thung Yang Daeng	0.1	0.0	0.2	0	0	0	0.0	0.0	0.1	0	0	0
Thailand	Thungkru	0.1	0.1	0.2	1	0	1	0.0	0.0	0.1	0	0	0
Thailand	Trakan Phutphon	0.1	0.1	0.2	1	1	2	0.0	0.0	0.1	0	0	0
Thailand	Tron	0.1	0.1	0.2	0	0	1	0.0	0.0	0.1	0	0	0
Thailand	U Thong	0.1	0.1	0.2	1	1	2	0.0	0.0	0.1	0	0	0
Thailand	Ubol Ratana	0.1	0.1	0.2	0	0	1	0.0	0.0	0.1	0	0	0
Thailand	Umphang	0.1	0.1	0.2	0	0	1	0.0	0.0	0.1	0	0	0
Thailand	Uthai	0.1	0.1	0.2	0	0	1	0.0	0.0	0.1	0	0	0
Thailand	Uthumphon Phisai	0.1	0.1	0.2	1	0	1	0.0	0.0	0.1	0	0	0
Thailand	Wachira Barami	0.1	0.1	0.2	0	0	1	0.0	0.0	0.1	0	0	0
Thailand	Waeng	0.1	0.1	0.2	0	0	1	0.0	0.0	0.1	0	0	0
Thailand	Waeng Noi	0.1	0.1	0.2	0	0	1	0.0	0.0	0.1	0	0	0
Thailand	Waeng Yai	0.1	0.1	0.2	0	0	0	0.0	0.0	0.1	0	0	0
Thailand	Wan Yai	0.1	0.1	0.2	0	0	0	0.0	0.0	0.1	0	0	0
Thailand	Wang Chan	0.1	0.0	0.1	0	0	0	0.0	0.0	0.1	0	0	0
Thailand	Wang Chin	0.1	0.1	0.2	0	0	1	0.0	0.0	0.1	0	0	0
Thailand	Wang Hin	0.1	0.1	0.2	0	0	1	0.0	0.0	0.1	0	0	0
Thailand	Wang Muang	0.1	0.1	0.2	0	0	0	0.0	0.0	0.1	0	0	0
Thailand	Wang Nam Yen	0.1	0.0	0.1	0	0	1	0.0	0.0	0.1	0	0	0
Thailand	Wang Noi	0.1	0.1	0.2	1	0	1	0.0	0.0	0.1	0	0	0

Table 3: Diarrhoea Mortality by Unit *(continued)*

Country	Unit	2000 mortality mean rate (per 10,000)	2000 mortality lower rate (per 10,000)	2000 mortality upper rate (per 10,000)	2000 mortality mean count	2000 mortality lower count	2000 mortality upper count	2017 mortality mean rate (per 10,000)	2017 mortality lower rate (per 10,000)	2017 mortality upper rate (per 10,000)	2017 mortality mean count	2017 mortality lower count	2017 mortality upper count
Thailand	Wang Nua	0.1	0.1	0.2	0	0	1	0.0	0.0	0.1	0	0	0
Thailand	Wang Num Khiaw	0.1	0.1	0.2	0	0	1	0.0	0.0	0.1	0	0	0
Thailand	Wang Pong	0.1	0.1	0.2	0	0	1	0.0	0.0	0.1	0	0	0
Thailand	Wang Sai Phun	0.1	0.1	0.2	0	0	0	0.0	0.0	0.1	0	0	0
Thailand	Wang Sam Mo	0.1	0.1	0.2	1	0	1	0.0	0.0	0.1	0	0	0
Thailand	Wang Saphung	0.1	0.1	0.2	1	0	1	0.0	0.0	0.1	0	0	0
Thailand	Wang Thong	0.1	0.1	0.2	1	1	2	0.0	0.0	0.1	0	0	0
Thailand	Wang Thonglang	0.1	0.1	0.2	1	0	1	0.0	0.0	0.1	0	0	0
Thailand	Wang Wiset	0.1	0.1	0.2	0	0	1	0.0	0.0	0.1	0	0	0
Thailand	Wanon Niwat	0.1	0.1	0.2	1	1	2	0.0	0.0	0.1	0	0	0
Thailand	Wapi Pathum	0.1	0.0	0.2	1	0	2	0.0	0.0	0.1	0	0	0
Thailand	Warin Chamrap	0.1	0.1	0.2	1	1	2	0.0	0.0	0.1	0	0	1
Thailand	Waritchaphum	0.1	0.1	0.2	0	0	1	0.0	0.0	0.1	0	0	0
Thailand	Wat Bot	0.1	0.1	0.2	0	0	1	0.0	0.0	0.1	0	0	0
Thailand	Wat Phleng	0.1	0.1	0.2	0	0	0	0.0	0.0	0.1	0	0	0
Thailand	Wat Sing	0.1	0.1	0.2	0	0	0	0.0	0.0	0.1	0	0	0
Thailand	Wattana	0.1	0.1	0.2	1	0	1	0.0	0.0	0.1	0	0	0
Thailand	Watthana Nakhon	0.1	0.0	0.1	1	0	1	0.0	0.0	0.1	0	0	0
Thailand	Wiang Chai	0.1	0.1	0.2	0	0	1	0.0	0.0	0.1	0	0	0
Thailand	Wiang Haeng	0.1	0.1	0.2	0	0	0	0.0	0.0	0.1	0	0	0
Thailand	Wiang Kao	0.1	0.1	0.2	0	0	0	0.0	0.0	0.1	0	0	0
Thailand	Wiang Pa Pao	0.1	0.1	0.2	1	0	1	0.0	0.0	0.1	0	0	0
Thailand	Wiang Sa	0.1	0.1	0.2	1	0	1	0.0	0.0	0.1	0	0	0
Thailand	Wiang Sa	0.1	0.1	0.2	0	0	1	0.0	0.0	0.1	0	0	0
Thailand	Wichian Buri	0.1	0.1	0.2	1	1	2	0.0	0.0	0.1	0	0	0
Thailand	Wieng Kaen	0.1	0.1	0.2	1	0	1	0.1	0.0	0.1	0	0	0
Thailand	Wihan Daeng	0.1	0.1	0.2	0	0	0	0.0	0.0	0.1	0	0	0
Thailand	Wiset Chai Chan	0.1	0.1	0.2	1	0	1	0.0	0.0	0.1	0	0	0
Thailand	Yaha	0.1	0.0	0.2	1	0	1	0.0	0.0	0.1	0	0	0
Thailand	Yan Ta Khao	0.1	0.1	0.2	1	0	1	0.0	0.0	0.1	0	0	0
Thailand	Yang Chum Noi	0.1	0.1	0.2	0	0	0	0.0	0.0	0.1	0	0	0
Thailand	Yang Si Surat	0.1	0.0	0.2	0	0	1	0.0	0.0	0.1	0	0	0
Thailand	Yang Talat	0.1	0.1	0.2	1	1	2	0.0	0.0	0.1	0	0	0
Thailand	Yannawa	0.1	0.1	0.2	1	0	1	0.0	0.0	0.1	0	0	0
Thailand	Yarang	0.1	0.0	0.2	1	0	1	0.0	0.0	0.1	0	0	0
Thailand	Yaring	0.1	0.0	0.2	1	0	1	0.0	0.0	0.1	0	0	0
Thailand	Yi-ngo	0.1	0.0	0.2	0	0	1	0.0	0.0	0.1	0	0	0
Timor-Leste	Aileu	1.3	1.0	1.8	3	2	4	0.4	0.3	0.5	2	1	2
Timor-Leste	Ainaro	1.3	1.0	1.7	3	2	4	0.4	0.3	0.5	1	1	1
Timor-Leste	Alas	1.1	0.9	1.4	1	1	2	0.3	0.3	0.4	0	0	0
Timor-Leste	Atabai	1.7	1.3	2.1	3	2	4	0.5	0.4	0.6	1	1	1
Timor-Leste	Atauro	1.6	1.2	2.1	2	1	2	0.4	0.3	0.5	0	0	1
Timor-Leste	Atsabe	1.4	1.1	1.8	5	4	7	0.4	0.4	0.5	2	1	2
Timor-Leste	Baguia	1.3	1.0	1.7	3	2	4	0.4	0.3	0.5	0	0	0
Timor-Leste	Balibó	1.7	1.4	2.1	5	4	6	0.5	0.4	0.6	1	1	2
Timor-Leste	Barique	1.3	1.0	1.7	1	1	2	0.3	0.3	0.4	0	0	0
Timor-Leste	Baucau	1.3	1.0	1.7	8	6	10	0.4	0.3	0.5	3	2	4
Timor-Leste	Bazar Tete	1.5	1.1	1.9	6	4	7	0.5	0.4	0.6	2	2	3
Timor-Leste	Bobonaro	1.4	1.1	1.8	6	4	7	0.4	0.4	0.5	1	1	2
Timor-Leste	Cailaco	1.5	1.2	2.0	3	2	3	0.5	0.4	0.6	1	1	1
Timor-Leste	Dili Barat	1.8	1.4	2.3	27	21	34	0.4	0.3	0.5	11	9	15
Timor-Leste	Dili Timur	1.8	1.3	2.2	15	12	19	0.4	0.3	0.5	5	4	7
Timor-Leste	Ermera	1.3	1.0	1.7	5	4	6	0.4	0.4	0.5	2	2	3
Timor-Leste	Fato Berliu	1.1	0.9	1.5	1	1	2	0.3	0.3	0.4	0	0	0
Timor-Leste	Fatu Fulic	1.5	1.2	1.8	0	0	1	0.4	0.4	0.5	0	0	0
Timor-Leste	Fatu Mean	1.4	1.2	1.8	1	1	1	0.4	0.3	0.5	0	0	0
Timor-Leste	Fohorem	1.5	1.2	1.9	1	1	1	0.4	0.4	0.5	0	0	0
Timor-Leste	Hato Builico	1.3	1.0	1.8	3	2	4	0.4	0.3	0.5	1	1	1

Table 3: Diarrhoea Mortality by Unit *(continued)*

Country	Unit	2000 mortality mean rate (per 10,000)	2000 mortality lower rate (per 10,000)	2000 mortality upper rate (per 10,000)	2000 mortality mean count	2000 mortality lower count	2000 mortality upper count	2017 mortality mean rate (per 10,000)	2017 mortality lower rate (per 10,000)	2017 mortality upper rate (per 10,000)	2017 mortality mean count	2017 mortality lower count	2017 mortality upper count
Timor-Leste	Hato Hudo	1.3	1.0	1.7	2	2	3	0.4	0.3	0.5	1	1	1
Timor-Leste	Hatólia	1.4	1.1	1.8	8	6	11	0.5	0.4	0.6	3	2	3
Timor-Leste	Iliomar	1.7	1.3	2.2	2	2	3	0.4	0.3	0.5	1	0	1
Timor-Leste	Laclo	1.8	1.4	2.3	3	2	4	0.4	0.3	0.5	1	0	1
Timor-Leste	Laclubar	1.4	1.1	1.8	2	1	2	0.4	0.3	0.4	1	1	1
Timor-Leste	Lacluta	1.5	1.2	2.0	1	1	2	0.4	0.3	0.5	0	0	0
Timor-Leste	Laga	1.4	1.1	1.8	4	3	5	0.4	0.3	0.5	1	1	1
Timor-Leste	Laleia	1.6	1.2	2.1	1	1	1	0.4	0.3	0.5	0	0	0
Timor-Leste	Lau Lara	1.4	1.1	1.8	2	1	2	0.4	0.3	0.5	1	1	1
Timor-Leste	Lautém	1.7	1.3	2.2	5	4	7	0.5	0.4	0.5	1	1	1
Timor-Leste	Lequidoe	1.4	1.1	1.8	1	1	2	0.4	0.3	0.5	0	0	0
Timor-Leste	Letefoho	1.4	1.0	1.7	5	3	6	0.4	0.4	0.5	1	1	2
Timor-Leste	Liquiçá	1.4	1.1	1.7	4	3	5	0.5	0.4	0.6	2	1	2
Timor-Leste	Lolotoi	1.5	1.2	1.9	2	2	2	0.4	0.4	0.5	0	0	1
Timor-Leste	Los Palos	1.9	1.5	2.5	10	7	12	0.5	0.4	0.5	2	2	3
Timor-Leste	Luro	1.6	1.2	2.0	2	2	3	0.4	0.3	0.5	0	0	0
Timor-Leste	Maliana	1.7	1.3	2.1	7	5	9	0.5	0.4	0.6	2	2	3
Timor-Leste	Manatuto	1.8	1.4	2.4	3	2	4	0.4	0.3	0.5	1	1	1
Timor-Leste	Mape	1.3	1.0	1.6	2	2	2	0.4	0.3	0.5	1	1	1
Timor-Leste	Maubara	1.5	1.2	1.9	4	4	6	0.5	0.4	0.6	1	1	2
Timor-Leste	Maubisse	1.4	1.0	1.8	6	5	8	0.4	0.3	0.5	1	1	1
Timor-Leste	Metinaro	1.8	1.4	2.3	1	1	1	0.4	0.3	0.5	0	0	0
Timor-Leste	Nitibe	1.6	1.3	2.1	4	3	5	0.5	0.4	0.6	1	1	1
Timor-Leste	Oe Silo	1.7	1.4	2.1	4	3	5	0.5	0.4	0.6	1	1	1
Timor-Leste	Ossu	1.4	1.0	1.8	4	3	6	0.4	0.3	0.5	1	1	1
Timor-Leste	Pante Macassar	1.7	1.4	2.2	11	8	13	0.6	0.5	0.7	4	3	5
Timor-Leste	Passabe	1.7	1.3	2.1	2	2	3	0.5	0.4	0.6	1	0	1
Timor-Leste	Quelicai	1.3	1.0	1.7	4	3	5	0.4	0.3	0.5	1	1	1
Timor-Leste	Railaco	1.4	1.0	1.7	2	2	3	0.4	0.4	0.5	1	1	1
Timor-Leste	Remexio	1.5	1.2	1.9	2	2	3	0.4	0.3	0.5	1	0	1
Timor-Leste	Same	1.2	0.9	1.5	5	4	7	0.3	0.3	0.4	1	1	2
Timor-Leste	Soibada	1.3	1.0	1.7	1	0	1	0.4	0.3	0.4	0	0	0
Timor-Leste	Suai Kota	1.6	1.3	2.0	6	5	7	0.4	0.3	0.5	2	1	2
Timor-Leste	Tilomar	1.6	1.3	1.9	2	1	2	0.4	0.4	0.5	1	0	1
Timor-Leste	Turiscai	1.2	0.9	1.5	1	1	2	0.4	0.3	0.4	0	0	0
Timor-Leste	Tutuala	2.0	1.5	2.6	2	1	2	0.5	0.4	0.6	0	0	0
Timor-Leste	Uato Carbau	1.4	1.1	1.9	1	1	2	0.4	0.3	0.5	0	0	0
Timor-Leste	Uatolari	1.6	1.2	2.0	5	4	6	0.4	0.3	0.5	1	1	1
Timor-Leste	Vemasse	1.4	1.0	1.8	2	2	3	0.4	0.3	0.5	1	0	1
Timor-Leste	Venilale	1.3	1.0	1.7	3	2	4	0.4	0.3	0.5	1	1	1
Timor-Leste	Viqueque	1.6	1.2	2.0	6	5	8	0.4	0.3	0.5	2	1	2
Vietnam	A Lưới	0.1	0.1	0.2	0	0	1	0.0	0.0	0.0	0	0	0
Vietnam	An Biên	0.1	0.0	0.1	1	0	1	0.0	0.0	0.0	0	0	0
Vietnam	An Dương	0.1	0.1	0.2	2	1	3	0.0	0.0	0.0	0	0	1
Vietnam	An Khê	0.1	0.1	0.2	1	0	1	0.0	0.0	0.0	0	0	0
Vietnam	An Lão	0.1	0.1	0.2	0	0	0	0.0	0.0	0.0	0	0	0
Vietnam	An Lão	0.1	0.1	0.2	1	1	2	0.0	0.0	0.0	0	0	0
Vietnam	An Minh	0.1	0.0	0.1	1	0	1	0.0	0.0	0.0	0	0	0
Vietnam	An Nhơn	0.1	0.1	0.2	2	1	3	0.0	0.0	0.0	0	0	0
Vietnam	An Phú	0.1	0.1	0.1	1	1	2	0.0	0.0	0.0	0	0	0
Vietnam	Ân Thi	0.1	0.1	0.2	1	1	2	0.0	0.0	0.0	0	0	0
Vietnam	Anh Sơn	0.1	0.1	0.2	1	1	1	0.0	0.0	0.0	0	0	0
Vietnam	Ayun Pa	0.1	0.1	0.2	0	0	1	0.0	0.0	0.0	0	0	0
Vietnam	Ba Bể	0.1	0.1	0.2	0	0	1	0.0	0.0	0.0	0	0	0
Vietnam	Ba Chẽ	0.1	0.0	0.1	0	0	0	0.0	0.0	0.0	0	0	0
Vietnam	Ba Đình	0.1	0.1	0.2	2	1	3	0.0	0.0	0.0	0	0	1
Vietnam	Ba Đồn	0.1	0.0	0.1	1	0	1	0.0	0.0	0.0	0	0	0
Vietnam	Bà Rịa	0.1	0.0	0.1	1	0	1	0.0	0.0	0.0	0	0	0
Vietnam	Bá Thước	0.1	0.0	0.1	1	0	1	0.0	0.0	0.0	0	0	0
Vietnam	Ba Tơ	0.1	0.1	0.2	1	0	1	0.0	0.0	0.0	0	0	0

Table 3: Diarrhoea Mortality by Unit *(continued)*

Country	Unit	2000 mortality mean rate (per 10,000)	2000 mortality lower rate (per 10,000)	2000 mortality upper rate (per 10,000)	2000 mortality mean count	2000 mortality lower count	2000 mortality upper count	2017 mortality mean rate (per 10,000)	2017 mortality lower rate (per 10,000)	2017 mortality upper rate (per 10,000)	2017 mortality mean count	2017 mortality lower count	2017 mortality upper count
Vietnam	Ba Tri	0.1	0.1	0.1	2	1	2	0.0	0.0	0.0	0	0	0
Vietnam	Ba Vì	0.1	0.1	0.2	3	2	5	0.0	0.0	0.0	0	0	1
Vietnam	Bác Ái	0.1	0.1	0.2	0	0	0	0.0	0.0	0.0	0	0	0
Vietnam	Bắc Bình	0.1	0.0	0.1	1	1	2	0.0	0.0	0.0	0	0	0
Vietnam	Bắc Giang	0.1	0.1	0.2	1	1	2	0.0	0.0	0.0	0	0	0
Vietnam	Bắc Hà	0.1	0.1	0.2	1	1	2	0.0	0.0	0.0	0	0	0
Vietnam	Bạc Liêu	0.1	0.0	0.1	1	0	1	0.0	0.0	0.0	0	0	0
Vietnam	Bắc Mê	0.1	0.1	0.2	1	0	1	0.0	0.0	0.0	0	0	0
Vietnam	Bắc Ninh	0.1	0.1	0.2	2	1	4	0.0	0.0	0.0	0	0	1
Vietnam	Bắc Quang	0.1	0.1	0.2	1	1	2	0.0	0.0	0.0	0	0	0
Vietnam	Bắc Sơn	0.1	0.0	0.1	1	0	1	0.0	0.0	0.0	0	0	0
Vietnam	Bắc Tân Uyên	0.1	0.0	0.1	0	0	1	0.0	0.0	0.0	0	0	0
Vietnam	Bắc Trà My	0.1	0.1	0.1	0	0	1	0.0	0.0	0.0	0	0	0
Vietnam	Bắc Từ Liêm	0.1	0.1	0.2	2	1	3	0.0	0.0	0.0	0	0	1
Vietnam	Bắc Yên	0.1	0.1	0.2	1	0	1	0.0	0.0	0.0	0	0	0
Vietnam	Bạch Long Vĩ	NA	NA	NA	NA	NA	NA	NA	NA	NA	NA	NA	NA
Vietnam	Bạch Thông	0.1	0.1	0.2	0	0	0	0.0	0.0	0.0	0	0	0
Vietnam	Bảo Lạc	0.1	0.1	0.2	1	0	1	0.0	0.0	0.0	0	0	0
Vietnam	Bảo Lâm	0.1	0.1	0.2	1	0	1	0.0	0.0	0.0	0	0	0
Vietnam	Bảo Lâm	0.1	0.1	0.2	1	1	2	0.0	0.0	0.0	0	0	0
Vietnam	Bảo Lộc	0.1	0.1	0.1	1	1	2	0.0	0.0	0.0	0	0	0
Vietnam	Bảo Thắng	0.1	0.1	0.2	2	1	3	0.0	0.0	0.0	0	0	1
Vietnam	Bảo Yên	0.1	0.1	0.2	1	1	2	0.0	0.0	0.0	0	0	0
Vietnam	Bát Xát	0.1	0.1	0.2	1	1	2	0.0	0.0	0.0	0	0	0
Vietnam	Bàu Bàng	0.1	0.0	0.1	0	0	0	0.0	0.0	0.0	0	0	0
Vietnam	Bến Cát	0.1	0.0	0.1	0	0	1	0.0	0.0	0.0	0	0	0
Vietnam	Bến Cầu	0.1	0.0	0.1	1	0	1	0.0	0.0	0.0	0	0	0
Vietnam	Bến Lức	0.1	0.0	0.1	1	1	2	0.0	0.0	0.0	0	0	0
Vietnam	Bến Tre	0.1	0.1	0.2	1	1	2	0.0	0.0	0.0	0	0	0
Vietnam	Biên Hòa	0.1	0.0	0.1	5	3	8	0.0	0.0	0.0	1	1	2
Vietnam	Bỉm Sơn	0.1	0.1	0.1	0	0	1	0.0	0.0	0.0	0	0	0
Vietnam	Bình Chánh	0.1	0.0	0.1	4	2	7	0.0	0.0	0.0	1	0	2
Vietnam	Bình Đại	0.1	0.1	0.2	1	1	2	0.0	0.0	0.0	0	0	0
Vietnam	Bình Gia	0.1	0.1	0.2	0	0	1	0.0	0.0	0.0	0	0	0
Vietnam	Bình Giang	0.1	0.1	0.2	1	1	2	0.0	0.0	0.0	0	0	0
Vietnam	Bình Liêu	0.1	0.0	0.1	0	0	0	0.0	0.0	0.0	0	0	0
Vietnam	Bình Long	0.1	0.0	0.1	0	0	1	0.0	0.0	0.0	0	0	0
Vietnam	Bình Lục	0.1	0.1	0.2	1	1	2	0.0	0.0	0.0	0	0	0
Vietnam	Bình Minh	0.1	0.1	0.2	1	0	1	0.0	0.0	0.0	0	0	0
Vietnam	Bình Sơn	0.1	0.1	0.1	1	1	2	0.0	0.0	0.0	0	0	0
Vietnam	Bình Tân	0.1	0.0	0.1	3	2	6	0.0	0.0	0.0	1	0	1
Vietnam	Bình Tân	0.1	0.1	0.1	1	0	1	0.0	0.0	0.0	0	0	0
Vietnam	Bình Thạnh	0.1	0.0	0.1	2	1	3	0.0	0.0	0.0	0	0	1
Vietnam	Bình Thuỷ	0.1	0.1	0.1	1	1	1	0.0	0.0	0.0	0	0	0
Vietnam	Bình Xuyên	0.1	0.1	0.2	1	1	2	0.0	0.0	0.0	0	0	0
Vietnam	Bố Trạch	0.1	0.0	0.1	1	1	2	0.0	0.0	0.0	0	0	0
Vietnam	Bù Đăng	0.1	0.1	0.1	1	1	2	0.0	0.0	0.0	0	0	0
Vietnam	Bù Đốp	0.1	0.0	0.1	0	0	1	0.0	0.0	0.0	0	0	0
Vietnam	Bù Gia Mập	0.1	0.0	0.1	1	0	1	0.0	0.0	0.0	0	0	0
Vietnam	Buôn Đôn	0.1	0.1	0.2	1	0	1	0.0	0.0	0.0	0	0	0
Vietnam	Buôn Ma Thuột	0.1	0.1	0.2	3	2	5	0.0	0.0	0.0	1	0	1
Vietnam	Cà Mau	0.1	0.0	0.1	1	1	2	0.0	0.0	0.0	0	0	0
Vietnam	Cái Bè	0.1	0.1	0.1	2	1	4	0.0	0.0	0.0	0	0	1
Vietnam	Cai Lậy	0.1	0.1	0.1	2	1	2	0.0	0.0	0.0	0	0	0
Vietnam	Cai Lậy (Thị xã)	0.1	0.1	0.1	1	1	2	0.0	0.0	0.0	0	0	0
Vietnam	Cái Nước	0.1	0.0	0.1	1	0	1	0.0	0.0	0.0	0	0	0
Vietnam	Cái Răng	0.1	0.1	0.2	1	1	2	0.0	0.0	0.0	0	0	0
Vietnam	Cẩm Giàng	0.1	0.1	0.2	1	1	2	0.0	0.0	0.0	0	0	0
Vietnam	Cẩm Khê	0.1	0.1	0.2	2	1	4	0.0	0.0	0.0	0	0	0
Vietnam	Cam Lâm	0.1	0.1	0.2	1	0	1	0.0	0.0	0.0	0	0	0

Table 3: Diarrhoea Mortality by Unit *(continued)*

Country	Unit	2000 mortality mean rate (per 10,000)	lower rate (per 10,000)	upper rate (per 10,000)	mean count	lower count	upper count	2017 mortality mean rate (per 10,000)	lower rate (per 10,000)	upper rate (per 10,000)	mean count	lower count	upper count
Vietnam	Cẩm Lệ	0.1	0.1	0.2	1	1	2	0.0	0.0	0.0	0	0	0
Vietnam	Cam Lộ	0.1	0.0	0.1	0	0	1	0.0	0.0	0.0	0	0	0
Vietnam	Cẩm Mỹ	0.1	0.0	0.1	1	1	1	0.0	0.0	0.0	0	0	0
Vietnam	Cẩm Phả	0.1	0.0	0.1	1	1	2	0.0	0.0	0.0	0	0	0
Vietnam	Cam Ranh	0.1	0.1	0.1	1	1	2	0.0	0.0	0.0	0	0	0
Vietnam	Cẩm Thủy	0.1	0.0	0.1	1	0	1	0.0	0.0	0.0	0	0	0
Vietnam	Cẩm Xuyên	0.1	0.1	0.2	1	1	2	0.0	0.0	0.0	0	0	0
Vietnam	Cần Đước	0.1	0.1	0.1	1	1	2	0.0	0.0	0.0	0	0	0
Vietnam	Cần Giờ	0.1	0.1	0.1	0	0	1	0.0	0.0	0.0	0	0	0
Vietnam	Cần Giuộc	0.1	0.1	0.1	1	1	2	0.0	0.0	0.0	0	0	0
Vietnam	Can Lộc	0.1	0.1	0.2	1	1	2	0.0	0.0	0.0	0	0	0
Vietnam	Càng Long	0.1	0.1	0.2	1	1	2	0.0	0.0	0.0	0	0	0
Vietnam	Cao Bằng	0.1	0.1	0.2	1	0	1	0.0	0.0	0.0	0	0	0
Vietnam	Cao Lãnh	0.1	0.1	0.1	2	1	2	0.0	0.0	0.0	0	0	0
Vietnam	Cao Lãnh (Thành phố)	0.1	0.1	0.1	1	1	2	0.0	0.0	0.0	0	0	0
Vietnam	Cao Lộc	0.1	0.1	0.1	1	0	1	0.0	0.0	0.0	0	0	0
Vietnam	Cao Phong	0.1	0.1	0.2	0	0	1	0.0	0.0	0.0	0	0	0
Vietnam	Cát Hải	0.1	0.1	0.1	0	0	0	0.0	0.0	0.0	0	0	0
Vietnam	Cát Tiên	0.1	0.1	0.1	0	0	0	0.0	0.0	0.0	0	0	0
Vietnam	Cầu Giấy	0.1	0.1	0.2	2	1	3	0.0	0.0	0.0	0	0	1
Vietnam	Cầu Kè	0.1	0.1	0.1	1	1	2	0.0	0.0	0.0	0	0	0
Vietnam	Cầu Ngang	0.1	0.0	0.1	1	1	2	0.0	0.0	0.0	0	0	0
Vietnam	Châu Đốc	0.1	0.0	0.1	1	0	1	0.0	0.0	0.0	0	0	0
Vietnam	Châu Đức	0.1	0.0	0.1	1	1	2	0.0	0.0	0.0	0	0	0
Vietnam	Châu Phú	0.1	0.0	0.1	2	1	3	0.0	0.0	0.0	0	0	1
Vietnam	Châu Thành	0.1	0.0	0.1	1	0	1	0.0	0.0	0.0	0	0	0
Vietnam	Châu Thành	0.1	0.1	0.1	1	0	1	0.0	0.0	0.0	0	0	0
Vietnam	Châu Thành	0.1	0.0	0.1	1	1	2	0.0	0.0	0.0	0	0	0
Vietnam	Châu Thành	0.1	0.1	0.1	2	1	2	0.0	0.0	0.0	0	0	0
Vietnam	Châu Thành	0.1	0.0	0.1	1	1	2	0.0	0.0	0.0	0	0	0
Vietnam	Châu Thành	0.1	0.1	0.2	1	0	1	0.0	0.0	0.0	0	0	0
Vietnam	Châu Thành	0.1	0.0	0.1	1	1	2	0.0	0.0	0.0	0	0	0
Vietnam	Châu Thành	0.1	0.0	0.1	1	1	2	0.0	0.0	0.0	0	0	0
Vietnam	Châu Thành	0.1	0.1	0.2	1	1	2	0.0	0.0	0.0	0	0	0
Vietnam	Châu Thành	0.1	0.1	0.2	2	1	3	0.0	0.0	0.0	0	0	0
Vietnam	Châu Thành A	0.1	0.1	0.1	1	1	2	0.0	0.0	0.0	0	0	0
Vietnam	Chi Lăng	0.1	0.0	0.1	1	0	1	0.0	0.0	0.0	0	0	0
Vietnam	Chí Linh	0.1	0.1	0.2	1	1	2	0.0	0.0	0.0	0	0	0
Vietnam	Chiêm Hóa	0.1	0.1	0.2	1	1	2	0.0	0.0	0.0	0	0	0
Vietnam	Chợ Đồn	0.1	0.1	0.2	0	0	1	0.0	0.0	0.0	0	0	0
Vietnam	Chợ Gạo	0.1	0.1	0.2	2	1	3	0.0	0.0	0.0	0	0	0
Vietnam	Chợ Lách	0.1	0.1	0.2	1	0	1	0.0	0.0	0.0	0	0	0
Vietnam	Chợ Mới	0.1	0.0	0.1	3	2	5	0.0	0.0	0.0	0	0	1
Vietnam	Chợ Mới	0.1	0.1	0.2	0	0	1	0.0	0.0	0.0	0	0	0
Vietnam	Chơn Thành	0.1	0.0	0.1	0	0	1	0.0	0.0	0.0	0	0	0
Vietnam	Chư Păh	0.1	0.1	0.2	1	0	1	0.0	0.0	0.0	0	0	0
Vietnam	Chư Prông	0.1	0.1	0.2	1	1	2	0.0	0.0	0.0	0	0	0
Vietnam	Chư Pưh	0.1	0.1	0.2	1	0	1	0.0	0.0	0.0	0	0	0
Vietnam	Chư Sê	0.1	0.1	0.2	1	1	2	0.0	0.0	0.0	0	0	0
Vietnam	Chương Mỹ	0.1	0.1	0.2	2	1	4	0.0	0.0	0.0	1	0	1
Vietnam	Cờ Đỏ	0.1	0.0	0.1	1	1	2	0.0	0.0	0.0	0	0	0
Vietnam	Cô Tô	0.1	0.0	0.1	0	0	0	0.0	0.0	0.0	0	0	0
Vietnam	Cồn Cỏ	NA	NA	NA	NA	NA	NA	NA	NA	NA	NA	NA	NA
Vietnam	Con Cuông	0.1	0.1	0.1	1	0	1	0.0	0.0	0.0	0	0	0
Vietnam	Củ Chi	0.1	0.0	0.1	2	1	3	0.0	0.0	0.0	0	0	1
Vietnam	Cư Jút	0.1	0.1	0.2	1	1	1	0.0	0.0	0.0	0	0	0
Vietnam	Cư Kuin	0.1	0.1	0.2	1	1	2	0.0	0.0	0.0	0	0	0
Vietnam	Cù Lao Dung	0.1	0.0	0.1	1	0	1	0.0	0.0	0.0	0	0	0
Vietnam	Cư M'gar	0.1	0.1	0.2	2	1	3	0.0	0.0	0.0	0	0	1

Table 3: Diarrhoea Mortality by Unit *(continued)*

Country	Unit	2000 mortality mean rate (per 10,000)	lower rate (per 10,000)	upper rate (per 10,000)	mean count	lower count	upper count	2017 mortality mean rate (per 10,000)	lower rate (per 10,000)	upper rate (per 10,000)	mean count	lower count	upper count
Vietnam	Cửa Lò	0.1	0.1	0.2	0	0	0	0.0	0.0	0.0	0	0	0
Vietnam	Đà Bắc	0.1	0.1	0.2	1	0	1	0.0	0.0	0.0	0	0	0
Vietnam	Đạ Huoai	0.1	0.1	0.1	0	0	0	0.0	0.0	0.0	0	0	0
Vietnam	Đa Krông	0.1	0.1	0.2	0	0	1	0.0	0.0	0.0	0	0	0
Vietnam	Đà Lạt	0.1	0.1	0.2	2	1	3	0.0	0.0	0.0	0	0	1
Vietnam	Đạ Tẻh	0.1	0.1	0.1	0	0	1	0.0	0.0	0.0	0	0	0
Vietnam	Đại Lộc	0.1	0.1	0.2	1	1	2	0.0	0.0	0.0	0	0	0
Vietnam	Đại Từ	0.1	0.1	0.2	2	1	2	0.0	0.0	0.0	0	0	0
Vietnam	Đăk Đoa	0.1	0.1	0.2	1	1	2	0.0	0.0	0.0	0	0	0
Vietnam	Đắk Glei	0.1	0.1	0.2	0	0	1	0.0	0.0	0.0	0	0	0
Vietnam	Đăk Glong	0.1	0.1	0.2	0	0	1	0.0	0.0	0.0	0	0	0
Vietnam	Đắk Hà	0.1	0.1	0.2	1	0	1	0.0	0.0	0.0	0	0	0
Vietnam	Đắk Mil	0.1	0.1	0.2	1	1	1	0.0	0.0	0.0	0	0	0
Vietnam	Đăk Pơ	0.1	0.1	0.2	1	0	1	0.0	0.0	0.0	0	0	0
Vietnam	Đắk R'Lấp	0.1	0.1	0.2	1	0	1	0.0	0.0	0.0	0	0	0
Vietnam	Đắk Song	0.1	0.1	0.2	1	0	1	0.0	0.0	0.0	0	0	0
Vietnam	Đắk Tô	0.1	0.1	0.2	0	0	1	0.0	0.0	0.0	0	0	0
Vietnam	Đầm Dơi	0.1	0.0	0.1	1	1	2	0.0	0.0	0.0	0	0	0
Vietnam	Đầm Hà	0.1	0.0	0.1	0	0	0	0.0	0.0	0.0	0	0	0
Vietnam	Đam Rông	0.1	0.1	0.2	0	0	1	0.0	0.0	0.0	0	0	0
Vietnam	Đan Phượng	0.1	0.1	0.2	1	1	2	0.0	0.0	0.0	0	0	0
Vietnam	Đất Đỏ	0.1	0.0	0.1	1	0	1	0.0	0.0	0.0	0	0	0
Vietnam	Dầu Tiếng	0.1	0.0	0.1	0	0	1	0.0	0.0	0.0	0	0	0
Vietnam	Dĩ An	0.1	0.0	0.1	1	1	2	0.0	0.0	0.0	0	0	1
Vietnam	Di Linh	0.1	0.1	0.2	1	1	2	0.0	0.0	0.0	0	0	0
Vietnam	Điện Bàn	0.1	0.1	0.2	2	1	3	0.0	0.0	0.0	0	0	0
Vietnam	Điện Biên	0.1	0.1	0.2	1	1	2	0.0	0.0	0.0	0	0	1
Vietnam	Điện Biên Đông	0.1	0.1	0.1	1	0	1	0.0	0.0	0.0	0	0	0
Vietnam	Điện Biên Phủ	0.1	0.0	0.1	0	0	1	0.0	0.0	0.0	0	0	0
Vietnam	Diễn Châu	0.1	0.1	0.1	2	1	4	0.0	0.0	0.0	0	0	1
Vietnam	Diên Khánh	0.1	0.1	0.2	1	1	2	0.0	0.0	0.0	0	0	0
Vietnam	Định Hóa	0.1	0.1	0.2	1	0	1	0.0	0.0	0.0	0	0	0
Vietnam	Đình Lập	0.1	0.0	0.1	0	0	0	0.0	0.0	0.0	0	0	0
Vietnam	Định Quán	0.1	0.0	0.1	1	1	2	0.0	0.0	0.0	0	0	0
Vietnam	Đô Lương	0.1	0.1	0.2	2	1	3	0.0	0.0	0.0	0	0	0
Vietnam	Đồ Sơn	0.1	0.1	0.2	0	0	1	0.0	0.0	0.0	0	0	0
Vietnam	Đoan Hùng	0.1	0.1	0.2	2	1	3	0.0	0.0	0.0	0	0	0
Vietnam	Đơn Dương	0.1	0.1	0.2	1	1	1	0.0	0.0	0.0	0	0	0
Vietnam	Đông Anh	0.1	0.1	0.2	3	2	5	0.0	0.0	0.0	1	0	1
Vietnam	Đống Đa	0.1	0.1	0.2	2	1	4	0.0	0.0	0.0	0	0	1
Vietnam	Đông Giang	0.1	0.1	0.2	0	0	0	0.0	0.0	0.0	0	0	0
Vietnam	Đông Hà	0.1	0.0	0.1	1	0	1	0.0	0.0	0.0	0	0	0
Vietnam	Đông Hải	0.1	0.0	0.1	1	0	1	0.0	0.0	0.0	0	0	0
Vietnam	Đông Hòa	0.1	0.1	0.2	1	1	2	0.0	0.0	0.0	0	0	0
Vietnam	Đông Hưng	0.1	0.1	0.2	2	1	3	0.0	0.0	0.0	0	0	0
Vietnam	Đồng Hỷ	0.1	0.1	0.2	1	0	1	0.0	0.0	0.0	0	0	0
Vietnam	Đồng Phú	0.1	0.0	0.1	1	0	1	0.0	0.0	0.0	0	0	0
Vietnam	Đông Sơn	0.1	0.0	0.1	1	0	1	0.0	0.0	0.0	0	0	0
Vietnam	Đông Triều	0.1	0.1	0.2	1	1	2	0.0	0.0	0.0	0	0	0
Vietnam	Đồng Văn	0.1	0.1	0.2	1	0	1	0.0	0.0	0.0	0	0	0
Vietnam	Đồng Xoài	0.1	0.0	0.1	1	0	1	0.0	0.0	0.0	0	0	0
Vietnam	Đồng Xuân	0.1	0.1	0.2	1	0	1	0.0	0.0	0.0	0	0	0
Vietnam	Đức Cơ	0.1	0.1	0.1	1	0	1	0.0	0.0	0.0	0	0	0
Vietnam	Đức Hòa	0.1	0.0	0.1	2	1	2	0.0	0.0	0.0	0	0	0
Vietnam	Đức Huệ	0.1	0.1	0.1	1	0	1	0.0	0.0	0.0	0	0	0
Vietnam	Đức Linh	0.1	0.0	0.1	1	1	1	0.0	0.0	0.0	0	0	0
Vietnam	Đức Phổ	0.1	0.1	0.2	1	1	2	0.0	0.0	0.0	0	0	0
Vietnam	Đức Thọ	0.1	0.1	0.2	1	1	2	0.0	0.0	0.0	0	0	0
Vietnam	Đức Trọng	0.1	0.1	0.2	2	1	2	0.0	0.0	0.0	0	0	1
Vietnam	Dương Kinh	0.1	0.1	0.2	1	0	1	0.0	0.0	0.0	0	0	0

Table 3: Diarrhoea Mortality by Unit *(continued)*

Country	Unit	2000 mortality mean rate (per 10,000)	lower rate (per 10,000)	upper rate (per 10,000)	mean count	lower count	upper count	2017 mortality mean rate (per 10,000)	lower rate (per 10,000)	upper rate (per 10,000)	mean count	lower count	upper count
Vietnam	Dương Minh Châu	0.1	0.0	0.1	1	0	1	0.0	0.0	0.0	0	0	0
Vietnam	Duy Tiên	0.1	0.1	0.2	1	1	2	0.0	0.0	0.0	0	0	0
Vietnam	Duy Xuyên	0.1	0.1	0.2	1	1	2	0.0	0.0	0.0	0	0	0
Vietnam	Duyên Hải	0.1	0.0	0.1	1	0	1	0.0	0.0	0.0	0	0	0
Vietnam	Duyên Hải (Thị xã)	0.1	0.0	0.1	0	0	1	0.0	0.0	0.0	0	0	0
Vietnam	Ea H'leo	0.1	0.1	0.2	1	1	2	0.0	0.0	0.0	0	0	0
Vietnam	Ea Kar	0.1	0.1	0.2	1	1	2	0.0	0.0	0.0	0	0	0
Vietnam	Ea Súp	0.1	0.1	0.2	1	0	1	0.0	0.0	0.0	0	0	0
Vietnam	Gia Bình	0.1	0.1	0.2	1	1	2	0.0	0.0	0.0	0	0	0
Vietnam	Gia Lâm	0.1	0.1	0.2	2	1	4	0.0	0.0	0.0	0	0	1
Vietnam	Gia Lộc	0.1	0.1	0.2	1	1	2	0.0	0.0	0.0	0	0	0
Vietnam	Gia Nghĩa	0.1	0.1	0.2	0	0	1	0.0	0.0	0.0	0	0	0
Vietnam	Giá Rai	0.1	0.0	0.1	1	0	1	0.0	0.0	0.0	0	0	0
Vietnam	Gia Viễn	0.1	0.1	0.2	1	1	2	0.0	0.0	0.0	0	0	0
Vietnam	Giang Thành	0.1	0.1	0.1	0	0	0	0.0	0.0	0.0	0	0	0
Vietnam	Giao Thủy	0.1	0.1	0.2	2	1	3	0.0	0.0	0.0	0	0	0
Vietnam	Gio Linh	0.1	0.0	0.1	1	0	1	0.0	0.0	0.0	0	0	0
Vietnam	Giồng Riềng	0.1	0.0	0.1	2	1	2	0.0	0.0	0.0	0	0	0
Vietnam	Giồng Trôm	0.1	0.1	0.2	1	1	2	0.0	0.0	0.0	0	0	0
Vietnam	Gò Công	0.1	0.1	0.2	1	1	1	0.0	0.0	0.0	0	0	0
Vietnam	Gò Công Đông	0.1	0.1	0.2	1	1	2	0.0	0.0	0.0	0	0	0
Vietnam	Gò Công Tây	0.1	0.1	0.2	1	1	2	0.0	0.0	0.0	0	0	0
Vietnam	Gò Dầu	0.1	0.0	0.1	1	0	1	0.0	0.0	0.0	0	0	0
Vietnam	Gò Quao	0.1	0.0	0.1	1	1	2	0.0	0.0	0.0	0	0	0
Vietnam	Gò Vấp	0.1	0.0	0.1	2	1	3	0.0	0.0	0.0	0	0	1
Vietnam	Hà Đông	0.1	0.1	0.2	2	1	4	0.0	0.0	0.0	0	0	1
Vietnam	Hà Giang	0.1	0.1	0.2	0	0	1	0.0	0.0	0.0	0	0	0
Vietnam	Hạ Hoà	0.1	0.1	0.2	2	1	3	0.0	0.0	0.0	0	0	0
Vietnam	Hạ Lang	0.1	0.1	0.2	0	0	0	0.0	0.0	0.0	0	0	0
Vietnam	Hạ Long	0.1	0.0	0.1	1	1	2	0.0	0.0	0.0	0	0	0
Vietnam	Hà Quảng	0.1	0.1	0.2	0	0	1	0.0	0.0	0.0	0	0	0
Vietnam	Hà Tiên	0.1	0.1	0.1	0	0	0	0.0	0.0	0.0	0	0	0
Vietnam	Hà Tĩnh	0.1	0.1	0.2	1	0	1	0.0	0.0	0.0	0	0	0
Vietnam	Hà Trung	0.1	0.1	0.1	1	1	1	0.0	0.0	0.0	0	0	0
Vietnam	Hải An	0.1	0.1	0.2	1	0	1	0.0	0.0	0.0	0	0	0
Vietnam	Hai Bà Trưng	0.1	0.1	0.2	2	1	3	0.0	0.0	0.0	0	0	1
Vietnam	Hải Châu	0.1	0.1	0.2	1	1	2	0.0	0.0	0.0	0	0	1
Vietnam	Hải Dương	0.1	0.1	0.2	2	1	4	0.0	0.0	0.0	0	0	1
Vietnam	Hải Hà	0.1	0.0	0.1	0	0	1	0.0	0.0	0.0	0	0	0
Vietnam	Hải Hậu	0.1	0.1	0.1	3	1	4	0.0	0.0	0.0	0	0	1
Vietnam	Hải Lăng	0.1	0.1	0.2	1	0	1	0.0	0.0	0.0	0	0	0
Vietnam	Hàm Tân	0.1	0.0	0.1	1	0	1	0.0	0.0	0.0	0	0	0
Vietnam	Hàm Thuận Bắc	0.1	0.0	0.1	1	1	2	0.0	0.0	0.0	0	0	0
Vietnam	Hàm Thuận Nam	0.1	0.0	0.1	1	0	1	0.0	0.0	0.0	0	0	0
Vietnam	Hàm Yên	0.1	0.1	0.2	1	1	2	0.0	0.0	0.0	0	0	0
Vietnam	Hậu Lộc	0.1	0.0	0.1	1	1	2	0.0	0.0	0.0	0	0	0
Vietnam	Hiệp Đức	0.1	0.1	0.1	0	0	1	0.0	0.0	0.0	0	0	0
Vietnam	Hiệp Hòa	0.1	0.1	0.2	2	1	3	0.0	0.0	0.0	0	0	0
Vietnam	Hoà An	0.1	0.1	0.2	1	0	1	0.0	0.0	0.0	0	0	0
Vietnam	Hoà Bình	0.1	0.0	0.1	1	0	1	0.0	0.0	0.0	0	0	0
Vietnam	Hòa Bình	0.1	0.1	0.2	1	1	1	0.0	0.0	0.0	0	0	0
Vietnam	Hoa Lư	0.1	0.1	0.2	1	0	1	0.0	0.0	0.0	0	0	0
Vietnam	Hòa Thành	0.1	0.0	0.1	1	0	1	0.0	0.0	0.0	0	0	0
Vietnam	Hòa Vang	0.1	0.1	0.2	1	1	2	0.0	0.0	0.0	0	0	0
Vietnam	Hoài Ân	0.1	0.1	0.2	1	1	1	0.0	0.0	0.0	0	0	0
Vietnam	Hoài Đức	0.1	0.1	0.2	2	1	3	0.0	0.0	0.0	0	0	1
Vietnam	Hoài Nhơn	0.1	0.1	0.2	2	1	3	0.0	0.0	0.0	0	0	0
Vietnam	Hoàn Kiếm	0.1	0.1	0.2	1	1	1	0.0	0.0	0.0	0	0	0

Table 3: Diarrhoea Mortality by Unit *(continued)*

Country	Unit	2000 mortality mean rate (per 10,000)	lower rate (per 10,000)	upper rate (per 10,000)	mean count	lower count	upper count	2017 mortality mean rate (per 10,000)	lower rate (per 10,000)	upper rate (per 10,000)	mean count	lower count	upper count
Vietnam	Hoằng Hóa	0.1	0.0	0.1	2	1	3	0.0	0.0	0.0	0	0	0
Vietnam	Hoàng Mai	0.1	0.1	0.2	4	2	7	0.0	0.0	0.0	1	0	1
Vietnam	Hoàng Mai	0.1	0.0	0.1	1	0	1	0.0	0.0	0.0	0	0	0
Vietnam	Hoàng Su Phì	0.1	0.1	0.2	1	0	1	0.0	0.0	0.0	0	0	0
Vietnam	Hoành Bồ	0.1	0.0	0.1	0	0	1	0.0	0.0	0.0	0	0	0
Vietnam	Hóc Môn	0.1	0.0	0.1	2	1	3	0.0	0.0	0.0	0	0	1
Vietnam	Hội An	0.1	0.1	0.2	1	0	1	0.0	0.0	0.0	0	0	0
Vietnam	Hòn Đất	0.1	0.0	0.1	1	1	2	0.0	0.0	0.0	0	0	0
Vietnam	Hớn Quản	0.1	0.0	0.1	1	0	1	0.0	0.0	0.0	0	0	0
Vietnam	Hồng Bàng	0.1	0.1	0.1	1	0	1	0.0	0.0	0.0	0	0	0
Vietnam	Hồng Dân	0.1	0.0	0.1	1	0	1	0.0	0.0	0.0	0	0	0
Vietnam	Hồng Lĩnh	0.1	0.1	0.2	0	0	1	0.0	0.0	0.0	0	0	0
Vietnam	Hồng Ngự	0.1	0.0	0.1	1	1	2	0.0	0.0	0.0	0	0	0
Vietnam	Hồng Ngự (Thị xã)	0.1	0.0	0.1	1	0	1	0.0	0.0	0.0	0	0	0
Vietnam	Huế	0.1	0.1	0.2	3	1	4	0.0	0.0	0.0	0	0	1
Vietnam	Hưng Hà	0.1	0.1	0.2	2	1	4	0.0	0.0	0.0	0	0	1
Vietnam	Hưng Nguyên	0.1	0.1	0.2	2	1	3	0.0	0.0	0.0	0	0	0
Vietnam	Hưng Yên	0.1	0.1	0.2	1	1	2	0.0	0.0	0.0	0	0	0
Vietnam	Hướng Hóa	0.1	0.1	0.1	1	0	1	0.0	0.0	0.0	0	0	0
Vietnam	Hương Khê	0.1	0.1	0.2	1	1	2	0.0	0.0	0.0	0	0	0
Vietnam	Hương Sơn	0.1	0.1	0.2	1	1	2	0.0	0.0	0.0	0	0	0
Vietnam	Hương Thủy	0.1	0.1	0.2	1	1	2	0.0	0.0	0.0	0	0	0
Vietnam	Hương Trà	0.1	0.1	0.2	1	1	2	0.0	0.0	0.0	0	0	0
Vietnam	Hữu Lũng	0.1	0.1	0.2	1	1	2	0.0	0.0	0.0	0	0	0
Vietnam	Ia Grai	0.1	0.1	0.2	1	1	1	0.0	0.0	0.0	0	0	0
Vietnam	Ia H' Drai	0.1	0.1	0.1	0	0	0	0.0	0.0	0.0	0	0	0
Vietnam	Ia Pa	0.1	0.1	0.2	1	0	1	0.0	0.0	0.0	0	0	0
Vietnam	KBang	0.1	0.1	0.2	1	0	1	0.0	0.0	0.0	0	0	0
Vietnam	Kế Sách	0.1	0.0	0.1	1	1	2	0.0	0.0	0.0	0	0	0
Vietnam	Khánh Sơn	0.1	0.1	0.2	0	0	0	0.0	0.0	0.0	0	0	0
Vietnam	Khánh Vĩnh	0.1	0.1	0.2	0	0	1	0.0	0.0	0.0	0	0	0
Vietnam	Khoái Châu	0.1	0.1	0.2	2	1	3	0.0	0.0	0.0	0	0	0
Vietnam	Kiến An	0.1	0.1	0.2	1	0	1	0.0	0.0	0.0	0	0	0
Vietnam	Kiên Hải	0.1	0.0	0.1	0	0	0	0.0	0.0	0.0	0	0	0
Vietnam	Kiên Lương	0.1	0.0	0.1	0	0	0	0.0	0.0	0.0	0	0	0
Vietnam	Kiến Thuỵ	0.1	0.1	0.2	1	1	2	0.0	0.0	0.0	0	0	0
Vietnam	Kiến Tường	0.1	0.1	0.1	0	0	1	0.0	0.0	0.0	0	0	0
Vietnam	Kiến Xương	0.1	0.1	0.2	2	1	3	0.0	0.0	0.0	0	0	0
Vietnam	Kim Bảng	0.1	0.1	0.2	1	1	2	0.0	0.0	0.0	0	0	0
Vietnam	Kim Bôi	0.1	0.1	0.2	1	1	1	0.0	0.0	0.0	0	0	0
Vietnam	Kim Động	0.1	0.1	0.2	1	1	2	0.0	0.0	0.0	0	0	0
Vietnam	Kim Sơn	0.1	0.1	0.1	1	1	2	0.0	0.0	0.0	0	0	0
Vietnam	Kim Thành	0.1	0.1	0.2	1	1	2	0.0	0.0	0.0	0	0	0
Vietnam	Kinh Môn	0.1	0.1	0.2	1	1	2	0.0	0.0	0.0	0	0	0
Vietnam	Kon Plông	0.1	0.1	0.2	0	0	0	0.0	0.0	0.0	0	0	0
Vietnam	Kon Rẫy	0.1	0.1	0.2	0	0	0	0.0	0.0	0.0	0	0	0
Vietnam	Kon Tum	0.1	0.1	0.2	2	1	3	0.0	0.0	0.0	0	0	1
Vietnam	Kông Chro	0.1	0.1	0.2	1	0	1	0.0	0.0	0.0	0	0	0
Vietnam	Krông A Na	0.1	0.1	0.2	1	1	1	0.0	0.0	0.0	0	0	0
Vietnam	Krông Bông	0.1	0.1	0.2	1	1	1	0.0	0.0	0.0	0	0	0
Vietnam	Krông Búk	0.1	0.1	0.2	1	0	1	0.0	0.0	0.0	0	0	0
Vietnam	Krông Năng	0.1	0.1	0.2	1	1	2	0.0	0.0	0.0	0	0	0
Vietnam	Krông Nô	0.1	0.1	0.2	1	0	1	0.0	0.0	0.0	0	0	0
Vietnam	Krông Pa	0.1	0.1	0.2	1	1	1	0.0	0.0	0.0	0	0	0
Vietnam	Krông Pắc	0.1	0.1	0.2	2	1	3	0.0	0.0	0.0	0	0	1
Vietnam	Kỳ Anh	0.1	0.0	0.1	1	0	1	0.0	0.0	0.0	0	0	0
Vietnam	Kỳ Anh (Thị xã)	0.1	0.0	0.1	0	0	1	0.0	0.0	0.0	0	0	0
Vietnam	Kỳ Sơn	0.1	0.1	0.2	0	0	0	0.0	0.0	0.0	0	0	0
Vietnam	Kỳ Sơn	0.1	0.1	0.1	1	0	1	0.0	0.0	0.0	0	0	0

Table 3: Diarrhoea Mortality by Unit *(continued)*

Country	Unit	2000 mortality mean rate (per 10,000)	lower rate (per 10,000)	upper rate (per 10,000)	mean count	lower count	upper count	2017 mortality mean rate (per 10,000)	lower rate (per 10,000)	upper rate (per 10,000)	mean count	lower count	upper count
Vietnam	La Gi	0.1	0.0	0.1	1	0	1	0.0	0.0	0.0	0	0	0
Vietnam	Lạc Dương	0.1	0.1	0.2	0	0	0	0.0	0.0	0.0	0	0	0
Vietnam	Lạc Sơn	0.1	0.1	0.1	1	1	2	0.0	0.0	0.0	0	0	0
Vietnam	Lạc Thủy	0.1	0.1	0.2	1	0	1	0.0	0.0	0.0	0	0	0
Vietnam	Lai Châu	0.1	0.1	0.2	0	0	0	0.0	0.0	0.0	0	0	0
Vietnam	Lai Vung	0.1	0.1	0.1	1	1	2	0.0	0.0	0.0	0	0	0
Vietnam	Lắk	0.1	0.1	0.2	1	0	1	0.0	0.0	0.0	0	0	0
Vietnam	Lâm Bình	0.1	0.1	0.2	0	0	1	0.0	0.0	0.0	0	0	0
Vietnam	Lâm Hà	0.1	0.1	0.2	1	1	2	0.0	0.0	0.0	0	0	0
Vietnam	Lâm Thao	0.1	0.1	0.2	2	1	3	0.0	0.0	0.0	0	0	0
Vietnam	Lang Chánh	0.1	0.0	0.1	0	0	1	0.0	0.0	0.0	0	0	0
Vietnam	Lạng Giang	0.1	0.1	0.2	2	1	3	0.0	0.0	0.0	0	0	0
Vietnam	Lạng Sơn	0.1	0.1	0.1	1	0	1	0.0	0.0	0.0	0	0	0
Vietnam	Lào Cai	0.1	0.1	0.2	2	1	3	0.0	0.0	0.0	0	0	1
Vietnam	Lập Thạch	0.1	0.1	0.2	1	1	2	0.0	0.0	0.0	0	0	0
Vietnam	Lấp Vò	0.1	0.1	0.1	1	1	2	0.0	0.0	0.0	0	0	0
Vietnam	Lê Chân	0.1	0.1	0.1	1	1	2	0.0	0.0	0.0	0	0	0
Vietnam	Lệ Thủy	0.1	0.0	0.1	1	1	2	0.0	0.0	0.0	0	0	0
Vietnam	Liên Chiểu	0.1	0.1	0.2	1	1	2	0.0	0.0	0.0	0	0	0
Vietnam	Lộc Bình	0.1	0.0	0.1	1	0	1	0.0	0.0	0.0	0	0	0
Vietnam	Lộc Hà	0.1	0.1	0.2	1	0	1	0.0	0.0	0.0	0	0	0
Vietnam	Lộc Ninh	0.1	0.0	0.1	1	0	1	0.0	0.0	0.0	0	0	0
Vietnam	Long Biên	0.1	0.1	0.2	3	1	4	0.0	0.0	0.0	1	0	1
Vietnam	Long Điền	0.1	0.0	0.1	1	0	1	0.0	0.0	0.0	0	0	0
Vietnam	Long Hồ	0.1	0.1	0.1	2	1	2	0.0	0.0	0.0	0	0	0
Vietnam	Long Khánh	0.1	0.0	0.1	1	0	1	0.0	0.0	0.0	0	0	0
Vietnam	Long Mỹ	0.1	0.0	0.1	1	0	1	0.0	0.0	0.0	0	0	0
Vietnam	Long Mỹ (Thị xã)	0.1	0.0	0.1	1	0	1	0.0	0.0	0.0	0	0	0
Vietnam	Long Phú	0.1	0.0	0.1	1	0	1	0.0	0.0	0.0	0	0	0
Vietnam	Long Thành	0.1	0.0	0.1	1	1	2	0.0	0.0	0.0	0	0	0
Vietnam	Long Xuyên	0.1	0.0	0.1	2	1	3	0.0	0.0	0.0	0	0	1
Vietnam	Lục Nam	0.1	0.1	0.2	2	1	3	0.0	0.0	0.0	0	0	0
Vietnam	Lục Ngạn	0.1	0.1	0.1	2	1	3	0.0	0.0	0.0	0	0	0
Vietnam	Lục Yên	0.1	0.1	0.2	1	1	2	0.0	0.0	0.0	0	0	0
Vietnam	Lương Sơn	0.1	0.1	0.2	1	1	2	0.0	0.0	0.0	0	0	0
Vietnam	Lương Tài	0.1	0.1	0.2	1	1	2	0.0	0.0	0.0	0	0	0
Vietnam	Lý Nhân	0.1	0.1	0.2	2	1	3	0.0	0.0	0.0	0	0	0
Vietnam	Lý Sơn	NA	NA	NA	NA	NA	NA	NA	NA	NA	NA	NA	NA
Vietnam	M'Đrắk	0.1	0.1	0.2	1	0	1	0.0	0.0	0.0	0	0	0
Vietnam	Mai Châu	0.1	0.1	0.2	0	0	1	0.0	0.0	0.0	0	0	0
Vietnam	Mai Sơn	0.1	0.1	0.2	2	1	3	0.0	0.0	0.0	0	0	1
Vietnam	Mang Thít	0.1	0.1	0.2	1	0	1	0.0	0.0	0.0	0	0	0
Vietnam	Mang Yang	0.1	0.1	0.2	1	0	1	0.0	0.0	0.0	0	0	0
Vietnam	Mê Linh	0.1	0.1	0.2	2	1	3	0.0	0.0	0.0	0	0	1
Vietnam	Mèo Vạc	0.1	0.1	0.2	1	0	1	0.0	0.0	0.0	0	0	0
Vietnam	Minh Hóa	0.1	0.0	0.1	0	0	1	0.0	0.0	0.0	0	0	0
Vietnam	Minh Long	0.1	0.1	0.2	0	0	0	0.0	0.0	0.0	0	0	0
Vietnam	Mỏ Cày Bắc	0.1	0.1	0.2	1	1	2	0.0	0.0	0.0	0	0	0
Vietnam	Mỏ Cày Nam	0.1	0.1	0.2	1	1	2	0.0	0.0	0.0	0	0	0
Vietnam	Mộ Đức	0.1	0.1	0.2	1	1	2	0.0	0.0	0.0	0	0	0
Vietnam	Mộc Châu	0.1	0.1	0.2	1	1	2	0.0	0.0	0.0	0	0	0
Vietnam	Mộc Hóa	0.1	0.1	0.1	0	0	0	0.0	0.0	0.0	0	0	0
Vietnam	Móng Cái	0.1	0.0	0.1	1	0	1	0.0	0.0	0.0	0	0	0
Vietnam	Mù Căng Chải	0.1	0.1	0.2	1	0	1	0.0	0.0	0.0	0	0	0
Vietnam	Mường Ảng	0.1	0.1	0.2	0	0	1	0.0	0.0	0.0	0	0	0
Vietnam	Mường Chà	0.1	0.1	0.2	0	0	1	0.0	0.0	0.0	0	0	0
Vietnam	Mường Khương	0.1	0.1	0.2	1	0	1	0.0	0.0	0.0	0	0	0
Vietnam	Mường La	0.1	0.1	0.2	1	1	2	0.0	0.0	0.0	0	0	0
Vietnam	Mường Lát	0.1	0.1	0.2	0	0	0	0.0	0.0	0.0	0	0	0
Vietnam	Mường Nhé	0.1	0.1	0.2	0	0	1	0.0	0.0	0.0	0	0	0

Table 3: Diarrhoea Mortality by Unit *(continued)*

Country	Unit	2000 mortality mean rate (per 10,000)	lower rate (per 10,000)	upper rate (per 10,000)	mean count	lower count	upper count	2017 mortality mean rate (per 10,000)	lower rate (per 10,000)	upper rate (per 10,000)	mean count	lower count	upper count
Vietnam	Mường Tè	0.1	0.1	0.2	0	0	0	0.0	0.0	0.0	0	0	0
Vietnam	Mỹ Đức	0.1	0.1	0.2	2	1	3	0.0	0.0	0.0	0	0	1
Vietnam	Mỹ Hào	0.1	0.1	0.2	1	1	2	0.0	0.0	0.0	0	0	0
Vietnam	Mỹ Lộc	0.1	0.1	0.2	1	1	2	0.0	0.0	0.0	0	0	0
Vietnam	Mỹ Tho	0.1	0.1	0.2	2	1	3	0.0	0.0	0.0	0	0	0
Vietnam	Mỹ Tú	0.1	0.0	0.1	1	0	1	0.0	0.0	0.0	0	0	0
Vietnam	Mỹ Xuyên	0.1	0.0	0.1	1	1	2	0.0	0.0	0.0	0	0	0
Vietnam	Nà Hang	0.1	0.1	0.2	0	0	1	0.0	0.0	0.0	0	0	0
Vietnam	Na Rì	0.1	0.1	0.2	0	0	1	0.0	0.0	0.0	0	0	0
Vietnam	Năm Căn	0.1	0.0	0.1	0	0	1	0.0	0.0	0.0	0	0	0
Vietnam	Nam Đàn	0.1	0.1	0.2	1	1	2	0.0	0.0	0.0	0	0	0
Vietnam	Nam Định	0.1	0.1	0.2	2	1	3	0.0	0.0	0.0	0	0	0
Vietnam	Nam Đông	0.1	0.1	0.2	0	0	0	0.0	0.0	0.0	0	0	0
Vietnam	Nam Giang	0.1	0.1	0.2	0	0	0	0.0	0.0	0.0	0	0	0
Vietnam	Nậm Nhùn	0.1	0.1	0.2	0	0	0	0.0	0.0	0.0	0	0	0
Vietnam	Nậm Pồ	0.1	0.1	0.2	0	0	1	0.0	0.0	0.0	0	0	0
Vietnam	Nam Sách	0.1	0.1	0.2	1	0	1	0.0	0.0	0.0	0	0	0
Vietnam	Nam Trà My	0.1	0.1	0.2	0	0	0	0.0	0.0	0.0	0	0	0
Vietnam	Nam Trực	0.1	0.1	0.2	2	1	3	0.0	0.0	0.0	0	0	0
Vietnam	Nam Từ Liêm	0.1	0.1	0.2	2	1	4	0.0	0.0	0.0	1	0	1
Vietnam	Ngã Bảy	0.1	0.0	0.1	1	0	1	0.0	0.0	0.0	0	0	0
Vietnam	Ngã Năm	0.1	0.0	0.1	1	0	1	0.0	0.0	0.0	0	0	0
Vietnam	Nga Sơn	0.1	0.1	0.1	1	1	2	0.0	0.0	0.0	0	0	0
Vietnam	Ngân Sơn	0.1	0.1	0.2	0	0	0	0.0	0.0	0.0	0	0	0
Vietnam	Nghi Lộc	0.1	0.1	0.2	2	1	2	0.0	0.0	0.0	0	0	0
Vietnam	Nghi Xuân	0.1	0.1	0.2	1	1	2	0.0	0.0	0.0	0	0	0
Vietnam	Nghĩa Đàn	0.1	0.1	0.1	1	1	2	0.0	0.0	0.0	0	0	0
Vietnam	Nghĩa Hành	0.1	0.1	0.2	1	1	1	0.0	0.0	0.0	0	0	0
Vietnam	Nghĩa Hưng	0.1	0.1	0.1	2	1	3	0.0	0.0	0.0	0	0	0
Vietnam	Nghĩa Lộ	0.1	0.1	0.2	0	0	0	0.0	0.0	0.0	0	0	0
Vietnam	Ngô Quyền	0.1	0.1	0.1	1	1	2	0.0	0.0	0.0	0	0	0
Vietnam	Ngọc Hiển	0.1	0.0	0.1	0	0	1	0.0	0.0	0.0	0	0	0
Vietnam	Ngọc Hồi	0.1	0.1	0.2	0	0	1	0.0	0.0	0.0	0	0	0
Vietnam	Ngọc Lặc	0.1	0.0	0.1	1	1	2	0.0	0.0	0.0	0	0	0
Vietnam	Ngũ Hành Sơn	0.1	0.1	0.2	1	0	1	0.0	0.0	0.0	0	0	0
Vietnam	Nguyên Bình	0.1	0.1	0.2	0	0	1	0.0	0.0	0.0	0	0	0
Vietnam	Nhà Bè	0.1	0.1	0.1	1	1	1	0.0	0.0	0.0	0	0	0
Vietnam	Nha Trang	0.1	0.1	0.2	3	2	5	0.0	0.0	0.0	1	0	1
Vietnam	Nho Quan	0.1	0.1	0.1	1	1	2	0.0	0.0	0.0	0	0	0
Vietnam	Nhơn Trạch	0.1	0.1	0.1	1	1	2	0.0	0.0	0.0	0	0	0
Vietnam	Như Thanh	0.1	0.0	0.1	1	0	1	0.0	0.0	0.0	0	0	0
Vietnam	Như Xuân	0.1	0.0	0.1	0	0	1	0.0	0.0	0.0	0	0	0
Vietnam	Ninh Bình	0.1	0.1	0.2	1	0	1	0.0	0.0	0.0	0	0	0
Vietnam	Ninh Giang	0.1	0.1	0.2	1	1	2	0.0	0.0	0.0	0	0	0
Vietnam	Ninh Hải	0.1	0.1	0.1	1	0	1	0.0	0.0	0.0	0	0	0
Vietnam	Ninh Hòa	0.1	0.1	0.2	2	1	3	0.0	0.0	0.0	0	0	1
Vietnam	Ninh Kiều	0.1	0.1	0.2	1	1	2	0.0	0.0	0.0	0	0	0
Vietnam	Ninh Phước	0.1	0.1	0.1	1	1	2	0.0	0.0	0.0	0	0	0
Vietnam	Ninh Sơn	0.1	0.1	0.2	1	0	1	0.0	0.0	0.0	0	0	0
Vietnam	Nông Cống	0.1	0.0	0.1	1	1	2	0.0	0.0	0.0	0	0	0
Vietnam	Nông Sơn	0.1	0.1	0.2	0	0	0	0.0	0.0	0.0	0	0	0
Vietnam	Núi Thành	0.1	0.0	0.1	1	1	2	0.0	0.0	0.0	0	0	0
Vietnam	Ô Môn	0.1	0.1	0.1	1	0	1	0.0	0.0	0.0	0	0	0
Vietnam	Pác Nặm	0.1	0.1	0.2	0	0	0	0.0	0.0	0.0	0	0	0
Vietnam	Phan Rang-Tháp Chàm	0.1	0.1	0.1	1	1	2	0.0	0.0	0.0	0	0	0
Vietnam	Phan Thiết	0.1	0.0	0.1	1	1	2	0.0	0.0	0.0	0	0	0
Vietnam	Phổ Yên	0.1	0.1	0.2	1	1	2	0.0	0.0	0.0	0	0	0
Vietnam	Phong Điền	0.1	0.1	0.1	1	0	1	0.0	0.0	0.0	0	0	0
Vietnam	Phong Điền	0.1	0.1	0.2	1	0	1	0.0	0.0	0.0	0	0	0

Country	Unit	2000 mortality						2017 mortality					
		mean rate (per 10,000)	lower rate (per 10,000)	upper rate (per 10,000)	mean count	lower count	upper count	mean rate (per 10,000)	lower rate (per 10,000)	upper rate (per 10,000)	mean count	lower count	upper count
Vietnam	Phong Thổ	0.1	0.1	0.2	1	0	1	0.0	0.0	0.0	0	0	0
Vietnam	Phú Bình	0.1	0.1	0.2	1	1	2	0.0	0.0	0.0	0	0	0
Vietnam	Phù Cát	0.1	0.1	0.2	2	1	3	0.0	0.0	0.0	0	0	0
Vietnam	Phù Cừ	0.1	0.1	0.2	1	0	1	0.0	0.0	0.0	0	0	0
Vietnam	Phú Giáo	0.1	0.0	0.1	0	0	1	0.0	0.0	0.0	0	0	0
Vietnam	Phú Hoà	0.1	0.1	0.2	1	1	2	0.0	0.0	0.0	0	0	0
Vietnam	Phú Lộc	0.1	0.1	0.2	1	1	2	0.0	0.0	0.0	0	0	0
Vietnam	Phú Lương	0.1	0.1	0.2	1	0	1	0.0	0.0	0.0	0	0	0
Vietnam	Phủ Lý	0.1	0.1	0.2	1	1	2	0.0	0.0	0.0	0	0	0
Vietnam	Phù Mỹ	0.1	0.1	0.2	2	1	3	0.0	0.0	0.0	0	0	0
Vietnam	Phú Nhuận	0.1	0.0	0.1	1	0	1	0.0	0.0	0.0	0	0	0
Vietnam	Phú Ninh	0.1	0.1	0.1	1	0	1	0.0	0.0	0.0	0	0	0
Vietnam	Phù Ninh	0.1	0.1	0.2	2	1	3	0.0	0.0	0.0	0	0	0
Vietnam	Phú Quí	NA	NA	NA	NA	NA	NA	NA	NA	NA	NA	NA	NA
Vietnam	Phú Quốc	0.1	0.0	0.1	1	0	1	0.0	0.0	0.0	0	0	0
Vietnam	Phú Riềng	0.1	0.0	0.1	1	0	1	0.0	0.0	0.0	0	0	0
Vietnam	Phú Tân	0.1	0.0	0.1	1	0	1	0.0	0.0	0.0	0	0	0
Vietnam	Phú Tân	0.1	0.0	0.1	2	1	3	0.0	0.0	0.0	0	0	0
Vietnam	Phú Thiện	0.1	0.1	0.2	1	0	1	0.0	0.0	0.0	0	0	0
Vietnam	Phú Thọ	0.1	0.1	0.2	1	1	2	0.0	0.0	0.0	0	0	0
Vietnam	Phú Vang	0.1	0.1	0.2	1	1	2	0.0	0.0	0.0	0	0	0
Vietnam	Phú Xuyên	0.1	0.1	0.2	2	1	3	0.0	0.0	0.0	0	0	1
Vietnam	Phù Yên	0.1	0.1	0.2	1	1	2	0.0	0.0	0.0	0	0	0
Vietnam	Phục Hoà	0.1	0.1	0.2	0	0	0	0.0	0.0	0.0	0	0	0
Vietnam	Phúc Thọ	0.1	0.1	0.2	2	1	3	0.0	0.0	0.0	0	0	1
Vietnam	Phúc Yên	0.1	0.1	0.2	1	0	1	0.0	0.0	0.0	0	0	0
Vietnam	Phụng Hiệp	0.1	0.0	0.1	2	1	3	0.0	0.0	0.0	0	0	0
Vietnam	Phước Long	0.1	0.0	0.1	0	0	0	0.0	0.0	0.0	0	0	0
Vietnam	Phước Long	0.1	0.0	0.1	1	0	1	0.0	0.0	0.0	0	0	0
Vietnam	Phước Sơn	0.1	0.1	0.2	0	0	0	0.0	0.0	0.0	0	0	0
Vietnam	Pleiku	0.1	0.1	0.2	2	1	3	0.0	0.0	0.0	0	0	1
Vietnam	Quận 1	0.1	0.0	0.1	1	1	2	0.0	0.0	0.0	0	0	0
Vietnam	Quận 10	0.1	0.0	0.1	1	1	2	0.0	0.0	0.0	0	0	0
Vietnam	Quận 11	0.1	0.0	0.1	1	1	1	0.0	0.0	0.0	0	0	0
Vietnam	Quận 12	0.1	0.0	0.1	2	1	4	0.0	0.0	0.0	1	0	1
Vietnam	Quận 2	0.1	0.0	0.1	1	1	2	0.0	0.0	0.0	0	0	0
Vietnam	Quận 3	0.1	0.0	0.1	1	0	1	0.0	0.0	0.0	0	0	0
Vietnam	Quận 4	0.1	0.0	0.1	0	0	1	0.0	0.0	0.0	0	0	0
Vietnam	Quận 5	0.1	0.0	0.1	1	0	1	0.0	0.0	0.0	0	0	0
Vietnam	Quận 6	0.1	0.0	0.1	1	1	2	0.0	0.0	0.0	0	0	0
Vietnam	Quận 7	0.1	0.0	0.1	1	1	2	0.0	0.0	0.0	0	0	0
Vietnam	Quận 8	0.1	0.0	0.1	2	1	3	0.0	0.0	0.0	0	0	1
Vietnam	Quận 9	0.1	0.0	0.1	1	1	2	0.0	0.0	0.0	0	0	1
Vietnam	Quản Bạ	0.1	0.0	0.2	0	0	1	0.0	0.0	0.0	0	0	0
Vietnam	Quan Hóa	0.1	0.0	0.2	0	0	1	0.0	0.0	0.0	0	0	0
Vietnam	Quan Sơn	0.1	0.1	0.1	0	0	0	0.0	0.0	0.0	0	0	0
Vietnam	Quang Bình	0.1	0.1	0.2	1	0	1	0.0	0.0	0.0	0	0	0
Vietnam	Quảng Điền	0.1	0.1	0.2	1	0	1	0.0	0.0	0.0	0	0	0
Vietnam	Quảng Ngãi	0.1	0.1	0.1	1	1	2	0.0	0.0	0.0	0	0	0
Vietnam	Quảng Ninh	0.1	0.0	0.1	1	0	1	0.0	0.0	0.0	0	0	0
Vietnam	Quảng Trạch	0.1	0.0	0.1	1	0	1	0.0	0.0	0.0	0	0	0
Vietnam	Quảng Trị	0.1	0.1	0.1	0	0	0	0.0	0.0	0.0	0	0	0
Vietnam	Quảng Uyên	0.1	0.1	0.2	0	0	1	0.0	0.0	0.0	0	0	0
Vietnam	Quảng Xương	0.1	0.0	0.1	1	1	2	0.0	0.0	0.0	0	0	0
Vietnam	Quảng Yên	0.1	0.1	0.1	1	1	2	0.0	0.0	0.0	0	0	0
Vietnam	Quế Phong	0.1	0.1	0.1	1	0	1	0.0	0.0	0.0	0	0	0
Vietnam	Quế Sơn	0.1	0.1	0.2	1	0	1	0.0	0.0	0.0	0	0	0
Vietnam	Quế Võ	0.1	0.1	0.2	1	1	2	0.0	0.0	0.0	0	0	0
Vietnam	Qui Nhơn	0.1	0.1	0.2	3	2	4	0.0	0.0	0.0	0	0	1
Vietnam	Quốc Oai	0.1	0.1	0.2	2	1	3	0.0	0.0	0.0	0	0	1

Table 3: Diarrhoea Mortality by Unit *(continued)*

Country	Unit	2000 mortality mean rate (per 10,000)	lower rate (per 10,000)	upper rate (per 10,000)	mean count	lower count	upper count	2017 mortality mean rate (per 10,000)	lower rate (per 10,000)	upper rate (per 10,000)	mean count	lower count	upper count
Vietnam	Quỳ Châu	0.1	0.1	0.1	0	0	1	0.0	0.0	0.0	0	0	0
Vietnam	Quỳ Hợp	0.1	0.1	0.1	1	1	2	0.0	0.0	0.0	0	0	0
Vietnam	Quỳnh Lưu	0.1	0.1	0.1	2	1	3	0.0	0.0	0.0	0	0	1
Vietnam	Quỳnh Nhai	0.1	0.1	0.2	1	0	1	0.0	0.0	0.0	0	0	0
Vietnam	Quỳnh Phụ	0.1	0.1	0.2	2	1	3	0.0	0.0	0.0	0	0	0
Vietnam	Rạch Giá	0.1	0.0	0.1	1	1	2	0.0	0.0	0.0	0	0	0
Vietnam	Sa Đéc	0.1	0.1	0.1	1	0	1	0.0	0.0	0.0	0	0	0
Vietnam	Sa Pa	0.1	0.1	0.2	1	0	1	0.0	0.0	0.0	0	0	0
Vietnam	Sa Thầy	0.1	0.1	0.2	1	0	1	0.0	0.0	0.0	0	0	0
Vietnam	Sầm Sơn	0.1	0.0	0.1	1	0	1	0.0	0.0	0.0	0	0	0
Vietnam	Si Ma Cai	0.1	0.1	0.2	0	0	1	0.0	0.0	0.0	0	0	0
Vietnam	Sìn Hồ	0.1	0.1	0.2	0	0	1	0.0	0.0	0.0	0	0	0
Vietnam	Sóc Sơn	0.1	0.1	0.2	3	1	4	0.0	0.0	0.0	1	0	1
Vietnam	Sóc Trăng	0.1	0.0	0.1	1	0	1	0.0	0.0	0.0	0	0	0
Vietnam	Sơn Động	0.1	0.0	0.1	1	0	1	0.0	0.0	0.0	0	0	0
Vietnam	Sơn Dương	0.1	0.1	0.2	2	1	3	0.0	0.0	0.0	0	0	0
Vietnam	Sơn Hà	0.1	0.1	0.2	1	0	1	0.0	0.0	0.0	0	0	0
Vietnam	Sơn Hòa	0.1	0.1	0.2	1	0	1	0.0	0.0	0.0	0	0	0
Vietnam	Sơn La	0.1	0.1	0.2	1	1	2	0.0	0.0	0.0	0	0	0
Vietnam	Sơn Tây	0.1	0.1	0.2	0	0	0	0.0	0.0	0.0	0	0	0
Vietnam	Sơn Tây	0.1	0.1	0.2	1	1	2	0.0	0.0	0.0	0	0	0
Vietnam	Sơn Tịnh	0.1	0.1	0.1	1	1	1	0.0	0.0	0.0	0	0	0
Vietnam	Sơn Trà	0.1	0.1	0.2	1	1	2	0.0	0.0	0.0	0	0	0
Vietnam	Sông Cầu	0.1	0.1	0.2	1	0	1	0.0	0.0	0.0	0	0	0
Vietnam	Sông Công	0.1	0.1	0.2	1	0	1	0.0	0.0	0.0	0	0	0
Vietnam	Sông Hinh	0.1	0.1	0.2	1	0	1	0.0	0.0	0.0	0	0	0
Vietnam	Sông Lô	0.1	0.1	0.2	1	1	2	0.0	0.0	0.0	0	0	0
Vietnam	Sông Mã	0.1	0.0	0.2	1	1	2	0.0	0.0	0.0	0	0	0
Vietnam	Sốp Cộp	0.1	0.1	0.2	0	0	1	0.0	0.0	0.0	0	0	0
Vietnam	Tam Bình	0.1	0.1	0.1	1	1	2	0.0	0.0	0.0	0	0	0
Vietnam	Tam Đảo	0.1	0.1	0.2	1	0	1	0.0	0.0	0.0	0	0	0
Vietnam	Tam Điệp	0.1	0.1	0.1	1	0	1	0.0	0.0	0.0	0	0	0
Vietnam	Tam Dương	0.1	0.1	0.2	1	1	2	0.0	0.0	0.0	0	0	0
Vietnam	Tam Đường	0.1	0.1	0.2	0	0	1	0.0	0.0	0.0	0	0	0
Vietnam	Tam Kỳ	0.1	0.1	0.1	1	0	1	0.0	0.0	0.0	0	0	0
Vietnam	Tam Nông	0.1	0.1	0.2	2	1	3	0.0	0.0	0.0	0	0	0
Vietnam	Tam Nông	0.1	0.0	0.1	1	0	1	0.0	0.0	0.0	0	0	0
Vietnam	Tân An	0.1	0.1	0.1	1	1	2	0.0	0.0	0.0	0	0	0
Vietnam	Tân Biên	0.1	0.1	0.1	1	0	1	0.0	0.0	0.0	0	0	0
Vietnam	Tân Bình	0.1	0.0	0.1	2	1	4	0.0	0.0	0.0	1	0	1
Vietnam	Tân Châu	0.1	0.0	0.1	1	1	2	0.0	0.0	0.0	0	0	0
Vietnam	Tân Châu	0.1	0.0	0.1	1	1	2	0.0	0.0	0.0	0	0	0
Vietnam	Tân Hiệp	0.1	0.0	0.1	1	1	2	0.0	0.0	0.0	0	0	0
Vietnam	Tân Hồng	0.1	0.0	0.1	1	0	1	0.0	0.0	0.0	0	0	0
Vietnam	Tân Hưng	0.1	0.1	0.1	0	0	1	0.0	0.0	0.0	0	0	0
Vietnam	Tân Kỳ	0.1	0.1	0.1	1	1	2	0.0	0.0	0.0	0	0	0
Vietnam	Tân Lạc	0.1	0.1	0.2	1	0	1	0.0	0.0	0.0	0	0	0
Vietnam	Tân Phú	0.1	0.0	0.1	2	1	3	0.0	0.0	0.0	0	0	1
Vietnam	Tân Phú	0.1	0.0	0.1	1	1	2	0.0	0.0	0.0	0	0	0
Vietnam	Tân Phú Đông	0.1	0.1	0.2	0	0	1	0.0	0.0	0.0	0	0	0
Vietnam	Tân Phước	0.1	0.1	0.1	1	0	1	0.0	0.0	0.0	0	0	0
Vietnam	Tân Sơn	0.1	0.1	0.2	1	1	2	0.0	0.0	0.0	0	0	0
Vietnam	Tân Thành	0.1	0.1	0.1	1	1	2	0.0	0.0	0.0	0	0	0
Vietnam	Tân Thạnh	0.1	0.0	0.1	1	0	1	0.0	0.0	0.0	0	0	0
Vietnam	Tân Trụ	0.1	0.1	0.1	1	0	1	0.0	0.0	0.0	0	0	0
Vietnam	Tân Uyên	0.1	0.0	0.1	0	0	1	0.0	0.0	0.0	0	0	0
Vietnam	Tân Uyên	0.1	0.1	0.2	0	0	1	0.0	0.0	0.0	0	0	0
Vietnam	Tân Yên	0.1	0.1	0.2	1	1	2	0.0	0.0	0.0	0	0	0
Vietnam	Tánh Linh	0.1	0.0	0.1	1	0	1	0.0	0.0	0.0	0	0	0
Vietnam	Tây Giang	0.1	0.1	0.2	0	0	0	0.0	0.0	0.0	0	0	0

Table 3: Diarrhoea Mortality by Unit *(continued)*

		2000 mortality						2017 mortality					
Country	Unit	mean rate (per 10,000)	lower rate (per 10,000)	upper rate (per 10,000)	mean count	lower count	upper count	mean rate (per 10,000)	lower rate (per 10,000)	upper rate (per 10,000)	mean count	lower count	upper count
Vietnam	Tây Hồ	0.1	0.1	0.2	1	1	2	0.0	0.0	0.0	0	0	0
Vietnam	Tây Hoà	0.1	0.1	0.2	1	1	2	0.0	0.0	0.0	0	0	0
Vietnam	Tây Ninh	0.1	0.0	0.1	1	0	1	0.0	0.0	0.0	0	0	0
Vietnam	Tây Sơn	0.1	0.1	0.2	2	1	2	0.0	0.0	0.0	0	0	0
Vietnam	Tây Trà	0.1	0.1	0.1	0	0	0	0.0	0.0	0.0	0	0	0
Vietnam	Thạch An	0.1	0.1	0.2	0	0	1	0.0	0.0	0.0	0	0	0
Vietnam	Thạch Hà	0.1	0.1	0.2	1	1	2	0.0	0.0	0.0	0	0	0
Vietnam	Thạch Thành	0.1	0.1	0.1	1	1	2	0.0	0.0	0.0	0	0	0
Vietnam	Thạch Thất	0.1	0.1	0.2	2	1	3	0.0	0.0	0.0	0	0	1
Vietnam	Thái Bình	0.1	0.1	0.2	2	1	2	0.0	0.0	0.0	0	0	0
Vietnam	Thái Hoà	0.1	0.0	0.1	1	0	1	0.0	0.0	0.0	0	0	0
Vietnam	Thái Nguyên	0.1	0.1	0.2	3	1	4	0.0	0.0	0.0	0	0	1
Vietnam	Thái Thụy	0.1	0.1	0.2	2	1	3	0.0	0.0	0.0	0	0	0
Vietnam	Than Uyên	0.1	0.1	0.2	0	0	1	0.0	0.0	0.0	0	0	0
Vietnam	Thăng Bình	0.1	0.1	0.1	2	1	2	0.0	0.0	0.0	0	0	0
Vietnam	Thanh Ba	0.1	0.1	0.2	2	1	4	0.0	0.0	0.0	0	0	0
Vietnam	Thanh Bình	0.1	0.0	0.1	1	1	2	0.0	0.0	0.0	0	0	0
Vietnam	Thanh Chương	0.1	0.1	0.2	2	1	3	0.0	0.0	0.0	0	0	1
Vietnam	Thanh Hà	0.1	0.1	0.2	1	1	2	0.0	0.0	0.0	0	0	0
Vietnam	Thanh Hóa	0.1	0.0	0.1	2	1	4	0.0	0.0	0.0	0	0	1
Vietnam	Thạnh Hóa	0.1	0.0	0.1	0	0	1	0.0	0.0	0.0	0	0	0
Vietnam	Thanh Khê	0.1	0.1	0.2	1	0	1	0.0	0.0	0.0	0	0	0
Vietnam	Thanh Liêm	0.1	0.1	0.2	1	1	2	0.0	0.0	0.0	0	0	0
Vietnam	Thanh Miện	0.1	0.1	0.2	1	1	2	0.0	0.0	0.0	0	0	0
Vietnam	Thanh Oai	0.1	0.1	0.2	2	1	3	0.0	0.0	0.0	0	0	1
Vietnam	Thành Phố Bắc Kạn	0.1	0.1	0.2	0	0	0	0.0	0.0	0.0	0	0	0
Vietnam	Thành Phố Đồng Hới	0.1	0.0	0.1	1	0	1	0.0	0.0	0.0	0	0	0
Vietnam	Thạnh Phú	0.1	0.1	0.1	1	1	2	0.0	0.0	0.0	0	0	0
Vietnam	Thanh Sơn	0.1	0.1	0.2	2	1	4	0.0	0.0	0.0	0	0	0
Vietnam	Thanh Thuỷ	0.1	0.1	0.2	1	1	2	0.0	0.0	0.0	0	0	0
Vietnam	Thanh Trì	0.1	0.1	0.2	3	1	4	0.0	0.0	0.0	1	0	1
Vietnam	Thạnh Trị	0.1	0.0	0.1	1	0	1	0.0	0.0	0.0	0	0	0
Vietnam	Thanh Xuân	0.1	0.1	0.2	2	1	2	0.0	0.0	0.0	0	0	1
Vietnam	Tháp Mười	0.1	0.0	0.1	1	1	2	0.0	0.0	0.0	0	0	0
Vietnam	Thị Xã Buôn Hồ	0.1	0.1	0.2	1	1	2	0.0	0.0	0.0	0	0	0
Vietnam	Thị Xã Mường Lay	0.1	0.1	0.2	0	0	0	0.0	0.0	0.0	0	0	0
Vietnam	Thiệu Hóa	0.1	0.0	0.1	1	1	2	0.0	0.0	0.0	0	0	0
Vietnam	Thọ Xuân	0.1	0.0	0.1	2	1	2	0.0	0.0	0.0	0	0	0
Vietnam	Thoại Sơn	0.1	0.0	0.1	1	1	2	0.0	0.0	0.0	0	0	0
Vietnam	Thới Bình	0.1	0.0	0.1	1	0	1	0.0	0.0	0.0	0	0	0
Vietnam	Thới Lai	0.1	0.0	0.1	1	0	1	0.0	0.0	0.0	0	0	0
Vietnam	Thống Nhất	0.1	0.0	0.1	1	1	2	0.0	0.0	0.0	0	0	0
Vietnam	Thông Nông	0.1	0.1	0.2	0	0	0	0.0	0.0	0.0	0	0	0
Vietnam	Thốt Nốt	0.1	0.1	0.1	1	1	1	0.0	0.0	0.0	0	0	0
Vietnam	Thủ Dầu Một	0.1	0.0	0.1	1	0	1	0.0	0.0	0.0	0	0	1
Vietnam	Thủ Đức	0.1	0.0	0.1	2	1	3	0.0	0.0	0.0	1	0	1
Vietnam	Thủ Thừa	0.1	0.0	0.1	1	0	1	0.0	0.0	0.0	0	0	0
Vietnam	Thuận An	0.1	0.0	0.1	1	1	2	0.0	0.0	0.0	1	0	1
Vietnam	Thuận Bắc	0.1	0.1	0.1	0	0	1	0.0	0.0	0.0	0	0	0
Vietnam	Thuận Châu	0.1	0.1	0.2	2	1	3	0.0	0.0	0.0	0	0	1
Vietnam	Thuận Nam	0.1	0.1	0.1	1	0	1	0.0	0.0	0.0	0	0	0
Vietnam	Thuận Thành	0.1	0.1	0.2	2	1	3	0.0	0.0	0.0	0	0	0
Vietnam	Thường Tín	0.1	0.1	0.2	2	1	3	0.0	0.0	0.0	0	0	1
Vietnam	Thường Xuân	0.1	0.0	0.1	1	0	1	0.0	0.0	0.0	0	0	0
Vietnam	Thuỷ Nguyên	0.1	0.1	0.1	3	2	4	0.0	0.0	0.0	0	0	1
Vietnam	Tiên Du	0.1	0.1	0.2	1	1	2	0.0	0.0	0.0	0	0	0
Vietnam	Tiền Hải	0.1	0.1	0.2	2	1	3	0.0	0.0	0.0	0	0	0

Table 3: Diarrhoea Mortality by Unit *(continued)*

Country	Unit	2000 mortality mean rate (per 10,000)	2000 mortality lower rate (per 10,000)	2000 mortality upper rate (per 10,000)	2000 mortality mean count	2000 mortality lower count	2000 mortality upper count	2017 mortality mean rate (per 10,000)	2017 mortality lower rate (per 10,000)	2017 mortality upper rate (per 10,000)	2017 mortality mean count	2017 mortality lower count	2017 mortality upper count
Vietnam	Tiên Lãng	0.1	0.1	0.2	1	1	2	0.0	0.0	0.0	0	0	0
Vietnam	Tiên Lữ	0.1	0.1	0.2	1	0	1	0.0	0.0	0.0	0	0	0
Vietnam	Tiên Phước	0.1	0.1	0.1	1	0	1	0.0	0.0	0.0	0	0	0
Vietnam	Tiên Yên	0.1	0.0	0.1	0	0	1	0.0	0.0	0.0	0	0	0
Vietnam	Tiểu Cần	0.1	0.0	0.1	1	0	1	0.0	0.0	0.0	0	0	0
Vietnam	Tịnh Biên	0.1	0.0	0.1	1	1	1	0.0	0.0	0.0	0	0	0
Vietnam	Tĩnh Gia	0.1	0.0	0.1	2	1	3	0.0	0.0	0.0	0	0	0
Vietnam	Trà Bồng	0.1	0.1	0.1	0	0	0	0.0	0.0	0.0	0	0	0
Vietnam	Trà Cú	0.1	0.0	0.1	1	1	2	0.0	0.0	0.0	0	0	0
Vietnam	Trà Lĩnh	0.1	0.1	0.2	0	0	0	0.0	0.0	0.0	0	0	0
Vietnam	Trà Ôn	0.1	0.1	0.2	1	1	2	0.0	0.0	0.0	0	0	0
Vietnam	Trà Vinh	0.1	0.0	0.1	1	0	1	0.0	0.0	0.0	0	0	0
Vietnam	Trạm Tấu	0.1	0.1	0.2	0	0	1	0.0	0.0	0.0	0	0	0
Vietnam	Trần Đề	0.1	0.0	0.1	1	0	2	0.0	0.0	0.0	0	0	0
Vietnam	Trần Văn Thời	0.1	0.0	0.1	1	1	2	0.0	0.0	0.0	0	0	0
Vietnam	Trấn Yên	0.1	0.1	0.2	1	0	1	0.0	0.0	0.0	0	0	0
Vietnam	Trảng Bàng	0.1	0.0	0.1	1	1	2	0.0	0.0	0.0	0	0	0
Vietnam	Trảng Bom	0.1	0.0	0.1	2	1	2	0.0	0.0	0.0	0	0	0
Vietnam	Tràng Định	0.1	0.1	0.2	1	0	1	0.0	0.0	0.0	0	0	0
Vietnam	Tri Tôn	0.1	0.0	0.1	1	1	2	0.0	0.0	0.0	0	0	0
Vietnam	Triệu Phong	0.1	0.0	0.1	1	1	2	0.0	0.0	0.0	0	0	0
Vietnam	Triệu Sơn	0.1	0.0	0.1	1	1	2	0.0	0.0	0.0	0	0	0
Vietnam	Trực Ninh	0.1	0.1	0.2	2	1	3	0.0	0.0	0.0	0	0	0
Vietnam	Trùng Khánh	0.1	0.1	0.2	0	0	1	0.0	0.0	0.0	0	0	0
Vietnam	Tứ Kỳ	0.1	0.1	0.2	1	1	2	0.0	0.0	0.0	0	0	0
Vietnam	Tu Mơ Rông	0.1	0.1	0.2	0	0	0	0.0	0.0	0.0	0	0	0
Vietnam	Tư Nghĩa	0.1	0.1	0.1	1	1	2	0.0	0.0	0.0	0	0	0
Vietnam	Từ Sơn	0.1	0.1	0.2	1	1	2	0.0	0.0	0.0	0	0	0
Vietnam	Tủa Chùa	0.1	0.1	0.2	1	0	1	0.0	0.0	0.0	0	0	0
Vietnam	Tuần Giáo	0.1	0.1	0.2	1	0	1	0.0	0.0	0.0	0	0	0
Vietnam	Tương Dương	0.1	0.1	0.2	1	0	1	0.0	0.0	0.0	0	0	0
Vietnam	Tuy An	0.1	0.1	0.2	1	1	2	0.0	0.0	0.0	0	0	0
Vietnam	Tuy Đức	0.1	0.1	0.2	0	0	1	0.0	0.0	0.0	0	0	0
Vietnam	Tuy Hoà	0.1	0.1	0.2	1	1	2	0.0	0.0	0.0	0	0	0
Vietnam	Tuy Phong	0.1	0.0	0.1	1	1	2	0.0	0.0	0.0	0	0	0
Vietnam	Tuy Phước	0.1	0.1	0.2	2	1	3	0.0	0.0	0.0	0	0	0
Vietnam	Tuyên Hóa	0.1	0.0	0.1	1	0	1	0.0	0.0	0.0	0	0	0
Vietnam	Tuyên Quang	0.1	0.1	0.2	1	1	2	0.0	0.0	0.0	0	0	0
Vietnam	U Minh	0.1	0.0	0.1	1	0	1	0.0	0.0	0.0	0	0	0
Vietnam	U Minh Thượng	0.1	0.0	0.1	0	0	1	0.0	0.0	0.0	0	0	0
Vietnam	Ứng Hòa	0.1	0.1	0.2	2	1	3	0.0	0.0	0.0	0	0	1
Vietnam	Uông Bí	0.1	0.1	0.1	1	0	1	0.0	0.0	0.0	0	0	0
Vietnam	Văn Bàn	0.1	0.1	0.2	1	1	2	0.0	0.0	0.0	0	0	0
Vietnam	Vân Canh	0.1	0.1	0.2	0	0	1	0.0	0.0	0.0	0	0	0
Vietnam	Văn Chấn	0.1	0.1	0.2	2	1	3	0.0	0.0	0.0	0	0	0
Vietnam	Vân Đồn	0.1	0.0	0.1	0	0	0	0.0	0.0	0.0	0	0	0
Vietnam	Văn Giang	0.1	0.1	0.2	1	1	2	0.0	0.0	0.0	0	0	0
Vietnam	Vân Hồ	0.1	0.1	0.2	1	0	1	0.0	0.0	0.0	0	0	0
Vietnam	Văn Lâm	0.1	0.1	0.2	1	1	2	0.0	0.0	0.0	0	0	0
Vietnam	Văn Lãng	0.1	0.1	0.2	0	0	1	0.0	0.0	0.0	0	0	0
Vietnam	Vạn Ninh	0.1	0.1	0.2	1	1	1	0.0	0.0	0.0	0	0	0
Vietnam	Văn Quan	0.1	0.0	0.1	0	0	1	0.0	0.0	0.0	0	0	0
Vietnam	Văn Yên	0.1	0.1	0.2	1	1	2	0.0	0.0	0.0	0	0	0
Vietnam	Vị Thanh	0.1	0.0	0.1	1	0	1	0.0	0.0	0.0	0	0	0
Vietnam	Vị Thuỷ	0.1	0.0	0.1	1	0	1	0.0	0.0	0.0	0	0	0
Vietnam	Vị Xuyên	0.1	0.1	0.2	1	1	2	0.0	0.0	0.0	0	0	0
Vietnam	Việt Trì	0.1	0.1	0.2	3	2	5	0.0	0.0	0.0	0	0	0
Vietnam	Việt Yên	0.1	0.1	0.2	2	1	3	0.0	0.0	0.0	0	0	0
Vietnam	Vinh	0.1	0.1	0.2	3	1	4	0.0	0.0	0.0	0	0	1
Vietnam	Vĩnh Bảo	0.1	0.1	0.2	2	1	3	0.0	0.0	0.0	0	0	0

Table 3: Diarrhoea Mortality by Unit *(continued)*

Country	Unit	2000 mortality mean rate (per 10,000)	lower rate (per 10,000)	upper rate (per 10,000)	mean count	lower count	upper count	2017 mortality mean rate (per 10,000)	lower rate (per 10,000)	upper rate (per 10,000)	mean count	lower count	upper count
Vietnam	Vĩnh Châu	0.1	0.0	0.1	1	1	2	0.0	0.0	0.0	0	0	0
Vietnam	Vĩnh Cửu	0.1	0.0	0.1	1	0	1	0.0	0.0	0.0	0	0	0
Vietnam	Vĩnh Hưng	0.1	0.1	0.1	0	0	1	0.0	0.0	0.0	0	0	0
Vietnam	Vĩnh Linh	0.1	0.0	0.1	1	0	1	0.0	0.0	0.0	0	0	0
Vietnam	Vĩnh Lộc	0.1	0.0	0.1	1	0	1	0.0	0.0	0.0	0	0	0
Vietnam	Vĩnh Lợi	0.1	0.0	0.1	1	0	1	0.0	0.0	0.0	0	0	0
Vietnam	Vĩnh Long	0.1	0.1	0.1	1	0	1	0.0	0.0	0.0	0	0	0
Vietnam	Vĩnh Thạnh	0.1	0.1	0.2	0	0	1	0.0	0.0	0.0	0	0	0
Vietnam	Vĩnh Thạnh	0.1	0.0	0.1	1	1	1	0.0	0.0	0.0	0	0	0
Vietnam	Vĩnh Thuận	0.1	0.0	0.1	1	0	1	0.0	0.0	0.0	0	0	0
Vietnam	Vĩnh Tường	0.1	0.1	0.2	2	1	4	0.0	0.0	0.0	0	0	1
Vietnam	Vĩnh Yên	0.1	0.1	0.2	1	1	2	0.0	0.0	0.0	0	0	0
Vietnam	Võ Nhai	0.1	0.1	0.1	1	0	1	0.0	0.0	0.0	0	0	0
Vietnam	Vụ Bản	0.1	0.1	0.2	2	1	2	0.0	0.0	0.0	0	0	0
Vietnam	Vũ Quang	0.1	0.1	0.2	0	0	1	0.0	0.0	0.0	0	0	0
Vietnam	Vũ Thư	0.1	0.1	0.2	2	1	4	0.0	0.0	0.0	0	0	1
Vietnam	Vũng Liêm	0.1	0.1	0.2	1	1	2	0.0	0.0	0.0	0	0	0
Vietnam	Vũng Tàu	0.1	0.0	0.1	2	1	3	0.0	0.0	0.0	0	0	1
Vietnam	Xín Mần	0.1	0.1	0.2	1	0	1	0.0	0.0	0.0	0	0	0
Vietnam	Xuân Lộc	0.1	0.0	0.1	1	1	2	0.0	0.0	0.0	0	0	0
Vietnam	Xuân Trường	0.1	0.1	0.2	2	1	2	0.0	0.0	0.0	0	0	0
Vietnam	Xuyên Mộc	0.1	0.0	0.1	1	1	2	0.0	0.0	0.0	0	0	0
Vietnam	Ý Yên	0.1	0.1	0.2	2	1	4	0.0	0.0	0.0	0	0	0
Vietnam	Yên Bái	0.1	0.1	0.2	1	1	2	0.0	0.0	0.0	0	0	0
Vietnam	Yên Bình	0.1	0.1	0.2	1	1	2	0.0	0.0	0.0	0	0	0
Vietnam	Yên Châu	0.1	0.1	0.2	1	0	1	0.0	0.0	0.0	0	0	0
Vietnam	Yên Định	0.1	0.0	0.1	1	1	2	0.0	0.0	0.0	0	0	0
Vietnam	Yên Dũng	0.1	0.1	0.2	1	1	2	0.0	0.0	0.0	0	0	0
Vietnam	Yên Khánh	0.1	0.1	0.1	1	1	2	0.0	0.0	0.0	0	0	0
Vietnam	Yên Lạc	0.1	0.1	0.2	2	1	3	0.0	0.0	0.0	0	0	0
Vietnam	Yên Lập	0.1	0.1	0.2	2	1	3	0.0	0.0	0.0	0	0	0
Vietnam	Yên Minh	0.1	0.1	0.2	1	0	1	0.0	0.0	0.0	0	0	0
Vietnam	Yên Mô	0.1	0.1	0.1	1	1	2	0.0	0.0	0.0	0	0	0
Vietnam	Yên Mỹ	0.1	0.1	0.2	1	1	2	0.0	0.0	0.0	0	0	0
Vietnam	Yên Phong	0.1	0.1	0.2	1	1	2	0.0	0.0	0.0	0	0	0
Vietnam	Yên Sơn	0.1	0.1	0.2	2	1	3	0.0	0.0	0.0	0	0	0
Vietnam	Yên Thành	0.1	0.1	0.1	2	1	4	0.0	0.0	0.0	0	0	1
Vietnam	Yên Thế	0.1	0.1	0.2	1	0	1	0.0	0.0	0.0	0	0	0
Vietnam	Yên Thủy	0.1	0.1	0.1	1	0	1	0.0	0.0	0.0	0	0	0
**Sub-Saharan Africa**													
Angola	Alto Cauale	6.5	4.9	8.7	128	96	170	1.9	1.5	2.4	14	11	18
Angola	Alto Zambeze	7.0	5.5	8.8	34	27	43	2.0	1.7	2.5	55	46	67
Angola	Ambaca	6.5	4.7	8.4	120	86	155	2.0	1.6	2.5	19	15	24
Angola	Amboim	8.9	6.9	11.3	253	194	321	2.5	1.9	3.1	115	89	144
Angola	Ambriz	7.5	5.7	9.9	34	26	45	2.3	1.9	3.0	8	6	10
Angola	Ambuila	6.6	4.9	8.7	53	39	70	1.9	1.5	2.5	6	4	7
Angola	Andulo	7.5	5.7	9.8	435	329	566	2.0	1.6	2.5	84	66	105
Angola	Baía Farta	9.4	6.6	12.2	155	109	199	2.7	2.1	3.4	50	39	63
Angola	Bailundo	9.2	7.0	12.0	508	383	658	2.4	1.9	2.9	125	99	153
Angola	Balombo	9.2	6.8	12.0	129	96	168	2.4	1.8	3.1	43	32	54
Angola	Banga	7.1	5.1	9.4	15	11	20	2.1	1.6	2.6	2	2	3
Angola	Belize	7.4	5.8	9.4	4	3	5	2.0	1.5	2.4	8	6	10
Angola	Bembe	6.7	4.8	8.9	84	61	112	2.0	1.6	2.5	10	8	13
Angola	Benguela	9.3	6.4	12.3	612	421	809	2.5	1.9	3.2	204	158	262
Angola	Bibala	7.3	5.6	9.6	31	24	41	2.4	1.8	3.0	31	24	39
Angola	Bocoio	8.9	6.5	11.5	197	144	255	2.4	1.8	3.0	65	50	81
Angola	Bolongongo	6.9	4.7	8.9	32	22	41	2.0	1.6	2.5	5	4	6
Angola	Buco Zau	7.1	5.5	9.0	7	6	9	2.0	1.5	2.4	14	11	18
Angola	Buengas	6.3	4.9	8.1	141	110	182	1.9	1.5	2.5	16	12	21
Angola	Bula Atumba	7.1	5.1	9.1	23	17	30	2.0	1.5	2.4	5	4	6

Table 3: Diarrhoea Mortality by Unit *(continued)*

		2000 mortality						2017 mortality					
Country	Unit	mean rate (per 10,000)	lower rate (per 10,000)	upper rate (per 10,000)	mean count	lower count	upper count	mean rate (per 10,000)	lower rate (per 10,000)	upper rate (per 10,000)	mean count	lower count	upper count
Angola	Bungo	6.1	4.6	8.2	105	79	140	2.0	1.5	2.5	12	9	16
Angola	Caála	7.6	5.7	9.7	395	298	507	2.2	1.7	2.8	110	87	140
Angola	Cabinda	6.9	5.2	8.9	83	63	107	1.8	1.4	2.3	223	173	286
Angola	Cacolo	7.0	5.2	8.6	34	25	41	2.0	1.6	2.5	13	10	16
Angola	Caconda	7.4	5.2	9.5	82	58	106	2.2	1.6	2.7	77	58	97
Angola	Cacuaco	6.9	5.0	9.2	85	61	114	1.8	1.4	2.3	451	346	578
Angola	Cacuzo	8.9	6.7	11.6	115	87	151	2.5	2.0	3.1	38	31	47
Angola	Cahama	7.1	5.1	9.5	41	29	55	2.0	1.6	2.7	33	26	43
Angola	Caiambambo	8.8	6.1	11.1	109	76	138	2.2	1.7	2.9	34	26	44
Angola	Calai	8.0	6.0	9.9	43	32	53	2.4	1.9	3.1	12	10	16
Angola	Calandula	8.1	6.1	10.5	113	85	145	2.4	1.9	2.9	41	32	51
Angola	Caluquembe	7.6	5.6	9.9	101	75	132	2.1	1.6	2.6	89	67	112
Angola	Camacuio	8.3	6.2	10.6	27	20	34	2.5	1.9	3.0	25	20	31
Angola	Camacupa	6.6	4.8	8.4	229	167	292	1.9	1.5	2.4	49	38	61
Angola	Camanongue	7.3	5.5	9.4	11	9	15	2.0	1.5	2.6	17	13	22
Angola	Cambambe	7.4	5.5	9.5	179	135	230	2.1	1.7	2.7	29	22	36
Angola	Cambulo	7.7	5.6	9.9	110	80	142	2.2	1.7	2.7	59	46	73
Angola	Cambundi-Catembo	8.7	6.6	11.3	60	45	78	2.3	1.8	3.0	20	15	26
Angola	Cameia	7.3	5.7	9.6	9	7	12	2.0	1.5	2.5	14	11	18
Angola	Cangandala	9.1	7.0	12.1	81	63	108	2.4	1.9	3.0	23	19	29
Angola	Caombo	8.8	6.5	11.4	31	23	40	2.2	1.8	2.8	10	8	12
Angola	Capenda	6.6	4.9	8.6	44	33	57	1.9	1.4	2.3	22	17	28
Angola	Cassongue	9.4	7.1	12.0	171	130	220	2.3	1.8	2.8	65	52	81
Angola	Catabola	6.6	4.9	8.6	196	145	255	1.9	1.4	2.5	41	31	53
Angola	Catchiungo	7.4	5.6	9.2	162	122	203	2.2	1.8	2.8	47	37	59
Angola	Caungula	7.2	5.3	9.4	26	19	34	2.1	1.6	2.5	13	10	16
Angola	Cazenga	6.6	4.6	9.0	44	31	60	1.9	1.4	2.4	261	196	338
Angola	Cazengo	7.5	5.6	9.8	344	258	449	2.0	1.6	2.5	52	41	65
Angola	Chibia	7.4	5.5	9.6	93	69	121	2.4	1.9	3.1	98	76	126
Angola	Chicomba	7.5	5.4	10.2	63	46	86	2.2	1.7	2.8	60	47	76
Angola	Chinguar	6.8	5.1	8.6	200	150	252	2.0	1.6	2.6	43	34	56
Angola	Chipindo	7.7	5.6	10.1	35	26	46	2.2	1.7	2.8	30	24	38
Angola	Chitato	7.5	5.5	9.9	181	134	238	2.3	1.7	2.8	100	76	122
Angola	Chitembo	6.6	5.1	8.2	117	90	145	1.9	1.5	2.3	24	19	30
Angola	Chongoroi	8.8	6.4	11.2	100	73	127	2.4	1.8	3.1	35	27	45
Angola	Conda	9.3	7.1	11.9	106	82	136	2.6	2.0	3.2	46	36	57
Angola	Cuaba Nzogo	8.9	6.5	11.6	23	17	30	2.5	1.9	3.1	8	6	10
Angola	Cuangar	8.0	6.3	10.1	53	42	67	2.4	1.9	2.9	12	10	15
Angola	Cuango	7.3	5.4	9.3	149	112	190	1.9	1.6	2.4	71	57	87
Angola	Cuanhama	6.8	5.1	8.8	203	151	262	1.7	1.3	2.1	141	109	178
Angola	Cubal	8.6	6.2	11.0	337	242	432	2.2	1.7	2.8	108	83	141
Angola	Cuchi	7.3	5.9	9.0	74	59	90	2.0	1.6	2.5	16	12	20
Angola	Cuemba	6.5	4.8	8.1	83	62	104	1.9	1.5	2.4	19	14	24
Angola	Cuilo	7.1	5.4	9.2	19	14	24	2.1	1.6	2.6	10	8	12
Angola	Cuimba	7.4	5.6	9.5	28	21	36	2.1	1.7	2.6	26	21	33
Angola	Cuito Cuanavale	7.4	5.9	9.1	69	55	86	2.1	1.7	2.4	15	12	17
Angola	Cunda-dia-Baza	8.4	6.1	10.8	17	13	22	2.2	1.7	2.8	6	5	7
Angola	Cunhinga	6.5	4.7	8.4	118	85	152	1.9	1.4	2.4	24	18	31
Angola	Curoca	6.4	4.7	8.1	23	17	29	1.9	1.5	2.4	19	15	24
Angola	Cuvelai	7.0	5.3	9.0	36	27	46	1.9	1.5	2.3	27	21	33
Angola	Dala	7.2	5.5	9.3	31	24	40	2.0	1.5	2.5	12	9	15
Angola	Damba	6.3	5.0	8.1	162	130	209	2.0	1.6	2.5	19	15	25
Angola	Dande	7.3	5.4	9.5	329	243	425	2.2	1.7	2.7	81	64	102
Angola	Dembos	7.0	5.1	9.3	40	29	54	2.1	1.6	2.6	9	7	12
Angola	Dirico	7.7	6.1	9.3	31	25	38	2.4	1.9	3.0	9	7	11
Angola	Ebo	8.9	6.7	11.4	178	134	227	2.4	1.8	3.0	75	58	93
Angola	Ekunha	8.0	6.1	10.7	131	100	177	2.3	1.8	2.9	35	28	45
Angola	Gambos	7.3	5.3	9.4	39	28	50	2.4	1.9	3.1	41	32	53
Angola	Ganda	8.0	6.0	10.2	252	187	321	2.2	1.7	2.8	89	69	112

Table 3: Diarrhoea Mortality by Unit *(continued)*

Country	Unit	2000 mortality mean rate (per 10,000)	lower rate (per 10,000)	upper rate (per 10,000)	mean count	lower count	upper count	2017 mortality mean rate (per 10,000)	lower rate (per 10,000)	upper rate (per 10,000)	mean count	lower count	upper count
Angola	Golungo Alto	7.3	5.4	9.4	68	51	89	2.1	1.6	2.6	10	8	13
Angola	Huambo	7.7	5.9	10.1	948	727	1240	2.1	1.7	2.7	260	206	329
Angola	Humpata	8.0	6.0	10.5	51	39	67	2.3	1.7	2.9	55	42	69
Angola	Icolo e Bengo	7.3	5.3	9.7	12	9	16	2.2	1.7	2.7	48	37	61
Angola	Ingombota	6.2	4.3	8.5	20	14	27	1.8	1.4	2.3	117	88	152
Angola	Jamba	7.6	5.9	9.9	52	41	68	2.2	1.7	2.7	49	38	61
Angola	Kilamba Kiaxi	6.7	4.6	9.1	61	43	83	1.8	1.4	2.4	365	272	471
Angola	Kuito	6.7	5.0	8.5	693	511	877	2.0	1.5	2.5	149	113	190
Angola	Kuvango	7.1	5.4	9.1	39	30	50	2.0	1.6	2.5	34	26	42
Angola	Landana	7.0	5.4	8.7	7	5	8	2.1	1.6	2.6	17	13	21
Angola	Léua	7.4	5.6	9.5	11	8	14	2.0	1.5	2.6	17	13	21
Angola	Libolo	8.1	6.3	10.2	87	68	110	2.3	1.8	2.8	37	30	46
Angola	Lobito	9.3	6.7	12.4	641	464	858	2.6	2.1	3.3	227	182	288
Angola	Londuimbale	8.9	6.7	11.7	217	163	286	2.5	2.0	3.1	59	46	73
Angola	Longonjo	8.0	6.0	10.7	133	99	178	2.2	1.7	2.8	36	28	46
Angola	Luau	7.1	5.4	9.3	28	21	36	2.0	1.5	2.5	43	34	55
Angola	Lubalo	6.6	4.9	8.2	21	16	26	1.9	1.5	2.3	11	9	14
[truncated: 799,406 more chars]
